# Diurnal Rhythms Result in Significant Changes in the Cellular Protein Complement in the Cyanobacterium *Cyanothece* 51142

**DOI:** 10.1371/journal.pone.0016680

**Published:** 2011-02-22

**Authors:** Jana Stöckel, Jon M. Jacobs, Thanura R. Elvitigala, Michelle Liberton, Eric A. Welsh, Ashoka D. Polpitiya, Marina A. Gritsenko, Carrie D. Nicora, David W. Koppenaal, Richard D. Smith, Himadri B. Pakrasi

**Affiliations:** 1 Department of Biology, Washington University, St. Louis, Missouri, United States of America; 2 Pacific Northwest National Laboratory, Richland, Washington, United States of America; 3 Center for Proteomics Translational Genomics Research Institute, Phoenix, Arizona, United States of America; King Abdullah University of Science and Technology, Saudi Arabia

## Abstract

*Cyanothece* sp. ATCC 51142 is a diazotrophic cyanobacterium notable for its ability to perform oxygenic photosynthesis and dinitrogen fixation in the same single cell. Previous transcriptional analysis revealed that the existence of these incompatible cellular processes largely depends on tightly synchronized expression programs involving ∼30% of genes in the genome. To expand upon current knowledge, we have utilized sensitive proteomic approaches to examine the impact of diurnal rhythms on the protein complement in *Cyanothece* 51142. We found that 250 proteins accounting for ∼5% of the predicted ORFs from the *Cyanothece* 51142 genome and 20% of proteins detected under alternating light/dark conditions exhibited periodic oscillations in their abundances. Our results suggest that altered enzyme activities at different phases during the diurnal cycle can be attributed to changes in the abundance of related proteins and key compounds. The integration of global proteomics and transcriptomic data further revealed that post-transcriptional events are important for temporal regulation of processes such as photosynthesis in *Cyanothece* 51142. This analysis is the first comprehensive report on global quantitative proteomics in a unicellular diazotrophic cyanobacterium and uncovers novel findings about diurnal rhythms.

## Introduction

The era of genome sequencing has provided a wealth of genomic information, ranging from entire human and plant genomes to hundreds of completely or partially annotated bacterial genomes. Current transcriptomic and proteomic technologies provide the opportunity to convert such static information into a more comprehensive picture of biological systems [1]–[2]. Recent efforts in analyzing the proteome of *Synechocystis* sp. PCC 6803 under various environmental conditions using high throughput proteomic technologies resulted in a protein coverage of ∼53% which represents the most complete functional and quantitative description of the proteome of any cyanobacterium till date [3]. The characterization of cyanobacterial biology is important because these gram negative photoautotrophs have the ability to perform oxygenic photosynthesis and thus significantly contribute to the global carbon and oxygen cycle. In addition, many strains are capable of atmospheric nitrogen fixation catalyzed by nitrogenase, a multiprotein enzyme highly sensitive to oxygen. Cyanobacteria are the only diazotrophic organisms which produce molecular oxygen as a by-product of photosynthesis, and have evolved different mechanisms to balance the presence of oxygen with the activity of an oxygen-sensitive enzyme [4].

*Cyanothece* sp. ATCC 51142 is a unicellular diazotrophic cyanobacterium with the ability to temporally separate the processes of oxygenic photosynthesis and dinitrogen fixation during a light/dark cycle. A recent DNA microarray study in *Cyanothece* 51142 showed that diurnal changes in cellular activities such as photosynthesis, respiration, and nitrogen fixation are vastly anticipated at the transcriptional level [5]. This analysis further revealed a co-regulation of essentially all central metabolic pathways, with the maximal expression of related genes at distinct phases throughout the diurnal cycle. These data subsequently raise the question of how these RNA profiles are reflected at the protein level and, in particular, how the temporal separation of oxygenic photosynthesis and nitrogen fixation are implemented. The utilization of sensitive high throughput proteomic approaches holds the potential to answer these questions and forms the basis for this study, which represents a more in-depth and expanded version of the limited proteogenomic data integrated into the *Cyanothece* 51142 genomic report [6].

To date, only a limited number of proteome studies are available that emphasize temporal changes of the cellular protein complement. Previous proteomics work in the diatom *L. polyedra*, using 2-dimensional polyacrylamide gel electrophoresis in combination with LC-MS/MS, found that 28 out of 900 (∼3%) identified proteins show changes in their abundance over a circadian period [7]. In the current study, we observed ∼68% of the proteins predicted from the completely sequenced *Cyanothece* 51142 genome. Quantitative proteome analysis identified ∼20% of the detected proteins as exhibiting measurable oscillations in abundance under alternating light/dark conditions during the diurnal cycle. Using previously obtained transcriptomics data in conjunction with the protein abundance profiles from this study, gene/protein expression correlations across a tightly regulated diurnal cycle revealed pathway and functional category specific differences. Thus, post-transcriptional events appear to play a significant role in the temporal regulation of processes such as photosynthesis in *Cyanothece* 51142. Our data also suggest that diurnal changes in activities of several enzymes involved in central metabolic processes are mainly attributed to changes in abundance of associated proteins and key components.

## Results and Discussion

### Proteome analysis identified 68% of the protein-encoding ORFs predicted from the *Cyanothece* 51142 genome

Proteome analysis was based on 5304 protein-encoding ORFs predicted from the final annotation of the *Cyanothece* 51142 genome [6]. To obtain a higher order of proteome coverage, proteins were extracted from cells grown under a variety of culture conditions and separation methods (Table 1). More than 1000 LC-MS/MS analyses from different *Cyanothece* 51142 cultures resulted in identification of 41,469 unique peptides (Table S1). In total, 3,616 different proteins were detected corresponding to 68.2% of the predicted *Cyanothece* 51142 ORFs (Table S2). Out of these 3,616 proteins, 79% are represented by multiple peptide identifications. Table 1 summarizes the analyses performed under all experimental conditions. The largest gains of unique peptide identifications were achieved using additional SCX-fractionations, mainly in combination with either subcellular fractionations and/or variations of growth conditions. This is particularly evident for the thylakoid membrane isolation and fractionation of samples obtained from cultures grown under 12 hour light/dark conditions in conjunction with SCX-fractionation (Table 1).

**Table 1:** Data sets contributing to the *Cyanothece* 51142 proteome coverage.

Growth condition	Illumination	Pre-MS Separation	MS/MS run #	Detected Peptides #	% Unique Peptides
+NO_3_	continuous light	none	2	3303	1.4
-NaCl	continuous light	none	2	3405	1.7
-Fe	continuous light	none	2	2989	3.1
-Fe/+Fe	continuous light	none	2	3642	1.4
-NO_3_/dark	12 h light/dark	none	130	13611	5.8
-NO_3_/light	12 h light/dark	none	134	15310	9.1
-NO_3_/dark	12 h light/dark	SCX	369	29056	17.1
-NO_3_/light	12 h light/dark	SCX	369	26797	12.1
-NO_3_	12 h light/dark	membrane	1	1274	0.3
-NO_3_	12 h light/dark	soluble	1	2047	0.2
-NO_3_	12 h light/dark	thylakoid	1	1593	1.5
-NO_3_	12 h light/dark	membrane-SCX	25	5058	4.7
-NO_3_	12 h light/dark	soluble-SCX	25	8352	5.4
-NO_3_	12 h light/dark	thylakoid-SCX	25	8535	19.8
-NO_3_	12 h light/dark	1D gel slices	7	4243	16.2

The % unique peptides column corresponds to the % of peptides that are not identified under any other condition. This table does not represent the entire number of datasets compiled within the peptide database, but includes and compares the majority of contributing datasets. All 12 hour time-course samples correspond to samples taken every 2 hours across either the 12 hour light or dark period for downstream LC-MS/MS analyses. Continuous light samples correspond to collection of cell cultures at a single time point after continuous light exposure.

### Global protein profiling during a diurnal period

In order to gain further insights into the diurnal lifestyle of *Cyanothece* 51142, and to correlate temporally regulated physiological phenomena with protein levels, a quantitative in-depth global proteome survey was performed. Digested peptides of samples collected from cultures grown under nitrogen fixing conditions over two consecutive diurnal periods (48 hours), sampled every 2 hours, were analyzed using LC-MS/MS. A total of 100 LC-MS/MS analyses were performed, which represent a subset of the >1000 LC-MS/MS analyses described above. The MS/MS spectra were identified and the sums of the spectra from all peptides for any particular protein were combined to obtain the count value for that time point (Figure S1; Table S3). To note, MS/MS spectrum count-based quantitation is peptide centric and subject to variations that are peptide specific, i.e. digestion, LC-separation, and ionization efficiencies. However, this relative quantitative approach has shown to be reliable when based on comparing the same peptides/proteins across multiple, but similar, samples provided that proper controls and experimental designs are in place [8], [9].

**Figure 1:**
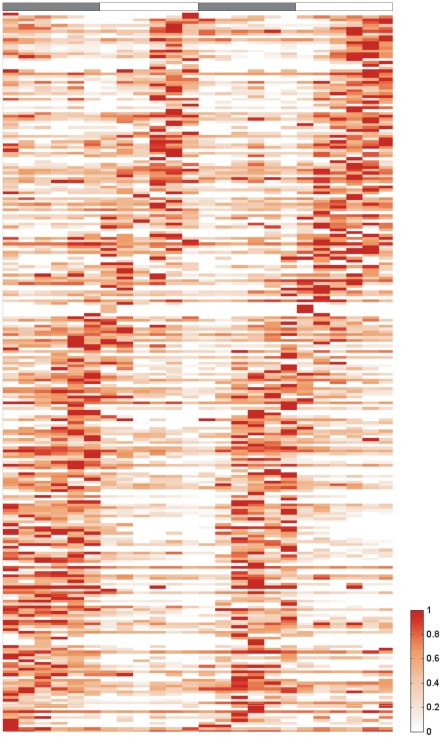
Hierarchical clustered heat map of proteins with diurnal abundance changes. The samples were taken from cultures grown under nitrogen fixing conditions in alternating light/dark cycles over a period of 48 hours. Each colored area in the template represents the relative spectral counts of the associated proteins. *White* colored areas reveal absence of data.

**Figure 2:**
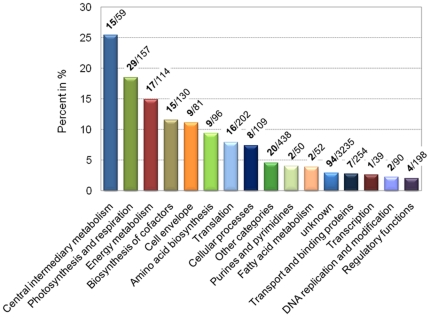
Functional category breakdown for all cyclic proteins. The graph shows the percentage of cyclic proteins within each functional category, calculated based on the total number of predicted proteins in each functional category. The number of cyclic proteins *vs*. the total number of proteins from each functional category is shown in bold. Functional categories for each gene are assigned as [6].

Out of a total of 1,232 proteins identified under these conditions, 250 proteins (20.3%) exhibited cyclic changes in abundance over both diurnal periods. Of these, 113 proteins show a higher abundance during the light, while 137 proteins exhibit higher protein levels during the dark period (Figure 1; Tables S4 and S5). The peak time distribution for all proteins with known functions is shown in Figure S2. The 250 proteins with oscillating profiles were identified using a combination of previously described cycle detection approaches [10], [11]. Figure 2 shows the functional category breakdown for these 250 proteins. The majority of proteins, 40.3%, belongs to central metabolism-based (central intermediary and energy) pathways, such as nitrogen fixation and glycogen degradation. In addition, 18.5% of the proteins are involved in photosynthesis and respiration and 11.5% and 11.1% of proteins are associated with the biosynthesis of cofactors and the cell envelope, respectively.

#### Integration of transcriptional and translational data

**Figure 3:**
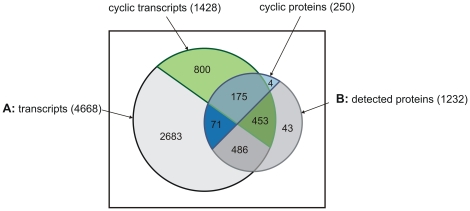
Venn diagram representing all transcripts and proteins with and without oscillating abundance profiles during a diurnal cycle. (A) Transcripts present on the microarray and (B) proteins detected under diurnal conditions. Transcripts identified as cyclic are shaded in *green*, while proteins identified as cyclic are in *blue*.

Previously conducted transcriptional analyses [5] provide the opportunity to integrate transcriptomic and proteomic data for this model system. Of particular interest are genes with diurnal expression profiles that lead to proteins with diurnal changes in their steady state abundance. Importantly, among this group of proteins are both low and high abundance proteins (Figure S3). Of the 250 cyclic proteins identified above, 175 overlap with genes previously identified as cyclic at the transcript level [5] (Figure 3). A more detailed survey of the 71 non-overlapping cyclic proteins revealed that 35 of the corresponding transcripts were additionally classified as diurnally expressed in a recent study [11], that 4 genes were not represented on previously used microarrays, and that the transcription level data of the remaining 32 genes showed either fold changes below the threshold level or high variations in the expression levels which impeded distinct classifications as cyclic or non-cyclic. In total, 70.0% of proteins (175 *vs*. 250) detected as cyclic with available orthogonal data show cyclic correlation at the transcriptomic level.

In contrast, only 27.9% of the 628 cycling genes (175 out of 628) for which proteins were detected are shown to be subsequently cyclic at the protein level. This observation implies that, even though transcription and translation in prokaryotes are tightly coupled [12] and protein abundances are assumed to mirror the mRNA levels, a significant extent of post-translational events, i.e. regulation of degradation rates, are important to determine the protein repertoire of *Cyanothece* 51142 during a diurnal period.

**Figure 4:**
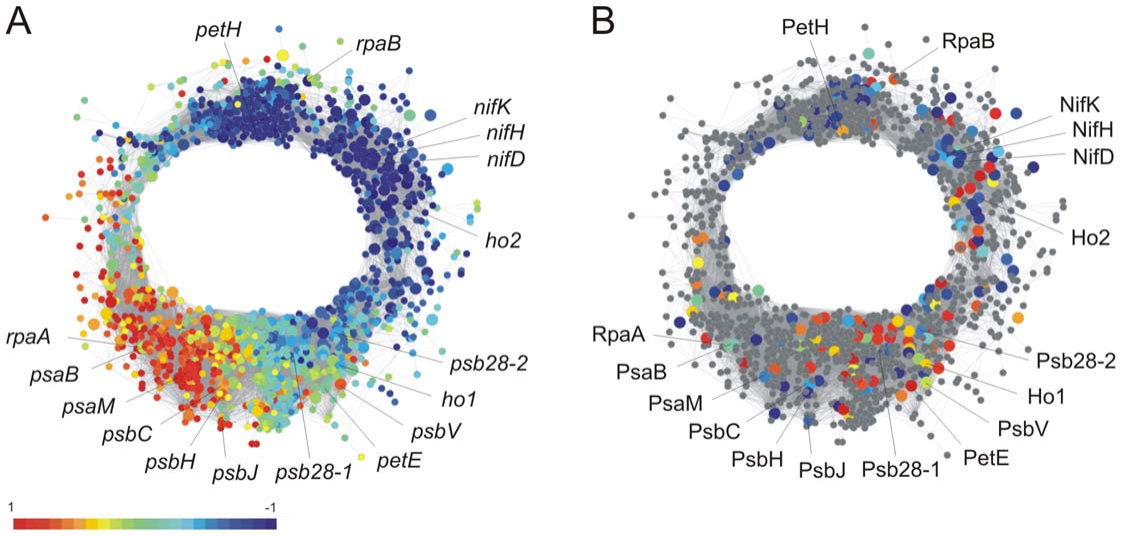
Integration of transcriptomic and proteomic data. (A) Co-expression network of previously obtained transcriptomic data which contains all genes with cyclic mRNA abundance that changed by at least 1.3-fold over the entire time course [5]. The network was visualized using Cytoscape version 2.5.1 [43]. Genes with Pearson correlation coefficients ≥0.9 are connected and each node corresponds to one gene. The genes are colored according to their relative mRNA abundance at time point L5 (five hours in the light cycle). (B) All genes in the co-expression network from panel A for which cyclically expressed proteins were detected are colored according to the relative protein abundance levels at time point L5. Genes without corresponding cyclic proteins are shown in *grey*. Other time points are shown in Figure S5.

A comparison of peak times in mRNA and protein abundances for the 175 cyclic proteins revealed differences depending on the cellular process or functional class (Table S6). For instance, the protein levels for genes involved in nitrogen fixation as well as in biosynthesis of cofactors essentially follow with an average time delay of about 3 hours the mRNA expression levels, suggesting that these genes are largely transcriptionally regulated. In contrast, the peak protein abundances of genes involved in photosynthesis varied greatly from their corresponding transcripts (Figure 4; Tables S4–S6; Figures S4 and S5). In fact, maximal abundance of almost all proteins detected as cyclic from this functional category is during the dark period, whereas the cognate mRNA shows highest peak expression during the light. Such differences in peak times of mRNA and protein abundances could be the result of modified synthesis and/or degradation rates of proteins and cognate mRNA's. Figure 4 highlights this aspect by comparing relative transcript and protein abundances from samples taken after 5 hours in the light cycle. The transcriptional co-expression network that originated from previous transcriptomic analysis [5] revealed a higher transcript abundance of genes involved in processes such as photosynthesis at this time (Figure 4A), whereas the relative abundance of several associated proteins shows the opposite phase (Figure 4B).

#### Protein abundance and biological activities

**Figure 5:**
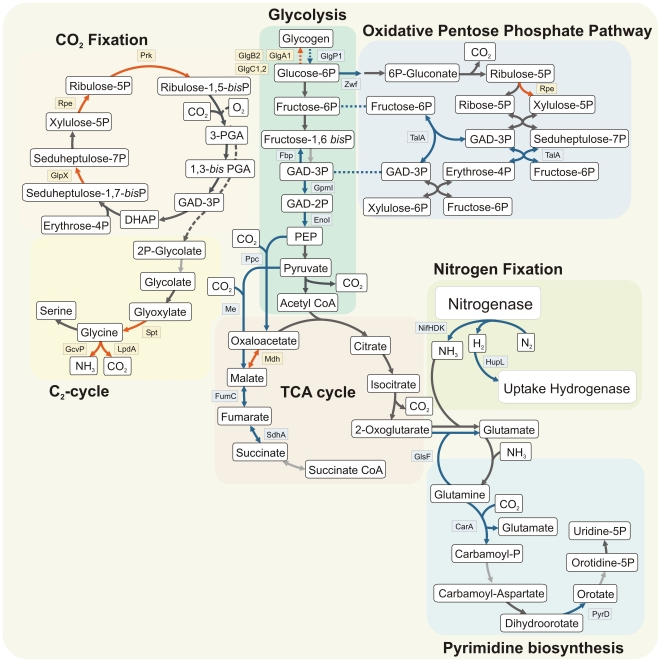
Overview of diurnal changes in the abundance of proteins involved in central metabolic pathways during a circadian cycle. Enzymatic steps involving proteins with maximal abundance during the dark are shown in *blue*, while proteins with peak expression during the light period are represented by *red* arrows. *Dark grey* colored arrows indicate proteins without significant changes in their abundance during a diurnal period while *light grey* colored arrows represent proteins that were not detected. GlgA1: glycogen synthase, GlgB2: 1,4-alpha-glucan branching enzyme, GlgC1,2: glucose-1-phosphate adenylyltransferase, GlgP1: glycogen phosphorylase, Zwf: glucose-6-phosphate dehydrogenase, TalA: transaldolase AB family, Fbp: fructose 1,6 bisphosphatase I, GpmI: 2,3-bisphosphoglycerate-independent phosphoglycerate mutase, EnoI: Enolase, Ppc: Phosphoenolpyruvate carboxylase, Me: malic oxidoreductase, Mdh: Malate dehydrogenase, FumC: fumarate hydratase, SdhA: succinate dehydrogenase subunit A, NifHDK nitrogenase subunits HDK, HupL: uptake hydrogenase large subunit, GlsF: ferredoxin-dependent glutamate synthase, CarA: carbamoyl phosphate synthase, PyrD: dihydroorotate dehydrogenase, Prk: phosphoribulokinase, Rpe: ribulose-phosphate 3-epimerase, GlpX: fructose-1,6-bisphosphatase II, Spt: serine:pyruvate/alanine:glyoxylate aminotransferase, GcvP: glycine carboxylase, LpdA: dihydrolipoamide dehydrogenase, 3-PGA: 3-phosphoglycerate, 1,3-bis PGA: 1,3 *bis*-phosphoglycerate, GAD-3P: glyceraldehyde-3-phosphate, DHAP: dihydroxy acetone phosphate.

Our analysis of the *Cyanothece* 51142 proteome revealed that the abundance of proteins related to central metabolic processes largely follows the expression pattern of their appropriate transcripts, although changes at the protein level are not as tightly regulated. Eight proteins involved in nitrogen fixation are highest during the dark period (Figure 5; Table S4), consistent with an observed peak in nitrogen fixation at this time [13]. In addition, several rate limiting enzymes involved in glycogen degradation, oxidative pentose phosphate pathway (OPP) and glycolysis are also more highly abundant during the dark cycle (Figure 5). One of these enzymes, the glucose-6-phosphate dehydrogenase Zwf (*cce_2536*), plays a critical role in carbon metabolism as it redirects the carbon flow into the OPP and initiates the oxidation of glucose concomitantly with the production of NADPH. The production of NADPH represents an essential catabolic route because it provides reductants for nitrogen fixation and respiration. Thus, a mutation of *zwf* in *N. punctiforme* resulted in loss of the nitrogenase activity [14].

The TCA cycle catalyzes the final steps in carbohydrate oxidation. Interestingly, the higher abundance of the fumarate hydratase subunit FumC (*cce_0396*) and SdhA (*cce_0663*), a subunit of the succinate dehydrogenase complex, during the dark suggests an elevated metabolic demand for TCA-cycle intermediates leading to succinate. This is also supported by the observation that the PEP carboxylase, which catalyzes the fixation of CO_2_ to produce oxaloacetate, and the malic enzyme (*cce_3246*), required for the carboxylation of pyruvate to form malate, are most abundant at this time. Both of these reactions channel glycolytic intermediates to the C4-acid path of the TCA-cycle while recapturing metabolically released CO_2_ and circumventing the conversion of PEP into pyruvate and/or the decarboxylation of pyruvate into acetyl-CoA (Figure 5). Anaplerotic CO_2_ fixation has previously been shown to be important in other unicellular photoautotrophs. Recent studies revealed that 10–15% of the cellular carbon in *Roseobacter denitrificans* is obtained through the activities of PEP carboxylase and malic enzyme [15]. The extent of this anaplerotic CO_2_ fixation is even more apparent in *Synechocystis* sp. PCC 6803, with 25% under mixotrophic conditions [16], and is essential for growth under photoautotrophic conditions in continuous light [17]. Anaplerotic CO_2_ fixation appears to play an important physiological role in *Cyanothece* 51142 in recapturing metabolically released CO_2_ and interconnecting the glycolytic pathway with the C4-acid branch of the TCA-cycle. However, because the succinate dehydrogenase participates in both the TCA-cycle and the respiratory electron transport chain [18], the higher abundance of enzymes covering the reactions to succinate might also be attributed to the peak in respiratory activity observed during the dark period [19]. In fact, different proteins associated with other respiratory complexes such as the NDH-complex, the cytochrome b*_6_*/f complex, the cytochrome c oxidase, and the ATP-synthase complex are detected at higher abundance during the dark (Table S4; Figure S2).

Our data also suggest that the cellular energy requirements during the dark are mainly met via oxidative phosphorylation, as the metabolic reactions leading to malate and oxaloacetate are favored over glycolytic reactions associated with substrate-level phosphorylation (Figure 5). In addition, the higher abundance of fructose-1,6- bisphosphatase I (*cce_4758*), an enzyme involved in gluconeogenesis, implies that the cellular dark metabolism of *Cyanothece* 51142 is not limited by ATP.

In contrast, various enzymes involved in the Calvin-Benson-Bassham cycle, the primary pathway for CO_2_ fixation, are most abundant during the light period (Figure 5). In addition, the protein levels of different enzymes involved in glycogen synthesis such as the 1,4-α-glucan branching enzyme GlgB2 (*cce_4595*) and the glucose-1-phosphate adenylyltransferases GlgC1 (*cce_0987*) and GlgC2 (*cce_2658*) are also maximal during the light phase (Figure 5). The peak abundances of proteins associated with Calvin-Benson-Bassham cycle and glycogen synthesis are in accordance with previous findings from transcriptional and physiological studies [5], [20].

The glycine carboxylase GcvP (*cce_2609*) and dihydrolipoamide dehydrogenase LpdA (*cce_0735*), two proteins of the glycine decarboxylase complex, also exhibit maximum abundance during the light period. The complex participates in the photorespiratory 2-phosphoglycolate metabolism and catalyzes the concomitant oxidative decarboxylation and deamination of glycine to CO_2_, NH_3_, and the transfer of methylene to tetrahydrofolate (THF). Even though it is well established that the low CO_2_ affinity of cyanobacterial Rubisco is compensated by an efficient carbon concentrating mechanism, recent studies showed that the oxygenase function of the Rubisco enzyme leading to 2-phosphoglycolate is essential for cyanobacterial growth [21]. In addition, accumulation of 2-phosphoglycolate seems to initiate the cellular adaptation to low CO_2_ conditions [22].

Notably, plastocyanin (*cce_0590*), the ferredoxin-NADPH oxidoreductase PetH (*cce_0994*), and the extrinsic cytochrome c-550 protein PsbV (*cce_2955*), which is implicated in stabilizing the manganese cluster of the oxygen evolving complex [23], are the proteins related to photosynthesis that are up-regulated during the light period (Table S4; Figure S2). Intriguingly, the electron carrier proteins from both the donor and acceptor side of PSI are up-regulated during the light period, whereas the structural subunits PsaB (*cce_0990*), PsaJ (*cce_1289*), and PsaM (*cce_4273*) of PSI, PsbH (*cce_0860*), a subunit of PSII, and both isoforms of Psb28 (*cce_1599*, *cce_2792*), are highly abundant during the dark period. Earlier studies in *Cyanothece* 51142 showed that the PsaA/B reaction center subunits of PSI are highly up-regulated during the dark period, while the transcript levels are maximal during the day [24]. Even though it is likely that light triggers a more rapid degradation of certain photosynthetic subunits during the day, followed by overall higher protein abundances during the dark period, additional factors are presumably at play to explain the significantly reduced photosynthetic capacity that is observed during this time [25].

The protein Psb28, previously identified as a non-stoichiometric component of PSII complexes in *Synechocystis* sp. PCC 6803 [26], might play an important role in this, since recent studies in *Synechocystis* sp. PCC 6803 suggested that Psb28 is required for the conversion of Mg-protoporphyrin monomethyl ester into protochlorophyllide [27]. In addition, a mutant lacking the protein shows a significantly higher PSII-mediated O_2_ evolution rate attributed to a more efficient PSII repair cycle [27]. Consequently, the Psb28 protein in *Cyanothece* 51142 may maintain the majority of PSII complexes in an intermediary state without properly assembled oxygen evolving complexes. The higher abundance of PsaB protein in *Cyanothece* 51142 during the dark period might be also attributed to elevated chlorophyll biosynthesis during this time, since in addition to Psb28-1 (*cce_1599*) and Psb28-2 (*cce_2792*), the abundance of several proteins involved in this process, including the uroporphyrinogen decarboxylase HemE (*cce_2966*), the Mg-protoporphyrin IX monomethyl ester oxidative cyclases (*cce_4412*), the protochlorophyllide reductase POR (*cce_0320*), and the geranylgeranyl hydrogenase ChlP (*cce_3146*), are increased (Table S4; Figure S2). A correlation between chlorophyll availability and cellular PSI content has been recently documented [28]. Taken together, our data suggest that a combination of transcriptional and post-transcriptional mechanisms controls the abundance of various structural and regulatory proteins involved in photosynthesis in order to modify the photosynthetic capacity and photosynthetic electron transport in *Cyanothece* 51142, thus facilitating the temporal separation of oxygenic photosynthesis and nitrogen fixation. Furthermore, our data suggest that *Cyanothece* 51142 uses the alternating dark phase to sustain and replenish key components required to reinitialize photosynthetic activity at the onset of light.

#### Adaptation to alternating light/dark conditions

The anticipation of impending environmental changes, such as diurnal light/dark rhythms, are well documented at the transcriptional level in *Cyanothece* 51142 [5] and evolved to facilitate temporal separation of various metabolic processes more efficiently. At the protein level, different light sensing proteins show changes in abundance over a diurnal period. For instance, the phytochrome A protein AphA (*cce_1983*) in *Cyanothece* 51142 is most abundant during the dark period. An earlier study revealed that the orthologous gene *cph1* in *Synechocystis* sp. PCC 6803 is significantly up-regulated during the dark, repressed by light, and has been suggested to play a role in adaptation to light/dark and dark/light transitions [29]. Our analysis also revealed that RpaA is highly abundant during the end of the light and beginning of the dark period, whereas RpaB is much more abundant during the light period. The proteins RpaA (*cce_0298*) and RpaB (*cce_4002*) are OmpR-type DNA-binding response regulators that have previously been found to control coupling of phycobilisomes to photosynthetic reaction centers [30]. Later studies uncovered that RpaA interacts as part of a two component system with the histidine kinase SasA that mediates a circadian timing signal from the posttranslational oscillator to the transcriptional machinery [31]. In contrast, RpaB was found to bind to a conserved promoter sequence of *hliB* and various PSI genes under low light conditions [32], [33]. Both HliB homologs in *Cyanothece* 51142 (*cce_0602* and *cce_4826*) are highly abundant at the beginning of each light period, which indicates cellular adaptations in response to light. HliB proteins also function to dissipate excess light energy [34], [35] and as transient carriers for chlorophyll [36]. In addition, the flavoproteins (*cce_3833* and *cce_3835*) are highly abundant during the late light period. Orthologous genes encoding the A-type flavoproteins Flv2 and Flv4 in *Synechocystis* sp. PCC 6803 have been recently suggested to participate in photoprotection of PSII [37]. The identification of diurnally regulated light sensing proteins is notable since external light cues are mediated to keep different metabolic processes in phase with environmental variations and thus convey cellular fitness. In addition, proteins involved in protection against excess light modulate cell physiology and are required to maintain cellular homeostasis.

In summary, organisms such as *Cyanothece* 51142 that separate different metabolic processes temporally during a diurnal cycle are suitable targets to study periodical changes of cellular components. The present study is a comprehensive report on global diurnal alterations of the cellular protein repertoire in *Cyanothece* 51142 and expands upon current knowledge concerning diurnal rhythms. We observed that 250 proteins accounting for ∼5% of the predicted ORFs from the *Cyanothece* 51142 genome and 20% of proteins detected under alternating light/dark conditions show diurnal changes in their abundance during a circadian cycle. The significant contrast between the number of cyclic transcripts and protein has led to the conclusion that even though cyclic protein abundances appear to be essentially transcriptionally regulated, post-translational processes may play a larger role than expected. In addition, variations in the correlation between maximal mRNA expression levels and protein abundances appear to be a pathway and gene category dependent phenomenon that contributes towards regulation of cellular activities such as photosynthesis.

In conclusion, global transcriptomic and proteomic analyses in conjunction with subsequent data integrations are valuable tools for studying different questions in biology and unraveling the complex networks that occur at the cellular level. In addition, ongoing improvements of both proteomic and transcriptomic approaches and technologies, along with a better understanding of post-translational events, will continue to advance the integration of such disparate quantitative values that hold the potential to explain cellular systemic regulation and control.

## Materials and Methods

### Growth conditions

Cells were routinely grown under continuous light conditions (50 µmol photons m^−2^ s^−1^ at 30°C) in ASP_2_ medium [13] with air bubbling. For nutrient starvation experiments *Cyanothece* 51142 cultures were grown in ASP_2_ media without added sodium chloride or iron for seven days. Iron recovery was initiated by adding 14.4 µM iron(III)chloride to a six day old iron starved *Cyanothece* 51142 culture and harvested after 24 hours. The samples for quantitative proteome analysis were derived from the same set of *Cyanothece* 51142 cultures that were previously used for global transcriptional analysis [5], with additional time resolution (every 2 hours) over the subset of samples previously profiled (every 4 hours). The cultures were grown for six days under nitrogen fixing conditions in 12 hours light/dark cycles prior to the start of sample collection. Samples were then harvested every 2 hours, starting one hour into the dark period, over two consecutive diurnal cycles.

### Sample preparation

The cultures were harvested by centrifugation at 5,000 x *g* at ambient temperature and washed 3 times in 100 mM ammonium bicarbonate buffer (pH 8.0). The cells were broken by two passages through a French Press at 20,000 psi and unbroken cells and cell debris were removed by centrifugation at 3,000 x *g* at 4°C. The cell lysates were fractionated by centrifugation at 150,000 x *g* at 4°C for 20 min and the membrane fractions were washed with 100 mM ammonium bicarbonate buffer (pH 8.0) and centrifuged again. The protein concentrations of soluble and insoluble fractions were determined using a bicinchoninic acid (BCA) assay (Pierce, Rockford, IL, USA) and stored at −80°C. Soluble proteins were denatured and reduced using 7 M urea, 2 M Thiourea and 5 mM Dithiothreitol (DTT) (Sigma-Aldrich, Saint Louis, MO, USA) at 60°C for 30 min. The insoluble fraction was treated identically in addition to sonication for 5 min prior to incubation. All samples were diluted 10 times in ammonium bicarbonate (soluble fraction in 100 mM solution and insoluble fraction in 50 mM solution) prior to tryptic digestion performed for 3 hours at 37°C with 1∶50 (w/w) trypsin-to-protein ratio. Desalting of digested samples was achieved by applying the SPE method. The peptides were purified using 1 mL SPE C18 columns (Discovery DSC-18, SUPELCO, Bellefonte, PA, USA), eluted with 80% acetonitrile and 0.1% trifluoroacetic acid and concentrated using a Speed-Vac SC 250 Express (Thermo Savant, Holbrook, NY, USA). A BCA assay was performed to determine the final peptide concentrations. For samples subjected to strong cation exchange chromatography (SCX), a PolySulfoethyl A, 200 mm×2.1 mm, 5 µM, 300-Å column with 10 mm×2.1 mm guard column (PolyLC, Inc., Columbia, MD, USA) with a flow rate of 0.2 mL/min was used. The SCX peptide fractionation step has been previously described [38], [39]. The peptides were resuspended in 900 µL of mobile phase A, and separated on an Agilent 1100 system (Agilent, Palo Alto, CA, USA) equipped with a quaternary pump, degasser, diode array detector, Peltier-cooled autosampler and fraction collector (both set at 4°C). Depending on the sample, a total of either 25 fractions (for a single time point analysis of total membrane, thylakoid membrane, and soluble protein samples) or 4 to 6 fractions (for insoluble and soluble proteins prepared from time point samples taken across the entire diurnal period) were collected.

### Reversed phase LC separation and MS/MS analysis of peptides

All samples, regardless of pre-MS separation, were subjected to the same LC-MS/MS analysis. This method coupled a constant pressure (5,000 psi) reversed phase capillary liquid chromatography system (150 µm i.d.×360 µm o.d.×65 cm capillary; Polymicro Technologies Inc., Phoenix, AZ, USA) at an approximate flow-rate of 400 nL/min, with a Finnigan LTQ ion trap mass spectrometer (ThermoFinnigan, San Jose, CA, USA) and an electrospray ionization source manufactured in-house and extensively reported previously [40]. All unfractionated samples and SCX fractions were analyzed *via* capillary RPLC-MS/MS. The instrument was operated in data-dependent mode with an m/z range of 400–2000. The ten most abundant ions from MS analysis were selected for further MS/MS analysis using a normalized collision energy setting of 35%. A dynamic exclusion of 1 min was applied to reduce repetitive analysis of the same abundant precursor ion.

### LC-MS/MS data analysis

ExtractMSn (version 4.0) and SEQUEST analysis software (Version v.27, Rev 12, Thermo Fisher Scientific, Waltham MA, USA) was used to match all MS/MS fragmentation spectra to sequences from the final annotation of the *Cyanothece* 51142 proteome downloaded from NCBI [6] which contains a total of 5304 protein entries. Search was performed using default parameters with no-enzyme rules within a +/−1.5 Da parent mass window, +/−0.5 fragment mass window, average parent mass, and monoisotopic fragment mass. The criteria selected for filtering are based on a method that utilizes a reverse database false positive model which has a ∼95% confidence over an entire protein dataset [41]. Specific filter criteria for this study to achieve this level of confidence include DelCN ≥0.1 coupled with Xcorr of ≥1.6 for full tryptic charge state +1, ≥2.4 for charge state +2, and ≥3.2 for charge state +3. For partial tryptic, Xcorr ≥4.3 for charge state +2 and ≥4.7 for charge state +3.

### Protein Rollup, quantitative cycle detection, statistical analyses, and integration of transcriptomic and proteomic data

Based on their sequences, the detected peptides were mapped onto their corresponding proteins (Tables S1, S7; Figure S6). If a sequence was non-unique to a single protein, it was counted as corresponding to each of the matching proteins. Spectral data for both membrane and soluble fractions, measured in duplicate, resulted in four count values per peptide for each time point. The protein abundance was determined by summing all observed peptides for each protein. The count values for proteins from soluble and membrane fractions were summed together for each of the four combinations of membrane plus soluble replicates (m1+s1, m1+s2, m2+s1, m2+s2) and their average and standard deviation calculated. These averaged abundance values were then used for the cycle detection.

A combination of trigonometric curve fitting, autocorrelation, and a Fourier Score based method was applied. Trigonometric curve fitting was performed using seven different trigonometric equations [10]. Autocorrelation analysis was utilized to calculate the Pearson correlation between all points *i* and *i*+*period*, from which optimal period and phases were minimized [10]. The cyclic protein levels were first assigned using autocorrelation fit cutoffs of r^2^≥0.5 and periods from 20 to 28 hours. Additional cyclic proteins were then added for autocorrelation fit cutoffs of r^2^≥0.25 for which the trigonometric fits corresponded to r^2^≥0.5 and for which both methods yielded periods within 20 to 28 hours. The Fourier score based method, using a reference sine/cosine function with a 24 hour period, was applied to calculate the Fourier scores of the original expression pattern as well as 10,000 randomly generated protein profiles. Using the Fourier score based method, a protein was classified as cyclic if the Fourier score for the random protein profiles, generated using different permutations of the original profile, exceeded the Fourier score for the original expression in less than 1% of the permutations (p-value <1%) [11]. Genes identified using this approach represented an expected false discovery rate of 0.5% [42]. The two cycle detection algorithms identified 166 different proteins with diurnal expression patterns.

Additional diurnally regulated proteins were detected by comparing protein abundances from time points that are separated by 12 hours or by analyzing the total spectral counts during dark and light periods. Protein abundance levels are classified as being significantly different if: (a) the difference between spectral counts is >1, (b) the fold-change between spectral counts is >1.5 and (c) the p-value computed using a two-sample t-test is <5%. These criteria must be satisfied for both 24 hour periods separately. Based on this criterion, 84 additional proteins with significant changes in their abundance between light and dark periods were identified. With the exception of six proteins, all proteins showed more than two fold abundance variation during a 24 hour period. The lists of proteins detected by each method were merged into the final set of 250 proteins with cyclic abundance levels.

The time difference between the maxima of mRNA and protein expression profiles was calculated for all genes with diurnal oscillations. For that purpose, each expression profile was approximated using the first oscillatory term of the Fourier series expansion. The time difference between two oscillations was computed as the phase difference of the approximated signals.

A co-expression network that contains all diurnally expressed genes with at least a 1.3-fold change between their maximum and minimum mRNA abundance has been generated previously [5] and is shown in Figure 4A. In order to identify the phase of their oscillations, each gene expression was mean deducted and scaled to values ranging between +1 and −1. In Figure 4B, in the same network, the relative abundance levels for proteins identified as cyclic are colored according to their relative abundance levels. Similar to the mRNA levels, the protein abundances are mean deducted and scaled between −1 and +1, and their relative expression levels are visible from the color of the node. Genes which were not detected as cyclic at the protein level are shown in *grey*.

## Supporting Information

Figure S1 **Overview of sample collection and preparation.** The cultures were grown under nitrogen fixing conditions and samples were collected over two consecutive diurnal periods. Each sample fraction was analyzed via LC-MS/MS. Out of a total of 100 LC-MS/MS analyses, 1232 proteins were identified.
(TIF)

Figure S2 **Peak time distribution of cyclic expressed proteins during a diurnal cycle.** The circle defines a polar coordinate plot with radians transformed into hours.
(TIF)

Figure S3 **Abundance values for all cyclic expressed proteins.** The abundance value corresponds to the total number of spectral counts for the most abundant peptide from each protein.
(TIF)

Figure S4 **Expression values for different mRNAs and corresponding proteins.** Relative expression values for (A) *nifH*, (B) *nifD*, (C) *nifK*, (D) *psaB*, and (E) *psaM* mRNA's and corresponding proteins involved in nitrogen fixation or photosynthesis are shown over the entire 48 hour time course.
(PDF)

Figure S5 **Integration of transcriptomic and proteomic data.** Co-expression network of previously obtained transcriptomic data which contains all genes with cyclic mRNA abundance that changed by at least 1.3-fold over the entire time course [5]. The genes are colored according to their relative mRNA abundance at different time points (left panel). All genes in the co-expression network for which cyclically expressed proteins were detected are colored according to the relative protein abundance levels at each time point (right panel). Genes without corresponding cyclic proteins are colored in *grey*. Shown are data for the time points D1, D5, D9, L1, L5, and L9 for the first 24 hours of the time course experiment.
(PDF)

Figure S6 **MS/MS spectra for all single-peptide-based protein identifications.** The spectra correspond to the proteins entries in Table S7.
(PDF)

Table S1 **Total number of detected peptides.** The table contains the protein identifications, the protein annotation settings, and the pre-MS separations parameters for all LC-MS/MS analyses. A total of 41,469 unique peptides were identified which resulted after redundant mapping across all protein identifiers in a total of 41,959 entries.
(XLS)

Table S2 **Total number of detected proteins.** The peptides from Table S1 resulted in 3,616 protein identifications. For proteins identified based on only one peptide identification, individual peptides and spectra information are provided in Figure S6 and Table S7.
(XLS)

Table S3 **Subset of identified peptides detected during the 48 hour light / dark time course.** The time points are labeled as D1, D3, D5, D7, D9, and D11 for 1, 3, 5, 7, 9, and 11 hours in the dark, and L1, L3, L5, L7, L9, and L11 for 1, 3, 5, 7, 9, and 11 hours in the light. A total of 6,848 unique peptides were utilized and mapped across 1,232 proteins. The subsequent protein roll-up, quantitative cycle detection, and statistical analysis are described in detail in the Experimental Methods section, with final results shown in Table S4.
(XLS)

Table S4 **Final normalized spectral counts for all proteins with cyclic changes in their abundance during the 48 hour light / dark time course.** The time points are labeled as D1, D3, D5, D7, D9, D11 for 1, 3, 5, 7, 9, and 11 hours in the dark, and L1, L3, L5, L7, L9, L11 for 1, 3, 5, 7, 9, and 11 hours in the light.
(XLS)

Table S5 **Means, standard deviations, intensity values and fold-changes for all proteins detected as cyclic expressed over two consecutive diurnal periods.** Spectral count data derived from two technical replicates for both membrane and soluble fractions and resulted in four count values per peptide for each time point. The protein abundance was determined by summing all observed peptides for each protein. These averaged abundance values were used for the cycle detection. The intensity values and fold-changes are given for both days separately. For a number of proteins the fold-changes are undefined (n.d.) because their minimum spectral count values are equivalent to zero. The time points are labeled as D1, D3, D5, D7, D9, and D11 for 1, 3, 5, 7, 9, and 11 hours in the dark, and L1, L3, L5, L7, L9, and L11 for 1, 3, 5, 7, 9, and 11 hours in the light.
(XLS)

Table S6 **Peak time maxima for mRNA and protein expression profiles with diurnal oscillation pattern.** Each expression profile was approximated using the first oscillatory term of the Fourier series expansion. The fold-changes between maximum and minimum intensities for mRNA's and proteins are given for each day separately. For a number of proteins the fold-changes are undefined (n.d.), because their minimum spectral count values are equivalent to zero.
(XLS)

Table S7 **Single-peptide-based protein identifications.** The total number of single-peptide-based protein identifications including accession number, protein annotation settings, and pre-MS separations parameters for the LC-MS/MS analyses. The corresponding MS/MS spectra are shown in identical order in Figure S6.
(XLS)
