# Supplementary figures and images for: Diurnal Rhythms Result in Significant Changes in the Cellular Protein Complement in the Cyanobacterium Cyanothece 51142
Source: PLoS One. 2011 Feb 22;6(2):e16680. doi: 10.1371/journal.pone.0016680 (PMC3043056; doi:10.1371/journal.pone.0016680)

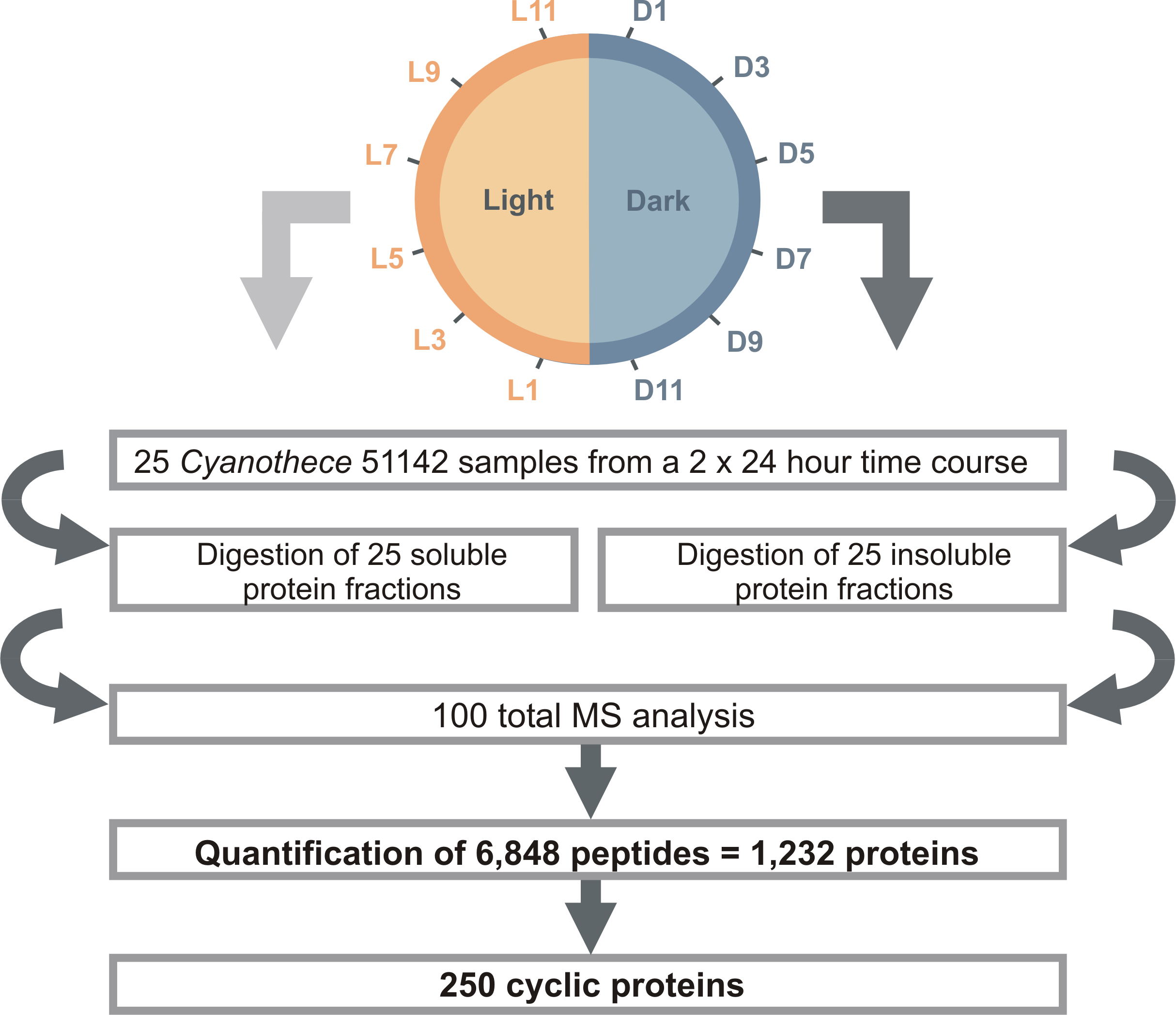

Supplement: Figure S1 — Overview of sample collection and preparation. The cultures were grown under nitrogen fixing conditions and samples were collected over two consecutive diurnal periods. Each sample fraction was analyzed via LC-MS/MS. Out of a total of 100 LC-MS/MS analyses, 1232 proteins were identified. (TIF) [file pone.0016680.s001.tif]

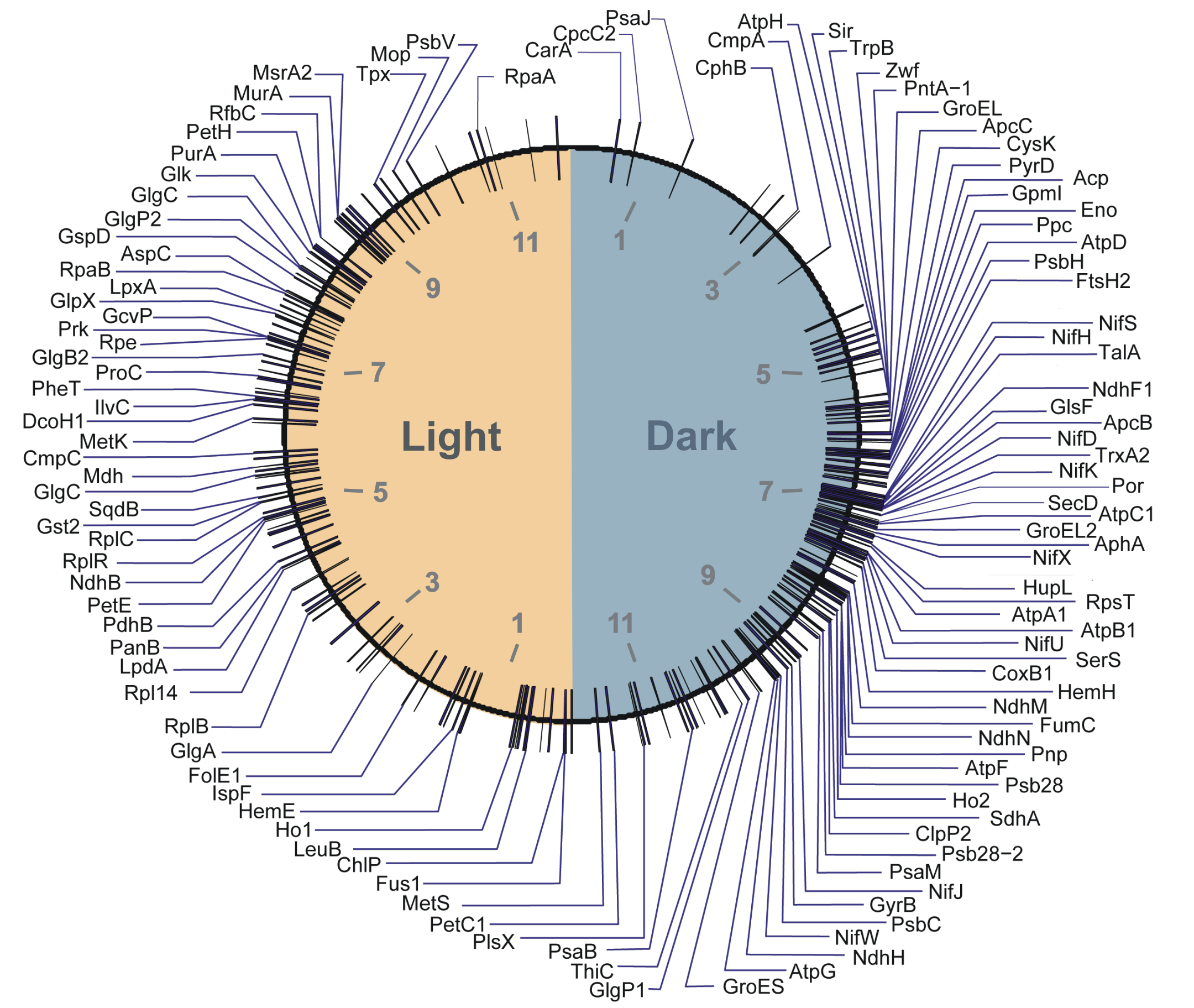

Supplement: Figure S2 — Peak time distribution of cyclic expressed proteins during a diurnal cycle. The circle defines a polar coordinate plot with radians transformed into hours. (TIF) [file pone.0016680.s002.tif]

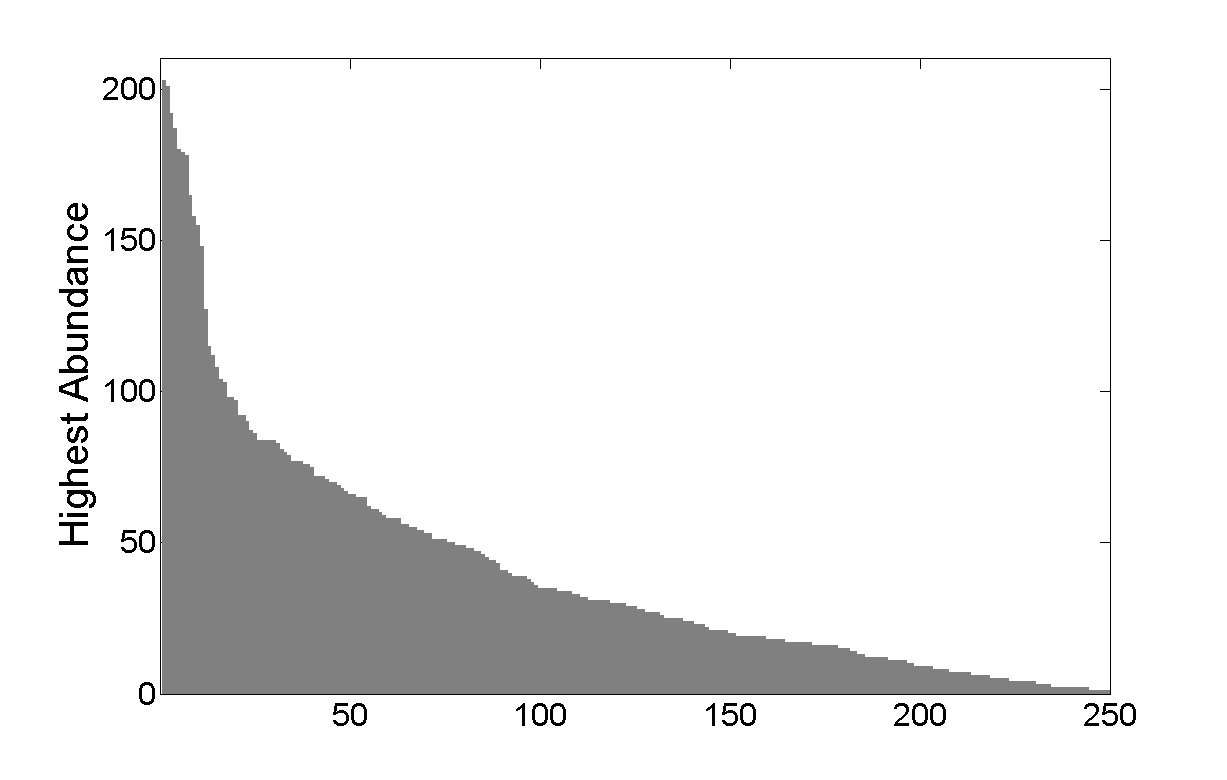

Supplement: Figure S3 — Abundance values for all cyclic expressed proteins. The abundance value corresponds to the total number of spectral counts for the most abundant peptide from each protein. (TIF) [file pone.0016680.s003.tif]

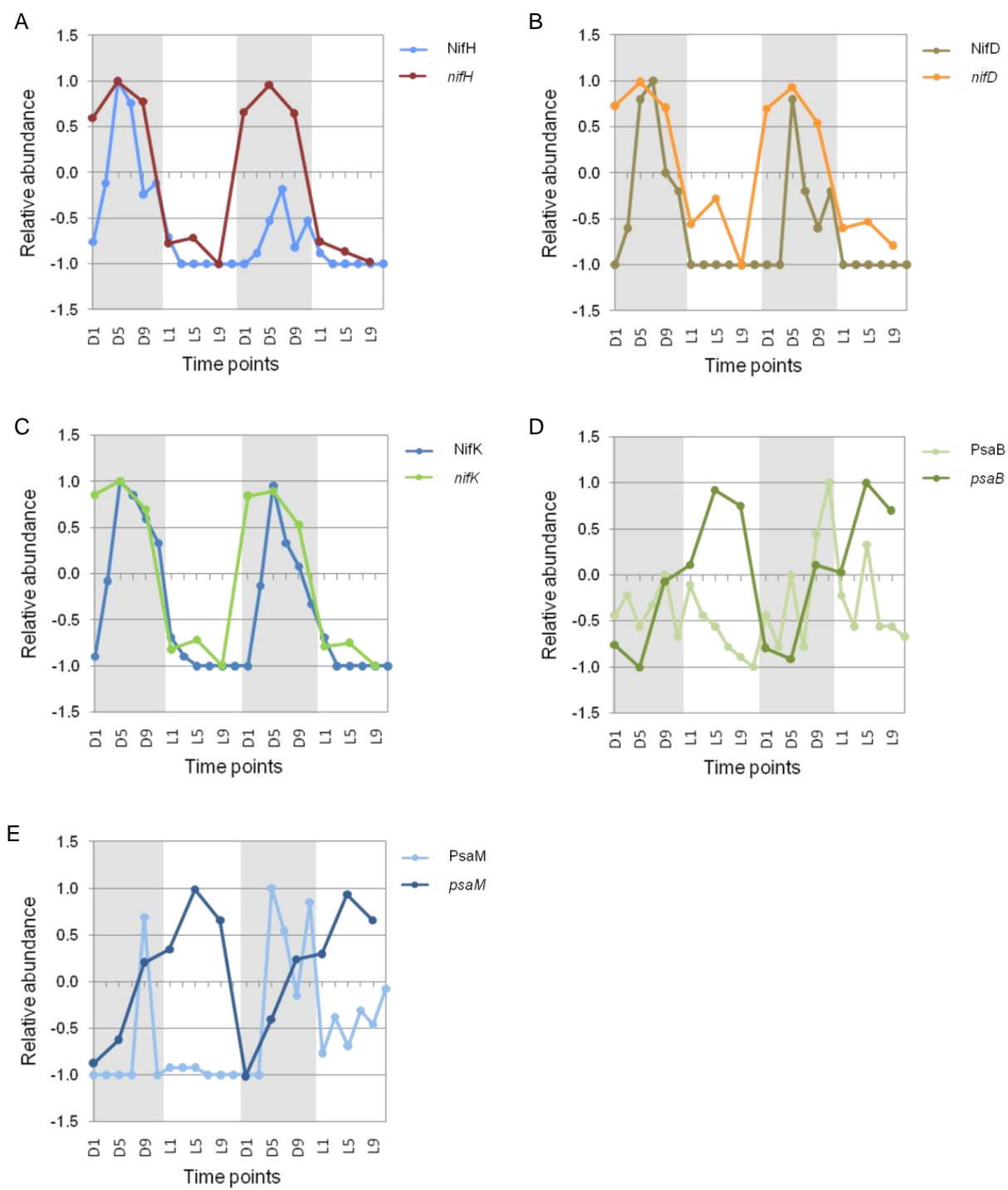

Figure S4

Supplement: Figure S4 — Expression values for different mRNAs and corresponding proteins. Relative expression values for (A) nifH, (B) nifD, (C) nifK, (D) psaB, and (E) psaM mRNA's and corresponding proteins involved in nitrogen fixation or photosynthesis are shown over the entire 48 hour time course. (PDF) [file pone.0016680.s004.pdf]

D1

Transcripts

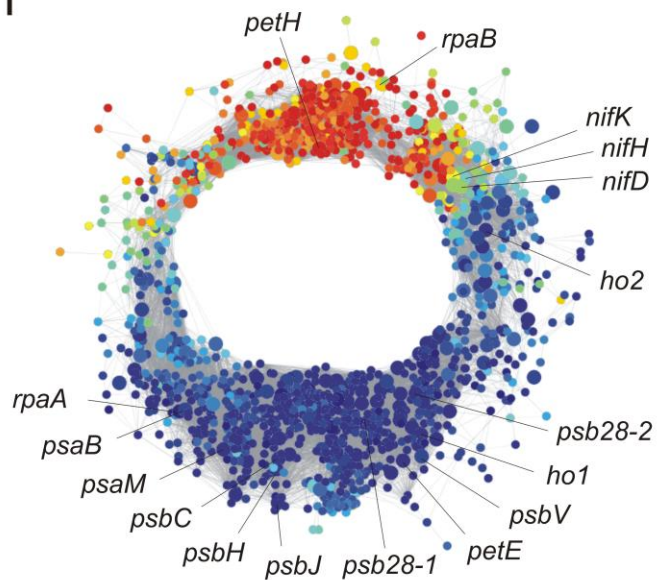

Proteins

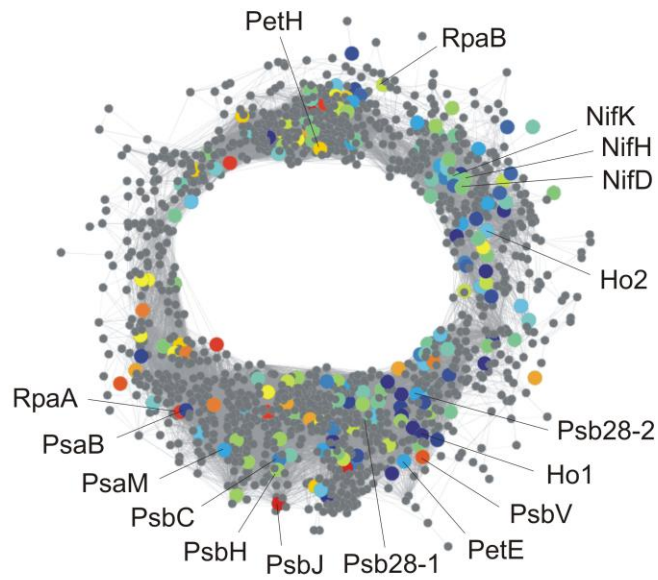

D5

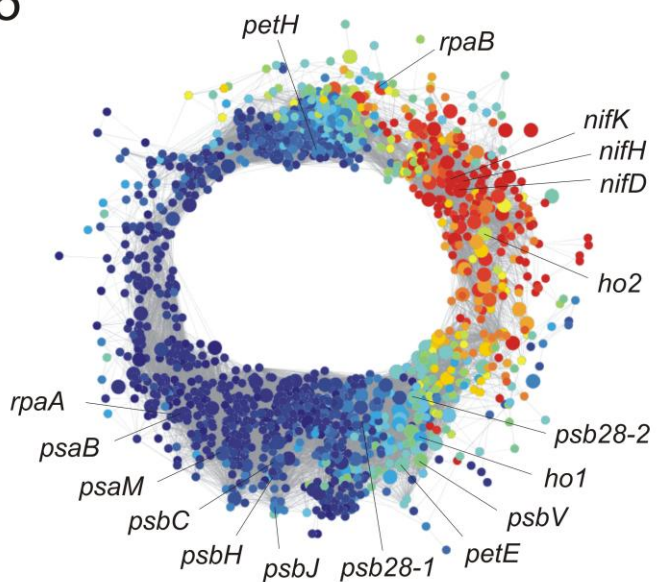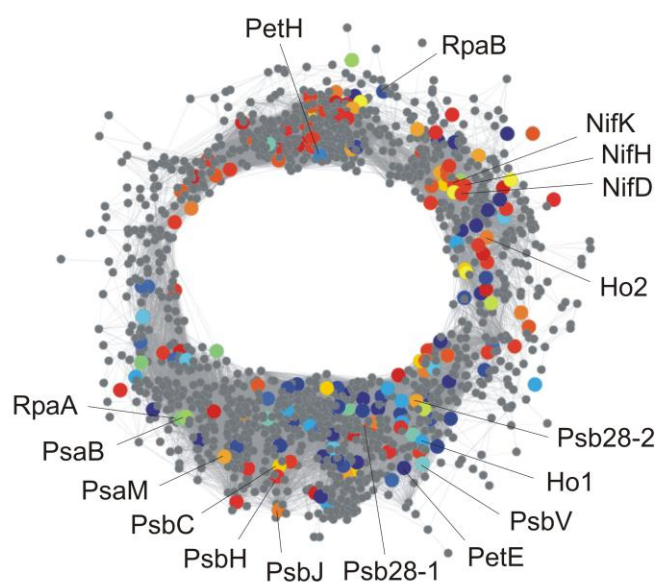

D9

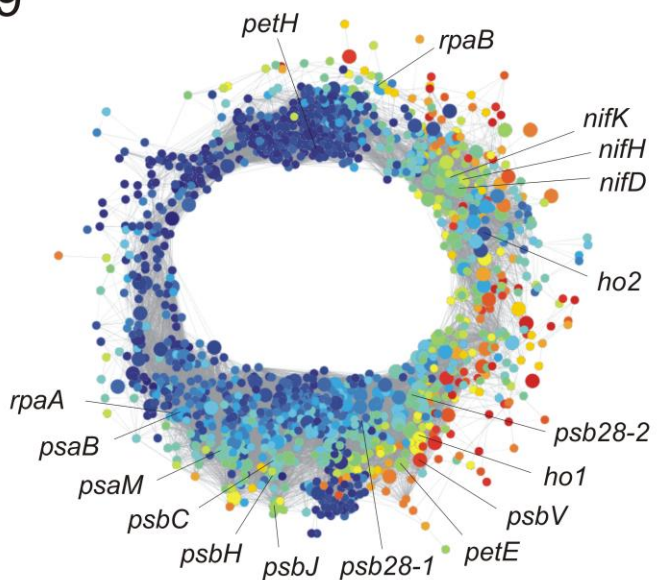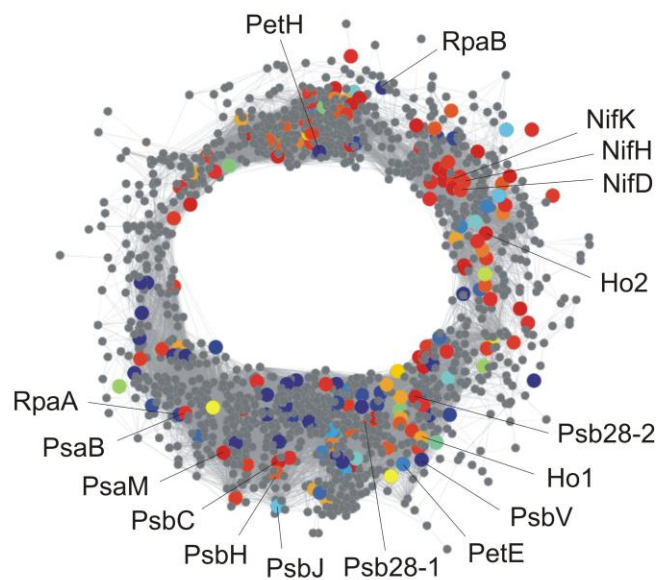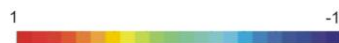

L1

Transcripts

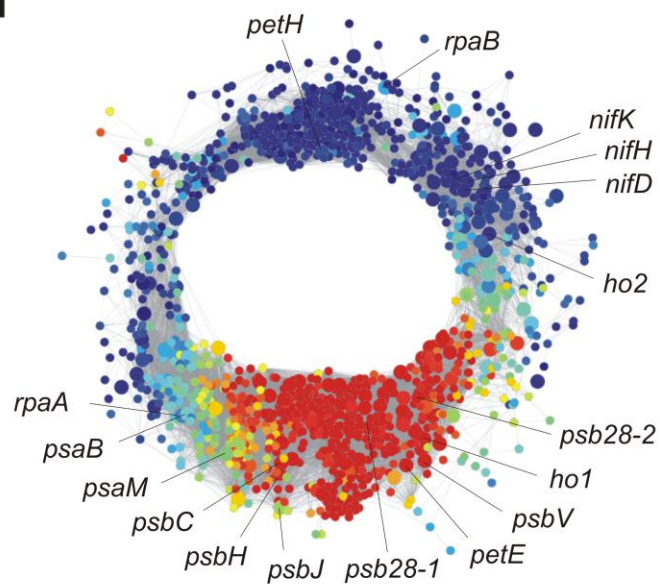

Proteins

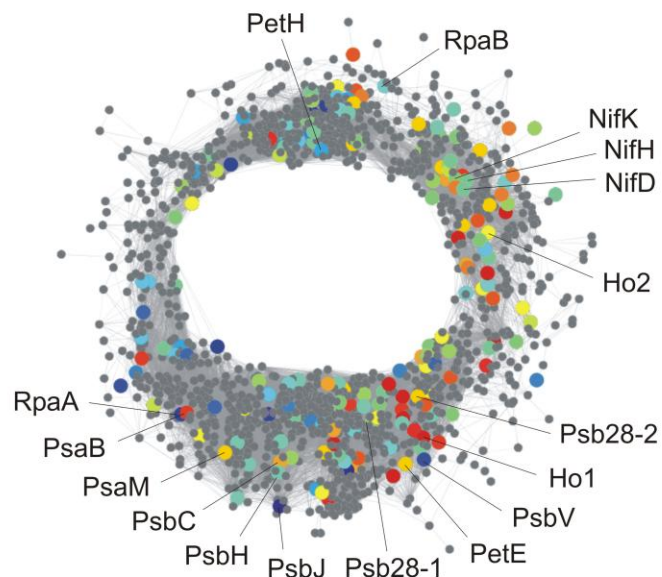

L5

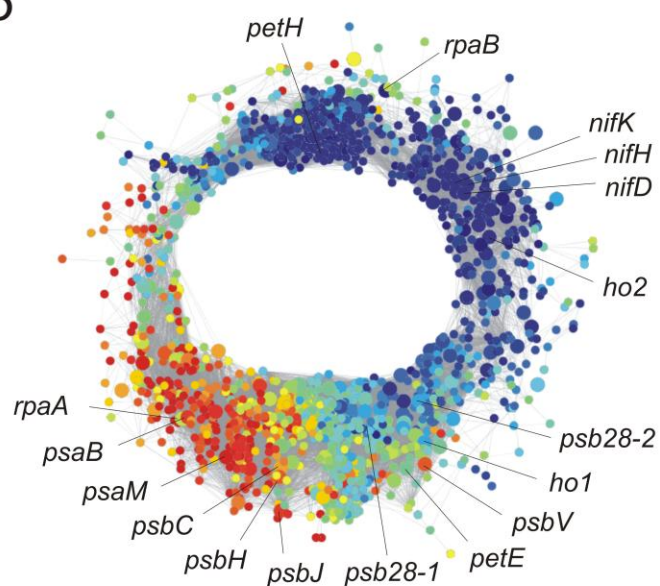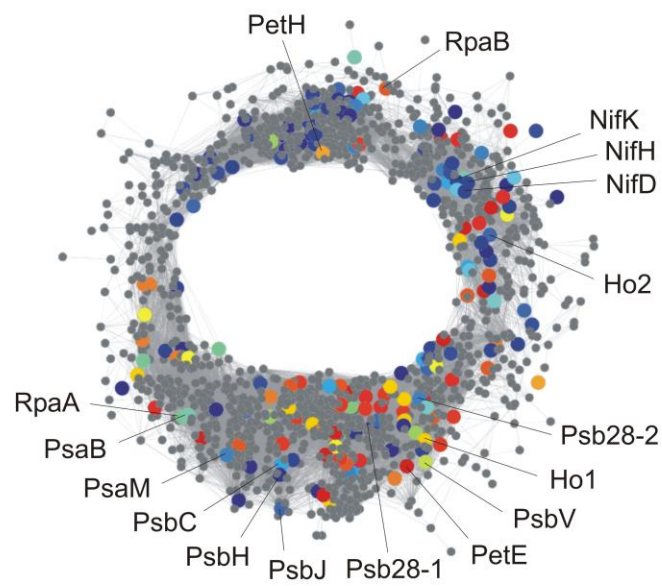

L9

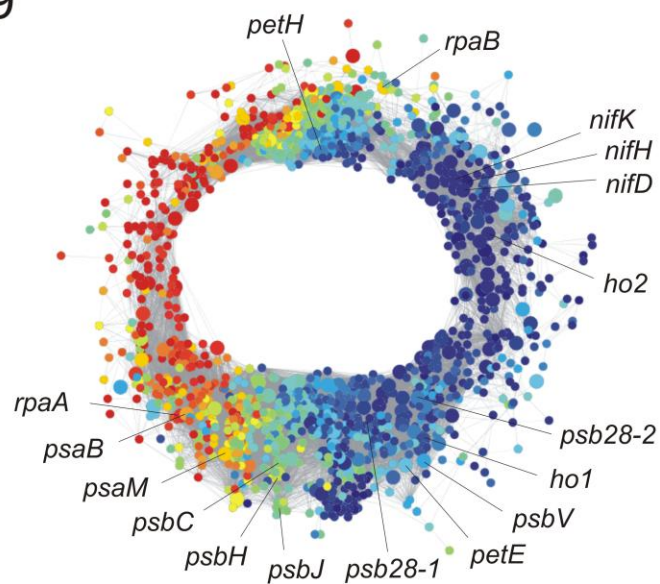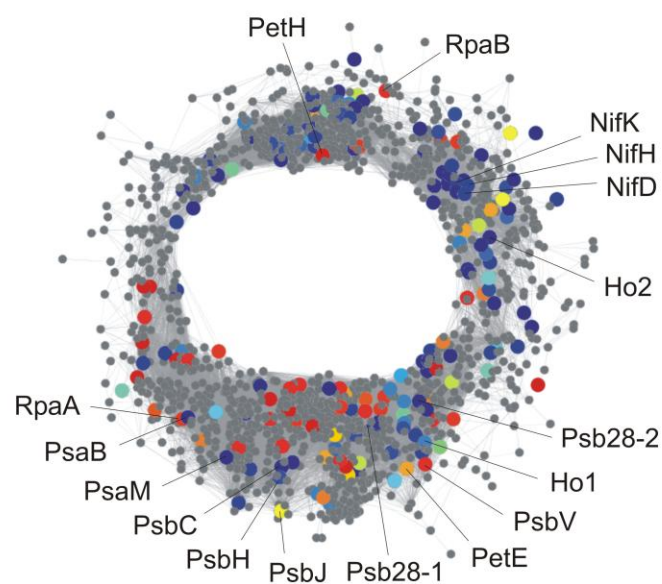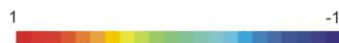

Figure S5

Supplement: Figure S5 — Integration of transcriptomic and proteomic data. Co-expression network of previously obtained transcriptomic data which contains all genes with cyclic mRNA abundance that changed by at least 1.3-fold over the entire time course [5]. The genes are colored according to their relative mRNA abundance at different time points (left panel). All genes in the co-expression network for which cyclically expressed proteins were detected are colored according to the relative protein abundance levels at each time point (right panel). Genes without corresponding cyclic proteins are colored in grey. Shown are data for the time points D1, D5, D9, L1, L5, and L9 for the first 24 hours of the time course experiment. (PDF) [file pone.0016680.s005.pdf]
