# Supplementary material for: Diurnal Rhythms Result in Significant Changes in the Cellular Protein Complement in the Cyanobacterium Cyanothece 51142
Source: PLoS One. 2011 Feb 22;6(2):e16680. doi: 10.1371/journal.pone.0016680 (PMC3043056; doi:10.1371/journal.pone.0016680)
Supplement: Figure S6 — MS/MS spectra for all single-peptide-based protein identifications. The spectra correspond to the proteins entries in Table S7. (PDF) [file pone.0016680.s006.pdf]

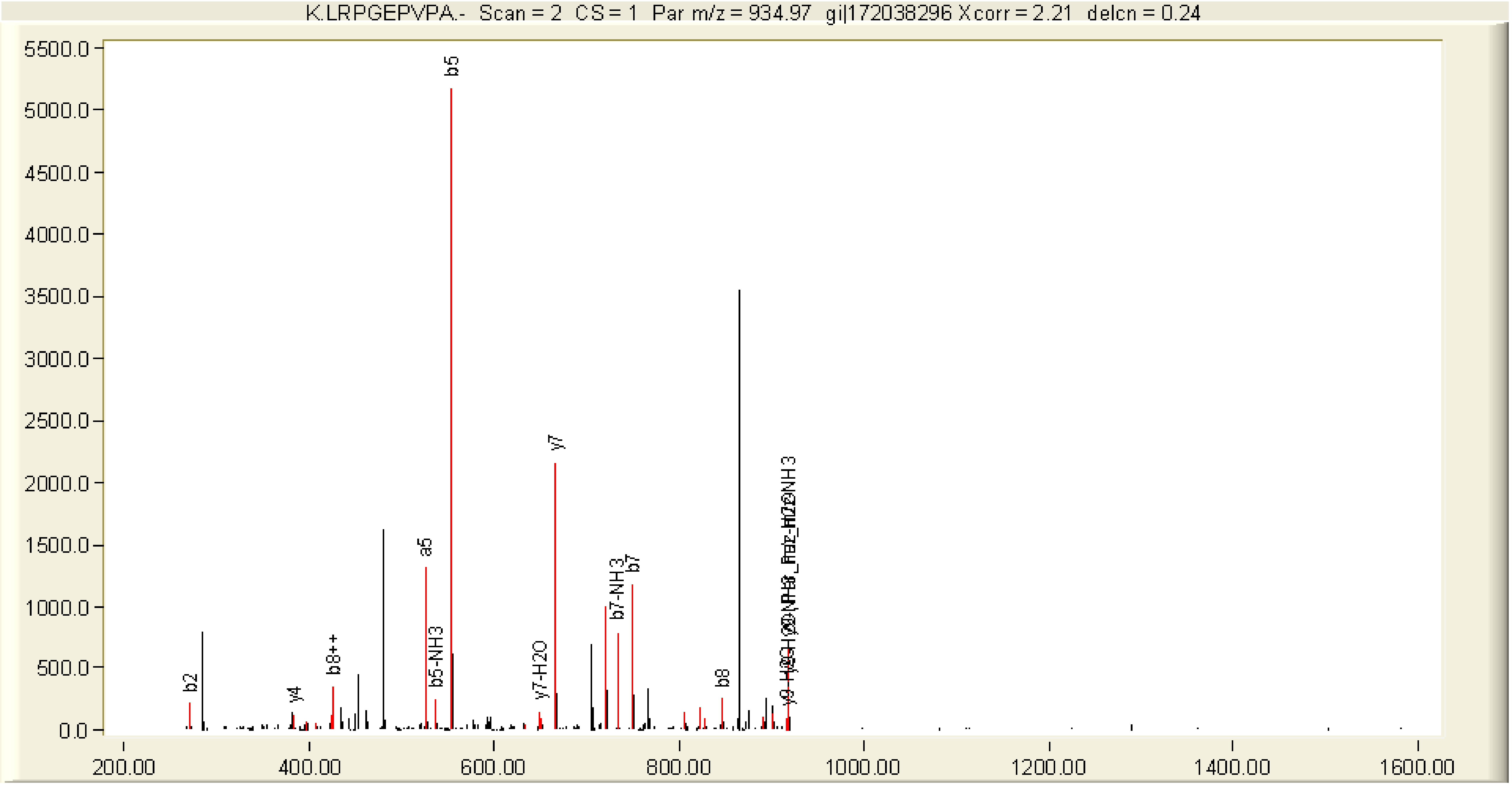

K.LRPGEPVPA.- Scan = 2 CS = 1 Par m/z = 934.97 gi|172038296 Xcorr = 2.21 delcn = 0.24
5500.0
5000.0
4500.0
4000.0
3500.0
3000.0
2500.0
2000.0
1500.0
1000.0
500.0
0.0
200.00
400.00
600.00
800.00
1000.00
1200.00
1400.00
1600.00
b2
y4
b8++
a5
b5-NH3
b5
y7-H2O
y7
b7-NH3
b7
b8

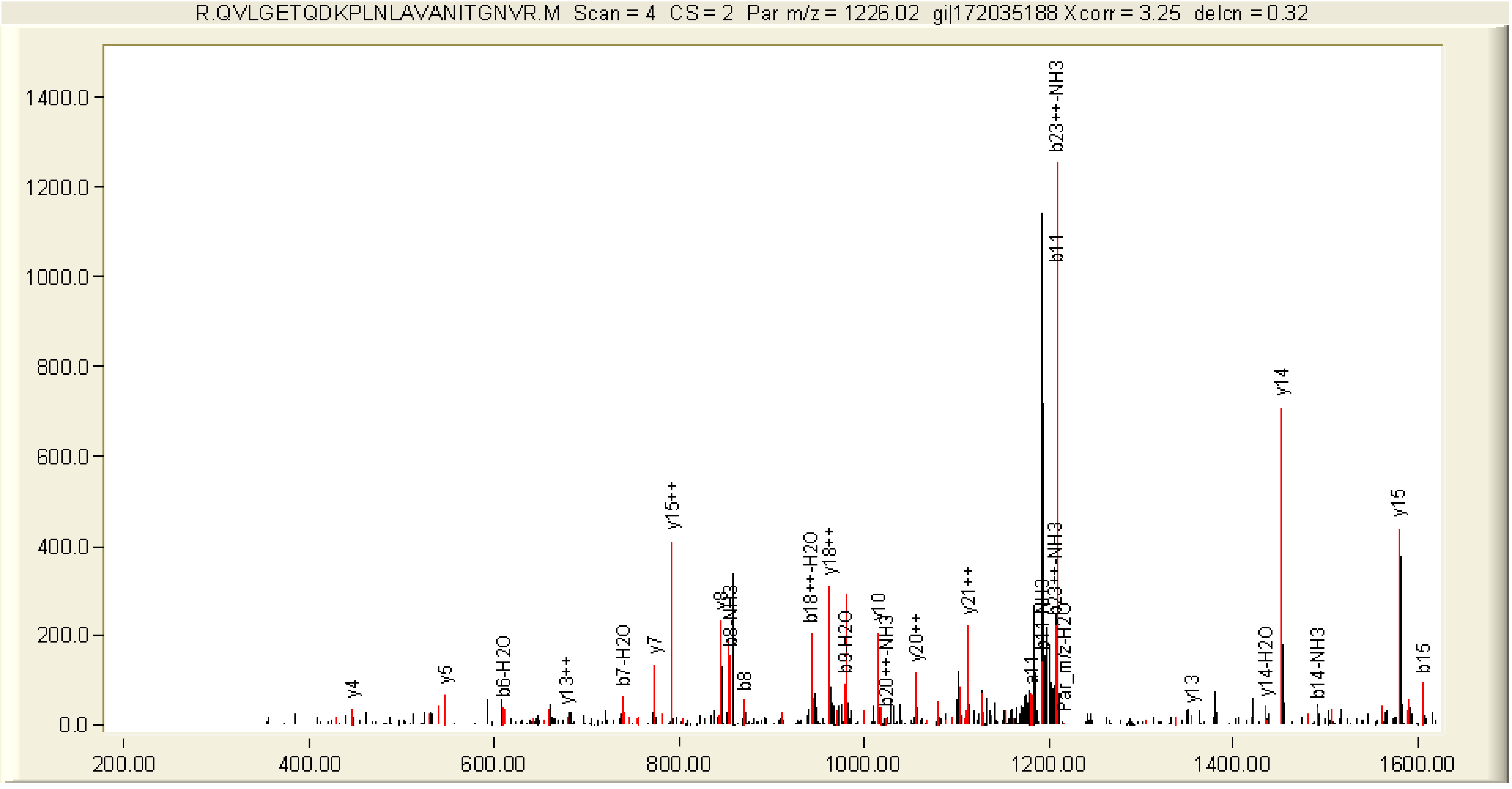
R.QVLGETQDKPLNLAVANITGNVR.M Scan = 4 CS = 2 Par m/z = 1226.02 gi|172035188 Xcorr = 3.25 delcn = 0.32
1400.0
1200.0
1000.0
800.0
600.0
400.0
200.0
0.0
200.00
400.00
600.00
800.00
1000.00
1200.00
1400.00
1600.00
y4
y5
b6-H2O
y13++
b7-H2O
y7
y15++
b8-NH3
b8
b18++-H2O
y18++
b9-H2O
y10
b20++-NH3
y20++
y21++
b23++-NH3
b11
Par_m/z-H2O
y13
y14-H2O
y14
b14-NH3
y15
b15

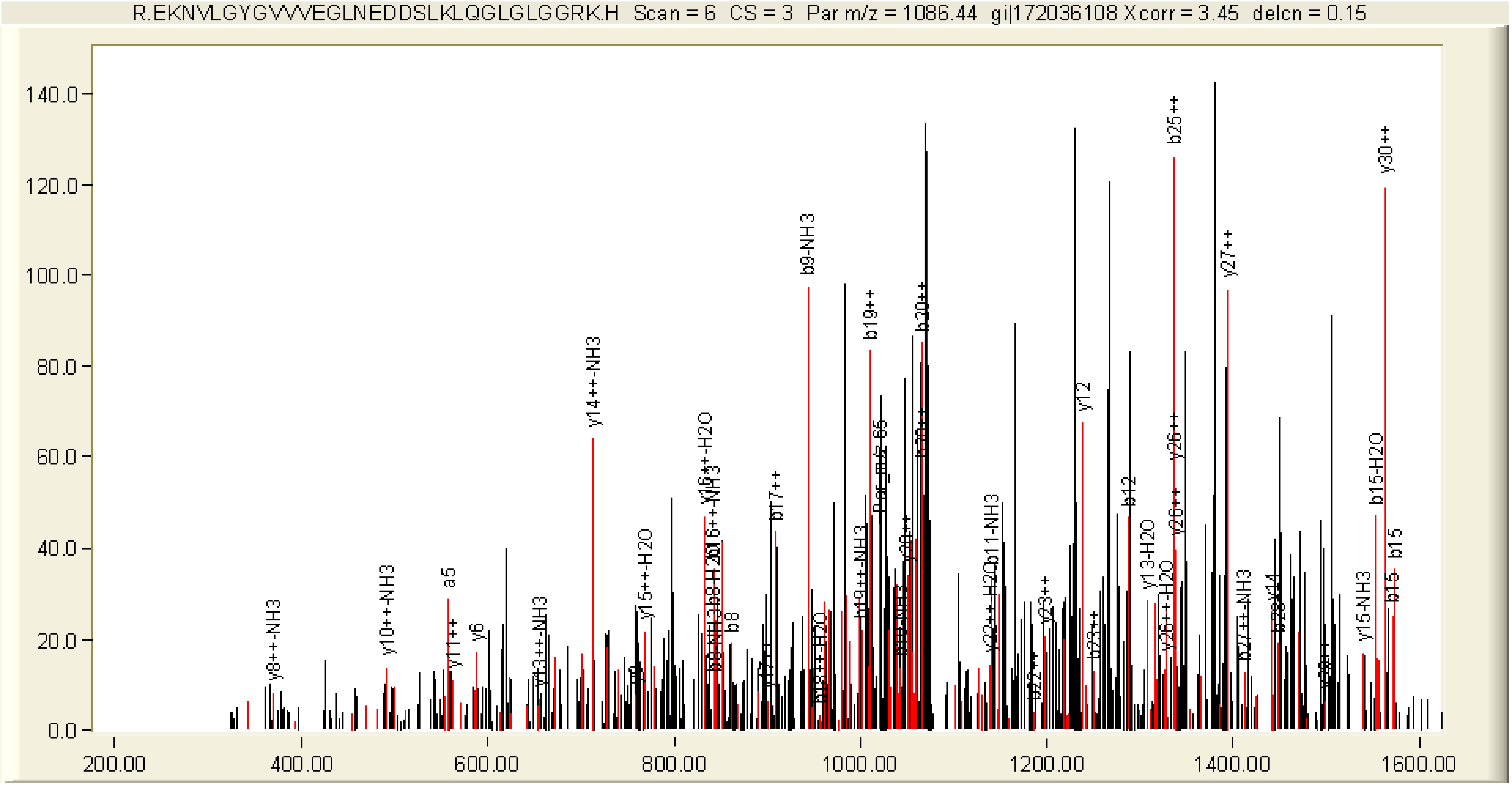
R.EKNVLGYGVVVEGLNEDDSLKLQGLGLGGRK.H Scan = 6 CS = 3 Par m/z = 1086.44 gi|172036108 Xcorr = 3.45 delcn = 0.15
140.0
120.0
100.0
80.0
60.0
40.0
20.0
0.0
200.00
400.00
600.00
800.00
1000.00
1200.00
1400.00
1600.00
y8++-NH3
y10++-NH3
a5
y11++
y6
y13++-NH3
y14++-NH3
y15++-H2O
b8
b9-NH3
b19++
b17++
y12
b11-NH3
b12
y13-H2O
b25++
y26++
y27++
y30++
b15-H2O
b15
y15-NH3
y23++

R.GVEPFFLAISQTNFLAAIK.L Scan = 8 CS = 2 Par m/z = 1033.57 gi|172038740 Xcorr = 2.51 delcn = 0.12

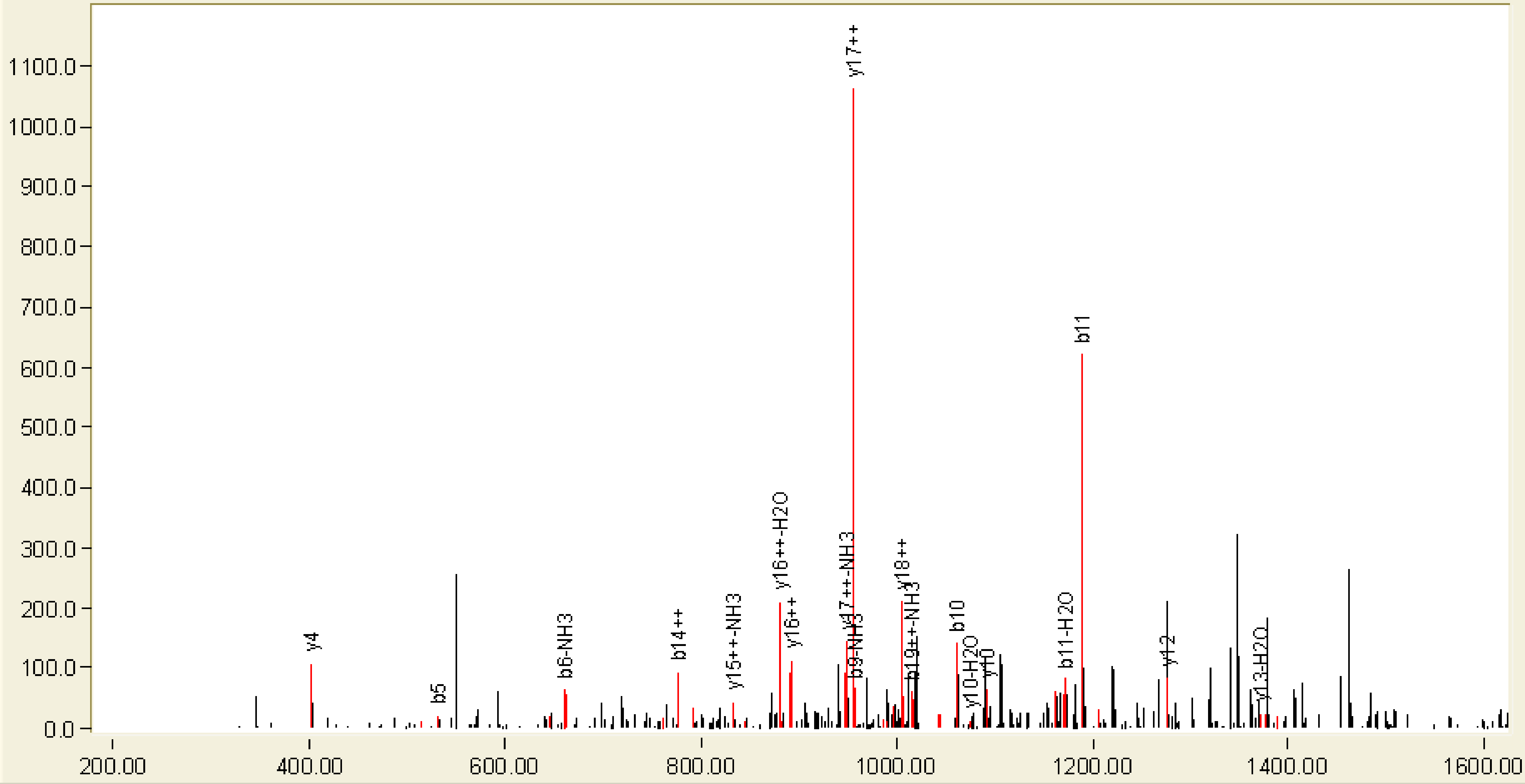

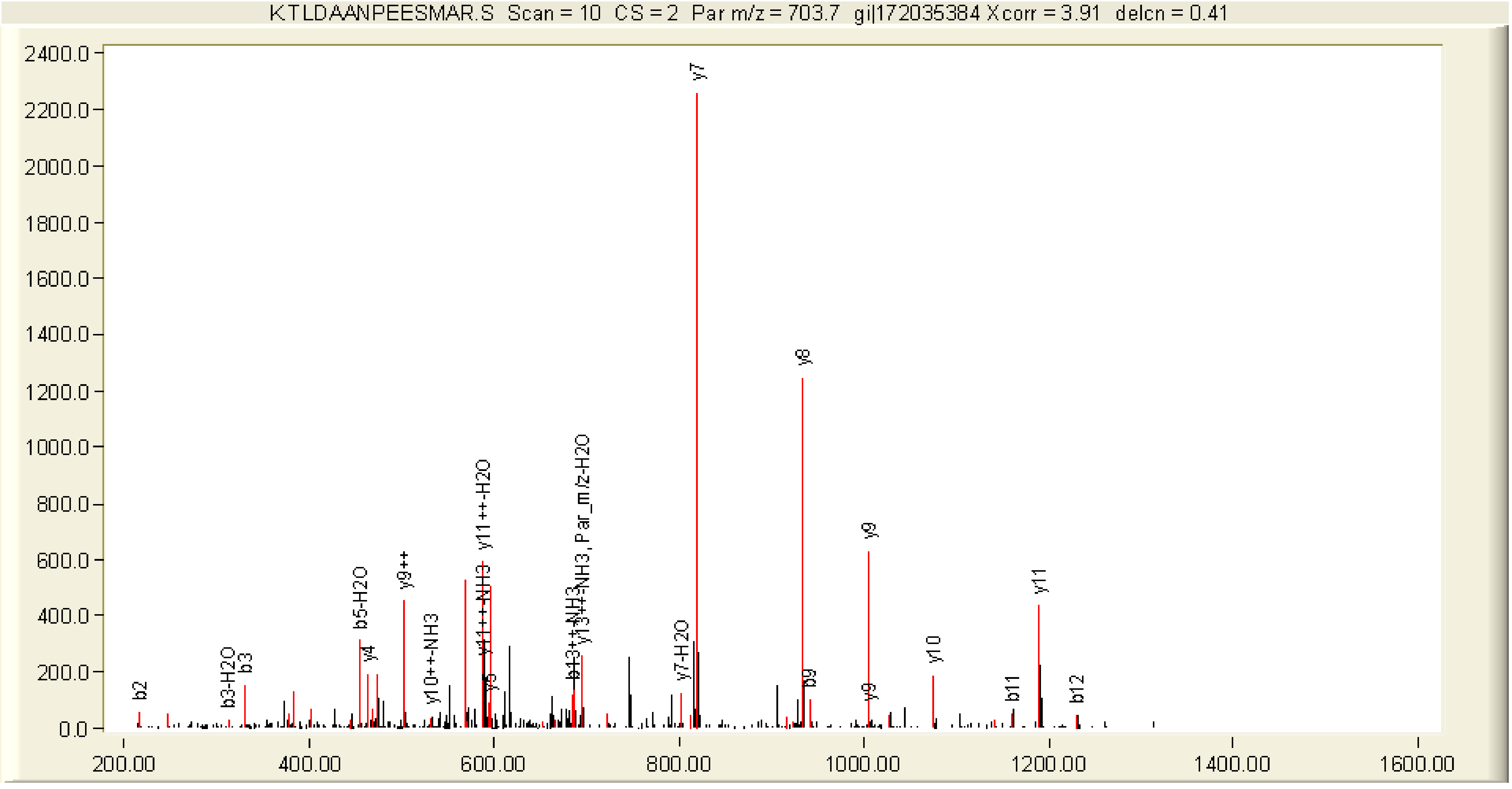
KTLDAANPEESMAR.S Scan = 10 CS = 2 Par m/z = 703.7 gi|172035384 Xcorr = 3.91 delcn = 0.41
2400.0
2200.0
2000.0
1800.0
1600.0
1400.0
1200.0
1000.0
800.0
600.0
400.0
200.0
0.0
200.00
400.00
600.00
800.00
1000.00
1200.00
1400.00
1600.00
b2
b3-H2O
b3
b5-H2O
y4
y9++
y10++-NH3
y11++-NH3
y11++-H2O
y5
y13++-NH3, Par_m/z-H2O
y7-H2O
y7
y8
b9
y9
y10
b11
y11
b12

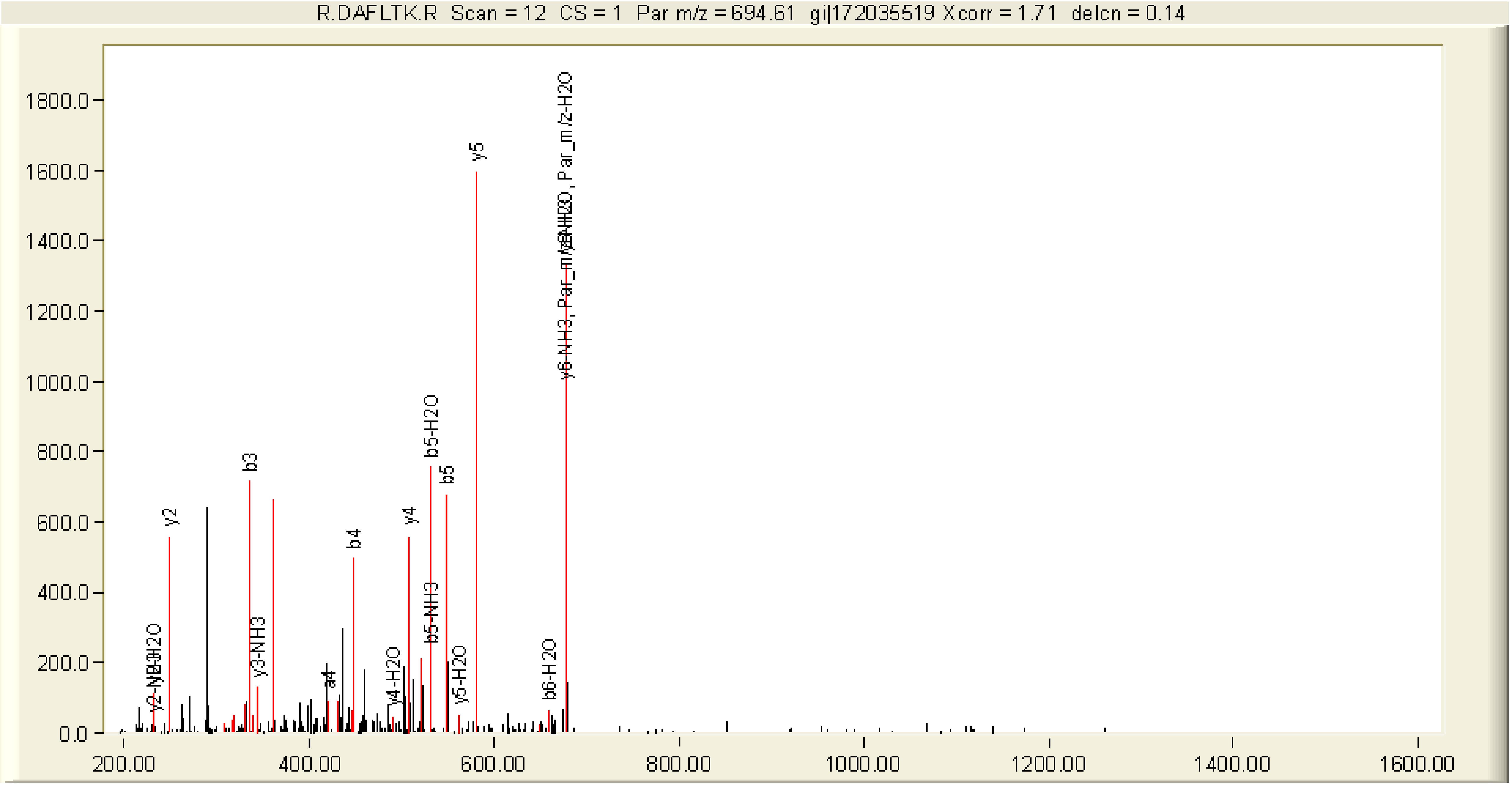
R.DAFLTK.R Scan = 12 CS = 1 Par m/z = 694.61 gi|172035519 Xcorr = 1.71 delcn = 0.14
1800.0
1600.0
1400.0
1200.0
1000.0
800.0
600.0
400.0
200.0
0.0
200.00
400.00
600.00
800.00
1000.00
1200.00
1400.00
1600.00
y2
b3
y3-NH3
b4
a4
y4
y4-H2O
b5-H2O
b5-NH3
b5
y5
y5-H2O
b6-H2O
y6-NH3, Par_m/z-H2O, Par_m/z-H2O

R.TIPTEADLLNR.A Scan = 14 CS = 2 Par m/z = 621.9 gi|172038390 Xcorr = 2.9 delcn = 0.2

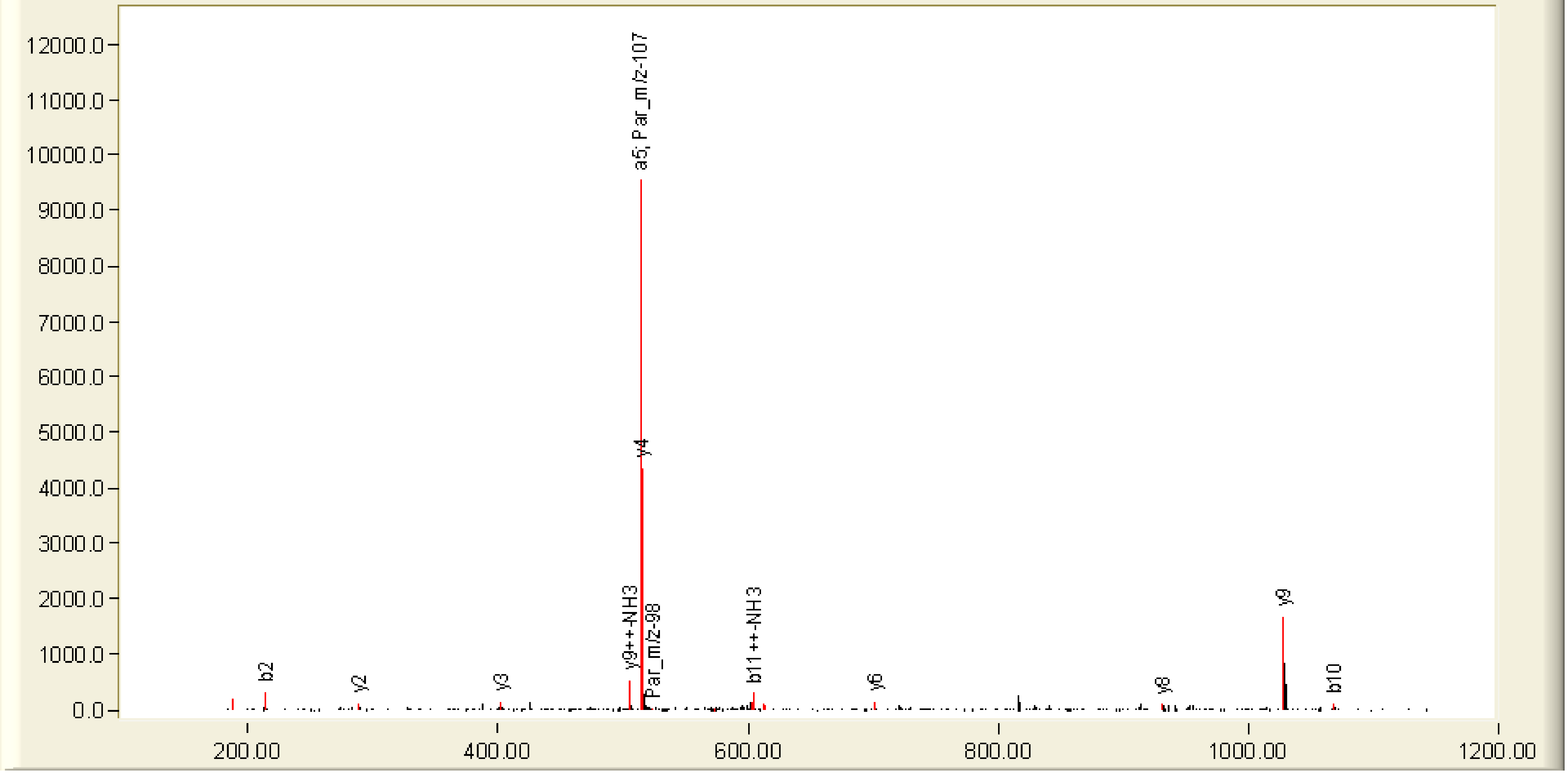

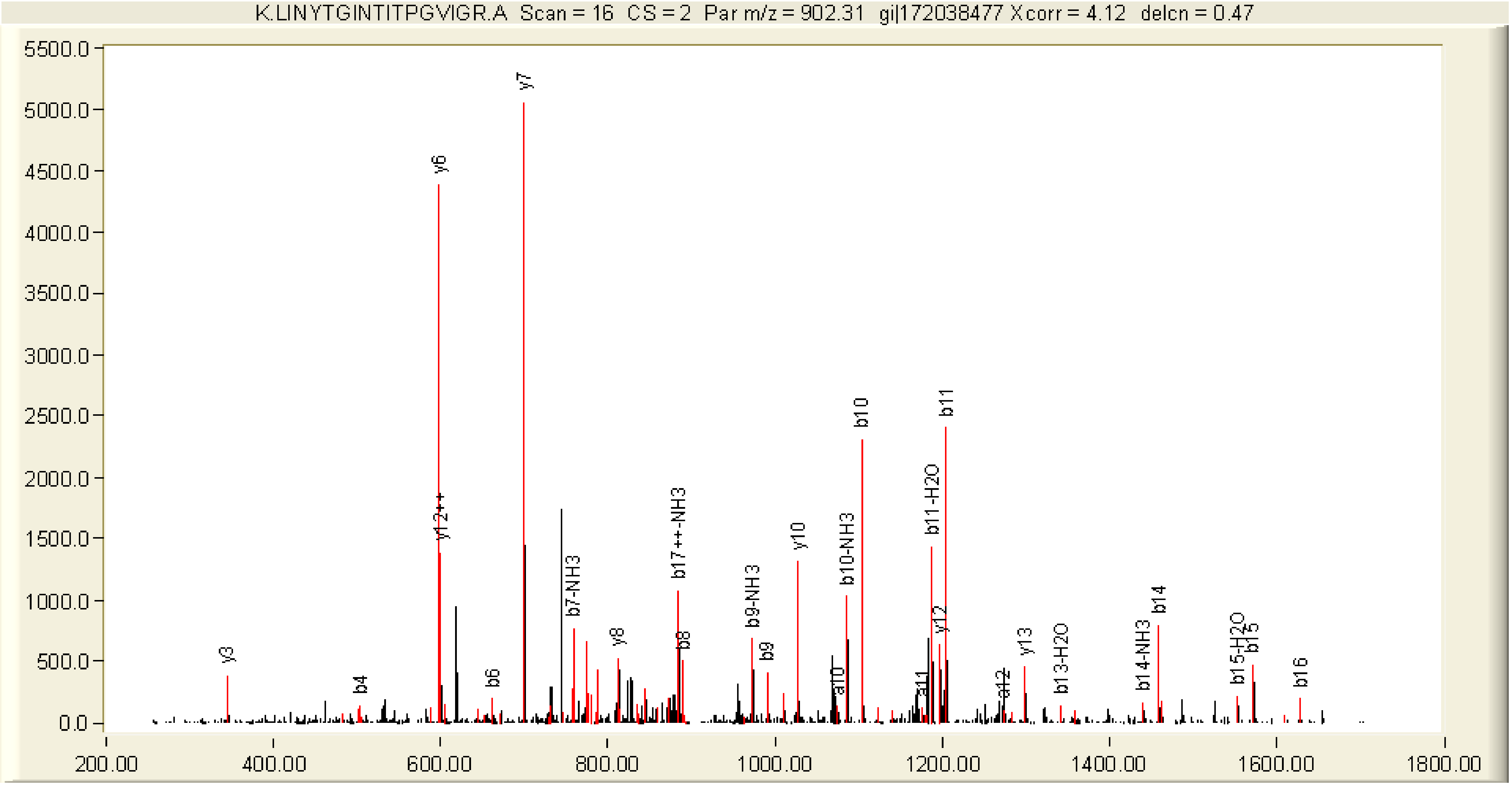

K.LINYTGINTITPGVIGR.A Scan = 16 CS = 2 Par m/z = 902.31 gi|172038477 Xcorr = 4.12 delcn = 0.47
5500.0
5000.0
4500.0
4000.0
3500.0
3000.0
2500.0
2000.0
1500.0
1000.0
500.0
0.0
200.00
400.00
600.00
800.00
1000.00
1200.00
1400.00
1600.00
1800.00
y7
y6
y12++
y3
b4
b6
b7-NH3
y8
b17++-NH3
b8
b9-NH3
b9
y10
a10
b10-NH3
b10
b11-H2O
b11
a11
y12
a12
y13
b13-H2O
b14-NH3
b14
b15-H2O
b15
b16

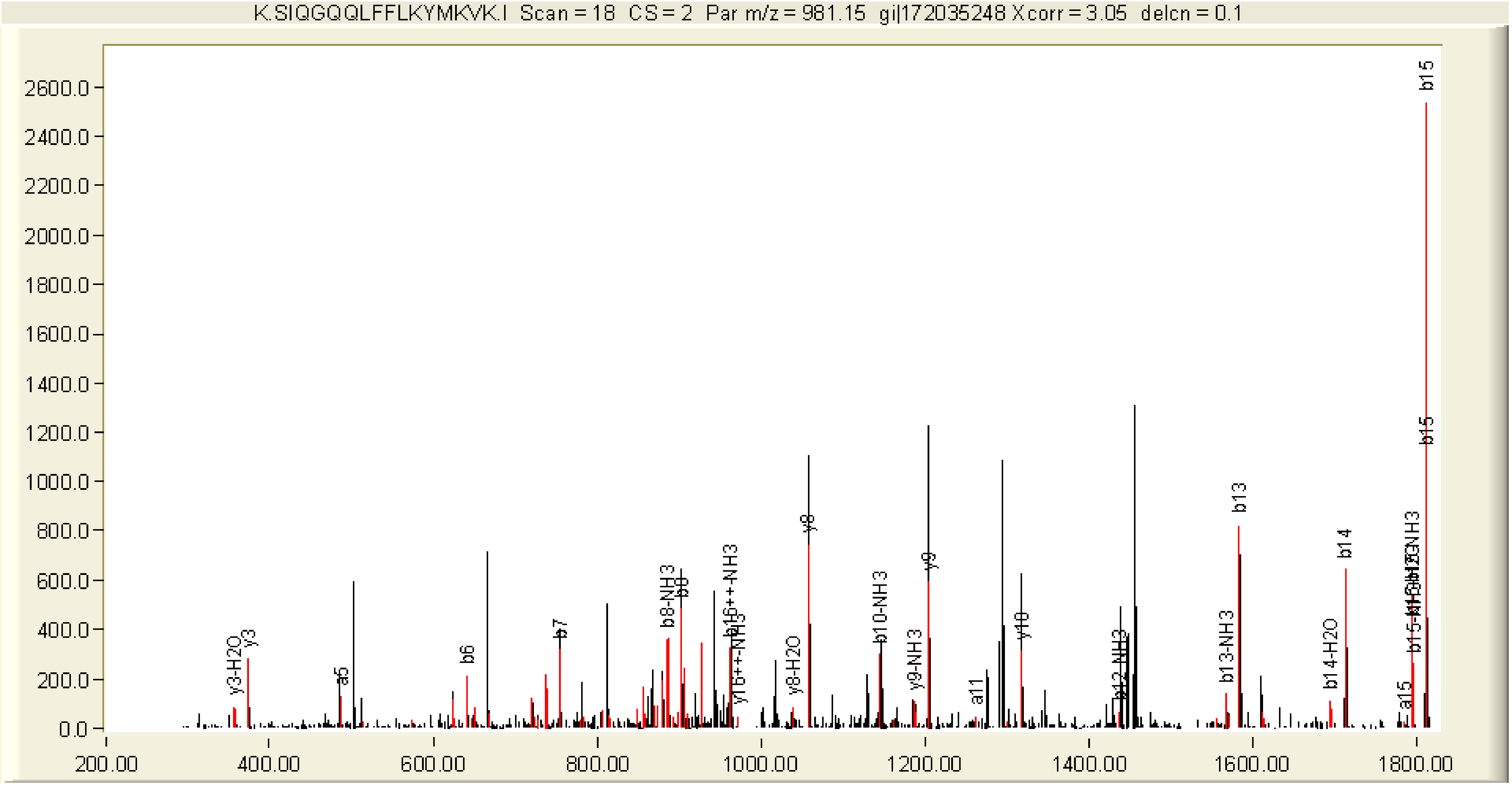
K.SIQGQQLFFLKYMKVK.I Scan = 18 CS = 2 Par m/z = 981.15 gi|172035248 Xcorr = 3.05 delcn = 0.1
2600.0
2400.0
2200.0
2000.0
1800.0
1600.0
1400.0
1200.0
1000.0
800.0
600.0
400.0
200.0
0.0
200.00
400.00
600.00
800.00
1000.00
1200.00
1400.00
1600.00
1800.00
y3-H2O
y3
a5
b6
b7
b8-NH3
b8
y8-H2O
y8
b10-NH3
y9-NH3
y9
a11
y10
b13-NH3
b13
b14-H2O
b14
a15
b15
b15

K.SGLFGTLVNLNPVTPLLVTTR.E Scan = 20 CS = 2 Par m/z = 1106.93 gi|172037005 Xcorr = 4.68 delcn = 0.5

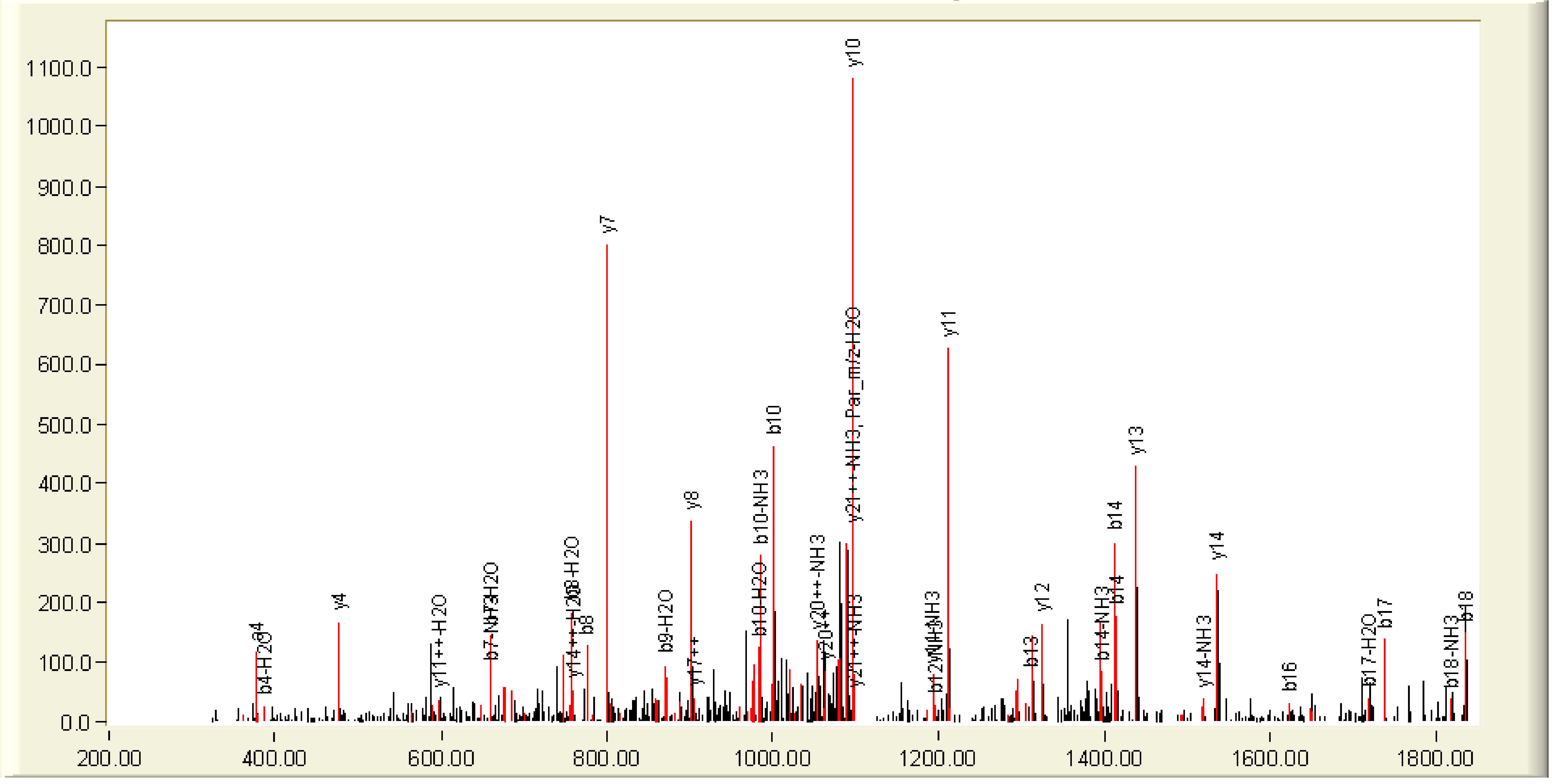

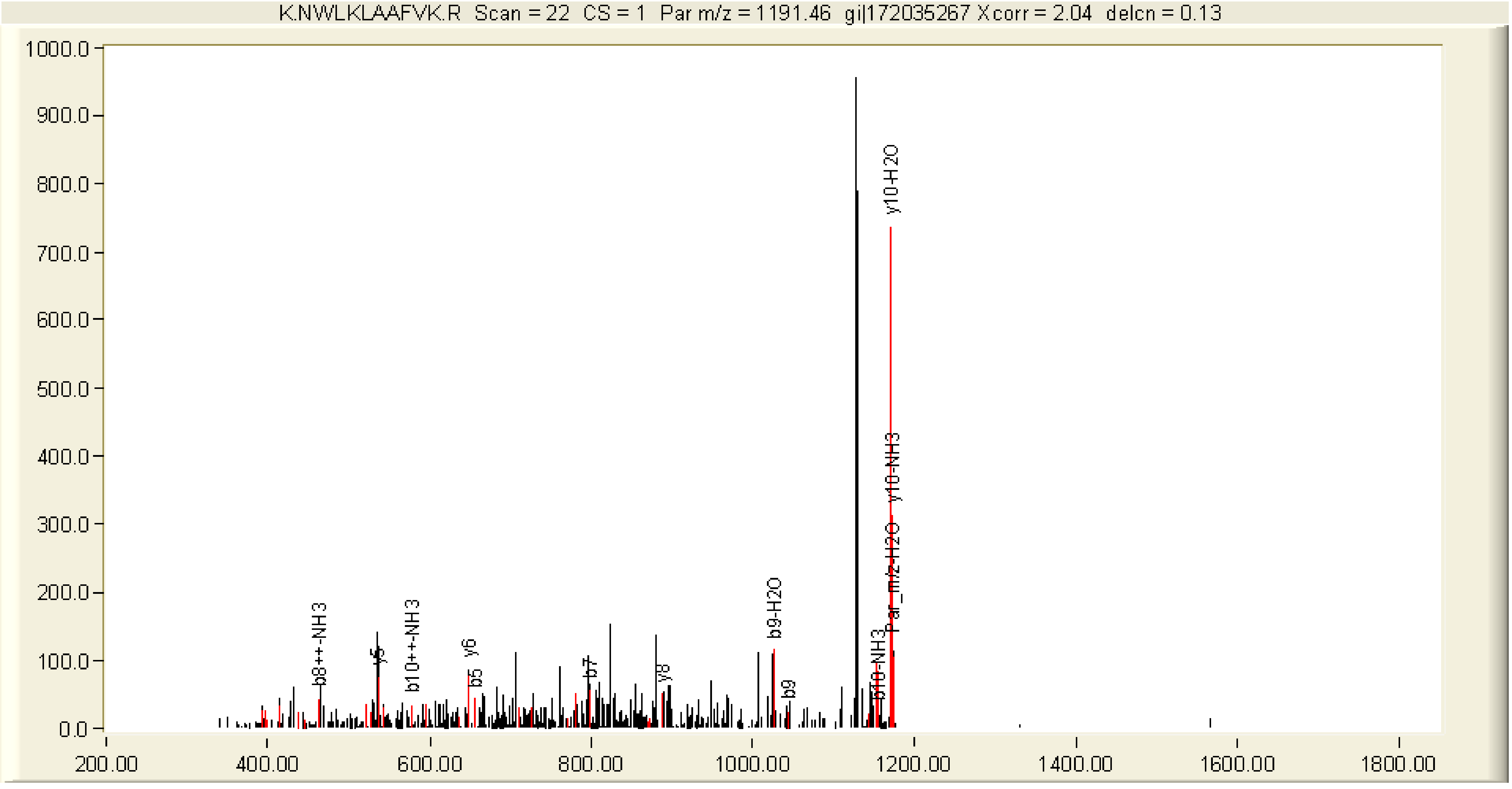
K.NWLKLAAFVK.R Scan = 22 CS = 1 Par m/z = 1191.46 gi|172035267 Xcorr = 2.04 delcn = 0.13
y10-H2O
y10-NH3
Par_m/z-H2O
b10-NH3
b9-H2O
b9
y8
b7
y6
b5
b10++-NH3
y5
b8++-NH3
1000.0
900.0
800.0
700.0
600.0
500.0
400.0
300.0
200.0
100.0
0.0
200.00
400.00
600.00
800.00
1000.00
1200.00
1400.00
1600.00
1800.00

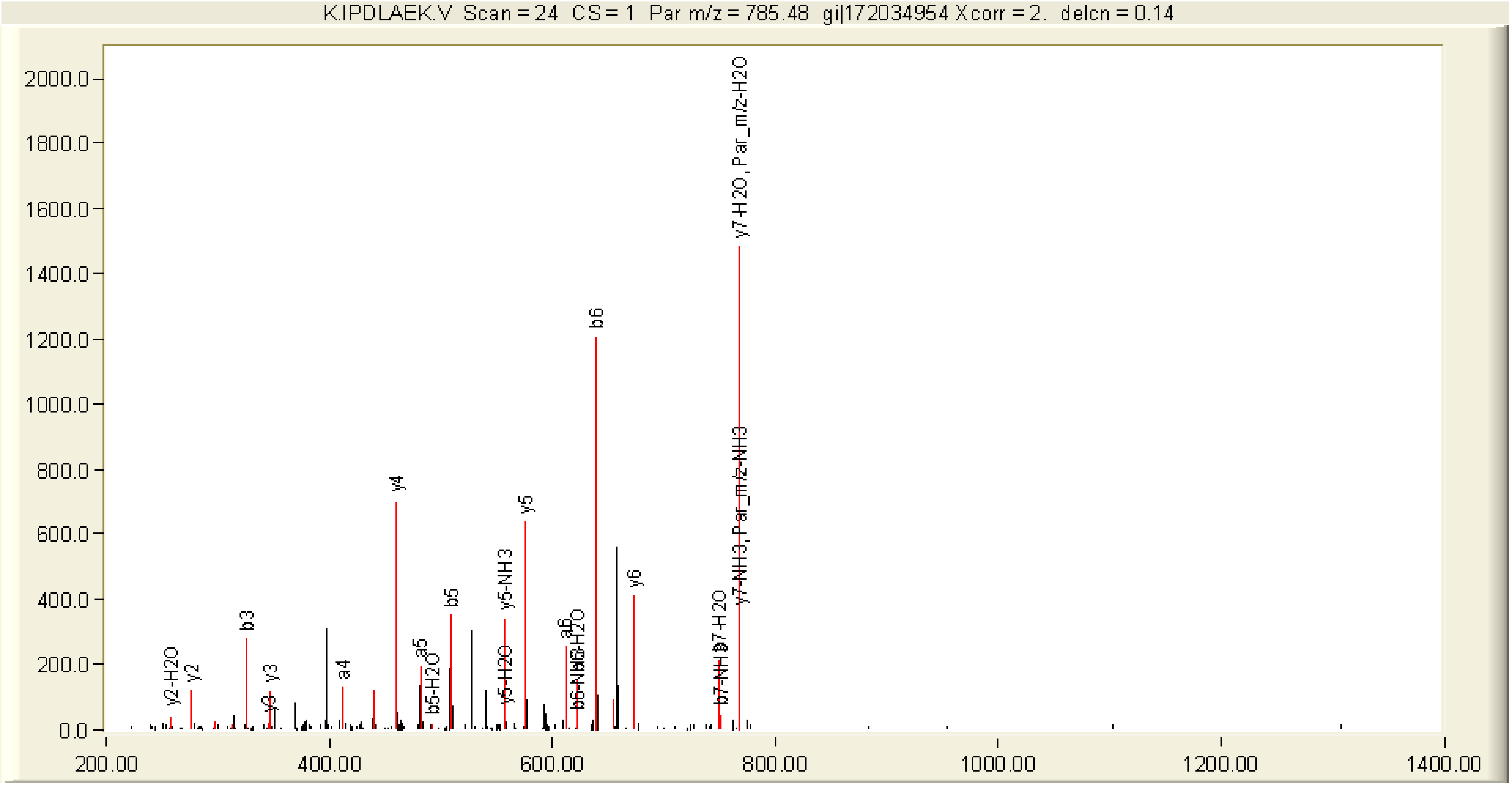
K.IPDLAEK.V Scan = 24 CS = 1 Par m/z = 785.48 gi|172034954 Xcorr = 2. delcn = 0.14
y7-H2O, Par_m/z-H2O
b6
y4
y5
y7-NH3, Par_m/z-NH3
y6
y5-NH3
b5
b3
b7-H2O
a5
b5-H2O
a4
y2-H2O
y2
y3
y5-H2O
b7-NH3
2000.0
1800.0
1600.0
1400.0
1200.0
1000.0
800.0
600.0
400.0
200.0
0.0
200.00
400.00
600.00
800.00
1000.00
1200.00
1400.00

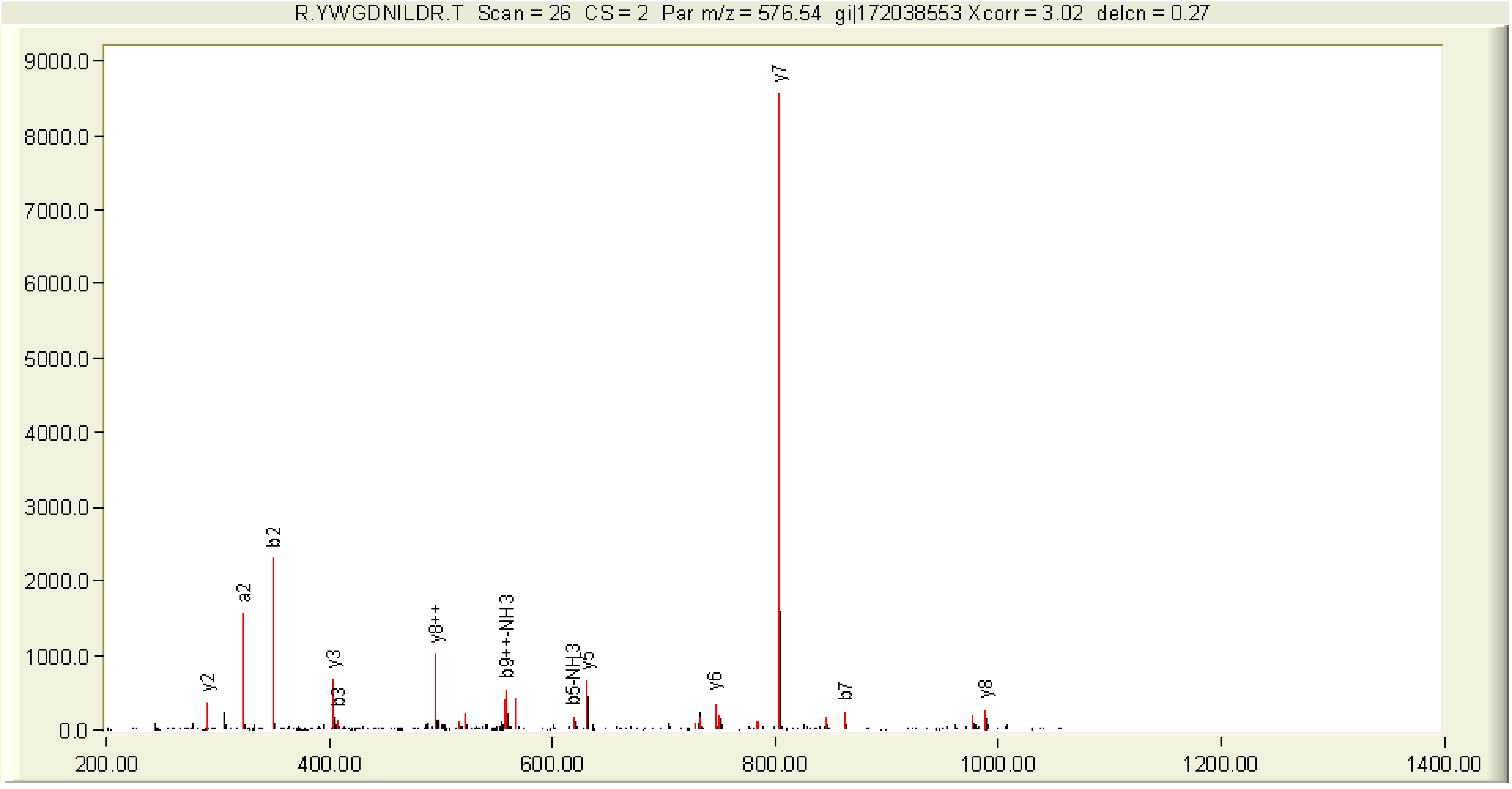

R.YWGDNILDR.T Scan = 26 CS = 2 Par m/z = 576.54 gi|172038553 Xcorr = 3.02 delcn = 0.27
9000.0
8000.0
7000.0
6000.0
5000.0
4000.0
3000.0
2000.0
1000.0
0.0
200.00
400.00
600.00
800.00
1000.00
1200.00
1400.00
y7
b2
a2
y8++
b9++-NH3
y3
b3
y2
b5-NH3
y5
y6
b7
y8

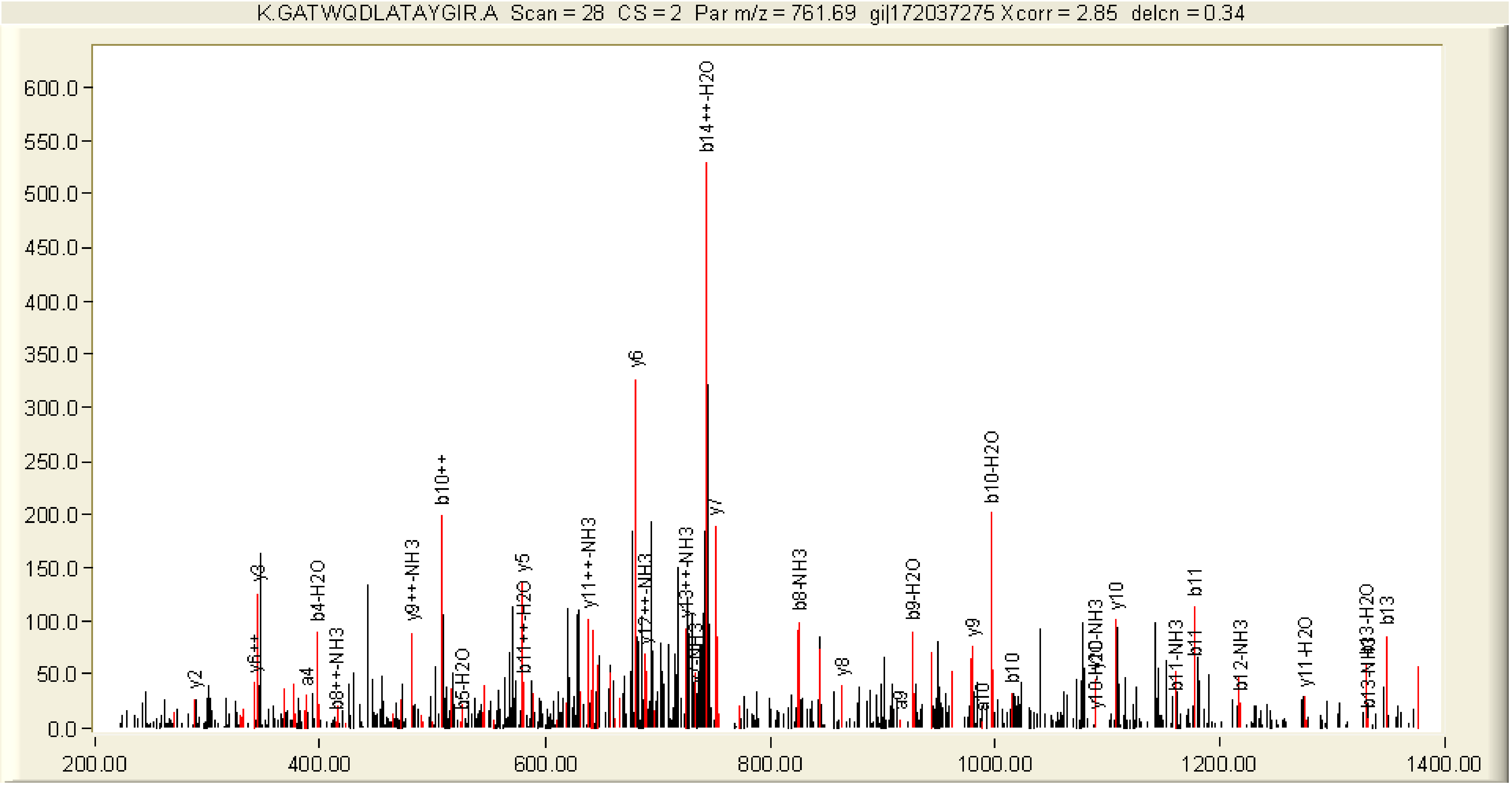
K.GATWQDLATAYGIR.A Scan = 28 CS = 2 Par m/z = 761.69 gi|172037275 Xcorr = 2.85 delcn = 0.34
600.0
550.0
500.0
450.0
400.0
350.0
300.0
250.0
200.0
150.0
100.0
50.0
0.0
200.00
400.00
600.00
800.00
1000.00
1200.00
1400.00
b14++-H2O
y6
b10-H2O
b10++
y7
y11++-NH3
b8-NH3
b9-H2O
b11
y10
b13
b4-H2O
y3
y5
y9++-NH3
y9
b12-NH3
y11-H2O
b10
y8
a4
y2
b8++-NH3
b5-H2O
b11-NH3
a9

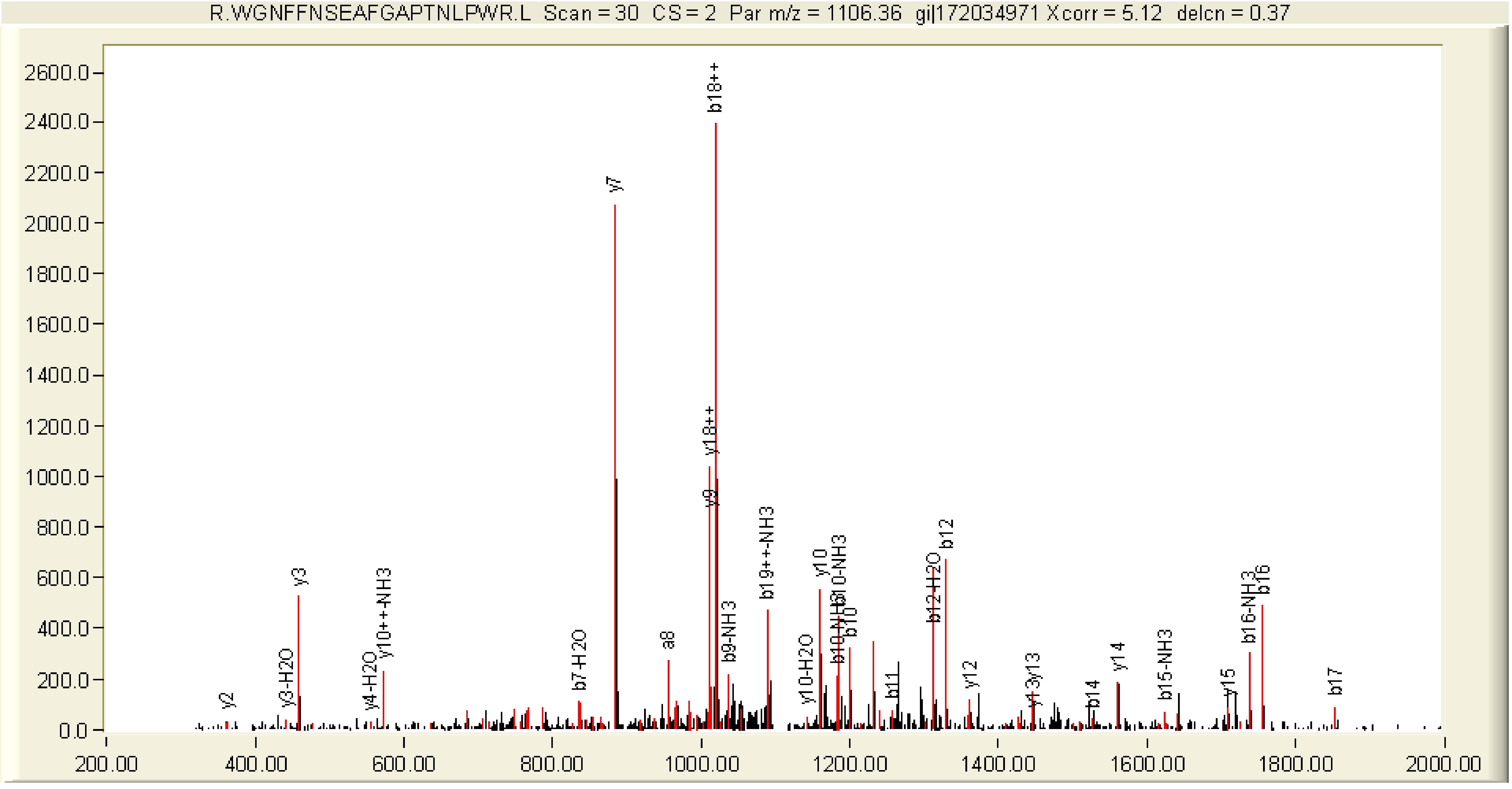
R.WGNFFNSEAFGAPTNLPWR.L Scan = 30 CS = 2 Par m/z = 1106.36 gi|172034971 Xcorr = 5.12 delcn = 0.37
2600.0
2400.0
2200.0
2000.0
1800.0
1600.0
1400.0
1200.0
1000.0
800.0
600.0
400.0
200.0
0.0
200.00
400.00
600.00
800.00
1000.00
1200.00
1400.00
1600.00
1800.00
2000.00
b18++
y7
y18++
y9
y3
y10++-NH3
b19++-NH3
y10
b12
b12-H2O
b16
b16-NH3
a8
b9-NH3
b7-H2O
y3-H2O
y4-H2O
y2
y10-H2O
b10
y12
y13
y14
b14
b15-NH3
y15
b17
b11

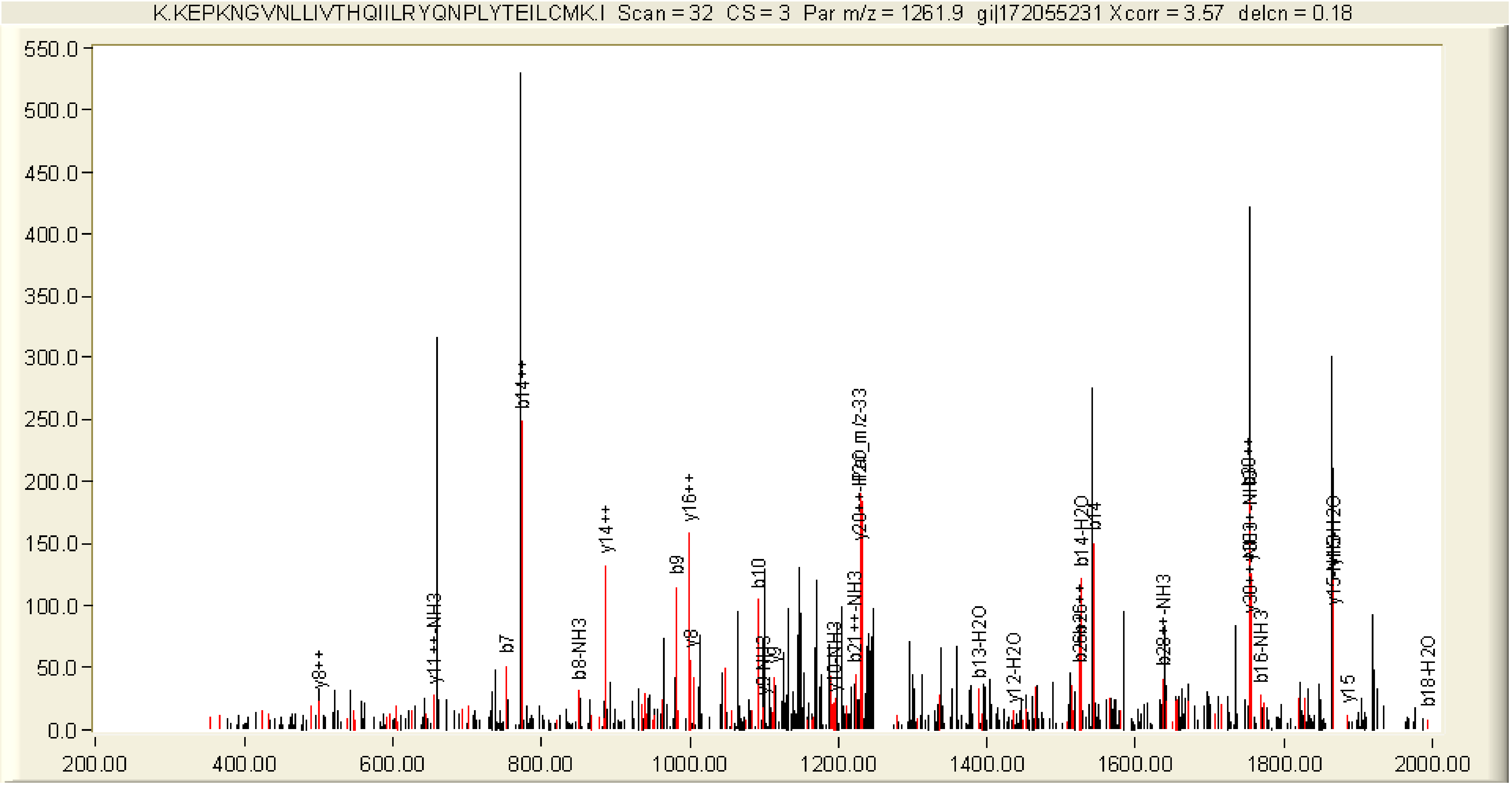
K.KEPKNGVNLLIVTHQIILRYQNPLYTEILCMK.I Scan = 32 CS = 3 Par m/z = 1261.9 gi|172055231 Xcorr = 3.57 delcn = 0.18
550.0
500.0
450.0
400.0
350.0
300.0
250.0
200.0
150.0
100.0
50.0
0.0
200.00
400.00
600.00
800.00
1000.00
1200.00
1400.00
1600.00
1800.00
2000.00
y8++
y11++-NH3
b7
b14++
b8-NH3
y14++
b9
y16++
y8
b10
b21++-NH3
b13-H2O
y12-H2O
b14-H2O
b14
b28++-NH3
b16-NH3
y15
b18-H2O

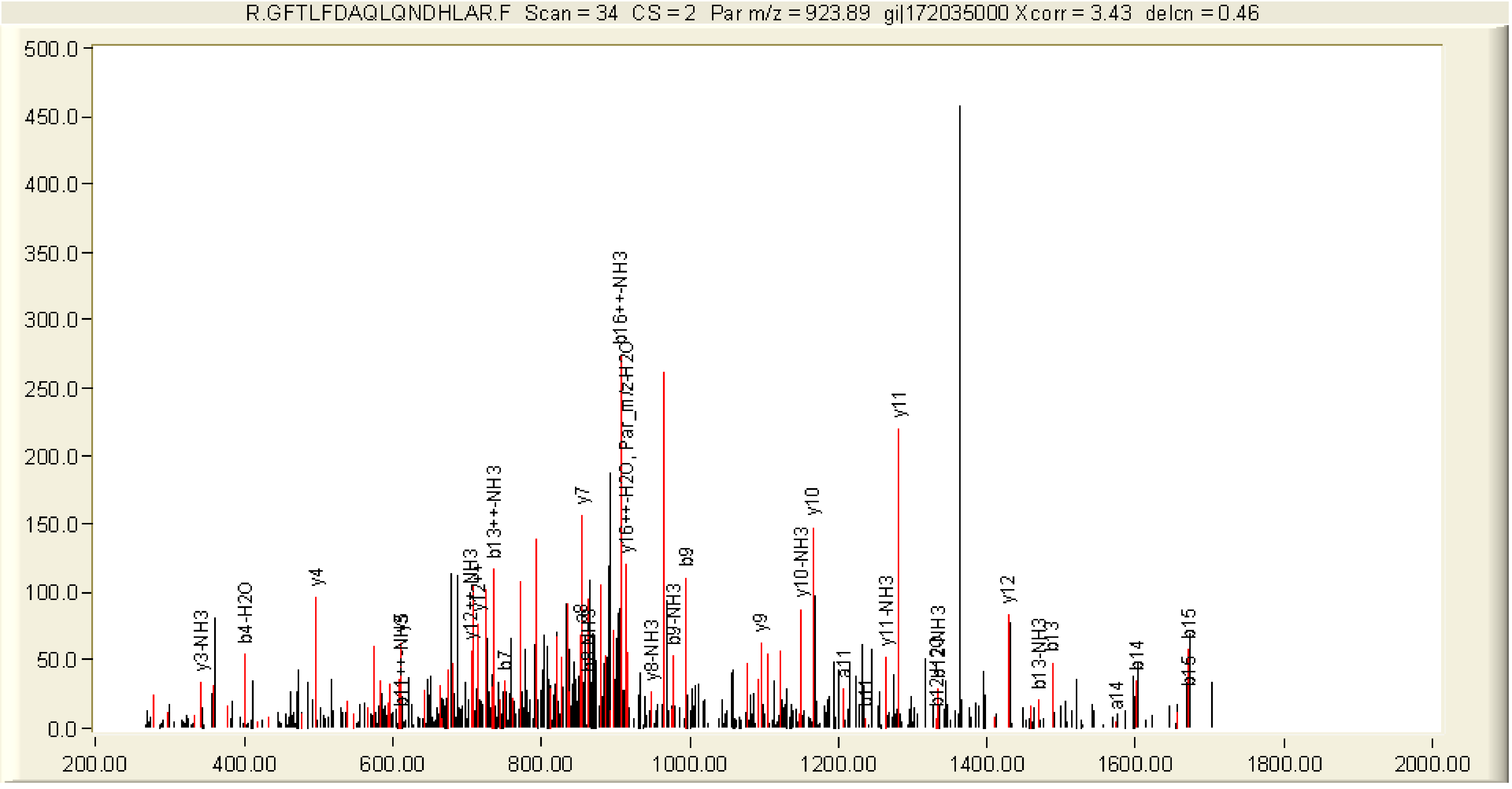
R.GFTLFDAQLQNDHLAR.F Scan = 34 CS = 2 Par m/z = 923.89 gi|172035000 Xcorr = 3.43 delcn = 0.46
500.0
450.0
400.0
350.0
300.0
250.0
200.0
150.0
100.0
50.0
0.0
200.00
400.00
600.00
800.00
1000.00
1200.00
1400.00
1600.00
1800.00
2000.00
y3-NH3
b4-H2O
y4
b7
b13++-NH3
y7
b16++-NH3
y16++-H2O, Par_m/z-H2O
y8-NH3
b9-NH3
b9
y9
y10-NH3
y10
a11
y11-NH3
y11
y12
b13-NH3
b13
a14
b14
b15
b15

R.LLAALPEREYQR.I Scan = 36 CS = 2 Par m/z = 730.67 gi|172037722 Xcorr = 3.1 delcn = 0.38

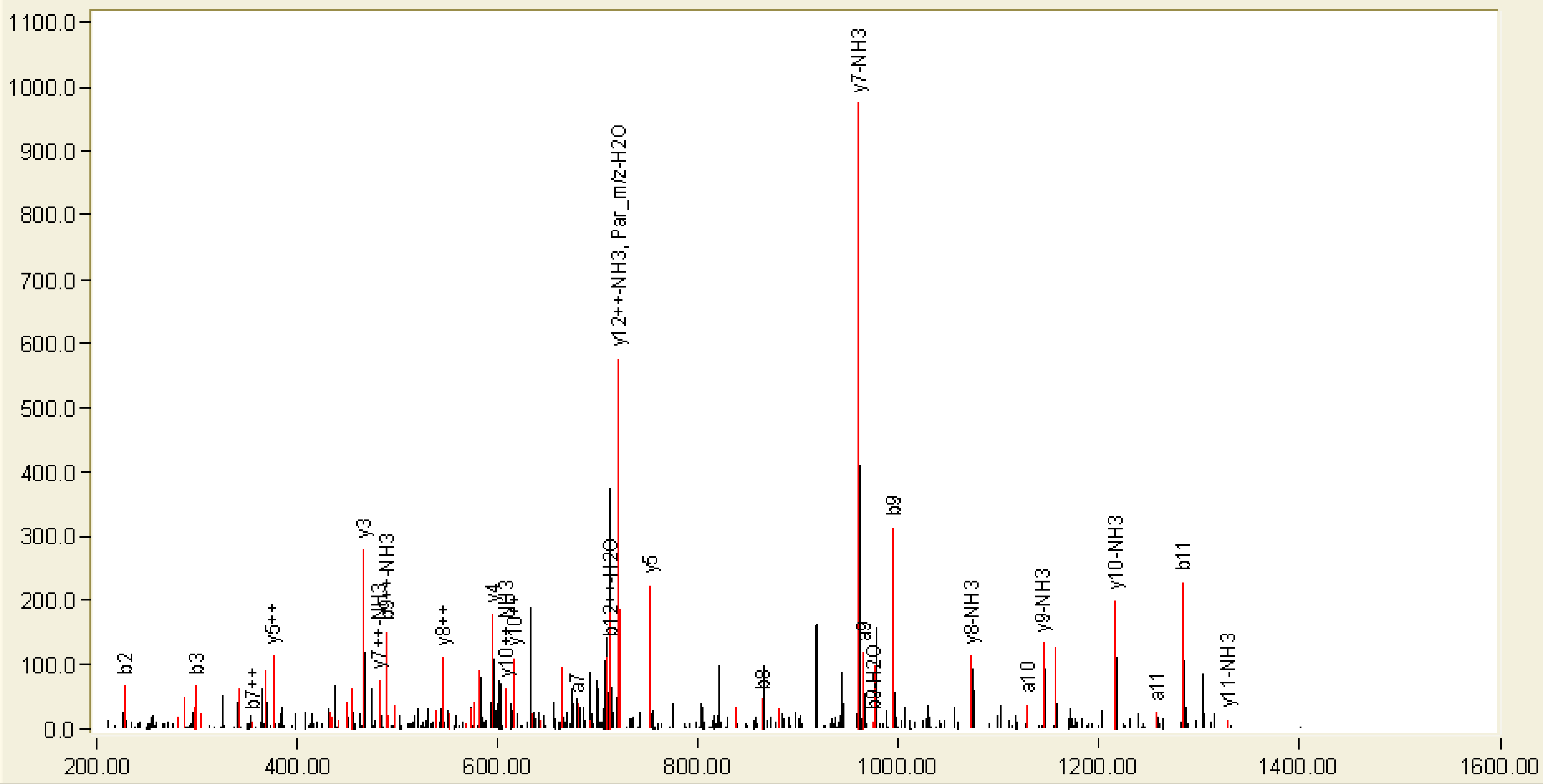

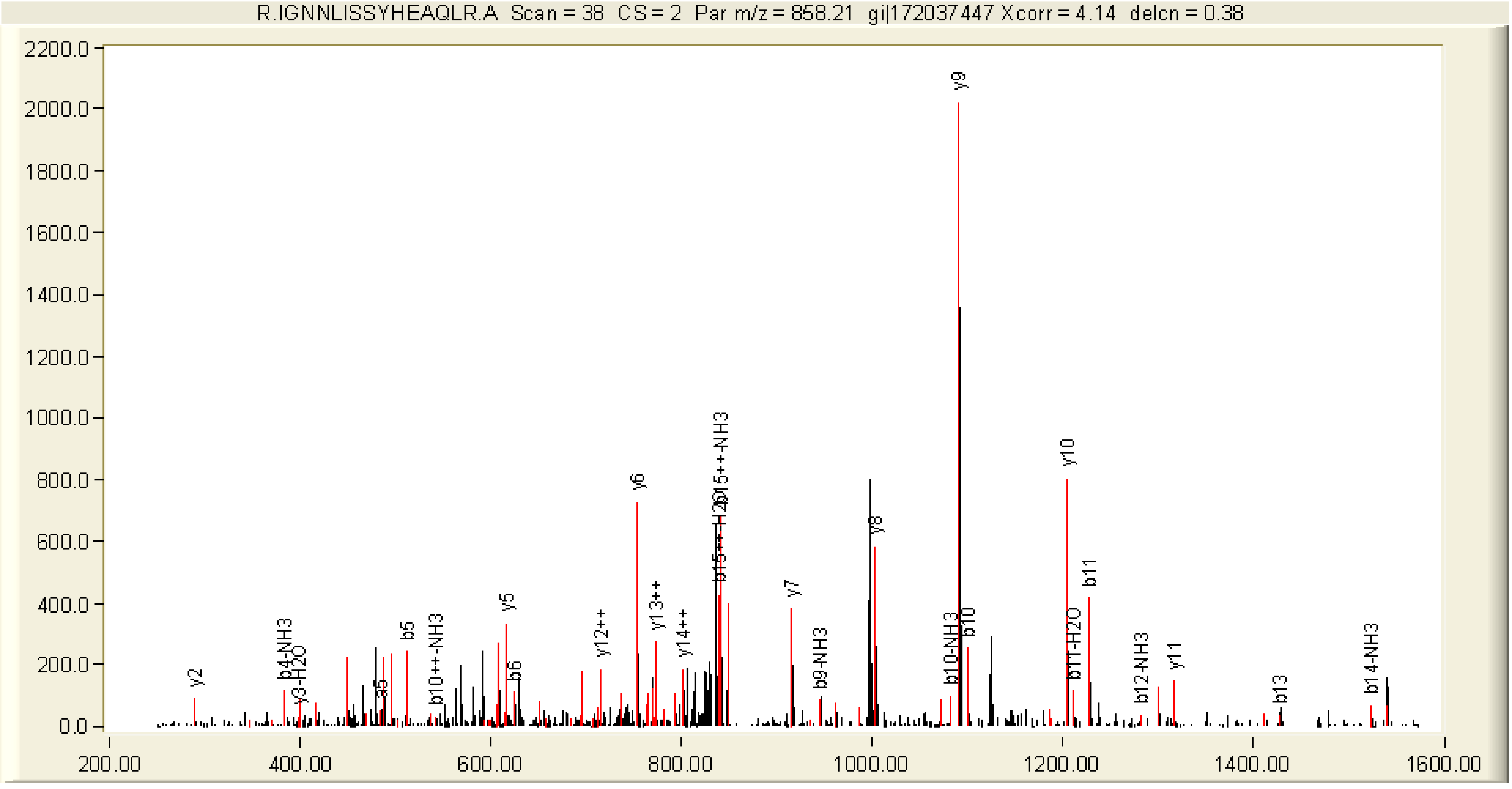

R.IGNNLISSYHEAQLR.A Scan = 38 CS = 2 Par m/z = 858.21 gi|172037447 Xcorr = 4.14 delcn = 0.38
2200.0
2000.0
1800.0
1600.0
1400.0
1200.0
1000.0
800.0
600.0
400.0
200.0
0.0
200.00
400.00
600.00
800.00
1000.00
1200.00
1400.00
1600.00
y2
b4-NH3
y3-H2O
a5
b5
b10++-NH3
y5
b6
y12++
y6
y13++
y14++
b15++-NH3
b15++-H2O
y7
b9-NH3
y8
y9
b10-NH3
b10
y10
b11-H2O
b11
b12-NH3
y11
b13
b14-NH3

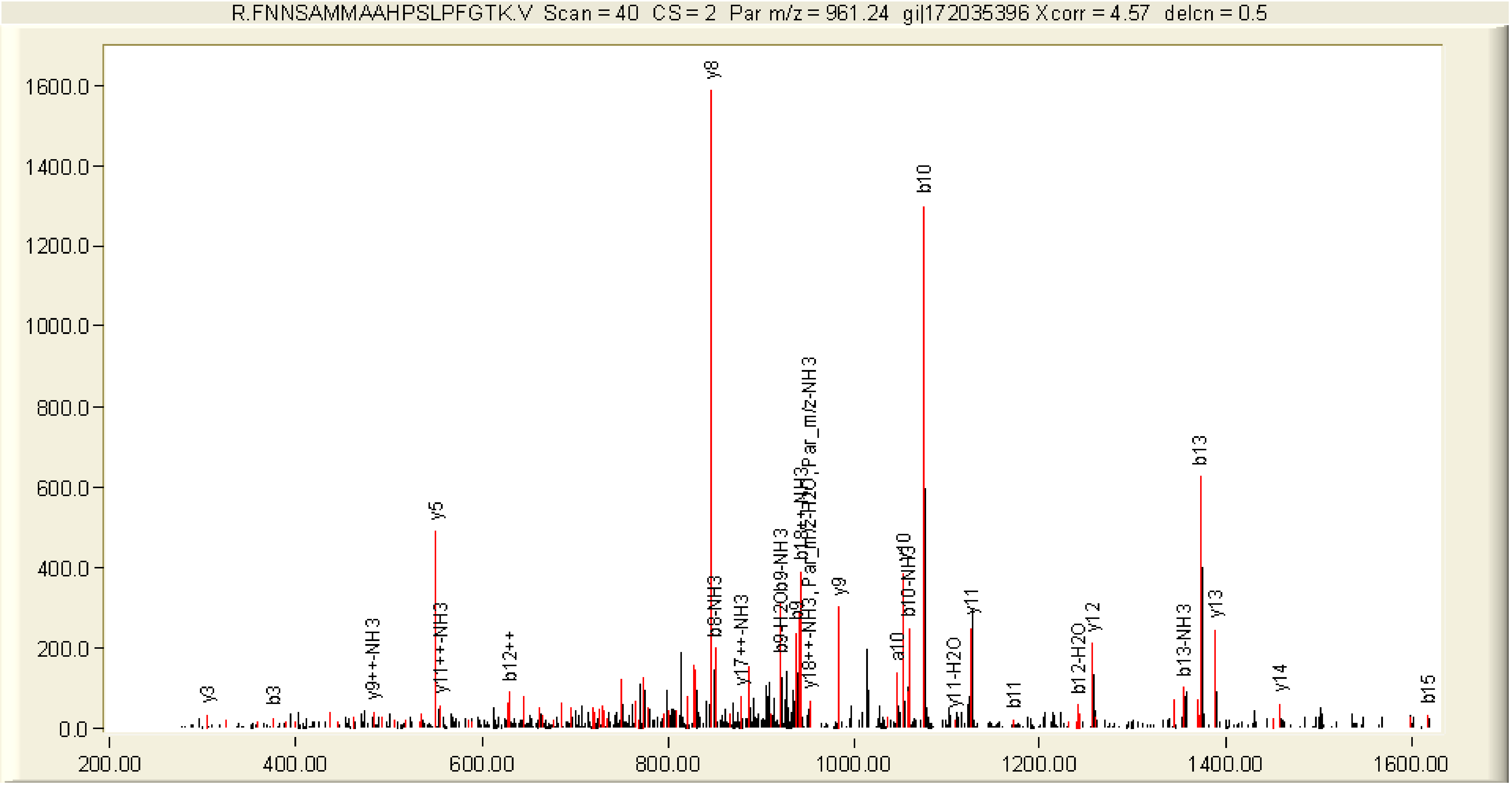
R.FNNSAMMAAHPSLPFGTK.V Scan = 40 CS = 2 Par m/z = 961.24 gi|172035396 Xcorr = 4.57 delcn = 0.5
1600.0
1400.0
1200.0
1000.0
800.0
600.0
400.0
200.0
0.0
200.00
400.00
600.00
800.00
1000.00
1200.00
1400.00
1600.00
y3
b3
y9++-NH3
y11++-NH3
y5
b12++
b8-NH3
y8
y17++-NH3
y9
a10
b10-NH3
b10
y11-H2O
y11
b11
b12-H2O
y12
b13-NH3
b13
y13
y14
b15

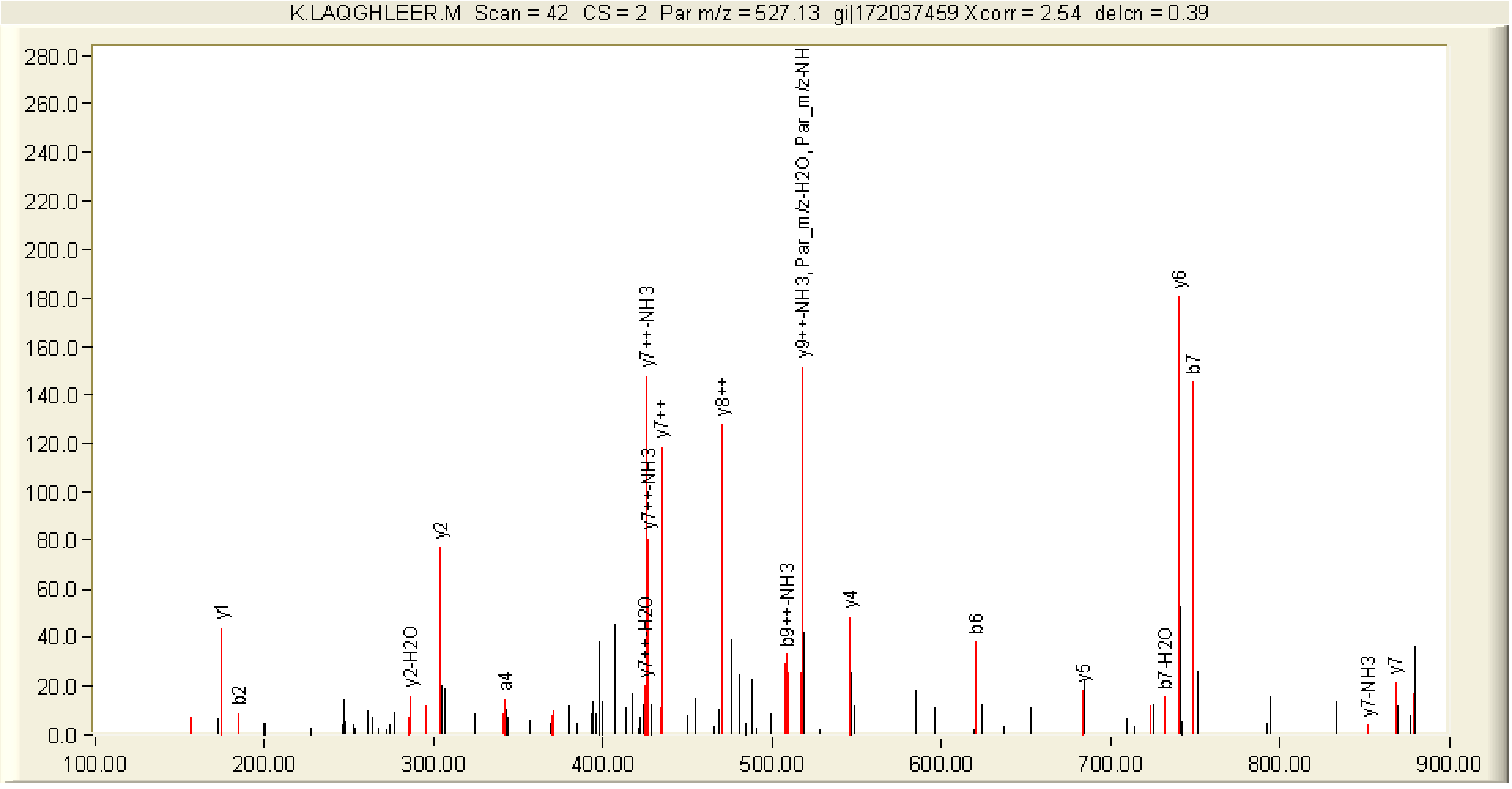

K.LAQGHLEER.M Scan = 42 CS = 2 Par m/z = 527.13 gi|172037459 Xcorr = 2.54 delcn = 0.39
280.0
260.0
240.0
220.0
200.0
180.0
160.0
140.0
120.0
100.0
80.0
60.0
40.0
20.0
0.0
100.00
200.00
300.00
400.00
500.00
600.00
700.00
800.00
900.00
y1
b2
y2-H2O
y2
a4
y7++-H2O
y7++-NH3
y7++-NH3
y7++
y8++
b9++-NH3
y9++-NH3, Par_m/z-H2O, Par_m/z-NH
y4
b6
y5
b7-H2O
y6
b7
y7-NH3
y7

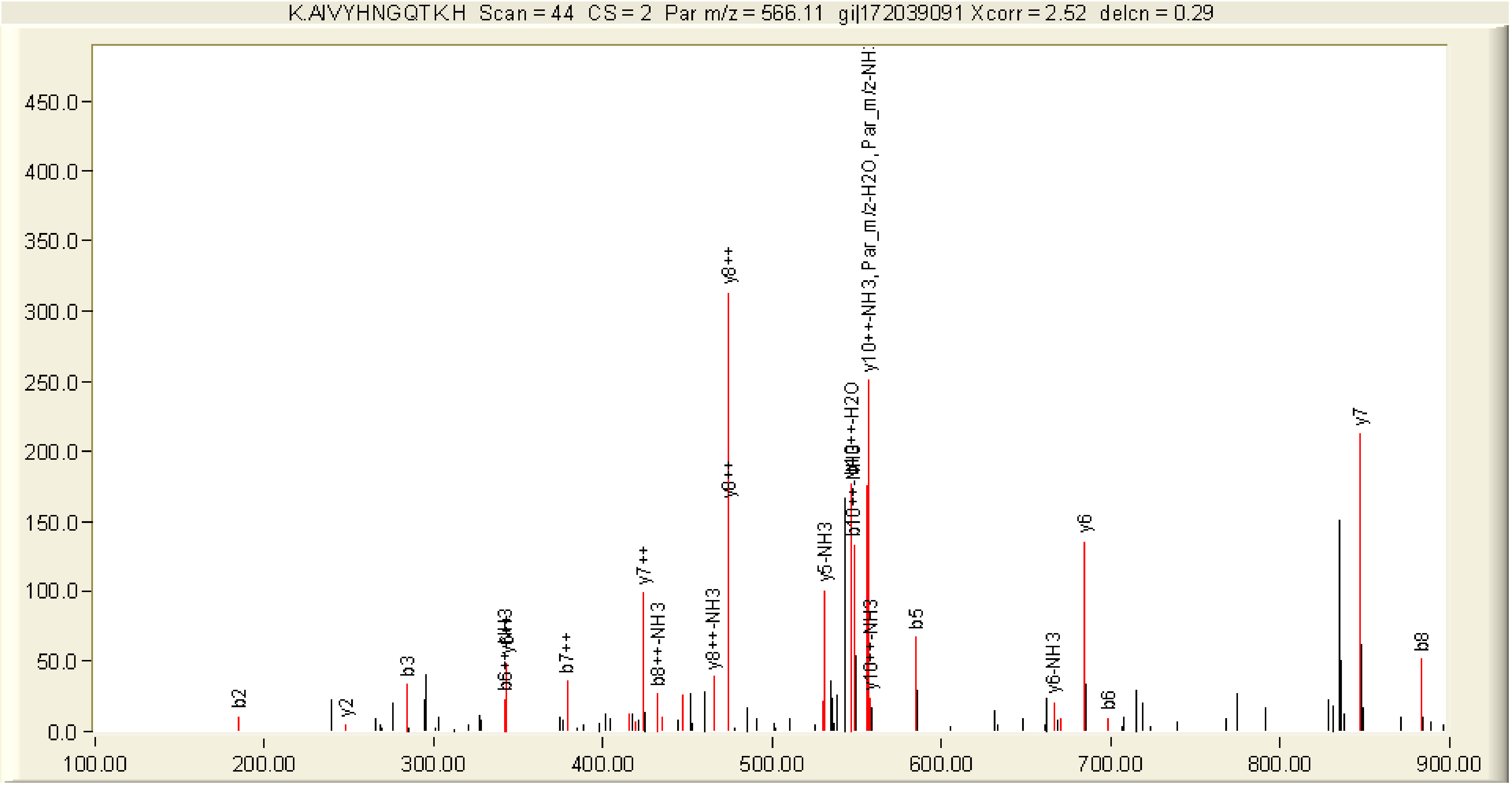

K.AIVYHNGQTK.H Scan = 44 CS = 2 Par m/z = 566.11 gi|172039091 Xcorr = 2.52 delcn = 0.29
y10++-NH3, Par_m/z-H2O, Par_m/z-NH3
y8++
y9++
y7
y6
y5-NH3
y7++
b8++-NH3
y8++-NH3
b10++-NH3
b10++-H2O
y10++-NH3
b5
y6-NH3
b8
b6
b7++
b3
y2
b2

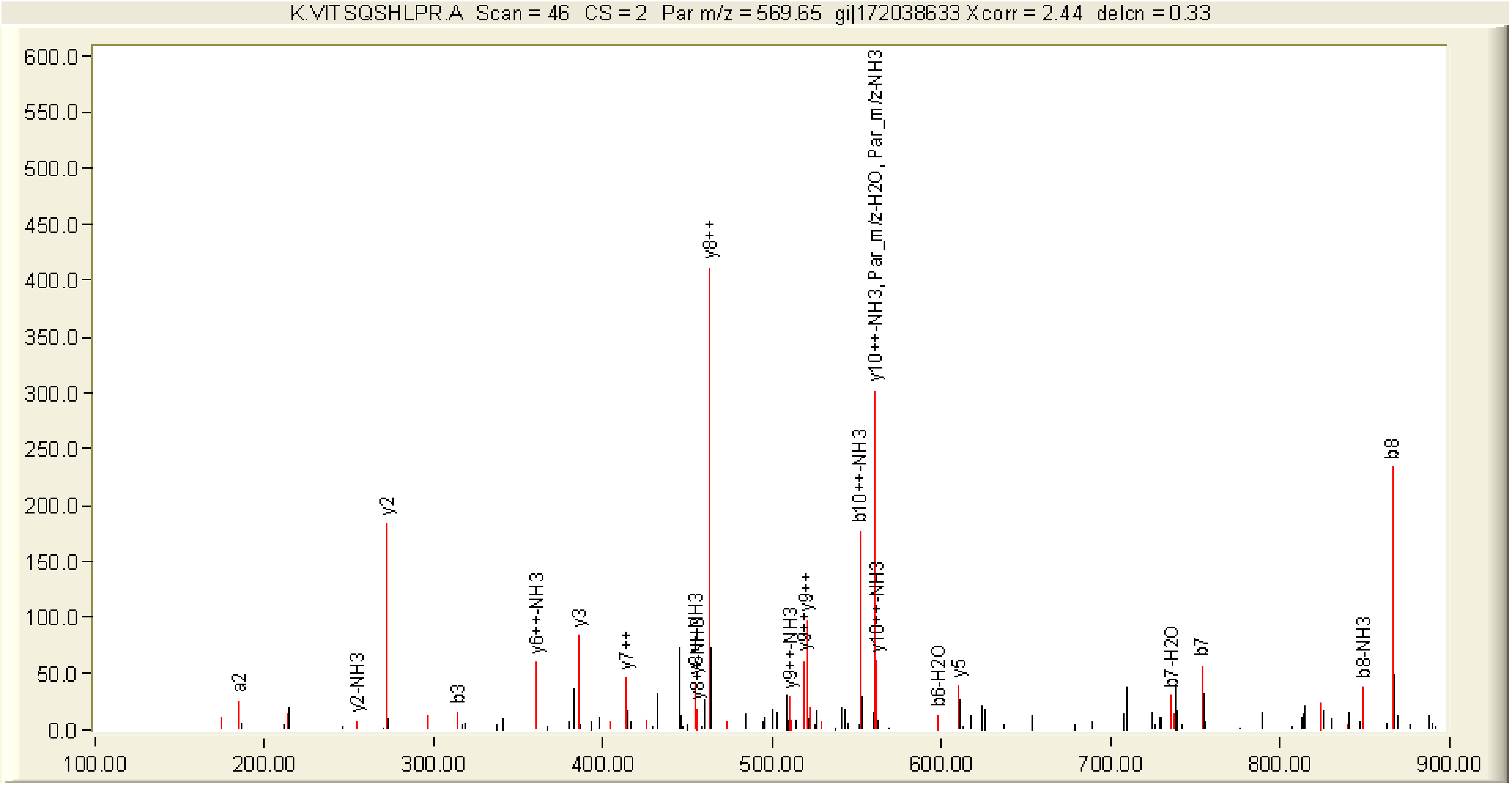
K.VITSQSHLPR.A Scan = 46 CS = 2 Par m/z = 569.65 gi|172038633 Xcorr = 2.44 delcn = 0.33
600.0
550.0
500.0
450.0
400.0
350.0
300.0
250.0
200.0
150.0
100.0
50.0
0.0
100.00
200.00
300.00
400.00
500.00
600.00
700.00
800.00
900.00
a2
y2-NH3
y2
b3
y6++-NH3
y3
y7++
y8++
y9++-NH3
y9++
b10++-NH3
y10++-NH3, Par_m/z-H2O, Par_m/z-NH3
y10++-NH3
b6-H2O
y5
b7-H2O
b7
b8-NH3
b8

R.VHASGPLNETSGR.V Scan = 48 CS = 2 Par m/z = 662.86 gi|172035992 Xcorr = 2.81 delcn = 0.4

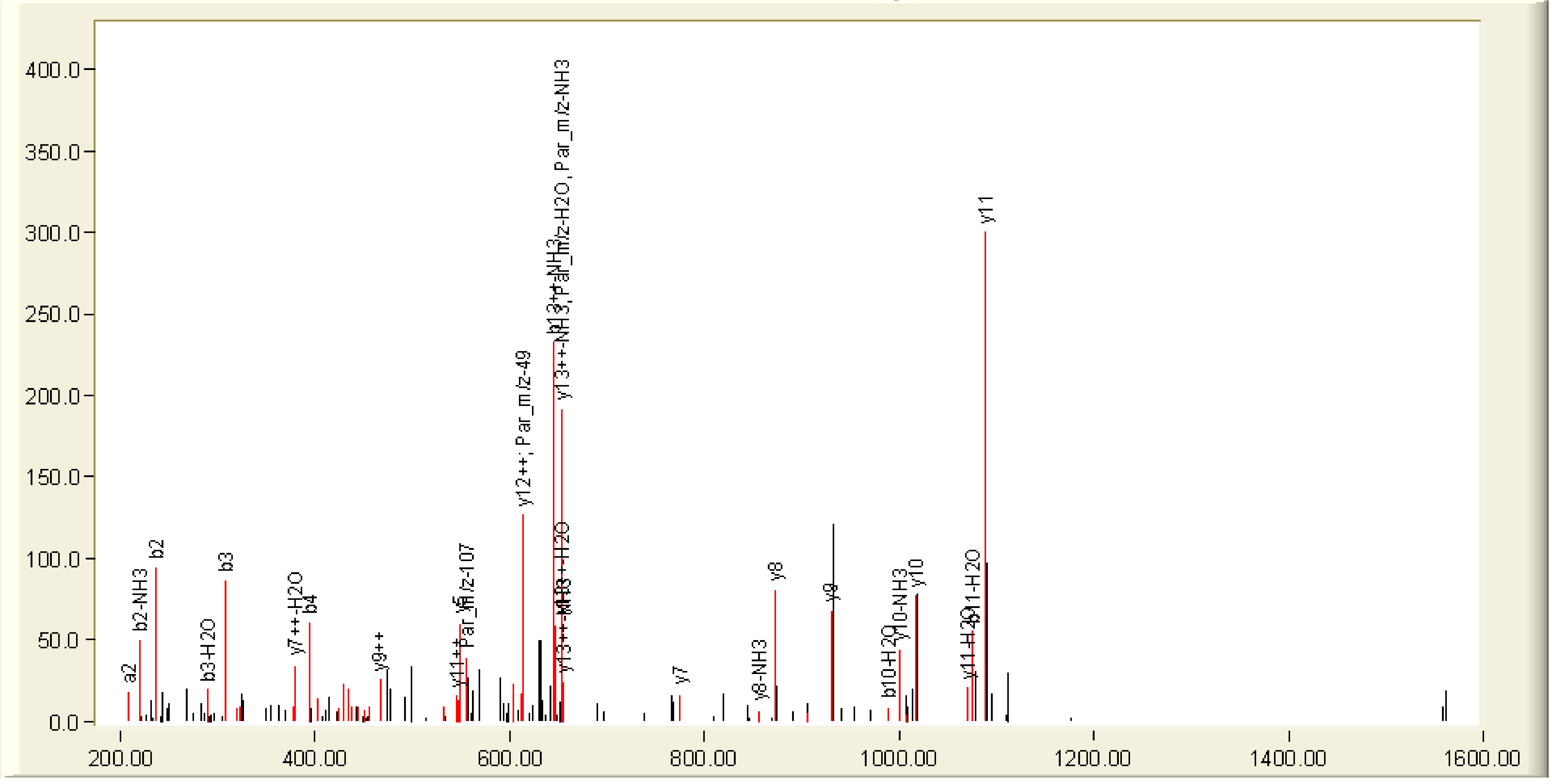

K.TSDSIFKNNLKNLKYQETGTSNKPLK.Y Scan = 50 CS = 3 Par m/z = 990.54 gi|172038196 Xcorr = 3.34 delcn = 0.11

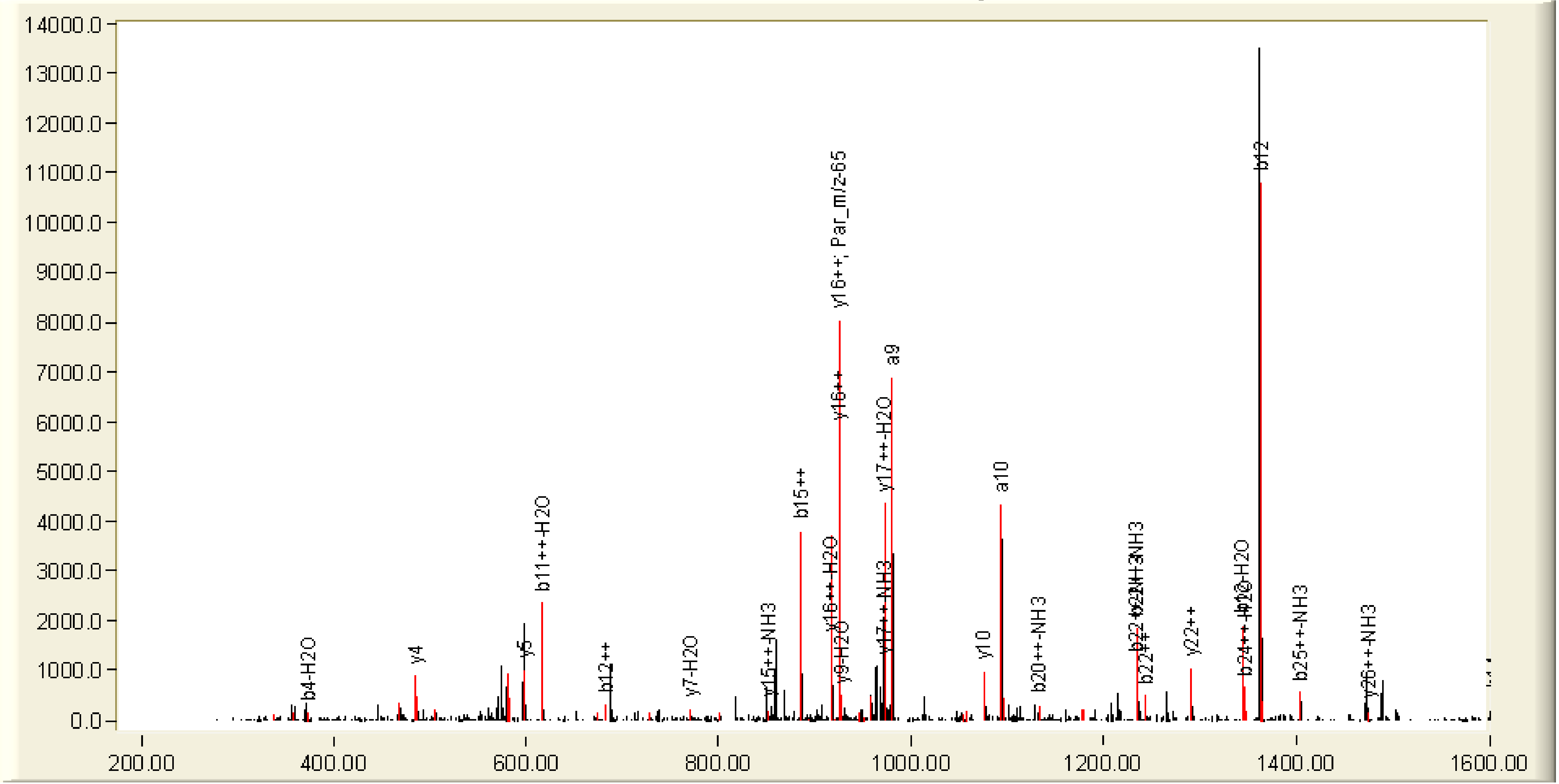

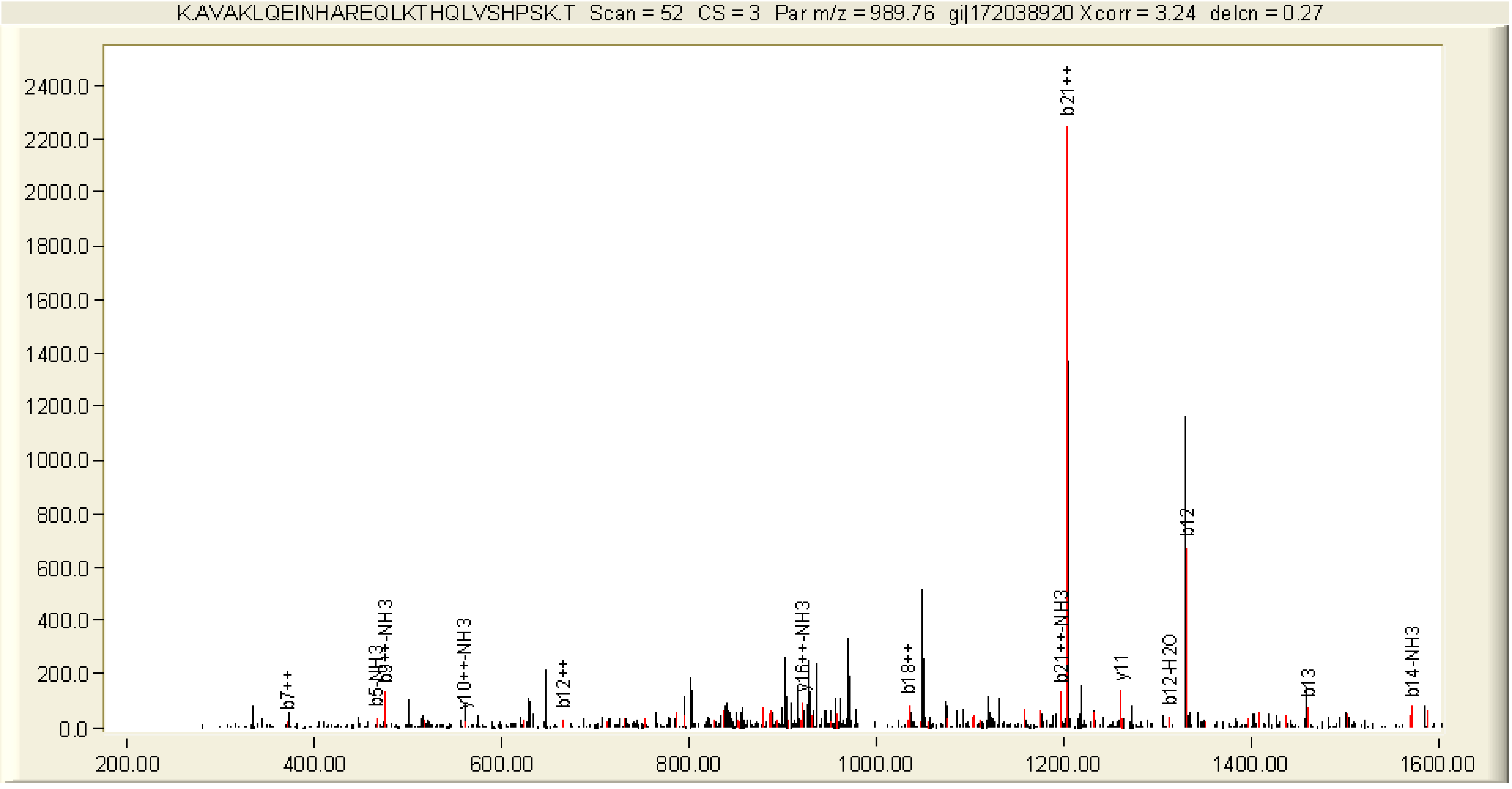

K.AVAKLQEINHAREQLKTHQLVSHPSK.T Scan = 52 CS = 3 Par m/z = 989.76 gi|172038920 Xcorr = 3.24 delcn = 0.27
2400.0
2200.0
2000.0
1800.0
1600.0
1400.0
1200.0
1000.0
800.0
600.0
400.0
200.0
0.0
200.00
400.00
600.00
800.00
1000.00
1200.00
1400.00
1600.00
b7++
b5-NH3
b9++-NH3
y10++-NH3
b12++
y16++-NH3
b18++
b21++-NH3
b21++
y11
b12H2O
b12
b13
b14-NH3

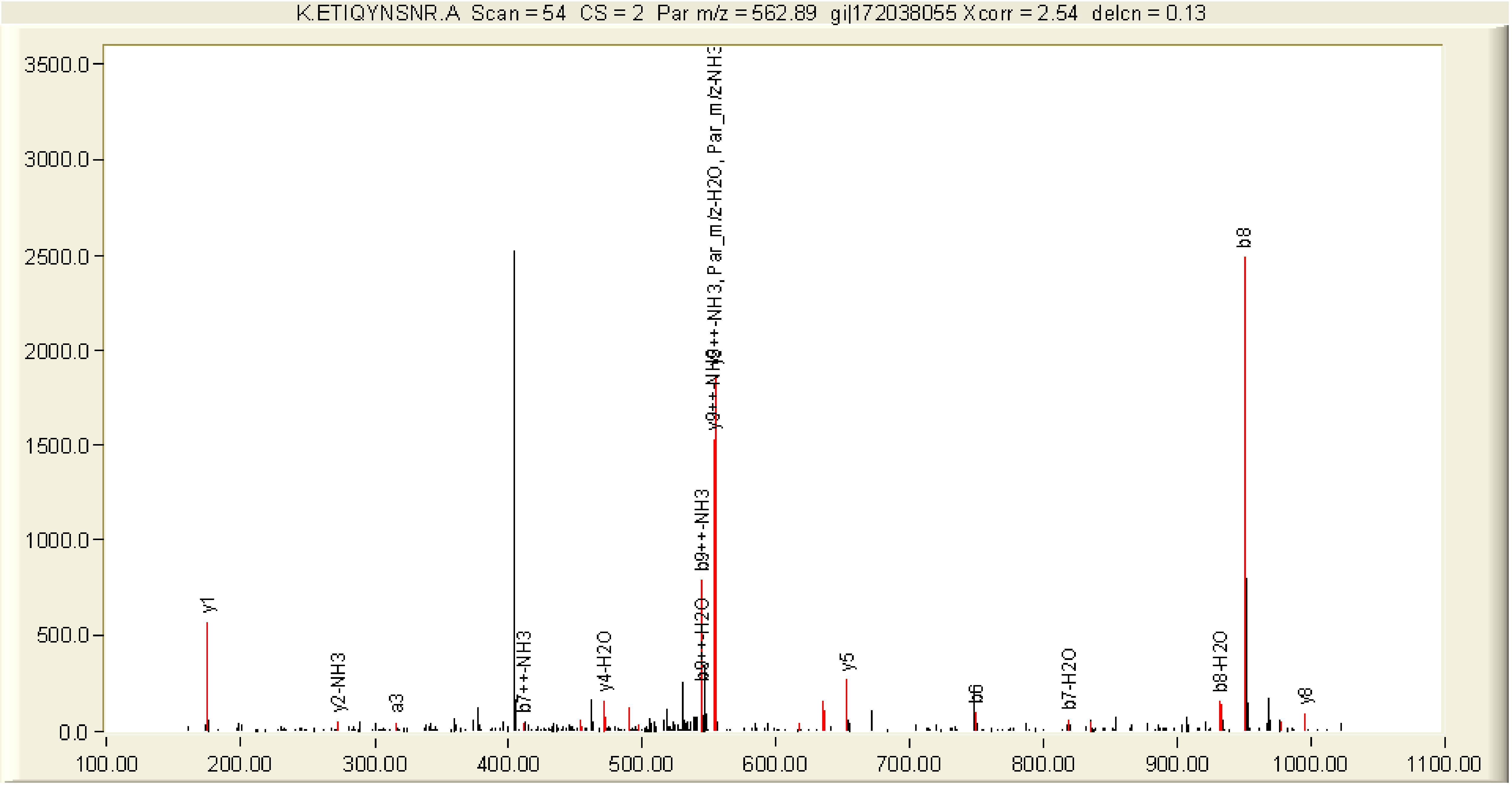

K.ETIQYNSNR.A Scan = 54 CS = 2 Par m/z = 562.89 gi|172038055 Xcorr = 2.54 delcn = 0.13
3500.0
3000.0
2500.0
2000.0
1500.0
1000.0
500.0
0.0
100.00
200.00
300.00
400.00
500.00
600.00
700.00
800.00
900.00
1000.00
1100.00
y1
y2-NH3
a3
b7++-NH3
y4-H2O
b9++-H2O
b9++-NH3
y9++-NH3, Par_m/z-H2O, Par_m/z-NH3
y5
b6
b7-H2O
b8-H2O
b8
y8

K.CFPTRKALAIMVR.V Scan = 56 CS = 1 Par m/z = 1504.8 gi|172054898 Xcorr = 2.23 delcn = 0.11

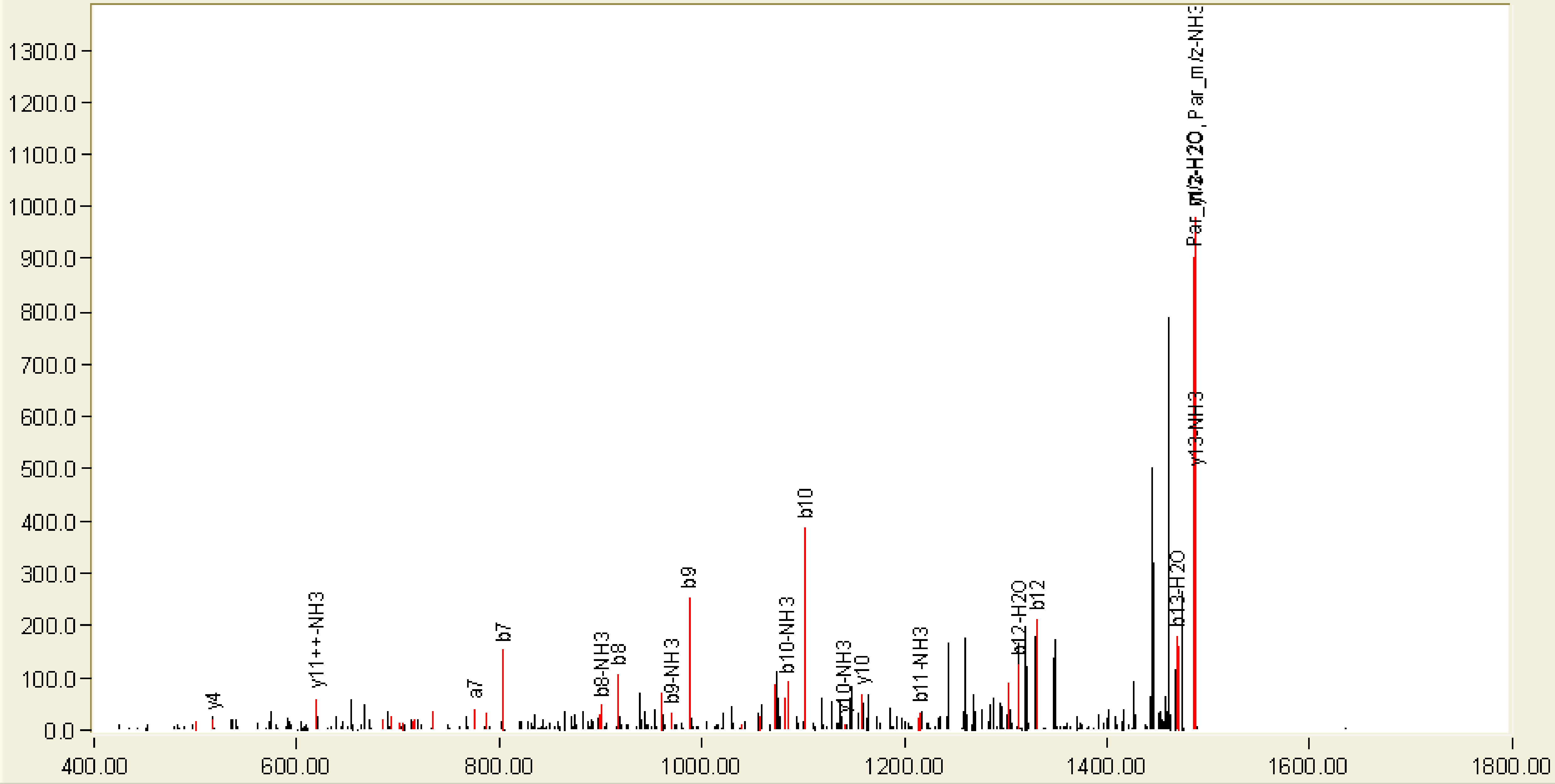

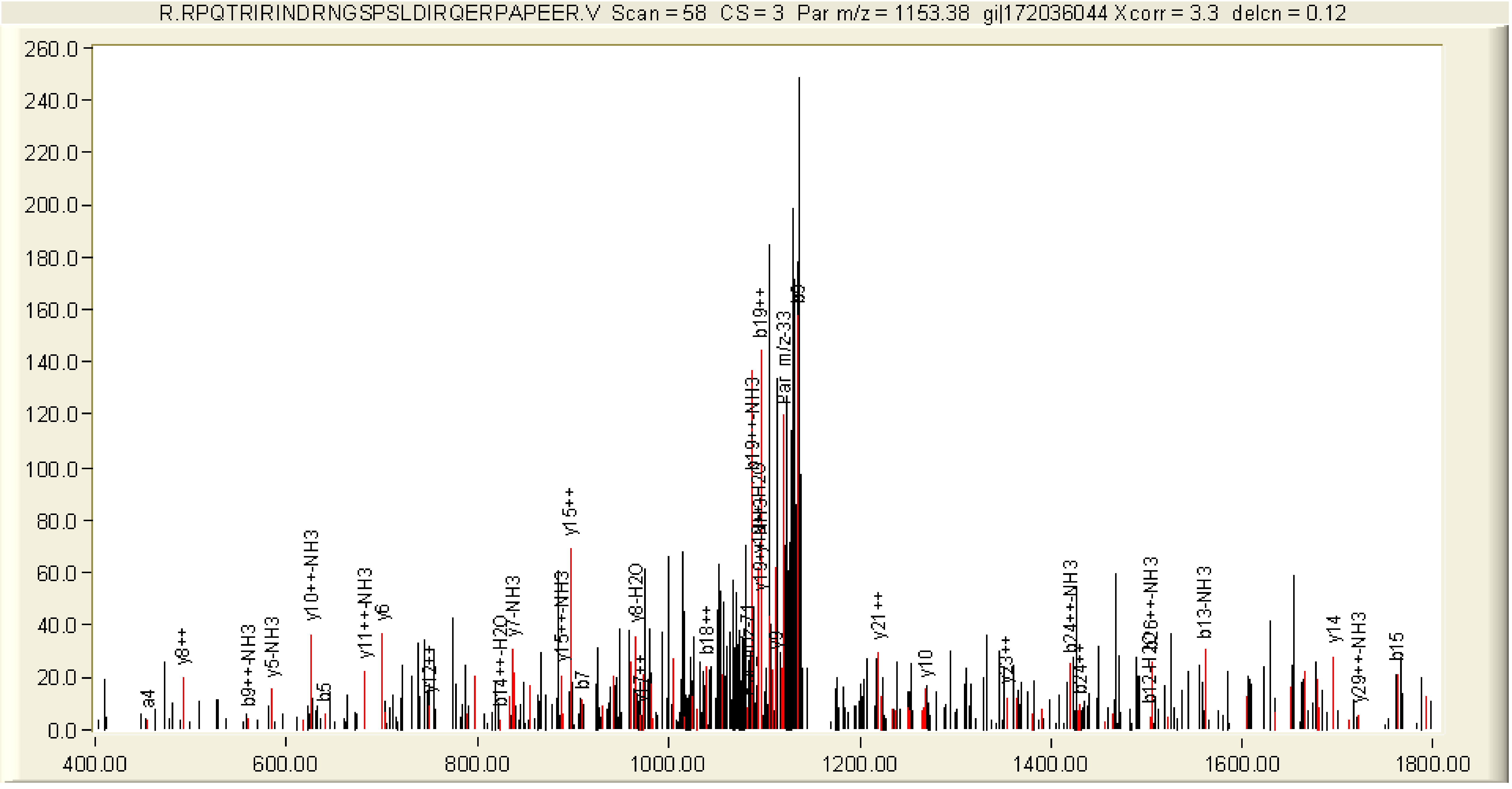
R.RPQTRIRINDRNGSPSLDIRQERPAPEER.V Scan = 58 CS = 3 Par m/z = 1153.38 gi|172036044 Xcorr = 3.3 delcn = 0.12
260.0
240.0
220.0
200.0
180.0
160.0
140.0
120.0
100.0
80.0
60.0
40.0
20.0
0.0
400.00
600.00
800.00
1000.00
1200.00
1400.00
1600.00
1800.00
a4
y8++
b9++-NH3
y5-NH3
y10++-NH3
b5
y11++-NH3
y6
y7-NH3
y15++
y15++-NH3
b7
y8-H2O
b18++
b19++
Par m/z-33
y9
y21++
y10
b24++-NH3
b13-NH3
y14
y29++-NH3
b15

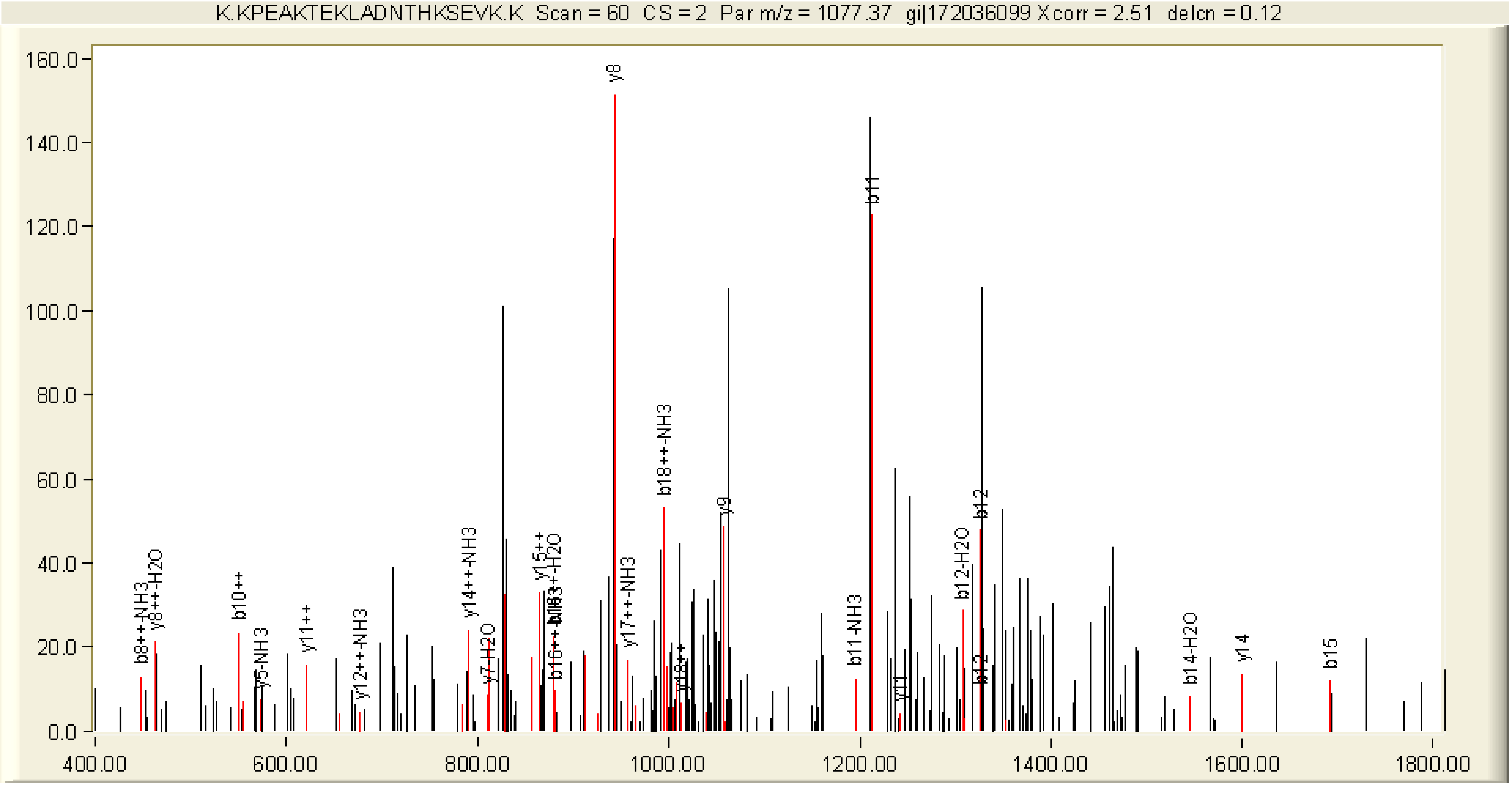
K.KPEAKTEKLADNTHKSEVK.K Scan = 60 CS = 2 Par m/z = 1077.37 gi|172036099 Xcorr = 2.51 delcn = 0.12
y8
b11
b18++-NH3
y9
b12
b12-H2O
y14++-NH3
y15++
y7-H2O
y17++-NH3
b10++
b8++-NH3
y8++-H2O
y5-NH3
y11++
y12++-NH3
b11-NH3
b14-H2O
y14
b15
y11
160.0
140.0
120.0
100.0
80.0
60.0
40.0
20.0
0.0
400.00
600.00
800.00
1000.00
1200.00
1400.00
1600.00
1800.00

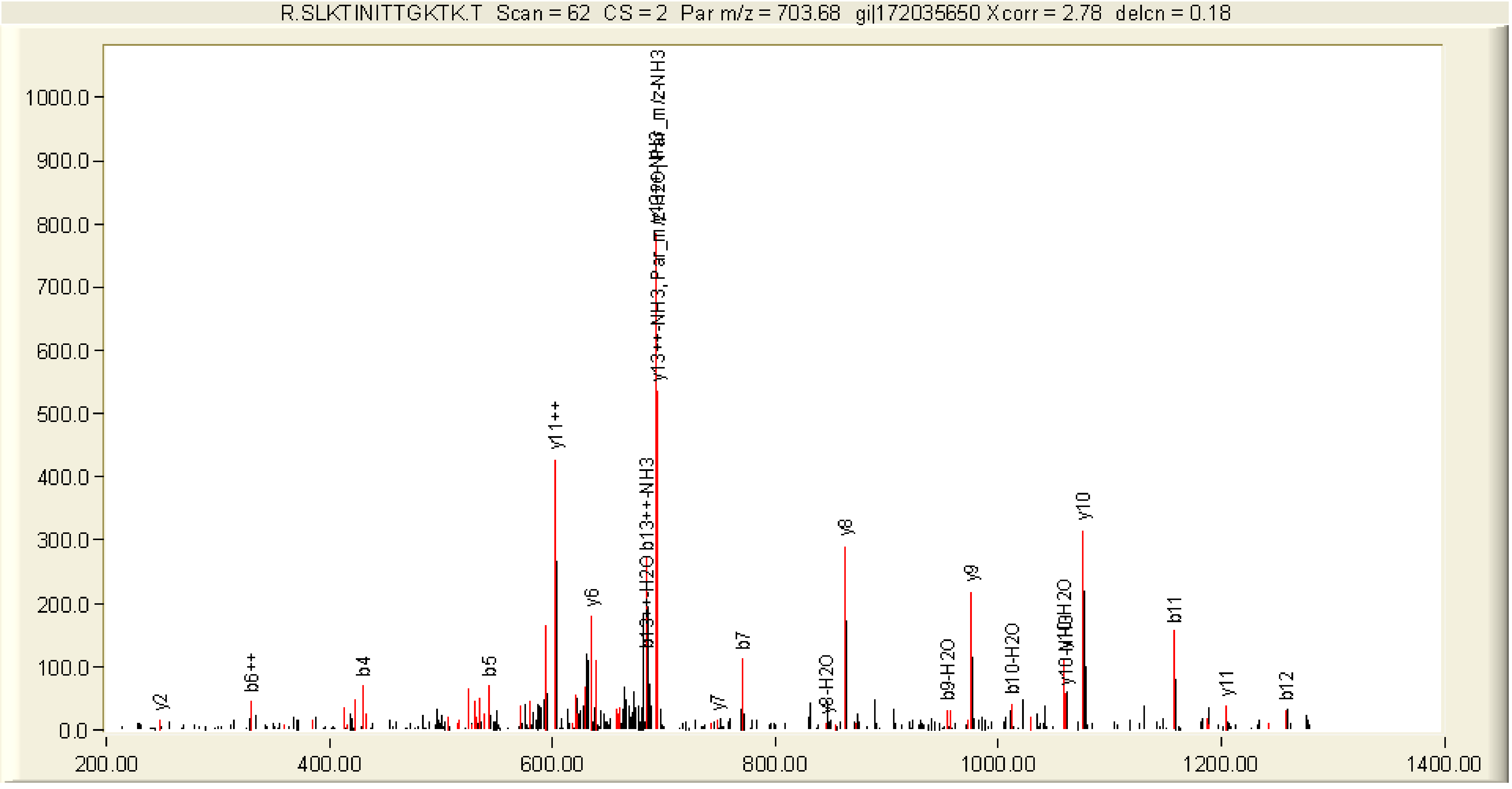
R.SLKTINITTGKTK.T Scan = 62 CS = 2 Par m/z = 703.68 gi|172035650 Xcorr = 2.78 delcn = 0.18
y2
b6++
b4
b5
y11++
y6
b13++-NH3
y13++-NH3, Par_m/z-NH3
y7
b7
y8-H2O
y8
b9-H2O
y9
b10-H2O
y10
b11
y11
b12
0.0
100.0
200.0
300.0
400.0
500.0
600.0
700.0
800.0
900.0
1000.0
200.00
400.00
600.00
800.00
1000.00
1200.00
1400.00

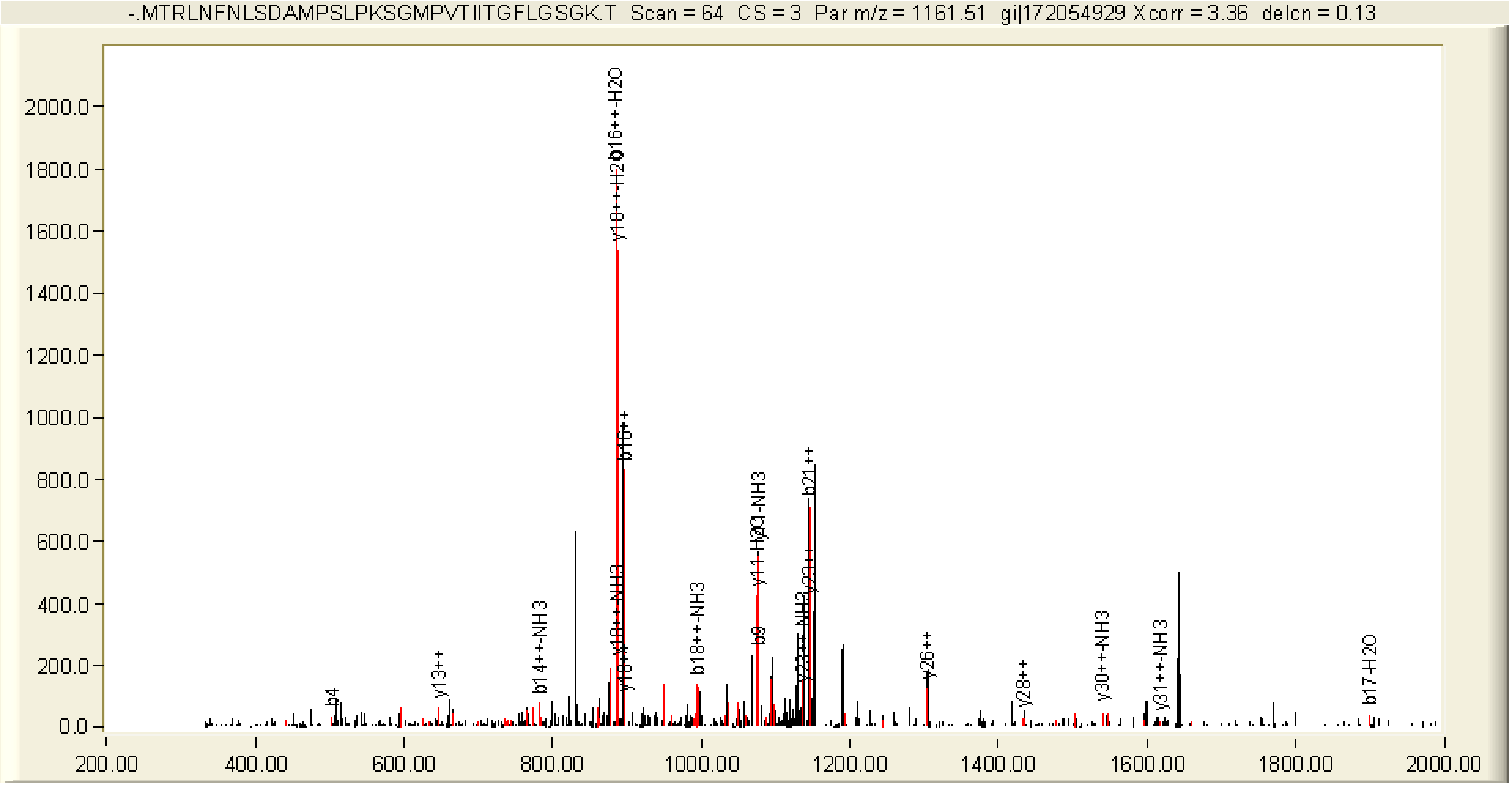

-.MTRLNFNLSDAMPSLPKSGMPVTIITGFLGSGK.T Scan = 64 CS = 3 Par m/z = 1161.51 gi|172054929 Xcorr = 3.36 delcn = 0.13
2000.0
1800.0
1600.0
1400.0
1200.0
1000.0
800.0
600.0
400.0
200.0
0.0
200.00
400.00
600.00
800.00
1000.00
1200.00
1400.00
1600.00
1800.00
2000.00
b16++-H2O
b16++
b21++
b4
y13++
b14++-NH3
b18++-NH3
b9
y26++
y28++
y30++-NH3
y31++-NH3
b17-H2O

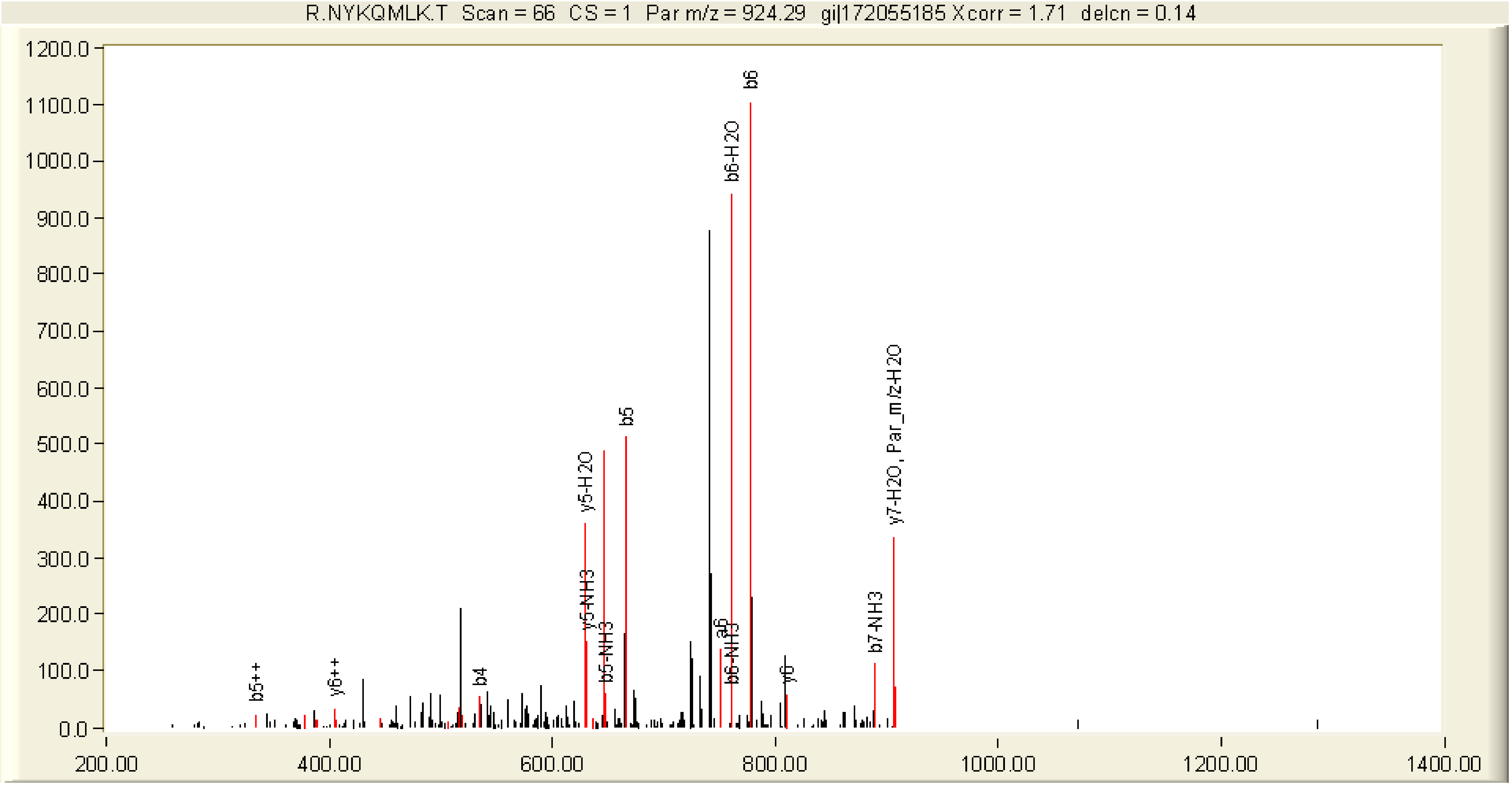
R.NYKQMLK.T Scan = 66 CS = 1 Par m/z = 924.29 gi|172055185 Xcorr = 1.71 delcn = 0.14
1200.0
1100.0
1000.0
900.0
800.0
700.0
600.0
500.0
400.0
300.0
200.0
100.0
0.0
200.00
400.00
600.00
800.00
1000.00
1200.00
1400.00
b6
b6-H2O
b5
y5-H2O
y7-H2O, Par_m/z-H2O
y5-NH3
b5-NH3
a6
b6-NH3
b7-NH3
y6
b4
b5++
y6++

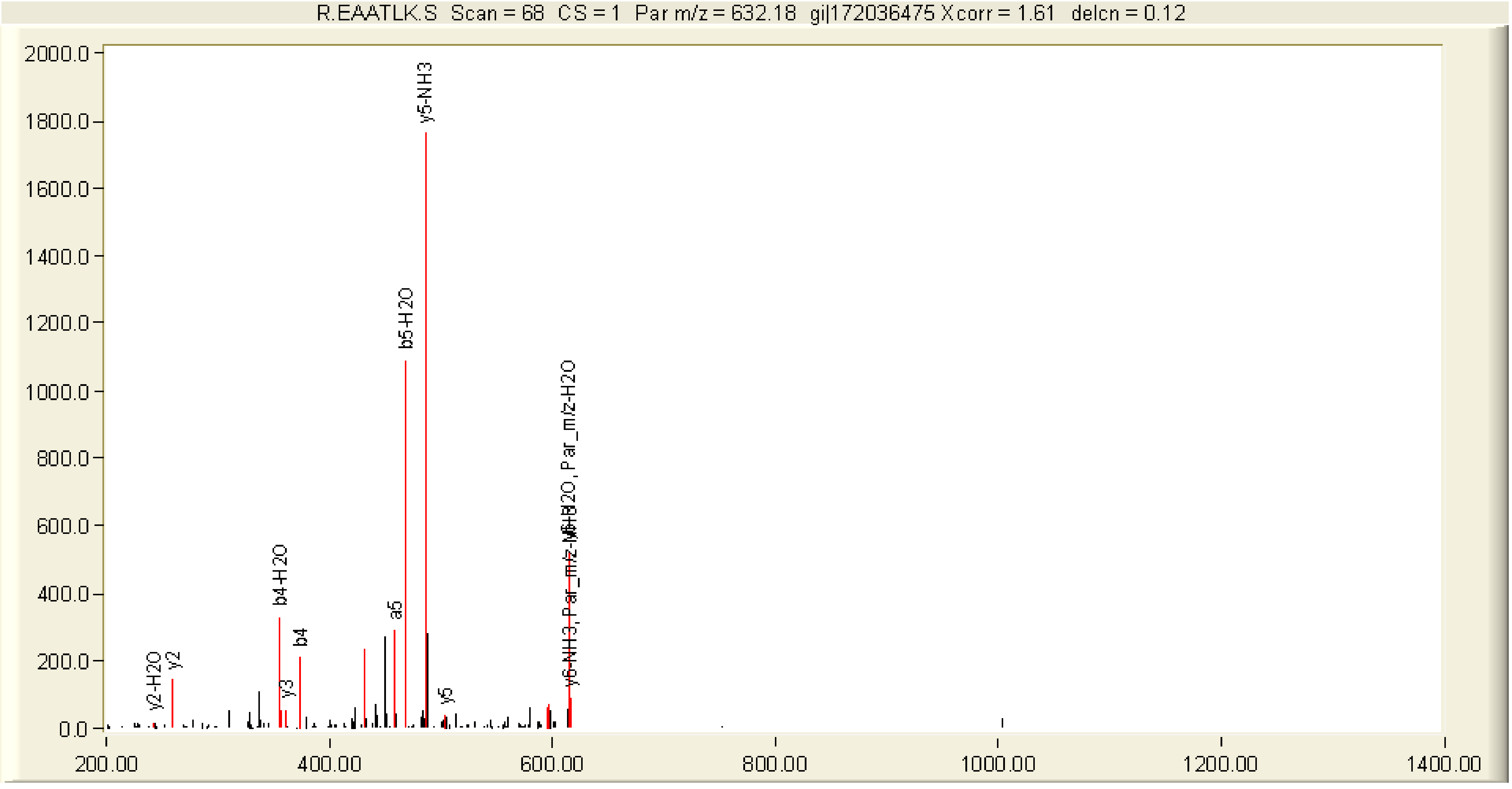

R.EAATLK.S Scan = 68 CS = 1 Par m/z = 632.18 gi|172036475 Xcorr = 1.61 delcn = 0.12
y5-NH3
b5-H2O
y6-NH3, Par_m/z-NH3
y6-H2O, Par_m/z-H2O
b4-H2O
a5
b4
y2-H2O
y2
y3
y5
2000.0
1800.0
1600.0
1400.0
1200.0
1000.0
800.0
600.0
400.0
200.0
0.0
200.00
400.00
600.00
800.00
1000.00
1200.00
1400.00

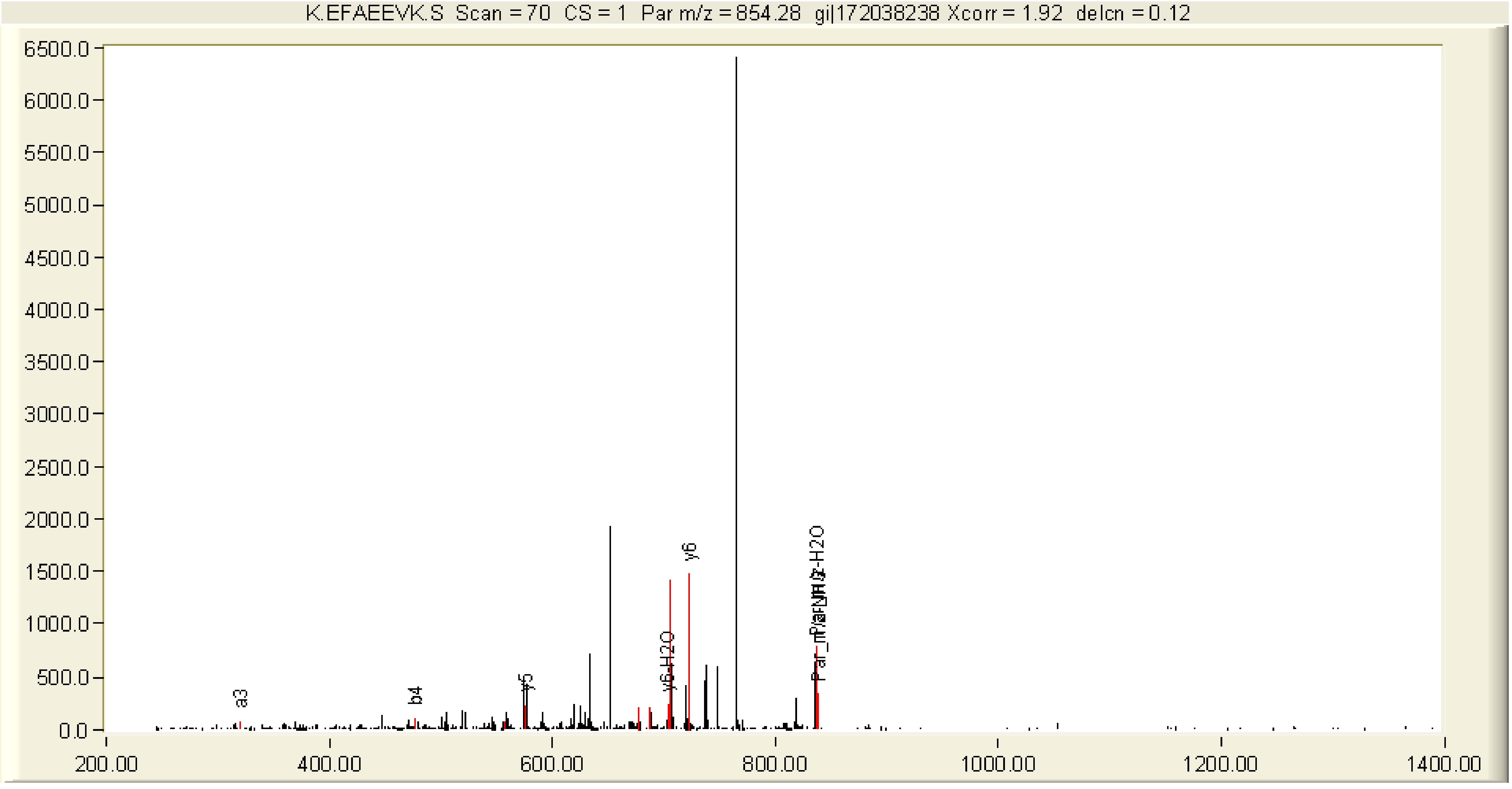
K.EFAEEVK.S Scan = 70 CS = 1 Par m/z = 854.28 gi|172038238 Xcorr = 1.92 delcn = 0.12
6500.0
6000.0
5500.0
5000.0
4500.0
4000.0
3500.0
3000.0
2500.0
2000.0
1500.0
1000.0
500.0
0.0
200.00
400.00
600.00
800.00
1000.00
1200.00
1400.00
a3
b4
y5
y6-H2O
y6
Par_m/z-H2O

R.LLTNSRLQLIDYPEVFALGDLAQISNKKK.S Scan = 72 CS = 3 Par m/z = 1097.81 gi|172038965 Xcorr = 3.2 delcn = 0.15

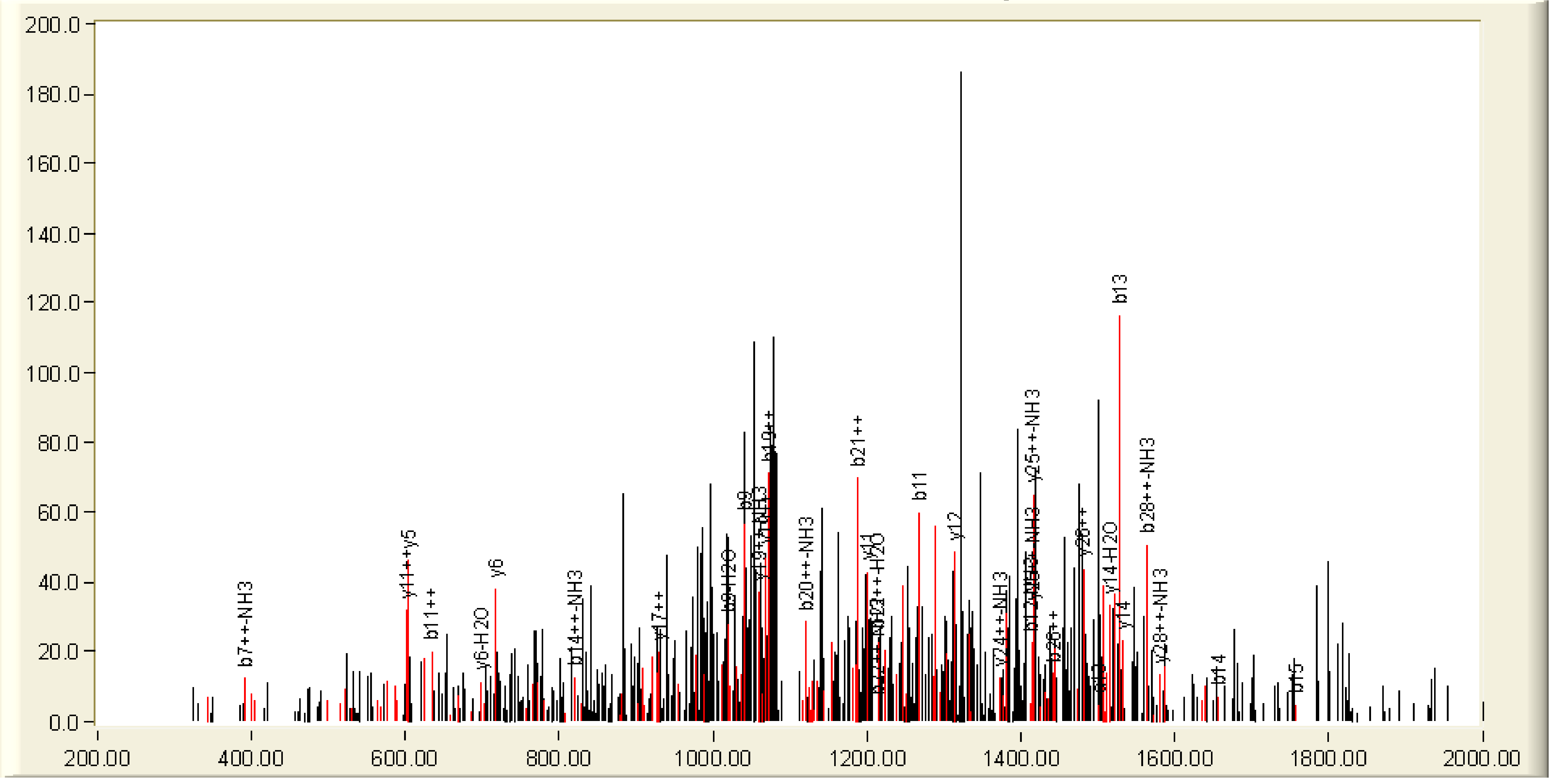

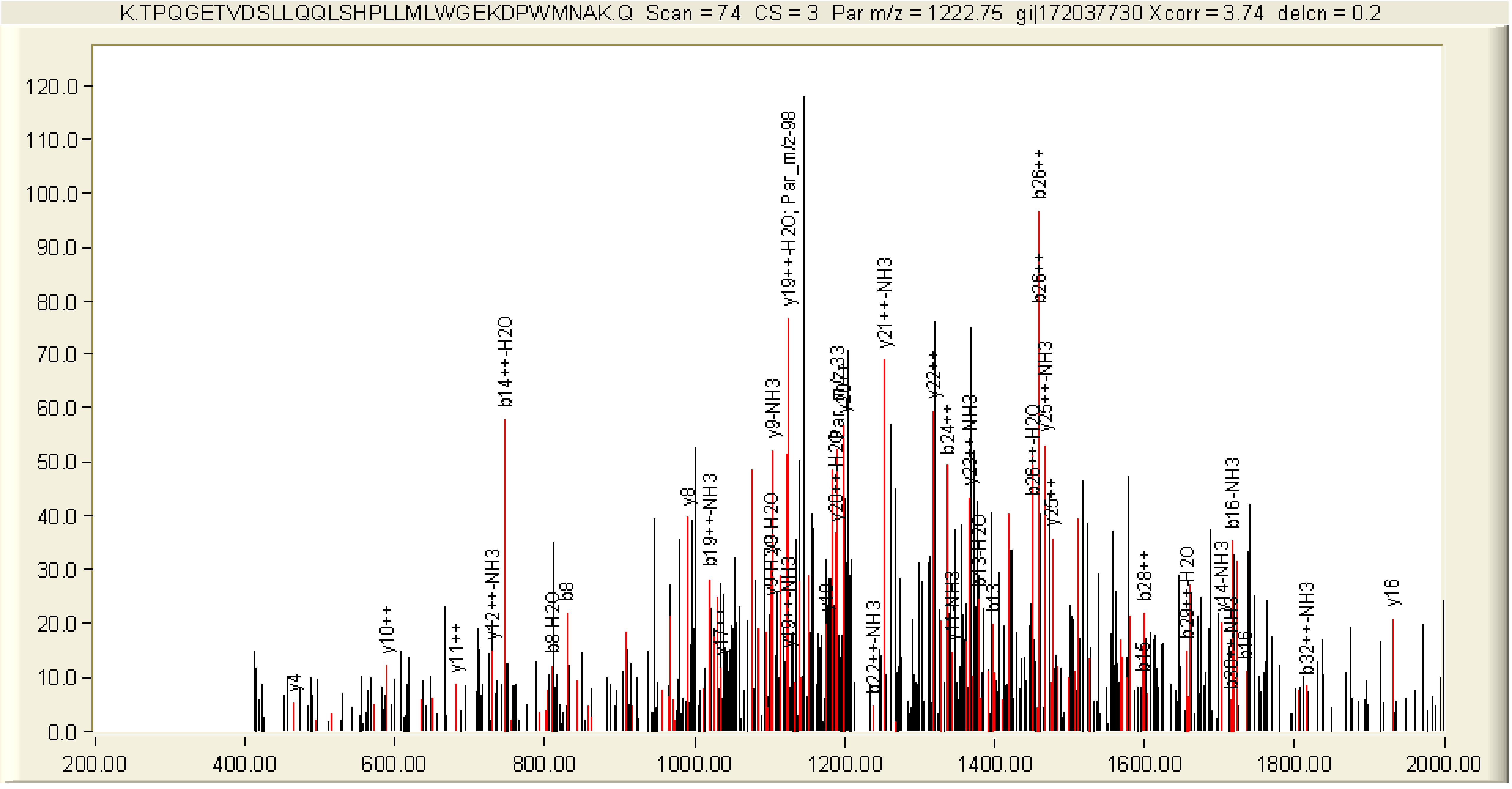
K.TPQGETVDSLLQQLSHPLLMLWGEKDPWMNAK.Q Scan = 74 CS = 3 Par m/z = 1222.75 gi|172037730 Xcorr = 3.74 delcn = 0.2
y19++-H2O; Par_m/z-98
b26++
y21++-NH3
b14++-H2O
y9-NH3
y22++
b24++
y25++-NH3
b26++-H2O
y8
b19++-NH3
b16-NH3
y25++
y12++-NH3
b28++
b8-H2O
b8
y10++
y11++
b22++-NH3
b32++-NH3
y16
y4
b15
b16
0.0
10.0
20.0
30.0
40.0
50.0
60.0
70.0
80.0
90.0
100.0
110.0
120.0
200.00
400.00
600.00
800.00
1000.00
1200.00
1400.00
1600.00
1800.00
2000.00

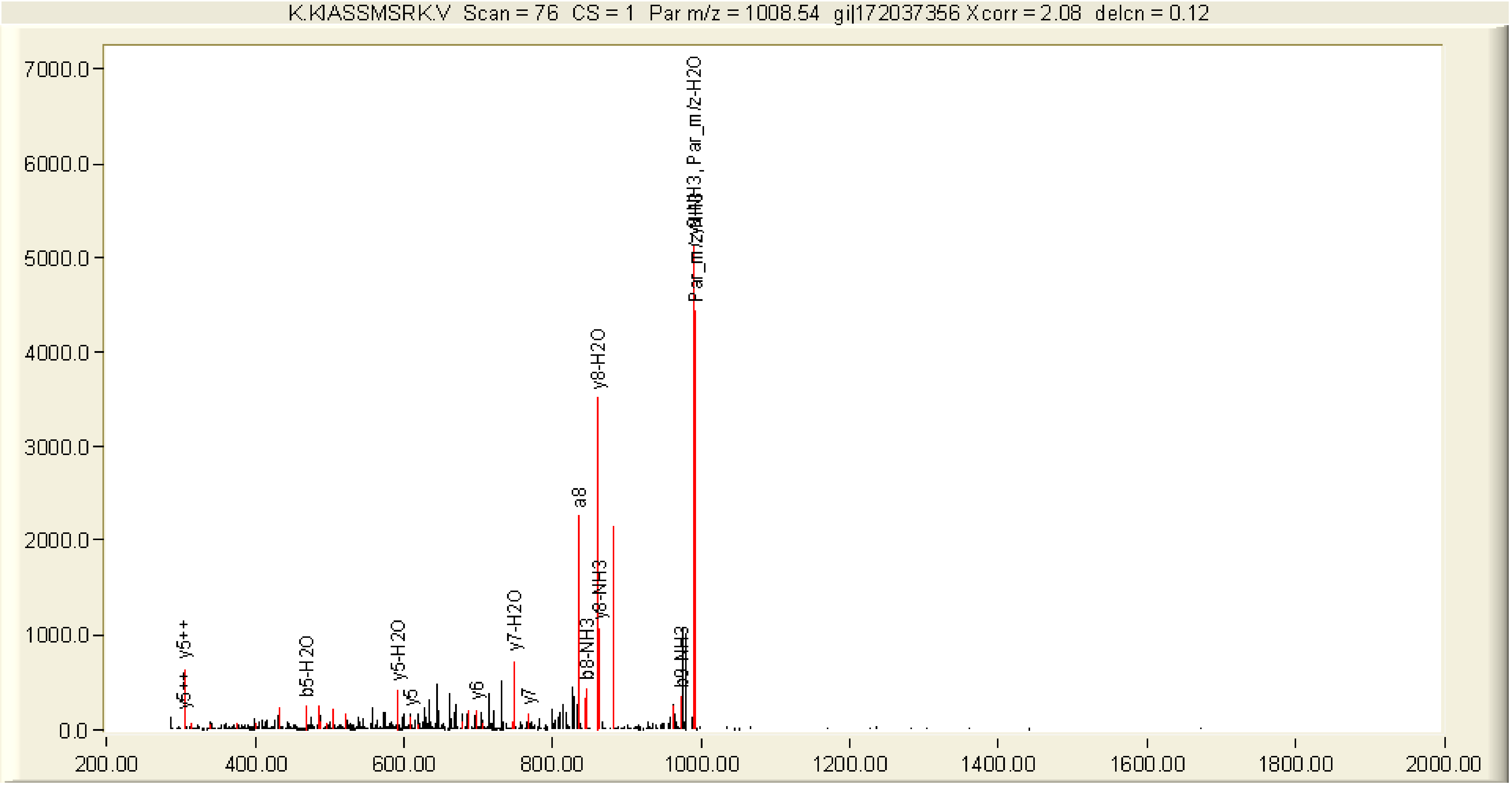
K.KIASSMSRK.V Scan = 76 CS = 1 Par m/z = 1008.54 gi|172037356 Xcorr = 2.08 delcn = 0.12
7000.0
6000.0
5000.0
4000.0
3000.0
2000.0
1000.0
0.0
200.00
400.00
600.00
800.00
1000.00
1200.00
1400.00
1600.00
1800.00
2000.00
Par_m/z-H2O
y8-H2O
a8
y8-NH3
b8-NH3
y7-H2O
y7
y6
y5
y5-H2O
b5-H2O
y5++

R.ALEHTIGIVAR.Q Scan = 78 CS = 2 Par m/z = 590.96 gi|172034960 Xcorr = 2.71 delcn = 0.47

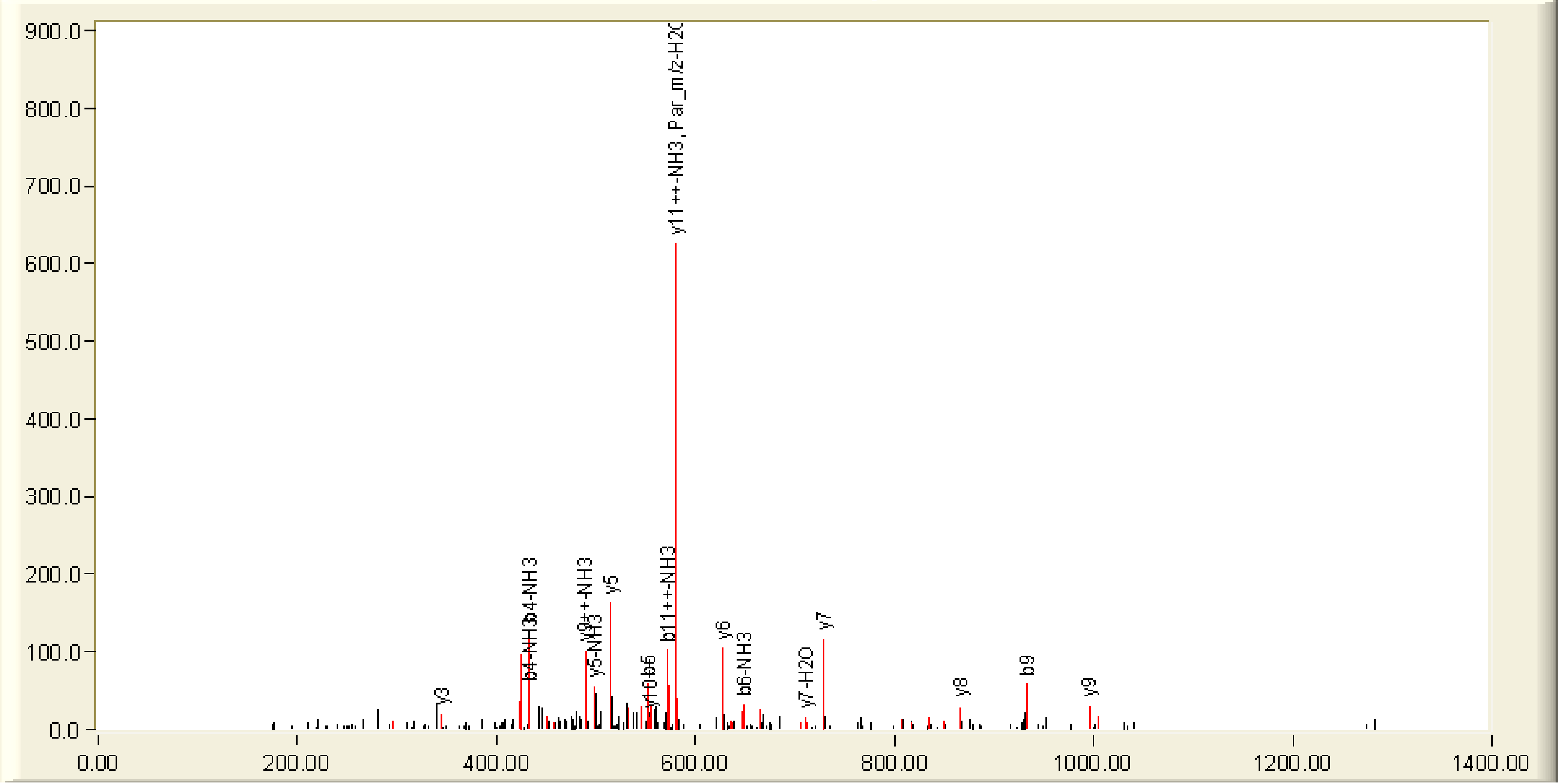

K.TLQKRHKIDGVRFSRLLR.T Scan = 80 CS = 2 Par m/z = 1113.37 gi|172038891 Xcorr = 2.69 delcn = 0.26

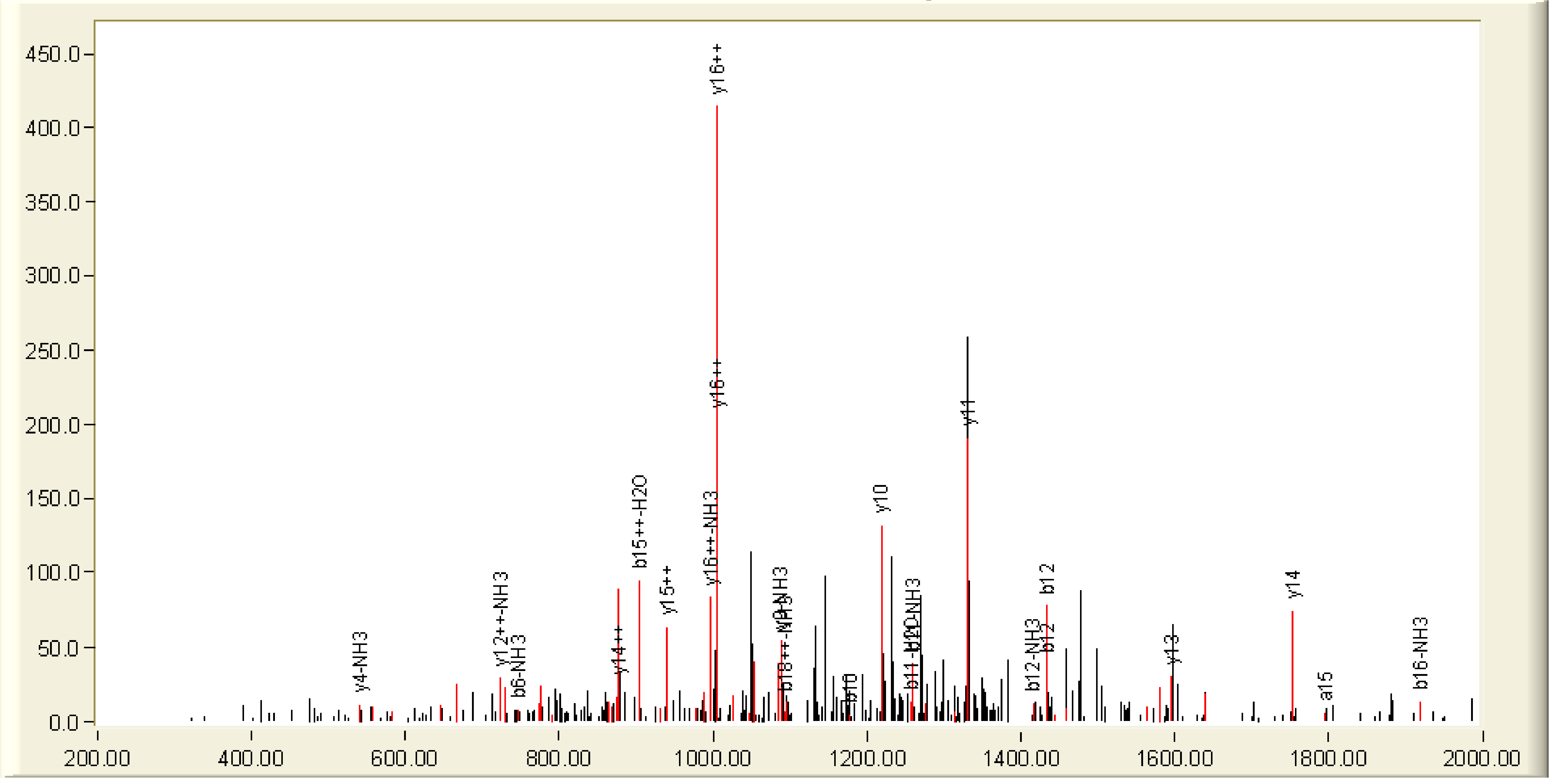

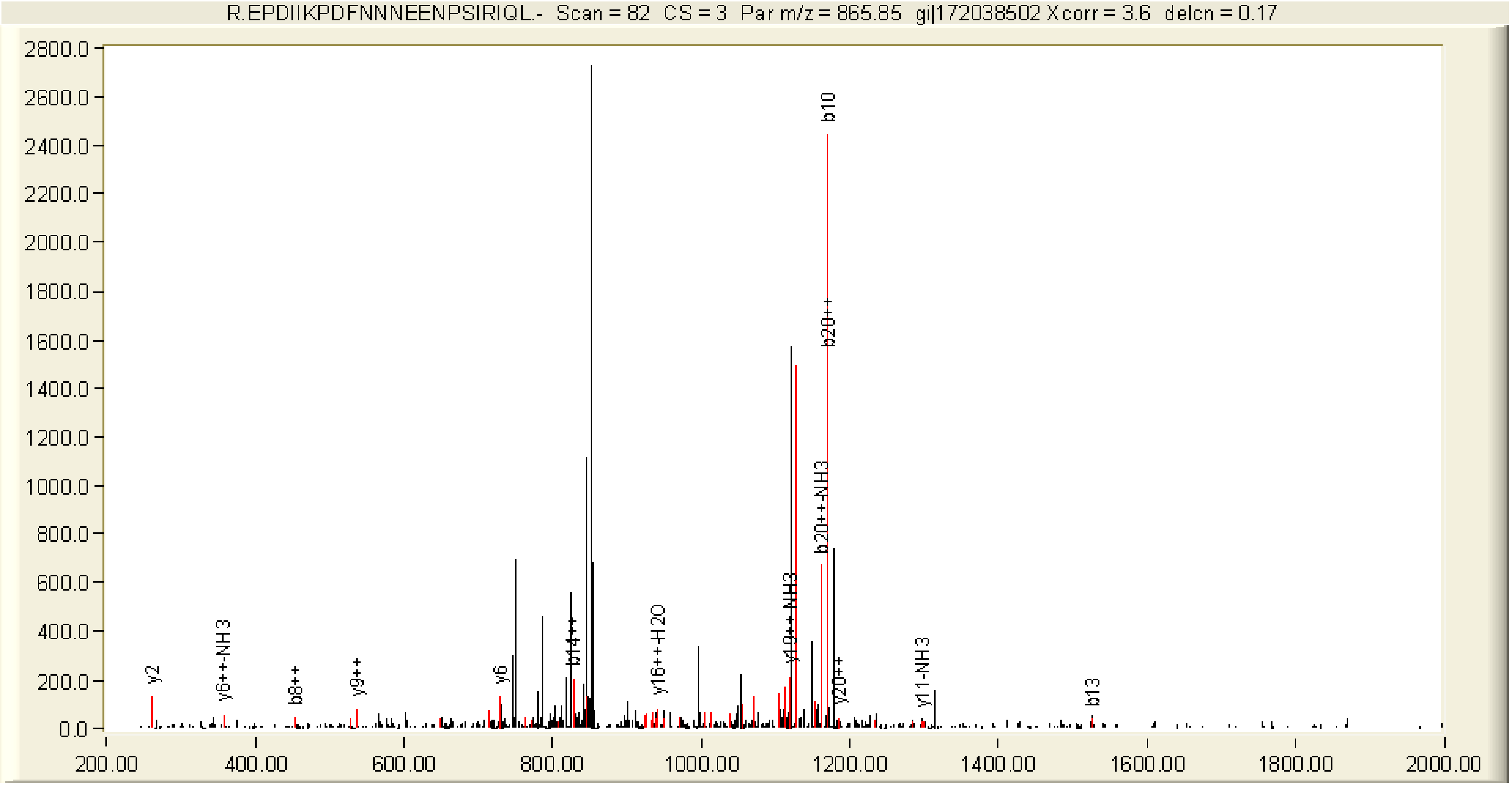
R.EPDIIKPDFNNNEENPSIRIQL.- Scan = 82 CS = 3 Par m/z = 865.85 gi|172038502 Xcorr = 3.6 delcn = 0.17
2800.0
2600.0
2400.0
2200.0
2000.0
1800.0
1600.0
1400.0
1200.0
1000.0
800.0
600.0
400.0
200.0
0.0
200.00
400.00
600.00
800.00
1000.00
1200.00
1400.00
1600.00
1800.00
2000.00
y2
y6++-NH3
b8++
y9++
y6
b14++
y16++-H2O
y19++-NH3
b20++-NH3
b20++
b10
y20++
y11-NH3
b13

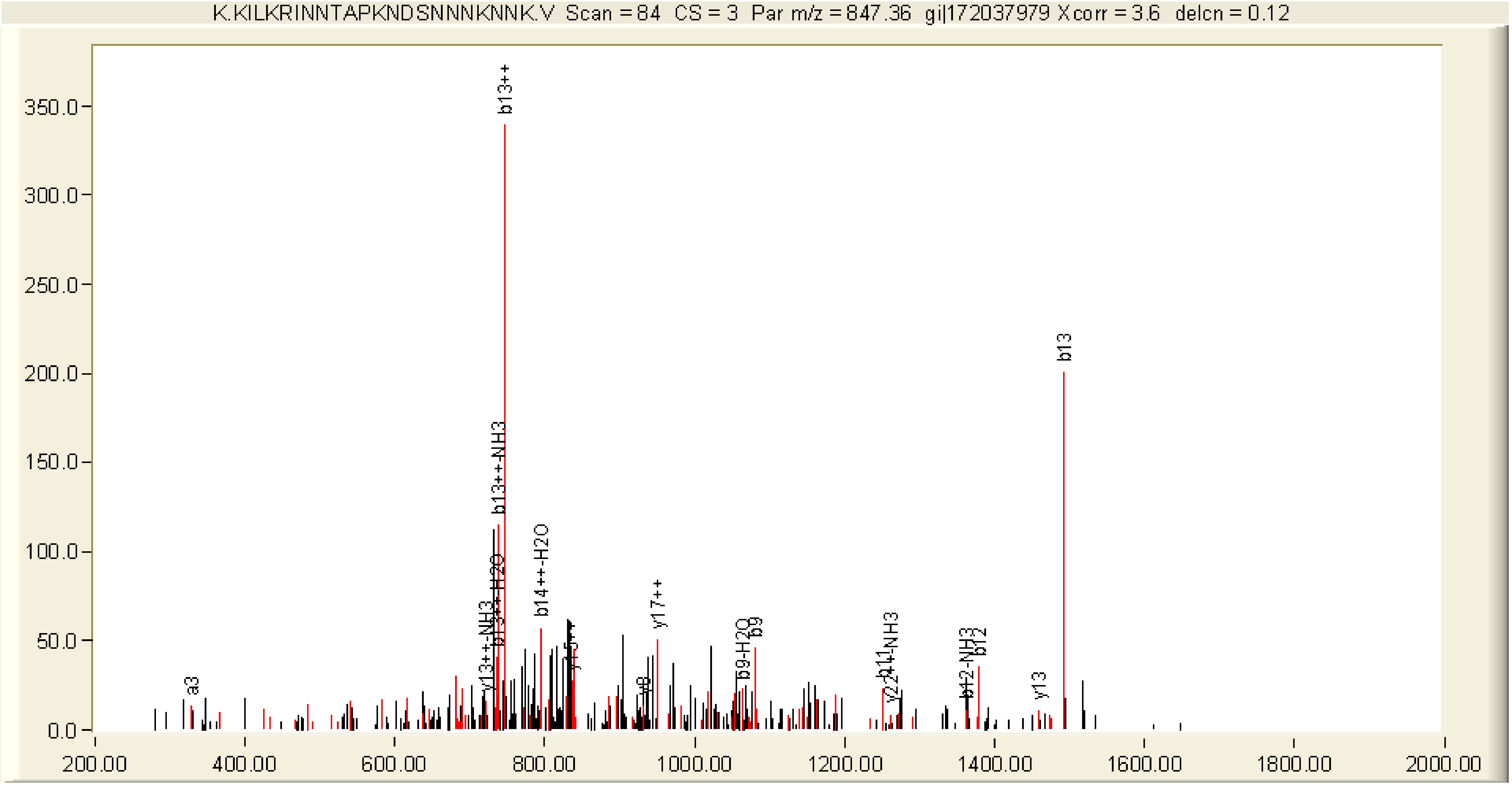
K.KILKRINNTAPKNDSNNNKNNK.V Scan = 84 CS = 3 Par m/z = 847.36 gi|172037979 Xcorr = 3.6 delcn = 0.12
b13++
b13++-NH3
b13++-H2O
y13++-NH3
b14++-H2O
y17++
b9-H2O
b9
y22++-NH3
b11
b12-NH3
b12
y13
b13
a3
y8
0.0
50.0
100.0
150.0
200.0
250.0
300.0
350.0
200.00
400.00
600.00
800.00
1000.00
1200.00
1400.00
1600.00
1800.00
2000.00

K.KANWTCQKCNRKCIEPGQVIPKDWTVSK.R Scan = 86 CS = 3 Par m/z = 1087.46 gi|172054994 Xcorr = 3.3 delcn = 0.21

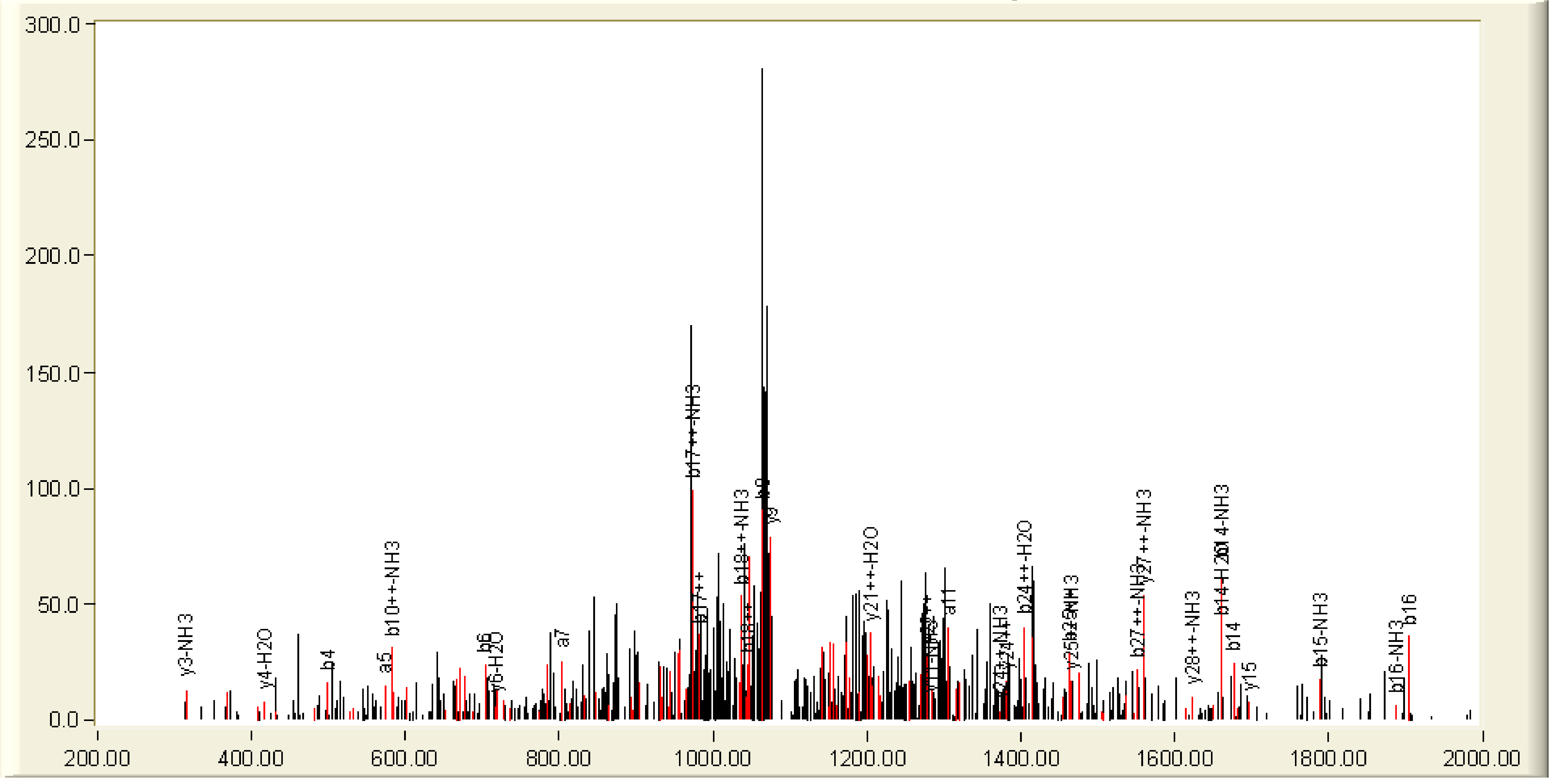

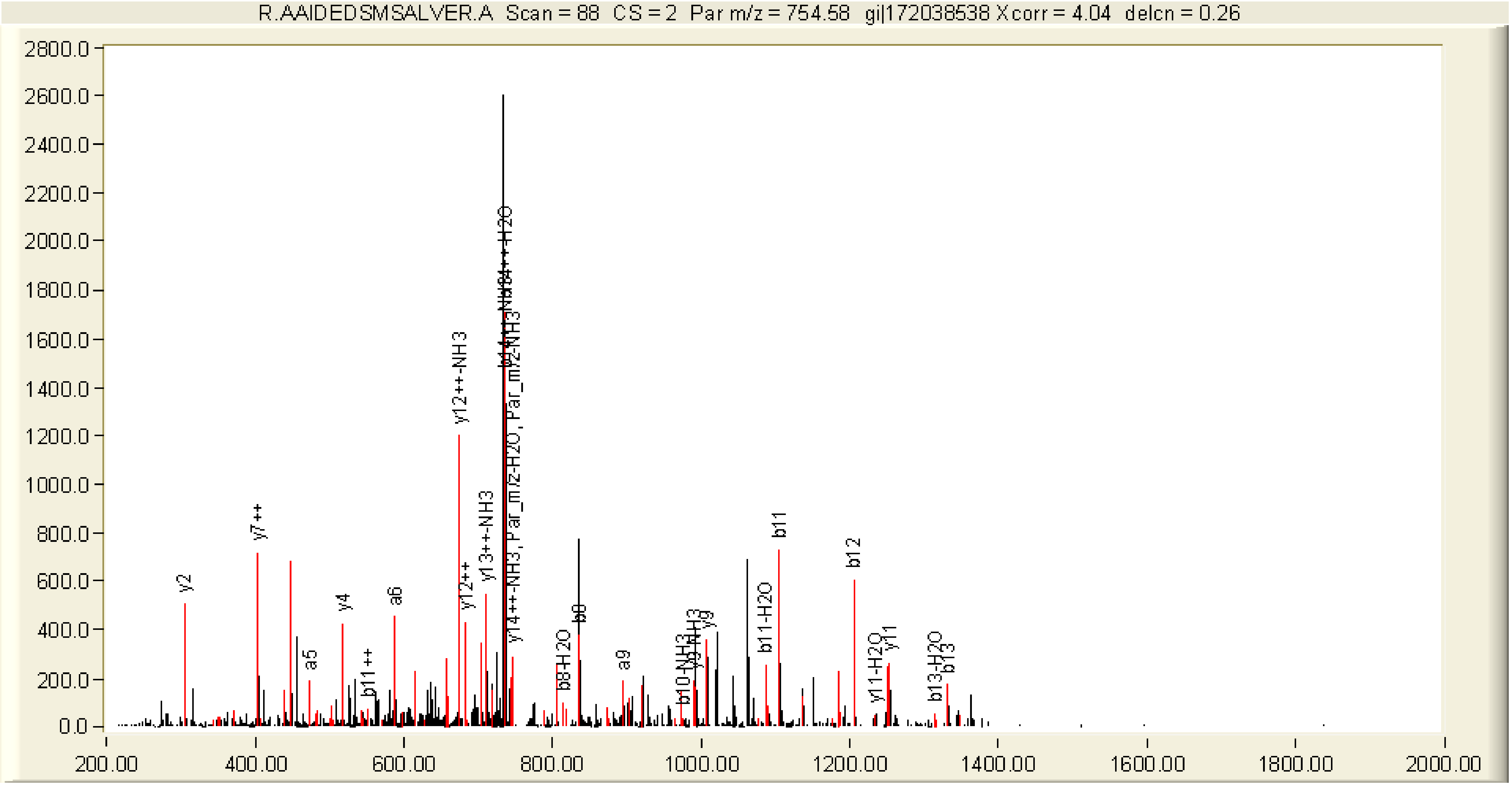

R.AAIDEDSMSALVER.A Scan = 88 CS = 2 Par m/z = 754.58 gi|172038538 Xcorr = 4.04 delcn = 0.26
2800.0
2600.0
2400.0
2200.0
2000.0
1800.0
1600.0
1400.0
1200.0
1000.0
800.0
600.0
400.0
200.0
0.0
200.00
400.00
600.00
800.00
1000.00
1200.00
1400.00
1600.00
1800.00
2000.00
y2
y7++
a5
y4
b11++
a6
y12++-NH3
y12++
y13++-NH3
y14++-NH3, Par_m/z-H2O, Par_m/z-NH3
b8-H2O
a9
b11
b11-H2O
b12
y11-H2O
y11
b13-H2O
b13

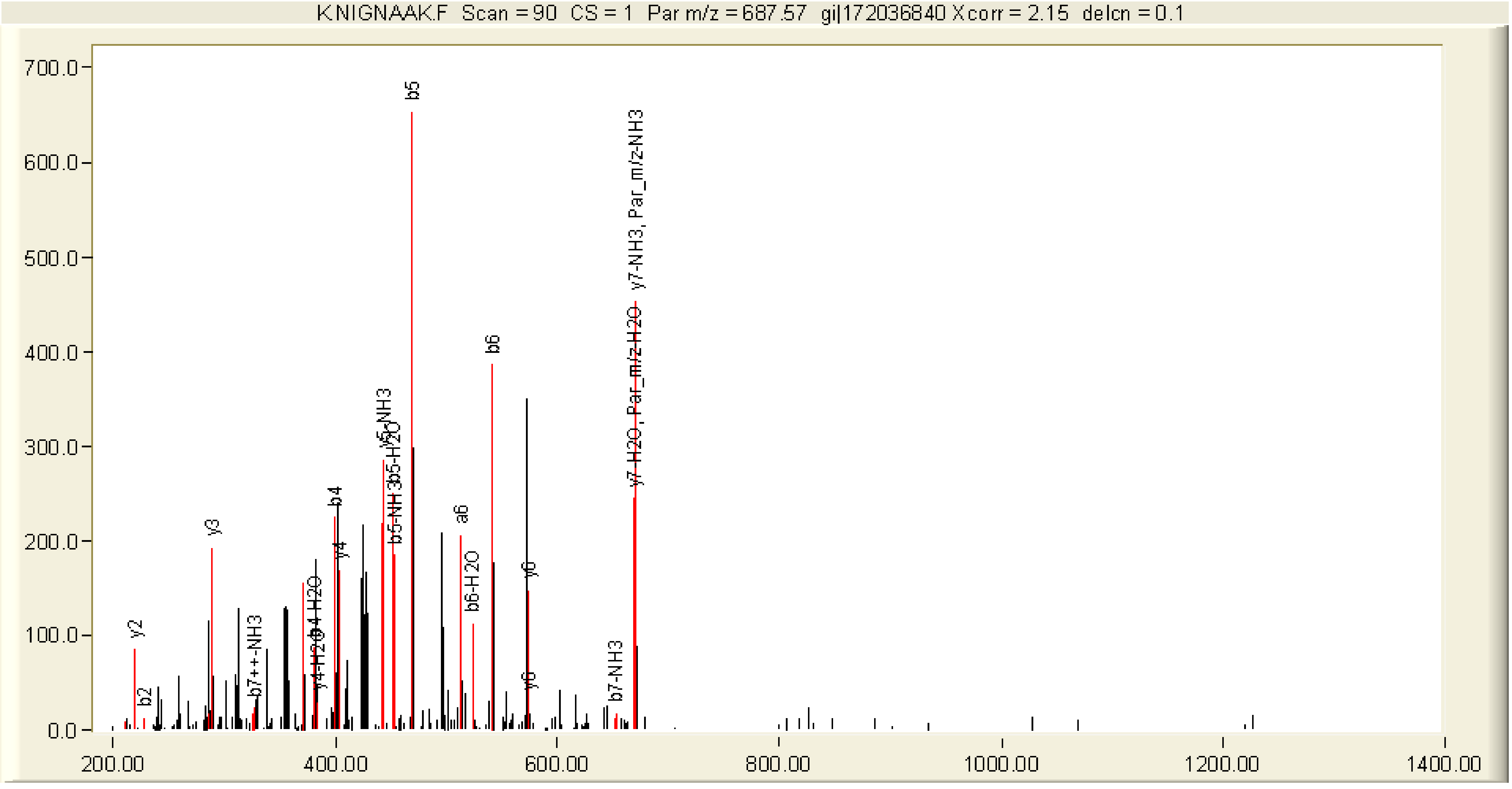

K.NIGNAAK.F Scan = 90 CS = 1 Par m/z = 687.57 gi|172036840 Xcorr = 2.15 delcn = 0.1
700.0
600.0
500.0
400.0
300.0
200.0
100.0
0.0
200.00
400.00
600.00
800.00
1000.00
1200.00
1400.00
b5
y7-NH3, Par_m/z-NH3
y7-H2O, Par_m/z-H2O
b6
y5-NH3
b5-H2O
b5-NH3
b4
y4
a6
b6-H2O
y3
y6
y2
b2
b7++-NH3
y4-H2O
y4-H2O
b7-NH3

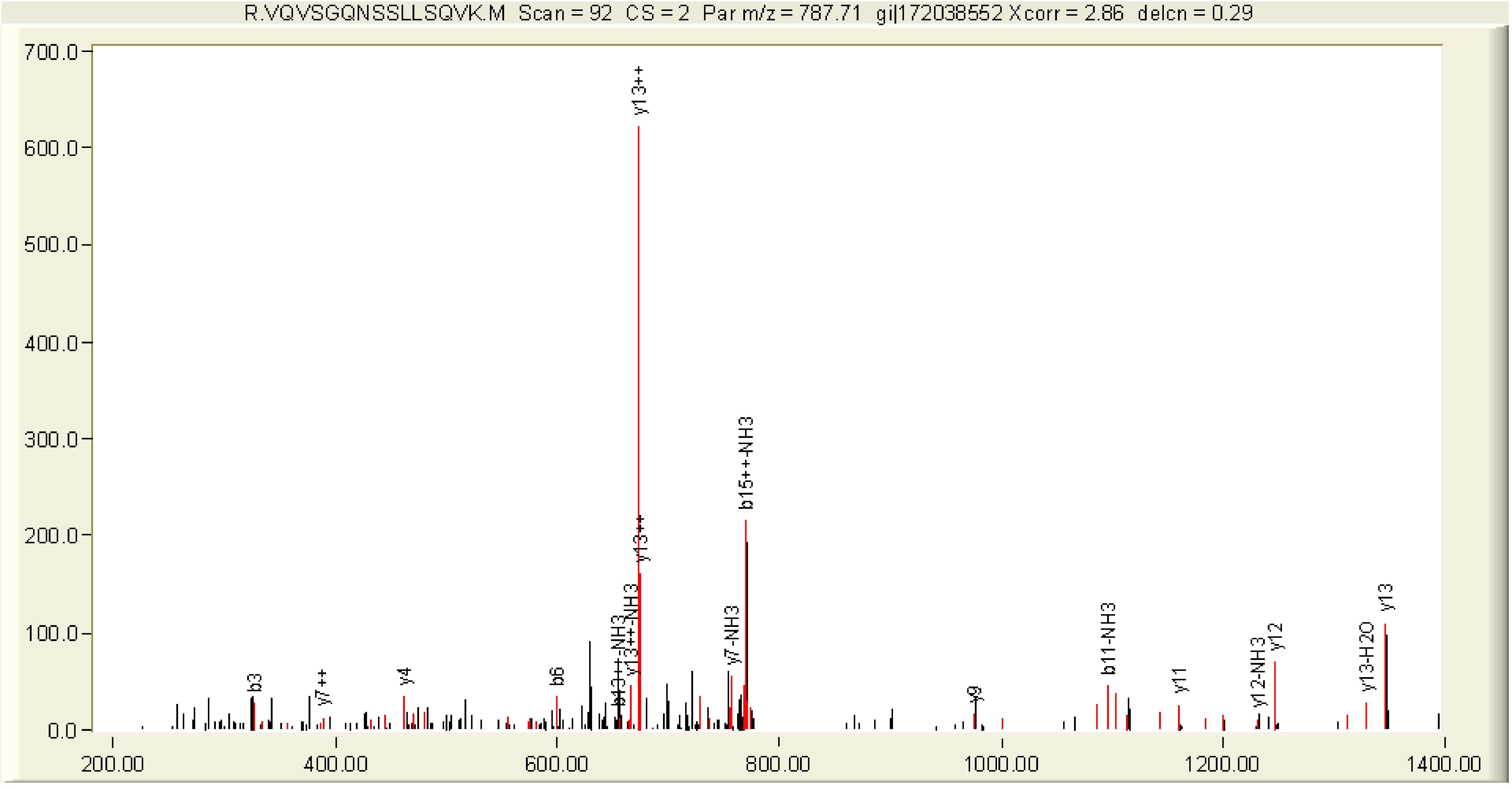
R.VQVSGQNSSLLSQVK.M Scan = 92 CS = 2 Par m/z = 787.71 gi|172038552 Xcorr = 2.86 delcn = 0.29
y13++
b15++-NH3
y13++
y13++-NH3
b13++-NH3
y7-NH3
b11-NH3
y13
y12
y12-NH3
y13-H2O
y11
y9
b6
y4
y7++
b3
700.0
600.0
500.0
400.0
300.0
200.0
100.0
0.0
200.00
400.00
600.00
800.00
1000.00
1200.00
1400.00

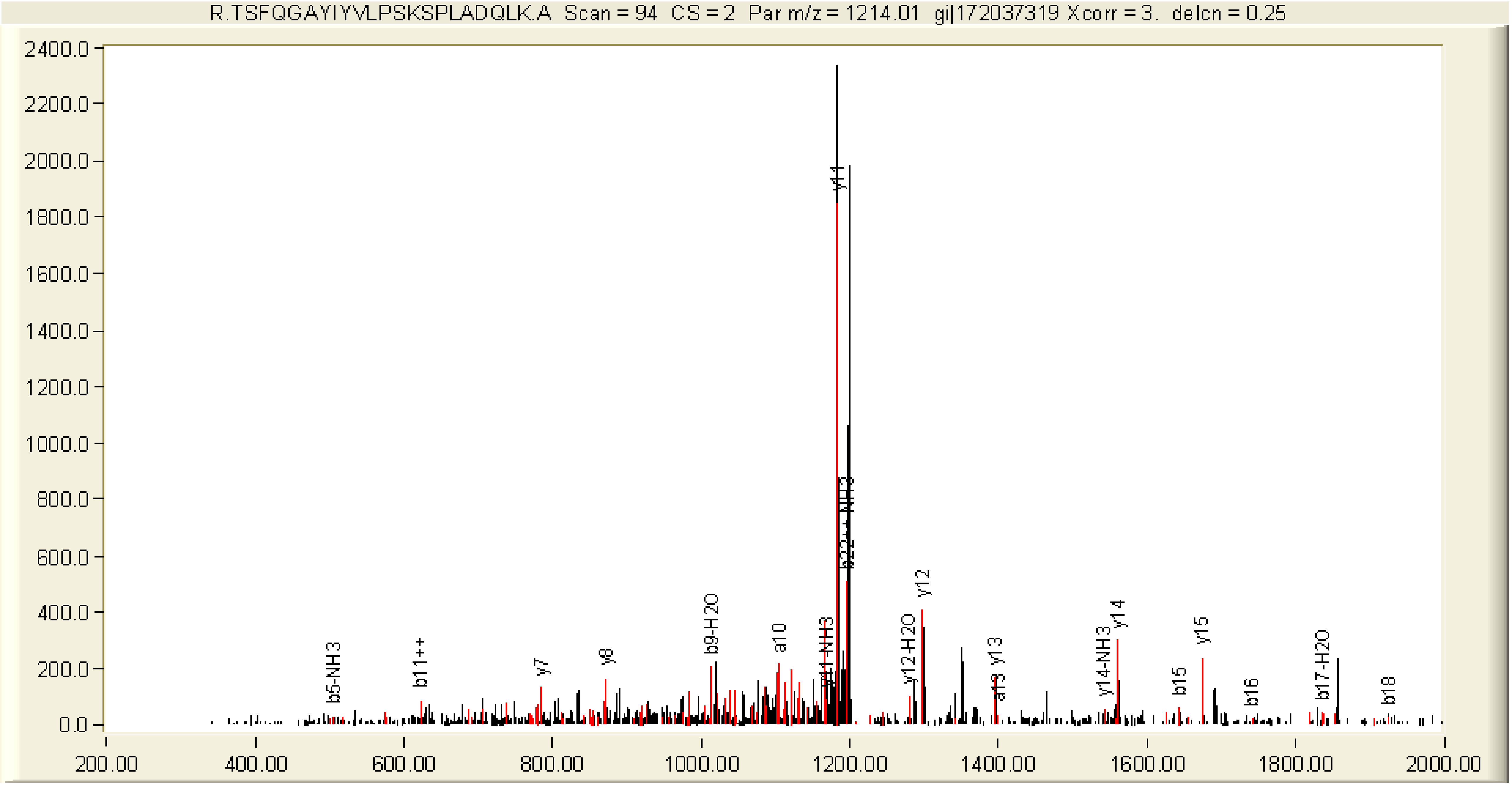

R.TSFQGAYIYVLPSKSPLADQLK.A Scan = 94 CS = 2 Par m/z = 1214.01 gi|172037319 Xcorr = 3. delcn = 0.25
2400.0
2200.0
2000.0
1800.0
1600.0
1400.0
1200.0
1000.0
800.0
600.0
400.0
200.0
0.0
200.00
400.00
600.00
800.00
1000.00
1200.00
1400.00
1600.00
1800.00
2000.00
y11
b5-NH3
b11++
y7
y8
b9-H2O
a10
y12-H2O
y12
a13
y13
y14-NH3
y14
b15
y15
b16
b17-H2O
b18

K.FLESYFDNLIKEK.S Scan = 96 CS = 2 Par m/z = 823.15 gi|172036657 Xcorr = 2.85 delcn = 0.24

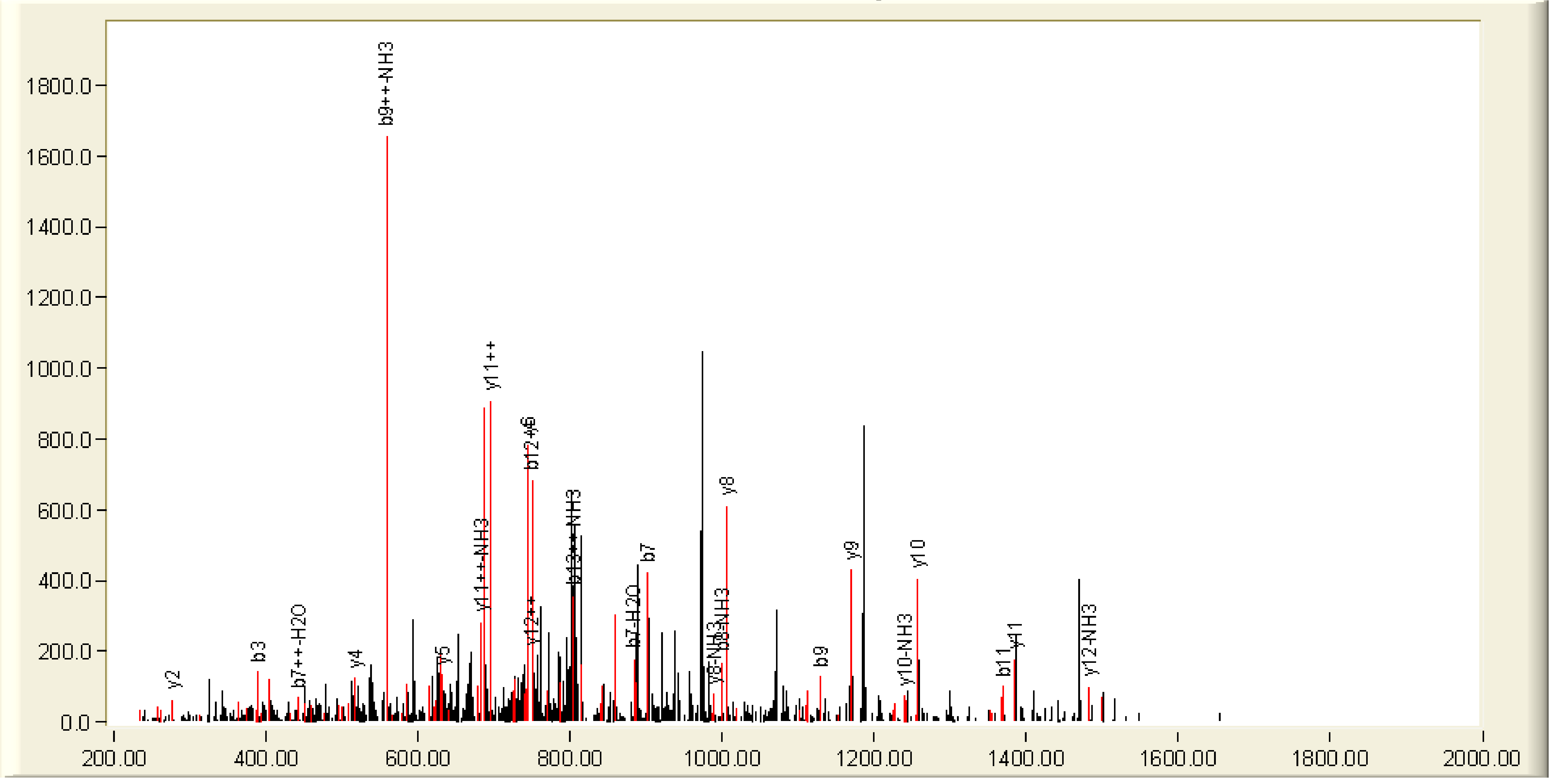

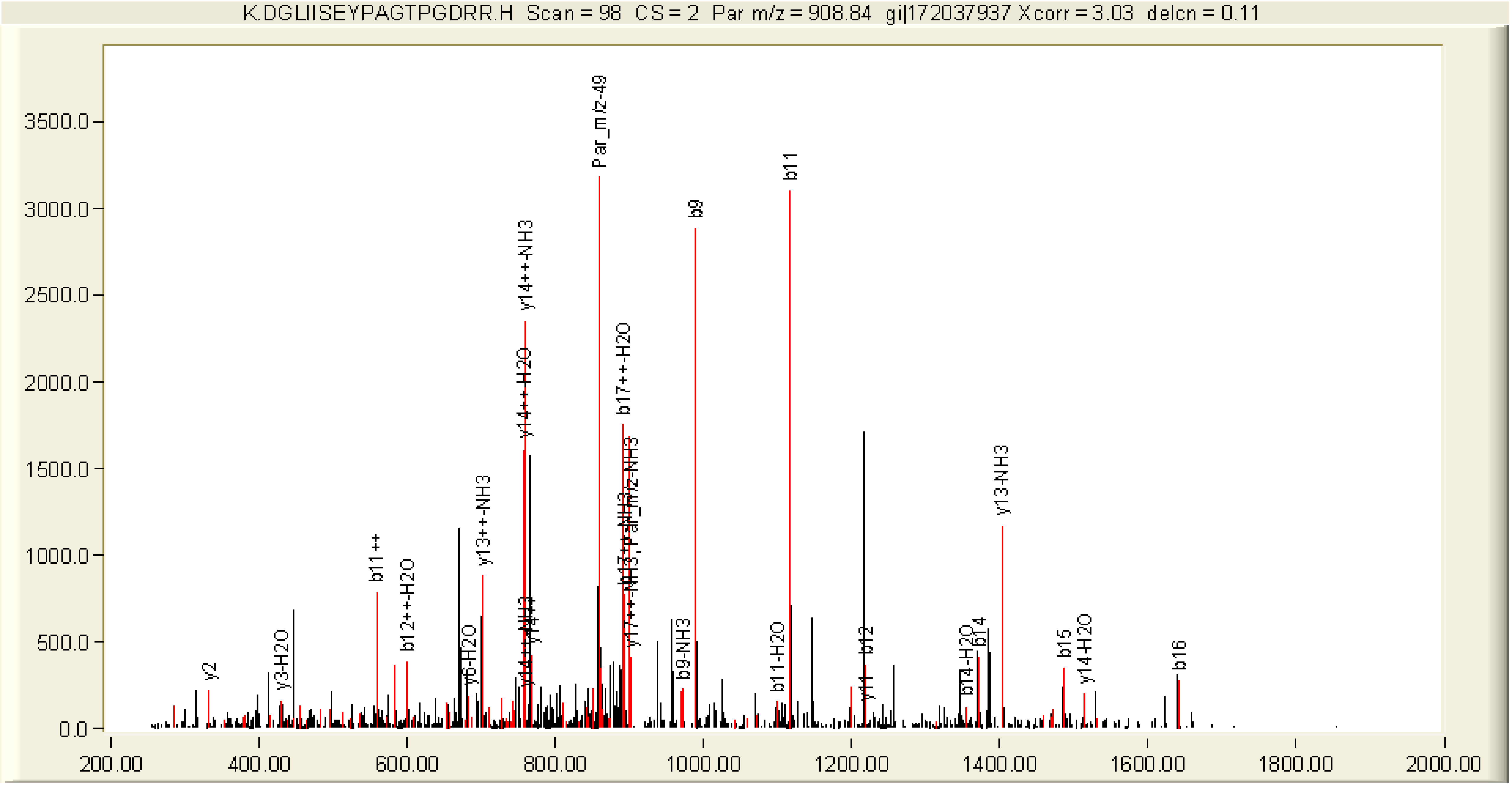

K.DGLIISEYPAGTPGDRR.H Scan = 98 CS = 2 Par m/z = 908.84 gi|172037937 Xcorr = 3.03 delcn = 0.11
Par_m/z-49
b11
b9
y14++-NH3
y14++-H2O
b17++-H2O
y13-NH3
y13++-NH3
b11++
b12++-H2O
y3-H2O
y2
y6-H2O
b9-NH3
b11-H2O
b12
y11
b14-H2O
b14
b15
y14-H2O
b16
3500.0
3000.0
2500.0
2000.0
1500.0
1000.0
500.0
0.0
200.00
400.00
600.00
800.00
1000.00
1200.00
1400.00
1600.00
1800.00
2000.00

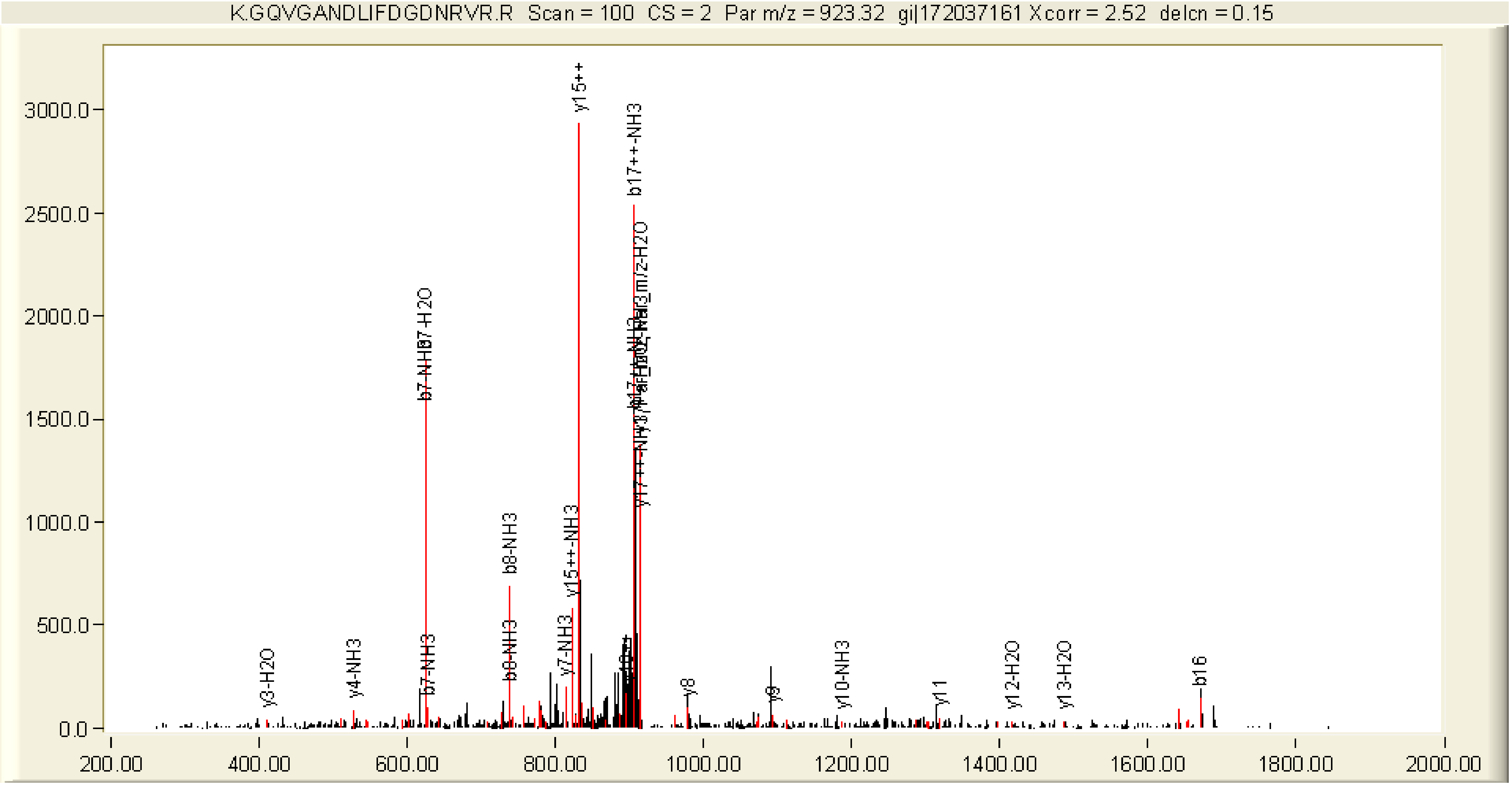
K.GGQVGANDLIFDGDNRVR.R Scan = 100 CS = 2 Par m/z = 923.32 gi|172037161 Xcorr = 2.52 delcn = 0.15
y15++
b17++-NH3
b8-NH3
y15++-NH3
y3-H2O
y4-NH3
b7-NH3
y7-NH3
y8
y9
y10-NH3
y11
y12-H2O
y13-H2O
b16
3000.0
2500.0
2000.0
1500.0
1000.0
500.0
0.0
200.00
400.00
600.00
800.00
1000.00
1200.00
1400.00
1600.00
1800.00
2000.00

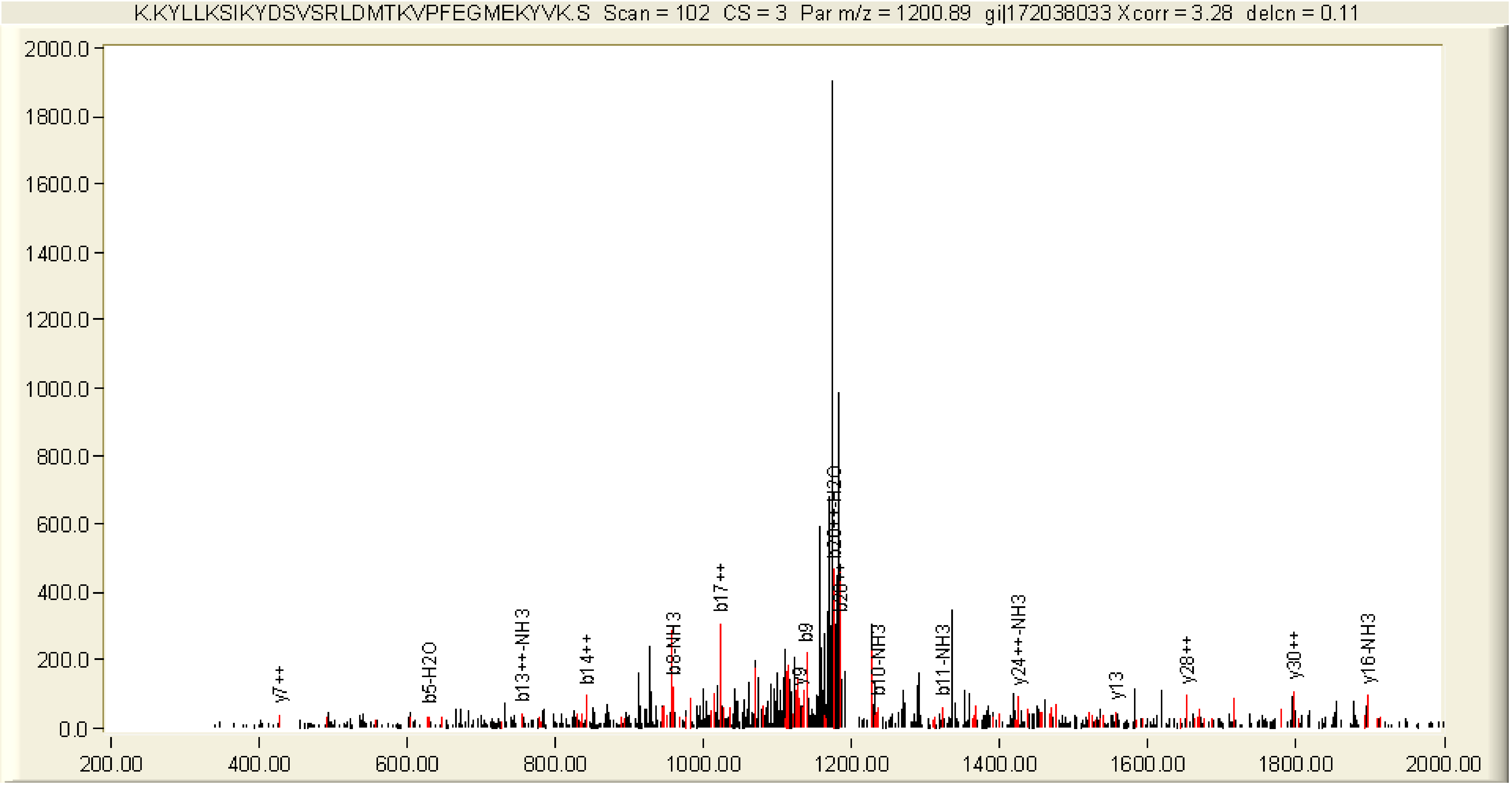
K.KYLLKSIKYDSVSRLDMTKVPFEGMEKYVK.S Scan = 102 CS = 3 Par m/z = 1200.89 gi|172038033 Xcorr = 3.28 delcn = 0.11
2000.0
1800.0
1600.0
1400.0
1200.0
1000.0
800.0
600.0
400.0
200.0
0.0
200.00
400.00
600.00
800.00
1000.00
1200.00
1400.00
1600.00
1800.00
2000.00
y7++
b5-H2O
b13++-NH3
b14++
b8-NH3
b17++
y9
b9
b20++
b10-NH3
b11-NH3
y24++-NH3
y13
y28++
y30++
y16-NH3

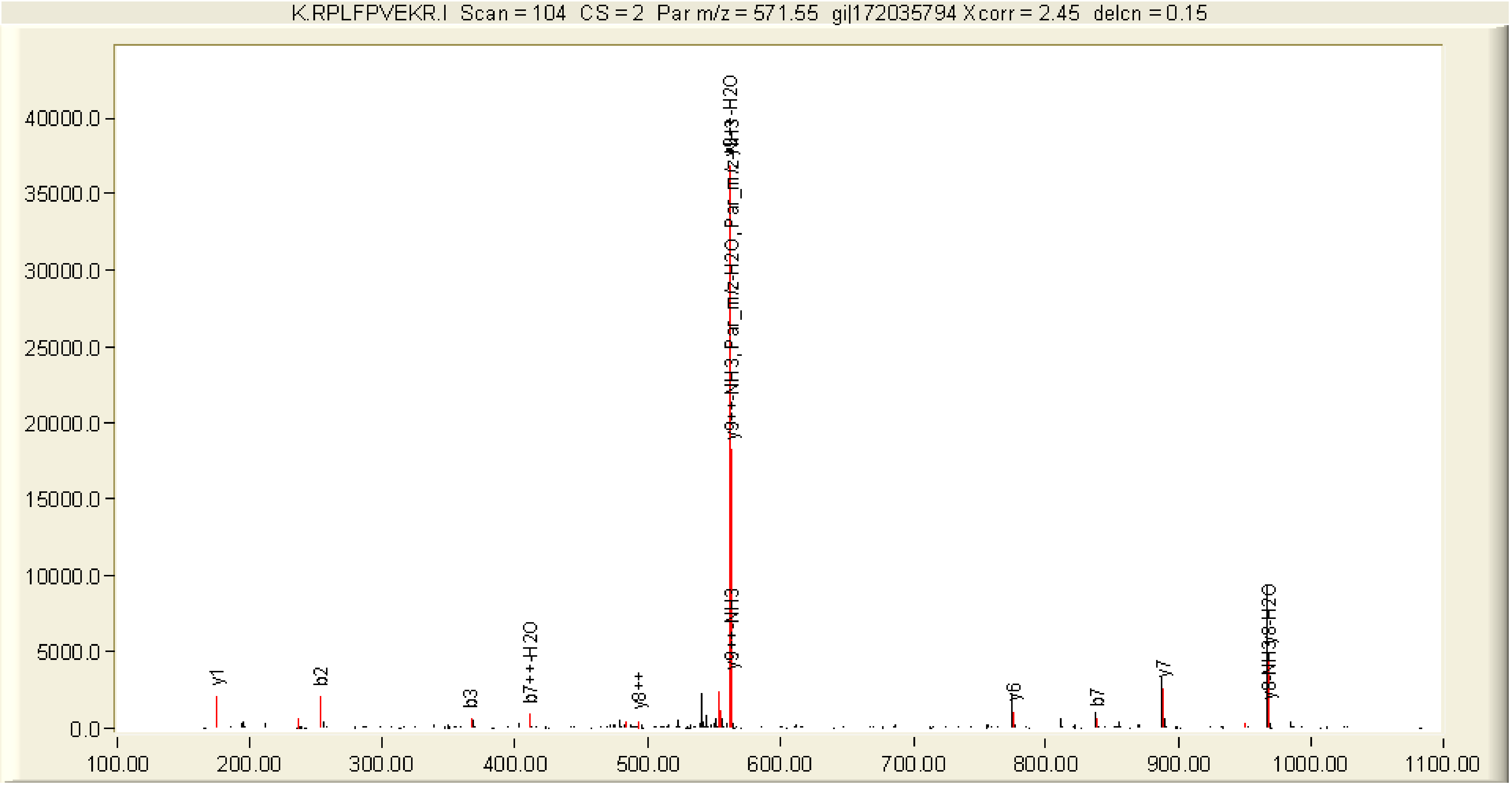
K.RPLFPVEKR.I Scan = 104 CS = 2 Par m/z = 571.55 gi|172035794 Xcorr = 2.45 delcn = 0.15
40000.0
35000.0
30000.0
25000.0
20000.0
15000.0
10000.0
5000.0
0.0
100.00
200.00
300.00
400.00
500.00
600.00
700.00
800.00
900.00
1000.00
1100.00
y1
b2
b3
b7++-H2O
y8++
y9++-NH3
y9++-NH3, Par_m/z-H2O, Par_m/z-NH3
y6
b7
y7
y8-H2O

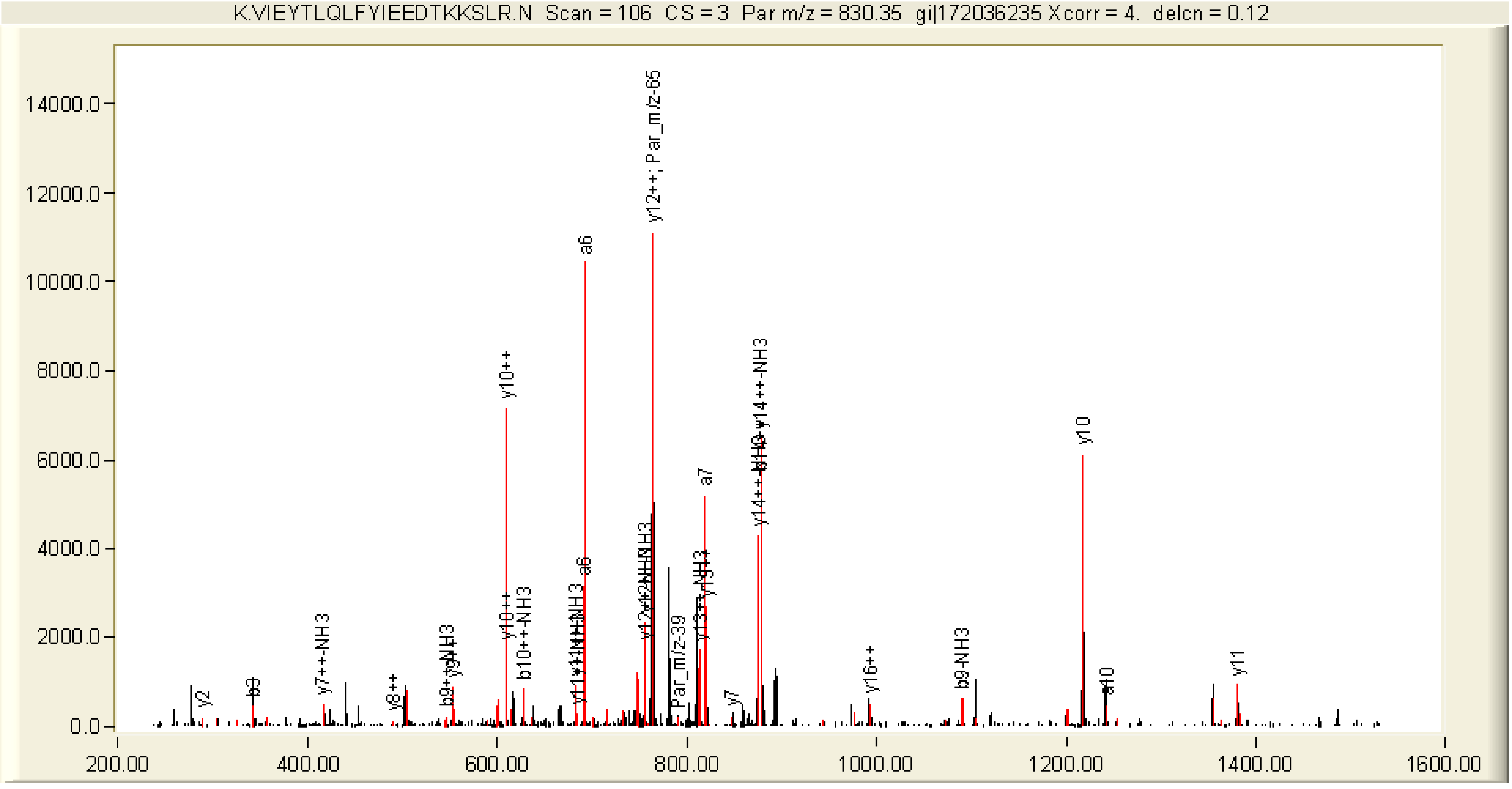
K.VIEYTLQLFYIEEDTKKSLR.N Scan = 106 CS = 3 Par m/z = 830.35 gi|172036235 Xcorr = 4. delcn = 0.12
14000.0
12000.0
10000.0
8000.0
6000.0
4000.0
2000.0
0.0
200.00
400.00
600.00
800.00
1000.00
1200.00
1400.00
1600.00
y12++; Par_m/z-65
a6
y10++
y14++-NH3
a7
y10
y2
b3
y7++-NH3
y8++
y10++-NH3
b10++-NH3
Par_m/z-39
y13++-NH3
y13++
y7
y16++
b9-NH3
a10
y11

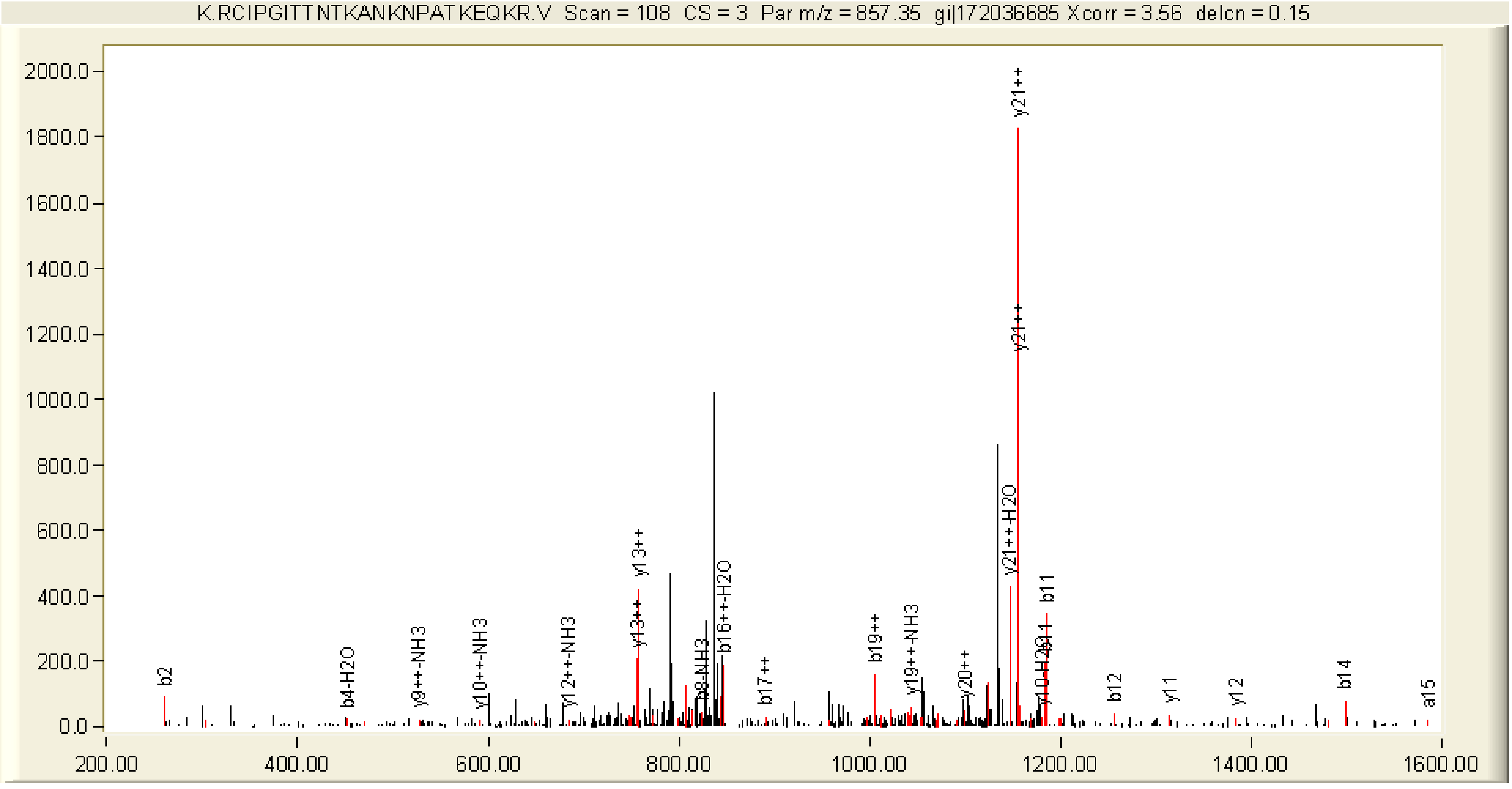

K.RCIPGITTNTKANKNPATKEQKR.V Scan = 108 CS = 3 Par m/z = 857.35 gi|172036685 Xcorr = 3.56 delcn = 0.15
2000.0
1800.0
1600.0
1400.0
1200.0
1000.0
800.0
600.0
400.0
200.0
0.0
200.00
400.00
600.00
800.00
1000.00
1200.00
1400.00
1600.00
y21++
y21++
y21++-H2O
y13++
y13++
b16++-H2O
b8-NH3
b11
b2
b4-H2O
y9++-NH3
y10++-NH3
y12++-NH3
b17++
b19++
y19++-NH3
y20++
b12
y11
y12
b14
a15

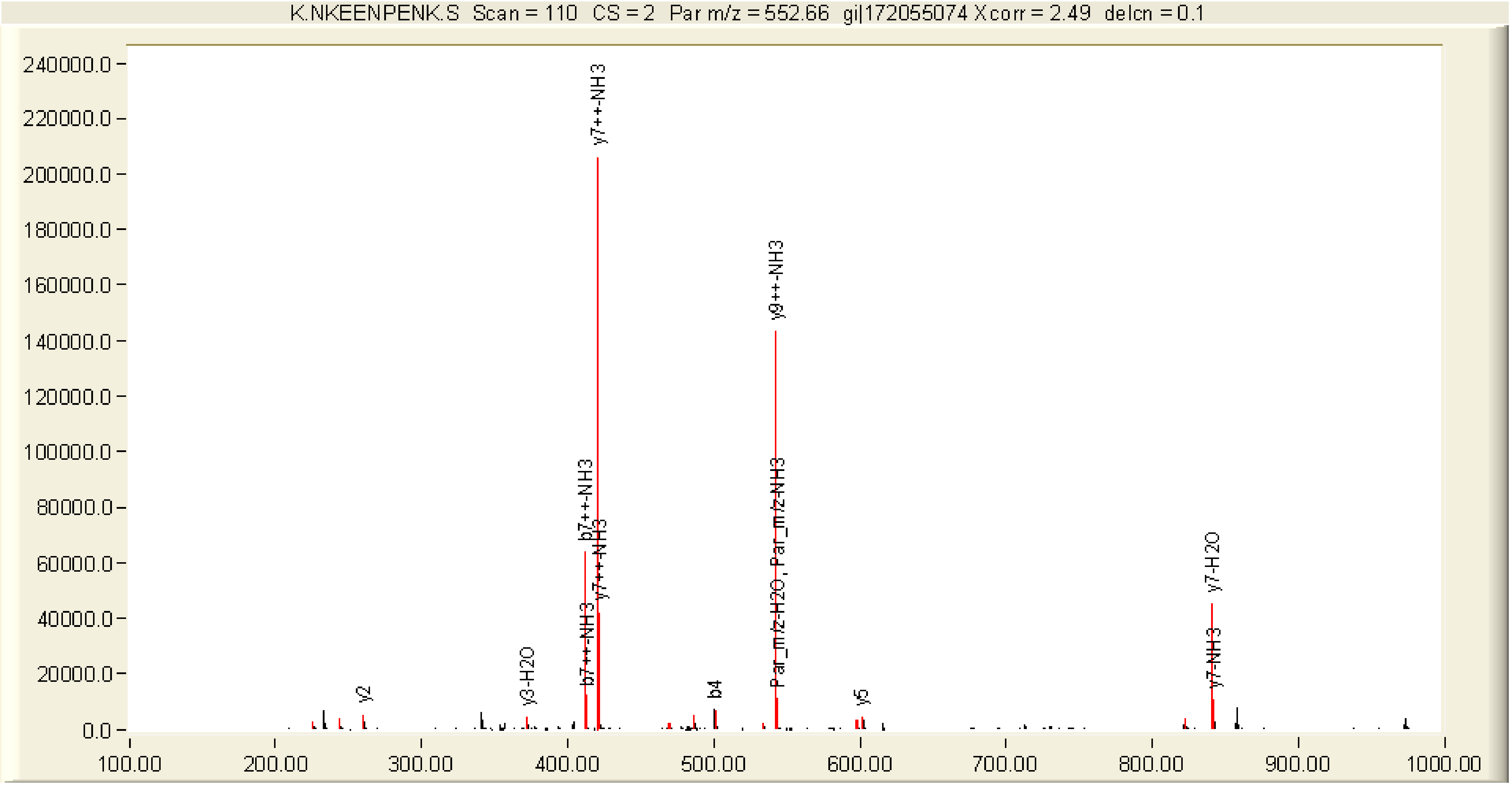

K.NKEENPENK.S Scan = 110 CS = 2 Par m/z = 552.66 gi|172055074 Xcorr = 2.49 delcn = 0.1
240000.0
220000.0
200000.0
180000.0
160000.0
140000.0
120000.0
100000.0
80000.0
60000.0
40000.0
20000.0
0.0
100.00
200.00
300.00
400.00
500.00
600.00
700.00
800.00
900.00
1000.00
y7++-NH3
y9++-NH3
b7++-NH3
y7++-NH3
Par_m/z-H2O, Par_m/z-NH3
y7-H2O
y7-NH3
b7++-NH3
y3-H2O
y2
b4
y5

R.GFWNADEETLEK.L Scan = 112 CS = 2 Par m/z = 721.36 gi|172035531 Xcorr = 2.99 delcn = 0.11

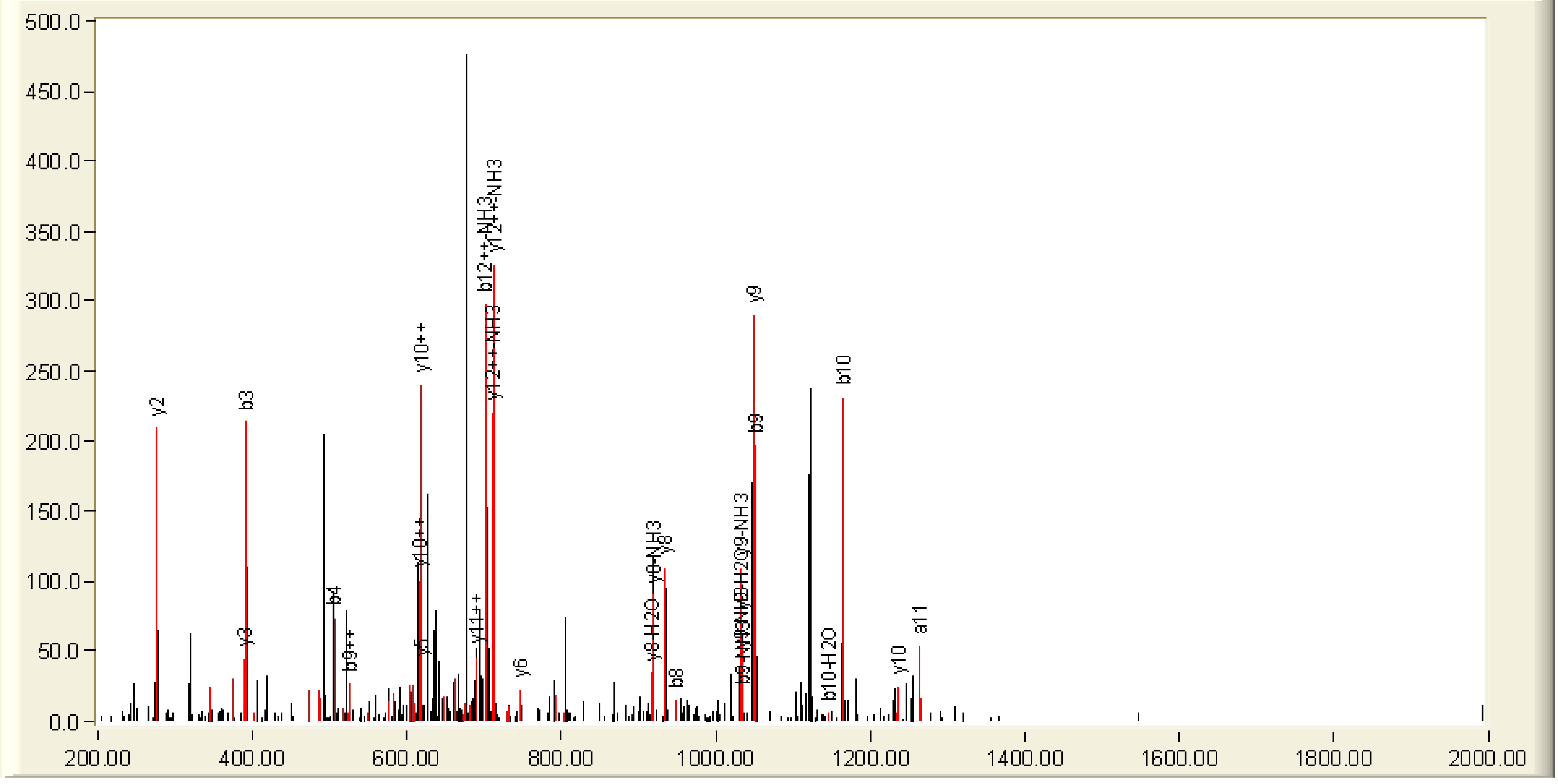

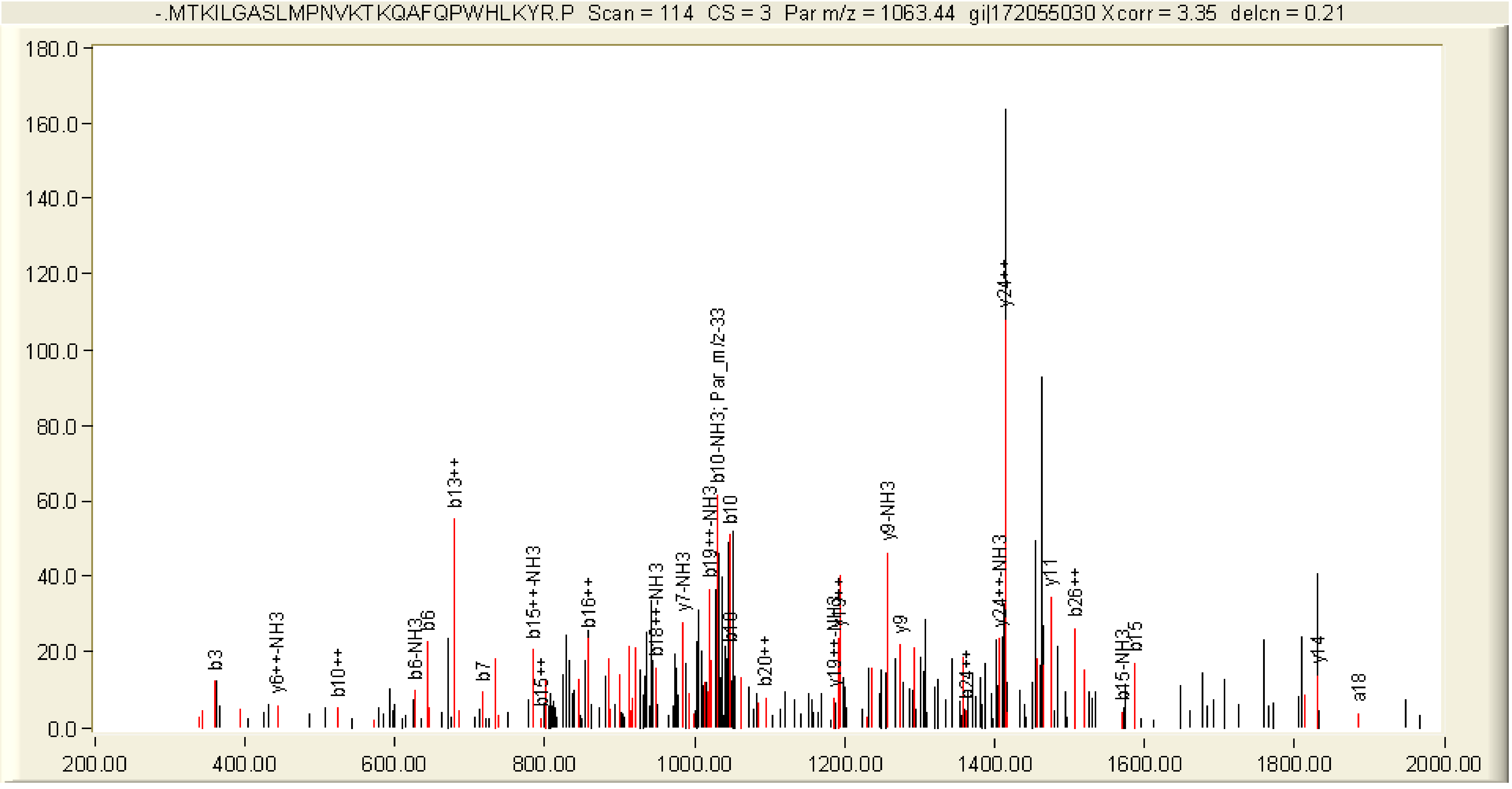
-.MTKILGASLMPNVKTKQAFQPWHLKYR.P Scan = 114 CS = 3 Par m/z = 1063.44 gi|172055030 Xcorr = 3.35 delcn = 0.21
180.0
160.0
140.0
120.0
100.0
80.0
60.0
40.0
20.0
0.0
200.00
400.00
600.00
800.00
1000.00
1200.00
1400.00
1600.00
1800.00
2000.00
b3
y6++-NH3
b10++
b6-NH3
b6
b13++
b7
b15++-NH3
b15++
b16++
b18++-NH3
y7-NH3
b19++-NH3
b10-NH3; Par_m/z-33
b10
b20++
y19++-NH3
y9-NH3
y9
y24++
y24++-NH3
y11
b26++
b15-NH3
b15
y14
a18

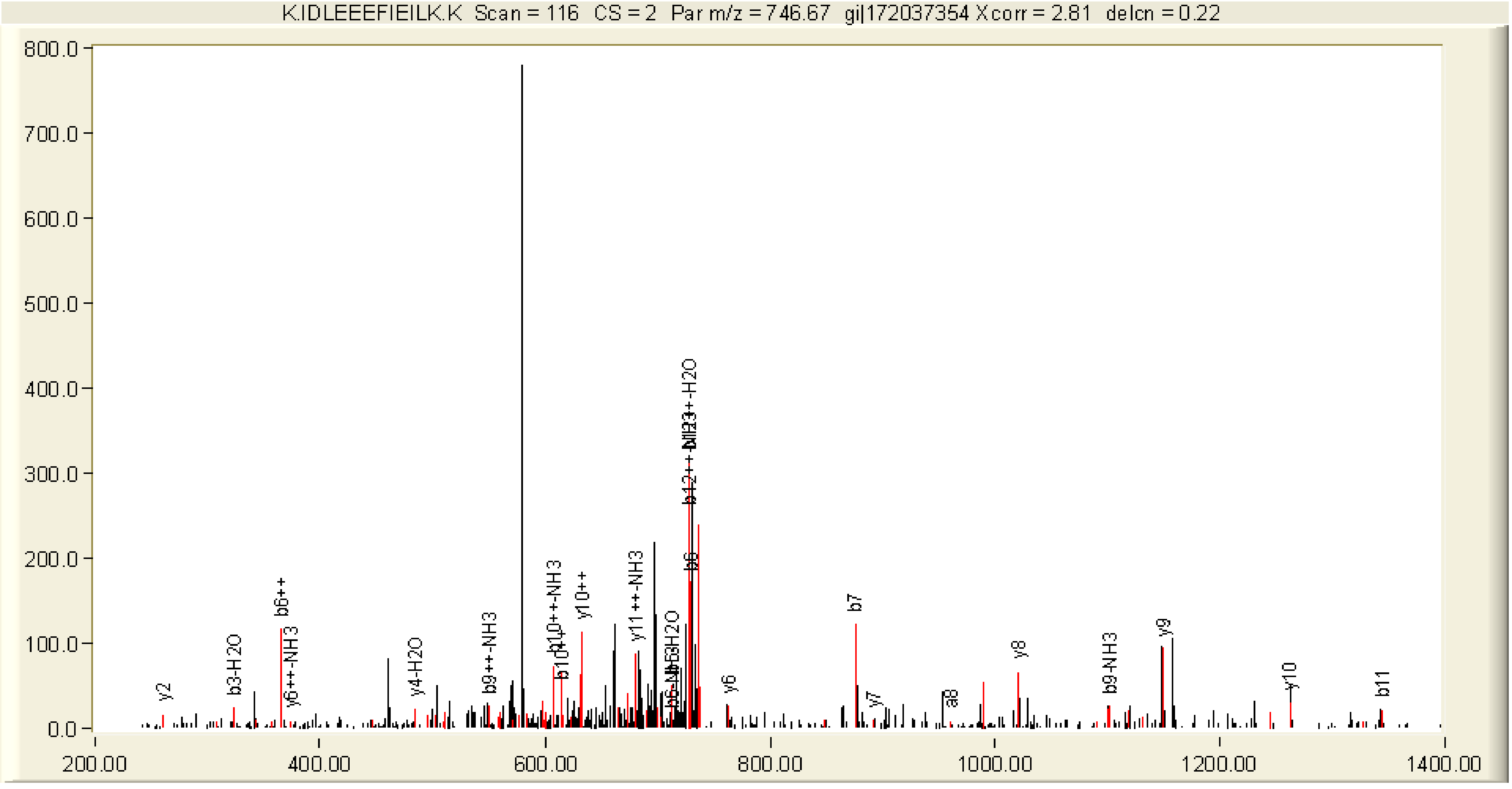

K.IDLEEEEFIEILK.K Scan = 116 CS = 2 Par m/z = 746.67 gi|172037354 Xcorr = 2.81 delcn = 0.22
800.0
700.0
600.0
500.0
400.0
300.0
200.0
100.0
0.0
200.00
400.00
600.00
800.00
1000.00
1200.00
1400.00
y2
b3-H2O
b6++
y6++-NH3
y4-H2O
b9++-NH3
b10++-NH3
b10++
y10++
y11++-NH3
b6-H2O
b6
b7
y6
y7
a8
y8
b9-NH3
y9
y10
b11

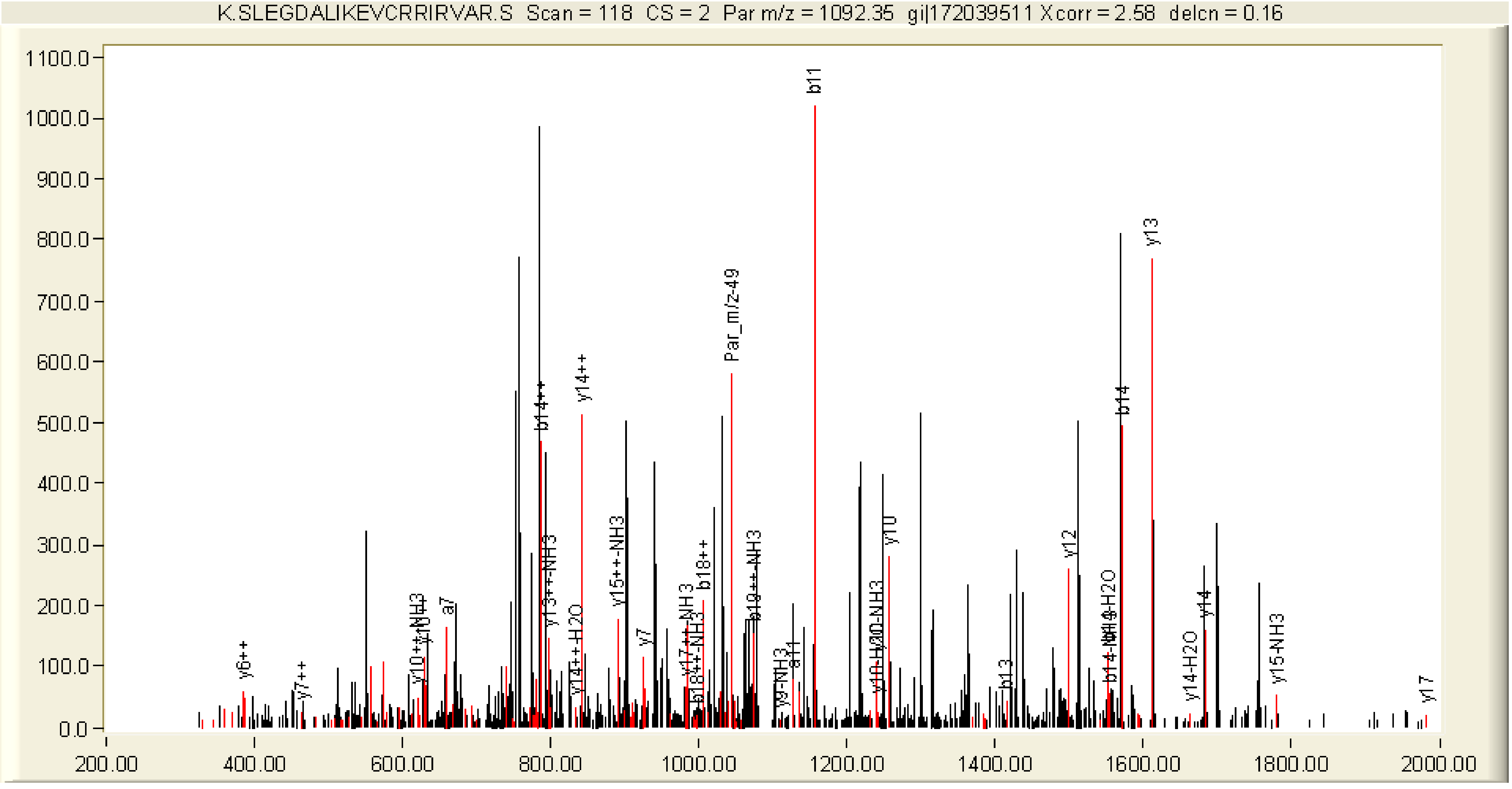
K.SLEGDALIKEVCRRIRVAR.S Scan = 118 CS = 2 Par m/z = 1092.35 gi|172039511 Xcorr = 2.58 delcn = 0.16
1100.0
1000.0
900.0
800.0
700.0
600.0
500.0
400.0
300.0
200.0
100.0
0.0
200.00
400.00
600.00
800.00
1000.00
1200.00
1400.00
1600.00
1800.00
2000.00
b11
y13
b14
Par_m/z-49
y14++
b14++
y13++-NH3
y15++-NH3
y10
y12
b18++
b19++-NH3
a7
y6++
y7++
y7
b13
y14
y14-H2O
y15-NH3
y17
a11
y9-NH3

K.RSGTQSVGIIGGFGLAAELAVR.E Scan = 120 CS = 2 Par m/z = 1080.78 gi|172035118 Xcorr = 2.87 delcn = 0.15

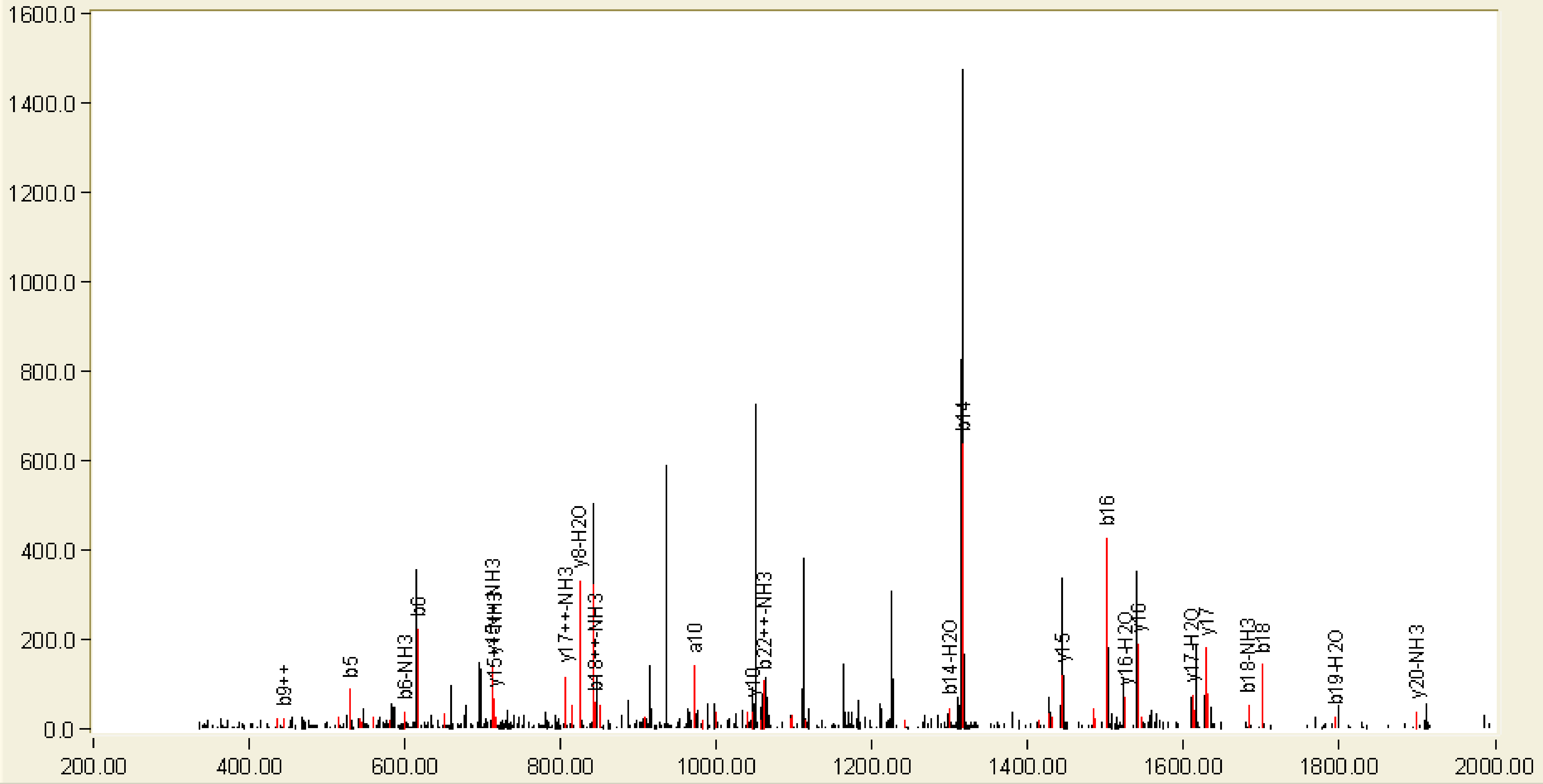

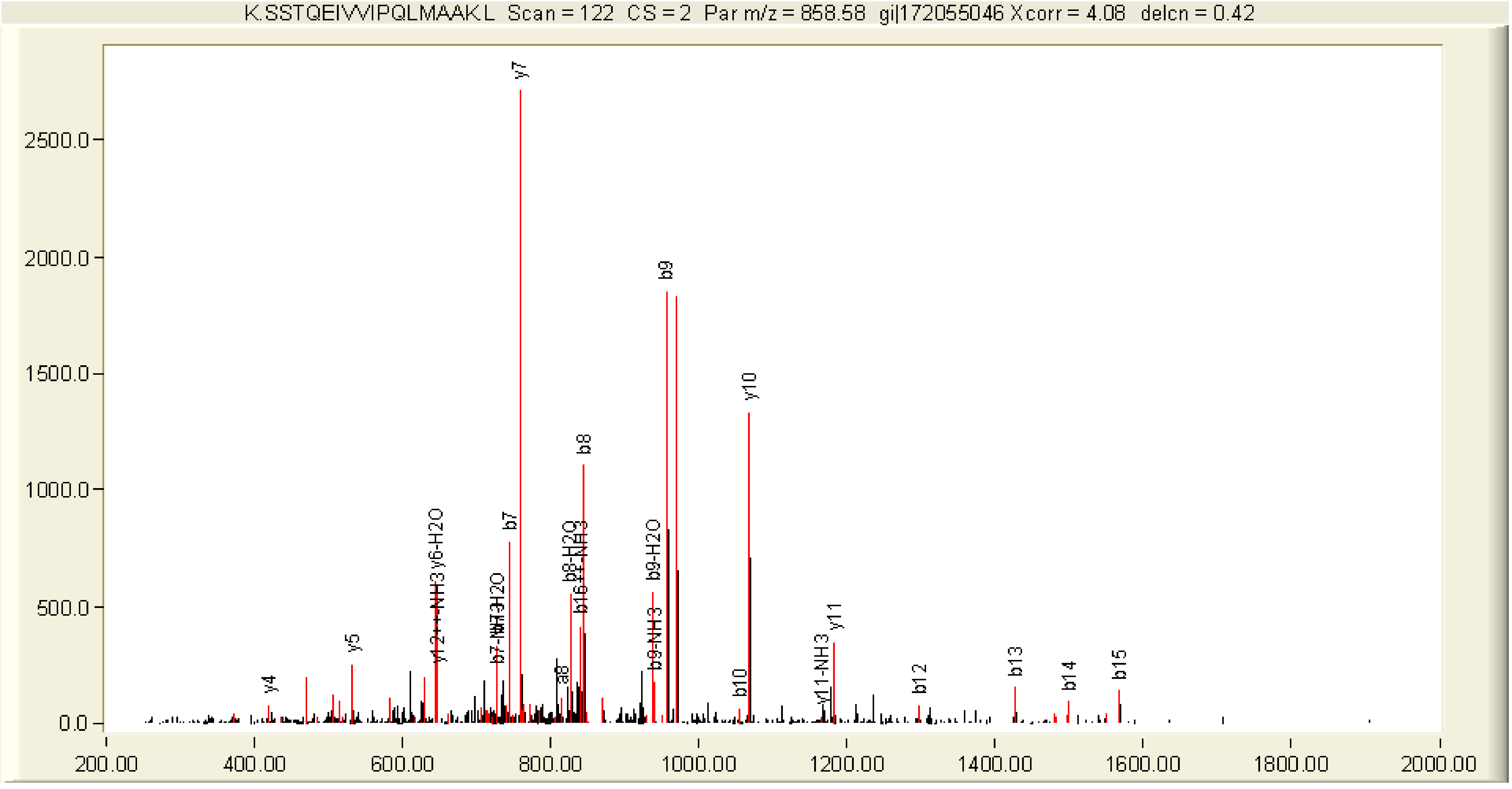

K.SSTQEIVVIPQLMAAK.L Scan = 122 CS = 2 Par m/z = 858.58 gi|172055046 Xcorr = 4.08 delcn = 0.42
y7
b9
y10
b8
b7
y6-H2O
b9-H2O
b8-H2O
b9-NH3
y11
y11-NH3
y5
y4
a8
b10
b12
b13
b14
b15
2500.0
2000.0
1500.0
1000.0
500.0
0.0
200.00
400.00
600.00
800.00
1000.00
1200.00
1400.00
1600.00
1800.00
2000.00

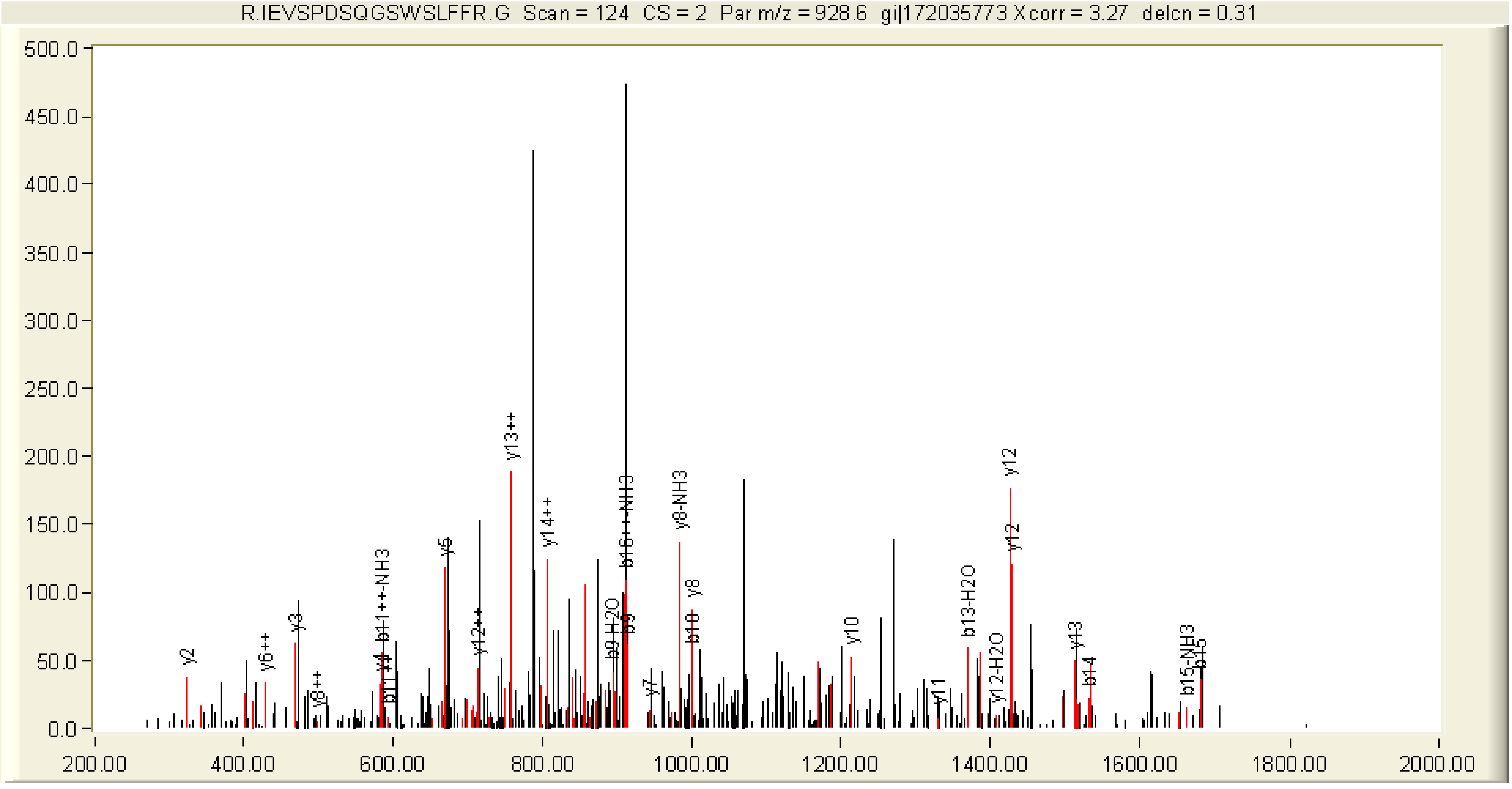

R.IEVSPDSQGSWSLFFR.G Scan = 124 CS = 2 Par m/z = 928.6 gi|172035773 Xcorr = 3.27 delcn = 0.31
0.0
50.0
100.0
150.0
200.0
250.0
300.0
350.0
400.0
450.0
500.0
200.00
400.00
600.00
800.00
1000.00
1200.00
1400.00
1600.00
1800.00
2000.00
y2
y6++
y3
y8++
b11++-NH3
y5
y12++
y13++
y14++
b9-H2O
b16++-NH3
y7
y8-NH3
y8
b10
y10
y11
b13-H2O
y12-H2O
y12
y13
b14
b15-NH3
b15

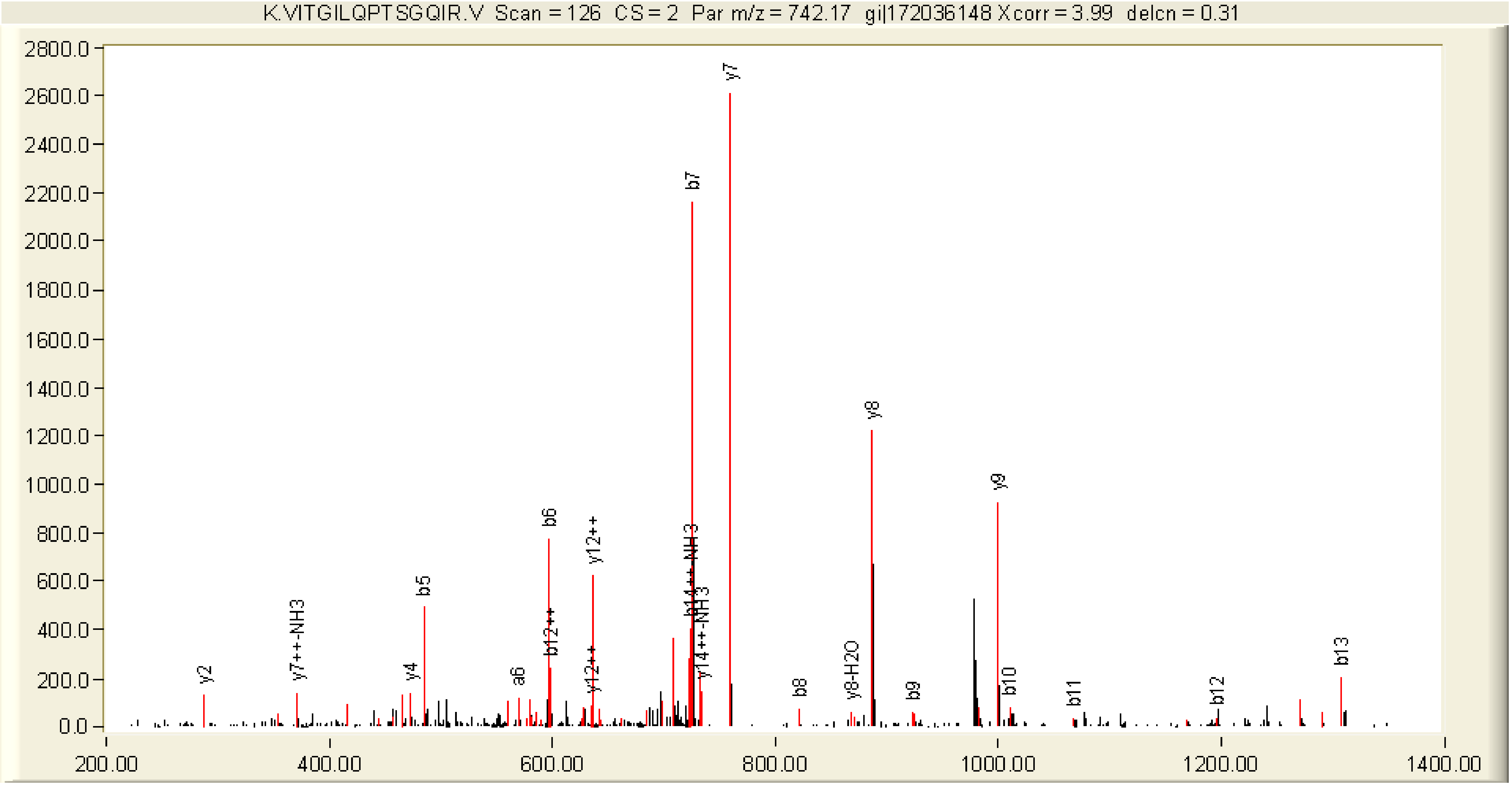

K.VITGILQPTSGQIR.V Scan = 126 CS = 2 Par m/z = 742.17 gi|172036148 Xcorr = 3.99 delcn = 0.31
y7
b7
y8
y9
b6
y12++
b14++-NH3
b14++-NH3
b5
y7++-NH3
b12++
y14++-NH3
y12++
y2
y4
a6
b8
y8-H2O
b9
b10
b11
b12
b13
2800.0
2600.0
2400.0
2200.0
2000.0
1800.0
1600.0
1400.0
1200.0
1000.0
800.0
600.0
400.0
200.0
0.0
200.00
400.00
600.00
800.00
1000.00
1200.00
1400.00

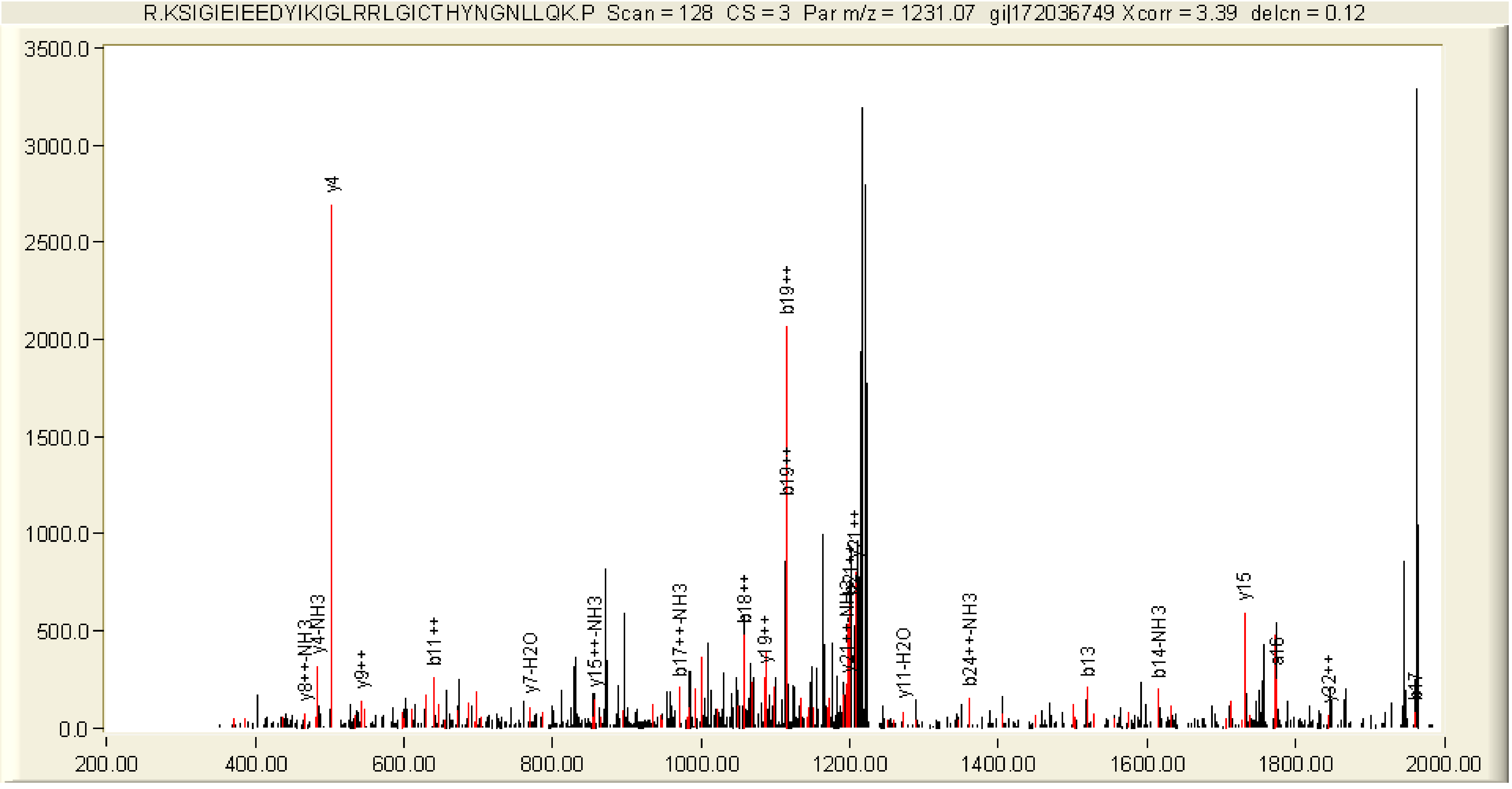

R.KSIGIEIEEDYIKIGLRRLGICTHYNGNLLQK.P Scan = 128 CS = 3 Par m/z = 1231.07 gi|172036749 Xcorr = 3.39 delcn = 0.12
3500.0
3000.0
2500.0
2000.0
1500.0
1000.0
500.0
0.0
200.00
400.00
600.00
800.00
1000.00
1200.00
1400.00
1600.00
1800.00
2000.00
y4
b19++
y8++-NH3
y4-NH3
y9++
b11 ++
y7-H2O
y15++-NH3
b17++-NH3
b18++
y19++
y11-H2O
b24++-NH3
b13
b14-NH3
y15
y32++

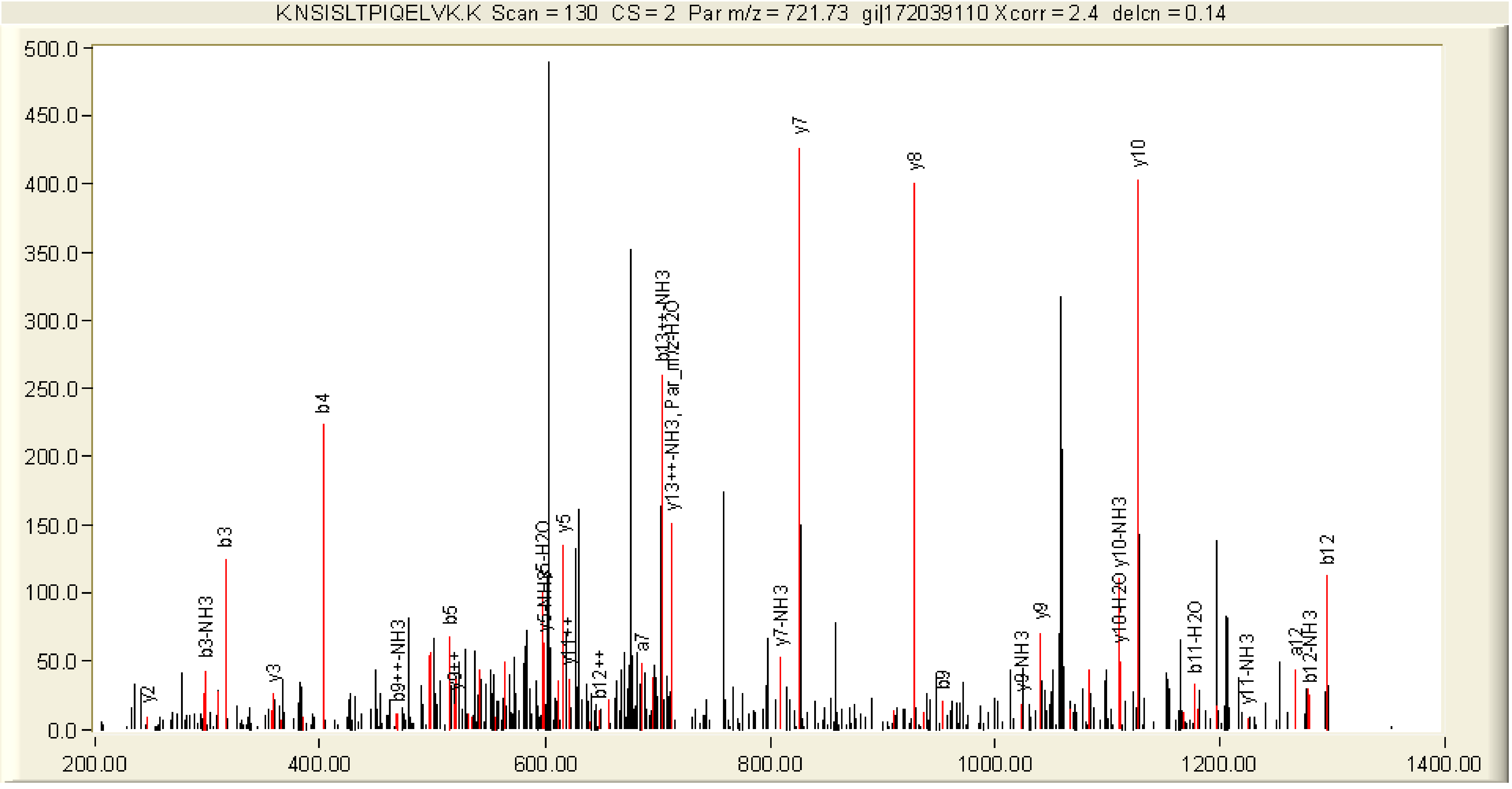

K.NSISLTPIQELVK.K Scan = 130 CS = 2 Par m/z = 721.73 gi|172039110 Xcorr = 2.4 delcn = 0.14
y7
y8
y10
b4
b3
b3-NH3
y3
y2
b9++-NH3
b5
y5
y5-H2O
y11++
b12++
a7
y13++-NH3, Par_m/z-H2O
b13++-NH3
y7-NH3
b9
y9-NH3
y9
y10-H2O
y10-NH3
b11-H2O
y11-NH3
a12
b12-NH3
b12
0.0
50.0
100.0
150.0
200.0
250.0
300.0
350.0
400.0
450.0
500.0
200.00
400.00
600.00
800.00
1000.00
1200.00
1400.00

K.MANYVHIPALSYSY.- Scan = 132 CS = 2 Par m/z = 815.64 gi|172035490 Xcorr = 2.98 delcn = 0.33

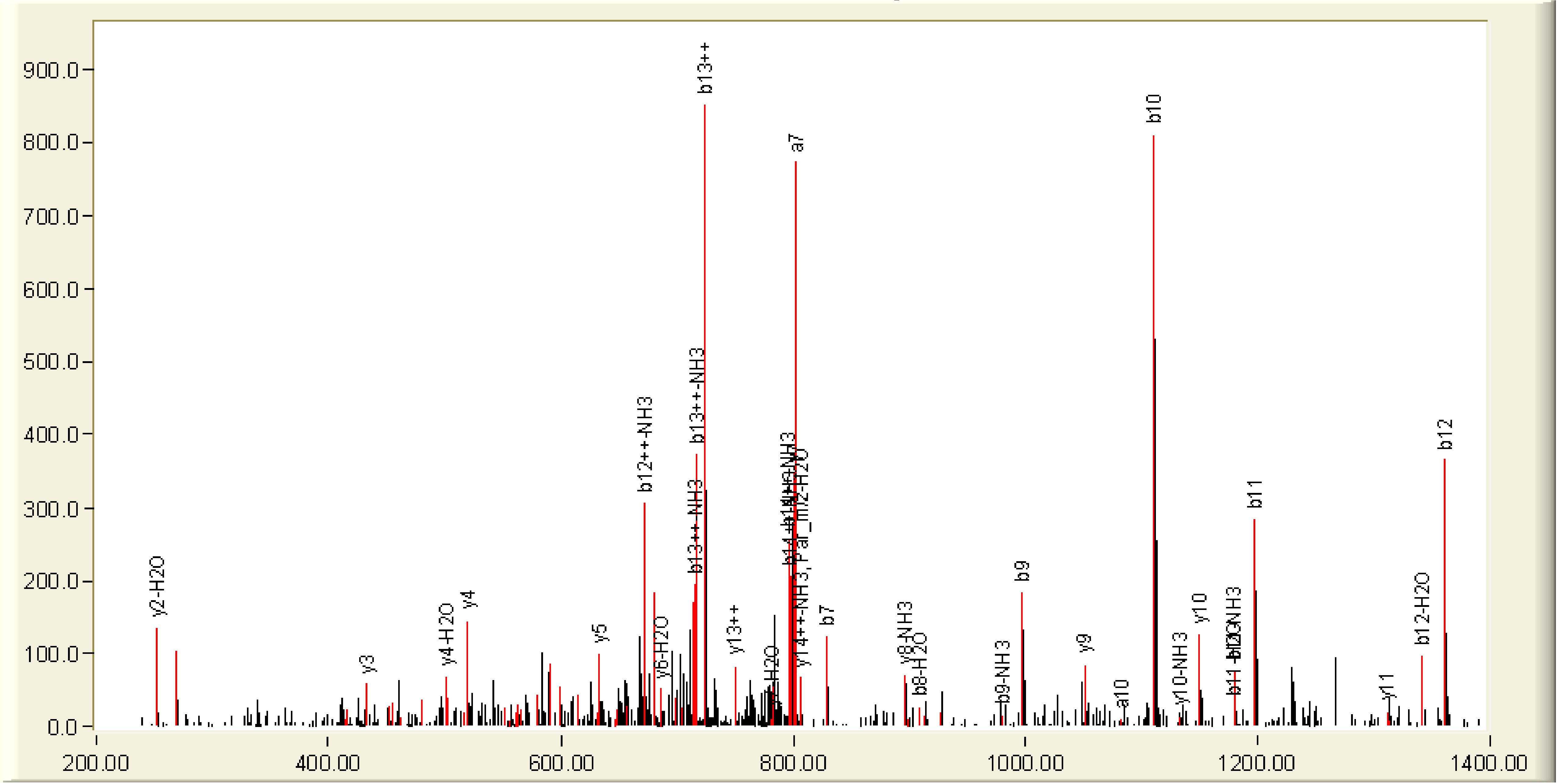

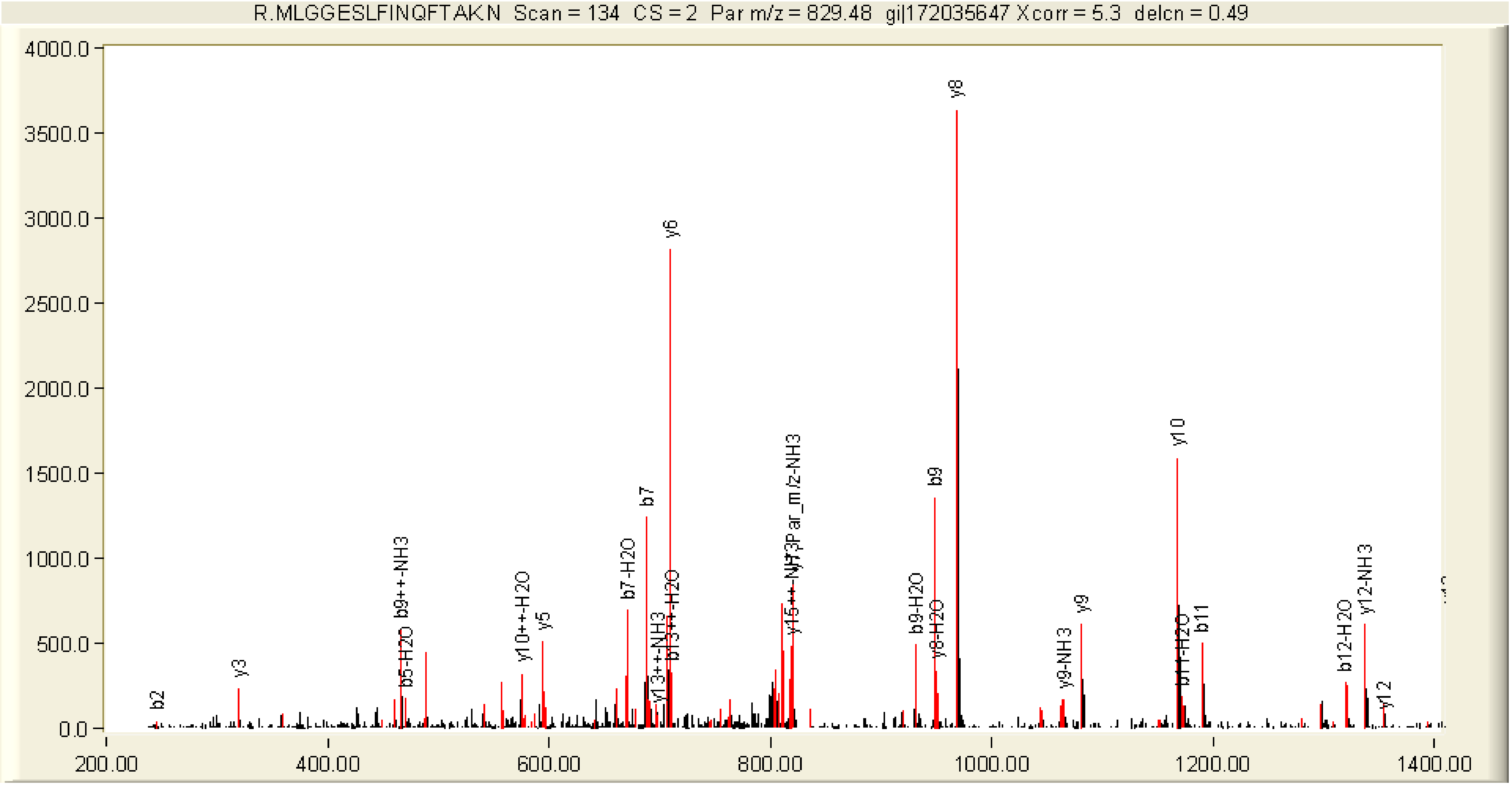

R.MLGGESLFINQFTAKN Scan = 134 CS = 2 Par m/z = 829.48 gi|172035647 Xcorr = 5.3 delcn = 0.49
4000.0
3500.0
3000.0
2500.0
2000.0
1500.0
1000.0
500.0
0.0
200.00
400.00
600.00
800.00
1000.00
1200.00
1400.00
b2
y3
b5-H2O
b9++-NH3
y10++-H2O
y5
b7-H2O
b7
y13++-NH3
b13++-H2O
y6
y15++-NH3
y7
Par_m/z-NH3
b9-H2O
y8-H2O
b9
y8
y9-NH3
y9
y10
b11-H2O
b11
b12-H2O
y12-NH3
y12

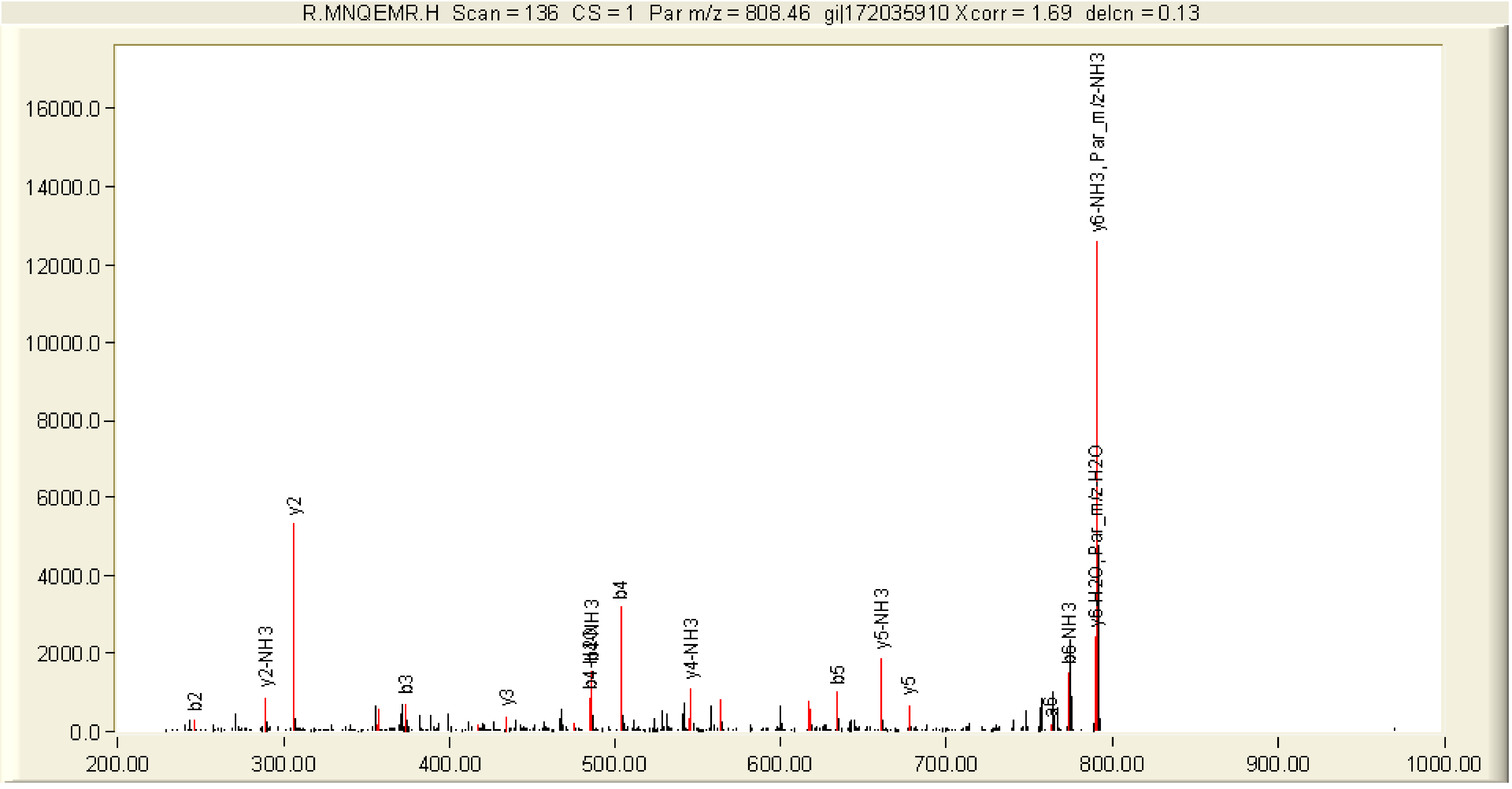

R.MNQEMR.H Scan = 136 CS = 1 Par m/z = 808.46 gi|172035910 Xcorr = 1.69 delcn = 0.13
16000.0
14000.0
12000.0
10000.0
8000.0
6000.0
4000.0
2000.0
0.0
200.00
300.00
400.00
500.00
600.00
700.00
800.00
900.00
1000.00
b2
y2-NH3
y2
b3
y3
b4
y4-NH3
b5
y5-NH3
y5
b6-NH3
y6-H2O, Par_m/z-H2O
y6-NH3, Par_m/z-NH3

R.YGSPDVLEYAEVTKPSPTAK.Q Scan = 138 CS = 2 Par m/z = 1076.71 gi|172039652 Xcorr = 5.08 delcn = 0.53

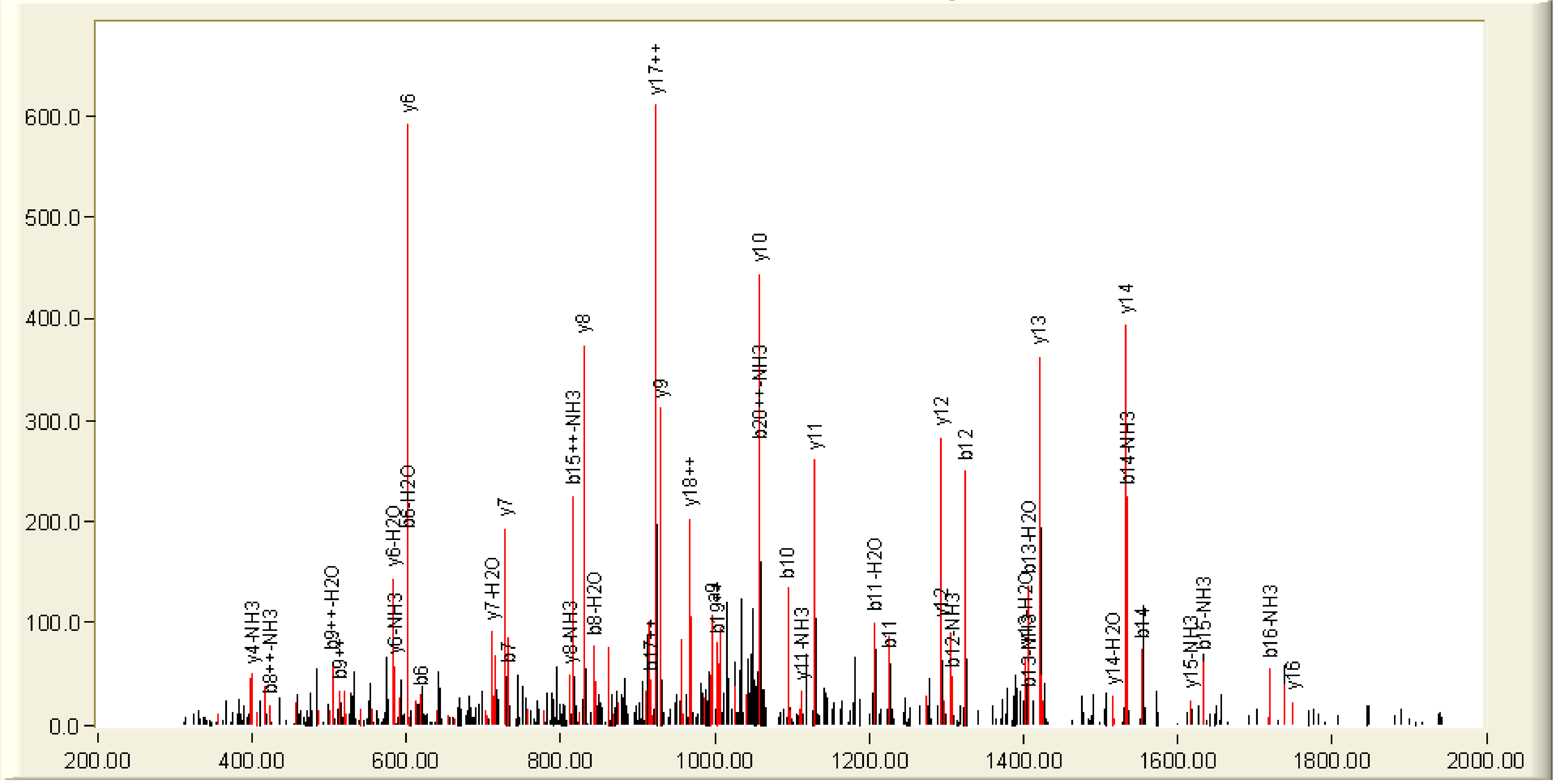

K.SVFTYLK.Q Scan = 140 CS = 1 Par m/z = 857.53 gi|172036592 Xcorr = 1.62 delcn = 0.12

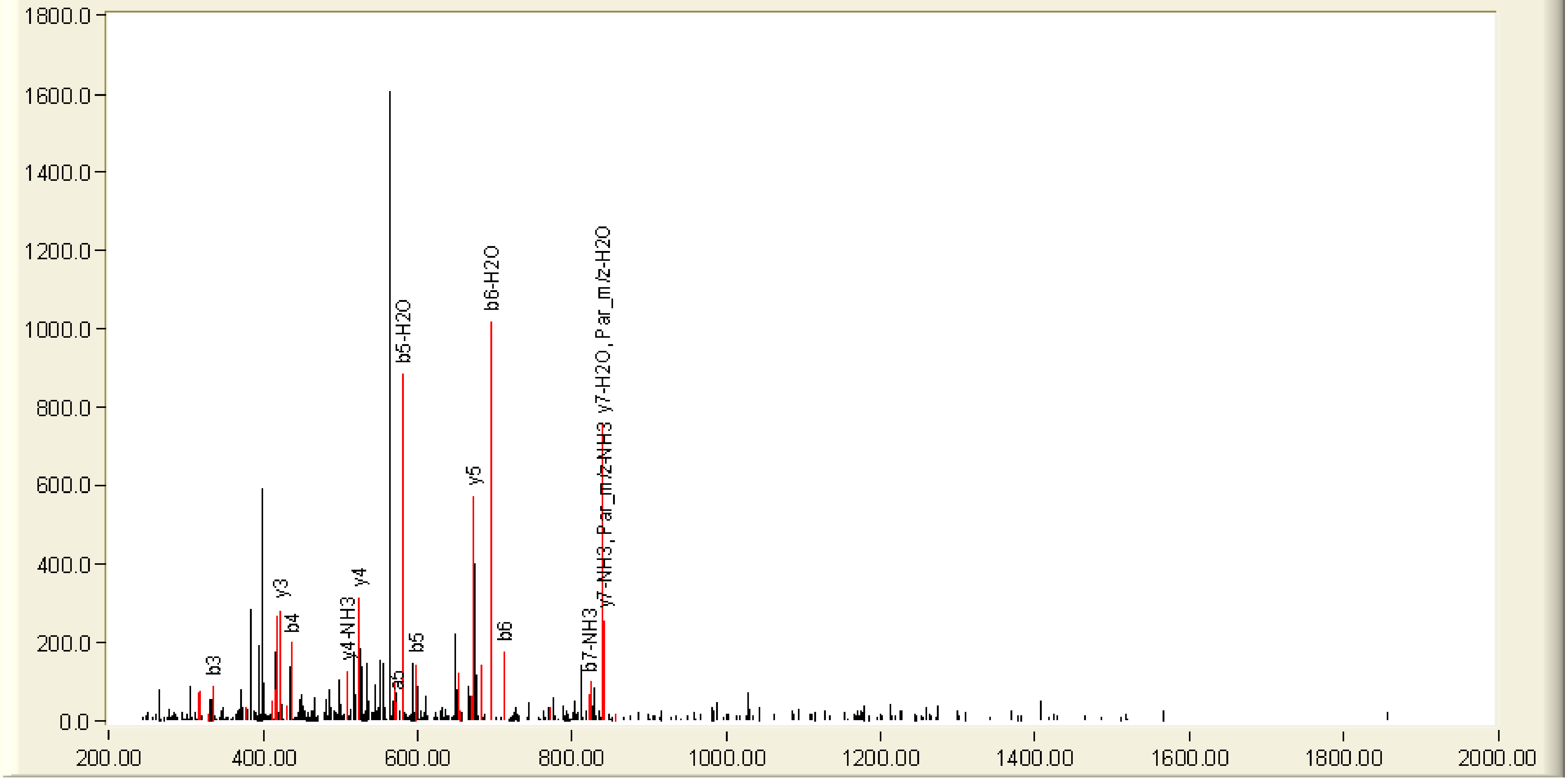

K.NAELTEVPIIVKDLSDSQAQR.F Scan = 142 CS = 2 Par m/z = 1164.89 gi|172055118 Xcorr = 3.19 delcn = 0.4

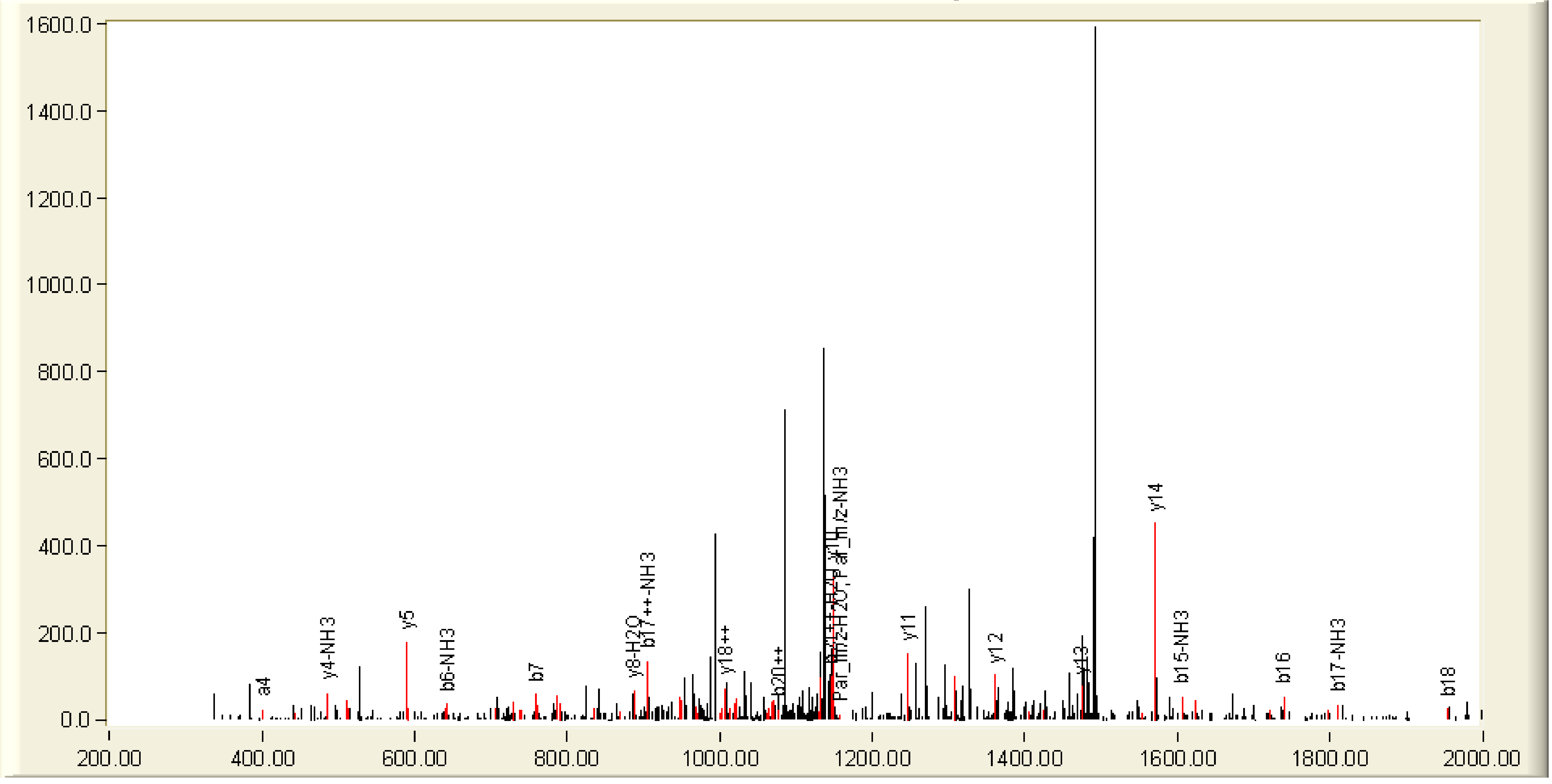

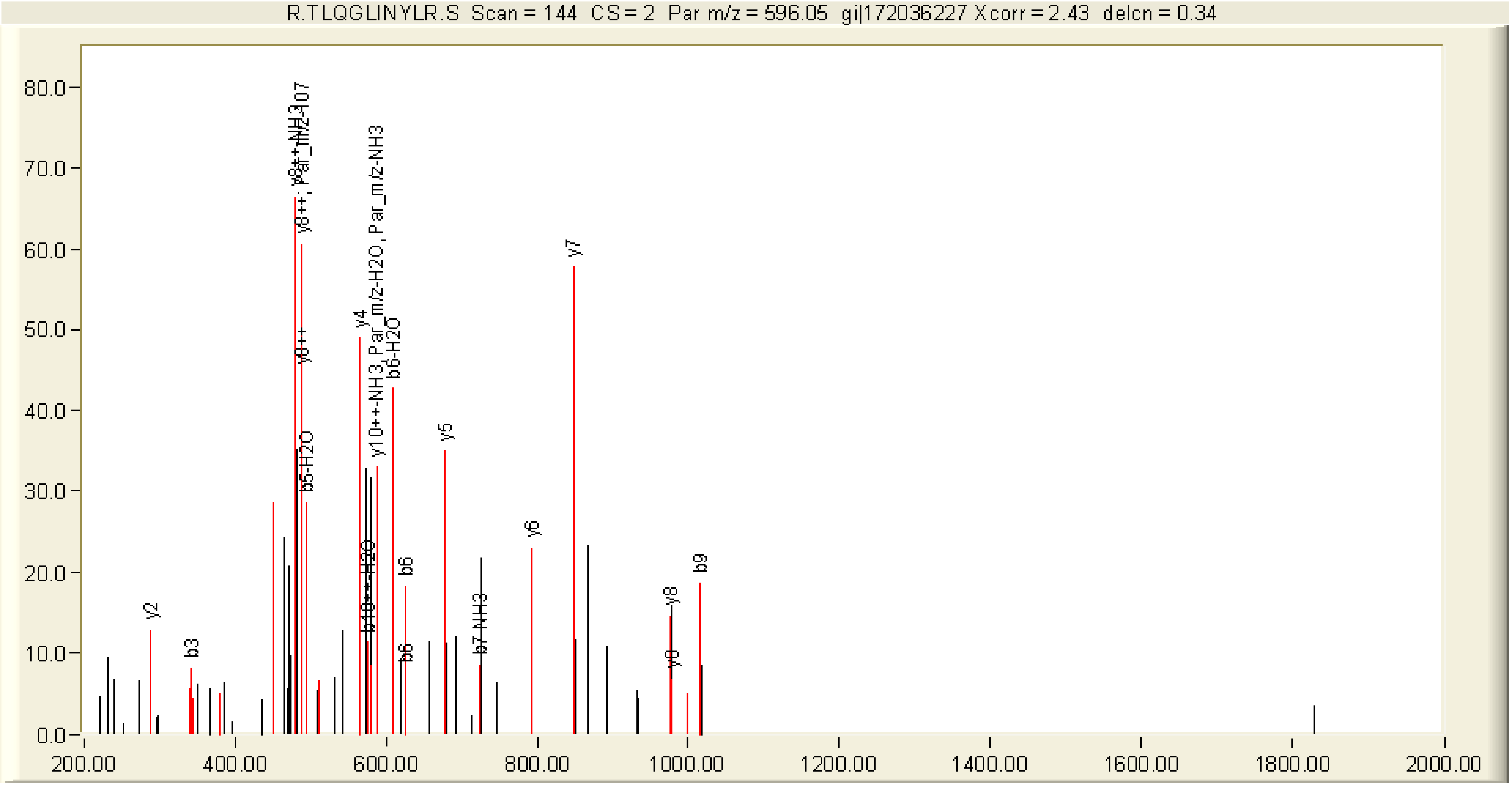
R.TLQGLINYLR.S Scan = 144 CS = 2 Par m/z = 596.05 gi|172036227 Xcorr = 2.43 delcn = 0.34
80.0
70.0
60.0
50.0
40.0
30.0
20.0
10.0
0.0
200.00
400.00
600.00
800.00
1000.00
1200.00
1400.00
1600.00
1800.00
2000.00
y2
b3
y8++; Par_m/z-107
y8++
b5-H2O
y4
y10++-NH3, Par_m/z-H2O, Par_m/z-NH3
b6-H2O
b6
b6
y5
b7-NH3
y6
y7
y8
b9

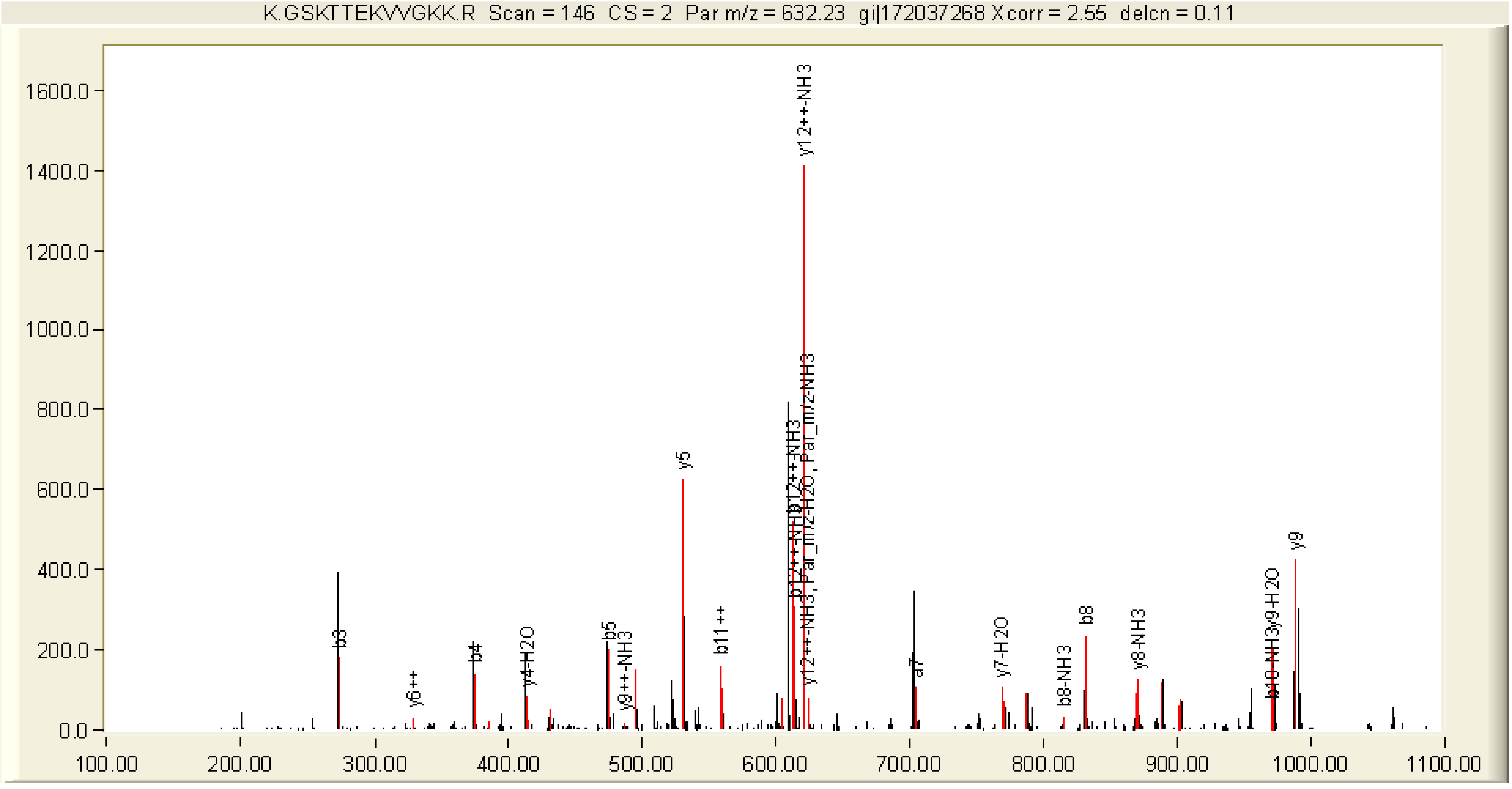

K.GSKTTEKVVGKK.R Scan = 146 CS = 2 Par m/z = 632.23 gi|172037268 Xcorr = 2.55 delcn = 0.11
1600.0
1400.0
1200.0
1000.0
800.0
600.0
400.0
200.0
0.0
100.00
200.00
300.00
400.00
500.00
600.00
700.00
800.00
900.00
1000.00
1100.00
b3
y6++
b4
y4-H2O
b5
y9++-NH3
y5
b11++
y12++-NH3
y12++-NH3, Par_m/z-NH3
a7
y7-H2O
b8-NH3
b8
y8-NH3
b10-NH3
y9-H2O
y9

R.LSYSQGTLEIMTPLFIHENTKV Scan = 148 CS = 3 Par m/z = 808.6 gi|172036320 Xcorr = 3.36 delcn = 0.13

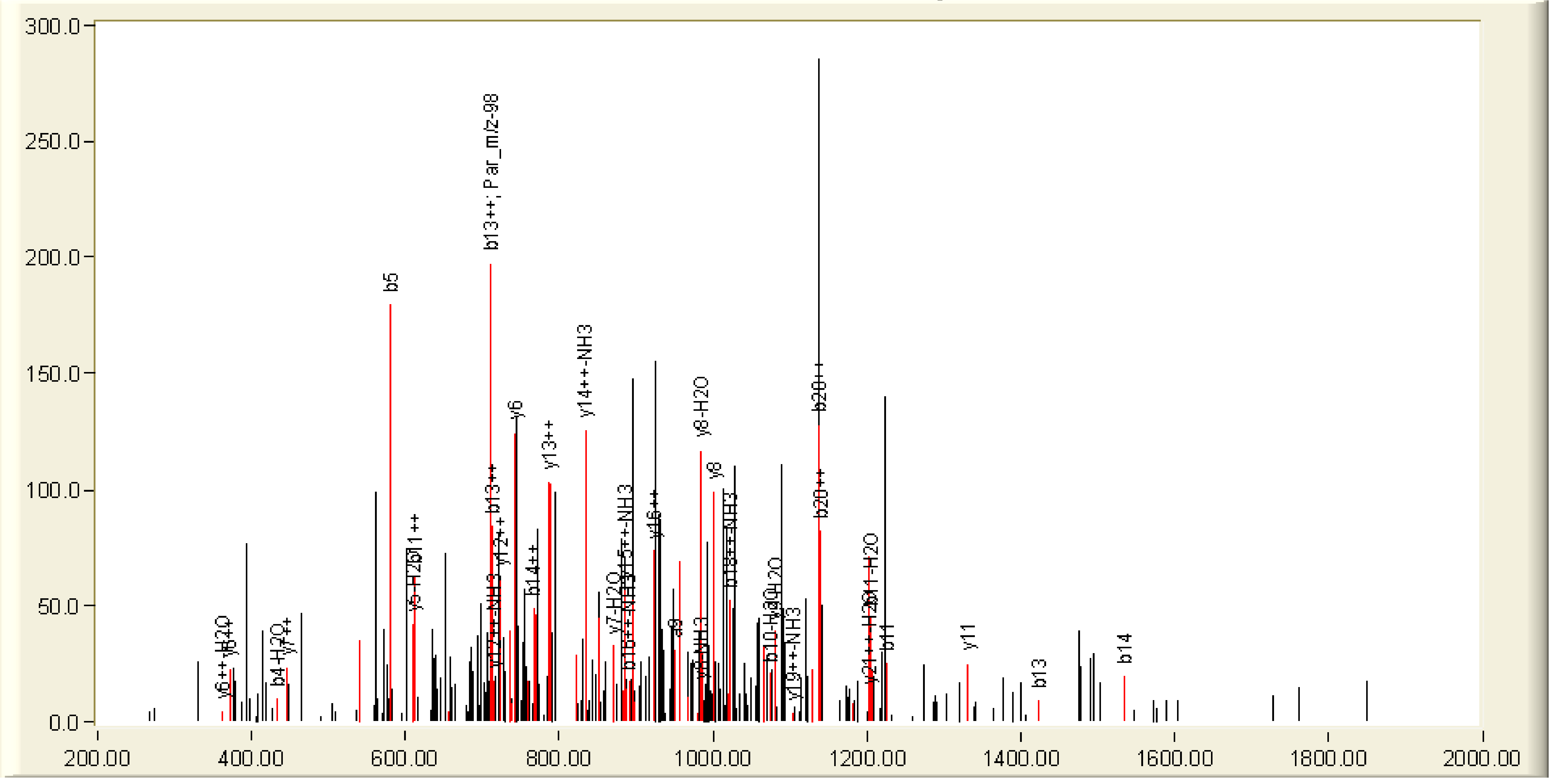

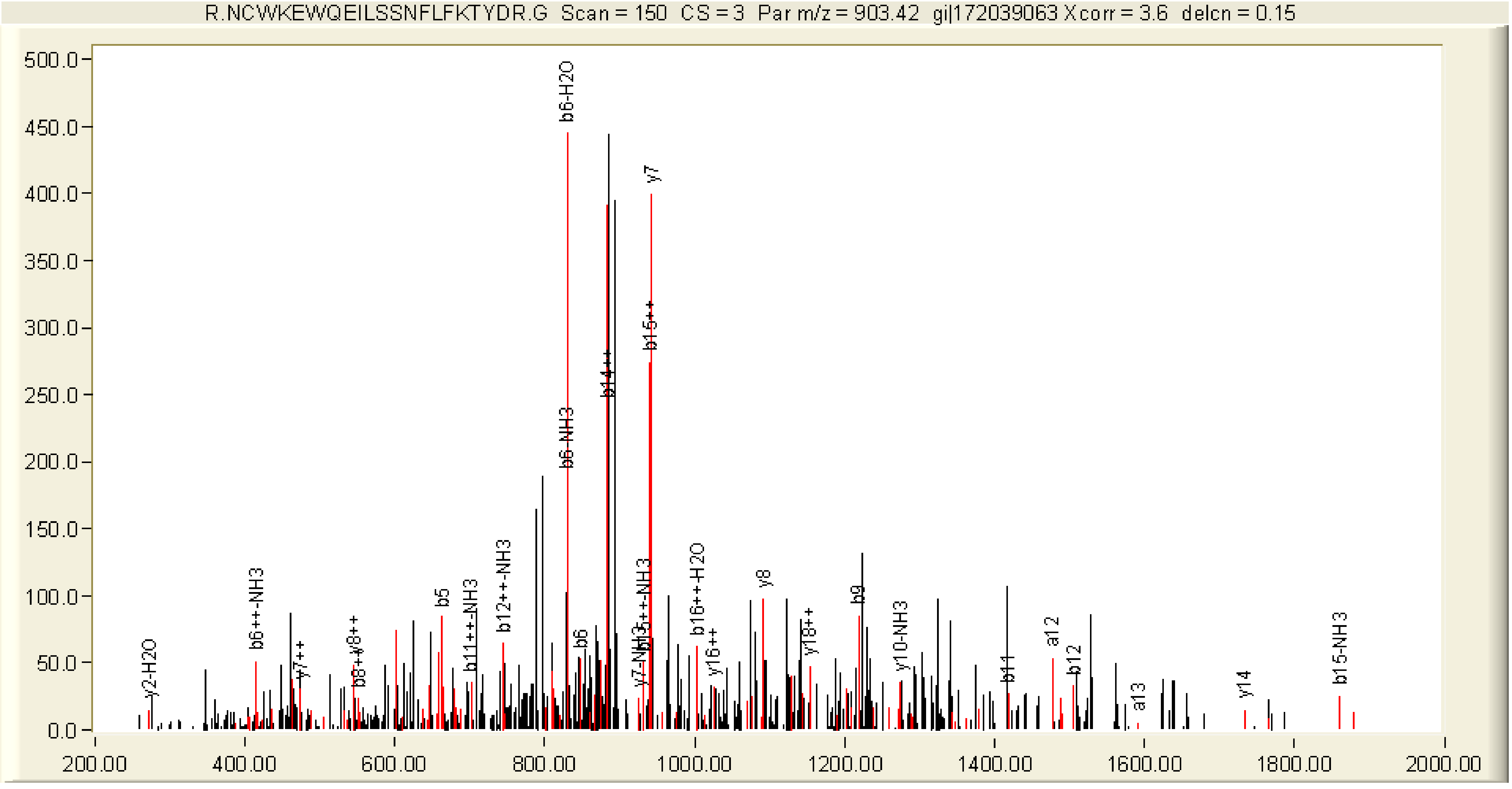
R.NCWKEWQEILSSNFLFKTYDR.G Scan = 150 CS = 3 Par m/z = 903.42 gi|172039063 Xcorr = 3.6 delcn = 0.15
500.0
450.0
400.0
350.0
300.0
250.0
200.0
150.0
100.0
50.0
0.0
200.00
400.00
600.00
800.00
1000.00
1200.00
1400.00
1600.00
1800.00
2000.00
b6-H2O
y7
b15++
b14++
b6-NH3
y2-H2O
b6++-NH3
y7++
b8++
y8++
b5
b11++-NH3
b12++-NH3
b6
y7-NH3
b16++-H2O
y16++
y8
y18++
b9
y10-NH3
b11
a12
b12
a13
y14
b15-NH3

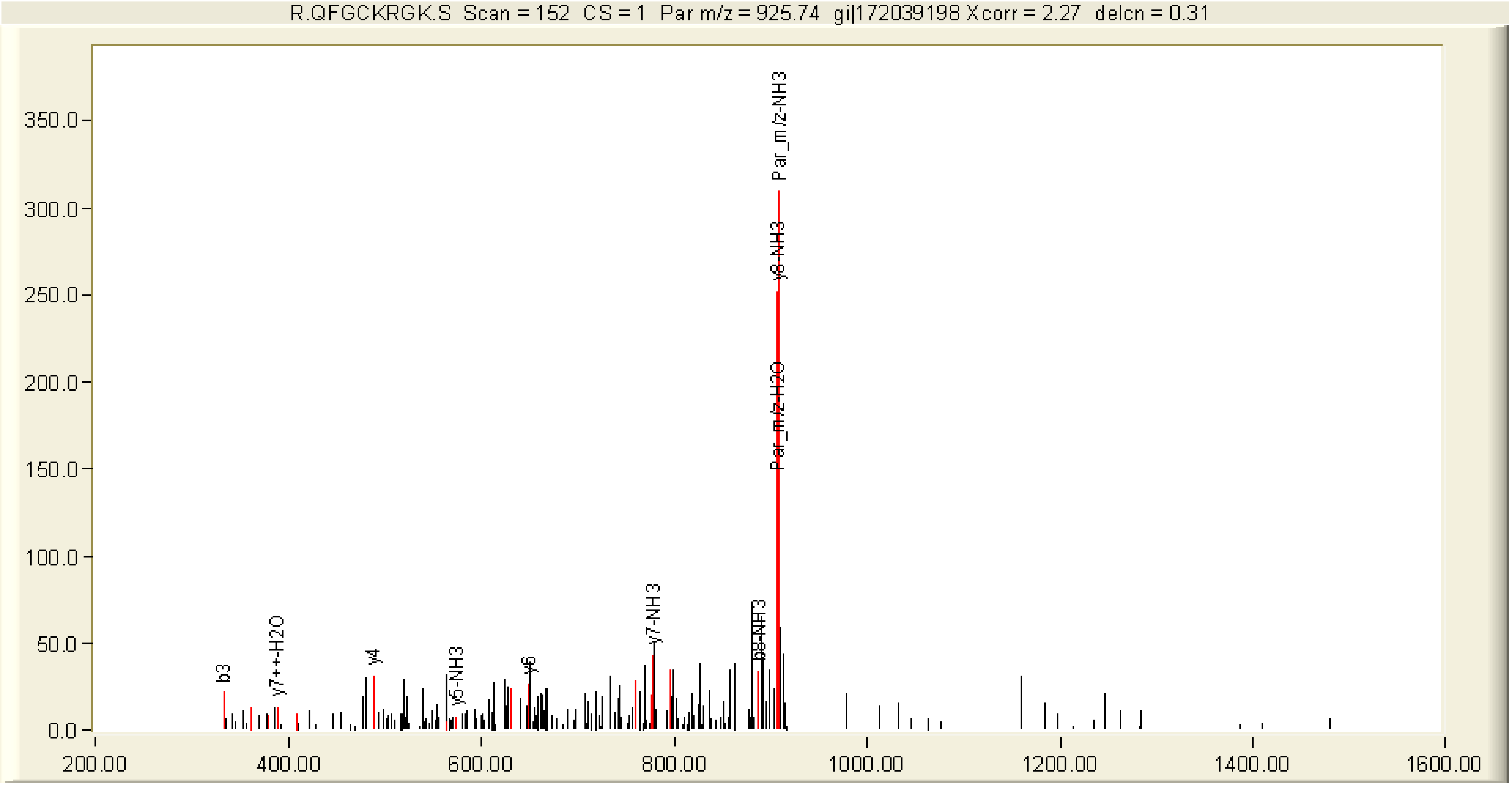
R.QFGCKRGK.S Scan = 152 CS = 1 Par m/z = 925.74 gi|172039198 Xcorr = 2.27 delcn = 0.31
Par_m/z-NH3
y8-NH3
Par_m/z-H2O
b3
y7++-H2O
y4
y5-NH3
y6
y7-NH3
b8-NH3
0.0
50.0
100.0
150.0
200.0
250.0
300.0
350.0
200.00
400.00
600.00
800.00
1000.00
1200.00
1400.00
1600.00

K.NALLSRHYRVRHLAGQTTGGHLLAVPKQRIK.R Scan = 154 CS = 3 Par m/z = 1164.88 gi|172037124 Xcorr = 3.34 delcn = 0.12

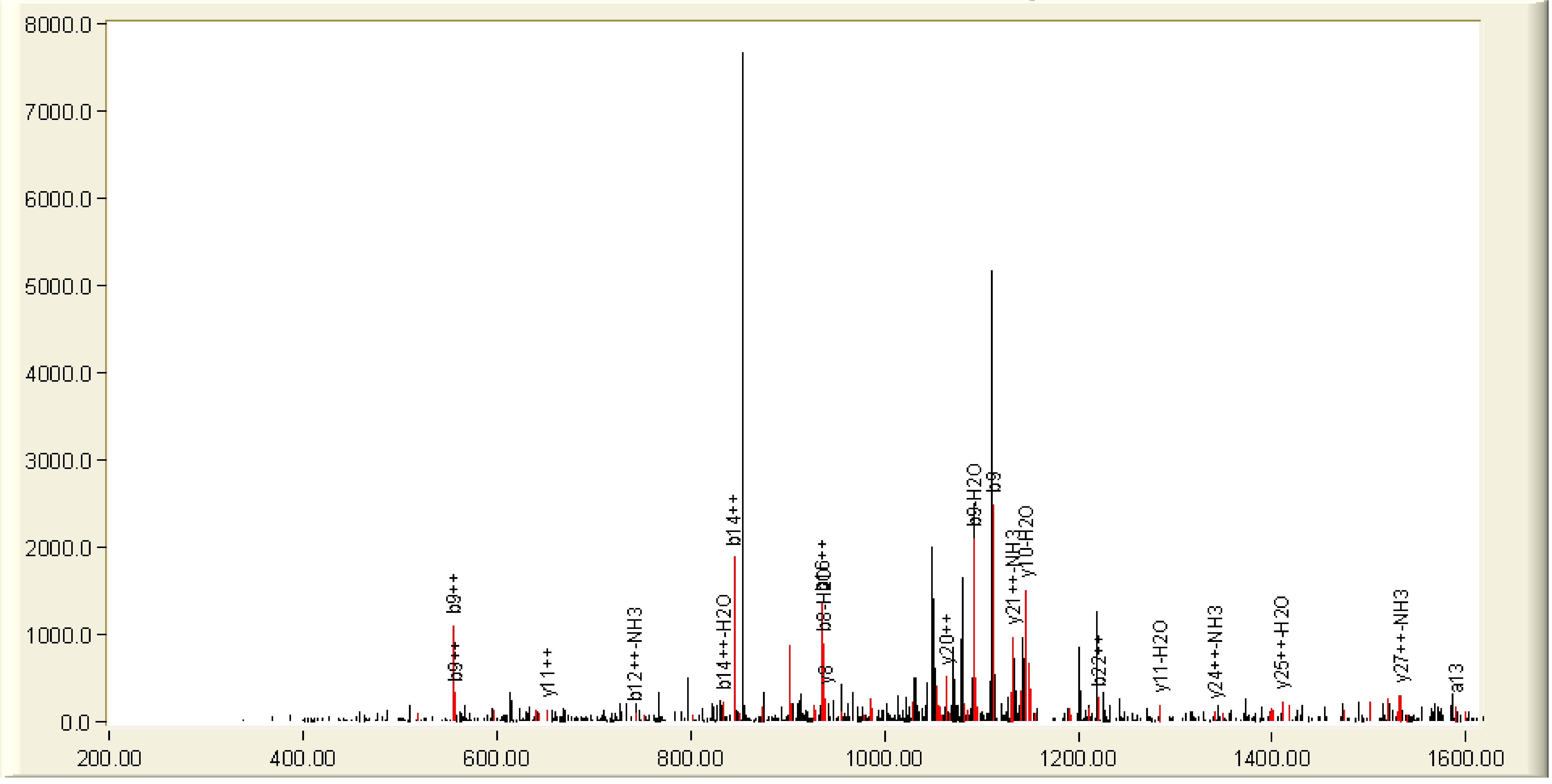

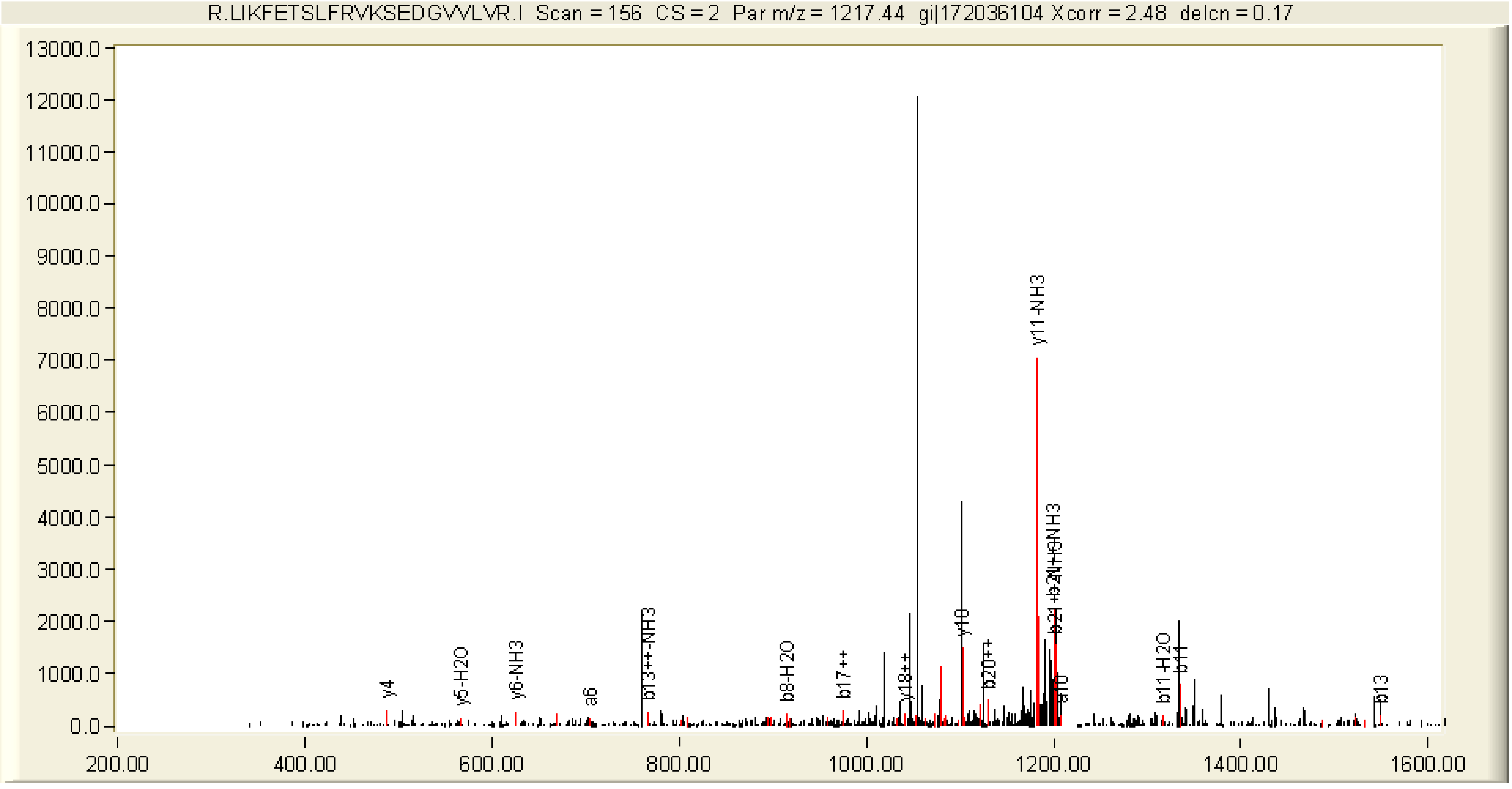
R.LIKFETSLFRVKSEDGVVLVR.I Scan = 156 CS = 2 Par m/z = 1217.44 gi|172036104 Xcorr = 2.48 delcn = 0.17
13000.0
12000.0
11000.0
10000.0
9000.0
8000.0
7000.0
6000.0
5000.0
4000.0
3000.0
2000.0
1000.0
0.0
200.00
400.00
600.00
800.00
1000.00
1200.00
1400.00
1600.00
y4
y5-H2O
y6-NH3
a6
b13++-NH3
b8-H2O
b17++
y18++
y10
b20++
y11-NH3
a10
b11-H2O
b11
b13

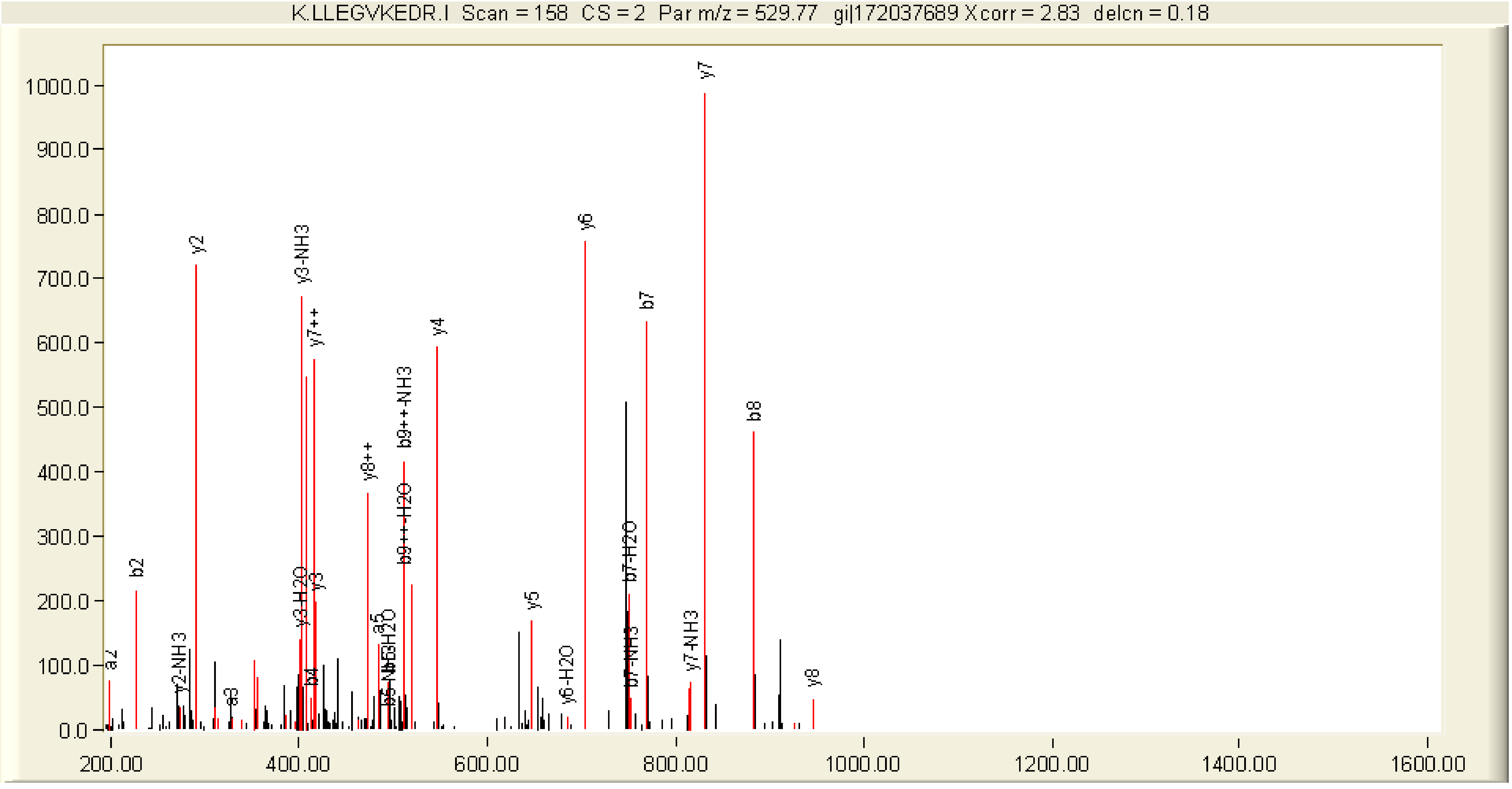
K.LLEGVKEDR.I Scan = 158 CS = 2 Par m/z = 529.77 gi|172037689 Xcorr = 2.83 delcn = 0.18
1000.0
900.0
800.0
700.0
600.0
500.0
400.0
300.0
200.0
100.0
0.0
200.00
400.00
600.00
800.00
1000.00
1200.00
1400.00
1600.00
y7
y6
y2
y3-NH3
b7
y7++
y4
b8
b9++-NH3
y8++
b9++-H2O
b7-H2O
y3-H2O
y3
b2
y5
y6-H2O
y7-NH3
y2-NH3
b7-NH3
b4
a3
y8

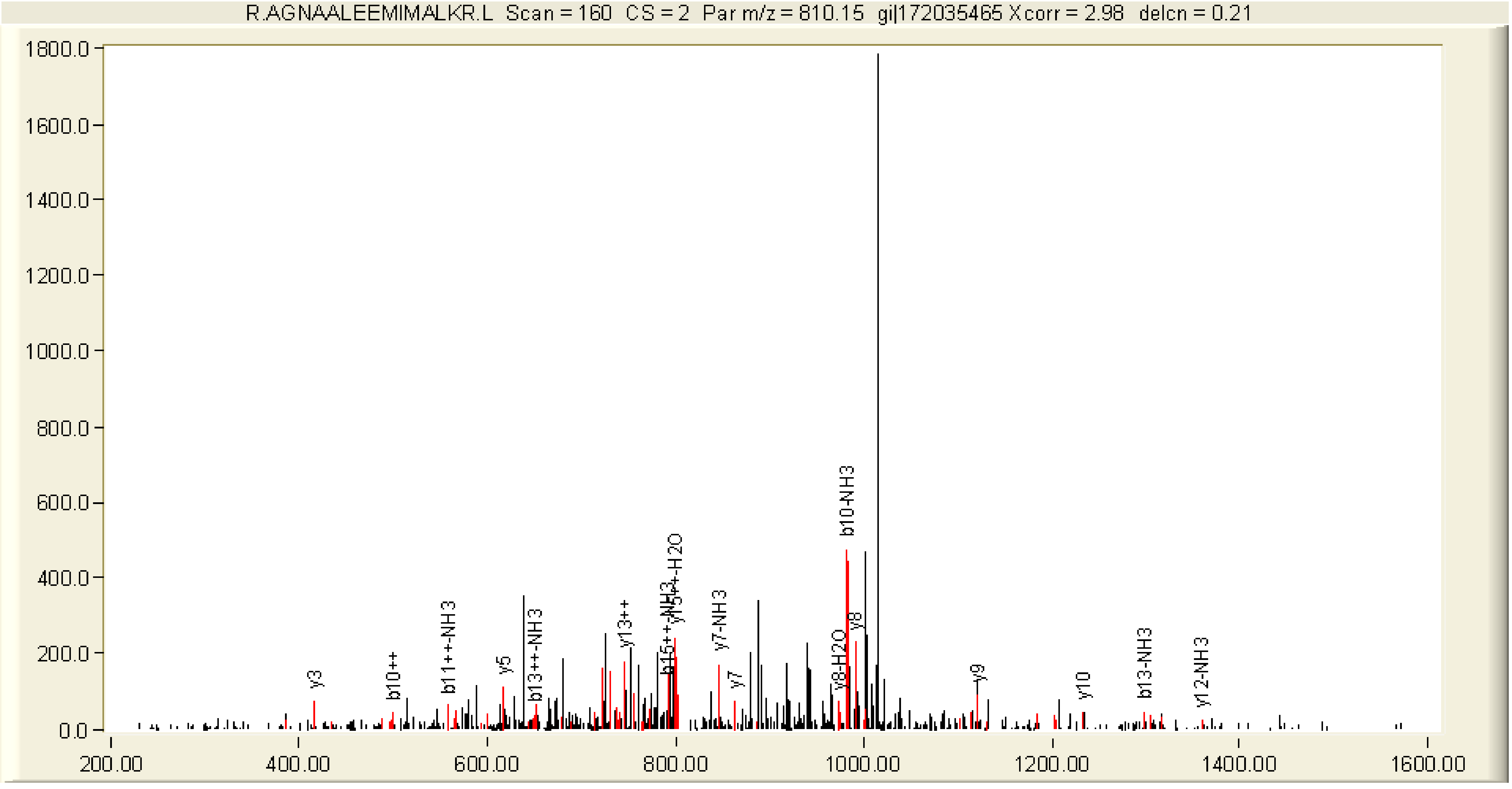

R.AGNAALEEMIMALKR.L Scan = 160 CS = 2 Par m/z = 810.15 gi|172035465 Xcorr = 2.98 delcn = 0.21
1800.0
1600.0
1400.0
1200.0
1000.0
800.0
600.0
400.0
200.0
0.0
200.00
400.00
600.00
800.00
1000.00
1200.00
1400.00
1600.00
y3
b10++
b11++-NH3
y5
b13++-NH3
y13++
y7-NH3
y7
y8-H2O
b10-NH3
y8
y9
y10
b13-NH3
y12-NH3

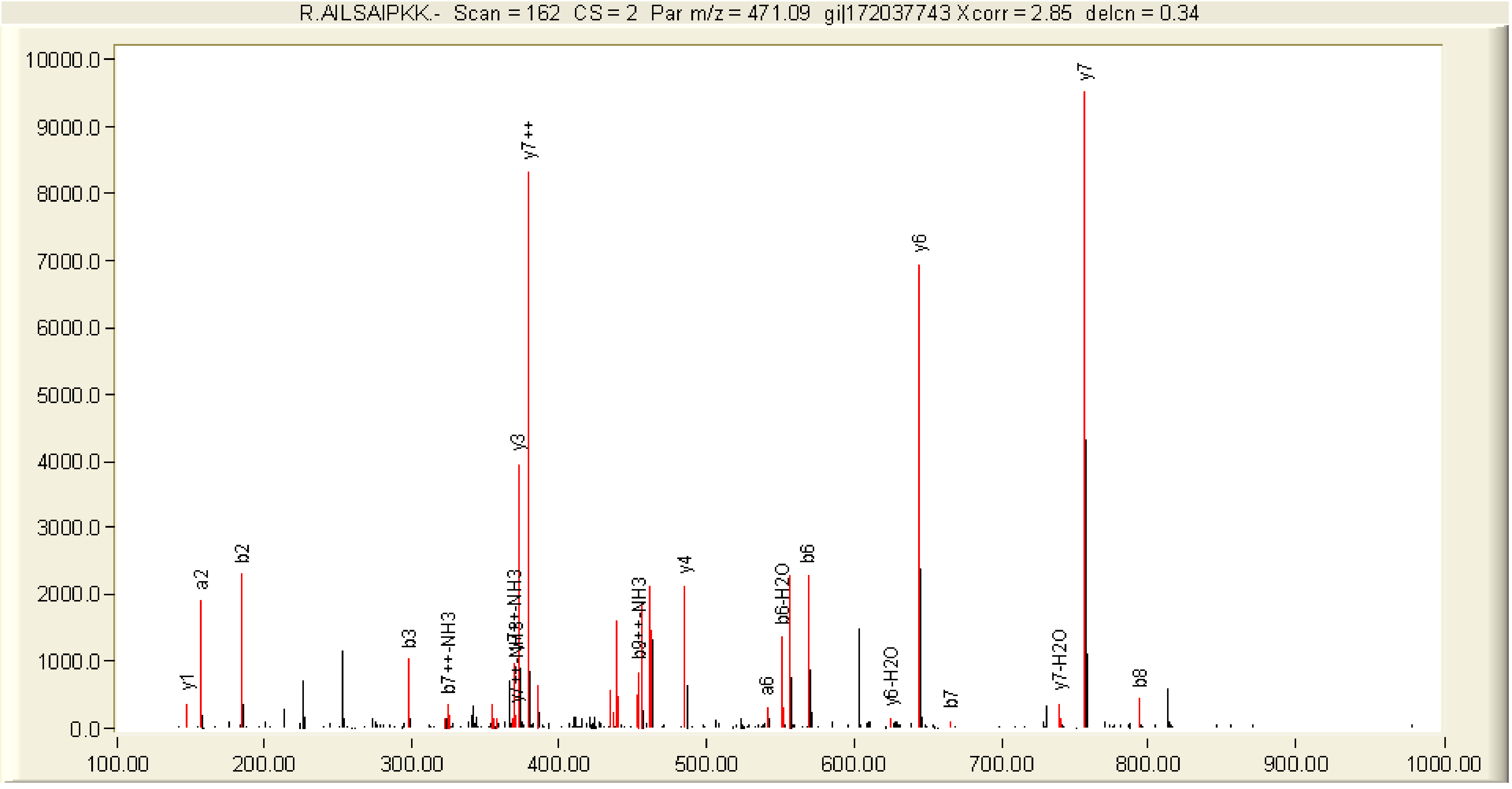
R.AILSAIPKK.- Scan = 162 CS = 2 Par m/z = 471.09 gi|172037743 Xcorr = 2.85 delcn = 0.34
10000.0
9000.0
8000.0
7000.0
6000.0
5000.0
4000.0
3000.0
2000.0
1000.0
0.0
100.00
200.00
300.00
400.00
500.00
600.00
700.00
800.00
900.00
1000.00
y1
a2
b2
b3
b7++-NH3
y3
y7++
b9++-NH3
y4
a6
b6-H2O
b6
y6-H2O
y6
b7
y7-H2O
y7
b8

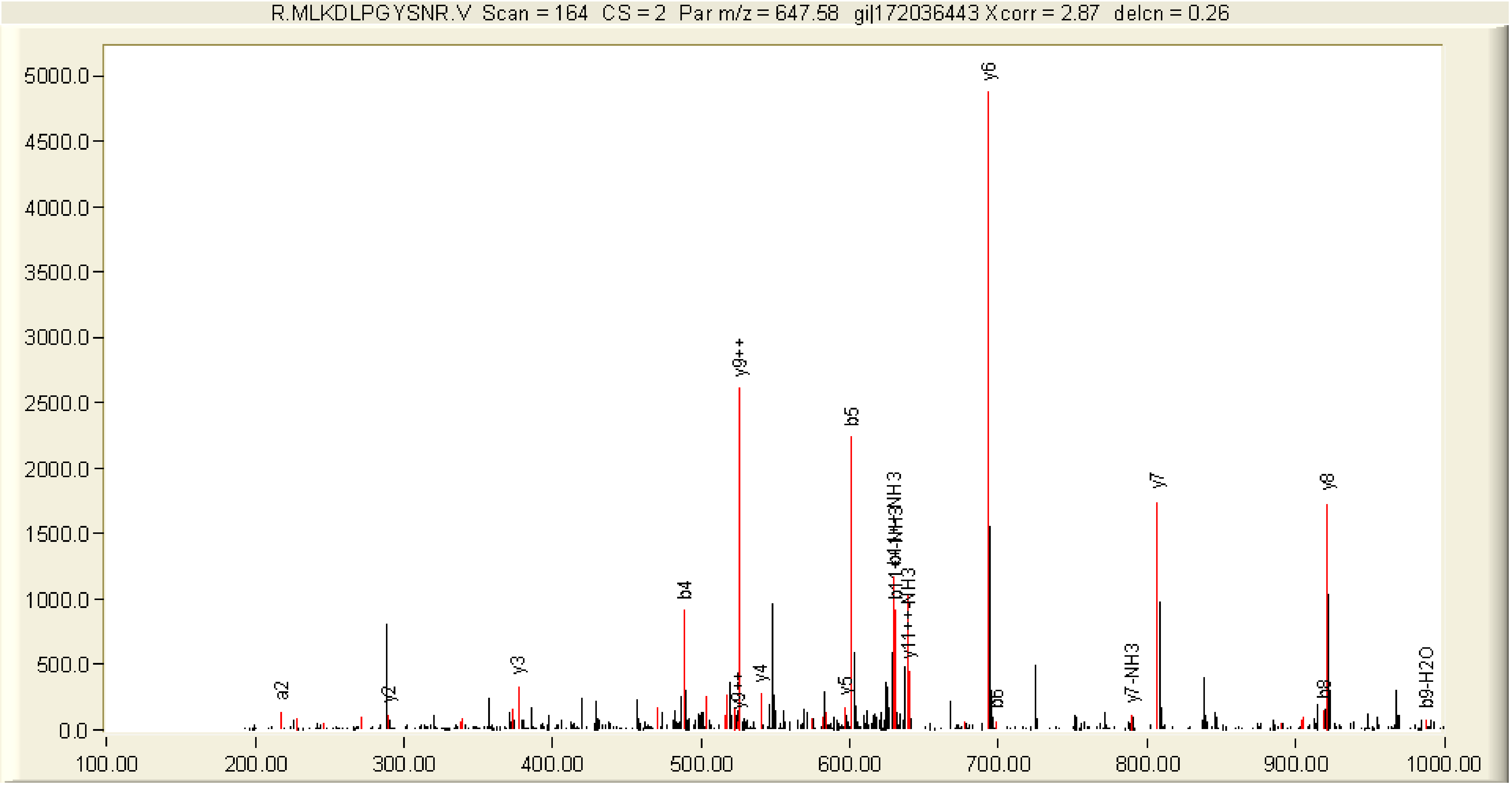
R.MLKDLPGYSNR.V Scan = 164 CS = 2 Par m/z = 647.58 gi|172036443 Xcorr = 2.87 delcn = 0.26
y6
y9++
b5
b4
y7
y8
y3
a2
y2
y4
y5
b6
y7-NH3
b8
b9-H2O
0.0
500.0
1000.0
1500.0
2000.0
2500.0
3000.0
3500.0
4000.0
4500.0
5000.0
100.00
200.00
300.00
400.00
500.00
600.00
700.00
800.00
900.00
1000.00

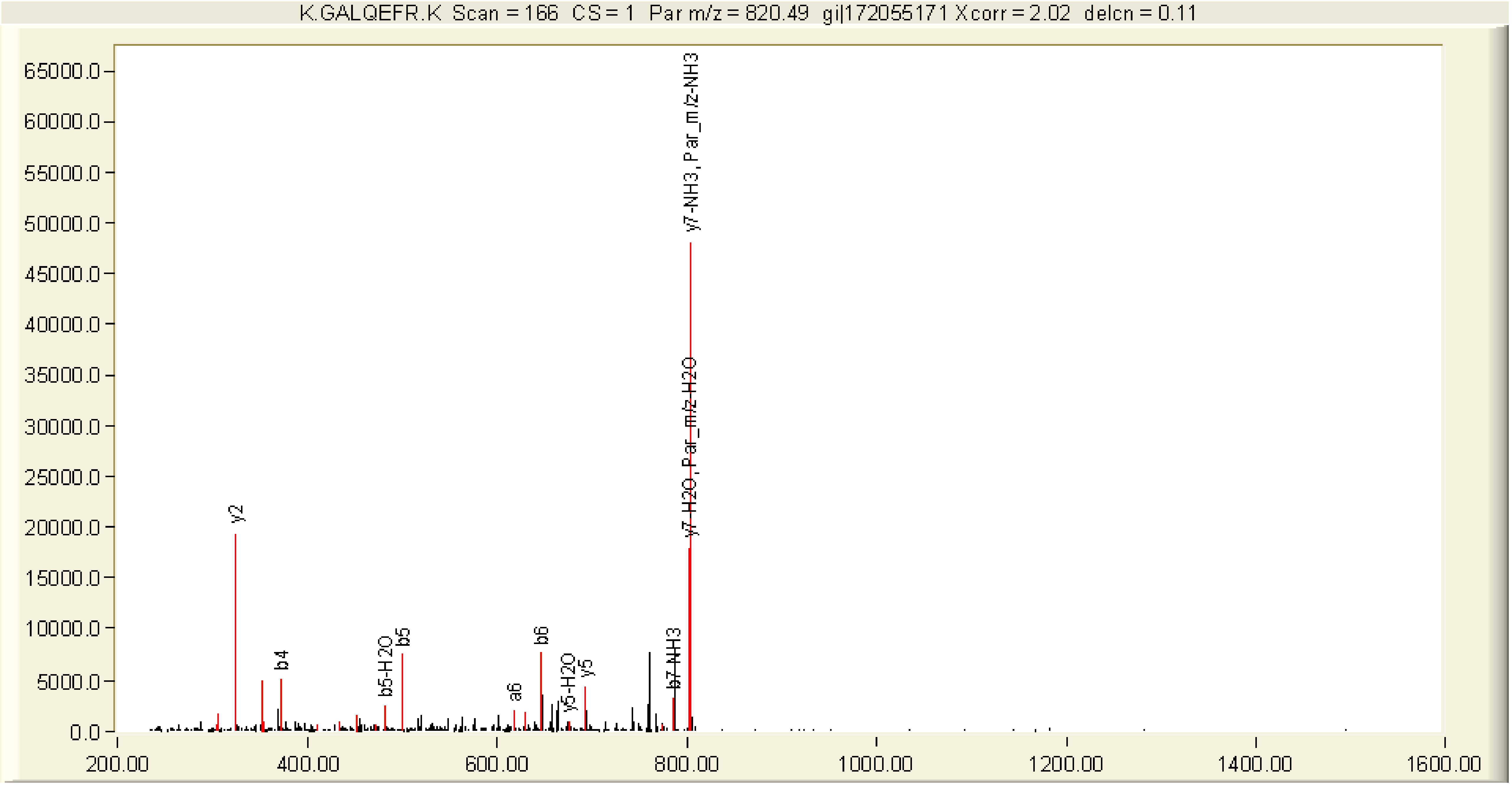

K.GALQEFR.K Scan = 166 CS = 1 Par m/z = 820.49 gi|172055171 Xcorr = 2.02 delcn = 0.11
65000.0
60000.0
55000.0
50000.0
45000.0
40000.0
35000.0
30000.0
25000.0
20000.0
15000.0
10000.0
5000.0
0.0
200.00
400.00
600.00
800.00
1000.00
1200.00
1400.00
1600.00
y2
b4
b5-H2O
b5
a6
b6
y5-H2O
y5
b7-NH3
y7-H2O, Par_m/z-H2O
y7-NH3, Par_m/z-NH3

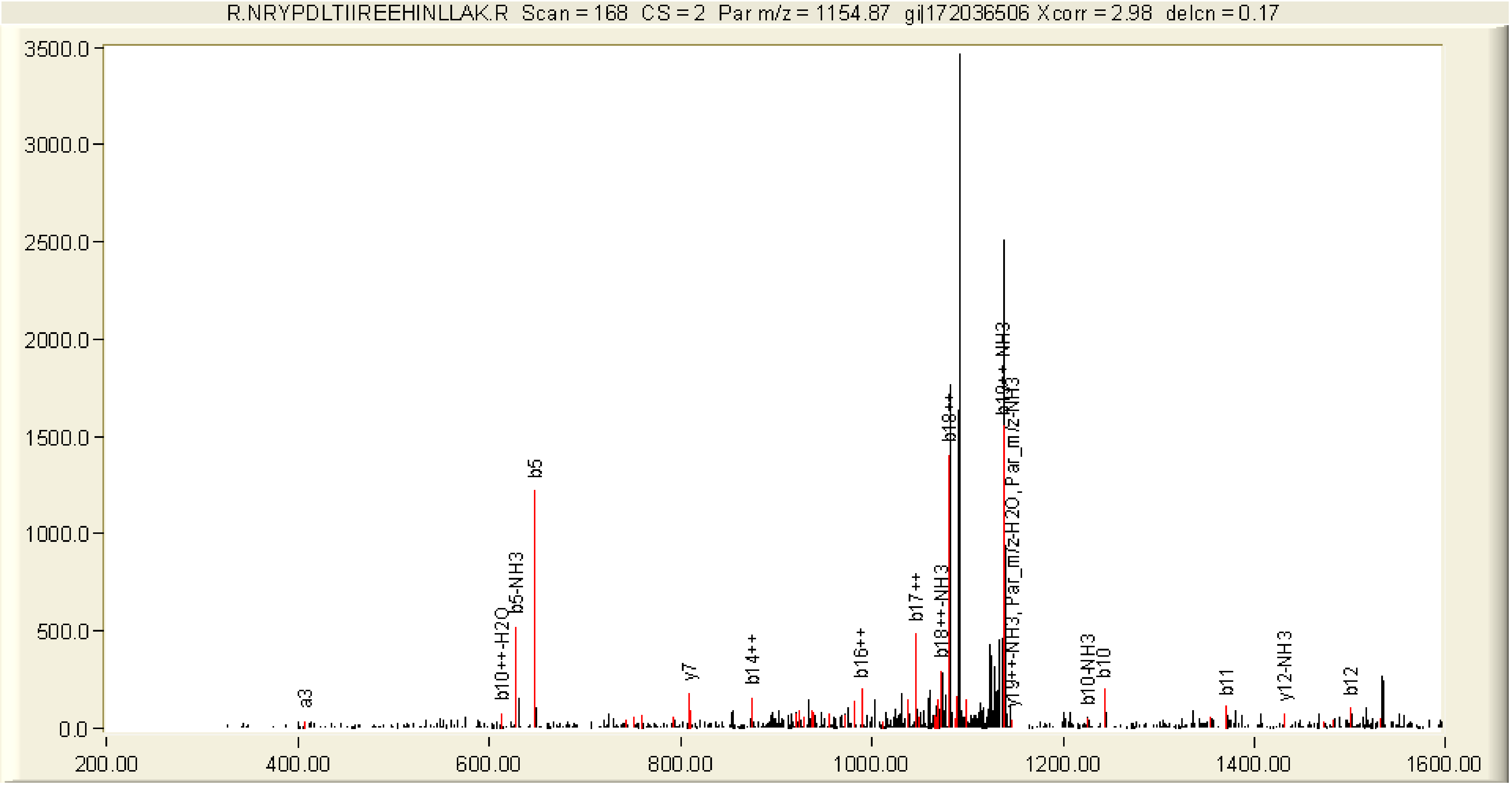

R.NRYPDLTIIREEHINLLAK.R Scan = 168 CS = 2 Par m/z = 1154.87 gi|172036506 Xcorr = 2.98 delcn = 0.17
3500.0
3000.0
2500.0
2000.0
1500.0
1000.0
500.0
0.0
200.00
400.00
600.00
800.00
1000.00
1200.00
1400.00
1600.00
a3
b10++-H2O
b5-NH3
b5
y7
b14++
b16++
b17++
b18++-NH3
b18++
b19++-NH3
y19++-NH3, Par_m/z-H2O, Par_m/z-NH3
b10-NH3
b10
b11
y12-NH3
b12

R.KFNVEIDRYNLPYFIPHR.C   Scan = 170   CS = 2   Par m/z = 1161.43   gi|172039146 Xcorr = 2.47   delcn = 0.28

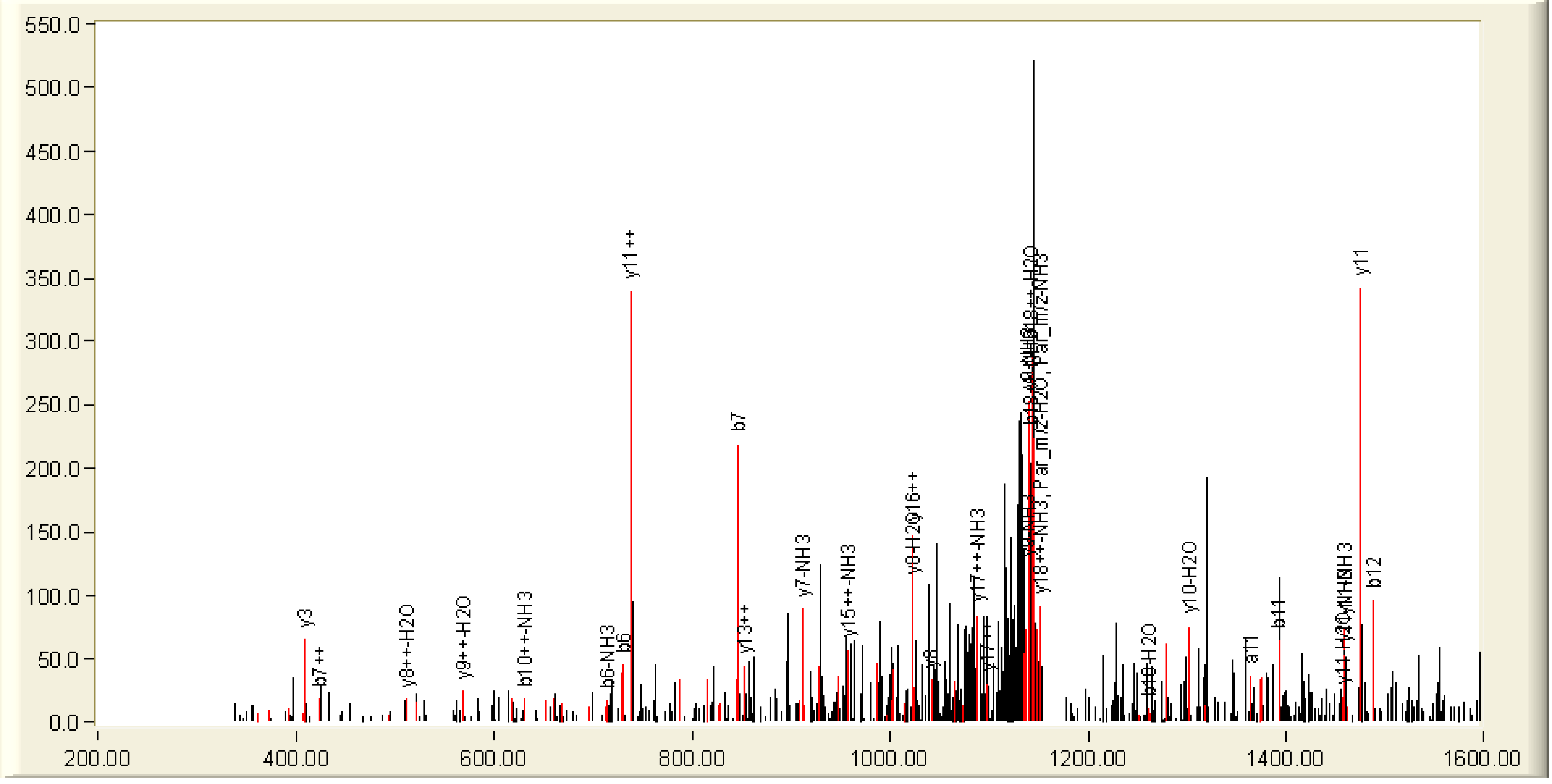

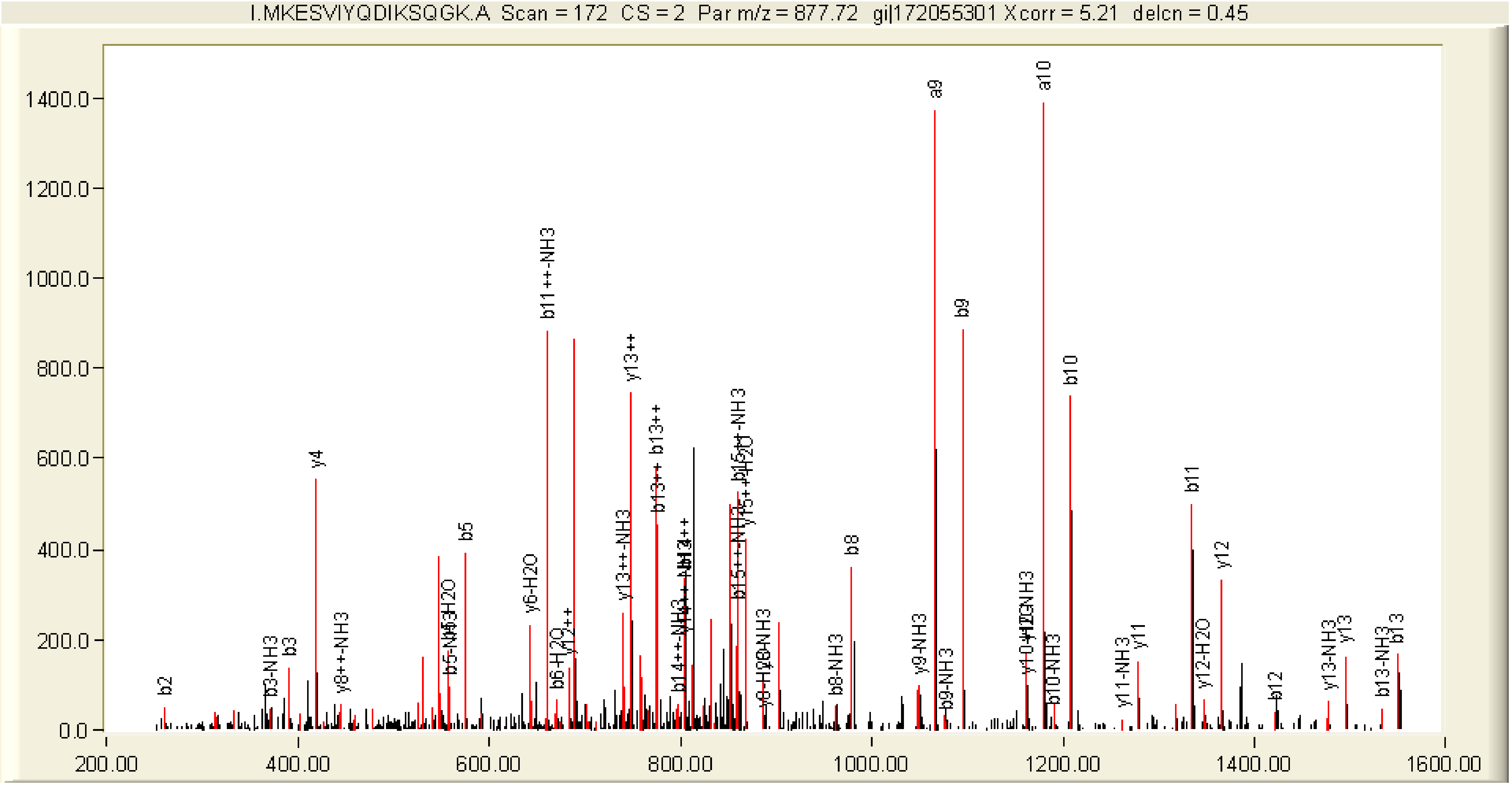

I.MKESVIYQDIKSQGK.A Scan = 172 CS = 2 Par m/z = 877.72 gi|172055301 Xcorr = 5.21 delcn = 0.45
1400.0
1200.0
1000.0
800.0
600.0
400.0
200.0
0.0
200.00
400.00
600.00
800.00
1000.00
1200.00
1400.00
1600.00
b2
b3-NH3
b3
y4
y8++-NH3
b5
y6-H2O
b11++-NH3
y13++
y13++-NH3
b13++
b8
b8-NH3
y9-NH3
b9-NH3
a9
b9
a10
b10
b10-NH3
y11-NH3
y11
b11
y12-H2O
y12
b12
y13-NH3
y13
b13-NH3
b13

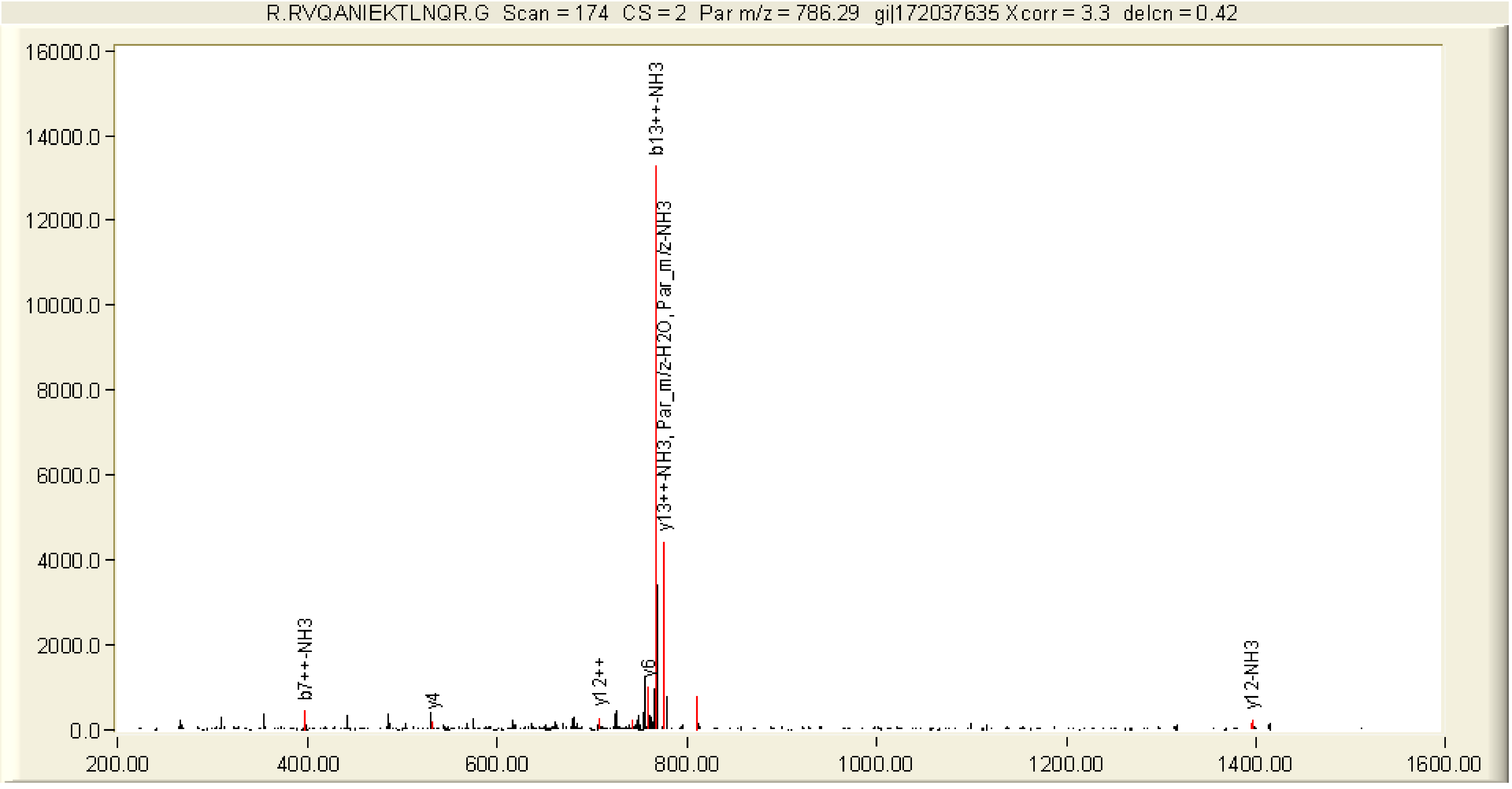

R.RVQANIEKTLNQR.G Scan = 174 CS = 2 Par m/z = 786.29 gi|172037635 Xcorr = 3.3 delcn = 0.42
b13++-NH3
y13++-NH3, Par_m/z-H2O, Par_m/z-NH3
b7++-NH3
y4
y12++
y6
y12-NH3
16000.0
14000.0
12000.0
10000.0
8000.0
6000.0
4000.0
2000.0
0.0
200.00
400.00
600.00
800.00
1000.00
1200.00
1400.00
1600.00

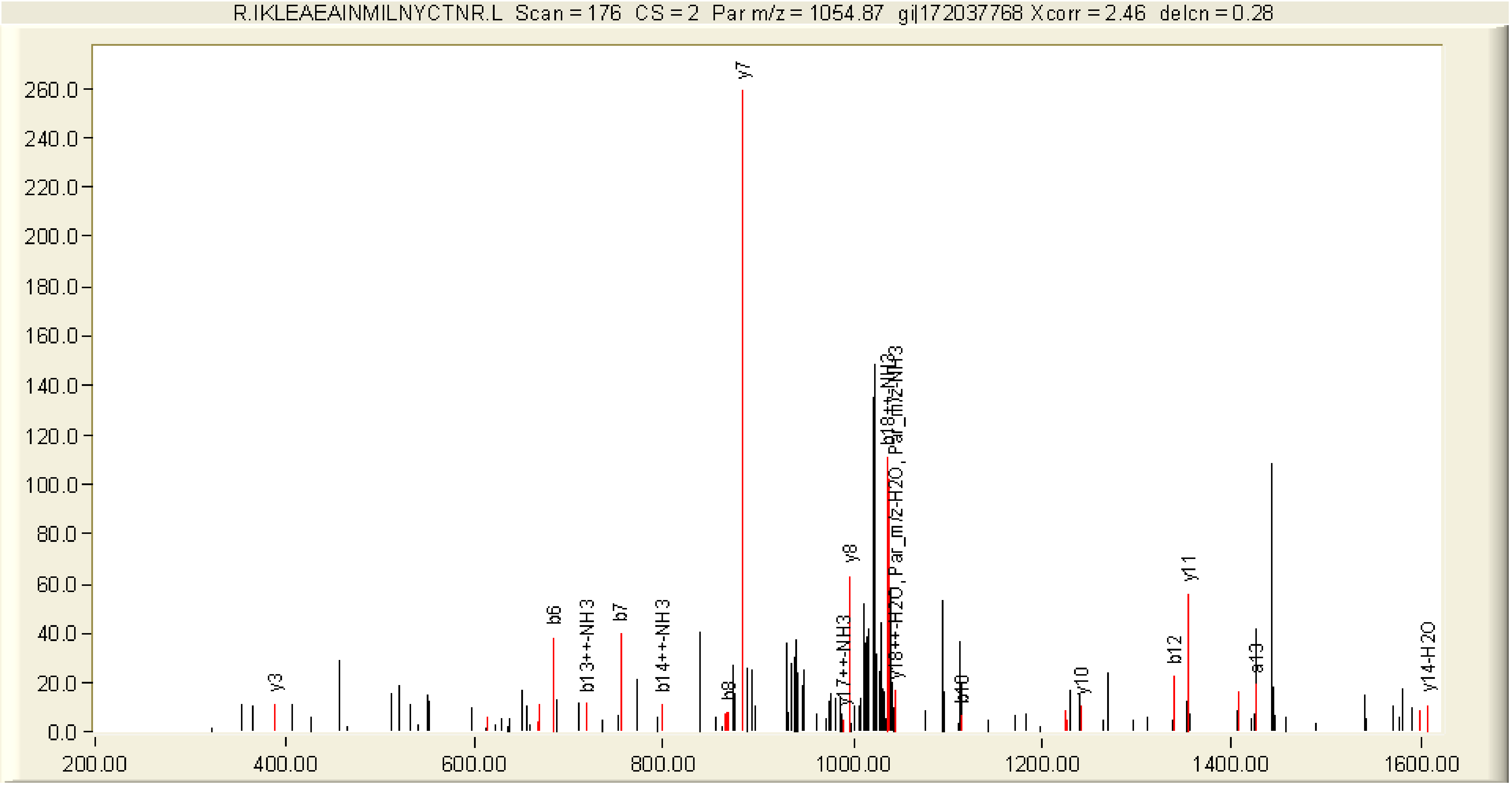
R.IKLEAEAINMILNYCTNR.L Scan = 176 CS = 2 Par m/z = 1054.87 gi|172037768 Xcorr = 2.46 delcn = 0.28
260.0
240.0
220.0
200.0
180.0
160.0
140.0
120.0
100.0
80.0
60.0
40.0
20.0
0.0
200.00
400.00
600.00
800.00
1000.00
1200.00
1400.00
1600.00
y3
b6
b13++-NH3
b7
b14++-NH3
b8
y7
y17++-NH3
y8
y18++-H2O, Par_m/z-H2O, Par_m/z-NH3
b18++-NH3
b10
y10
b12
y11
a13
y14-H2O

K.RIETLLLALLATARSNIPSLLTSPTATNVGSSPTVISVR.S Scan = 178 CS = 3 Par m/z = 1345.16 gi|172054905 Xcorr = 3.34 delcn = 0.16

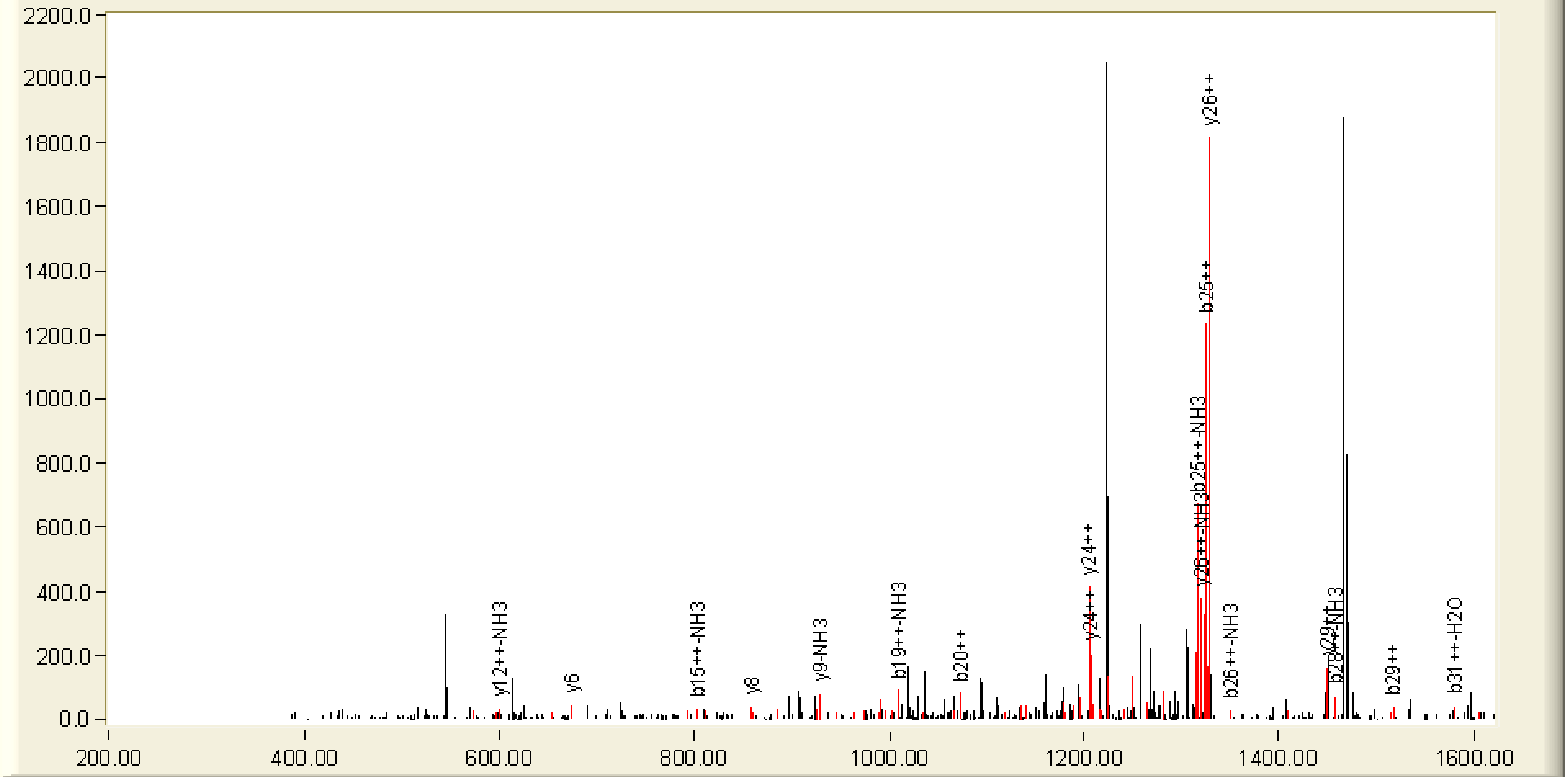

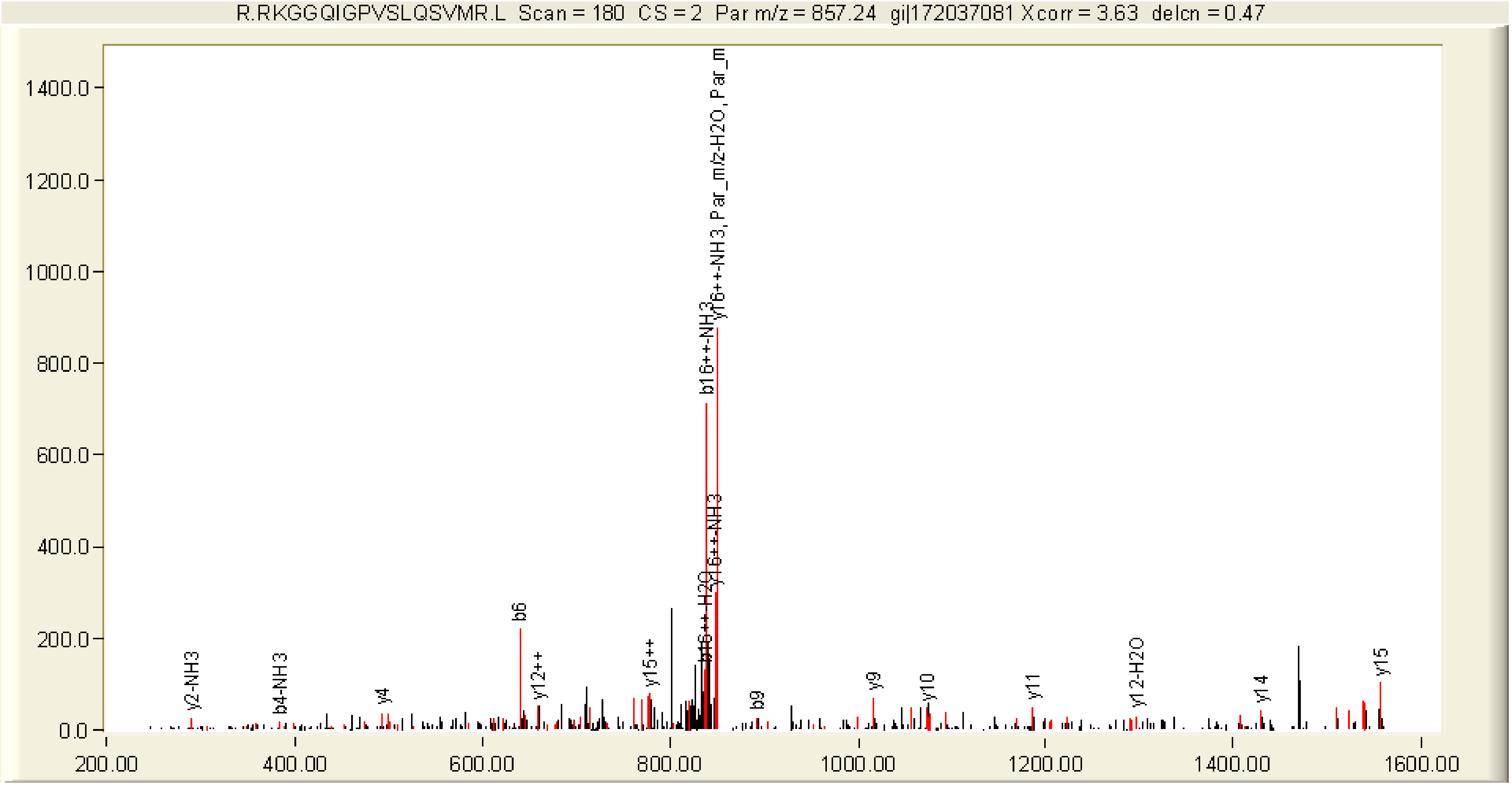

R.RKGGQIGPVSLQSVMR.L Scan = 180 CS = 2 Par m/z = 857.24 gi|172037081 Xcorr = 3.63 delcn = 0.47
1400.0
1200.0
1000.0
800.0
600.0
400.0
200.0
0.0
200.00
400.00
600.00
800.00
1000.00
1200.00
1400.00
1600.00
y2-NH3
b4-NH3
y4
b6
y12++
y15++
b16++-H2O
b16++-NH3
y16++-NH3
y16++-NH3, Par_m/z-H2O, Par_m
b9
y9
y10
y11
y12-H2O
y14
y15

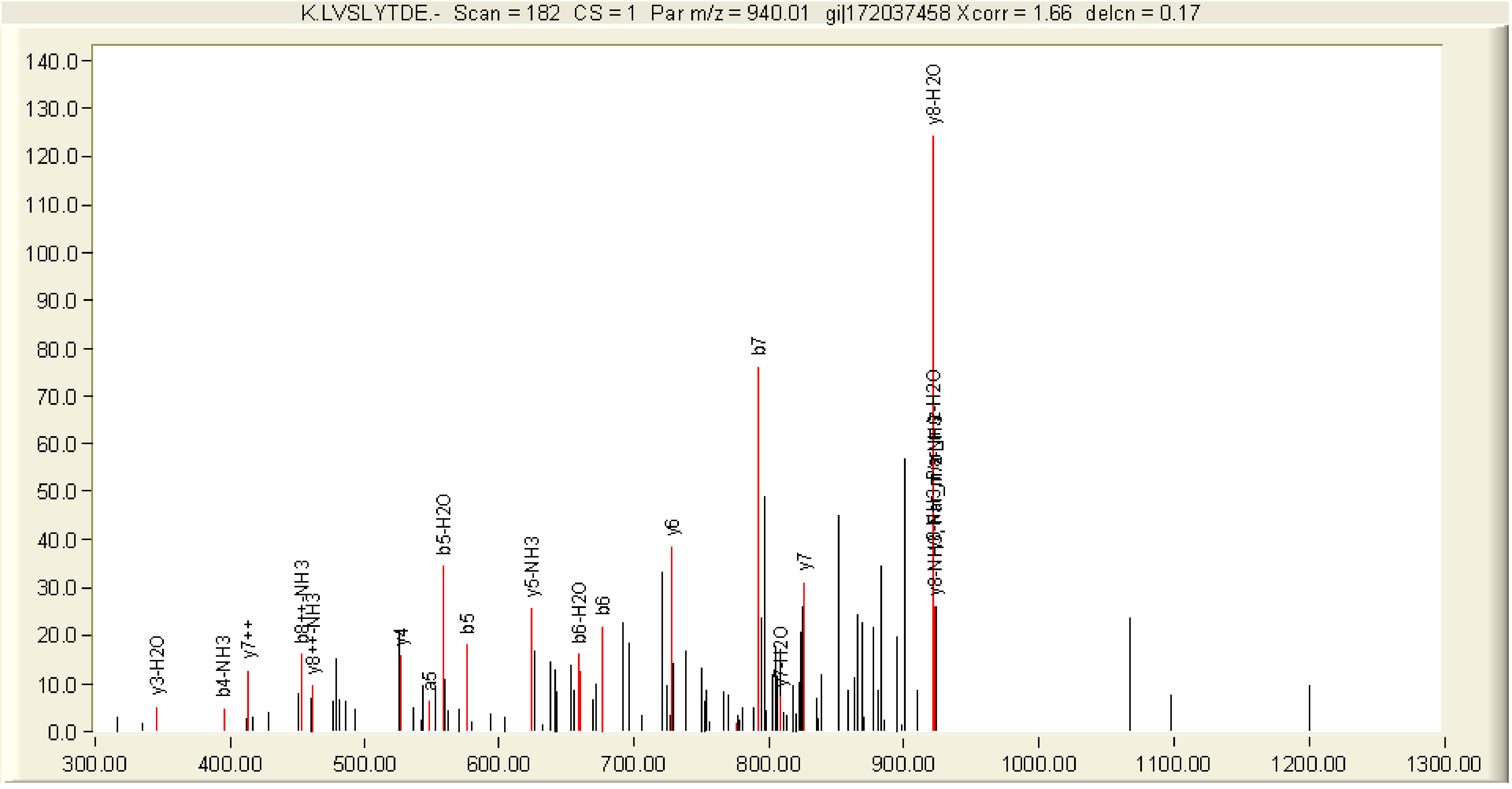
K.LVSLYTDE.- Scan = 182 CS = 1 Par m/z = 940.01 gi|172037458 Xcorr = 1.66 delcn = 0.17
y8-H2O
b7
y6
y7
b5-H2O
y5-NH3
b6-H2O
b6
b5
a5
y4
y3-H2O
b4-NH3
y7++
y8-NH3
0.0
10.0
20.0
30.0
40.0
50.0
60.0
70.0
80.0
90.0
100.0
110.0
120.0
130.0
140.0
300.00
400.00
500.00
600.00
700.00
800.00
900.00
1000.00
1100.00
1200.00
1300.00

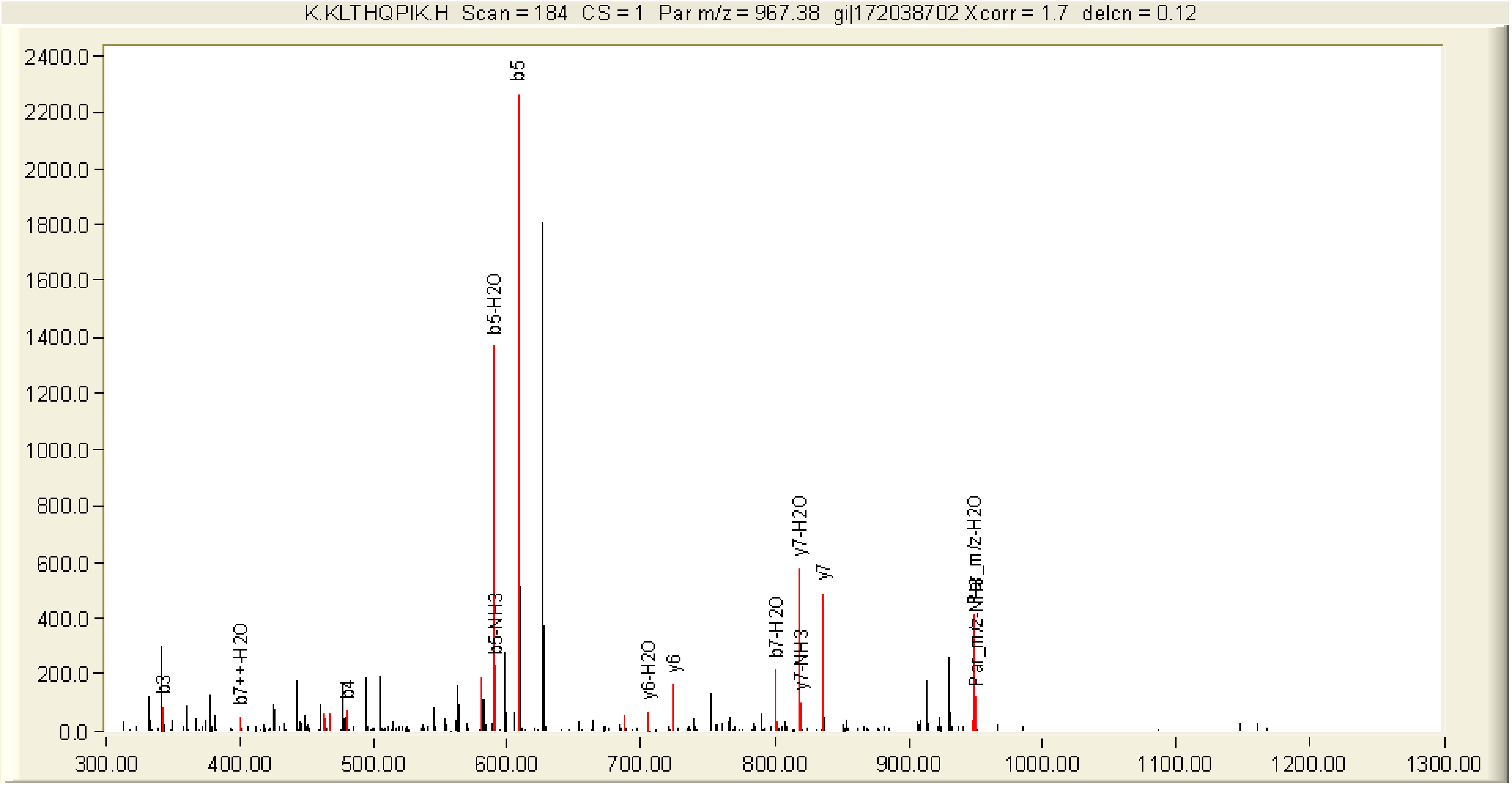
K.KLTHQPIK.H Scan = 184 CS = 1 Par m/z = 967.38 gi|172038702 Xcorr = 1.7 delcn = 0.12
b5
b5-H2O
b5-NH3
b3
b7++H2O
b4
y6-H2O
y6
b7-H2O
y7-H2O
y7-NH3
y7
Par_m/z-H2O
2400.0
2200.0
2000.0
1800.0
1600.0
1400.0
1200.0
1000.0
800.0
600.0
400.0
200.0
0.0
300.00
400.00
500.00
600.00
700.00
800.00
900.00
1000.00
1100.00
1200.00
1300.00

K.NQYLGEAIVTFIGLELGAMSHSLSDYWVSHFK.Q Scan = 186 CS = 3 Par m/z = 1206.41 gi|172036796 Xcorr = 3.42 delcn = 0.21

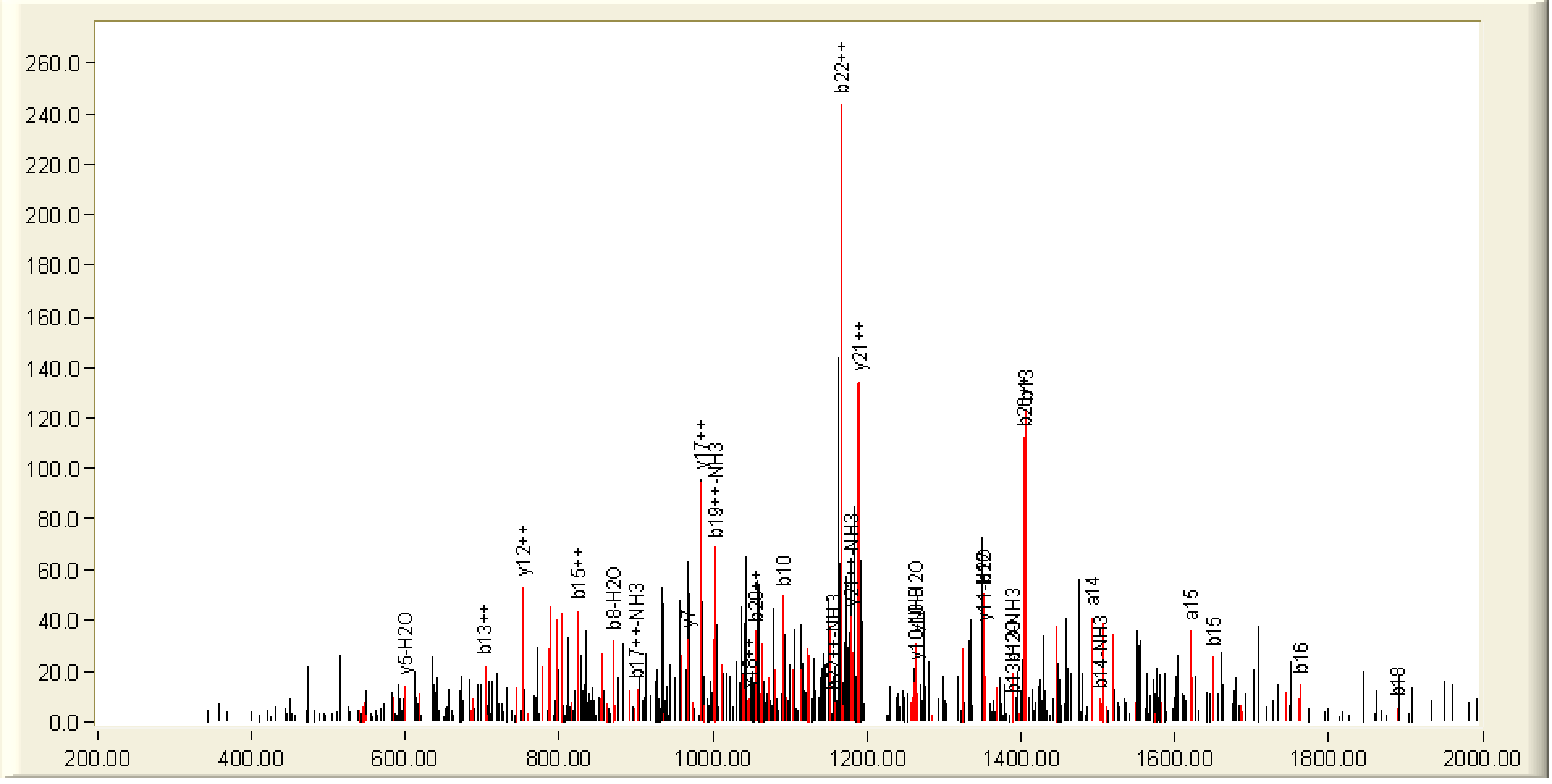

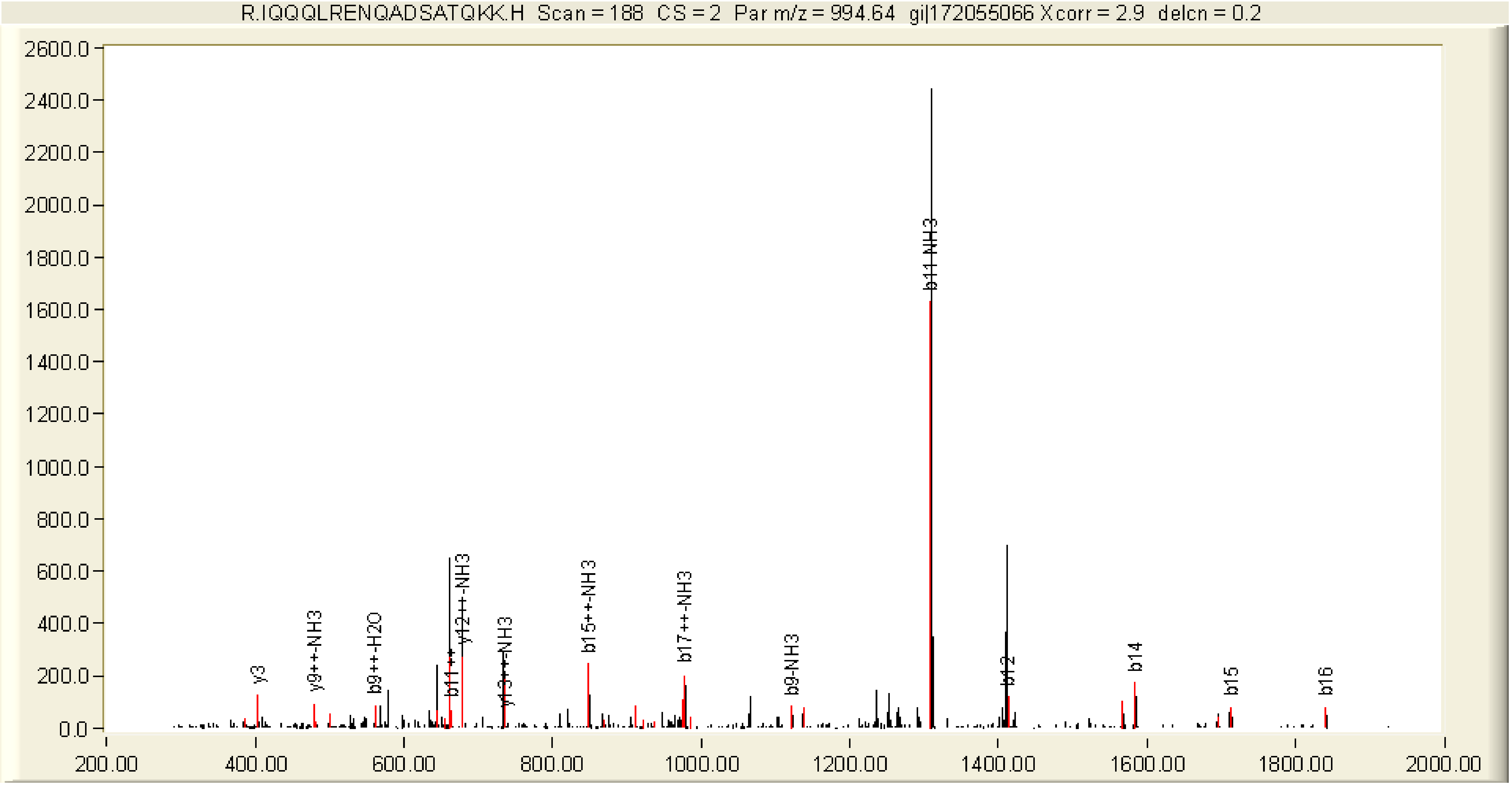
R.IQQQLRENQADSATQKK.H Scan = 188 CS = 2 Par m/z = 994.64 gi|172055066 Xcorr = 2.9 delcn = 0.2
2600.0
2400.0
2200.0
2000.0
1800.0
1600.0
1400.0
1200.0
1000.0
800.0
600.0
400.0
200.0
0.0
200.00
400.00
600.00
800.00
1000.00
1200.00
1400.00
1600.00
1800.00
2000.00
y3
y9++-NH3
b9++-H2O
b11++
y12++-NH3
y13++-NH3
b15++-NH3
b17++-NH3
b9-NH3
b11-NH3
b12
b14
b15
b16

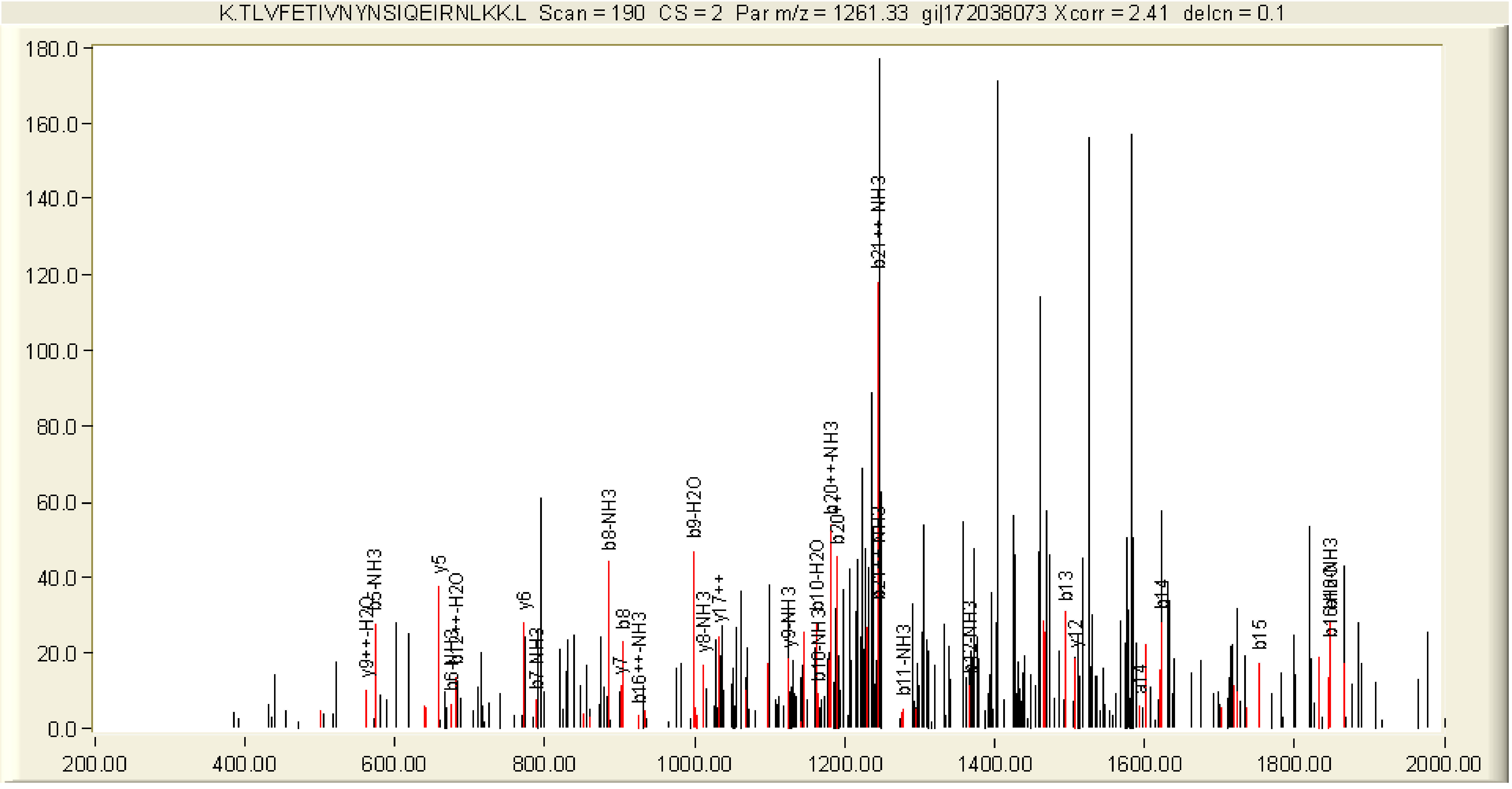

K.TLVFETIVNYNSIQEIRNLKK.L Scan = 190 CS = 2 Par m/z = 1261.33 gi|172038073 Xcorr = 2.41 delcn = 0.1
180.0
160.0
140.0
120.0
100.0
80.0
60.0
40.0
20.0
0.0
200.00
400.00
600.00
800.00
1000.00
1200.00
1400.00
1600.00
1800.00
2000.00
b21++-NH3
b20++-NH3
b9-H2O
b8-NH3
b5-NH3
y5
y6
b8
y7
y17++
b13
b15
b11-NH3

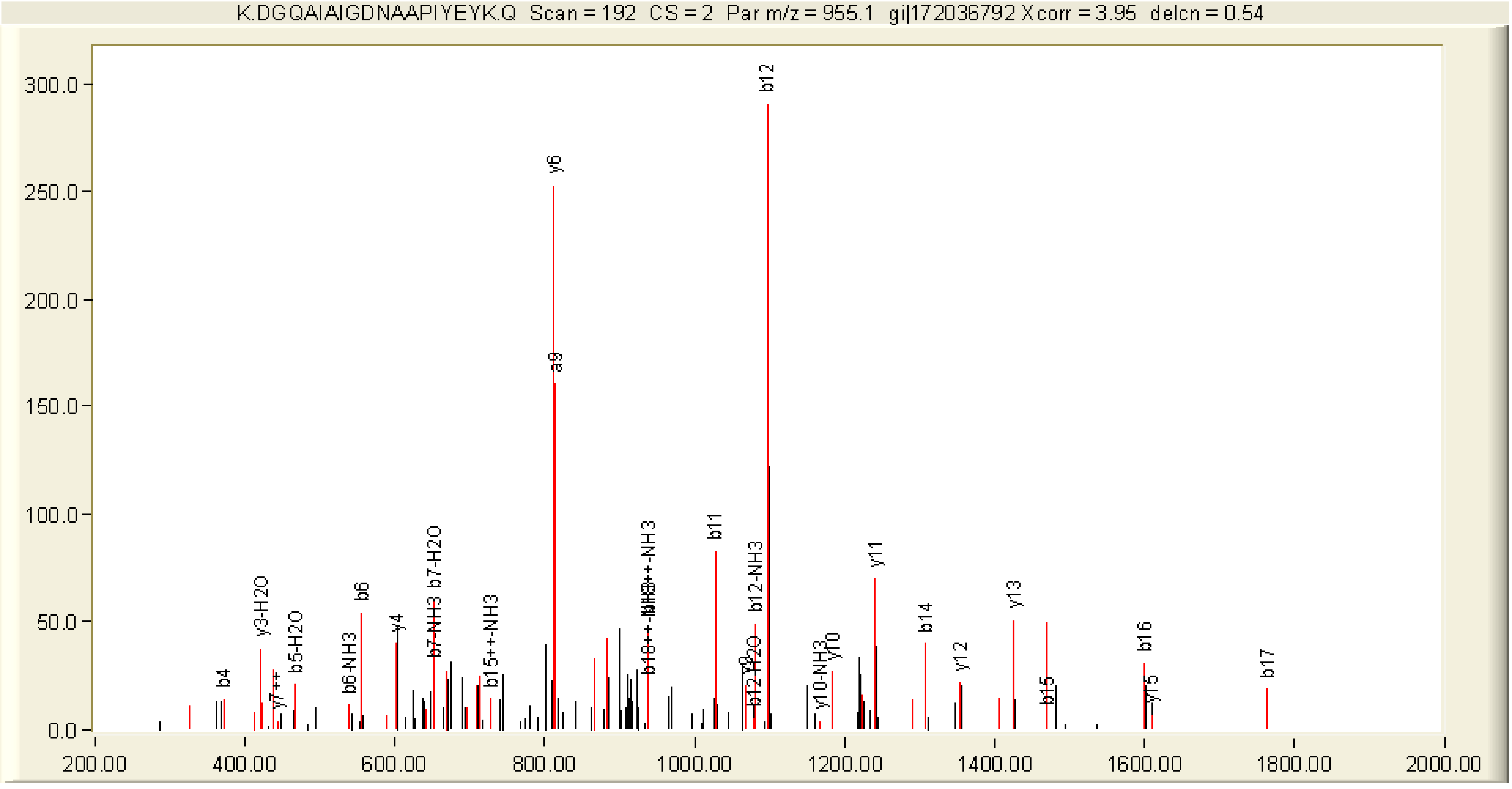
K.DGQAIAIGDNAAPIYEYK.Q Scan = 192 CS = 2 Par m/z = 955.1 gi|172036792 Xcorr = 3.95 delcn = 0.54
300.0
250.0
200.0
150.0
100.0
50.0
0.0
200.00
400.00
600.00
800.00
1000.00
1200.00
1400.00
1600.00
1800.00
2000.00
b12
y6
a9
b11
y11
b12-NH3
b7-NH3 b7-H2O
b15++-NH3
y3-H2O
b5-H2O
b6
b6-NH3
y4
y7++
b4
b12-H2O
y10-NH3
y10
b14
y12
y13
b15
b16
y15
b17

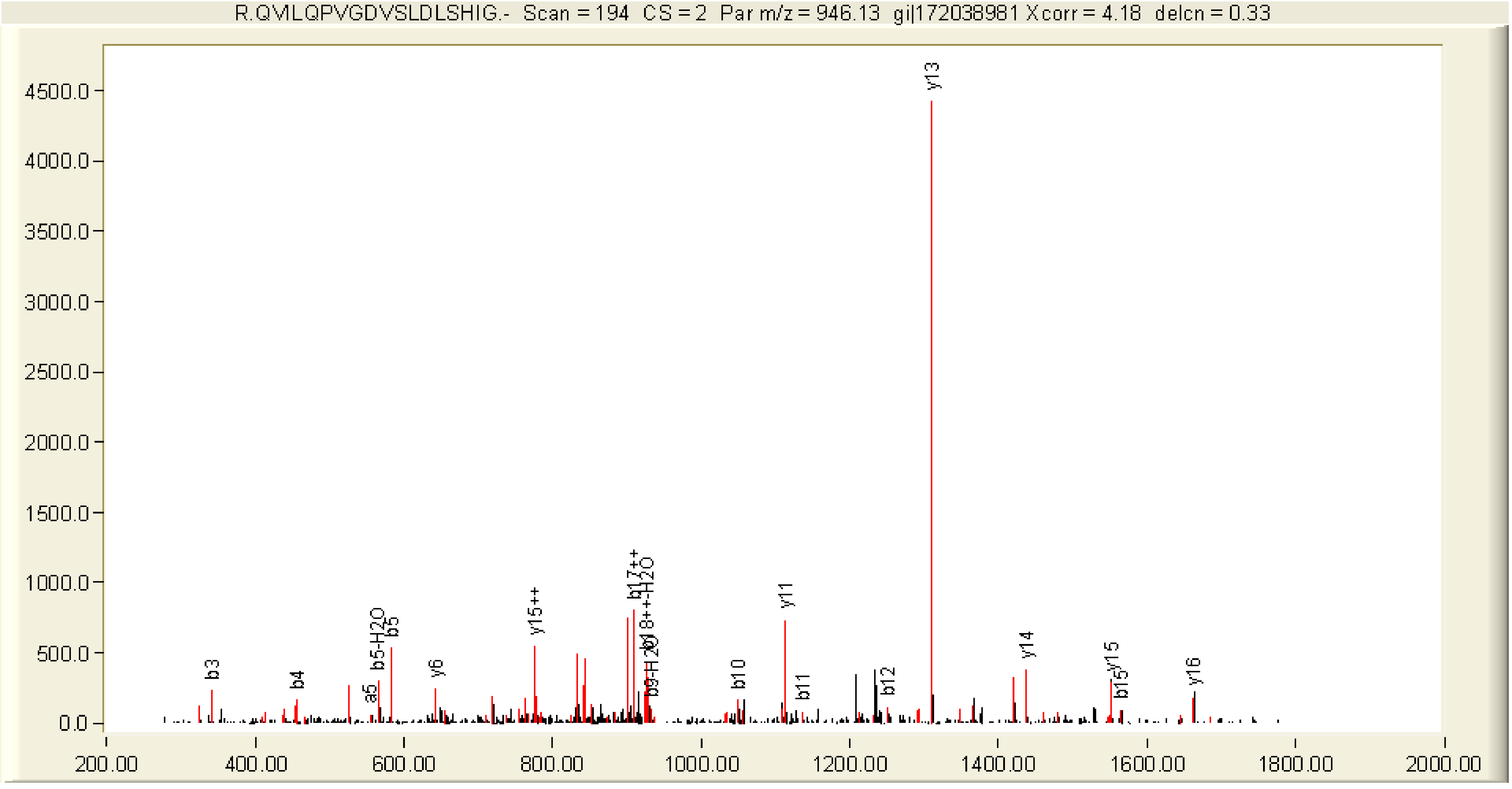
R.QVILQPVGDVSLDLSHIG.- Scan = 194 CS = 2 Par m/z = 946.13 gi|172038981 Xcorr = 4.18 delcn = 0.33
4500.0
4000.0
3500.0
3000.0
2500.0
2000.0
1500.0
1000.0
500.0
0.0
200.00
400.00
600.00
800.00
1000.00
1200.00
1400.00
1600.00
1800.00
2000.00
b3
b4
a5
b5-H2O
b5
y6
y15++
b17++
b10
y11
b11
b12
y13
y14
b15
y15
y16

K.QLATDQSVNEVNLAFLTEHGYL.- Scan = 196 CS = 2 Par m/z = 1231.76 gi|172037257 Xcorr = 3.57 delcn = 0.44

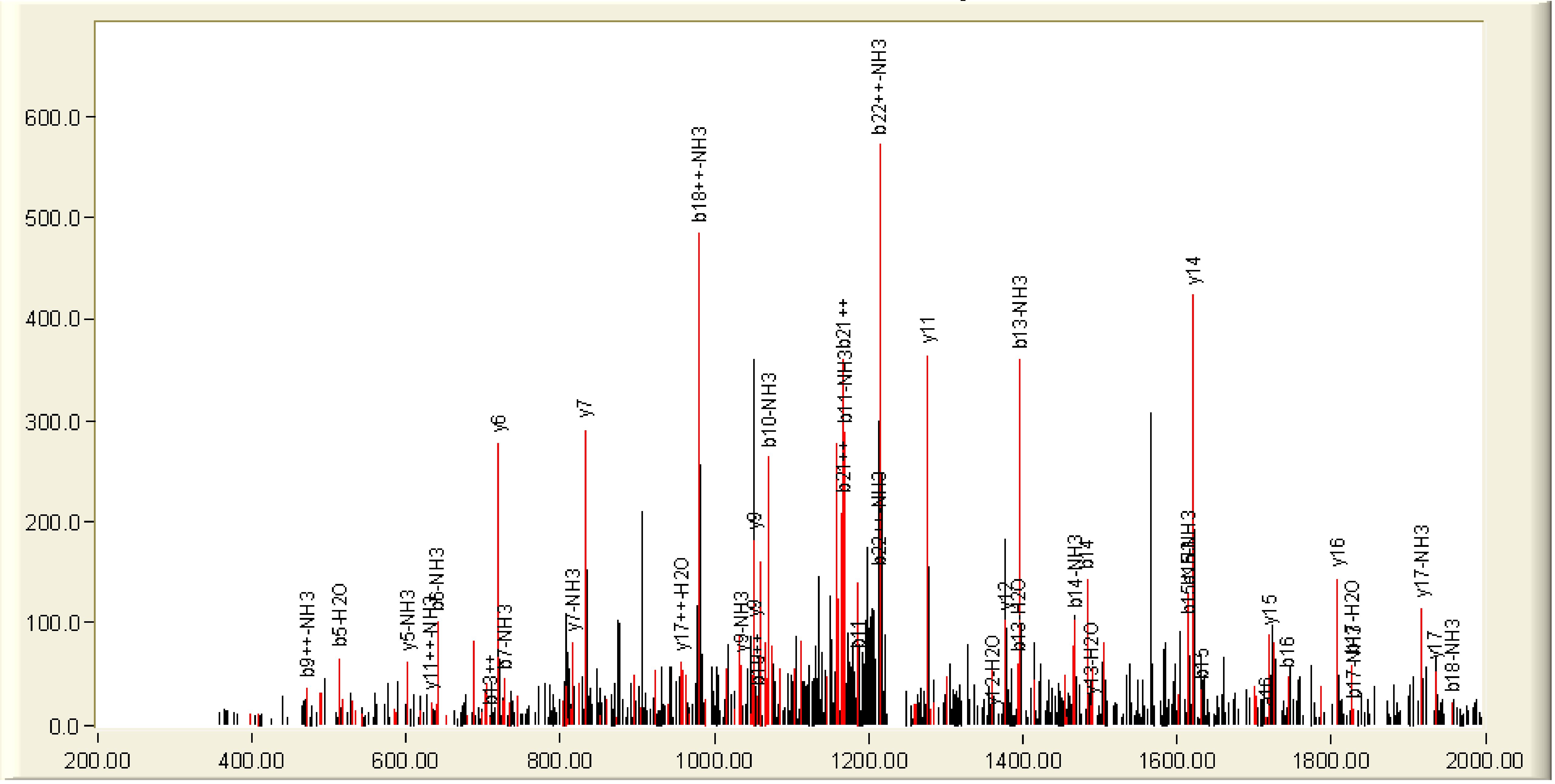

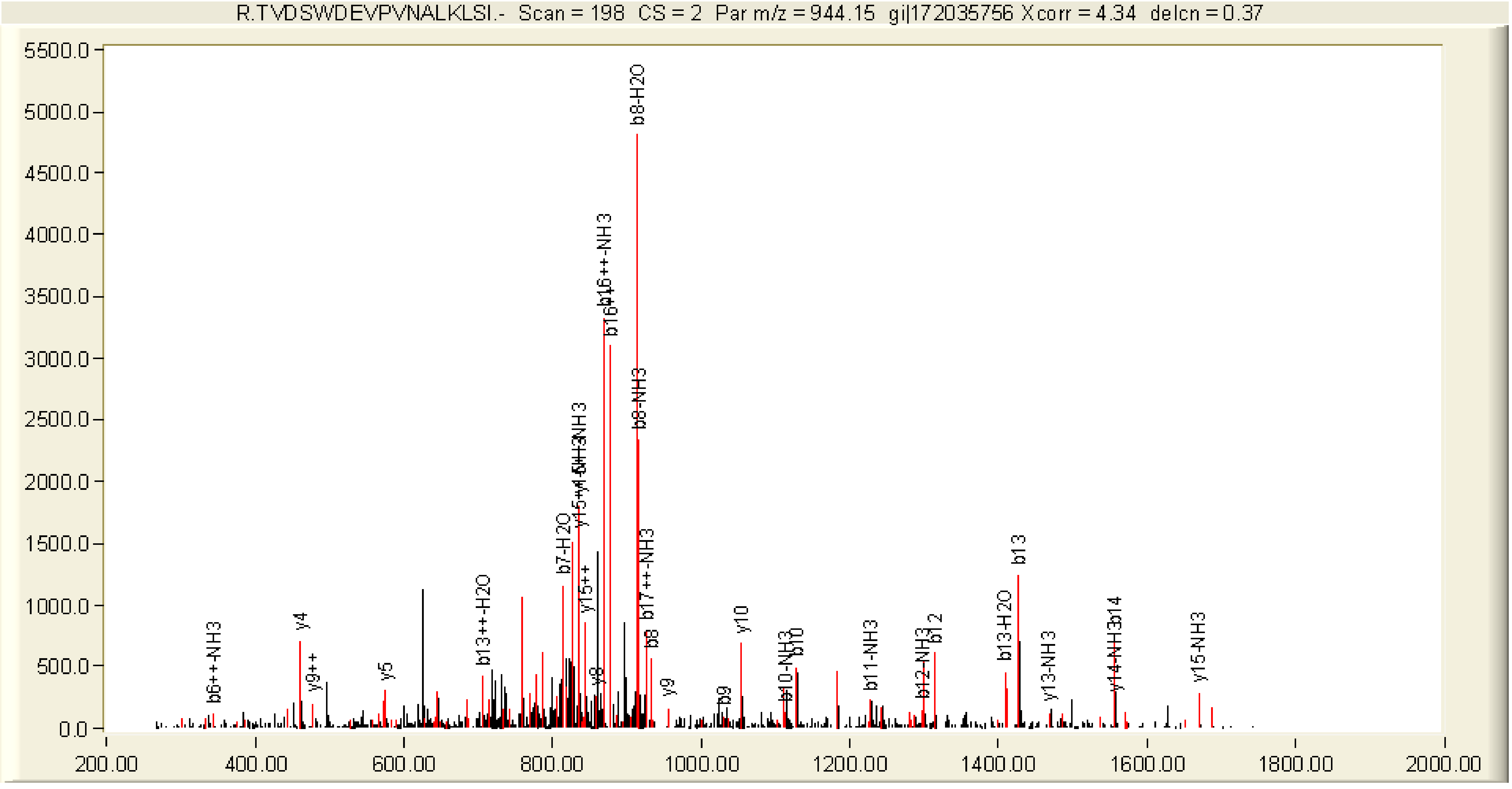

R.TVDSWDEVPVNALKLSI.- Scan = 198 CS = 2 Par m/z = 944.15 gi|172035756 Xcorr = 4.34 delcn = 0.37
b8-H2O
b16++-NH3
b8-NH3
b7-H2O
b13++-H2O
b17++-NH3
y4
y9++
y5
b6++-NH3
y15++
y8
b8
y9
b9
y10
b10-NH3
b10
b11-NH3
b12-NH3
b12
b13-H2O
b13
y13-NH3
y14-NH3
b14
y15-NH3
5500.0
5000.0
4500.0
4000.0
3500.0
3000.0
2500.0
2000.0
1500.0
1000.0
500.0
0.0
200.00
400.00
600.00
800.00
1000.00
1200.00
1400.00
1600.00
1800.00
2000.00

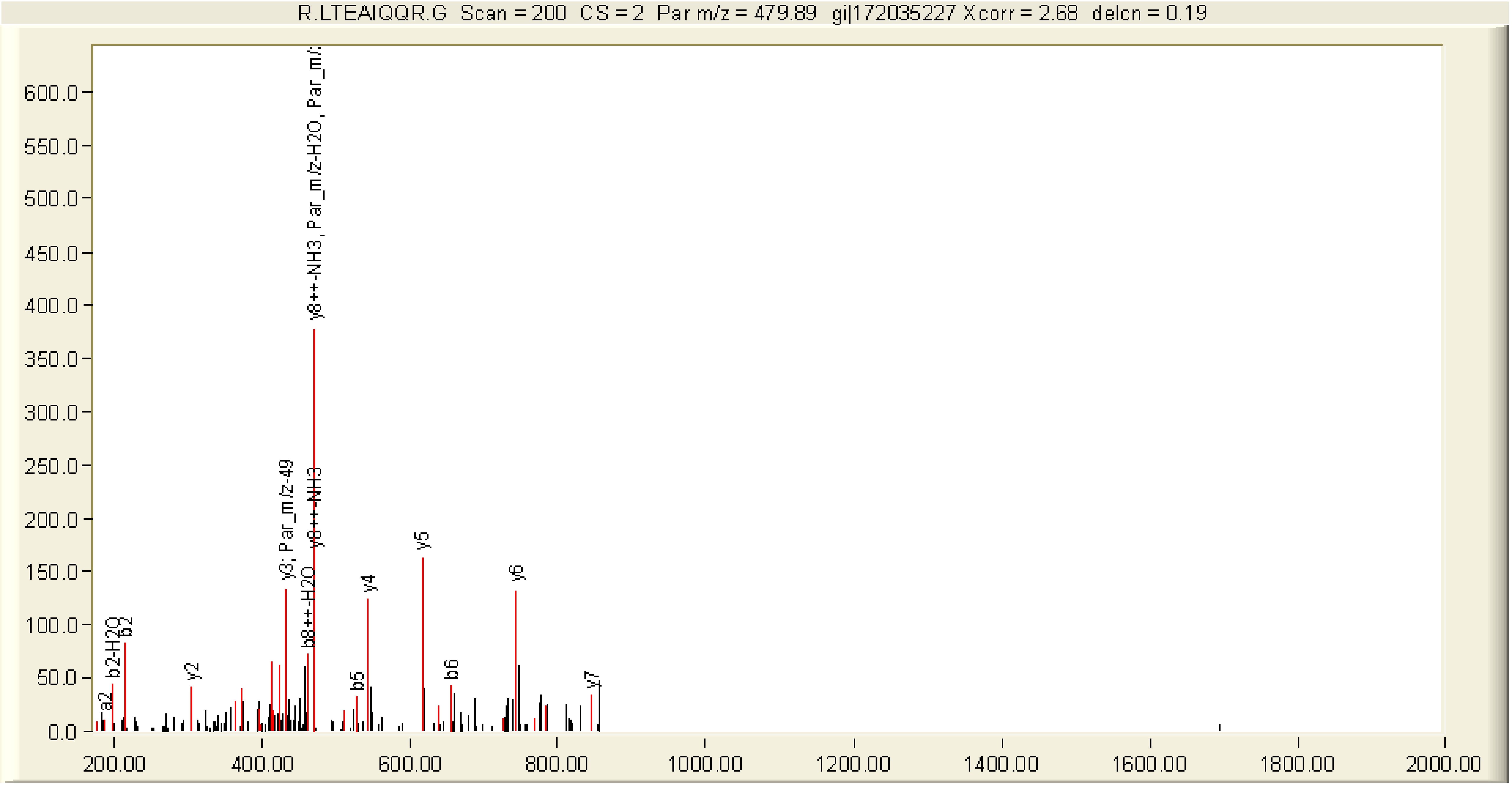

R.LTEAIQQR.G Scan = 200 CS = 2 Par m/z = 479.89 gi|172035227 Xcorr = 2.68 delcn = 0.19
y8++-NH3, Par_m/z-NH3, Par_m/z-H2O, Par_m/
y3; Par_m/z-49
y8++-NH3
b8++-H2O
y5
y4
y6
b2-H2O
b2
a2
y2
b5
b6
y7
600.0
550.0
500.0
450.0
400.0
350.0
300.0
250.0
200.0
150.0
100.0
50.0
0.0
200.00
400.00
600.00
800.00
1000.00
1200.00
1400.00
1600.00
1800.00
2000.00

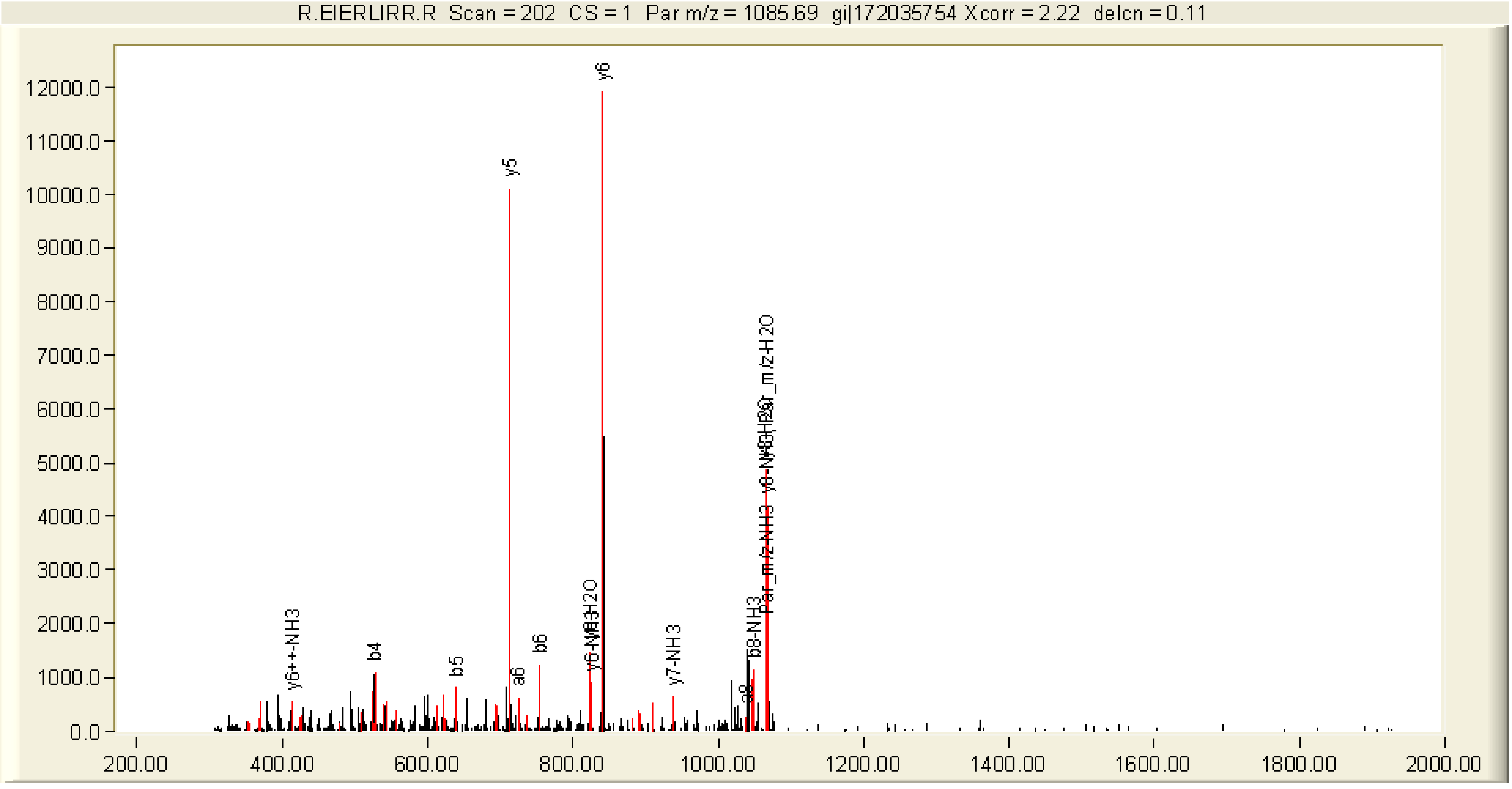
R.EIERLIRR.R Scan = 202 CS = 1 Par m/z = 1085.69 gi|172035754 Xcorr = 2.22 delcn = 0.11
y6
y5
y6++-NH3
b4
b5
a6
b6
y7-NH3
b8-NH3
a8
Par_m/z-NH3
Par_m/z-H2O
12000.0
11000.0
10000.0
9000.0
8000.0
7000.0
6000.0
5000.0
4000.0
3000.0
2000.0
1000.0
0.0
200.00
400.00
600.00
800.00
1000.00
1200.00
1400.00
1600.00
1800.00
2000.00

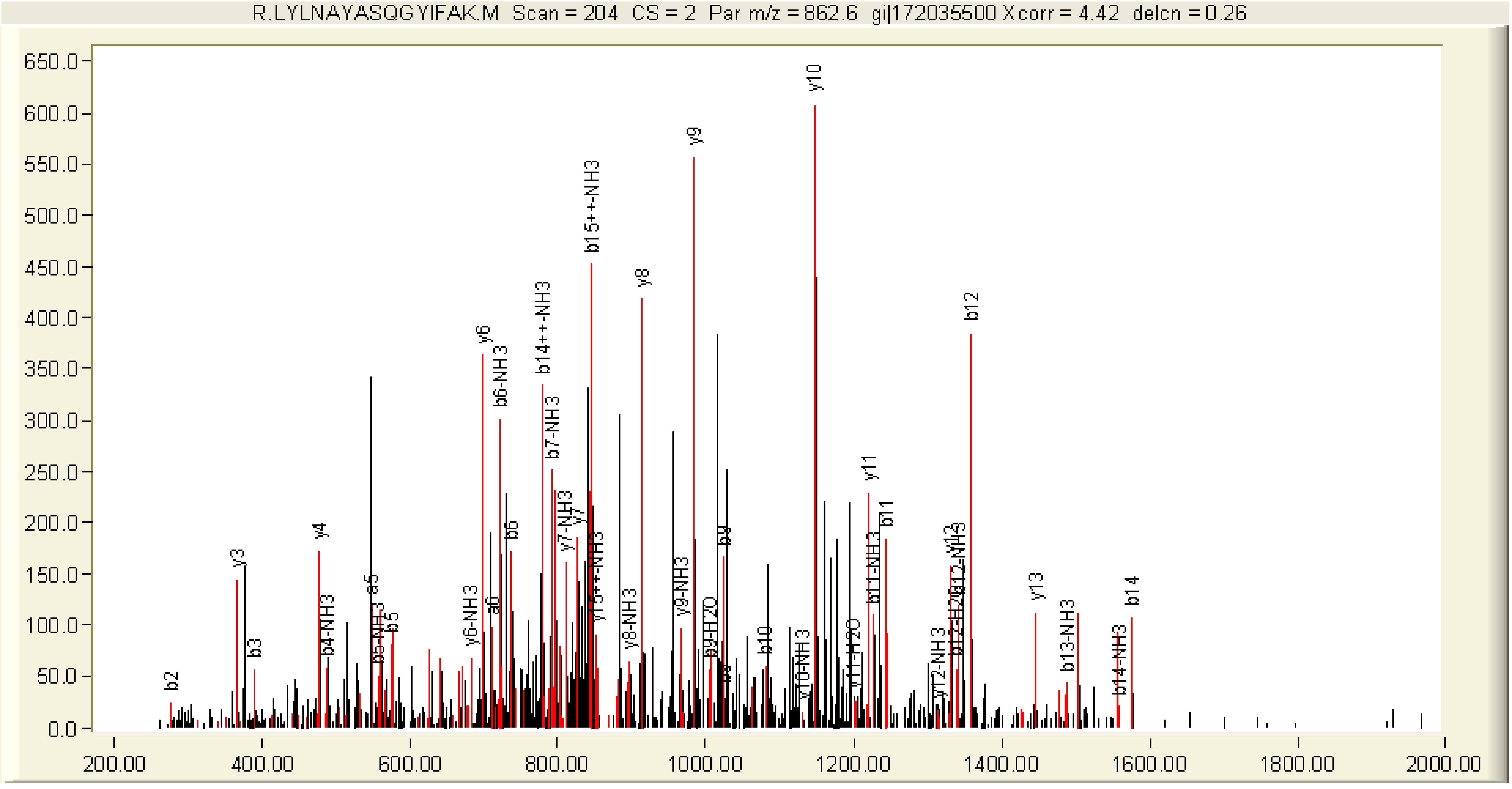

R.LYLNAYASQGYIFAK.M Scan = 204 CS = 2 Par m/z = 862.6 gi|172035500 Xcorr = 4.42 delcn = 0.26
650.0
600.0
550.0
500.0
450.0
400.0
350.0
300.0
250.0
200.0
150.0
100.0
50.0
0.0
200.00
400.00
600.00
800.00
1000.00
1200.00
1400.00
1600.00
1800.00
2000.00
y10
y9
b15++-NH3
y8
b12
y6
b6-NH3
b14++-NH3
b7-NH3
y11
b11
y7-NH3
y7
b6
y4
y3
b3
b2
b4-NH3
a5
b5-NH3
b5
y6-NH3
a6
y15++-NH3
y8-NH3
y9-NH3
b9
b10
y10-NH3
y11-H2O
b11-NH3
y12-NH3
y13
b13-NH3
b14-NH3
b14

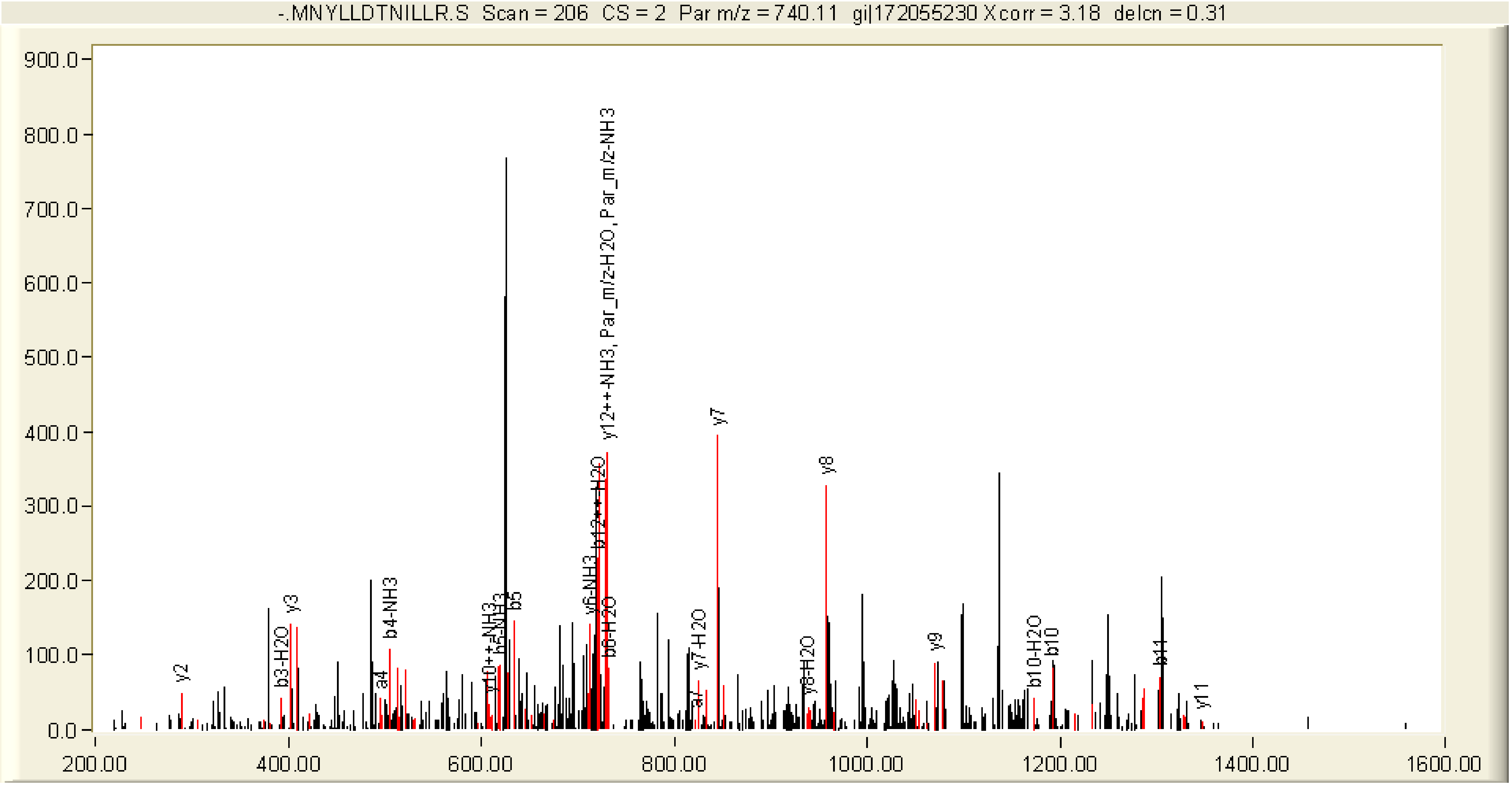
-.MNYLLDTNILLR.S Scan = 206 CS = 2 Par m/z = 740.11 gi|172055230 Xcorr = 3.18 delcn = 0.31
900.0
800.0
700.0
600.0
500.0
400.0
300.0
200.0
100.0
0.0
200.00
400.00
600.00
800.00
1000.00
1200.00
1400.00
1600.00
y2
b3-H2O
y3
a4
b4-NH3
b5
y6-NH3
b6-H2O
y12++-NH3, Par_m/z-H2O, Par_m/z-NH3
y7-H2O
y7
y8-H2O
y8
y9
b10-H2O
b10
b11
y11

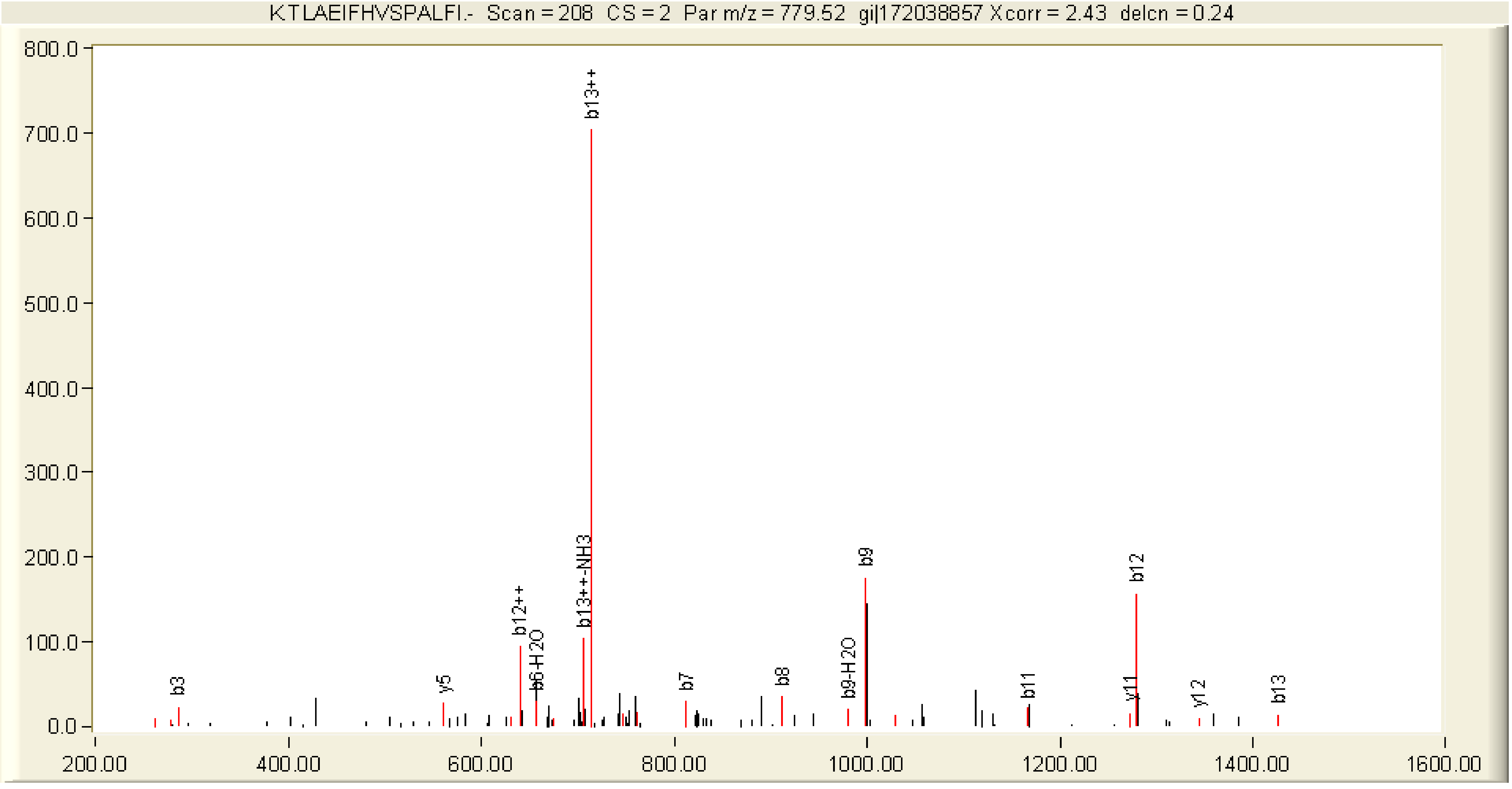

KTLAEIFHVSPALFI.- Scan = 208 CS = 2 Par m/z = 779.52 gi|172038857 Xcorr = 2.43 delcn = 0.24
800.0
700.0
600.0
500.0
400.0
300.0
200.0
100.0
0.0
200.00
400.00
600.00
800.00
1000.00
1200.00
1400.00
1600.00
b13++
b13++-NH3
b12++
b6-H2O
b3
y5
b7
b8
b9-H2O
b9
b11
y11
b12
y12
b13

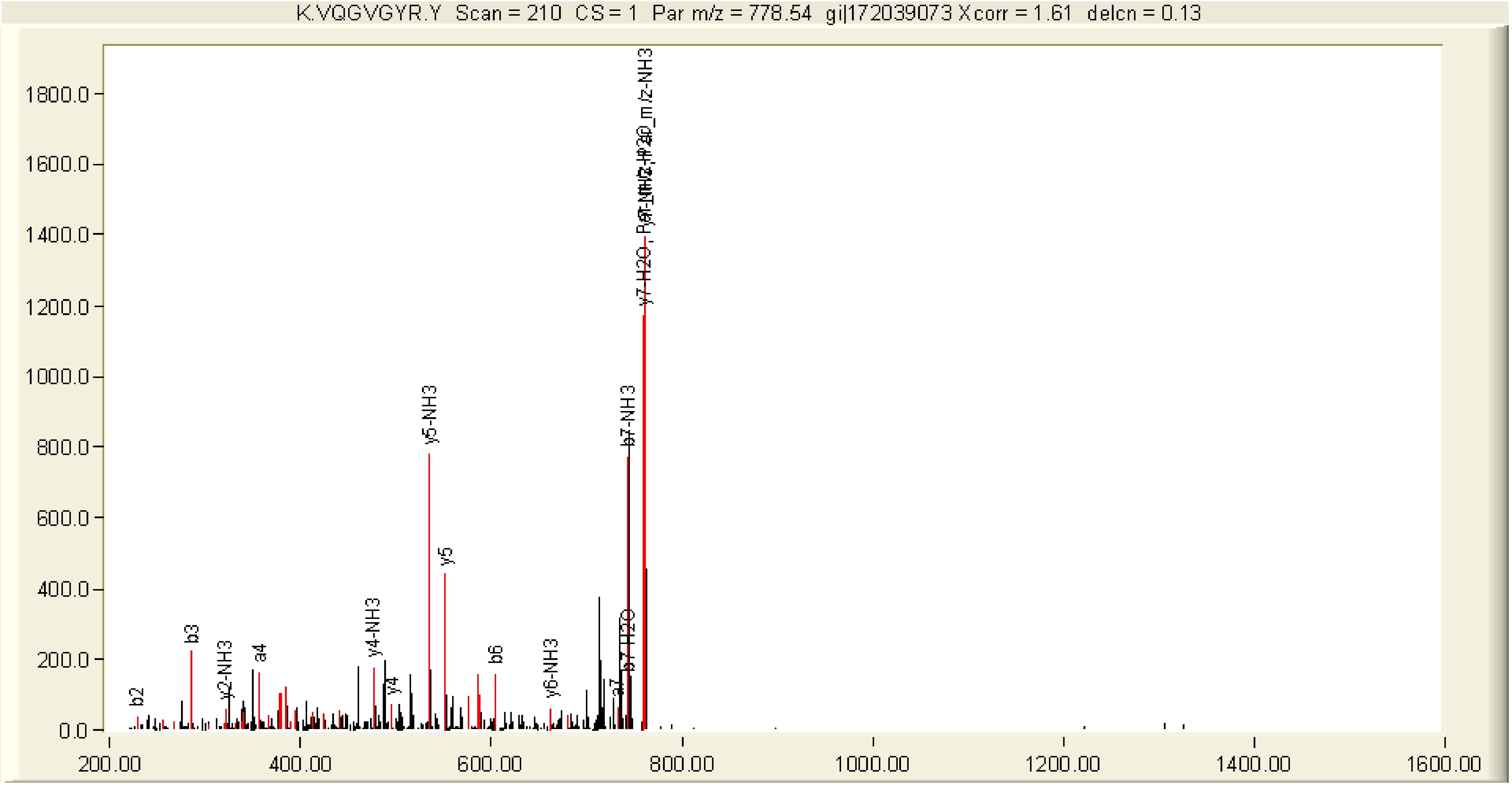

K.VQGVGYR.Y Scan = 210 CS = 1 Par m/z = 778.54 gi|172039073 Xcorr = 1.61 delcn = 0.13
1800.0
1600.0
1400.0
1200.0
1000.0
800.0
600.0
400.0
200.0
0.0
200.00
400.00
600.00
800.00
1000.00
1200.00
1400.00
1600.00
b2
b3
y2-NH3
a4
y4-NH3
y4
y5-NH3
y5
b6
y6-NH3
a7
b7-H2O
b7-NH3
y7-H2O

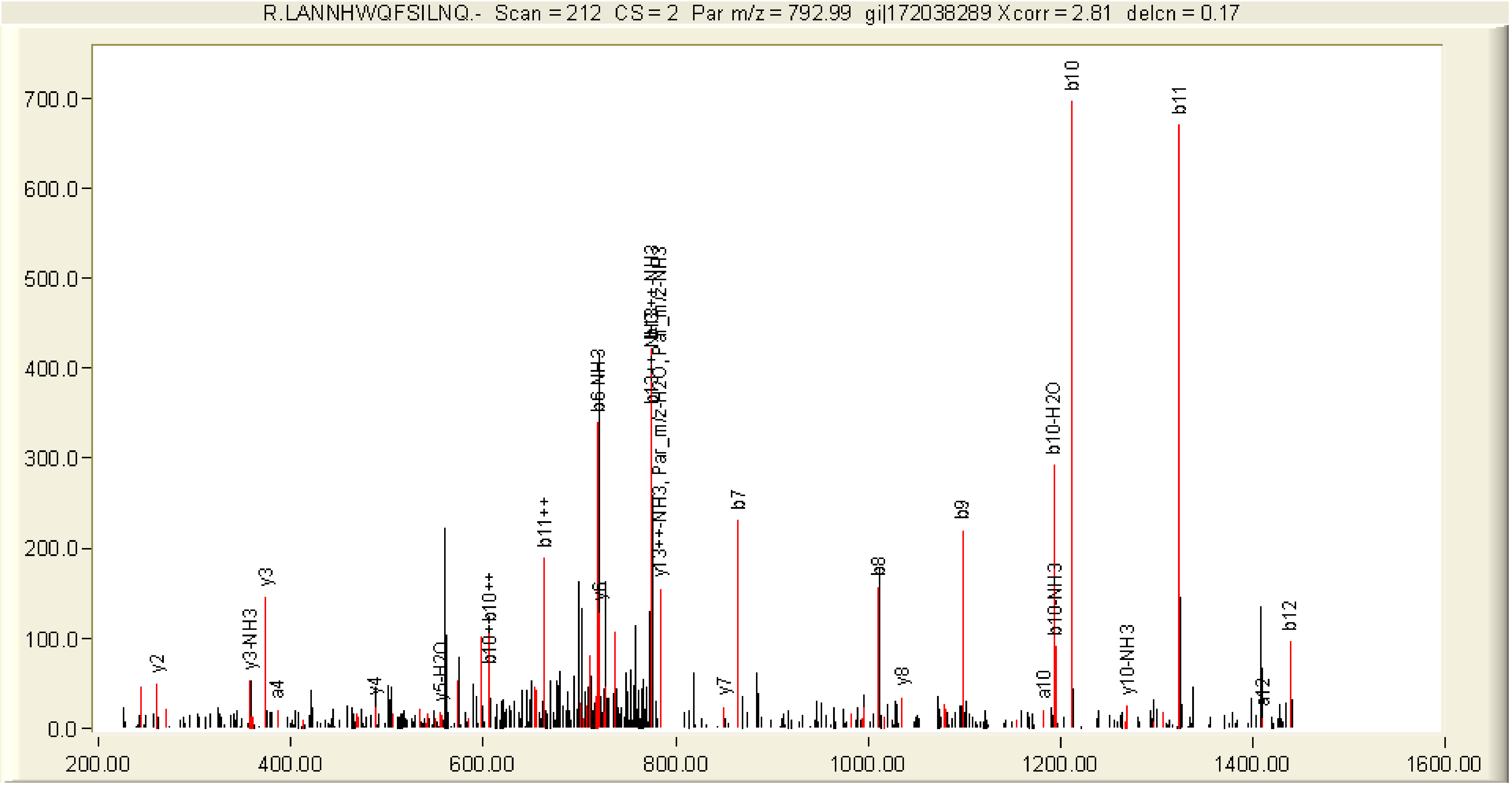

R.LANNHWQFSILNQ.- Scan = 212 CS = 2 Par m/z = 792.99 gi|172038289 Xcorr = 2.81 delcn = 0.17
700.0
600.0
500.0
400.0
300.0
200.0
100.0
0.0
200.00
400.00
600.00
800.00
1000.00
1200.00
1400.00
1600.00
y2
y3-NH3
y3
a4
y4
y5-H2O
b11++
b6-NH3
y6
b7
y7
b8
y8
b9
a10
b10-NH3
b10-H2O
b10
y10-NH3
b11
a12
b12

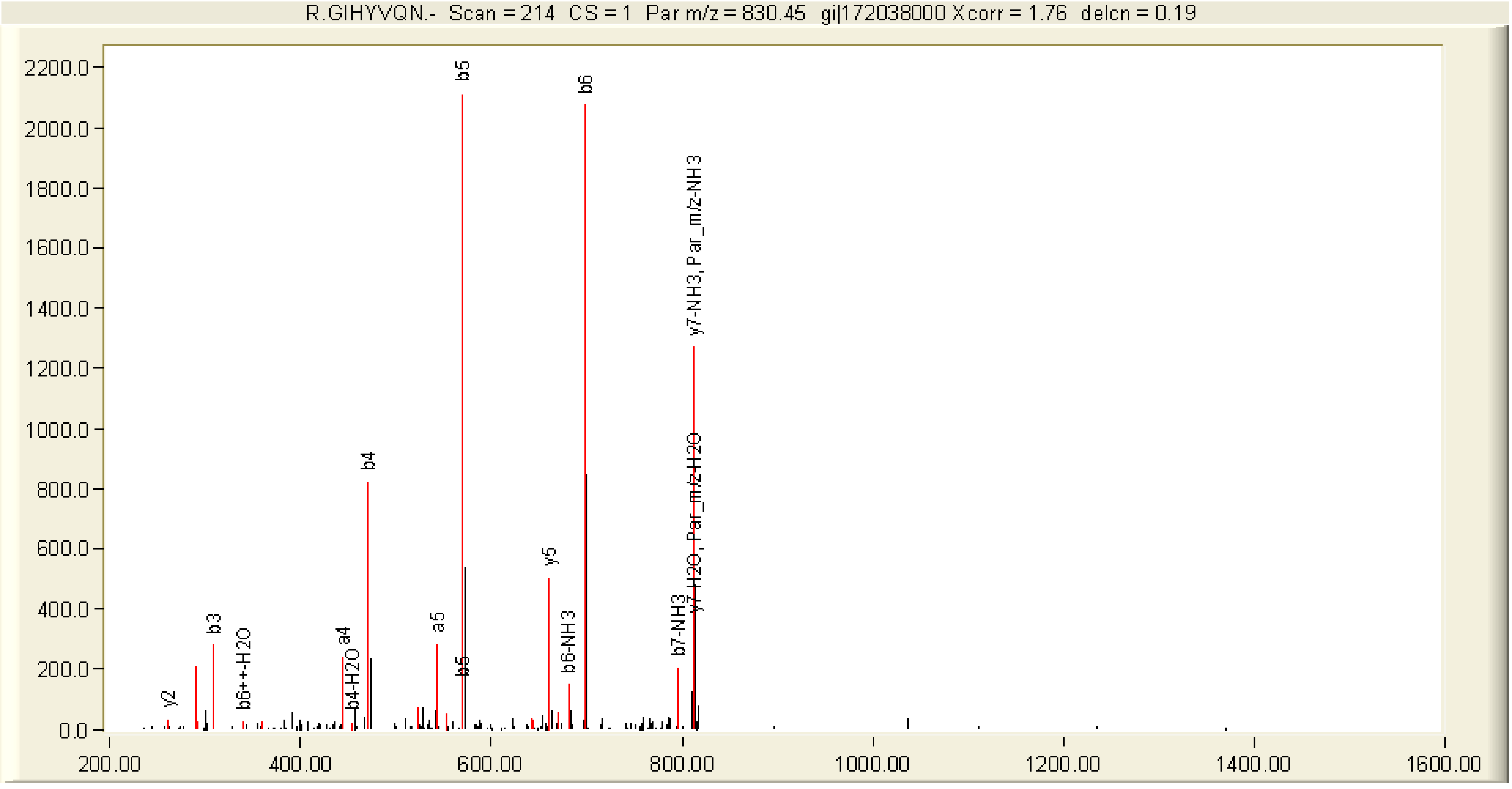
R.GIHYVQN.- Scan = 214 CS = 1 Par m/z = 830.45 gi|172038000 Xcorr = 1.76 delcn = 0.19
b5
b6
y7-NH3, Par_m/z-NH3
b4
y7-H2O, Par_m/z-H2O
y5
a5
b6-NH3
b7-NH3
b3
b6++-H2O
a4
b4-H2O
b5
y2
2200.0
2000.0
1800.0
1600.0
1400.0
1200.0
1000.0
800.0
600.0
400.0
200.0
0.0
200.00
400.00
600.00
800.00
1000.00
1200.00
1400.00
1600.00

R.EAIESFKEDLPTILDSLR.E Scan = 216 CS = 2 Par m/z = 1040.11 gi|172036013 Xcorr = 2.4 delcn = 0.11

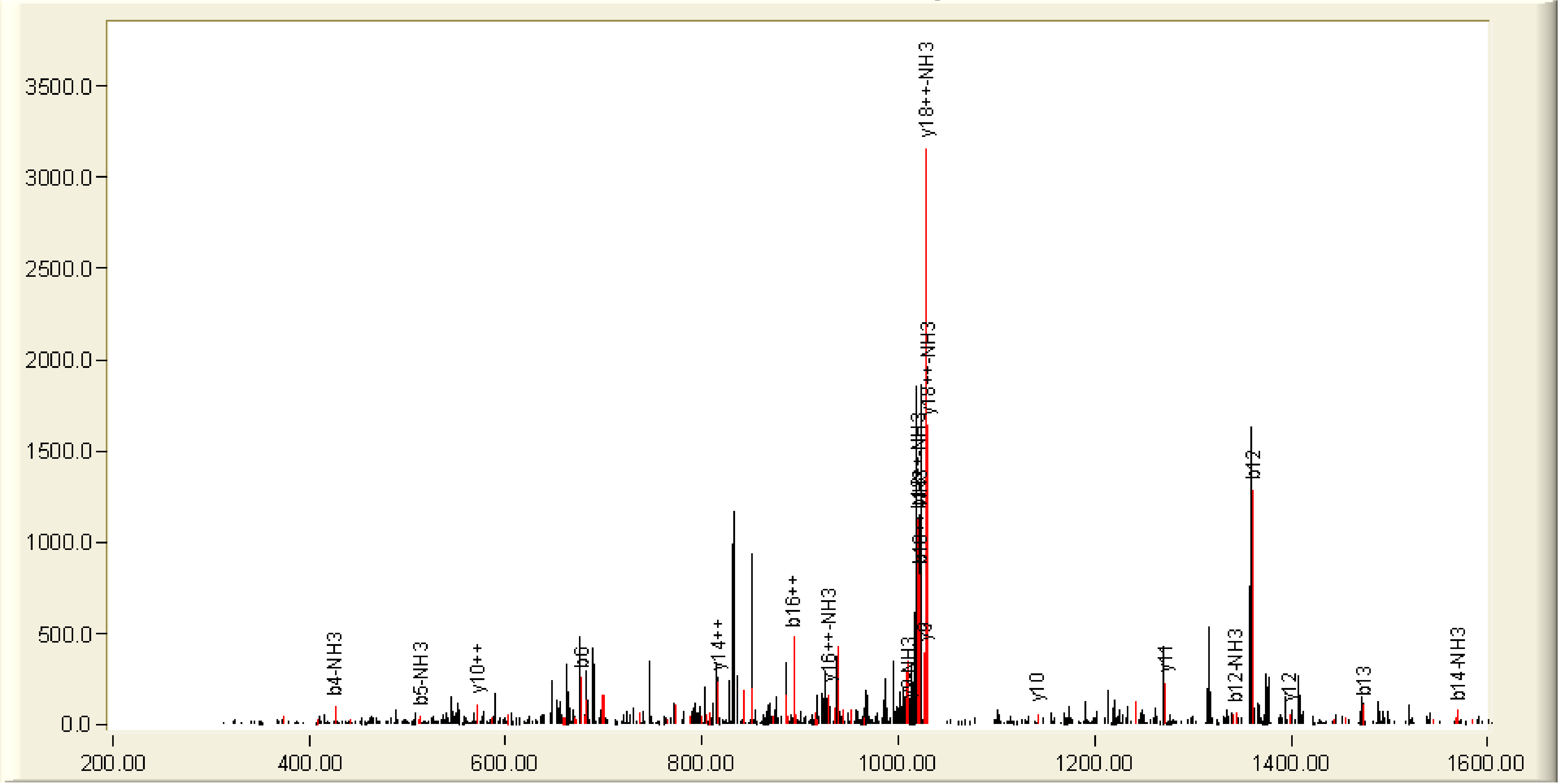

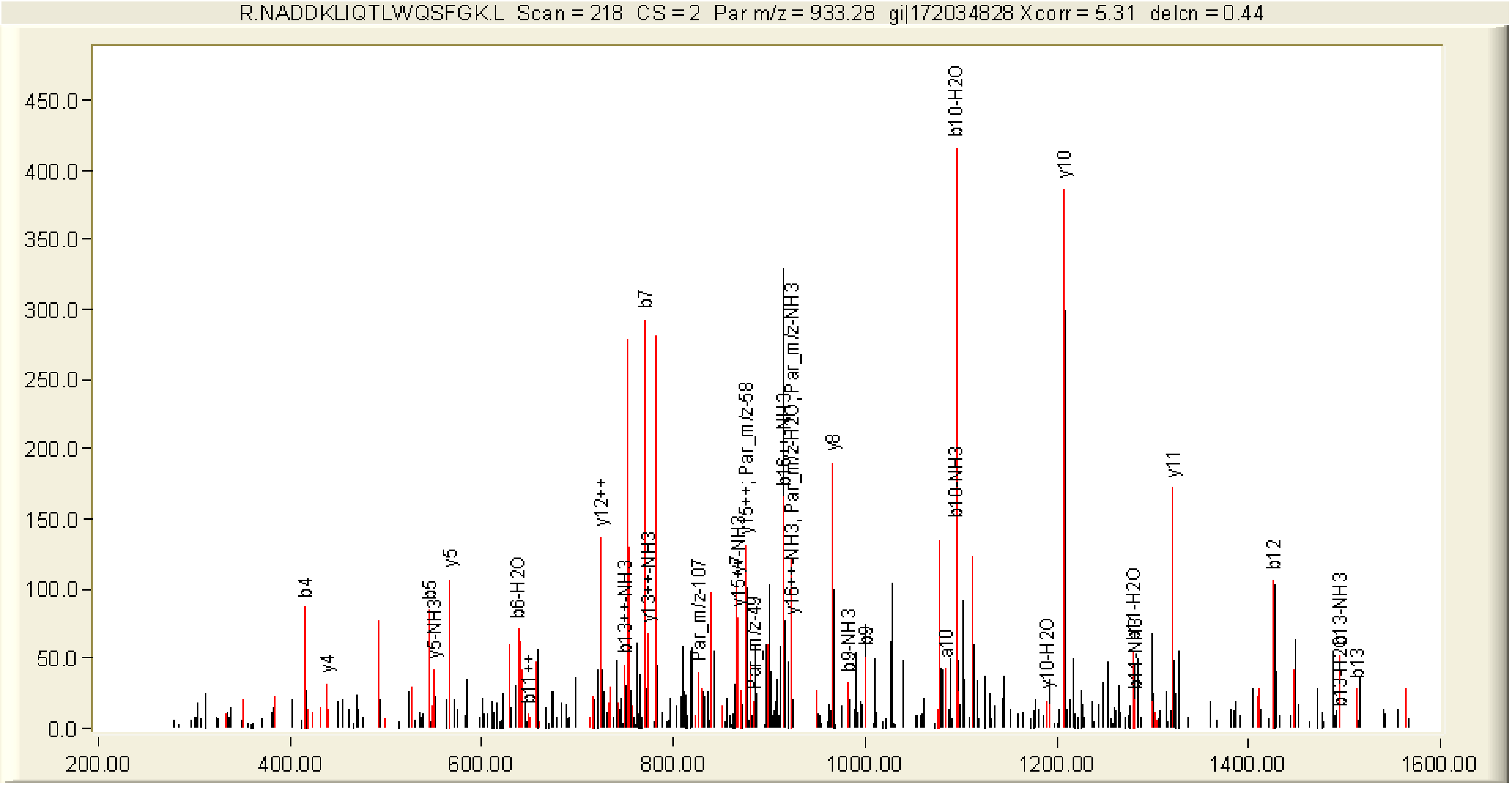
R.NADDKLIQTLWQSFGK.L Scan = 218 CS = 2 Par m/z = 933.28 gi|172034828 Xcorr = 5.31 delcn = 0.44
450.0
400.0
350.0
300.0
250.0
200.0
150.0
100.0
50.0
0.0
200.00
400.00
600.00
800.00
1000.00
1200.00
1400.00
1600.00
b4
y4
y5-NH3b5
y5
b6-H2O
b11++
y12++
b13++-NH3
b7
y13++-NH3
Par_m/z-107
Par_m/z-49
y8
b9-NH3
b9
a10
b10-NH3
b10-H2O
y10
y10-H2O
y11
b12
b13-NH3
b13

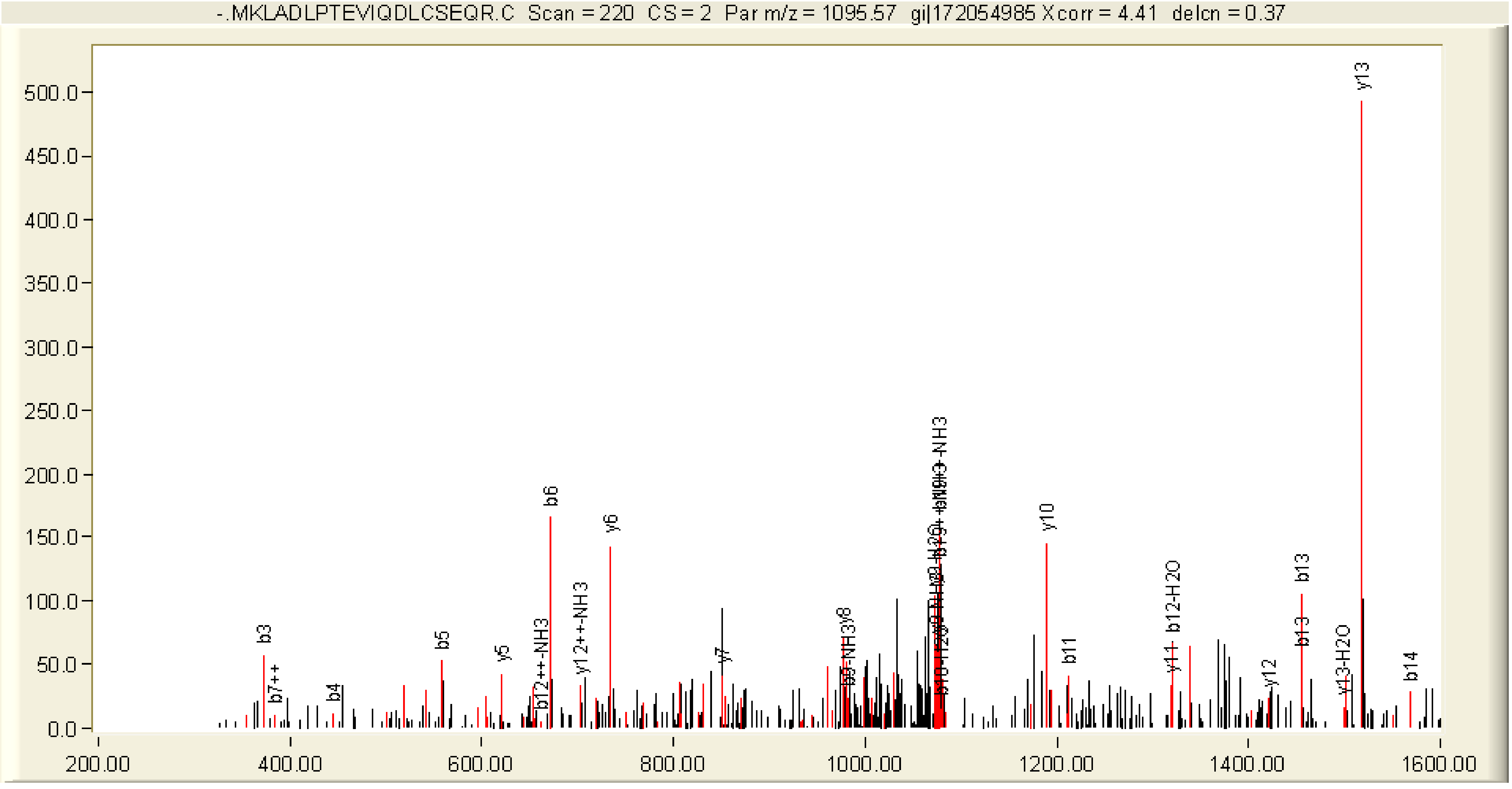

-.MKLADLPTEVIQDLCSEQR.C Scan = 220 CS = 2 Par m/z = 1095.57 gi|172054985 Xcorr = 4.41 delcn = 0.37
y13
b6
y6
y10
b13
b12-H2O
b3
b5
y5
b7++
b4
b12++-NH3
y12++-NH3
y7
b11
y11
y12
y13-H2O
b14
0.0
50.0
100.0
150.0
200.0
250.0
300.0
350.0
400.0
450.0
500.0
200.00
400.00
600.00
800.00
1000.00
1200.00
1400.00
1600.00

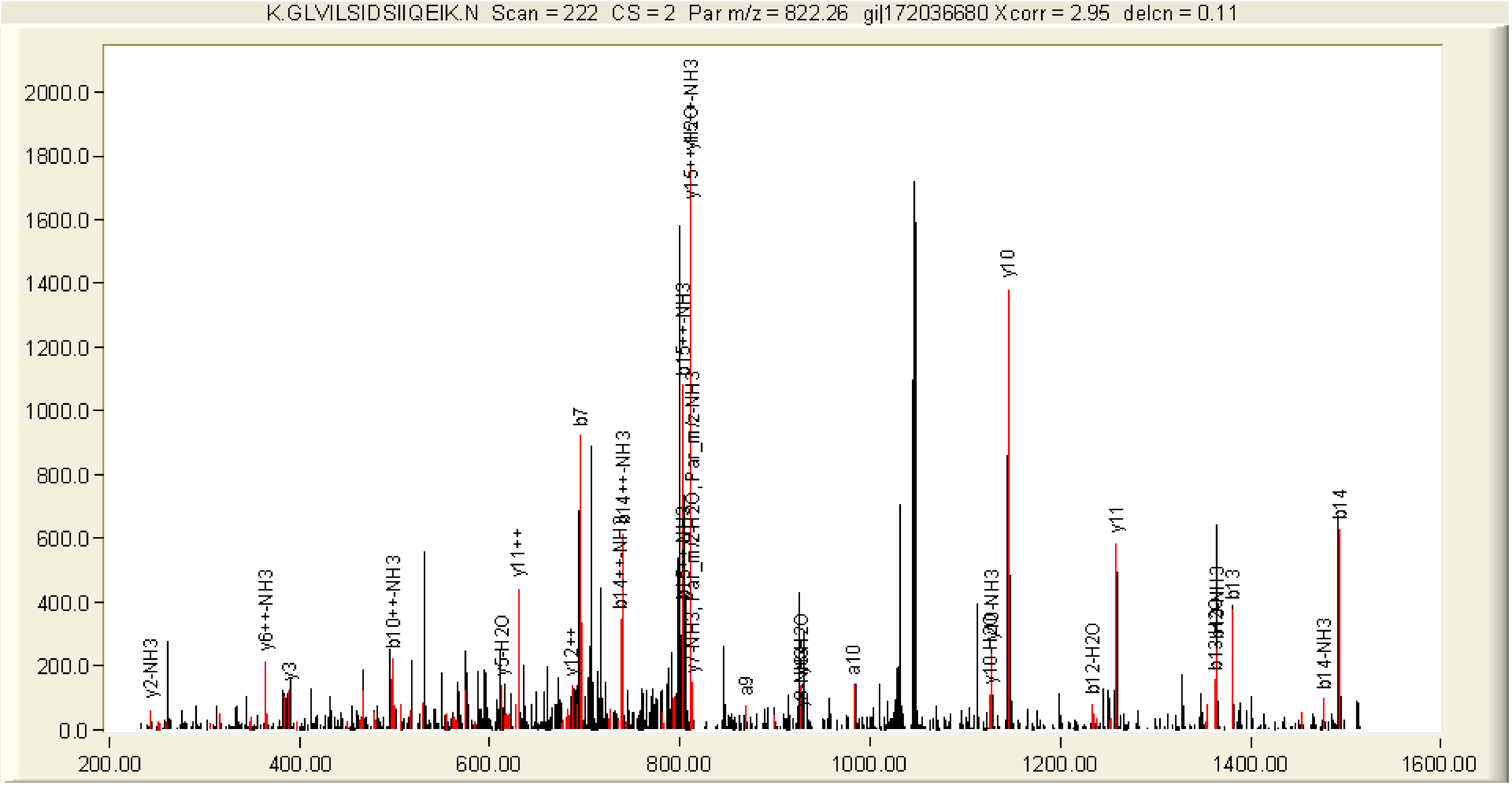

K.GLVILSIDSIIQEIK.N Scan = 222 CS = 2 Par m/z = 822.26 gi|172036680 Xcorr = 2.95 delcn = 0.11
2000.0
1800.0
1600.0
1400.0
1200.0
1000.0
800.0
600.0
400.0
200.0
0.0
200.00
400.00
600.00
800.00
1000.00
1200.00
1400.00
1600.00
y2-NH3
y6++-NH3
y3
b10++-NH3
y5-H2O
y11++
y12++
b7
b14++-NH3
b15++-NH3
a9
a10
y10-H2O
y10
b12-H2O
y11
b13
b14-NH3
b14

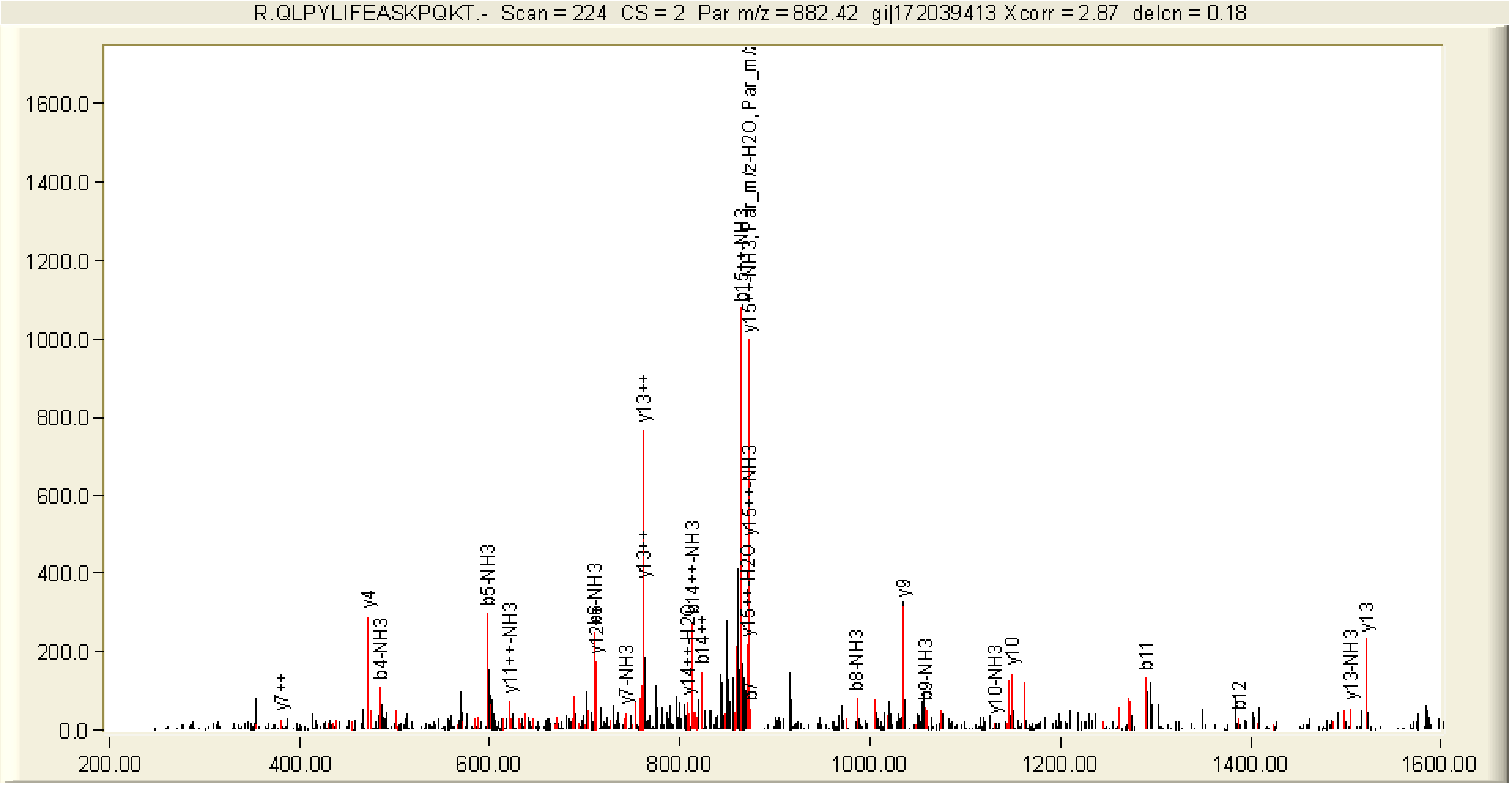

R.QLPYLIFEASKPQKT.- Scan = 224 CS = 2 Par m/z = 882.42 gi|172039413 Xcorr = 2.87 delcn = 0.18
1600.0
1400.0
1200.0
1000.0
800.0
600.0
400.0
200.0
0.0
200.00
400.00
600.00
800.00
1000.00
1200.00
1400.00
1600.00
y7++
y4
b4-NH3
b5-NH3
y11++-NH3
y7-NH3
y13++
y14++-NH3
b14++
y9
b8-NH3
b9-NH3
y10-NH3
y10
b11
b12
y13-NH3
y13
y15++-NH3
y15++-H2O
y15

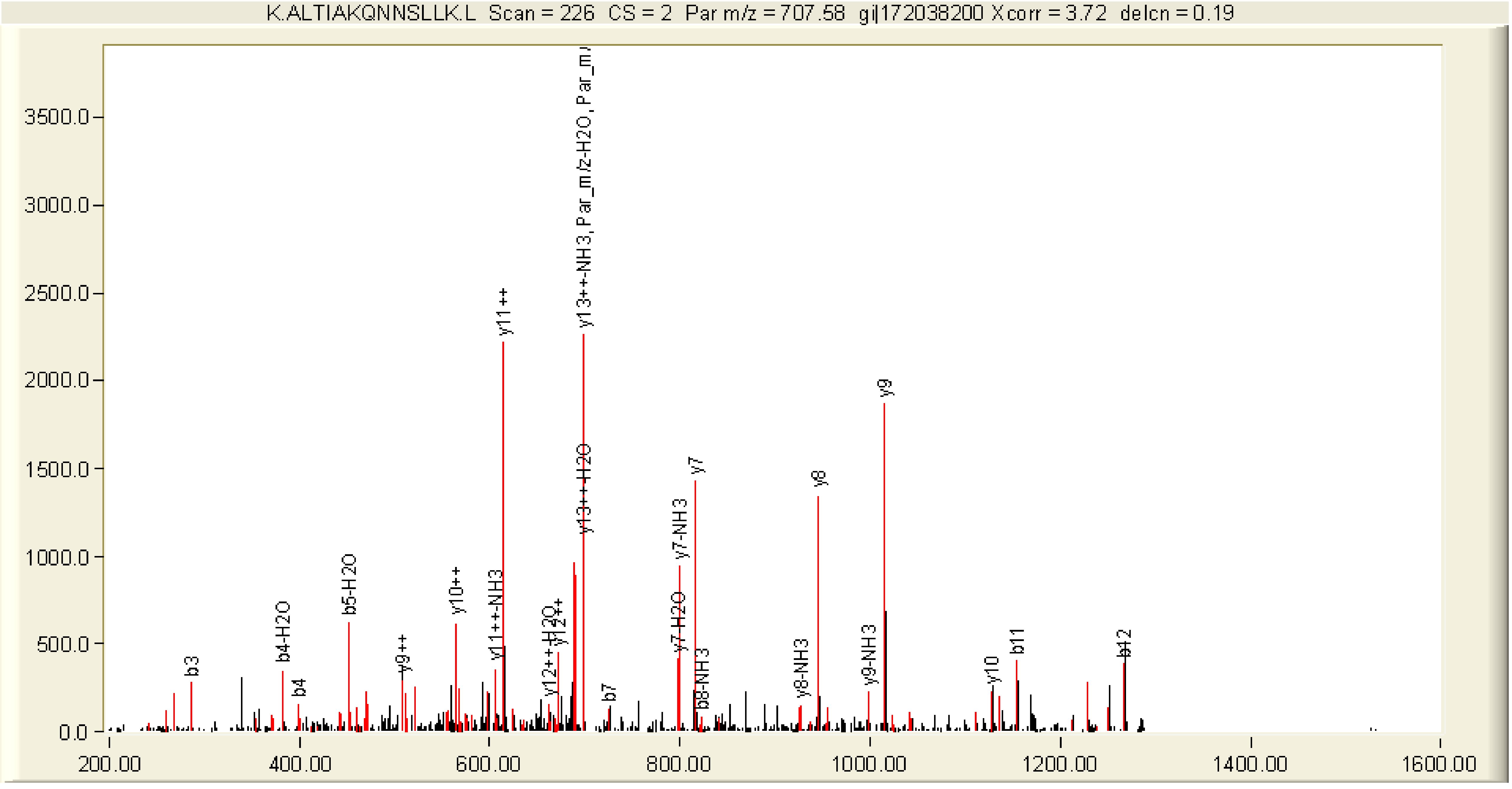

K.ALTIAKQNNSLLK.L Scan = 226 CS = 2 Par m/z = 707.58 gi|172038200 Xcorr = 3.72 delcn = 0.19
3500.0
3000.0
2500.0
2000.0
1500.0
1000.0
500.0
0.0
200.00
400.00
600.00
800.00
1000.00
1200.00
1400.00
1600.00
b3
b4-H2O
b4
b5-H2O
y9++
y10++
y11++-NH3
y11++
y12++-H2O
y12++
y13++-H2O
y13++-NH3, Par_m/z-H2O, Par_m/z
b7
y7-H2O
y7-NH3
y7
b8-NH3
y8-NH3
y8
y9-NH3
y9
y10
b11
b12

K.IEGCLDLDFIDLDVPLTFYSCYFSEGIR.L Scan = 228 CS = 3 Par m/z = 1081.72 gi|17203717 8 Xcorr = 3.47 delcn = 0.14

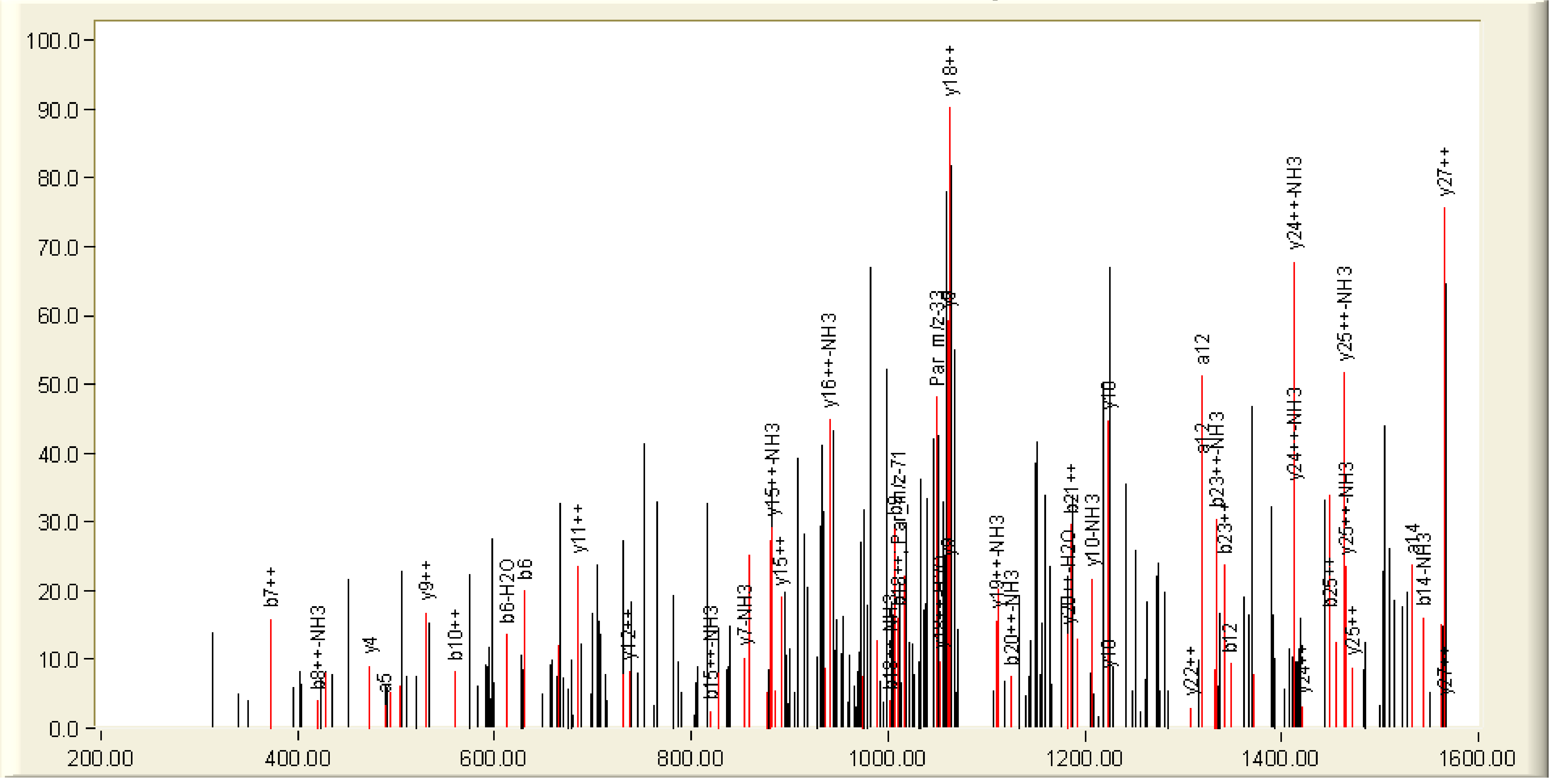

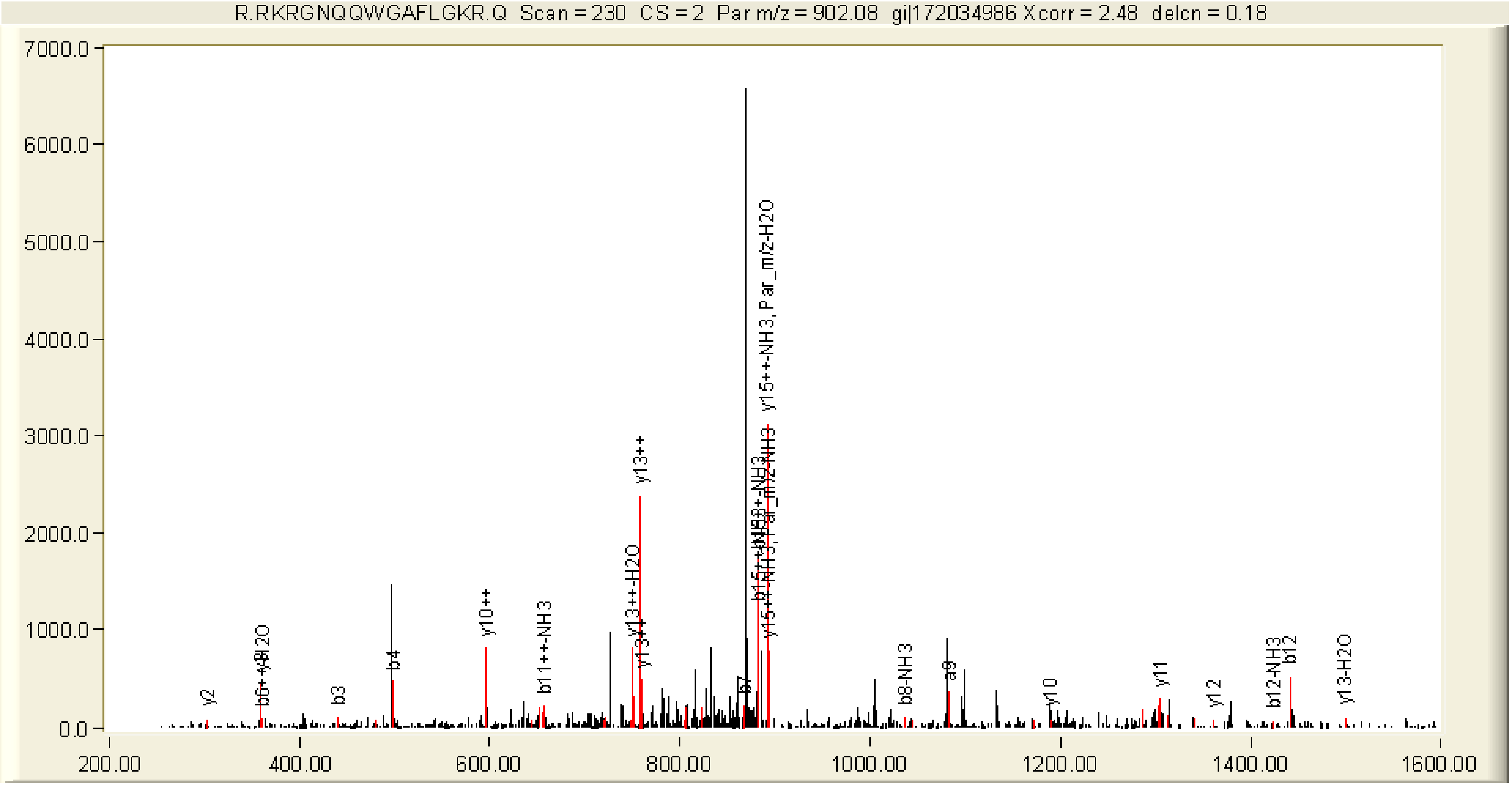

R.RKRGNQQWGAFLGKR.Q Scan = 230 CS = 2 Par m/z = 902.08 gi|172034986 Xcorr = 2.48 delcn = 0.18
7000.0
6000.0
5000.0
4000.0
3000.0
2000.0
1000.0
0.0
200.00
400.00
600.00
800.00
1000.00
1200.00
1400.00
1600.00
y2
b3
b4
y10++
b11++-NH3
y13++-H2O
y13++
b7
y15++-NH3, Par_m/z-H2O
b8-NH3
a9
y10
y11
y12
b12-NH3
b12
y13-H2O

R.YSIIDHSFSFPFHAR.F Scan = 232 CS = 2 Par m/z = 913.48 gi|172037123 Xcorr = 4.26 delcn = 0.52

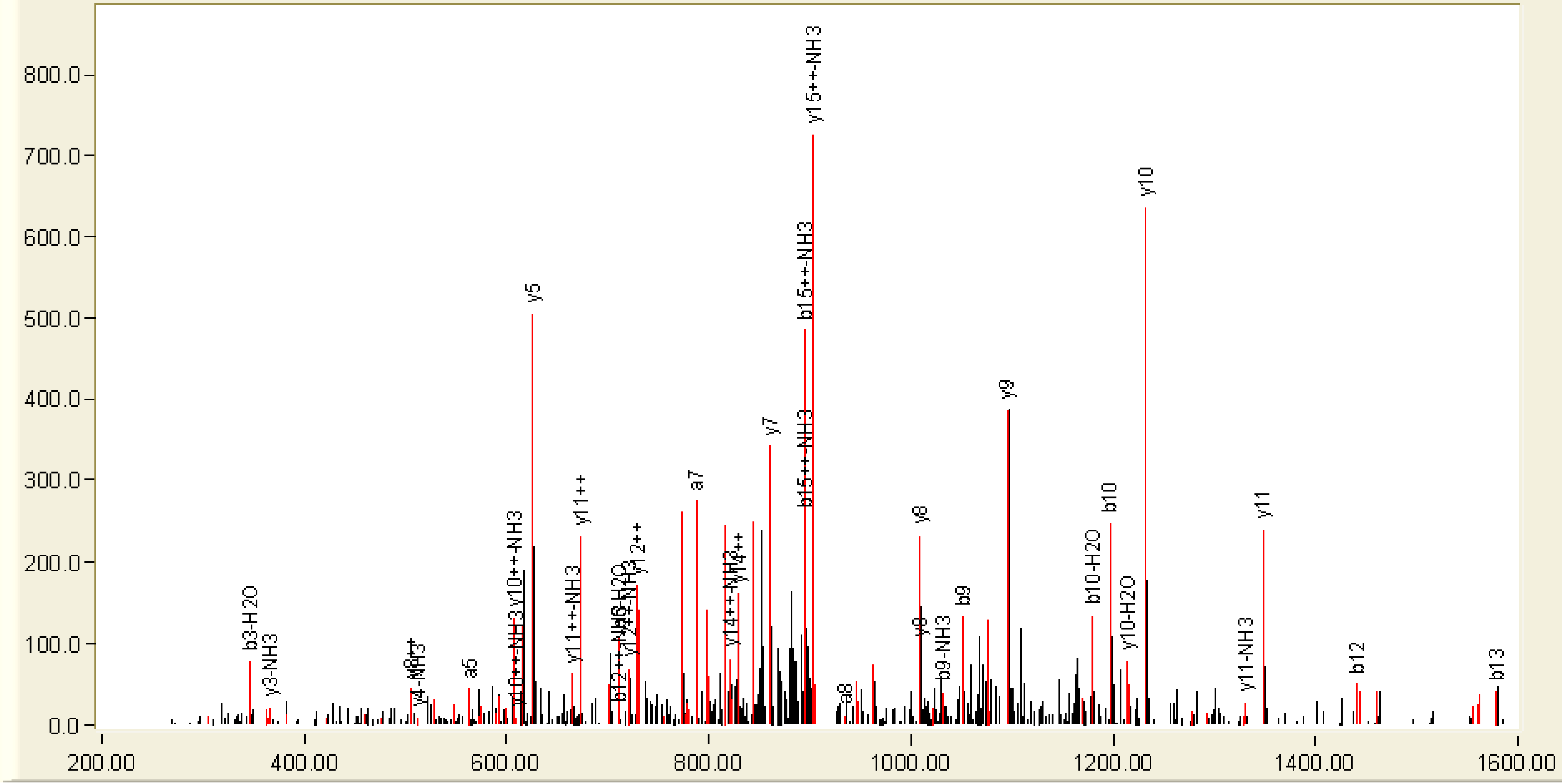

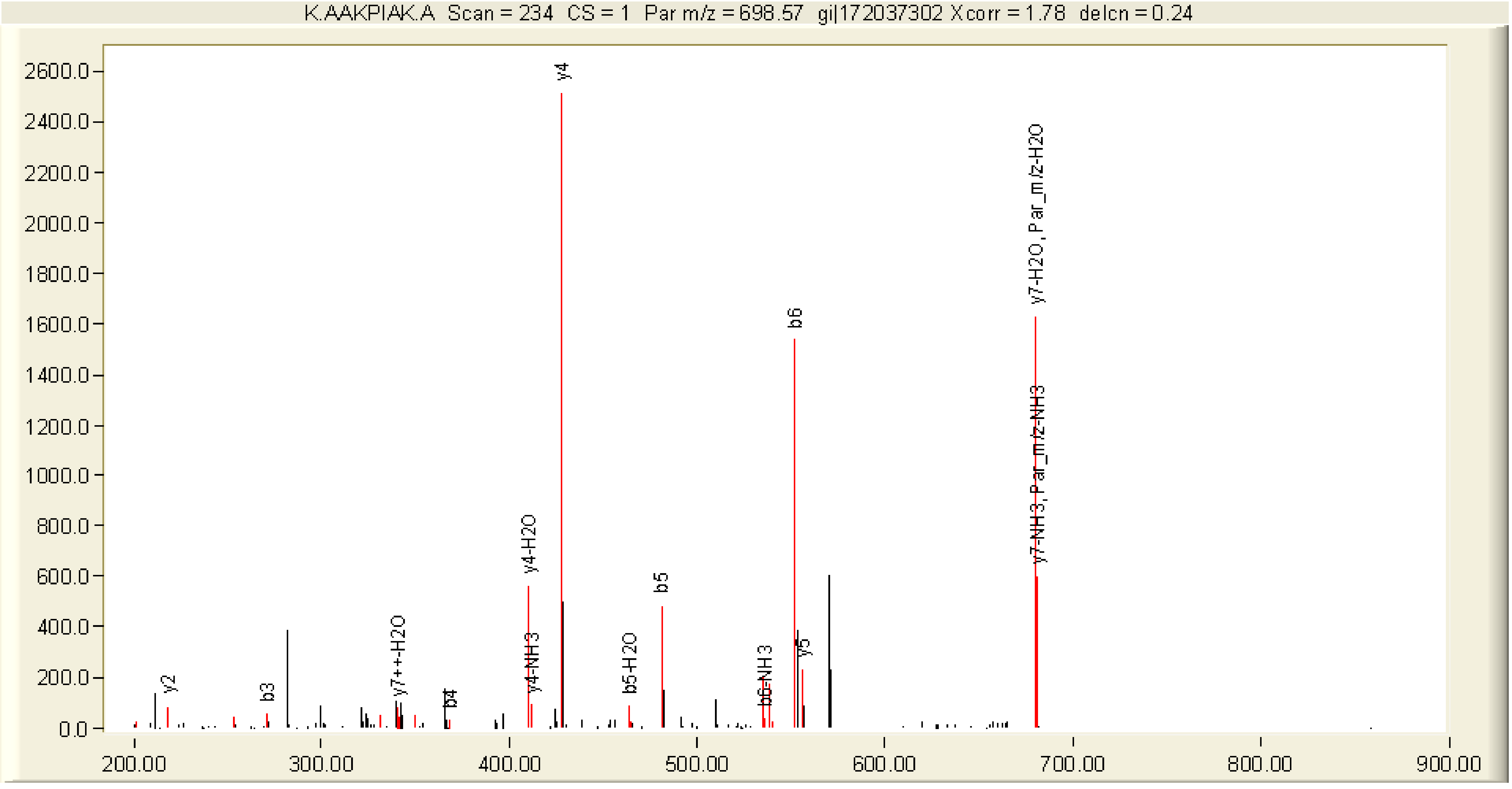

K.AAKPIAK.A Scan = 234 CS = 1 Par m/z = 698.57 gi|172037302 Xcorr = 1.78 delcn = 0.24
2600.0
2400.0
2200.0
2000.0
1800.0
1600.0
1400.0
1200.0
1000.0
800.0
600.0
400.0
200.0
0.0
200.00
300.00
400.00
500.00
600.00
700.00
800.00
900.00
y4
y7-H2O, Par_m/z-H2O
b6
y7-NH3, Par_m/z-NH3
y4-H2O
b5
y7++-H2O
y4-NH3
b5-H2O
b6-NH3
y5
y2
b3
b4

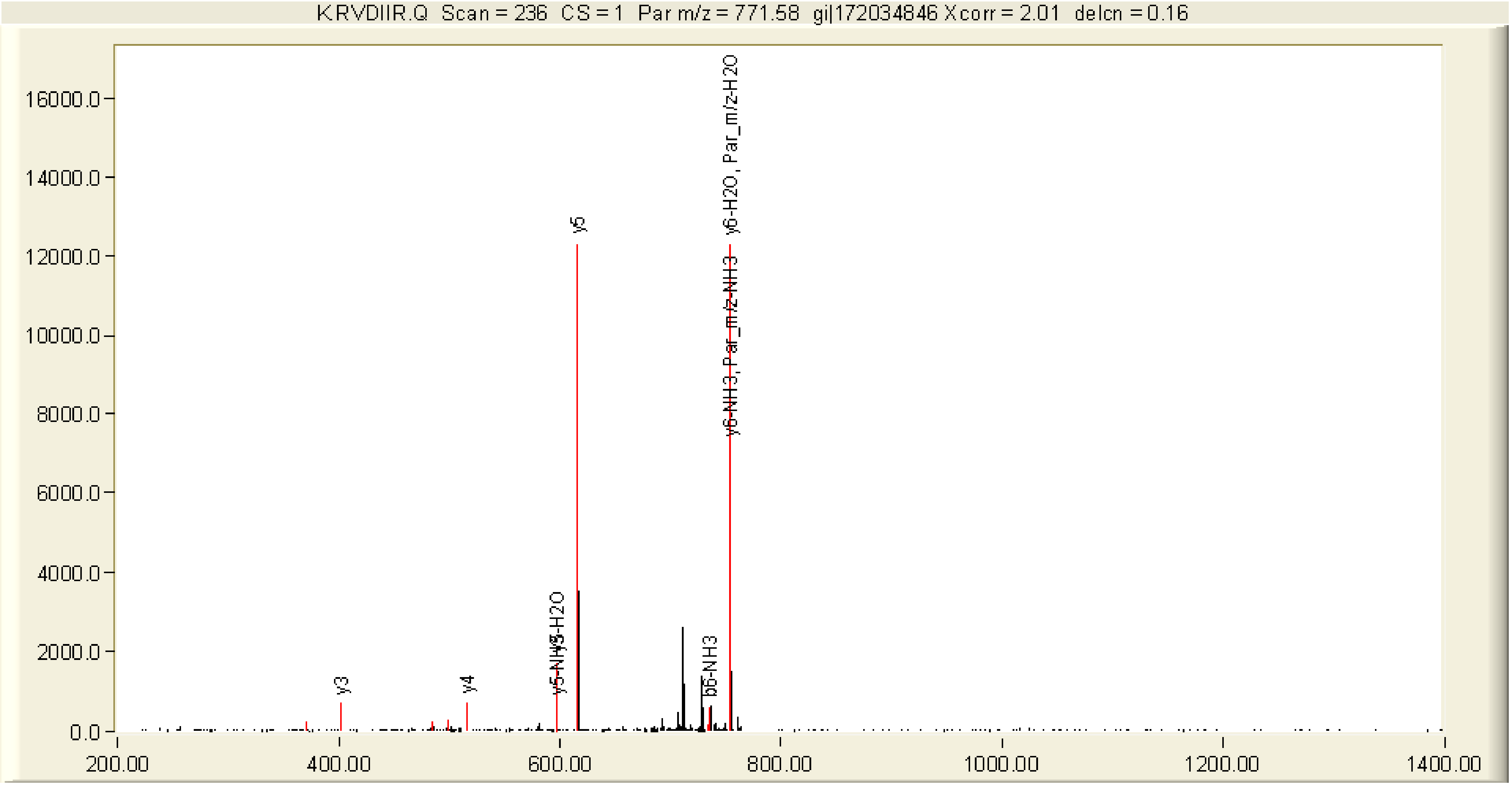

KRVDIIR.Q Scan = 236 CS = 1 Par m/z = 771.58 gi|172034846 Xcorr = 2.01 delcn = 0.16
y6-H2O, Par_m/z-H2O
y5
y6-NH3, Par_m/z-NH3
y3
y4
b6-NH3
16000.0
14000.0
12000.0
10000.0
8000.0
6000.0
4000.0
2000.0
0.0
200.00
400.00
600.00
800.00
1000.00
1200.00
1400.00

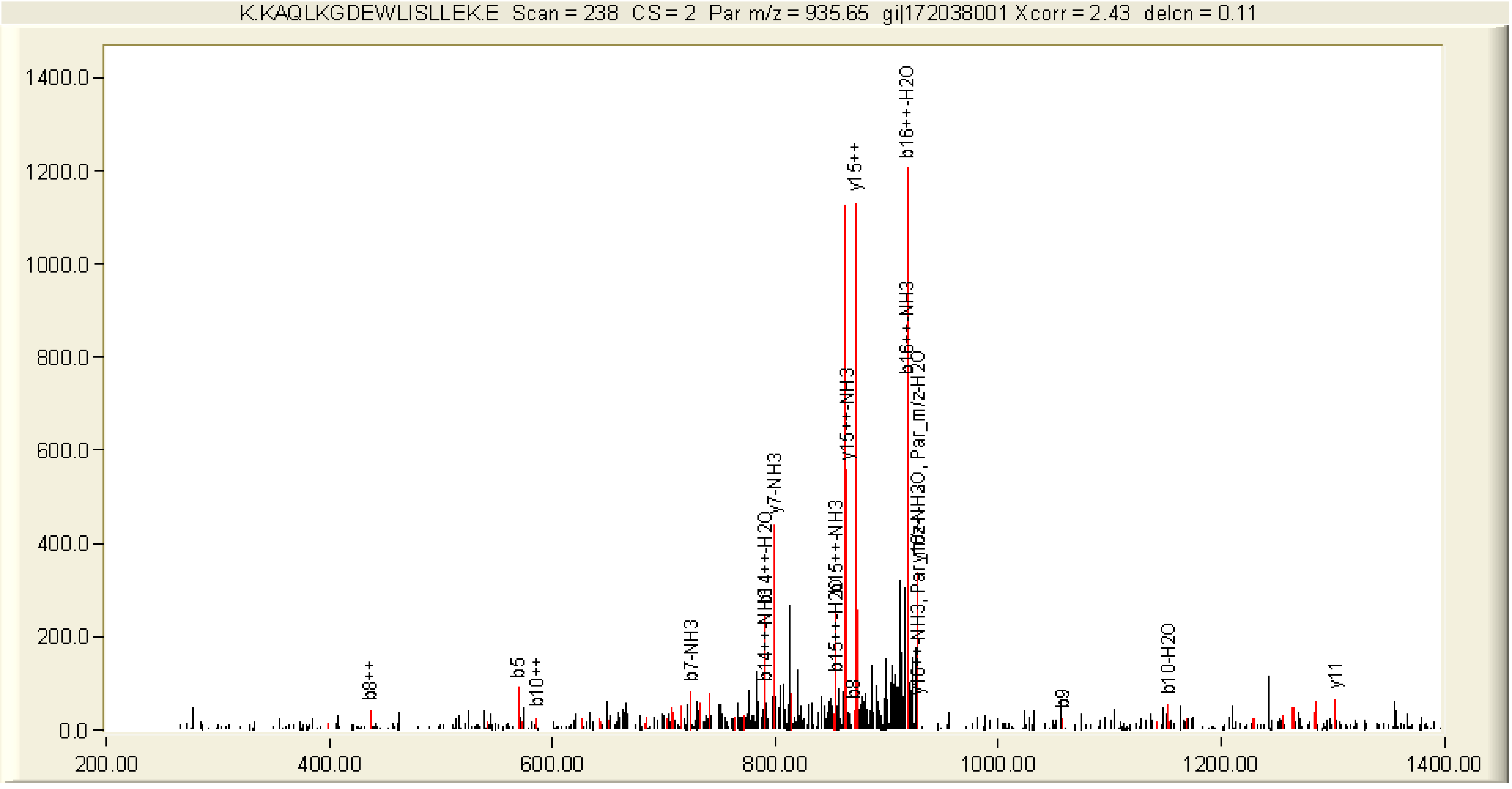

K.KAQLKGDEWLISLLEK.E Scan = 238 CS = 2 Par m/z = 935.65 gi|172038001 Xcorr = 2.43 delcn = 0.11
1400.0
1200.0
1000.0
800.0
600.0
400.0
200.0
0.0
200.00
400.00
600.00
800.00
1000.00
1200.00
1400.00
b8++
b5
b10++
b7-NH3
y7-NH3
b15++-NH3
y15++-NH3
y15++
b16++-H2O
b16++-NH3
b8
b9
b10-H2O
y11

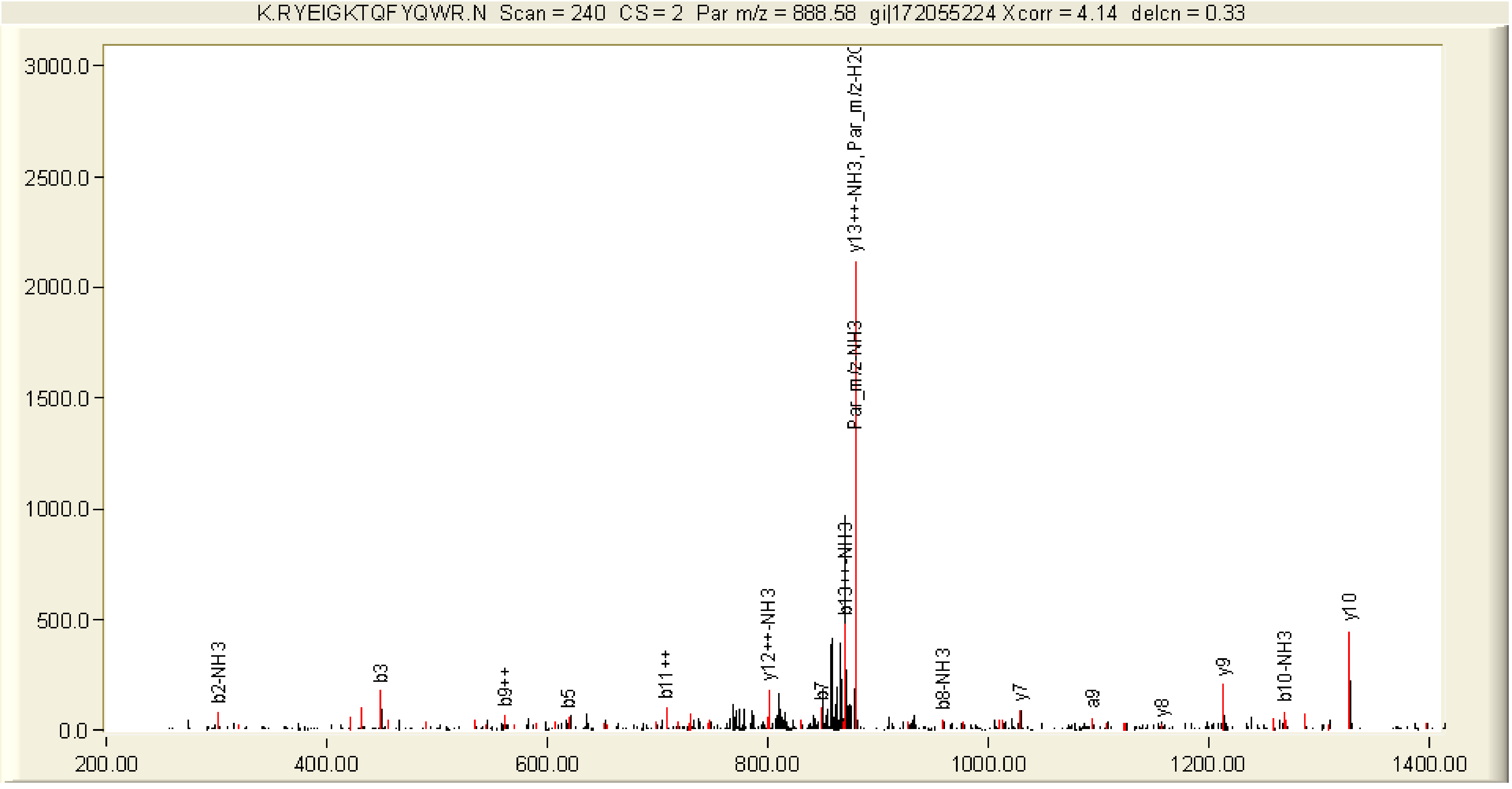

K.RYEIGKTQFYQWR.N Scan = 240 CS = 2 Par m/z = 888.58 gi|172055224 Xcorr = 4.14 delcn = 0.33
3000.0
2500.0
2000.0
1500.0
1000.0
500.0
0.0
200.00
400.00
600.00
800.00
1000.00
1200.00
1400.00
y13++-NH3, Par_m/z-H2O
Par_m/z-NH3
b13++-NH3
y12++-NH3
b2-NH3
b3
b9++
b5
b11++
b7
b8-NH3
y7
a9
y8
y9
b10-NH3
y10

K.KYPYPHSMSKEELDNIFR.S Scan = 242 CS = 2 Par m/z = 1127.89 gi|172035814 Xcorr = 2.5 delcn = 0.12

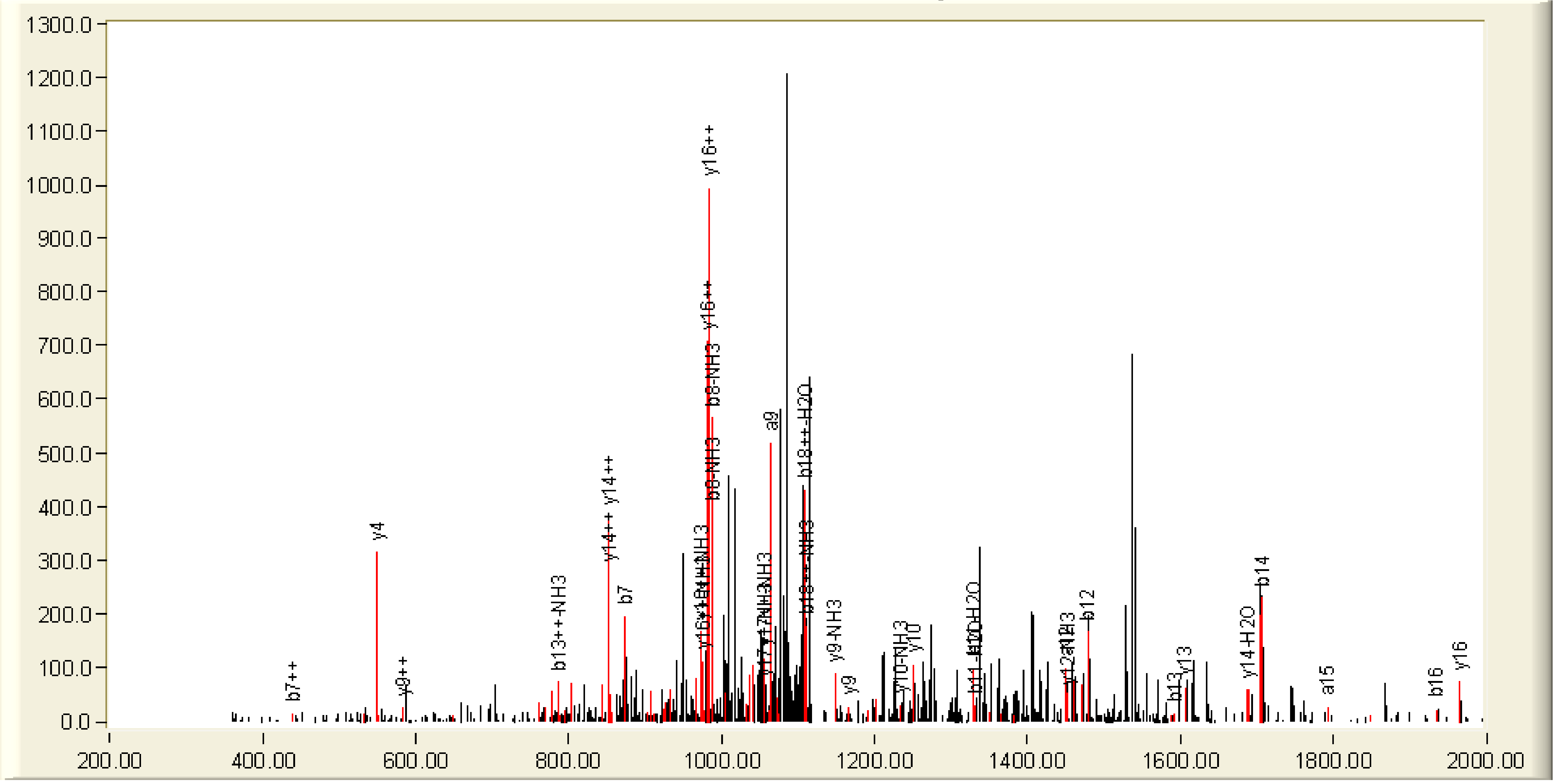

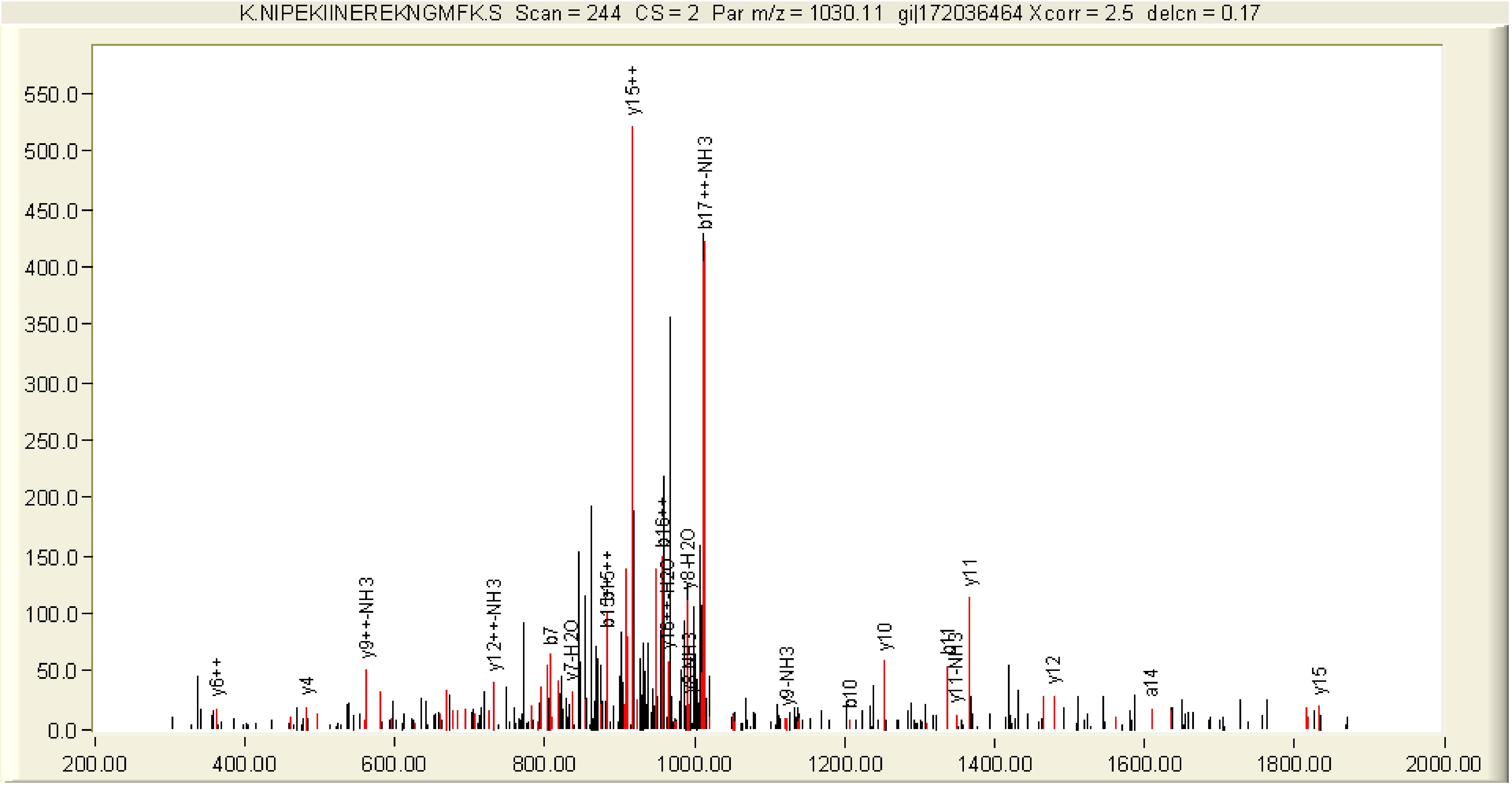
K.NIPEKIINEREKNGMFK.S Scan = 244 CS = 2 Par m/z = 1030.11 gi|172036464 Xcorr = 2.5 delcn = 0.17
y15++
b17++-NH3
y11
y10
y9++-NH3
y12++-NH3
b7
y6++
y4
y9-NH3
b10
y12
a14
y15
y8-H2O
0.0
50.0
100.0
150.0
200.0
250.0
300.0
350.0
400.0
450.0
500.0
550.0
200.00
400.00
600.00
800.00
1000.00
1200.00
1400.00
1600.00
1800.00
2000.00

R.QANVAIVEPNYGYYFHPDPEVEGDPTGQVLYQALSELNQEYGVK.E Scan = 246 CS = 3 Par m/z = 1648.68 gi|172039653 Xcorr = 5.19 delcn = 0.51

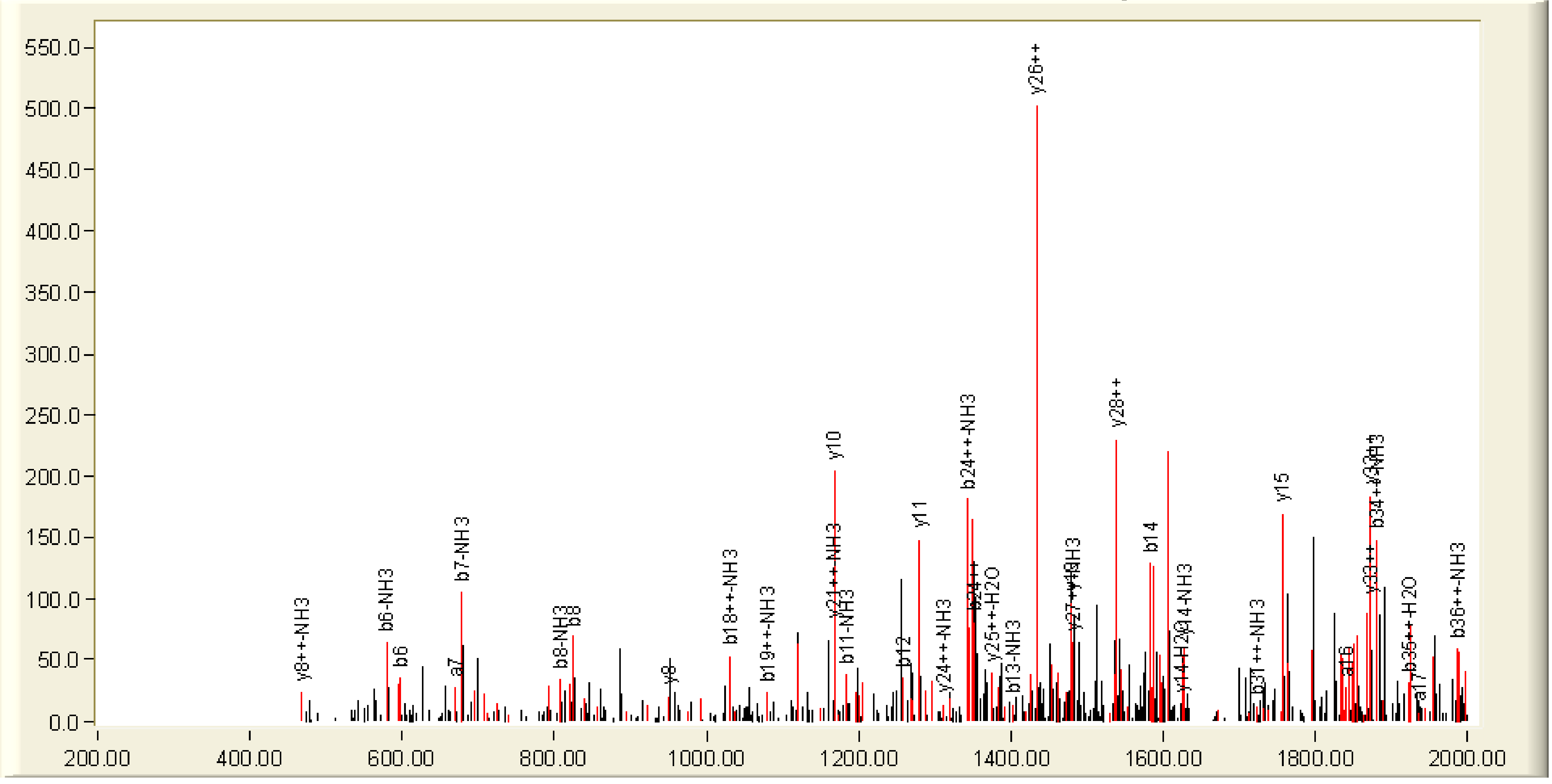

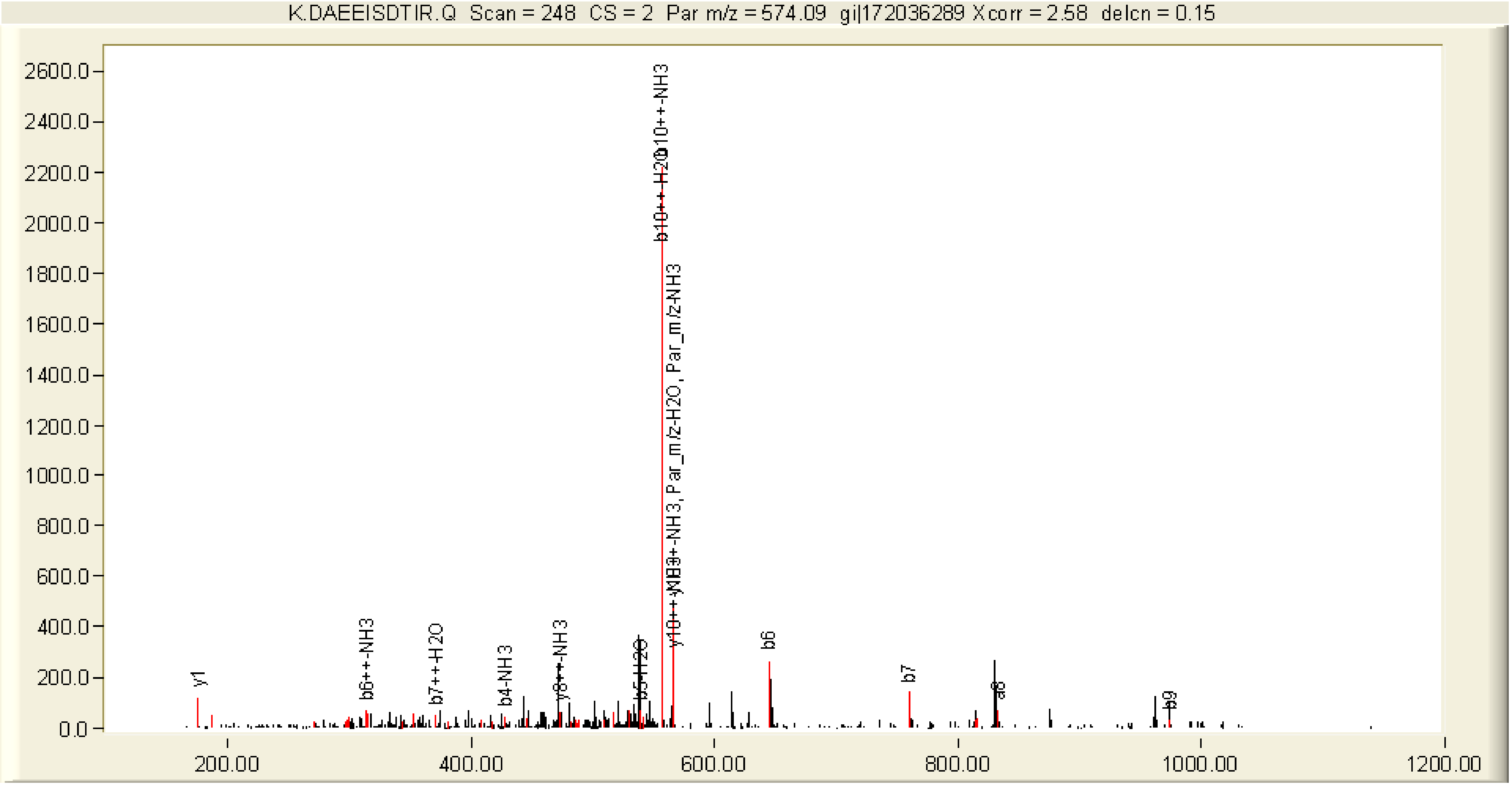
K.DAEEISDTIR.Q Scan = 248 CS = 2 Par m/z = 574.09 gi|172036289 Xcorr = 2.58 delcn = 0.15
y1
b6++-NH3
b7++-H2O
b4-NH3
y8++-NH3
b5-H2O
b10++-H2O
b10++-NH3
y10++-NH3, Par_m/z-NH3, Par_m/z-H2O, Par_m/z-NH3
b6
b7
a8
b9
2600.0
2400.0
2200.0
2000.0
1800.0
1600.0
1400.0
1200.0
1000.0
800.0
600.0
400.0
200.0
0.0
200.00
400.00
600.00
800.00
1000.00
1200.00

K.EENEATKMLDILNSPK.I Scan = 250 CS = 2 Par m/z = 918.02 gi|172037195 Xcorr = 3.07 delcn = 0.13

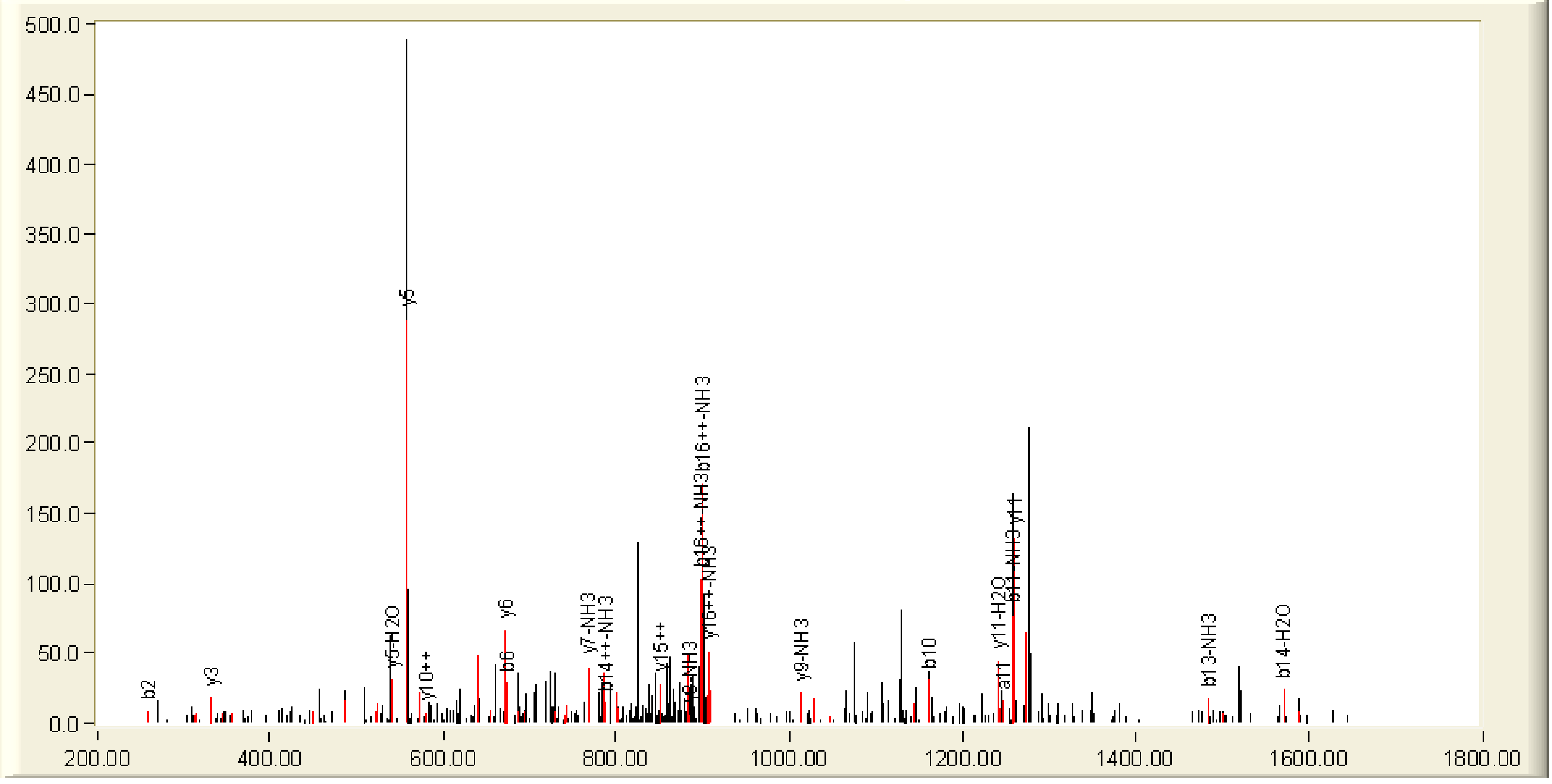

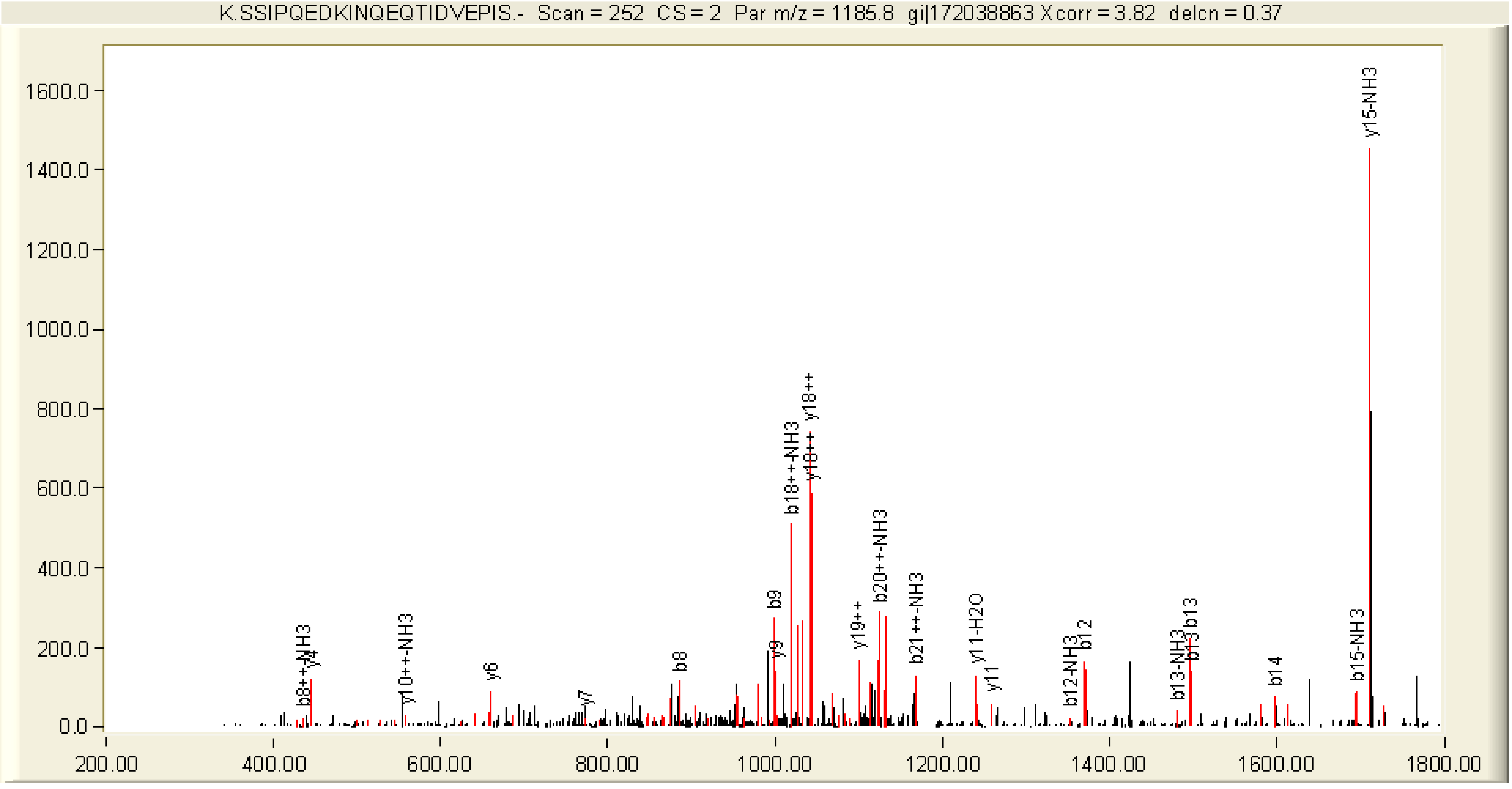

K.SSIPQEDKINQEQTIDVEPIS.- Scan = 252 CS = 2 Par m/z = 1185.8 gi|172038863 Xcorr = 3.82 delcn = 0.37
1600.0
1400.0
1200.0
1000.0
800.0
600.0
400.0
200.0
0.0
200.00
400.00
600.00
800.00
1000.00
1200.00
1400.00
1600.00
1800.00
y15-NH3
y18++
b18++-NH3
y18++
b20++-NH3
b9
y19++
b21++-NH3
y11-H2O
y11
b12
b12-NH3
b13
b13-NH3
b14
b15-NH3
b8
y9
y7
y6
y10++-NH3
b8++-NH3
y4

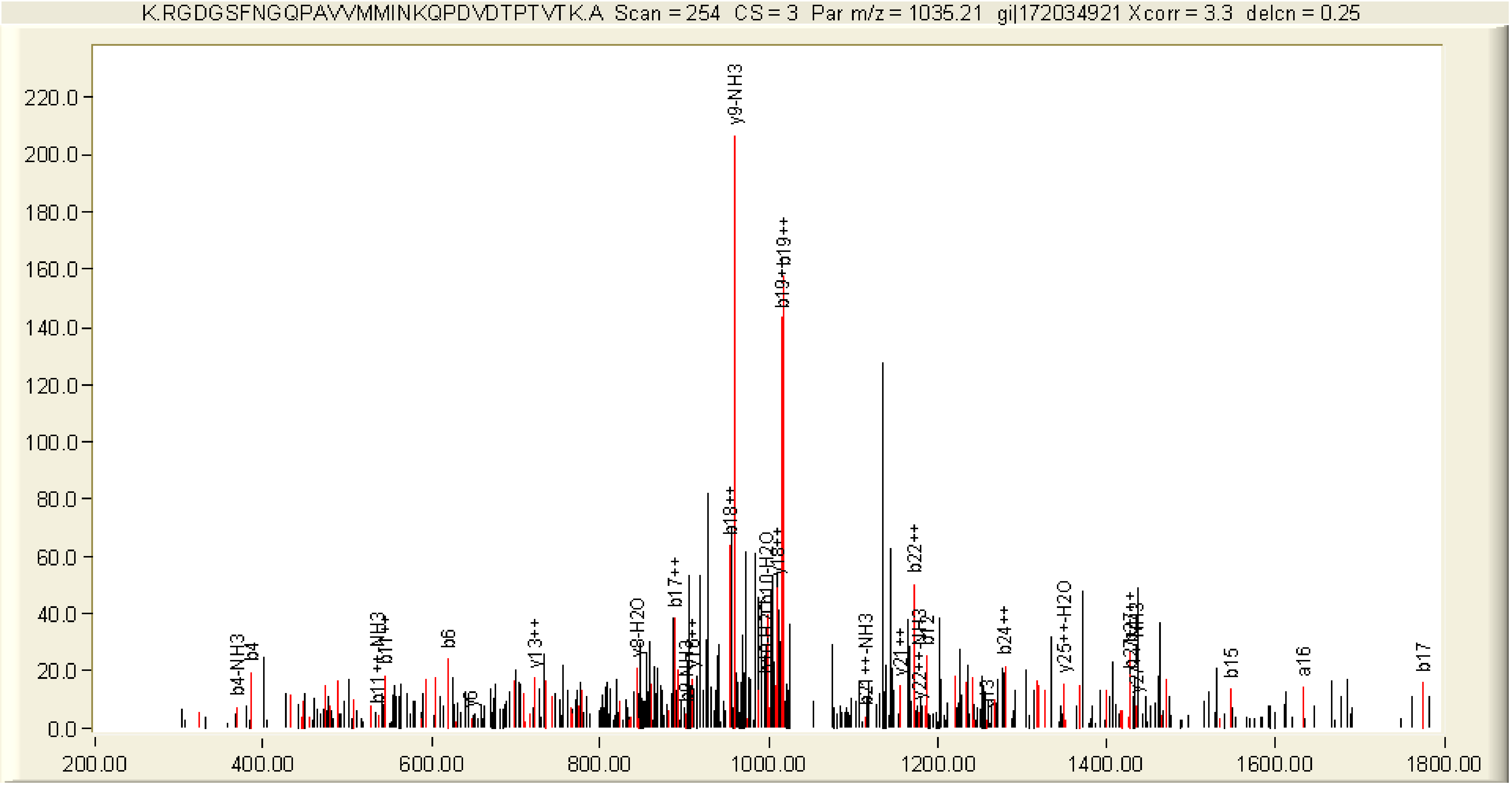
K.RGDGSFNGQPAVVMMINKQPDVDTPTVTK.A Scan = 254 CS = 3 Par m/z = 1035.21 gi|172034921 Xcorr = 3.3 delcn = 0.25
220.0
200.0
180.0
160.0
140.0
120.0
100.0
80.0
60.0
40.0
20.0
0.0
200.00
400.00
600.00
800.00
1000.00
1200.00
1400.00
1600.00
1800.00
y9-NH3
b19++
b18++
b22++
b17++
y13++
b6
b4-NH3
b4
b24++
y25++-H2O
b15
a16
b17

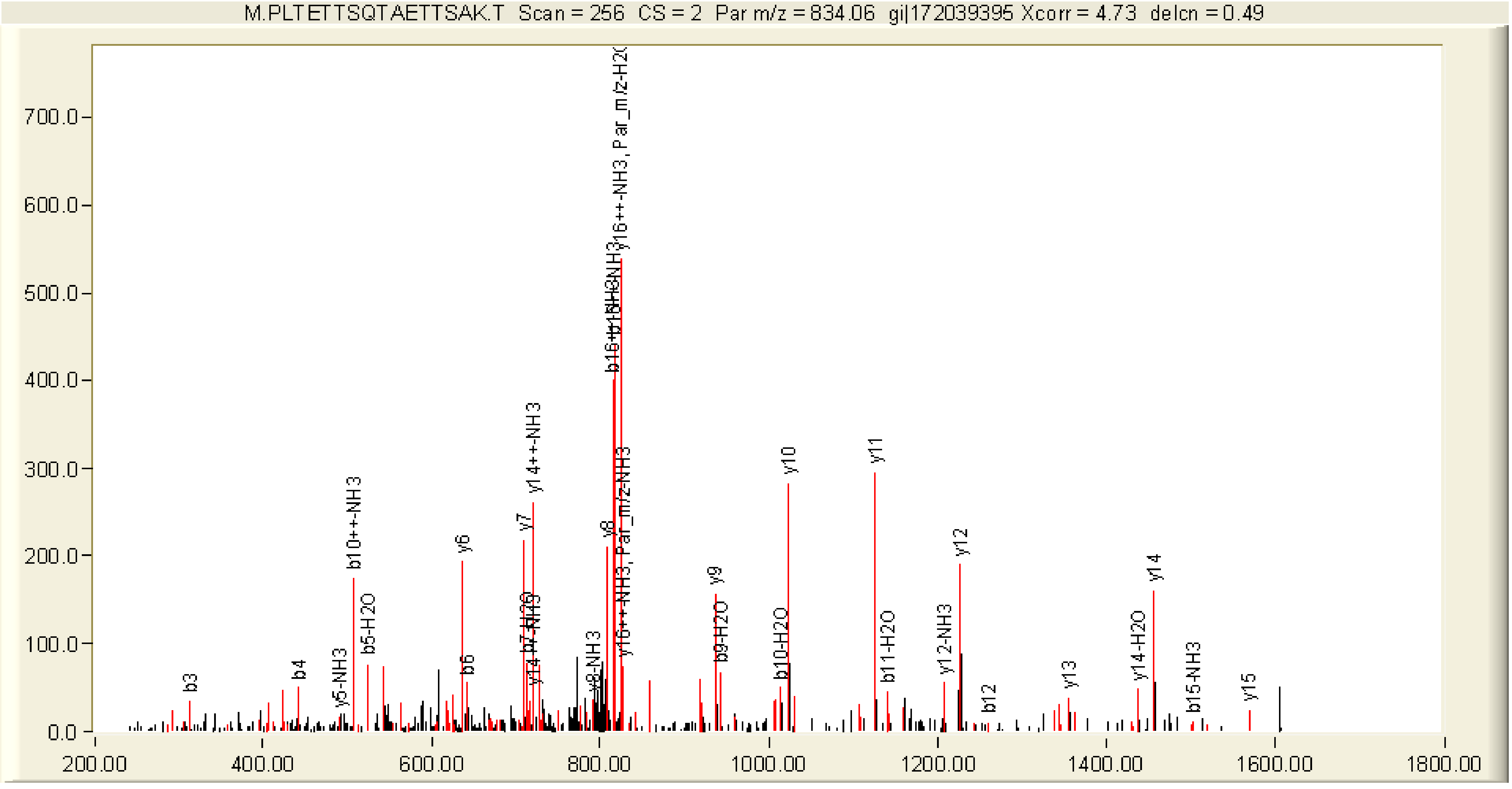
M.PLTETTSQTAETTSAK.T Scan = 256 CS = 2 Par m/z = 834.06 gi|172039395 Xcorr = 4.73 delcn = 0.49
700.0
600.0
500.0
400.0
300.0
200.0
100.0
0.0
200.00
400.00
600.00
800.00
1000.00
1200.00
1400.00
1600.00
1800.00
b3
b4
y5-NH3
b10++-NH3
b5-H2O
y6
b6
y7
y14++-NH3
y8
y16++-NH3, Par_m/z-H2O
y16++-NH3, Par_m/z-NH3
y8-NH3
y9
b9-H2O
y10
b10-H2O
y11
b11-H2O
y12
y12-NH3
b12
y13
y14
y14-H2O
b15-NH3
y15

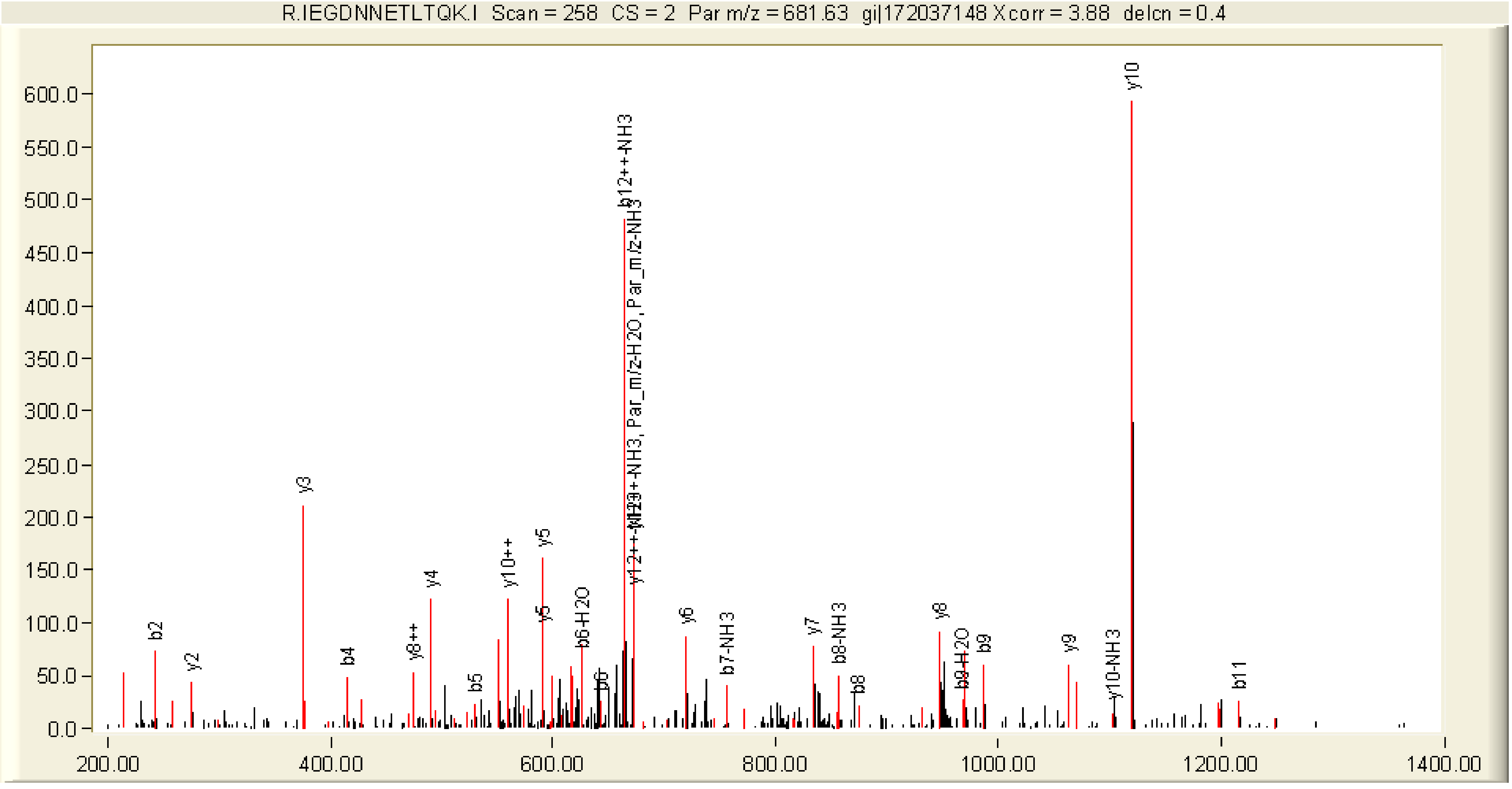

R.IEGDNNETLTQK.I Scan = 258 CS = 2 Par m/z = 681.63 gi|172037148 Xcorr = 3.88 delcn = 0.4
600.0
550.0
500.0
450.0
400.0
350.0
300.0
250.0
200.0
150.0
100.0
50.0
0.0
200.00
400.00
600.00
800.00
1000.00
1200.00
1400.00
b2
y2
y3
b4
y8++
y4
b5
y10++
y5
y5
b6-H2O
b6
b12++-NH3
y12++-NH3
y6
b7-NH3
y7
b8-NH3
b8
y8
b9-H2O
b9
y9
y10-NH3
y10
b11

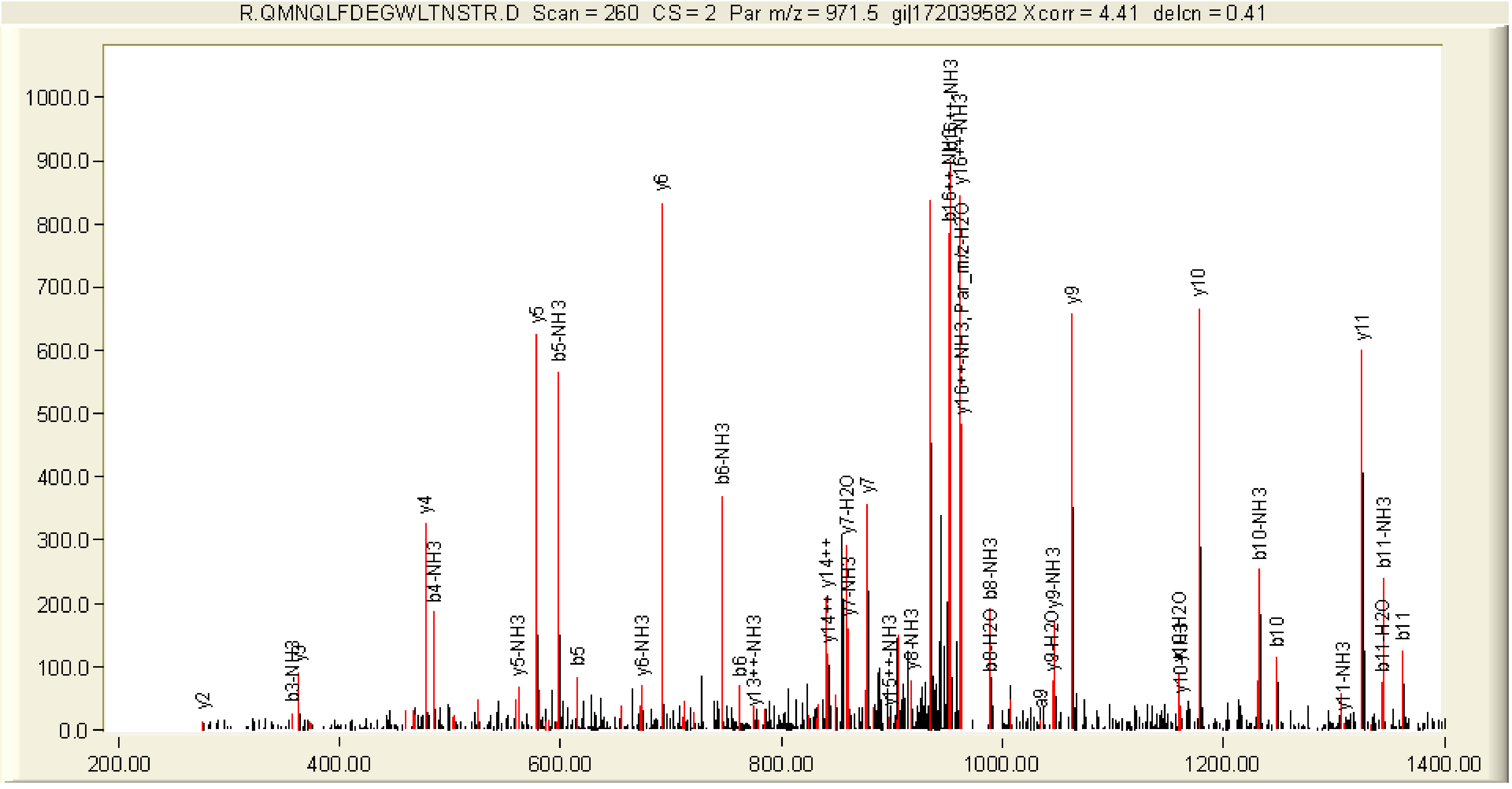
R.QMNQLFDEGWLTNSTR.D Scan = 260 CS = 2 Par m/z = 971.5 gi|172039582 Xcorr = 4.41 delcn = 0.41
1000.0
900.0
800.0
700.0
600.0
500.0
400.0
300.0
200.0
100.0
0.0
200.00
400.00
600.00
800.00
1000.00
1200.00
1400.00
y2
y4
b4-NH3
y5-NH3
y5
b5-NH3
b5
y6-NH3
y6
b6-NH3
b6
y14++
y7-H2O
y7
y8-NH3
y16++-NH3,Par_m/z-H2O
b8-NH3
b8-H2O
a9
y9-H2O
y9-NH3
y9
y10
b10-NH3
b10
y11-NH3
y11
b11-NH3
b11-H2O
b11

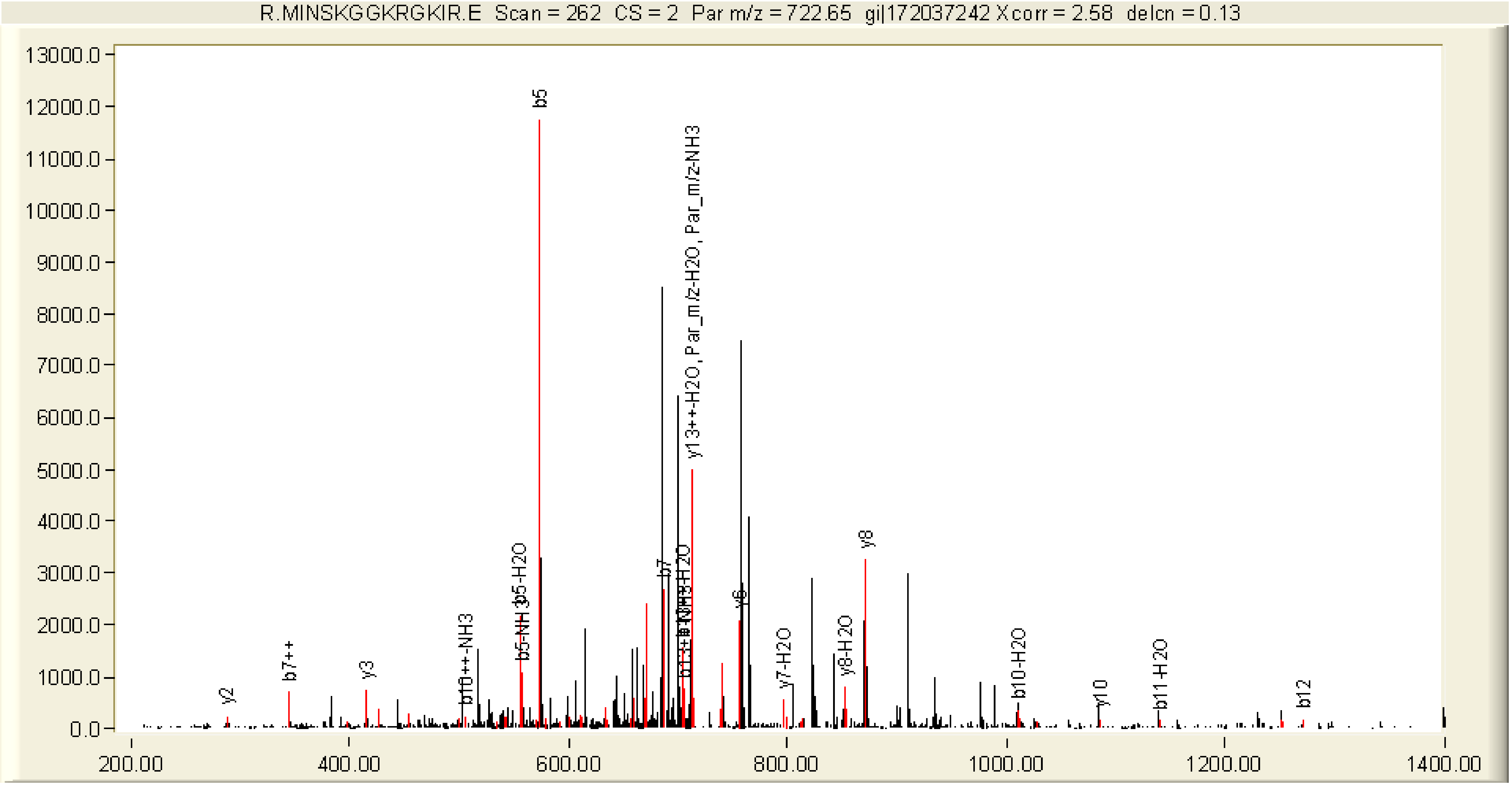
R.MINSKGGKRGKIR.E Scan = 262 CS = 2 Par m/z = 722.65 gi|172037242 Xcorr = 2.58 delcn = 0.13
13000.0
12000.0
11000.0
10000.0
9000.0
8000.0
7000.0
6000.0
5000.0
4000.0
3000.0
2000.0
1000.0
0.0
200.00
400.00
600.00
800.00
1000.00
1200.00
1400.00
b5
y13++-H2O, Par_m/z-H2O, Par_m/z-NH3
y8
b7
y6
y2
b7++
y3
b10++-NH3
b5-NH3
b5-H2O
y7-H2O
y8-H2O
b10-H2O
y10
b11-H2O
b12

K.IIKDNYPEIK.V Scan = 264 CS = 2 Par m/z = 617.19 gi|172039572 Xcorr = 2.84 delcn = 0.13

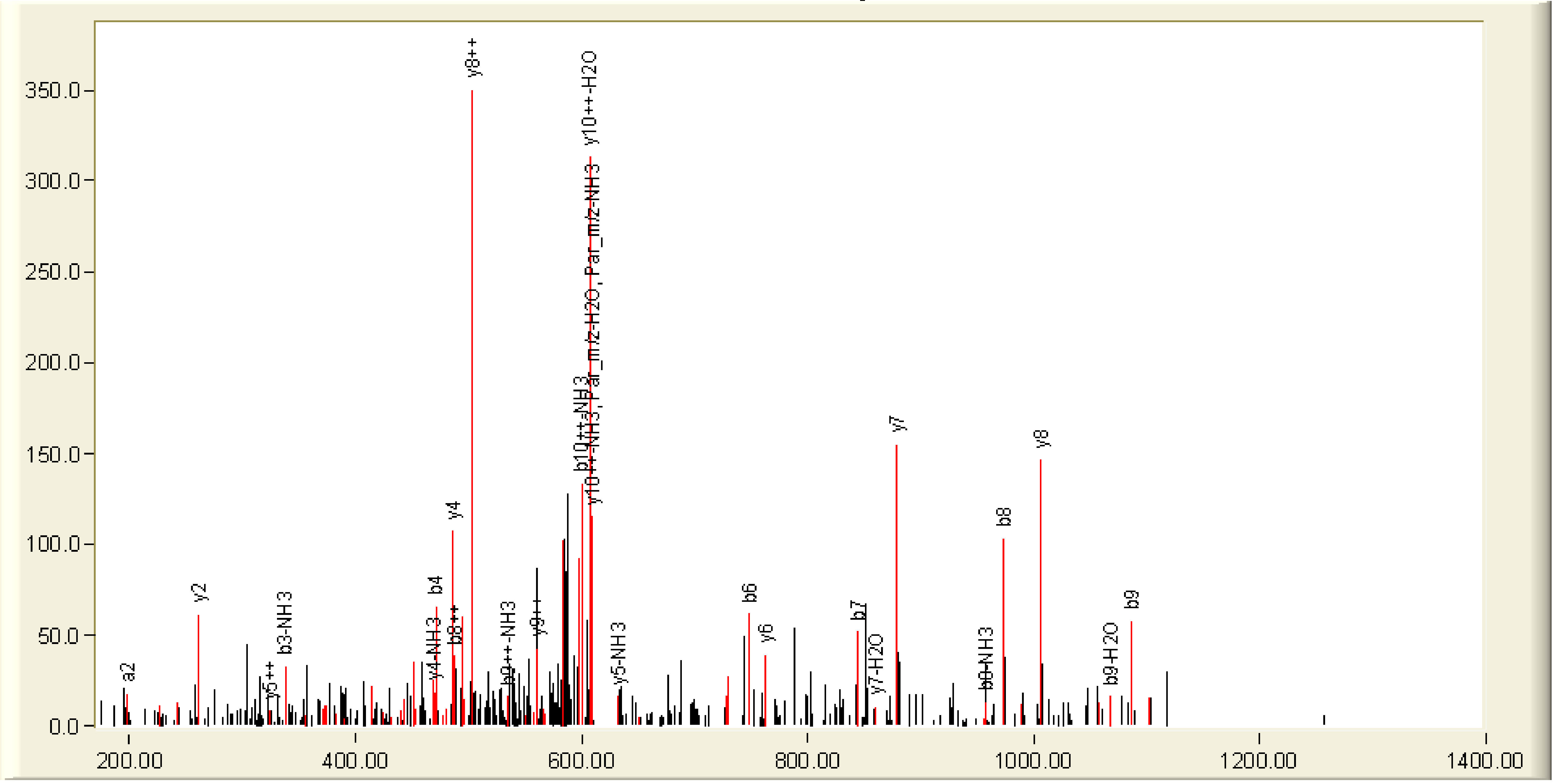

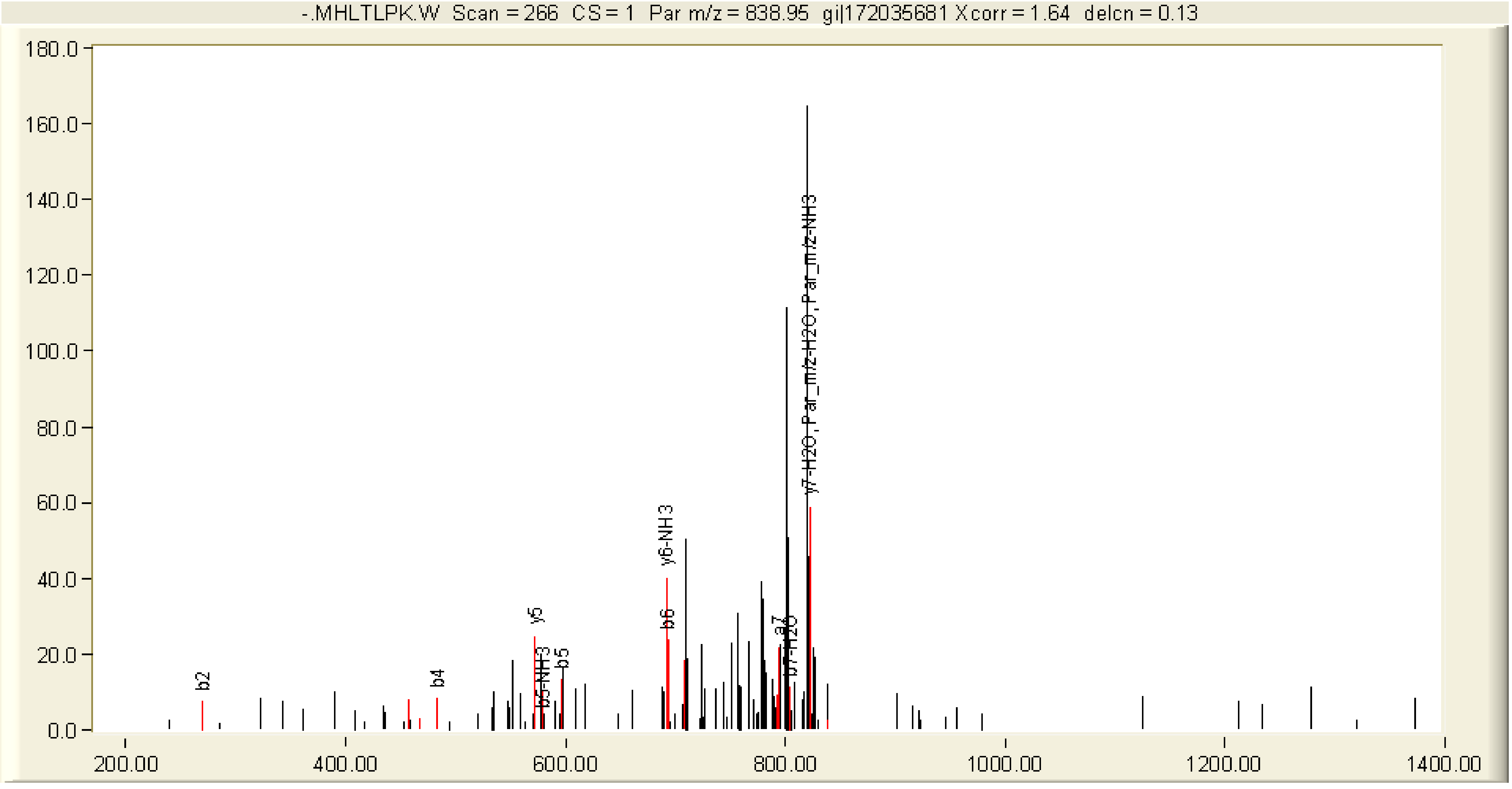
-.MHLTLPK.W Scan = 266 CS = 1 Par m/z = 838.95 gi|172035681 Xcorr = 1.64 delcn = 0.13
180.0
160.0
140.0
120.0
100.0
80.0
60.0
40.0
20.0
0.0
200.00
400.00
600.00
800.00
1000.00
1200.00
1400.00
b2
b4
y5
b5-NH3
b5
b6
y6-NH3
a7
b7-H2O
y7-H2O, Par_m/z-H2O, Par_m/z-NH3

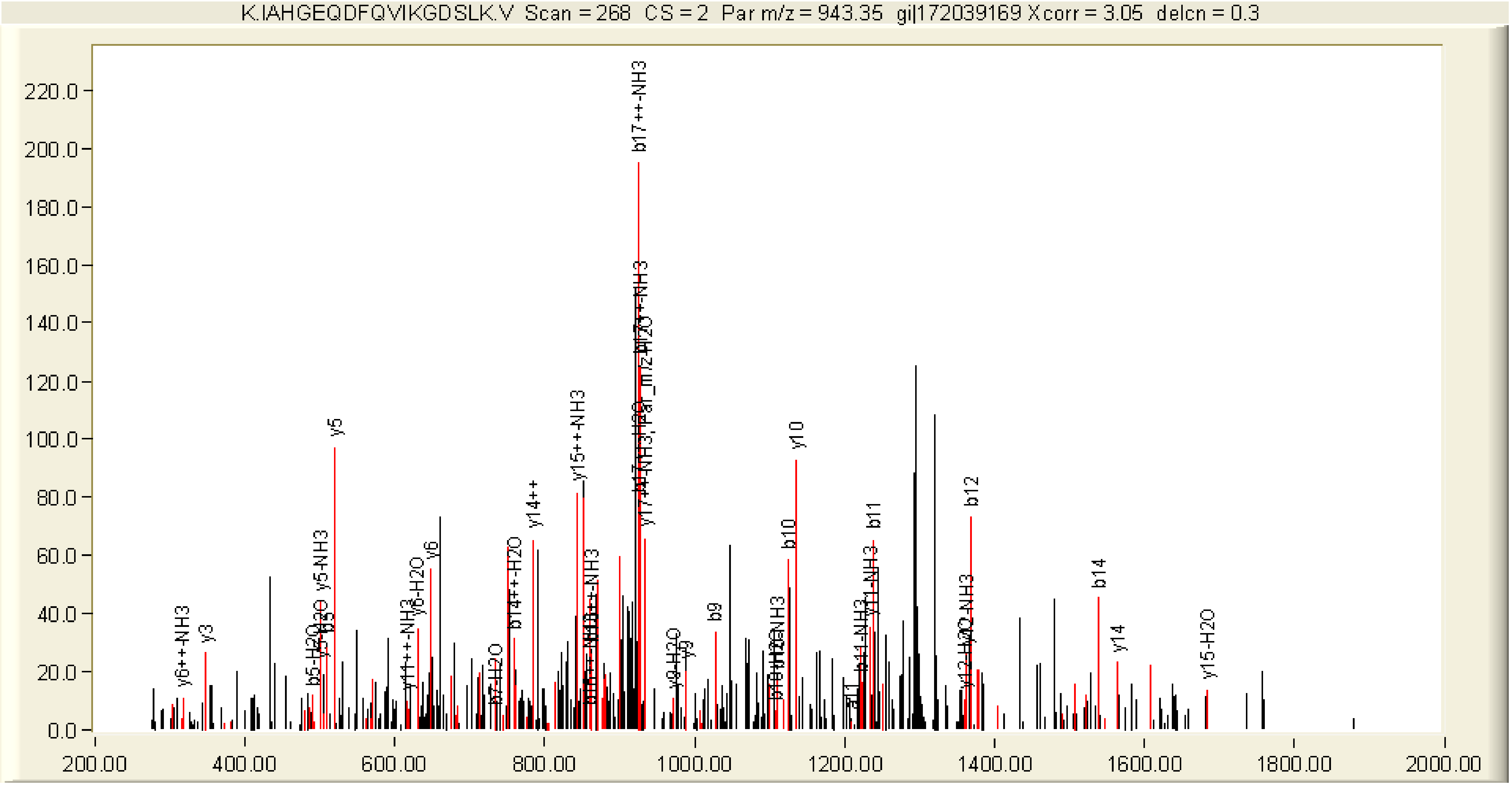

K.IAHGEQDFQVIKGDSLK.V Scan = 268 CS = 2 Par m/z = 943.35 gi|172039169 Xcorr = 3.05 delcn = 0.3
220.0
200.0
180.0
160.0
140.0
120.0
100.0
80.0
60.0
40.0
20.0
0.0
200.00
400.00
600.00
800.00
1000.00
1200.00
1400.00
1600.00
1800.00
2000.00
b17++-NH3
y5
y10
y15++-NH3
b12
b11
b10
y14++
y6
y5-NH3
y6-H2O
b14++-H2O
b14
y3
y6++-NH3
b9
y14
y15-H2O
y11-NH3
y11++-NH3
b5-H2O

K.SDYFSHVSHELR.T Scan = 270 CS = 2 Par m/z = 738.91 gi|172035081 Xcorr = 2.71 delcn = 0.32

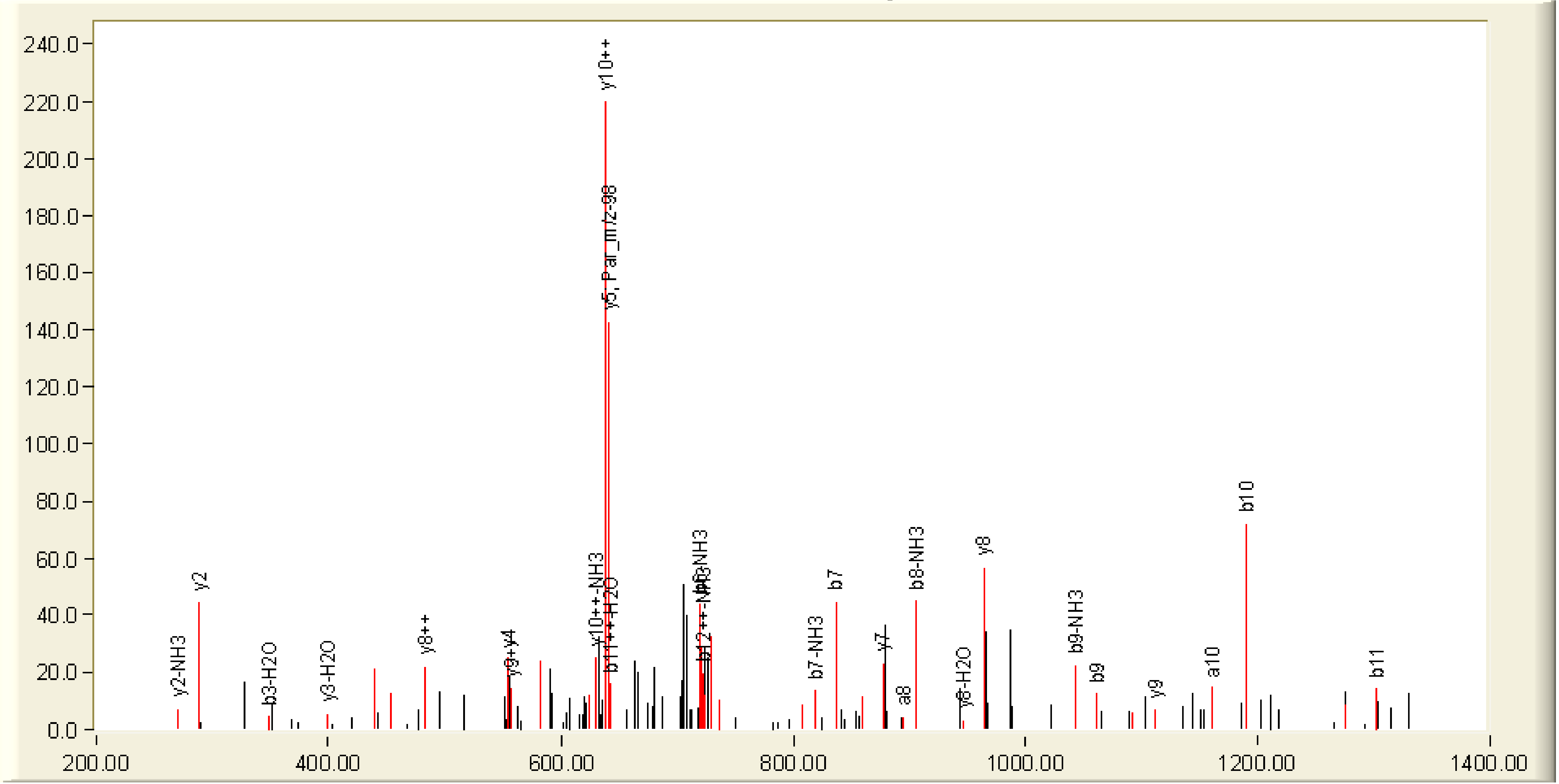

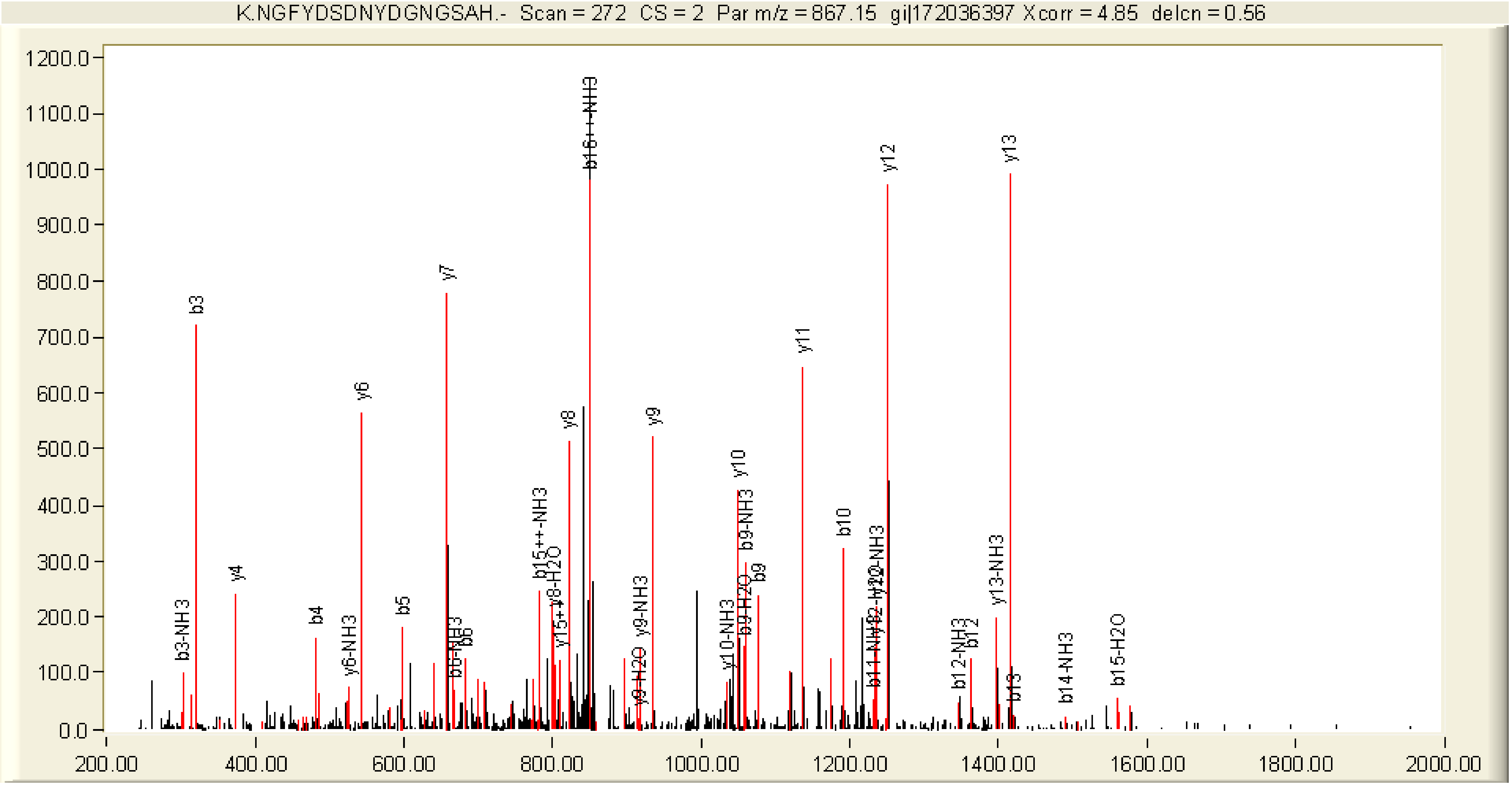
K.NGFYDSDNYDGNGSAH.- Scan = 272 CS = 2 Par m/z = 867.15 gi|172036397 Xcorr = 4.85 delcn = 0.56
b3
y7
y6
y8
y9
y10
y11
y12
y13
b10
b9
b9-NH3
b3-NH3
y4
b4
y6-NH3
b5
b15++-NH3
y9-NH3
y10-NH3
y13-NH3
b12-NH3
b12
b13
b14-NH3
b15-H2O
1200.0
1100.0
1000.0
900.0
800.0
700.0
600.0
500.0
400.0
300.0
200.0
100.0
0.0
200.00
400.00
600.00
800.00
1000.00
1200.00
1400.00
1600.00
1800.00
2000.00

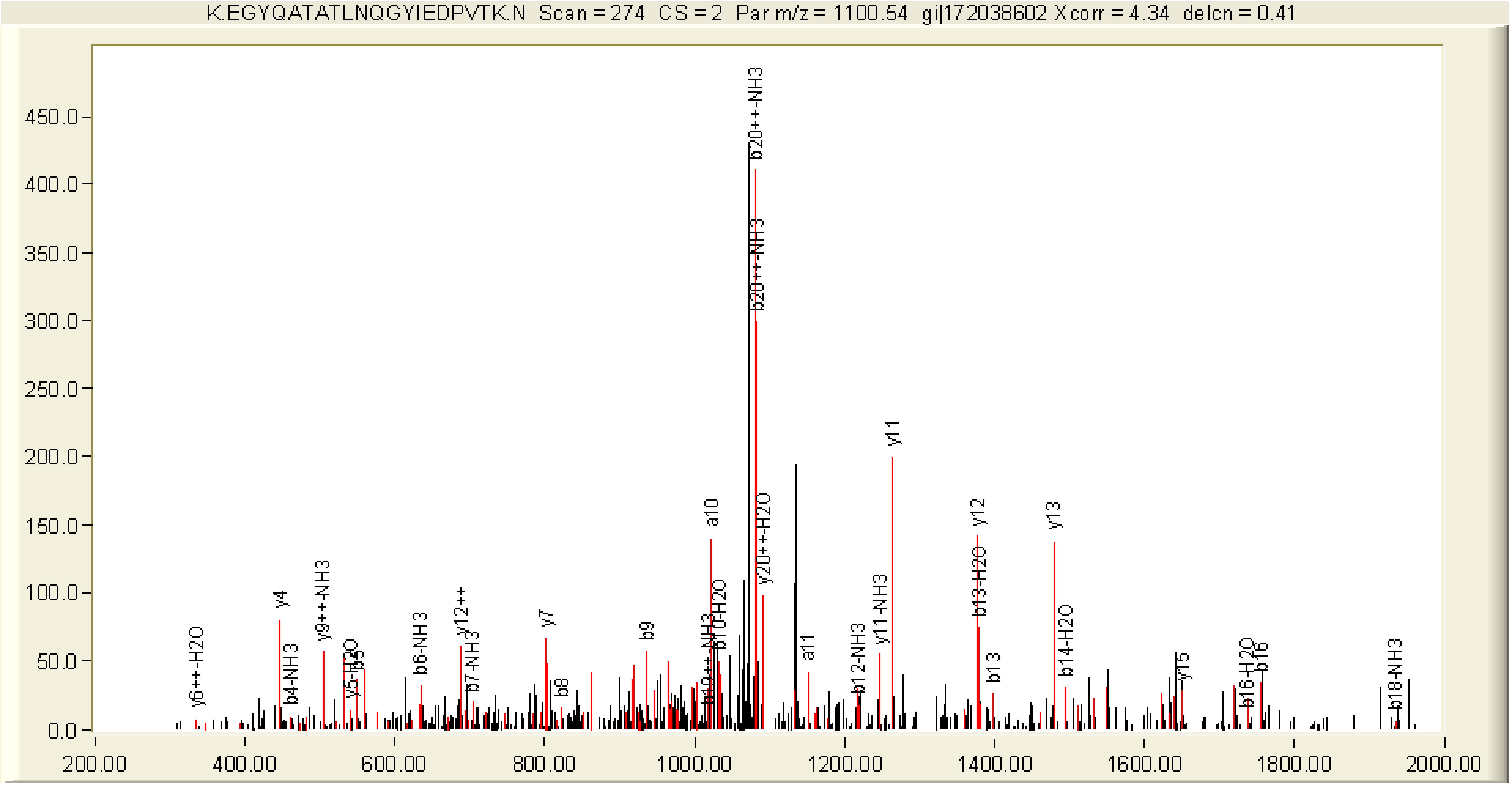
K.EGYQATATLNQGYIEDPVTK.N Scan = 274 CS = 2 Par m/z = 1100.54 gi|172038602 Xcorr = 4.34 delcn = 0.41
450.0
400.0
350.0
300.0
250.0
200.0
150.0
100.0
50.0
0.0
200.00
400.00
600.00
800.00
1000.00
1200.00
1400.00
1600.00
1800.00
2000.00
y6++-H2O
y4
b4-NH3
y9++-NH3
y5-H2O
b6-NH3
y12++
b7-NH3
y7
b8
b9
a10
b10-H2O
b20++-NH3
b20++-NH3
y20++-H2O
a11
b12-NH3
y11-NH3
y11
y12
b13-H2O
b13
y13
b14-H2O
y15
b16-H2O
b16
b18-NH3

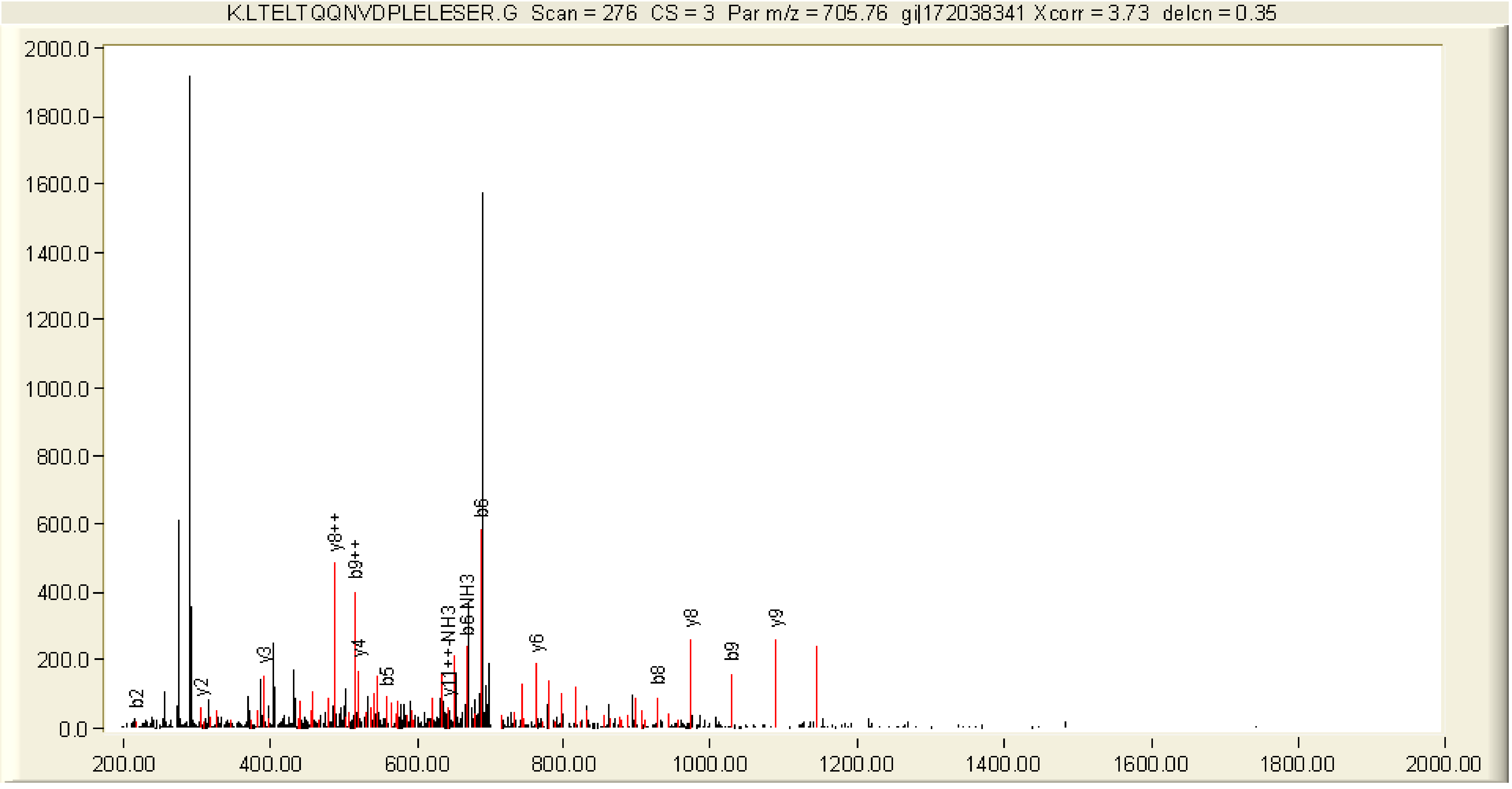

K.LTELTQQNVDPLELESER.G Scan = 276 CS = 3 Par m/z = 705.76 gi|172038341 Xcorr = 3.73 delcn = 0.35
2000.0
1800.0
1600.0
1400.0
1200.0
1000.0
800.0
600.0
400.0
200.0
0.0
200.00
400.00
600.00
800.00
1000.00
1200.00
1400.00
1600.00
1800.00
2000.00
b2
y2
y3
y8++
b9++
y4
b5
b6-NH3
b6
y6
b8
y8
b9
y9

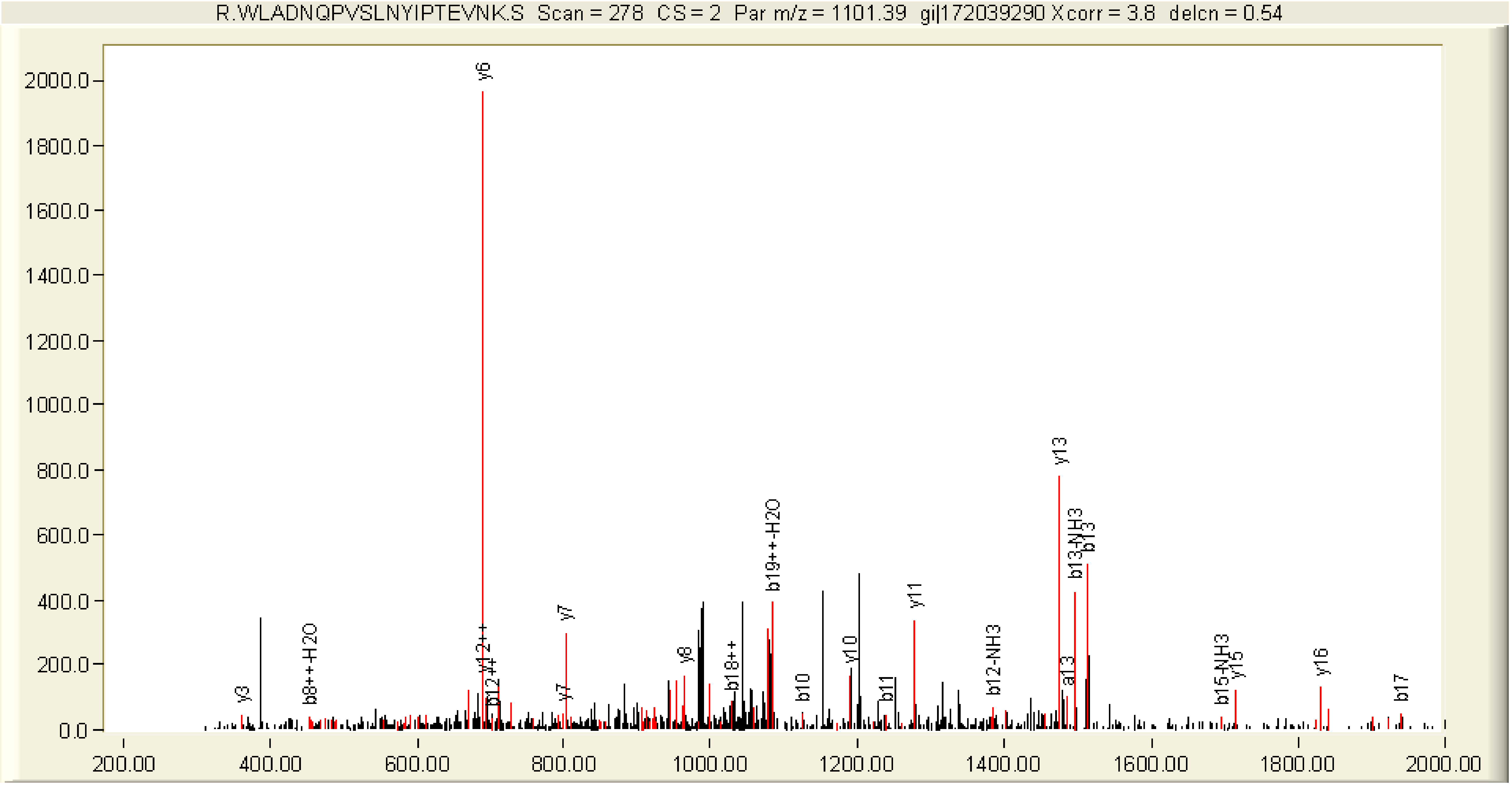

R.WLADNQPVSLNYIPTEVNK.S Scan = 278 CS = 2 Par m/z = 1101.39 gi|172039290 Xcorr = 3.8 delcn = 0.54
2000.0
1800.0
1600.0
1400.0
1200.0
1000.0
800.0
600.0
400.0
200.0
0.0
200.00
400.00
600.00
800.00
1000.00
1200.00
1400.00
1600.00
1800.00
2000.00
y6
y13
b19++-H2O
b13-NH3
b13
y11
y7
b8++-H2O
y12++
b12++
y3
y8
b18++
b10
y10
b11
b12-NH3
a13
b15-NH3
y15
y16
b17

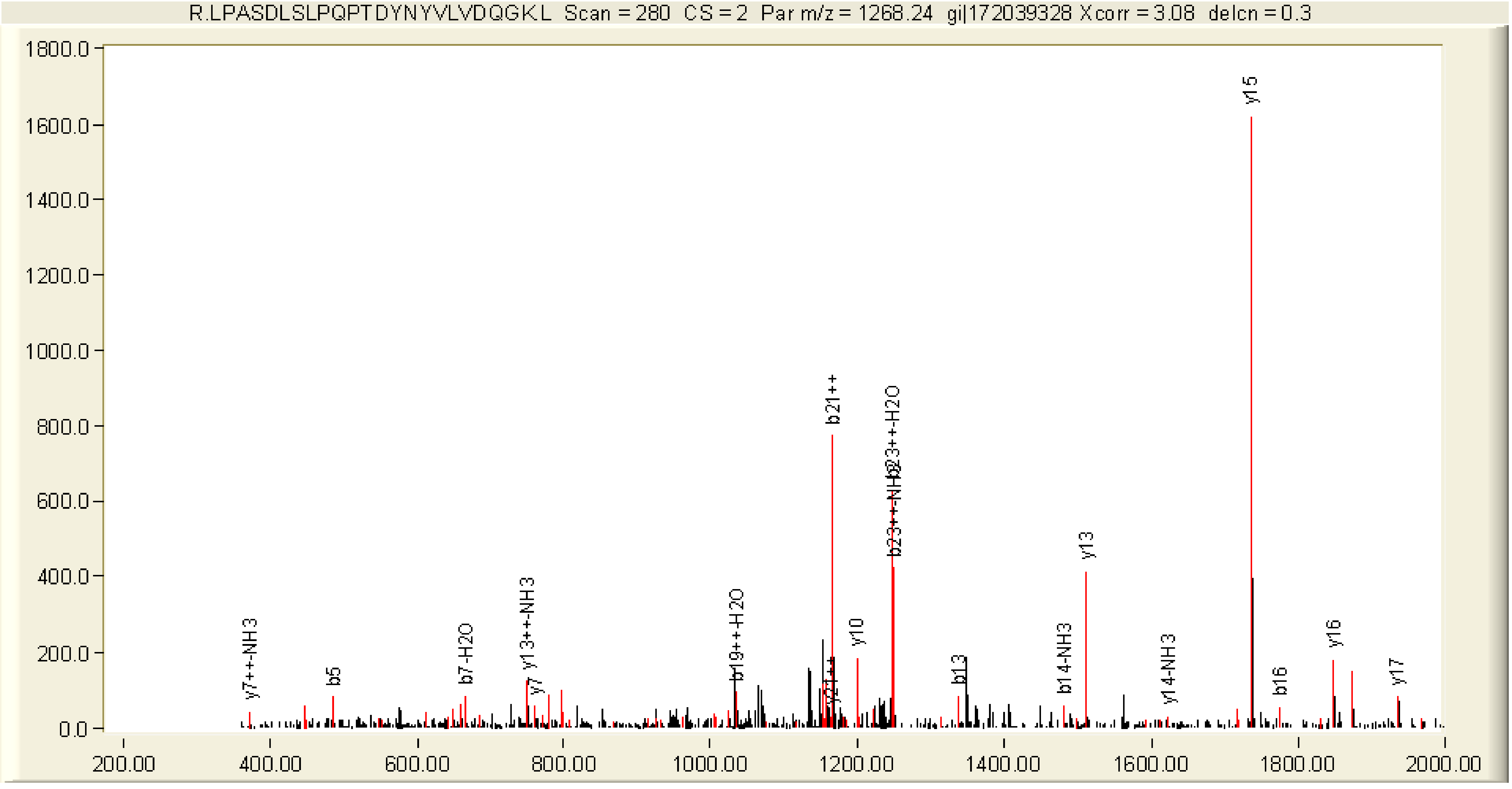
R.LPASDLSLPQPTDYNYVLVDQGKL Scan = 280 CS = 2 Par m/z = 1268.24 gi|172039328 Xcorr = 3.08 delcn = 0.3
y15
b21++
b23++-H2O
b23++-NH3
y13
y10
y7++-NH3
b5
b7-H2O
y13++-NH3
y7
b19++-H2O
y21++
b13
b14-NH3
y14-NH3
b16
y16
y17
1800.0
1600.0
1400.0
1200.0
1000.0
800.0
600.0
400.0
200.0
0.0
200.00
400.00
600.00
800.00
1000.00
1200.00
1400.00
1600.00
1800.00
2000.00

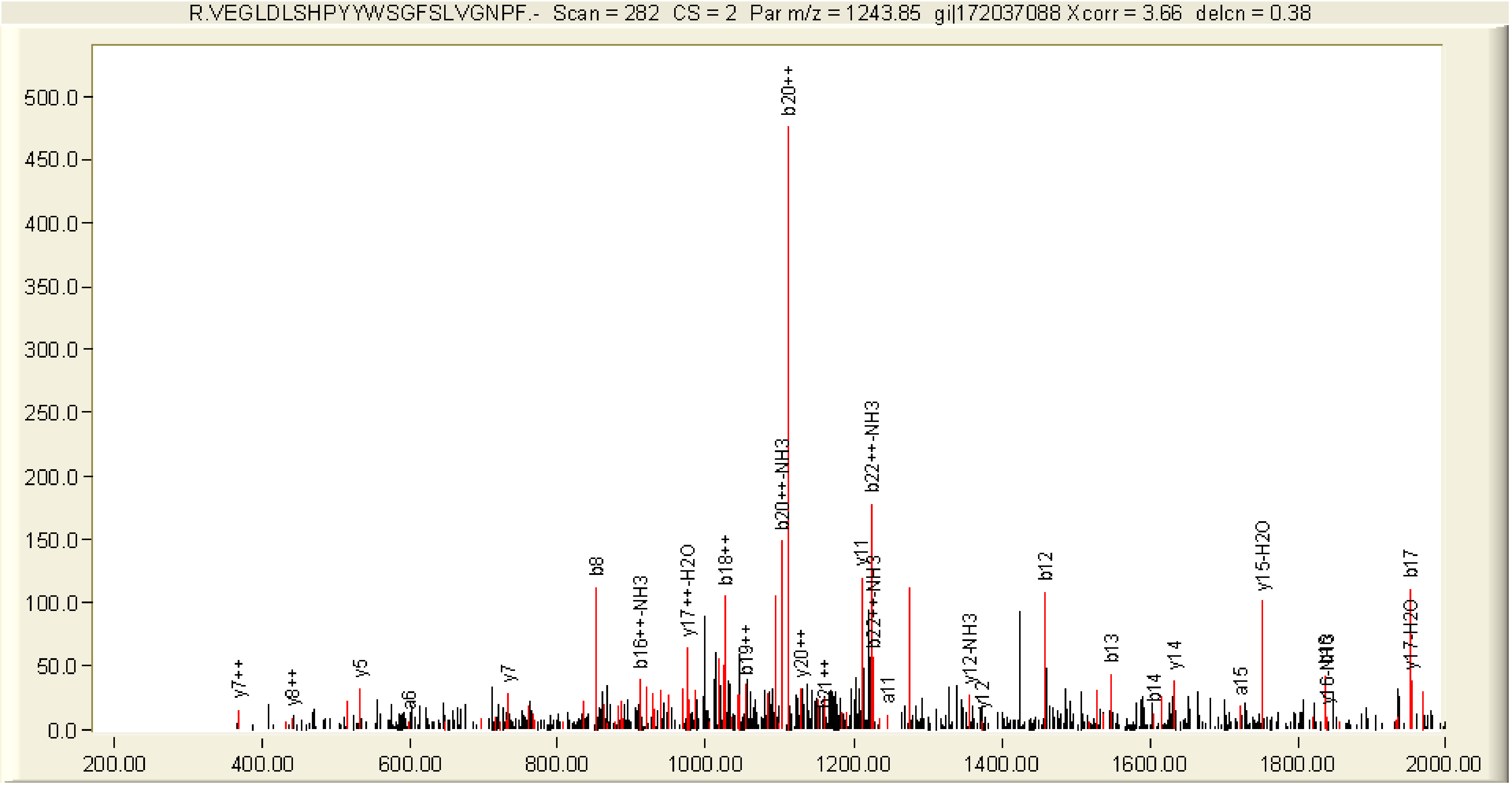
R.VEGLDLSHPYYWSGFSLVGNPF.- Scan = 282 CS = 2 Par m/z = 1243.85 gi|172037088 Xcorr = 3.66 delcn = 0.38
500.0
450.0
400.0
350.0
300.0
250.0
200.0
150.0
100.0
50.0
0.0
200.00
400.00
600.00
800.00
1000.00
1200.00
1400.00
1600.00
1800.00
2000.00
b20++
b20++-NH3
b22++-NH3
b8
b16++-NH3
y17++-H2O
b18++
b19++
y20++
y11
a11
y12-NH3
b12
b13
b14
y14
a15
y15-H2O
b17
y17-H2O
y7++
y8++
y5
a6
y7

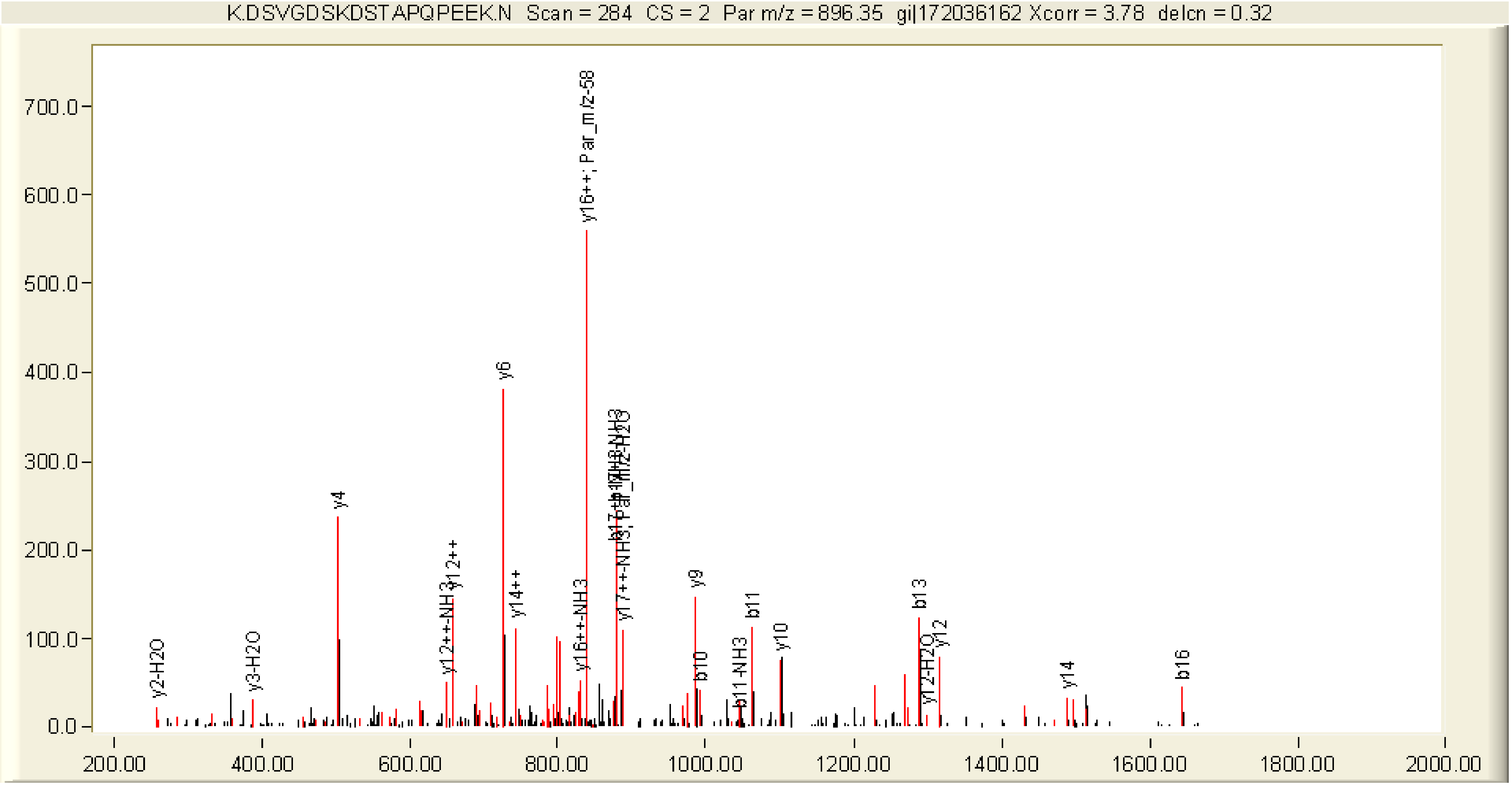

K.DSVGDSKDSTAPQPEEK.N Scan = 284 CS = 2 Par m/z = 896.35 gi|172036162 Xcorr = 3.78 delcn = 0.32
y16++; Par_m/z-58
y6
y4
y12++
y12++-NH3
y14++
y16++-NH3
y17++-NH3
y9
b10
b11
b11-NH3
y10
b13
y12
y12-H2O
y14
b16
y2-H2O
y3-H2O
0.0
100.0
200.0
300.0
400.0
500.0
600.0
700.0
200.00
400.00
600.00
800.00
1000.00
1200.00
1400.00
1600.00
1800.00
2000.00

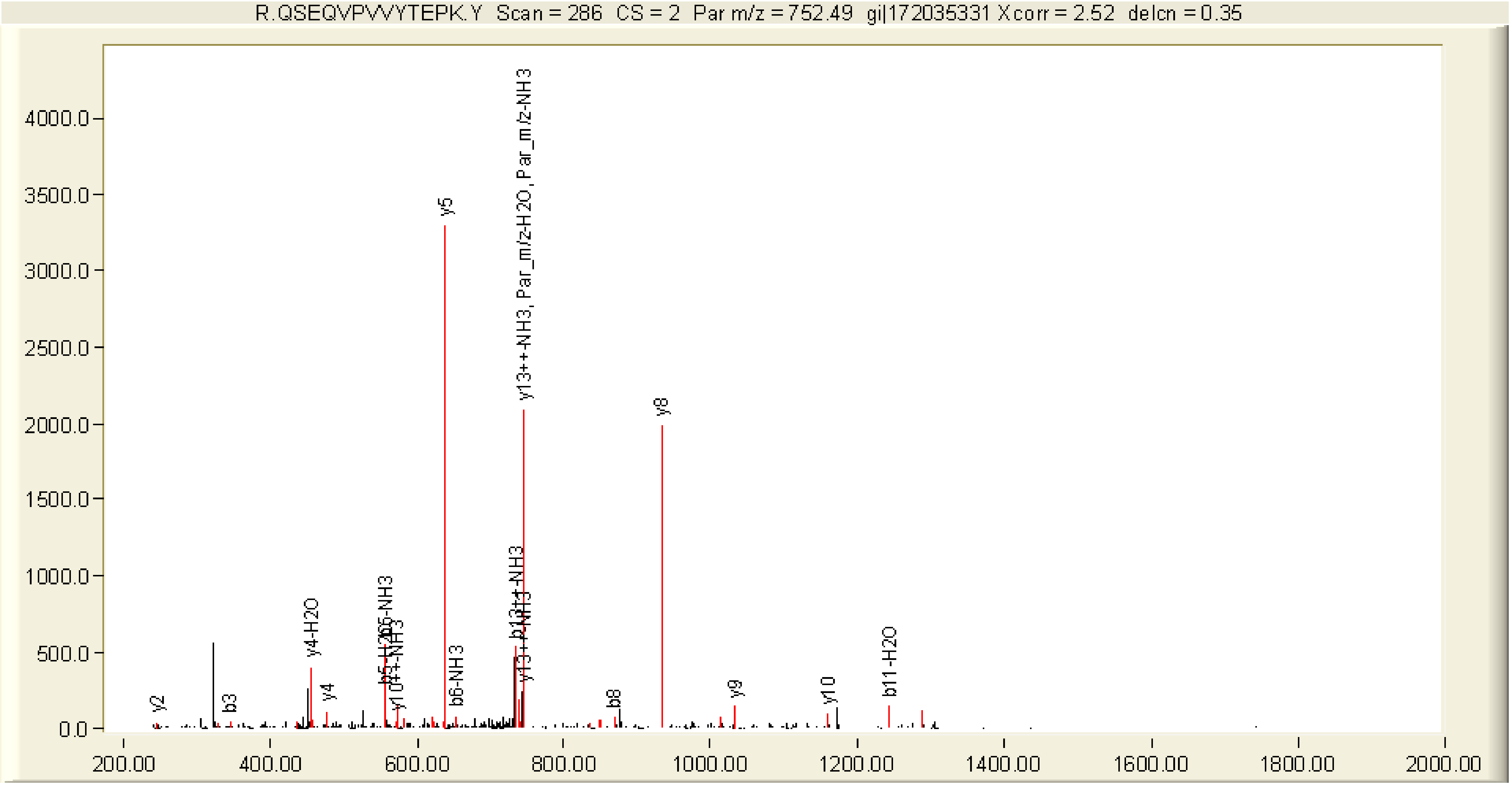
R.QSEQVPVVYTEPK.Y Scan = 286 CS = 2 Par m/z = 752.49 gi|172035331 Xcorr = 2.52 delcn = 0.35
4000.0
3500.0
3000.0
2500.0
2000.0
1500.0
1000.0
500.0
0.0
200.00
400.00
600.00
800.00
1000.00
1200.00
1400.00
1600.00
1800.00
2000.00
y5
y13++-NH3, Par_m/z-H2O, Par_m/z-NH3
y8
y4-H2O
y4
y2
b3
b6-NH3
b8
y9
y10
b11-H2O

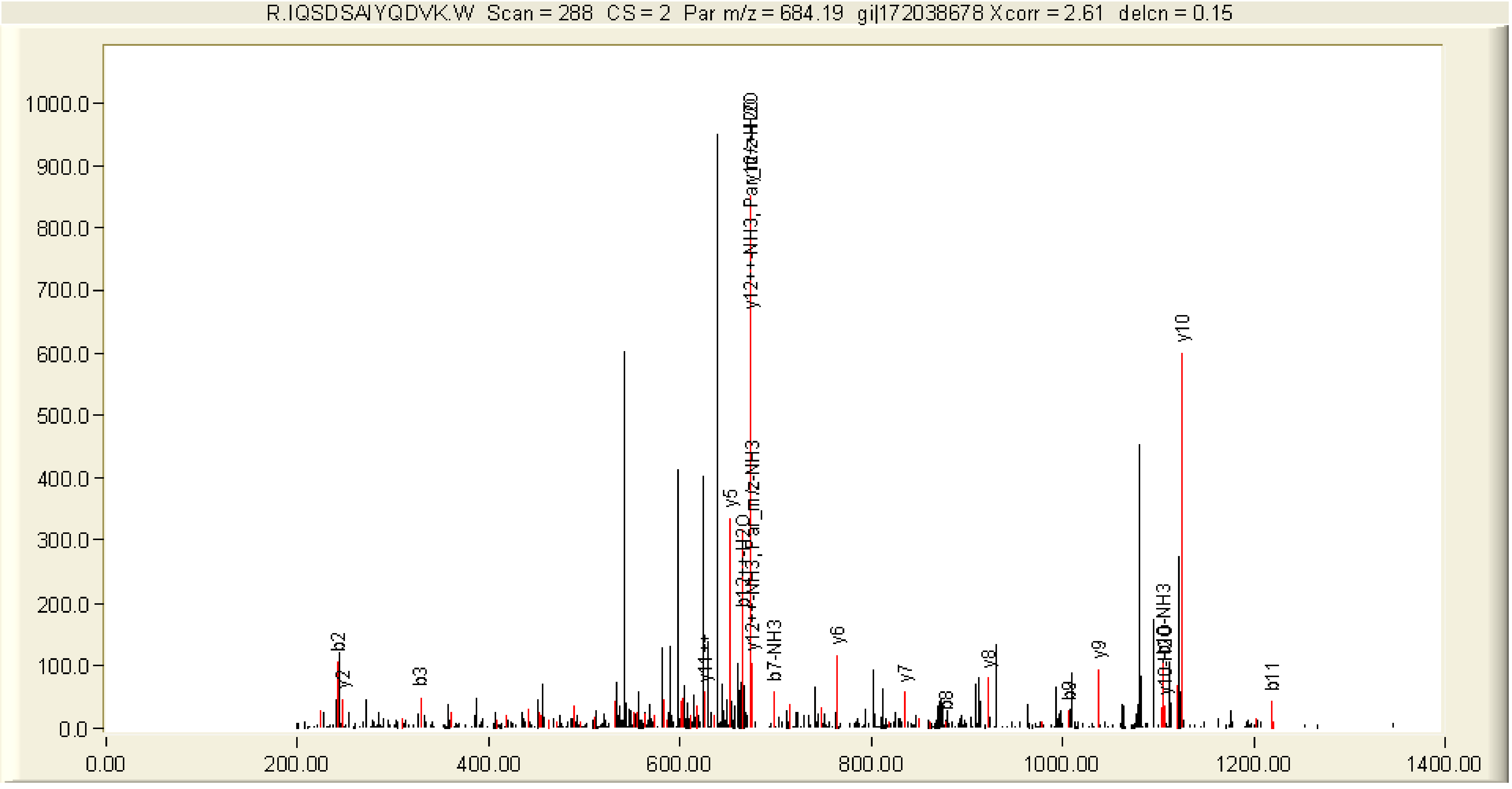
R.IQSDSAIYQDVK.W Scan = 288 CS = 2 Par m/z = 684.19 gi|172038678 Xcorr = 2.61 delcn = 0.15
1000.0
900.0
800.0
700.0
600.0
500.0
400.0
300.0
200.0
100.0
0.0
0.00
200.00
400.00
600.00
800.00
1000.00
1200.00
1400.00
b2
y2
b3
y5
y6
y7
y8
y9
y10
b7-NH3
b8
b9
b11
y10-NH3

R.LQGAPGLSGPDYK.V Scan = 289 CS = 2 Par m/z = 651.76 gi|172037920 Xcorr = 2.41 delcn = 0.21

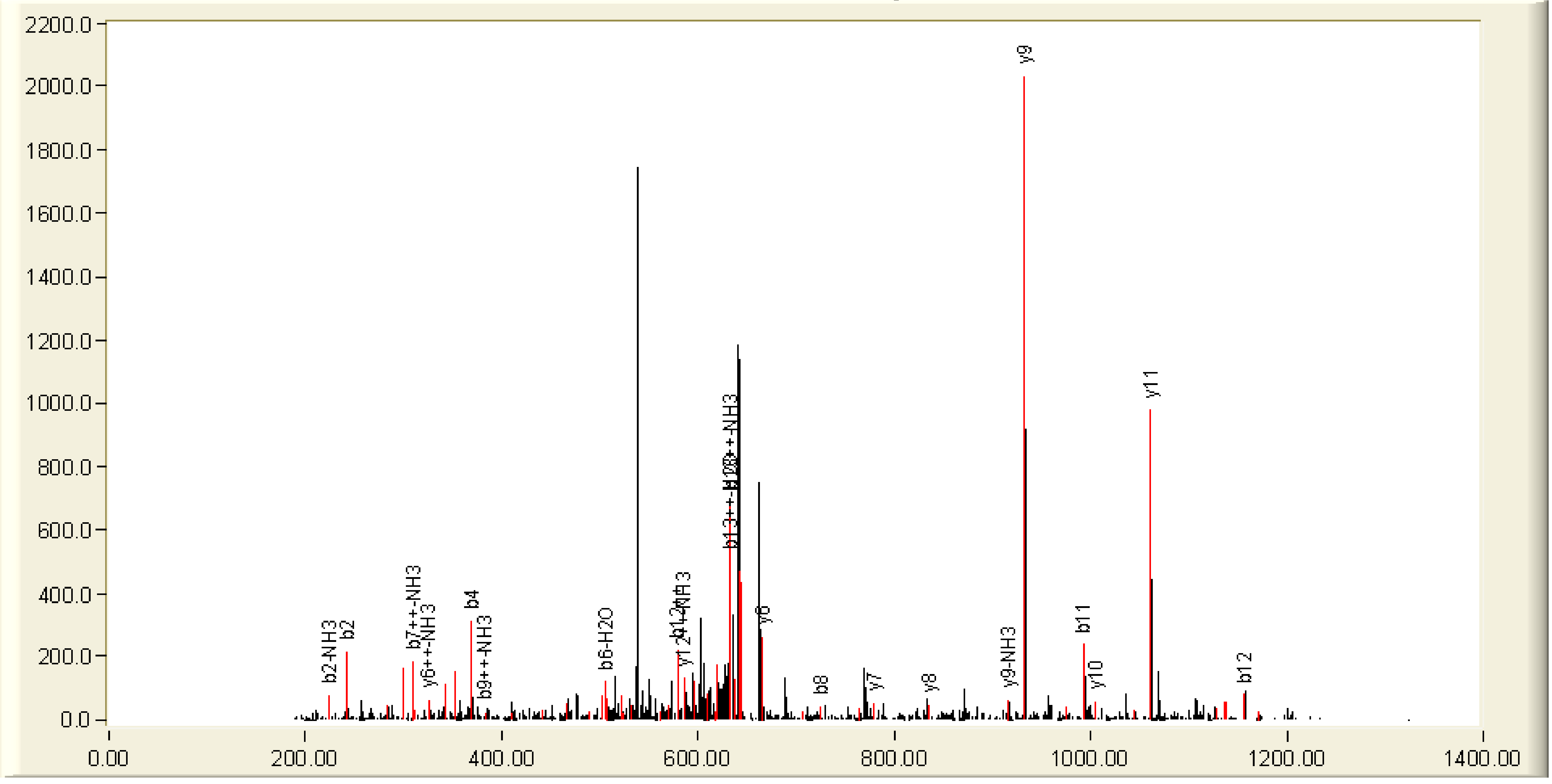

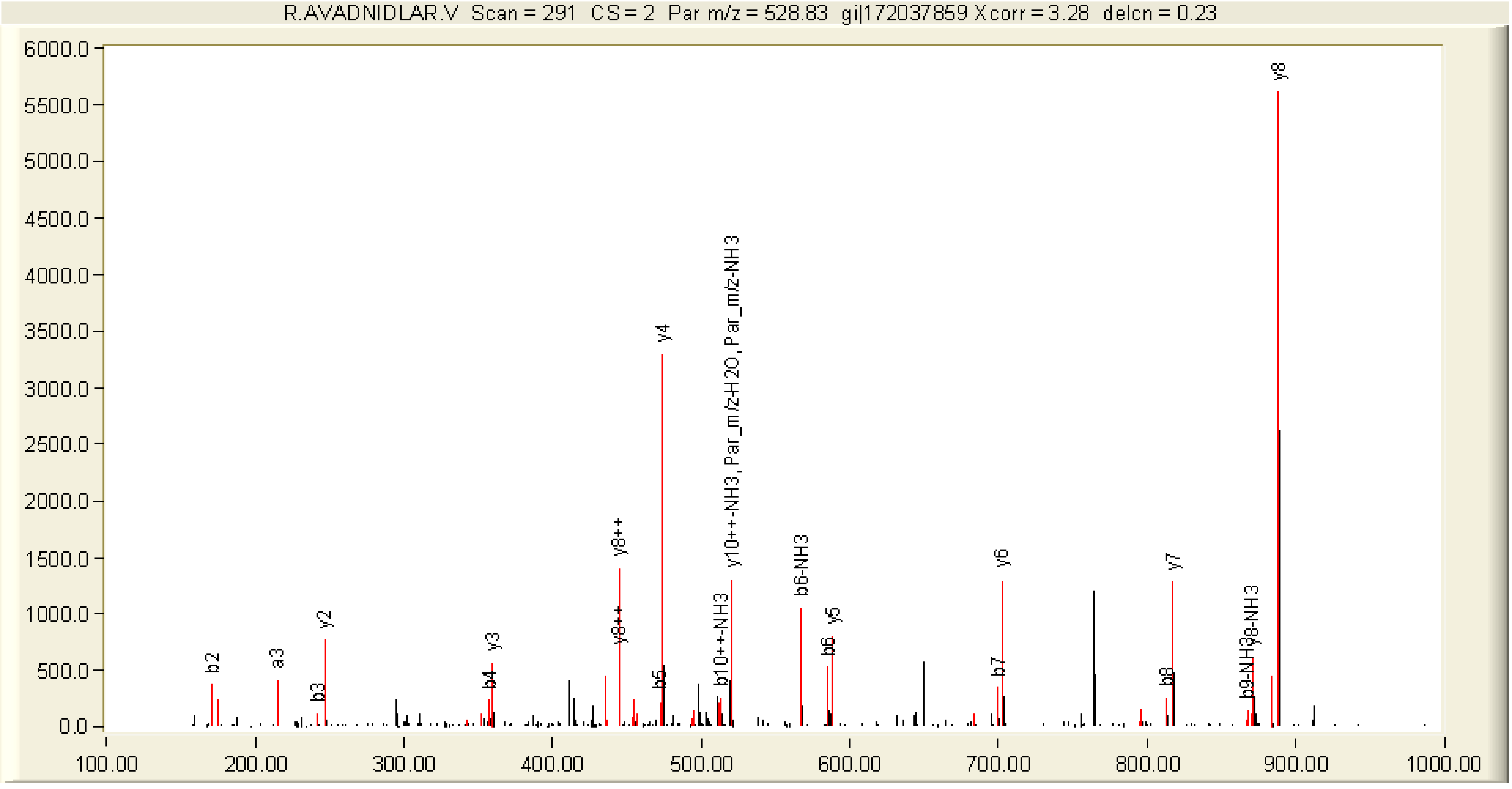
R.AVADNIDLAR.V Scan = 291 CS = 2 Par m/z = 528.83 gi|172037859 Xcorr = 3.28 delcn = 0.23
y8
y4
y10++-NH3, Par_m/z-H2O, Par_m/z-NH3
y8++
b6-NH3
y6
y7
y8-NH3
b9-NH3
y5
b6
b10++-NH3
y8++
y2
y3
a3
b2
b3
b4
b5
b7
b8
6000.0
5500.0
5000.0
4500.0
4000.0
3500.0
3000.0
2500.0
2000.0
1500.0
1000.0
500.0
0.0
100.00
200.00
300.00
400.00
500.00
600.00
700.00
800.00
900.00
1000.00

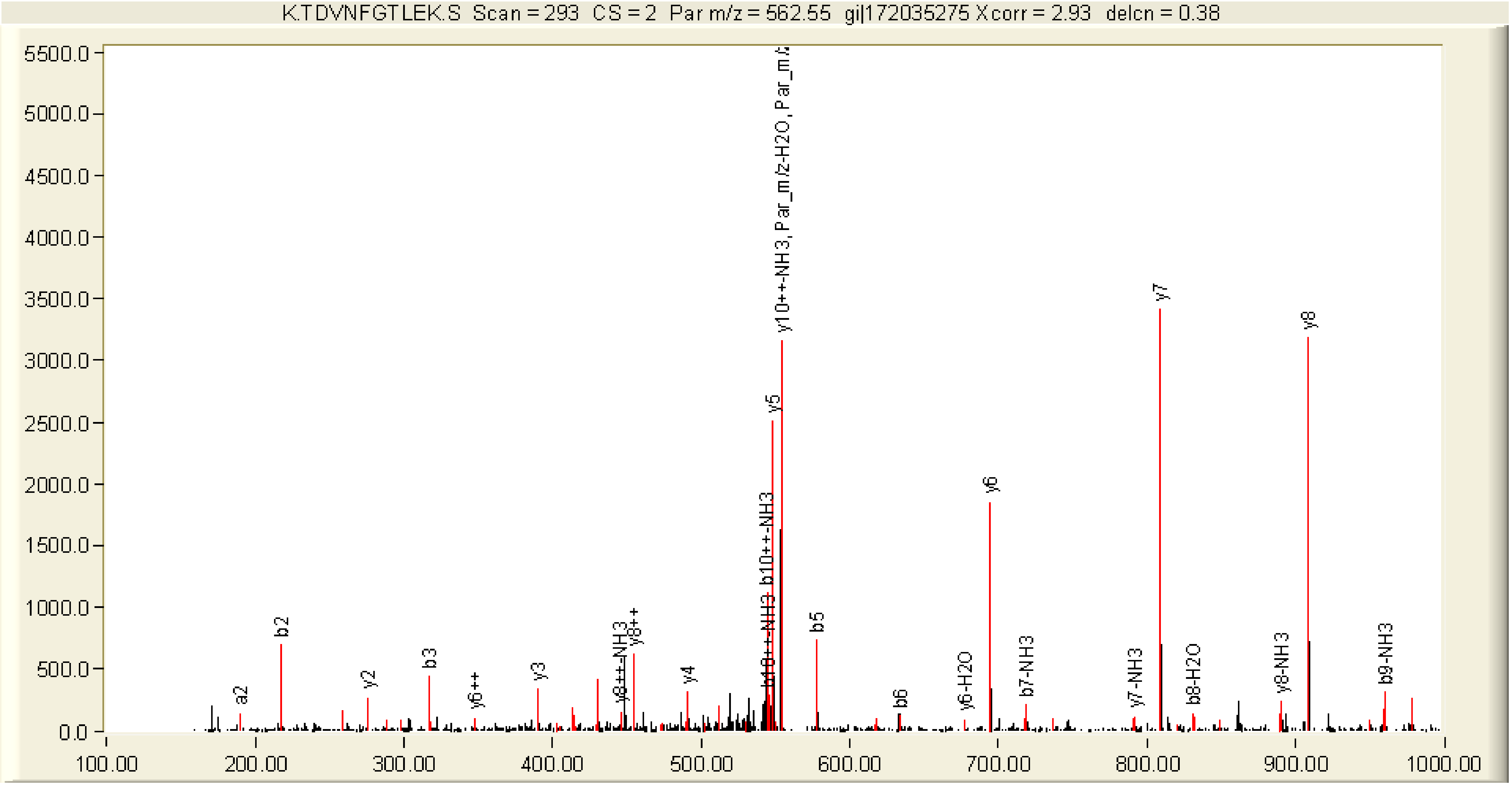

K.TDVNFGTLEK.S Scan = 293 CS = 2 Par m/z = 562.55 gi|172035275 Xcorr = 2.93 delcn = 0.38
5500.0
5000.0
4500.0
4000.0
3500.0
3000.0
2500.0
2000.0
1500.0
1000.0
500.0
0.0
100.00
200.00
300.00
400.00
500.00
600.00
700.00
800.00
900.00
1000.00
a2
b2
y2
b3
y6++
y3
y8++-NH3
y8++
y4
b10++-NH3
y5
y10++-NH3, Par_m/z-H2O, Par_m/z
b5
b6
y6-H2O
y6
b7-NH3
y7-NH3
y7
b8-H2O
y8-NH3
y8
b9-NH3

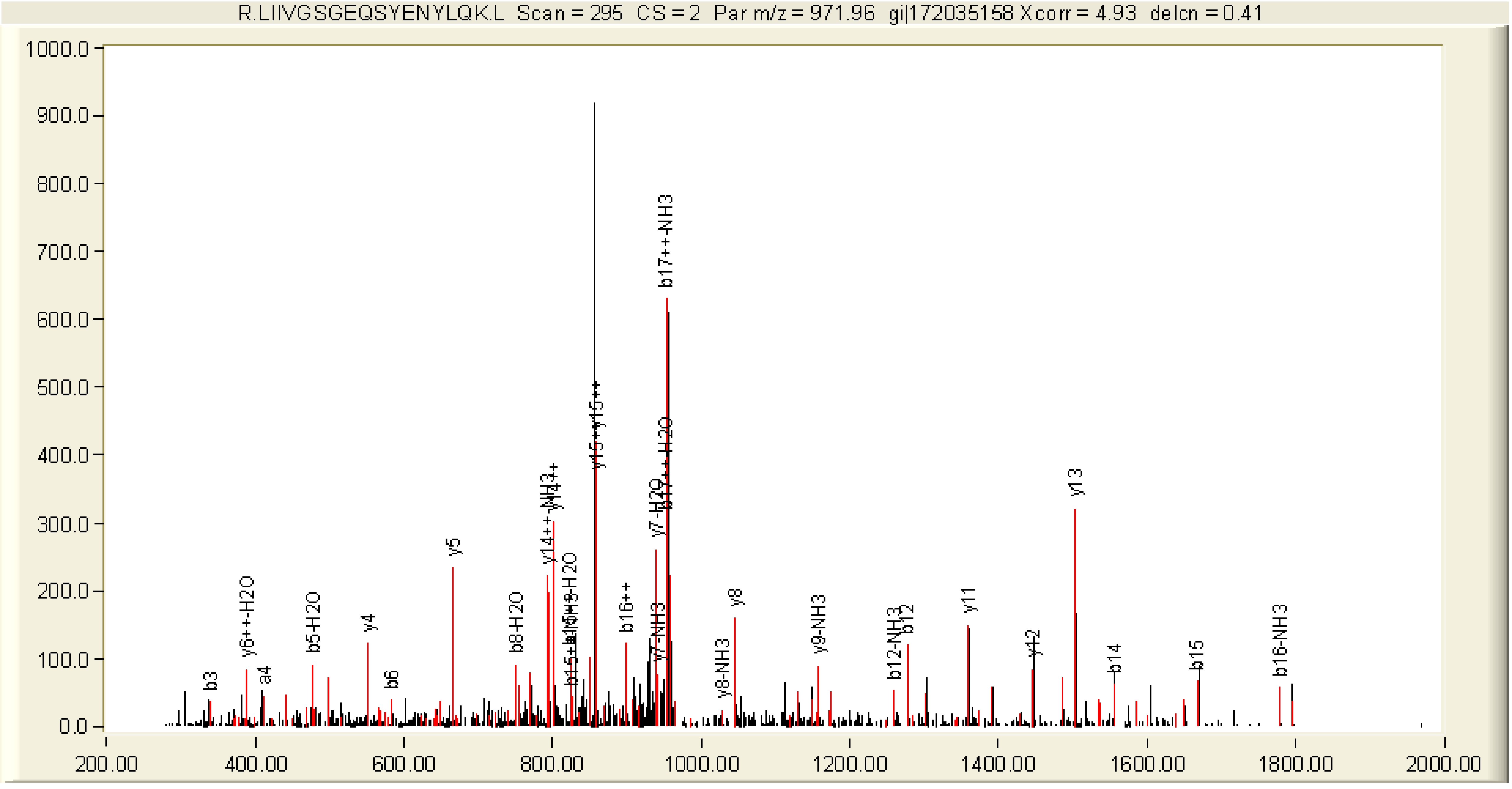
R.LIIVGSGEQSYENYLQK.L Scan = 295 CS = 2 Par m/z = 971.96 gi|172035158 Xcorr = 4.93 delcn = 0.41
1000.0
900.0
800.0
700.0
600.0
500.0
400.0
300.0
200.0
100.0
0.0
200.00
400.00
600.00
800.00
1000.00
1200.00
1400.00
1600.00
1800.00
2000.00
b3
y6++-H2O
a4
b5-H2O
y4
b6
y5
b8-H2O
y14++-NH3
y14++
y15++
b16++
y7-H2O
y7-NH3
b17++-H2O
b17++-NH3
y8-NH3
y8
y9-NH3
b12-NH3
b12
y11
y12
y13
b14
b15
b16-NH3

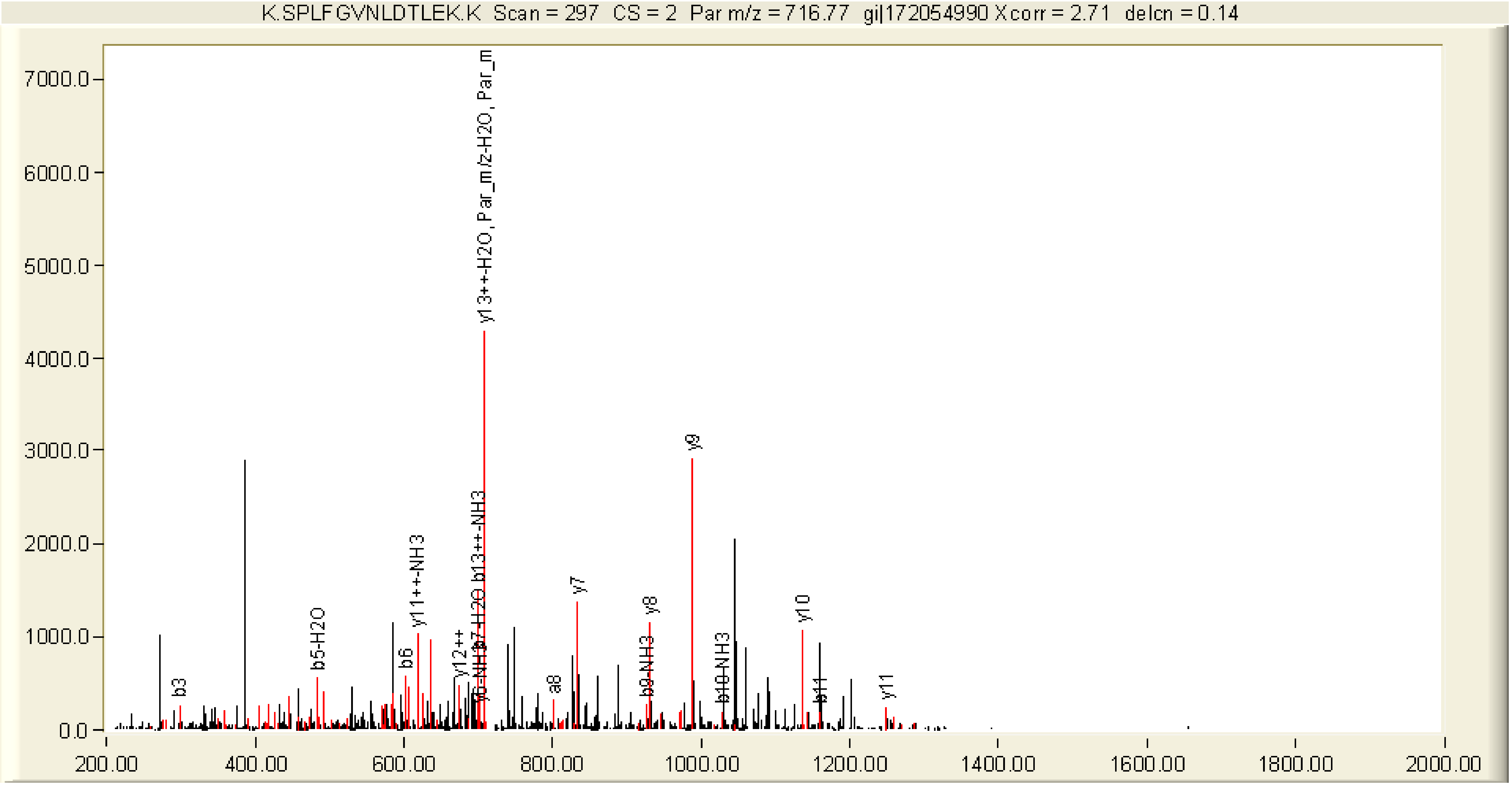

K.SPLFGVNLDTLEK.K Scan = 297 CS = 2 Par m/z = 716.77 gi|172054990 Xcorr = 2.71 delcn = 0.14
y13++-H2O, Par_m/z-H2O, Par_m
y9
y7
y8
y10
y11++-NH3
b13++-NH3
b5-H2O
b6
y12++
a8
b9-NH3
b10-NH3
b3
b11
y11
7000.0
6000.0
5000.0
4000.0
3000.0
2000.0
1000.0
0.0
200.00
400.00
600.00
800.00
1000.00
1200.00
1400.00
1600.00
1800.00
2000.00

R.LQVLPWEETAVQYYSK.I Scan = 299 CS = 2 Par m/z = 979.35 gi|172035497 Xcorr = 3.36 delcn = 0.35

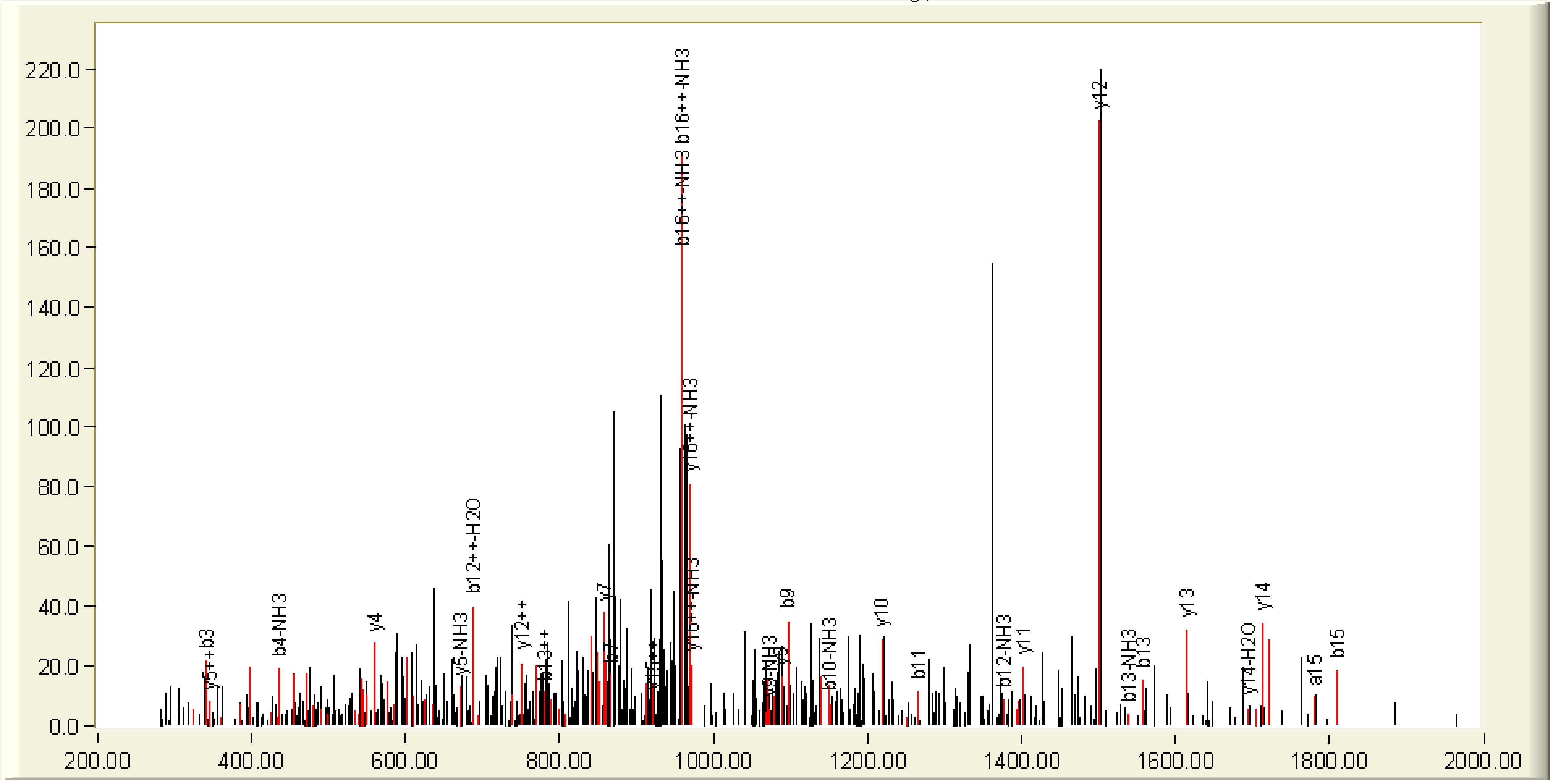

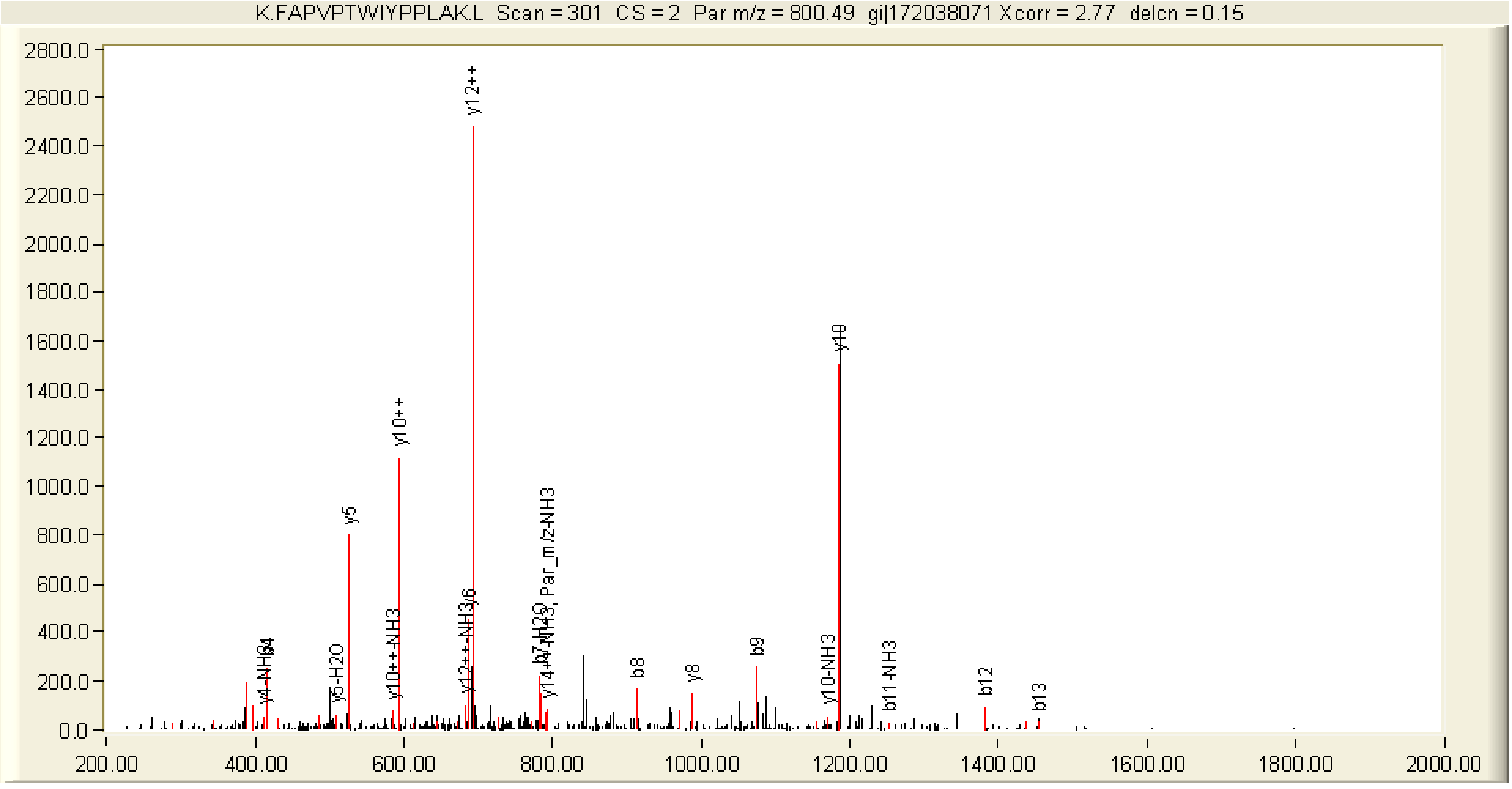

K.FAPVPTWIYPPLAK.L Scan = 301 CS = 2 Par m/z = 800.49 gi|172038071 Xcorr = 2.77 delcn = 0.15
y12++
y10
y10++
y5
y14++-NH3, Par_m/z-NH3
y5-H2O
y10++-NH3
y12++-NH3
b8
y8
b9
y10-NH3
b11-NH3
b12
b13
2800.0
2600.0
2400.0
2200.0
2000.0
1800.0
1600.0
1400.0
1200.0
1000.0
800.0
600.0
400.0
200.0
0.0
200.00
400.00
600.00
800.00
1000.00
1200.00
1400.00
1600.00
1800.00
2000.00

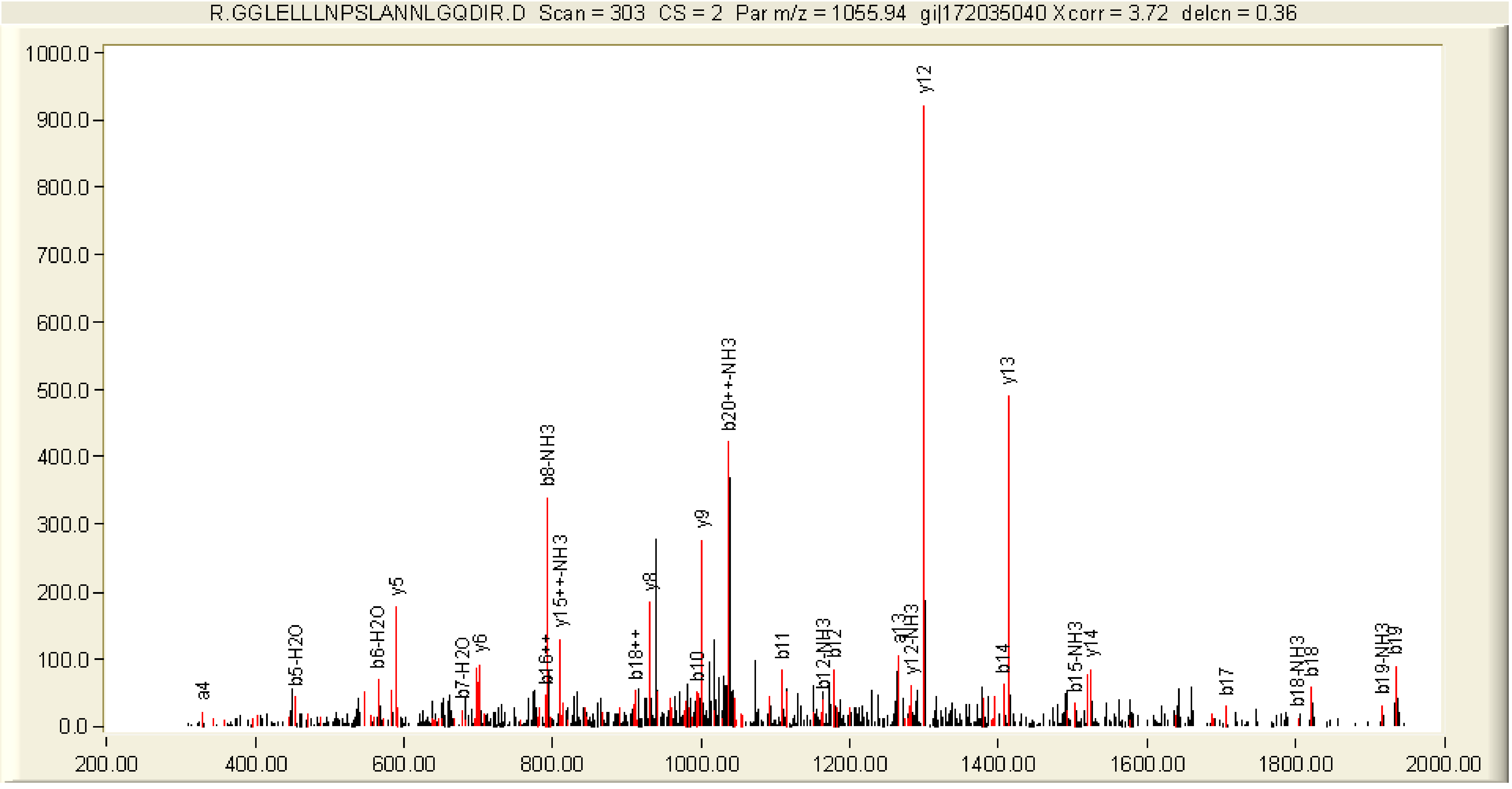
R.GGLELLLNPSLANNLGQDIR.D Scan = 303 CS = 2 Par m/z = 1055.94 gi|172035040 Xcorr = 3.72 delcn = 0.36
1000.0
900.0
800.0
700.0
600.0
500.0
400.0
300.0
200.0
100.0
0.0
200.00
400.00
600.00
800.00
1000.00
1200.00
1400.00
1600.00
1800.00
2000.00
a4
b5-H2O
b6-H2O
y5
b7-H2O
y6
b8-NH3
y15++-NH3
b18++
y8
b10
y9
b20++-NH3
b11
b12-NH3
b12
y12-NH3
y12
b14
y13
b15-NH3
y14
b17
b18-NH3
b18
b19-NH3
b19

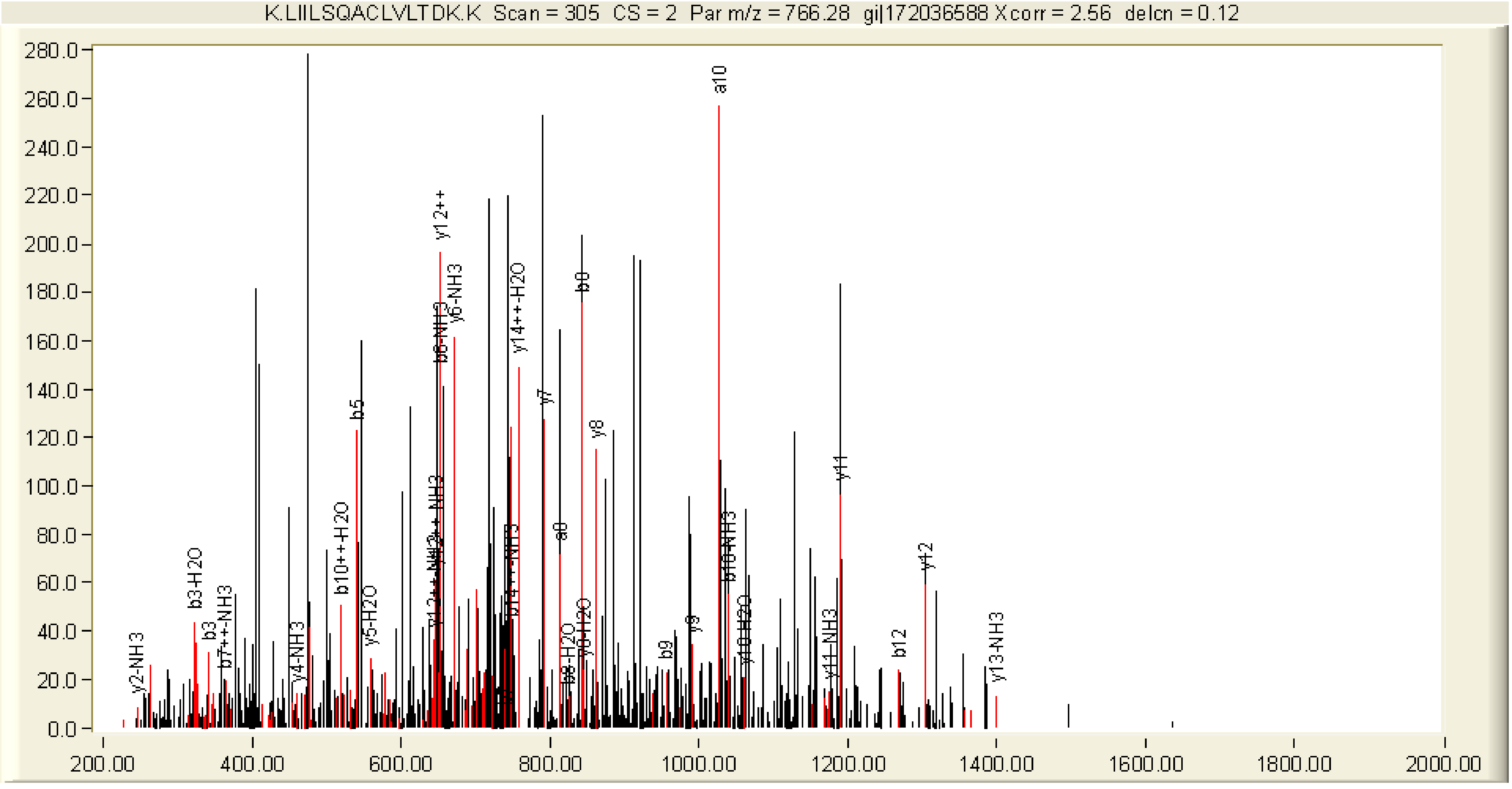
K.LIILSQACLVLTDK.K Scan = 305 CS = 2 Par m/z = 766.28 gi|172036588 Xcorr = 2.56 delcn = 0.12
280.0
260.0
240.0
220.0
200.0
180.0
160.0
140.0
120.0
100.0
80.0
60.0
40.0
20.0
0.0
200.00
400.00
600.00
800.00
1000.00
1200.00
1400.00
1600.00
1800.00
2000.00
y2-NH3
b3-H2O
b3
b7++-NH3
y4-NH3
b10++-H2O
b5
y5-H2O
y12++
y6-NH3
y14++-H2O
y7
a8
y8
b9
y9
a10
y11
b12
y12
y13-NH3

K.SFLLLNPLIPIIESIR.Q Scan = 307 CS = 2 Par m/z = 920.06 gi|172036147 Xcorr = 2.49 delcn = 0.19

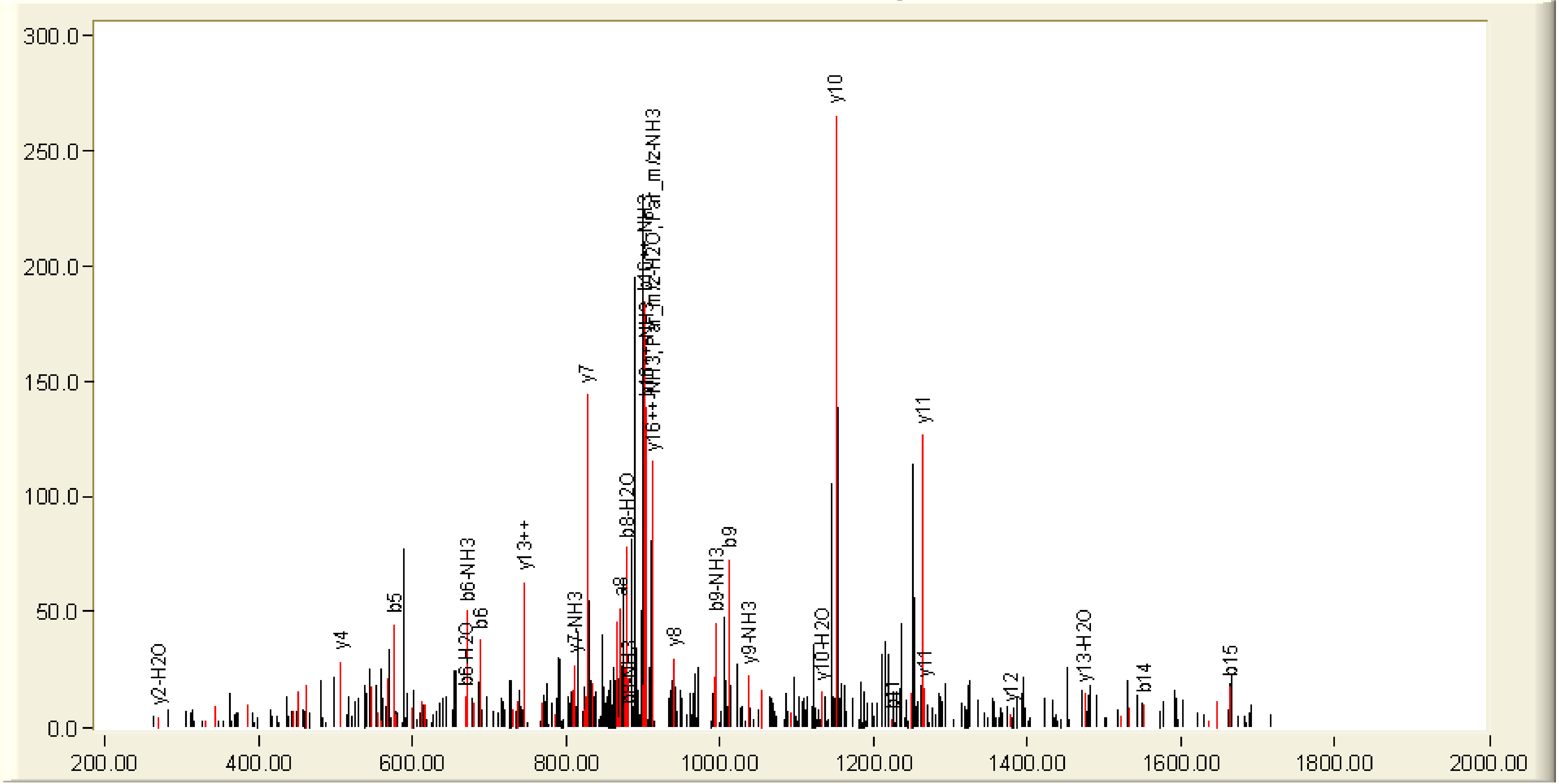

KIPNNTYYQTVVK.D Scan = 309 CS = 2 Par m/z = 721.56 gi|172035749 Xcorr = 3.06 delcn = 0.46

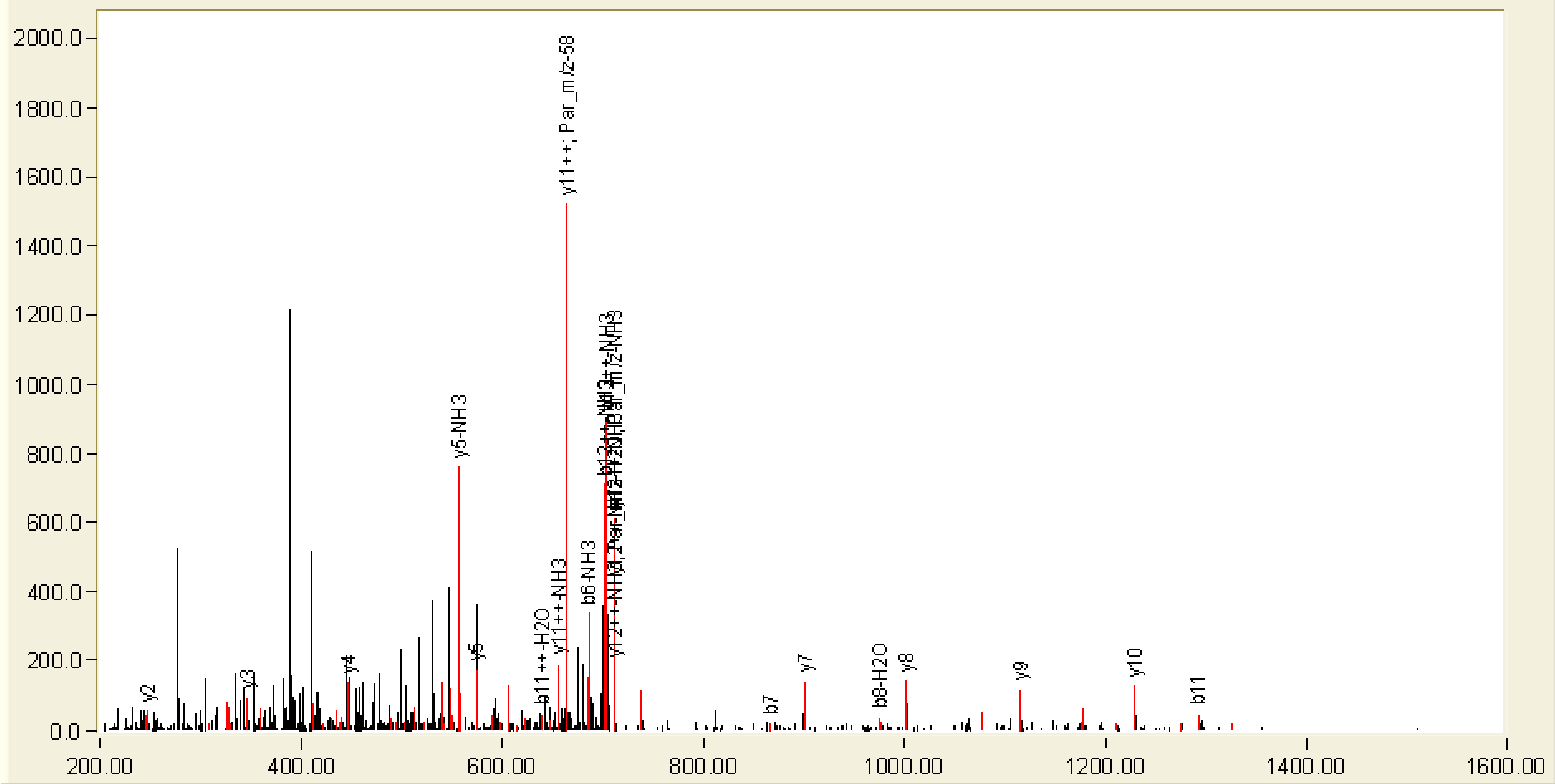

K.LLSPAQTQAYMASK.G Scan = 311 CS = 2 Par m/z = 755.4 gi|172037631 Xcorr = 2.89 delcn = 0.46

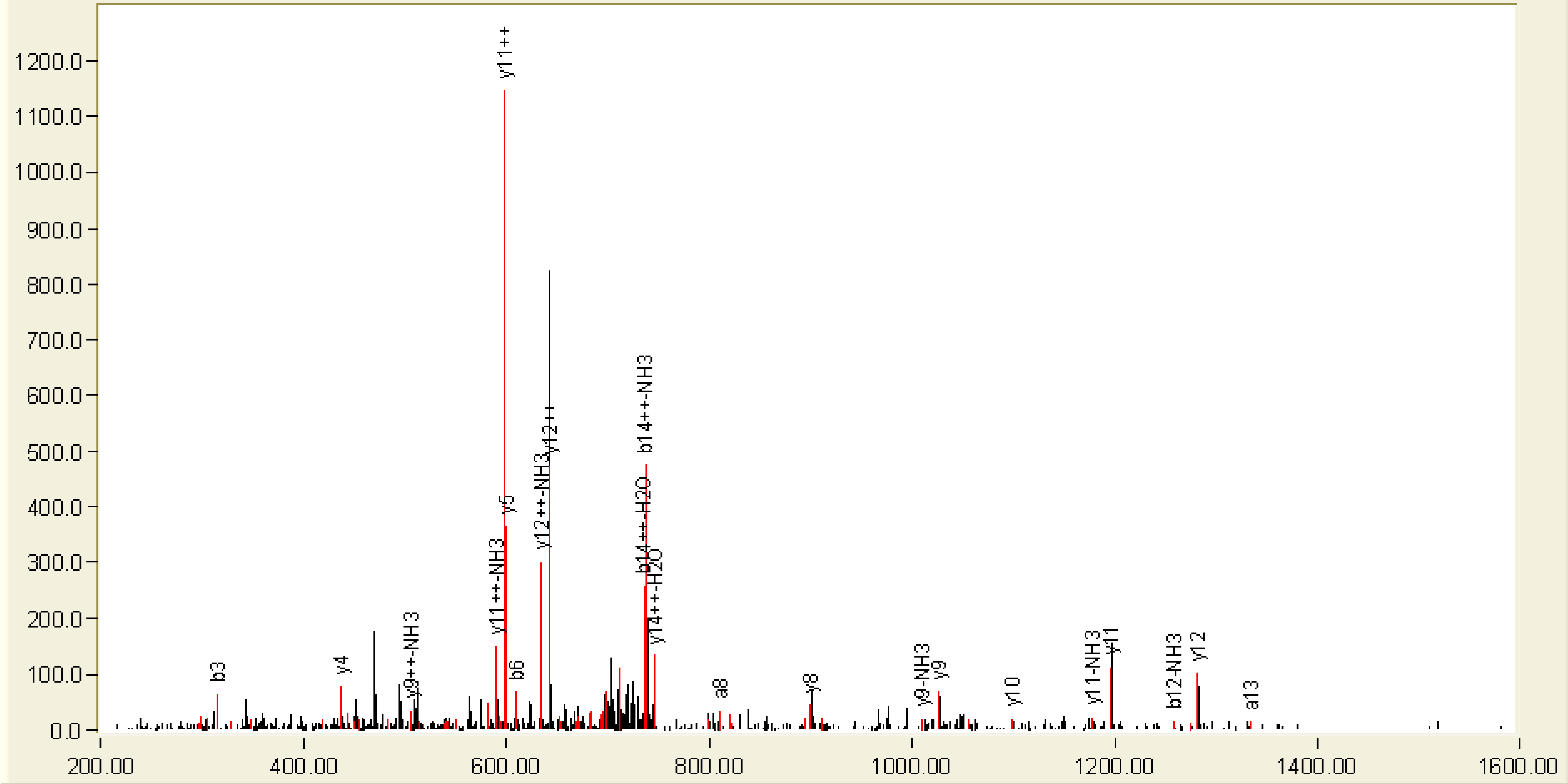

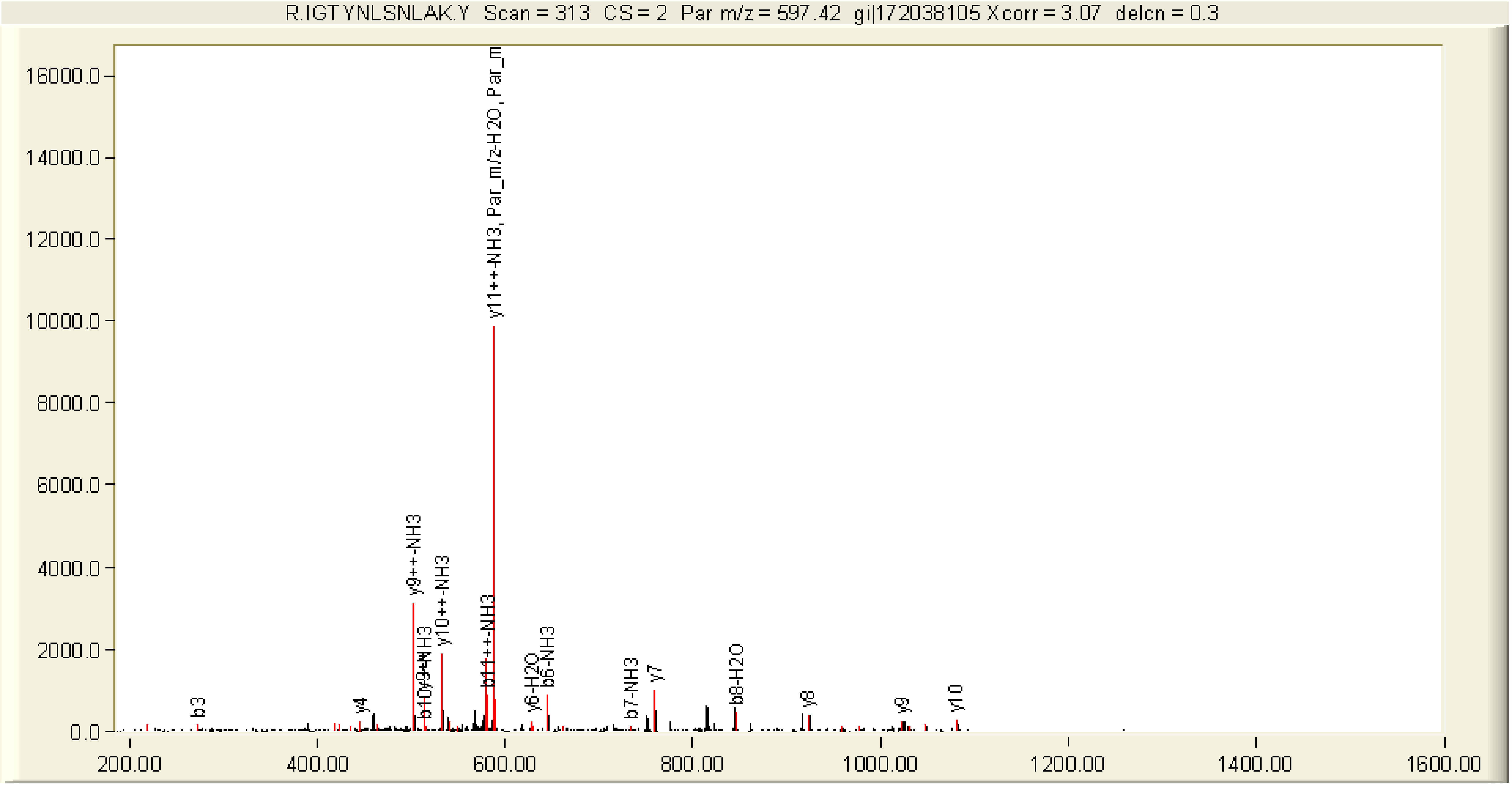
R.IGTYNLSNLAK.Y Scan = 313 CS = 2 Par m/z = 597.42 gi|172038105 Xcorr = 3.07 delcn = 0.3
16000.0
14000.0
12000.0
10000.0
8000.0
6000.0
4000.0
2000.0
0.0
200.00
400.00
600.00
800.00
1000.00
1200.00
1400.00
1600.00
y11++-NH3, Par_m/z-H2O, Par_m
y9++-NH3
y10++-NH3
b11++-NH3
y6-H2O
b6-NH3
b7-NH3
y7
b8-H2O
y8
y9
y10
b3
y4

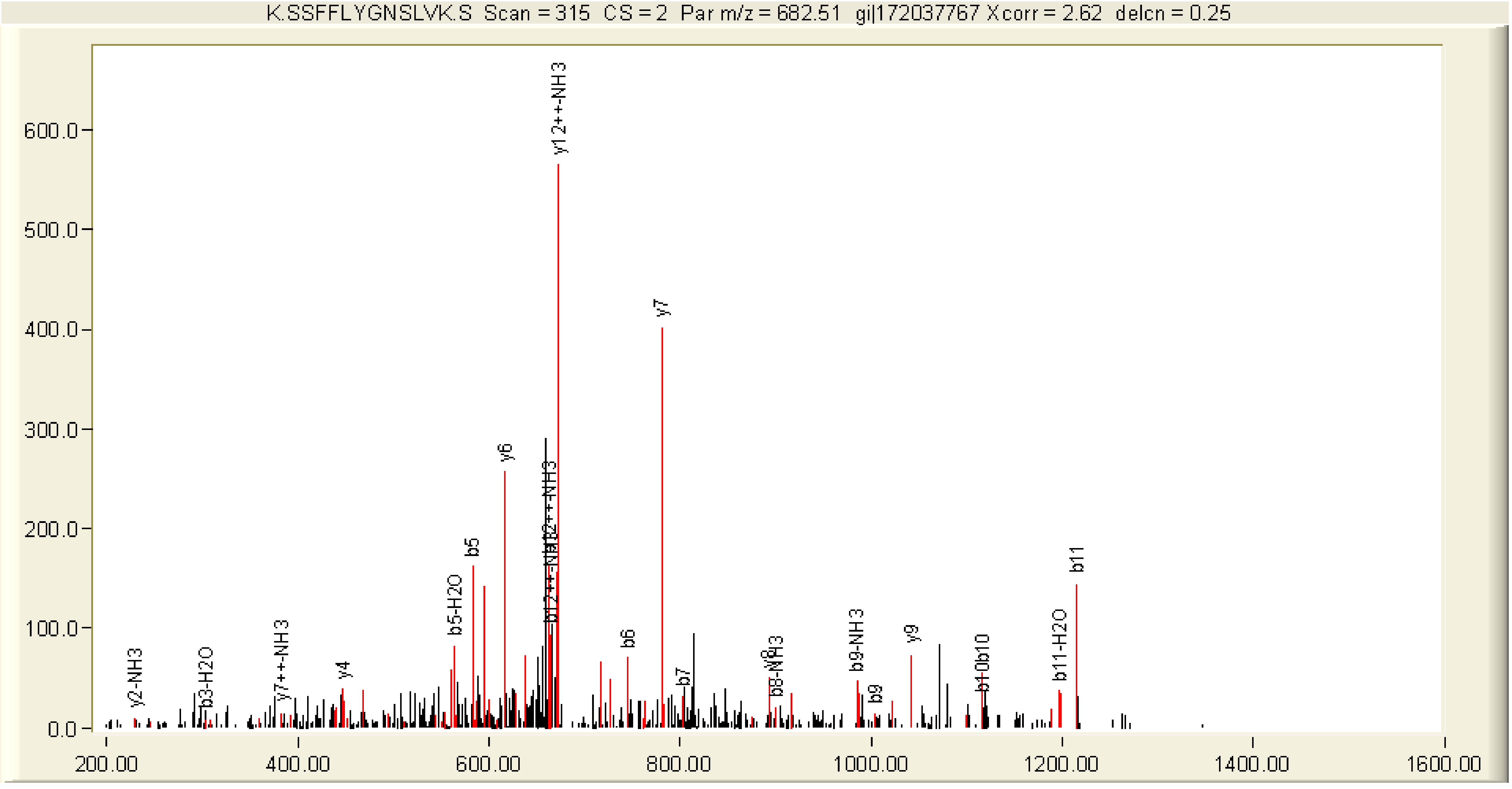
K.SSFFLYGNSLVK.S Scan = 315 CS = 2 Par m/z = 682.51 gi|172037767 Xcorr = 2.62 delcn = 0.25
y12++-NH3
y7
y6
b5
b5-H2O
y2-NH3
b3-H2O
y7++-NH3
y4
b6
b7
b8-NH3
b9-NH3
b9
y9
b10
b11-H2O
b11
0.0
100.0
200.0
300.0
400.0
500.0
600.0
200.00
400.00
600.00
800.00
1000.00
1200.00
1400.00
1600.00

K.LICGASFQDLPAIR.H Scan = 317 CS = 2 Par m/z = 754.3 gi|172037265 Xcorr = 2.91 delcn = 0.14

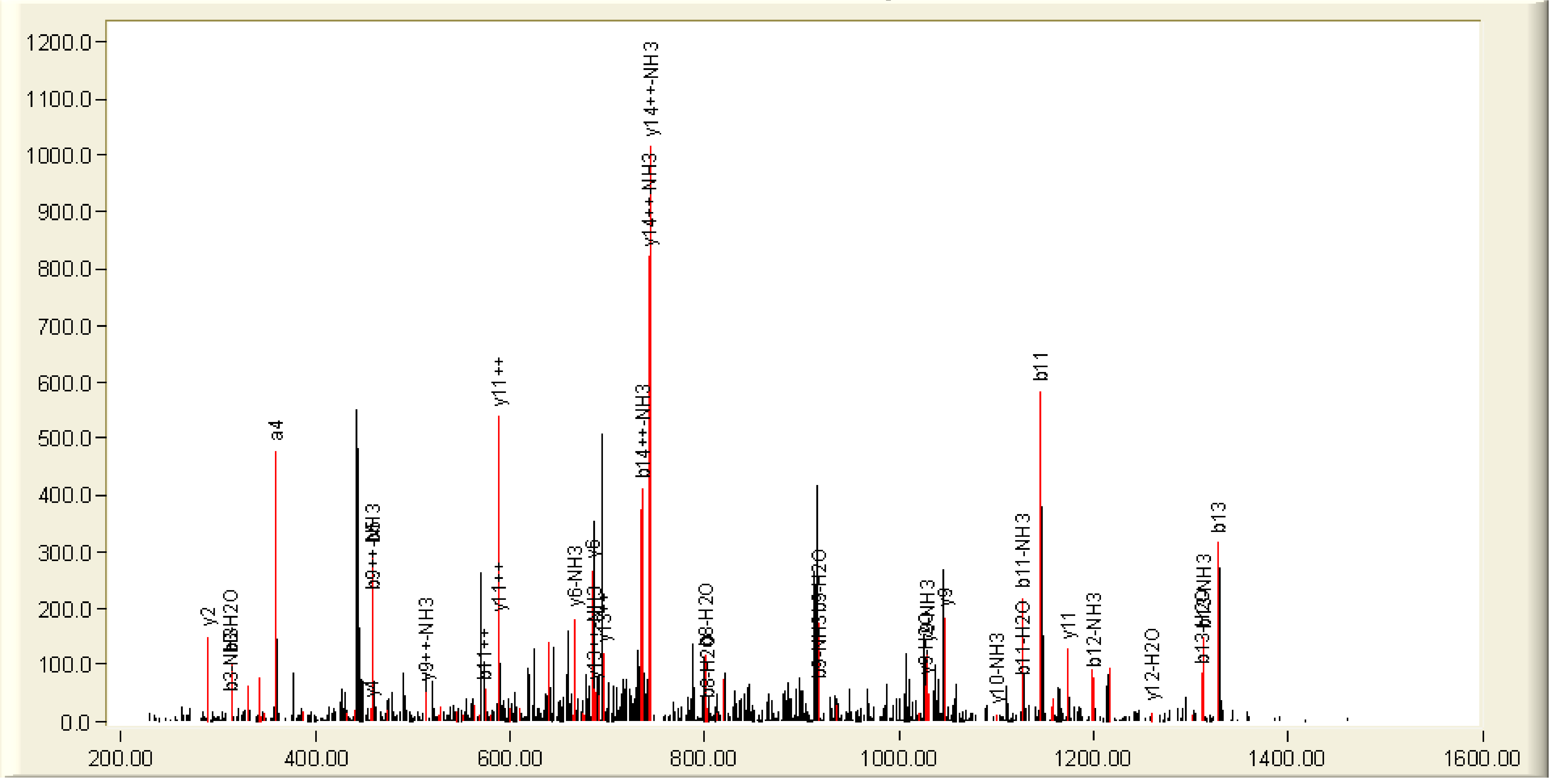

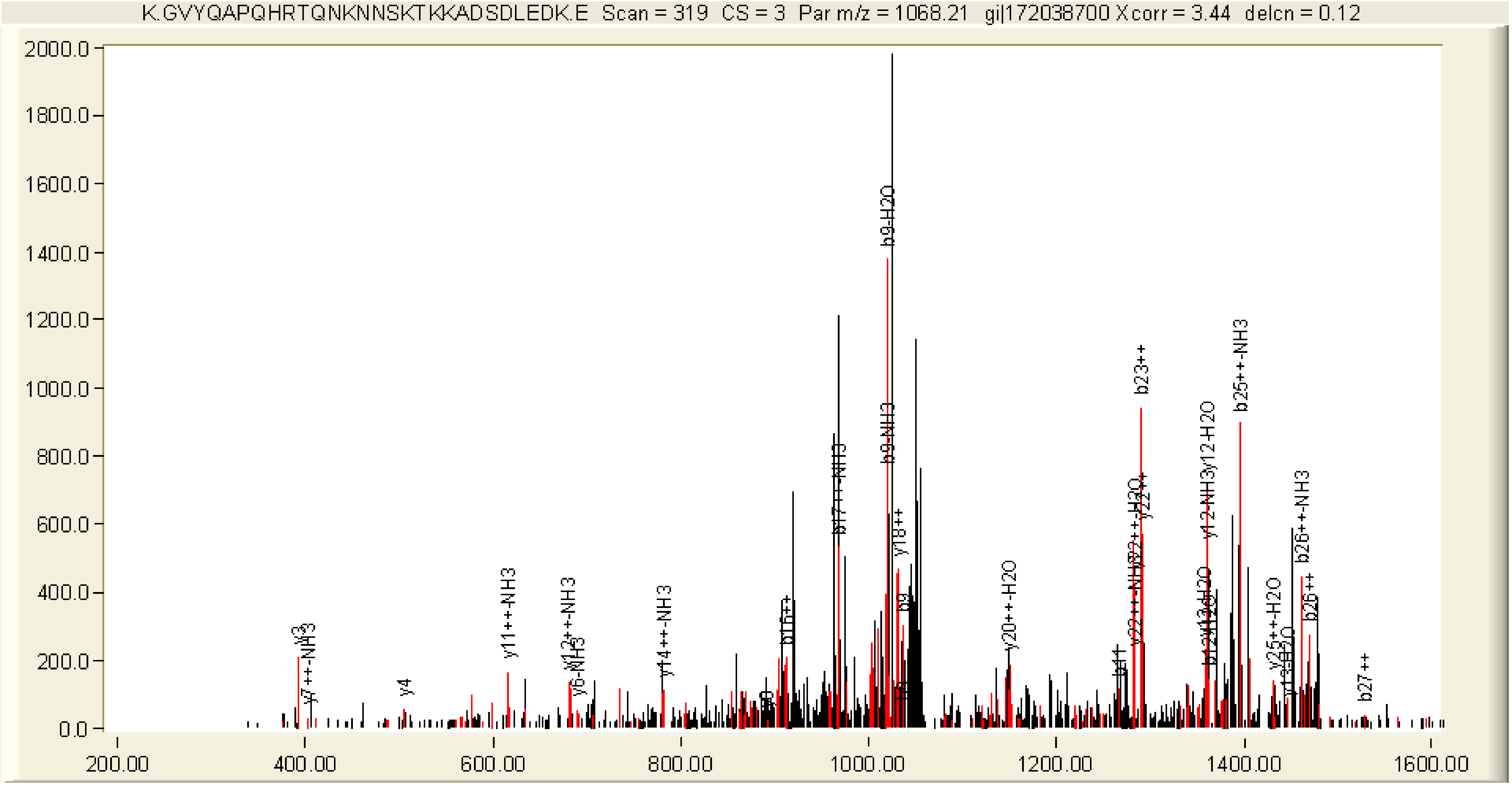

K.GVYQAPQHRTQNKNNSKTKKADSDLEDK.E Scan = 319 CS = 3 Par m/z = 1068.21 gi|172038700 Xcorr = 3.44 delcn = 0.12
2000.0
1800.0
1600.0
1400.0
1200.0
1000.0
800.0
600.0
400.0
200.0
0.0
200.00
400.00
600.00
800.00
1000.00
1200.00
1400.00
1600.00
y7++-NH3
y4
y11++-NH3
y14++-NH3
b9-H2O
b9-NH3
y18++
y20++-H2O
b23++
b25++-NH3
b26++-NH3
y25++-H2O
b27++

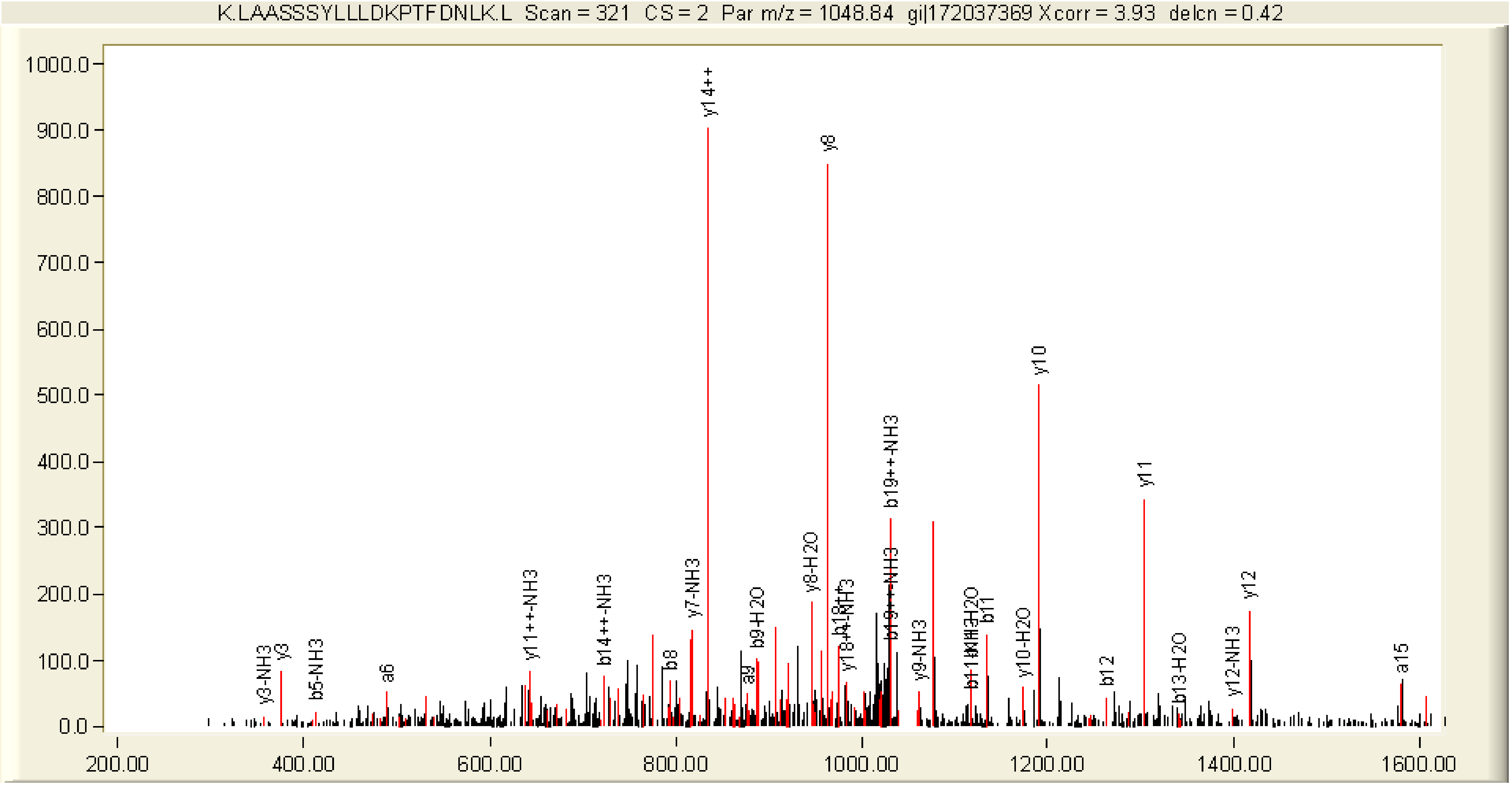
K.LAASSSYLLLDKPTFDNLK.L Scan = 321 CS = 2 Par m/z = 1048.84 gi|172037369 Xcorr = 3.93 delcn = 0.42
1000.0
900.0
800.0
700.0
600.0
500.0
400.0
300.0
200.0
100.0
0.0
200.00
400.00
600.00
800.00
1000.00
1200.00
1400.00
1600.00
y14++
y8
y10
b19++-NH3
y11
y8-H2O
y7-NH3
y11++-NH3
b14++-NH3
b9-H2O
y12
b11
y10-H2O
y9-NH3
b13-H2O
y12-NH3
b12
a15
y3-NH3
y3
b5-NH3
a6
b8
a9

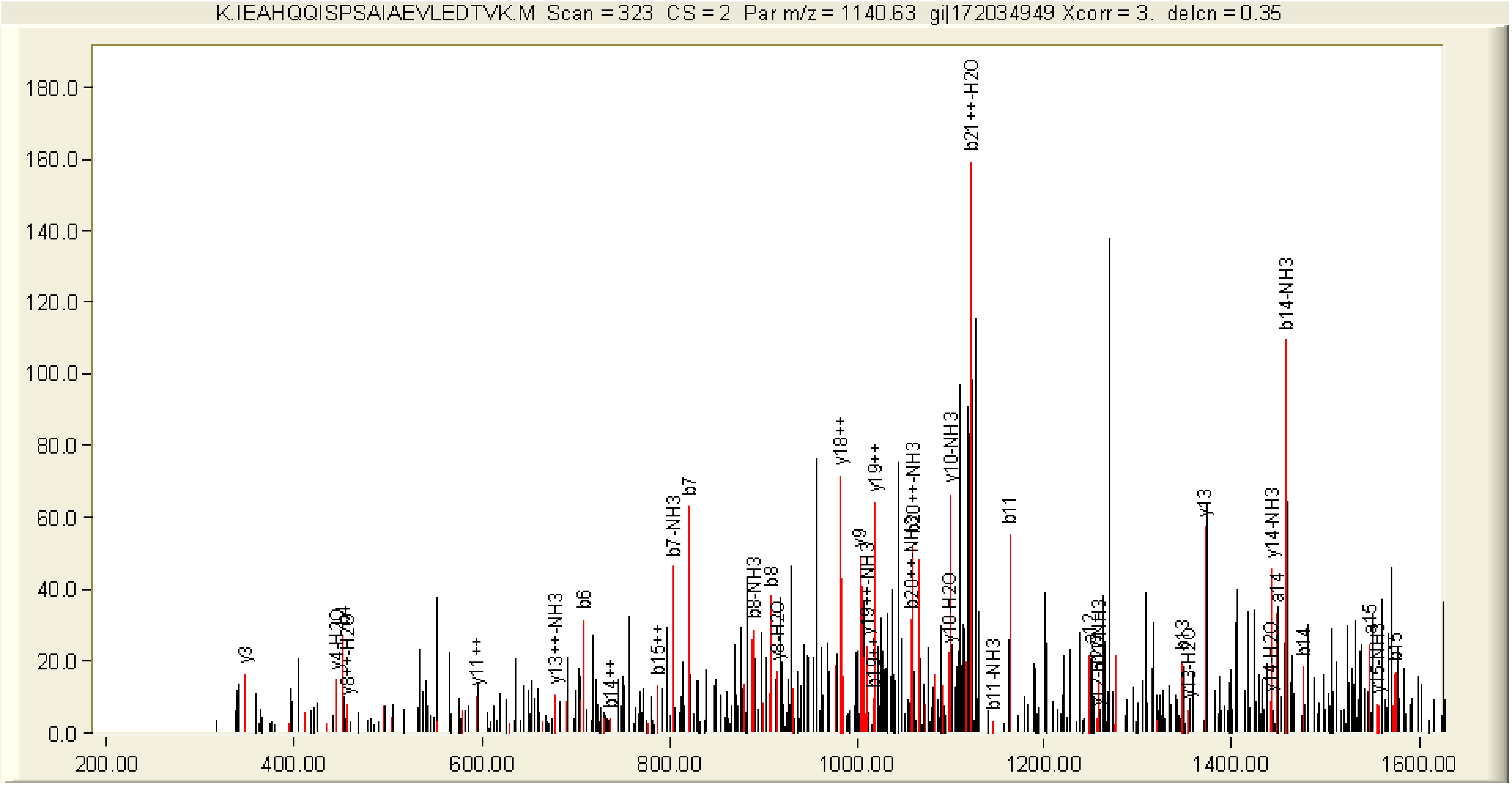
K.IEAHQQISPSAIAEVLEDTVK.M Scan = 323 CS = 2 Par m/z = 1140.63 gi|172034949 Xcorr = 3. delcn = 0.35
180.0
160.0
140.0
120.0
100.0
80.0
60.0
40.0
20.0
0.0
200.00
400.00
600.00
800.00
1000.00
1200.00
1400.00
1600.00
b21++-H2O
b14-NH3
y18++
y19++
b20++-NH3
y10-NH3
b7
b7-NH3
b11
y13
y14-NH3
a14
b8
b8-NH3
b6
y13++-NH3
b15++
b14++
y11++
y3
b11-NH3
y10-H2O
b14

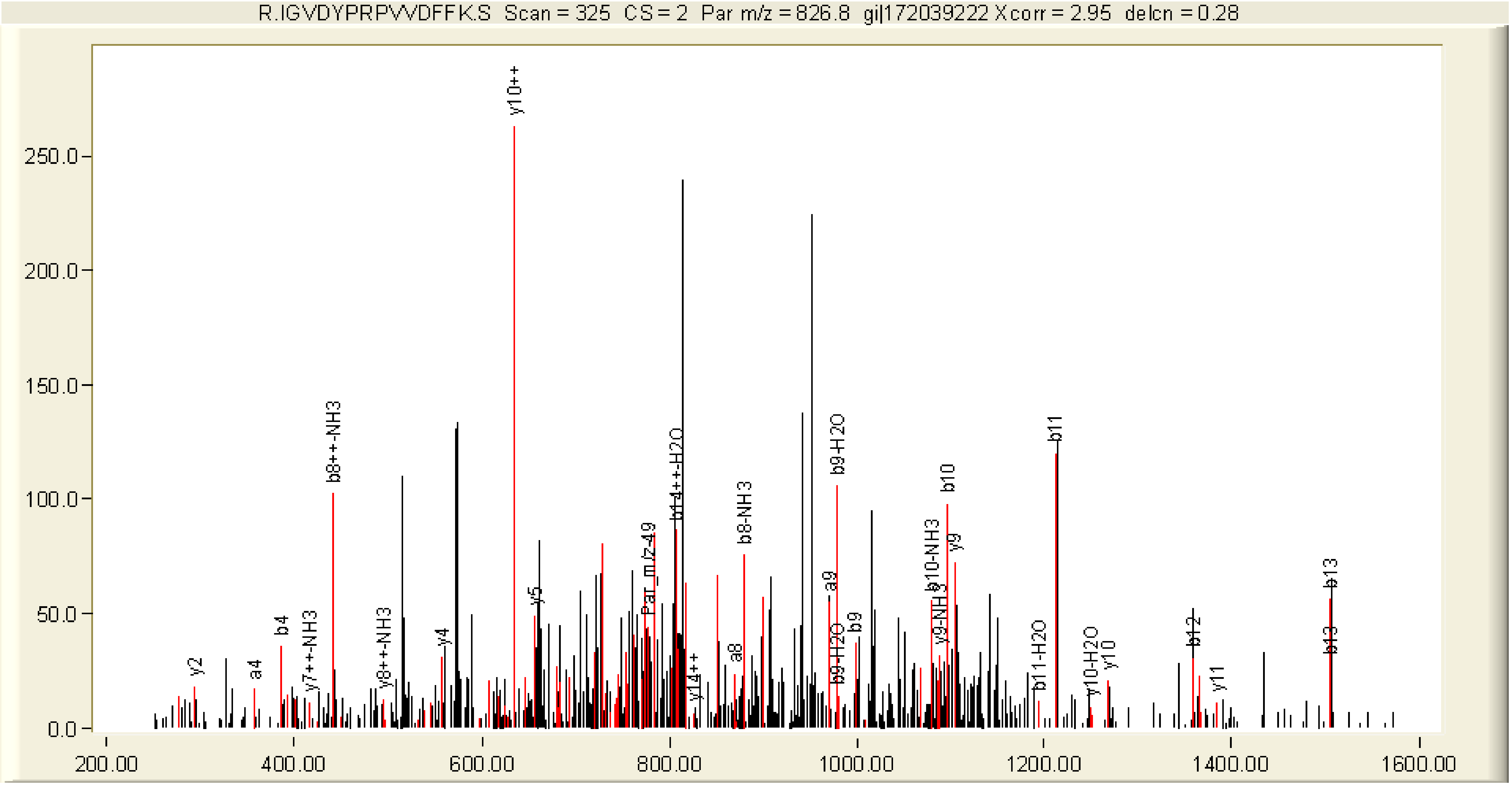

R.IGVDYPRPVVDFFK.S Scan = 325 CS = 2 Par m/z = 826.8 gi|172039222 Xcorr = 2.95 delcn = 0.28
y10++
b8++-NH3
b11
b9-H2O
b10
b11++-H2O
b8-NH3
Par_m/z-49
y9
b10-NH3
b13
a9
b10-NH3
y9-NH3
y5
b12
b4
b9
b11-H2O
y10-H2O
y10
b13
y7++-NH3
y8++-NH3
y4
y2
a4
a8
b9-H2O
y14++
y11
250.0
200.0
150.0
100.0
50.0
0.0
200.00
400.00
600.00
800.00
1000.00
1200.00
1400.00
1600.00

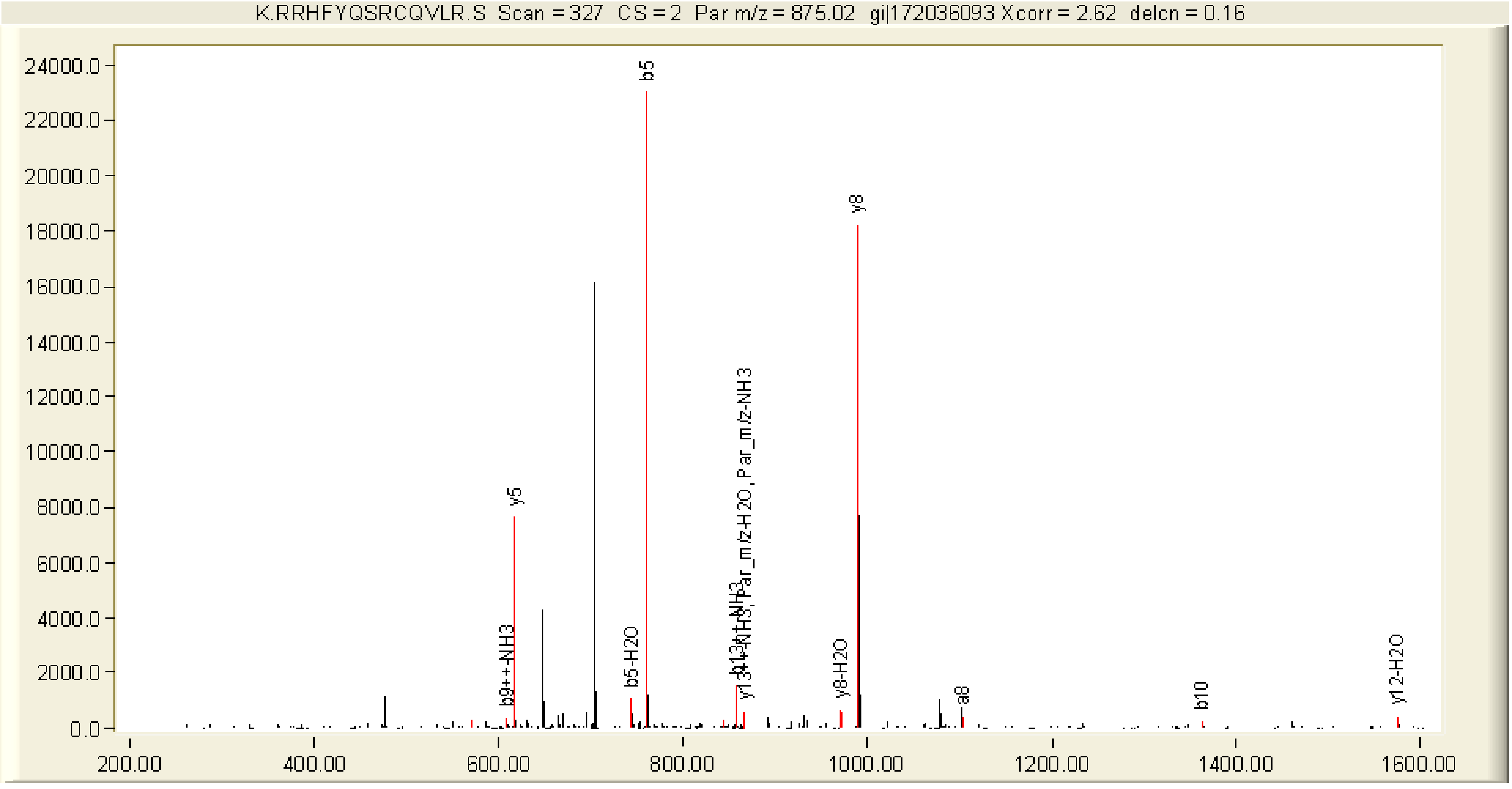

K.RRHFYQSRCQVLR.S Scan = 327 CS = 2 Par m/z = 875.02 gi|172036093 Xcorr = 2.62 delcn = 0.16
24000.0
22000.0
20000.0
18000.0
16000.0
14000.0
12000.0
10000.0
8000.0
6000.0
4000.0
2000.0
0.0
200.00
400.00
600.00
800.00
1000.00
1200.00
1400.00
1600.00
b5
y8
y5
b9++-NH3
b5-H2O
Par_m/z-H2O, Par_m/z-NH3
y8-H2O
a8
b10
y12-H2O

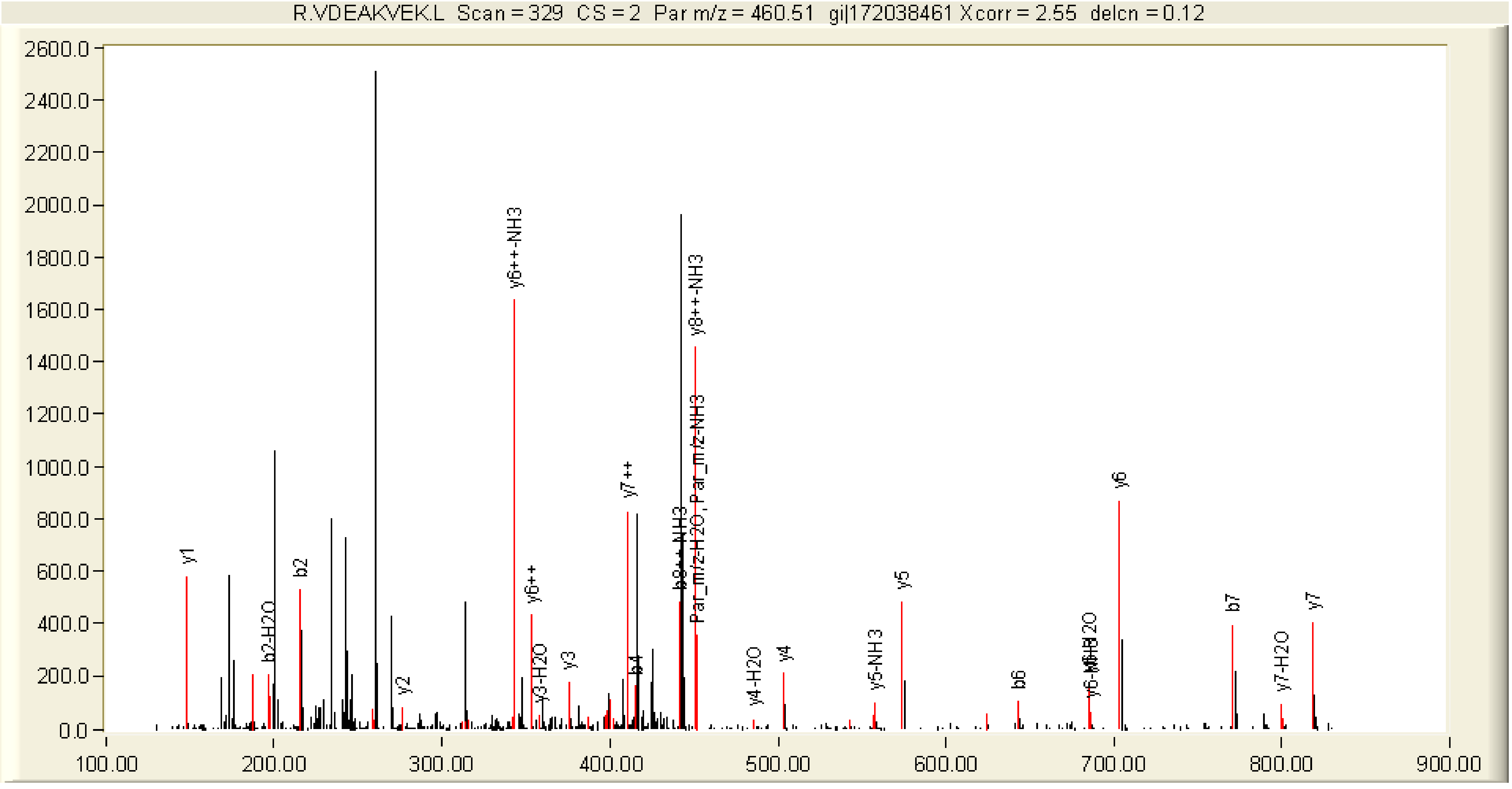
R.VDEAKVEK.L Scan = 329 CS = 2 Par m/z = 460.51 gi|172038461 Xcorr = 2.55 delcn = 0.12
2600.0
2400.0
2200.0
2000.0
1800.0
1600.0
1400.0
1200.0
1000.0
800.0
600.0
400.0
200.0
0.0
100.00
200.00
300.00
400.00
500.00
600.00
700.00
800.00
900.00
y1
b2-H2O
b2
y2
y6++-NH3
y6++
y3-H2O
y3
y7++
b4
y8++-NH3
Par_m/z-H2O, Par_m/z-NH3
y4-H2O
y4
y5-NH3
y5
b6
y6
b7
y7-H2O
y7

K.ALEKQEFNDDIIR.N Scan = 331 CS = 2 Par m/z = 796.32 gi|172036085 Xcorr = 3.02 delcn = 0.36

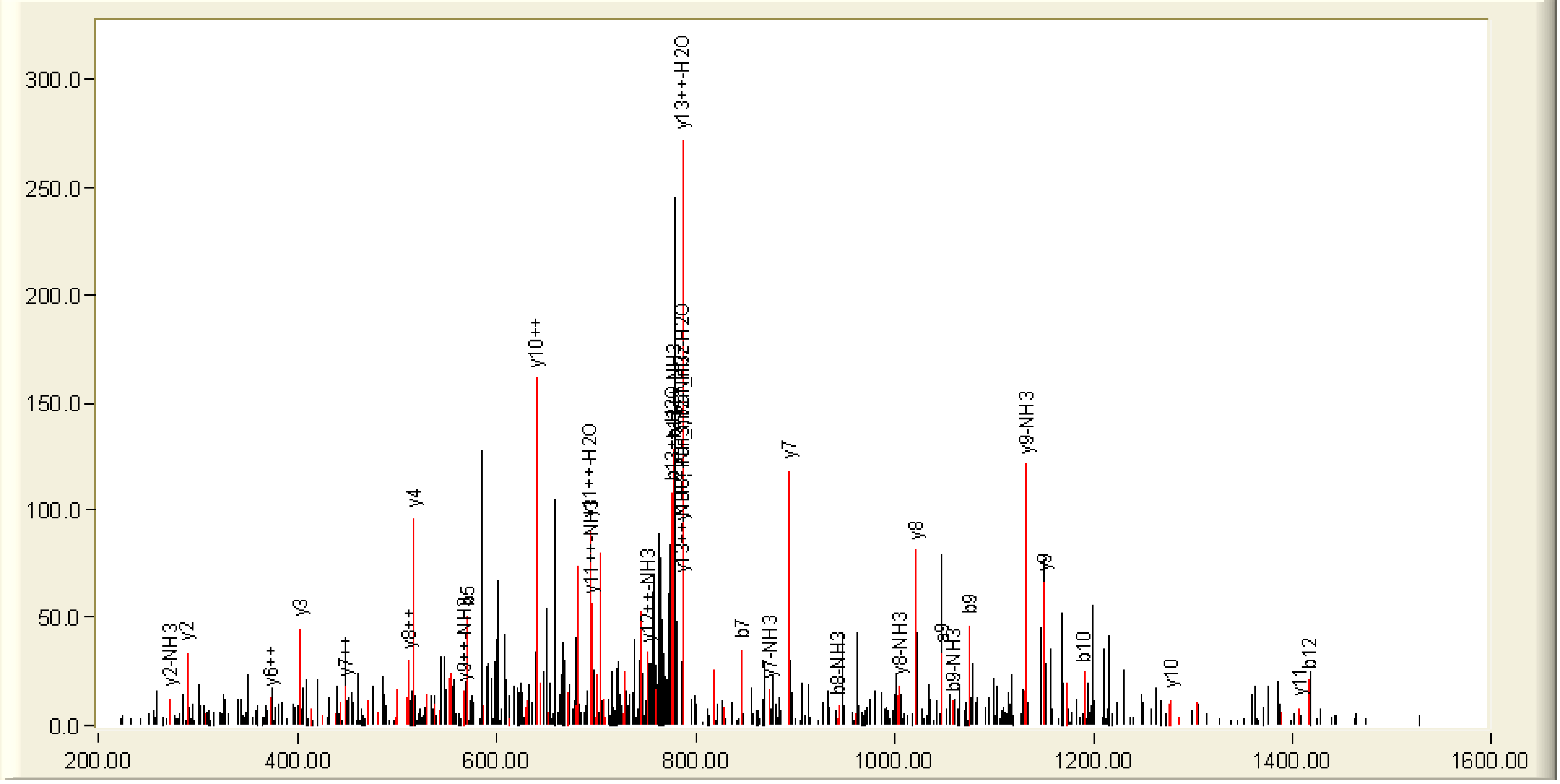

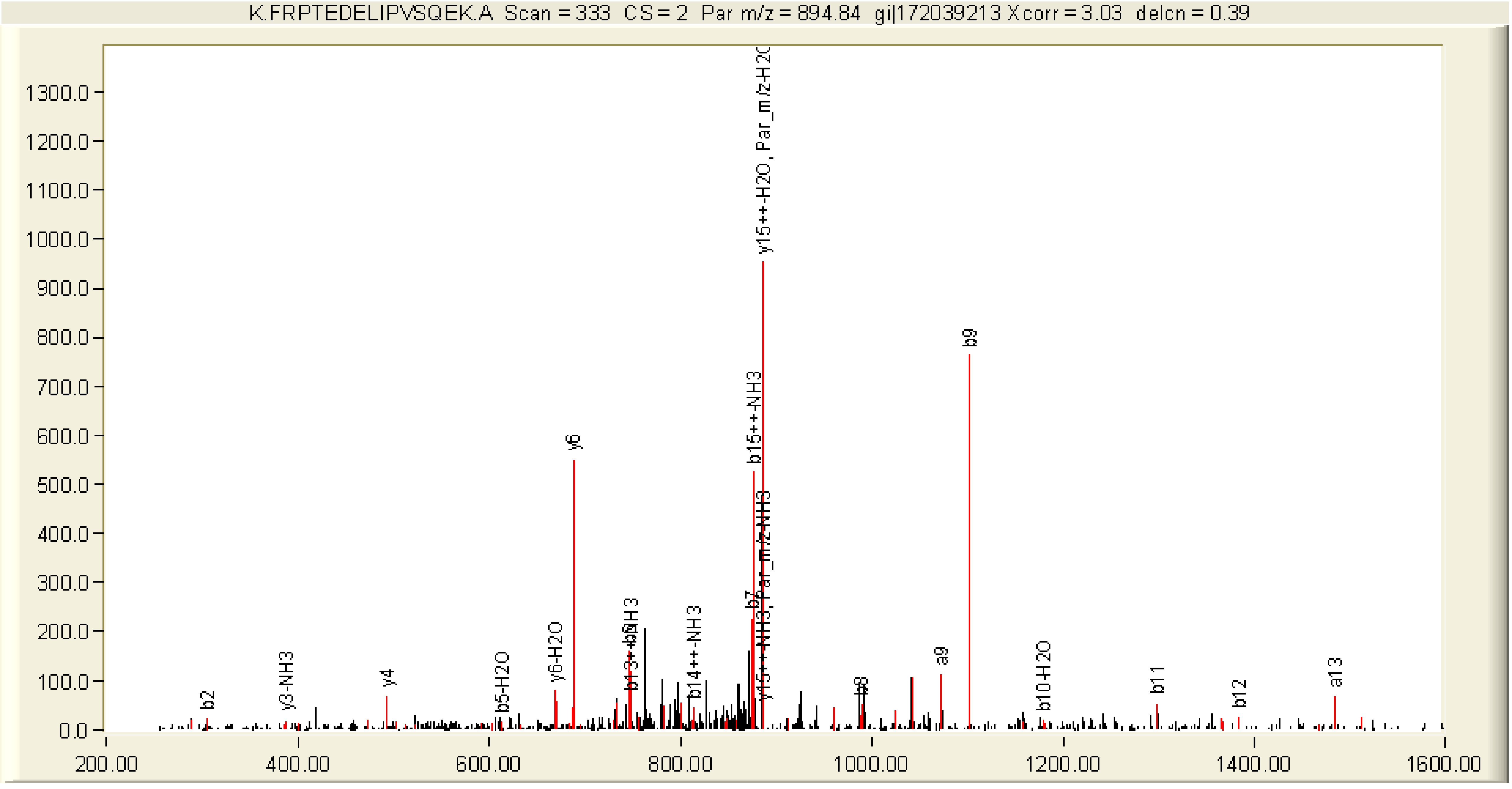
K.FRPTEDELIPVSQEK.A Scan = 333 CS = 2 Par m/z = 894.84 gi|172039213 Xcorr = 3.03 delcn = 0.39
y15++-H2O, Par_m/z-H2O
b9
y6
b15++-NH3
y15++-NH3, Par_m/z-NH3
b7
b14++-NH3
y6-H2O
b5-H2O
y4
y3-NH3
b2
a9
b8
b10-H2O
b11
b12
a13
0.0
100.0
200.0
300.0
400.0
500.0
600.0
700.0
800.0
900.0
1000.0
1100.0
1200.0
1300.0
200.00
400.00
600.00
800.00
1000.00
1200.00
1400.00
1600.00

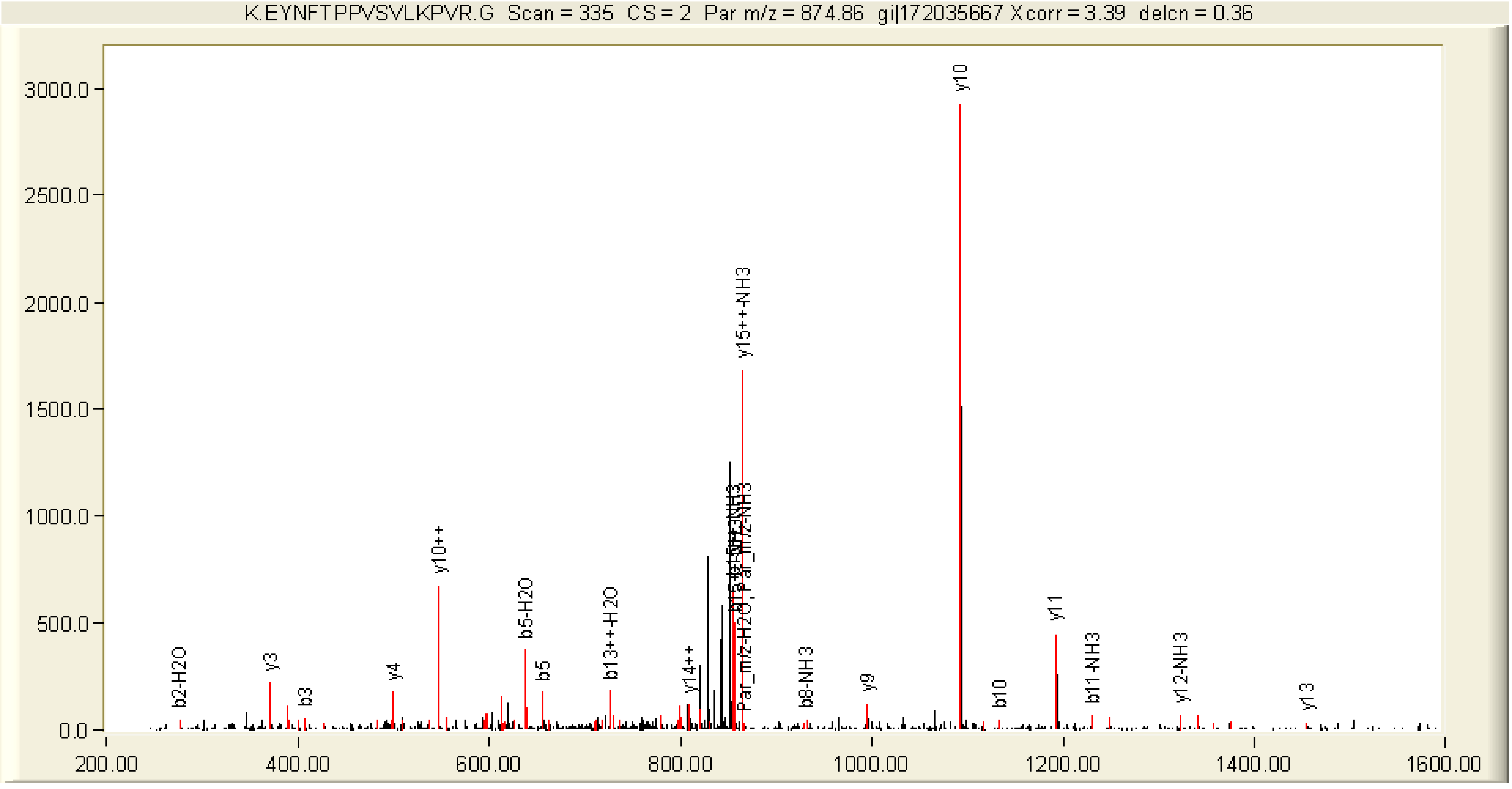
K.EYNFTPPVSVLKPVR.G Scan = 335 CS = 2 Par m/z = 874.86 gi|172035667 Xcorr = 3.39 delcn = 0.36
3000.0
2500.0
2000.0
1500.0
1000.0
500.0
0.0
200.00
400.00
600.00
800.00
1000.00
1200.00
1400.00
1600.00
y10
y15++-NH3
y10++
b5-H2O
b13++-H2O
y11
b11-NH3
y12-NH3
b2-H2O
y3
b3
y4
b5
y14++
b8-NH3
y9
b10
y13

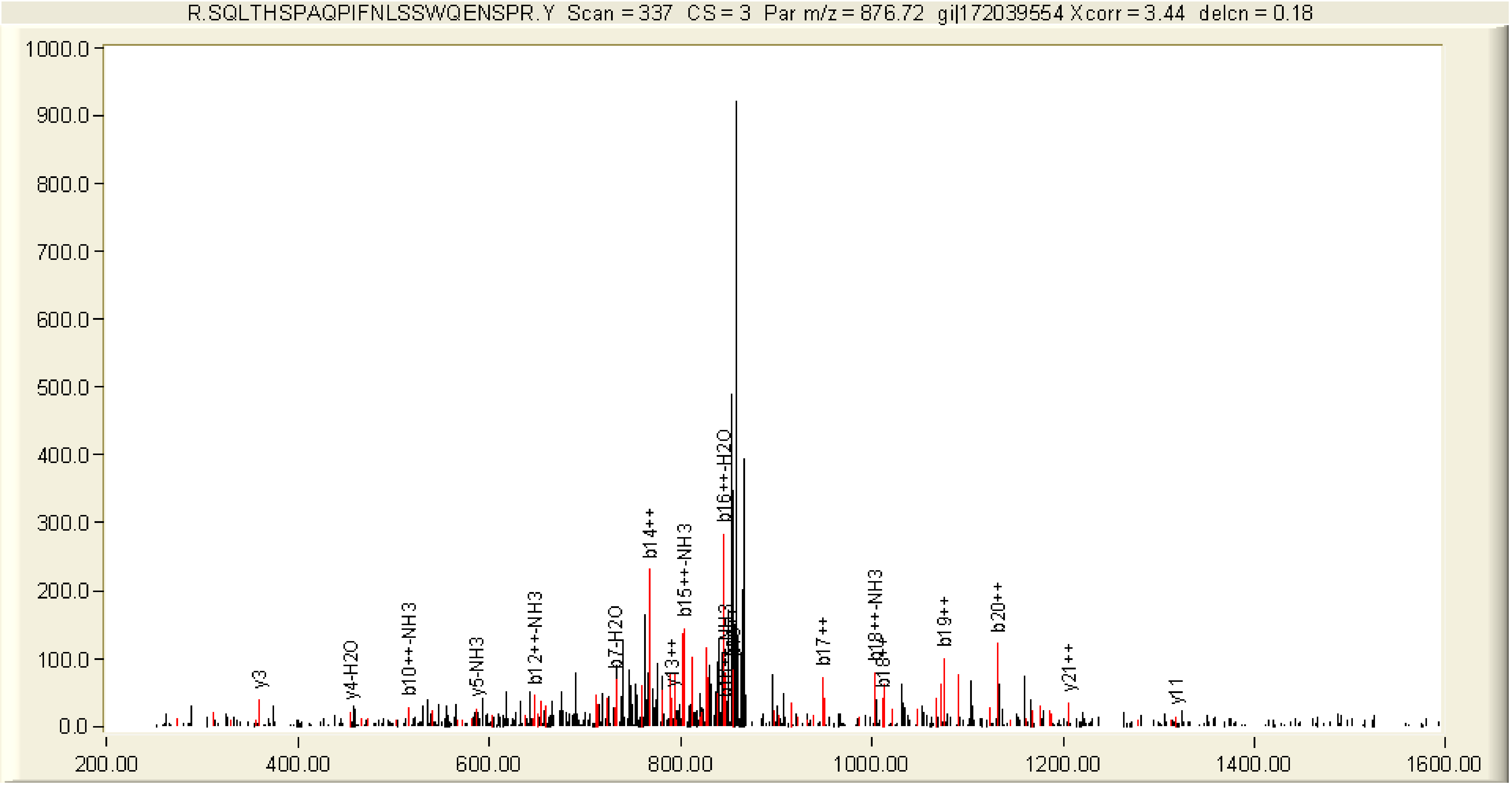

R.SQLTHSPAQPIFNLSSWQENSPR.Y Scan = 337 CS = 3 Par m/z = 876.72 gi|172039554 Xcorr = 3.44 delcn = 0.18
1000.0
900.0
800.0
700.0
600.0
500.0
400.0
300.0
200.0
100.0
0.0
y3
y4-H2O
b10++-NH3
y5-NH3
b12++-NH3
b7-H2O
b14++
y13++
b15++-NH3
b16++-H2O
b17++
b18++-NH3
b19++
b20++
y21++
y11
200.00
400.00
600.00
800.00
1000.00
1200.00
1400.00
1600.00

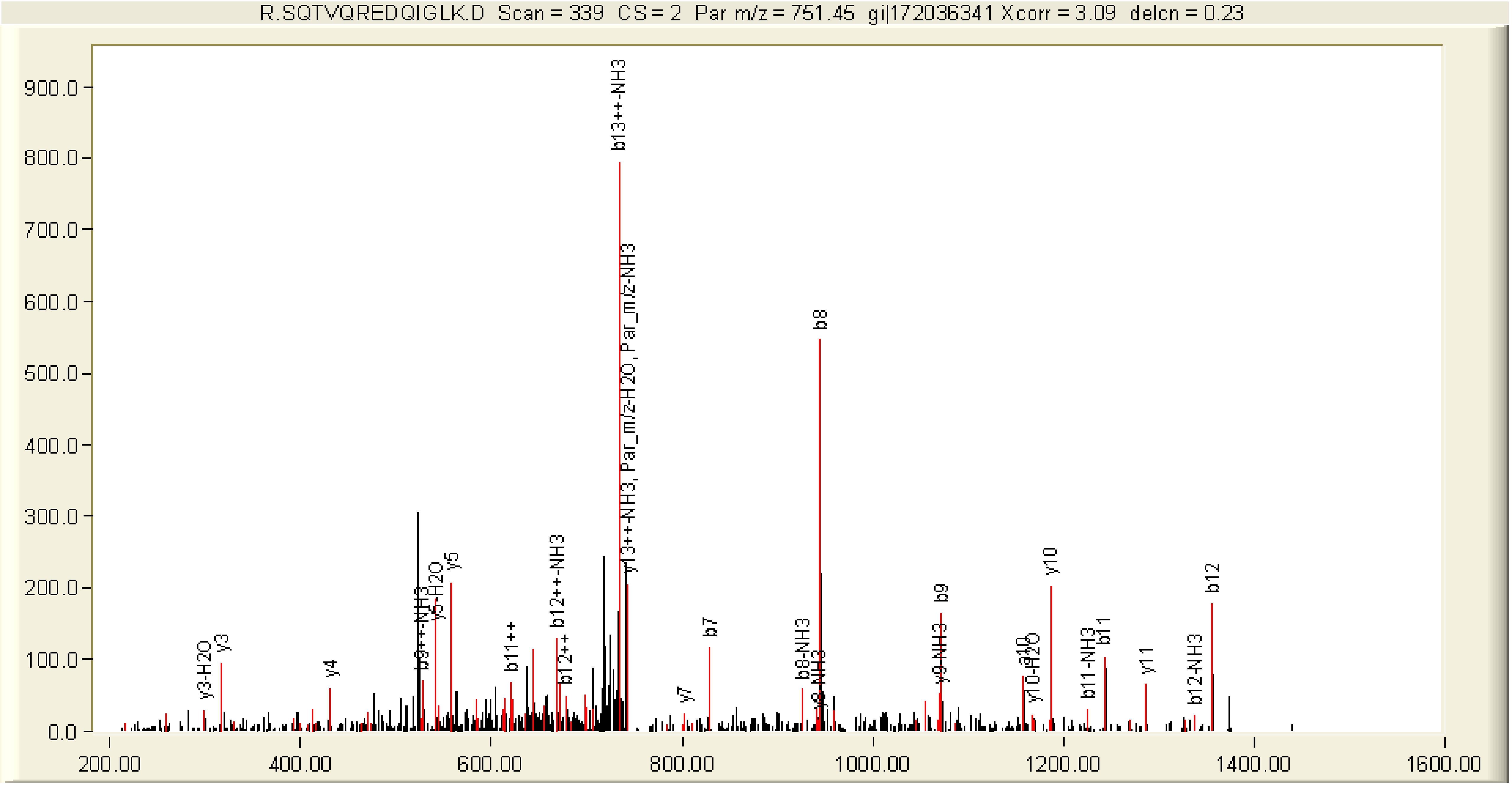
R.SQTVQREDQIGLK.D Scan = 339 CS = 2 Par m/z = 751.45 gi|172036341 Xcorr = 3.09 delcn = 0.23
900.0
800.0
700.0
600.0
500.0
400.0
300.0
200.0
100.0
0.0
200.00
400.00
600.00
800.00
1000.00
1200.00
1400.00
1600.00
b13++-NH3
y13++-NH3, Par_m/z-H2O, Par_m/z-NH3
b8
y3-H2O
y3
y4
y5-H2O
y5
b11++
b12++-NH3
b12++
y7
b7
b8-NH3
b9
y9-NH3
y10-H2O
y10
b11-NH3
b11
y11
b12-NH3
b12

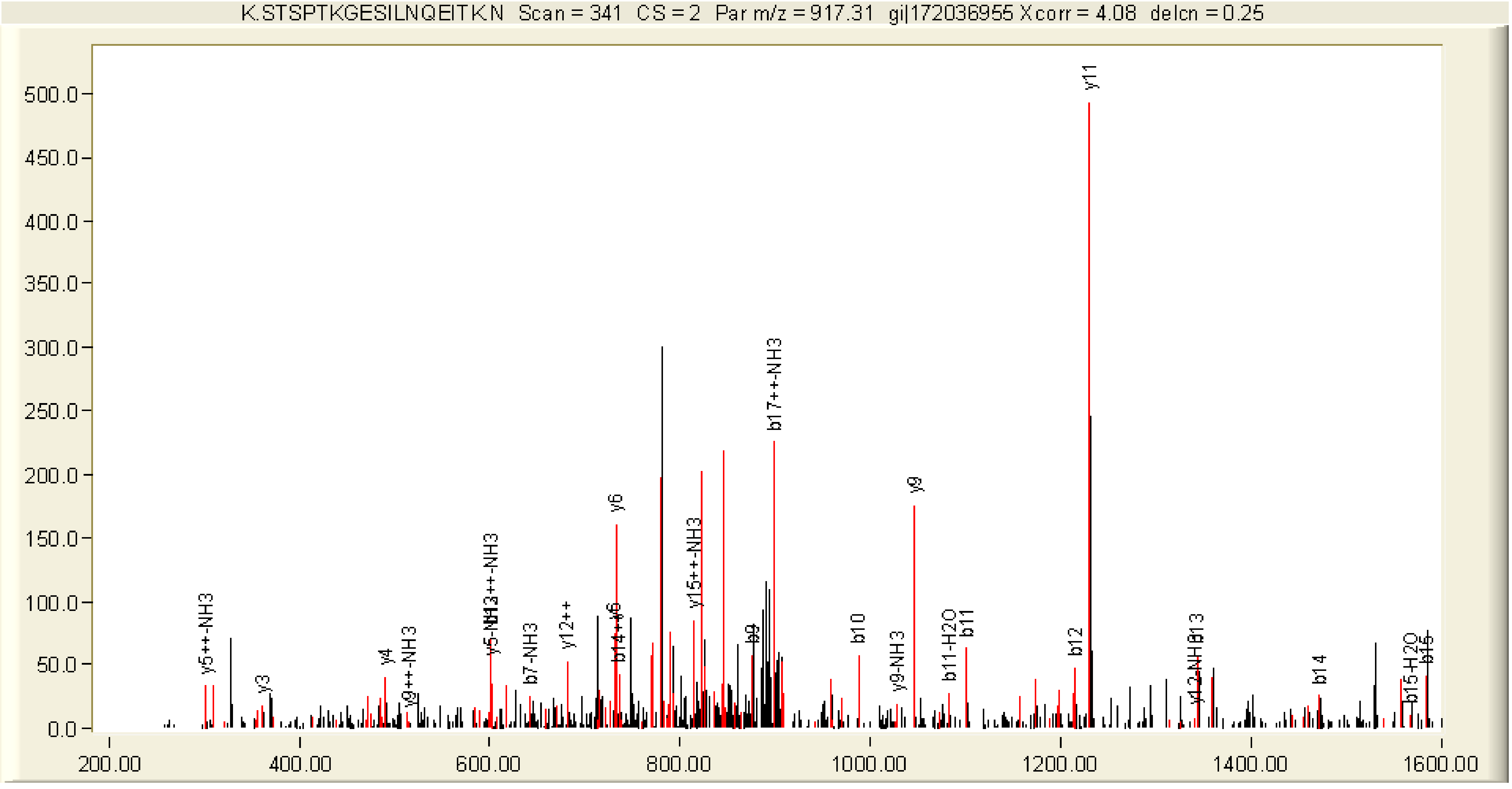
K.STSPTKGESILNQEITK.N Scan = 341 CS = 2 Par m/z = 917.31 gi|172036955 Xcorr = 4.08 delcn = 0.25
500.0
450.0
400.0
350.0
300.0
250.0
200.0
150.0
100.0
50.0
0.0
200.00
400.00
600.00
800.00
1000.00
1200.00
1400.00
1600.00
y5++-NH3
y3
y4
y9++-NH3
b13++-NH3
b7-NH3
y12++
y6
y15++-NH3
b17++-NH3
b10
y9-NH3
y9
b11-H2O
b11
b12
y11
b13
b14
b15-H2O

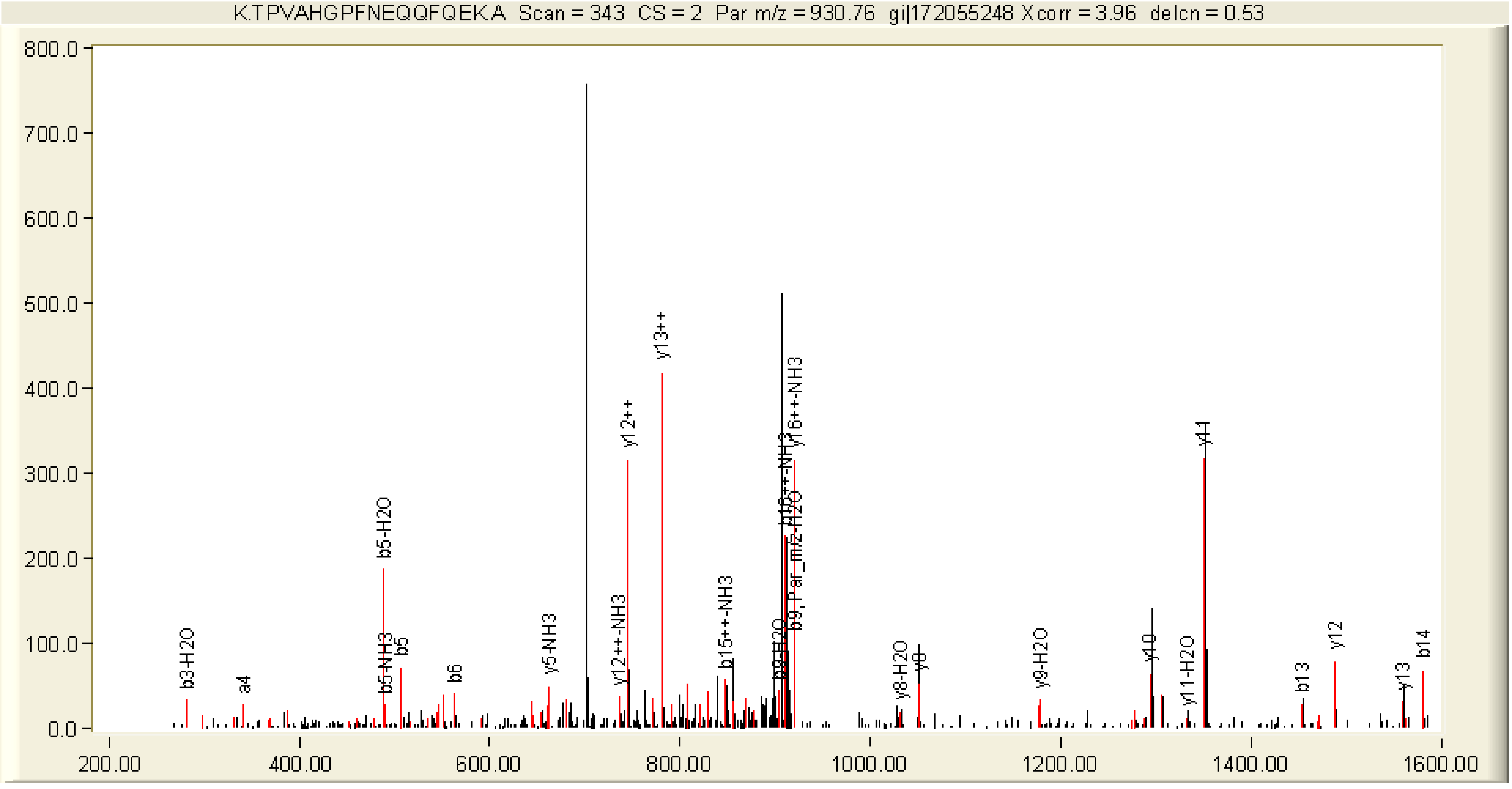
K.TPVAHGPFNEQQFQEKA Scan = 343 CS = 2 Par m/z = 930.76 gi|172055248 Xcorr = 3.96 delcn = 0.53
800.0
700.0
600.0
500.0
400.0
300.0
200.0
100.0
0.0
200.00
400.00
600.00
800.00
1000.00
1200.00
1400.00
1600.00
b3-H2O
a4
b5-H2O
b5-NH3
b5
b6
y5-NH3
y12++-NH3
y12++
y13++
b15++-NH3
y16++-NH3
b9_Par_m/z-H2O
y8-H2O
y9-H2O
y10
y11-H2O
y11
b13
y12
y13
b14

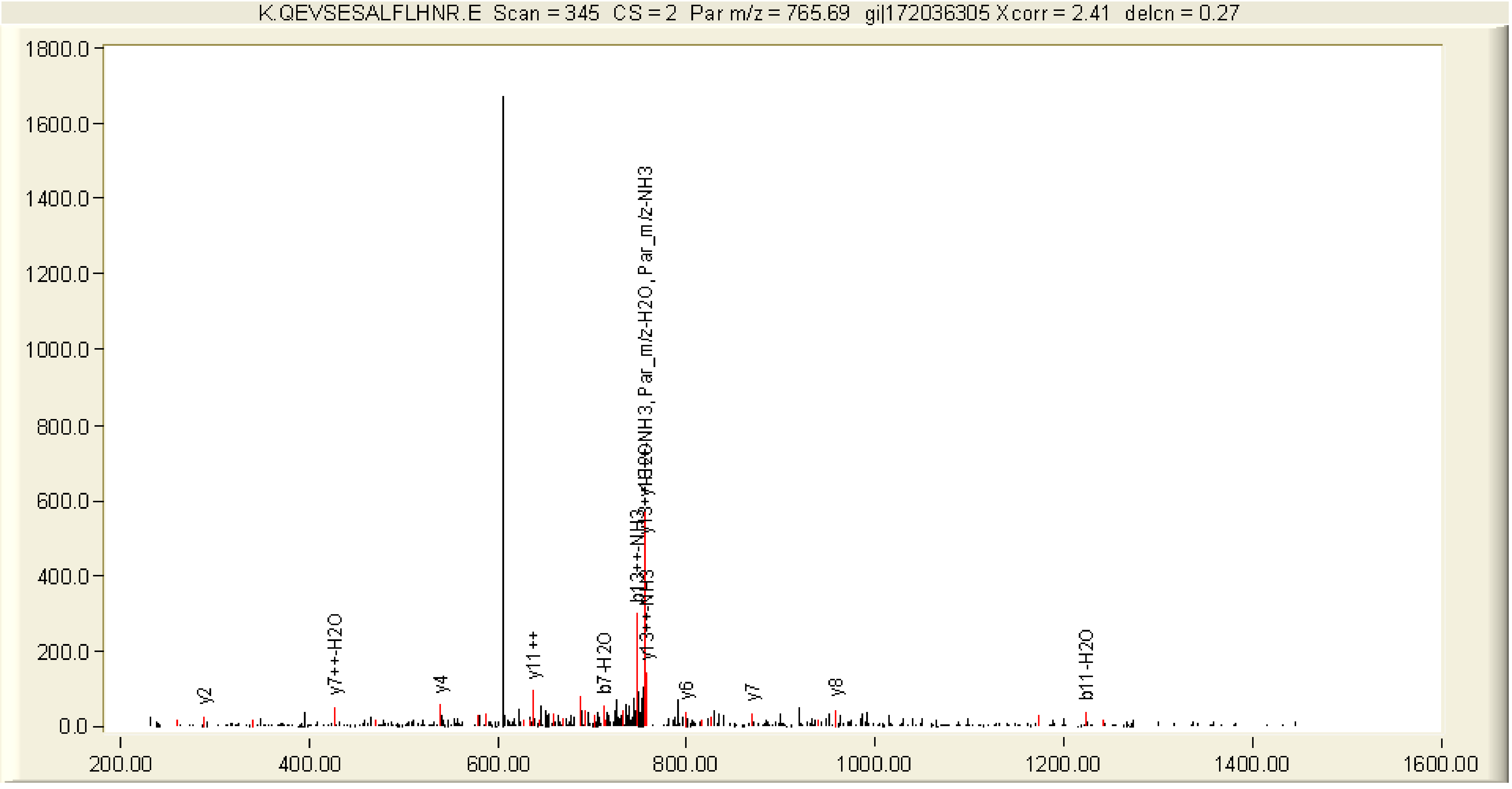

K.QEVSESALFLHNR.E Scan = 345 CS = 2 Par m/z = 765.69 gi|172036305 Xcorr = 2.41 delcn = 0.27
1800.0
1600.0
1400.0
1200.0
1000.0
800.0
600.0
400.0
200.0
0.0
200.00
400.00
600.00
800.00
1000.00
1200.00
1400.00
1600.00
y2
y7++-H2O
y4
y11++
b7-H2O
y6
y7
y8
b11-H2O

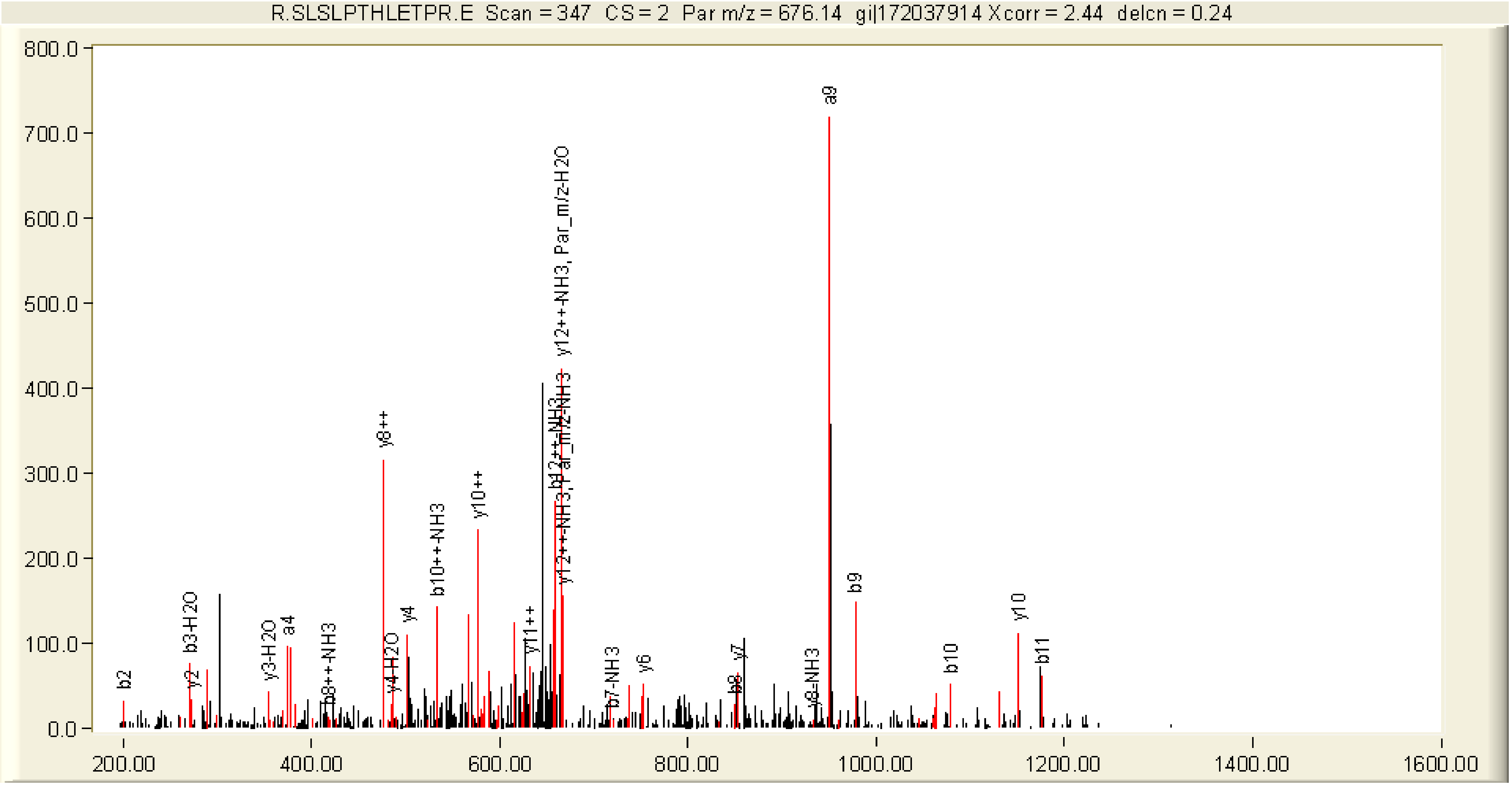
R.SLSLPTHLETPR.E Scan = 347 CS = 2 Par m/z = 676.14 gi|172037914 Xcorr = 2.44 delcn = 0.24
800.0
700.0
600.0
500.0
400.0
300.0
200.0
100.0
0.0
200.00
400.00
600.00
800.00
1000.00
1200.00
1400.00
1600.00
a9
y12++-NH3, Par_m/z-H2O
y8++
y10++
b10++-NH3
b3-H2O
y3-H2O
a4
y4
y4-H2O
b8++-NH3
b2
y2
y11++
b7-NH3
y6
b8
y7
y8-NH3
b9
b10
y10
b11

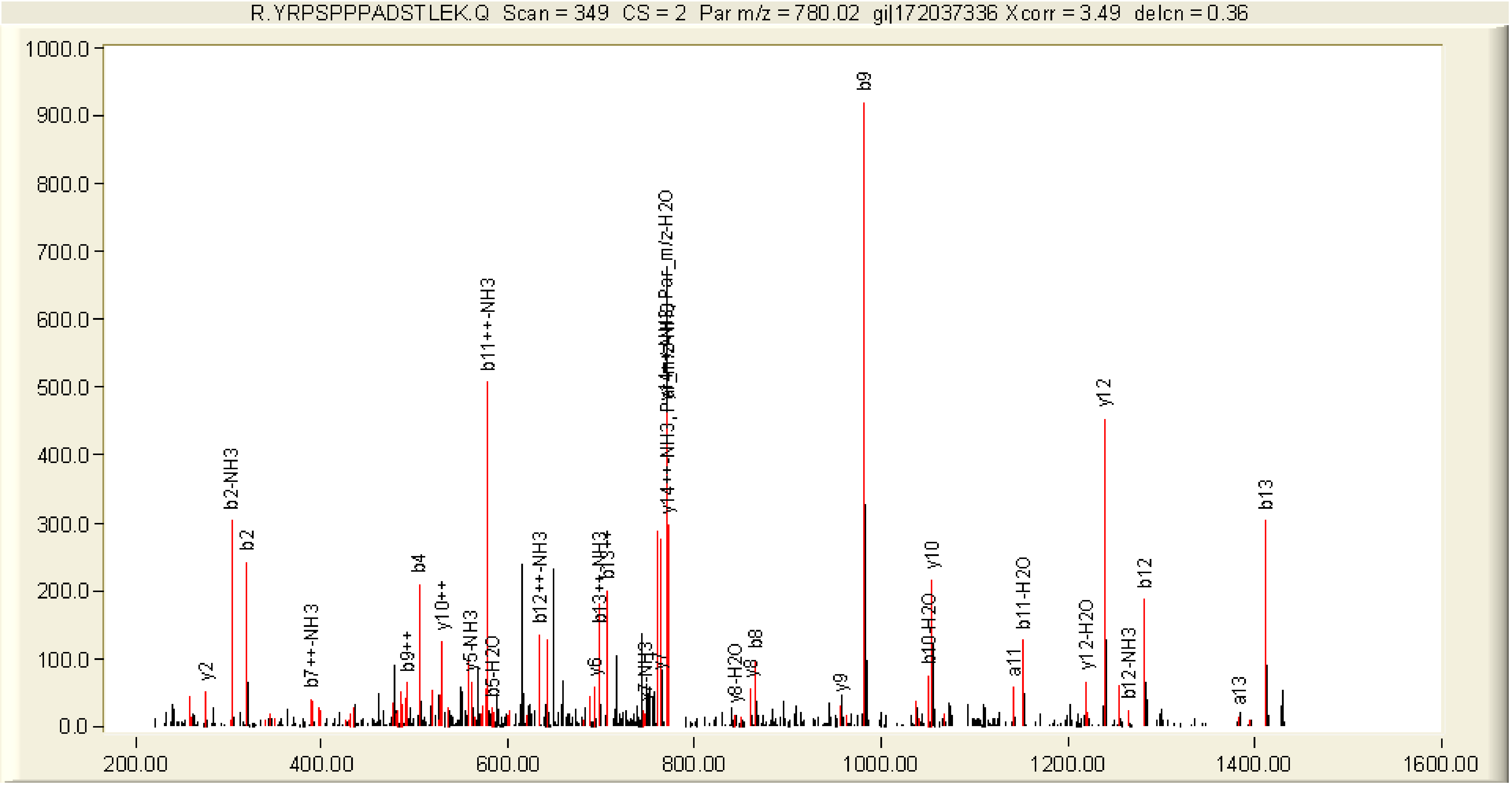
R.YRPSPPPADSTLEK.Q Scan = 349 CS = 2 Par m/z = 780.02 gi|172037336 Xcorr = 3.49 delcn = 0.36
1000.0
900.0
800.0
700.0
600.0
500.0
400.0
300.0
200.0
100.0
0.0
200.00
400.00
600.00
800.00
1000.00
1200.00
1400.00
1600.00
y2
b2-NH3
b2
b7++-NH3
b9++
b4
y10++
y5-NH3
b5-H2O
b11++-NH3
b12++-NH3
y6
b13++-NH3
b13++
y7-NH3
y7
y14++-NH3, Par_m/z-H2O
y8-H2O
y8
b8
y9
b9
b10-H2O
y10
a11
b11-H2O
y12-H2O
y12
b12-NH3
b12
a13
b13

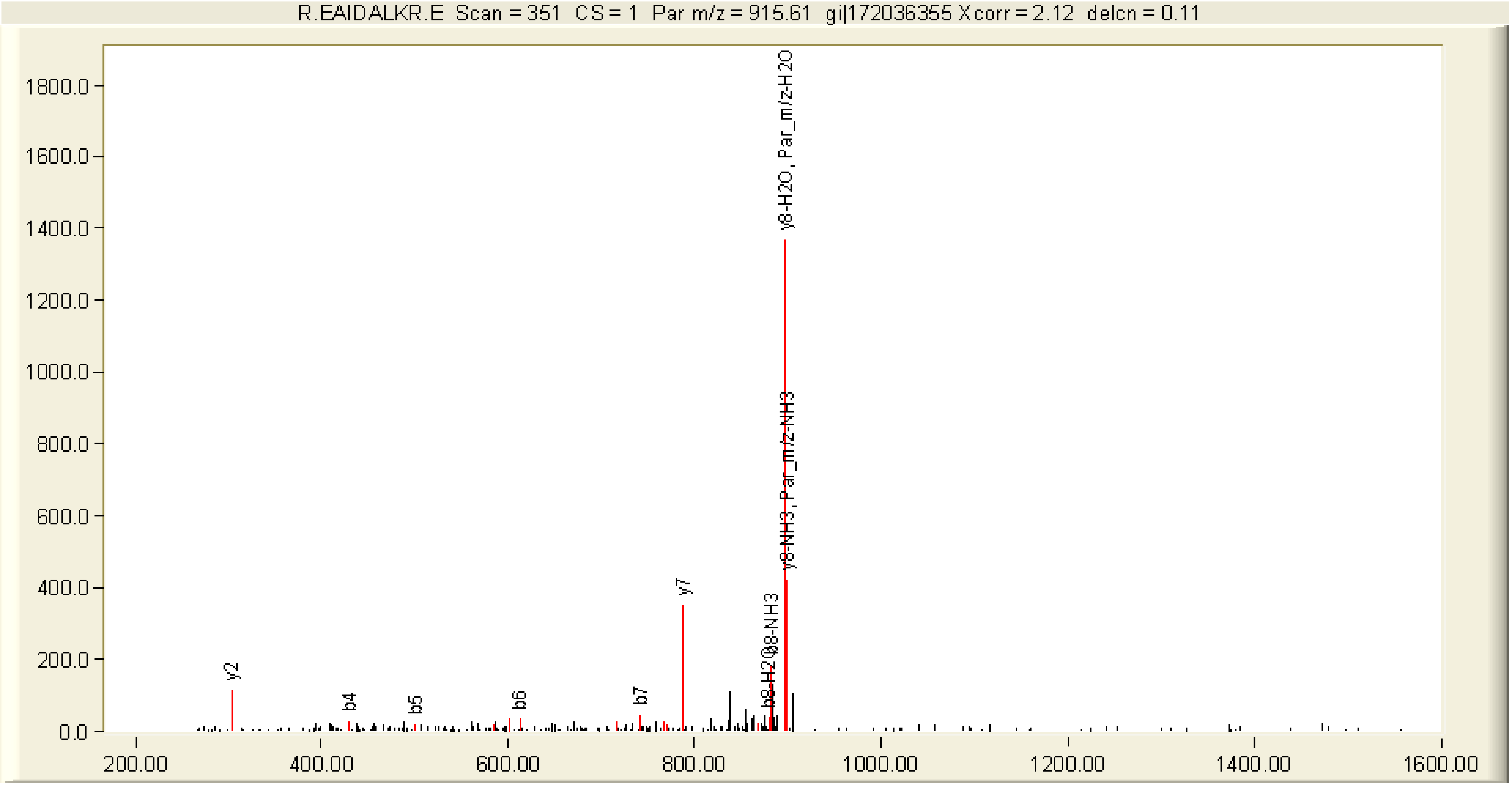
R.EAIDALKR.E Scan = 351 CS = 1 Par m/z = 915.61 gi|172036355 Xcorr = 2.12 delcn = 0.11
1800.0
1600.0
1400.0
1200.0
1000.0
800.0
600.0
400.0
200.0
0.0
200.00
400.00
600.00
800.00
1000.00
1200.00
1400.00
1600.00
y2
b4
b5
b6
b7
y7
b8-H2O
b8-NH3
y8-NH3, Par_m/z-NH3
y8-H2O, Par_m/z-H2O

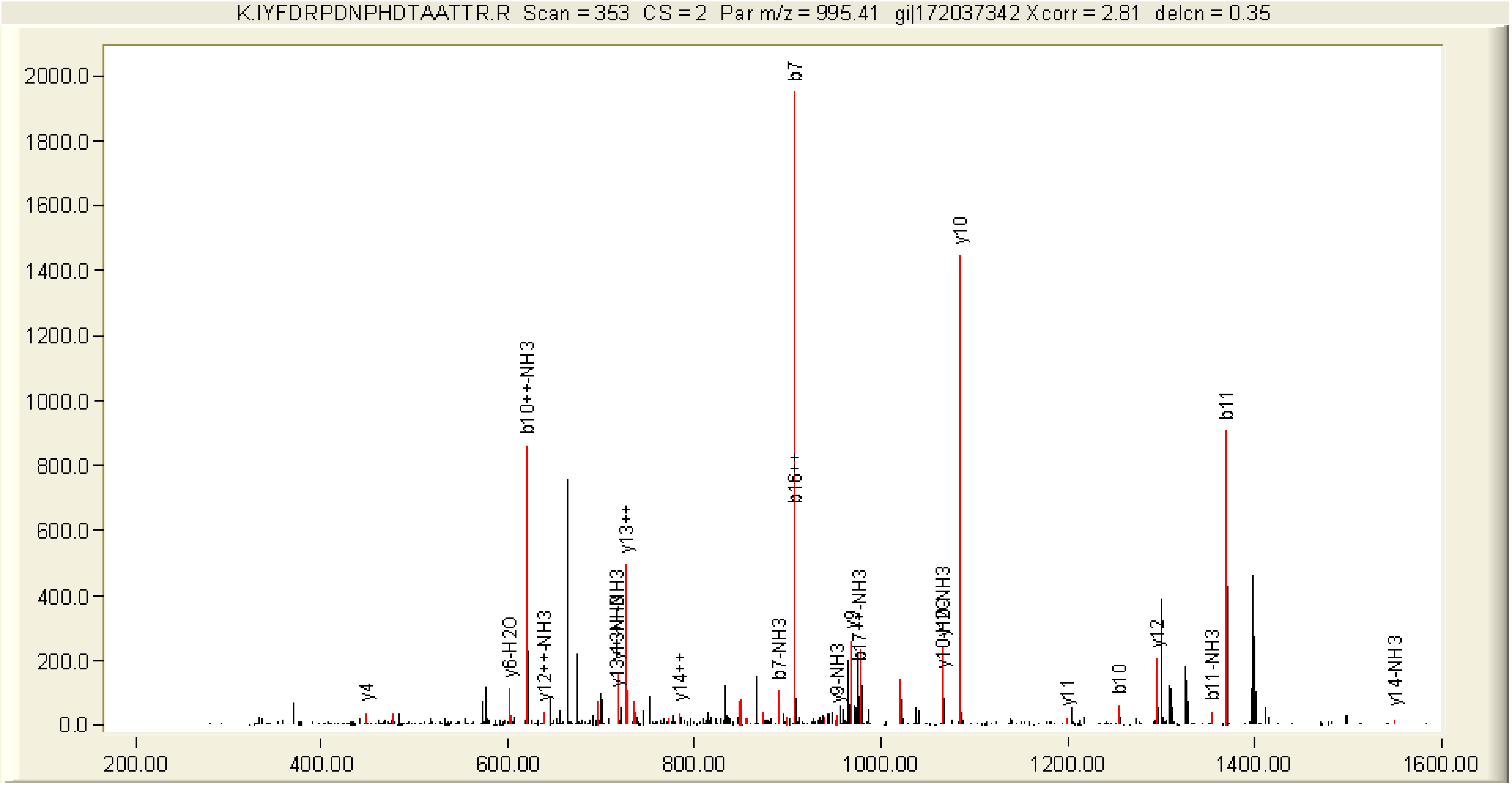
K.IYFDRPDNPHDTAATTR.R Scan = 353 CS = 2 Par m/z = 995.41 gi|172037342 Xcorr = 2.81 delcn = 0.35
2000.0
1800.0
1600.0
1400.0
1200.0
1000.0
800.0
600.0
400.0
200.0
0.0
200.00
400.00
600.00
800.00
1000.00
1200.00
1400.00
1600.00
b7
y10
b10++-NH3
b11
b16++
y13++
y4
y6-H2O
y12++-NH3
y14++
b7-NH3
y9-NH3
y11
b10
y12
b11-NH3
y14-NH3

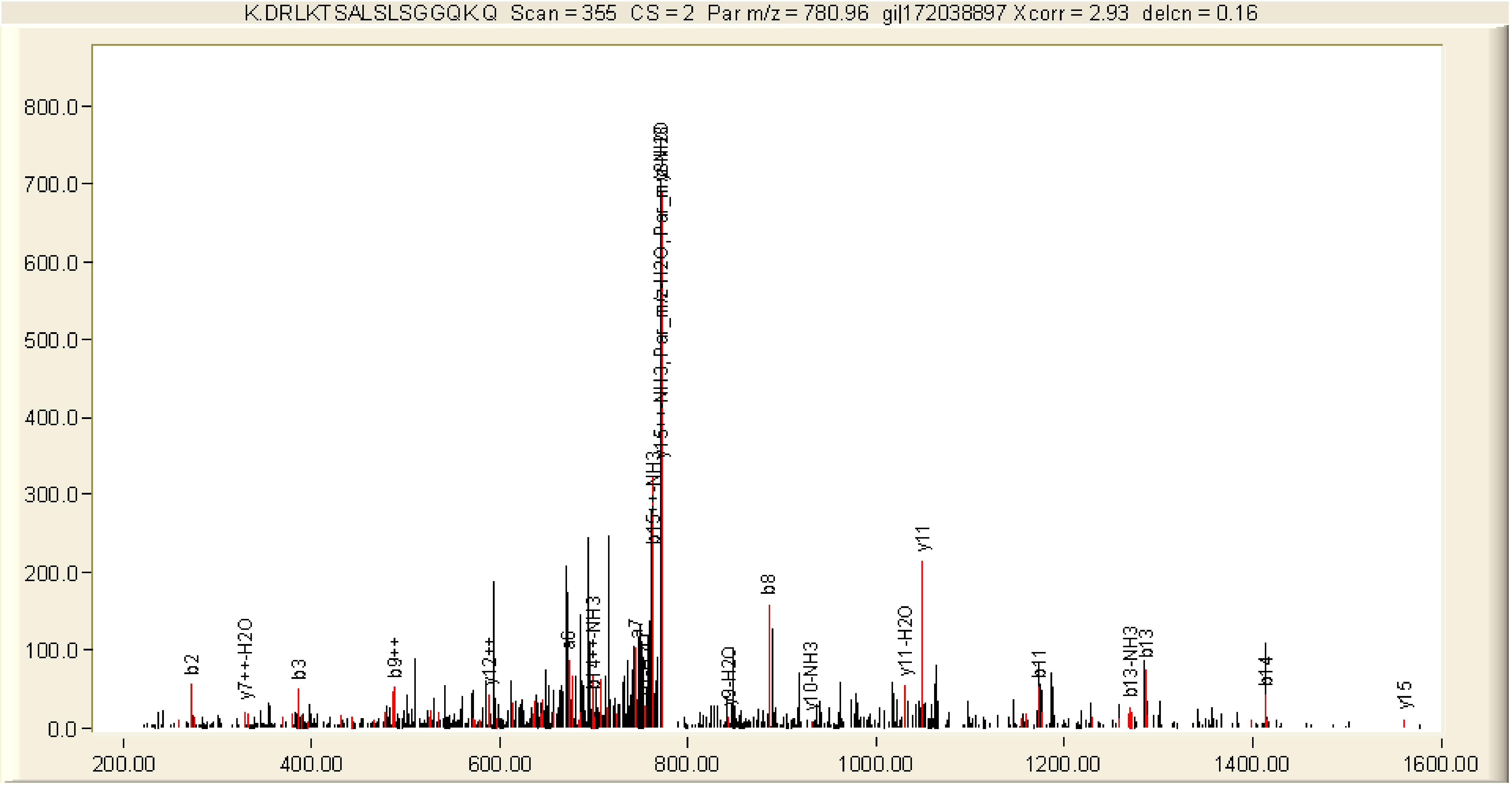

K.DRLKTSALSLSGGQKQ Scan = 355 CS = 2 Par m/z = 780.96 gi|172038897 Xcorr = 2.93 delcn = 0.16
800.0
700.0
600.0
500.0
400.0
300.0
200.0
100.0
0.0
200.00
400.00
600.00
800.00
1000.00
1200.00
1400.00
1600.00
b2
y7++-H2O
b3
b9++
y12++
a6
b14++-NH3
a7
b15++-NH3
y9-H2O
b8
y10-NH3
y11-H2O
y11
b11
b13-NH3
b13
b14
y15

K.ALEVQPHALINQK.L Scan = 357 CS = 2 Par m/z = 731.31 gi|172036154 Xcorr = 4.24 delcn = 0.4

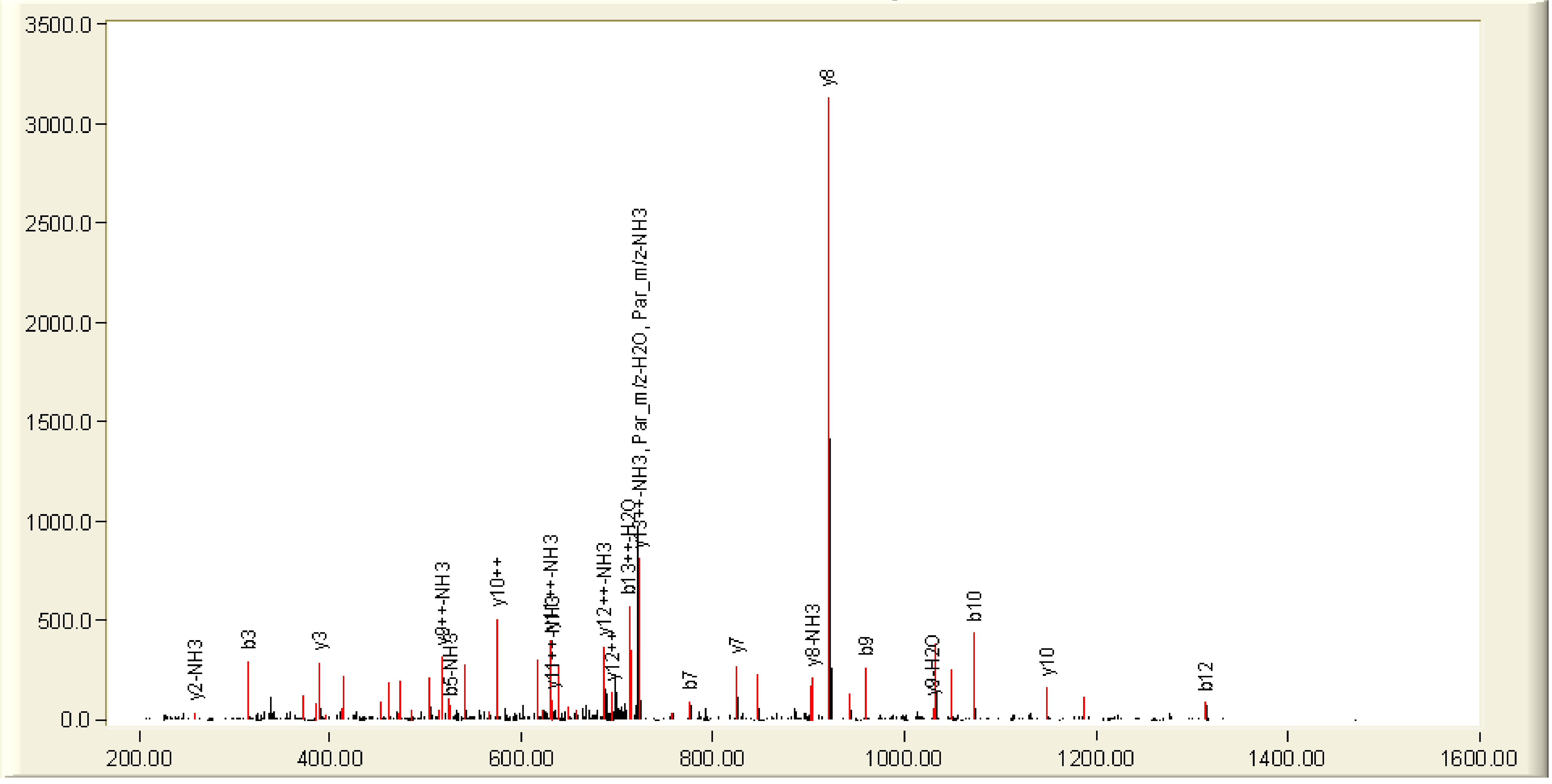

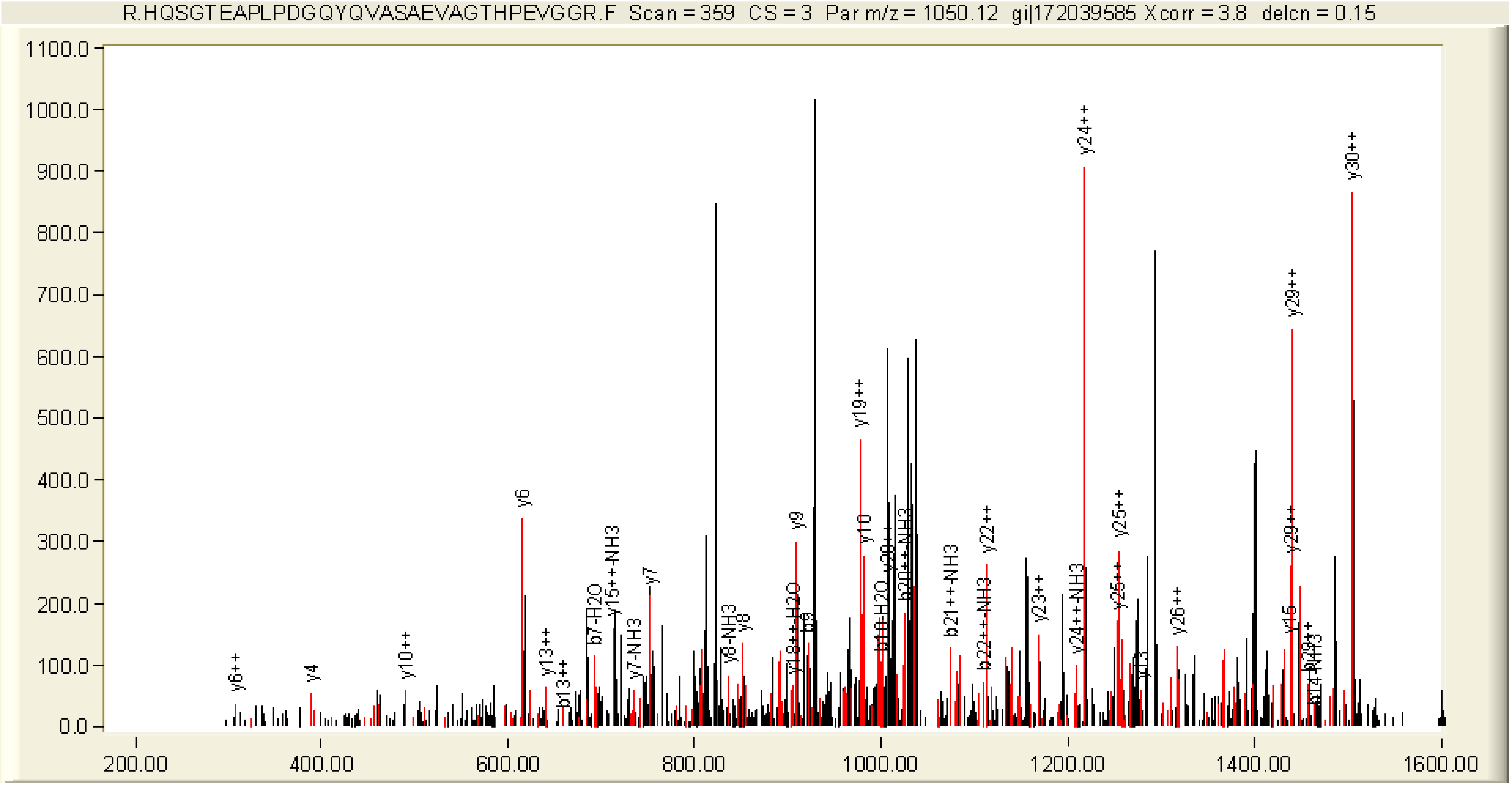
R.HQSGTEAPLPDGQYQVASAEVAGTHPEVGGR.F Scan = 359 CS = 3 Par m/z = 1050.12 gi|172039585 Xcorr = 3.8 delcn = 0.15
1100.0
1000.0
900.0
800.0
700.0
600.0
500.0
400.0
300.0
200.0
100.0
0.0
200.00
400.00
600.00
800.00
1000.00
1200.00
1400.00
1600.00
y6++
y4
y10++
y6
y13++
b13++
b7-H2O
y15++-NH3
y7-NH3
y7
y8-NH3
y8
y9
y19++
y10
b21++-NH3
y22++
b22++-NH3
y23++
y24++
y24++-NH3
y25++
y26++
y29++
y30++
y15

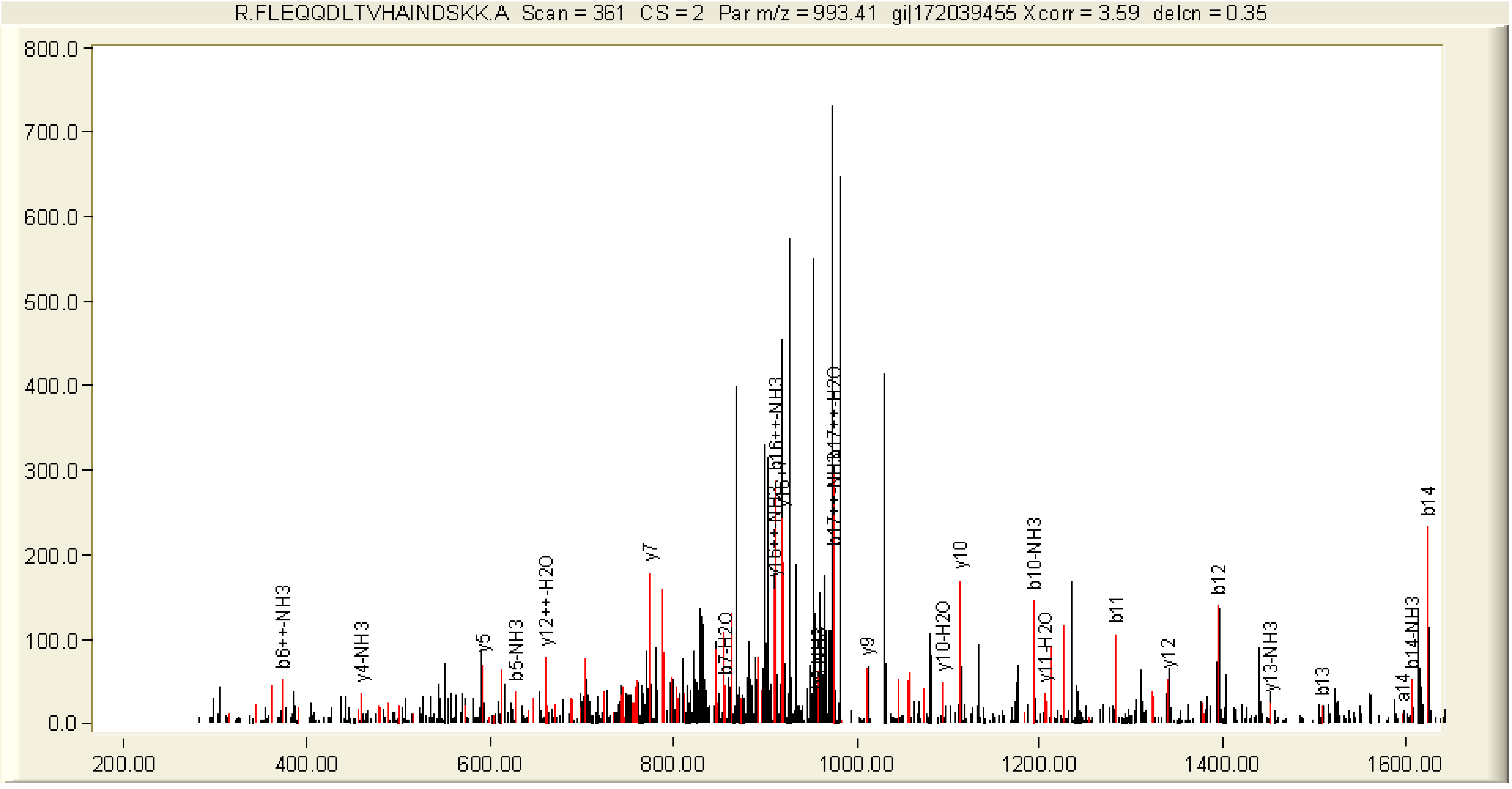
R.FLEQQDLTVHAINDSKK.A Scan = 361 CS = 2 Par m/z = 993.41 gi|172039455 Xcorr = 3.59 delcn = 0.35
800.0
700.0
600.0
500.0
400.0
300.0
200.0
100.0
0.0
200.00
400.00
600.00
800.00
1000.00
1200.00
1400.00
1600.00
b6++-NH3
y4-NH3
y5
b5-NH3
y12++-H2O
y7
b7-H2O
b16++-NH3
b17++-H2O
y9
y10-H2O
y10
b10-NH3
y11-H2O
b11
y12
b12
y13-NH3
b13
a14
b14-NH3
b14

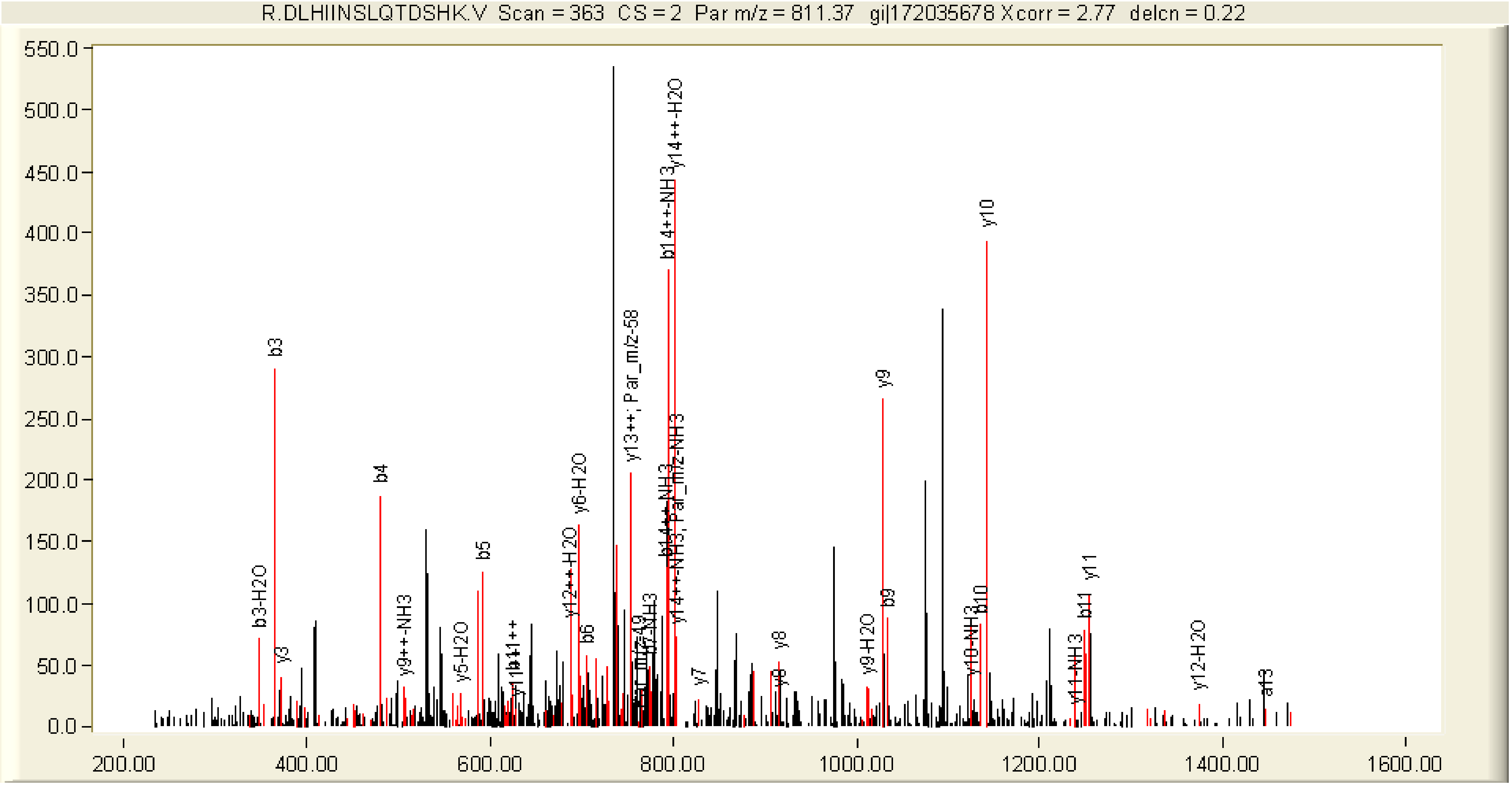
R.DLHIINSLQTDSHK.V Scan = 363 CS = 2 Par m/z = 811.37 gi|172035678 Xcorr = 2.77 delcn = 0.22
550.0
500.0
450.0
400.0
350.0
300.0
250.0
200.0
150.0
100.0
50.0
0.0
200.00
400.00
600.00
800.00
1000.00
1200.00
1400.00
1600.00
b3-H2O
b3
y3
b4
y9++-NH3
y5-H2O
b5
b6
y12++-H2O
y6-H2O
y13++; Par_m/z-58
b14++-NH3
y14++-H2O
y14++-NH3; Par_m/z-NH3
y7
y8
y9
y9-H2O
b9
y10
b10
y10-NH3
y11
b11
y11-NH3
y12-H2O
a13

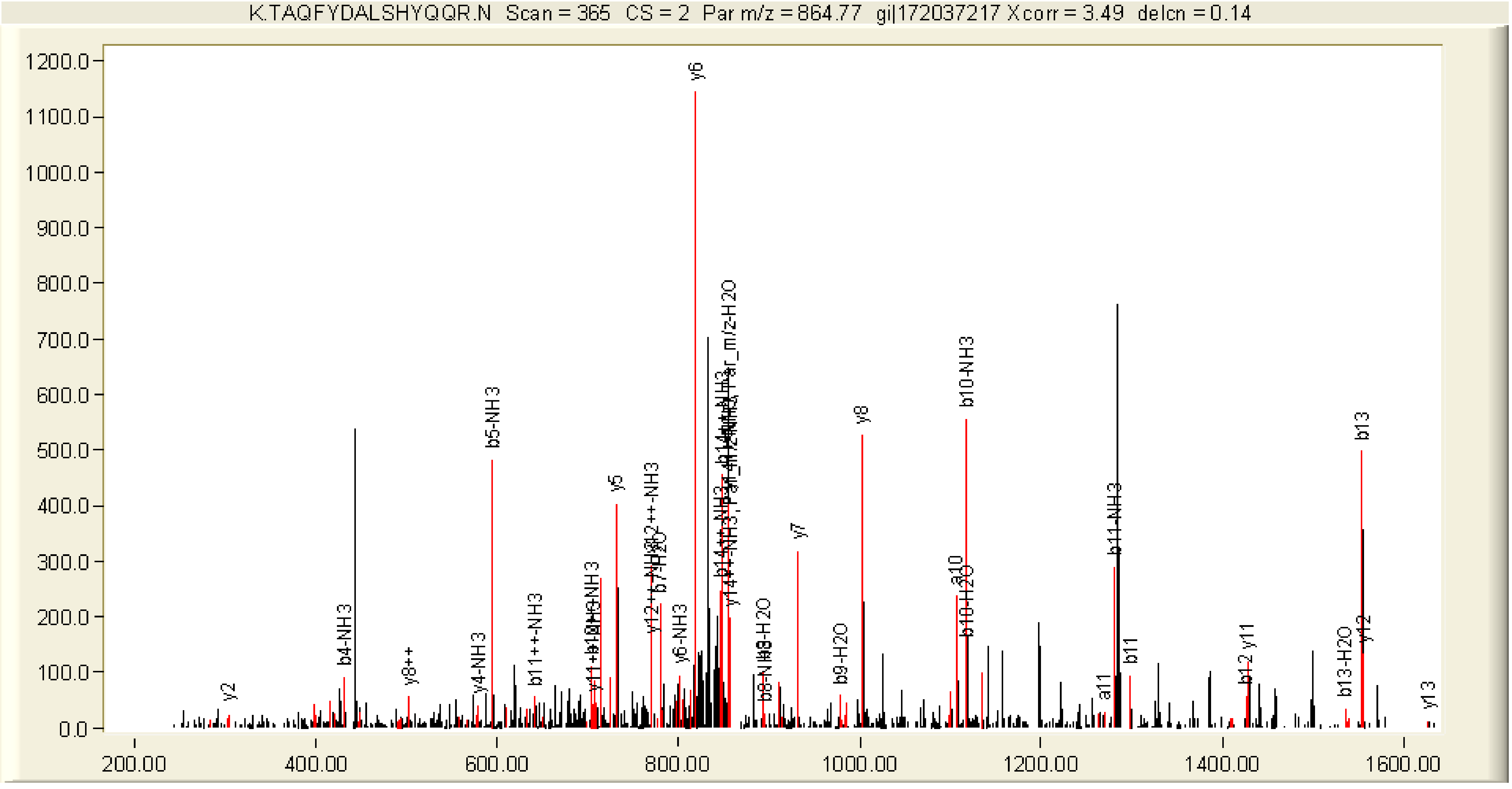

K.TAQFYDALSHYQQR.N Scan = 365 CS = 2 Par m/z = 864.77 gi|172037217 Xcorr = 3.49 delcn = 0.14
1200.0
1100.0
1000.0
900.0
800.0
700.0
600.0
500.0
400.0
300.0
200.0
100.0
0.0
200.00
400.00
600.00
800.00
1000.00
1200.00
1400.00
1600.00
y6
y5
y7
y8
b10-NH3
b5-NH3
b4-NH3
y8++
y4-NH3
b11++-NH3
y6-NH3
b9-H2O
b11-NH3
b13
b13-H2O
y12
y13
a11
b11
y11
a10
y2

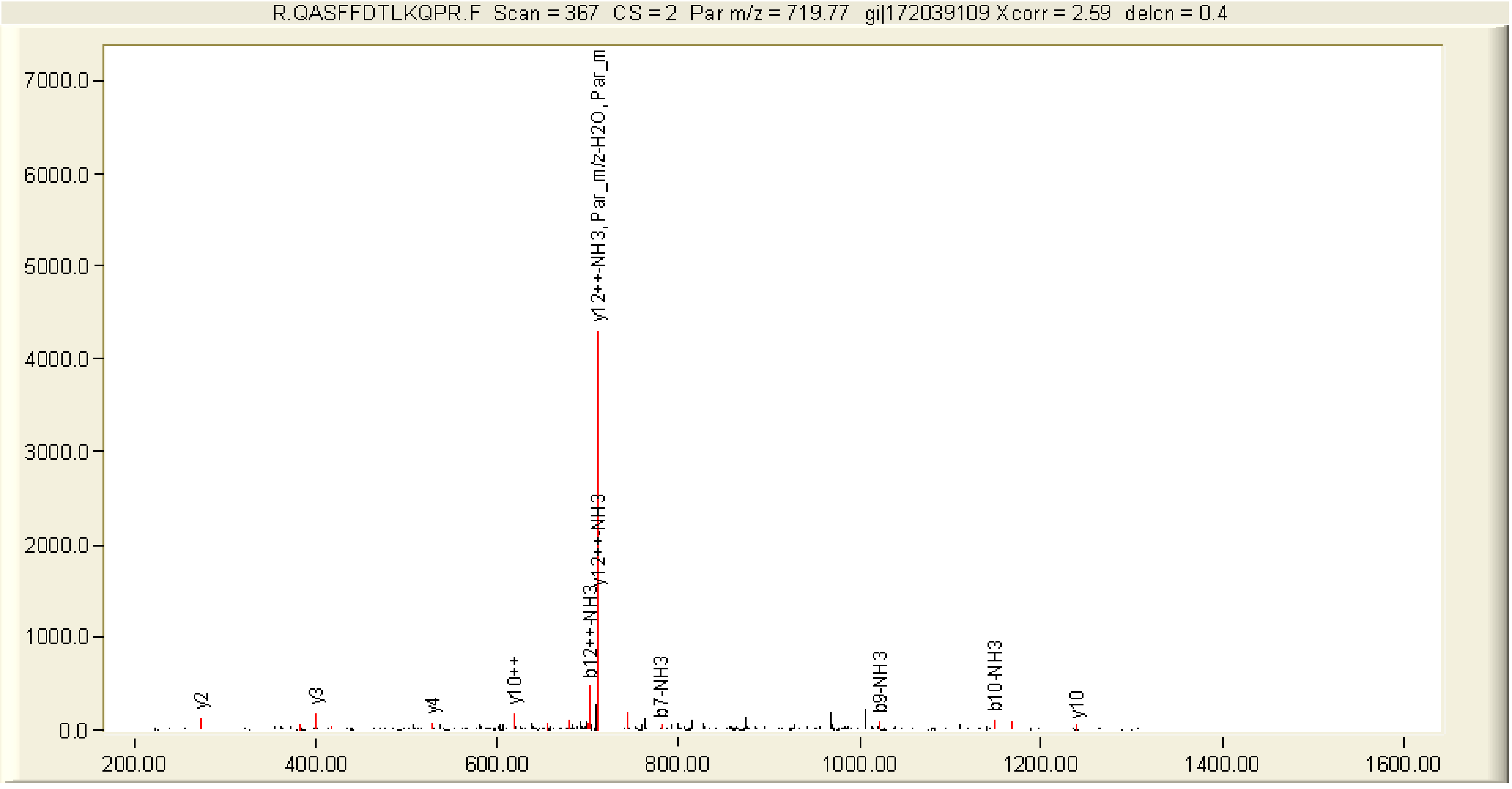
R.QASFFDTLKQPR.F Scan = 367 CS = 2 Par m/z = 719.77 gi|172039109 Xcorr = 2.59 delcn = 0.4
7000.0
6000.0
5000.0
4000.0
3000.0
2000.0
1000.0
0.0
200.00
400.00
600.00
800.00
1000.00
1200.00
1400.00
1600.00
y12++-NH3, Par_m/z-H2O, Par_m
y12++-NH3
b12++-NH3
y2
y3
y4
y10++
b7-NH3
b9-NH3
b10-NH3
y10

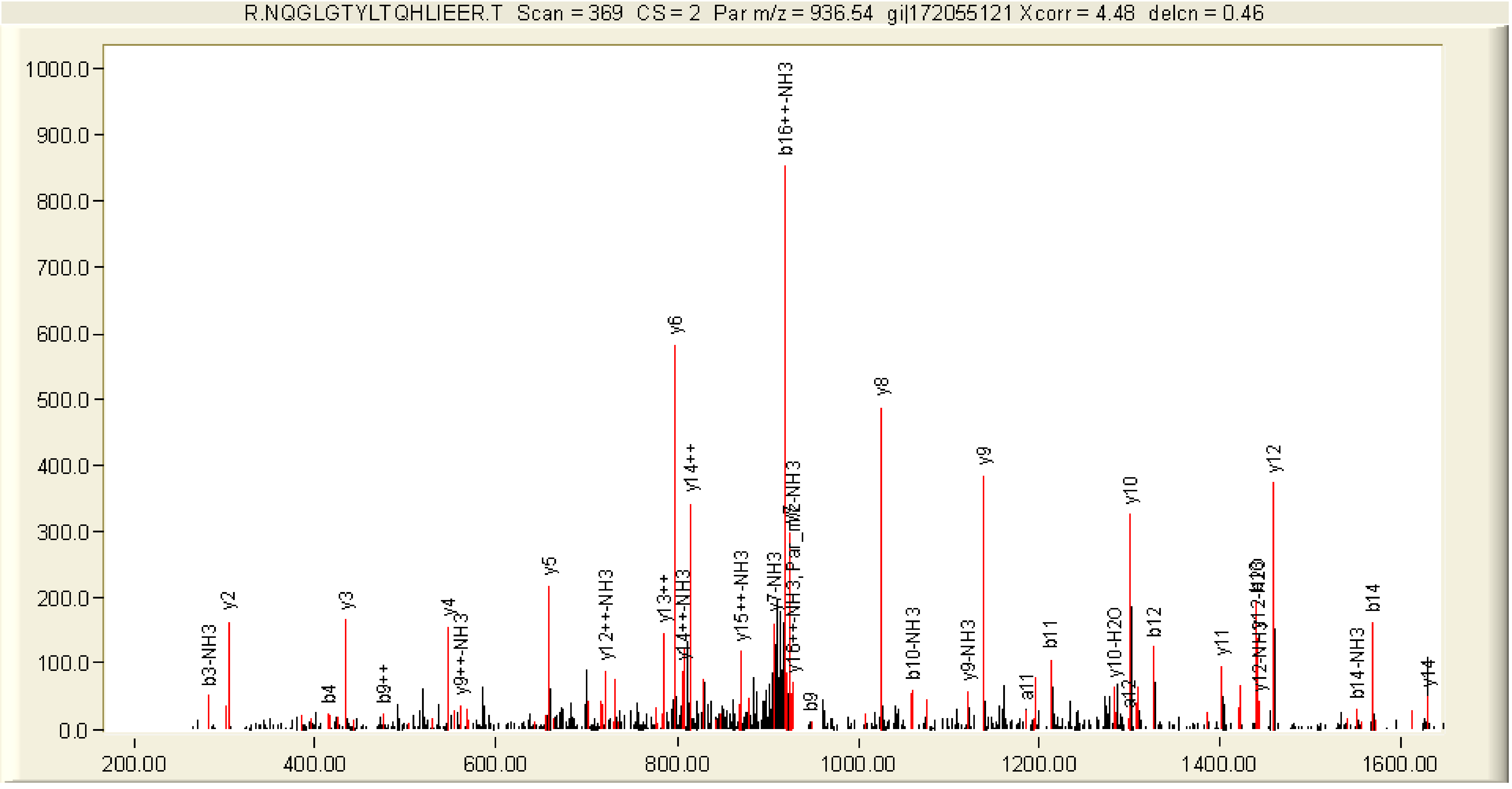
R.NQGLGTYLTQHLIEER.T Scan = 369 CS = 2 Par m/z = 936.54 gi|172055121 Xcorr = 4.48 delcn = 0.46

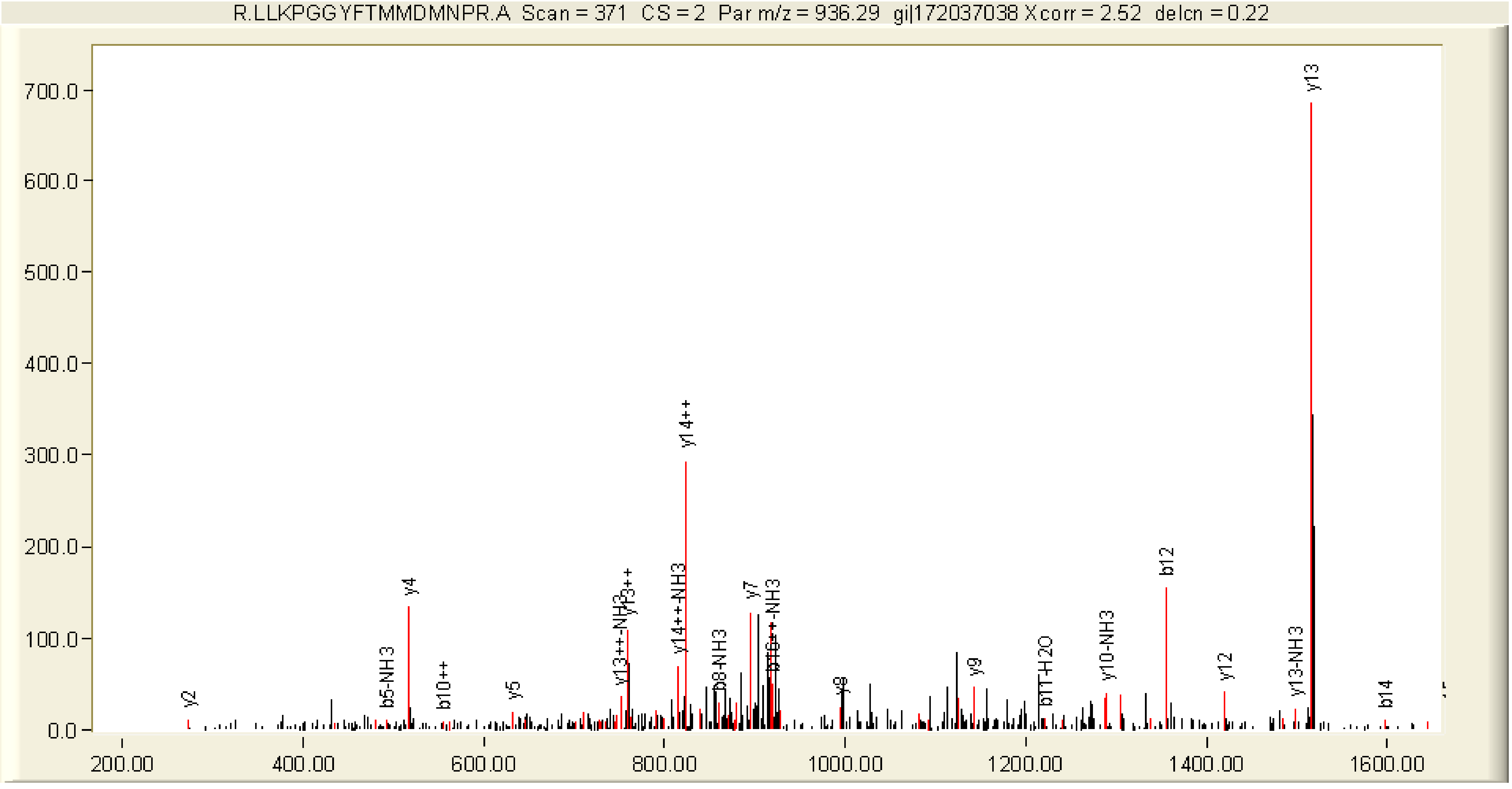
R.LLKPGGYFTMMDMNPR.A Scan = 371 CS = 2 Par m/z = 936.29 gi|172037038 Xcorr = 2.52 delcn = 0.22
700.0
600.0
500.0
400.0
300.0
200.0
100.0
0.0
200.00
400.00
600.00
800.00
1000.00
1200.00
1400.00
1600.00
y13
y14++
b12
y4
y7
y2
b5-NH3
b10++
y5
y14++-NH3
b8-NH3
y9
b11-H2O
y10-NH3
y12
y13-NH3
b14

K.IISQTGLIHSLFER.R Scan = 373 CS = 2 Par m/z = 807.73 gi|172036226 Xcorr = 3.47 delcn = 0.48

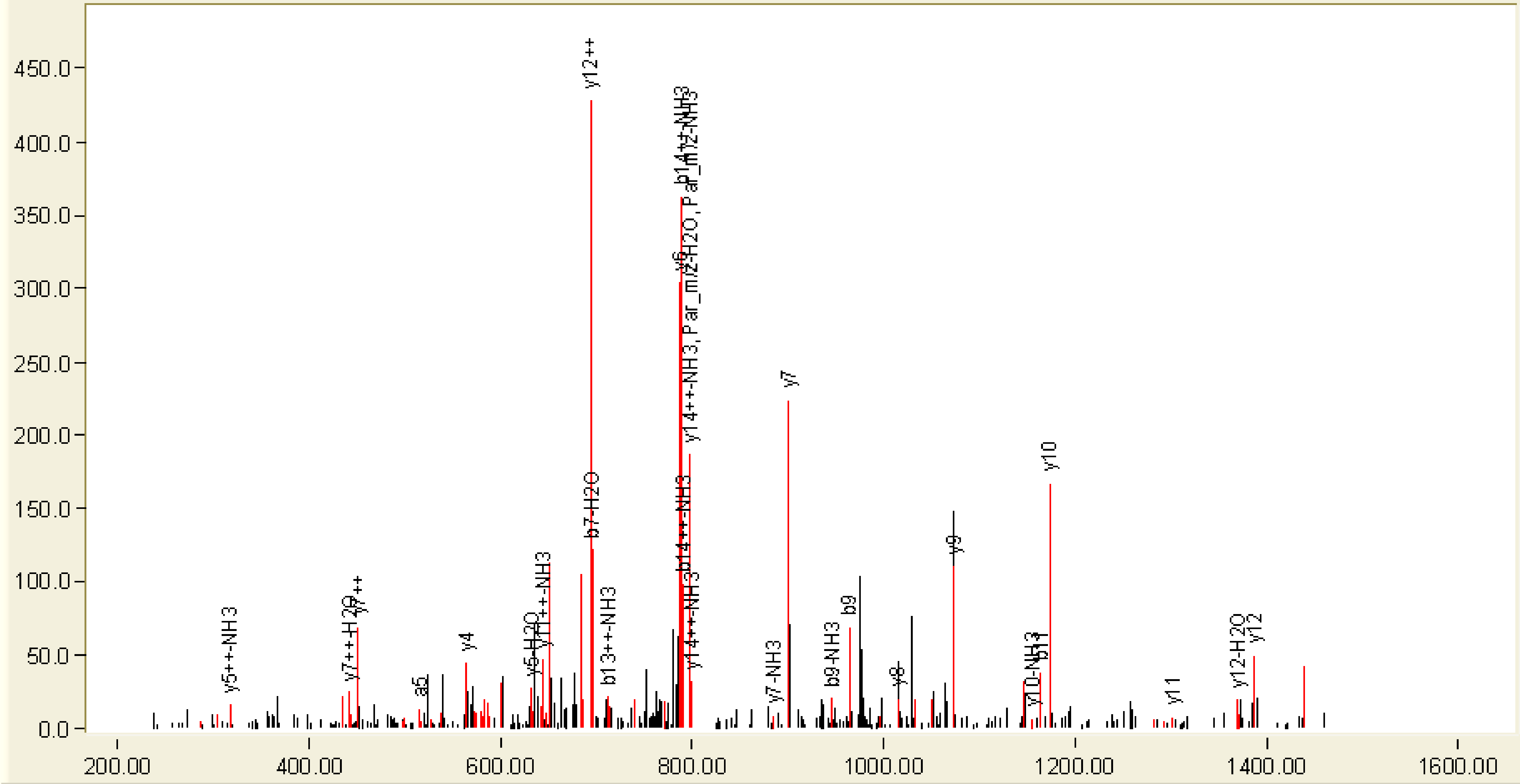

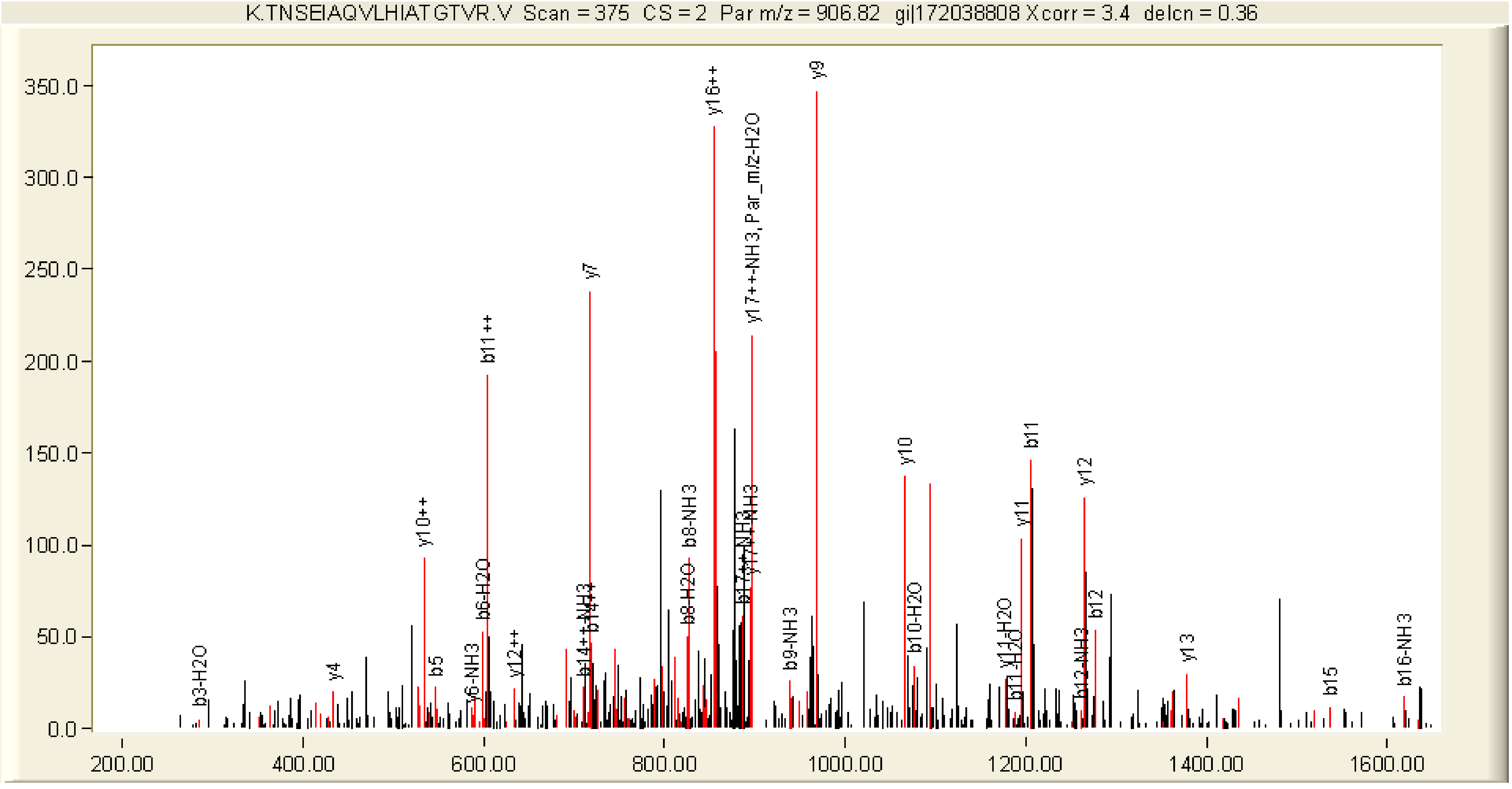
K.TNSEIAQVLHIATGTVR.V Scan = 375 CS = 2 Par m/z = 906.82 gi|172038808 Xcorr = 3.4 delcn = 0.36
350.0
300.0
250.0
200.0
150.0
100.0
50.0
0.0
200.00
400.00
600.00
800.00
1000.00
1200.00
1400.00
1600.00
b3-H2O
y4
y10++
b5
y6-NH3
b6-H2O
b11++
y12++
b14++-NH3
b14++
y7
b8-H2O
b8-NH3
y16++
y17++-NH3, Par_m/z-H2O
y9
b9-NH3
y10
b10-H2O
y11
b11
y12
b12
b12-NH3
y13
b15
b16-NH3

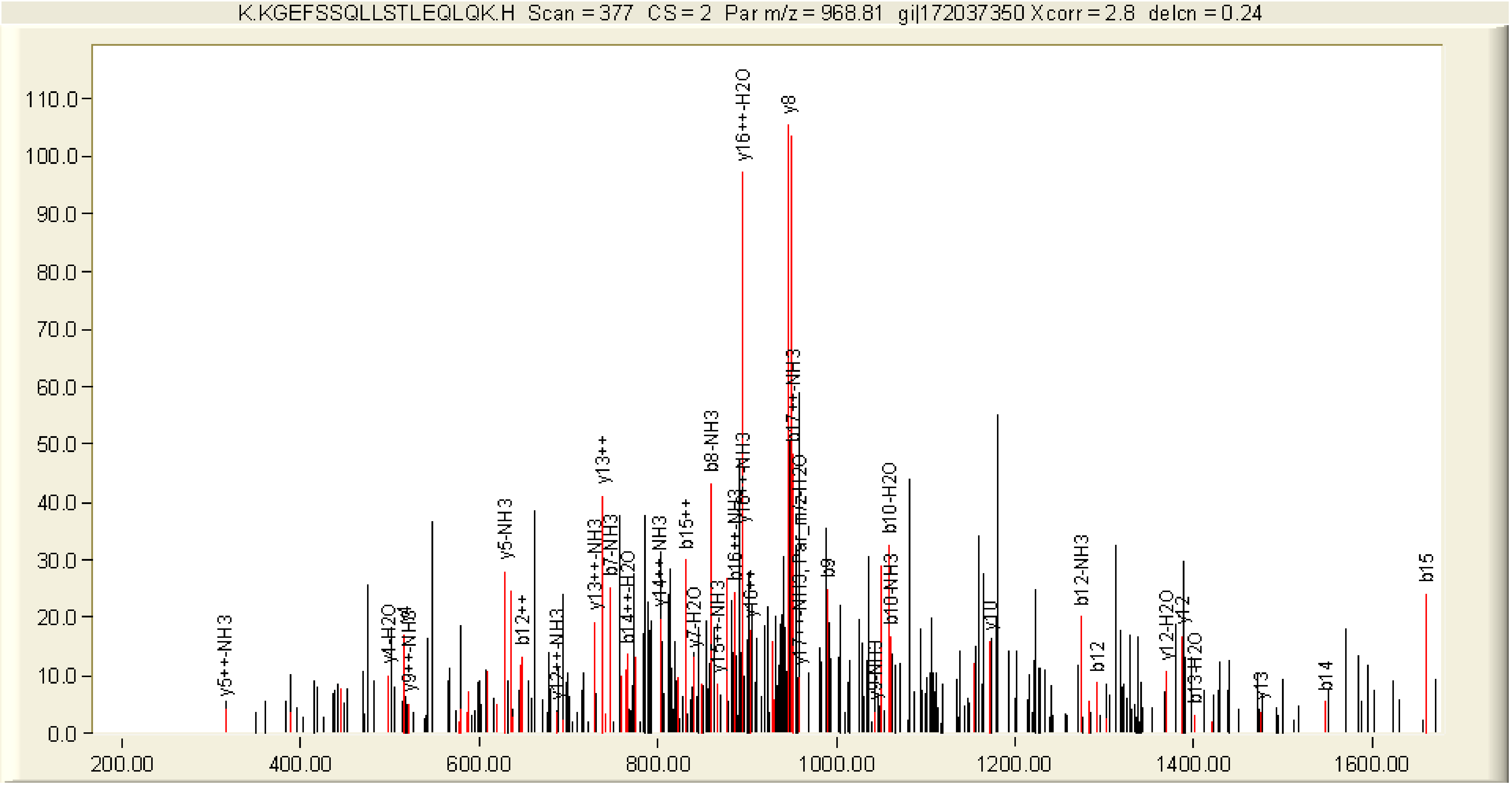

K.KGEFSSQLLSTLEQLQK.H Scan = 377 CS = 2 Par m/z = 968.81 gi|172037350 Xcorr = 2.8 delcn = 0.24
110.0
100.0
90.0
80.0
70.0
60.0
50.0
40.0
30.0
20.0
10.0
0.0
200.00
400.00
600.00
800.00
1000.00
1200.00
1400.00
1600.00
y16++-H2O
y8
y5++-NH3
y4-H2O
y5-NH3
b12++
y13++
y13++-NH3
b7-NH3
b8-NH3
b15++
b10-H2O
b10-NH3
b9
y10
b12-NH3
b12
y12-H2O
y12
b13-H2O
y13
b14
b15

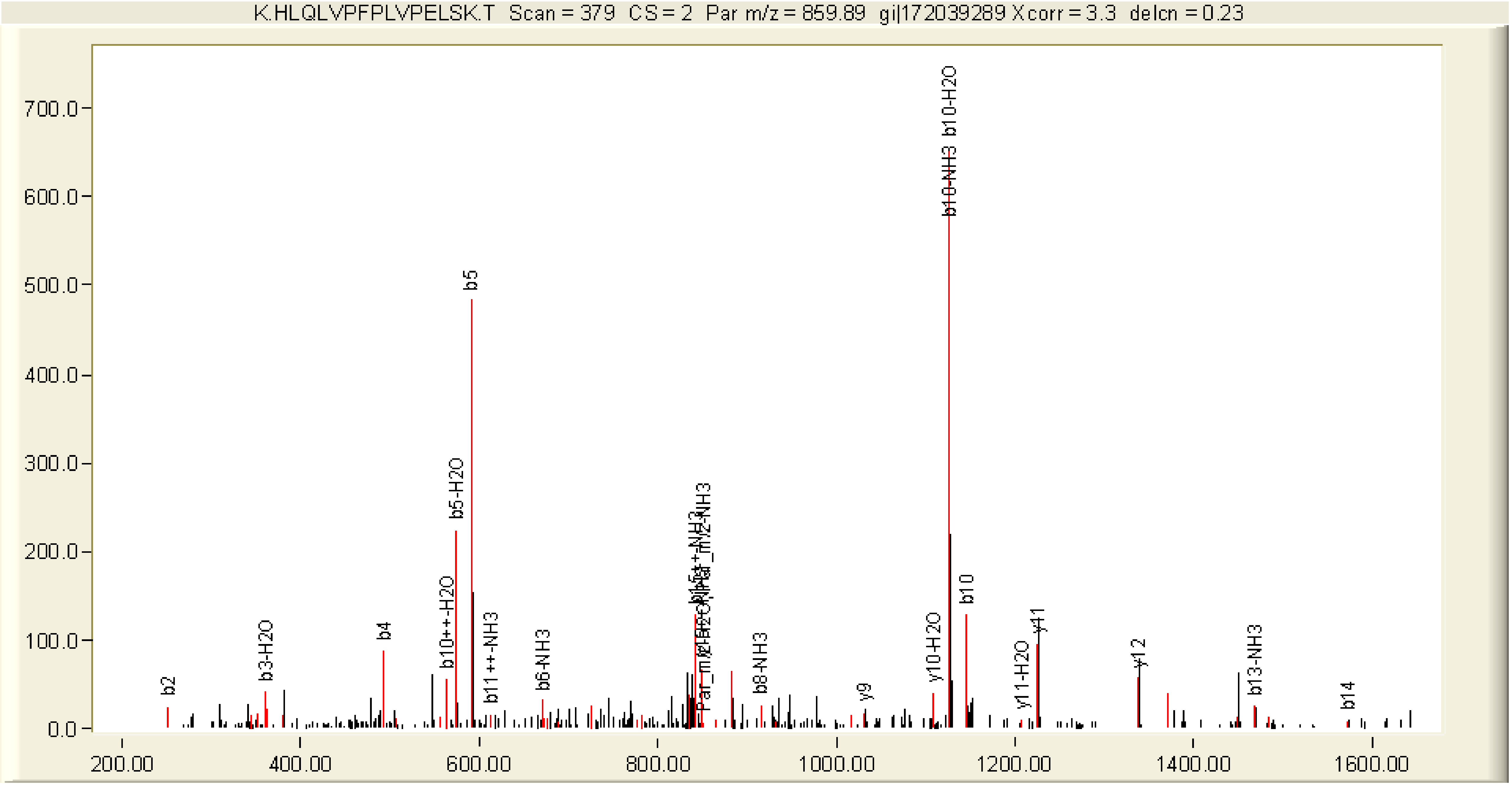
K.HLQLVPFPLVPELSK.T Scan = 379 CS = 2 Par m/z = 859.89 gi|172039289 Xcorr = 3.3 delcn = 0.23
700.0
600.0
500.0
400.0
300.0
200.0
100.0
0.0
200.00
400.00
600.00
800.00
1000.00
1200.00
1400.00
1600.00
b2
b3-H2O
b4
b10++-H2O
b5-H2O
b5
b11++-NH3
b6-NH3
b8-NH3
y9
y10-H2O
b10-NH3 b10-H2O
b10
y11-H2O
y11
y12
b13-NH3
b14

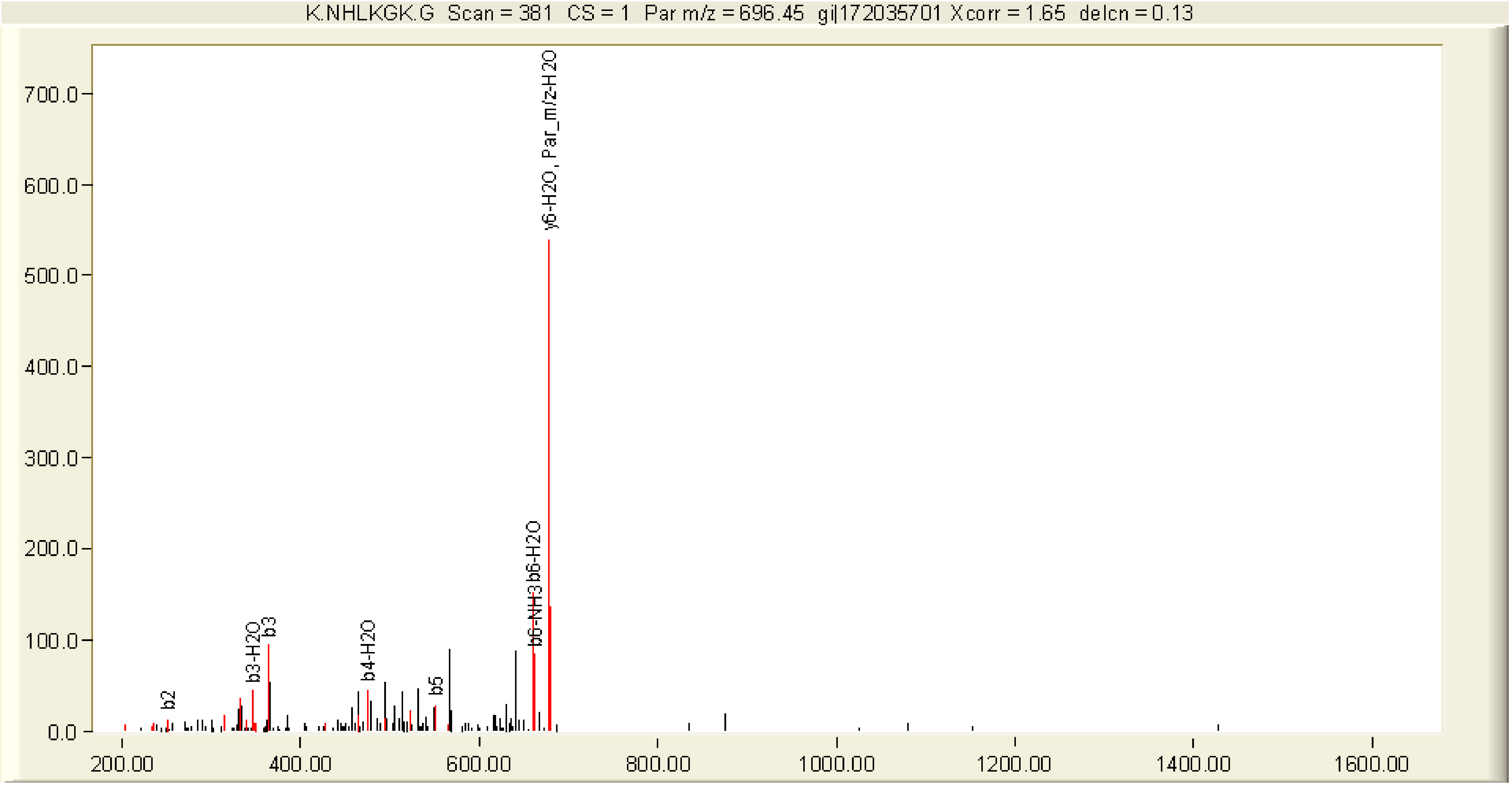

K.NHLKGK.G Scan = 381 CS = 1 Par m/z = 696.45 gi|172035701 Xcorr = 1.65 delcn = 0.13
700.0
600.0
500.0
400.0
300.0
200.0
100.0
0.0
200.00
400.00
600.00
800.00
1000.00
1200.00
1400.00
1600.00
b2
b3-H2O
b3
b4-H2O
b5
b6-NH3
b6-H2O
y6-H2O, Par_m/z-H2O

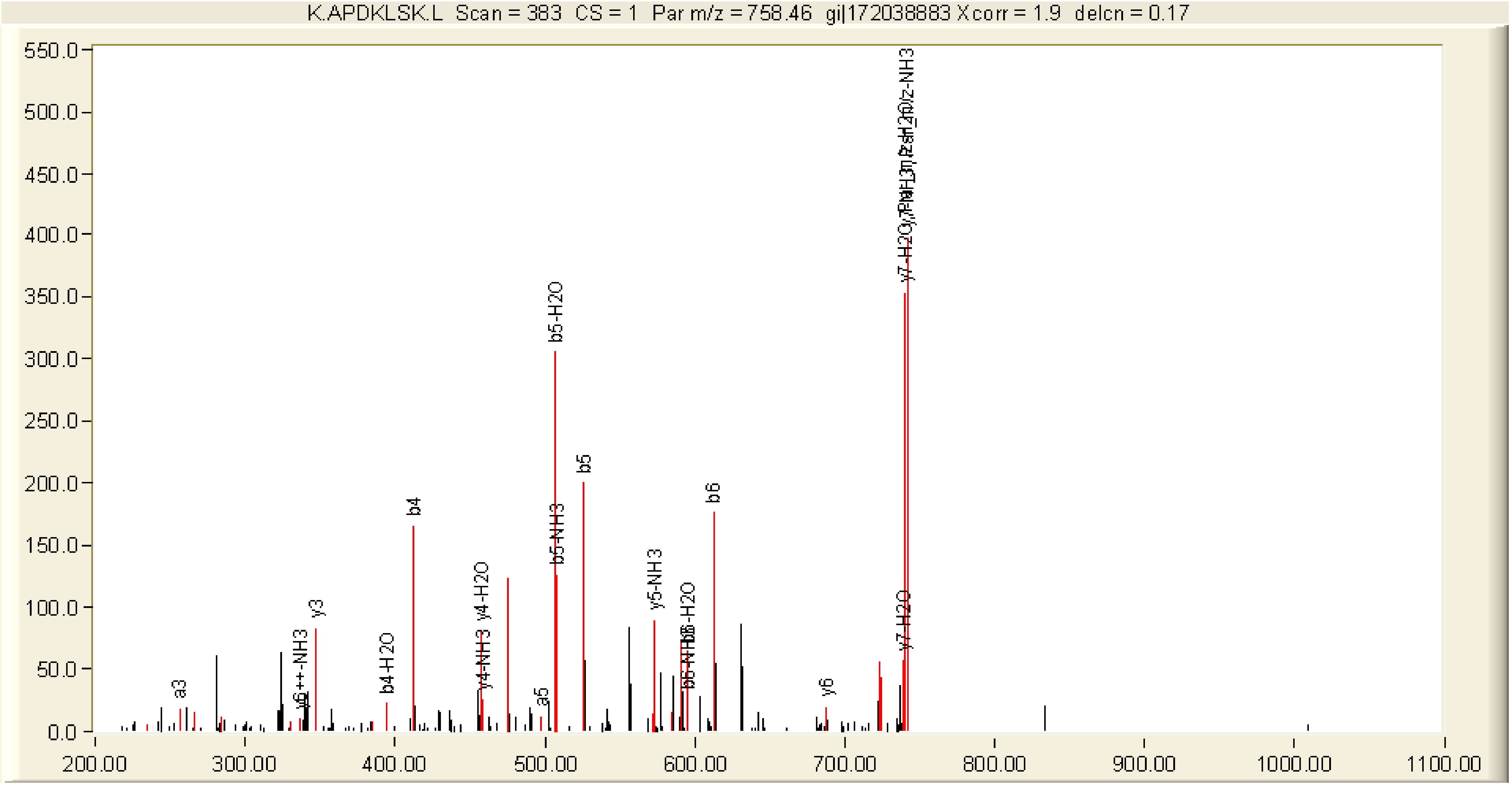

K.APDKLSK.L Scan = 383 CS = 1 Par m/z = 758.46 gi|172038883 Xcorr = 1.9 delcn = 0.17
550.0
500.0
450.0
400.0
350.0
300.0
250.0
200.0
150.0
100.0
50.0
0.0
200.00
300.00
400.00
500.00
600.00
700.00
800.00
900.00
1000.00
1100.00
a3
y6++-NH3
y3
b4-H2O
b4
y4-NH3
y4-H2O
a5
b5-NH3
b5-H2O
b5
y5-NH3
b6-H2O
b6
y6
y7-H2O

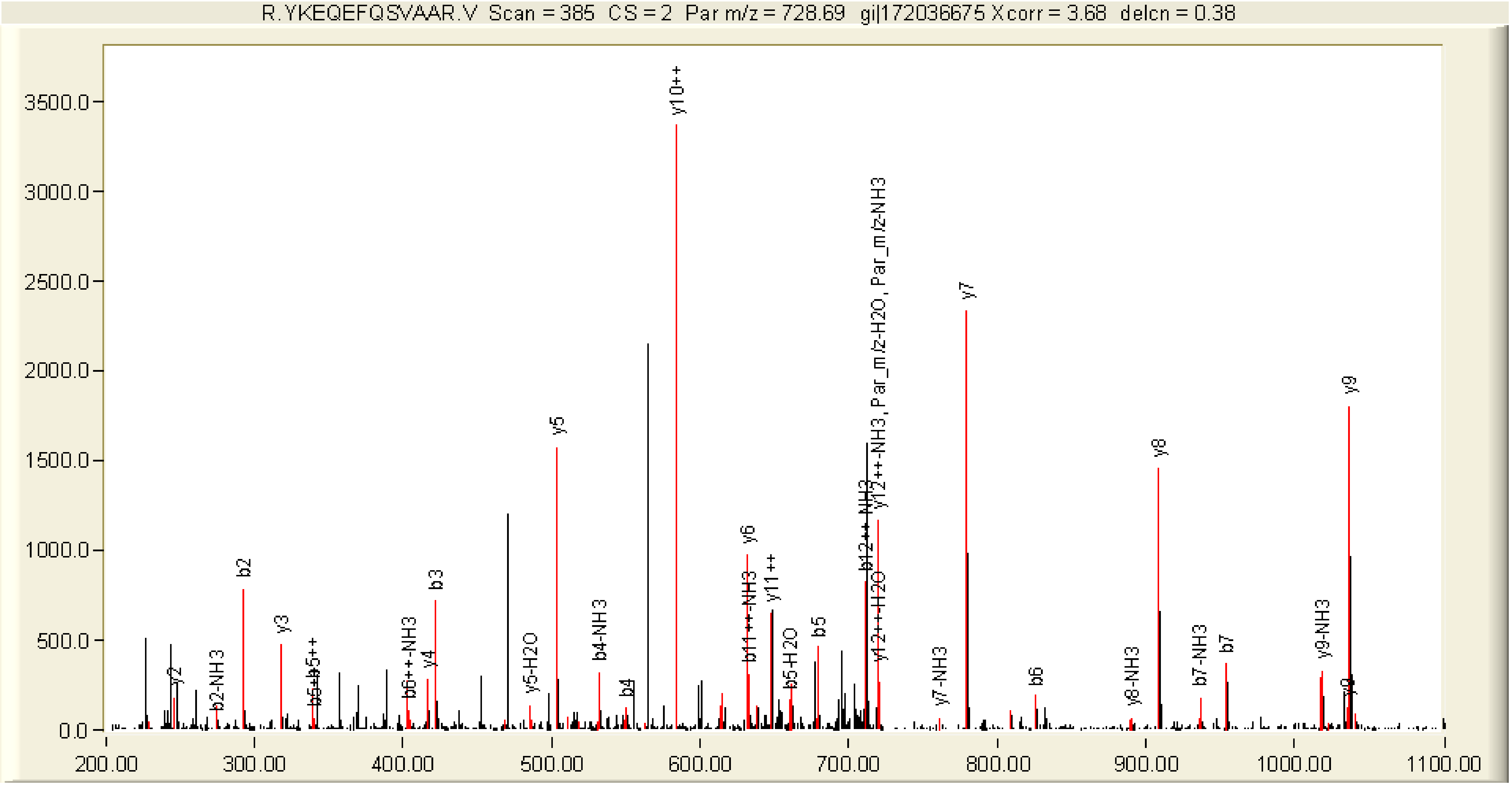
R.YKEQEFQSVAAR.V Scan = 385 CS = 2 Par m/z = 728.69 gi|172036675 Xcorr = 3.68 delcn = 0.38
3500.0
3000.0
2500.0
2000.0
1500.0
1000.0
500.0
0.0
200.00
300.00
400.00
500.00
600.00
700.00
800.00
900.00
1000.00
1100.00
y2
b2-NH3
b2
y3
b5+b5++
b6++-NH3
y4
b3
y5-H2O
y5
b4-NH3
b4
y10++
b11++-NH3
y6
y11++
b5-H2O
b5
b12++-NH3
y12++-H2O
y12++-NH3, Par_m/z-H2O, Par_m/z-NH3
y7-NH3
y7
b6
y8-NH3
y8
b7-NH3
b7
y9-NH3
y9

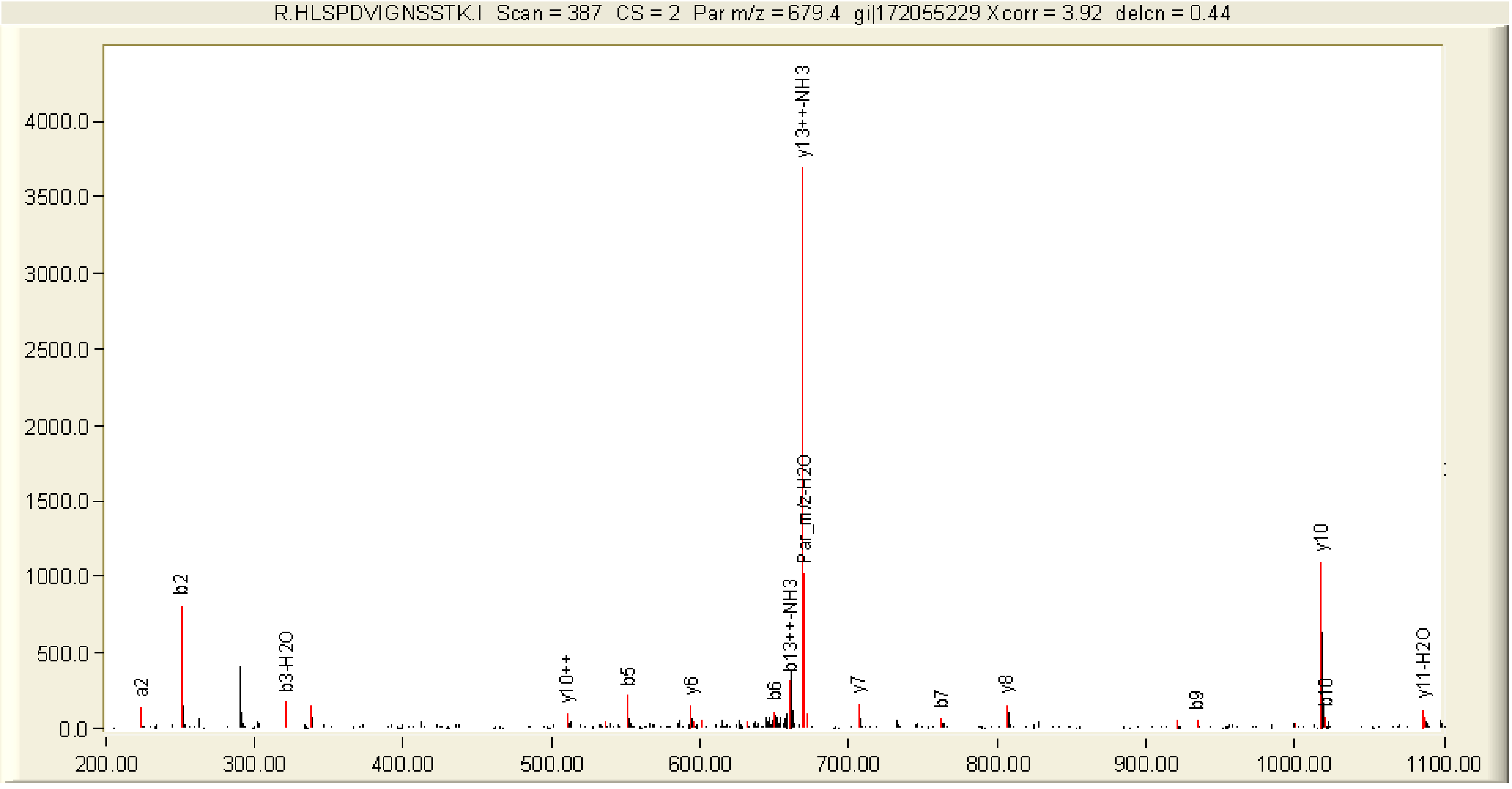
R.HLSPDVIGNSSTK.I Scan = 387 CS = 2 Par m/z = 679.4 gi|172055229 Xcorr = 3.92 delcn = 0.44
4000.0
3500.0
3000.0
2500.0
2000.0
1500.0
1000.0
500.0
0.0
200.00
300.00
400.00
500.00
600.00
700.00
800.00
900.00
1000.00
1100.00
y13++-NH3
Par_m/z-H2O
b13++-NH3
a2
b2
b3-H2O
y10++
b5
y6
b6
y7
b7
y8
b9
y10
b10
y11-H2O

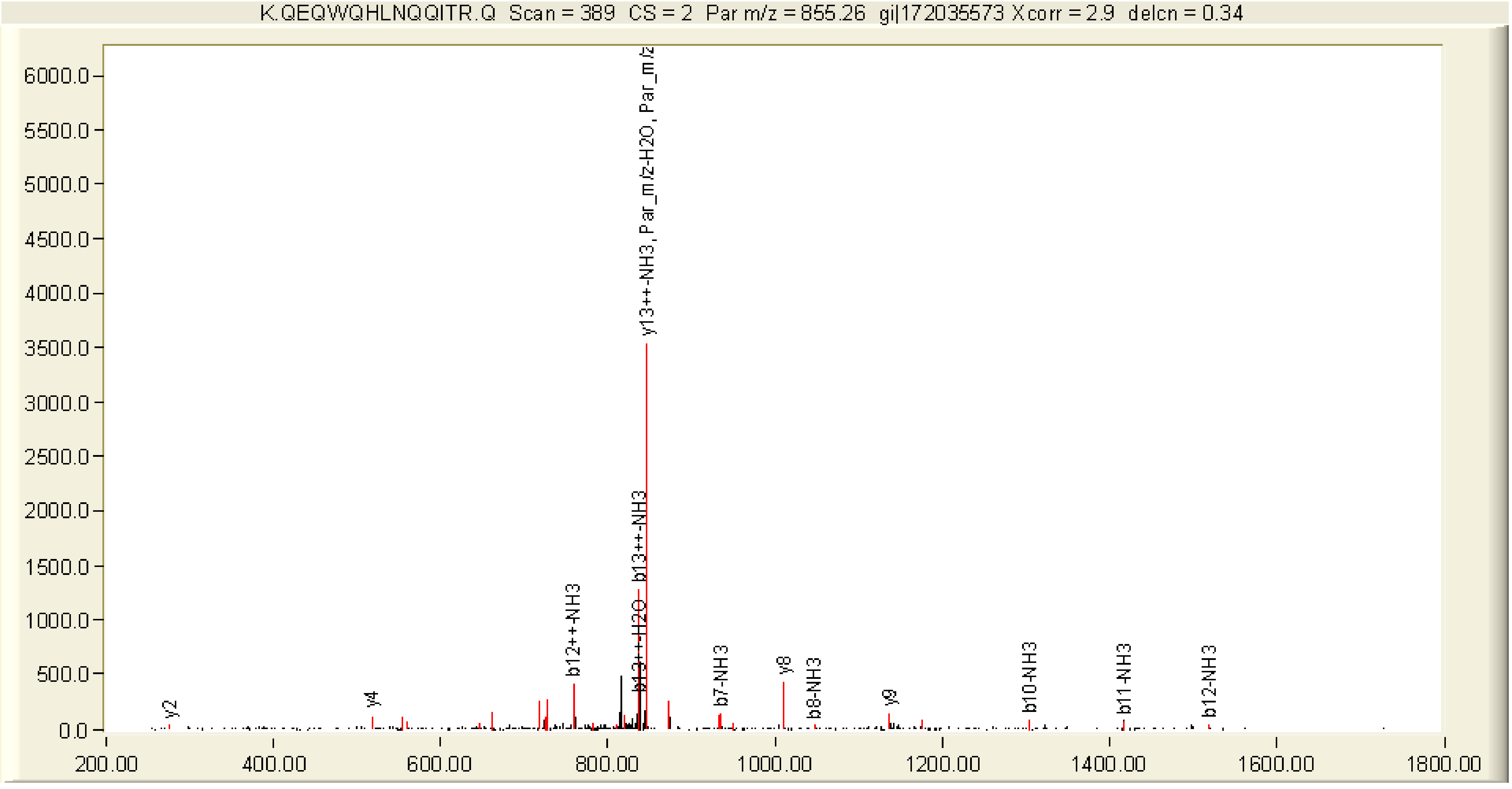
K.QEQWQHLNQQITR.Q Scan = 389 CS = 2 Par m/z = 855.26 gi|172035573 Xcorr = 2.9 delcn = 0.34
6000.0
5500.0
5000.0
4500.0
4000.0
3500.0
3000.0
2500.0
2000.0
1500.0
1000.0
500.0
0.0
200.00
400.00
600.00
800.00
1000.00
1200.00
1400.00
1600.00
1800.00
y13++-NH3, Par_m/z-H2O, Par_m/z
b13++-NH3
b13++-H2O
b12++-NH3
y2
y4
b7-NH3
y8
b8-NH3
y9
b10-NH3
b11-NH3
b12-NH3

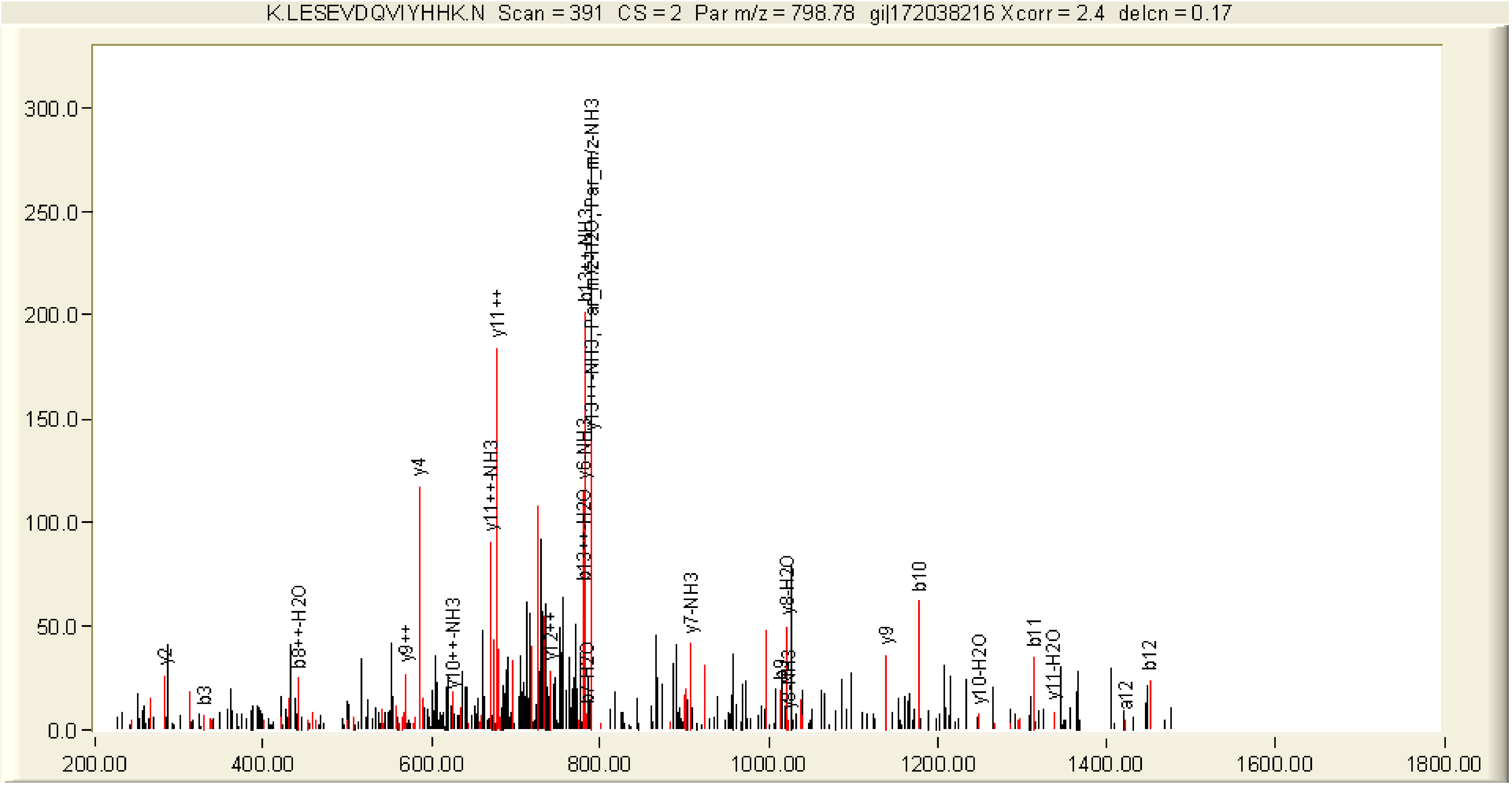
K.LESEVDQVIYHHK.N Scan = 391 CS = 2 Par m/z = 798.78 gi|172038216 Xcorr = 2.4 delcn = 0.17
300.0
250.0
200.0
150.0
100.0
50.0
0.0
200.00
400.00
600.00
800.00
1000.00
1200.00
1400.00
1600.00
1800.00
y2
b3
b8++-H2O
y9++
y4
y10++-NH3
y11++-NH3
y11++
y12++
b13++-H2O
y6-NH3
y7-NH3
y8-H2O
y9
b10
y10-H2O
b11
y11-H2O
a12
b12

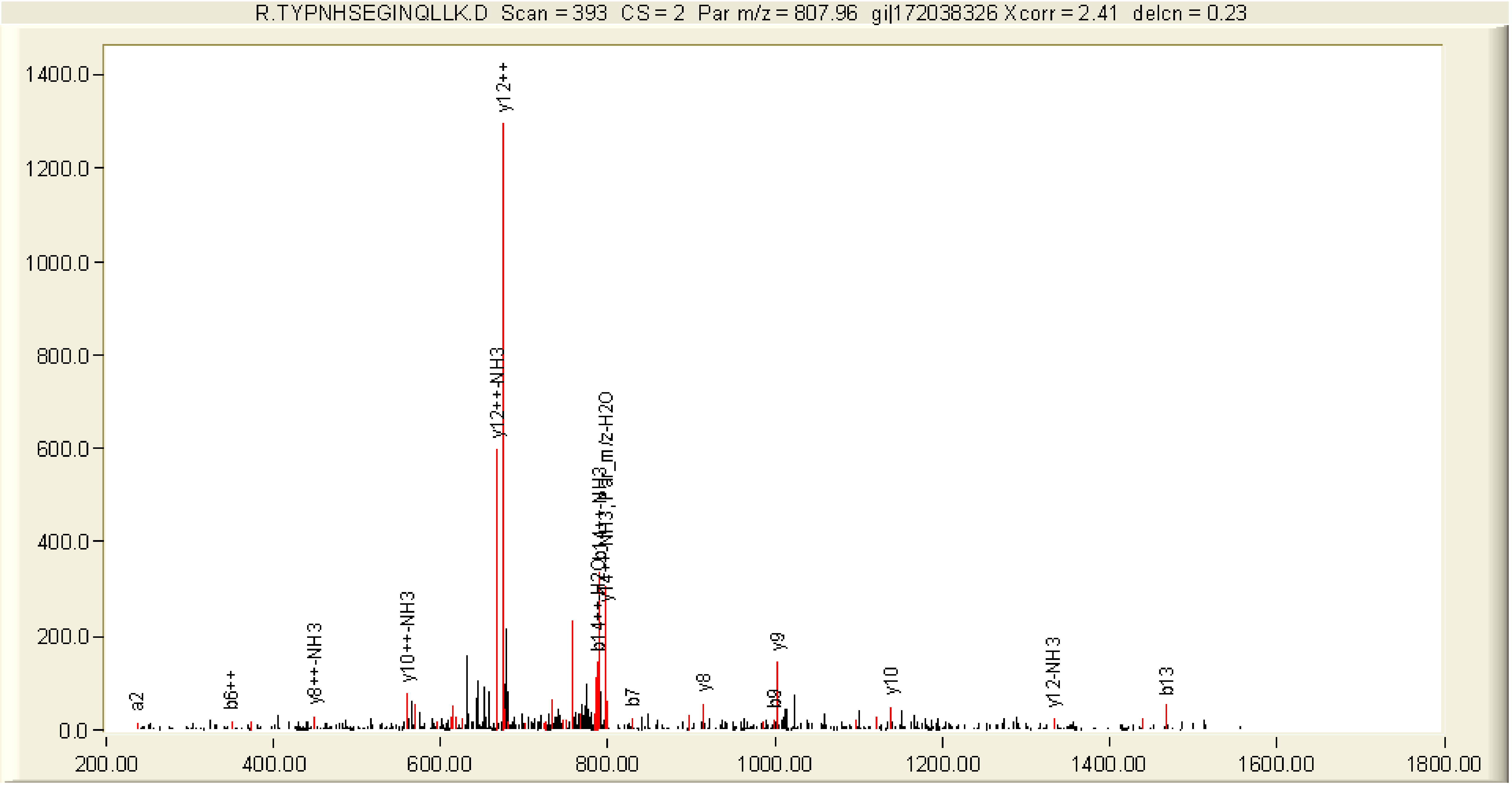
R.TYPNHSEGINQLLK.D Scan = 393 CS = 2 Par m/z = 807.96 gi|172038326 Xcorr = 2.41 delcn = 0.23
y12++
y12++-NH3
y10++-NH3
y8++-NH3
b6++
a2
b7
y8
y9
b9
y10
y12-NH3
b13
1400.0
1200.0
1000.0
800.0
600.0
400.0
200.0
0.0
200.00
400.00
600.00
800.00
1000.00
1200.00
1400.00
1600.00
1800.00

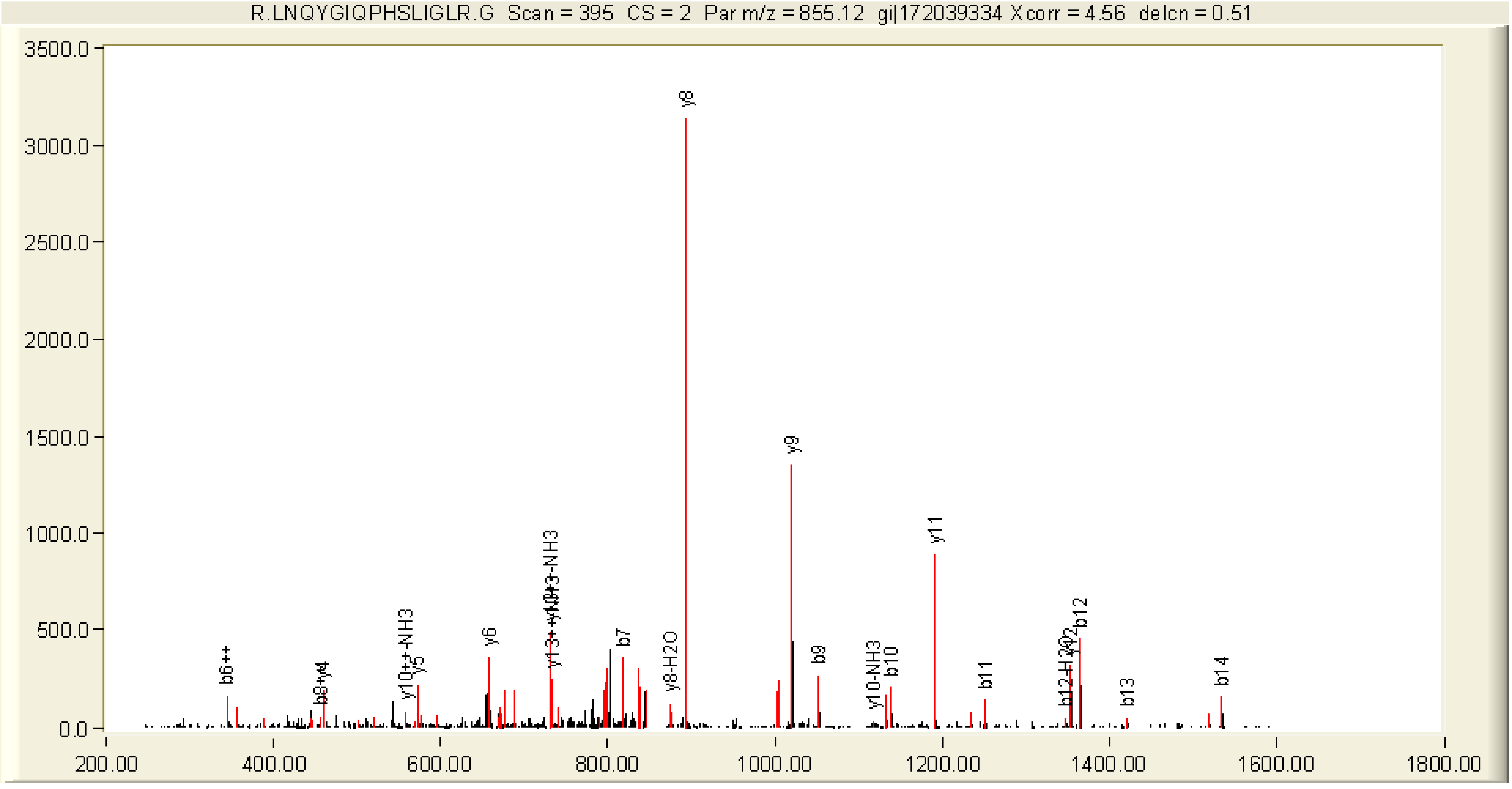
R.LNQYGIQPHSLIGLR.G Scan = 395 CS = 2 Par m/z = 855.12 gi|172039334 Xcorr = 4.56 delcn = 0.51
3500.0
3000.0
2500.0
2000.0
1500.0
1000.0
500.0
0.0
200.00
400.00
600.00
800.00
1000.00
1200.00
1400.00
1600.00
1800.00
y8
y9
y11
b12
b14
b13
b11
b10
y10-NH3
b9
y8-H2O
b7
y6
y5
b6++
b12-H2O
y13++-NH3

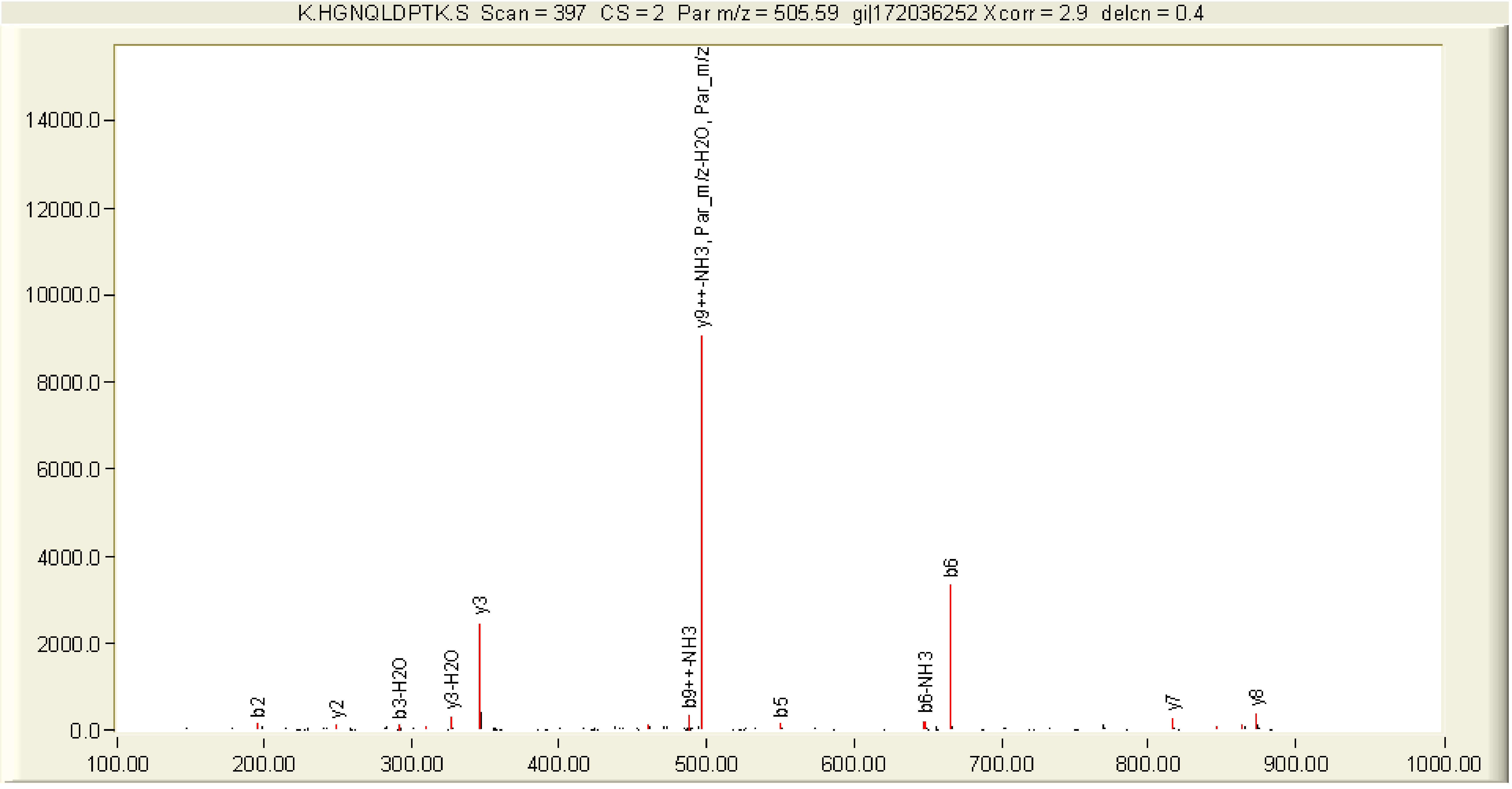

K.HGNQLDPTK.S Scan = 397 CS = 2 Par m/z = 505.59 gi|172036252 Xcorr = 2.9 delcn = 0.4
14000.0
12000.0
10000.0
8000.0
6000.0
4000.0
2000.0
0.0
100.00
200.00
300.00
400.00
500.00
600.00
700.00
800.00
900.00
1000.00
y9++-NH3, Par_m/z-H2O, Par_m/z
b6
y3
b2
y2
b3-H2O
y3-H2O
b9++-NH3
b5
b6-NH3
y7
y8

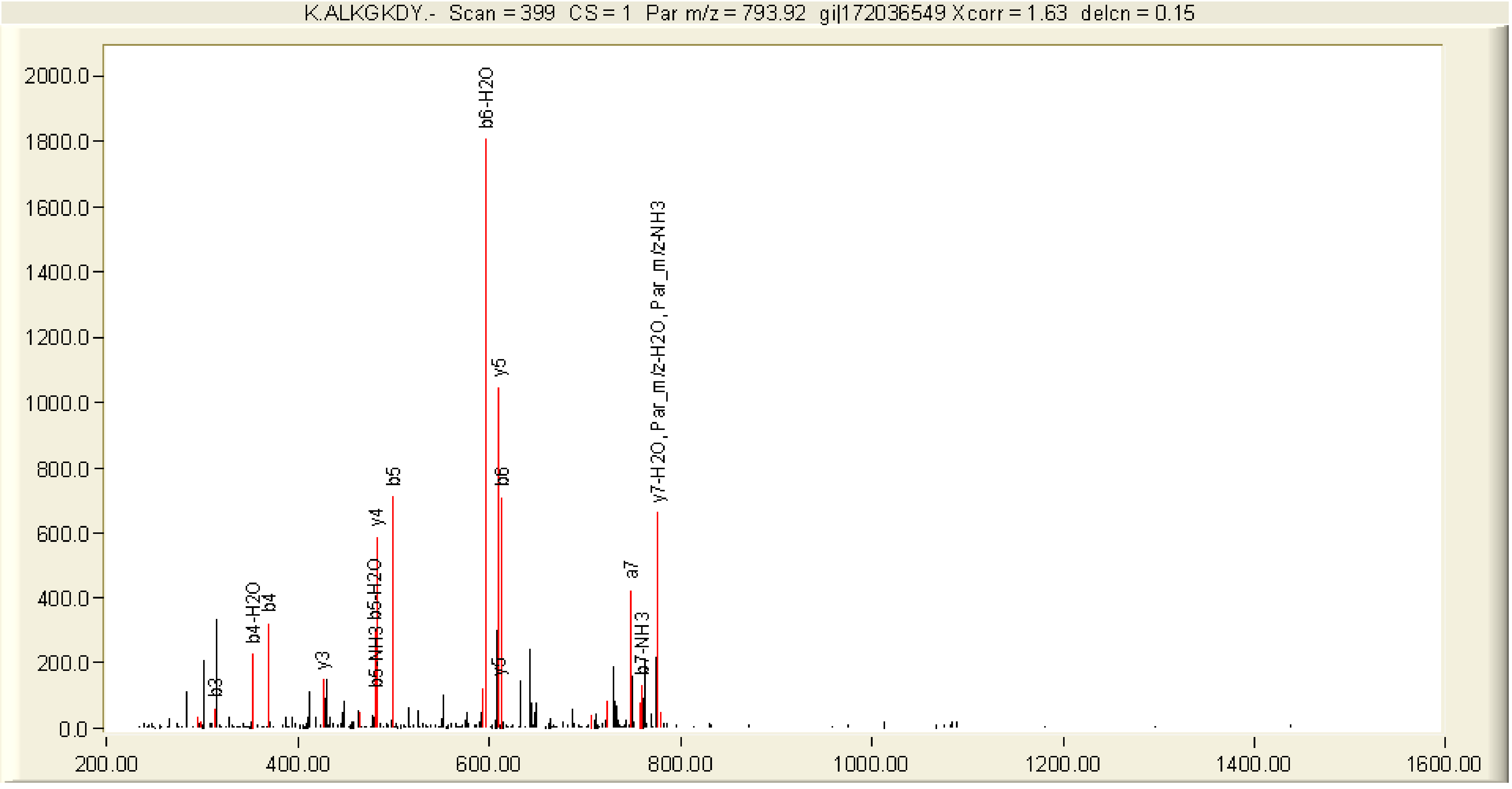

K.ALKGKDY.- Scan = 399 CS = 1 Par m/z = 793.92 gi|172036549 Xcorr = 1.63 delcn = 0.15
2000.0
1800.0
1600.0
1400.0
1200.0
1000.0
800.0
600.0
400.0
200.0
0.0
200.00
400.00
600.00
800.00
1000.00
1200.00
1400.00
1600.00
b6-H2O
y5
b6
b5
y4
b5-NH3 b5-H2O
b4-H2O
b4
b3
y3
y5
a7
b7-NH3
y7-H2O, Par_m/z-H2O, Par_m/z-NH3

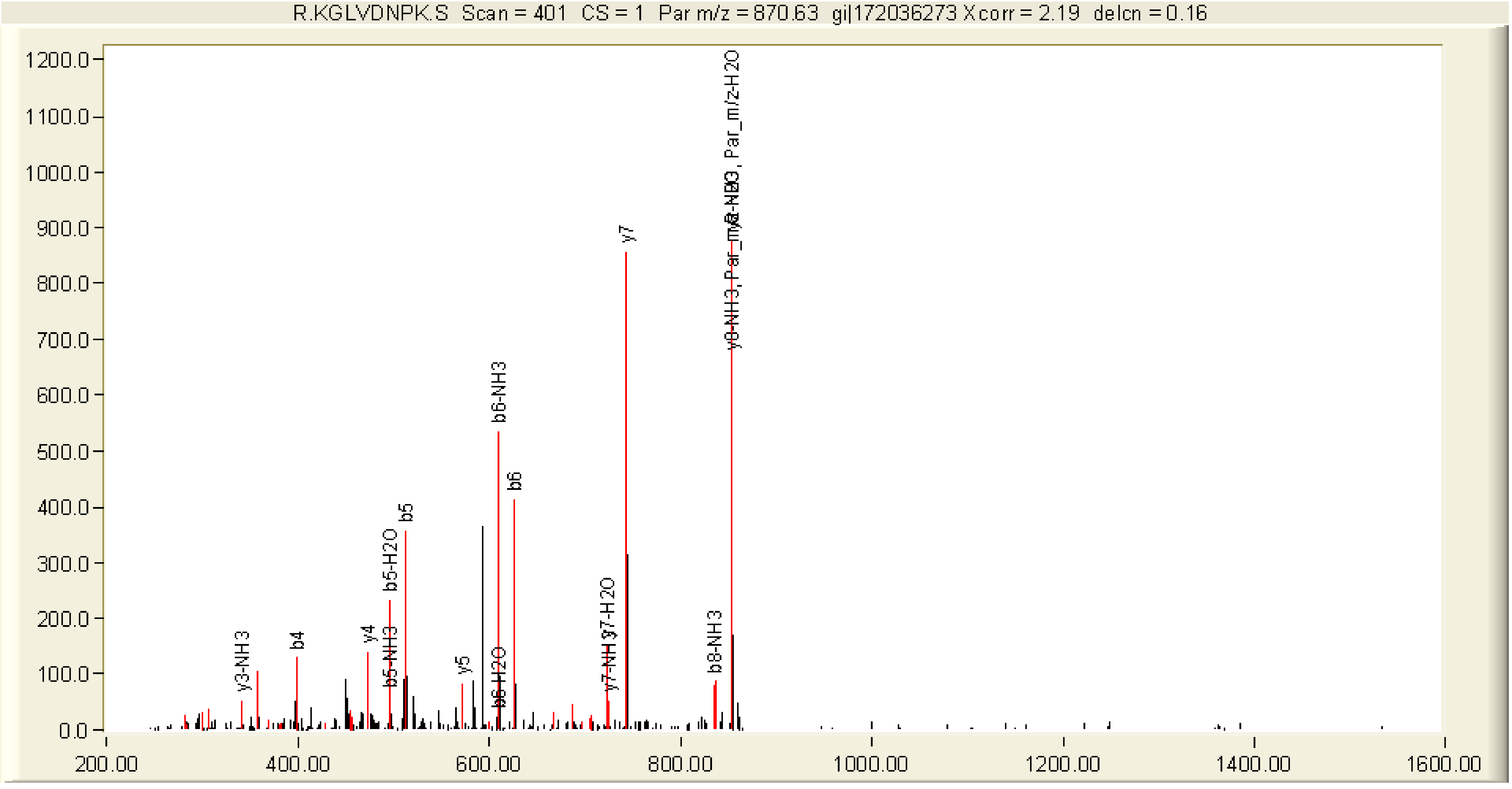
R.KGLVDNPK.S Scan = 401 CS = 1 Par m/z = 870.63 gi|172036273 Xcorr = 2.19 delcn = 0.16
y3-NH3
b4
y4
b5-NH3
b5-H2O
b5
y5
b6-H2O
b6-NH3
b6
y7-NH3
y7-H2O
y7
b8-NH3
Par_m/z-H2O

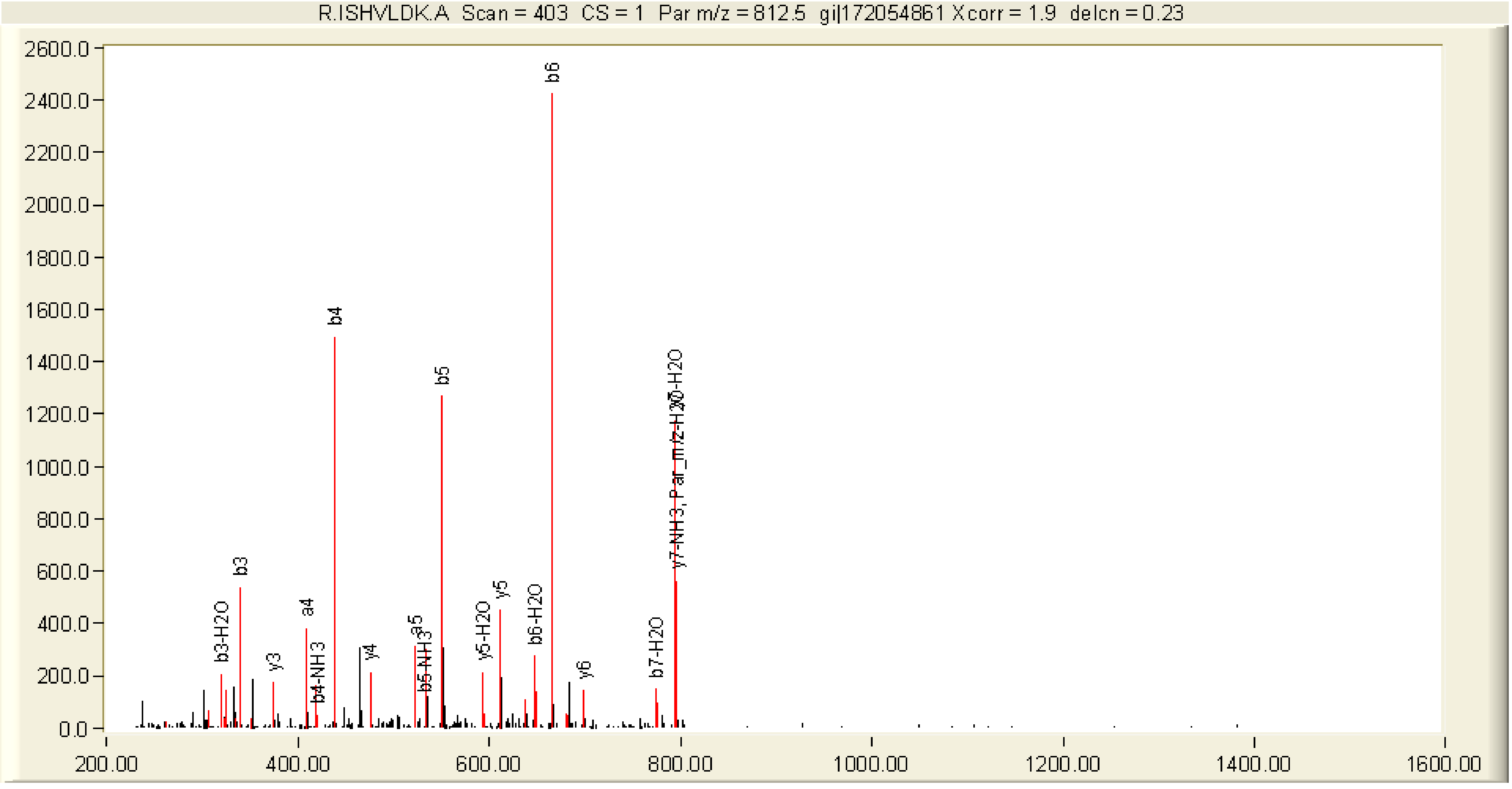

R.ISHVLDK.A Scan = 403 CS = 1 Par m/z = 812.5 gi|172054861 Xcorr = 1.9 delcn = 0.23
2600.0
2400.0
2200.0
2000.0
1800.0
1600.0
1400.0
1200.0
1000.0
800.0
600.0
400.0
200.0
0.0
200.00
400.00
600.00
800.00
1000.00
1200.00
1400.00
1600.00
b6
b4
b5
b3
b3-H2O
y3
a4
b4-NH3
y4
a5
b5-NH3
y5-H2O
y5
b6-H2O
y6
b7-H2O
y7-NH3, Par_m/z-H2O

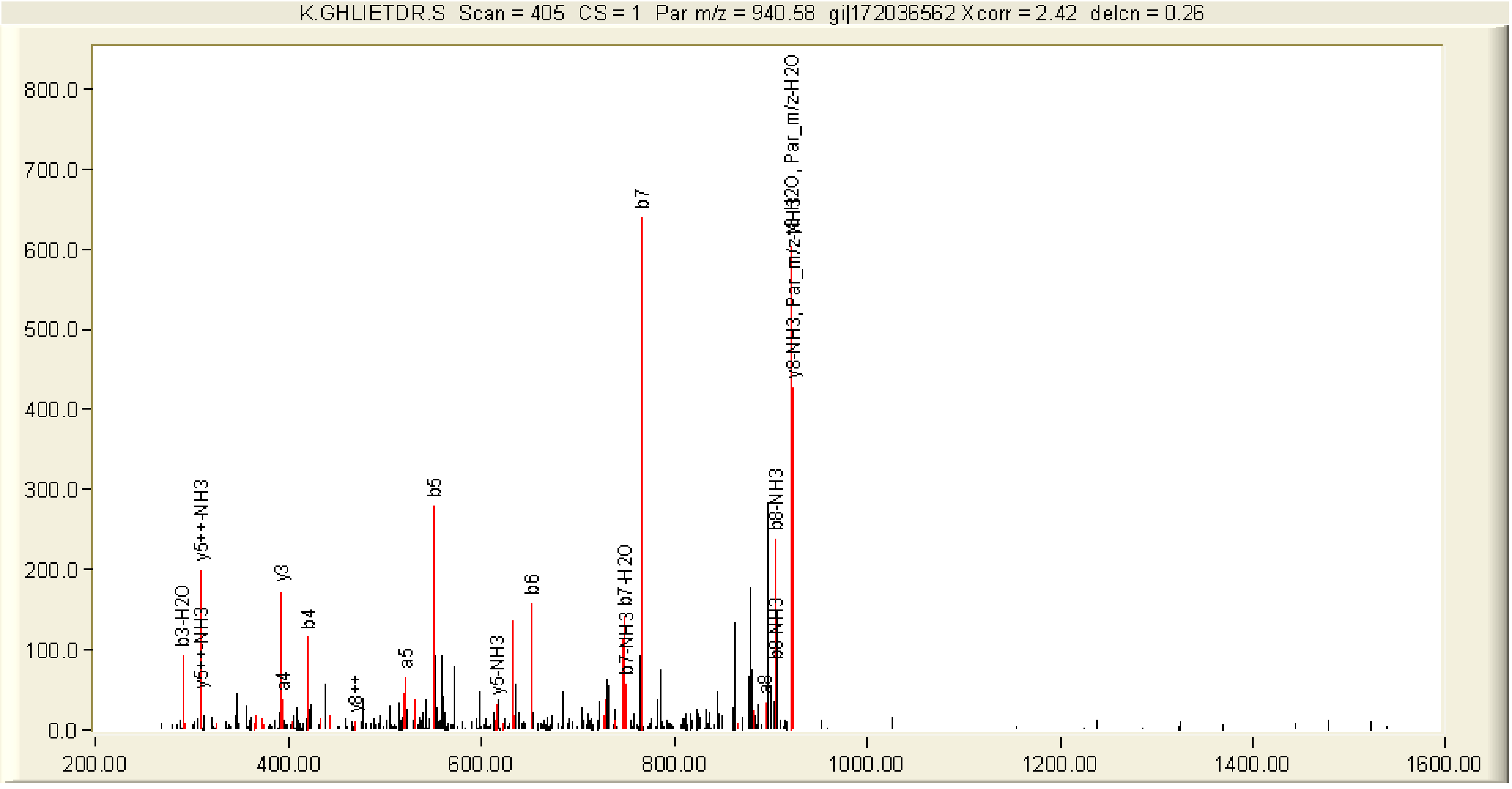
K.GHLIETDR.S Scan = 405 CS = 1 Par m/z = 940.58 gi|172036562 Xcorr = 2.42 delcn = 0.26
800.0
700.0
600.0
500.0
400.0
300.0
200.0
100.0
0.0
200.00
400.00
600.00
800.00
1000.00
1200.00
1400.00
1600.00
b3-H2O
y5++-NH3
y5++-NH3
y3
a4
b4
y8++
a5
b5
y5-NH3
b6
b7-NH3
b7-H2O
b7
a8
b8-NH3
b8-NH3
y8-NH3, Par_m/z-H2O, Par_m/z-H2O

K.KSQFSEQENSIK.S Scan = 407 CS = 2 Par m/z = 713.63 gi|172038439 Xcorr = 2.47 delcn = 0.19

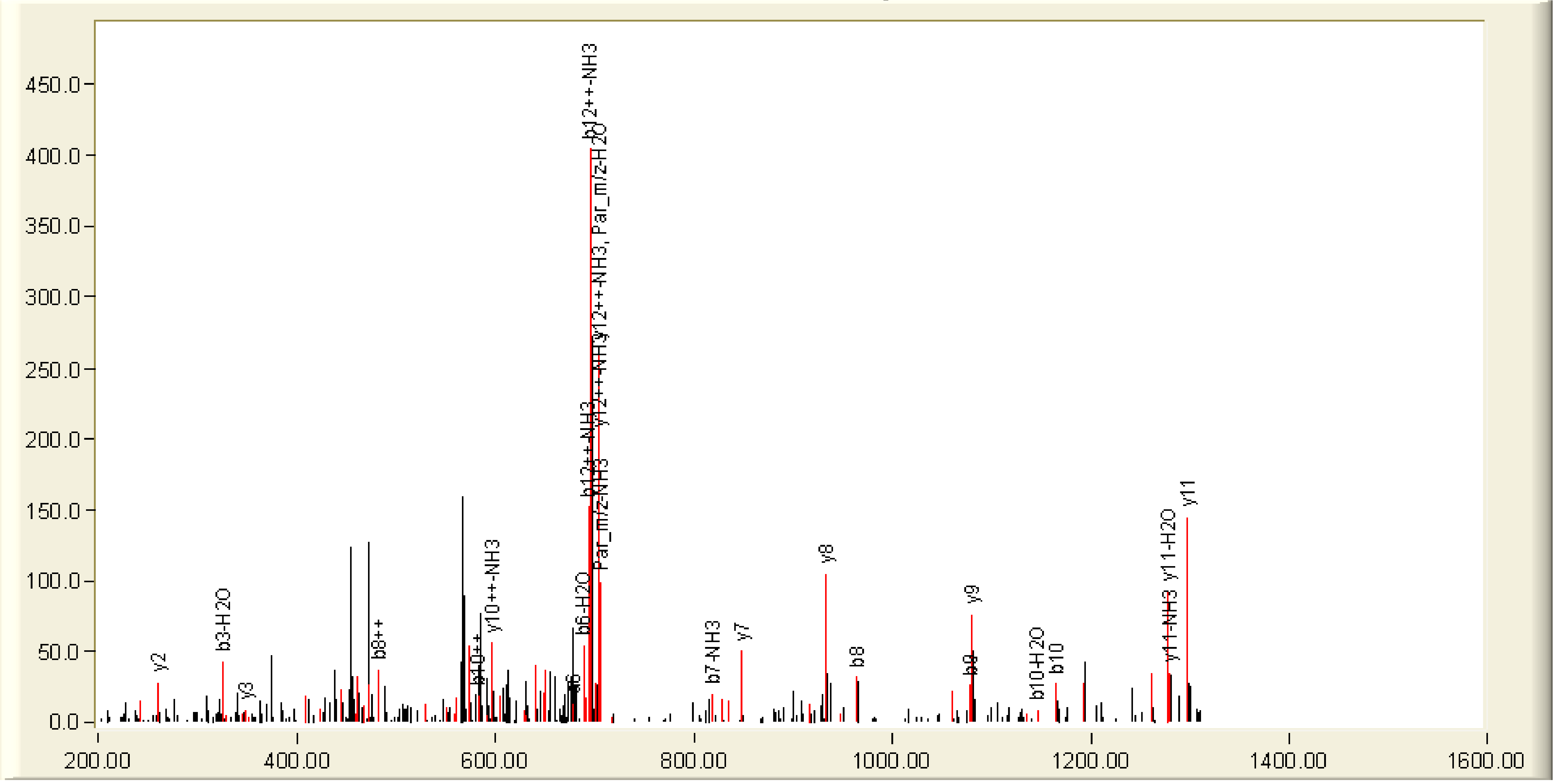

R.HLVESGVVSR.Q Scan = 409 CS = 1 Par m/z = 1082.68 gi|172035453 Xcorr = 2.8 delcn = 0.32

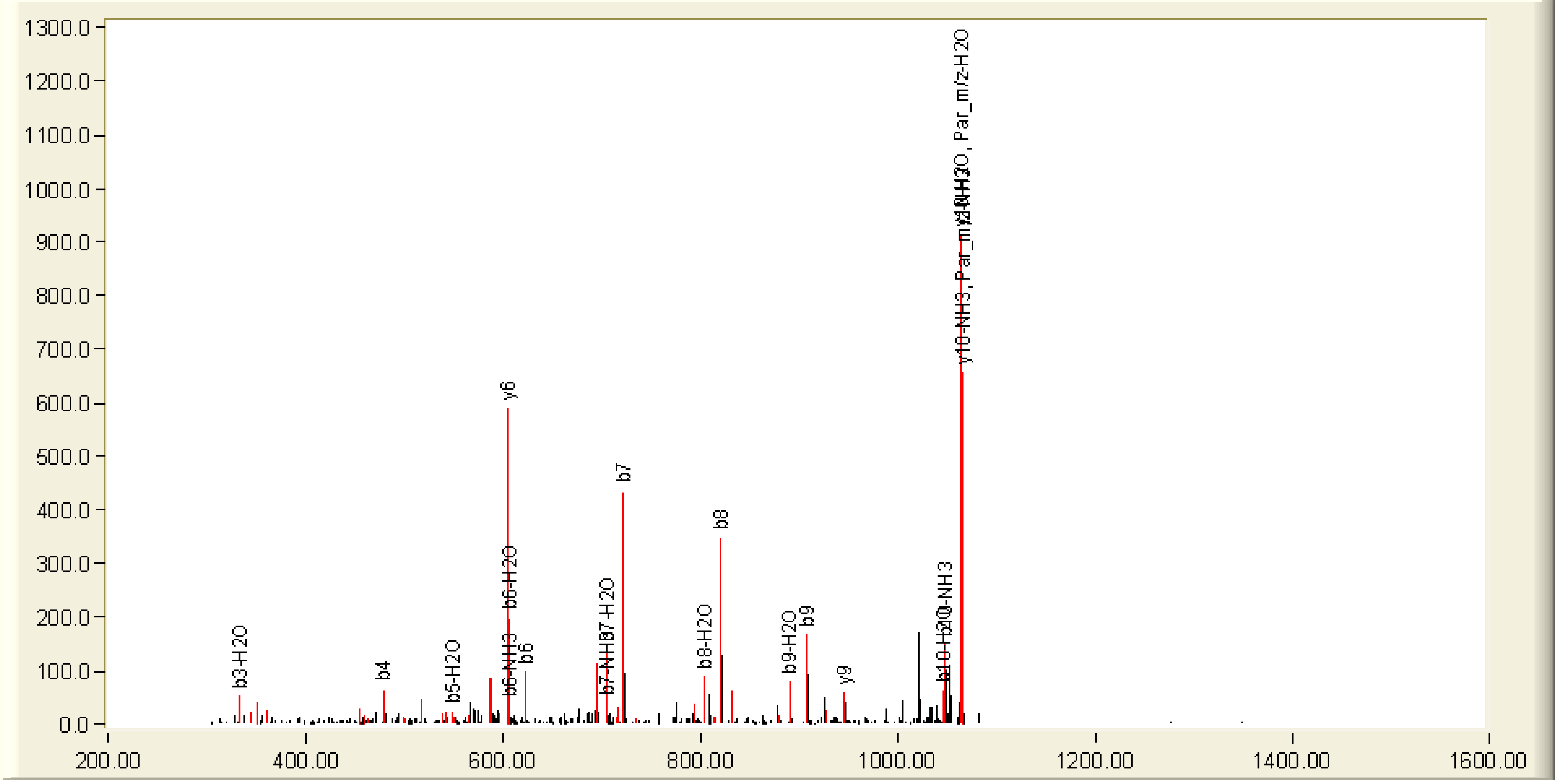

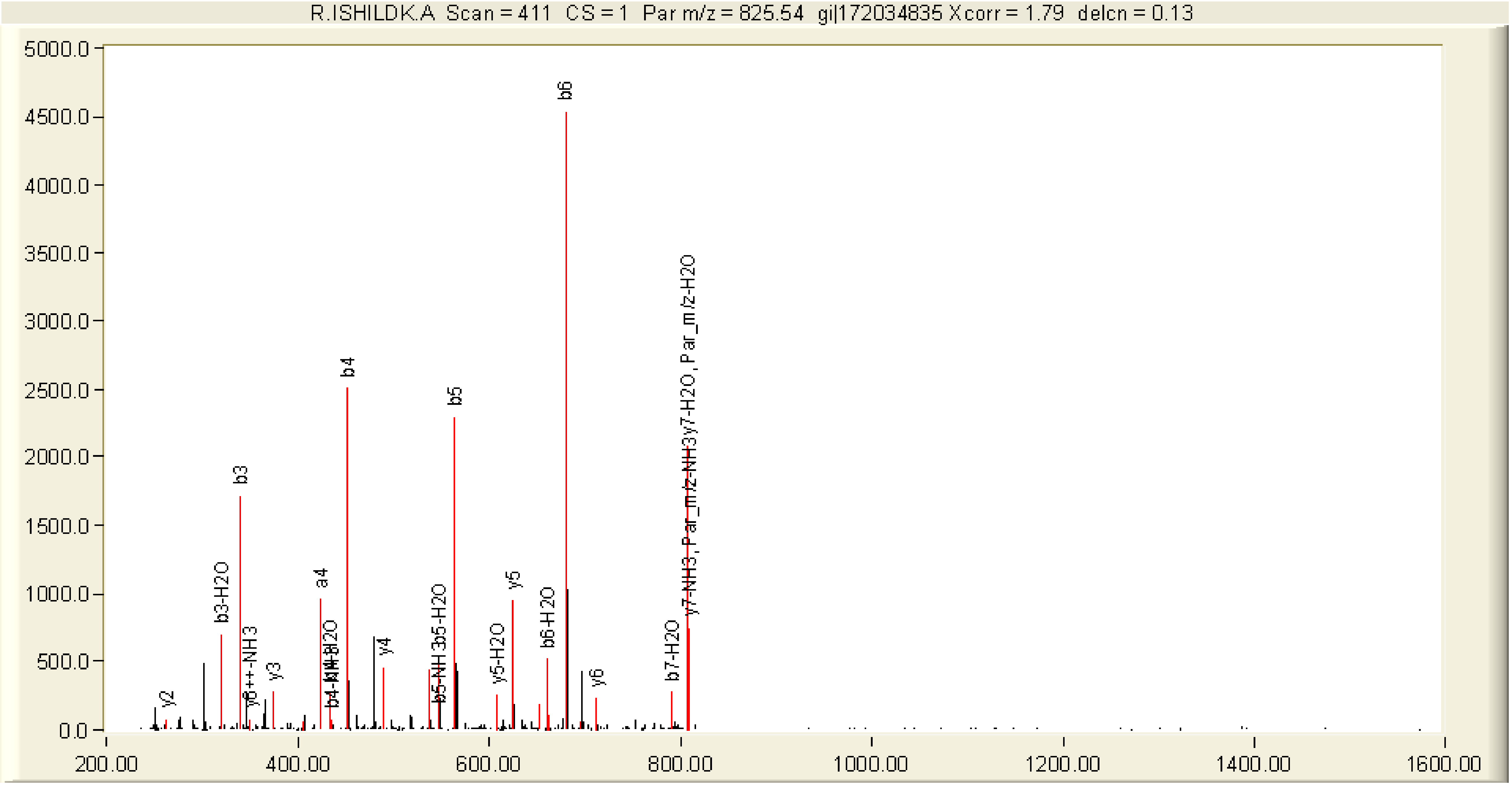
R.ISHILDK.A Scan = 411 CS = 1 Par m/z = 825.54 gi|172034835 Xcorr = 1.79 delcn = 0.13
5000.0
4500.0
4000.0
3500.0
3000.0
2500.0
2000.0
1500.0
1000.0
500.0
0.0
200.00
400.00
600.00
800.00
1000.00
1200.00
1400.00
1600.00
b6
b4
b5
b3
b3-H2O
a4
y5
b6-H2O
y7-NH3, Par_m/z-NH3 y7-H2O, Par_m/z-H2O
b5-H2O
y5-H2O
b7-H2O
y4
y3
y2
y6
b5-NH3

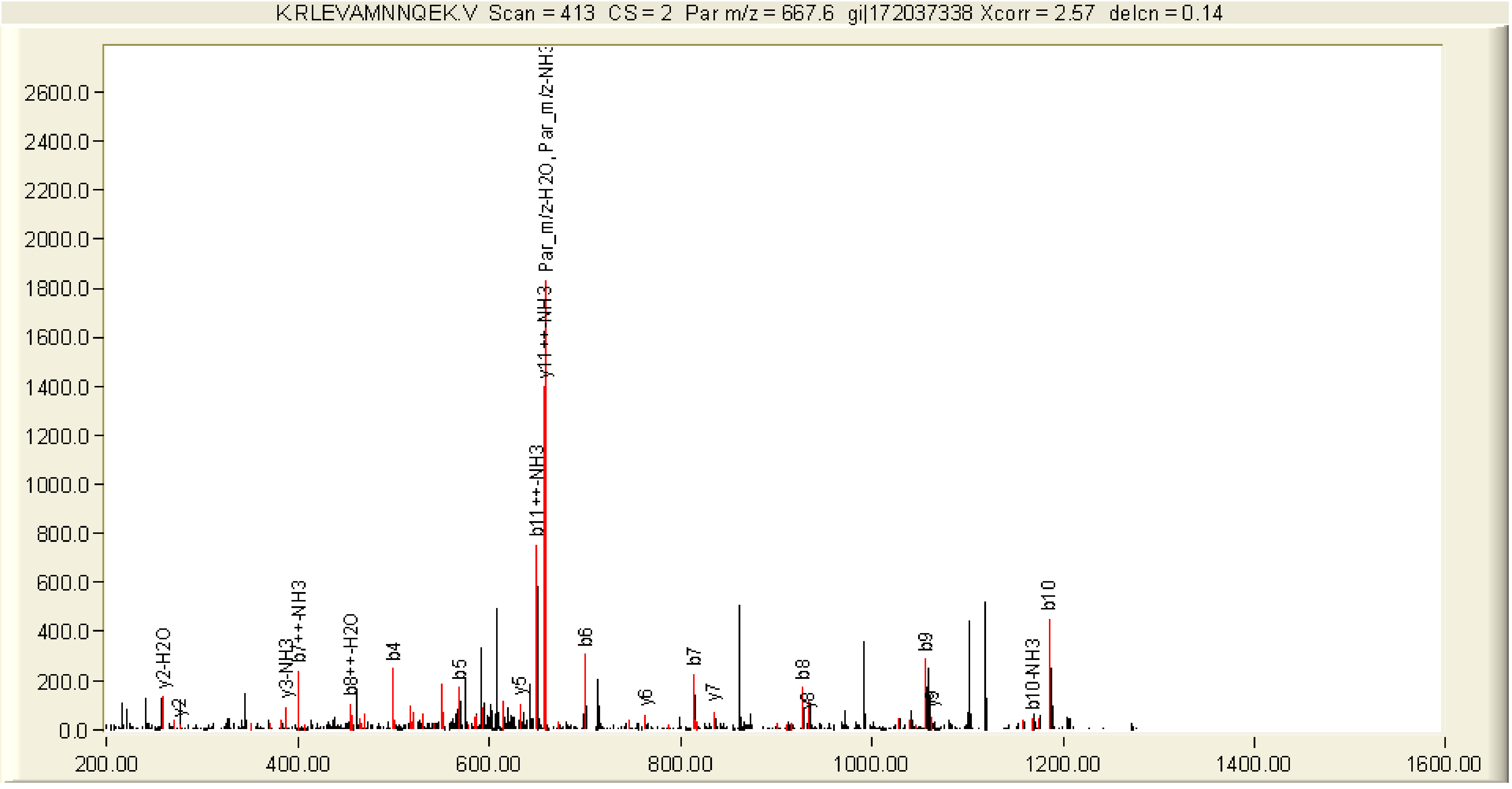

K.RLEVAMNNQEK.V Scan = 413 CS = 2 Par m/z = 667.6 gi|172037338 Xcorr = 2.57 delcn = 0.14
2600.0
2400.0
2200.0
2000.0
1800.0
1600.0
1400.0
1200.0
1000.0
800.0
600.0
400.0
200.0
0.0
200.00
400.00
600.00
800.00
1000.00
1200.00
1400.00
1600.00
y2-H2O
y2
y3-NH3
b7++-NH3
b8++-H2O
b4
b5
y5
b11++-NH3
y11++-NH3
Par_m/z-H2O, Par_m/z-NH3
b6
y6
b7
y7
b8
y8
b9
y9
b10-NH3
b10

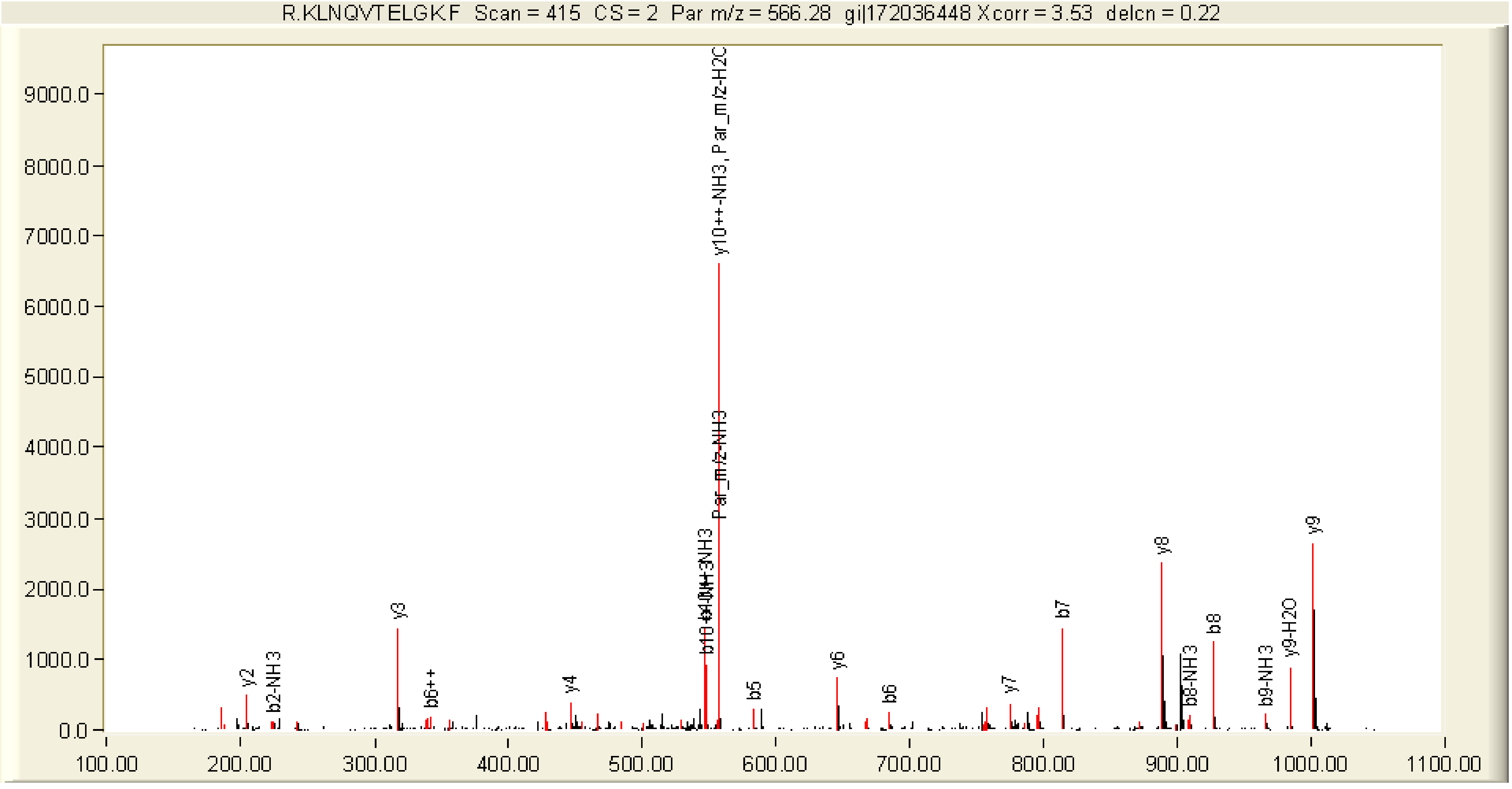
R.KLNQVTELGKF Scan = 415 CS = 2 Par m/z = 566.28 gi|172036448 Xcorr = 3.53 delcn = 0.22
9000.0
8000.0
7000.0
6000.0
5000.0
4000.0
3000.0
2000.0
1000.0
0.0
100.00
200.00
300.00
400.00
500.00
600.00
700.00
800.00
900.00
1000.00
1100.00
y10++-NH3, Par_m/z-H2O
Par_m/z-NH3
y2
b2-NH3
y3
b6++
y4
b5
y6
b6
y7
b7
y8
b8-NH3
b8
b9-NH3
y9-H2O
y9

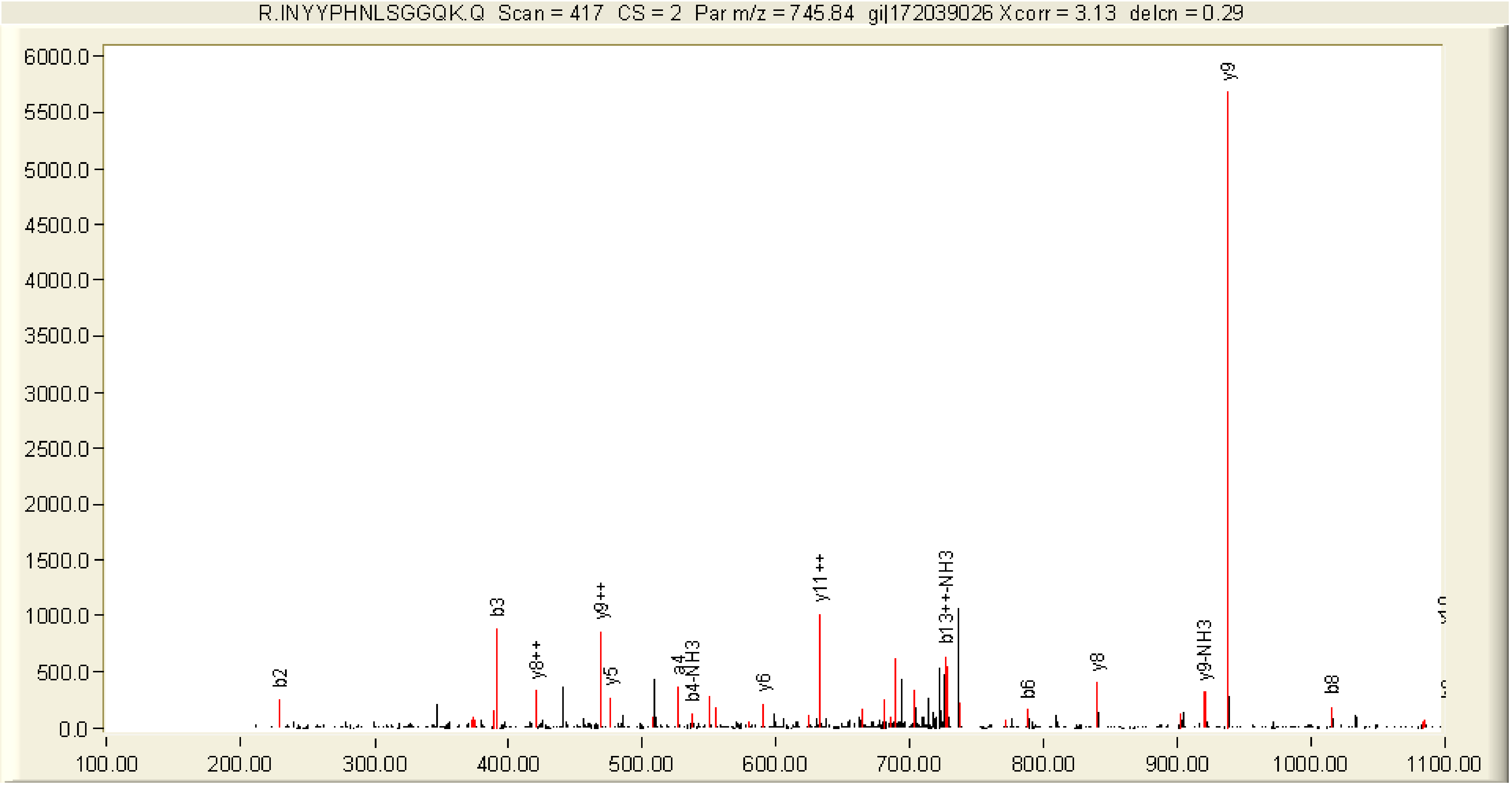

R.INYYPHNLSGGQK.Q Scan = 417 CS = 2 Par m/z = 745.84 gi|172039026 Xcorr = 3.13 delcn = 0.29
6000.0
5500.0
5000.0
4500.0
4000.0
3500.0
3000.0
2500.0
2000.0
1500.0
1000.0
500.0
0.0
100.00
200.00
300.00
400.00
500.00
600.00
700.00
800.00
900.00
1000.00
1100.00
b2
b3
y8++
y9++
y5
a4
b4-NH3
y6
y11++
b13++-NH3
b6
y8
y9-NH3
y9
b8

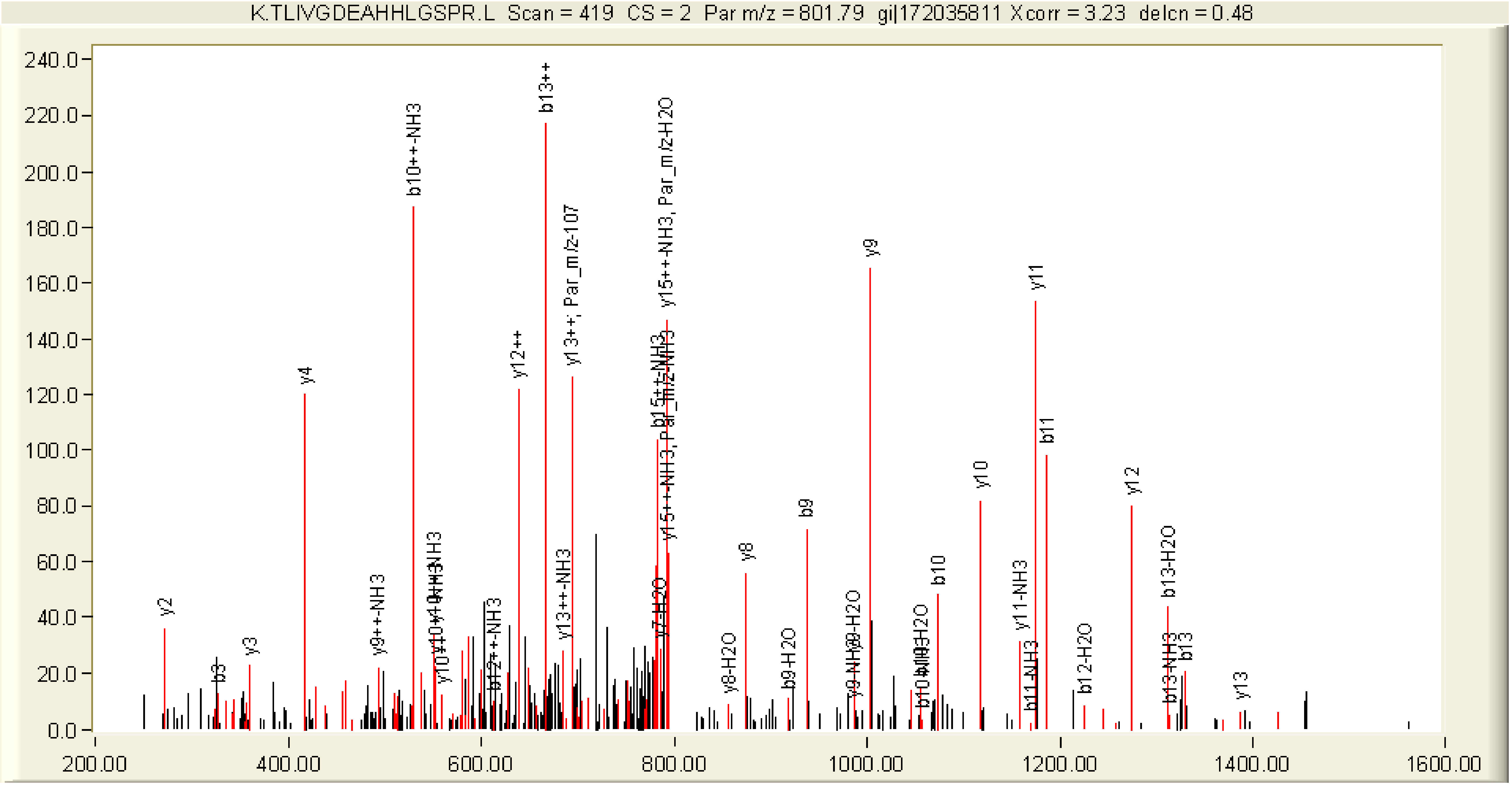
K.TLIVGDEAHHLGSPR.L Scan = 419 CS = 2 Par m/z = 801.79 gi|172035811 Xcorr = 3.23 delcn = 0.48
240.0
220.0
200.0
180.0
160.0
140.0
120.0
100.0
80.0
60.0
40.0
20.0
0.0
200.00
400.00
600.00
800.00
1000.00
1200.00
1400.00
1600.00
y2
b3
y3
y4
y9++-NH3
b10++-NH3
y12++
b13++
y13++; Par_m/z-107
y13++-NH3
y15++-NH3, Par_m/z-H2O
y8
y8-H2O
b9-H2O
b9
y9
b10
y10
y11-NH3
y11
b11
b11-NH3
b12-H2O
y12
b13-H2O
b13-NH3
b13
y13

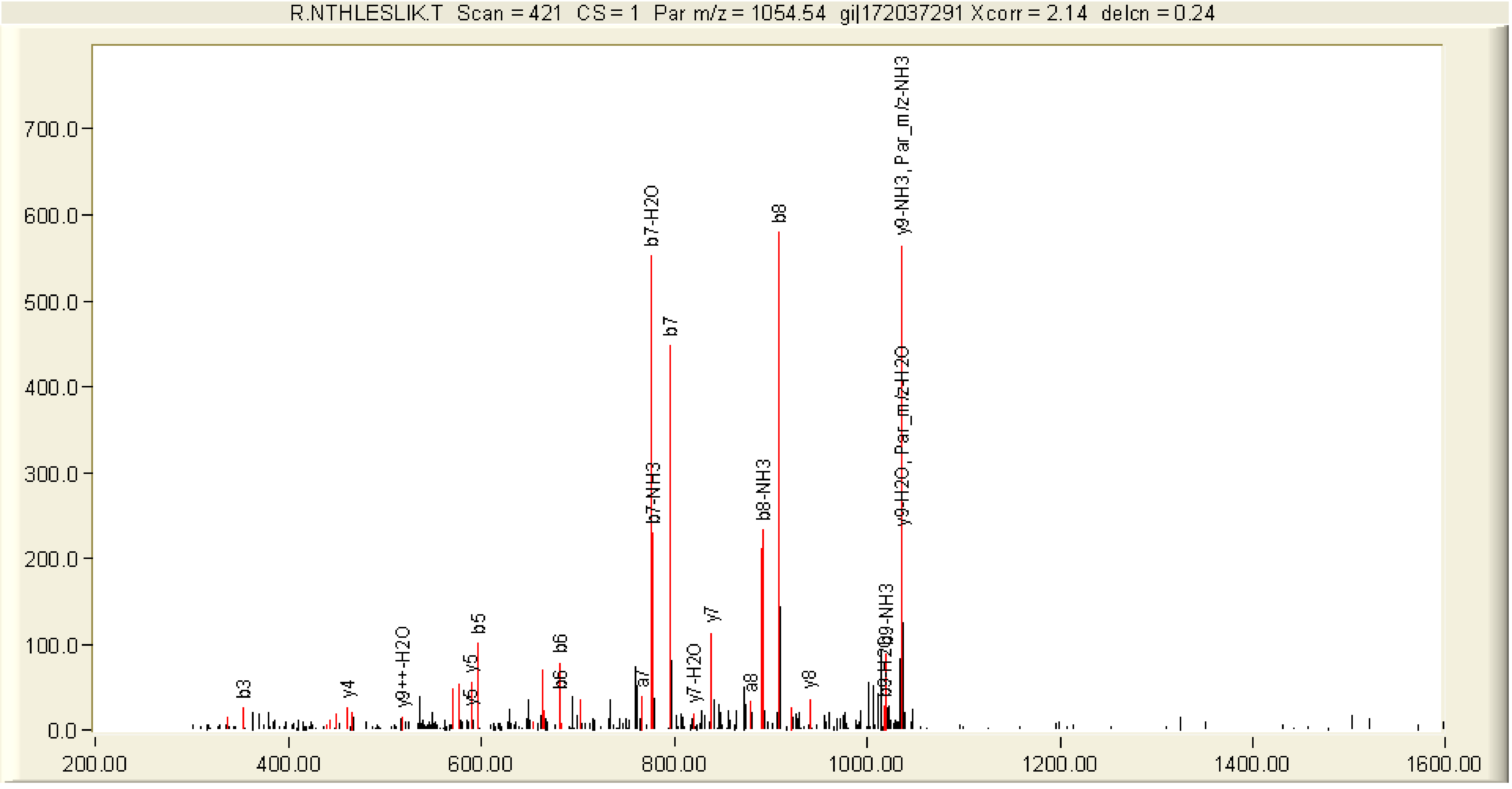
R.NTHLESLIK.T Scan = 421 CS = 1 Par m/z = 1054.54 gi|172037291 Xcorr = 2.14 delcn = 0.24
b3
y4
y9++-H2O
y5
b5
b6
a7
b7-NH3
b7-H2O
b7
y7-H2O
y7
a8
b8-NH3
b8
y8
y9-NH3
y9-H2O, Par_m/z-H2O
y9-NH3, Par_m/z-NH3
0.0
100.0
200.0
300.0
400.0
500.0
600.0
700.0
200.00
400.00
600.00
800.00
1000.00
1200.00
1400.00
1600.00

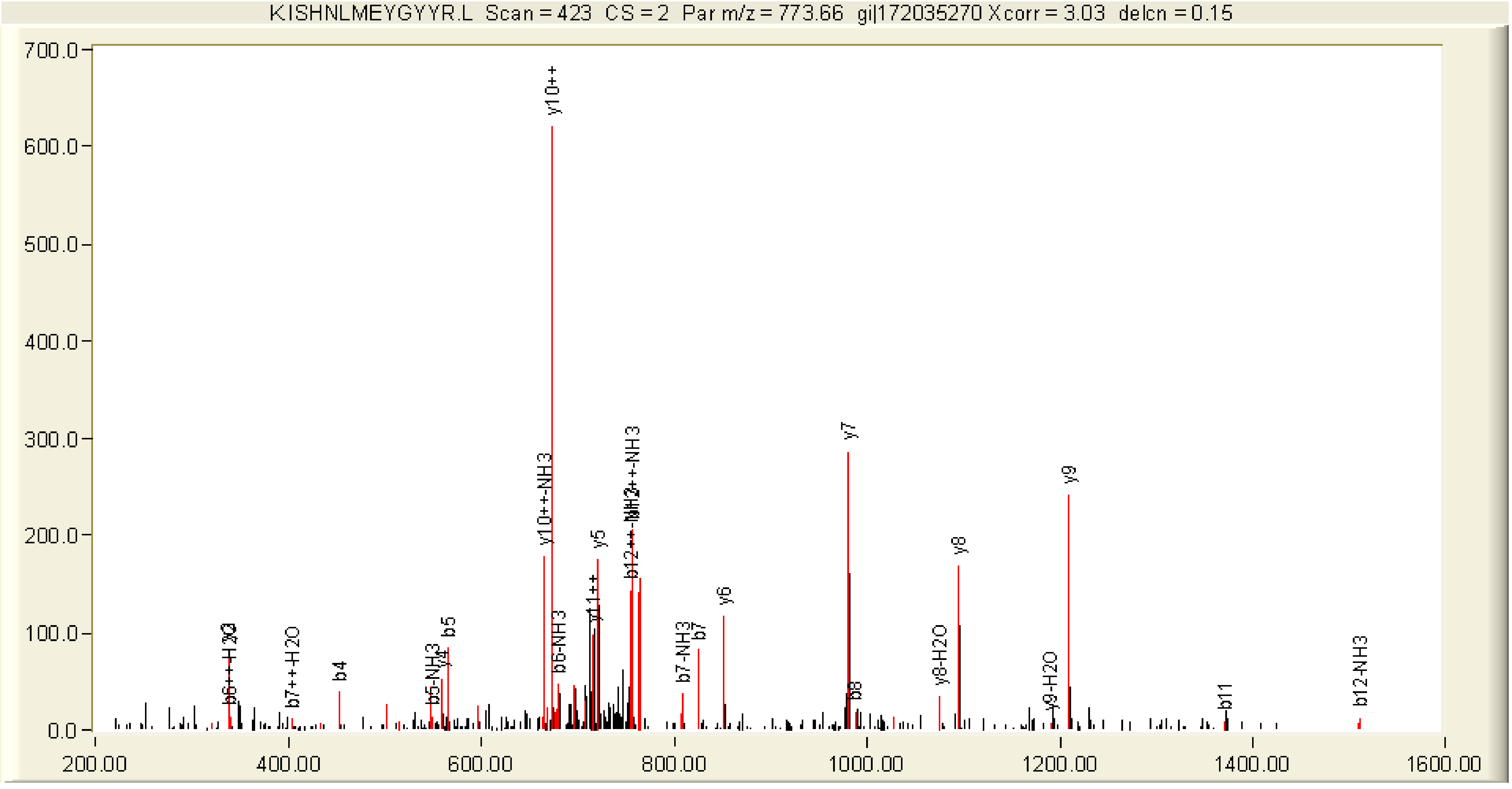

KISHNLMEYGYYR.L Scan = 423 CS = 2 Par m/z = 773.66 gi|172035270 Xcorr = 3.03 delcn = 0.15
700.0
600.0
500.0
400.0
300.0
200.0
100.0
0.0
200.00
400.00
600.00
800.00
1000.00
1200.00
1400.00
1600.00
y10++
y10++-NH3
y7
y9
y5
y8
y6
b5
b4
b7
b7-NH3
b6-NH3
b5-NH3
b7++-H2O
y8-H2O
y9-H2O
b8
b11
b12-NH3

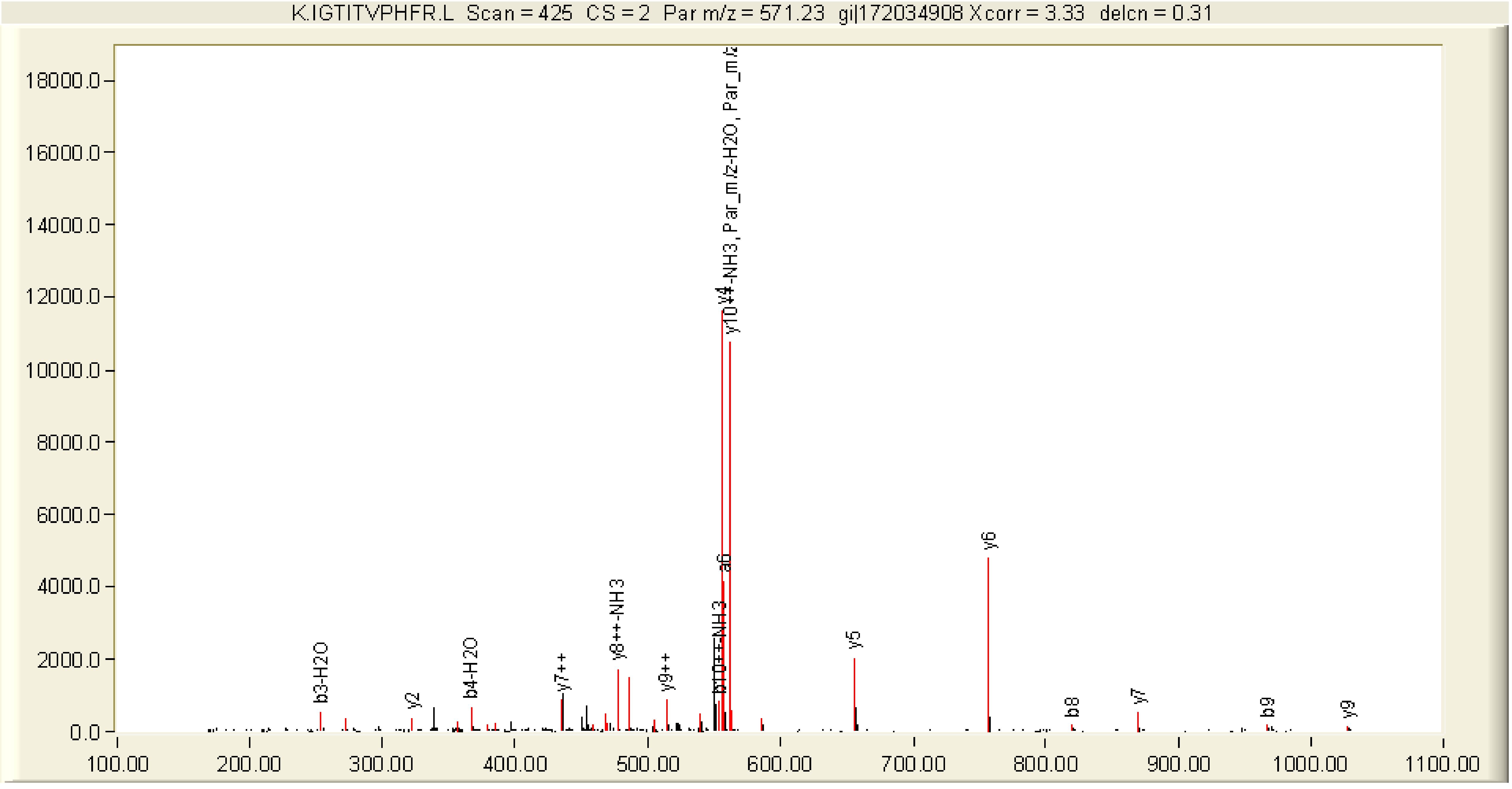
K.IGTITVPHFR.L Scan = 425 CS = 2 Par m/z = 571.23 gi|172034908 Xcorr = 3.33 delcn = 0.31
y10, y4-NH3, Par_m/z-H2O, Par_m/z
a6
y6
y5
b3-H2O
y2
b4-H2O
y7++
y8++-NH3
y9++
b10++-NH3
b8
y7
b9
y9
0.0
2000.0
4000.0
6000.0
8000.0
10000.0
12000.0
14000.0
16000.0
18000.0
100.00
200.00
300.00
400.00
500.00
600.00
700.00
800.00
900.00
1000.00
1100.00

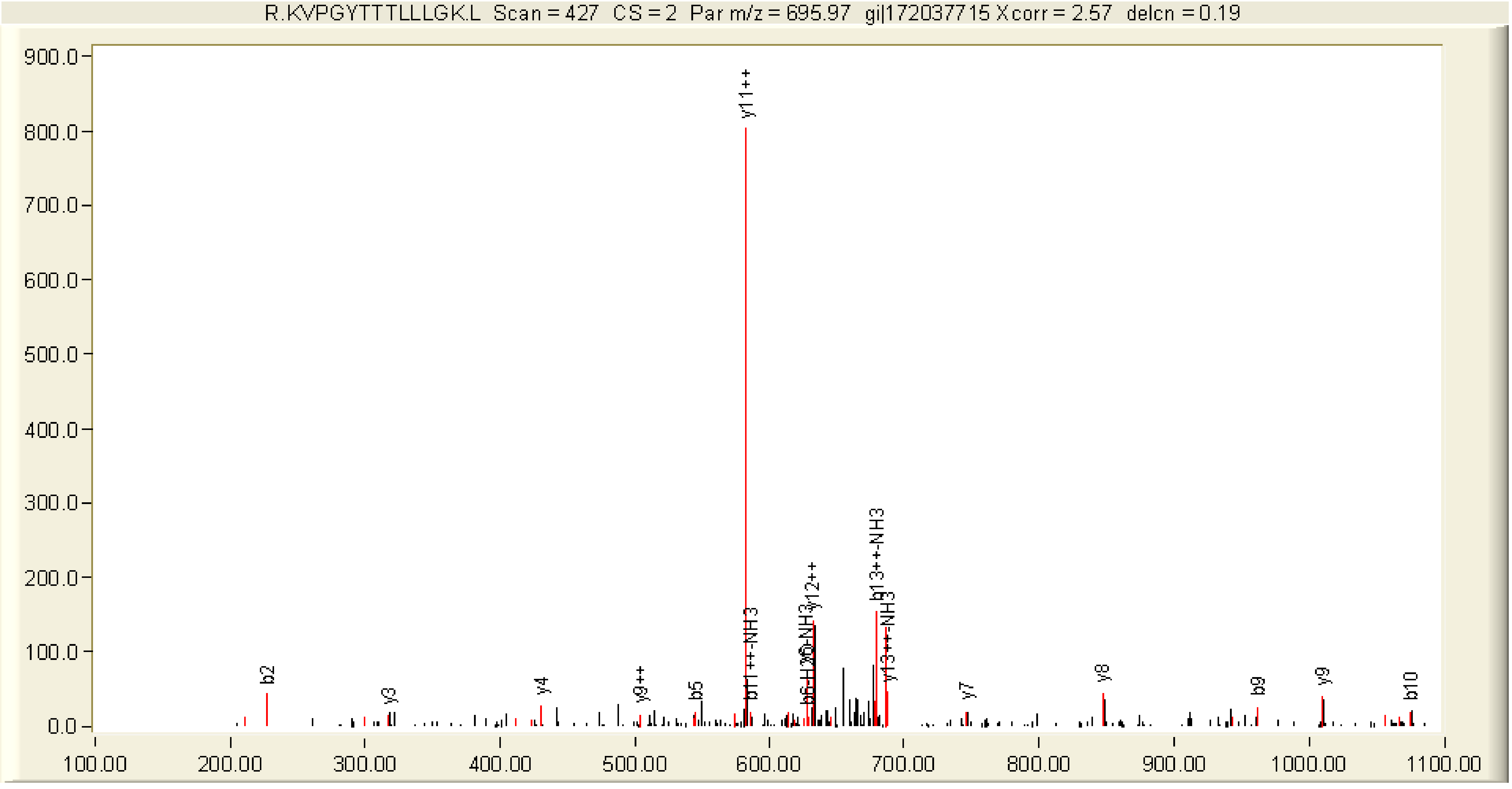

R.KVPGYTTTLLLGK.L Scan = 427 CS = 2 Par m/z = 695.97 gi|172037715 Xcorr = 2.57 delcn = 0.19
900.0
800.0
700.0
600.0
500.0
400.0
300.0
200.0
100.0
0.0
100.00
200.00
300.00
400.00
500.00
600.00
700.00
800.00
900.00
1000.00
1100.00
y11++
y12++
b13++-NH3
y13++-NH3
b11++-NH3
b2
y3
y4
y9++
b5
y7
y8
b9
y9
b10

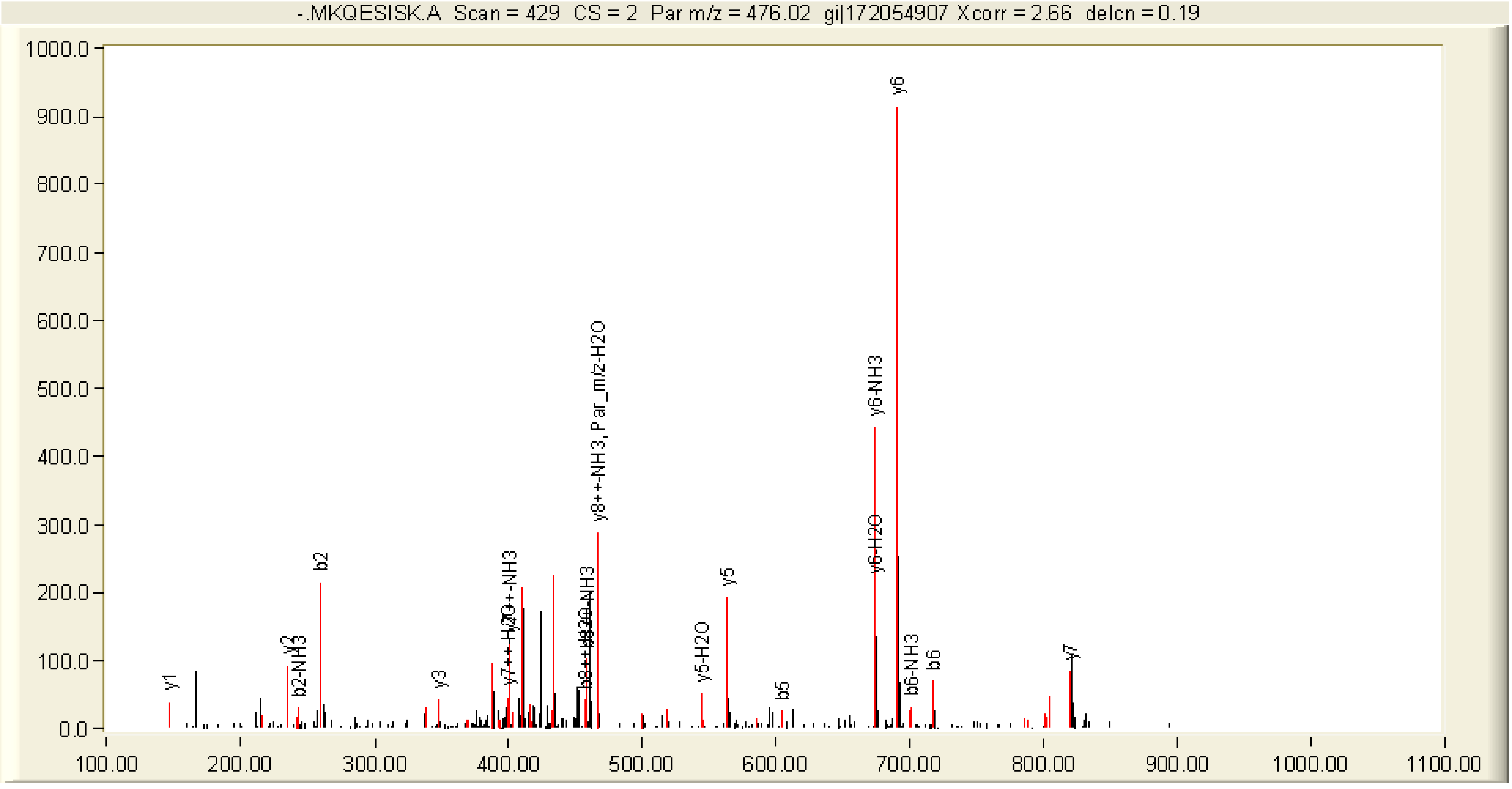

-.MKQESISK.A Scan = 429 CS = 2 Par m/z = 476.02 gi|172054907 Xcorr = 2.66 delcn = 0.19
y6
y6-NH3
y8++-NH3, Par_m/z-H2O
y6-H2O
y5
b2
y7++-NH3
y5-H2O
b6-NH3
b6
b2-NH3
y1
y3
b5
y7
1000.0
900.0
800.0
700.0
600.0
500.0
400.0
300.0
200.0
100.0
0.0
100.00
200.00
300.00
400.00
500.00
600.00
700.00
800.00
900.00
1000.00
1100.00

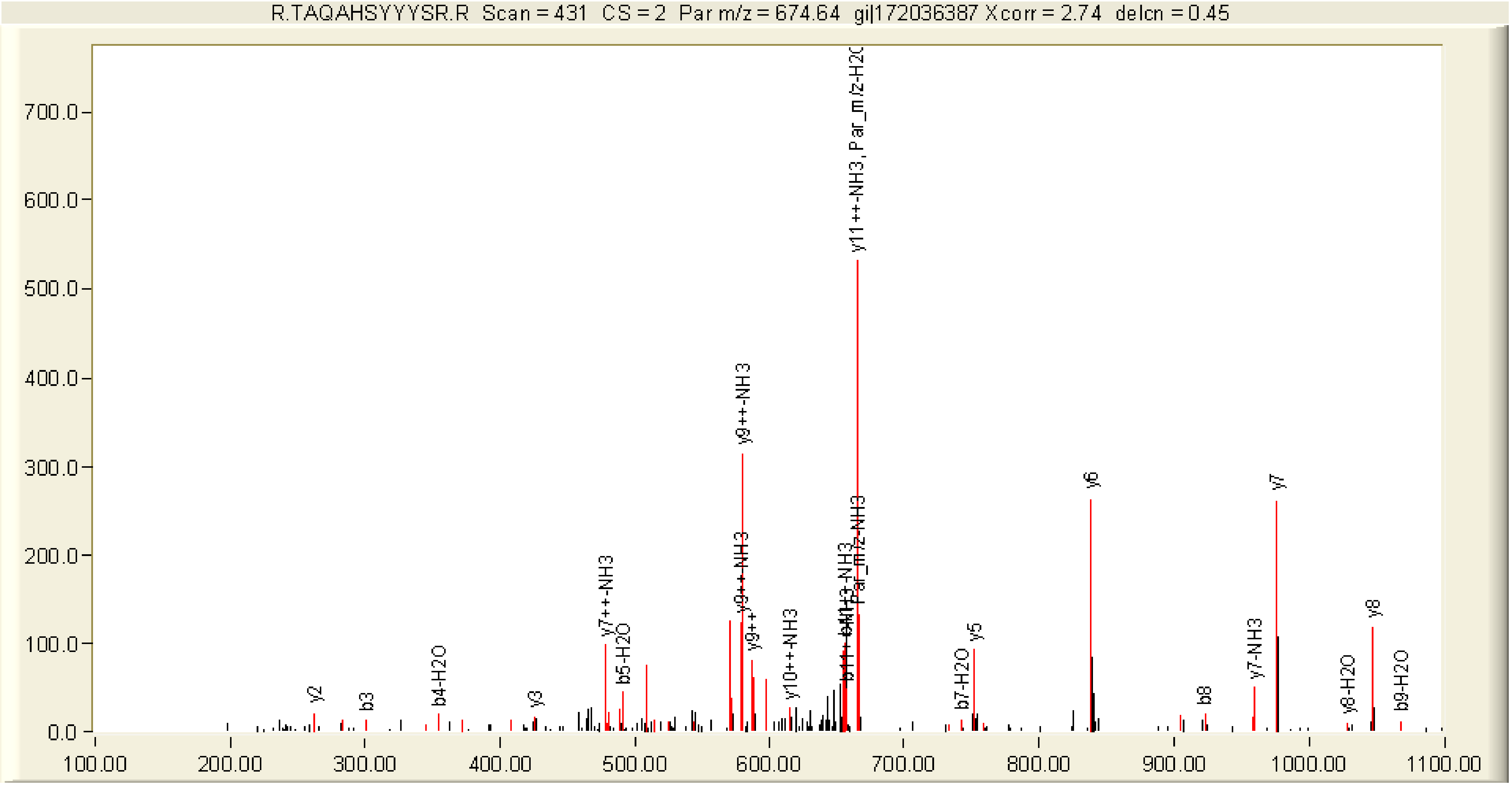

R.TAQAHSYYYSR.R Scan = 431 CS = 2 Par m/z = 674.64 gi|172036387 Xcorr = 2.74 delcn = 0.45
y11++-NH3, Par_m/z-H2O
y9++-NH3
y6
y7
y7++-NH3
b5-H2O
y9++-NH3
y9++
y10++-NH3
Par_m/z-NH3
y5
y8
y2
b3
b4-H2O
y3
b7-H2O
b8
y7-NH3
y8-H2O
b9-H2O
700.0
600.0
500.0
400.0
300.0
200.0
100.0
0.0
100.00
200.00
300.00
400.00
500.00
600.00
700.00
800.00
900.00
1000.00
1100.00

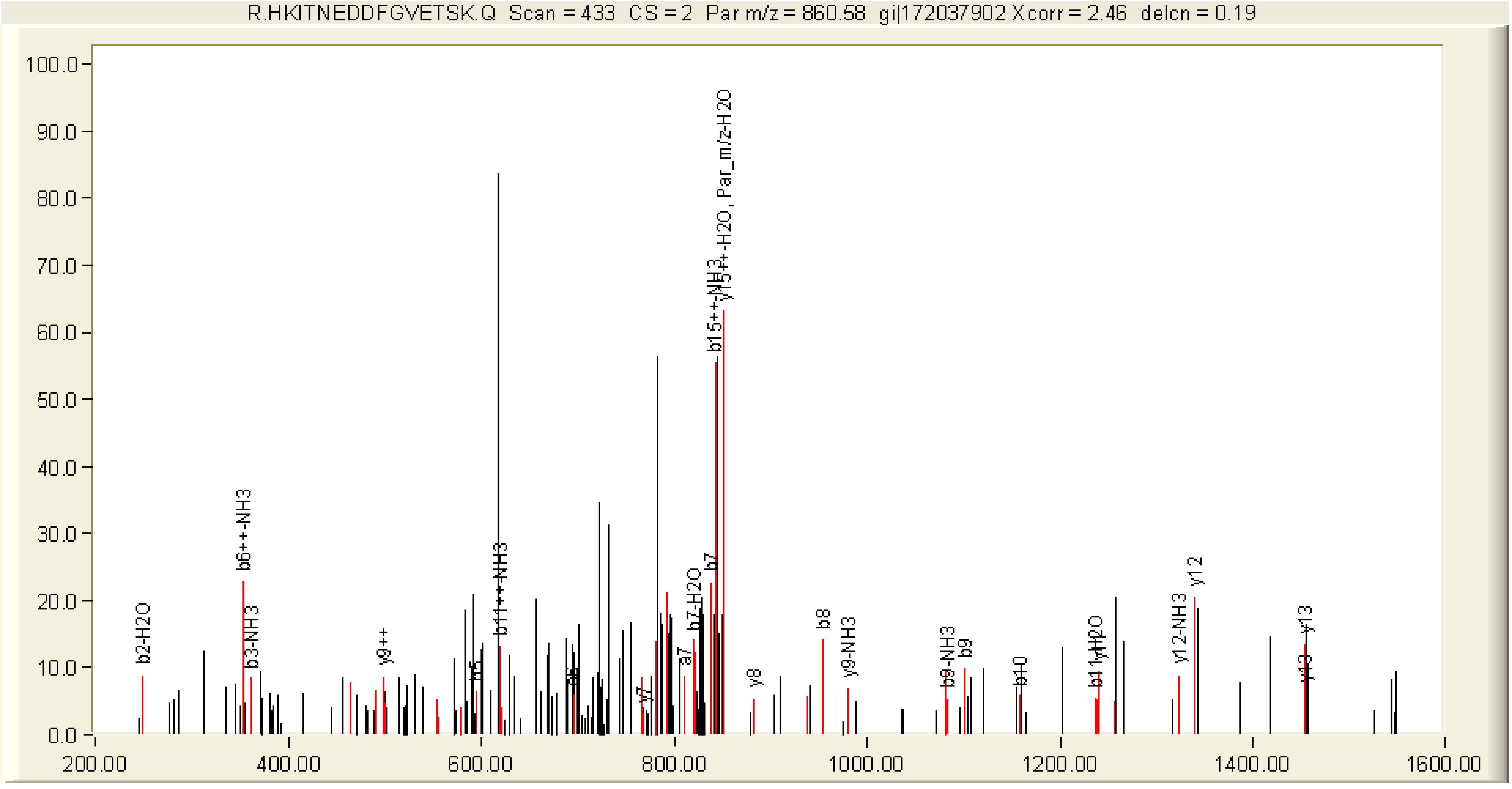

R.HKITNEDDFGVETSK.Q Scan = 433 CS = 2 Par m/z = 860.58 gi|172037902 Xcorr = 2.46 delcn = 0.19
100.0
90.0
80.0
70.0
60.0
50.0
40.0
30.0
20.0
10.0
0.0
200.00
400.00
600.00
800.00
1000.00
1200.00
1400.00
1600.00
b2-H2O
b6++-NH3
b3-NH3
y9++
b5
b11++-NH3
a7
b7-H2O
b7
y7
b15++-NH3
y15++-H2O, Par_m/z-H2O
y8
b8
y9-NH3
b9-NH3
b9
b10
b11-H2O
y12-NH3
y12
y13

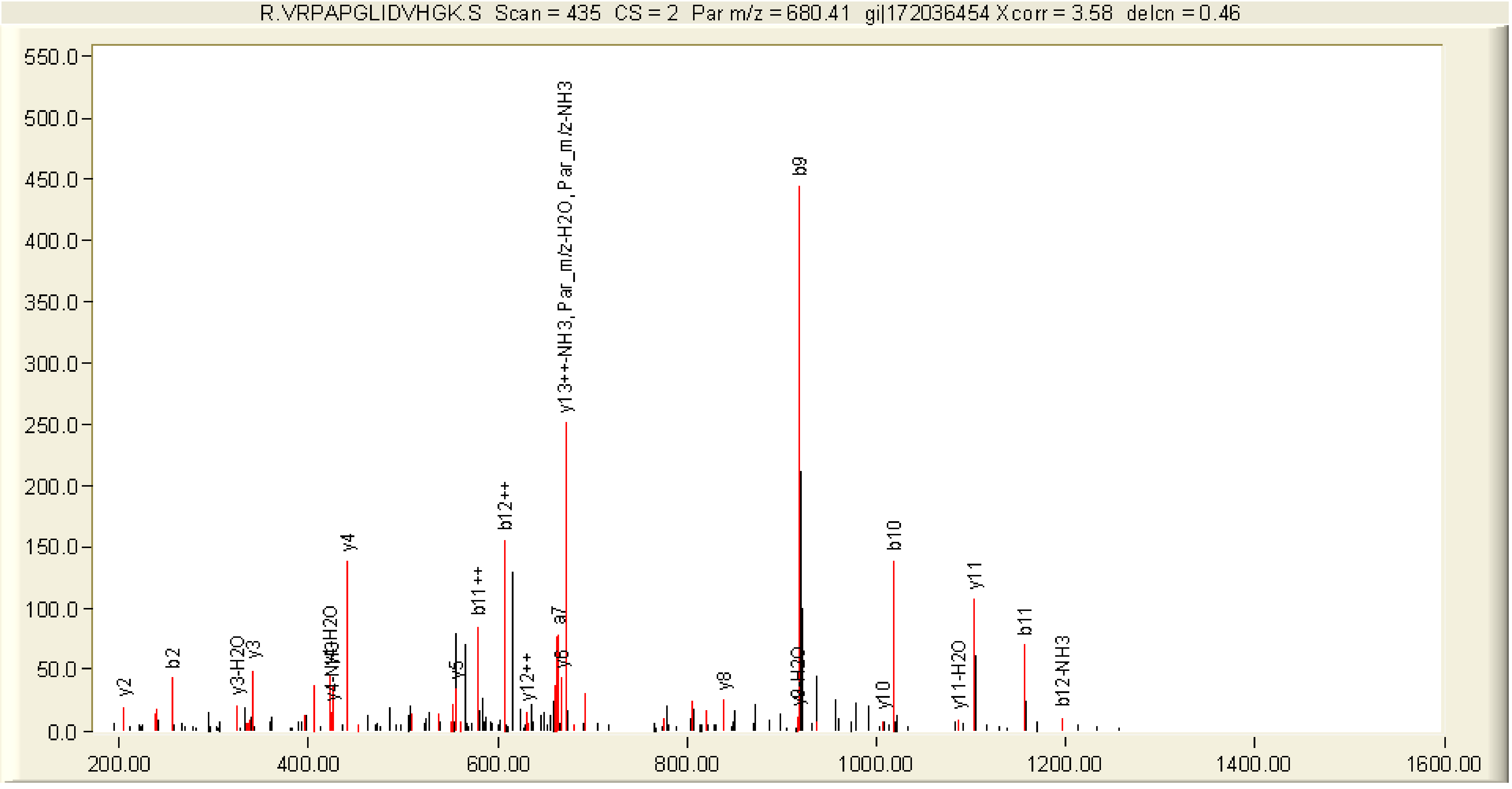

R.VRPAPGLIDVHGK.S Scan = 435 CS = 2 Par m/z = 680.41 gi|172036454 Xcorr = 3.58 delcn = 0.46
550.0
500.0
450.0
400.0
350.0
300.0
250.0
200.0
150.0
100.0
50.0
0.0
200.00
400.00
600.00
800.00
1000.00
1200.00
1400.00
1600.00
y2
b2
y3-H2O
y3
y4
b11++
b12++
y12++
y5
a7
y6
y13++-NH3, Par_m/z-H2O, Par_m/z-NH3
y8
b9
b10
y10
y11-H2O
y11
b11
b12-NH3

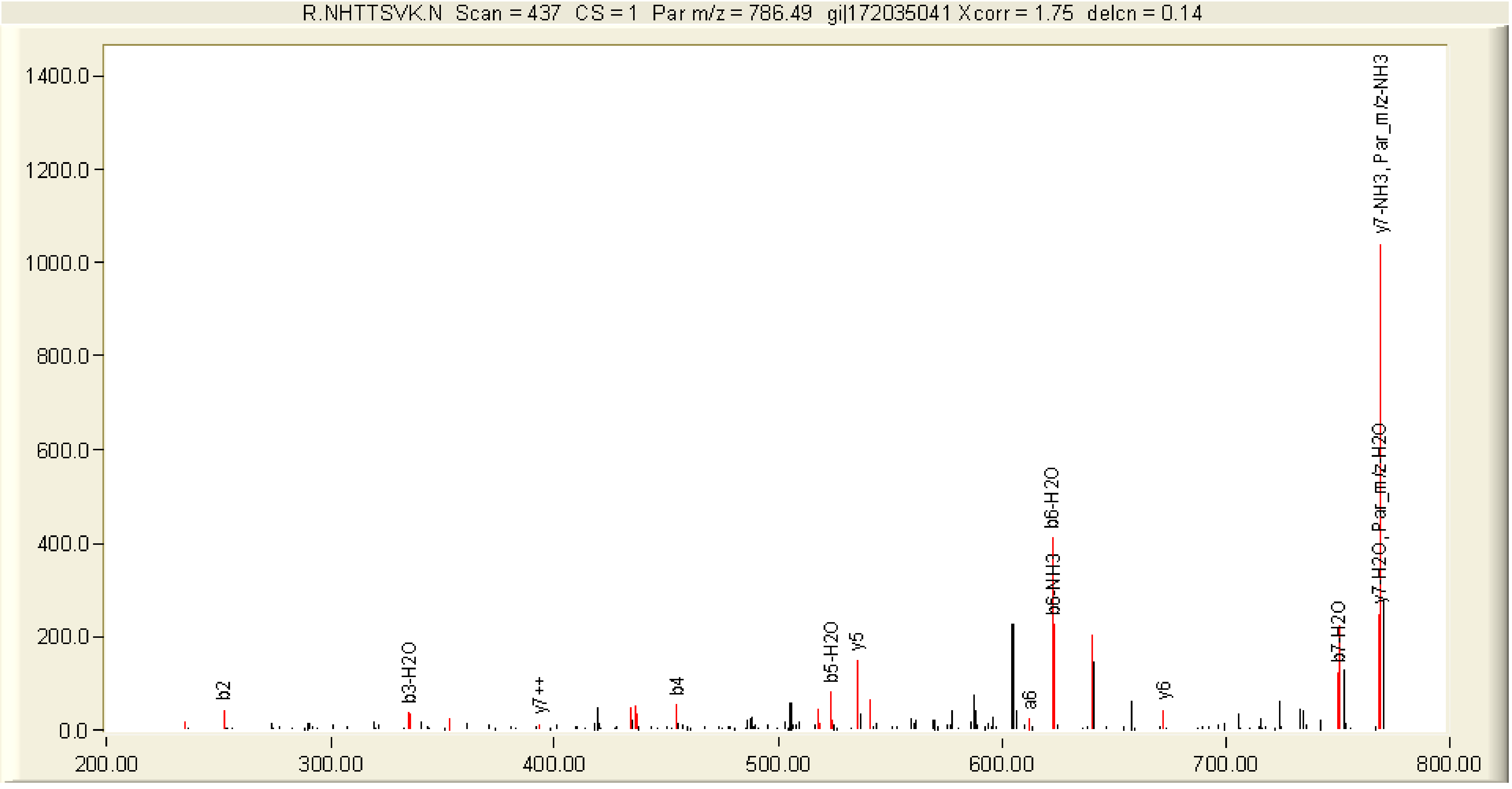

R.NHTTSVK.N Scan = 437 CS = 1 Par m/z = 786.49 gi|172035041 Xcorr = 1.75 delcn = 0.14
1400.0
1200.0
1000.0
800.0
600.0
400.0
200.0
0.0
200.00
300.00
400.00
500.00
600.00
700.00
800.00
b2
b3-H2O
y7++
b4
b5-H2O
y5
a6
b6-NH3
b6-H2O
y6
b7-H2O
y7-H2O, Par_m/z-H2O
y7-NH3, Par_m/z-NH3

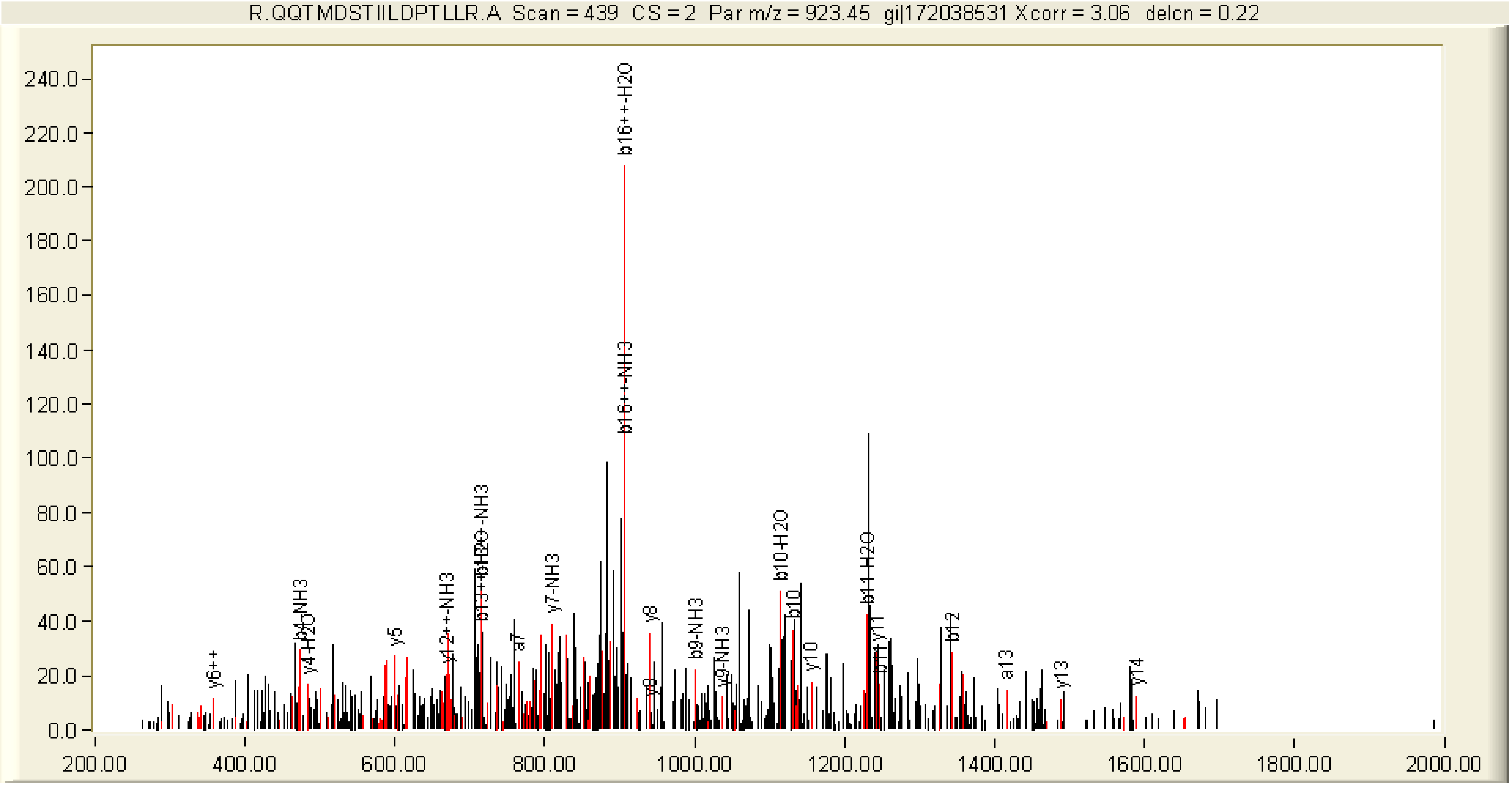
R.QQTMDSTIILDPTLLR.A Scan = 439 CS = 2 Par m/z = 923.45 gi|172038531 Xcorr = 3.06 delcn = 0.22
b16++-H2O
b16++-NH3
y6++
b4-NH3
y4-H2O
y5
y12++-NH3
b13++-H2O-NH3
a7
y7-NH3
y8
b9-NH3
y9-NH3
b10-H2O
b10
y10
b11-H2O
y11
b12
a13
y13
y14
0.0
20.0
40.0
60.0
80.0
100.0
120.0
140.0
160.0
180.0
200.0
220.0
240.0
200.00
400.00
600.00
800.00
1000.00
1200.00
1400.00
1600.00
1800.00
2000.00

K.WSILYDLQLIFK.T Scan = 441 CS = 2 Par m/z = 769.76 gi|172038005 Xcorr = 2.46 delcn = 0.12

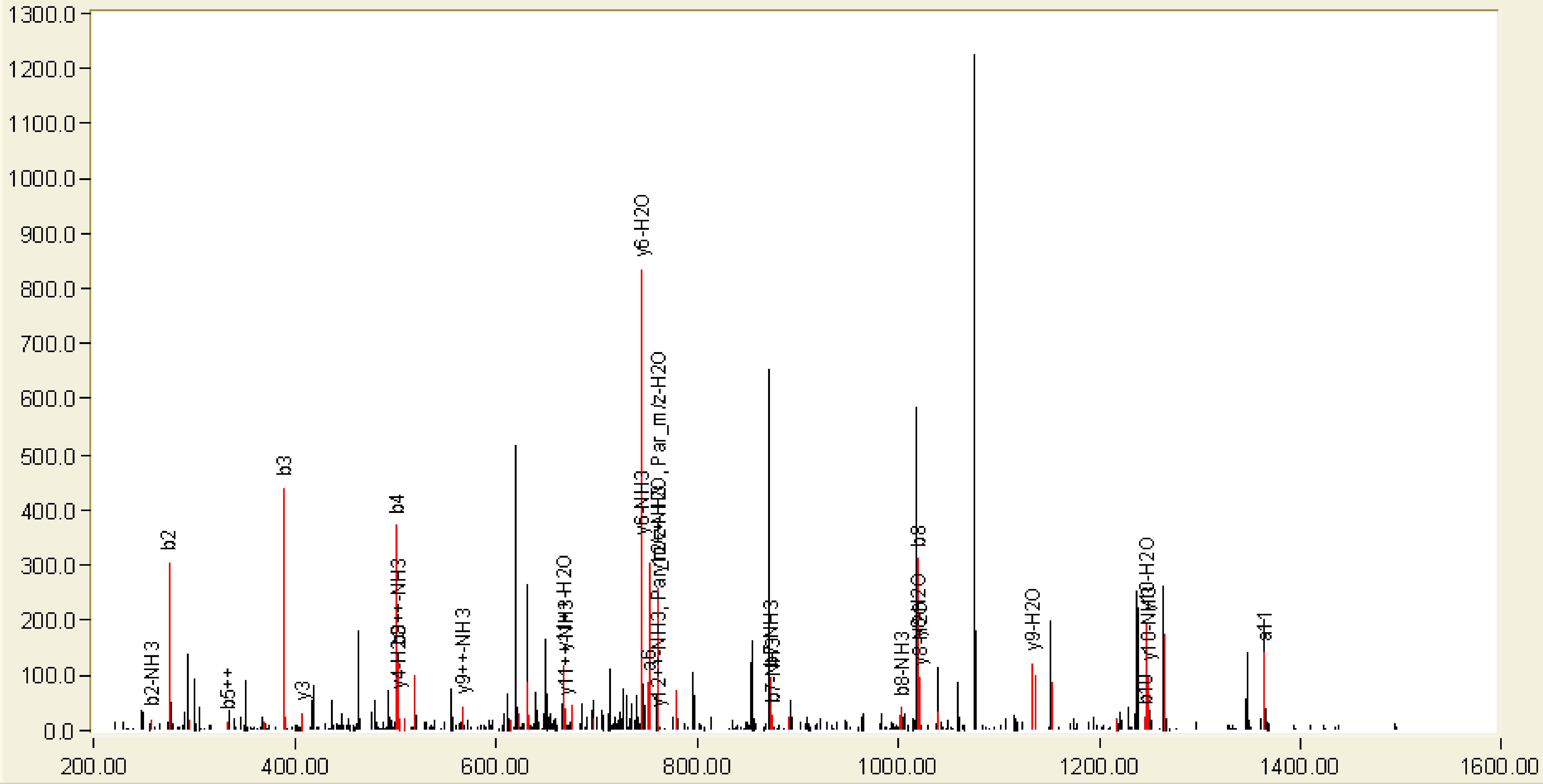

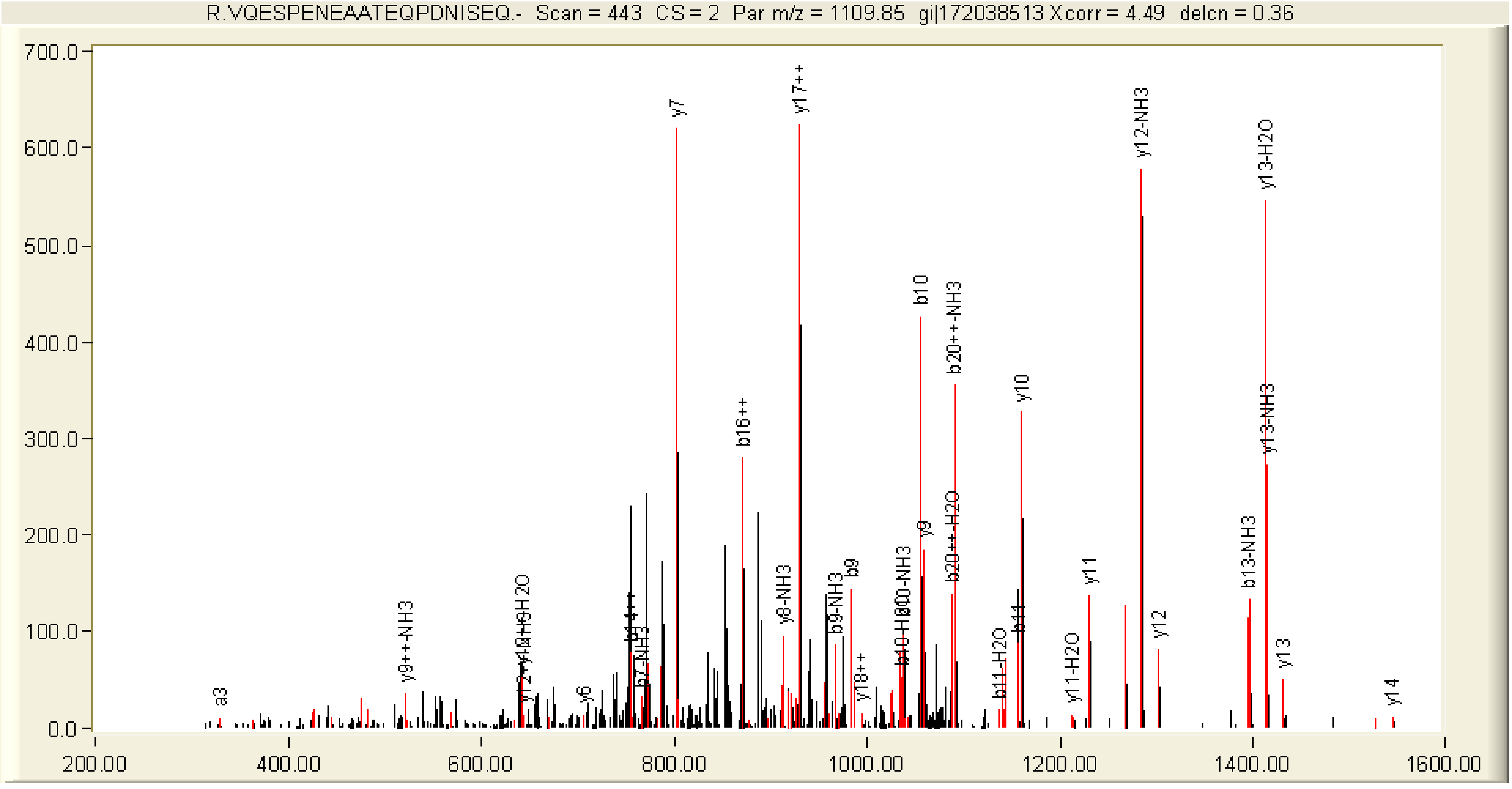

R.VQESPENEAATEQPDNISEQ.- Scan = 443 CS = 2 Par m/z = 1109.85 gi|172038513 Xcorr = 4.49 delcn = 0.36
700.0
600.0
500.0
400.0
300.0
200.0
100.0
0.0
200.00
400.00
600.00
800.00
1000.00
1200.00
1400.00
1600.00
a3
y9++-NH3
y6
y7
b16++
y17++
y8-NH3
b9-NH3
b9
y18++
b10
y9
b20++-NH3
b20++-H2O
y10
b11-H2O
y11-H2O
y11
y12-NH3
y12
b13-NH3
y13-H2O
y13-NH3
y13
y14

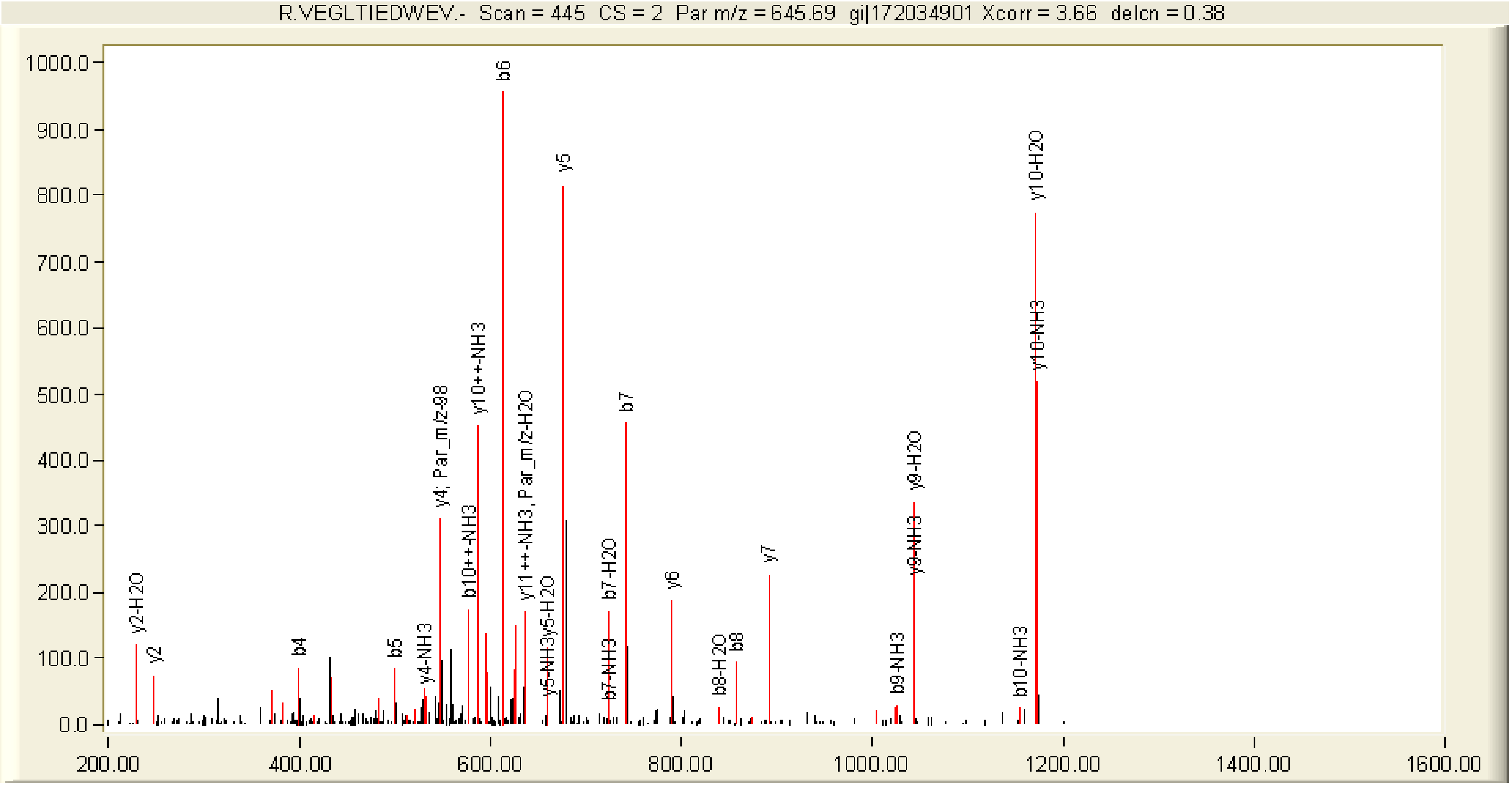
R.VEGLTIEDWEV.- Scan = 445 CS = 2 Par m/z = 645.69 gi|172034901 Xcorr = 3.66 delcn = 0.38
1000.0
900.0
800.0
700.0
600.0
500.0
400.0
300.0
200.0
100.0
0.0
y2-H2O
y2
b4
b5
y4-NH3
y4; Par_m/z-98
b10++-NH3
y10++-NH3
b6
y11 ++-NH3, Par_m/z-H2O
y5-NH3y5-H2O
y5
b7-NH3
b7-H2O
b7
y6
b8-H2O
b8
y7
b9-NH3
y9-NH3
y9-H2O
b10-NH3
y10-NH3
y10-H2O
200.00
400.00
600.00
800.00
1000.00
1200.00
1400.00
1600.00

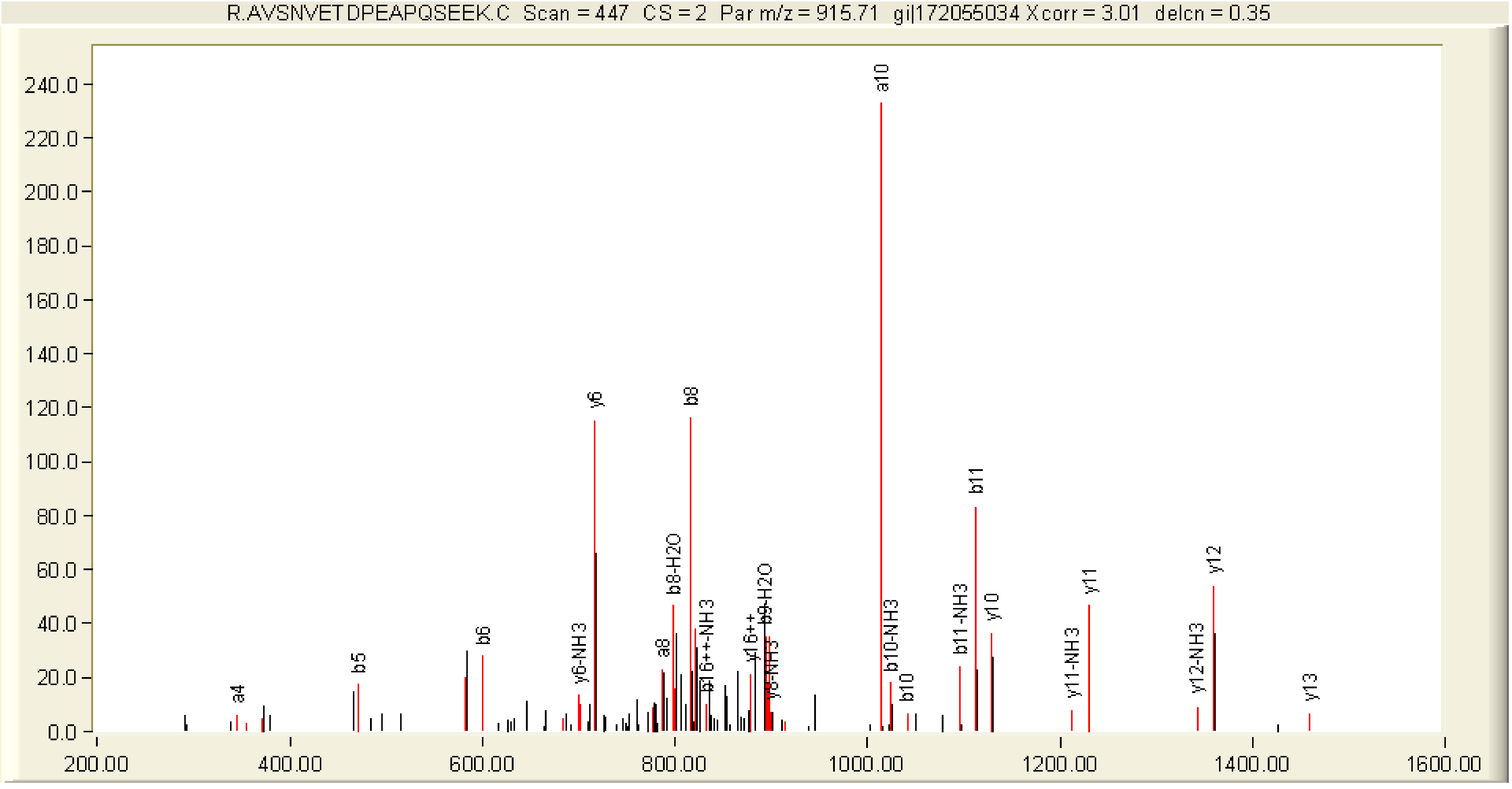

R.AVSNVETDPEAPQSEEK.C Scan = 447 CS = 2 Par m/z = 915.71 gi|172055034 Xcorr = 3.01 delcn = 0.35
240.0
220.0
200.0
180.0
160.0
140.0
120.0
100.0
80.0
60.0
40.0
20.0
0.0
200.00
400.00
600.00
800.00
1000.00
1200.00
1400.00
1600.00
a4
b5
b6
y6-NH3
y6
a8
b8-H2O
b8
b16++-NH3
y16++
b9-H2O
y8-NH3
a10
b10-NH3
b10
b11-NH3
b11
y10
y11-NH3
y11
y12-NH3
y12
y13

R.DSGYTVIGTTDAK.D Scan = 449 CS = 2 Par m/z = 664.85 gi|172039456 Xcorr = 3.71 delcn = 0.56

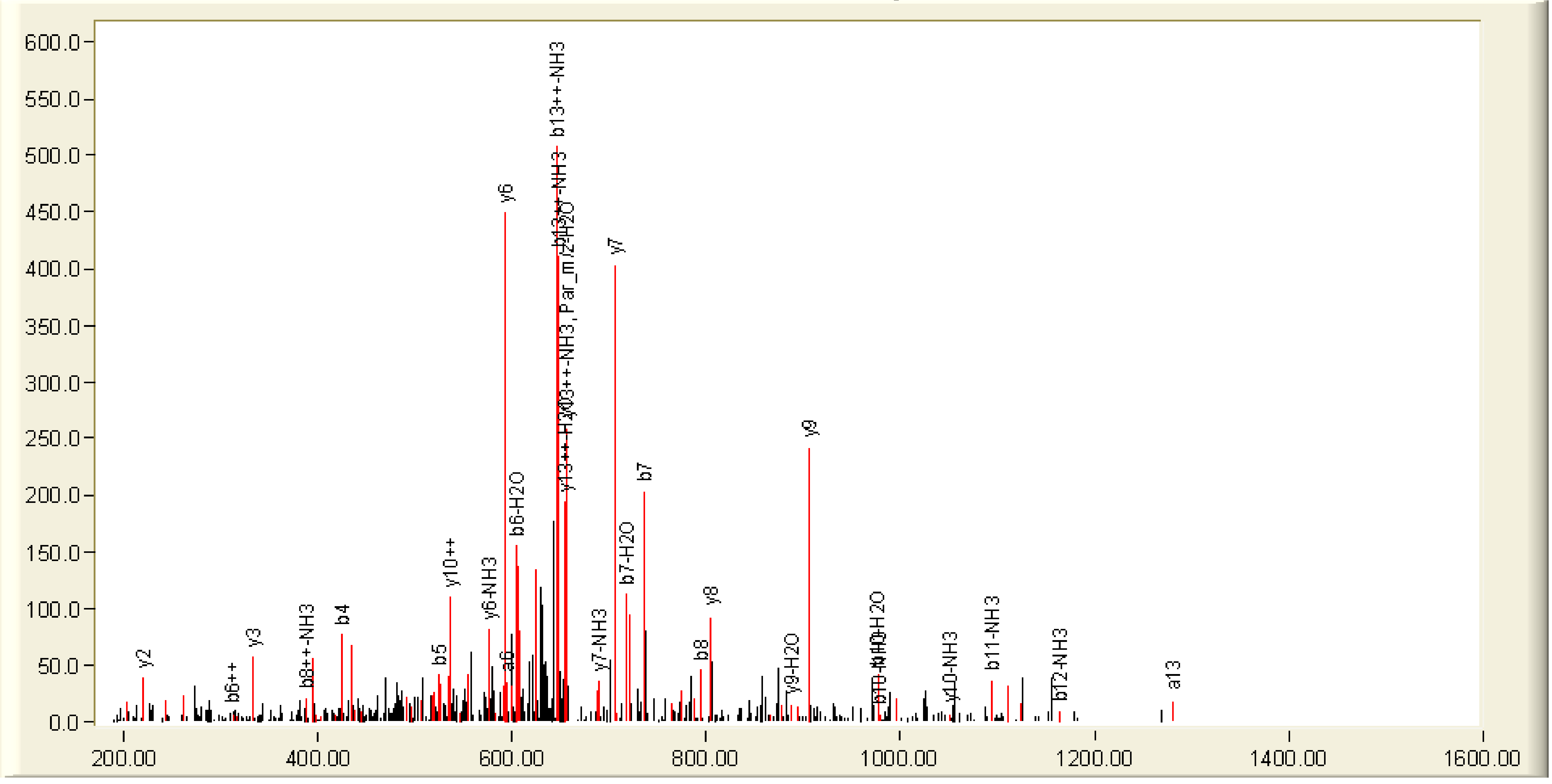

R.LASGENTTAGDLDAMTEQAK.V Scan = 451 CS = 2 Par m/z = 1012.64 gi|172039148 Xcorr = 5.21 delcn = 0.49

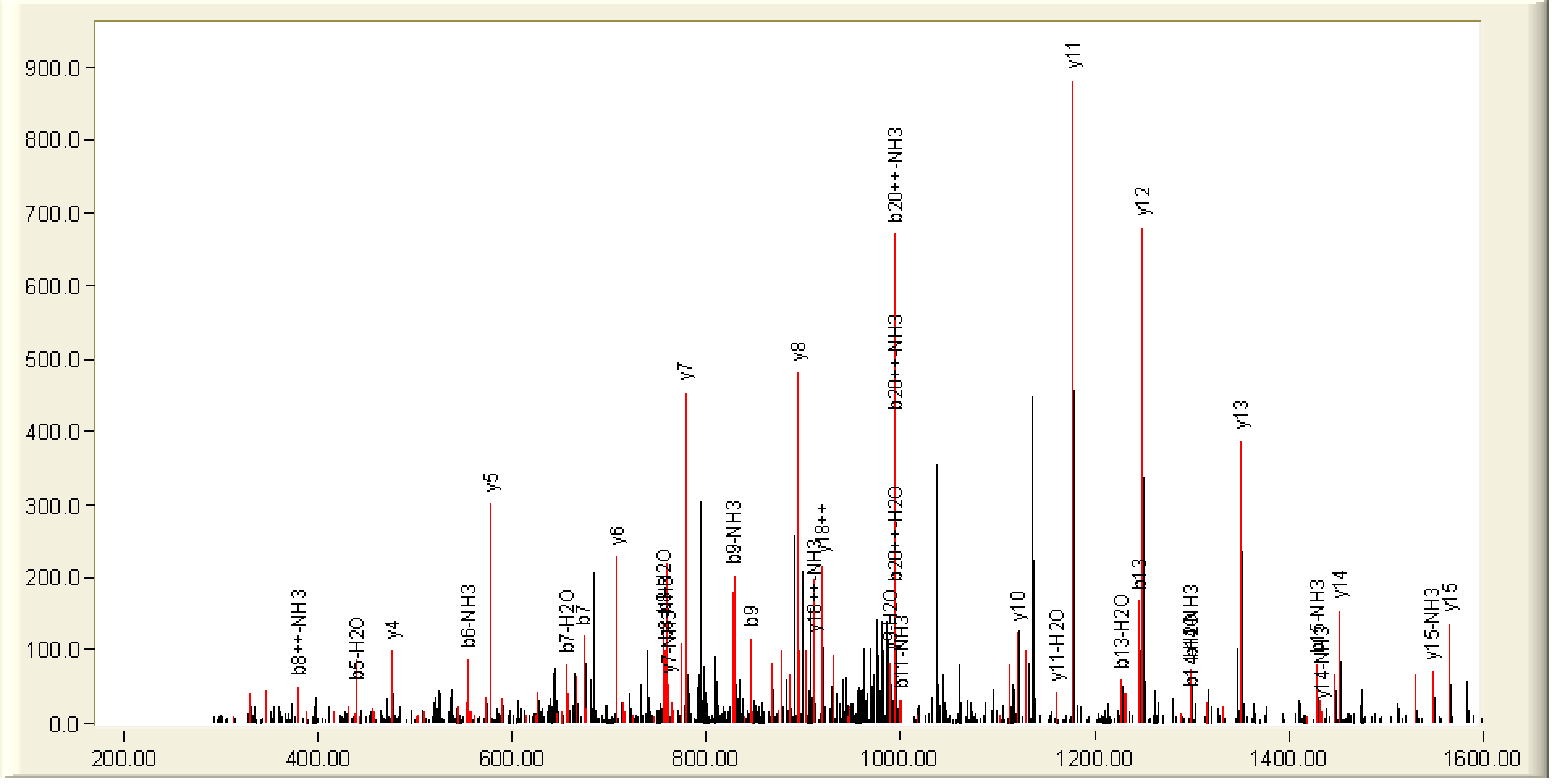

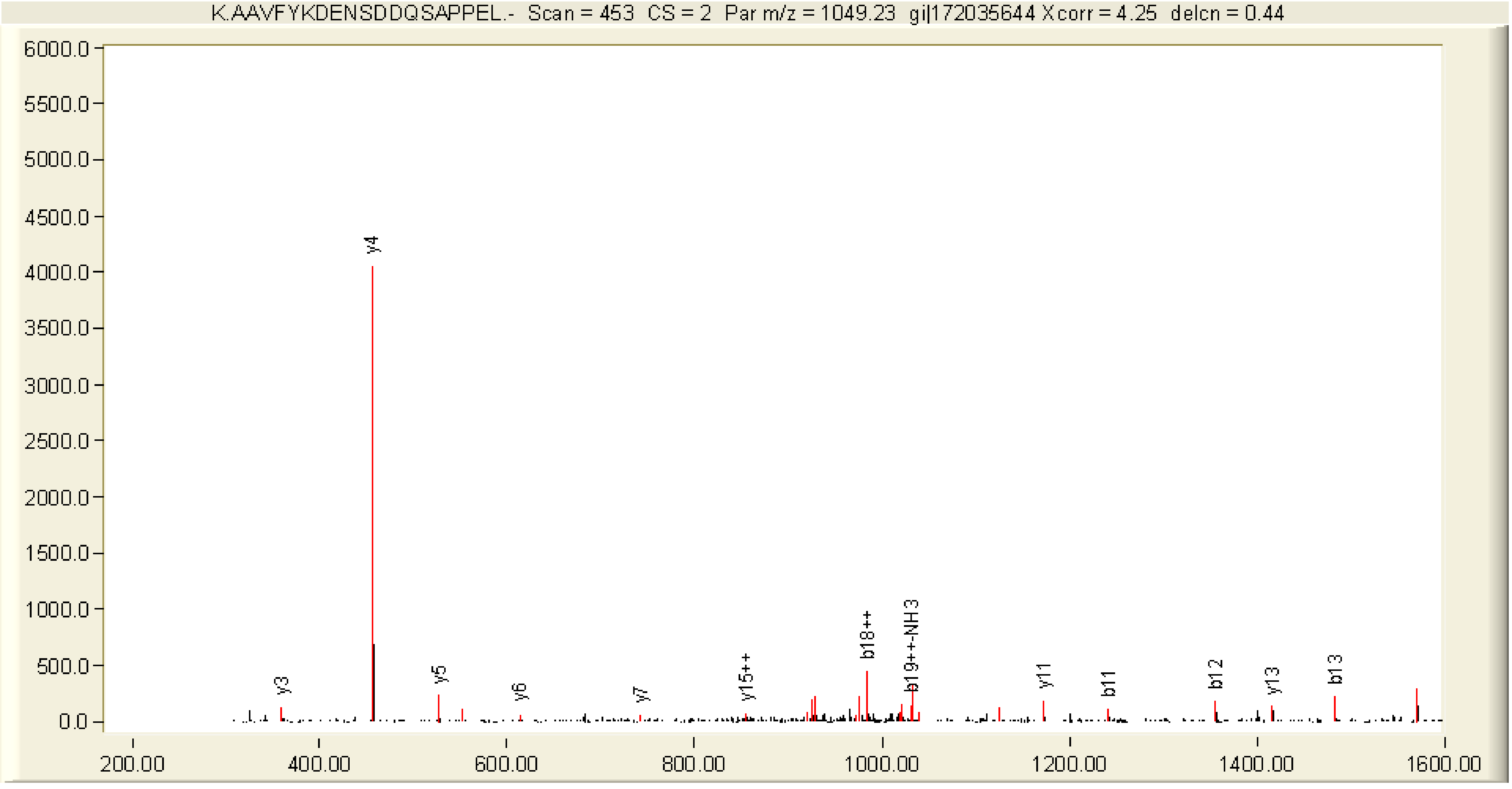
K.AAVFYKDENSDDQSAPPEL.- Scan = 453 CS = 2 Par m/z = 1049.23 gi|172035644 Xcorr = 4.25 delcn = 0.44
6000.0
5500.0
5000.0
4500.0
4000.0
3500.0
3000.0
2500.0
2000.0
1500.0
1000.0
500.0
0.0
200.00
400.00
600.00
800.00
1000.00
1200.00
1400.00
1600.00
y3
y4
y5
y6
y7
y15++
b18++
b19++-NH3
y11
b11
b12
y13
b13

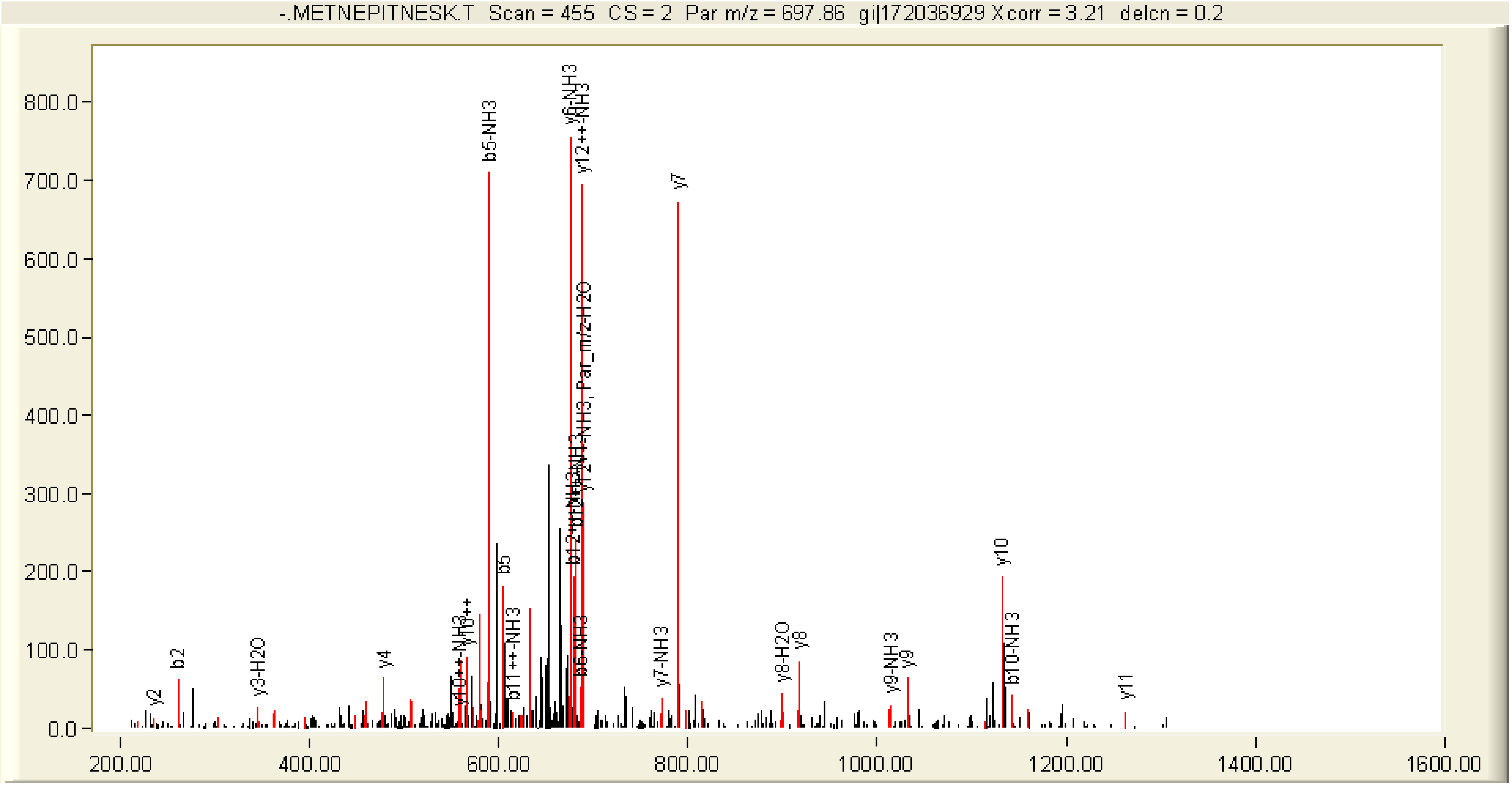

-.METNEPITNESK.T Scan = 455 CS = 2 Par m/z = 697.86 gi|172036929 Xcorr = 3.21 delcn = 0.2
800.0
700.0
600.0
500.0
400.0
300.0
200.0
100.0
0.0
200.00
400.00
600.00
800.00
1000.00
1200.00
1400.00
1600.00
b5-NH3
y6-NH3
y12++-NH3
y7
b5
y2
b2
y3-H2O
y4
y10++
b11++-NH3
b6-NH3
y7-NH3
y8-H2O
y8
y9-NH3
y9
y10
b10-NH3
y11

K.AQAEINMEQNASQSQQQTK.E Scan = 457 CS = 3 Par m/z = 712.6 gi|172055100 Xcorr = 3.24 delcn = 0.15

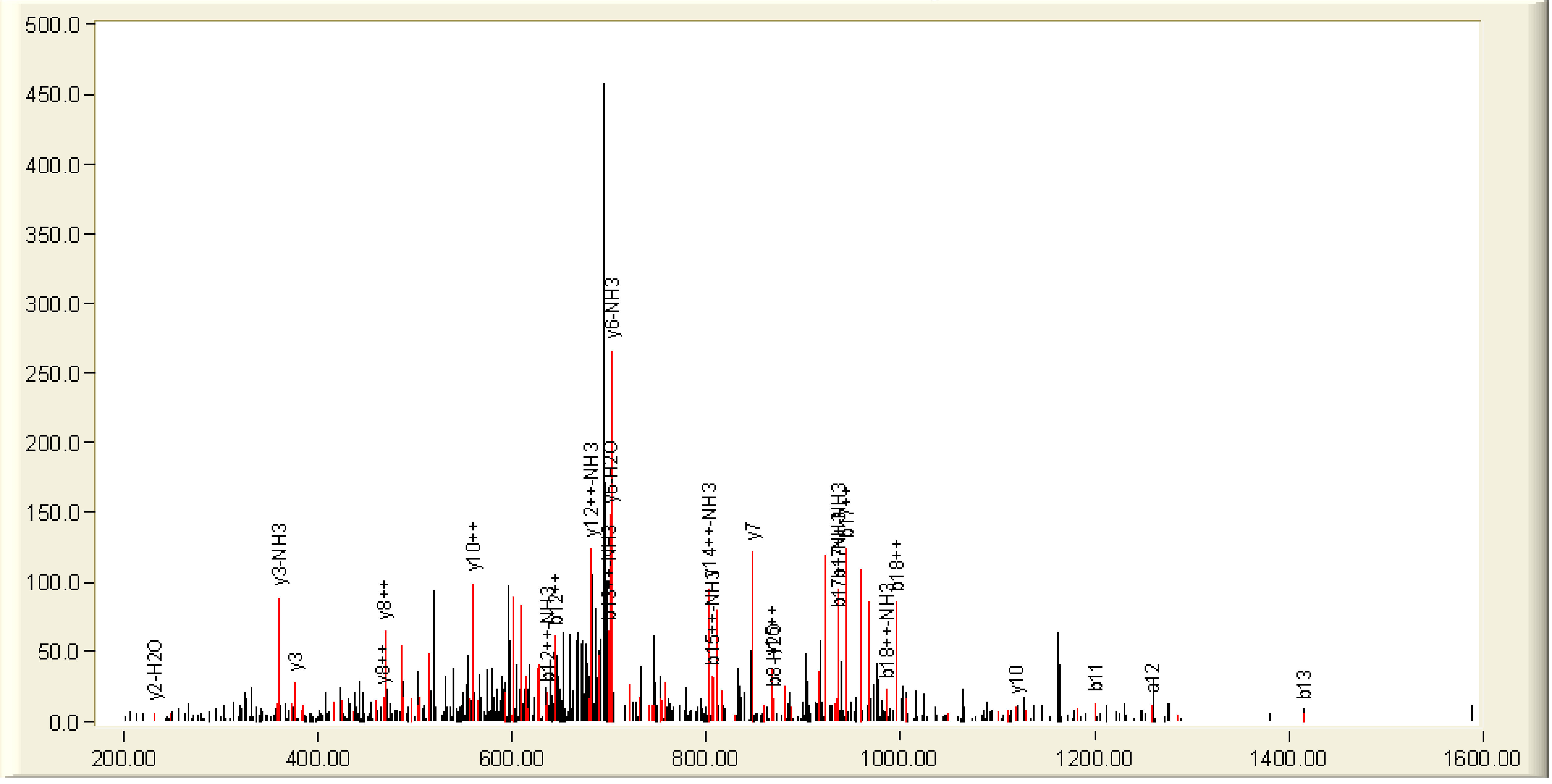

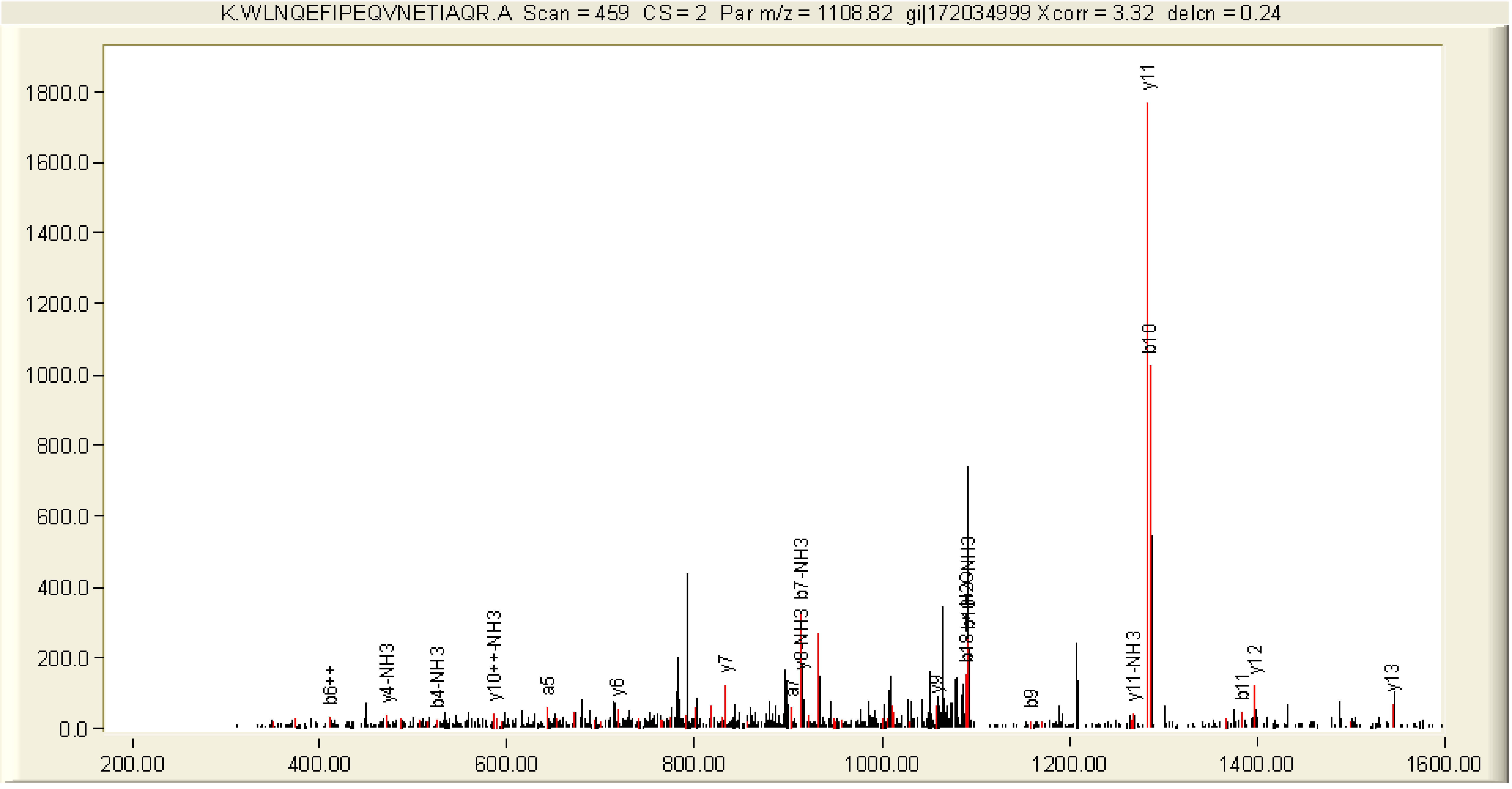
K.WLNQEFIPEQVNETIAQR.A Scan = 459 CS = 2 Par m/z = 1108.82 gi|172034999 Xcorr = 3.32 delcn = 0.24
0.0
200.0
400.0
600.0
800.0
1000.0
1200.0
1400.0
1600.0
1800.0
200.00
400.00
600.00
800.00
1000.00
1200.00
1400.00
1600.00
b6++
y4-NH3
b4-NH3
y10++-NH3
a5
y6
y7
a7
y8-NH3
b7-NH3
y9
b9
y11-NH3
y11
b10
b11
y12
y13

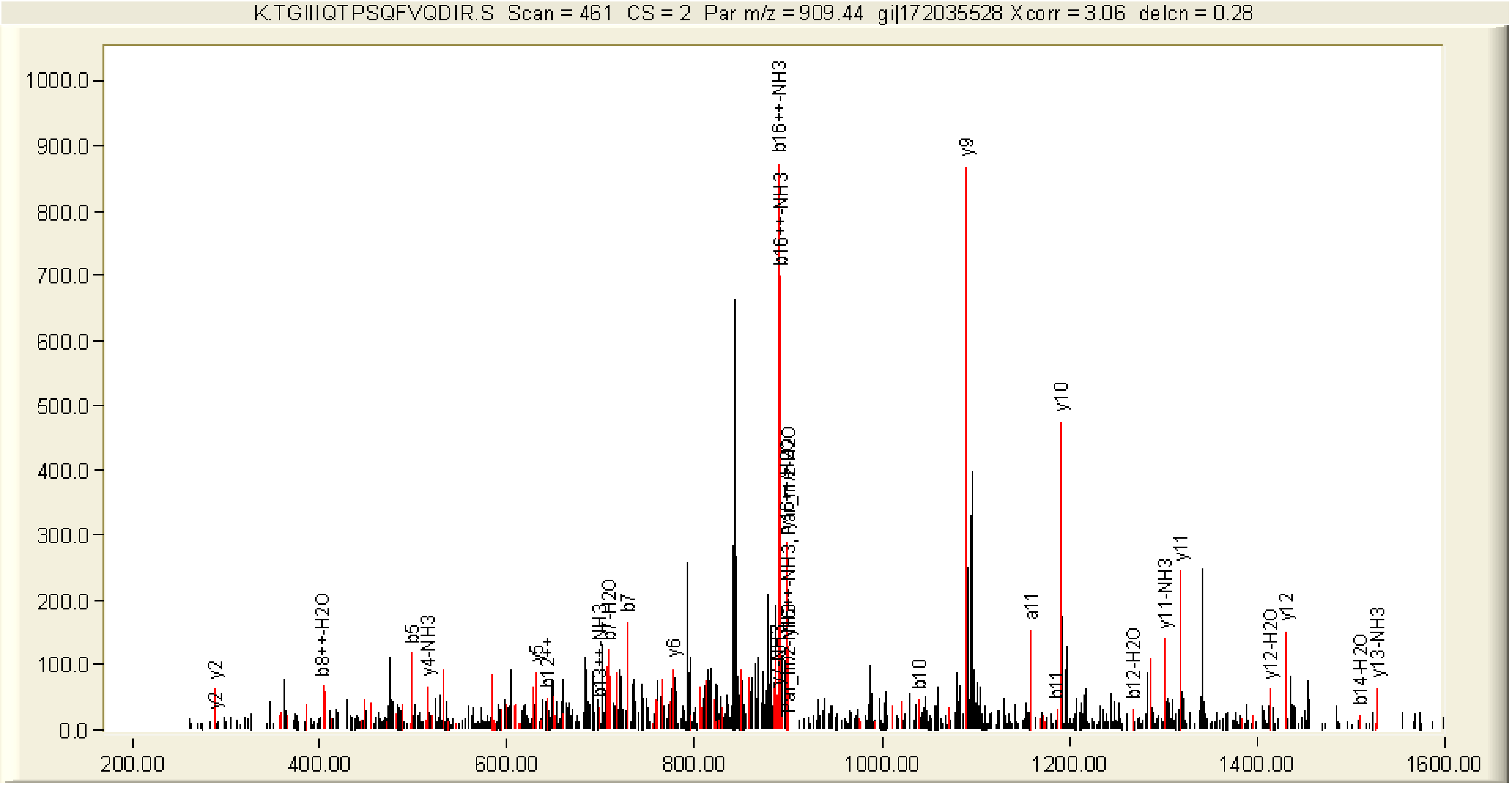
K.TGIIIQTPSQFVQDIR.S Scan = 461 CS = 2 Par m/z = 909.44 gi|172035528 Xcorr = 3.06 delcn = 0.28
1000.0
900.0
800.0
700.0
600.0
500.0
400.0
300.0
200.0
100.0
0.0
200.00
400.00
600.00
800.00
1000.00
1200.00
1400.00
1600.00
b16++-NH3
y9
y10
a11
y11-NH3
y11
y12-H2O
y12
b14-H2O
y13-NH3
b12-H2O
b11
b10
b8++-H2O
b5
y4-NH3
y2
b7
y6
b7-H2O

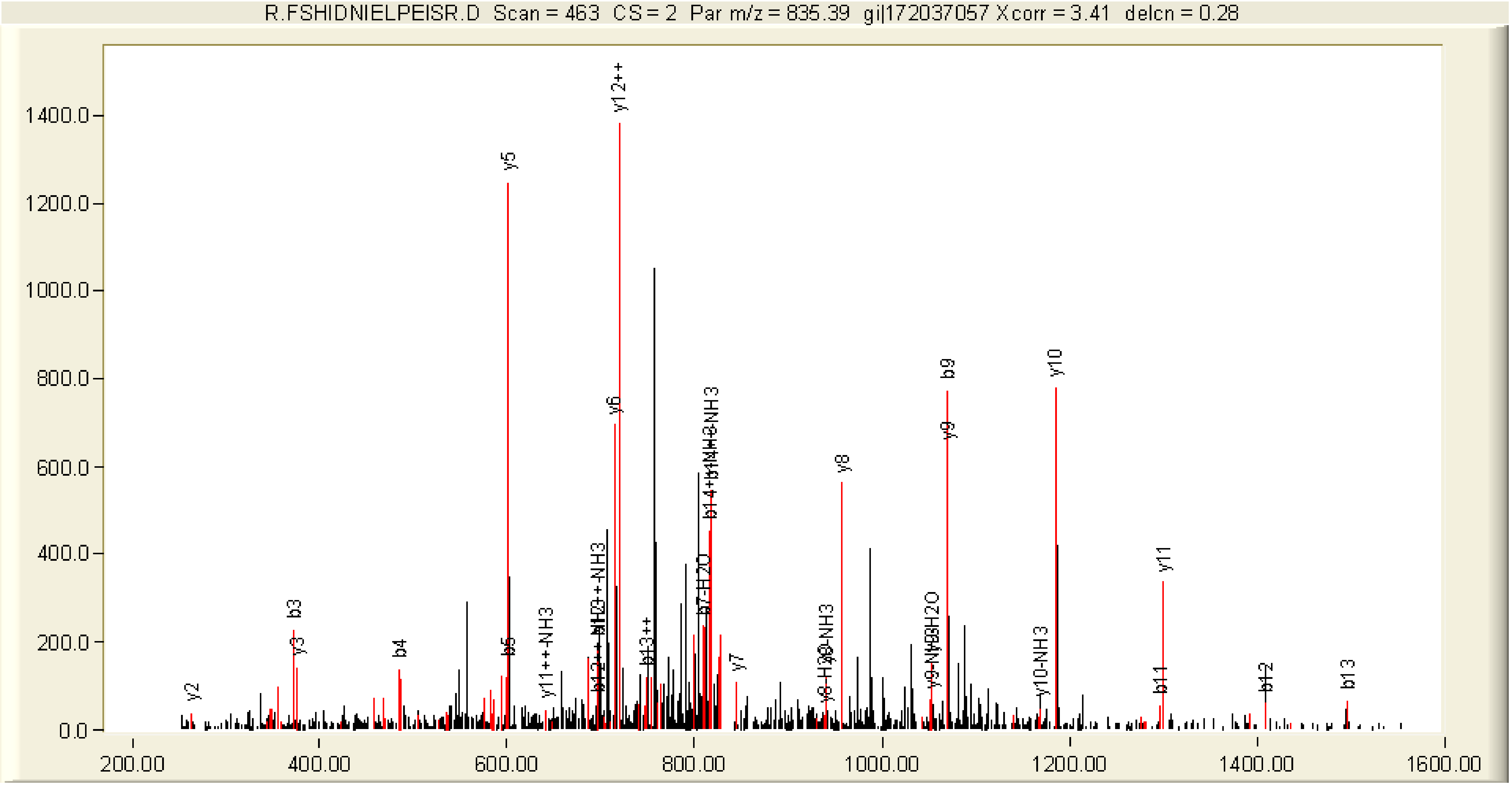
R.FSHIDNIELPEISR.D Scan = 463 CS = 2 Par m/z = 835.39 gi|172037057 Xcorr = 3.41 delcn = 0.28
0.0
200.0
400.0
600.0
800.0
1000.0
1200.0
1400.0
200.00
400.00
600.00
800.00
1000.00
1200.00
1400.00
1600.00
y2
b3
y3
b4
b5
y5
y11++-NH3
y6
y12++
b13++
b7-H2O
y7
y8
b9
y9
y10
y10-NH3
y11
b11
b12
b13

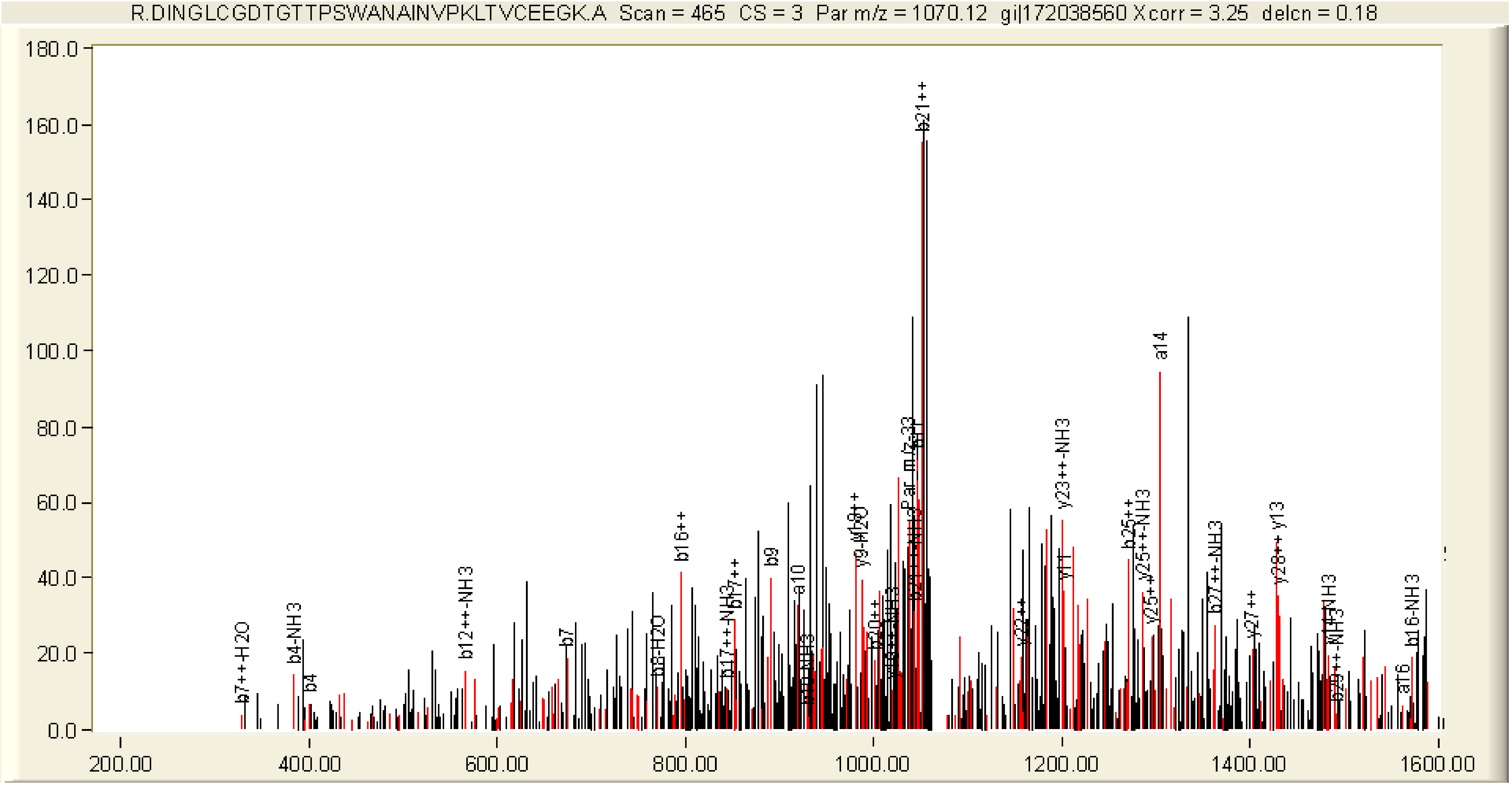
R.DINGLCGDTGTTPSWANAINVPKLTVCEEGK.A Scan = 465 CS = 3 Par m/z = 1070.12 gi|172038560 Xcorr = 3.25 delcn = 0.18
180.0
160.0
140.0
120.0
100.0
80.0
60.0
40.0
20.0
0.0
200.00
400.00
600.00
800.00
1000.00
1200.00
1400.00
1600.00
b21++
a14
y23++-NH3
b25++
y28++ y13
b16++
b9
a10
b12++-NH3
b4-NH3
b4
b7++-H2O
b7
b8-H2O
y11
b27++-NH3
y27++
b16-NH3
a16

R.SRVDADGVVR.L Scan = 467 CS = 2 Par m/z = 537.4 gi|172034900 Xcorr = 3.32 delcn = 0.31

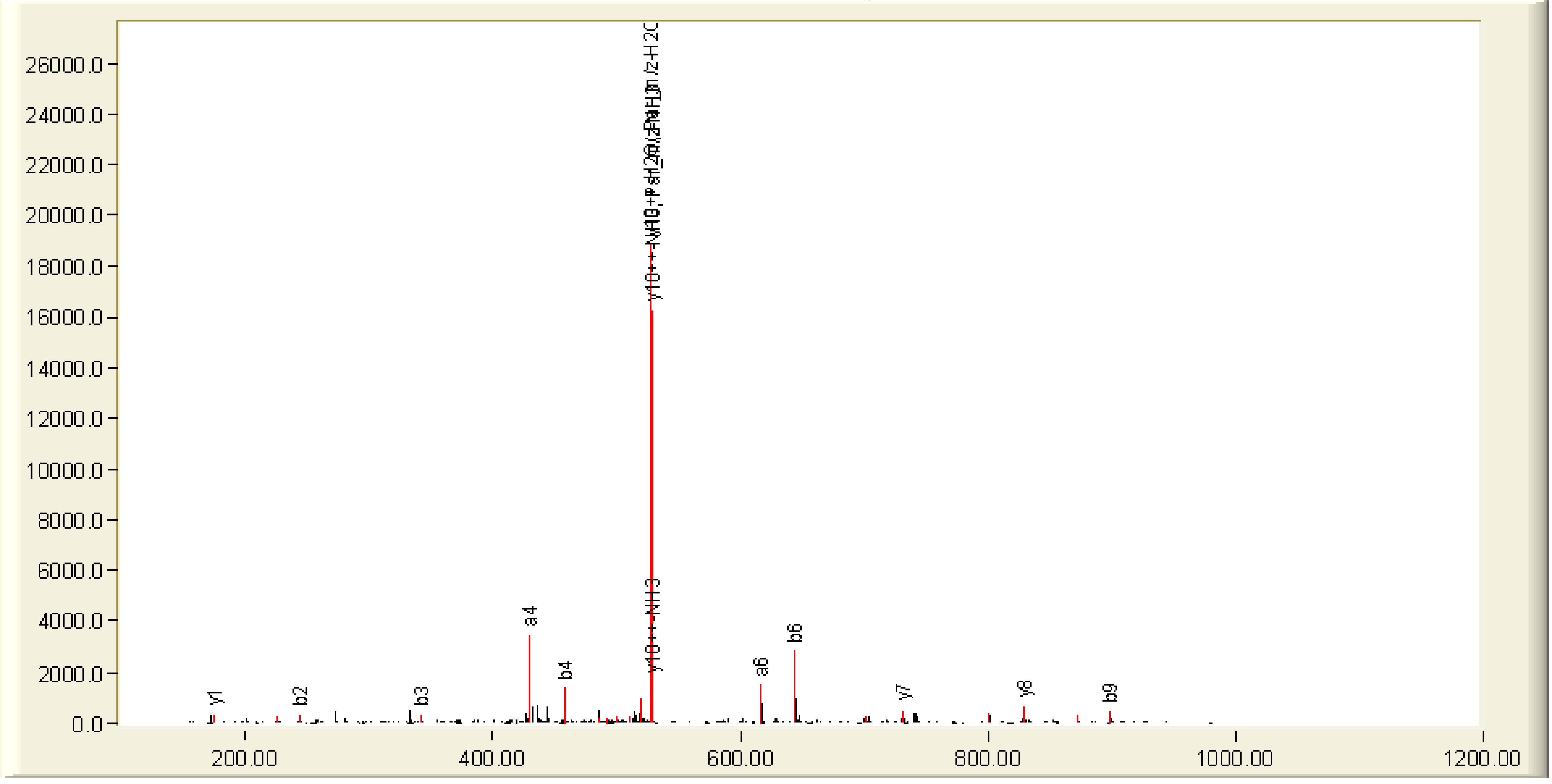

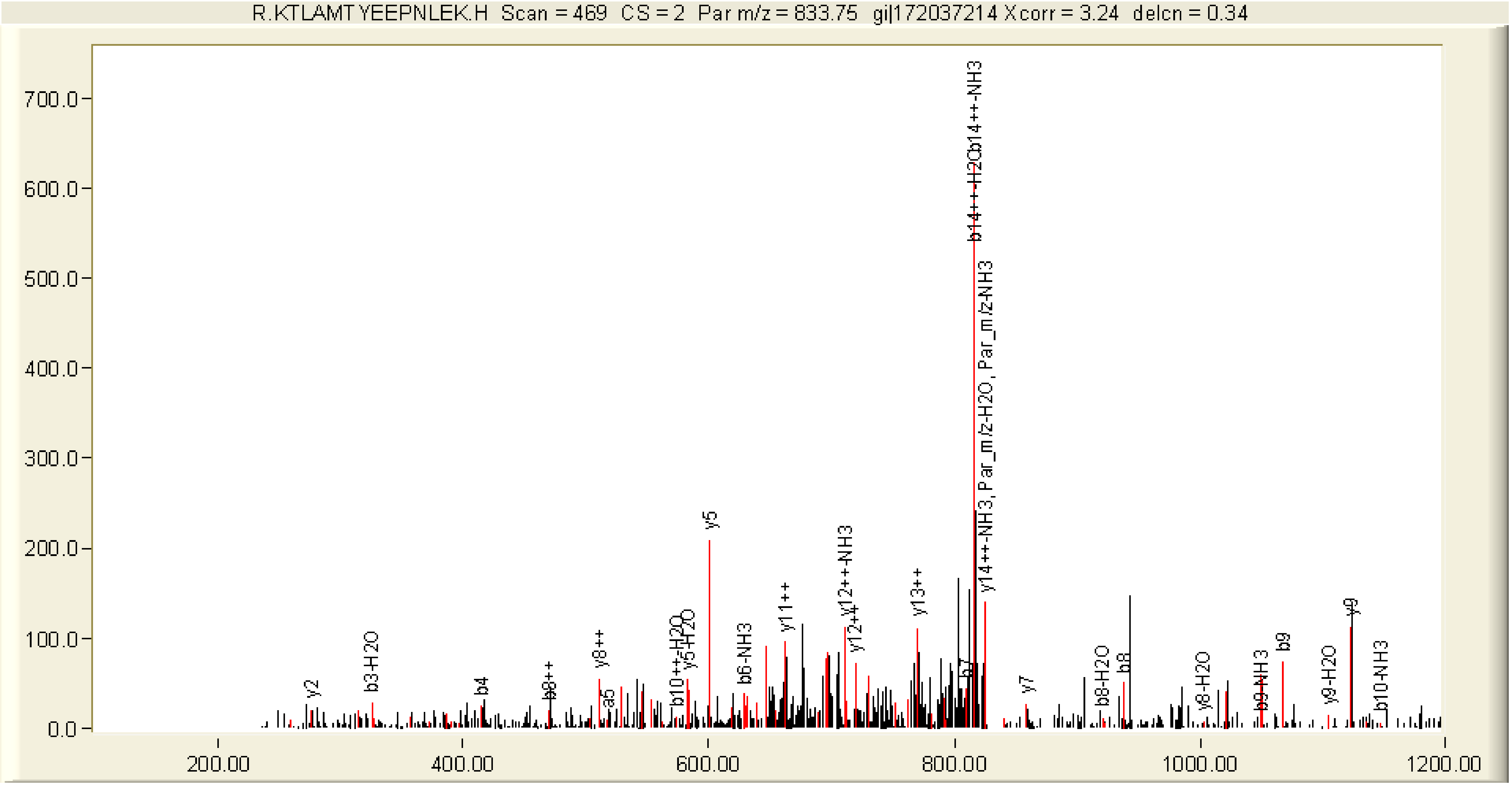
R.KTLAMTYEEPNLEK.H Scan = 469 CS = 2 Par m/z = 833.75 gi|172037214 Xcorr = 3.24 delcn = 0.34
700.0
600.0
500.0
400.0
300.0
200.0
100.0
0.0
200.00
400.00
600.00
800.00
1000.00
1200.00
b14++-H2O
b14++-NH3
y14++-NH3, Par_m/z-NH3, Par_m/z-H2O, Par_m/z-NH3
y5
y12++-NH3
y13++
y11++
b6-NH3
y5-H2O
y8++
b8++
b4
b3-H2O
y2
a5
y7
b8-H2O
b8
y8-H2O
b9-NH3
b9
y9-H2O
y9
b10-NH3

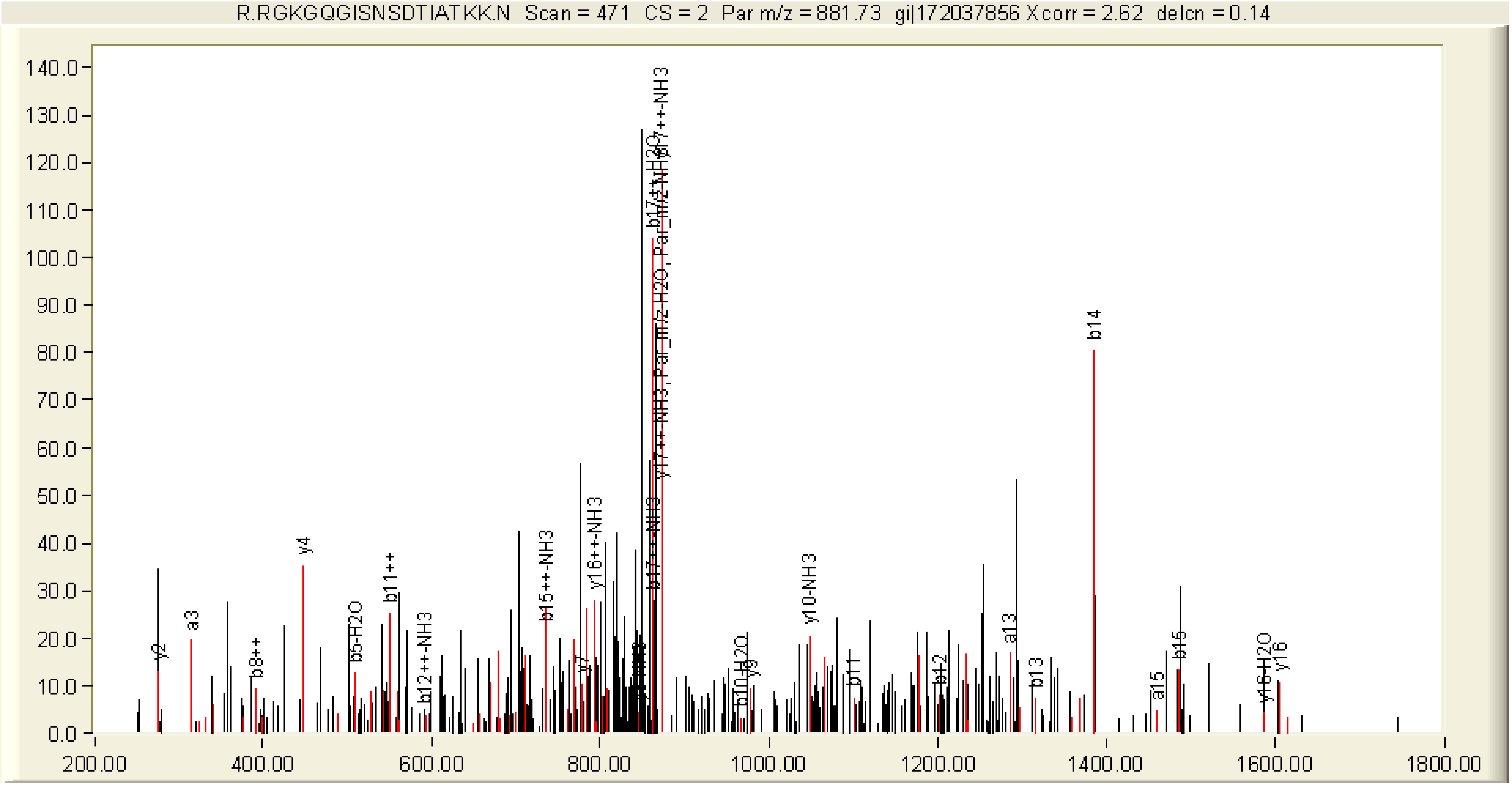

R.RGKGQGISNSDTIATKK.N Scan = 471 CS = 2 Par m/z = 881.73 gi|172037856 Xcorr = 2.62 delcn = 0.14
140.0
130.0
120.0
110.0
100.0
90.0
80.0
70.0
60.0
50.0
40.0
30.0
20.0
10.0
0.0
200.00
400.00
600.00
800.00
1000.00
1200.00
1400.00
1600.00
1800.00
y2
a3
b8++
y4
b5-H2O
b11++
b12++-NH3
b15++-NH3
y7
y16++-NH3
b17++-NH3
y10-NH3
b10-H2O
y9
b11
b12
a13
b13
b14
a15
b15
y16-H2O
y16

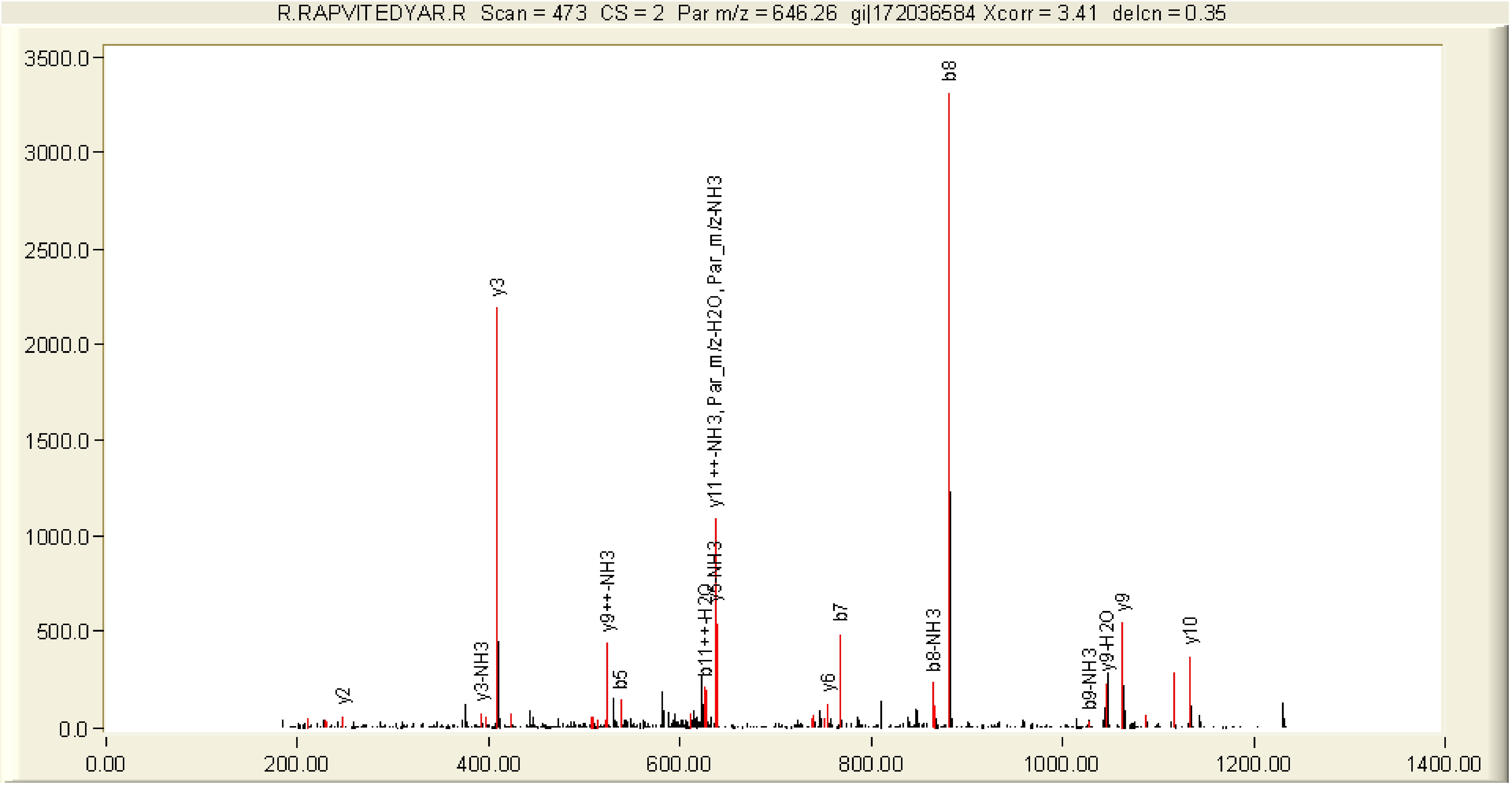

R.RAPVITEDYAR.R Scan = 473 CS = 2 Par m/z = 646.26 gi|172036584 Xcorr = 3.41 delcn = 0.35
b8
y11++-NH3, Par_m/z-H2O, Par_m/z-NH3
y3
y9++-NH3
y5-NH3
b11++-H2O
b7
b8-NH3
y9
y9-H2O
b9-NH3
y10
y3-NH3
b5
y6
y2
3500.0
3000.0
2500.0
2000.0
1500.0
1000.0
500.0
0.0
0.00
200.00
400.00
600.00
800.00
1000.00
1200.00
1400.00

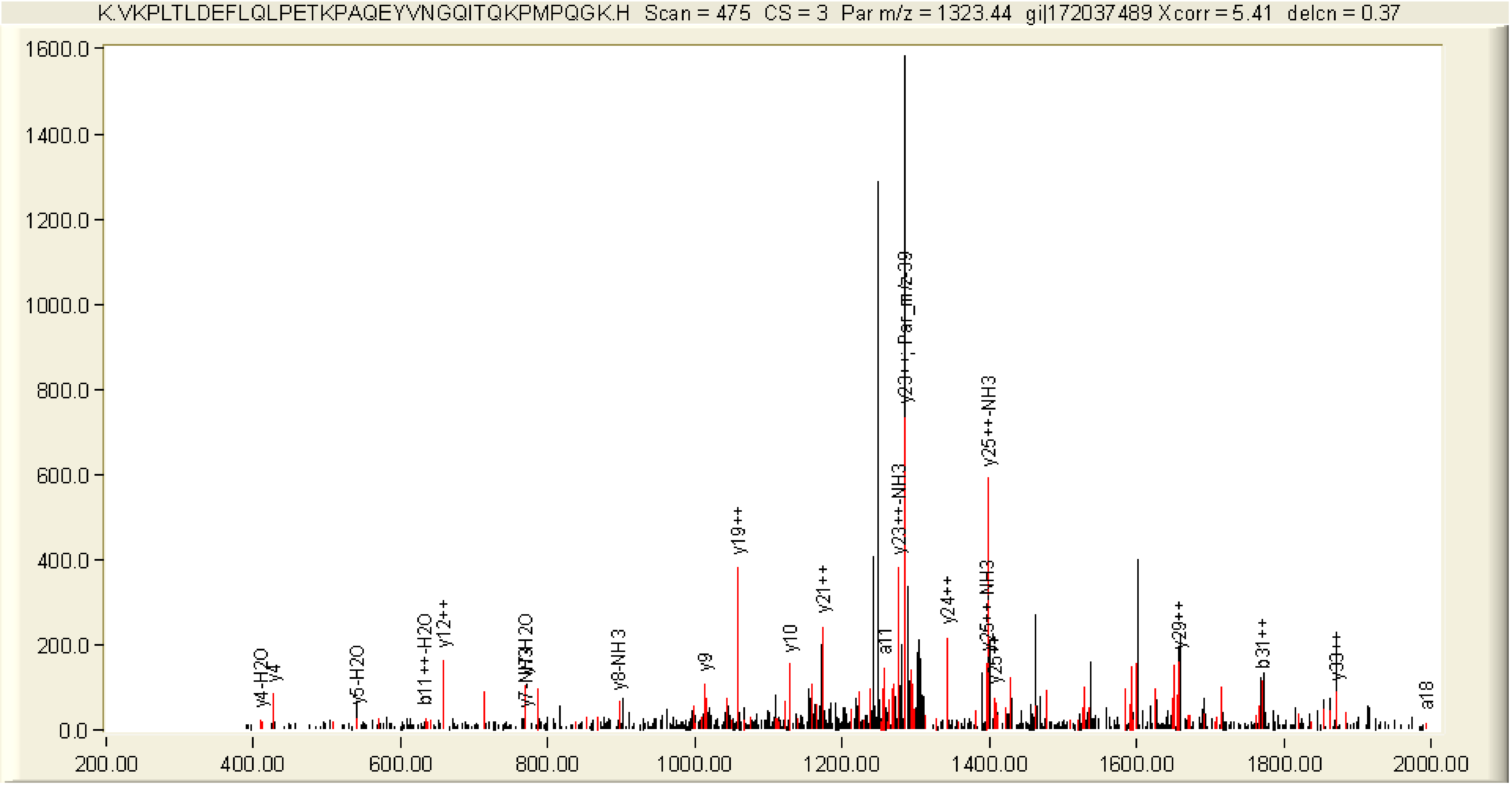
K.VKPLTLDEFLQLPETKPAQEYVNGQITQKPMPQGK.H Scan = 475 CS = 3 Par m/z = 1323.44 gi|172037489 Xcorr = 5.41 delcn = 0.37
1600.0
1400.0
1200.0
1000.0
800.0
600.0
400.0
200.0
0.0
200.00
400.00
600.00
800.00
1000.00
1200.00
1400.00
1600.00
1800.00
2000.00
y4-H2O
y4
y5-H2O
b11++-H2O
y12++
y8-NH3
y9
y19++
y10
y21++
a11
y23++-NH3
y24++
y25++-NH3
y29++
b31++
y33++
a18

K.MAGASANHNLLTGK.L Scan = 477 CS = 2 Par m/z = 692.71 gi|172038967 Xcorr = 3.57 delcn = 0.37

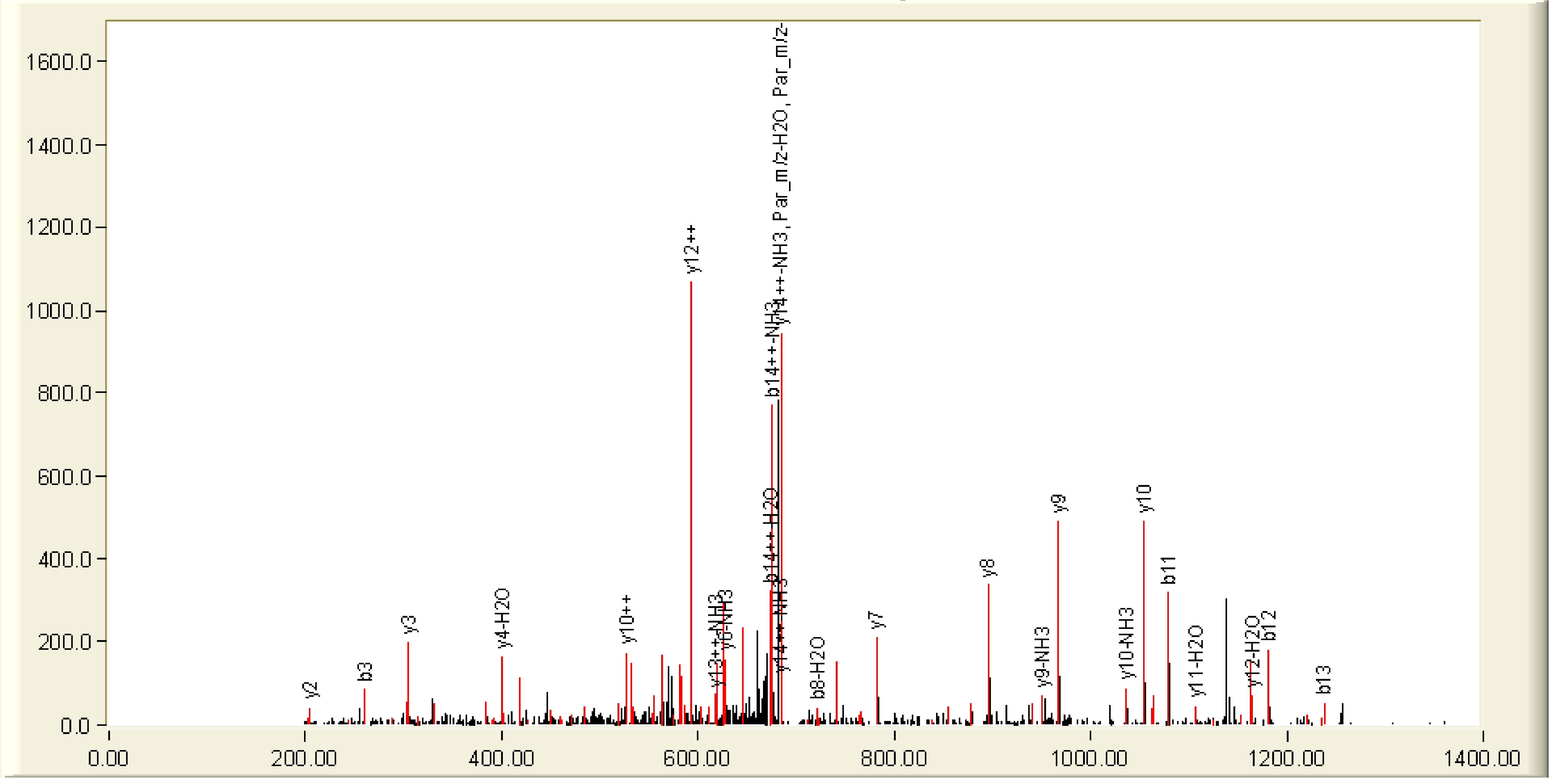

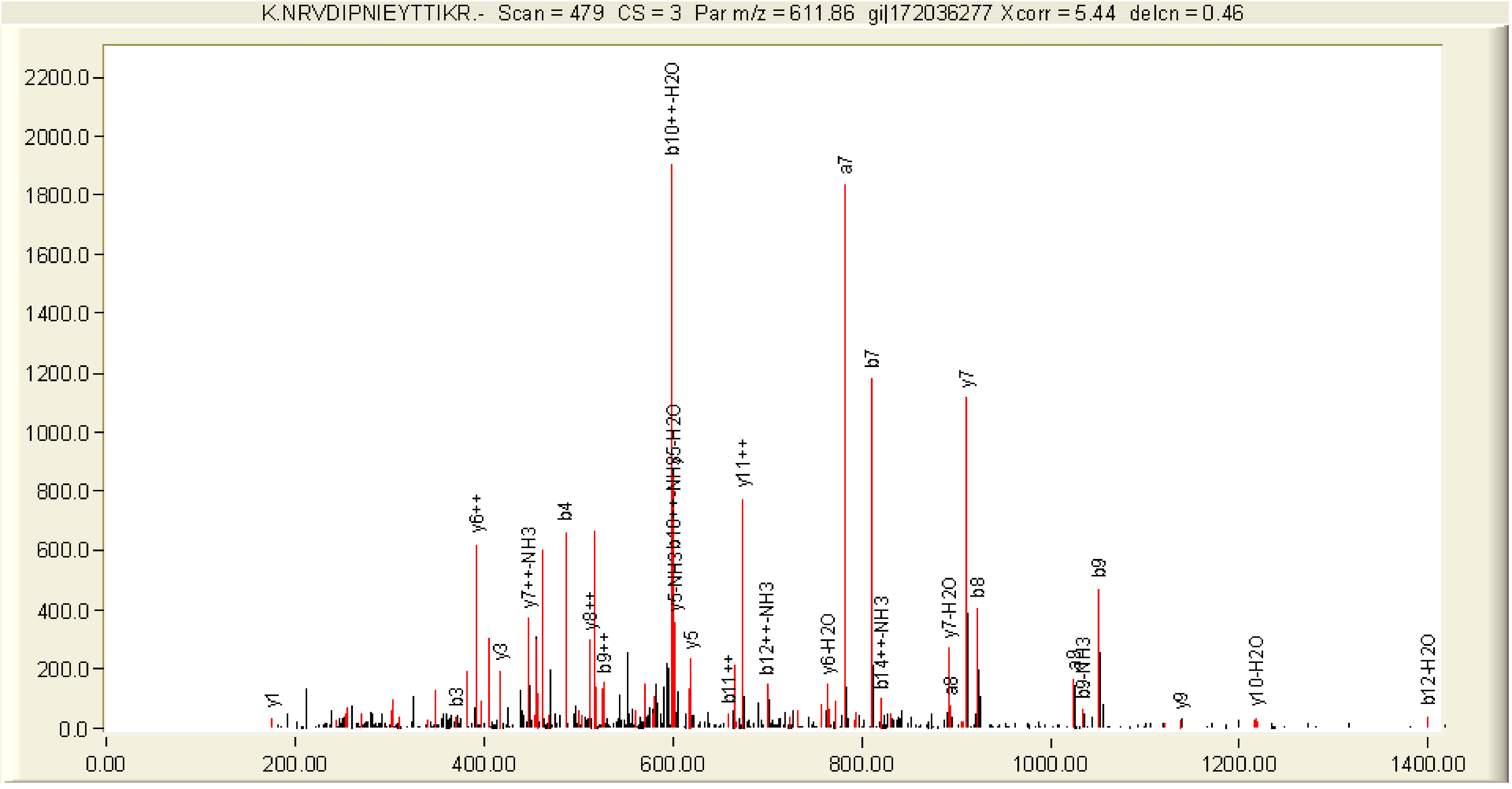

K.NRVDIPNIEYTTIKR.- Scan = 479 CS = 3 Par m/z = 611.86 gi|172036277 Xcorr = 5.44 delcn = 0.46
2200.0
2000.0
1800.0
1600.0
1400.0
1200.0
1000.0
800.0
600.0
400.0
200.0
0.0
0.00
200.00
400.00
600.00
800.00
1000.00
1200.00
1400.00
y1
b3
y6++
y3
y7++-NH3
b4
y8++
b9++
b10++-H2O
y5-NH3
y5
b11++
y11++
b12++-NH3
y6-H2O
a7
b7
b14++-NH3
a8
y7-H2O
y7
b8
b9
y9
y10-H2O
b12-H2O

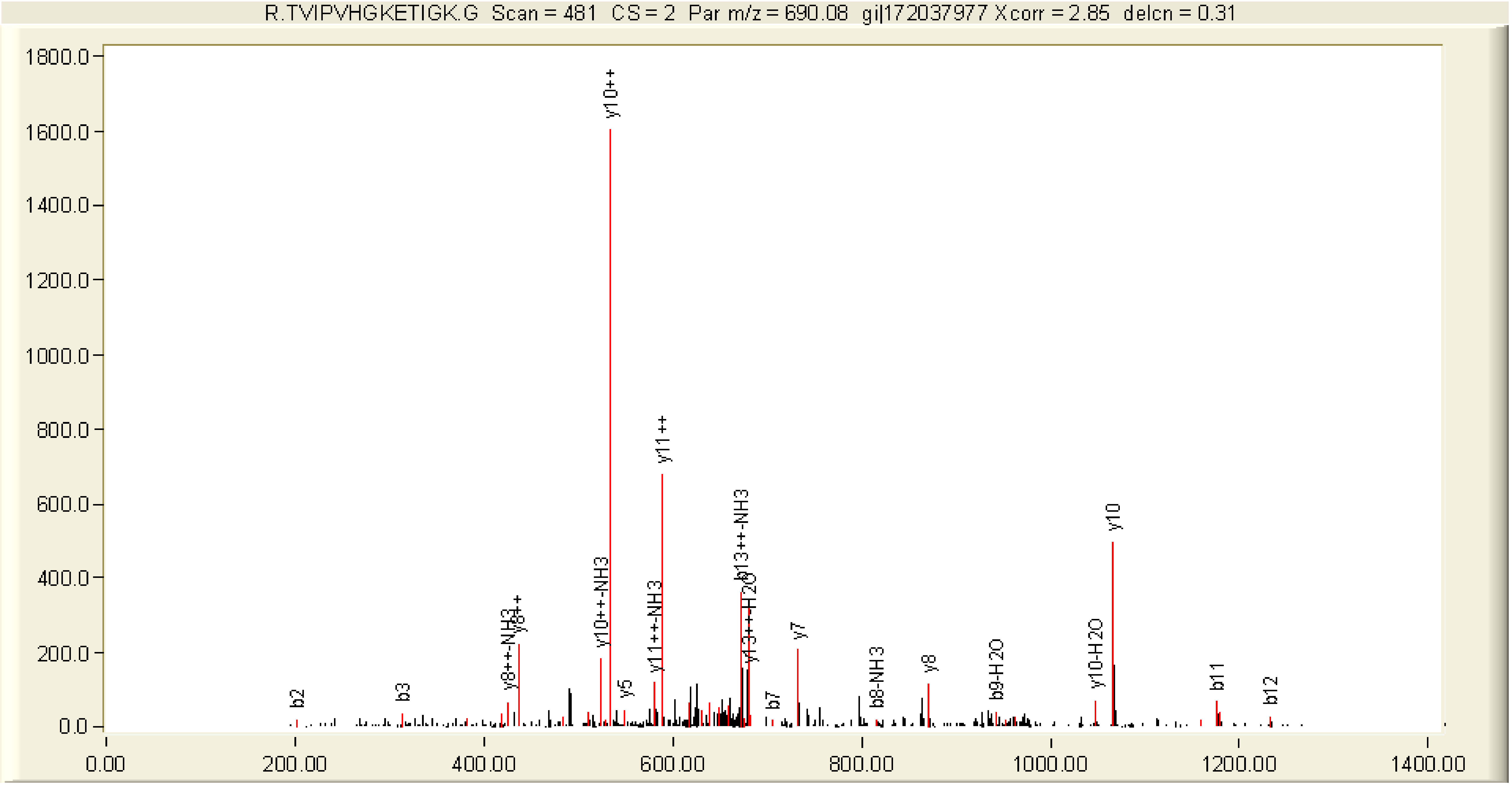

R.TVIPVHGKETIGK.G Scan = 481 CS = 2 Par m/z = 690.08 gi|172037977 Xcorr = 2.85 delcn = 0.31
1800.0
1600.0
1400.0
1200.0
1000.0
800.0
600.0
400.0
200.0
0.0
0.00
200.00
400.00
600.00
800.00
1000.00
1200.00
1400.00
y10++
y11++
b13++-NH3
y10
y8++-NH3
y8++
y10++-NH3
y11++-NH3
y7
b8-NH3
y8
b9-H2O
y10-H2O
b11
b12
b2
b3
y5
b7

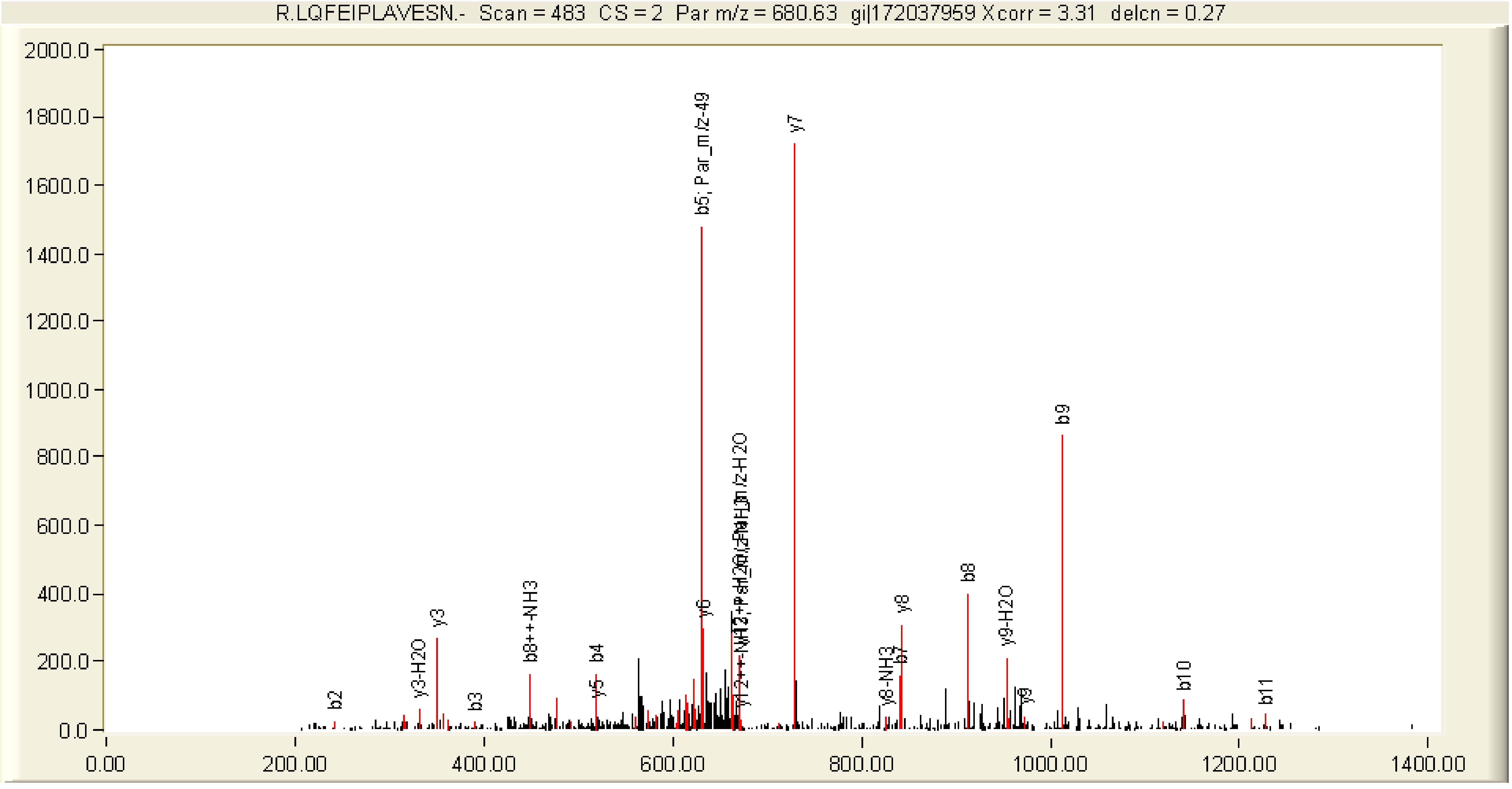

R.LQFEIPLAVESN.- Scan = 483 CS = 2 Par m/z = 680.63 gi|172037959 Xcorr = 3.31 delcn = 0.27
2000.0
1800.0
1600.0
1400.0
1200.0
1000.0
800.0
600.0
400.0
200.0
0.0
0.00
200.00
400.00
600.00
800.00
1000.00
1200.00
1400.00
b5; Par_m/z-49
y7
b9
b8
y9-H2O
y8
b7
y8-NH3
y6
y3
y3-H2O
b8++-NH3
b4
y5
b3
b2
b10
b11
y9

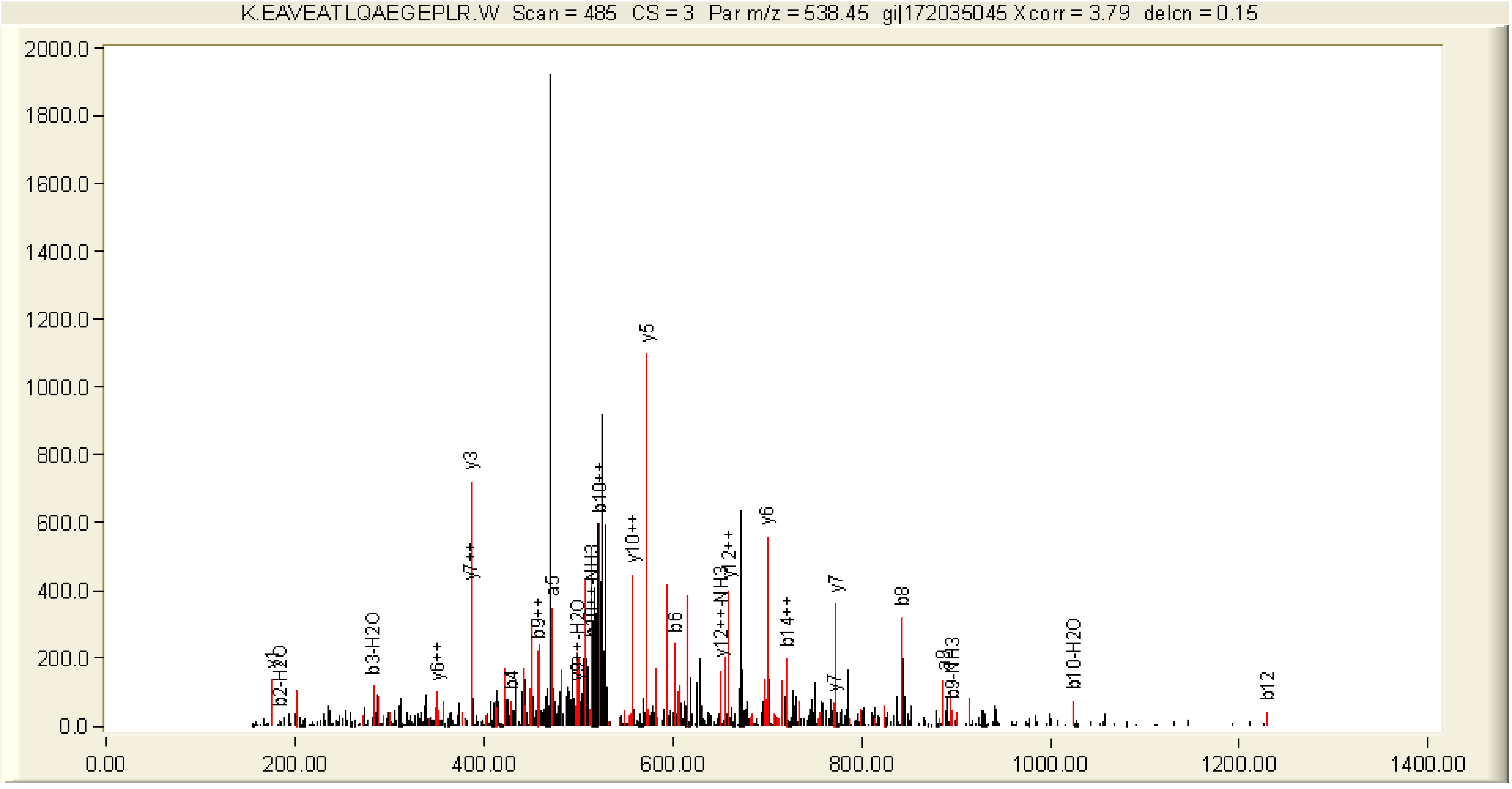
K.EAVEATLQAEGEPLR.W Scan = 485 CS = 3 Par m/z = 538.45 gi|172035045 Xcorr = 3.79 delcn = 0.15
2000.0
1800.0
1600.0
1400.0
1200.0
1000.0
800.0
600.0
400.0
200.0
0.0
0.00
200.00
400.00
600.00
800.00
1000.00
1200.00
1400.00
y5
y3
b10++
y6
y10++
y7++
y12++
a5
y7
b8
b9++
b6
b14++
b3-H2O
b10-H2O
b12
y6++
b4
b9-NH3
y7

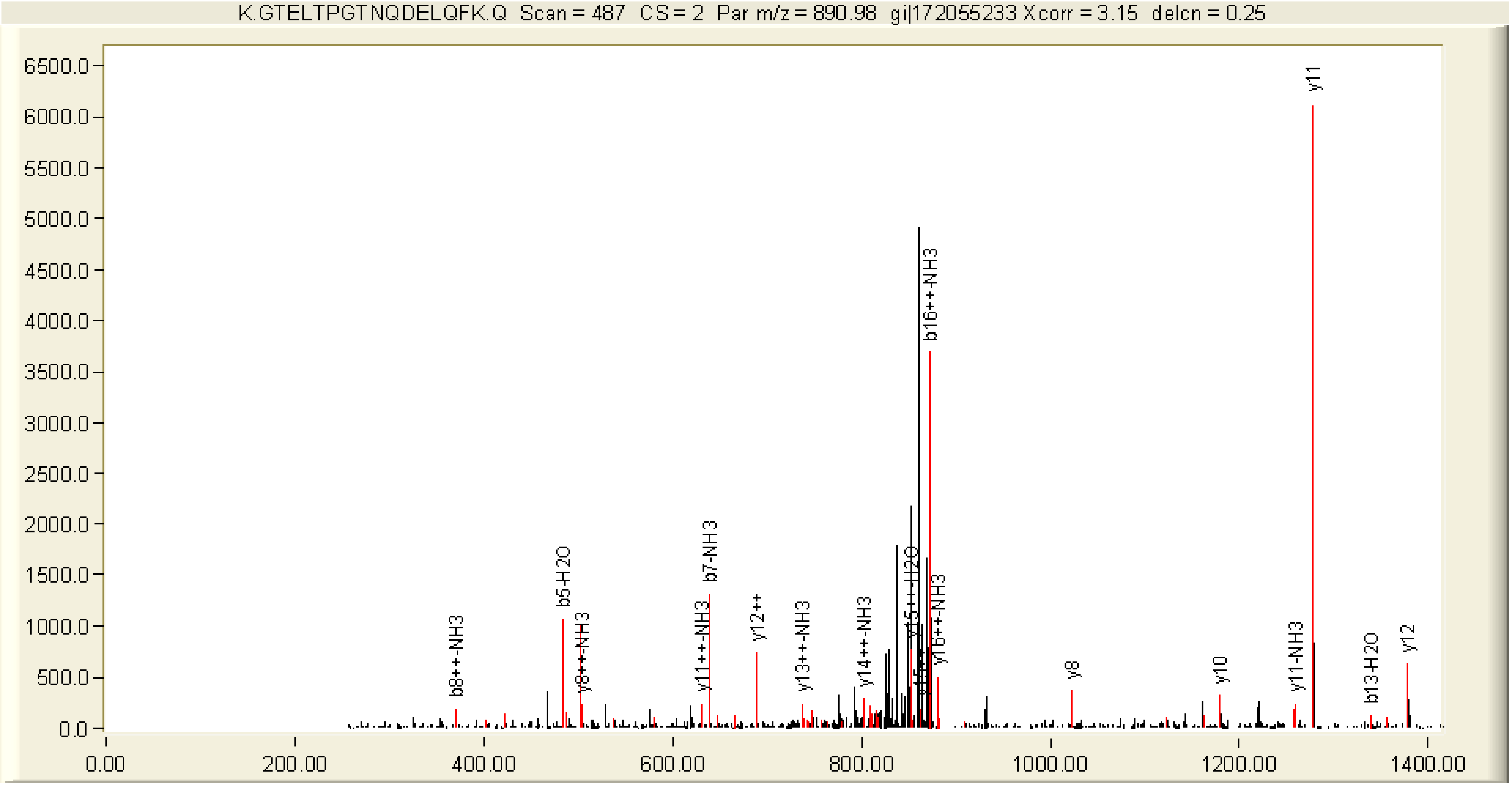
K.GTELTPGTNQDELQFK.Q Scan = 487 CS = 2 Par m/z = 890.98 gi|172055233 Xcorr = 3.15 delcn = 0.25
y11
b16++-NH3
b5-H2O
b7-NH3
b8++-NH3
y8++-NH3
y11++-NH3
y12++
y13++-NH3
y14++-NH3
y15++-H2O
y16++-NH3
y8
y10
y11-NH3
b13-H2O
y12
0.0
500.0
1000.0
1500.0
2000.0
2500.0
3000.0
3500.0
4000.0
4500.0
5000.0
5500.0
6000.0
6500.0
0.00
200.00
400.00
600.00
800.00
1000.00
1200.00
1400.00

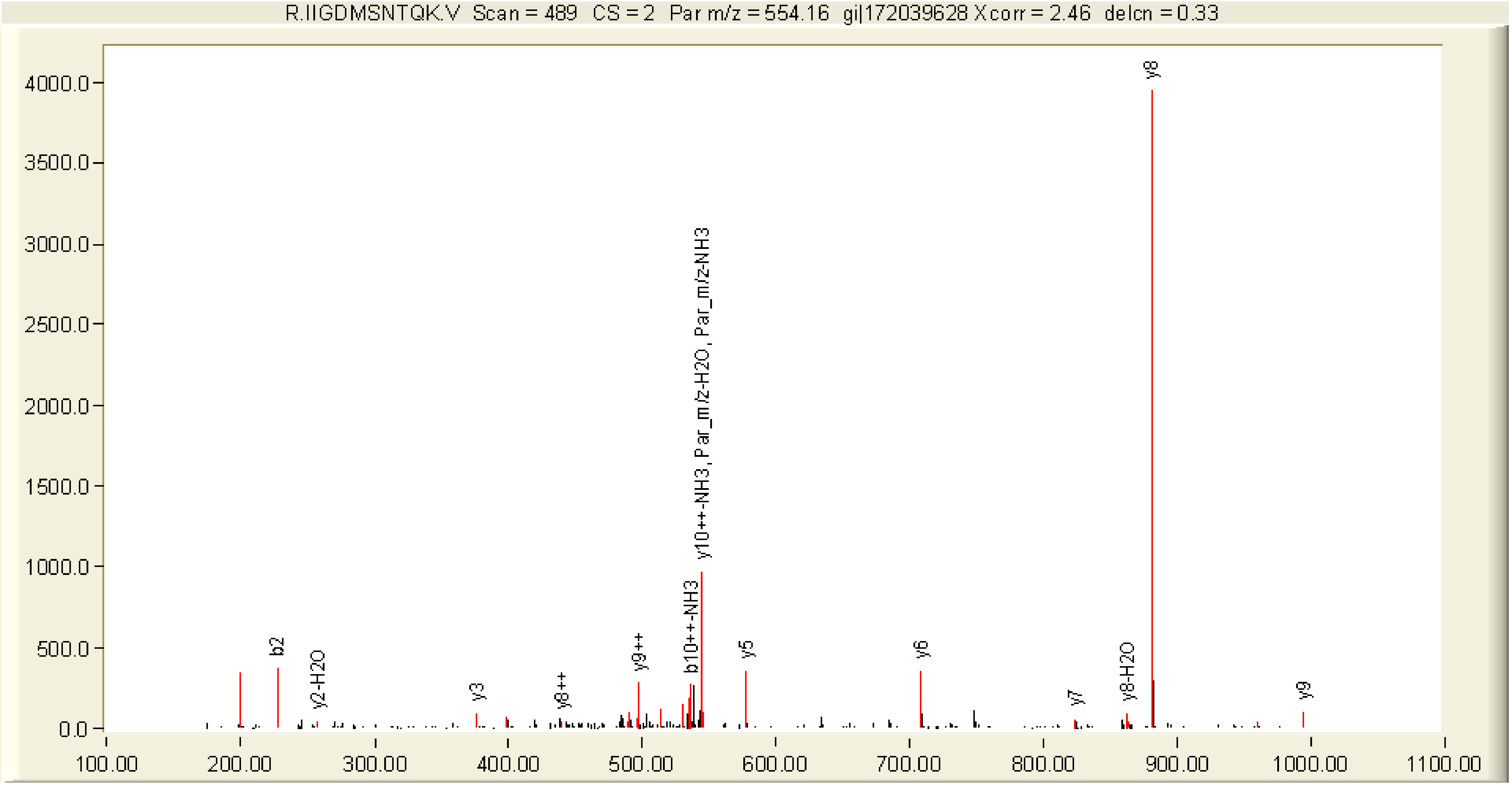
R.IIGDMSNTQK.V Scan = 489 CS = 2 Par m/z = 554.16 gi|172039628 Xcorr = 2.46 delcn = 0.33
4000.0
3500.0
3000.0
2500.0
2000.0
1500.0
1000.0
500.0
0.0
y8
y10++-NH3, Par_m/z-NH3, Par_m/z-H2O, Par_m/z-NH3
b2
y2-H2O
y3
y8++
y9++
b10++-NH3
y5
y6
y7
y8-H2O
y9
100.00
200.00
300.00
400.00
500.00
600.00
700.00
800.00
900.00
1000.00
1100.00

R.TLESLAVEGYR.S Scan = 491 CS = 2 Par m/z = 620.67 gi|172055082 Xcorr = 3.29 delcn = 0.3

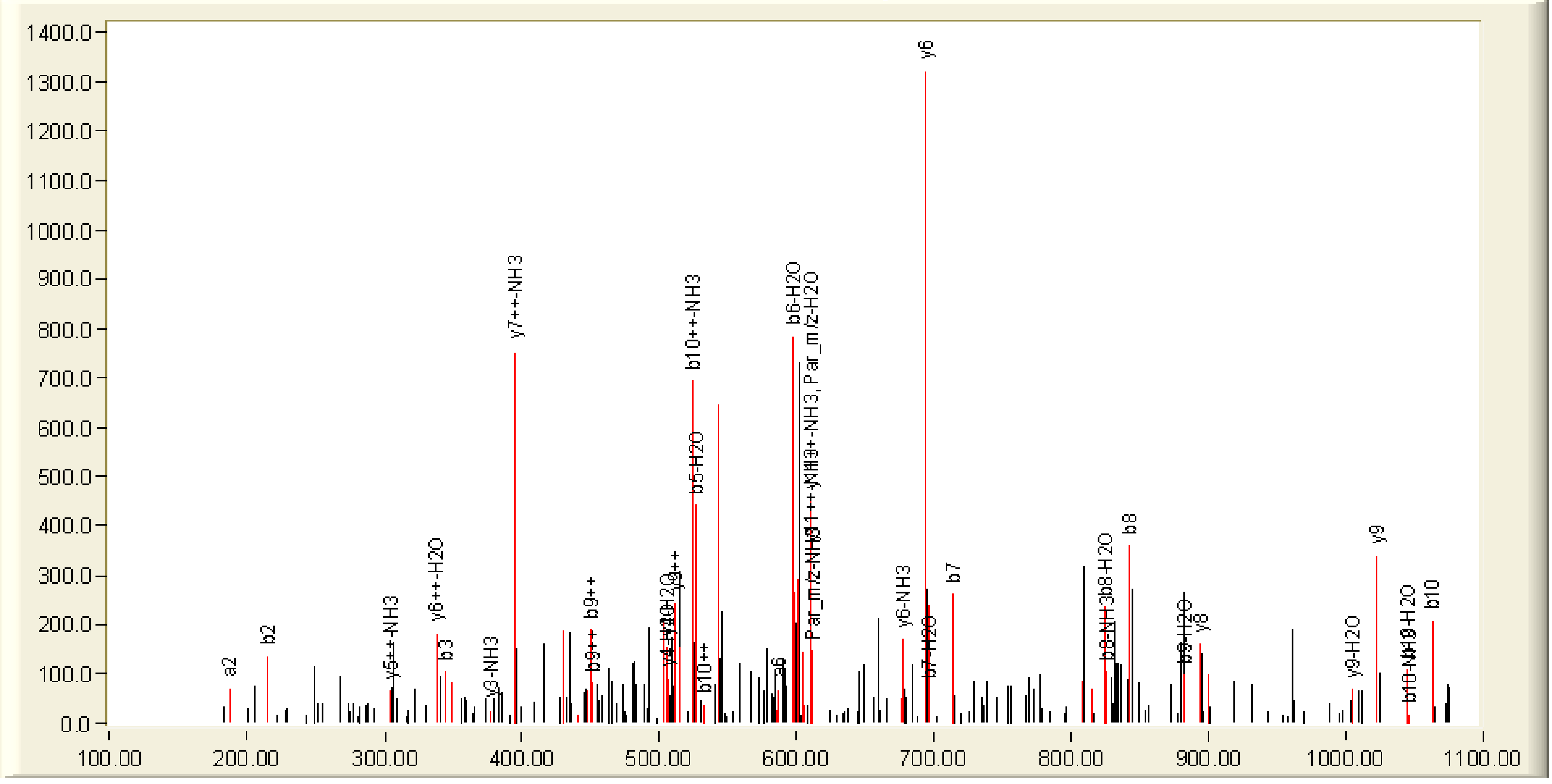

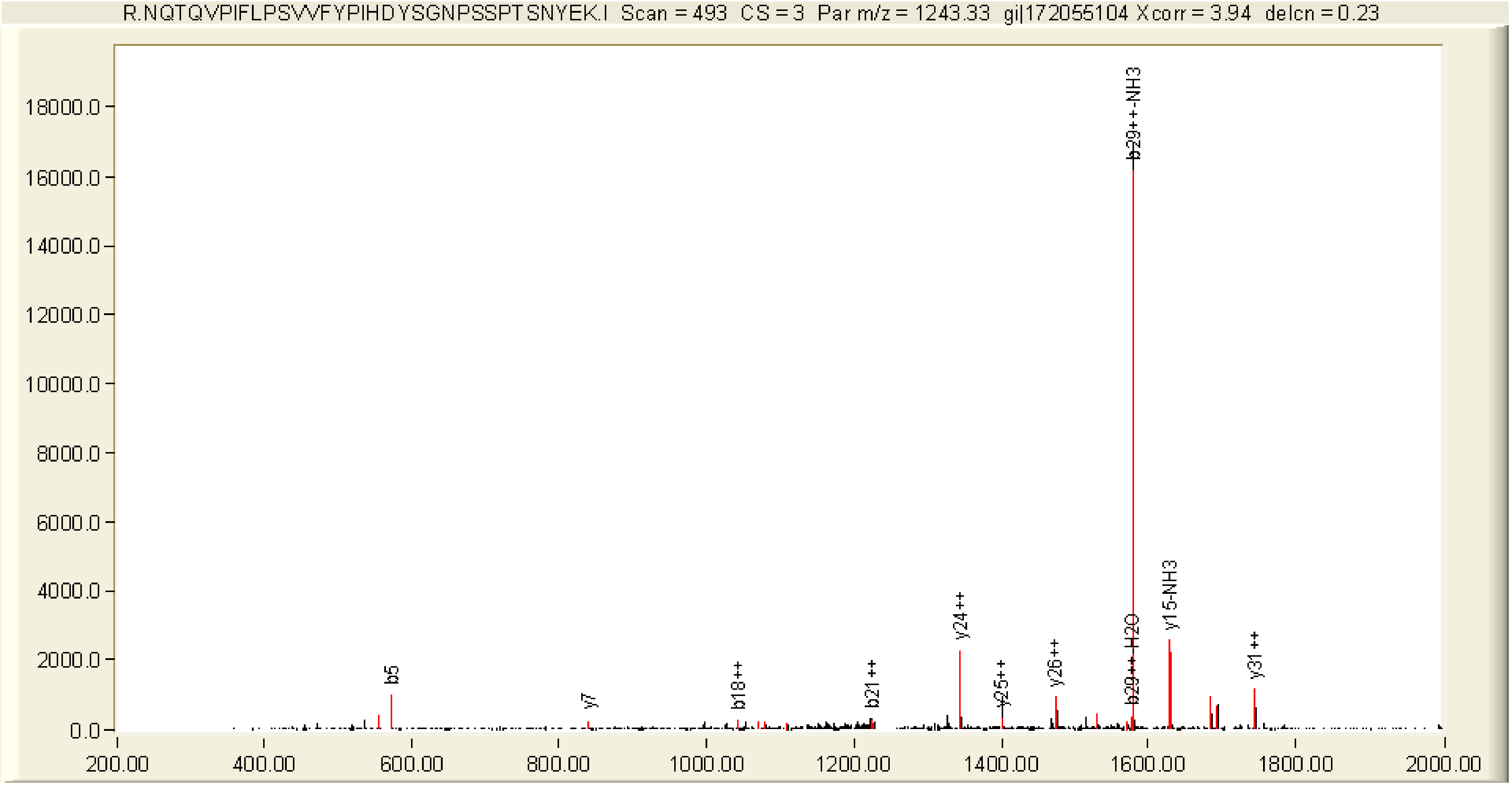

R.NQTQVPIFLPSVVFYPIHDYSGNPSSPTSNYEK.I Scan = 493 CS = 3 Par m/z = 1243.33 gi|172055104 Xcorr = 3.94 delcn = 0.23
b29++-NH3
y15-NH3
y24++
b29++-H2O
y26++
y31++
y25++
b5
b18++
b21++
y7
18000.0
16000.0
14000.0
12000.0
10000.0
8000.0
6000.0
4000.0
2000.0
0.0
200.00
400.00
600.00
800.00
1000.00
1200.00
1400.00
1600.00
1800.00
2000.00

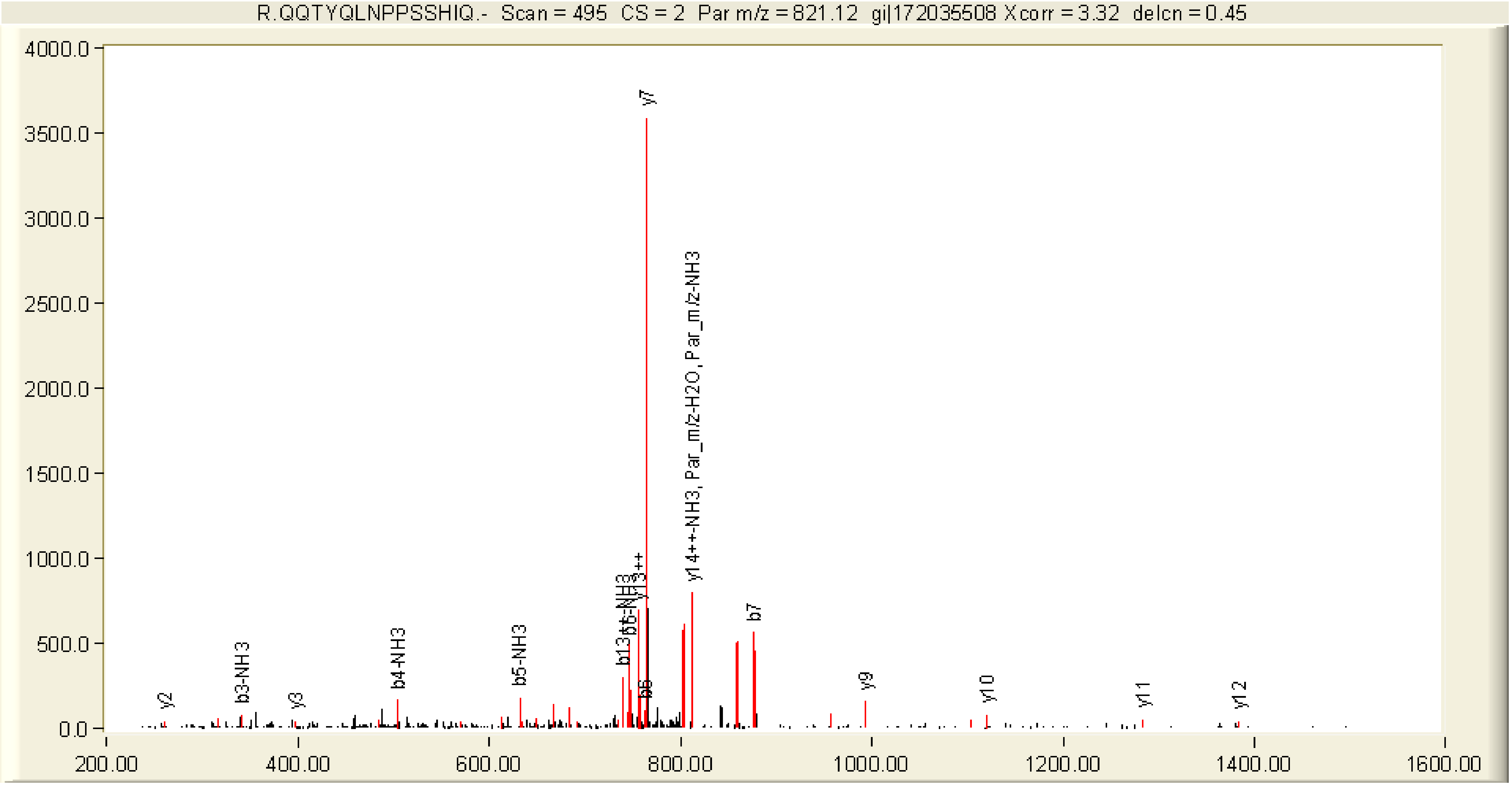
R.QQTYQLNPPSSHIQ.- Scan = 495 CS = 2 Par m/z = 821.12 gi|172035508 Xcorr = 3.32 delcn = 0.45
4000.0
3500.0
3000.0
2500.0
2000.0
1500.0
1000.0
500.0
0.0
200.00
400.00
600.00
800.00
1000.00
1200.00
1400.00
1600.00
y7
y14++-NH3, Par_m/z-H2O, Par_m/z-NH3
b7
y2
b3-NH3
y3
b4-NH3
b5-NH3
b6
y9
y10
y11
y12

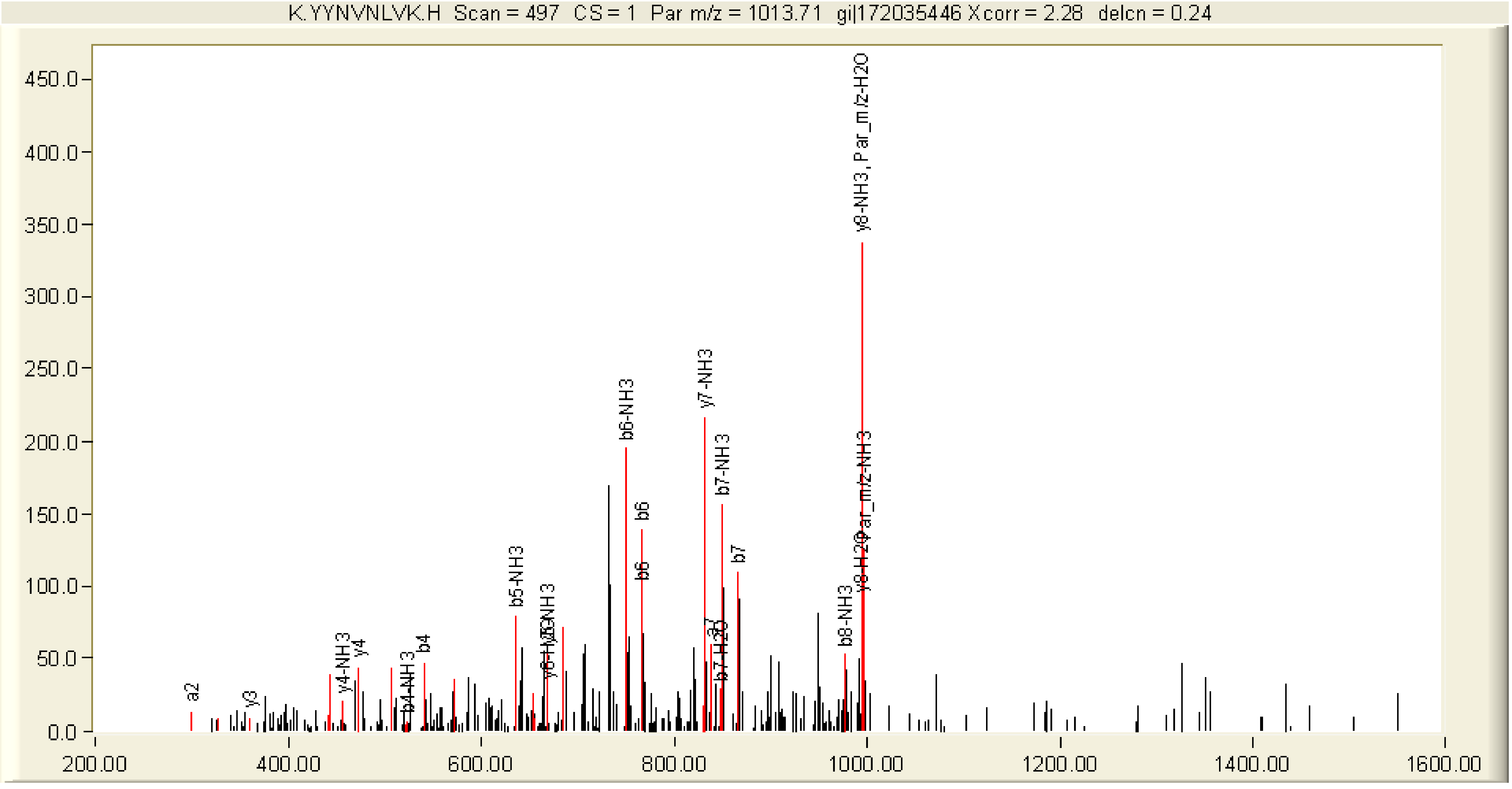

K.YYNVNLVK.H Scan = 497 CS = 1 Par m/z = 1013.71 gi|172035446 Xcorr = 2.28 delcn = 0.24
y8-NH3, Par_m/z-H2O
y7-NH3
b6-NH3
b7-NH3
b6
b7
b5-NH3
b8-NH3
y4-NH3
y4
b4
b4-NH3
a2
y3

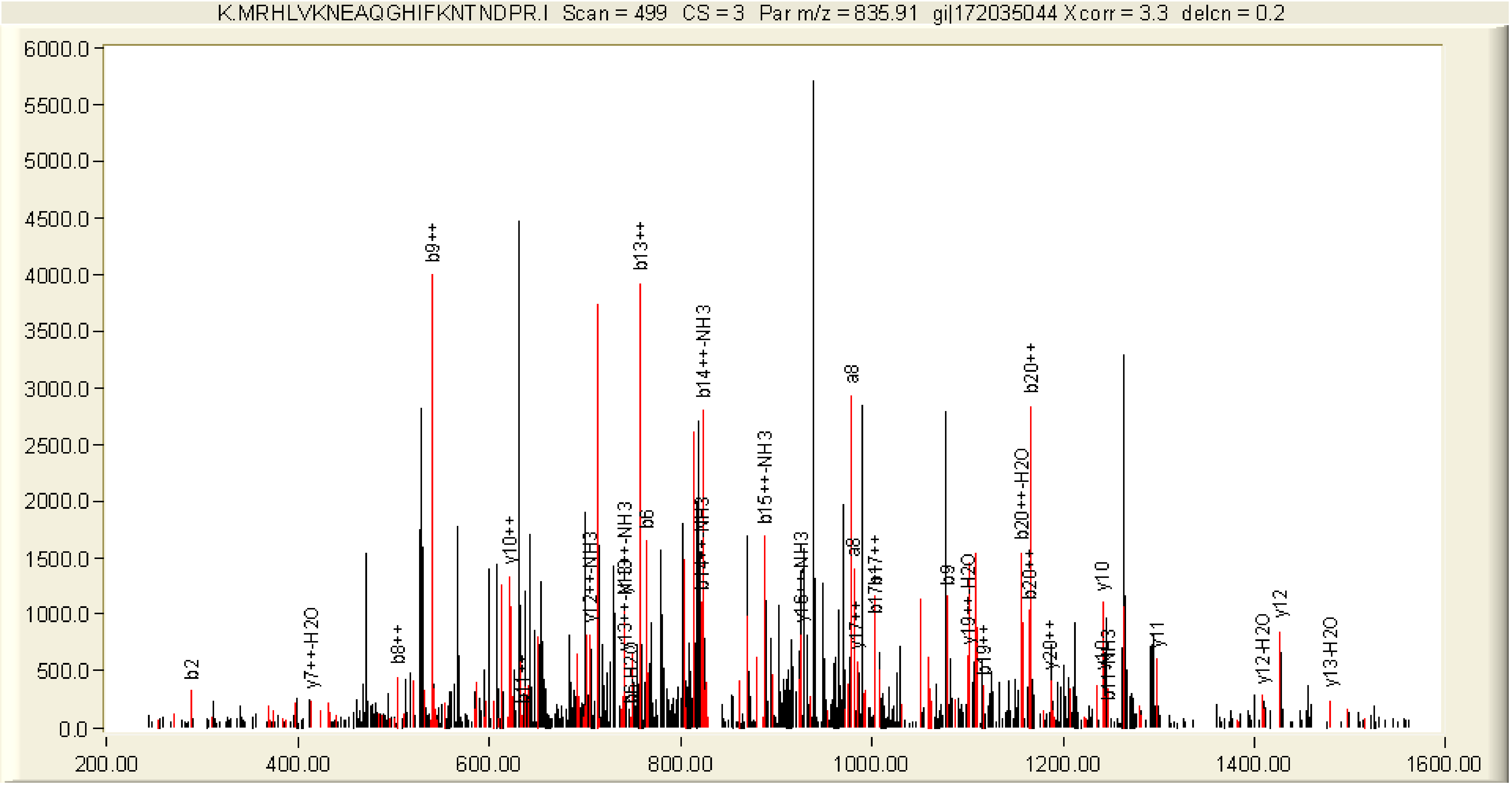
K.MRHLVKNEAQGHIFKNTNDPR.I Scan = 499 CS = 3 Par m/z = 835.91 gi|172035044 Xcorr = 3.3 delcn = 0.2
6000.0
5500.0
5000.0
4500.0
4000.0
3500.0
3000.0
2500.0
2000.0
1500.0
1000.0
500.0
0.0
200.00
400.00
600.00
800.00
1000.00
1200.00
1400.00
1600.00
b2
y7++-H2O
b8++
b9++
y10++
y12++-NH3
b13++
b6
b14++-NH3
b15++-NH3
a8
b20++
b20++-H2O
b9
y10
y11
y12-H2O
y12
y13-H2O
y20++
b17++
y17++
b19++

K.PVSSVLTCTGAVFIASPFLFLDFK.Q Scan = 501 CS = 3 Par m/z = 853.97 gi|172039126 Xcorr = 3.26 delcn = 0.17

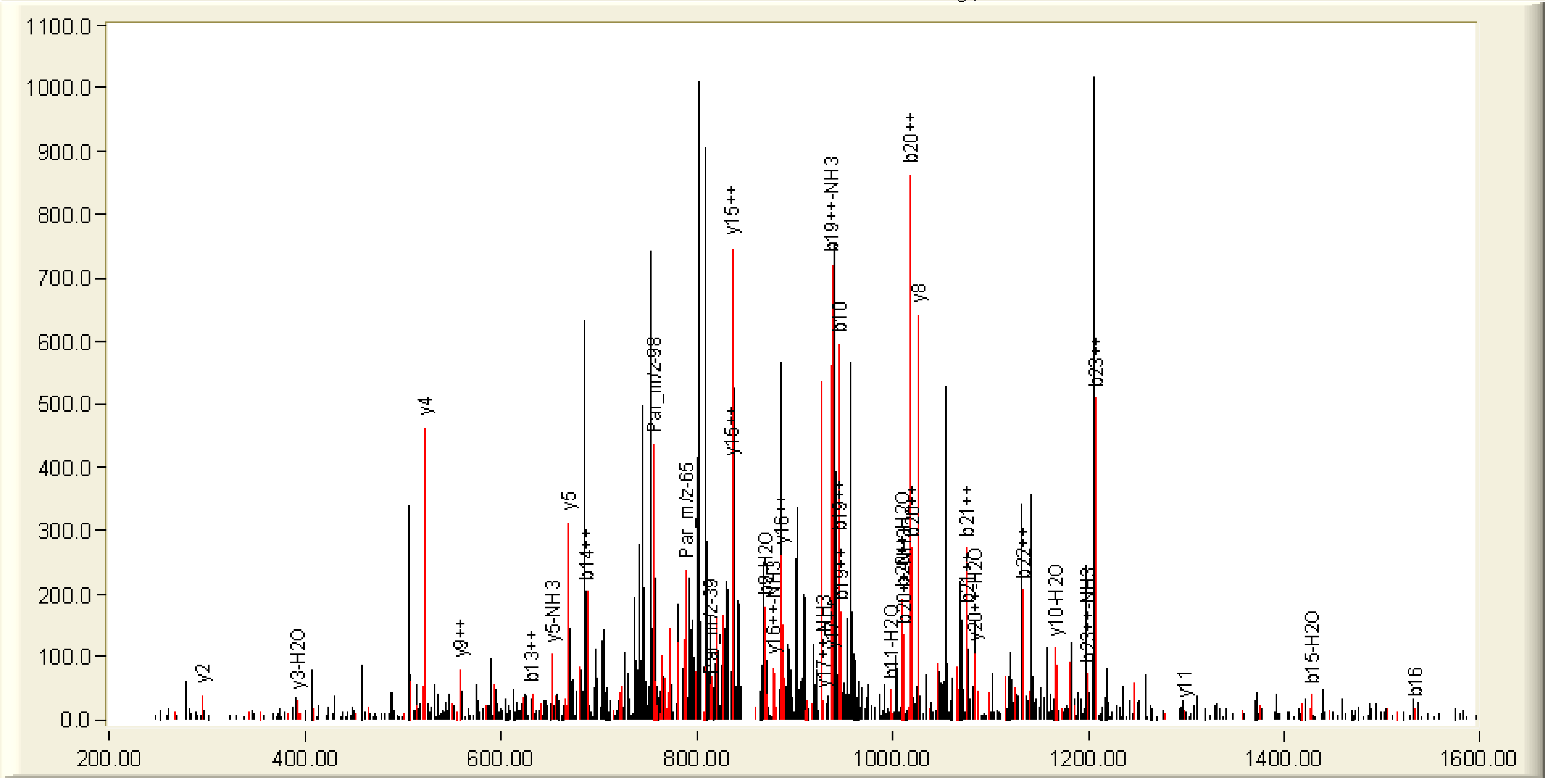

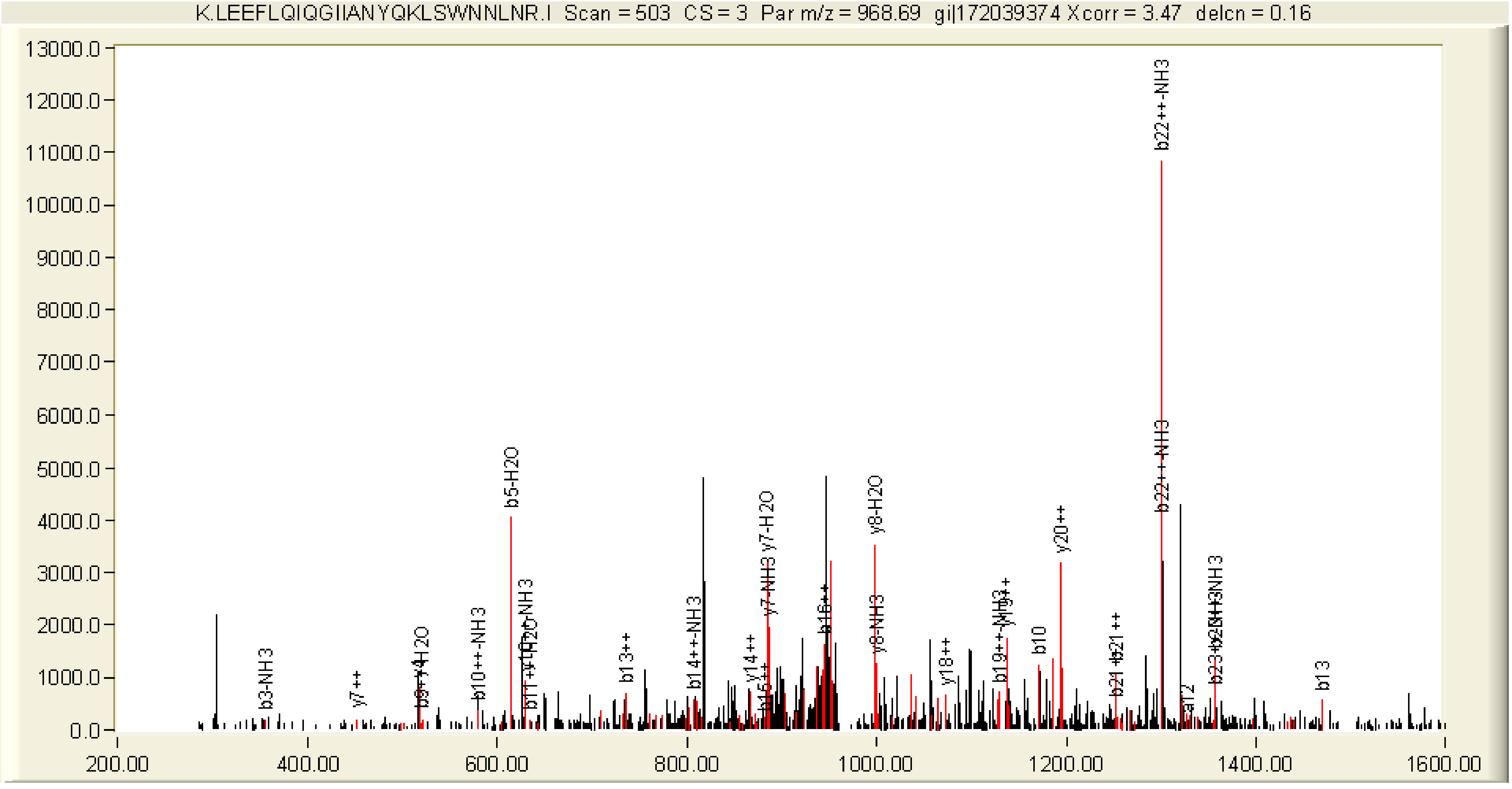
K.LEEFLQIQGIIANYQKLSWNNLNR.I Scan = 503 CS = 3 Par m/z = 968.69 gi|172039374 Xcorr = 3.47 delcn = 0.16
13000.0
12000.0
11000.0
10000.0
9000.0
8000.0
7000.0
6000.0
5000.0
4000.0
3000.0
2000.0
1000.0
0.0
200.00
400.00
600.00
800.00
1000.00
1200.00
1400.00
1600.00
b22++-NH3
b5-H2O
y8-H2O
y20++
y7-NH3 y7-H2O
b3-NH3
y7++
b10++-NH3
b13++
b14++-NH3
y14++
b16++
y8-NH3
y18++
b10
b21++
b13

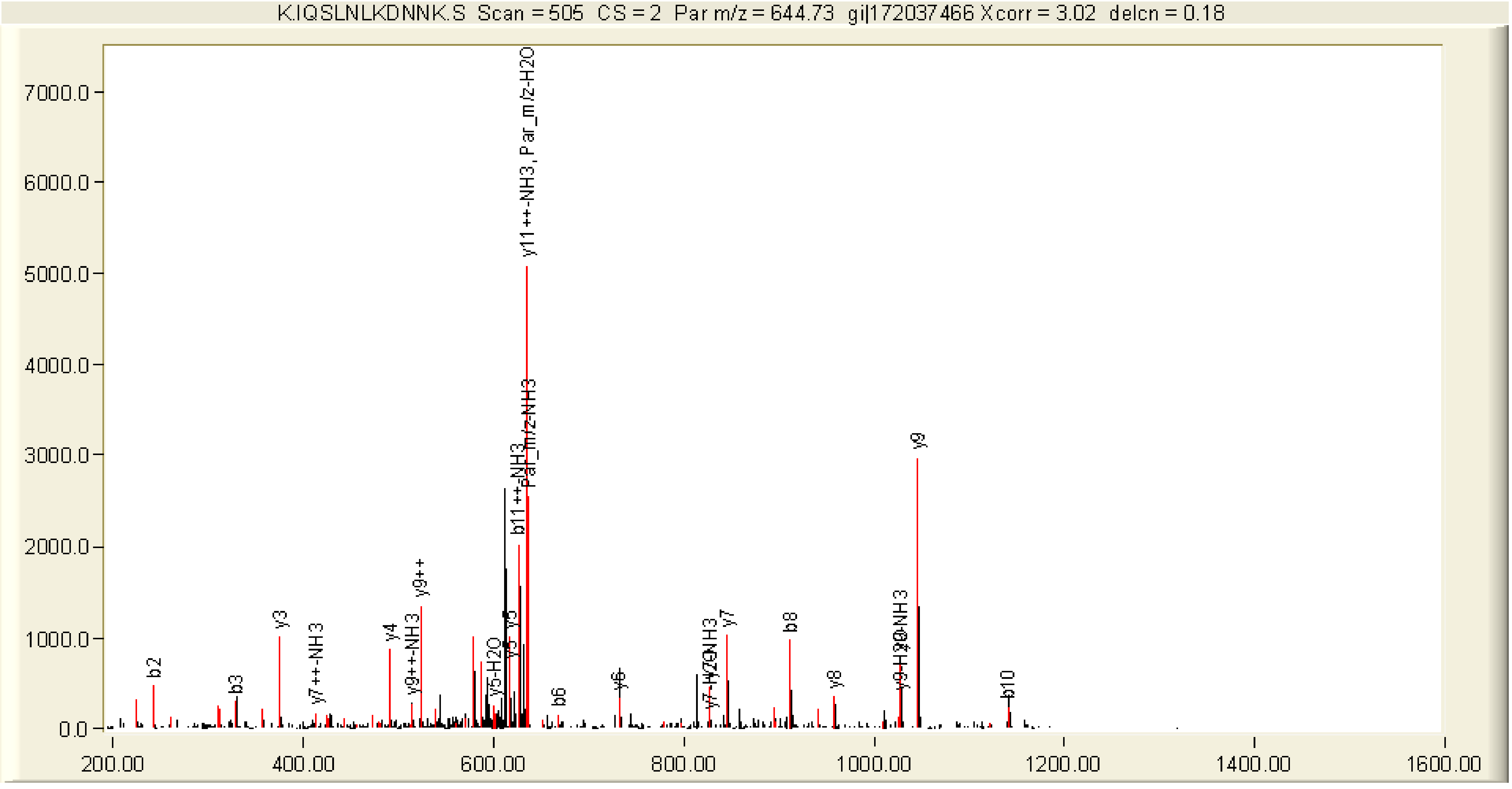

K.IQSLNLKDNNK.S Scan = 505 CS = 2 Par m/z = 644.73 gi|172037466 Xcorr = 3.02 delcn = 0.18
y11++-NH3, Par_m/z-H2O
Par_m/z-NH3
b11++-NH3
y9
y9++
y3
y4
y9++-NH3
y7++-NH3
b2
b3
y5-H2O
y5
b6
y6
y7-NH3
y7
b8
y8
b10
0.0
1000.0
2000.0
3000.0
4000.0
5000.0
6000.0
7000.0
200.00
400.00
600.00
800.00
1000.00
1200.00
1400.00
1600.00

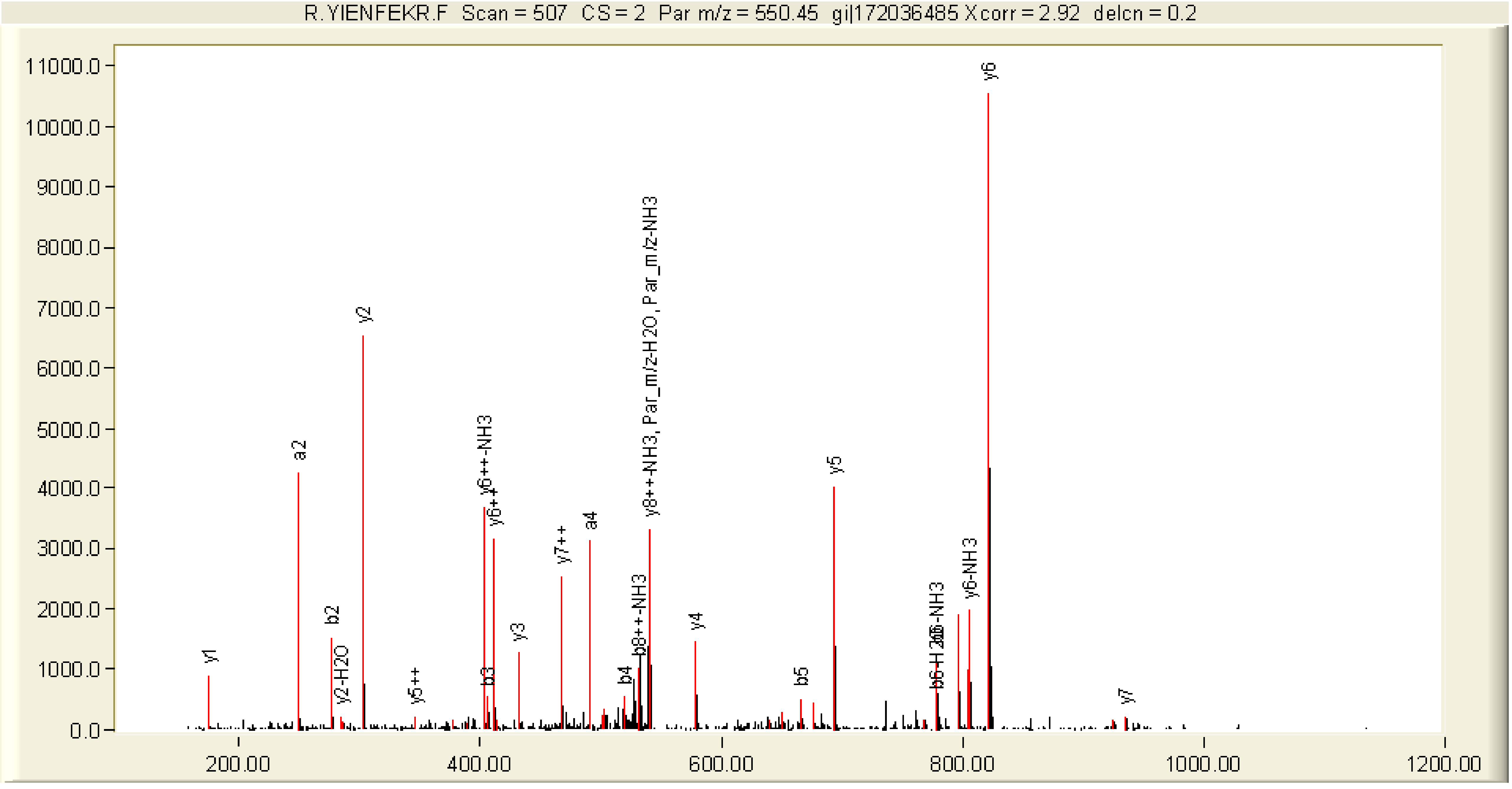
R.YIENFEKR.F Scan = 507 CS = 2 Par m/z = 550.45 gi|172036485 Xcorr = 2.92 delcn = 0.2
11000.0
10000.0
9000.0
8000.0
7000.0
6000.0
5000.0
4000.0
3000.0
2000.0
1000.0
0.0
200.00
400.00
600.00
800.00
1000.00
1200.00
y1
a2
b2
y2-H2O
y2
y5++
b3
y6++-NH3
y6++
y3
y7++
a4
b4
b8++-NH3
y8++-NH3, Par_m/z-H2O, Par_m/z-NH3
y4
b5
y5
b6-H2O
b6-NH3
y6-NH3
y6
y7

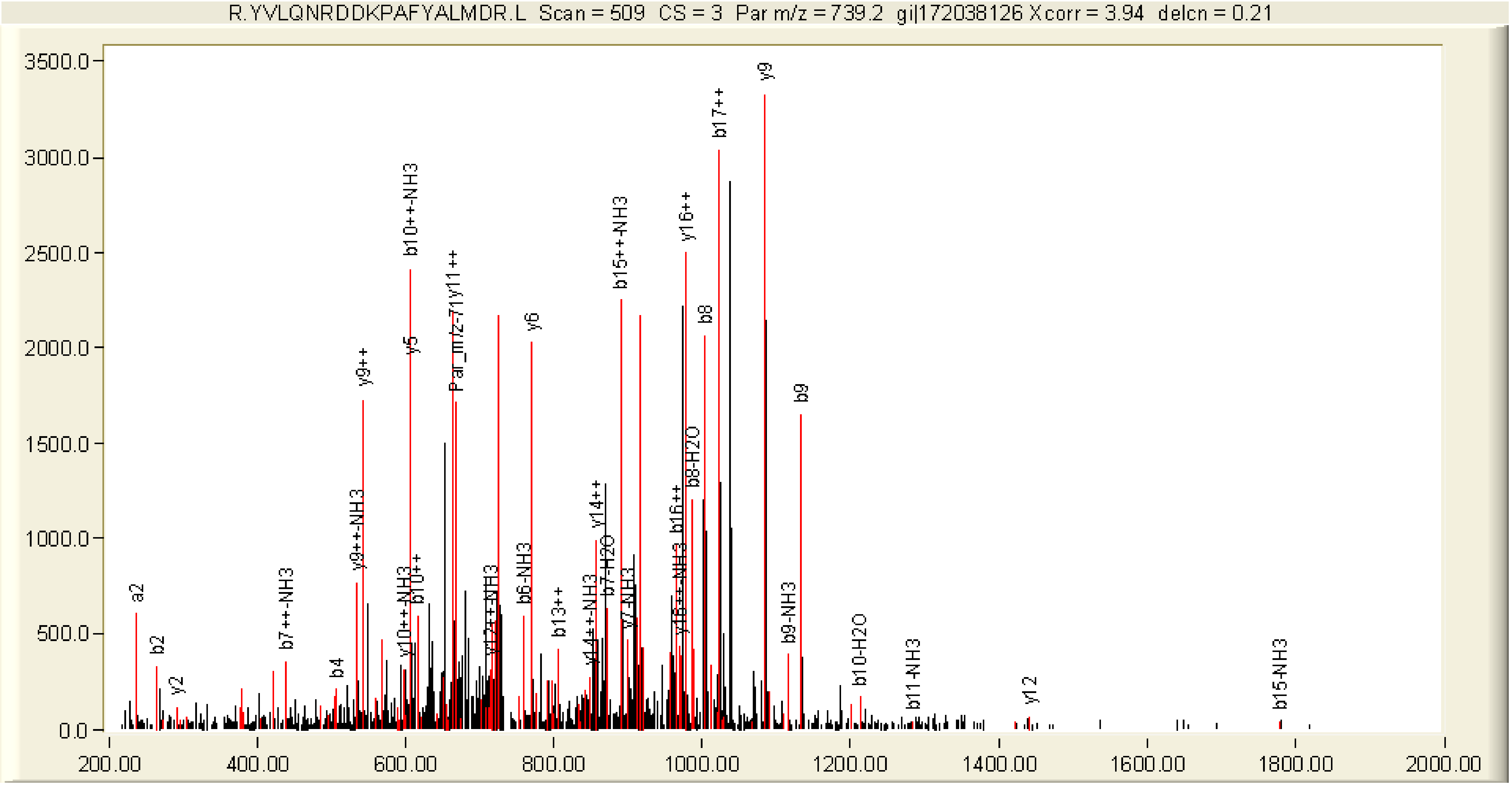
R.YVLQNRDDKPAFYALMDR.L Scan = 509 CS = 3 Par m/z = 739.2 gi|172038126 Xcorr = 3.94 delcn = 0.21
3500.0
3000.0
2500.0
2000.0
1500.0
1000.0
500.0
0.0
200.00
400.00
600.00
800.00
1000.00
1200.00
1400.00
1600.00
1800.00
2000.00
a2
b2
y2
b7++-NH3
b4
y9++-NH3
y9++
y10++-NH3
b10++
y5
b10++-NH3
Par_m/z-71y11++
y12++-NH3
b6-NH3
y6
b13++
y14++-NH3
y14++
b7-H2O
y7-NH3
b15++-NH3
y16++-NH3
b16++
y16++
b8-H2O
b8
b17++
y9
b9-NH3
b9
b10-H2O
b11-NH3
y12
b15-NH3

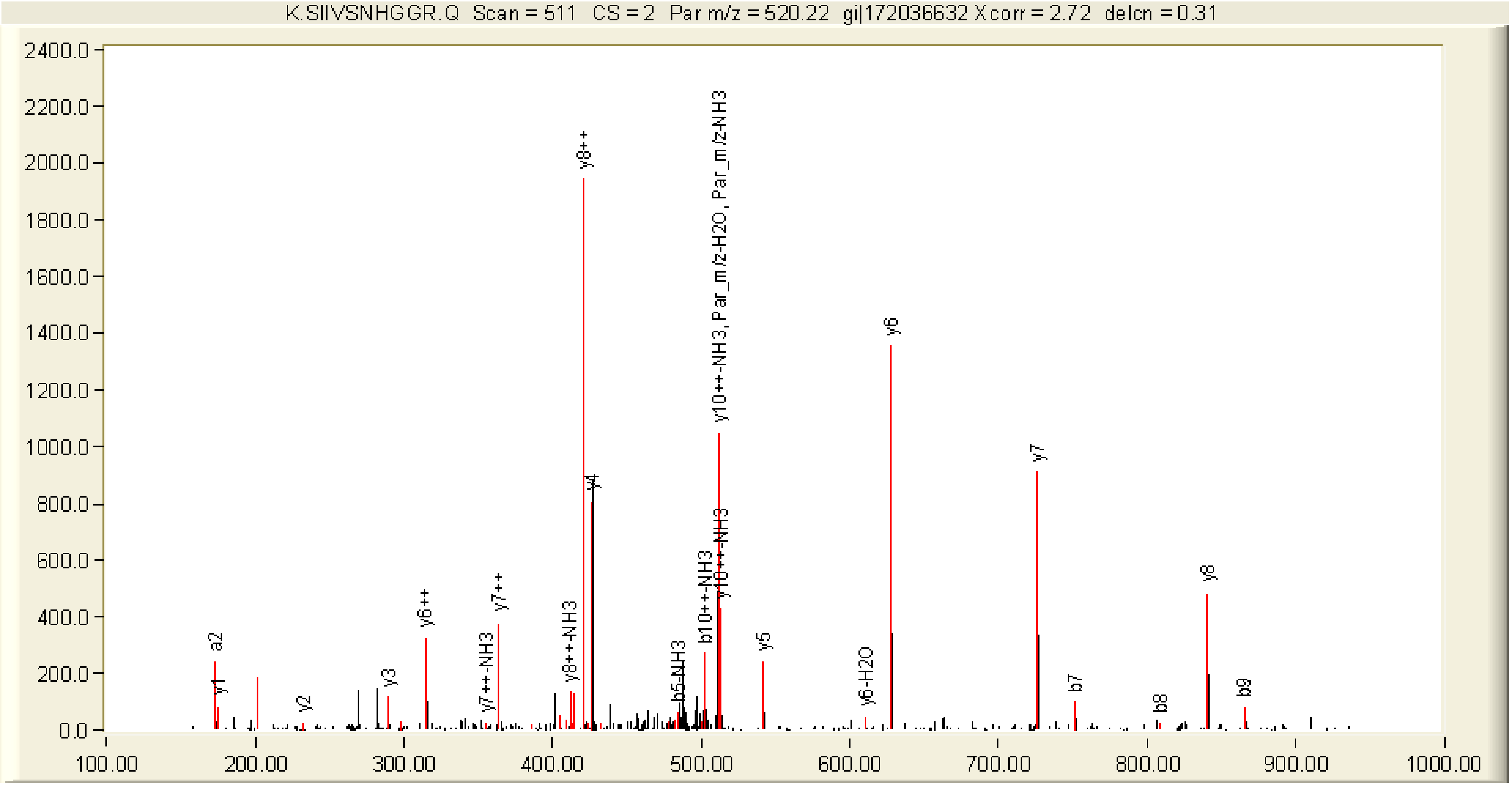
K.SIIVSNHGGR.Q Scan = 511 CS = 2 Par m/z = 520.22 gi|172036632 Xcorr = 2.72 delcn = 0.31
2400.0
2200.0
2000.0
1800.0
1600.0
1400.0
1200.0
1000.0
800.0
600.0
400.0
200.0
0.0
100.00
200.00
300.00
400.00
500.00
600.00
700.00
800.00
900.00
1000.00
a2
y1
y2
y3
y6++
y7++-NH3
y7++
y8++-NH3
y8++
y4
b5-NH3
b10++-NH3
y10++-NH3
y10++-NH3, Par_m/z-H2O, Par_m/z-NH3
y5
y6-H2O
y6
y7
b7
b8
y8
b9

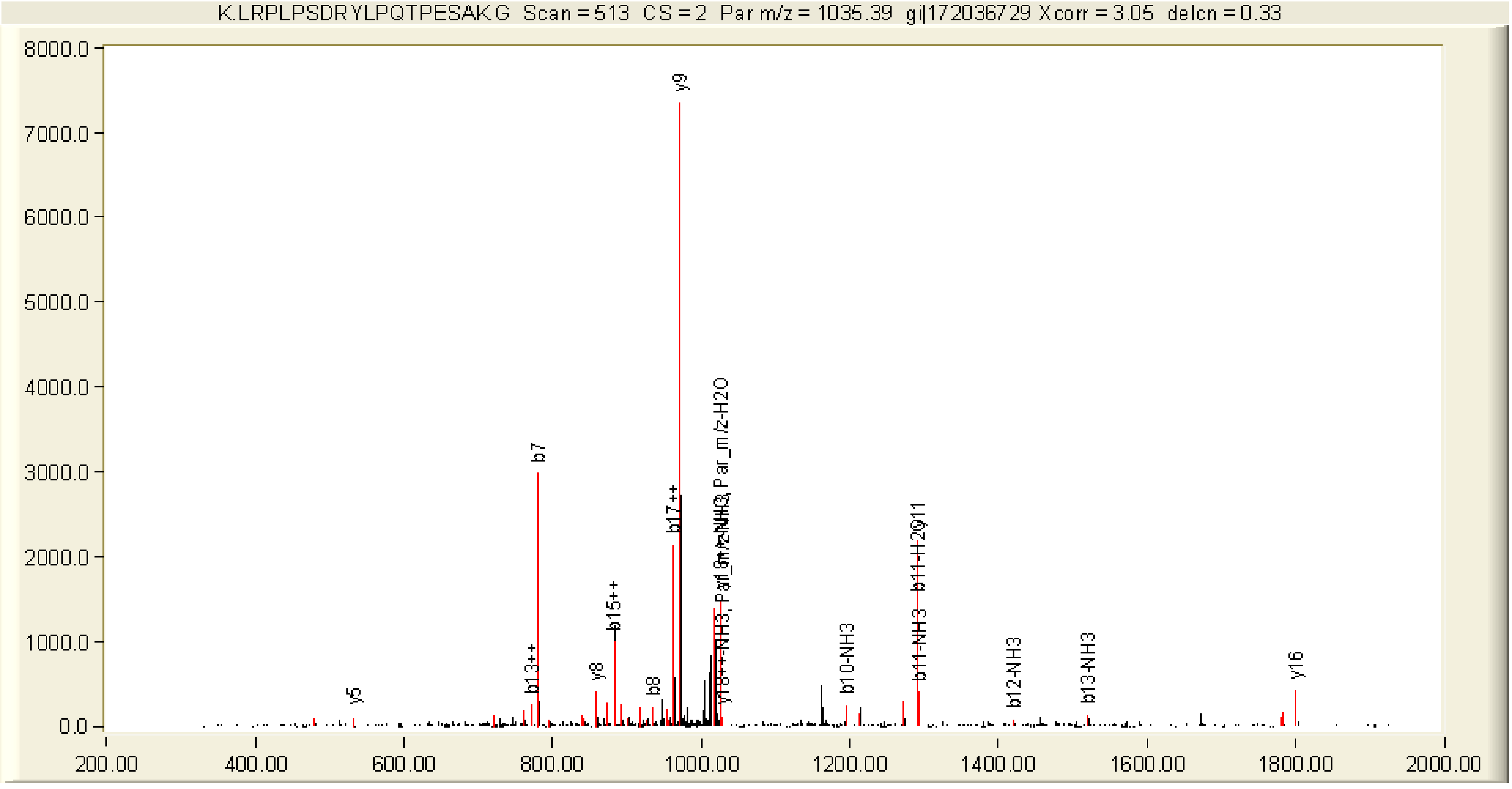

K.LRPLPSDRYLPQTPESAK.G Scan = 513 CS = 2 Par m/z = 1035.39 gi|172036729 Xcorr = 3.05 delcn = 0.33
8000.0
7000.0
6000.0
5000.0
4000.0
3000.0
2000.0
1000.0
0.0
200.00
400.00
600.00
800.00
1000.00
1200.00
1400.00
1600.00
1800.00
2000.00
y5
b13++
b7
y8
b15++
b8
b17++
y9
Par_m/z-H2O
b10-NH3
b11-NH3
b12-NH3
b13-NH3
y16

R.HGVQEILLELPAGTFDPKKEDSFSAAKR.E Scan = 515 CS = 3 Par m/z = 1029.96 gi|172034979 Xcorr = 4.04 delcn = 0.44

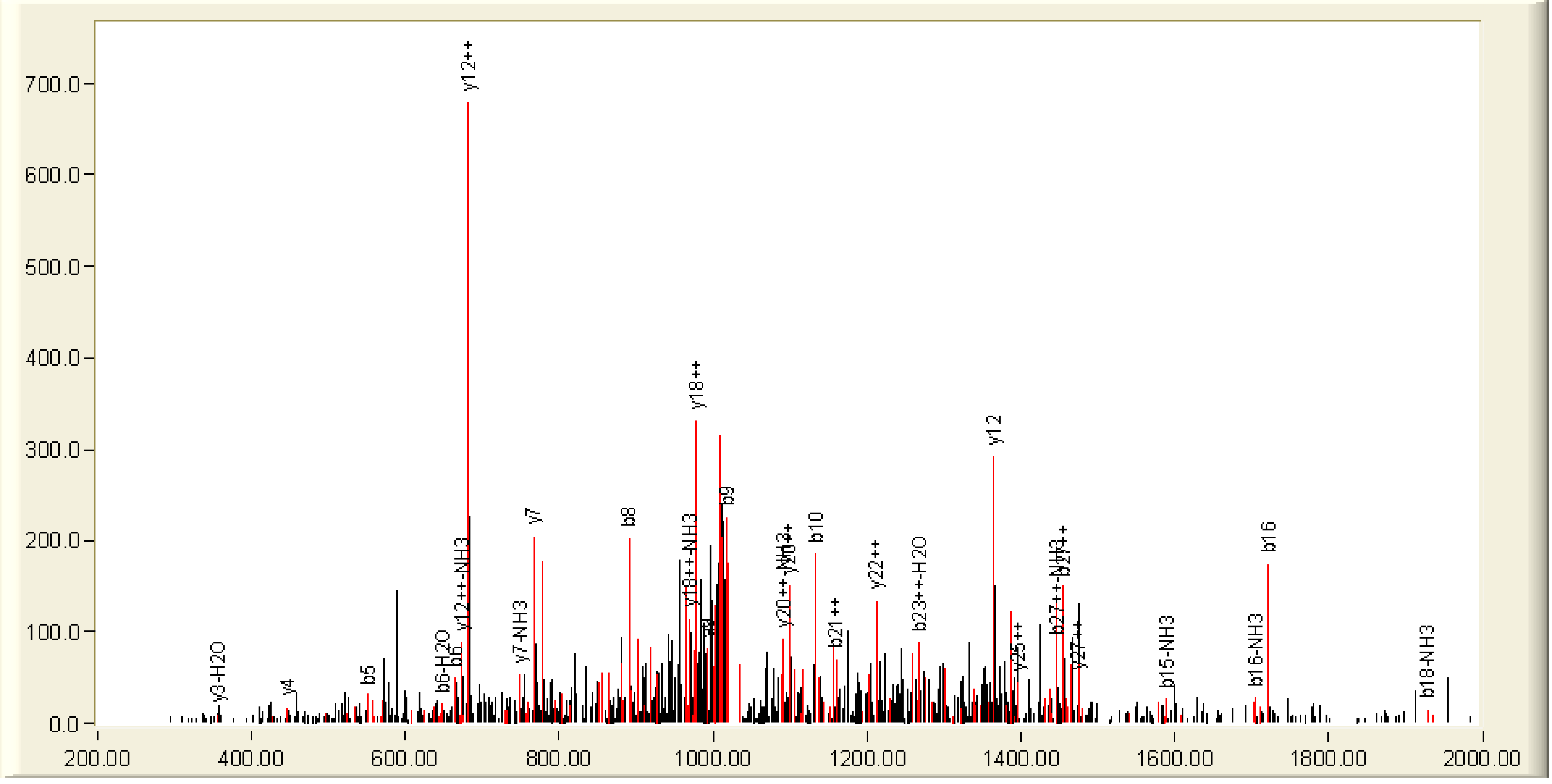

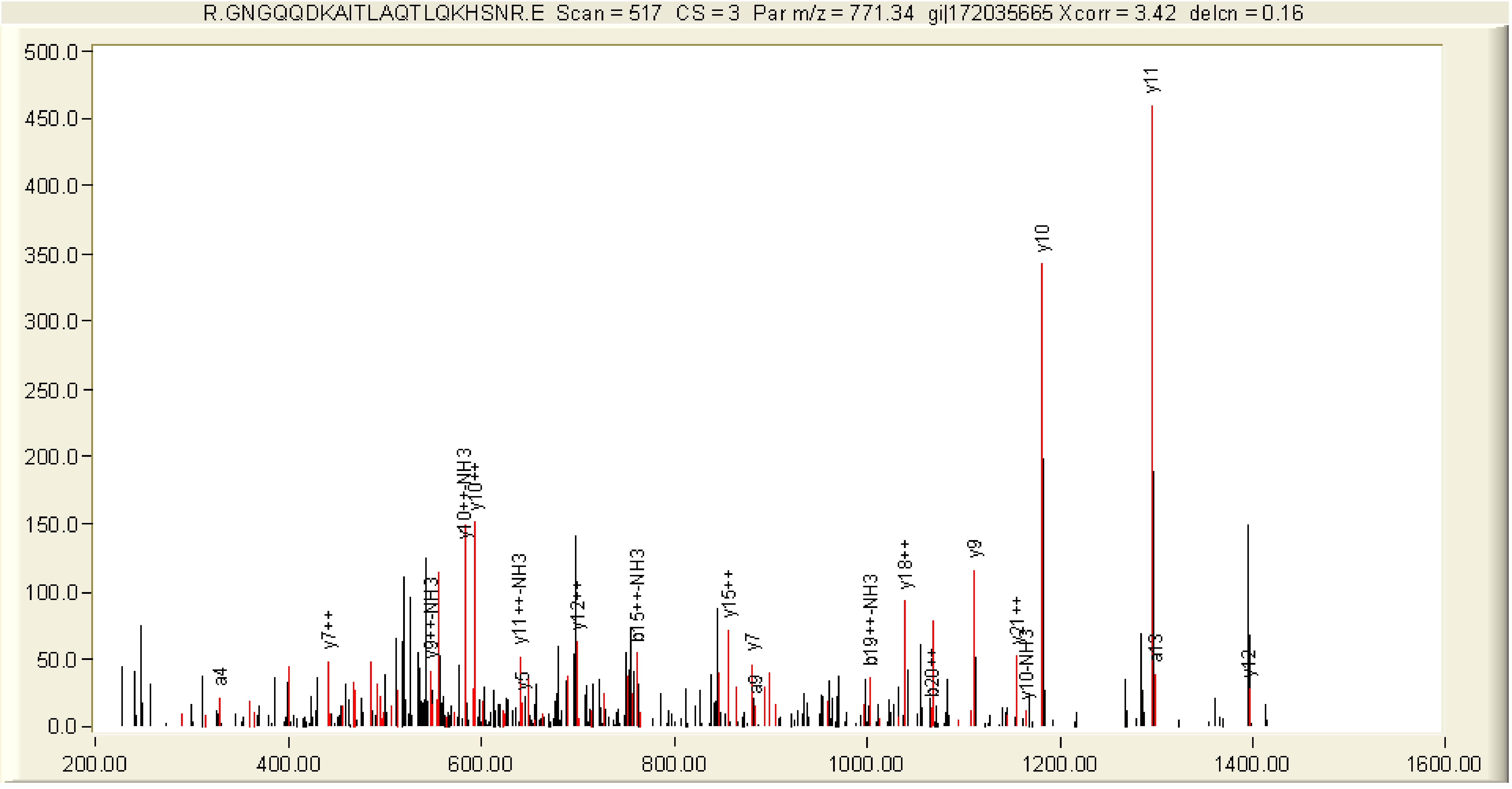

R.GNGQQDKAITLAQTLQKHSNR.E Scan = 517 CS = 3 Par m/z = 771.34 gi|172035665 Xcorr = 3.42 delcn = 0.16
500.0
450.0
400.0
350.0
300.0
250.0
200.0
150.0
100.0
50.0
0.0
200.00
400.00
600.00
800.00
1000.00
1200.00
1400.00
1600.00
y11
y10
a4
y7++
y9++-NH3
y10++-NH3
y10++
y11++-NH3
y5
y12++
b15++-NH3
y15++
y7
a9
b19++-NH3
y18++
b20++
y9
y21++
y10-NH3
a13
y12

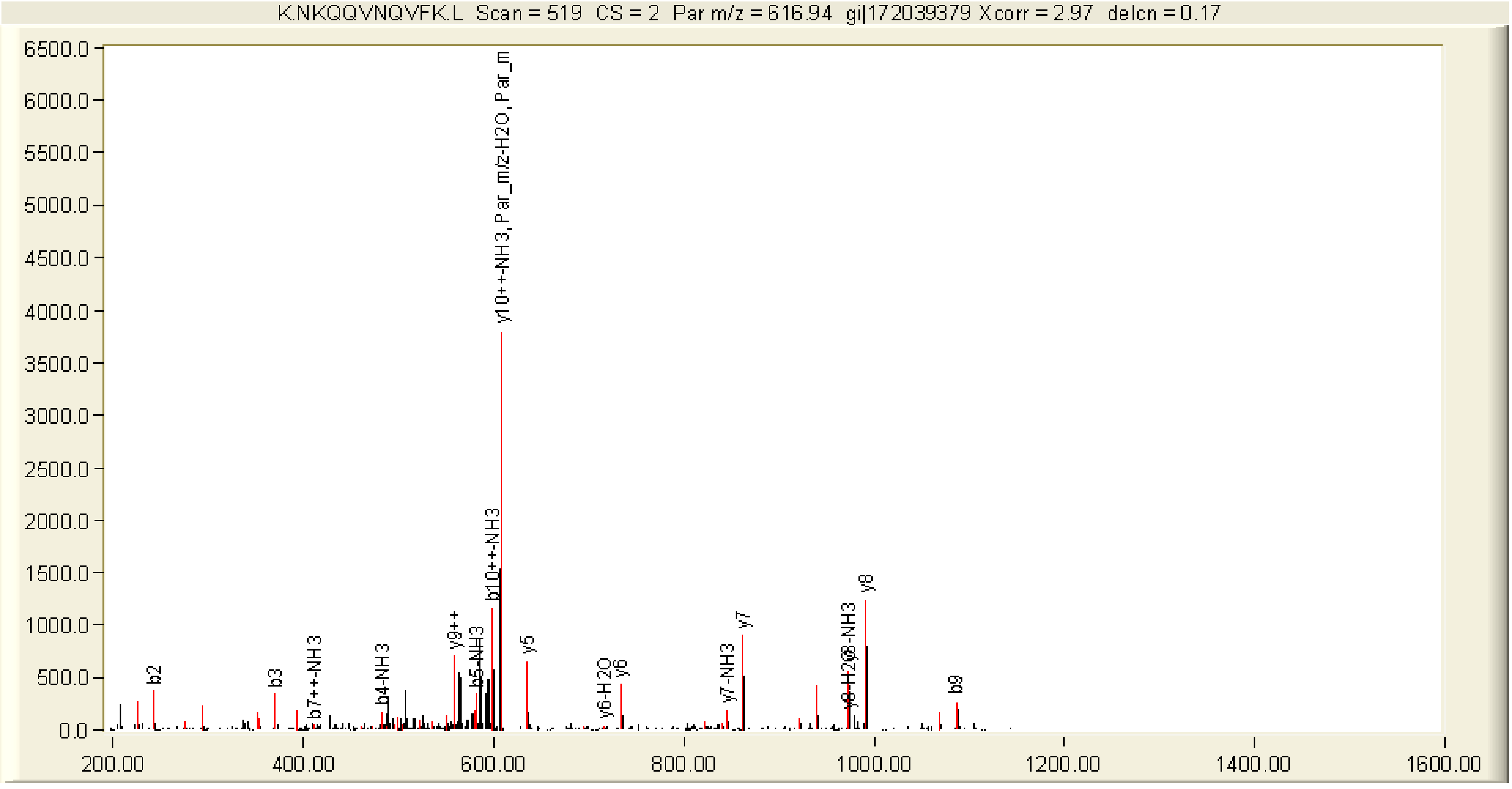

K.NKQQVNQVFK.L Scan = 519 CS = 2 Par m/z = 616.94 gi|172039379 Xcorr = 2.97 delcn = 0.17
6500.0
6000.0
5500.0
5000.0
4500.0
4000.0
3500.0
3000.0
2500.0
2000.0
1500.0
1000.0
500.0
0.0
200.00
400.00
600.00
800.00
1000.00
1200.00
1400.00
1600.00
y10++-NH3, Par_m/z-H2O, Par_m
b10++-NH3
y9++
b5-NH3
b2
b3
b7++-NH3
b4-NH3
y5
y6-H2O
y6
y7-NH3
y7
y8
b9

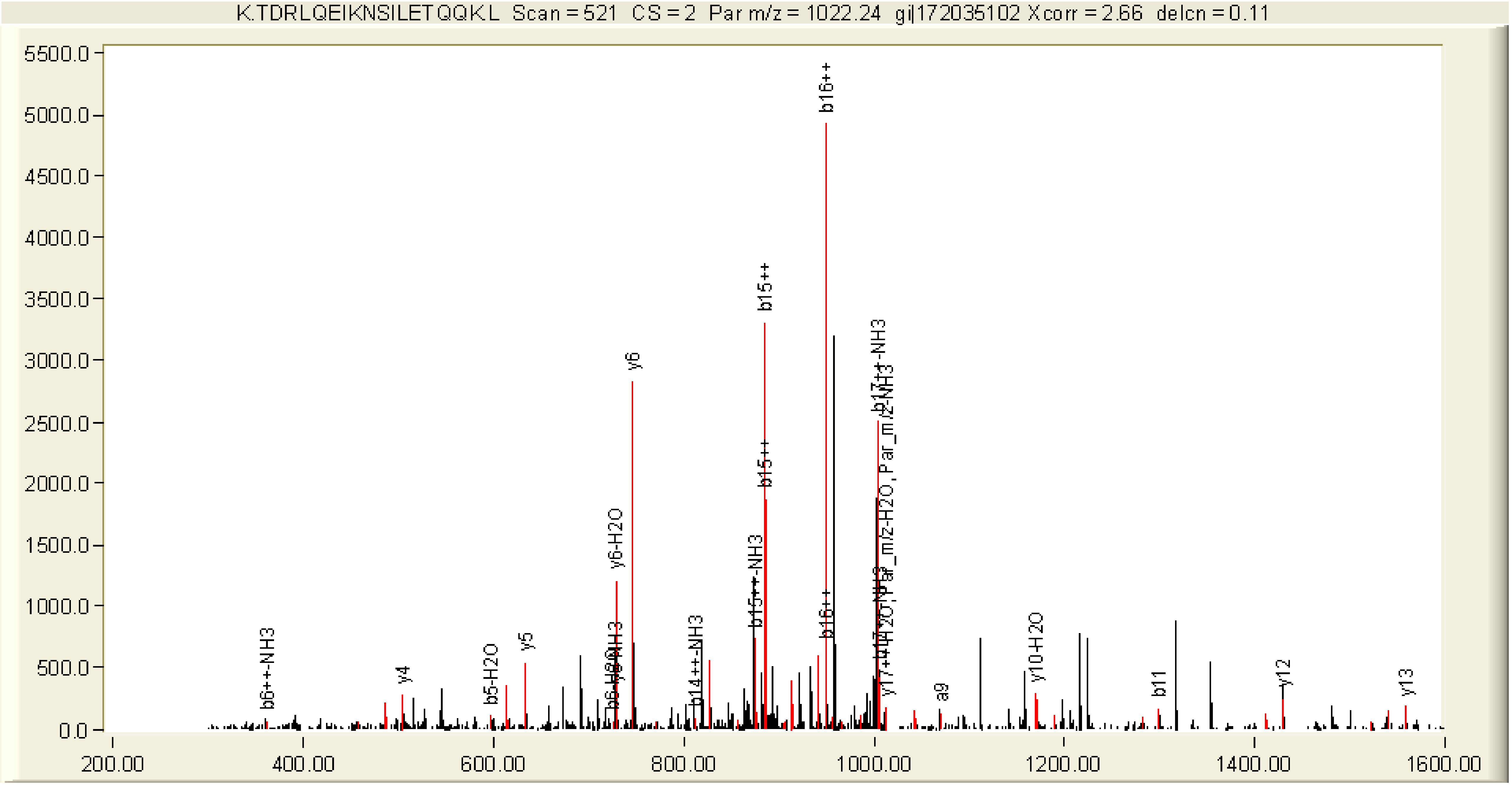
K.TDRLQEIKNSILETQQKL Scan = 521 CS = 2 Par m/z = 1022.24 gi|172035102 Xcorr = 2.66 delcn = 0.11
b16++
b15++
y6
y6-H2O
b15++
b15++-NH3
b16++
y5
b5-H2O
y4
b6++-NH3
b14++-NH3
a9
y10-H2O
b11
y12
y13
0.0
500.0
1000.0
1500.0
2000.0
2500.0
3000.0
3500.0
4000.0
4500.0
5000.0
5500.0
200.00
400.00
600.00
800.00
1000.00
1200.00
1400.00
1600.00

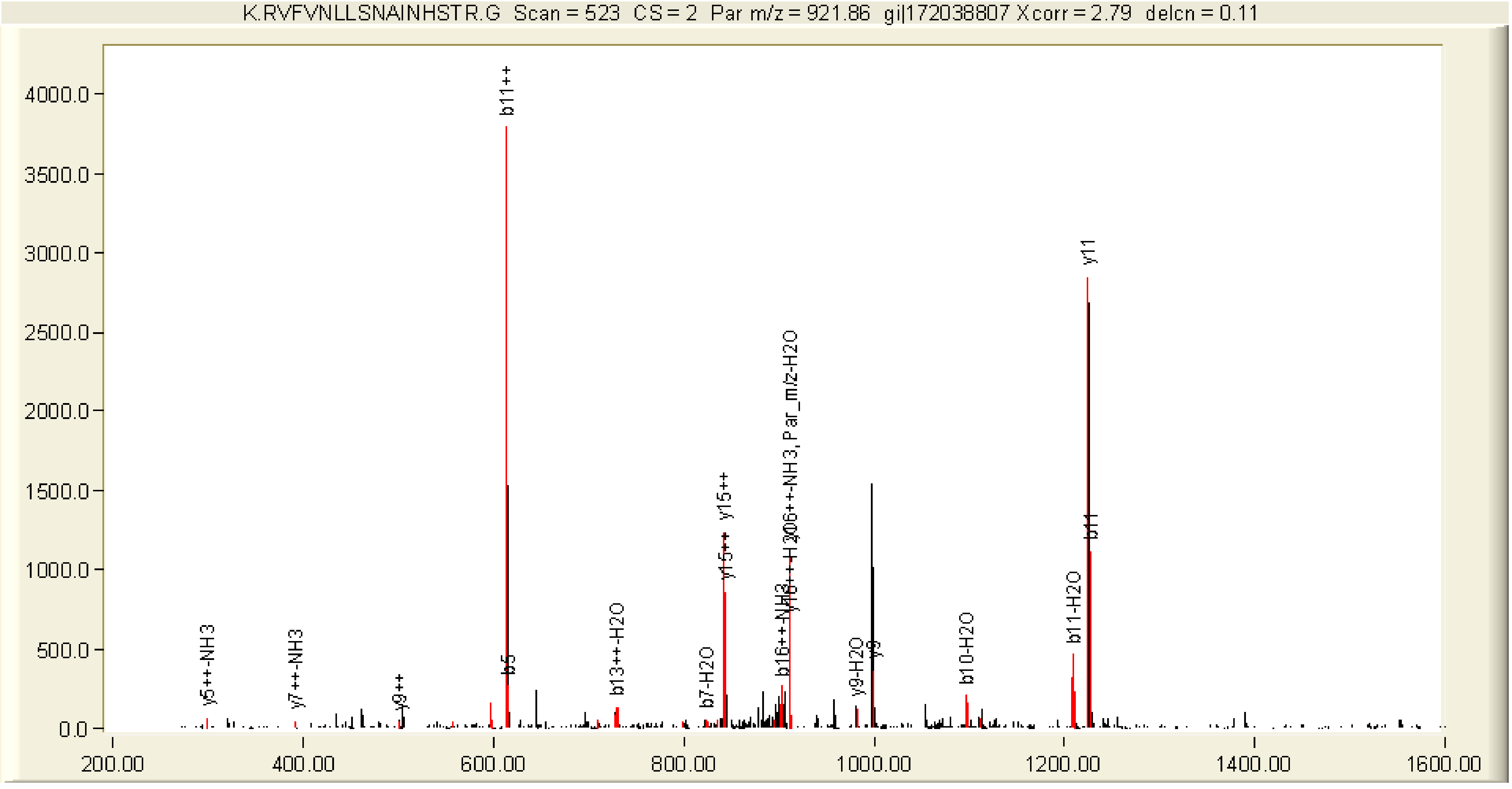

K.RVFVNLLSNAINHSTR.G Scan = 523 CS = 2 Par m/z = 921.86 gi|172038807 Xcorr = 2.79 delcn = 0.11
4000.0
3500.0
3000.0
2500.0
2000.0
1500.0
1000.0
500.0
0.0
200.00
400.00
600.00
800.00
1000.00
1200.00
1400.00
1600.00
b11++
y11
y15++
y16++-NH3, Par_m/z-H2O
b11
y15++
b16++-NH3
y5++-NH3
y7++-NH3
y9++
b5
b13++-H2O
b7-H2O
y9-H2O
y9
b10-H2O
b11-H2O

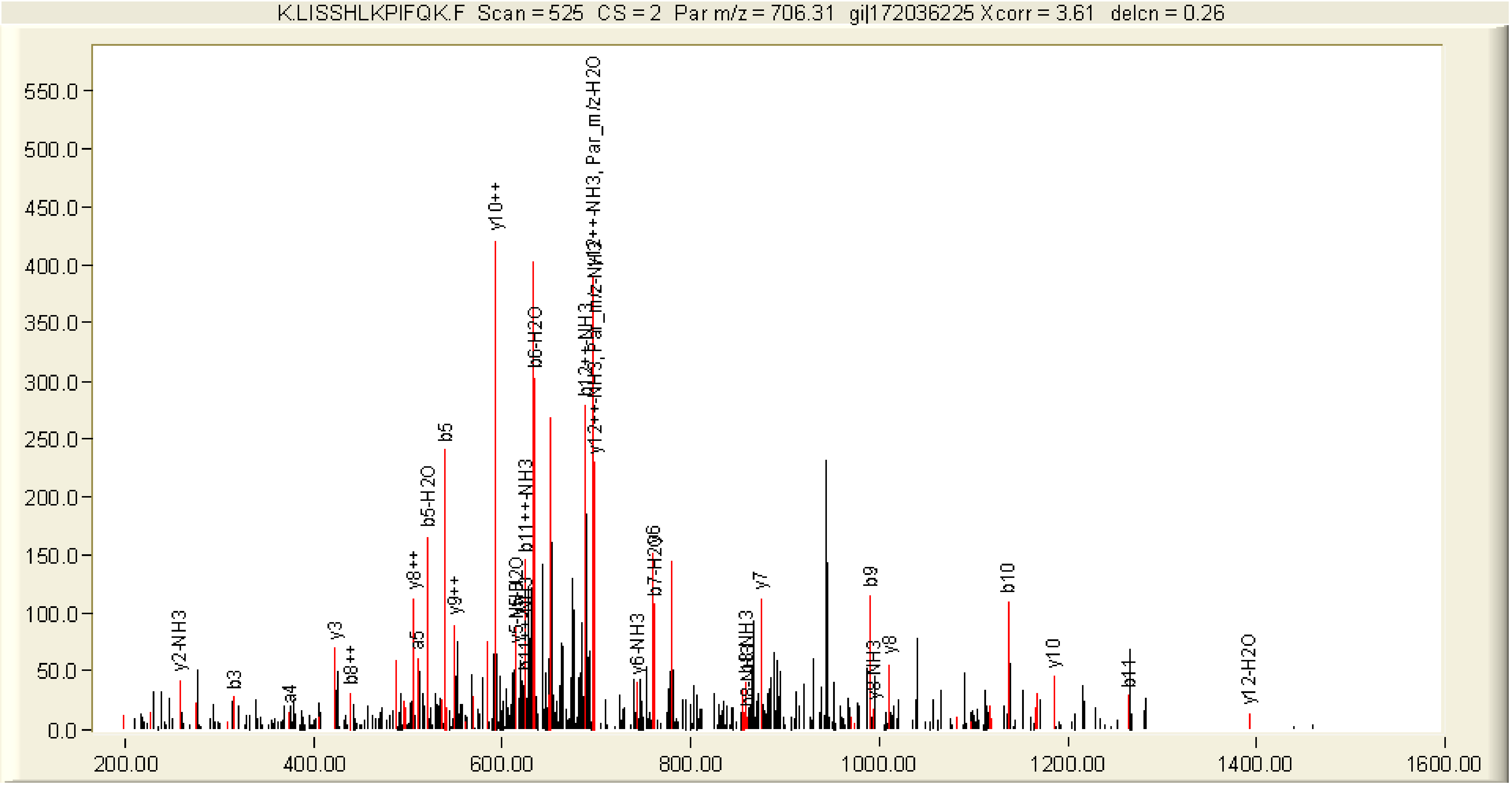
K.LISSHLKPIFQK.F Scan = 525 CS = 2 Par m/z = 706.31 gi|172036225 Xcorr = 3.61 delcn = 0.26
y10++
b6-H2O
b5
b5-H2O
b11++-NH3
y8++
y9++
a5
y3
b8++
b3
a4
y2-NH3
y6-NH3
y7
b9
y8
b10
y10
b11
y12-H2O
0.0
50.0
100.0
150.0
200.0
250.0
300.0
350.0
400.0
450.0
500.0
550.0
200.00
400.00
600.00
800.00
1000.00
1200.00
1400.00
1600.00

R.KLAIKEGK.R Scan = 527 CS = 2 Par m/z = 444.2 gi|172035797 Xcorr = 2.54 delcn = 0.11

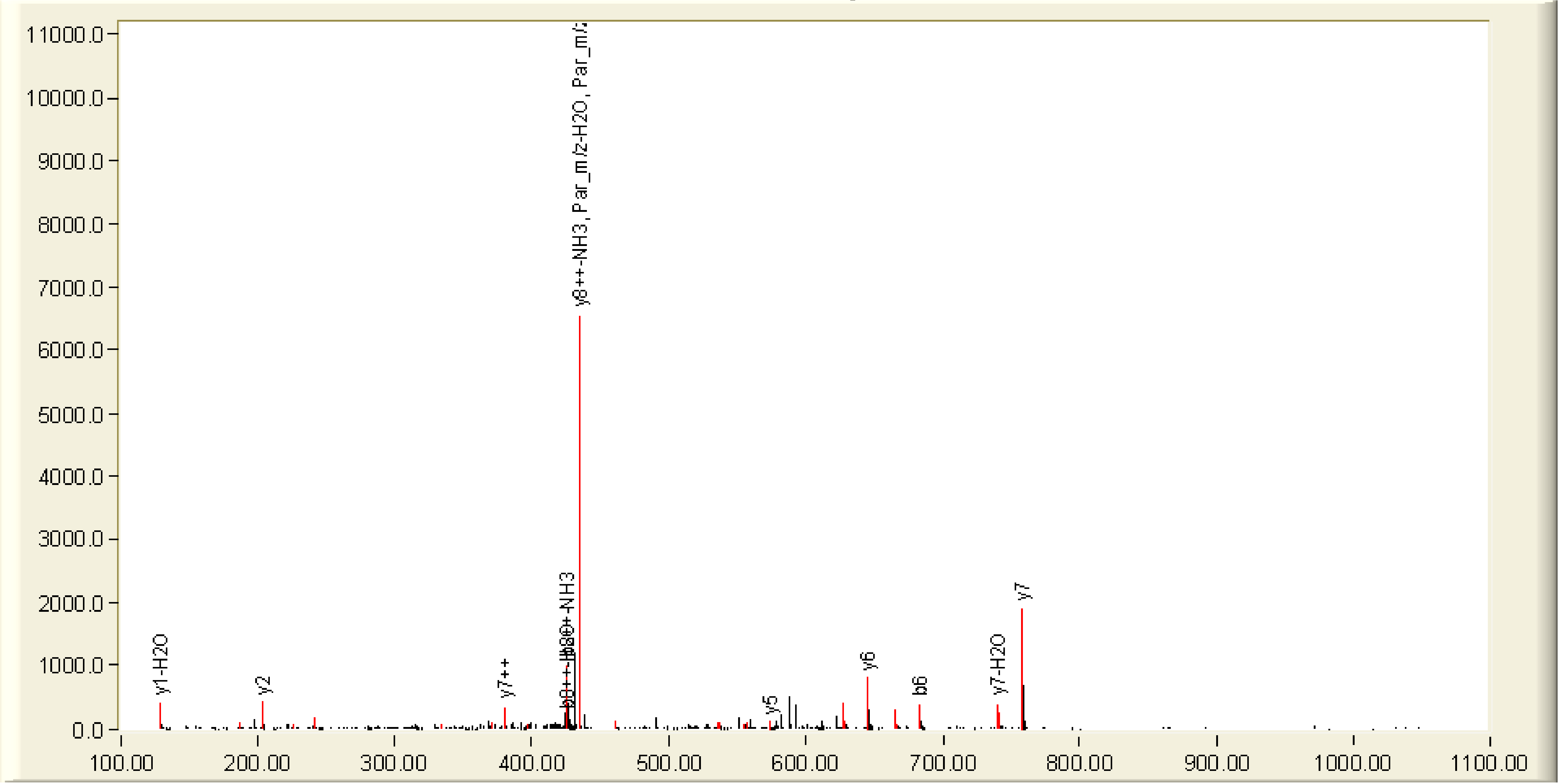

K.TQQINVTVEADGDIFVDR.Q Scan = 529 CS = 2 Par m/z = 1010.73 gi|172037966 Xcorr = 3.23 delcn = 0.16

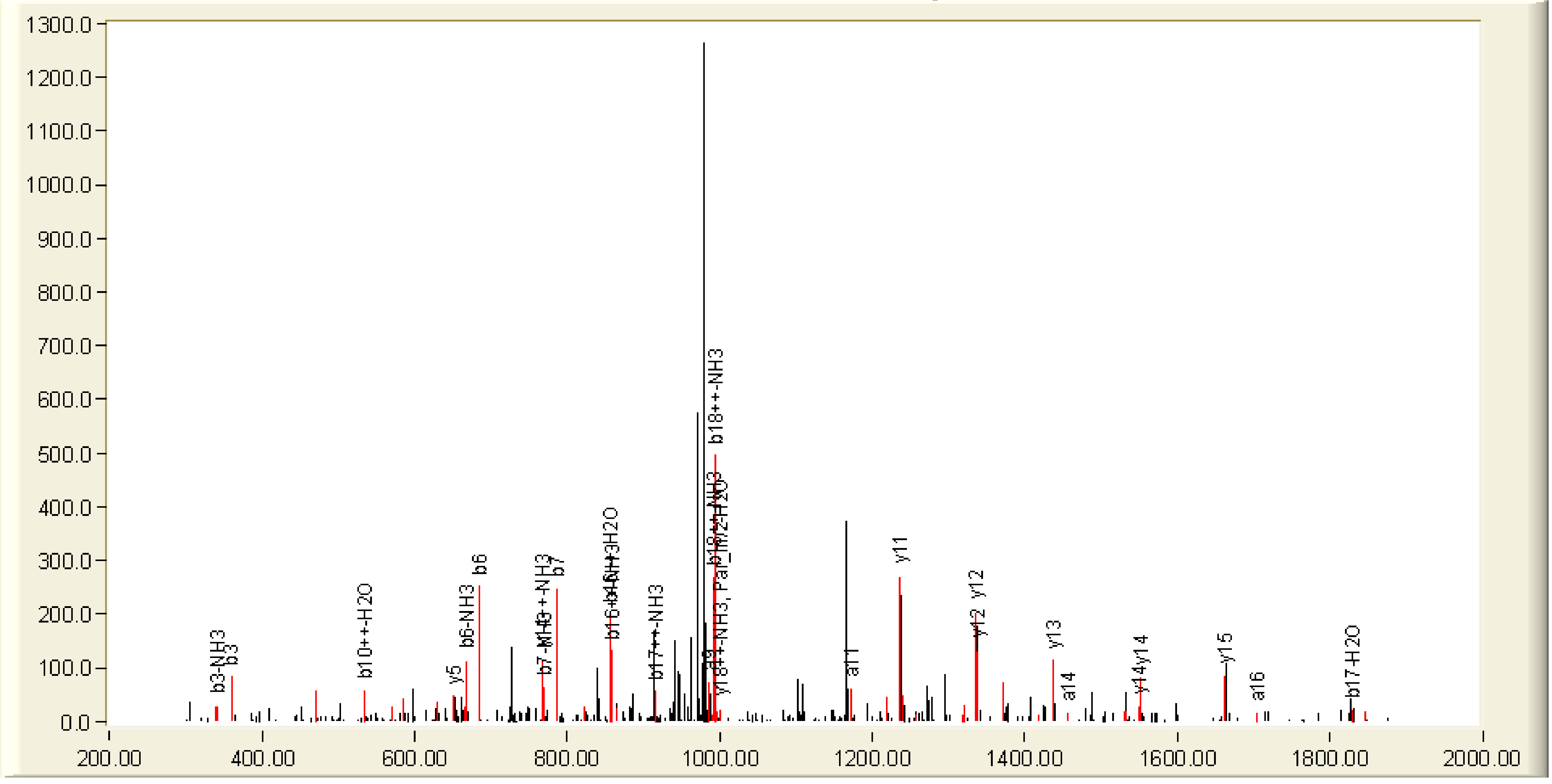

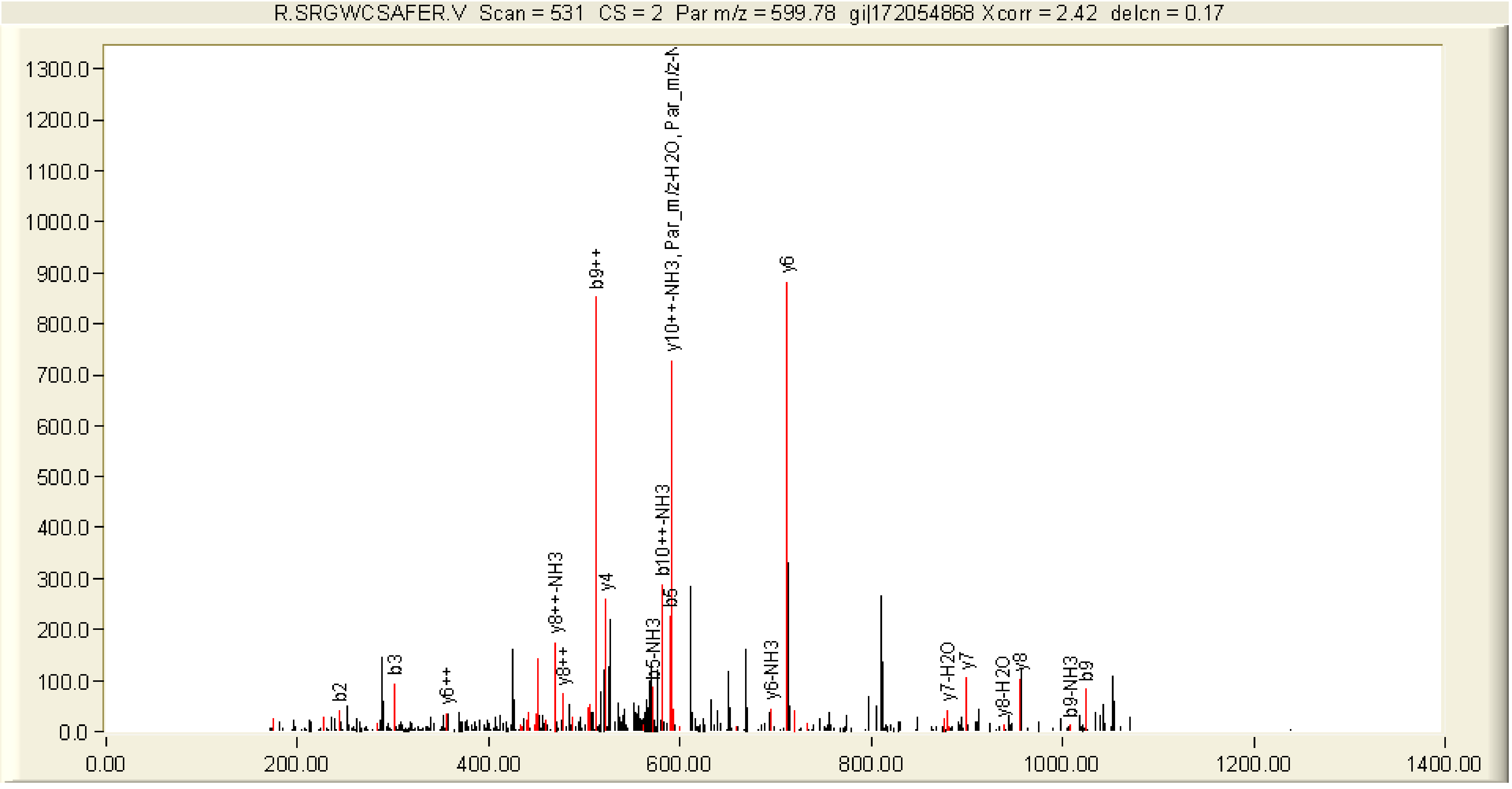
R.SRGWCSAFER.V Scan = 531 CS = 2 Par m/z = 599.78 gi|172054868 Xcorr = 2.42 delcn = 0.17
b9++
y10++-NH3, Par_m/z-H2O, Par_m/z-N
y6
b10++-NH3
y4
b5
y8++-NH3
y8++
b5-NH3
b2
b3
y6++
y6-NH3
y7-H2O
y7
y8-H2O
y8
b9-NH3
b9
0.00
200.00
400.00
600.00
800.00
1000.00
1200.00
1400.00
0.0
100.0
200.0
300.0
400.0
500.0
600.0
700.0
800.0
900.0
1000.0
1100.0
1200.0
1300.0

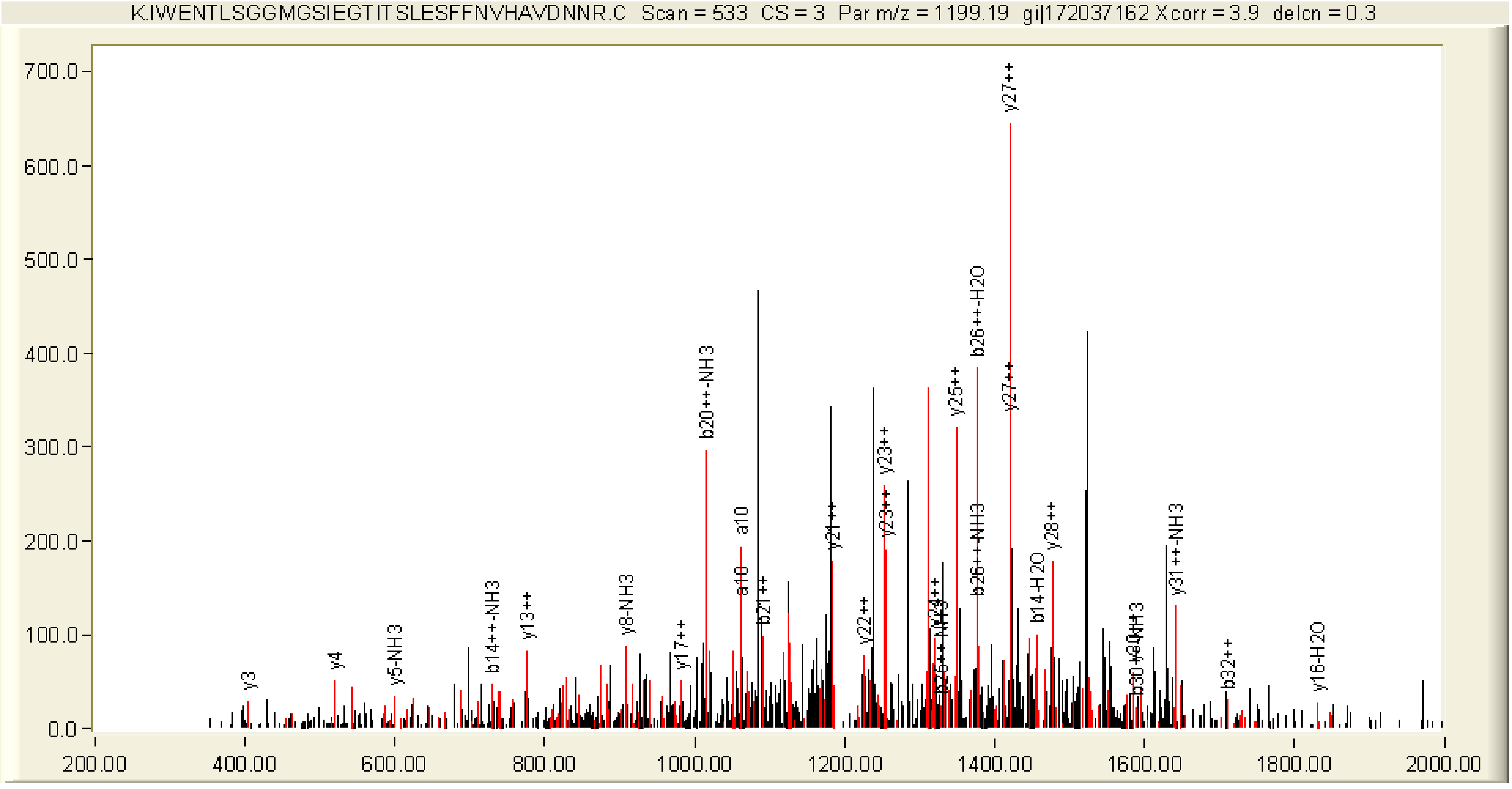

K.IWENTLSGGMGSIEGTITSLESFFNVHAVDNNR.C Scan = 533 CS = 3 Par m/z = 1199.19 gi|172037162 Xcorr = 3.9 delcn = 0.3
700.0
600.0
500.0
400.0
300.0
200.0
100.0
0.0
200.00
400.00
600.00
800.00
1000.00
1200.00
1400.00
1600.00
1800.00
2000.00
y3
y4
y5-NH3
b14++-NH3
y13++
y8-NH3
y17++
b20++-NH3
a10
b21++
y21++
y22++
y23++
y25++
b26++-H2O
b26++-NH3
y27++
b14-H2O
y28++
y31++-NH3
b32++
y16-H2O

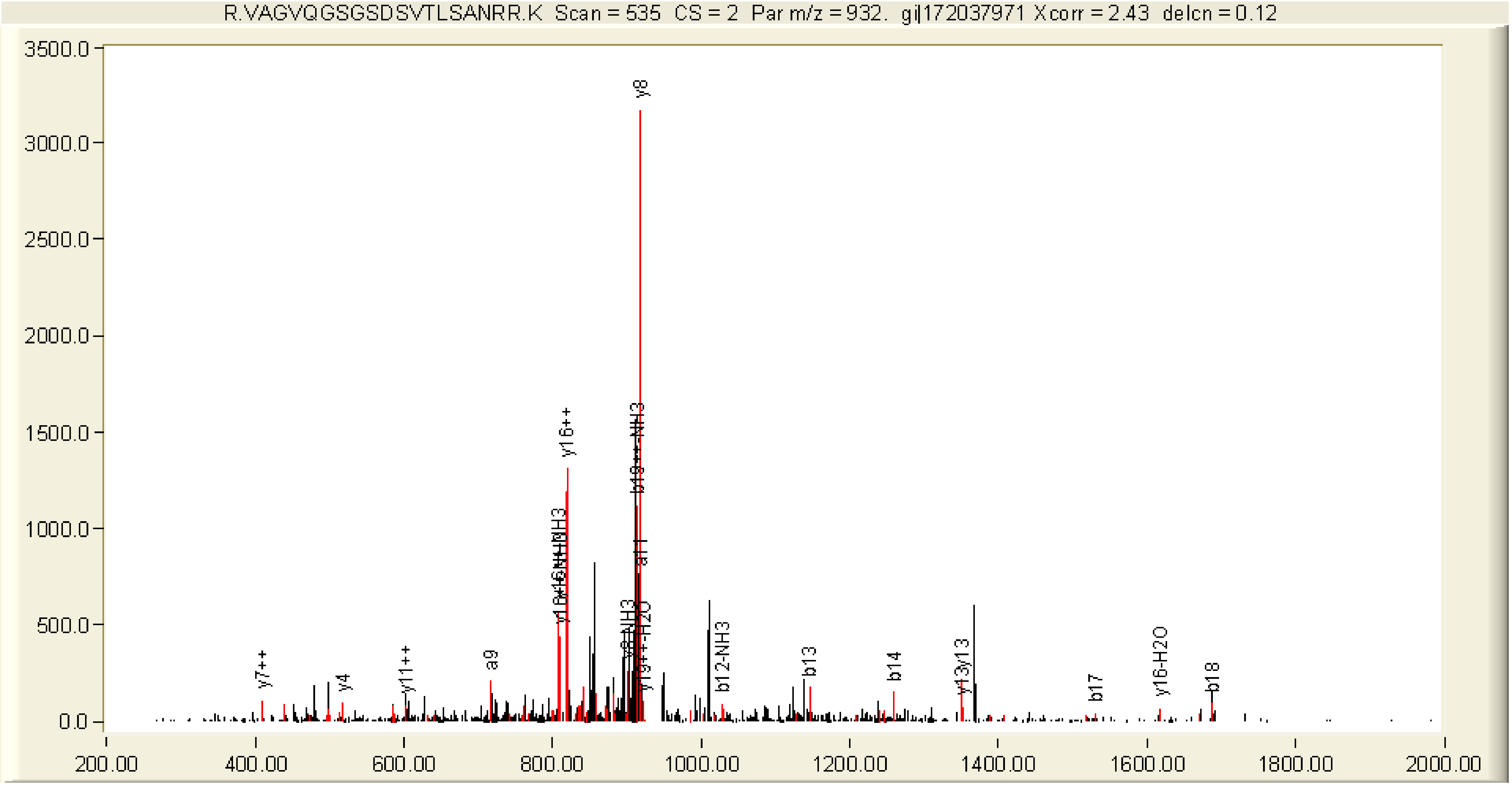
R.VAGVQGSGSDSVTLSANRR.K Scan = 535 CS = 2 Par m/z = 932. gi|172037971 Xcorr = 2.43 delcn = 0.12
3500.0
3000.0
2500.0
2000.0
1500.0
1000.0
500.0
0.0
200.00
400.00
600.00
800.00
1000.00
1200.00
1400.00
1600.00
1800.00
2000.00
y8
y16++
y7++
y4
y11++
a9
b12-NH3
b13
b14
b17
y16-H2O
b18

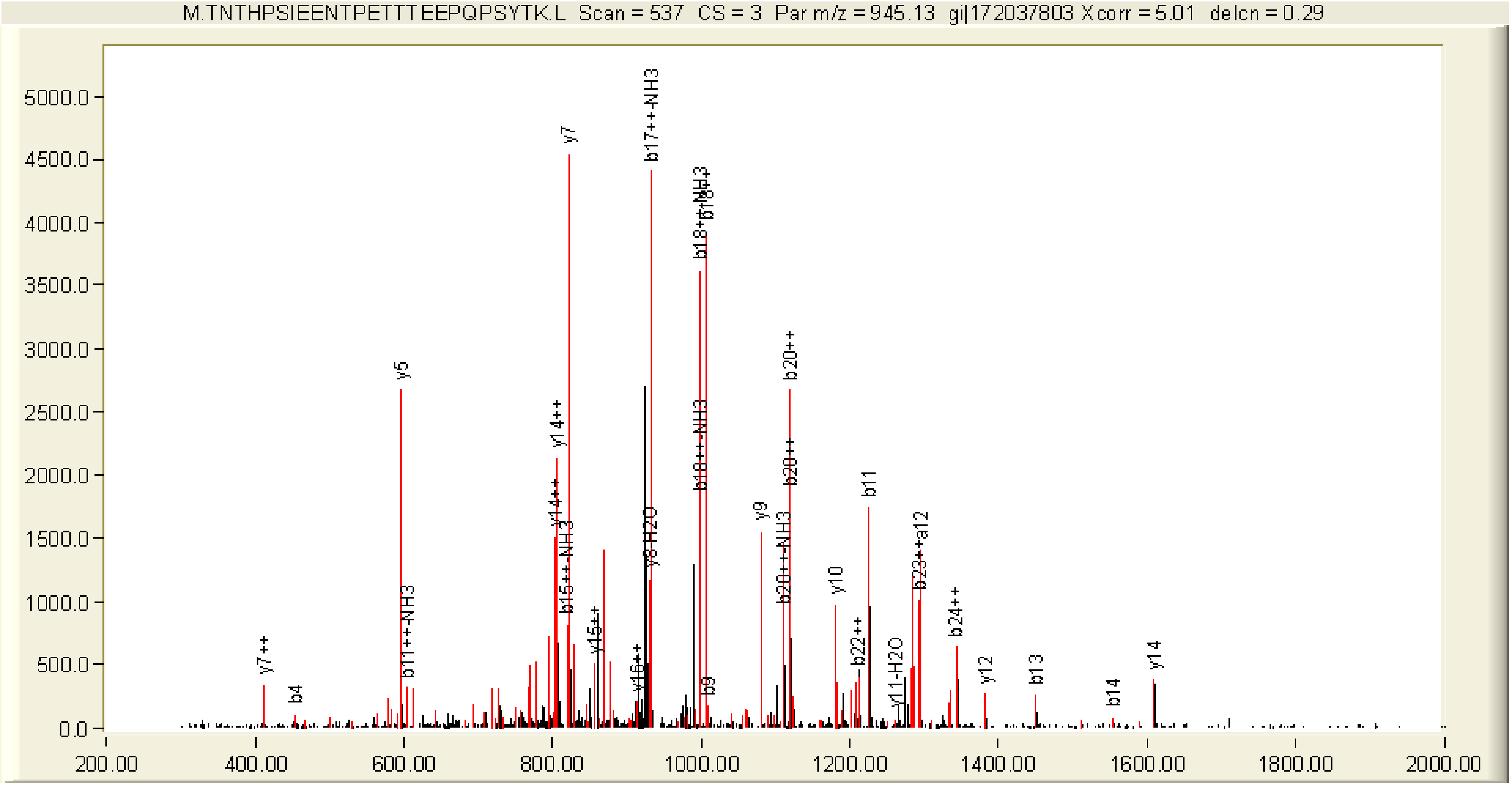
M.TNTHPSIEENTPETTTEEPQPSYTK.L Scan = 537 CS = 3 Par m/z = 945.13 gi|172037803 Xcorr = 5.01 delcn = 0.29
5000.0
4500.0
4000.0
3500.0
3000.0
2500.0
2000.0
1500.0
1000.0
500.0
0.0
200.00
400.00
600.00
800.00
1000.00
1200.00
1400.00
1600.00
1800.00
2000.00
y7++
b4
y5
b11++-NH3
y14++
b15++-NH3
y15++
y16++
y7
b17++-NH3
y8-H2O
b9
y9
b20++-NH3
b20++
y10
b22++
b11
y11-H2O
b23++a12
b24++
y12
b13
b14
y14

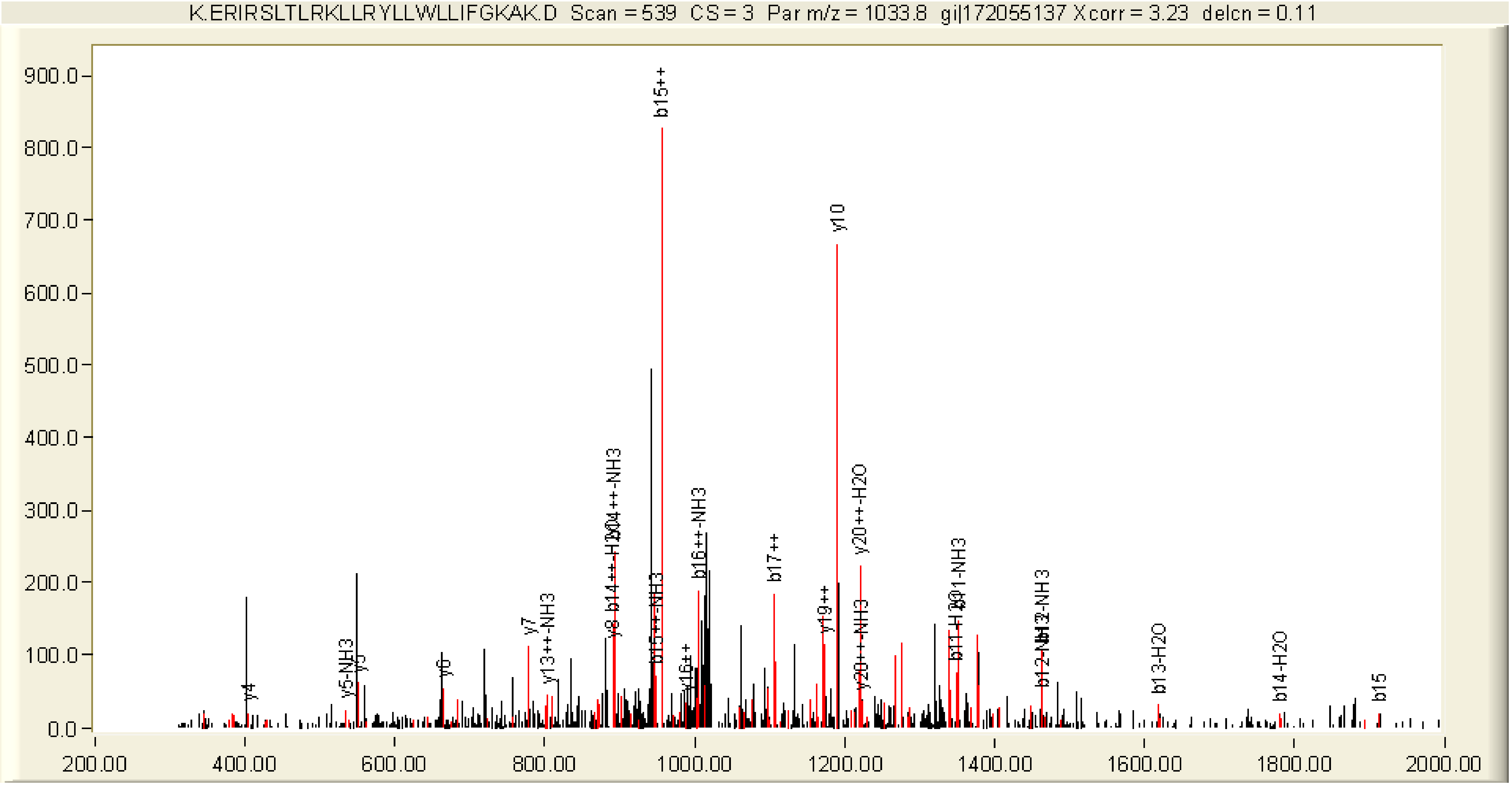

K.ERIRSLTLRKLLRYLLWLLIFGKAK.D Scan = 539 CS = 3 Par m/z = 1033.8 gi|172055137 Xcorr = 3.23 delcn = 0.11
900.0
800.0
700.0
600.0
500.0
400.0
300.0
200.0
100.0
0.0
200.00
400.00
600.00
800.00
1000.00
1200.00
1400.00
1600.00
1800.00
2000.00
b15++
y10
y20++-H2O
b16++-NH3
b17++
y19++
y20++-NH3
b11-NH3
b12-NH3
b13-H2O
b14-H2O
b15
y7
y13++-NH3
y6
y5-NH3
y5
y4
y16++

K.KRLKNLPVNAVSLR.E Scan = 541 CS = 2 Par m/z = 805.72 gi|172055120 Xcorr = 2.55 delcn = 0.13

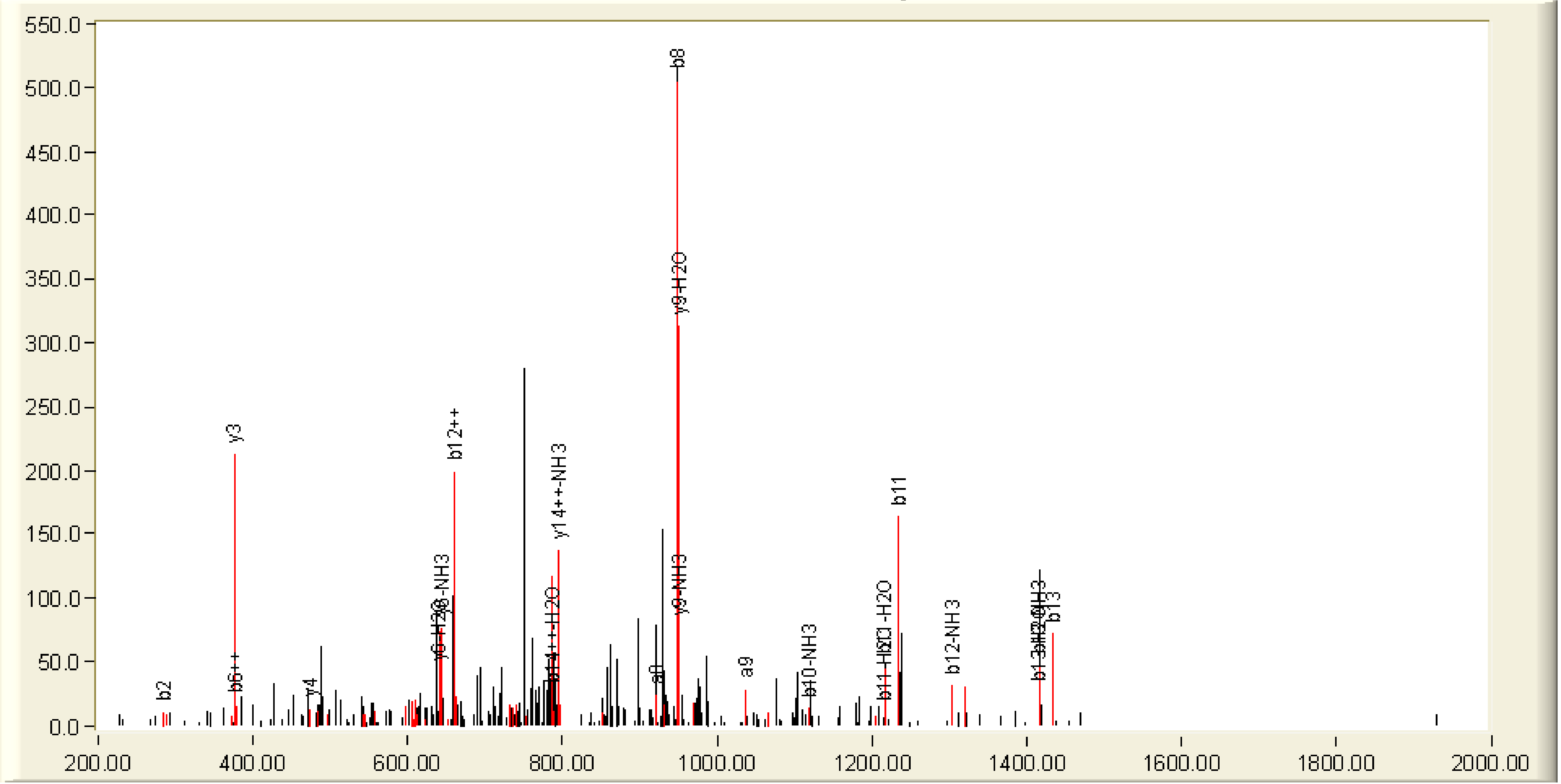

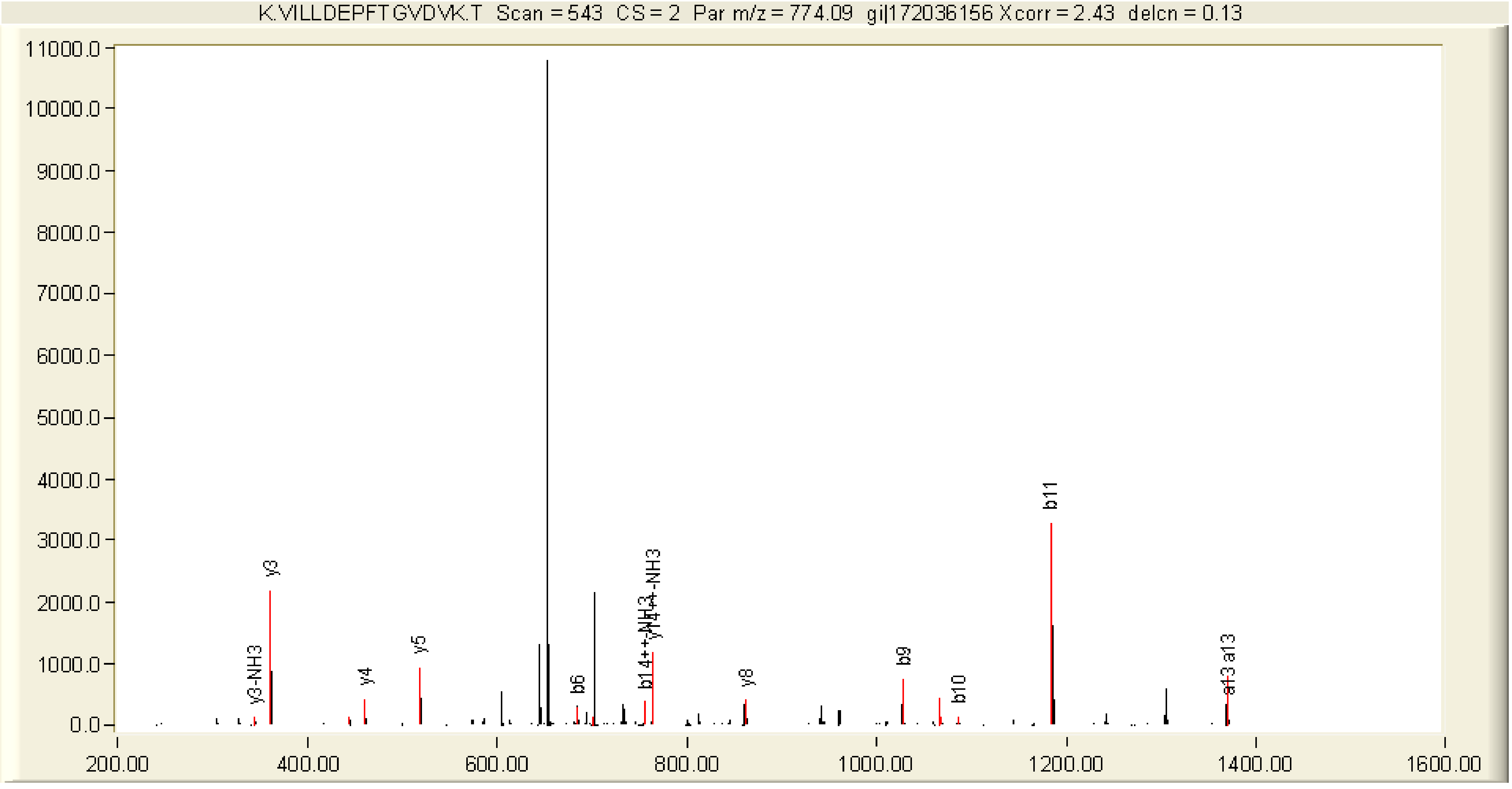
K.VILLDEPFTGVDVK.T Scan = 543 CS = 2 Par m/z = 774.09 gi|172036156 Xcorr = 2.43 delcn = 0.13
11000.0
10000.0
9000.0
8000.0
7000.0
6000.0
5000.0
4000.0
3000.0
2000.0
1000.0
0.0
200.00
400.00
600.00
800.00
1000.00
1200.00
1400.00
1600.00
y3-NH3
y3
y4
y5
b6
b14++-NH3
y14++-NH3
y8
b9
b10
b11
a13 a13

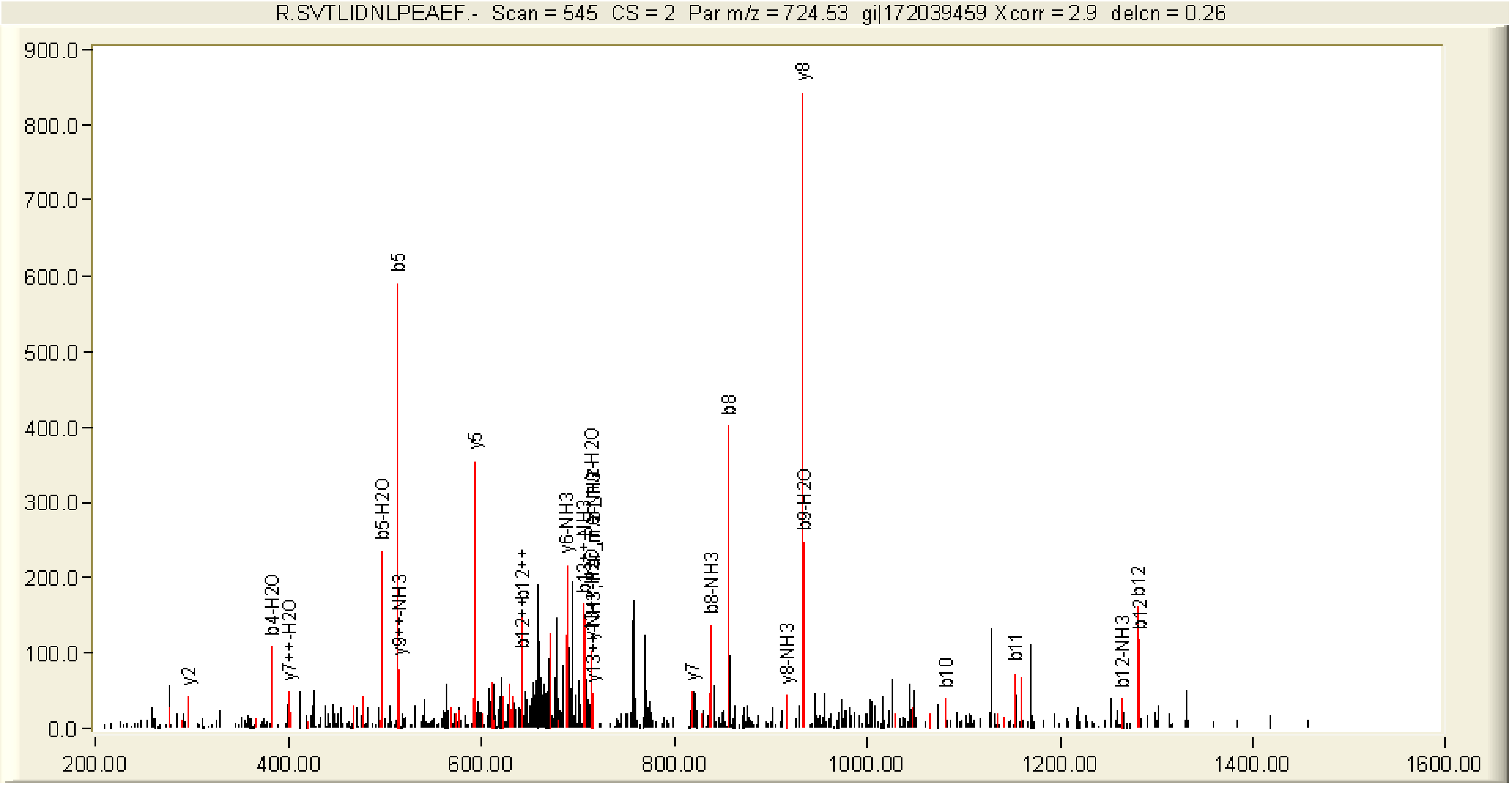
R.SVTLIDNLPEAEF.- Scan = 545 CS = 2 Par m/z = 724.53 gi|172039459 Xcorr = 2.9 delcn = 0.26
900.0
800.0
700.0
600.0
500.0
400.0
300.0
200.0
100.0
0.0
200.00
400.00
600.00
800.00
1000.00
1200.00
1400.00
1600.00
y2
b4-H2O
y7++-H2O
b5-H2O
b5
y9++-NH3
y5
b12++b12++
y6-NH3
b8-NH3
b8
y7
y8-NH3
y8
b9-H2O
b10
b11
b12-NH3
b12b12

R.SFPQDVELLVANAS.- Scan = 547 CS = 2 Par m/z = 746.49 gi|172037094 Xcorr = 3.63 delcn = 0.13

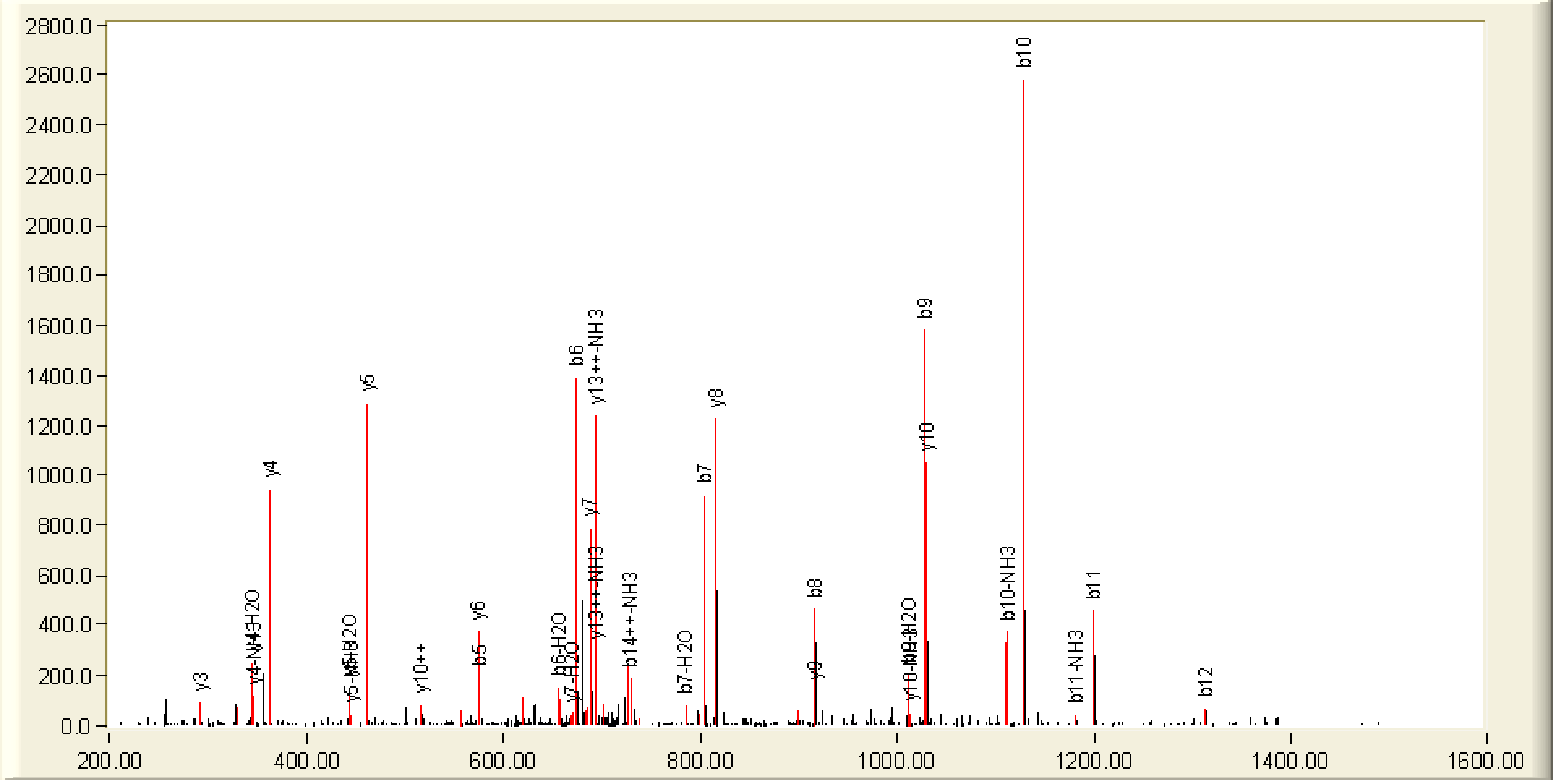

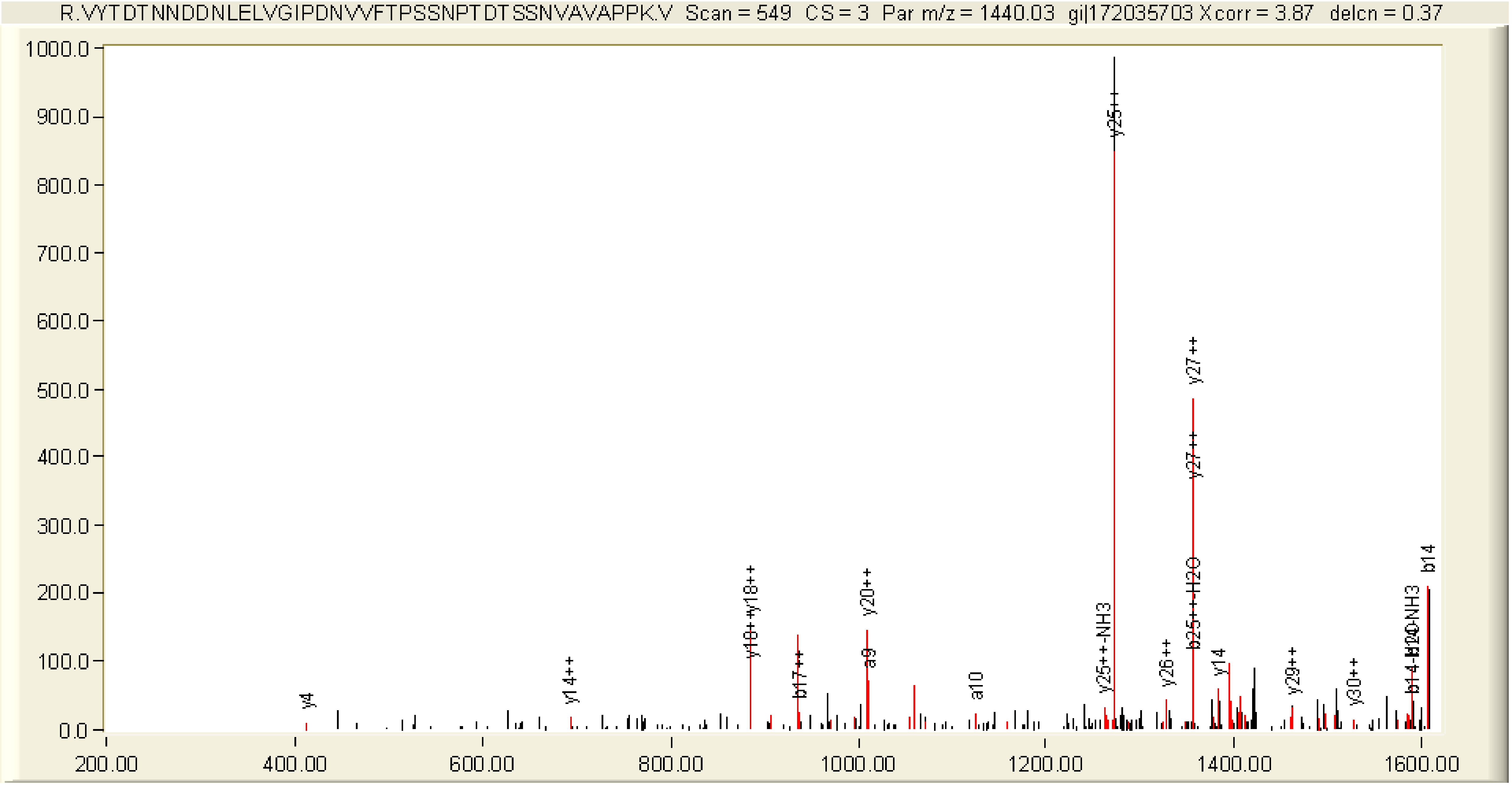
R.VYTDTNNDDNLELVGIPDNVVFTPSSNPTDTSSNVAVAPPK.V Scan = 549 CS = 3 Par m/z = 1440.03 gi|172035703 Xcorr = 3.87 delcn = 0.37
1000.0
900.0
800.0
700.0
600.0
500.0
400.0
300.0
200.0
100.0
0.0
200.00
400.00
600.00
800.00
1000.00
1200.00
1400.00
1600.00
y4
y14++
b17++
y20++
a9
a10
y25++-NH3
y25++
y26++
y27++
b25++-H2O
y14
y29++
y30++
b14

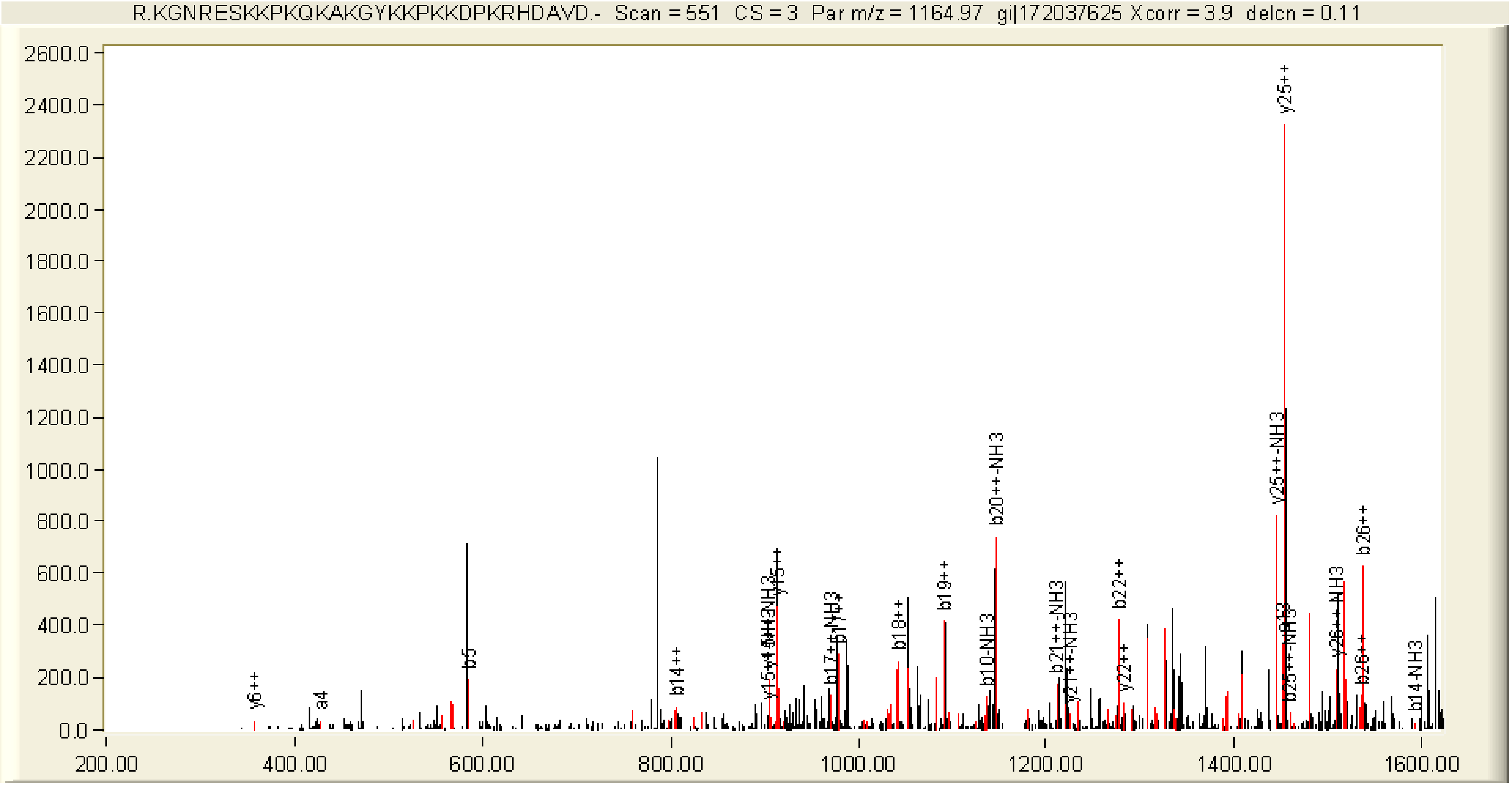
R.KGNRESKKPKQKAKGYKKPKKDPKRHDAVD.- Scan = 551 CS = 3 Par m/z = 1164.97 gi|172037625 Xcorr = 3.9 delcn = 0.11
0.0
200.0
400.0
600.0
800.0
1000.0
1200.0
1400.0
1600.0
1800.0
2000.0
2200.0
2400.0
2600.0
200.00
400.00
600.00
800.00
1000.00
1200.00
1400.00
1600.00
y6++
a4
b5
b14++
b18++
b19++
b10-NH3
b20++-NH3
b21++-NH3
b22++
y22++
y25++
y25++-NH3
b26++
b14-NH3

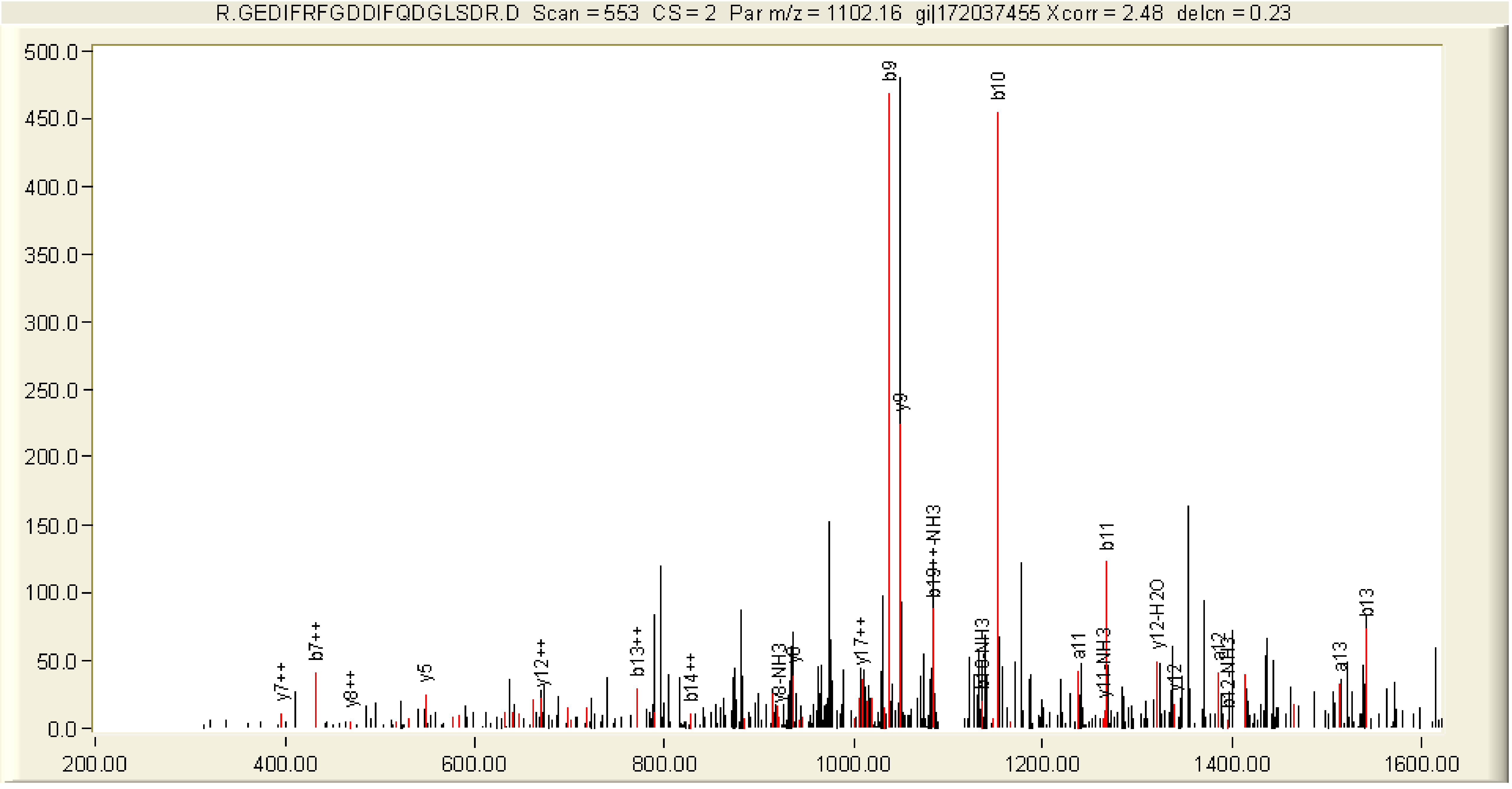
R.GEDIFRFGDDIFQDGLSDR.D Scan = 553 CS = 2 Par m/z = 1102.16 gi|172037455 Xcorr = 2.48 delcn = 0.23
500.0
450.0
400.0
350.0
300.0
250.0
200.0
150.0
100.0
50.0
0.0
200.00
400.00
600.00
800.00
1000.00
1200.00
1400.00
1600.00
y7++
b7++
y8++
y5
y12++
b13++
b14++
y8-NH3
b9
y9
b10
y17++
a11
b11
y11-NH3
y12-H2O
y12
a13
b13

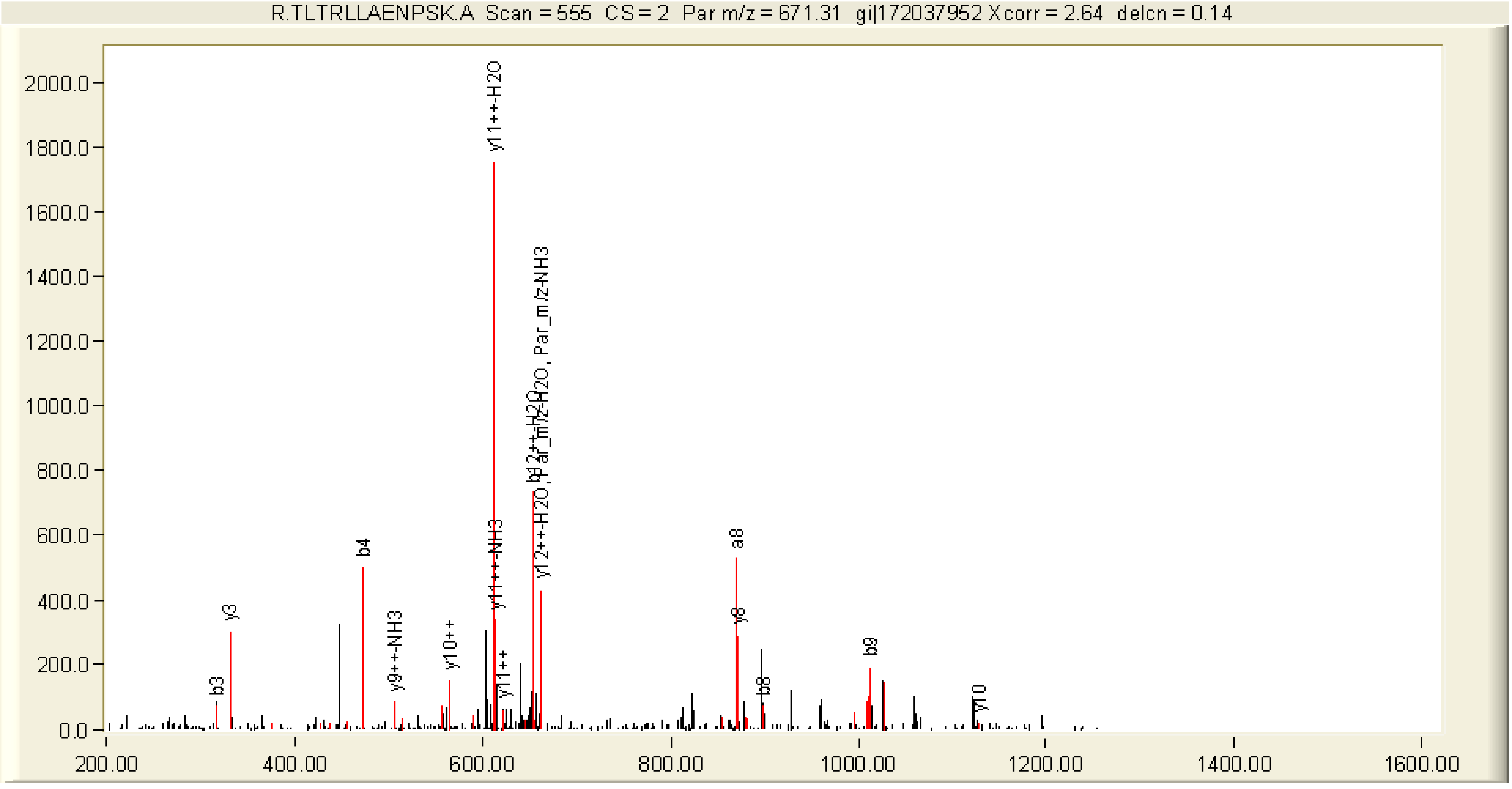

R.TLTRLLAENPSK.A Scan = 555 CS = 2 Par m/z = 671.31 gi|172037952 Xcorr = 2.64 delcn = 0.14
y11++-H2O
y12++-H2O, Par_m/z-H2O, Par_m/z-NH3
b12++-H2O
y11++-NH3
b4
a8
y3
y9++-NH3
y10++
y11++
y8
b9
b8
b3
y10
2000.0
1800.0
1600.0
1400.0
1200.0
1000.0
800.0
600.0
400.0
200.0
0.0
200.00
400.00
600.00
800.00
1000.00
1200.00
1400.00
1600.00

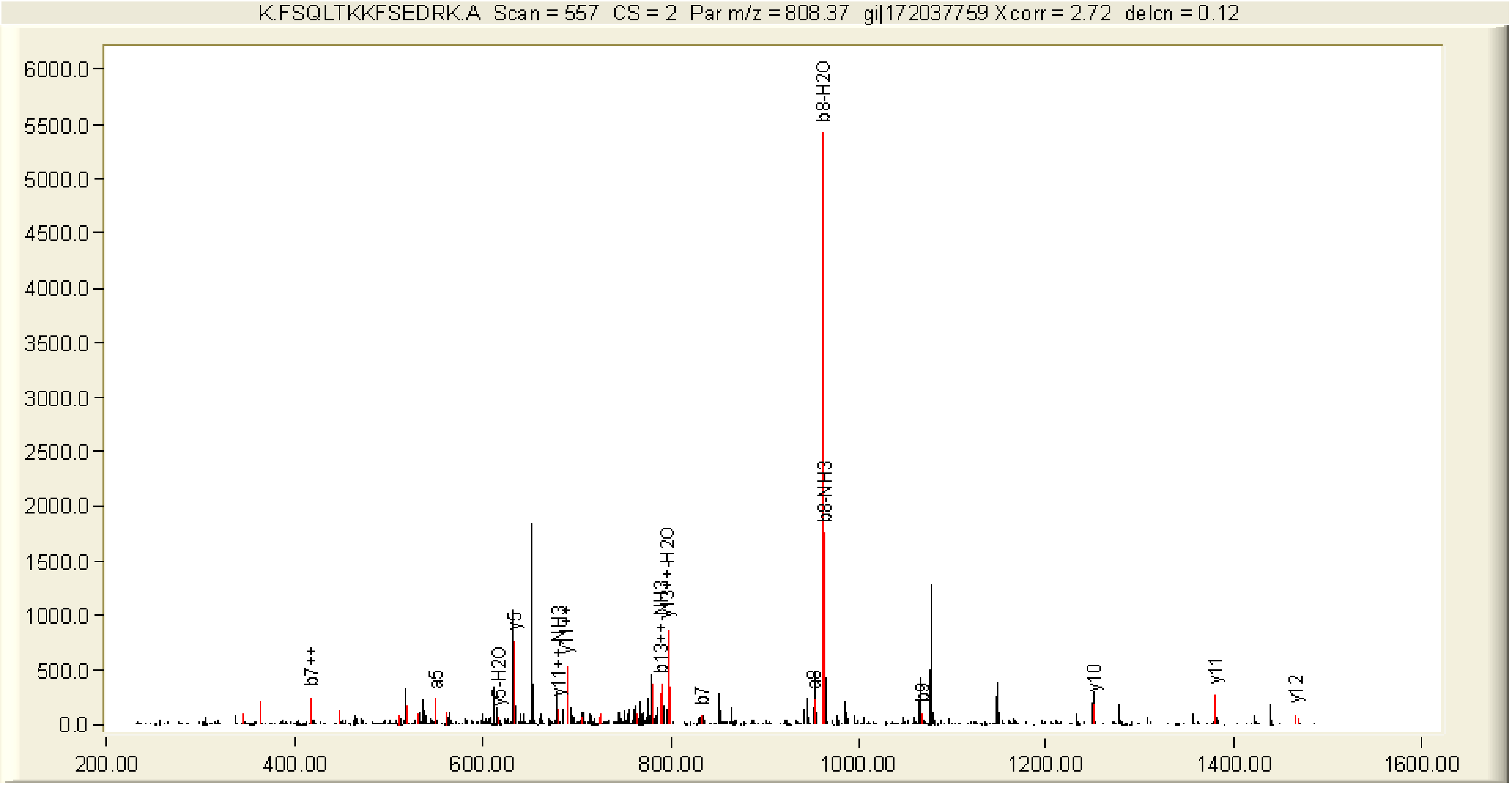
K.FSQLTKKFSEDRK.A Scan = 557 CS = 2 Par m/z = 808.37 gi|172037759 Xcorr = 2.72 delcn = 0.12
6000.0
5500.0
5000.0
4500.0
4000.0
3500.0
3000.0
2500.0
2000.0
1500.0
1000.0
500.0
0.0
200.00
400.00
600.00
800.00
1000.00
1200.00
1400.00
1600.00
b8-H2O
b8-NH3
b7++
a5
y5-H2O
y5
b7
a8
b9
y10
y11
y12

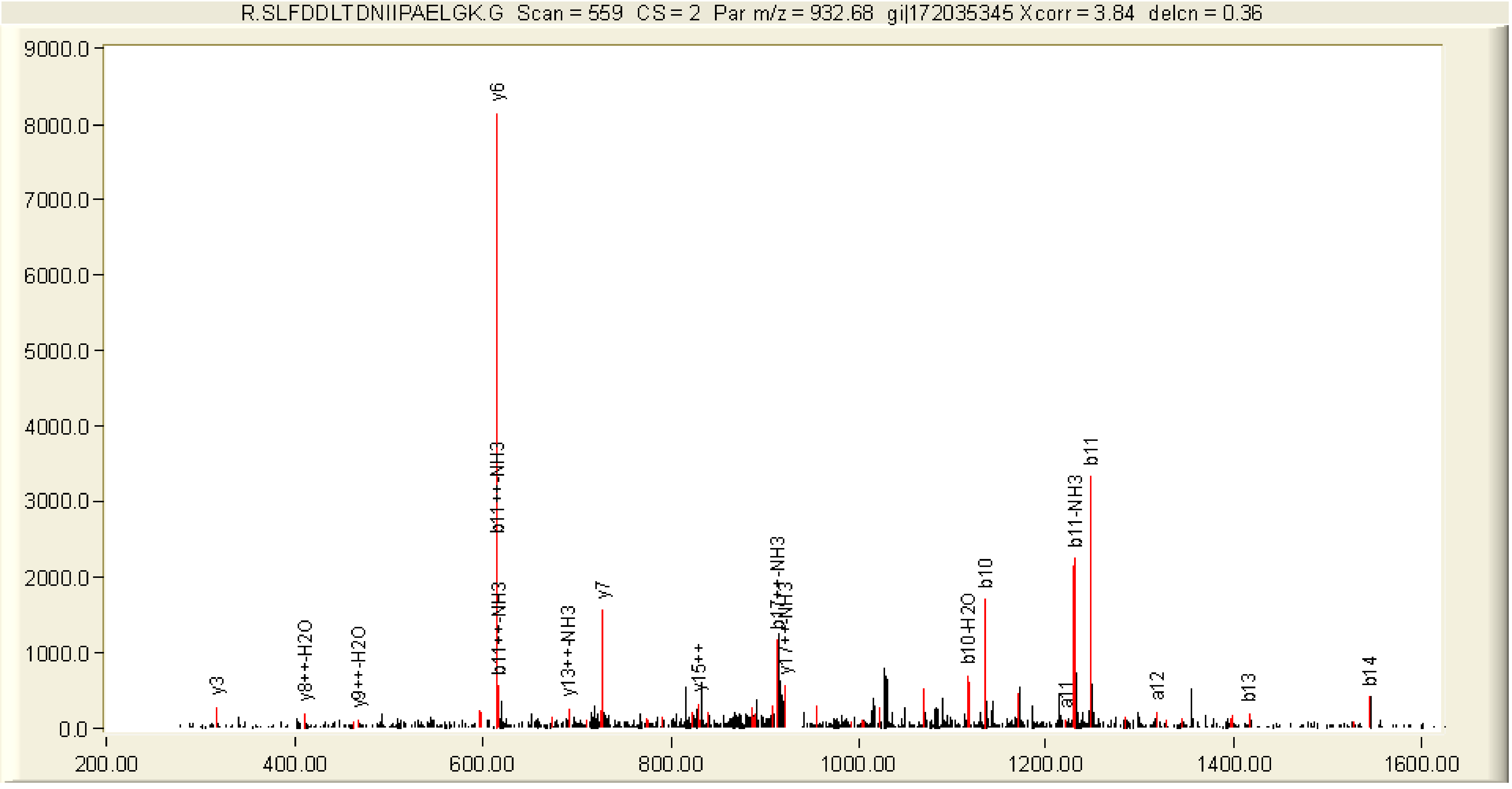
R.SLFDDLTDNIIPAELGK.G Scan = 559 CS = 2 Par m/z = 932.68 gi|172035345 Xcorr = 3.84 delcn = 0.36
y6
b11++-NH3
b11-NH3
b11
b10
b10-H2O
y7
y13++-NH3
y15++
y17++-NH3
y3
y8++-H2O
y9++-H2O
a11
a12
b13
b14
9000.0
8000.0
7000.0
6000.0
5000.0
4000.0
3000.0
2000.0
1000.0
0.0
200.00
400.00
600.00
800.00
1000.00
1200.00
1400.00
1600.00

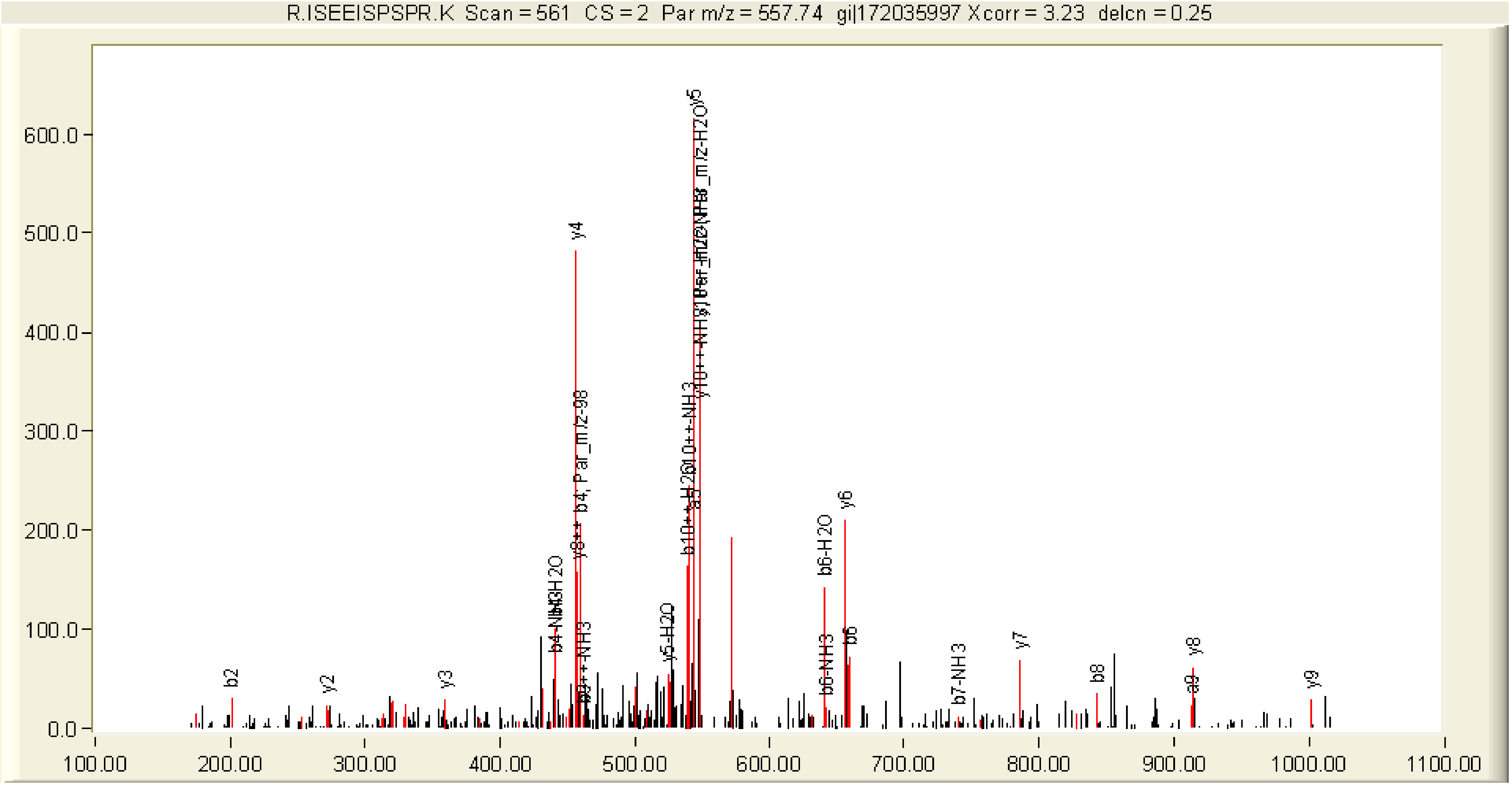
R.ISEEISPSPR.K Scan = 561 CS = 2 Par m/z = 557.74 gi|172035997 Xcorr = 3.23 delcn = 0.25
600.0
500.0
400.0
300.0
200.0
100.0
0.0
100.00
200.00
300.00
400.00
500.00
600.00
700.00
800.00
900.00
1000.00
1100.00
b2
y2
y3
y4
b4, Par_m/z-98
y8++
y5-H2O
b6-H2O
y6
b6-NH3
b6
b7-NH3
y7
b8
y8
a9
y9

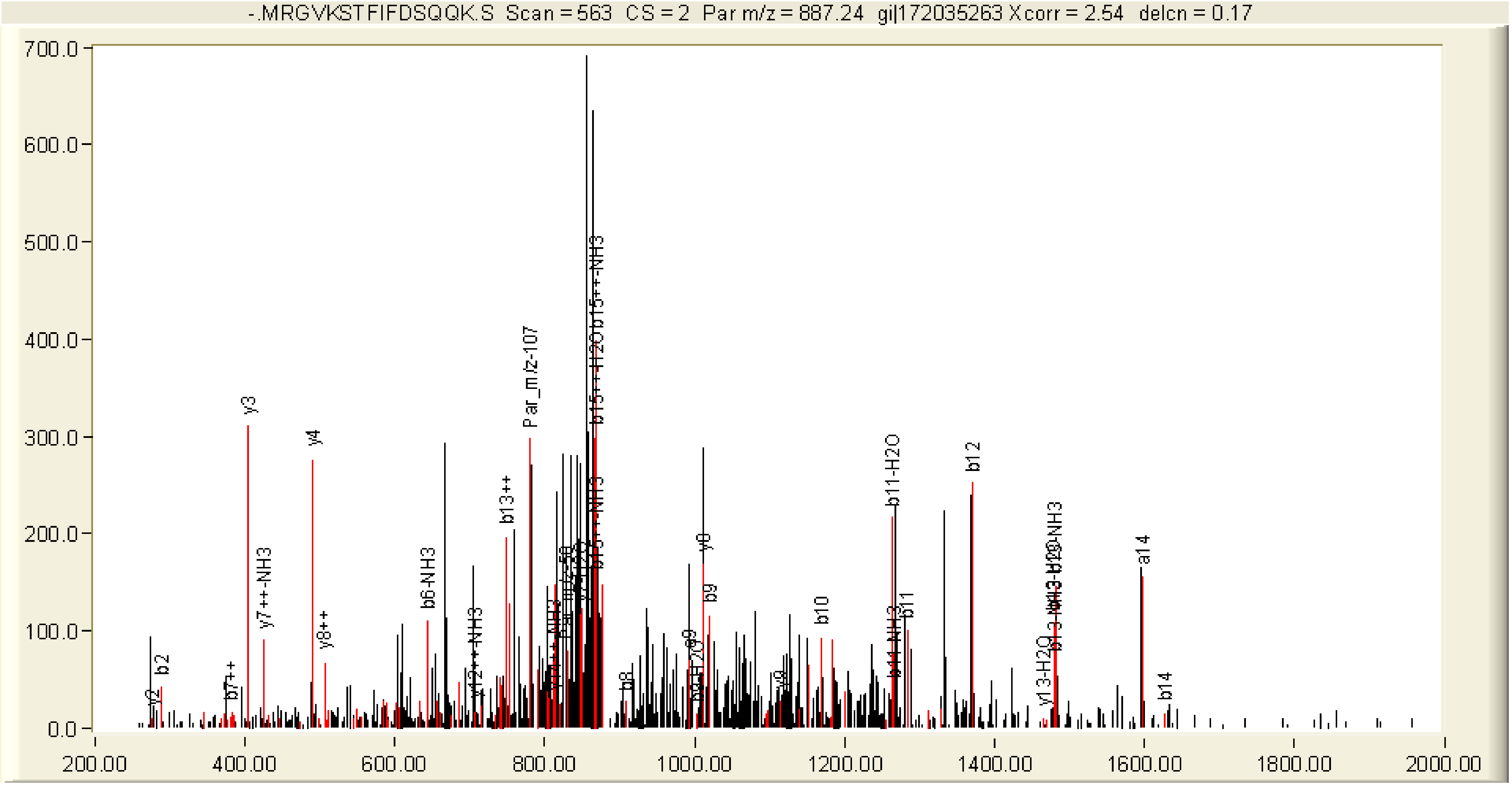
-.MRGVKSTFIFDSQQK.S Scan = 563 CS = 2 Par m/z = 887.24 gi|172035263 Xcorr = 2.54 delcn = 0.17
0.0
100.0
200.0
300.0
400.0
500.0
600.0
700.0
200.00
400.00
600.00
800.00
1000.00
1200.00
1400.00
1600.00
1800.00
2000.00
y2
b2
b7++
y3
y7++-NH3
y4
y8++
b6-NH3
y12++-NH3
b13++
Par_m/z-107
b15++-H2O
b15++-NH3
y8
b8
b9
b10
b11-H2O
b11
b12
y13-H2O
a14
b14

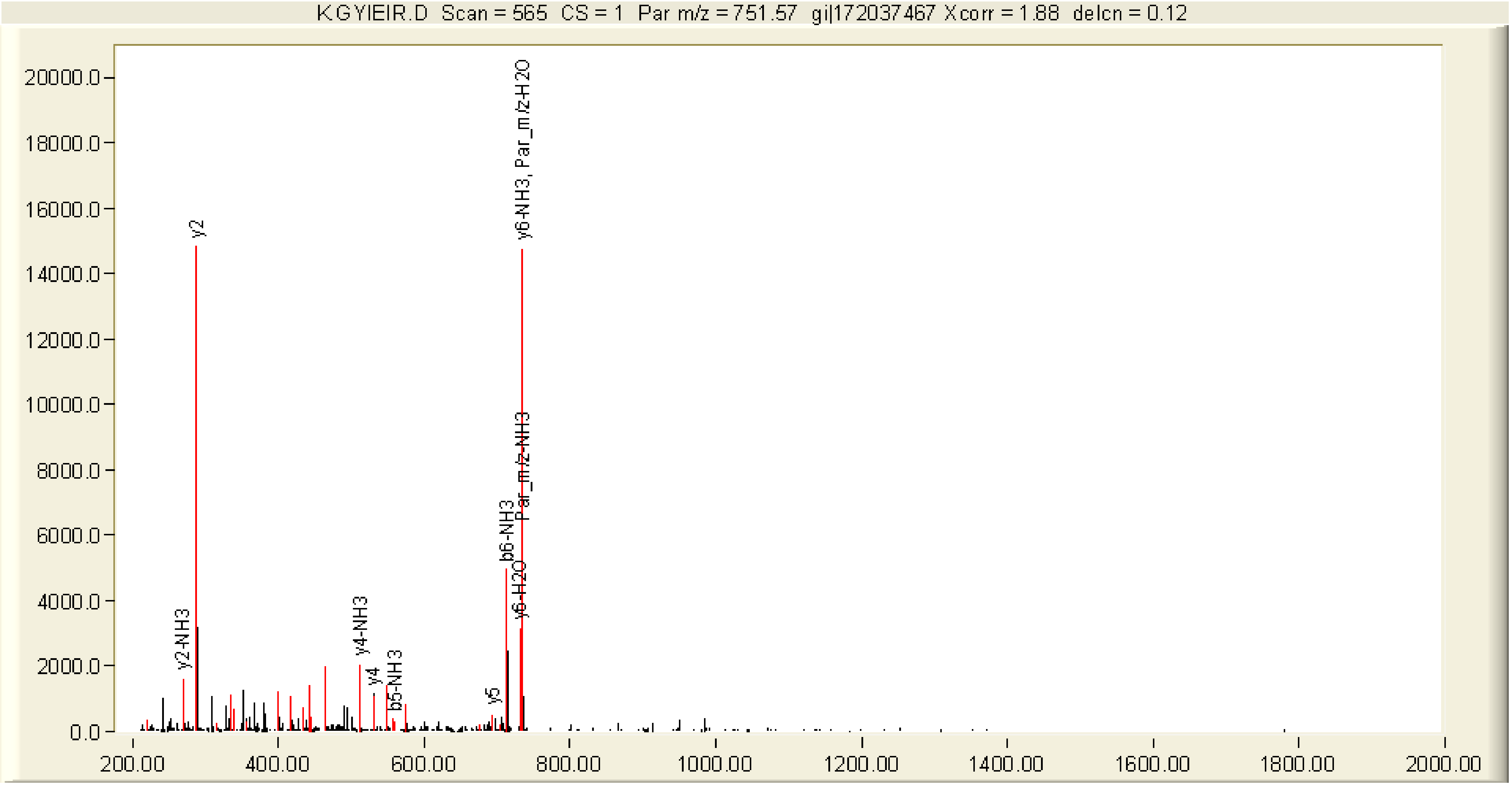
KGYIEIR.D Scan = 565 CS = 1 Par m/z = 751.57 gi|172037467 Xcorr = 1.88 delcn = 0.12
y2
y6-NH3, Par_m/z-H2O
Par_m/z-NH3
b6-NH3
y6-H2O
y2-NH3
y4-NH3
y4
b5-NH3
y5
20000.0
18000.0
16000.0
14000.0
12000.0
10000.0
8000.0
6000.0
4000.0
2000.0
0.0
200.00
400.00
600.00
800.00
1000.00
1200.00
1400.00
1600.00
1800.00
2000.00

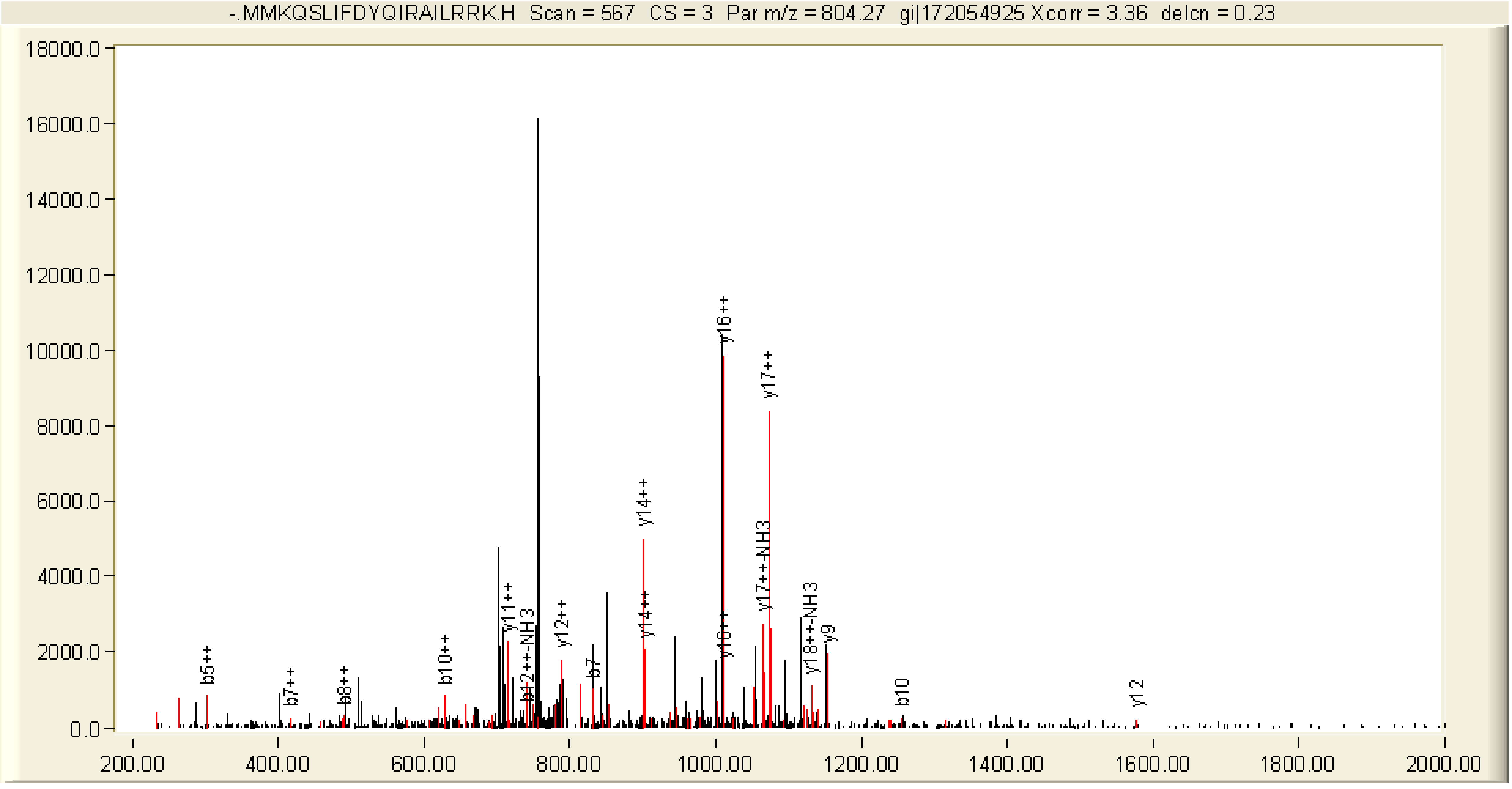
-.MMKQSLIFDYQIRAILRRK.H Scan = 567 CS = 3 Par m/z = 804.27 gi|172054925 Xcorr = 3.36 delcn = 0.23
18000.0
16000.0
14000.0
12000.0
10000.0
8000.0
6000.0
4000.0
2000.0
0.0
200.00
400.00
600.00
800.00
1000.00
1200.00
1400.00
1600.00
1800.00
2000.00
b5++
b7++
b8++
b10++
y11++
b12++-NH3
y12++
b7
y14++
y14++
y16++
y16++
y17++
y17++-NH3
y18++-NH3
y9
b10
y12

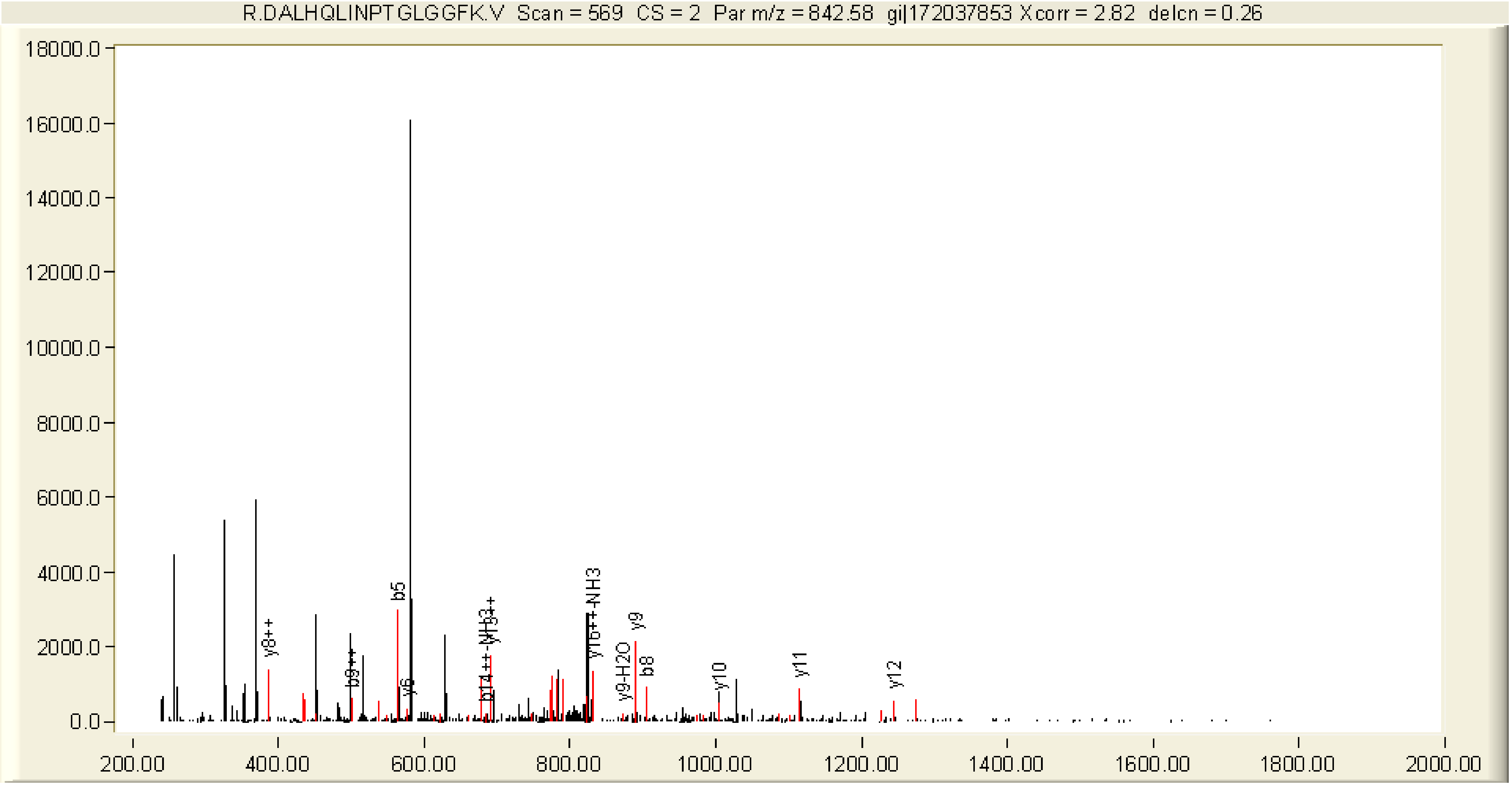

R.DALHQLINPTGLGGFK.V Scan = 569 CS = 2 Par m/z = 842.58 gi|172037853 Xcorr = 2.82 delcn = 0.26
18000.0
16000.0
14000.0
12000.0
10000.0
8000.0
6000.0
4000.0
2000.0
0.0
200.00
400.00
600.00
800.00
1000.00
1200.00
1400.00
1600.00
1800.00
2000.00
y8++
b9++
b5
y6
b14++-NH3
y13++
y16++-NH3
y9-H2O
y9
b8
y10
y11
y12

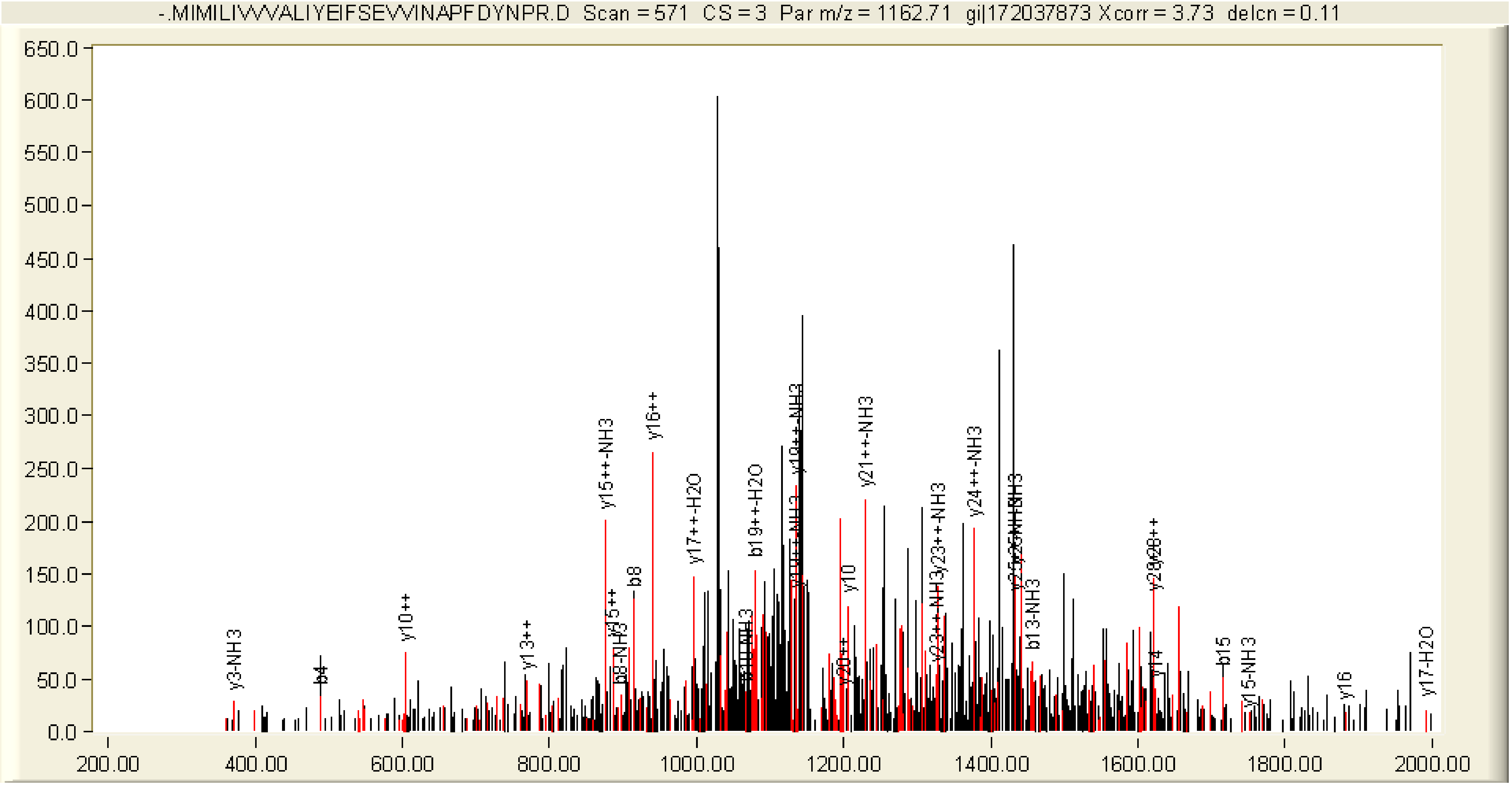
-.MIMILIVVVALIYEIFSEVVINAPFDYNPR.D Scan = 571 CS = 3 Par m/z = 1162.71 gi|172037873 Xcorr = 3.73 delcn = 0.11
0.0
50.0
100.0
150.0
200.0
250.0
300.0
350.0
400.0
450.0
500.0
550.0
600.0
650.0
200.00
400.00
600.00
800.00
1000.00
1200.00
1400.00
1600.00
1800.00
2000.00
y3-NH3
b4
y10++
y13++
y15++-NH3
b8-NH3
b8
y16++
y17++-H2O
b19++-H2O
y21++-NH3
y10
y20++
y23++-NH3
y24++-NH3
b13-NH3
y14
b15
y15-NH3
y16
y17-H2O

K.SGKLVKEVIAGK.Q Scan = 573 CS = 1 Par m/z = 1228.71 gi|172037077 Xcorr = 1.86 delcn = 0.11

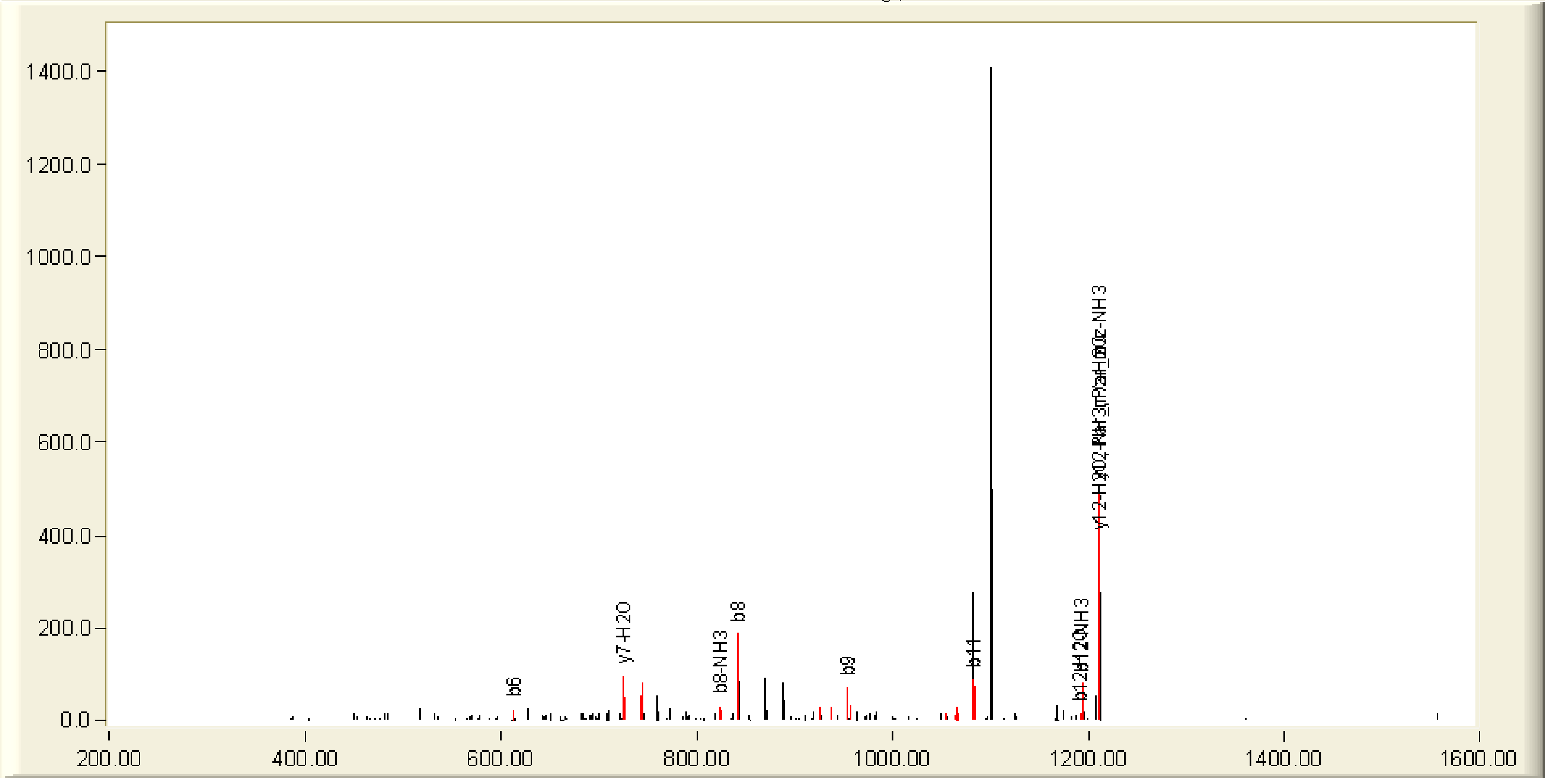

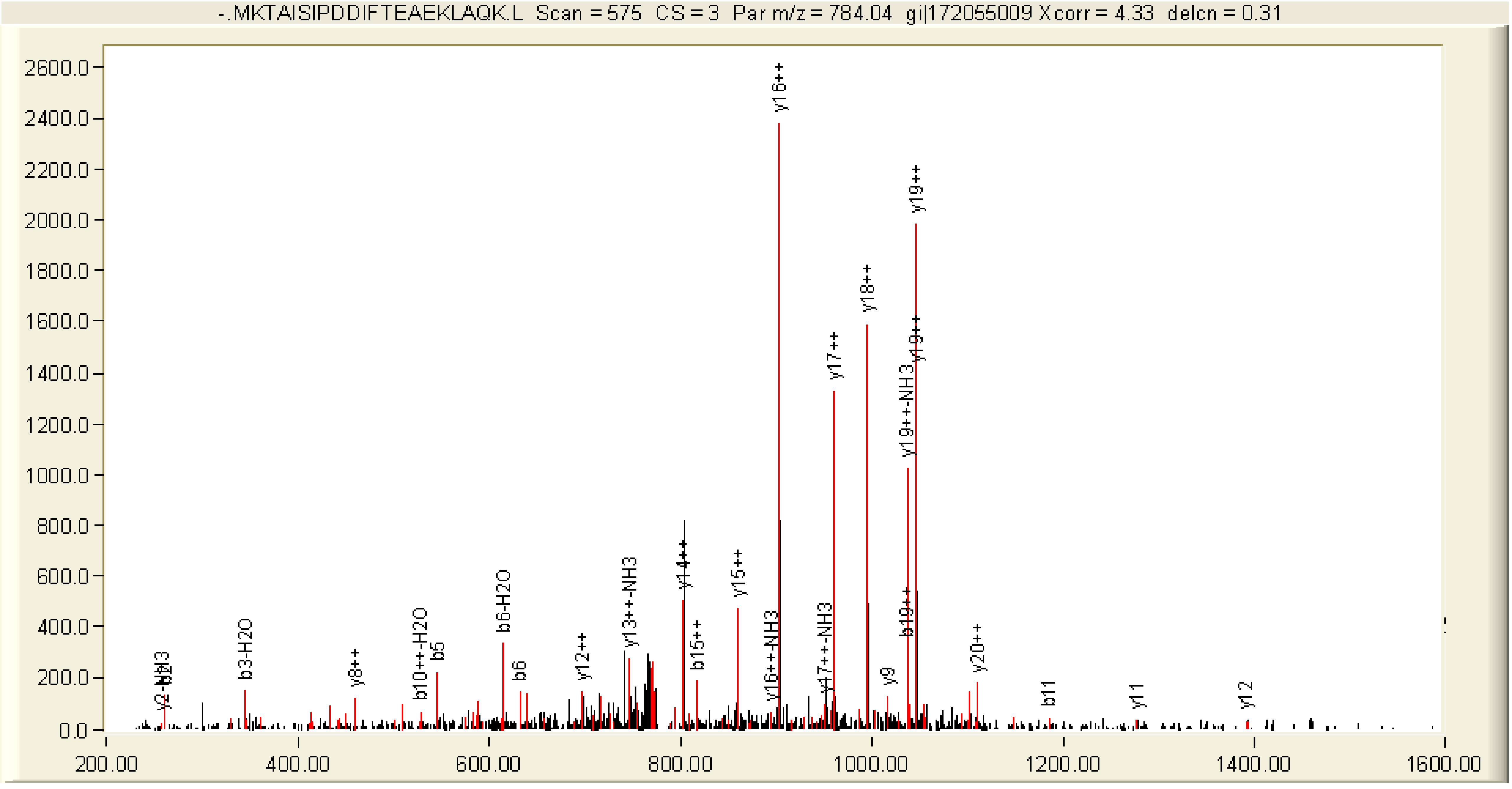
-.MKTAISIPDDIFTEAEKLAQK.L Scan = 575 CS = 3 Par m/z = 784.04 gi|172055009 Xcorr = 4.33 delcn = 0.31
y16++
y19++
y18++
y17++
y19++-NH3
y14++
y15++
y13++-NH3
b6-H2O
b15++
y16++-NH3
y17++-NH3
b19++
y20++
b3-H2O
b10++-H2O
b5
b6
y12++
y8++
y9
b11
y11
y12
y2-NH3
2600.0
2400.0
2200.0
2000.0
1800.0
1600.0
1400.0
1200.0
1000.0
800.0
600.0
400.0
200.0
0.0
200.00
400.00
600.00
800.00
1000.00
1200.00
1400.00
1600.00

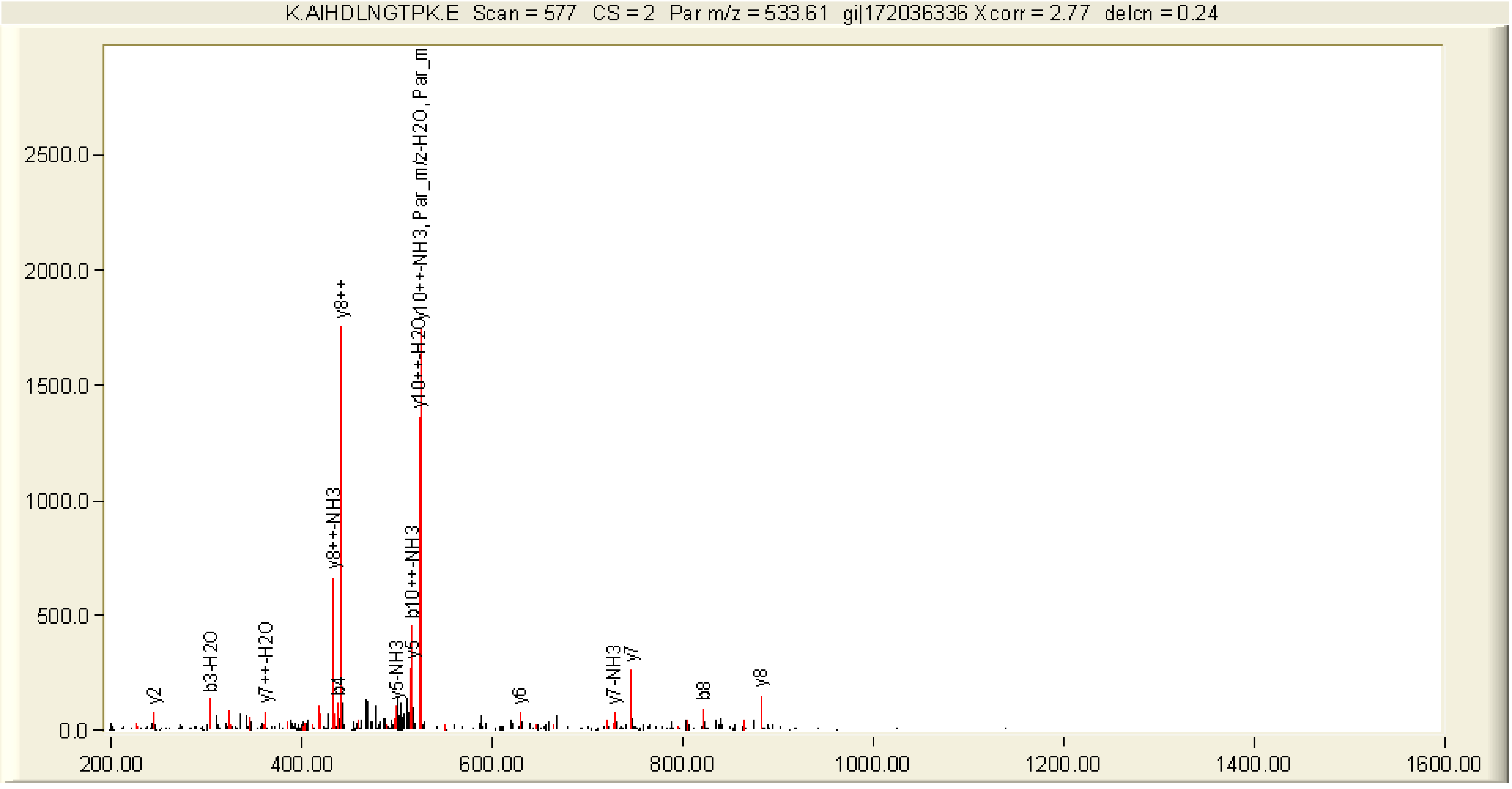

K.AIHDLNGTPK.E Scan = 577 CS = 2 Par m/z = 533.61 gi|172036336 Xcorr = 2.77 delcn = 0.24
2500.0
2000.0
1500.0
1000.0
500.0
0.0
200.00
400.00
600.00
800.00
1000.00
1200.00
1400.00
1600.00
y2
b3-H2O
y7++-H2O
y8++-NH3
y8++
b4
y5-NH3
y5
b10++-NH3
y10++-H2O
y10++-NH3, Par_m/z-H2O, Par_m
y6
y7-NH3
y7
b8
y8

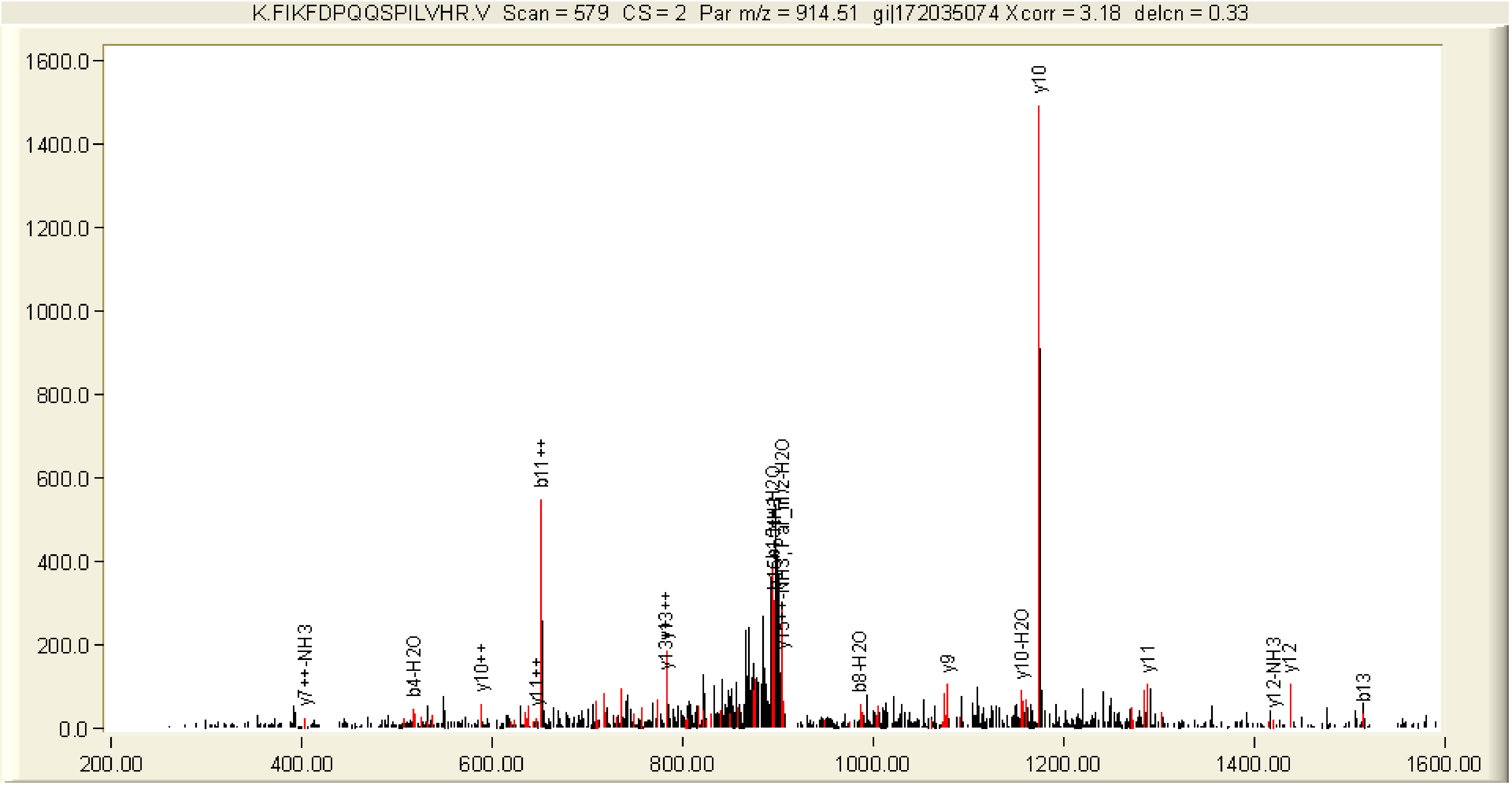

K.FIKFDPQQSPILVHR.V Scan = 579 CS = 2 Par m/z = 914.51 gi|172035074 Xcorr = 3.18 delcn = 0.33
0.0
200.0
400.0
600.0
800.0
1000.0
1200.0
1400.0
1600.0
200.00
400.00
600.00
800.00
1000.00
1200.00
1400.00
1600.00
y7++-NH3
b4-H2O
y10++
y11++
b11++
b8-H2O
y9
y10-H2O
y10
y11
y12-NH3
y12
b13

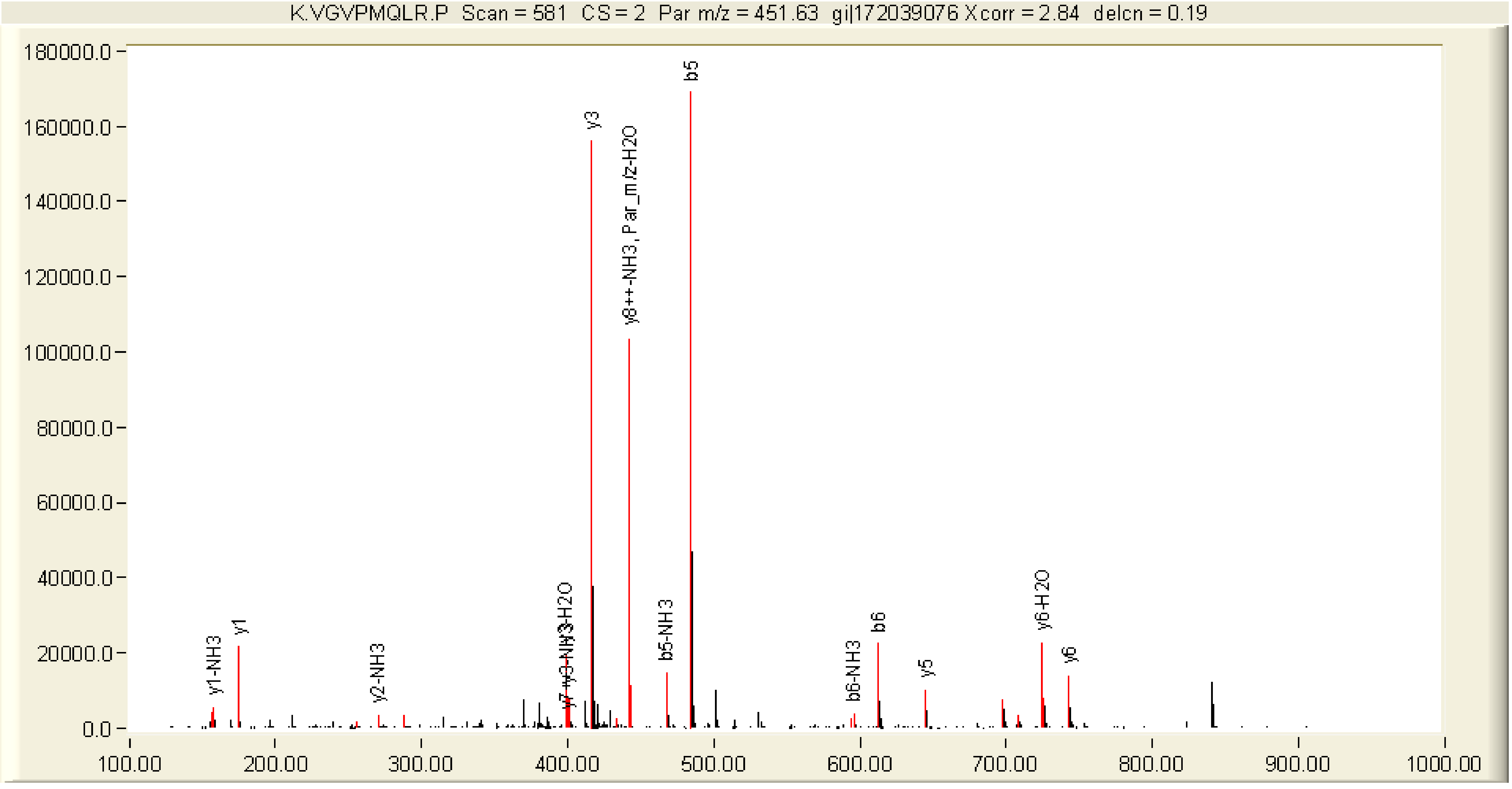

K.VGVPMQLR.P Scan = 581 CS = 2 Par m/z = 451.63 gi|172039076 Xcorr = 2.84 delcn = 0.19
180000.0
160000.0
140000.0
120000.0
100000.0
80000.0
60000.0
40000.0
20000.0
0.0
100.00
200.00
300.00
400.00
500.00
600.00
700.00
800.00
900.00
1000.00
y1-NH3
y1
y2-NH3
y3
y8++-NH3, Par_m/z-H2O
b5
b5-NH3
b6-NH3
b6
y5
y6-H2O
y6

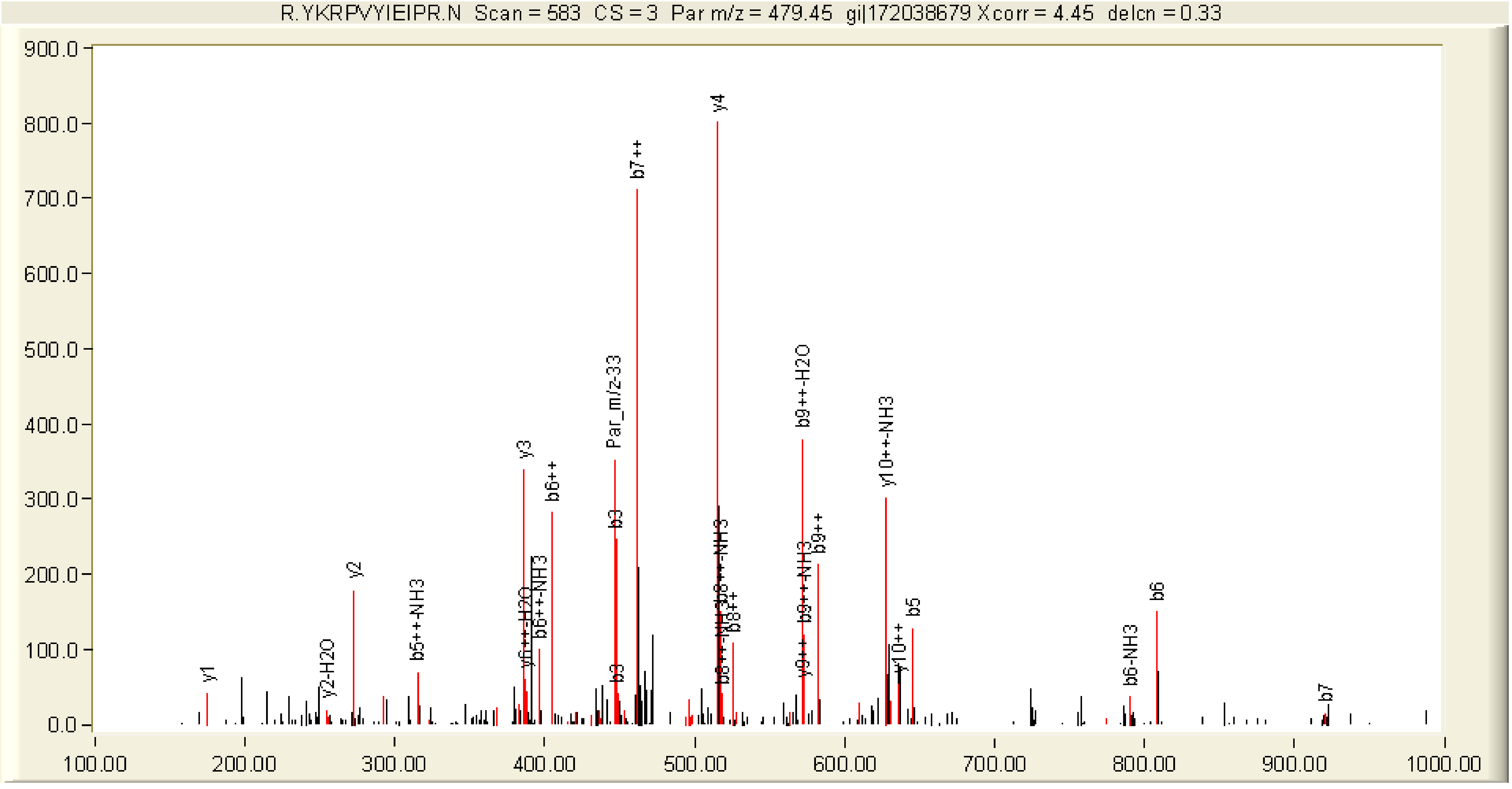
R.YKRPVYIEIPR.N Scan = 583 CS = 3 Par m/z = 479.45 gi|172038679 Xcorr = 4.45 delcn = 0.33
y1
y2-H2O
y2
b5++-NH3
y3
b6++-NH3
b6++
Par_m/z-33
b3
b7++
y4
b9++-H2O
b9++
y9++
y10++-NH3
y10++
b5
b6-NH3
b6
b7

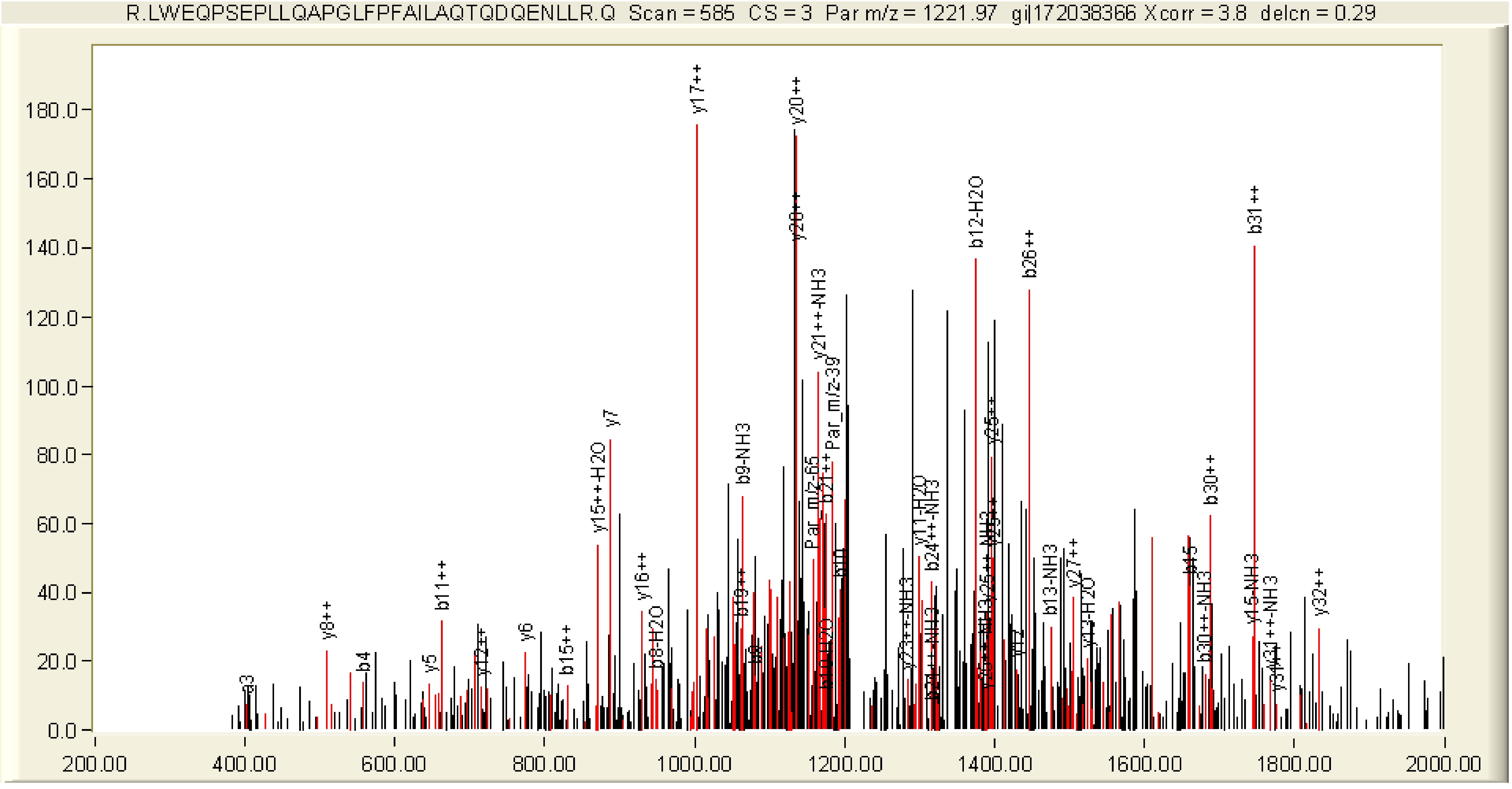
R.LWEQPSEPLLQAPGLFPFAILAQTQDQENLLR.Q Scan = 585 CS = 3 Par m/z = 1221.97 gi|172038366 Xcorr = 3.8 delcn = 0.29
180.0
160.0
140.0
120.0
100.0
80.0
60.0
40.0
20.0
0.0
200.00
400.00
600.00
800.00
1000.00
1200.00
1400.00
1600.00
1800.00
2000.00
y17++
y20++
b12-H2O
b31++
b26++
y21++-NH3
Par_m/z-39
y7
y15++-H2O
b9-NH3
b30++
y16++
b11++
y8++
y32++
b13-NH3
y27++
b4
y5
y6
b15++
a3

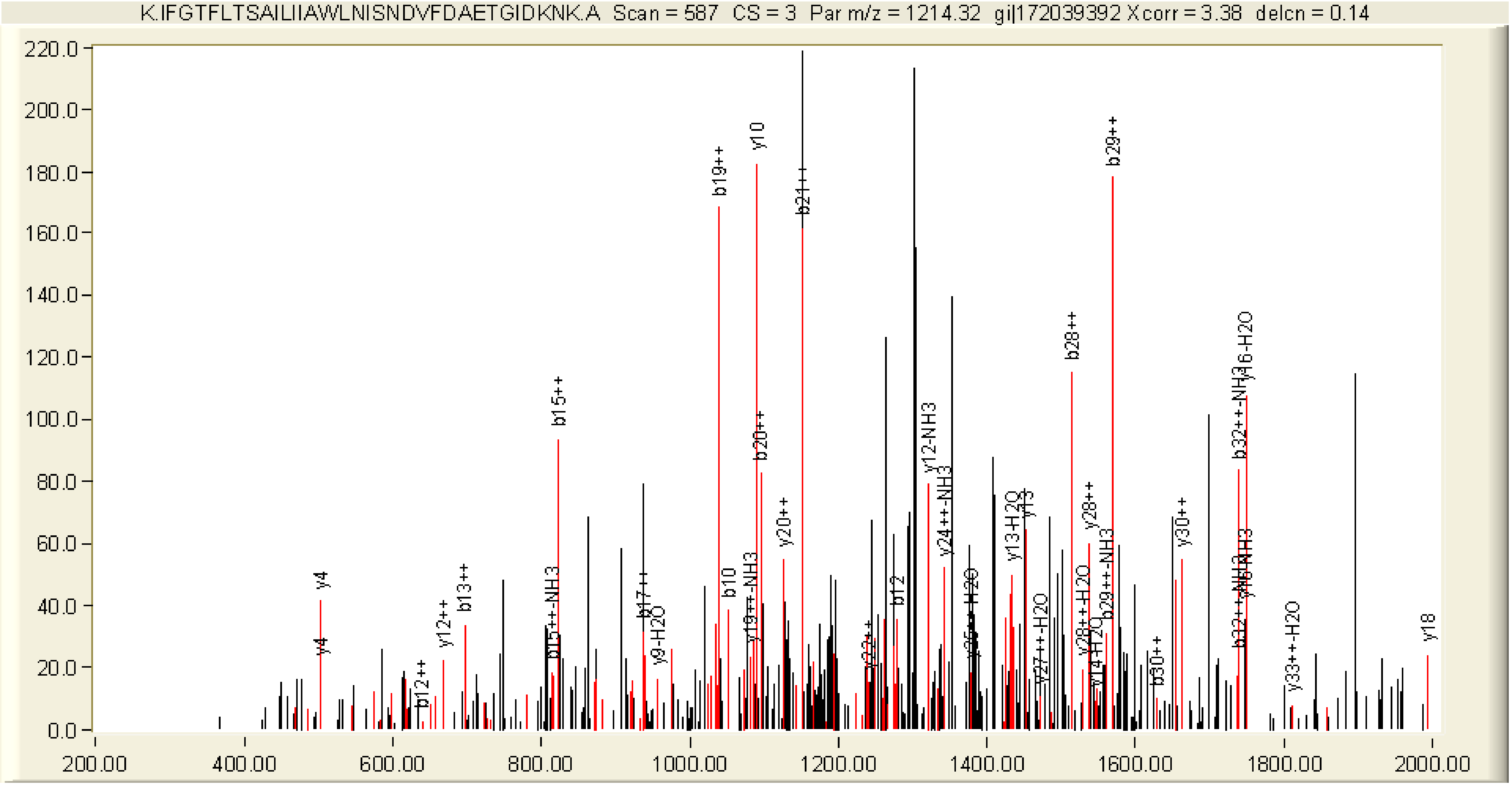
K.IFGTFLTSAILIIAWLNISNDVFDAETGIDKNK.A Scan = 587 CS = 3 Par m/z = 1214.32 gi|172039392 Xcorr = 3.38 delcn = 0.14
220.0
200.0
180.0
160.0
140.0
120.0
100.0
80.0
60.0
40.0
20.0
0.0
200.00
400.00
600.00
800.00
1000.00
1200.00
1400.00
1600.00
1800.00
2000.00
y4
y4
b12++
y12++
b13++
b15++
b15++-NH3
b17++
y9-H2O
b19++
b10
y10
y19++-NH3
b20++
y20++
b21++
b12
y12-NH3
y24++-NH3
y13-H2O
y13
y27++-H2O
b28++
y28++
b29++
b29++-NH3
b30++
y30++
b32++-NH3
y16-H2O
y33++-H2O
y18

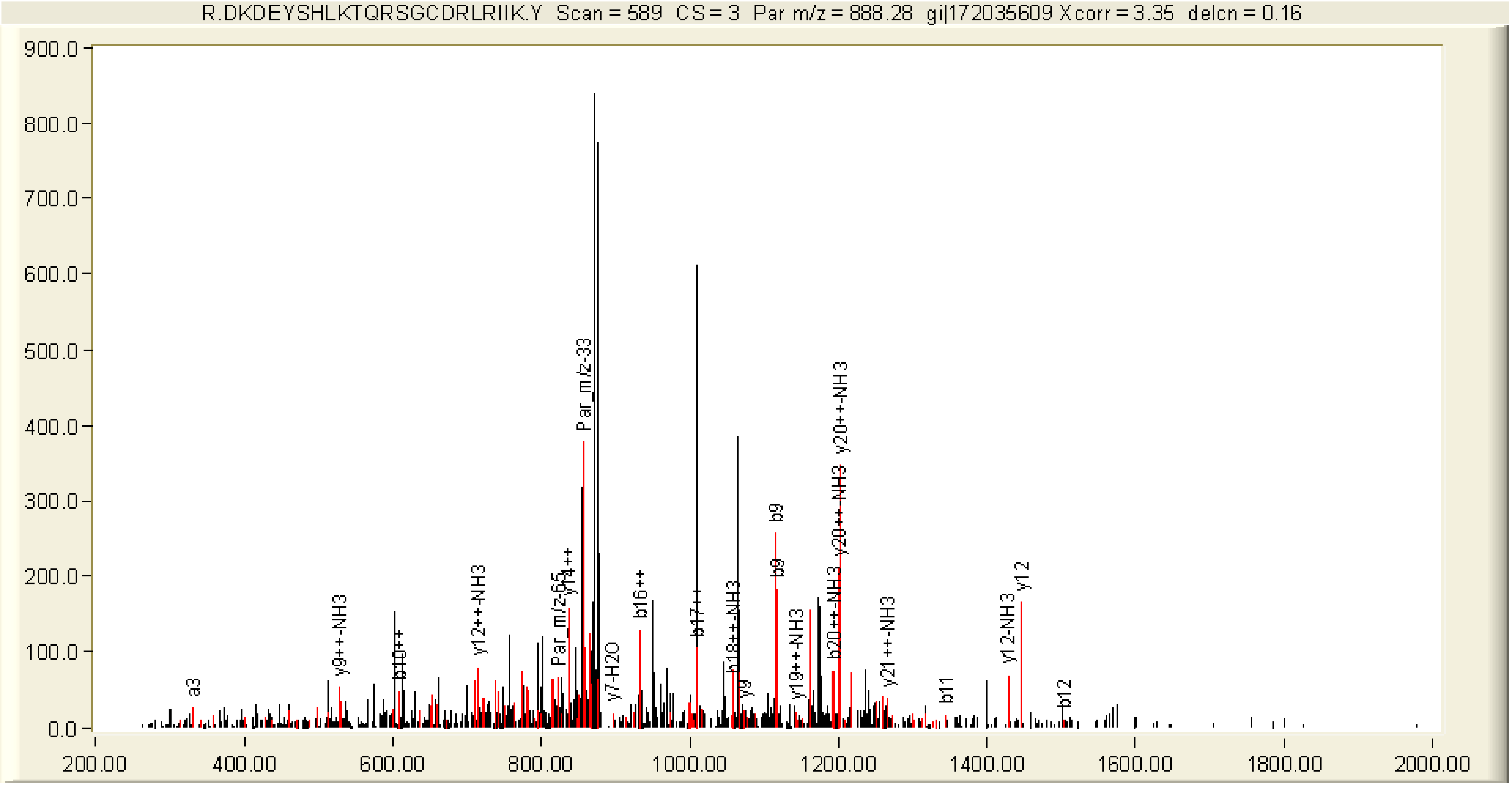

R.DKDEYSHLKTQRSGCDRLRIIK.Y Scan = 589 CS = 3 Par m/z = 888.28 gi|172035609 Xcorr = 3.35 delcn = 0.16
Par_m/z-33
y20++-NH3
b9
y20++-NH3
b9
y14++
Par_m/z-65
y12++-NH3
y9++-NH3
b10++
a3
b16++
b17++
b18++-NH3
y9
y19++-NH3
b20++-NH3
y21++-NH3
y7-H2O
b11
y12-NH3
y12
b12

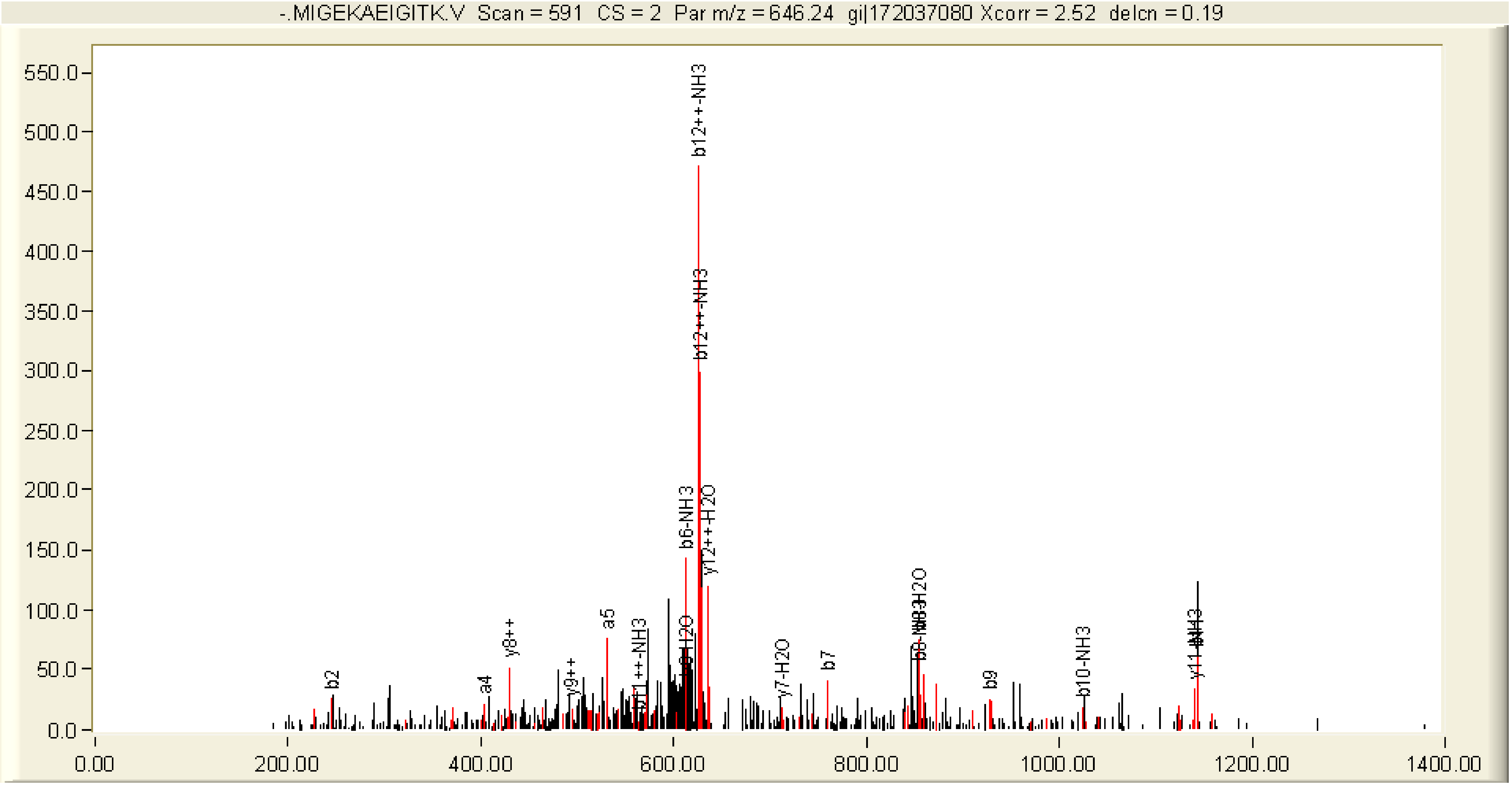
-.MIGEKAEIGITK.V Scan = 591 CS = 2 Par m/z = 646.24 gi|172037080 Xcorr = 2.52 delcn = 0.19
550.0
500.0
450.0
400.0
350.0
300.0
250.0
200.0
150.0
100.0
50.0
0.0
0.00
200.00
400.00
600.00
800.00
1000.00
1200.00
1400.00
b12++-NH3
b12++-NH3
b6-NH3
y12++-H2O
b2
a4
y8++
y9++
a5
y7-H2O
b7
b9
b10-NH3

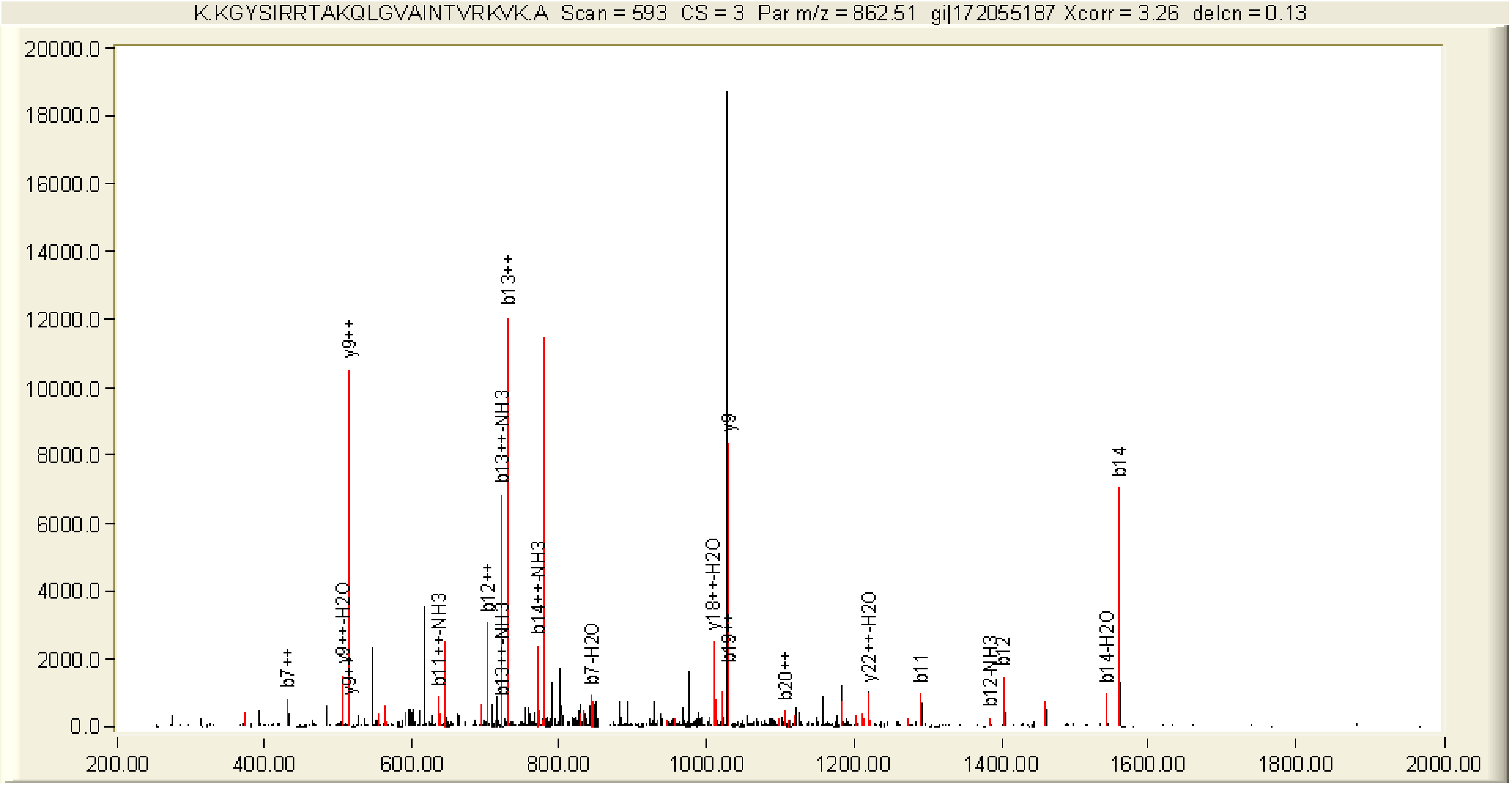
K.KGYSIRRTAKQLGVAINTVRKVK.A Scan = 593 CS = 3 Par m/z = 862.51 gi|172055187 Xcorr = 3.26 delcn = 0.13
20000.0
18000.0
16000.0
14000.0
12000.0
10000.0
8000.0
6000.0
4000.0
2000.0
0.0
200.00
400.00
600.00
800.00
1000.00
1200.00
1400.00
1600.00
1800.00
2000.00
b7++
y9++-H2O
y9++
b11++-NH3
b12++
b13++-NH3
b13++
b14++-NH3
b7-H2O
y18++-H2O
y9
b20++
y22++-H2O
b11
b12-NH3
b12
b14-H2O
b14

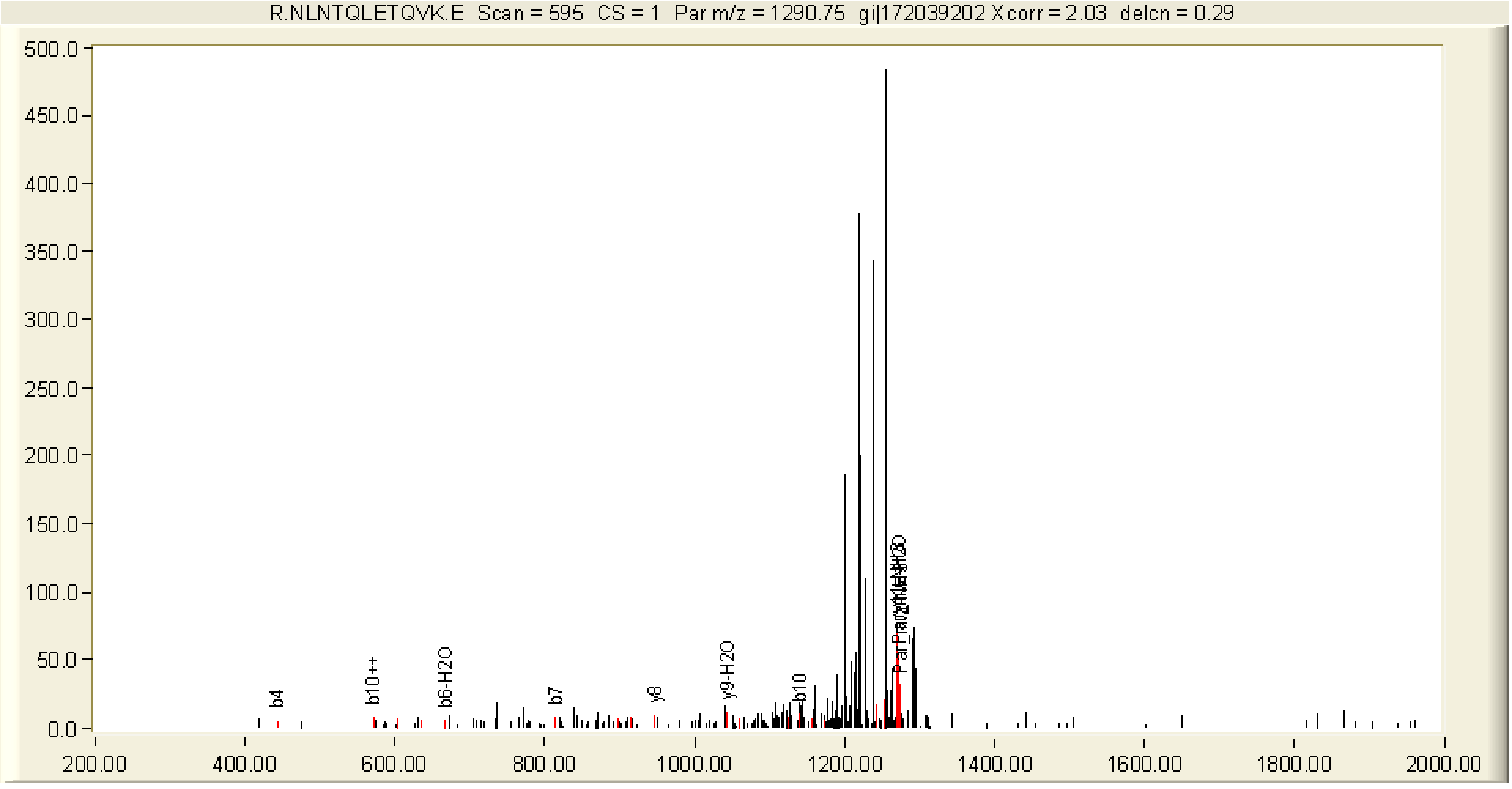
R.NLNTQLETQVK.E Scan = 595 CS = 1 Par m/z = 1290.75 gi|172039202 Xcorr = 2.03 delcn = 0.29
500.0
450.0
400.0
350.0
300.0
250.0
200.0
150.0
100.0
50.0
0.0
b4
b10++
b6-H2O
b7
y8
y9-H2O
b10
200.00
400.00
600.00
800.00
1000.00
1200.00
1400.00
1600.00
1800.00
2000.00

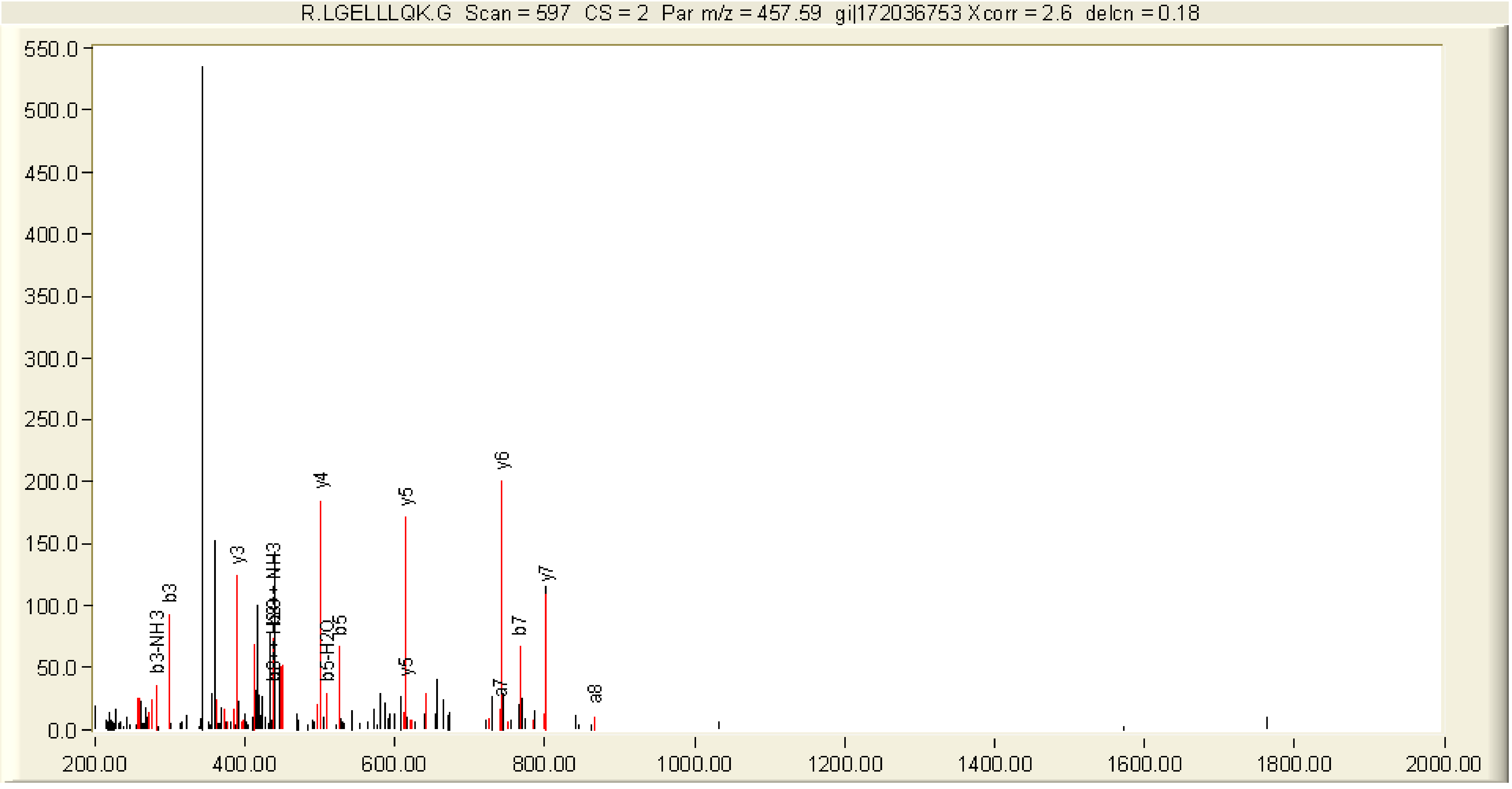
R.LGELLLQK.G Scan = 597 CS = 2 Par m/z = 457.59 gi|172036753 Xcorr = 2.6 delcn = 0.18
550.0
500.0
450.0
400.0
350.0
300.0
250.0
200.0
150.0
100.0
50.0
0.0
200.00
400.00
600.00
800.00
1000.00
1200.00
1400.00
1600.00
1800.00
2000.00
b3-NH3
b3
y3
y4
b5-H2O
b5
y5
y5
y6
a7
b7
y7
a8

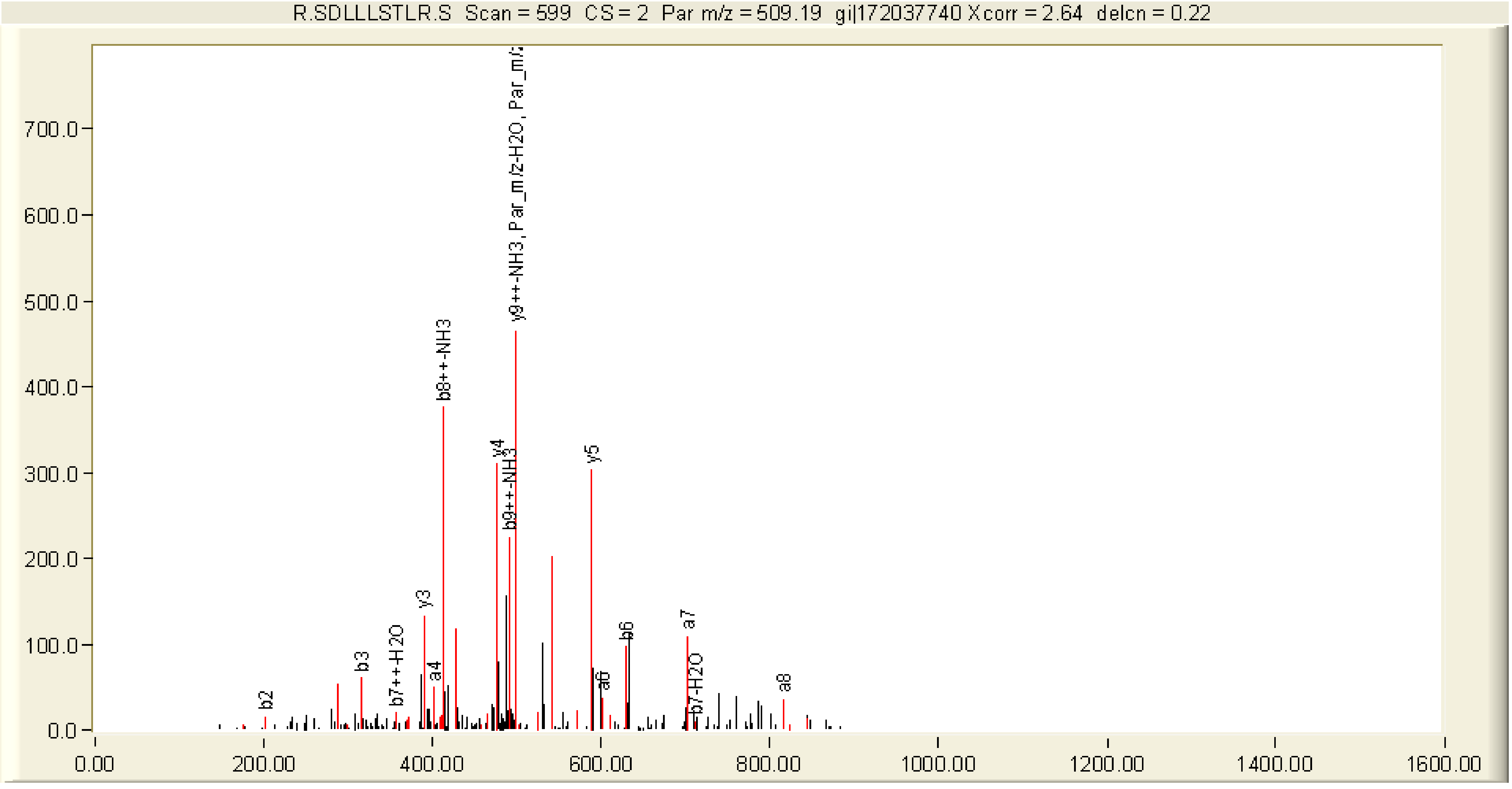
R.SDLLLSTLR.S Scan = 599 CS = 2 Par m/z = 509.19 gi|172037740 Xcorr = 2.64 delcn = 0.22
700.0
600.0
500.0
400.0
300.0
200.0
100.0
0.0
0.00
200.00
400.00
600.00
800.00
1000.00
1200.00
1400.00
1600.00
b2
b3
b7++-H2O
y3
a4
b8++-NH3
y4
b9++-NH3
y9++-NH3, Par_m/z-H2O, Par_m/z
y5
a6
b6
a7
b7-H2O
a8

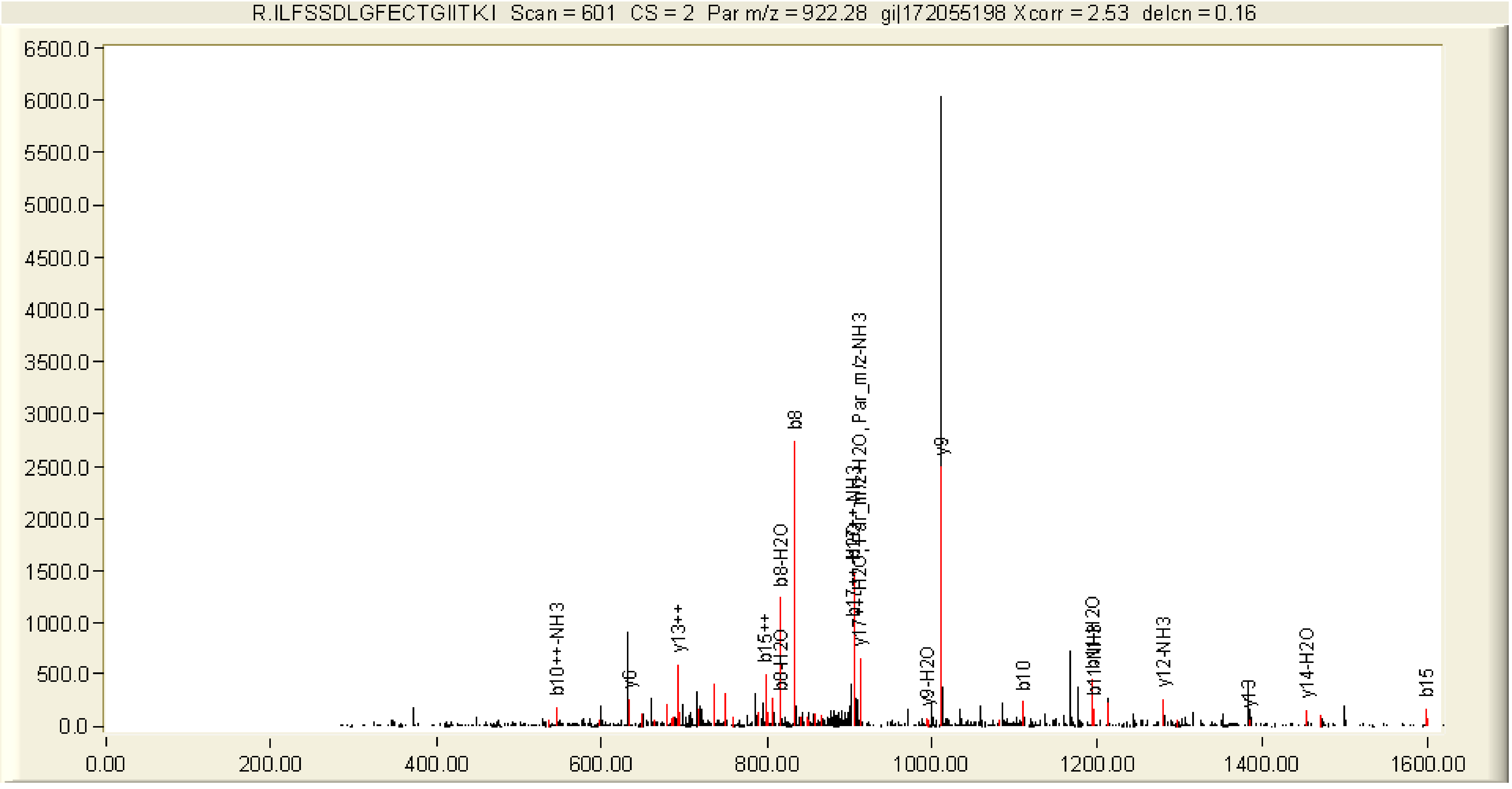
R.ILFSSDLGFECTGIITK.I Scan = 601 CS = 2 Par m/z = 922.28 gi|172055198 Xcorr = 2.53 delcn = 0.16
b10++-NH3
y6
y13++
b15++
b8-H2O
b8
y9
y9-H2O
b10
y12-NH3
y14-H2O
b15
0.0
500.0
1000.0
1500.0
2000.0
2500.0
3000.0
3500.0
4000.0
4500.0
5000.0
5500.0
6000.0
6500.0
0.00
200.00
400.00
600.00
800.00
1000.00
1200.00
1400.00
1600.00

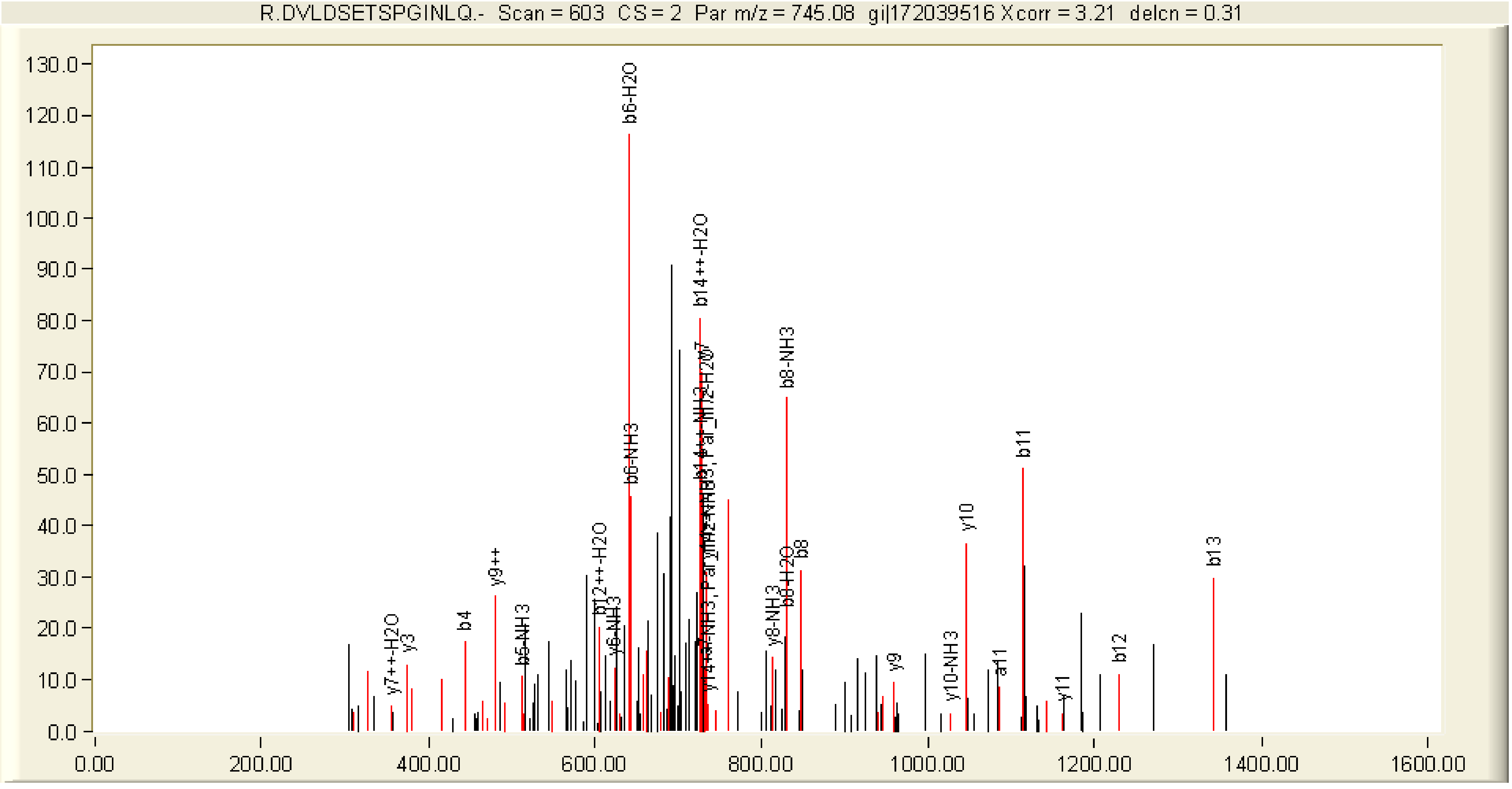

R.DVLDSETSPGINLQ.- Scan = 603 CS = 2 Par m/z = 745.08 gi|172039516 Xcorr = 3.21 delcn = 0.31
130.0
120.0
110.0
100.0
90.0
80.0
70.0
60.0
50.0
40.0
30.0
20.0
10.0
0.0
0.00
200.00
400.00
600.00
800.00
1000.00
1200.00
1400.00
1600.00
b6-H2O
b14++-H2O
b8-NH3
b6-NH3
b11
y10
b13
b8
b8-H2O
y8-NH3
b12++-H2O
y6-NH3
y9++
b4
b5-NH3
y7++-H2O
y3
y9
y10-NH3
a11
y11
b12

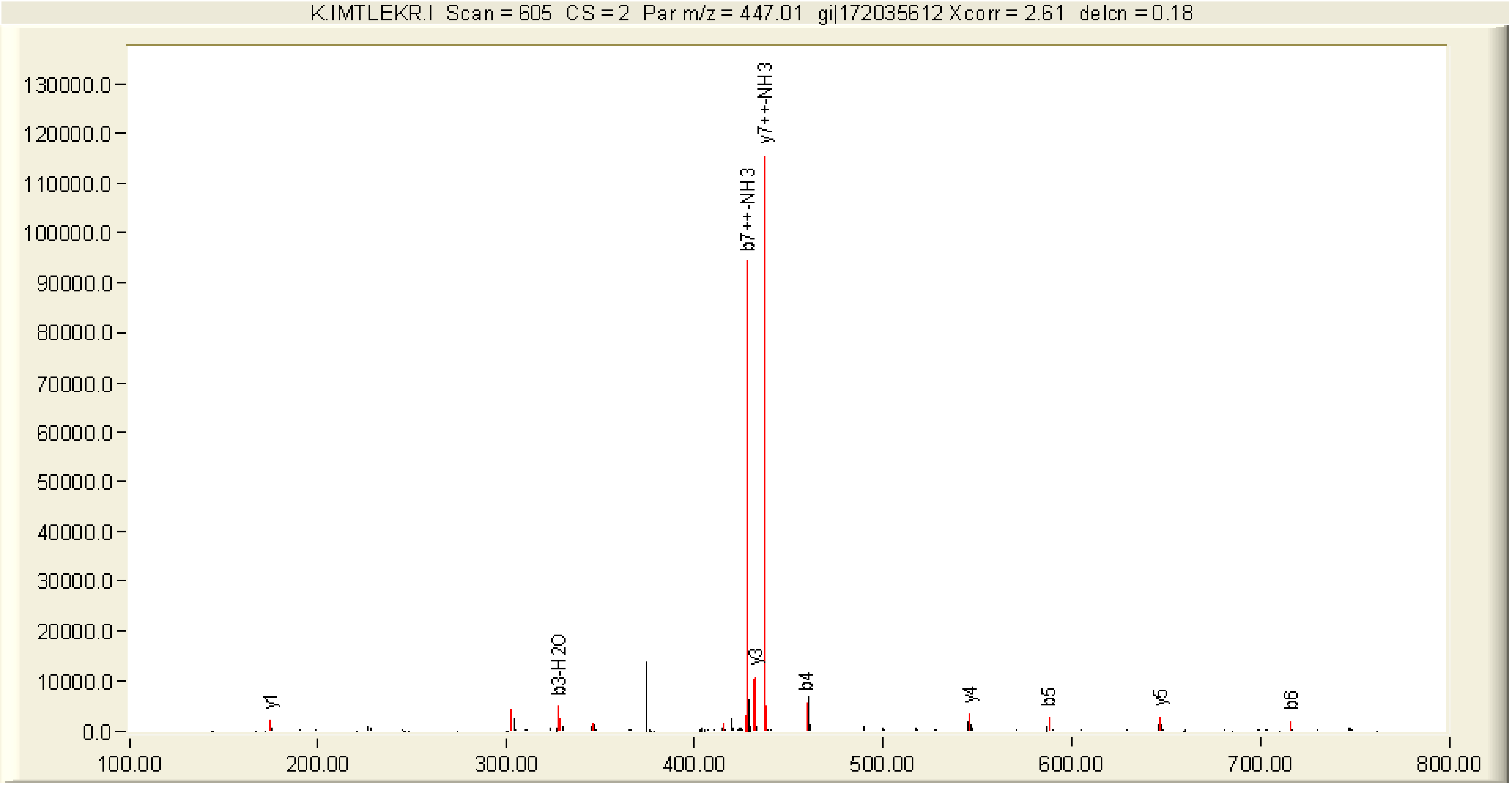
K.IMTLEKR.I Scan = 605 CS = 2 Par m/z = 447.01 gi|172035612 Xcorr = 2.61 delcn = 0.18
130000.0
120000.0
110000.0
100000.0
90000.0
80000.0
70000.0
60000.0
50000.0
40000.0
30000.0
20000.0
10000.0
0.0
100.00
200.00
300.00
400.00
500.00
600.00
700.00
800.00
y1
b3-H2O
b7++-NH3
y7++-NH3
y3
b4
y4
b5
y5
b6

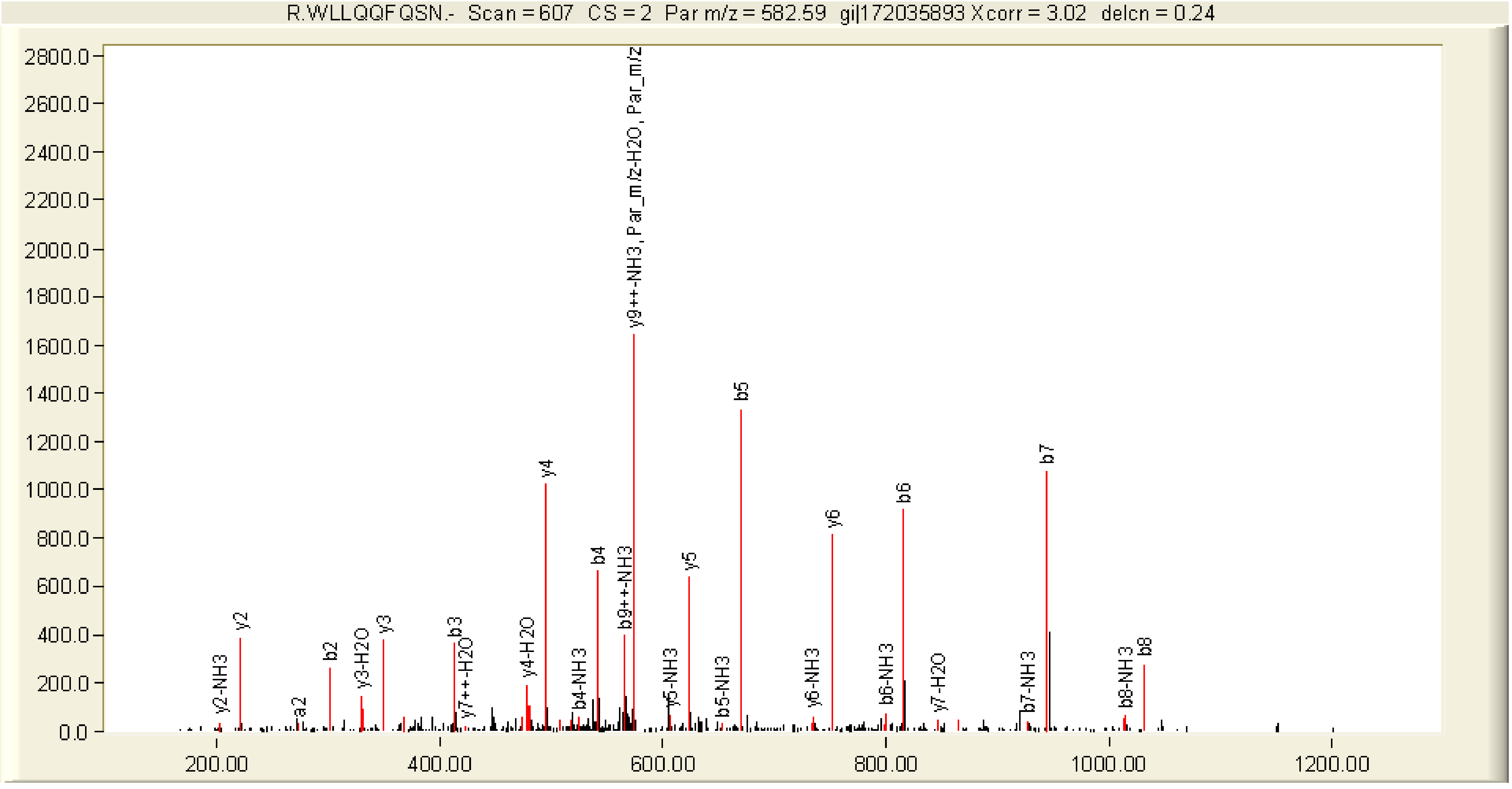
R.WLLQQFQSN.- Scan = 607 CS = 2 Par m/z = 582.59 gi|172035893 Xcorr = 3.02 delcn = 0.24
y9++-NH3, Par_m/z-H2O, Par_m/z
b5
b7
y4
b6
y6
b4
b9++-NH3
y5
y2
y3
b3
b2
b8
y2-NH3
a2
y3-H2O
y7++-H2O
y4-H2O
b4-NH3
y5-NH3
b5-NH3
y6-NH3
b6-NH3
y7-H2O
b7-NH3
b8-NH3
0.0
200.0
400.0
600.0
800.0
1000.0
1200.0
1400.0
1600.0
1800.0
2000.0
2200.0
2400.0
2600.0
2800.0
200.00
400.00
600.00
800.00
1000.00
1200.00

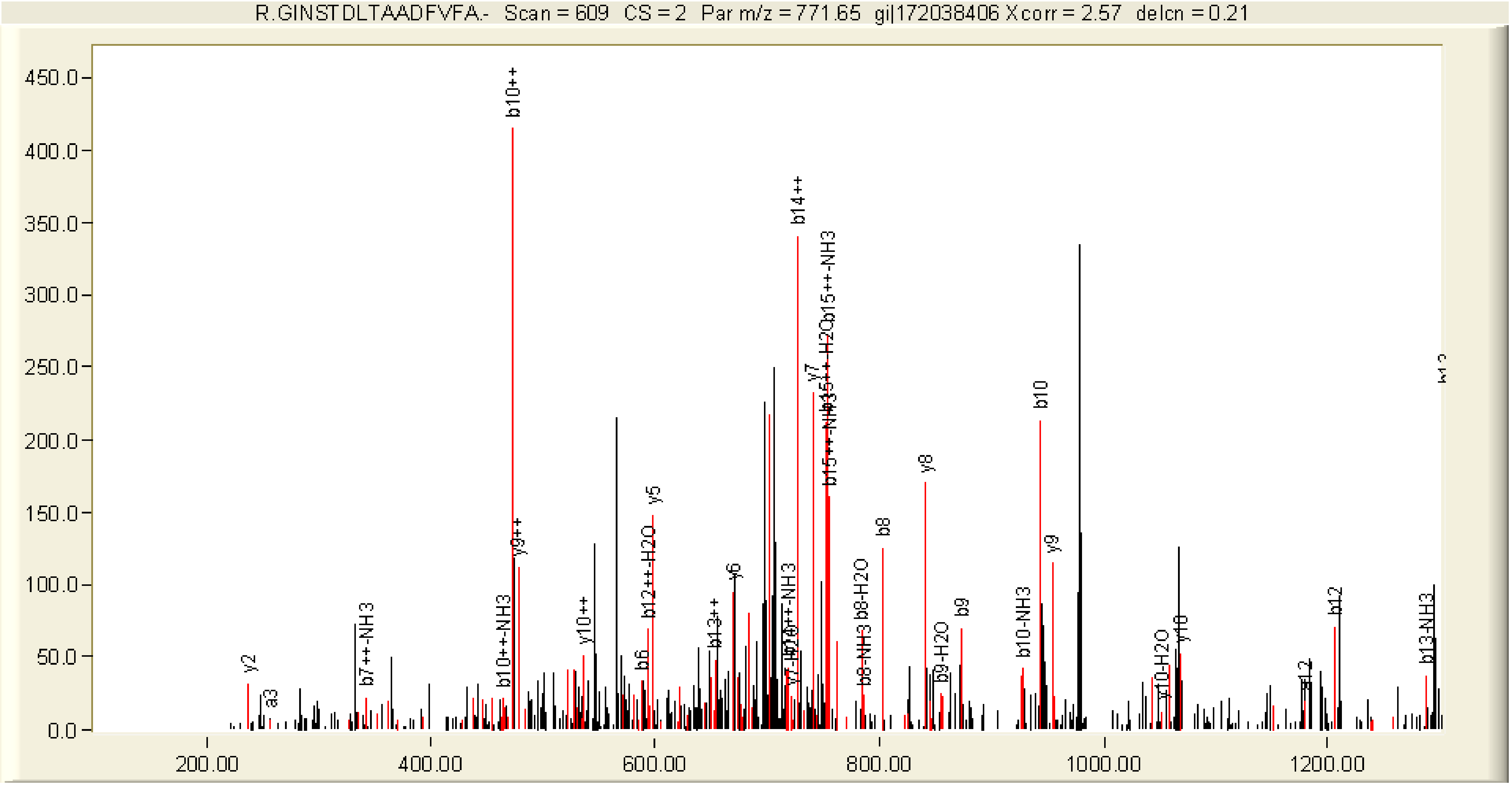
R.GINSTDLTAADFVFA.- Scan = 609 CS = 2 Par m/z = 771.65 gi|172038406 Xcorr = 2.57 delcn = 0.21
450.0
400.0
350.0
300.0
250.0
200.0
150.0
100.0
50.0
0.0
200.00
400.00
600.00
800.00
1000.00
1200.00
b10++
b14++
b15++-NH3
y7
b10
y8
y5
b8
y9++
y9
y6
b12++-H2O
b7++-NH3
b10++-NH3
y10++
b13++
b8-H2O
b8-NH3
b9
b9-H2O
b10-NH3
y10-H2O
y10
b12
b13-NH3
y2
a3
b6

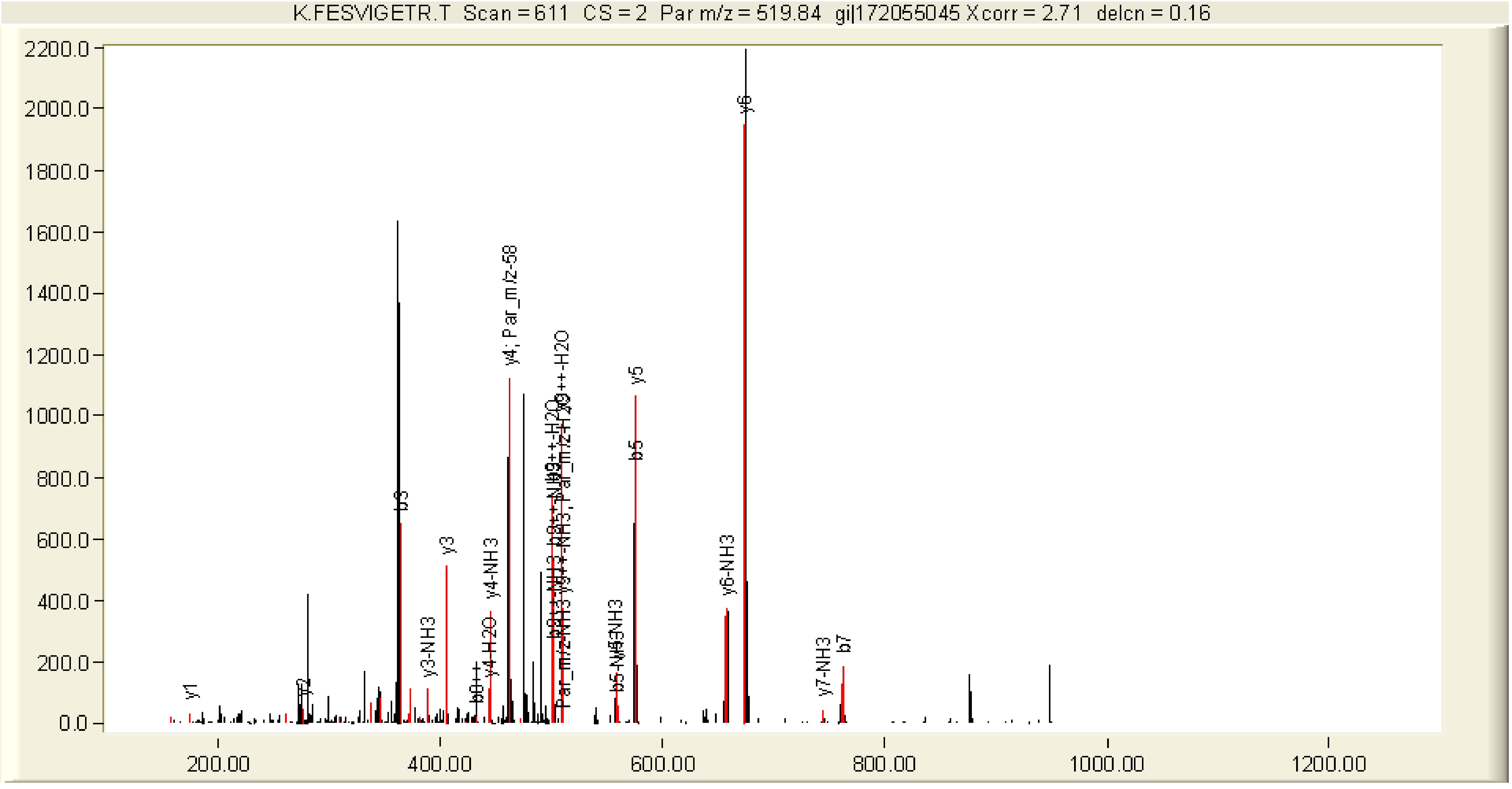

K.FESVIGETR.T Scan = 611 CS = 2 Par m/z = 519.84 gi|172055045 Xcorr = 2.71 delcn = 0.16
2200.0
2000.0
1800.0
1600.0
1400.0
1200.0
1000.0
800.0
600.0
400.0
200.0
0.0
200.00
400.00
600.00
800.00
1000.00
1200.00
y1
b3
y3-NH3
y3
y4-NH3
y4-H2O
y4; Par_m/z-58
y5
b5
y6-NH3
y6
y7-NH3
b7

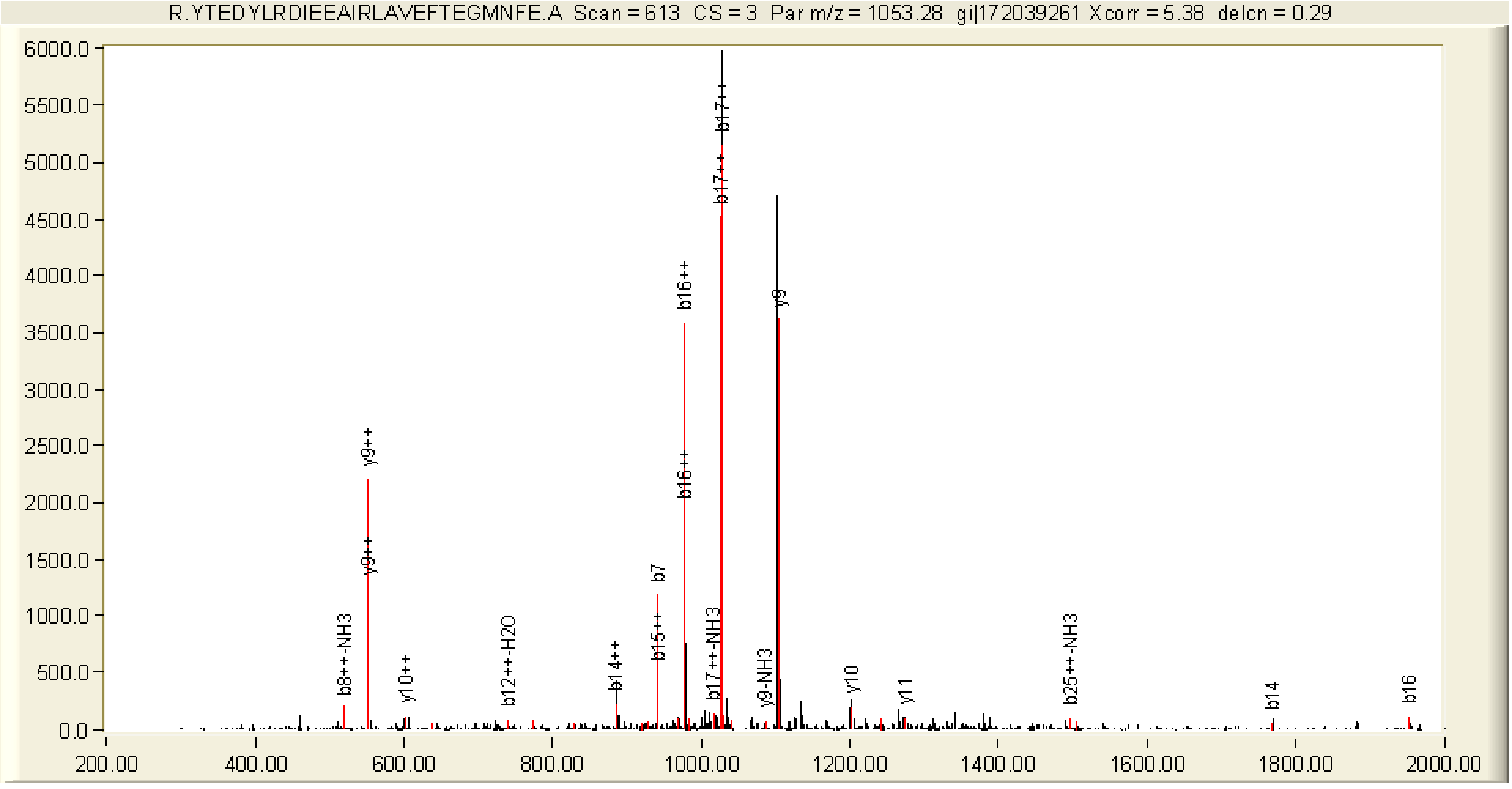
R.YTEDYLRDIEEAIRLAVEFTEGMNFE.A Scan = 613 CS = 3 Par m/z = 1053.28 gi|172039261 Xcorr = 5.38 delcn = 0.29
6000.0
5500.0
5000.0
4500.0
4000.0
3500.0
3000.0
2500.0
2000.0
1500.0
1000.0
500.0
0.0
200.00
400.00
600.00
800.00
1000.00
1200.00
1400.00
1600.00
1800.00
2000.00
b8++-NH3
y9++
y9++
y10++
b12++-H2O
b14++
b15++
b7
b16++
b16++
b17++-NH3
b17++
b17++
y9-NH3
y9
y10
y11
b25++-NH3
b14
b16

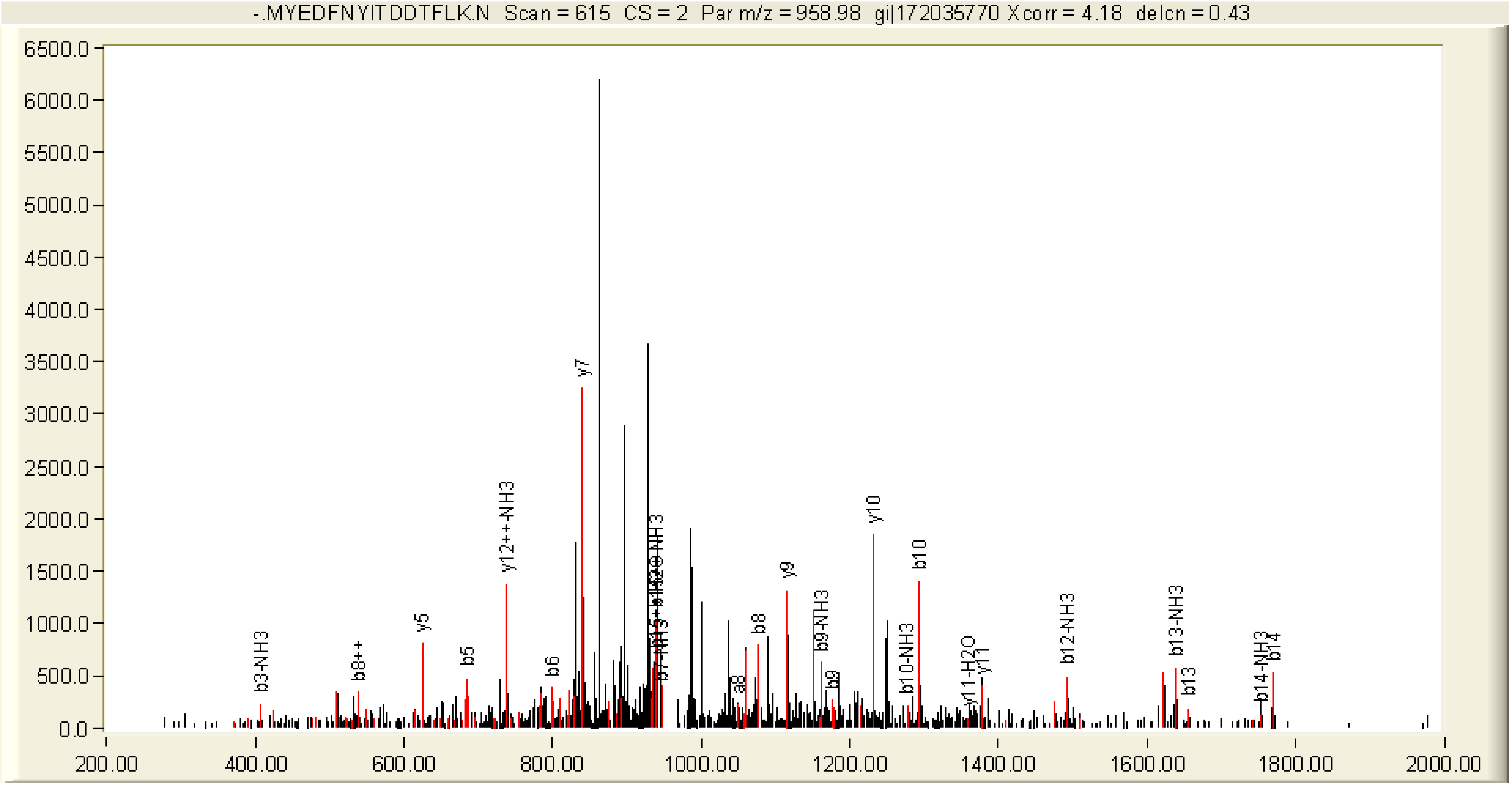
-.MYEDFNYITDDTFLK.N Scan = 615 CS = 2 Par m/z = 958.98 gi|172035770 Xcorr = 4.18 delcn = 0.43
6500.0
6000.0
5500.0
5000.0
4500.0
4000.0
3500.0
3000.0
2500.0
2000.0
1500.0
1000.0
500.0
0.0
200.00
400.00
600.00
800.00
1000.00
1200.00
1400.00
1600.00
1800.00
2000.00
b3-NH3
b8++
y5
b5
y12++-NH3
b6
y7
a8
b8
y9
b9-NH3
b9
y10
b10-NH3
b10
y11-H2O
y11
b12-NH3
b13-NH3
b13
b14-NH3
b14

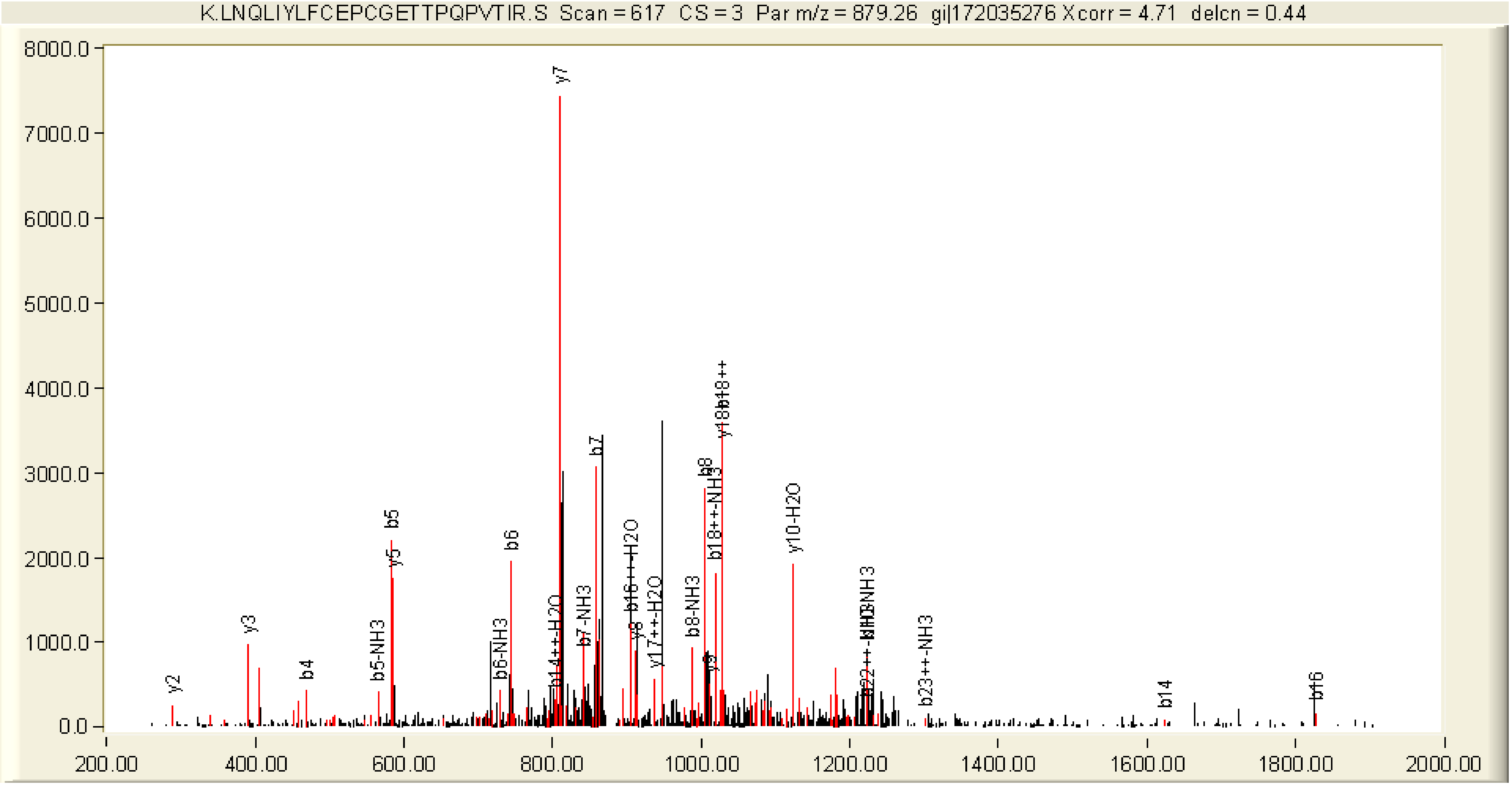

K.LNQLIYLFCEPCGETTPQPVTIR.S Scan = 617 CS = 3 Par m/z = 879.26 gi|172035276 Xcorr = 4.71 delcn = 0.44
8000.0
7000.0
6000.0
5000.0
4000.0
3000.0
2000.0
1000.0
0.0
200.00
400.00
600.00
800.00
1000.00
1200.00
1400.00
1600.00
1800.00
2000.00
y7
y2
y3
b4
b5-NH3
b5
y5
b6-NH3
b6
b7-NH3
b7
y17++-H2O
b8-NH3
b8
y10-H2O
b14
b16
b23++-NH3

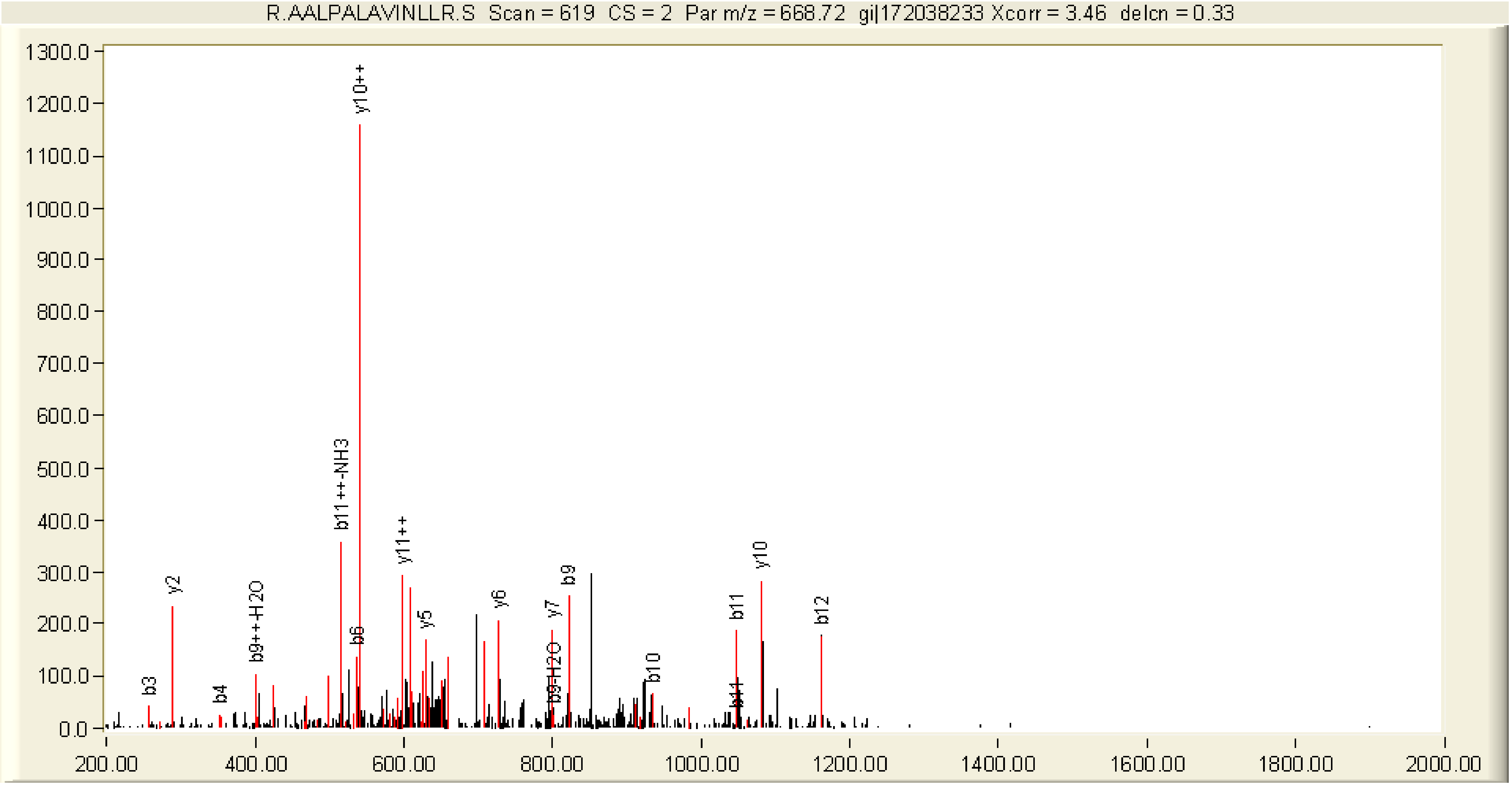
R.AALPALAVINLLR.S Scan = 619 CS = 2 Par m/z = 668.72 gi|172038233 Xcorr = 3.46 delcn = 0.33
1300.0
1200.0
1100.0
1000.0
900.0
800.0
700.0
600.0
500.0
400.0
300.0
200.0
100.0
0.0
200.00
400.00
600.00
800.00
1000.00
1200.00
1400.00
1600.00
1800.00
2000.00
y10++
b11++-NH3
y11++
y2
b9++-H2O
b3
b4
b6
y5
y6
y7
b9
b9-H2O
b10
b11
y10
b12

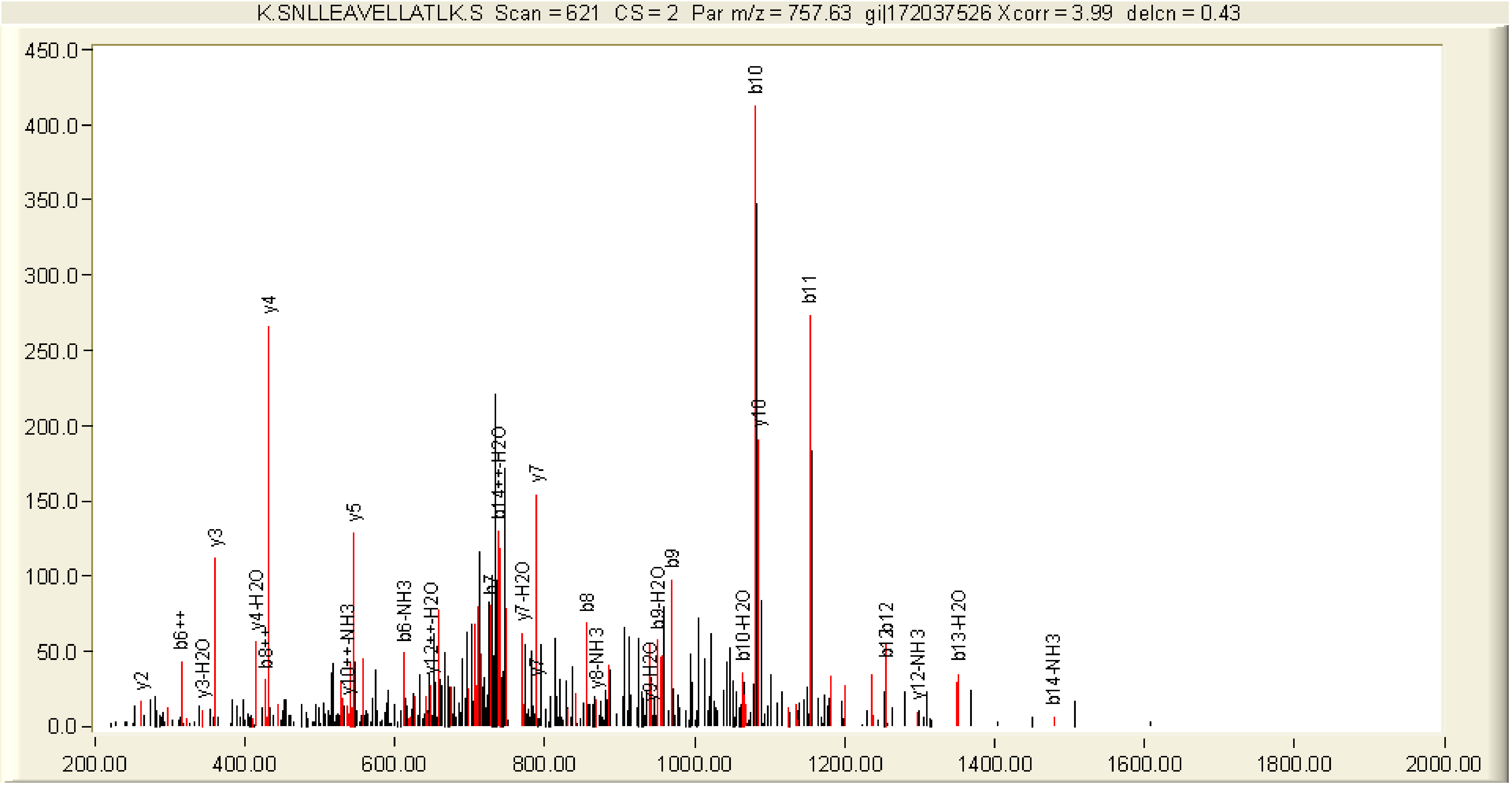
K.SNLLEAVELLATLK.S Scan = 621 CS = 2 Par m/z = 757.63 gi|172037526 Xcorr = 3.99 delcn = 0.43
b10
b11
y4
y10
b14++-H2O
y7
y5
y3
b9
y4-H2O
b7
y7-H2O
b8
b6-NH3
y12++-H2O
b9-H2O
b10-H2O
b13-H2O
b12
b6++
y8-NH3
y12-NH3
b14-NH3
y3-H2O
b8++
y10++-NH3
y2
0.0
50.0
100.0
150.0
200.0
250.0
300.0
350.0
400.0
450.0
200.00
400.00
600.00
800.00
1000.00
1200.00
1400.00
1600.00
1800.00
2000.00

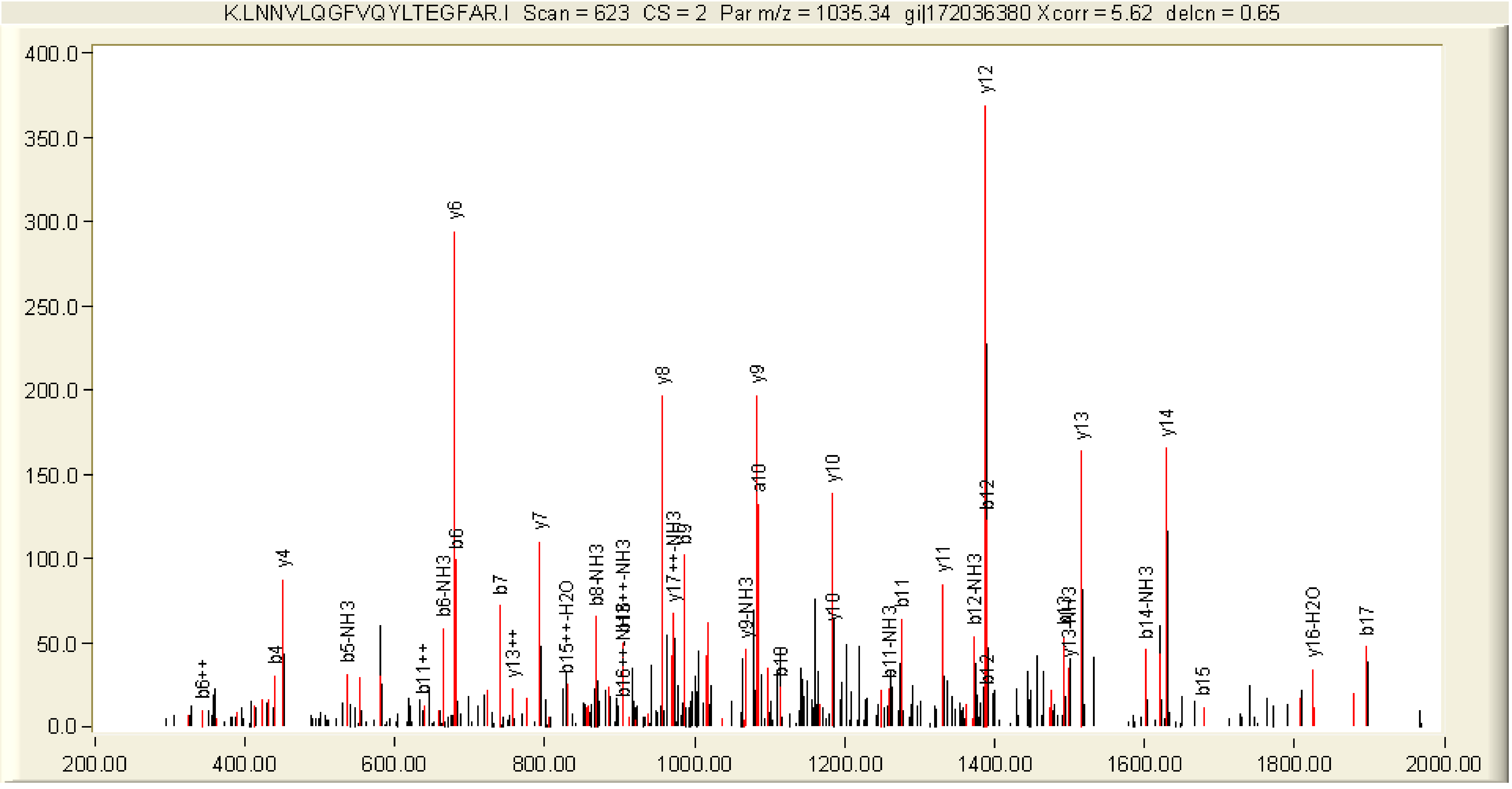

K.LNNVLQGFVQYLTEGFAR.I Scan = 623 CS = 2 Par m/z = 1035.34 gi|172036380 Xcorr = 5.62 delcn = 0.65
400.0
350.0
300.0
250.0
200.0
150.0
100.0
50.0
0.0
200.00
400.00
600.00
800.00
1000.00
1200.00
1400.00
1600.00
1800.00
2000.00
y12
y6
y8
y9
y13
y14
a10
y10
b12
y7
b6
y4
b6-NH3
b8-NH3
b7
b15++-H2O
y9-NH3
b11
y11
b12-NH3
b14-NH3
y16-H2O
b17
b5-NH3
b4
b6++
b11++
y13++
b10
b11-NH3
b15

R.IAMLQLLIDNIDPPPHNIYLSK.K Scan = 625 CS = 3 Par m/z = 840.72 gi|172037671 Xcorr = 3.53 delcn = 0.23

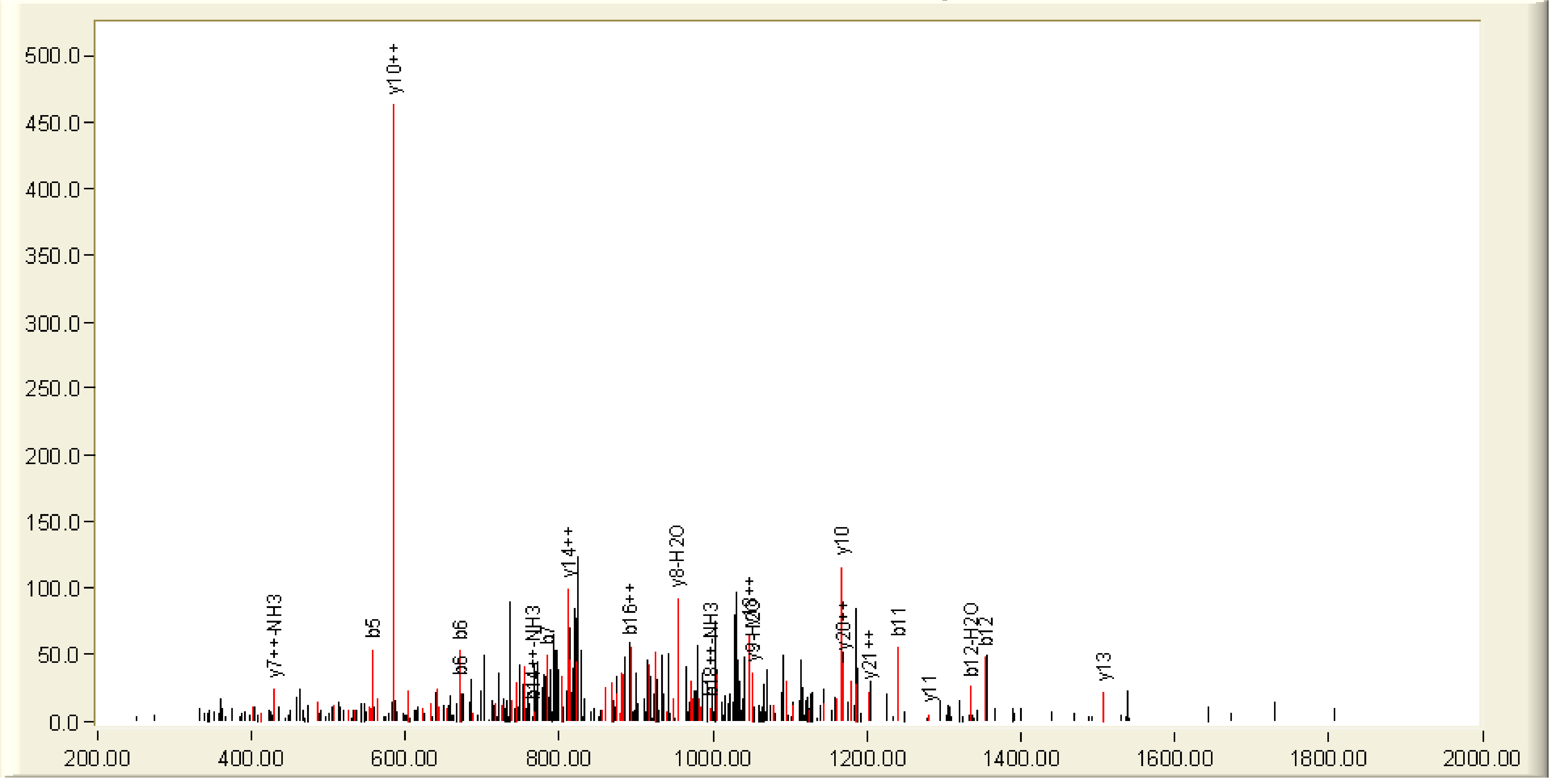

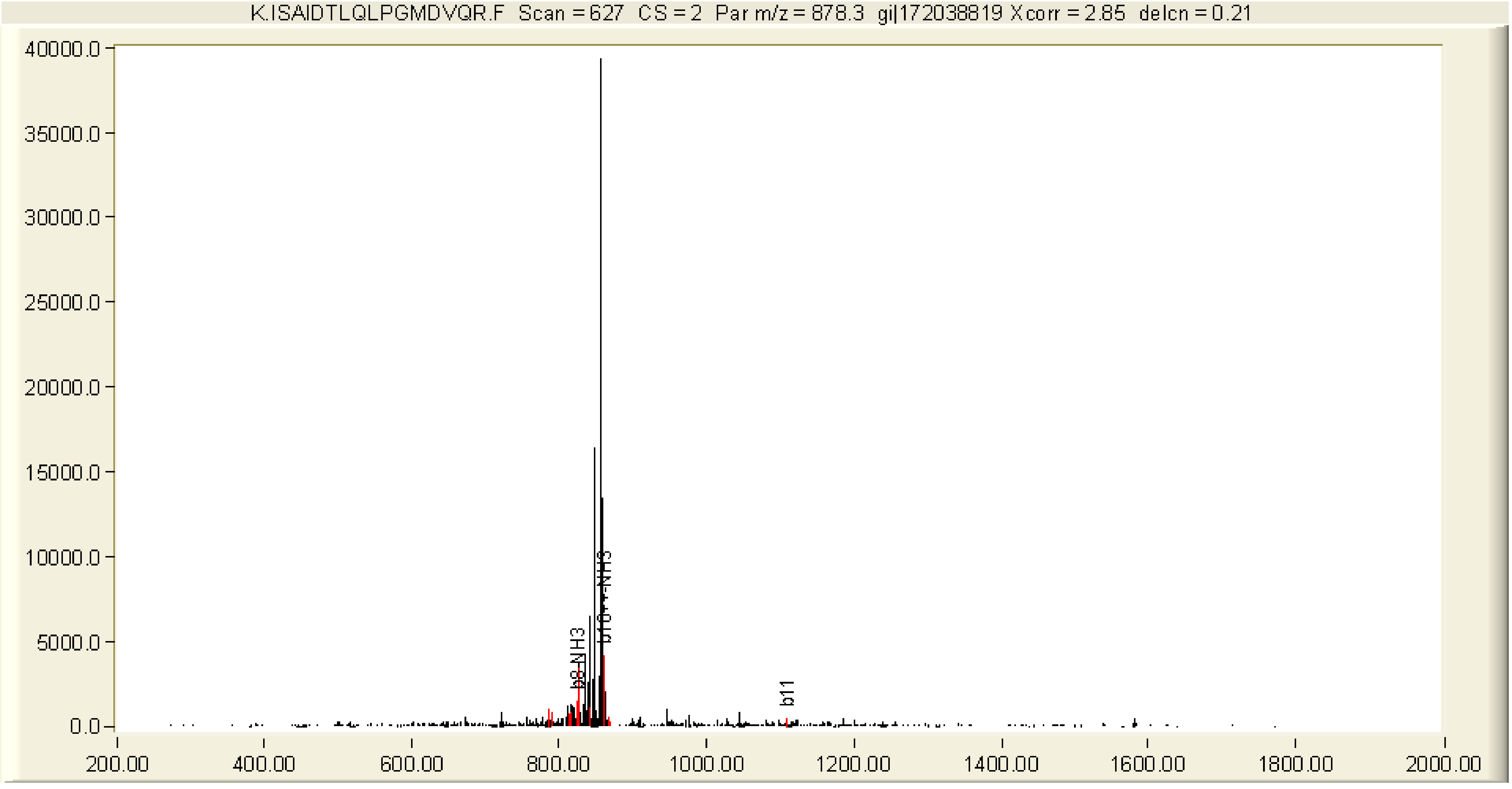
K.ISAIDTLQLPGMDVQR.F Scan = 627 CS = 2 Par m/z = 878.3 gi|172038819 Xcorr = 2.85 delcn = 0.21
40000.0
35000.0
30000.0
25000.0
20000.0
15000.0
10000.0
5000.0
0.0
b8-NH3
b16++-NH3
b11
200.00
400.00
600.00
800.00
1000.00
1200.00
1400.00
1600.00
1800.00
2000.00

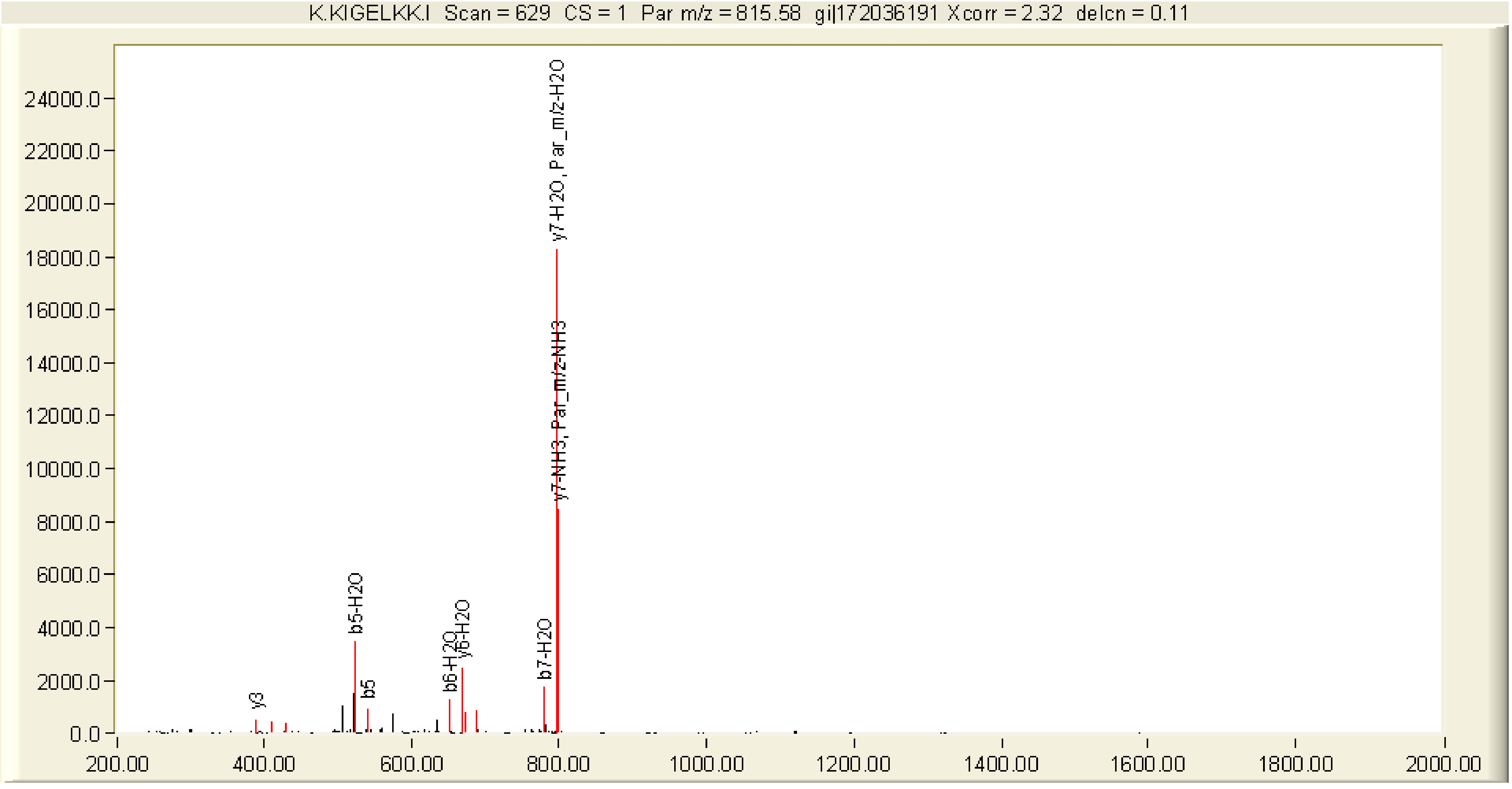
K.KIGELKK.I Scan = 629 CS = 1 Par m/z = 815.58 gi|172036191 Xcorr = 2.32 delcn = 0.11
y7-H2O, Par_m/z-H2O
y7-NH3, Par_m/z-NH3
b5-H2O
b5
y3
b6-H2O
y6-H2O
b7-H2O
24000.0
22000.0
20000.0
18000.0
16000.0
14000.0
12000.0
10000.0
8000.0
6000.0
4000.0
2000.0
0.0
200.00
400.00
600.00
800.00
1000.00
1200.00
1400.00
1600.00
1800.00
2000.00

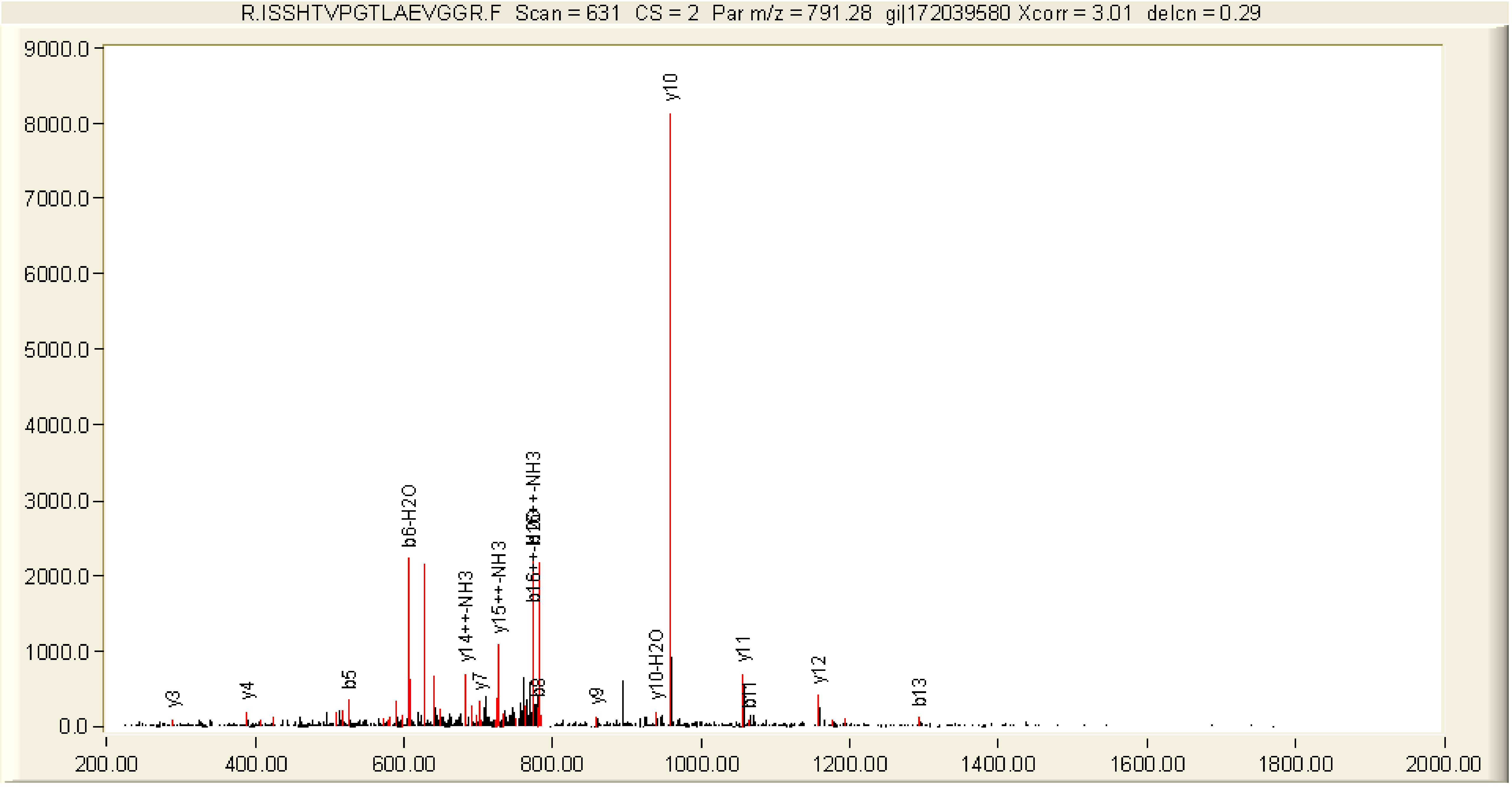
R.ISSHTVPGTLAEVGGR.F Scan = 631 CS = 2 Par m/z = 791.28 gi|172039580 Xcorr = 3.01 delcn = 0.29
9000.0
8000.0
7000.0
6000.0
5000.0
4000.0
3000.0
2000.0
1000.0
0.0
y10
b6-H2O
y14++-NH3
y15++-NH3
y7
y3
y4
b5
b8
y9
y10-H2O
y11
b11
y12
b13
200.00
400.00
600.00
800.00
1000.00
1200.00
1400.00
1600.00
1800.00
2000.00

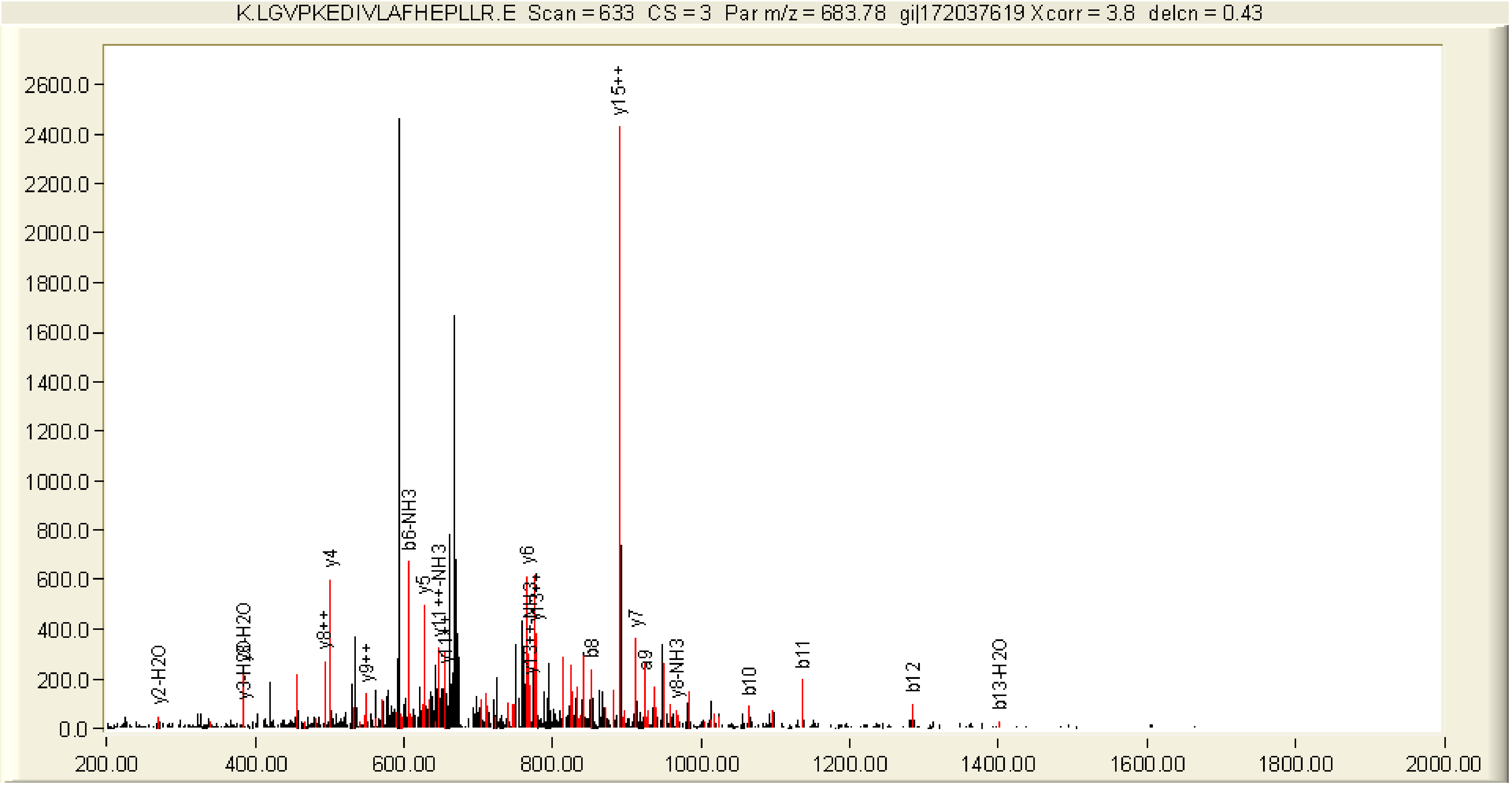
K.LGVPKEDIVLAFHEPLLR.E Scan = 633 CS = 3 Par m/z = 683.78 gi|172037619 Xcorr = 3.8 delcn = 0.43
2600.0
2400.0
2200.0
2000.0
1800.0
1600.0
1400.0
1200.0
1000.0
800.0
600.0
400.0
200.0
0.0
200.00
400.00
600.00
800.00
1000.00
1200.00
1400.00
1600.00
1800.00
2000.00
y15++
b6-NH3
y4
y5
y8++
y9++
y2-H2O
y6
y7
a9
b8
y8-NH3
b10
b11
b12
b13-H2O

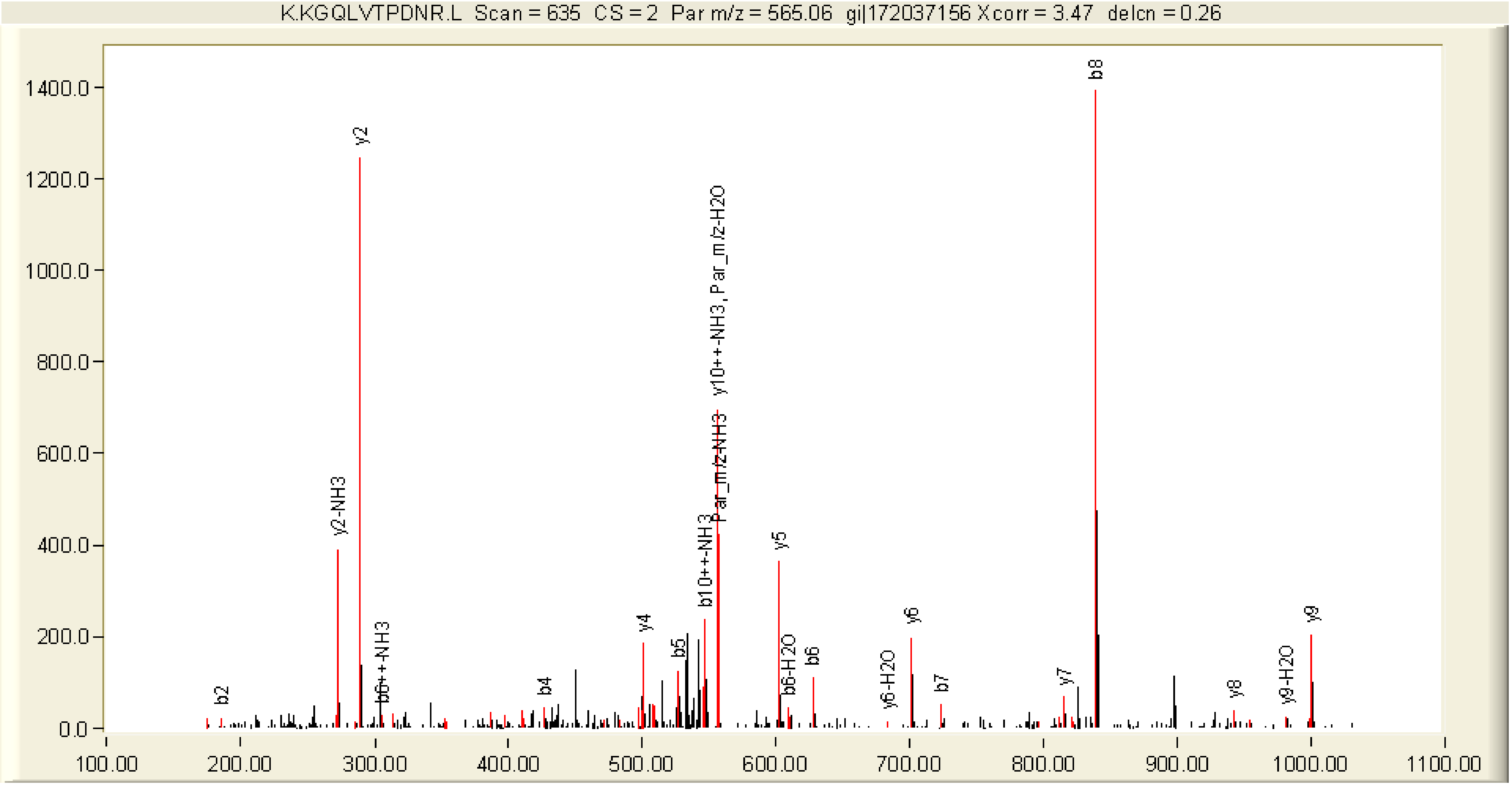
K.KGQLVTPDNR.L Scan = 635 CS = 2 Par m/z = 565.06 gi|172037156 Xcorr = 3.47 delcn = 0.26
1400.0
1200.0
1000.0
800.0
600.0
400.0
200.0
0.0
100.00
200.00
300.00
400.00
500.00
600.00
700.00
800.00
900.00
1000.00
1100.00
b2
y2-NH3
y2
b6++-NH3
b4
y4
b5
b10++-NH3
Par_m/z-NH3
y10++-NH3, Par_m/z-H2O
y5
b6-H2O
b6
y6-H2O
y6
b7
y7
b8
y8
y9-H2O
y9

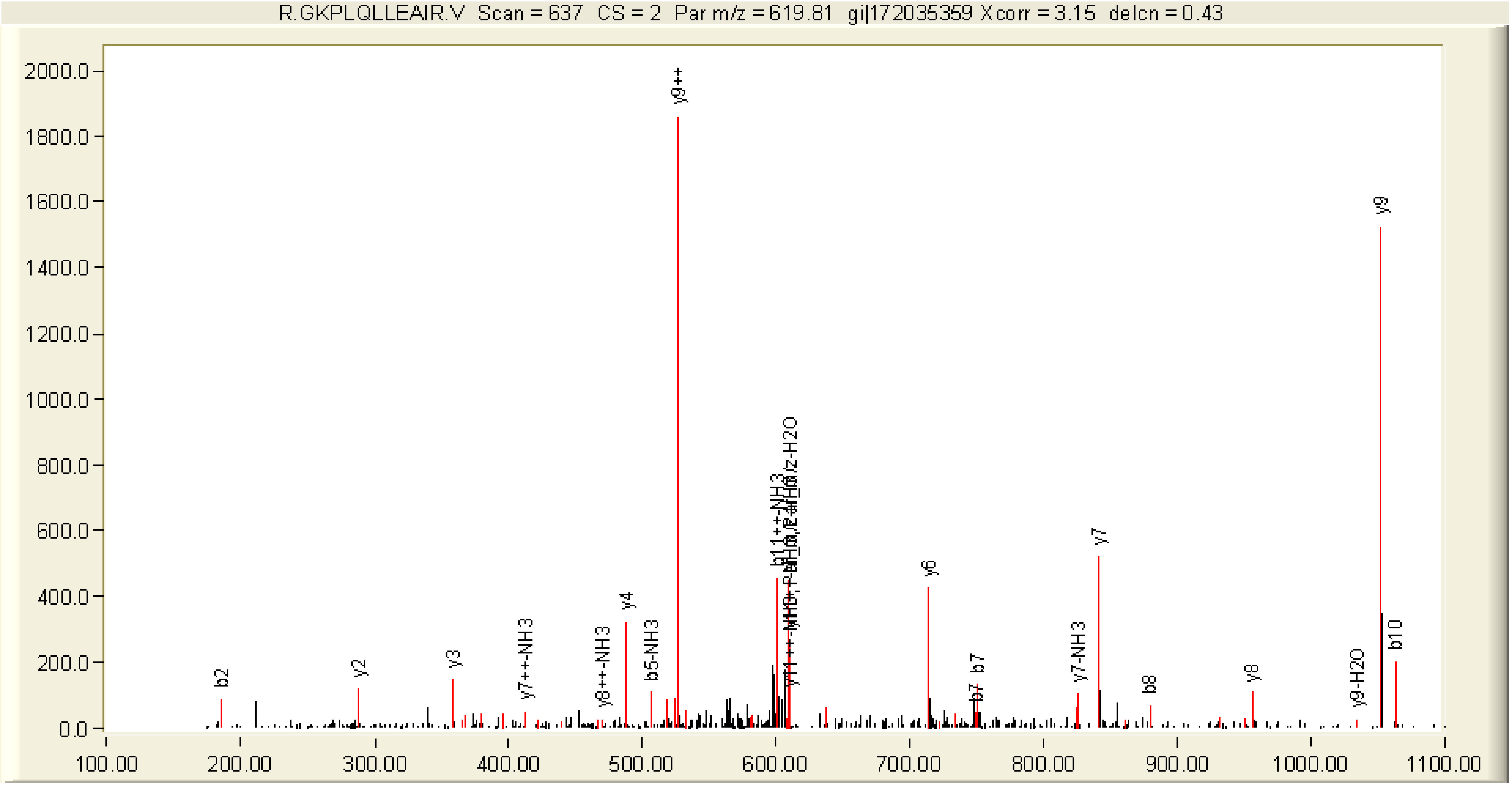
R.GKPLQLLEAIR.V Scan = 637 CS = 2 Par m/z = 619.81 gi|172035359 Xcorr = 3.15 delcn = 0.43
2000.0
1800.0
1600.0
1400.0
1200.0
1000.0
800.0
600.0
400.0
200.0
0.0
100.00
200.00
300.00
400.00
500.00
600.00
700.00
800.00
900.00
1000.00
1100.00
b2
y2
y3
y7++-NH3
y8++-NH3
y4
b5-NH3
y9++
y6
b7
y7-NH3
y7
b8
y8
y9-H2O
y9
b10

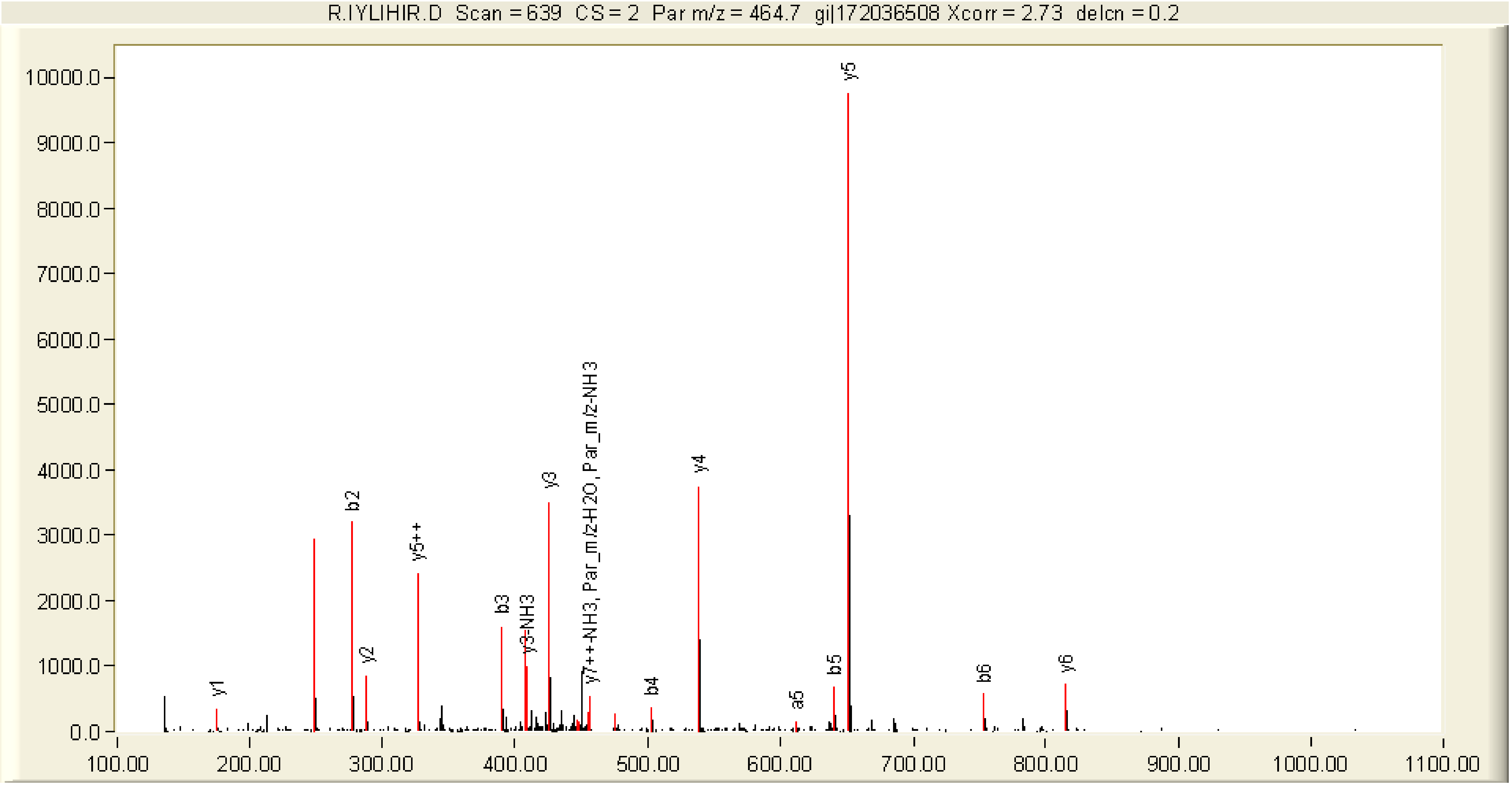

R.IYLIHIR.D Scan = 639 CS = 2 Par m/z = 464.7 gi|172036508 Xcorr = 2.73 delcn = 0.2
10000.0
9000.0
8000.0
7000.0
6000.0
5000.0
4000.0
3000.0
2000.0
1000.0
0.0
100.00
200.00
300.00
400.00
500.00
600.00
700.00
800.00
900.00
1000.00
1100.00
y1
b2
y2
y5++
b3
y3-NH3
y3
y7++-NH3, Par_m/z-H2O, Par_m/z-NH3
b4
y4
a5
b5
y5
b6
y6

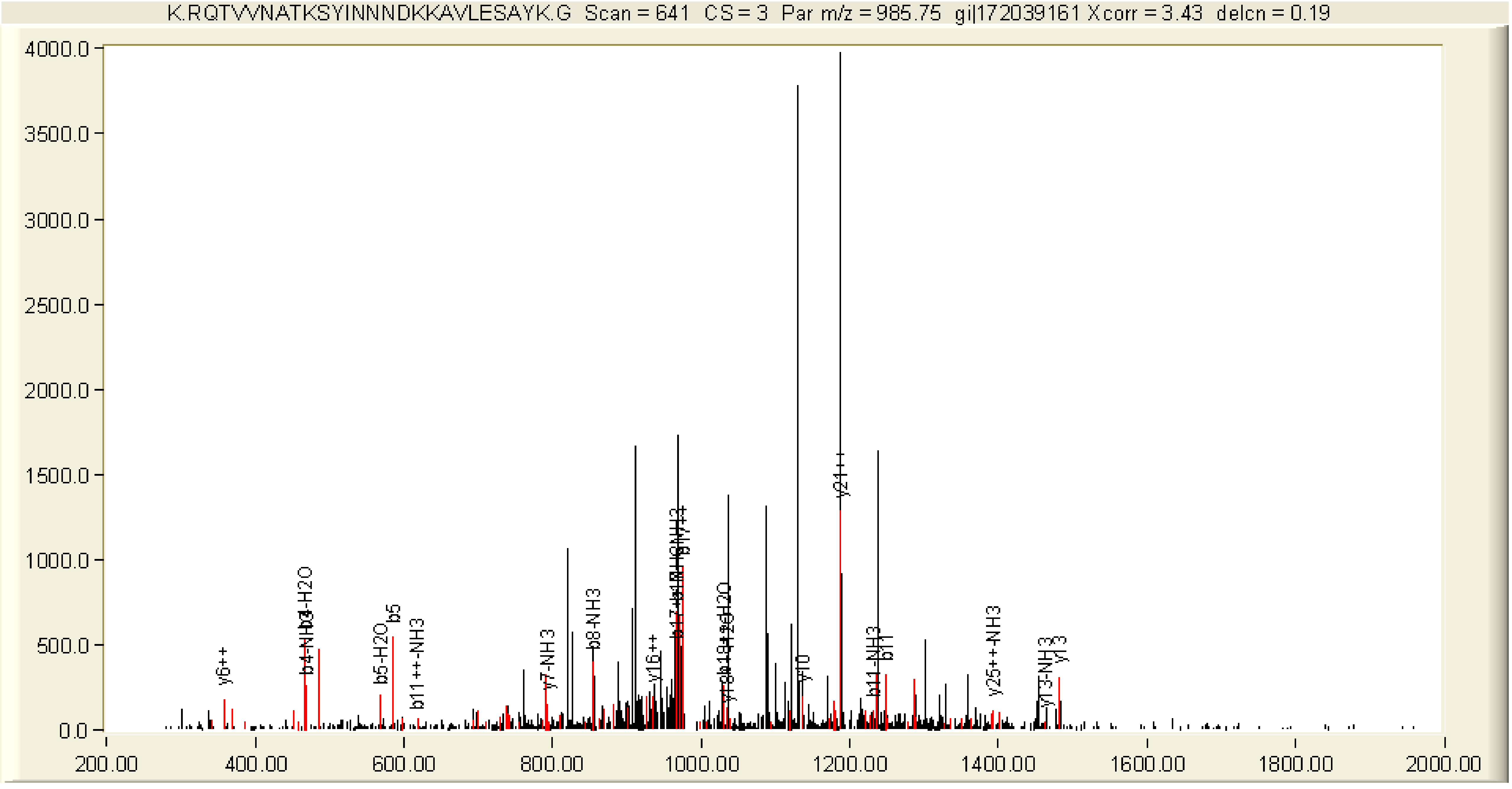
K.RQTVVNATKSYINNNDKKAVLESAYK.G Scan = 641 CS = 3 Par m/z = 985.75 gi|172039161 Xcorr = 3.43 delcn = 0.19
4000.0
3500.0
3000.0
2500.0
2000.0
1500.0
1000.0
500.0
0.0
200.00
400.00
600.00
800.00
1000.00
1200.00
1400.00
1600.00
1800.00
2000.00
y6++
b4-H2O
b5-H2O
b5
b11++-NH3
y7-NH3
b8-NH3
y16++
y21++
y10
b11-NH3
b11
y25++-NH3
y13-NH3
y13

K.TYQNGIQALHEISLTIDCGIFGLLGPNGAGK.S Scan = 643 CS = 3 Par m/z = 1068.75 gi|172037969 Xcorr = 3.25 delcn = 0.14

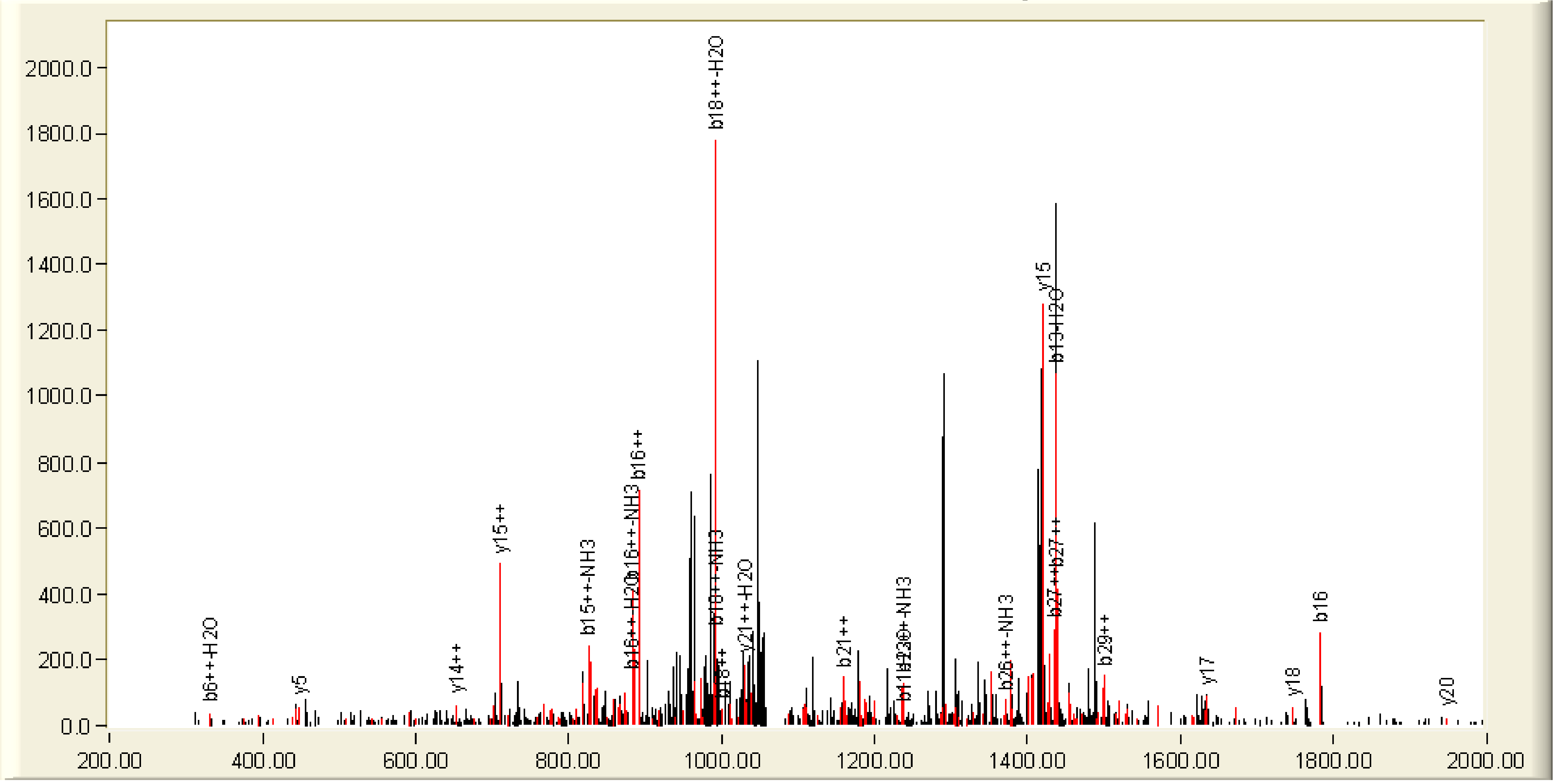

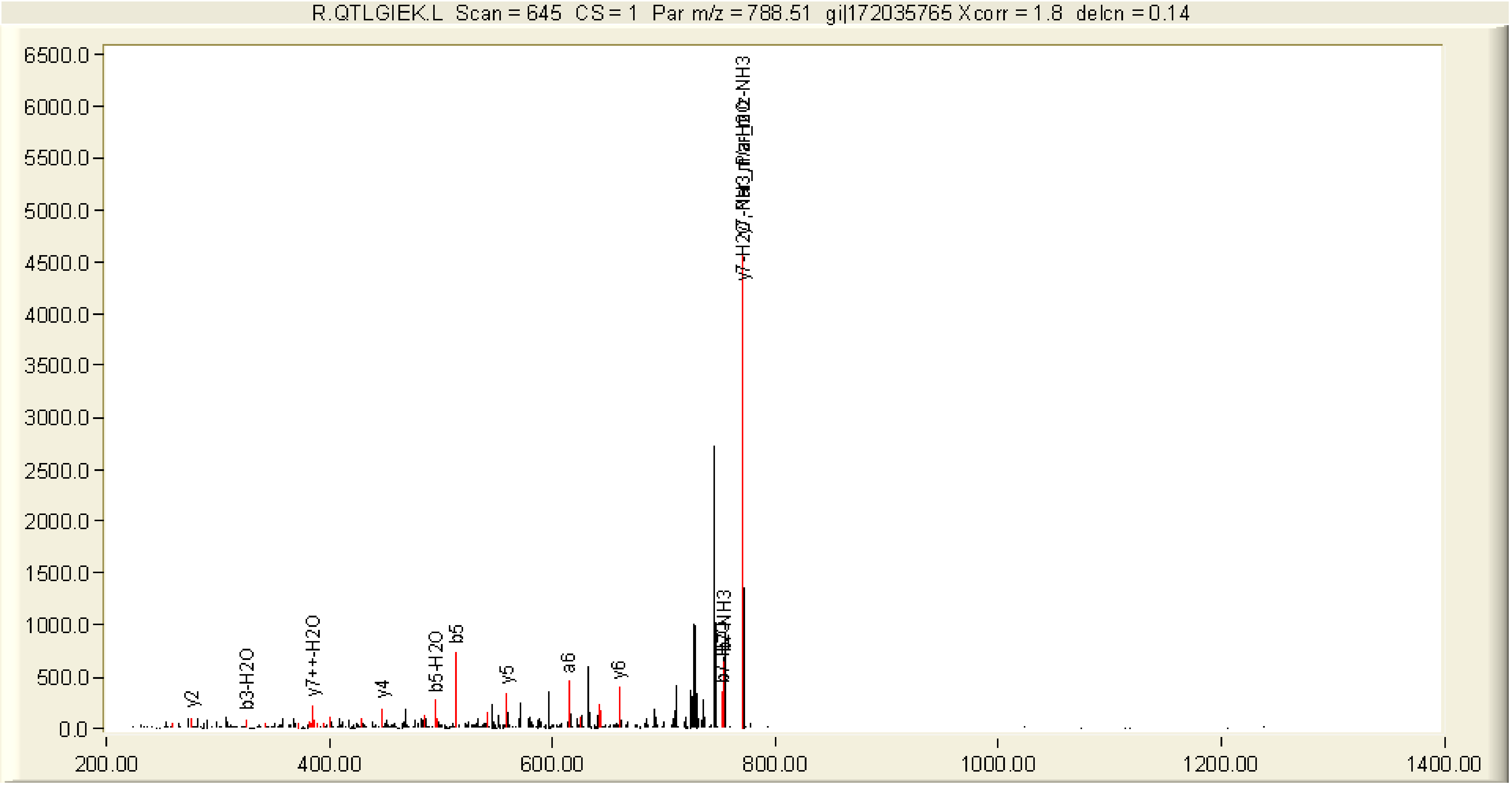

R.QTLGIEK.L Scan = 645 CS = 1 Par m/z = 788.51 gi|172035765 Xcorr = 1.8 delcn = 0.14
6500.0
6000.0
5500.0
5000.0
4500.0
4000.0
3500.0
3000.0
2500.0
2000.0
1500.0
1000.0
500.0
0.0
200.00
400.00
600.00
800.00
1000.00
1200.00
1400.00
y2
b3-H2O
y7++-H2O
y4
b5-H2O
b5
y5
a6
y6

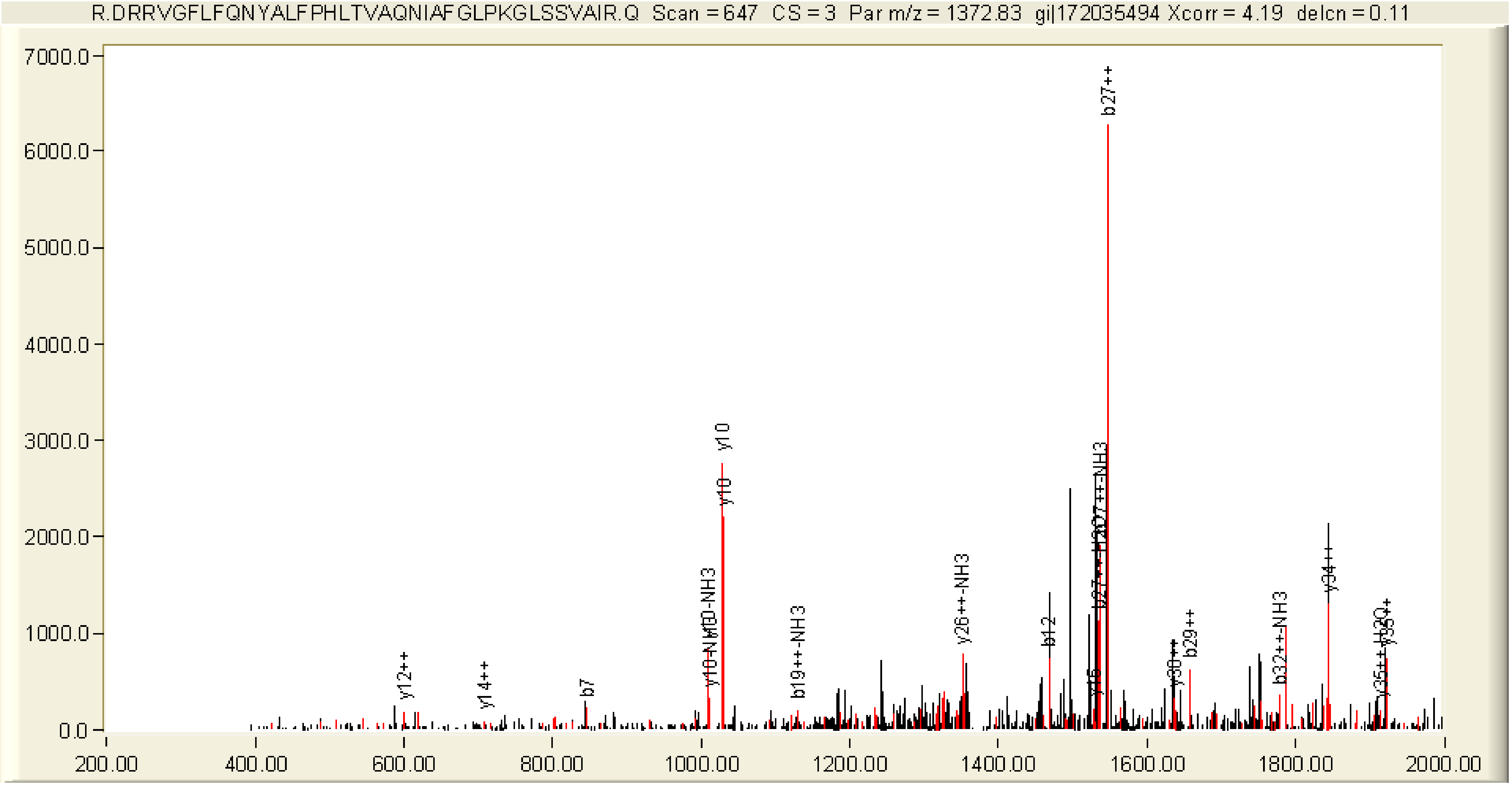
R.DRRVGFLFQNYALFPHLTVAQNIAFGLPKGLSSVAIR.Q Scan = 647 CS = 3 Par m/z = 1372.83 gi|172035494 Xcorr = 4.19 delcn = 0.11
7000.0
6000.0
5000.0
4000.0
3000.0
2000.0
1000.0
0.0
200.00
400.00
600.00
800.00
1000.00
1200.00
1400.00
1600.00
1800.00
2000.00
b27++
y10
y12++
y14++
b7
y10-NH3
b19++-NH3
y26++-NH3
b12
b29++
y34++
b32++-NH3

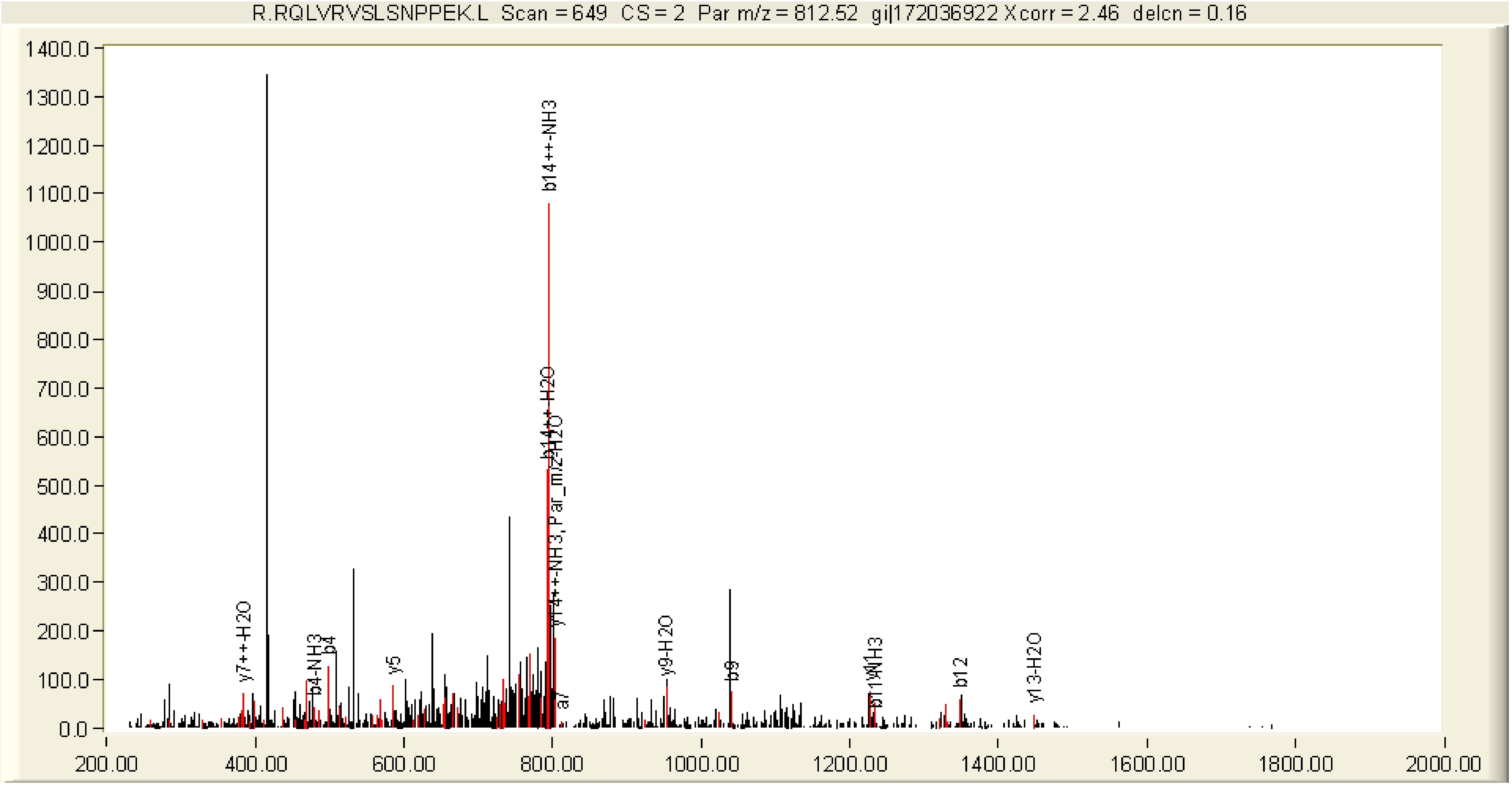
R.RQLVRVSLSNPPEK.L Scan = 649 CS = 2 Par m/z = 812.52 gi|172036922 Xcorr = 2.46 delcn = 0.16
b14++-NH3
b14++-H2O
y14++-NH3, Par_m/z-H2O
y7++-H2O
b4-NH3
b4
y5
a7
y9-H2O
b9
b12
y13-H2O
1400.0
1300.0
1200.0
1100.0
1000.0
900.0
800.0
700.0
600.0
500.0
400.0
300.0
200.0
100.0
0.0
200.00
400.00
600.00
800.00
1000.00
1200.00
1400.00
1600.00
1800.00
2000.00

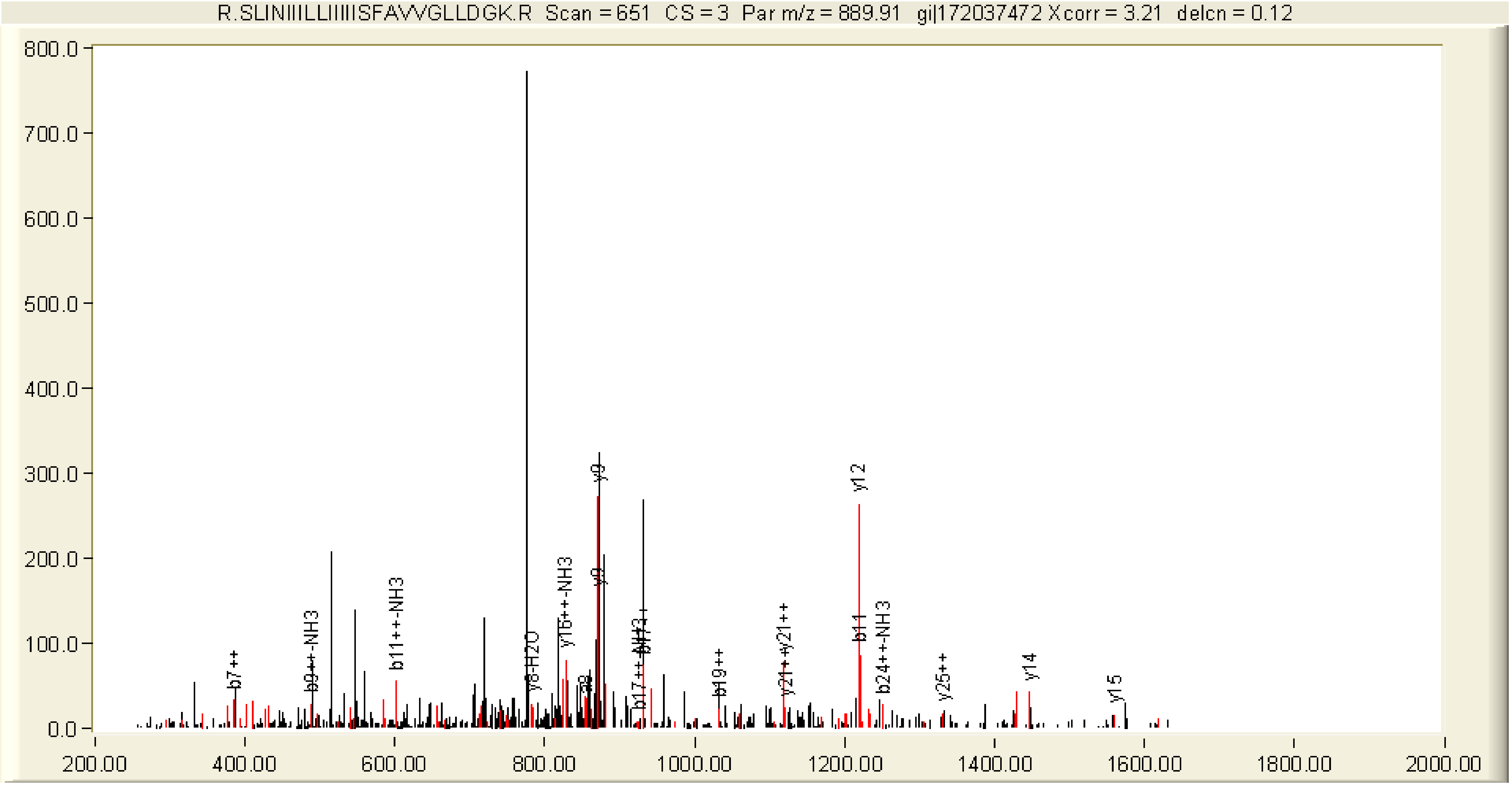
R.SLINIIIILLIIIIISFAVVGLLDGK.R Scan = 651 CS = 3 Par m/z = 889.91 gi|172037472 Xcorr = 3.21 delcn = 0.12
800.0
700.0
600.0
500.0
400.0
300.0
200.0
100.0
0.0
200.00
400.00
600.00
800.00
1000.00
1200.00
1400.00
1600.00
1800.00
2000.00
b7++
b11++-NH3
y16++-NH3
y9
y12
b19++
y21++
b11
b24++-NH3
y25++
y14
y15

K.EAFVSEEVSVKKEVDR.D Scan = 653 CS = 2 Par m/z = 925.24 gi|172039561 Xcorr = 3.49 delcn = 0.2

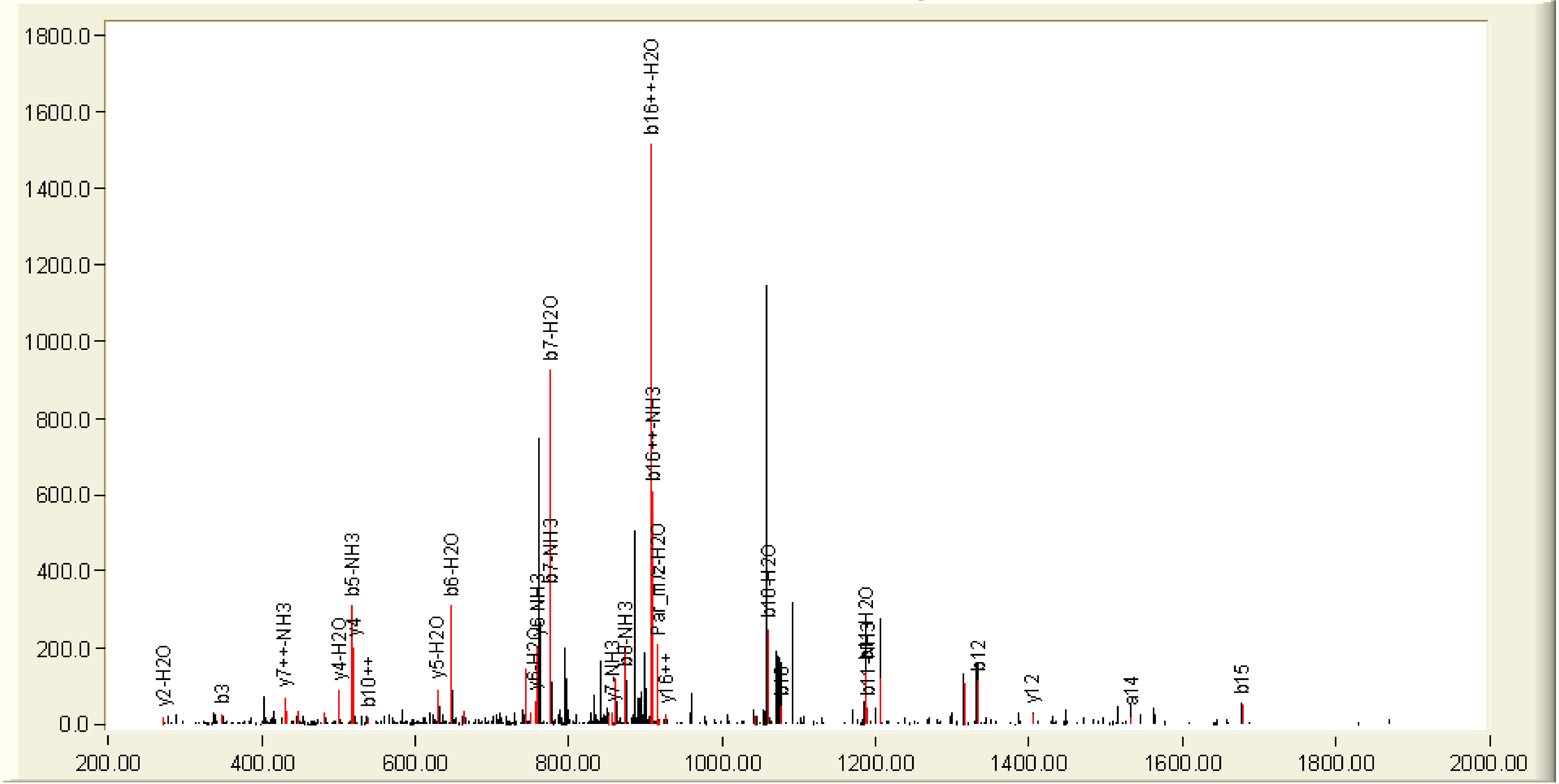

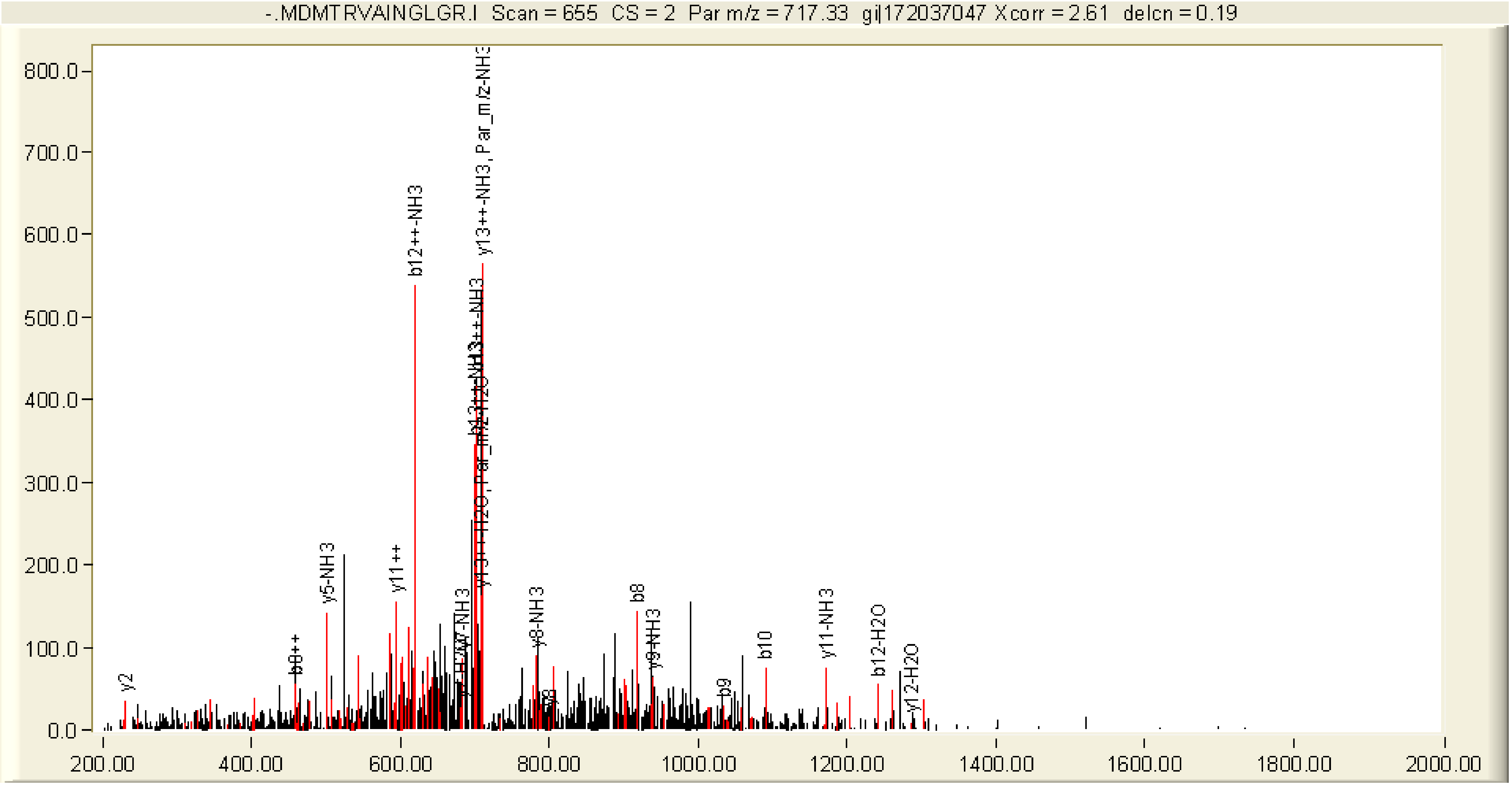

-.MDMTRVAINGLGR.I Scan = 655 CS = 2 Par m/z = 717.33 gi|172037047 Xcorr = 2.61 delcn = 0.19
800.0
700.0
600.0
500.0
400.0
300.0
200.0
100.0
0.0
200.00
400.00
600.00
800.00
1000.00
1200.00
1400.00
1600.00
1800.00
2000.00
y2
y5-NH3
y11++
b12++-NH3
y13++-NH3, Par_m/z-NH3
y8-NH3
b8
y9-NH3
b9
b10
y11-NH3
b12-H2O
y12-H2O

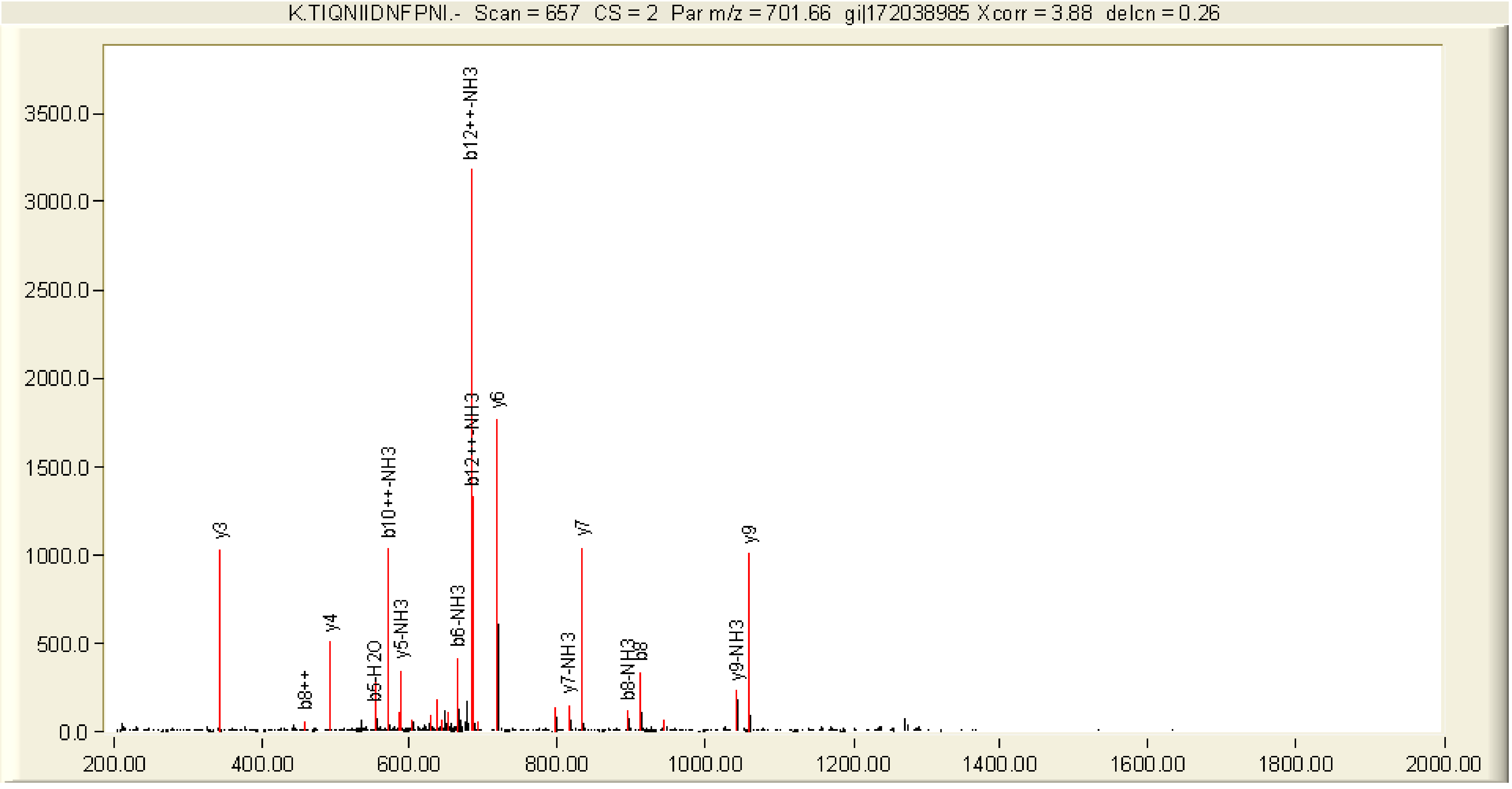

K.TIQNIIDNFPNI.- Scan = 657 CS = 2 Par m/z = 701.66 gi|172038985 Xcorr = 3.88 delcn = 0.26
3500.0
3000.0
2500.0
2000.0
1500.0
1000.0
500.0
0.0
200.00
400.00
600.00
800.00
1000.00
1200.00
1400.00
1600.00
1800.00
2000.00
b12++-NH3
y6
b12++-NH3
b10++-NH3
y3
y7
y9
y4
b8++
b5-H2O
y5-NH3
b6-NH3
y7-NH3
b8-NH3
b8
y9-NH3

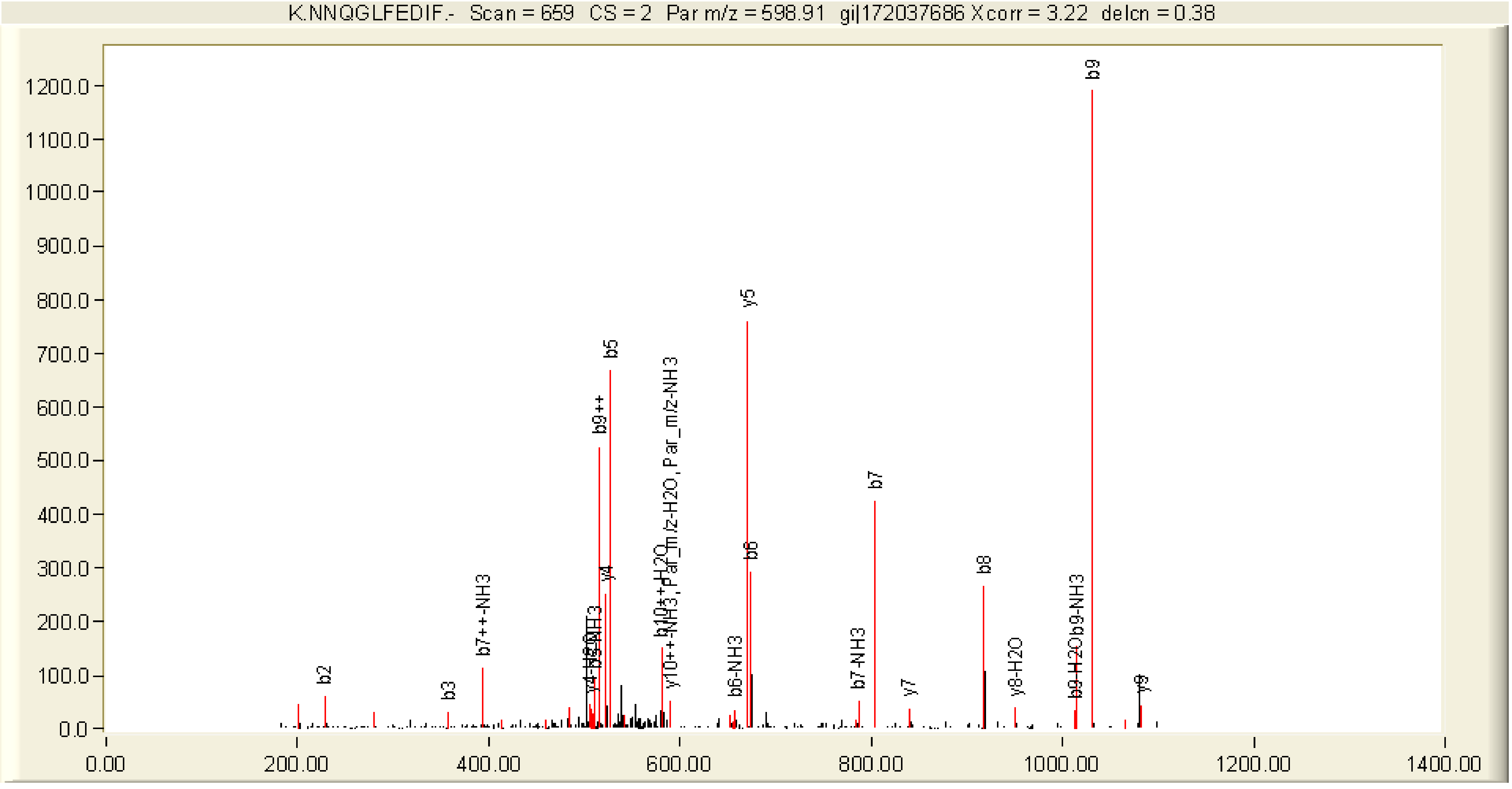

K.NNQGLFEDIF.- Scan = 659 CS = 2 Par m/z = 598.91 gi|172037686 Xcorr = 3.22 delcn = 0.38
1200.0
1100.0
1000.0
900.0
800.0
700.0
600.0
500.0
400.0
300.0
200.0
100.0
0.0
0.00
200.00
400.00
600.00
800.00
1000.00
1200.00
1400.00
b2
b3
b7++-NH3
b9++
b5
y4
y10++-H2O, Par_m/z-H2O, Par_m/z-NH3
y5
b6
b6-NH3
b7-NH3
b7
y7
b8
y8-H2O
b9-H2O
b9-NH3
b9

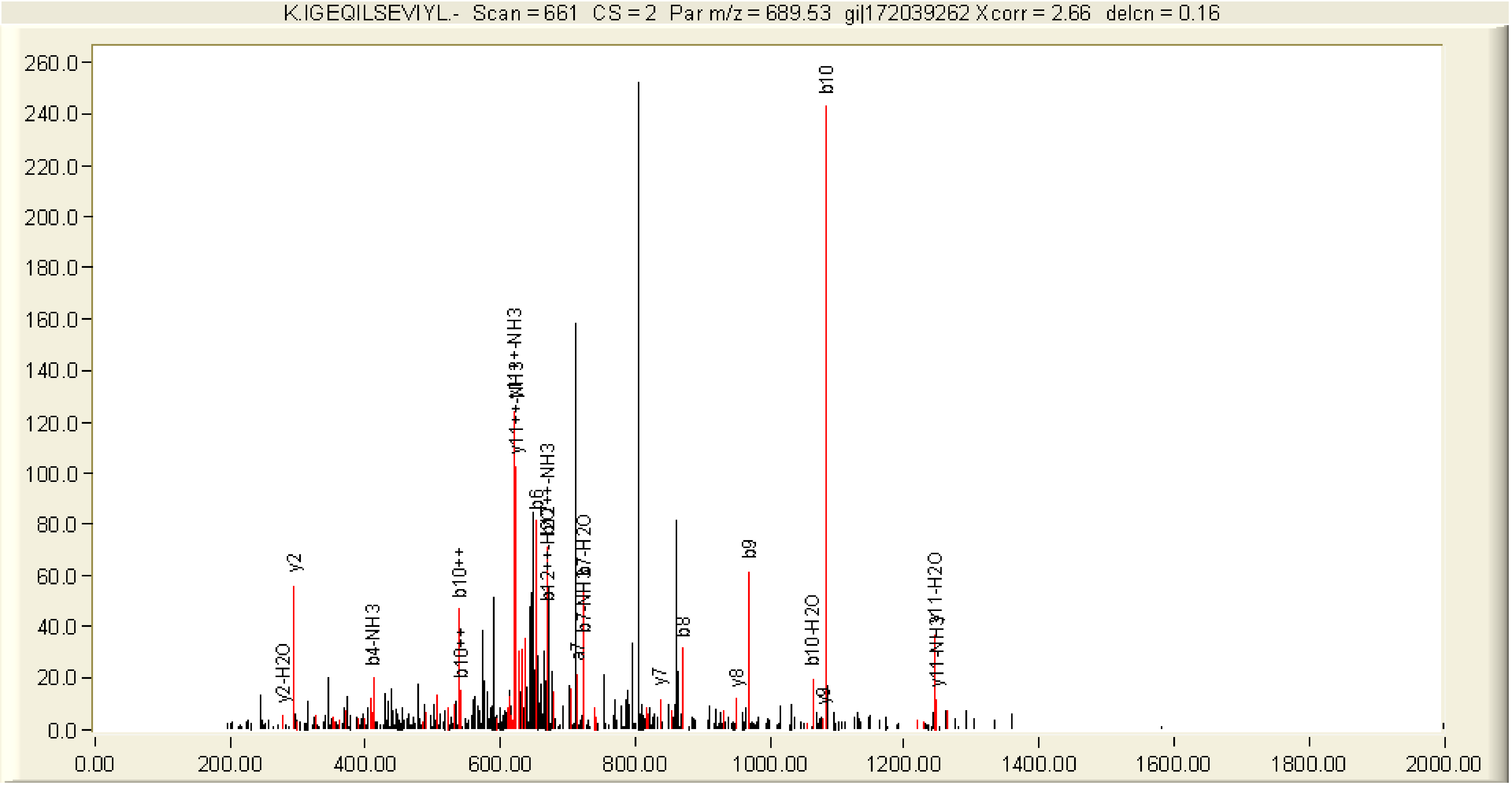
K.IGEQILSEVIYL.- Scan = 661 CS = 2 Par m/z = 689.53 gi|172039262 Xcorr = 2.66 delcn = 0.16
260.0
240.0
220.0
200.0
180.0
160.0
140.0
120.0
100.0
80.0
60.0
40.0
20.0
0.0
0.00
200.00
400.00
600.00
800.00
1000.00
1200.00
1400.00
1600.00
1800.00
2000.00
b10
y2
y2-H2O
b4-NH3
b10++
b6
b7-H2O
a7
b8
b9
y7
y8
b10-H2O
y11-H2O
y11-NH3

K.ELTTTEEAVKGVYDAKSYYK.I Scan = 663 CS = 3 Par m/z = 765.62 gi|172035343 Xcorr = 3.2 delcn = 0.12

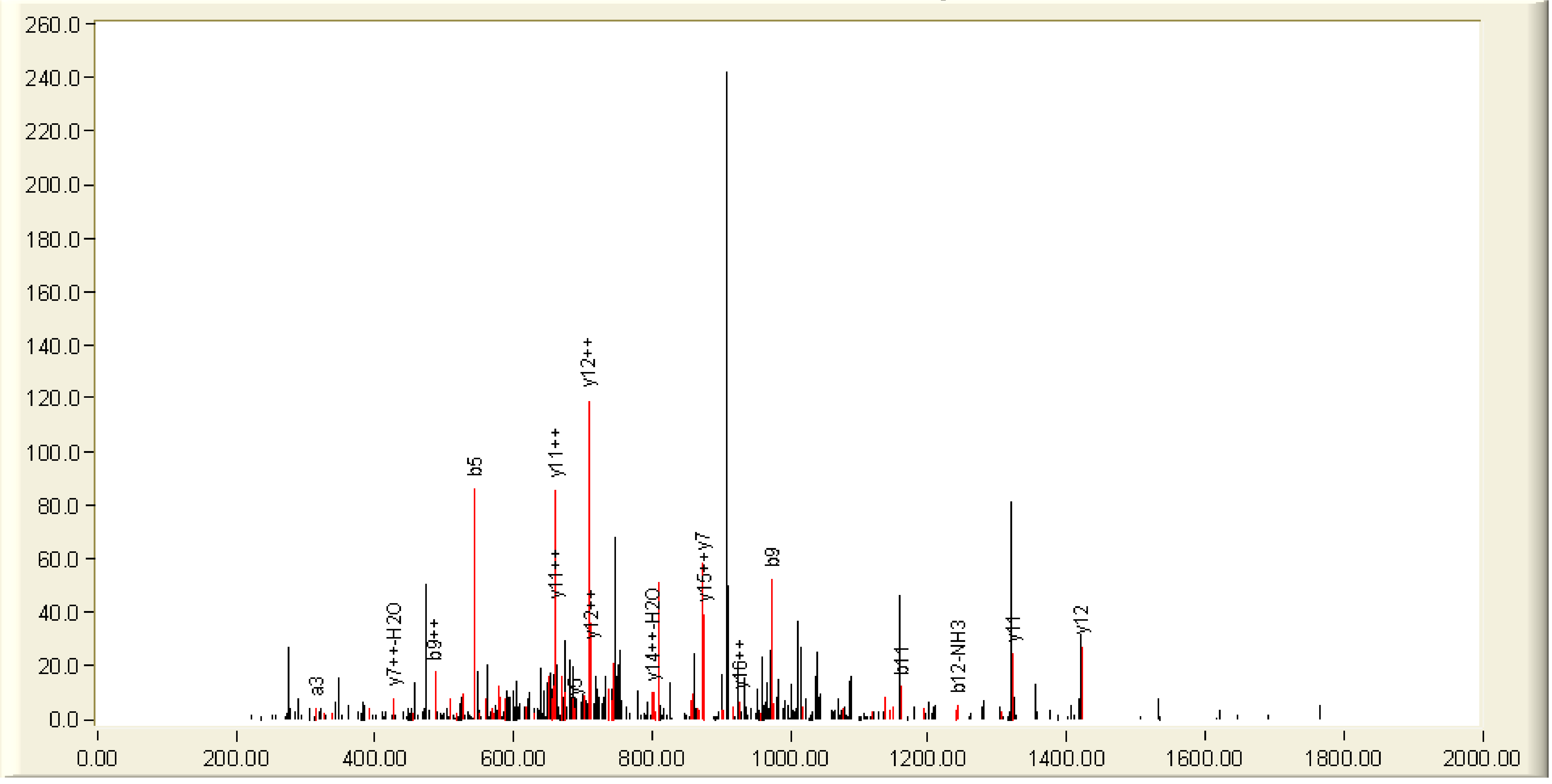

K.ALPDLQLEPLVEEEGNADH.- Scan = 665 CS = 2 Par m/z = 1045.81 gi|172034870 Xcorr = 3.75 delcn = 0.37

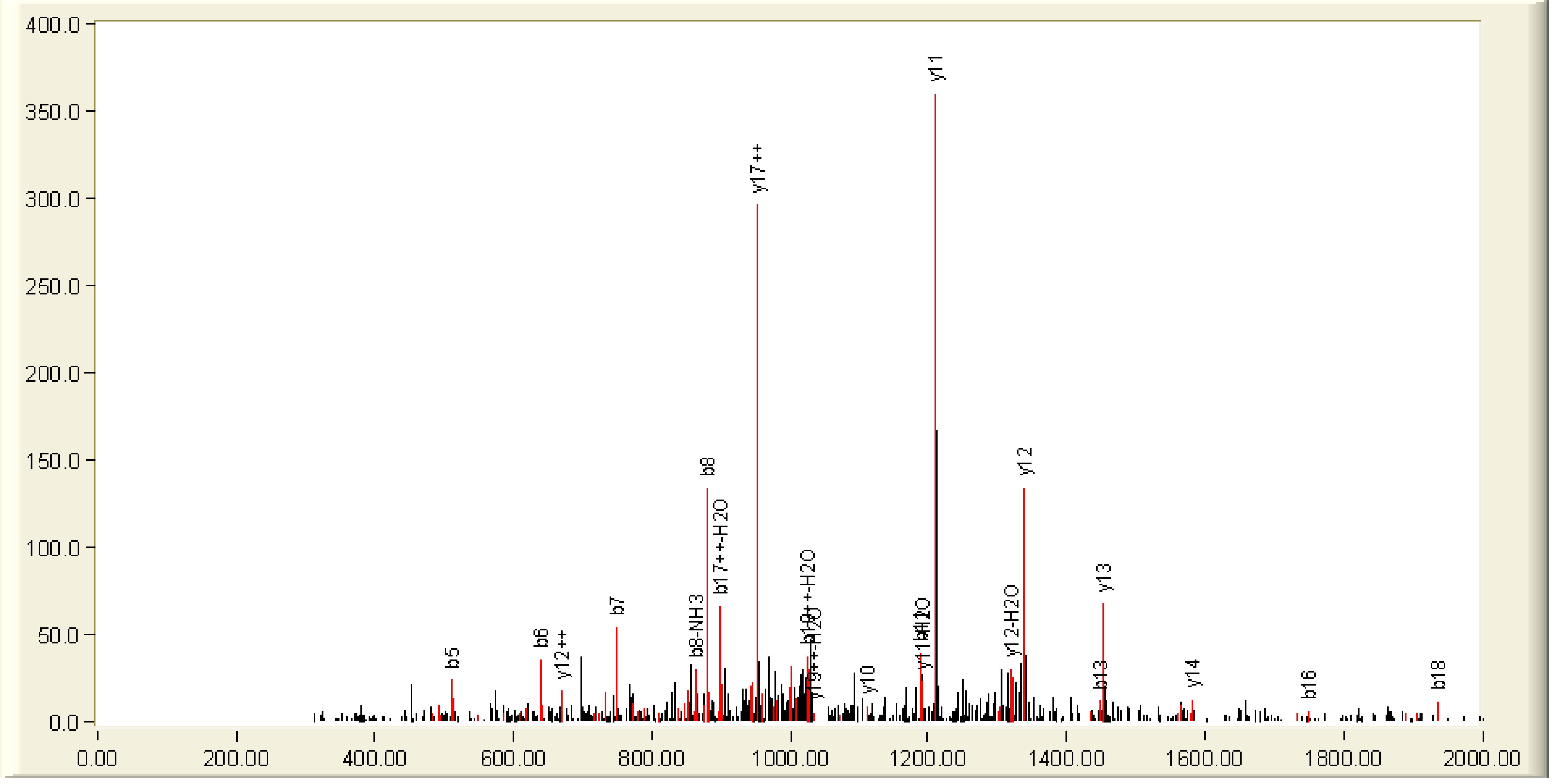

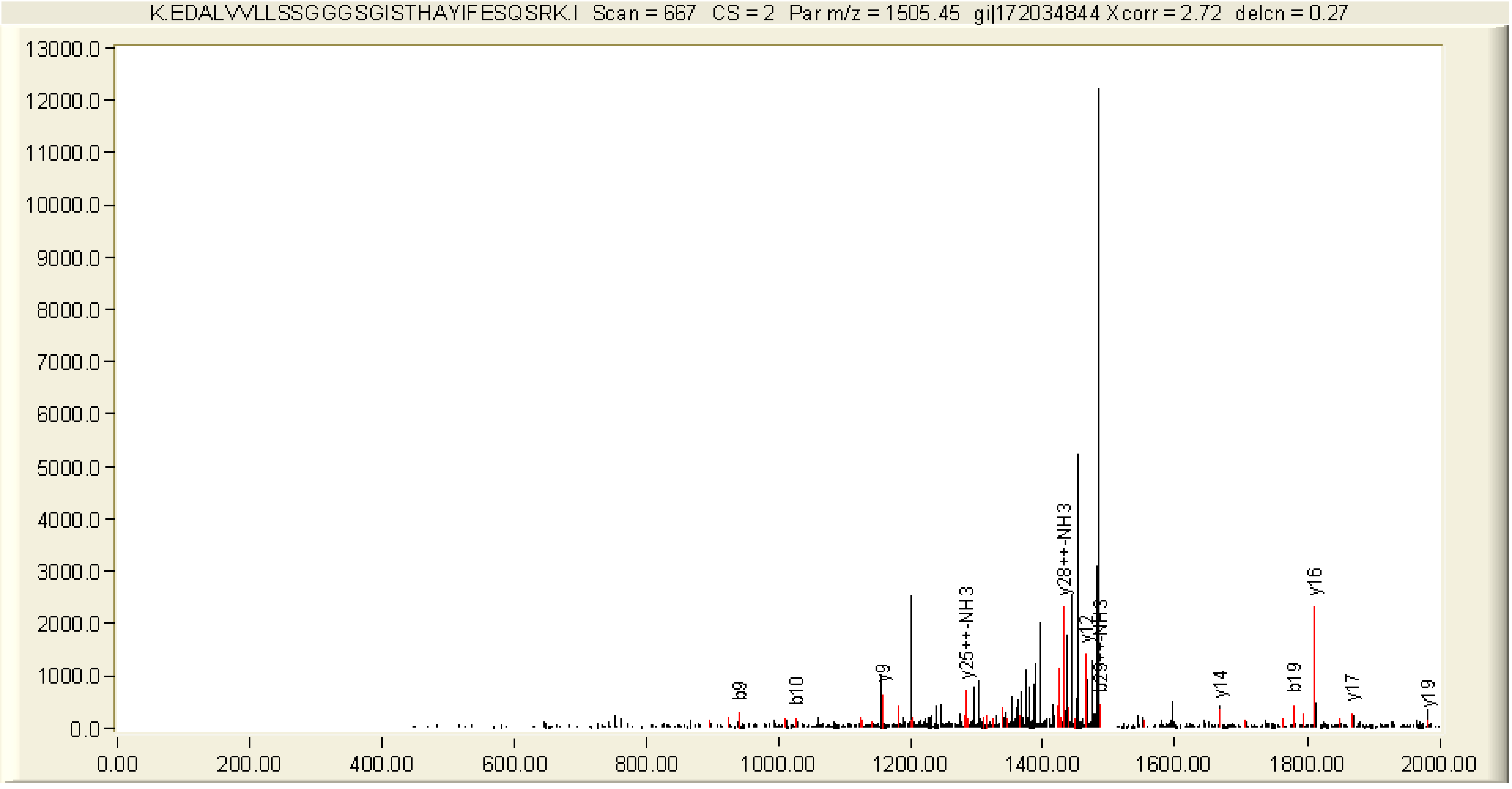
K.EDALVVLLSSGGGSGISTHAYIFESQSRK.I Scan = 667 CS = 2 Par m/z = 1505.45 gi|172034844 Xcorr = 2.72 delcn = 0.27
13000.0
12000.0
11000.0
10000.0
9000.0
8000.0
7000.0
6000.0
5000.0
4000.0
3000.0
2000.0
1000.0
0.0
0.00
200.00
400.00
600.00
800.00
1000.00
1200.00
1400.00
1600.00
1800.00
2000.00
b9
b10
y9
y25++-NH3
y28++-NH3
y12
y14
b19
y16
y17
y19

R.GRDPGYDNSNNSSGGSDAAAIVGGALVVGAIAAAIAGGSNNNSSDGR.T Scan = 669 CS = 3 Par m/z = 1436.07 gi|172038051 Xcorr = 6.02 delcn = 0.48

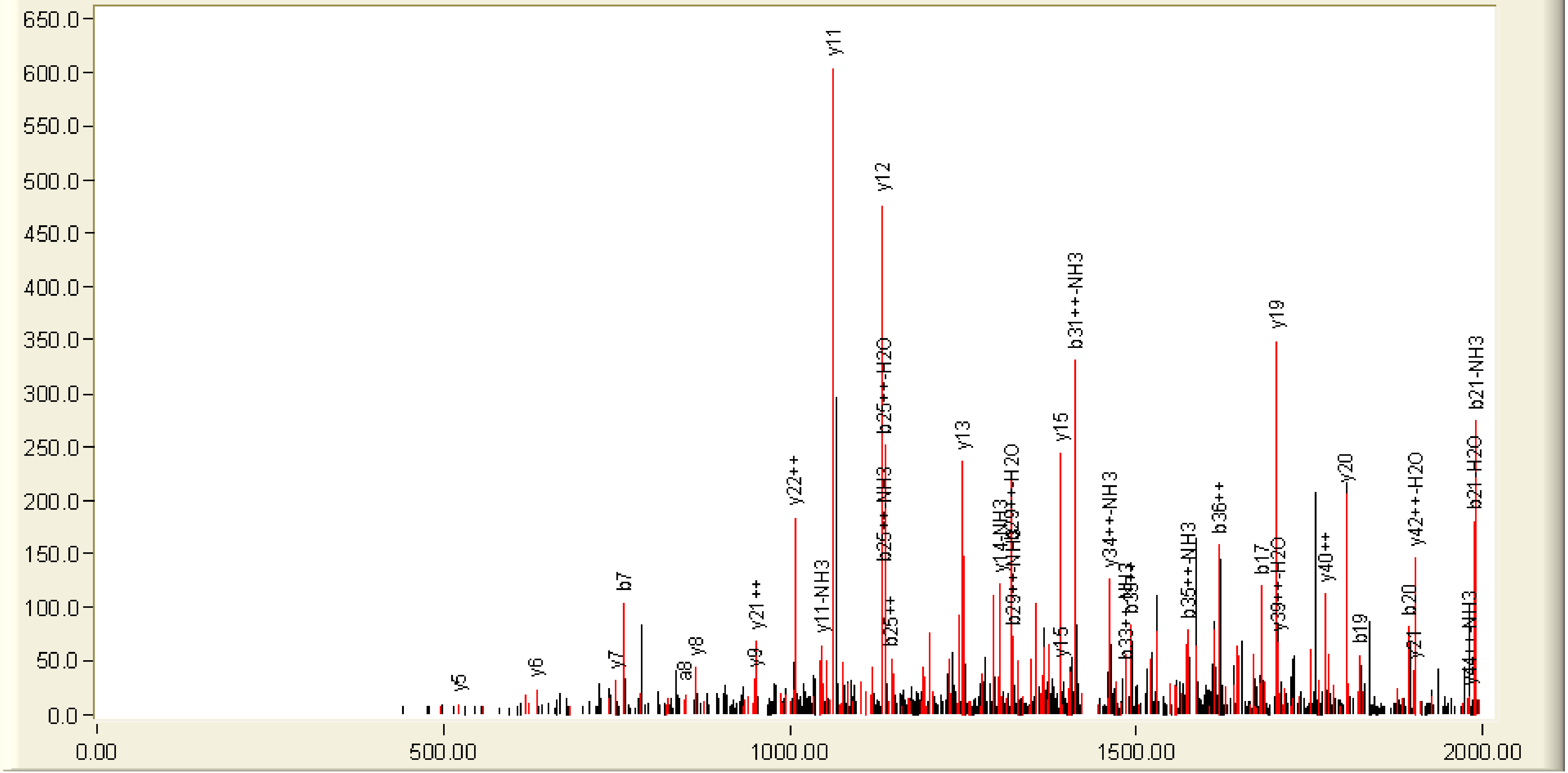

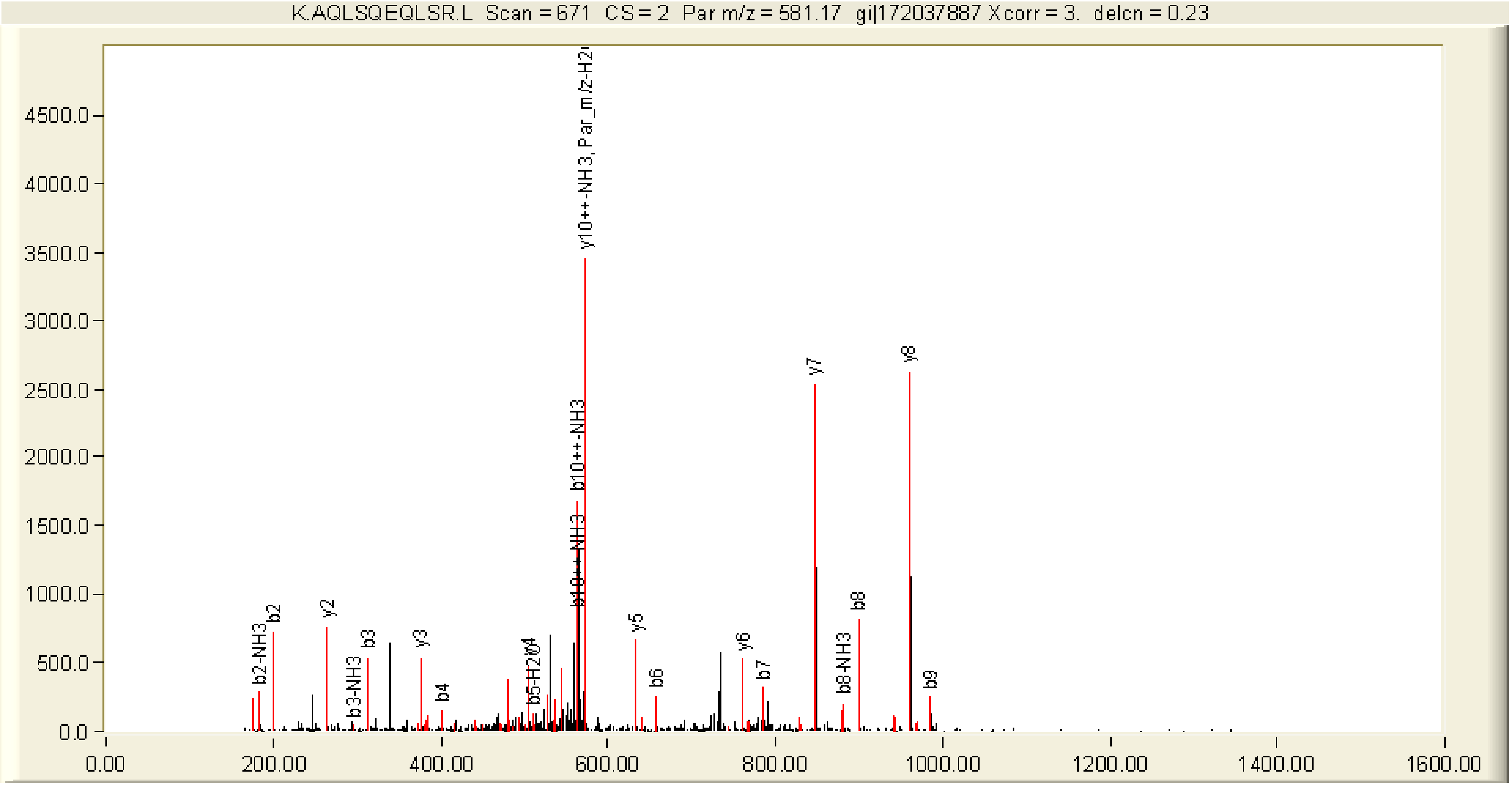
K.AQLSQEQLSR.L Scan = 671 CS = 2 Par m/z = 581.17 gi|172037887 Xcorr = 3. delcn = 0.23
y10++-NH3, Par_m/z-H2
b10++-NH3
b10++-NH3
y7
y8
b8
b8-NH3
y5
b6
y6
b7
b9
b2-NH3
b2
y2
b3-NH3
b3
y3
b4
b5-H2O
4500.0
4000.0
3500.0
3000.0
2500.0
2000.0
1500.0
1000.0
500.0
0.0
0.00
200.00
400.00
600.00
800.00
1000.00
1200.00
1400.00
1600.00

K.VGTRGTEDPYETPDTPEK.V Scan = 673 CS = 2 Par m/z = 998.27 gi|172055111 Xcorr = 2.78 delcn = 0.15

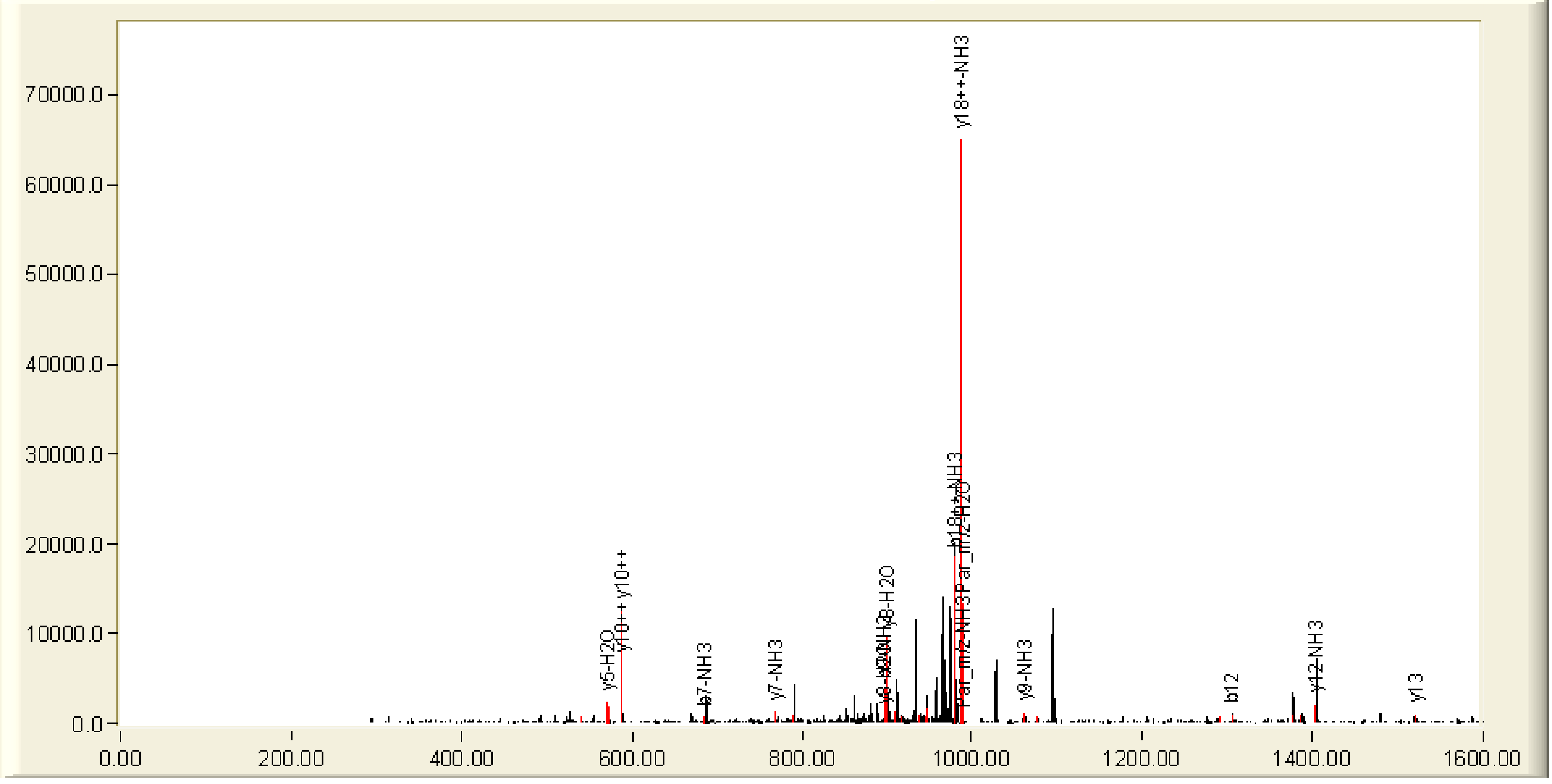

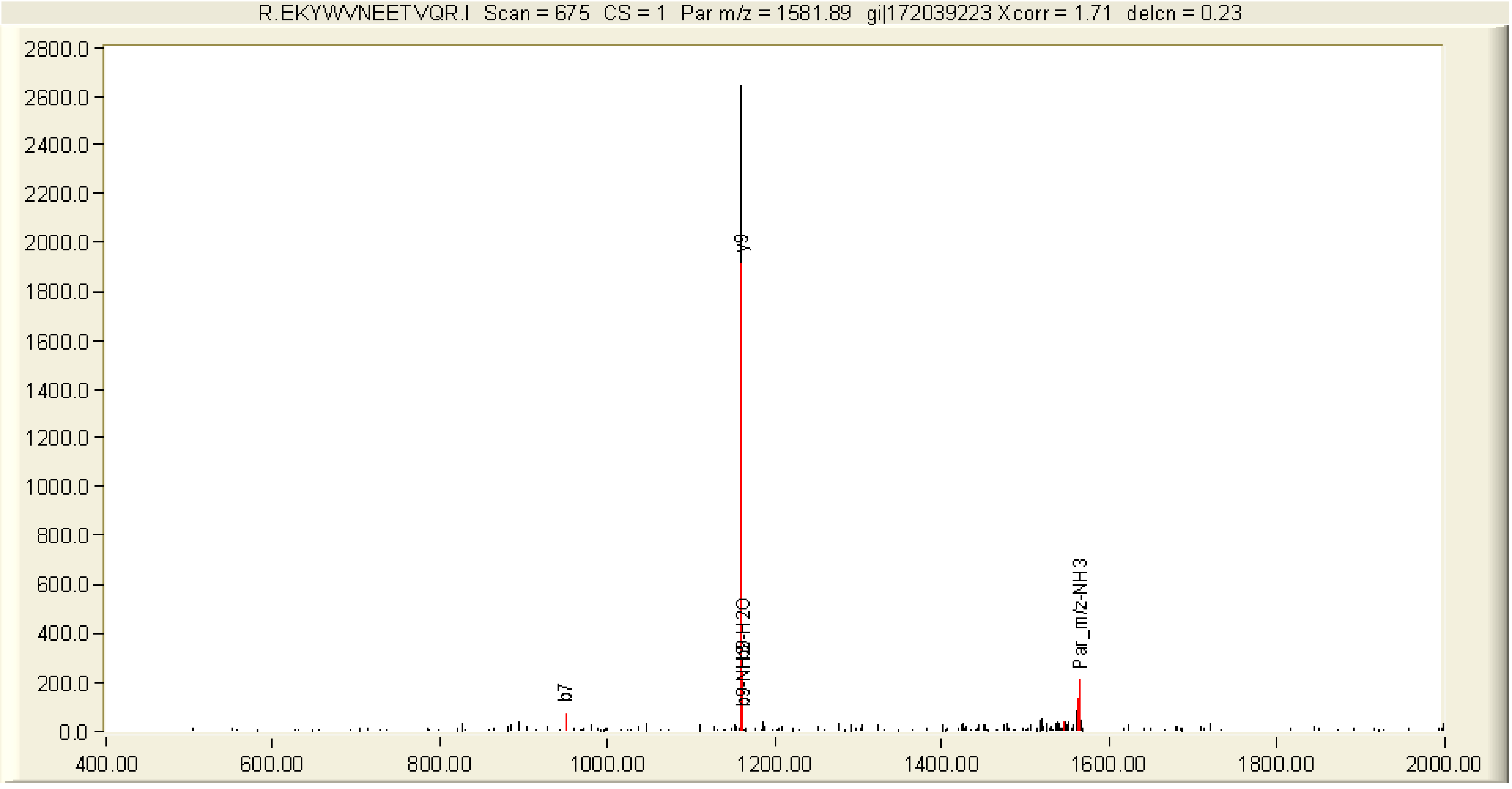
R.EKYWVNEETVQR.I Scan = 675 CS = 1 Par m/z = 1581.89 gi|172039223 Xcorr = 1.71 delcn = 0.23
y9
b7
Par_m/z-NH3
0.0
200.0
400.0
600.0
800.0
1000.0
1200.0
1400.0
1600.0
1800.0
2000.0
2200.0
2400.0
2600.0
2800.0
400.00
600.00
800.00
1000.00
1200.00
1400.00
1600.00
1800.00
2000.00

K.QTLSQQEMLLMR.S Scan = 677 CS = 2 Par m/z = 740.58 gi|172054982 Xcorr = 2.96 delcn = 0.16

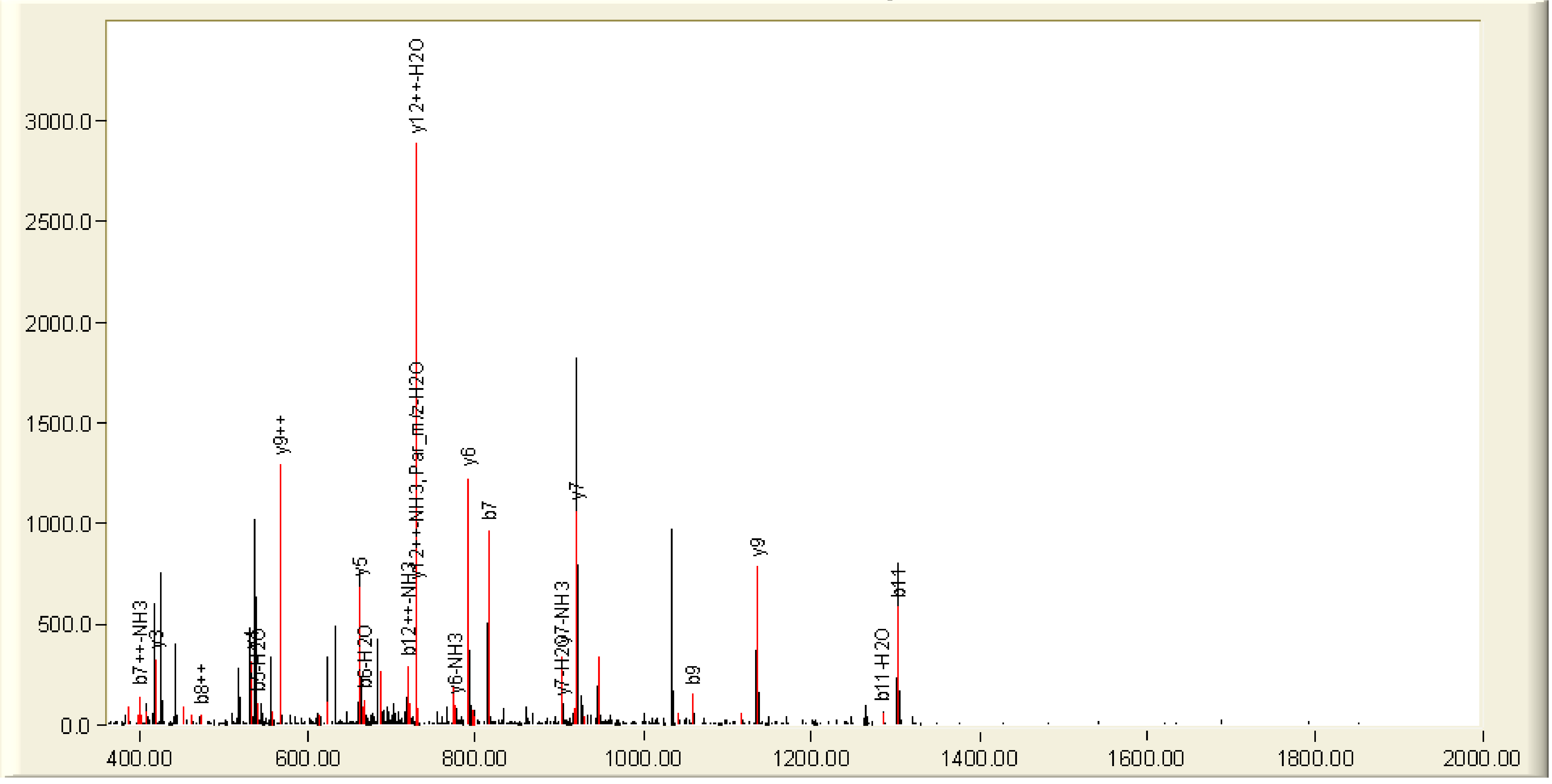

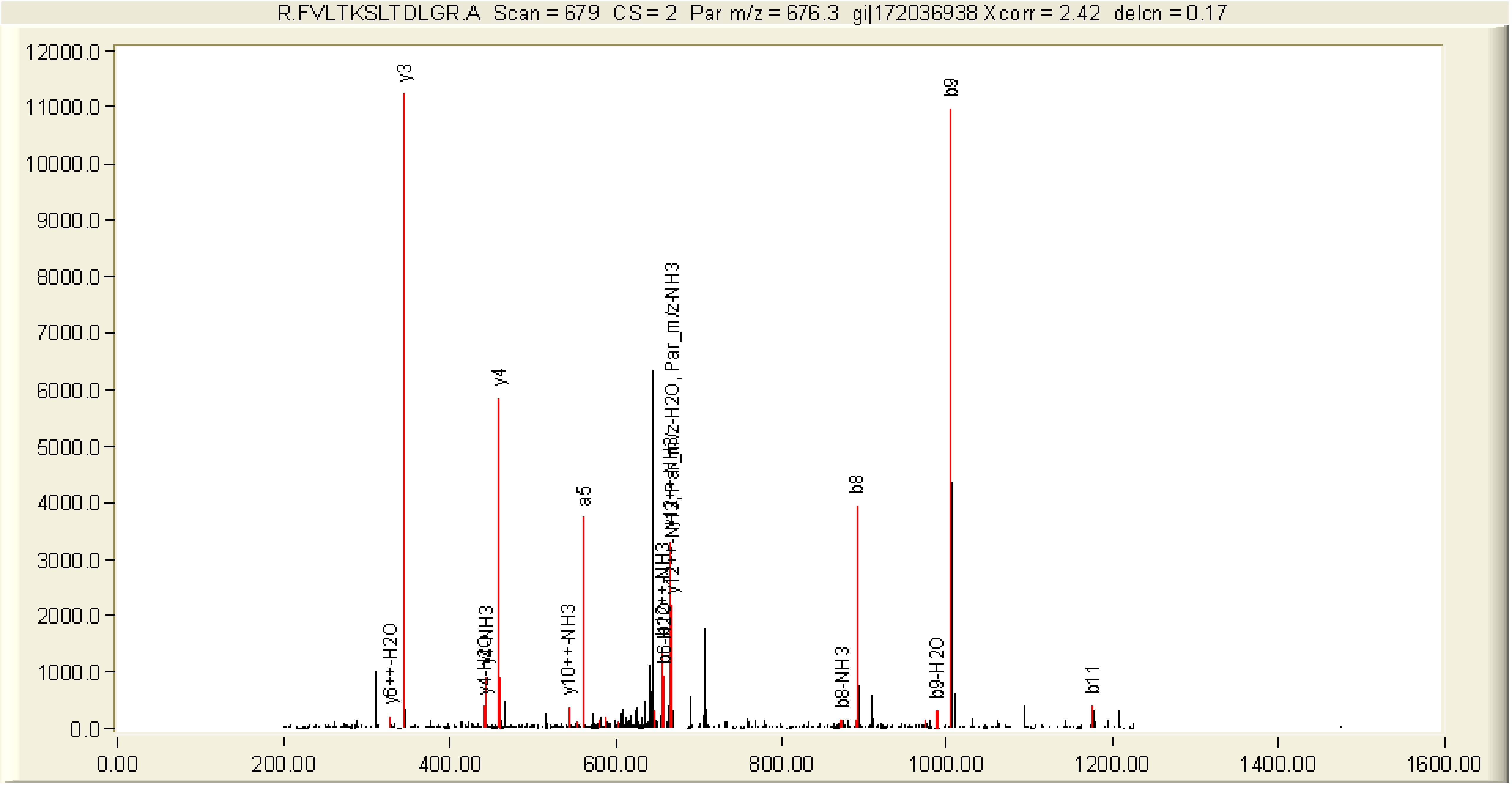

R.FVLTKSLTDLGR.A Scan = 679 CS = 2 Par m/z = 676.3 gi|172036938 Xcorr = 2.42 delcn = 0.17
12000.0
11000.0
10000.0
9000.0
8000.0
7000.0
6000.0
5000.0
4000.0
3000.0
2000.0
1000.0
0.0
0.00
200.00
400.00
600.00
800.00
1000.00
1200.00
1400.00
1600.00
y3
b9
y4
a5
b8
y6++-H2O
y10++-NH3
b8-NH3
b9-H2O
b11
Par_m/z-NH3

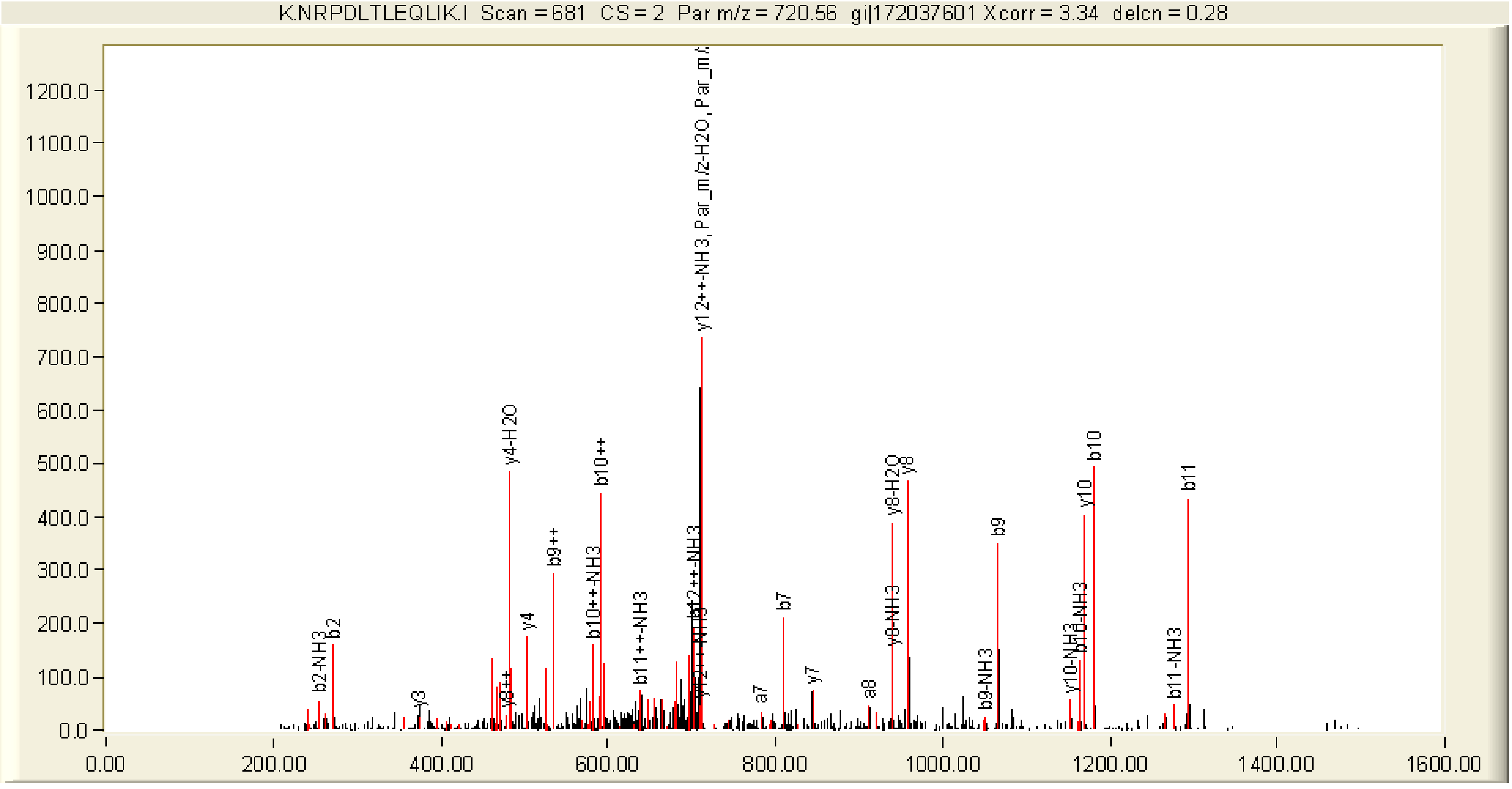
K.NRPDLTLEQLIK.I Scan = 681 CS = 2 Par m/z = 720.56 gi|172037601 Xcorr = 3.34 delcn = 0.28
y12++-NH3, Par_m/z-H2O, Par_m/
y4-H2O
b10++
b9++
b10++-NH3
b11++-NH3
y12++-NH3
b2-NH3
b2
y3
y8++
y4
a7
b7
y7
a8
y8-H2O
y8
y8-NH3
b9
b9-NH3
y10
b10
y10-NH3
b10-NH3
b11
b11-NH3
1200.0
1100.0
1000.0
900.0
800.0
700.0
600.0
500.0
400.0
300.0
200.0
100.0
0.0
0.00
200.00
400.00
600.00
800.00
1000.00
1200.00
1400.00
1600.00

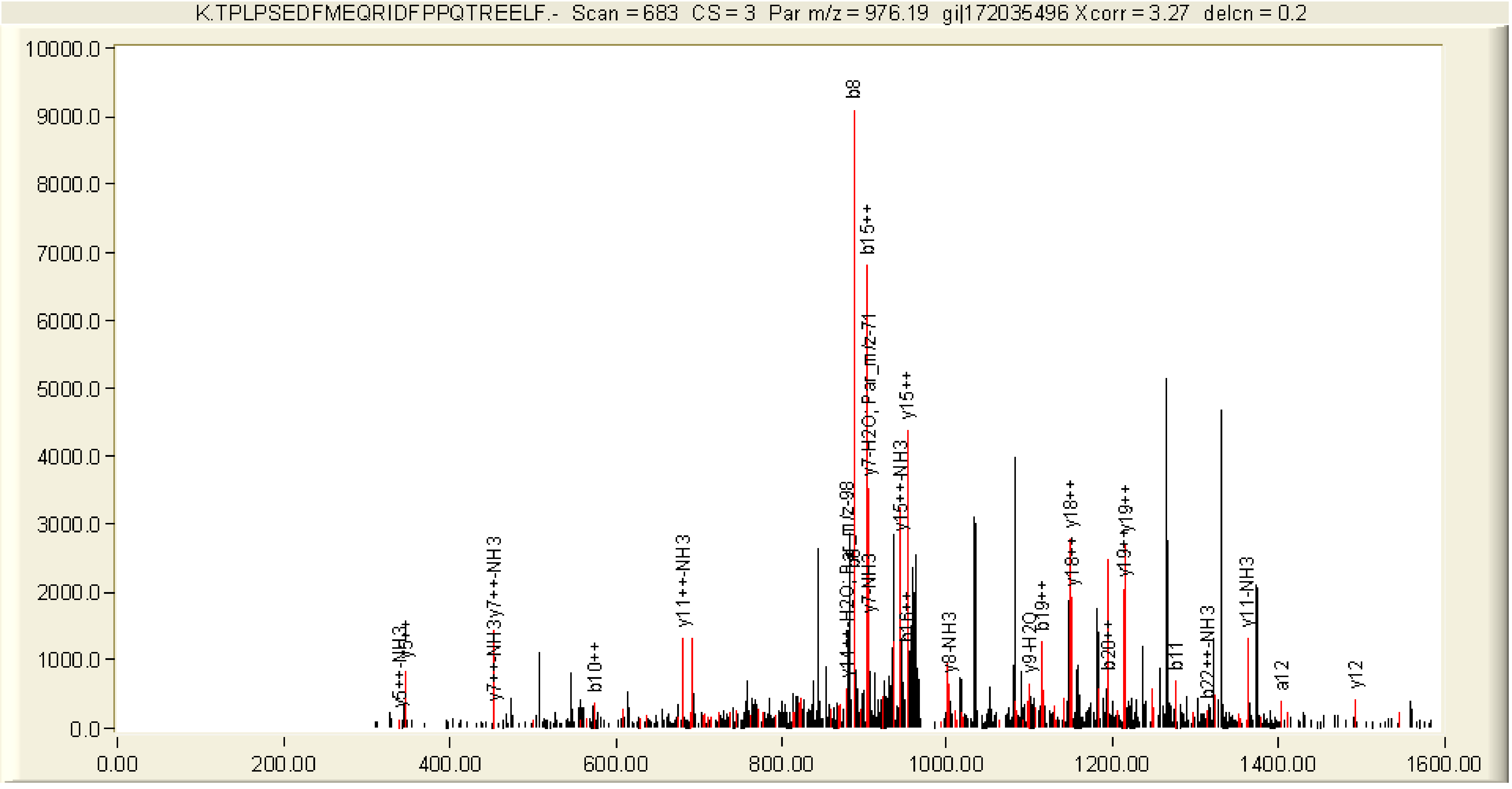

K.TPLPSEDFMEQRIDFPPQTREELF.- Scan = 683 CS = 3 Par m/z = 976.19 gi|172035496 Xcorr = 3.27 delcn = 0.2
10000.0
9000.0
8000.0
7000.0
6000.0
5000.0
4000.0
3000.0
2000.0
1000.0
0.0
0.00
200.00
400.00
600.00
800.00
1000.00
1200.00
1400.00
1600.00
b8
b15++
y7-H2O,Par_m/z-71
y15++
y15++-NH3
y7-NH3
y18++
y19++
y11++-NH3
y7++-NH3y7++-NH3
b10++
y8-NH3
y9-H2O
b19++
b20++
b11
b22++-NH3
y11-NH3
a12
y12

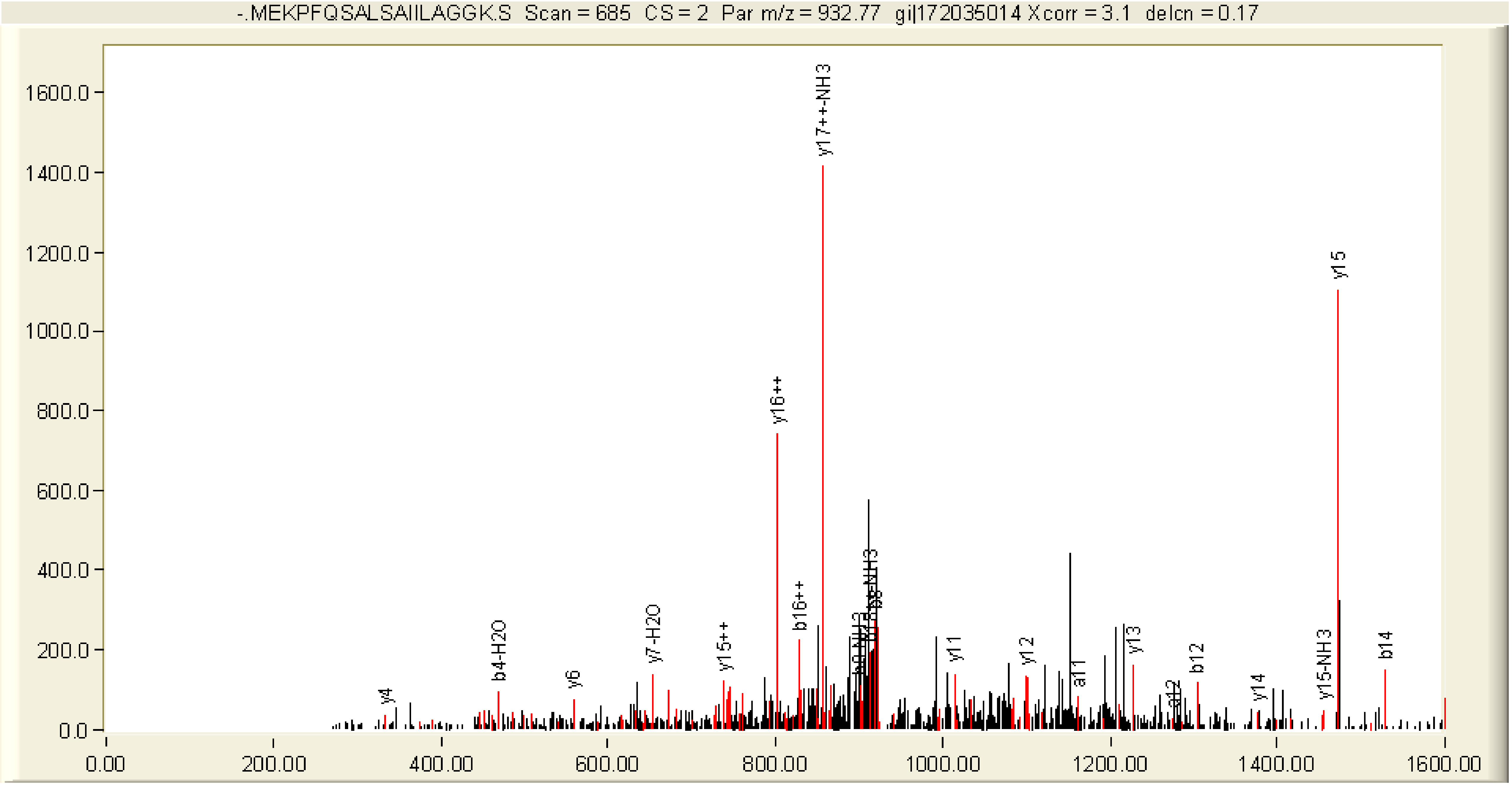

-.MEKPFQSALSAIILAGGK.S Scan = 685 CS = 2 Par m/z = 932.77 gi|172035014 Xcorr = 3.1 delcn = 0.17
1600.0
1400.0
1200.0
1000.0
800.0
600.0
400.0
200.0
0.0
0.00
200.00
400.00
600.00
800.00
1000.00
1200.00
1400.00
1600.00
y4
b4-H2O
y6
y7-H2O
y15++
y16++
b16++
y17++-NH3
y11
y12
a11
y13
b12
y14
y15-NH3
y15
b14

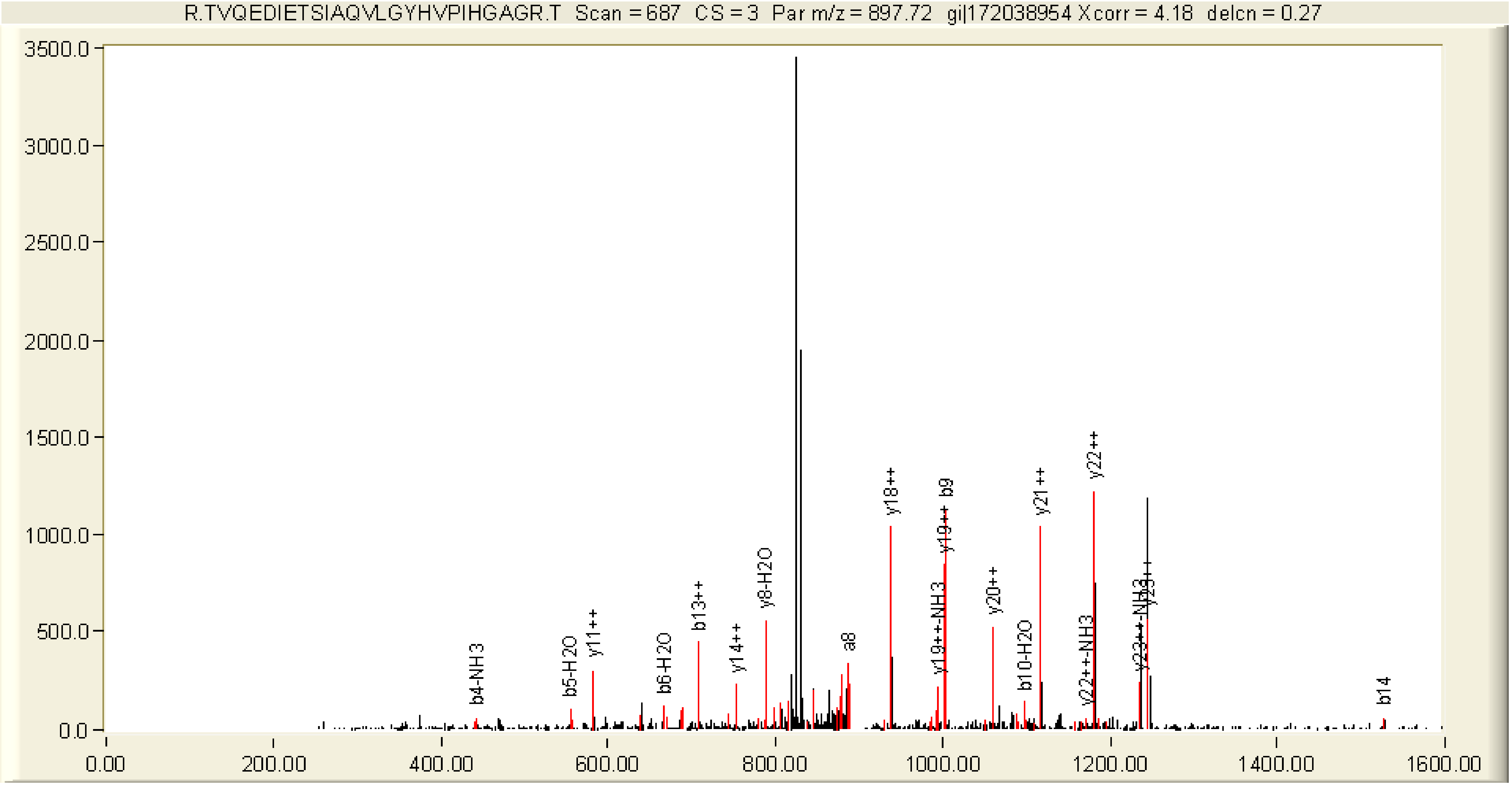
R.TVQEDIETSIAQVLGYHVPIHGAGR.T Scan = 687 CS = 3 Par m/z = 897.72 gi|172038954 Xcorr = 4.18 delcn = 0.27
3500.0
3000.0
2500.0
2000.0
1500.0
1000.0
500.0
0.0
0.00
200.00
400.00
600.00
800.00
1000.00
1200.00
1400.00
1600.00
b4-NH3
b5-H2O
y11++
b6-H2O
b13++
y14++
y8-H2O
a8
y18++
y19++-NH3
y19++
b9
y20++
b10-H2O
y21++
y22++-NH3
y22++
y23++-NH3
y23++
b14

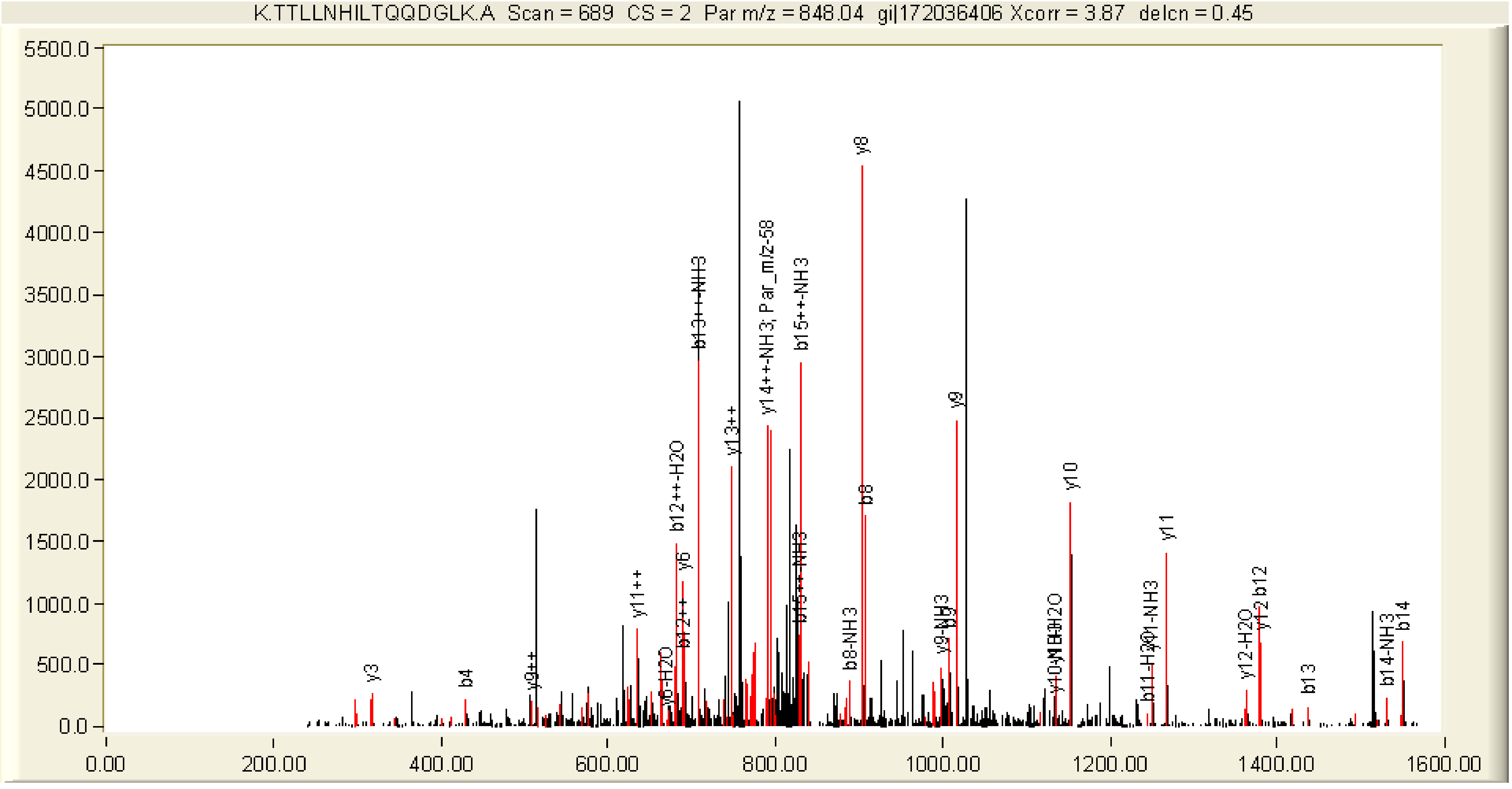
K.TTLLNHILTQQDGLK.A Scan = 689 CS = 2 Par m/z = 848.04 gi|172036406 Xcorr = 3.87 delcn = 0.45
5500.0
5000.0
4500.0
4000.0
3500.0
3000.0
2500.0
2000.0
1500.0
1000.0
500.0
0.0
0.00
200.00
400.00
600.00
800.00
1000.00
1200.00
1400.00
1600.00
y3
b4
y9++
y11++
b12++-H2O
y6
b12++
b13++-NH3
y13++
y14++-NH3; Par_m/z-58
b15++-NH3
b8-NH3
y8
b8
y9
y10
y11
b11-H2O
y12-H2O
b12
b13
b14-NH3
b14

R.SQITIKDVLEEIEEREGSIKPGDSAVR.E Scan = 691 CS = 3 Par m/z = 1000.34 gi|172038689 Xcorr = 5.14 delcn = 0.43

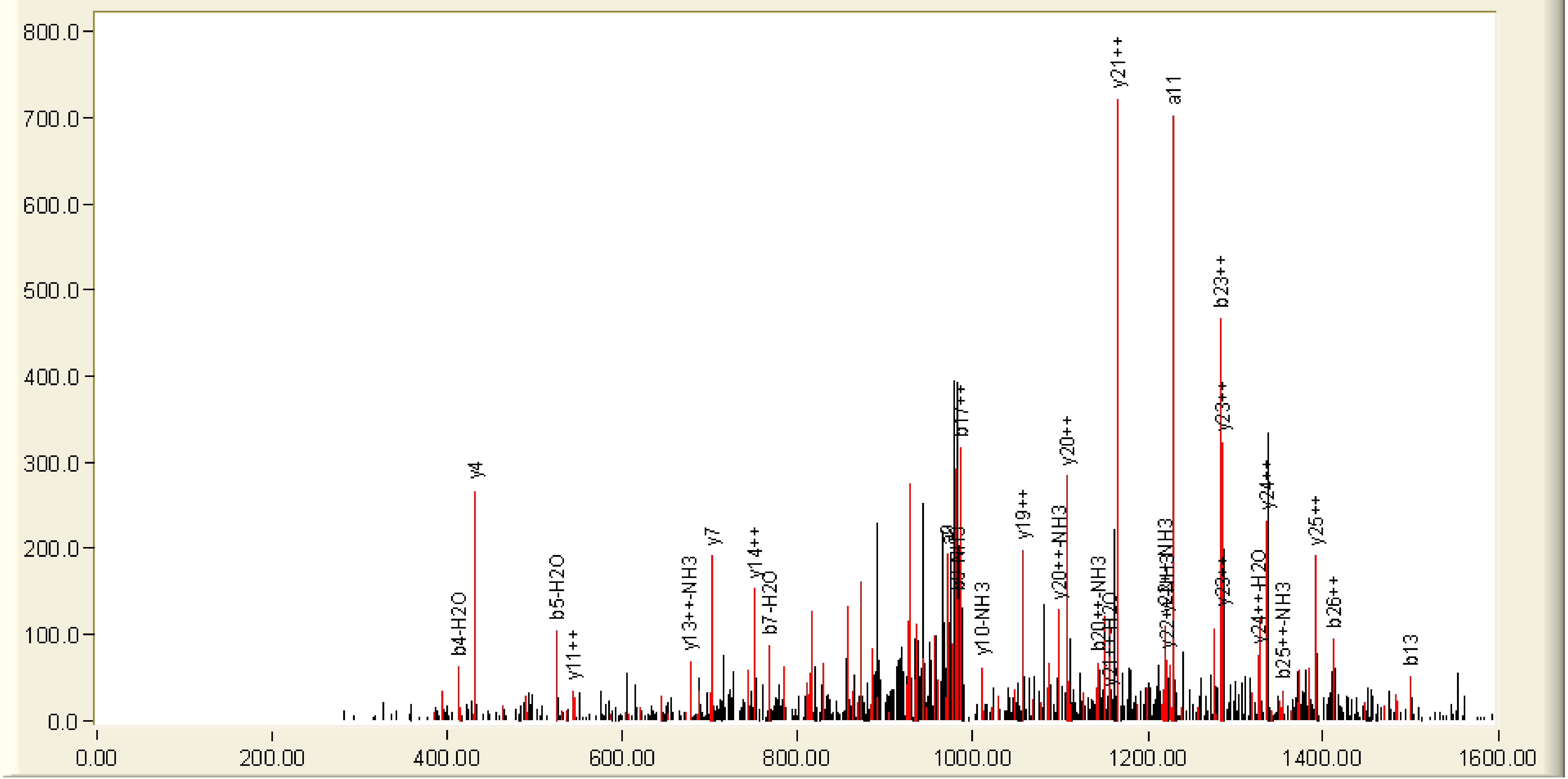

R.NDGKLAYYSLRDKR.I Scan = 693 CS = 2 Par m/z = 849.58 gi|172054940 Xcorr = 2.51 delcn = 0.15

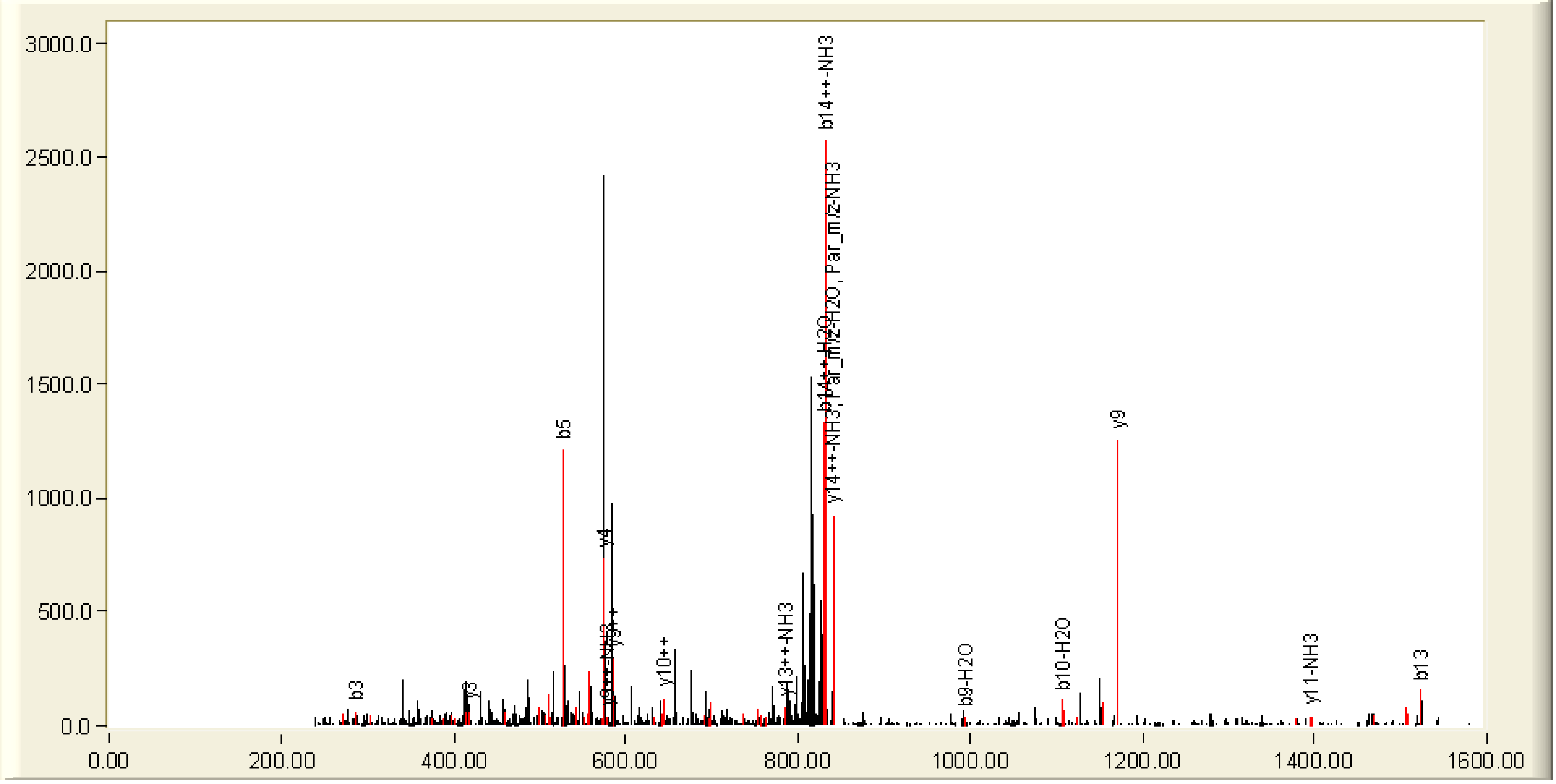

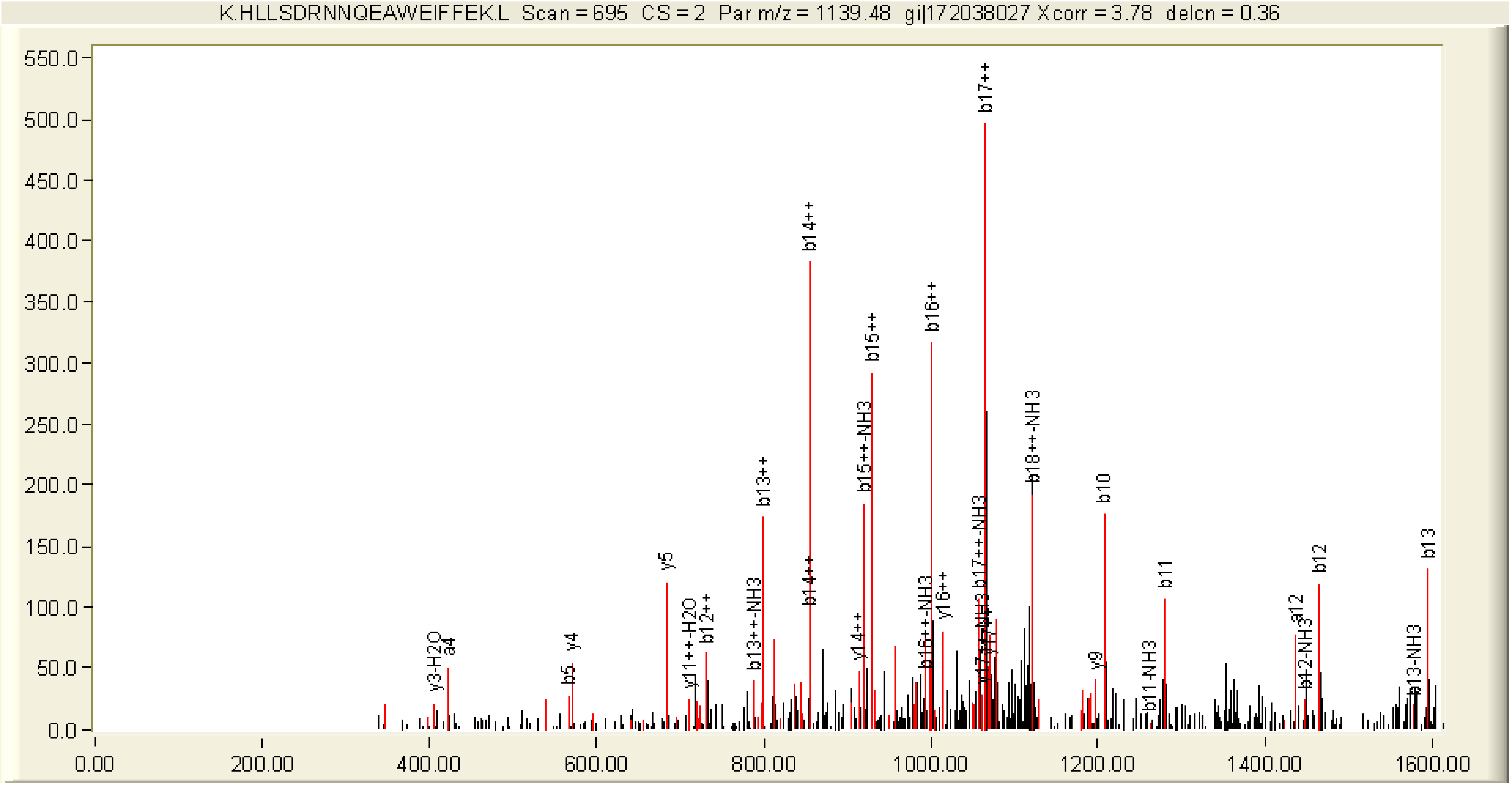
K.HLLSDRNNQEAWEIFFEK.L Scan = 695 CS = 2 Par m/z = 1139.48 gi|172038027 Xcorr = 3.78 delcn = 0.36
550.0
500.0
450.0
400.0
350.0
300.0
250.0
200.0
150.0
100.0
50.0
0.0
0.00
200.00
400.00
600.00
800.00
1000.00
1200.00
1400.00
1600.00
y3-H2O
a4
b5
y4
y5
y11++-H2O
b12++
b13++-NH3
b13++
b14++
b14++
y14++
b15++-NH3
b15++
b16++-NH3
b16++
y16++
b17++-NH3
b17++
b18++-NH3
y9
b10
b11-NH3
b11
a12
b12-NH3
b12
b13-NH3
b13

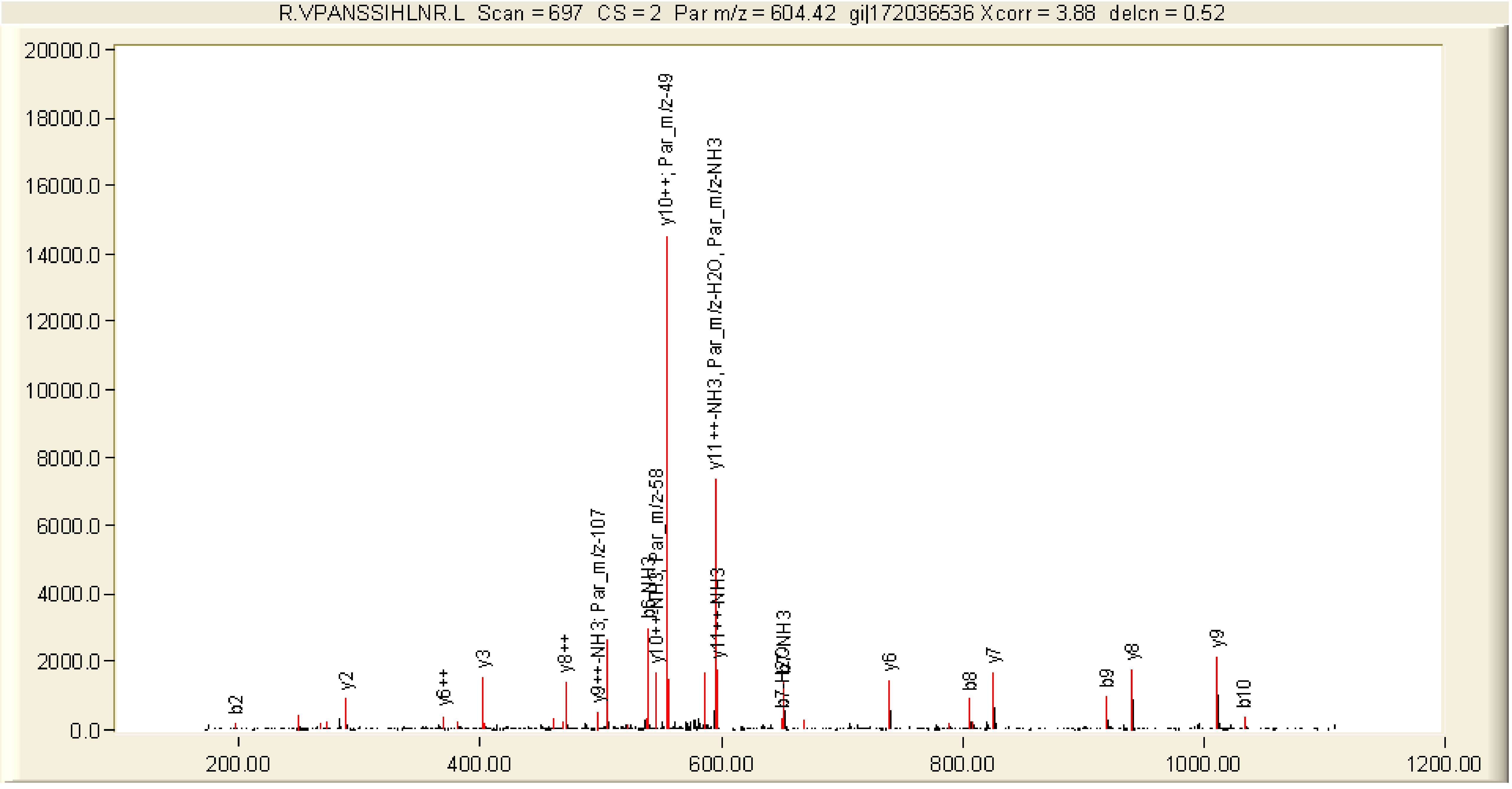

R.VPANSSIHLNR.L Scan = 697 CS = 2 Par m/z = 604.42 gi|172036536 Xcorr = 3.88 delcn = 0.52
20000.0
18000.0
16000.0
14000.0
12000.0
10000.0
8000.0
6000.0
4000.0
2000.0
0.0
200.00
400.00
600.00
800.00
1000.00
1200.00
b2
y2
y6++
y3
y8++
y9++-NH3; Par_m/z-107
y10++; Par_m/z-49
Par_m/z-58
y11++-NH3, Par_m/z-H2O, Par_m/z-NH3
y6
b8
y7
b9
y8
y9
b10

K.IIAHHQDMSLEALLK.F Scan = 699 CS = 2 Par m/z = 860.7 gi|172037921 Xcorr = 3.25 delcn = 0.16

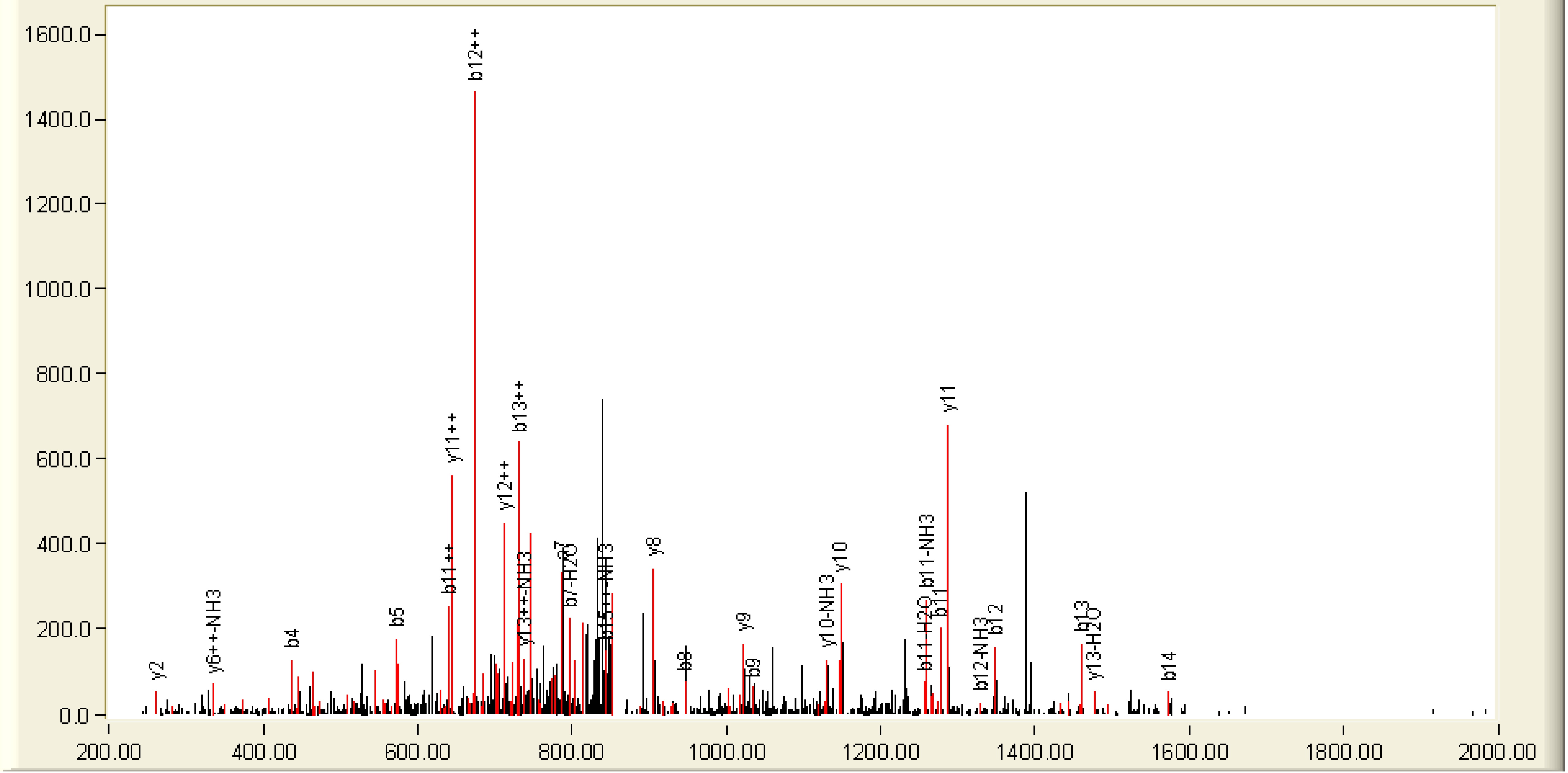

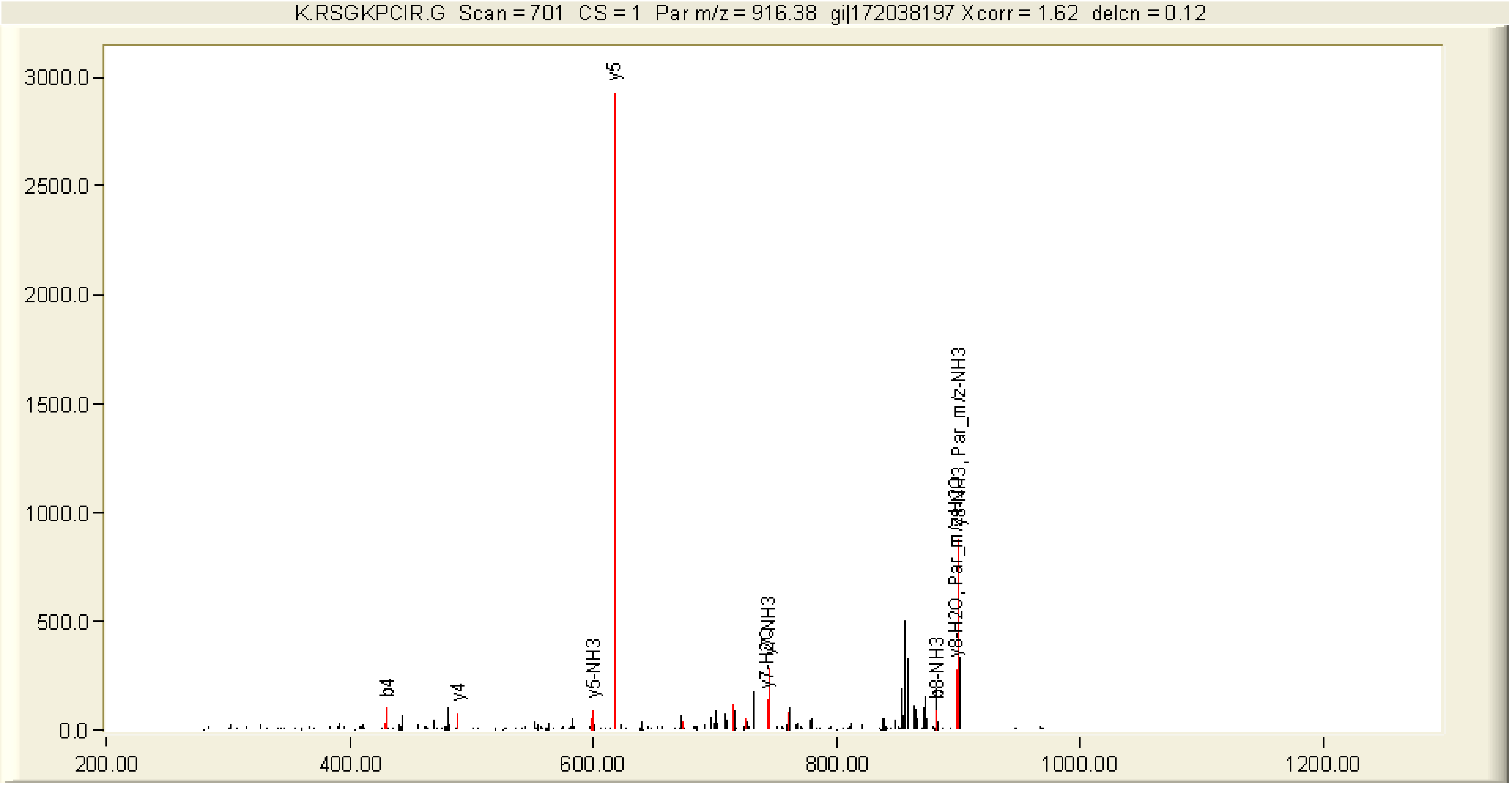
K.RSGKPCIR.G Scan = 701 CS = 1 Par m/z = 916.38 gi|172038197 Xcorr = 1.62 delcn = 0.12
3000.0
2500.0
2000.0
1500.0
1000.0
500.0
0.0
200.00
400.00
600.00
800.00
1000.00
1200.00
y5
b4
y4
y5-NH3
b8-NH3

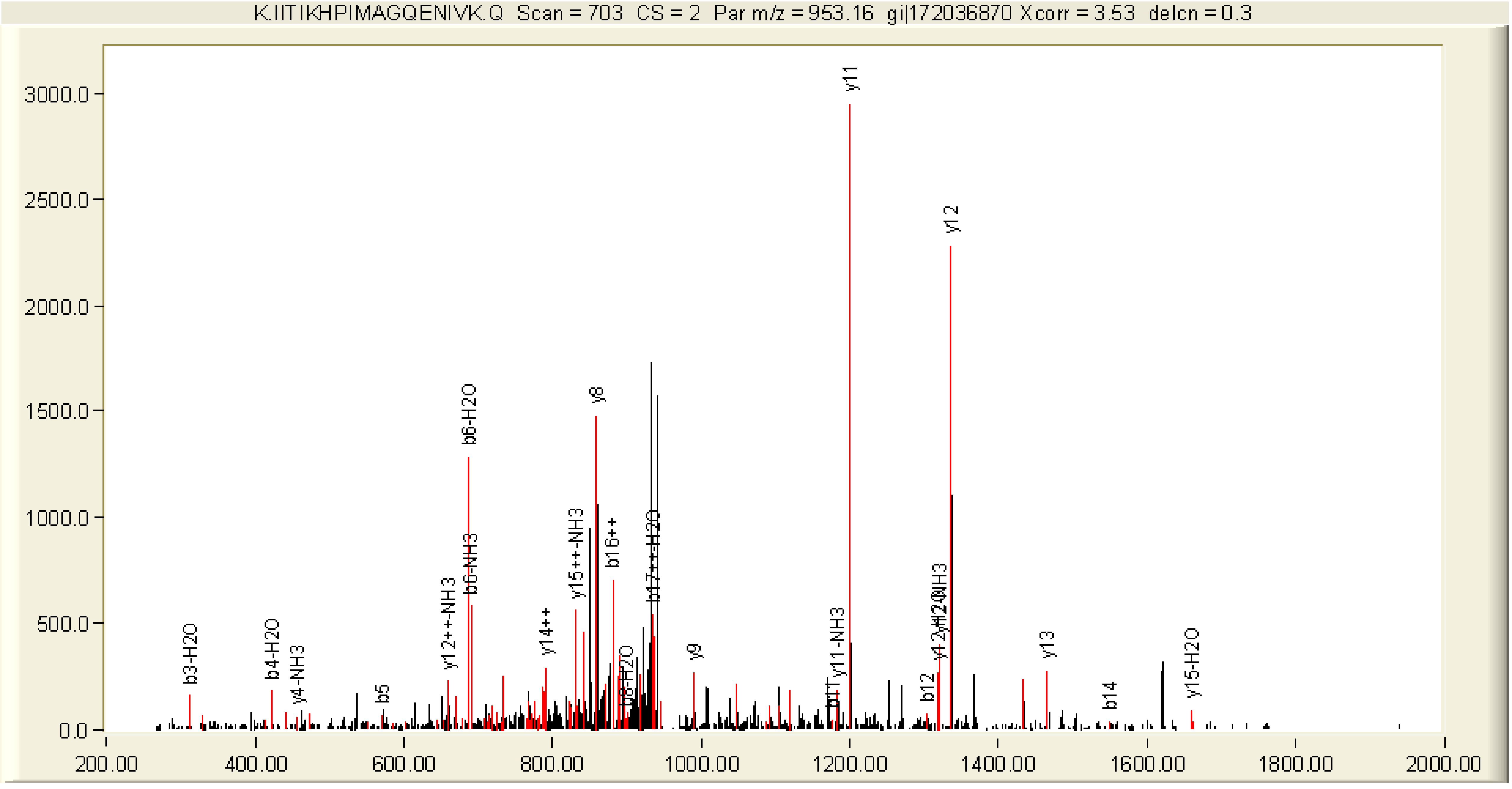

K.IITIKHPIMAGQENIVK.Q Scan = 703 CS = 2 Par m/z = 953.16 gi|172036870 Xcorr = 3.53 delcn = 0.3
3000.0
2500.0
2000.0
1500.0
1000.0
500.0
0.0
200.00
400.00
600.00
800.00
1000.00
1200.00
1400.00
1600.00
1800.00
2000.00
y11
y12
b6-H2O
y8
b6-NH3
y15++-NH3
b16++
y12++-NH3
y14++
b3-H2O
b4-H2O
y4-NH3
b5
y9
y11-NH3
b11
b12
y13
b14
y15-H2O

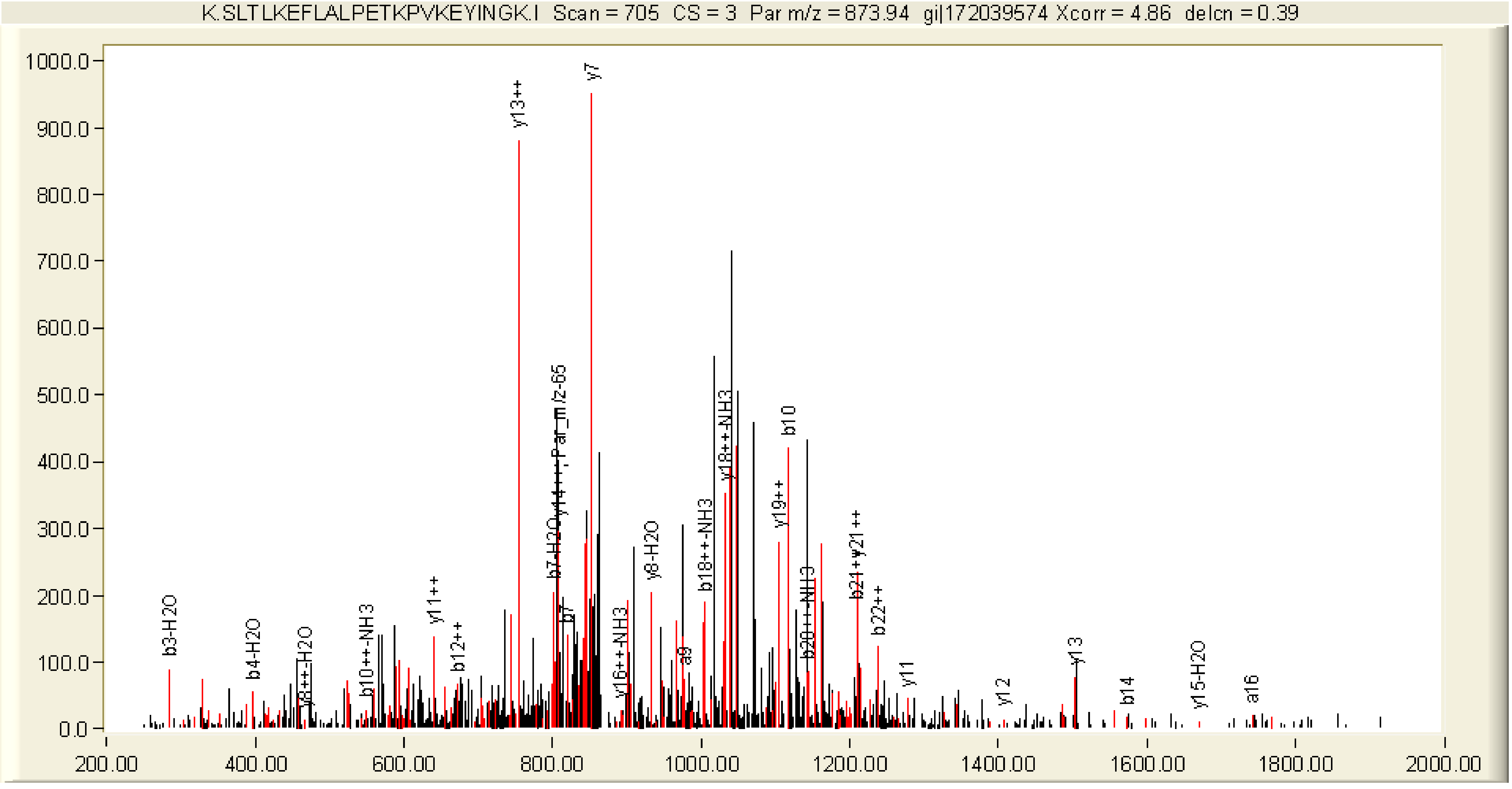

K.SLTLKEFLALPETKPVKEYINGK.I Scan = 705 CS = 3 Par m/z = 873.94 gi|172039574 Xcorr = 4.86 delcn = 0.39
1000.0
900.0
800.0
700.0
600.0
500.0
400.0
300.0
200.0
100.0
0.0
200.00
400.00
600.00
800.00
1000.00
1200.00
1400.00
1600.00
1800.00
2000.00
b3-H2O
b4-H2O
y8++-H2O
b10++-NH3
y11++
b12++
y13++
b7-H2O
b7
y7
y16++-NH3
y8-H2O
a9
b18++-NH3
y18++-NH3
y19++
b10
b20++-NH3
b21++y21++
b22++
y11
y12
y13
b14
y15-H2O
a16

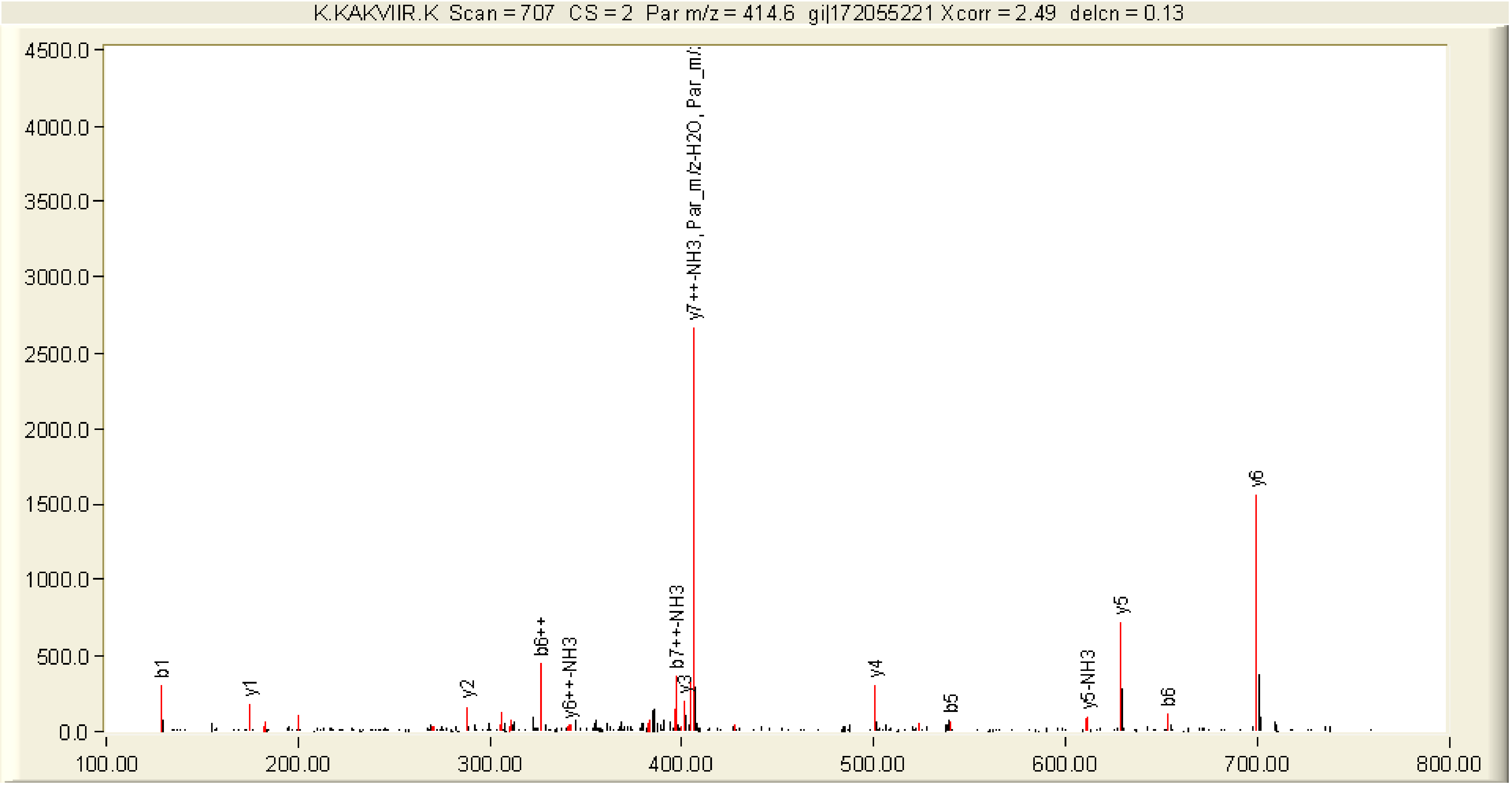
K.KAKVIIR.K Scan = 707 CS = 2 Par m/z = 414.6 gi|172055221 Xcorr = 2.49 delcn = 0.13
4500.0
4000.0
3500.0
3000.0
2500.0
2000.0
1500.0
1000.0
500.0
0.0
100.00
200.00
300.00
400.00
500.00
600.00
700.00
800.00
b1
y1
y2
b6++
y6++-NH3
b7++-NH3
y3
y7++-NH3, Par_m/z-H2O, Par_m/z
y4
b5
y5-NH3
y5
b6
y6

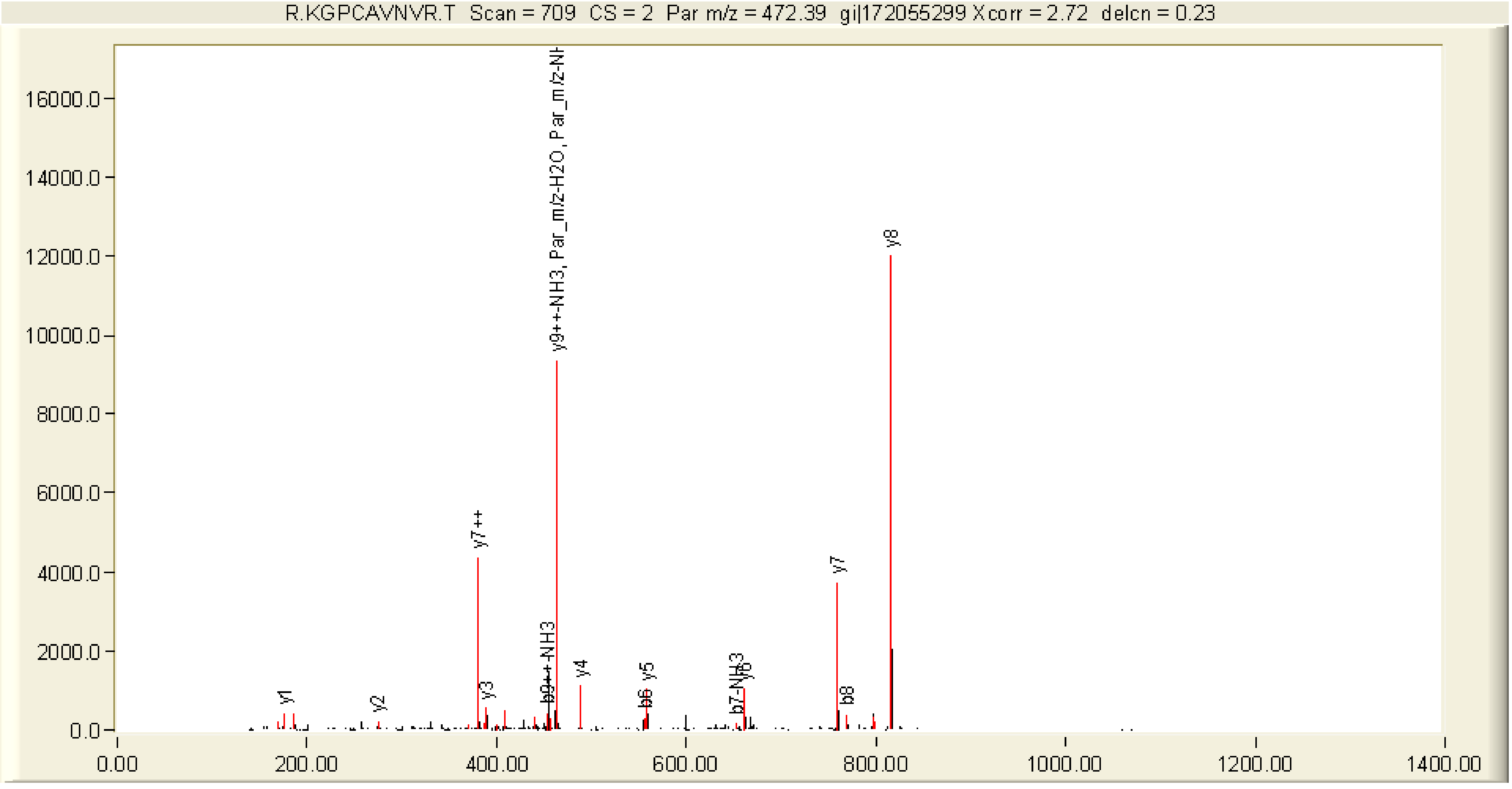
R.KGPCAVNVR.T Scan = 709 CS = 2 Par m/z = 472.39 gi|172055299 Xcorr = 2.72 delcn = 0.23
16000.0
14000.0
12000.0
10000.0
8000.0
6000.0
4000.0
2000.0
0.0
0.00
200.00
400.00
600.00
800.00
1000.00
1200.00
1400.00
y9++-NH3, Par_m/z-H2O, Par_m/z-NH
y8
y7++
y7
y1
y2
y3
y4
y5
b6
b8
b7-NH3
y6

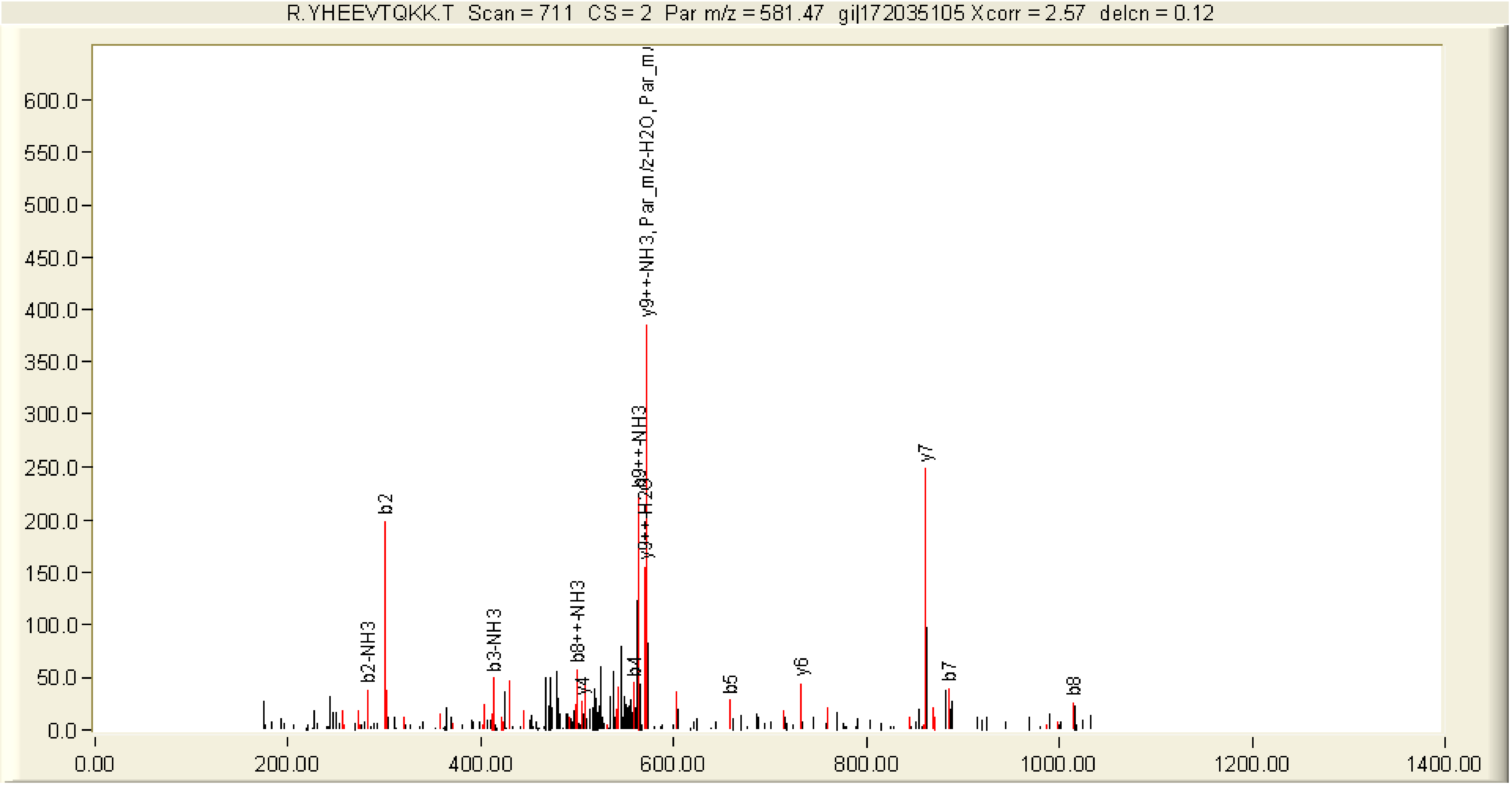
R.YHEEVTQKK.T Scan = 711 CS = 2 Par m/z = 581.47 gi|172035105 Xcorr = 2.57 delcn = 0.12
600.0
550.0
500.0
450.0
400.0
350.0
300.0
250.0
200.0
150.0
100.0
50.0
0.0
0.00
200.00
400.00
600.00
800.00
1000.00
1200.00
1400.00
b2-NH3
b2
b3-NH3
b8++-NH3
y4
b4
y9++-NH3, Par_m/z-H2O, Par_m/
b9++-NH3
y9++-H2O
b5
y6
y7
b7
b8

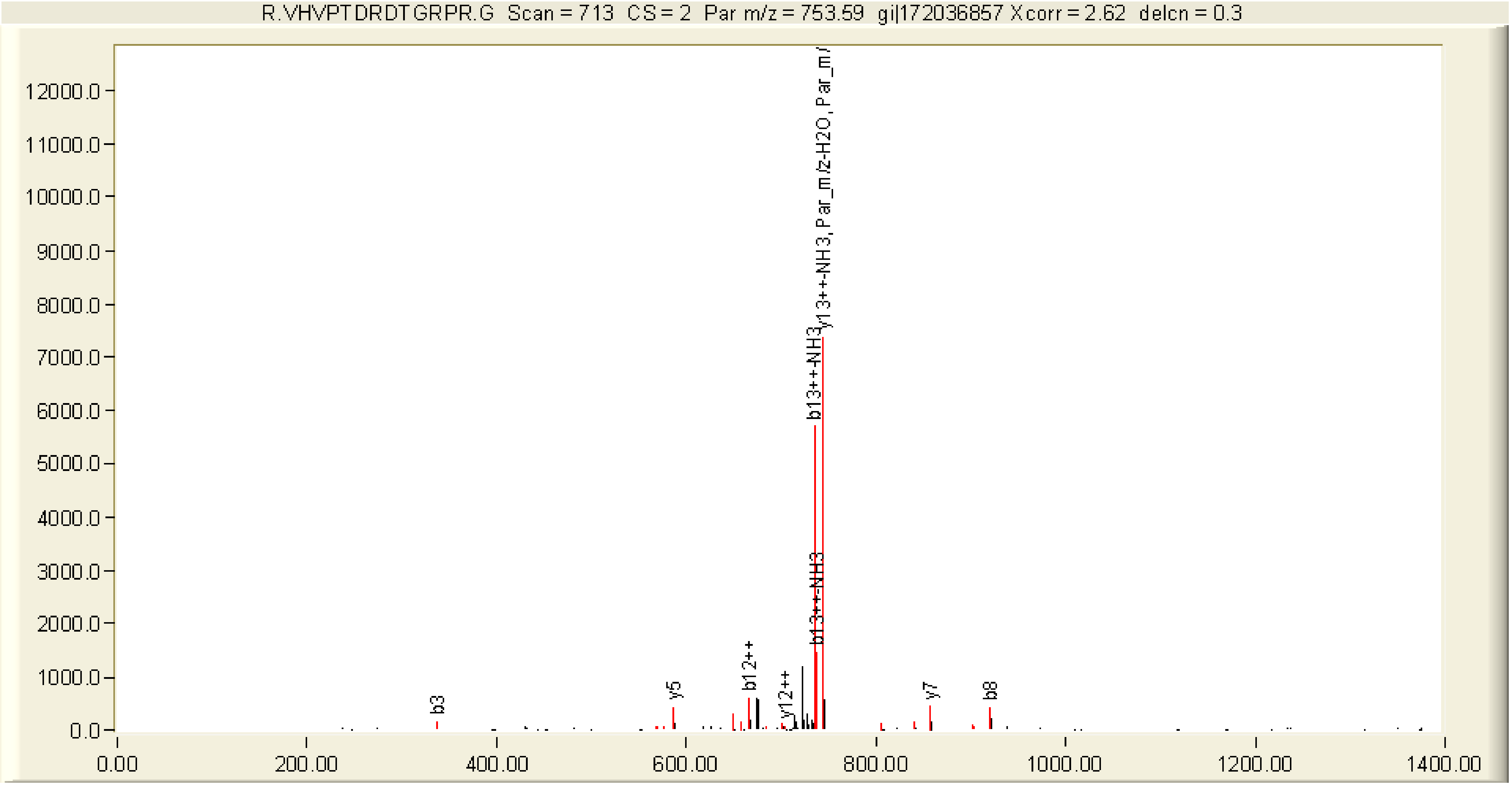

R.VHVPTDRDTGRPR.G Scan = 713 CS = 2 Par m/z = 753.59 gi|172036857 Xcorr = 2.62 delcn = 0.3
12000.0
11000.0
10000.0
9000.0
8000.0
7000.0
6000.0
5000.0
4000.0
3000.0
2000.0
1000.0
0.0
0.00
200.00
400.00
600.00
800.00
1000.00
1200.00
1400.00
b3
y5
b12++
y12++
b13++-NH3
b13++-NH3
y13++-NH3, Par_m/z-H2O, Par_m/
y7
b8

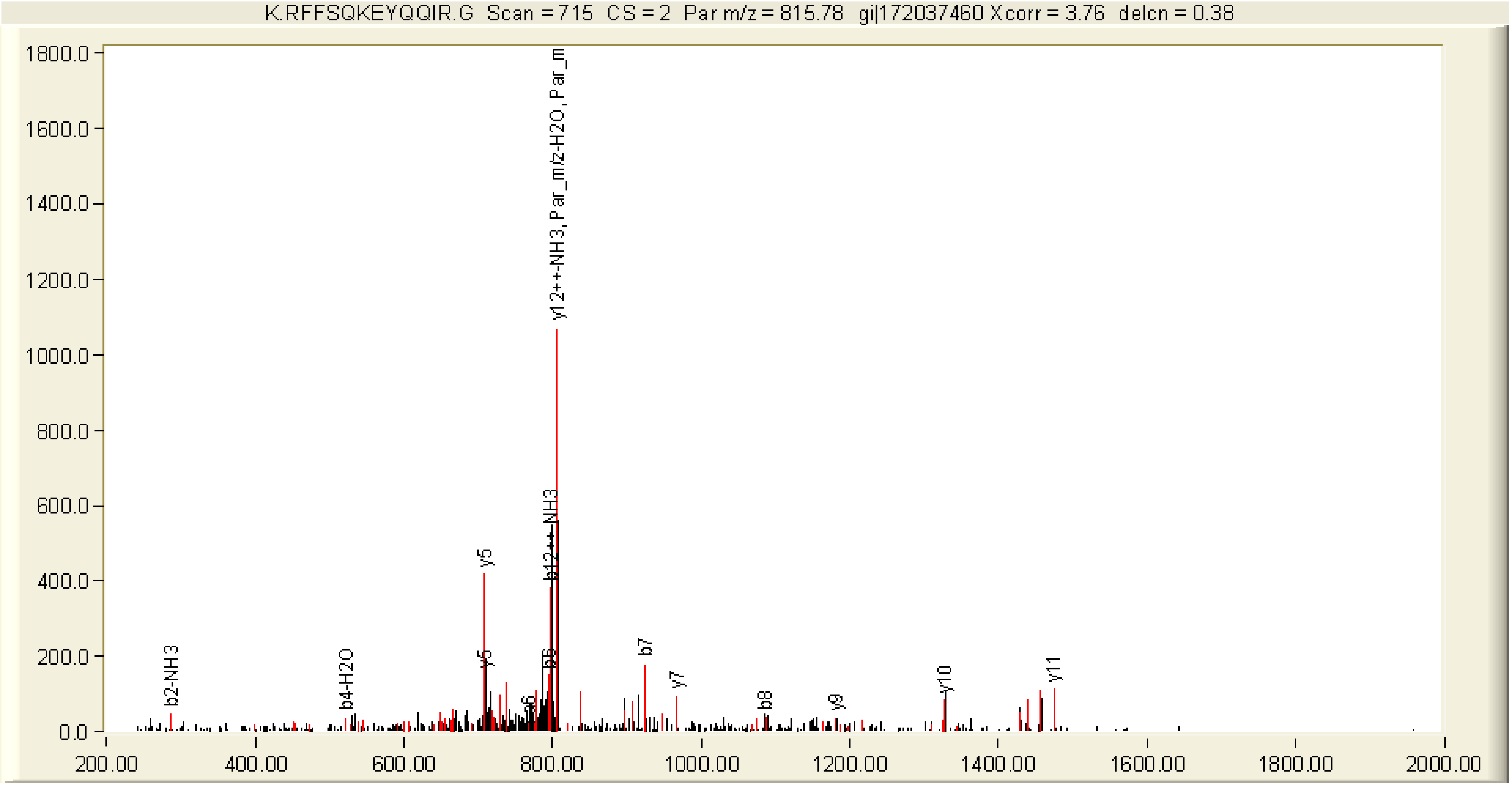

K.RFFSQKEYQQIR.G Scan = 715 CS = 2 Par m/z = 815.78 gi|172037460 Xcorr = 3.76 delcn = 0.38
y12++-NH3, Par_m/z-H2O, Par_m
b12++-NH3
y5
b2-NH3
b4-H2O
b7
y7
b8
y9
y10
y11
0.0
200.0
400.0
600.0
800.0
1000.0
1200.0
1400.0
1600.0
1800.0
200.00
400.00
600.00
800.00
1000.00
1200.00
1400.00
1600.00
1800.00
2000.00

K.RPLTNKINLVR.P Scan = 717 CS = 2 Par m/z = 663.83 gi|172037514 Xcorr = 2.78 delcn = 0.21

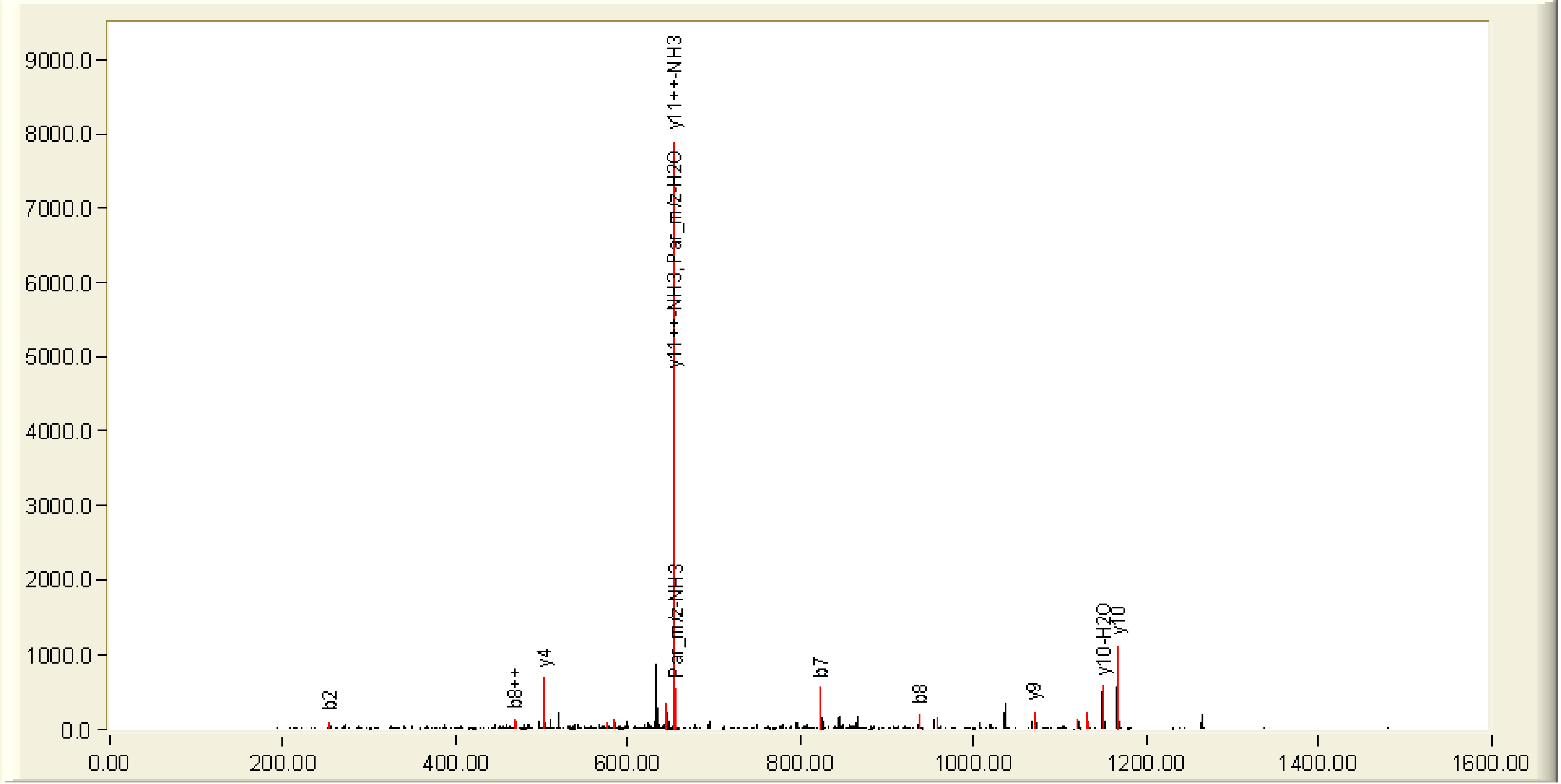

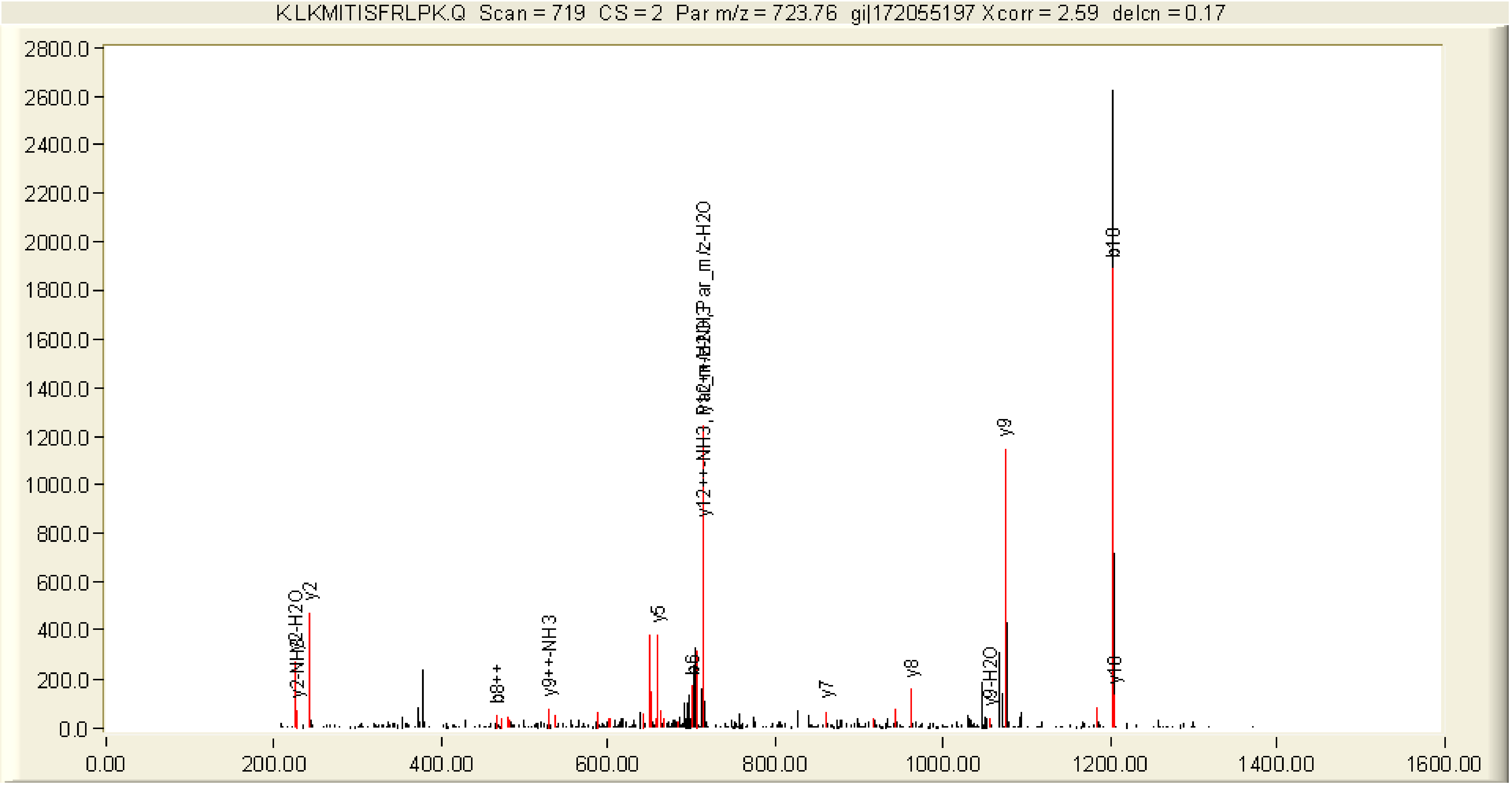

KLLKMITISFRLPK.Q Scan = 719 CS = 2 Par m/z = 723.76 gi|172055197 Xcorr = 2.59 delcn = 0.17
y2-NH3
y2-H2O
y2
b8++
y9++-NH3
y5
b6
y12++-NH3, Par_m/z-H2O
y7
y8
y9-H2O
y9
b10
y10
0.00
200.00
400.00
600.00
800.00
1000.00
1200.00
1400.00
1600.00
0.0
200.0
400.0
600.0
800.0
1000.0
1200.0
1400.0
1600.0
1800.0
2000.0
2200.0
2400.0
2600.0
2800.0

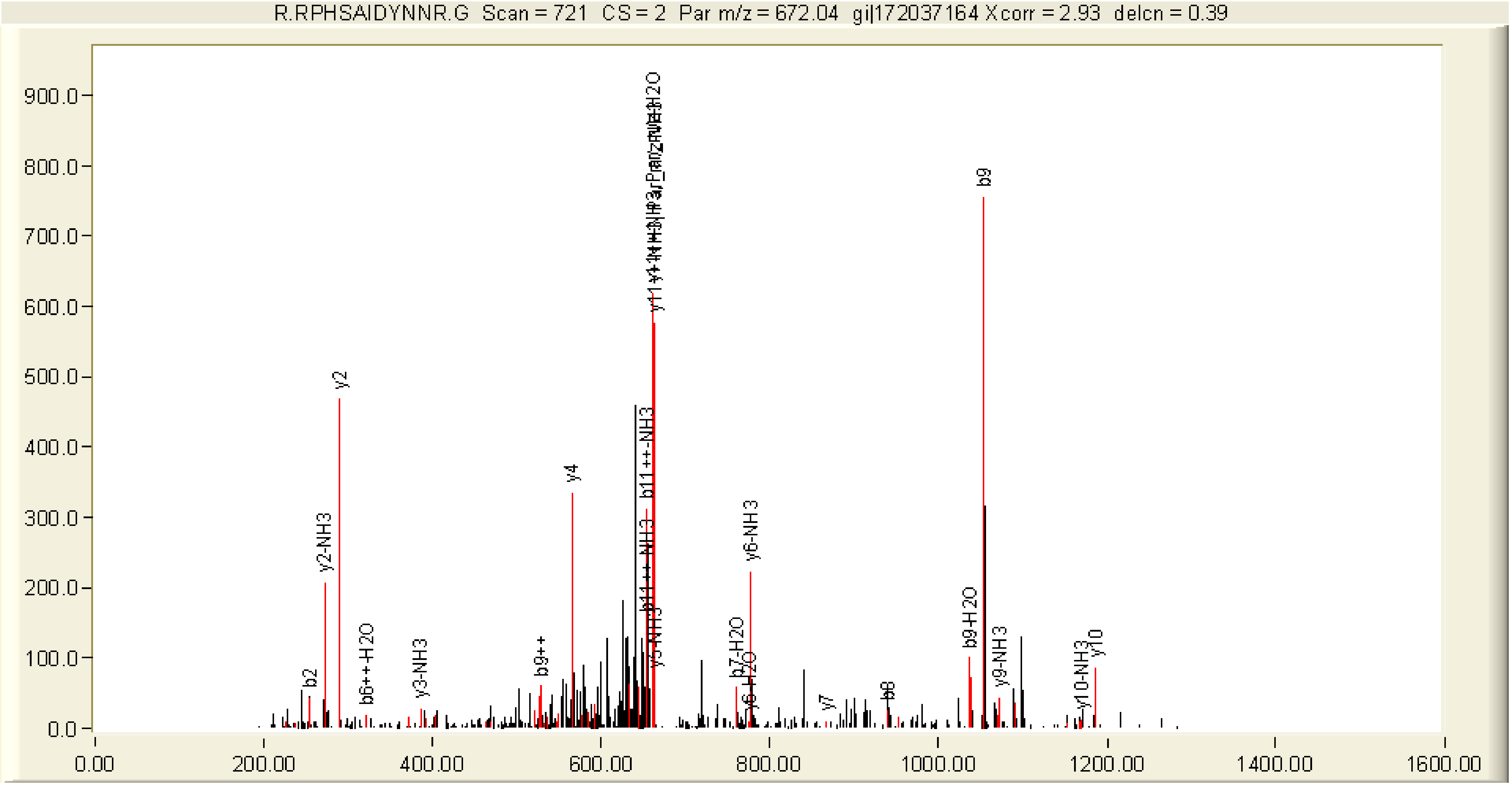

R.RPHSAIDYNNR.G Scan = 721 CS = 2 Par m/z = 672.04 gi|172037164 Xcorr = 2.93 delcn = 0.39
900.0
800.0
700.0
600.0
500.0
400.0
300.0
200.0
100.0
0.0
0.00
200.00
400.00
600.00
800.00
1000.00
1200.00
1400.00
1600.00
b2
y2-NH3
y2
b6++-H2O
y3-NH3
b9++
y4
b11++-NH3
y6-NH3
b7-H2O
y7
b8
b9-H2O
b9
y9-NH3
y10-NH3
y10

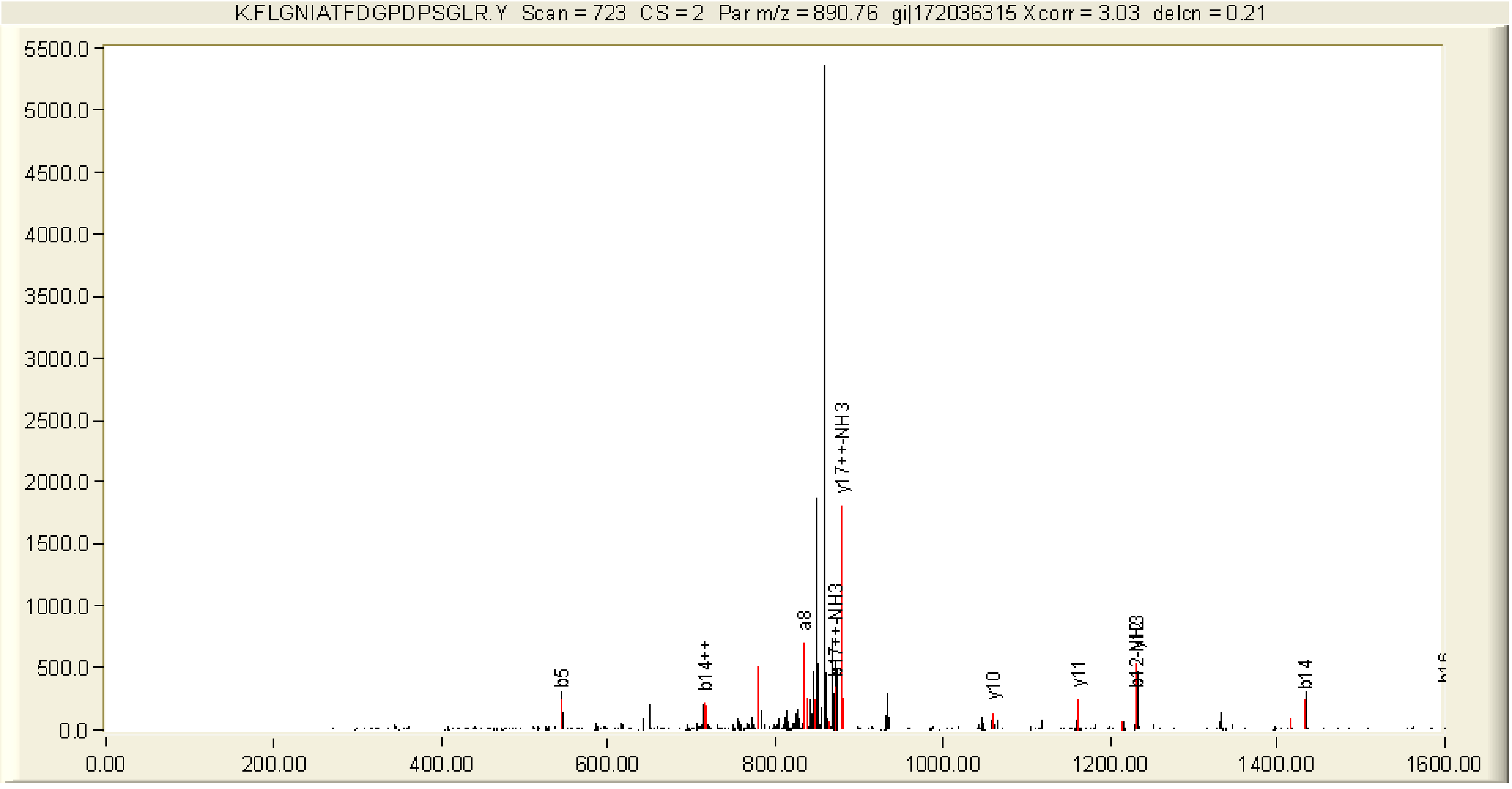

K.FLGNIATFDGPDPSGLR.Y Scan = 723 CS = 2 Par m/z = 890.76 gi|172036315 Xcorr = 3.03 delcn = 0.21
5500.0
5000.0
4500.0
4000.0
3500.0
3000.0
2500.0
2000.0
1500.0
1000.0
500.0
0.0
0.00
200.00
400.00
600.00
800.00
1000.00
1200.00
1400.00
1600.00
b5
b14++
a8
y17++-NH3
y10
y11
b14

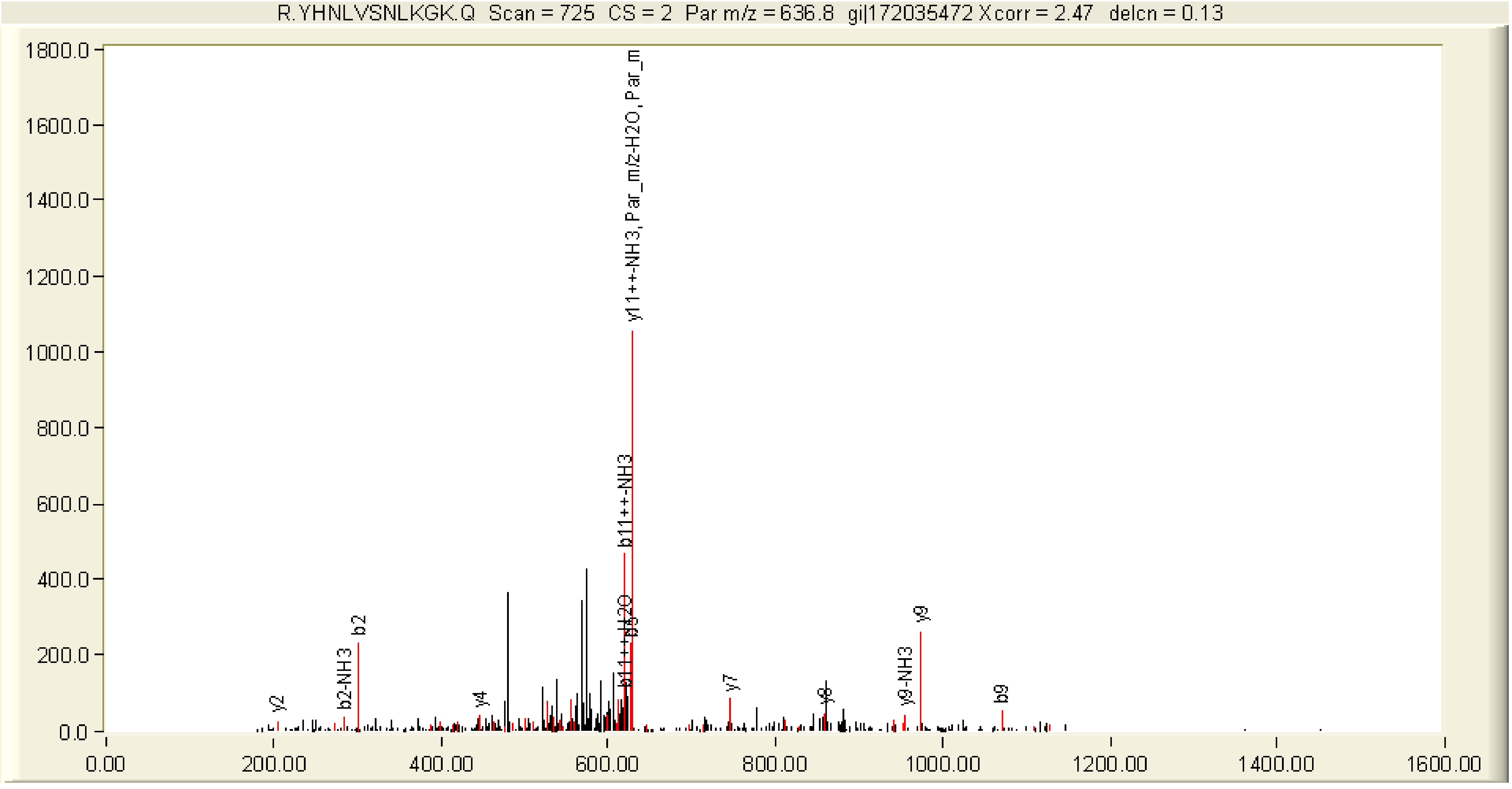
R.YHNLVSNLKGK.Q Scan = 725 CS = 2 Par m/z = 636.8 gi|172035472 Xcorr = 2.47 delcn = 0.13
y11++-NH3, Par_m/z-H2O, Par_m
b11++-NH3
b11++-H2O
b5
b2
b2-NH3
y2
y4
y7
y8
y9-NH3
y9
b9
1800.0
1600.0
1400.0
1200.0
1000.0
800.0
600.0
400.0
200.0
0.0
0.00
200.00
400.00
600.00
800.00
1000.00
1200.00
1400.00
1600.00

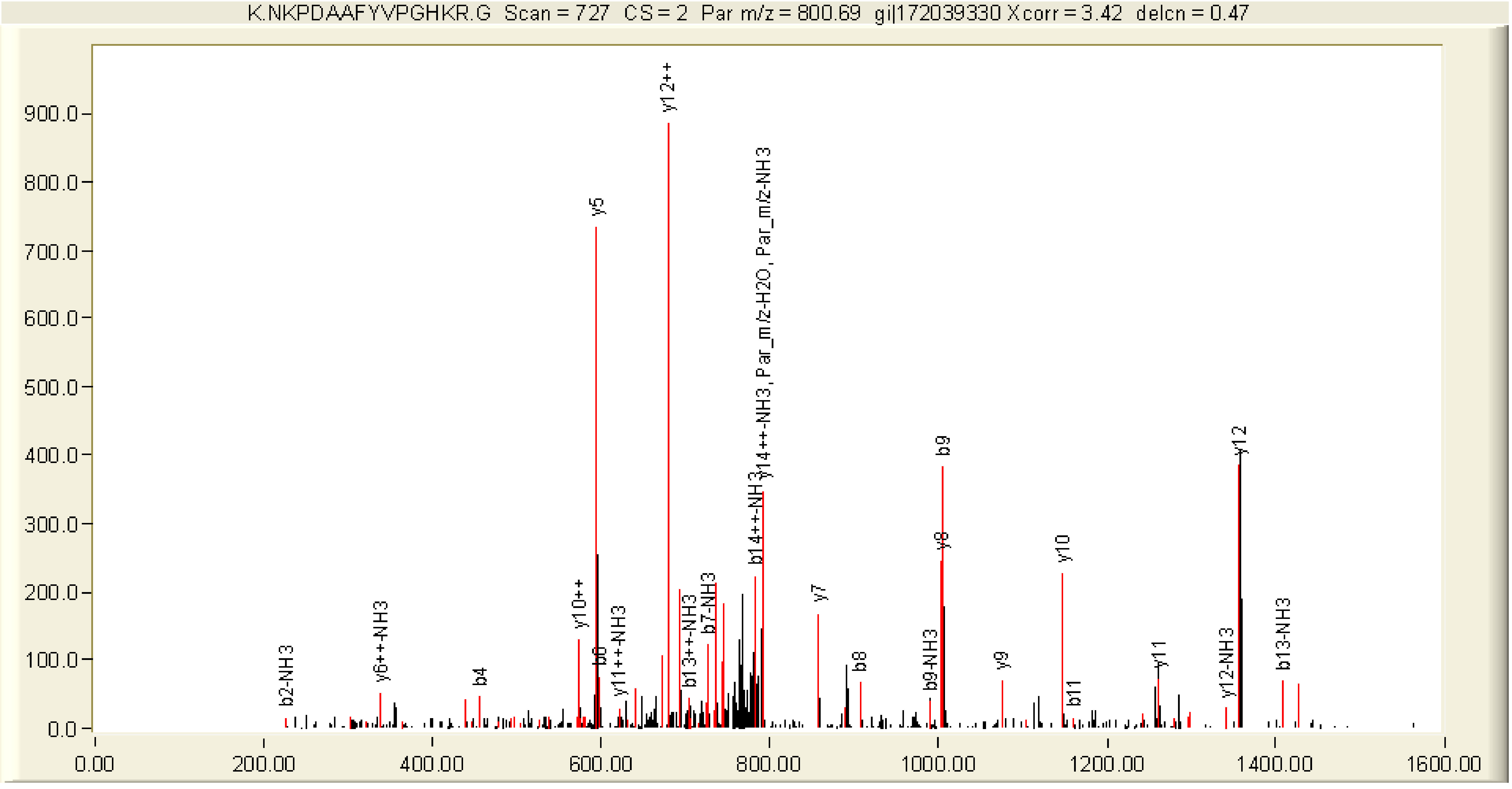
K.NKPDAAFYVPGHKR.G Scan = 727 CS = 2 Par m/z = 800.69 gi|172039330 Xcorr = 3.42 delcn = 0.47
900.0
800.0
700.0
600.0
500.0
400.0
300.0
200.0
100.0
0.0
0.00
200.00
400.00
600.00
800.00
1000.00
1200.00
1400.00
1600.00
y12++
y5
y14++-NH3, Par_m/z-H2O, Par_m/z-NH3
b14++-NH3
b9
y12
y8
y10
y7
y10++
b7-NH3
b13++-NH3
y11++-NH3
y6++-NH3
b2-NH3
b4
b6
b8
b9-NH3
y9
b11
y11
y12-NH3
b13-NH3

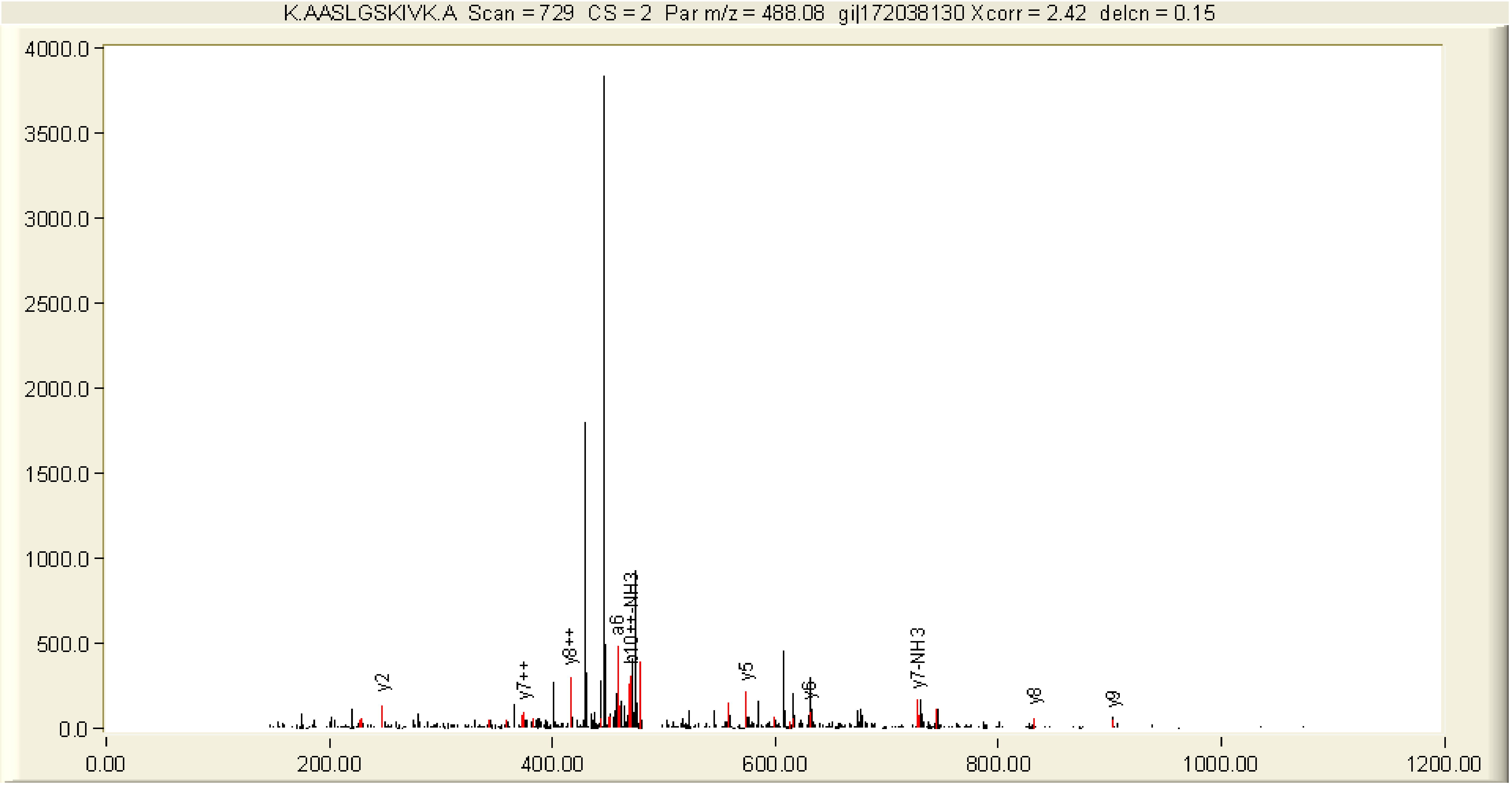
K.AASLGSKIVK.A Scan = 729 CS = 2 Par m/z = 488.08 gi|172038130 Xcorr = 2.42 delcn = 0.15
4000.0
3500.0
3000.0
2500.0
2000.0
1500.0
1000.0
500.0
0.0
0.00
200.00
400.00
600.00
800.00
1000.00
1200.00
y2
y7++
y8++
a6
b10++-NH3
y5
y6
y7-NH3
y8
y9

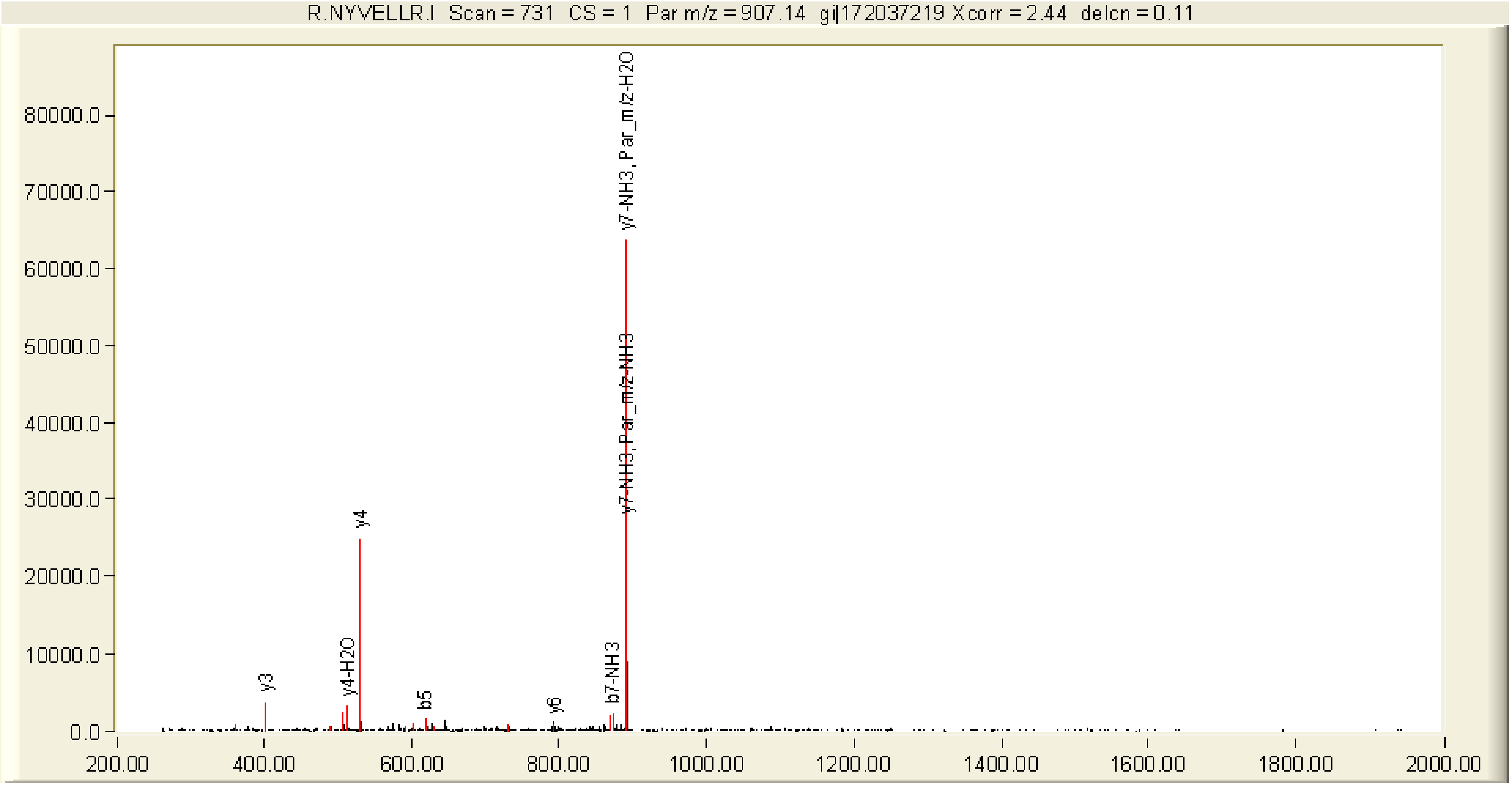
R.NYVELLR.I Scan = 731 CS = 1 Par m/z = 907.14 gi|172037219 Xcorr = 2.44 delcn = 0.11
y7-NH3, Par_m/z-H2O
y7-NH3, Par_m/z-NH3
y4
y4-H2O
y3
b5
y6
b7-NH3
80000.0
70000.0
60000.0
50000.0
40000.0
30000.0
20000.0
10000.0
0.0
200.00
400.00
600.00
800.00
1000.00
1200.00
1400.00
1600.00
1800.00
2000.00

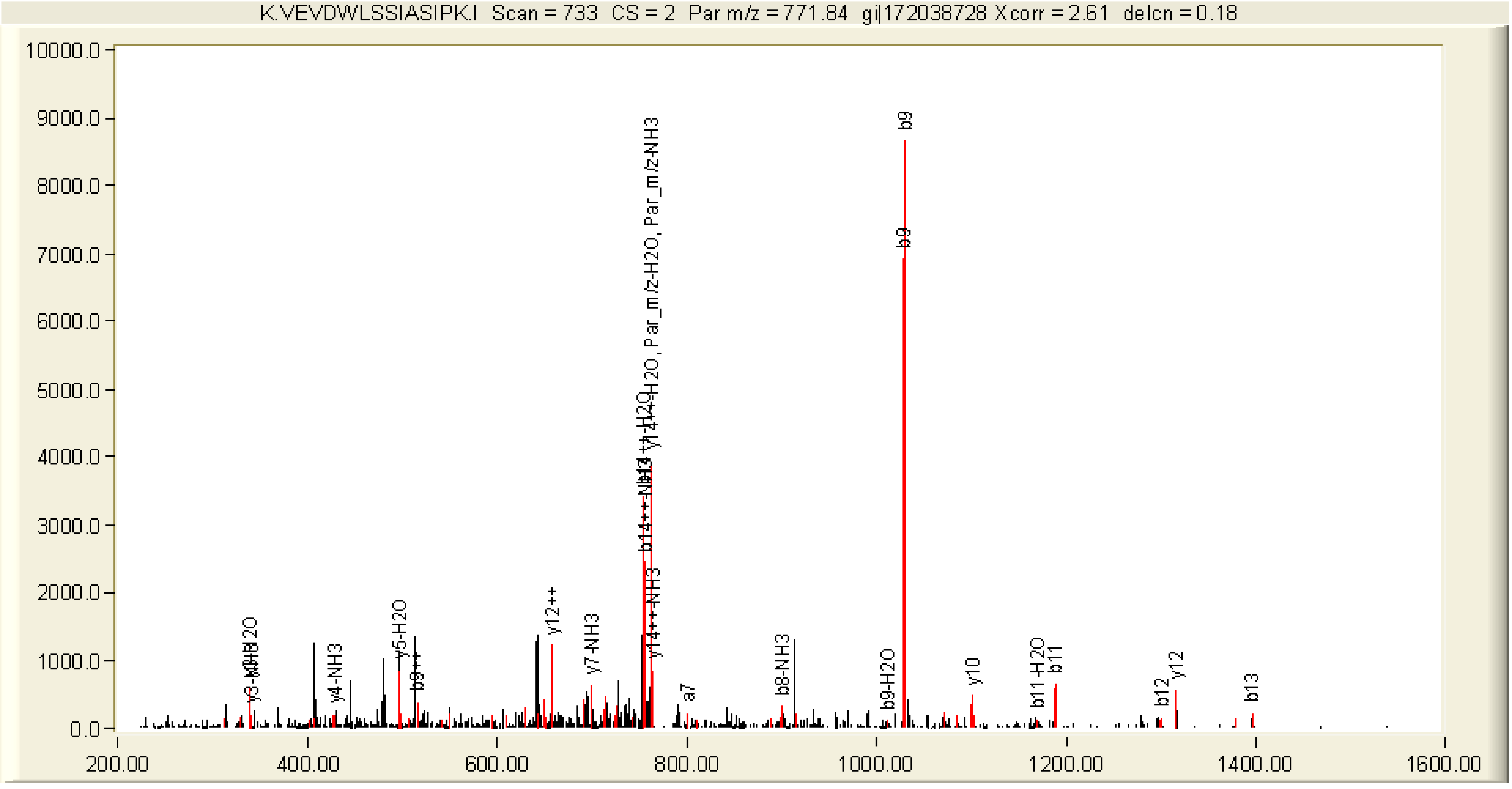

K.VEVDWLSSIASIPK.I Scan = 733 CS = 2 Par m/z = 771.84 gi|172038728 Xcorr = 2.61 delcn = 0.18
10000.0
9000.0
8000.0
7000.0
6000.0
5000.0
4000.0
3000.0
2000.0
1000.0
0.0
200.00
400.00
600.00
800.00
1000.00
1200.00
1400.00
1600.00
y4-NH3
y5-H2O
y12++
y7-NH3
Par_m/z-H2O, Par_m/z-NH3
b14++-NH3
y14++-NH3
a7
b8-NH3
b9-H2O
b9
b9
y10
b11-H2O
b11
b12
y12
b13

R.TEFRYGKFHRVIPLPSR.V Scan = 735 CS = 2 Par m/z = 1053.37 gi|172039610 Xcorr = 2.82 delcn = 0.14

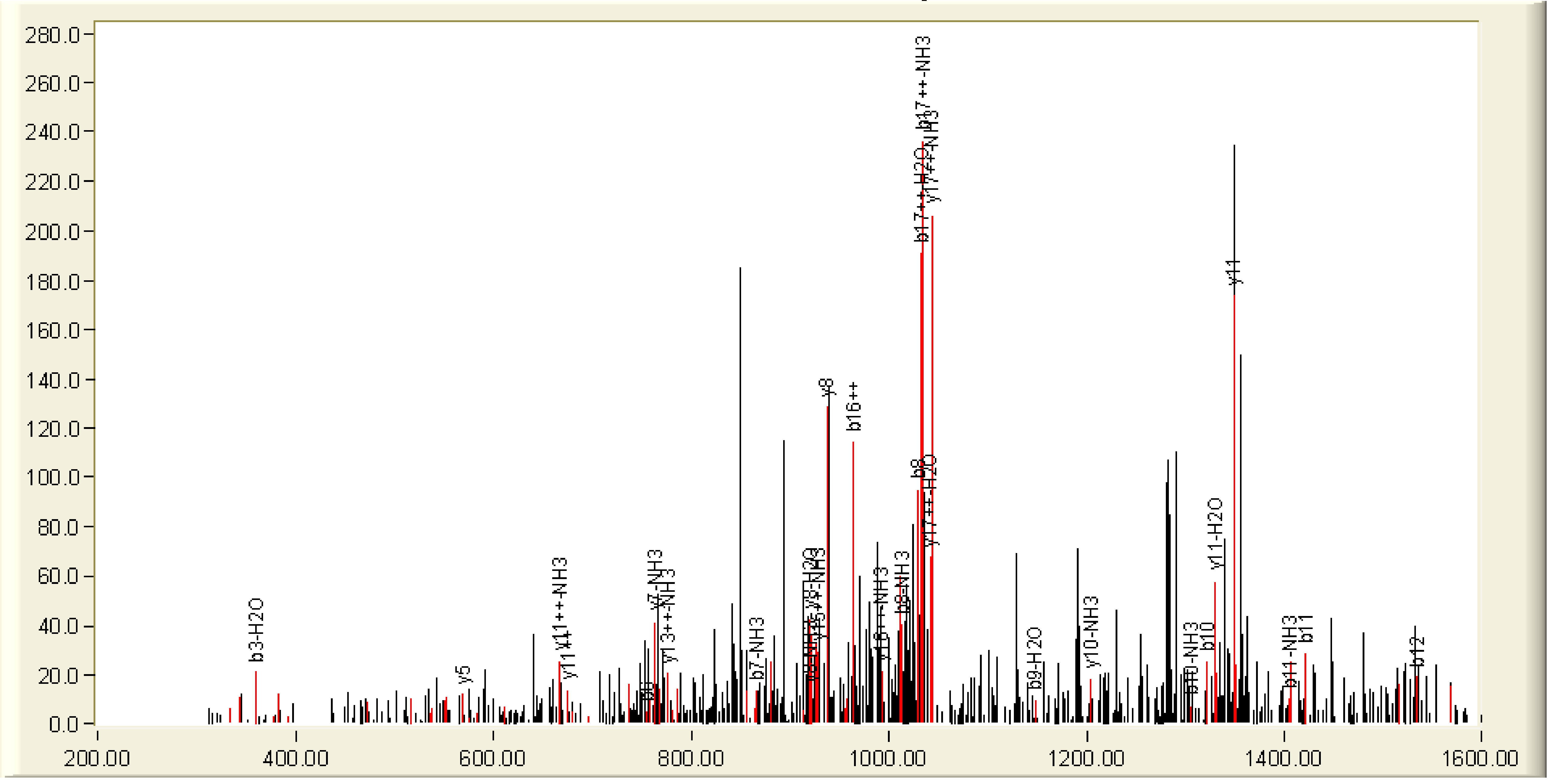

K.ASVKERKAYYEYFKRTANRNSEENEGRGN.- Scan = 737 CS = 3 Par m/z = 1157.17 gi|172055033 Xcorr = 3.29 delcn = 0.16

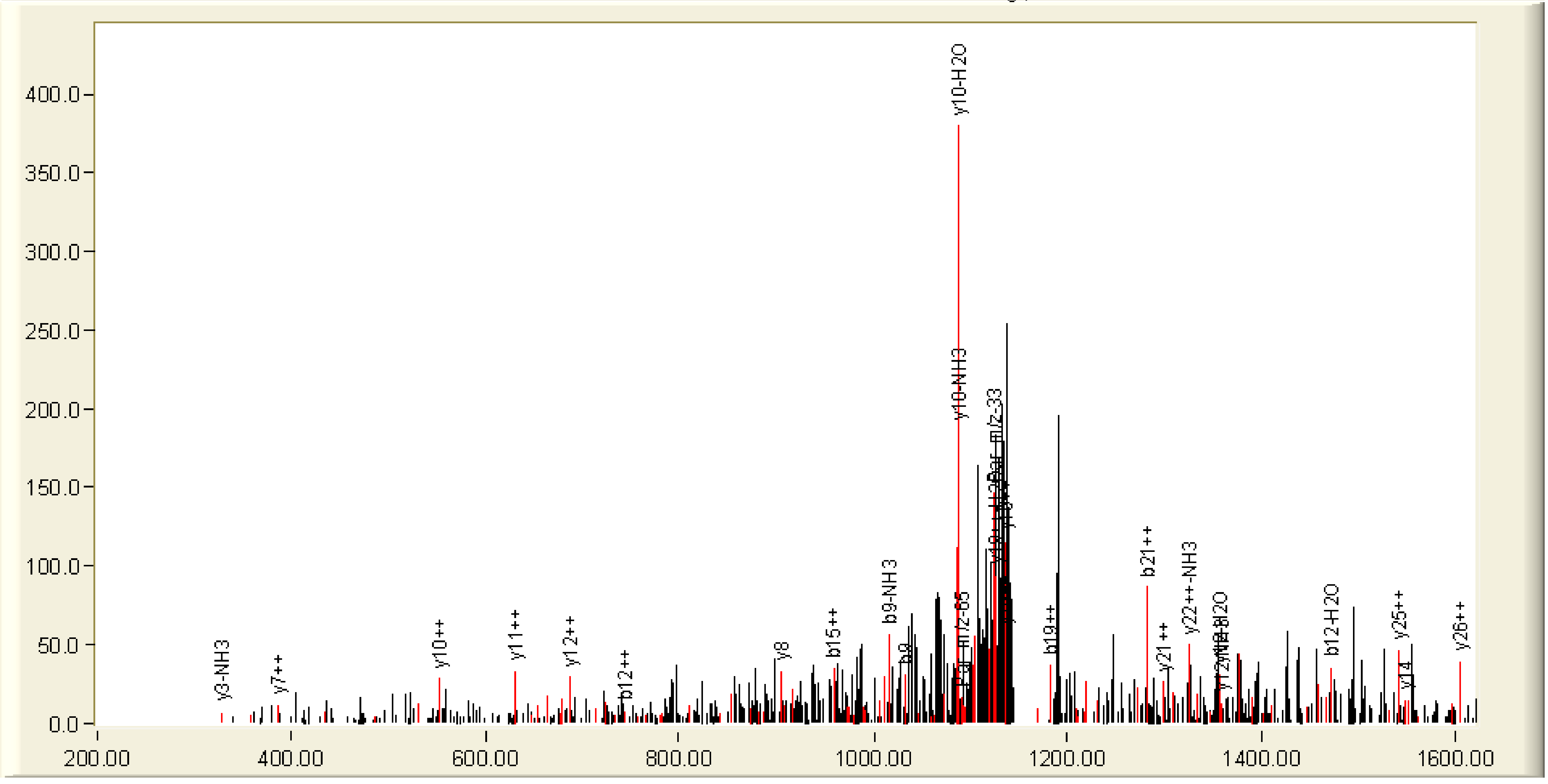

R.SHSAFLNGYSPEDEGLYDDY.- Scan = 739 CS = 2 Par m/z = 1140.13 gi|172037756 Xcorr = 2.46 delcn = 0.31

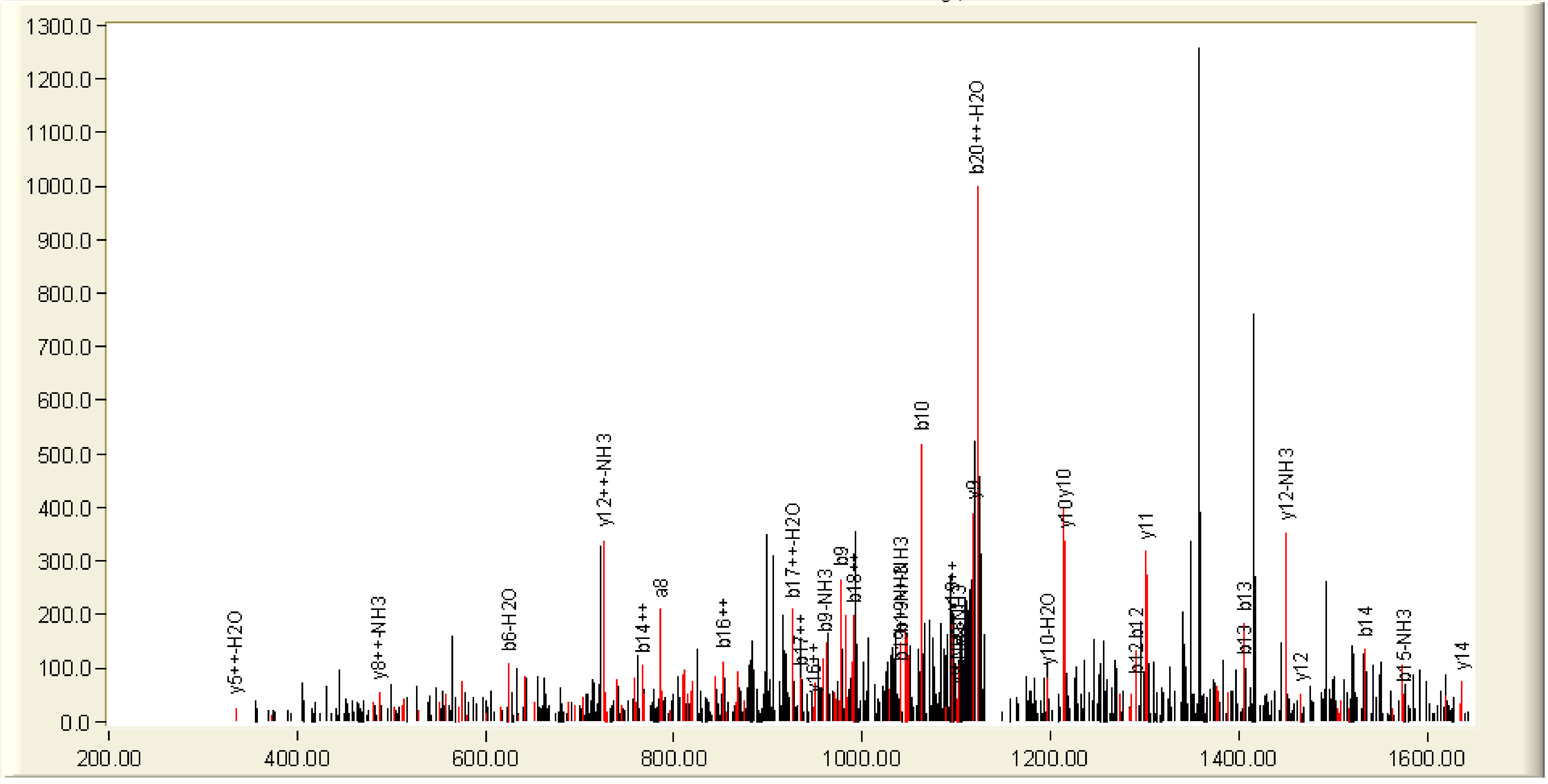

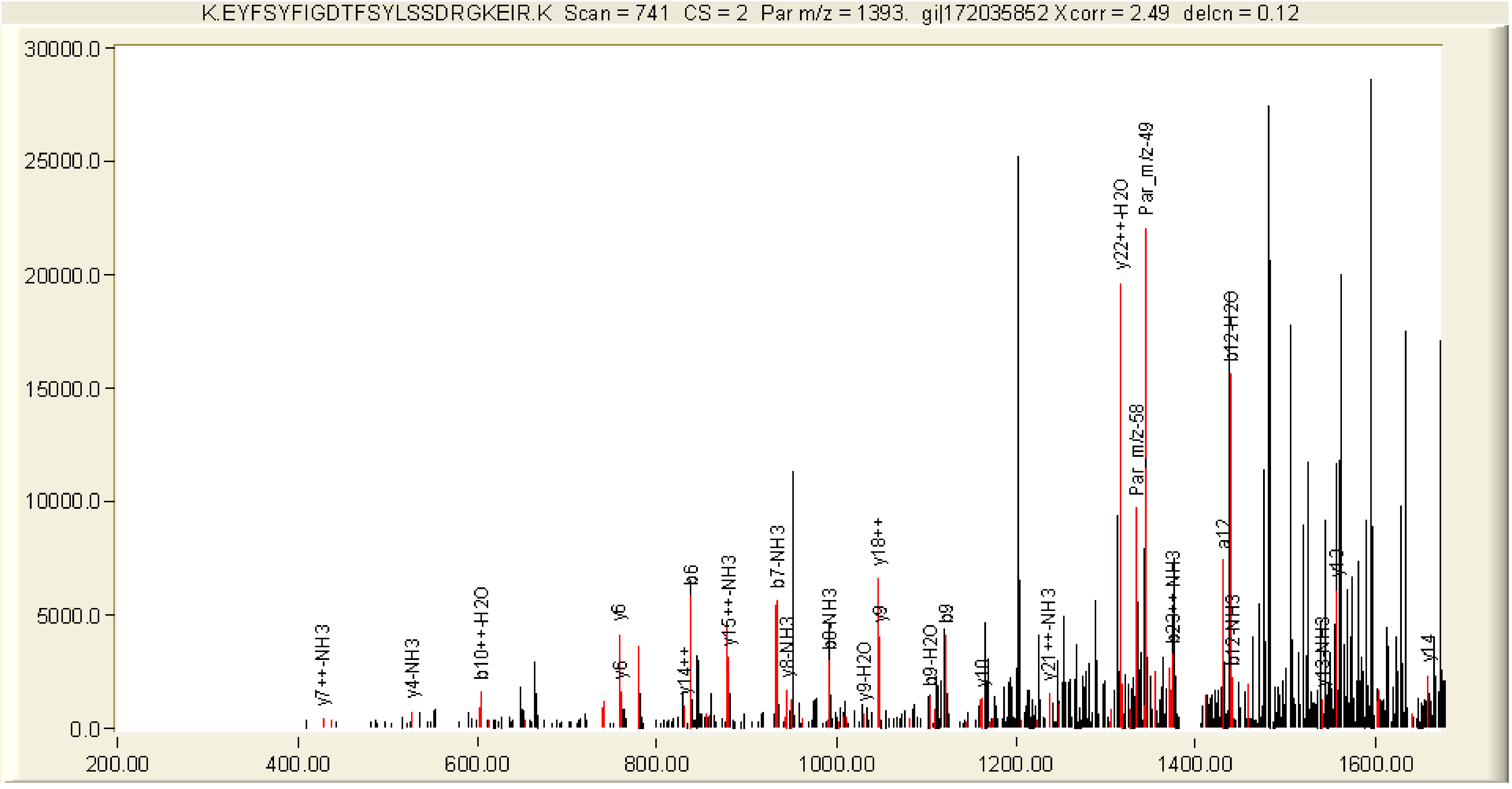

K.EYFSYFIGDTFSYLSSDRGKEIR.K Scan = 741 CS = 2 Par m/z = 1393. gi|172035852 Xcorr = 2.49 delcn = 0.12
30000.0
25000.0
20000.0
15000.0
10000.0
5000.0
0.0
200.00
400.00
600.00
800.00
1000.00
1200.00
1400.00
1600.00
y7++-NH3
y4-NH3
b10++-H2O
y6
y6
y14++
b6
y15++-NH3
b7-NH3
y8-NH3
b8-NH3
y9-H2O
y18++
y9
b9-H2O
b9
y10
y21++-NH3
y22++-H2O
Par_m/z-58
Par_m/z-49
b23++-NH3
a12
b12-H2O
b12-NH3
y13-NH3
y13
y14

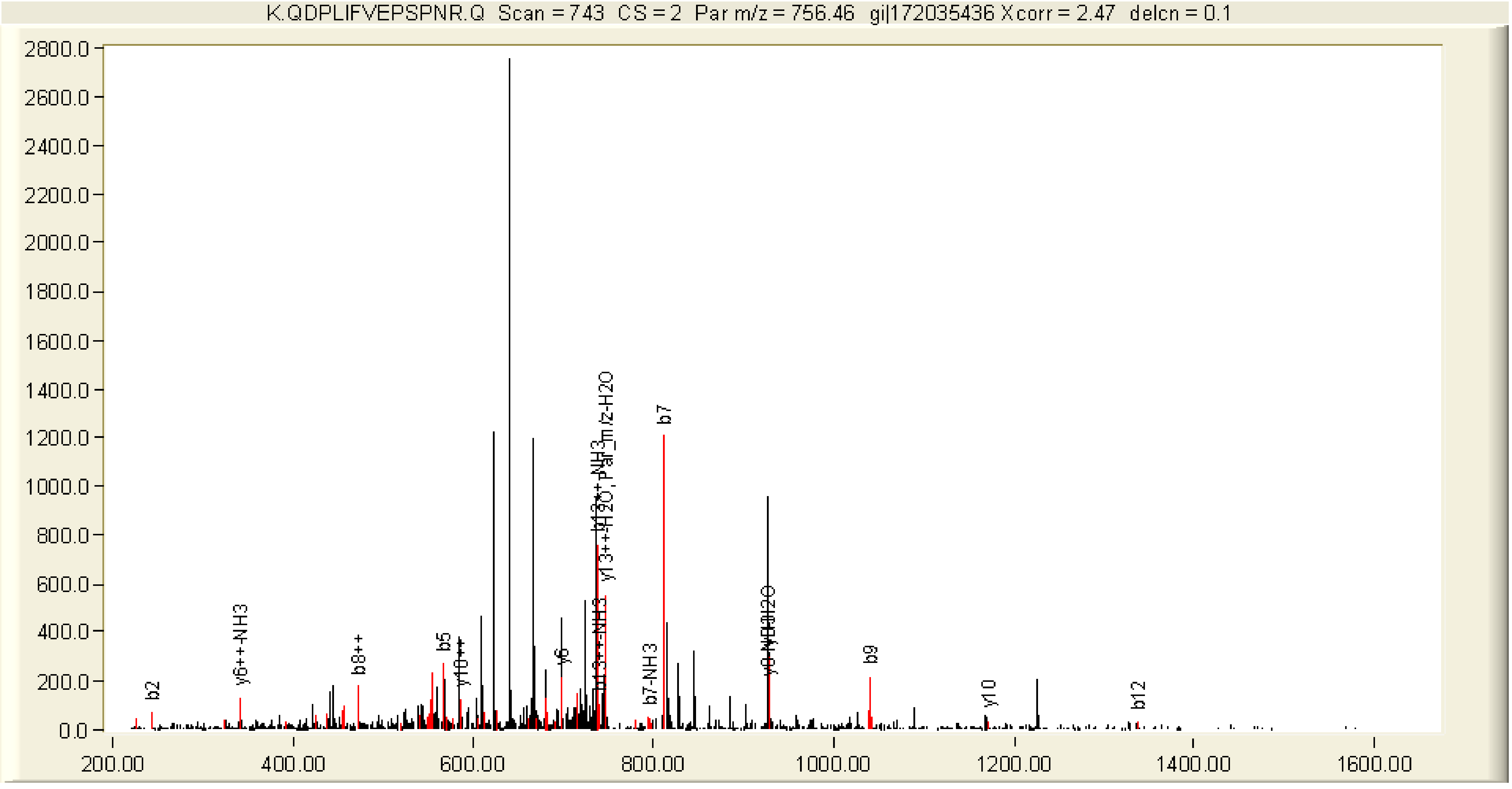
K.QDPLIFVEPSPNR.Q Scan = 743 CS = 2 Par m/z = 756.46 gi|172035436 Xcorr = 2.47 delcn = 0.1
2800.0
2600.0
2400.0
2200.0
2000.0
1800.0
1600.0
1400.0
1200.0
1000.0
800.0
600.0
400.0
200.0
0.0
200.00
400.00
600.00
800.00
1000.00
1200.00
1400.00
1600.00
b2
y6++-NH3
b8++
b5
y10++
y6
b7-NH3
b7
b9
y10
b12

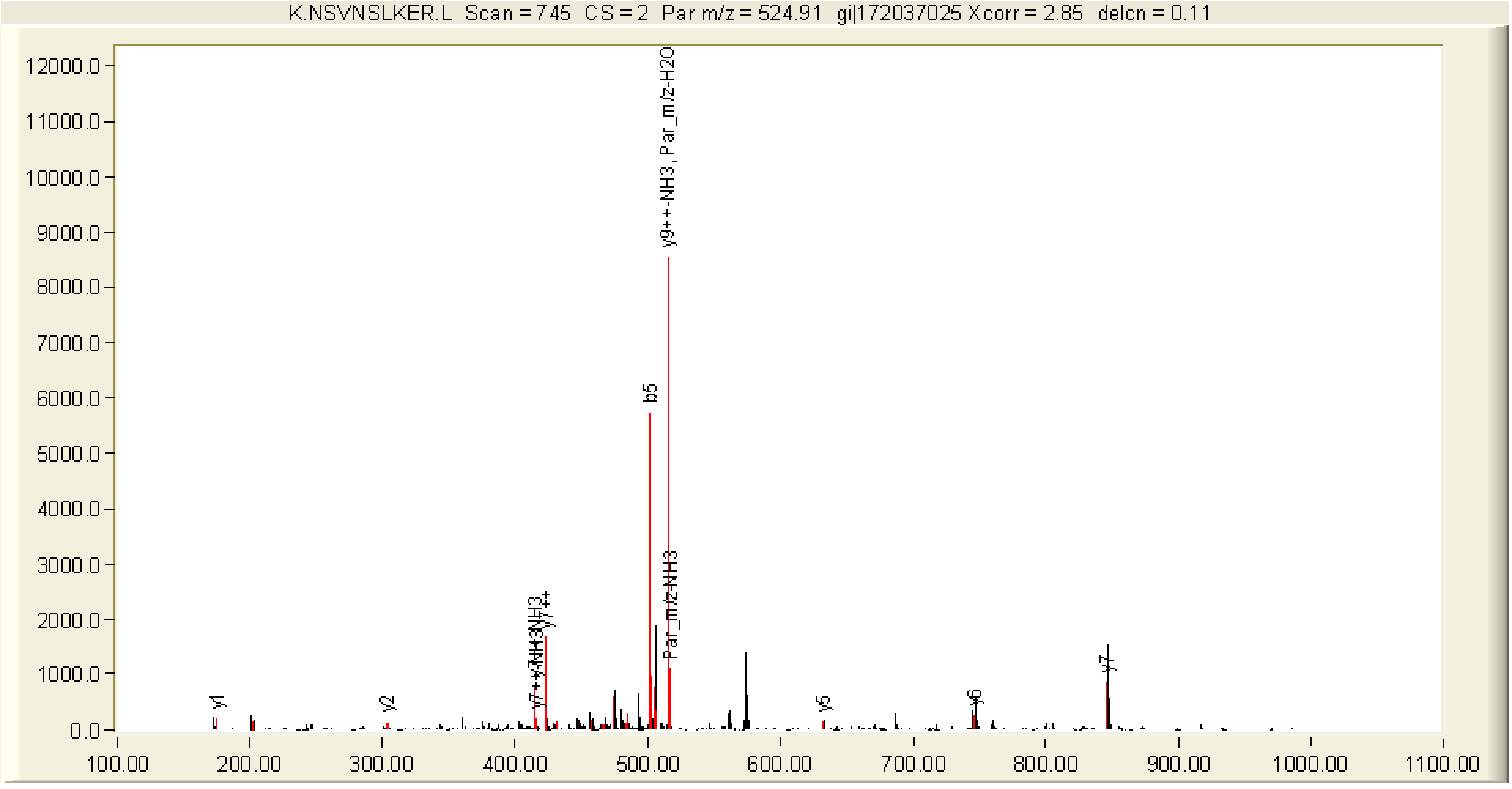
K.NSVNSLKER.L Scan = 745 CS = 2 Par m/z = 524.91 gi|172037025 Xcorr = 2.85 delcn = 0.11
12000.0
11000.0
10000.0
9000.0
8000.0
7000.0
6000.0
5000.0
4000.0
3000.0
2000.0
1000.0
0.0
100.00
200.00
300.00
400.00
500.00
600.00
700.00
800.00
900.00
1000.00
1100.00
y1
y2
y7++
b5
y9++-NH3, Par_m/z-H2O
Par_m/z-NH3
y5
y6
y7

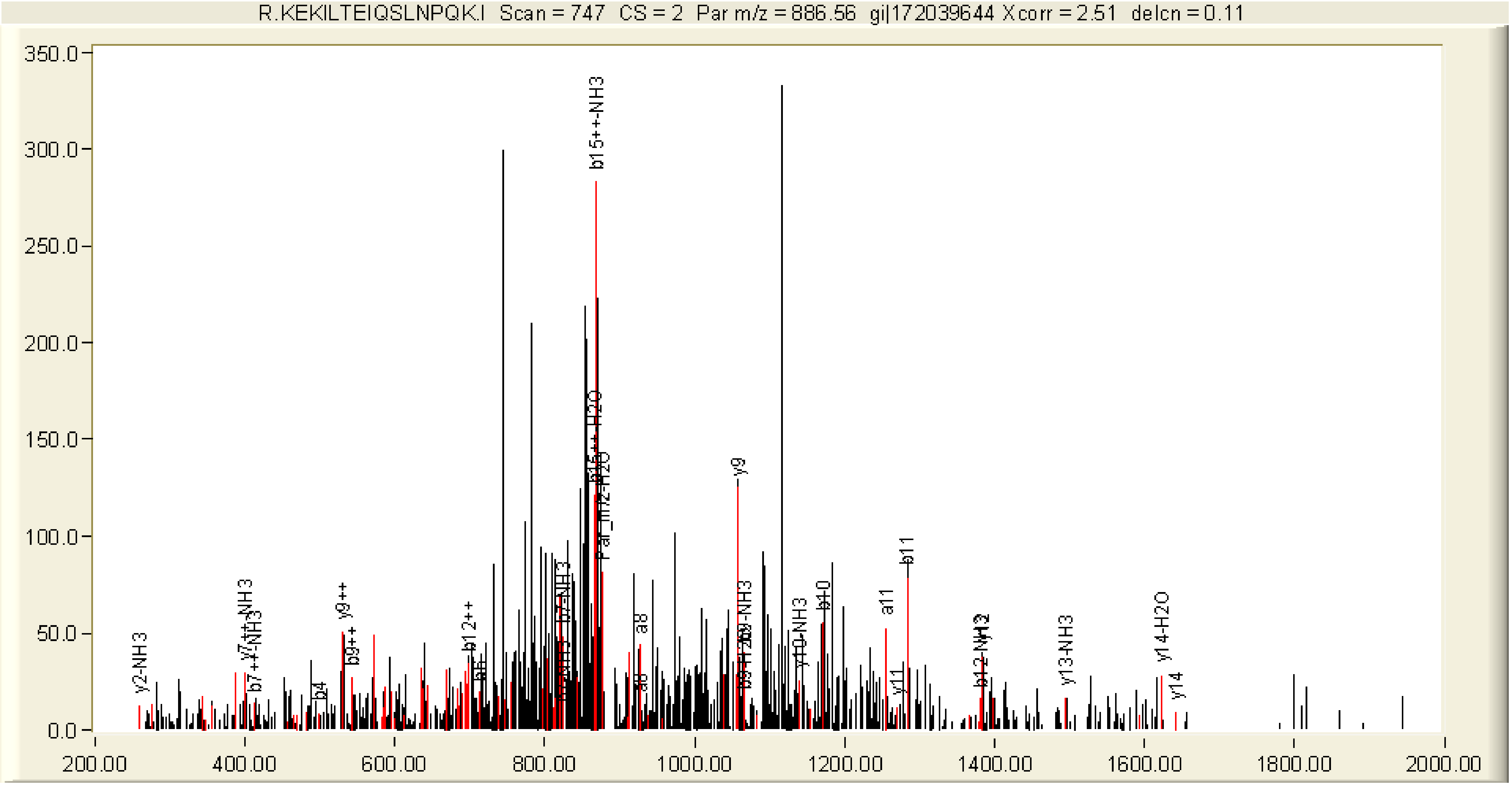

R.KEKILTEIQSLNPQK.I Scan = 747 CS = 2 Par m/z = 886.56 gi|172039644 Xcorr = 2.51 delcn = 0.11
350.0
300.0
250.0
200.0
150.0
100.0
50.0
0.0
200.00
400.00
600.00
800.00
1000.00
1200.00
1400.00
1600.00
1800.00
2000.00
b15++-NH3
y9
b11
a11
b10
a8
y9++
b9++
b12++
y2-NH3
b4
y11
y13-NH3
y14-H2O
y14

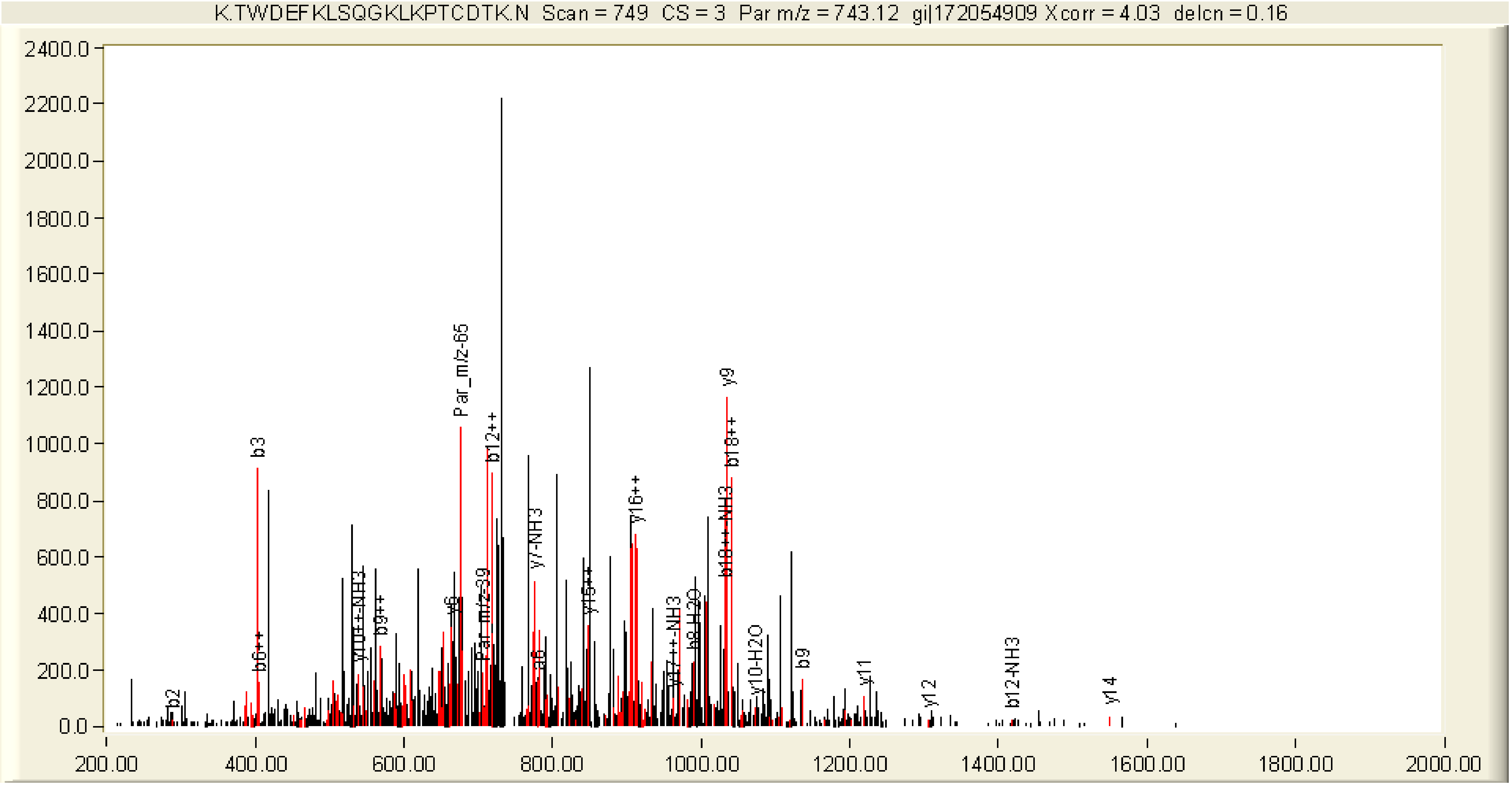

K.TWDEFKLSQGKLKPTCDTK.N Scan = 749 CS = 3 Par m/z = 743.12 gi|172054909 Xcorr = 4.03 delcn = 0.16
2400.0
2200.0
2000.0
1800.0
1600.0
1400.0
1200.0
1000.0
800.0
600.0
400.0
200.0
0.0
b2
b3
b6++
b9++
y6
Par_m/z-65
Par_m/z-39
b12++
a6
y7-NH3
y16++
y9
b18++
y10-H2O
b9
y11
y12
b12-NH3
y14
200.00
400.00
600.00
800.00
1000.00
1200.00
1400.00
1600.00
1800.00
2000.00

K.GYLFVLVLGLGLALHSLLVAYAVVIKVER.T Scan = 751 CS = 3 Par m/z = 1043.48 gi|172038449 Xcorr = 3.36 delcn = 0.18

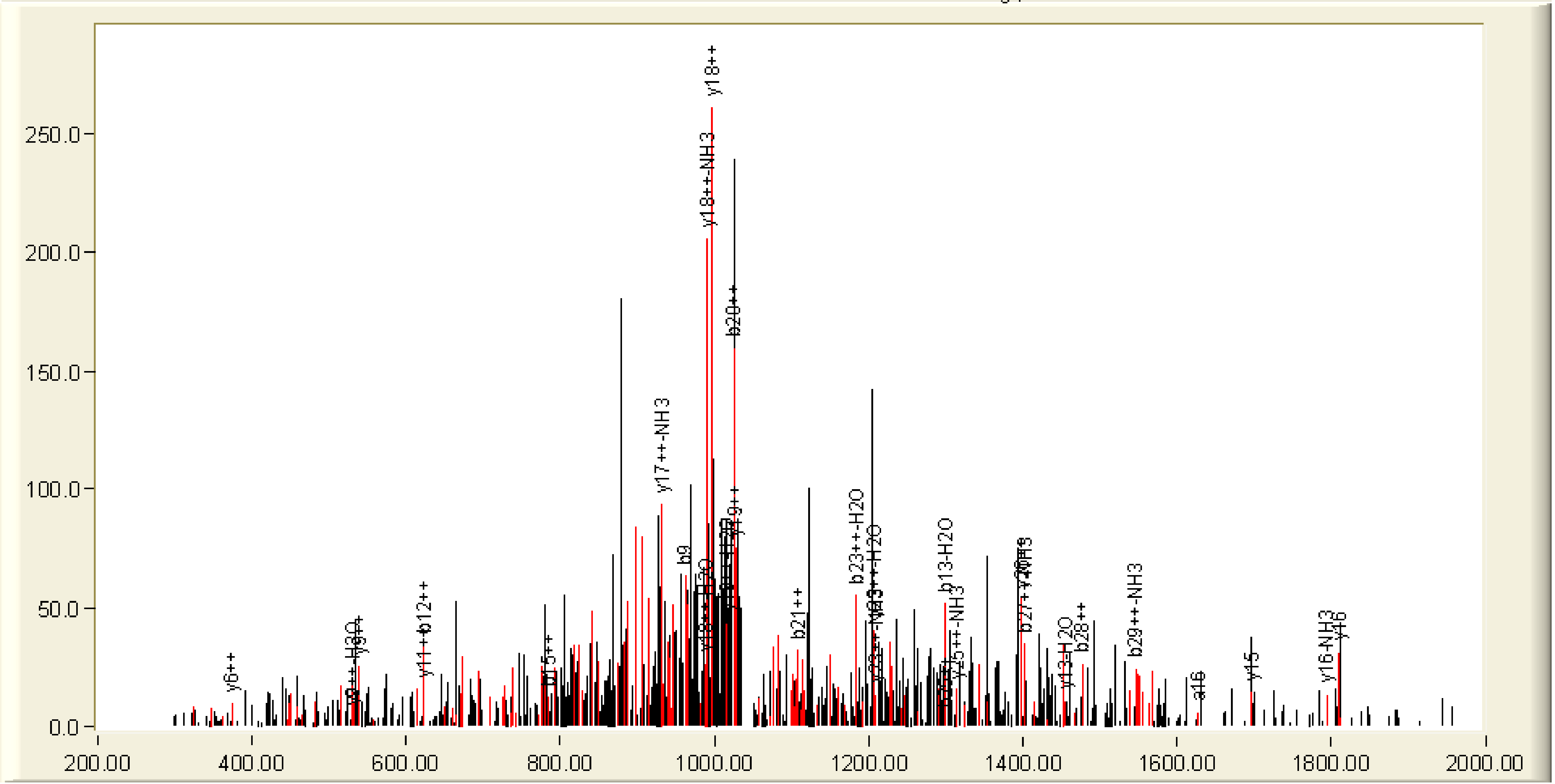

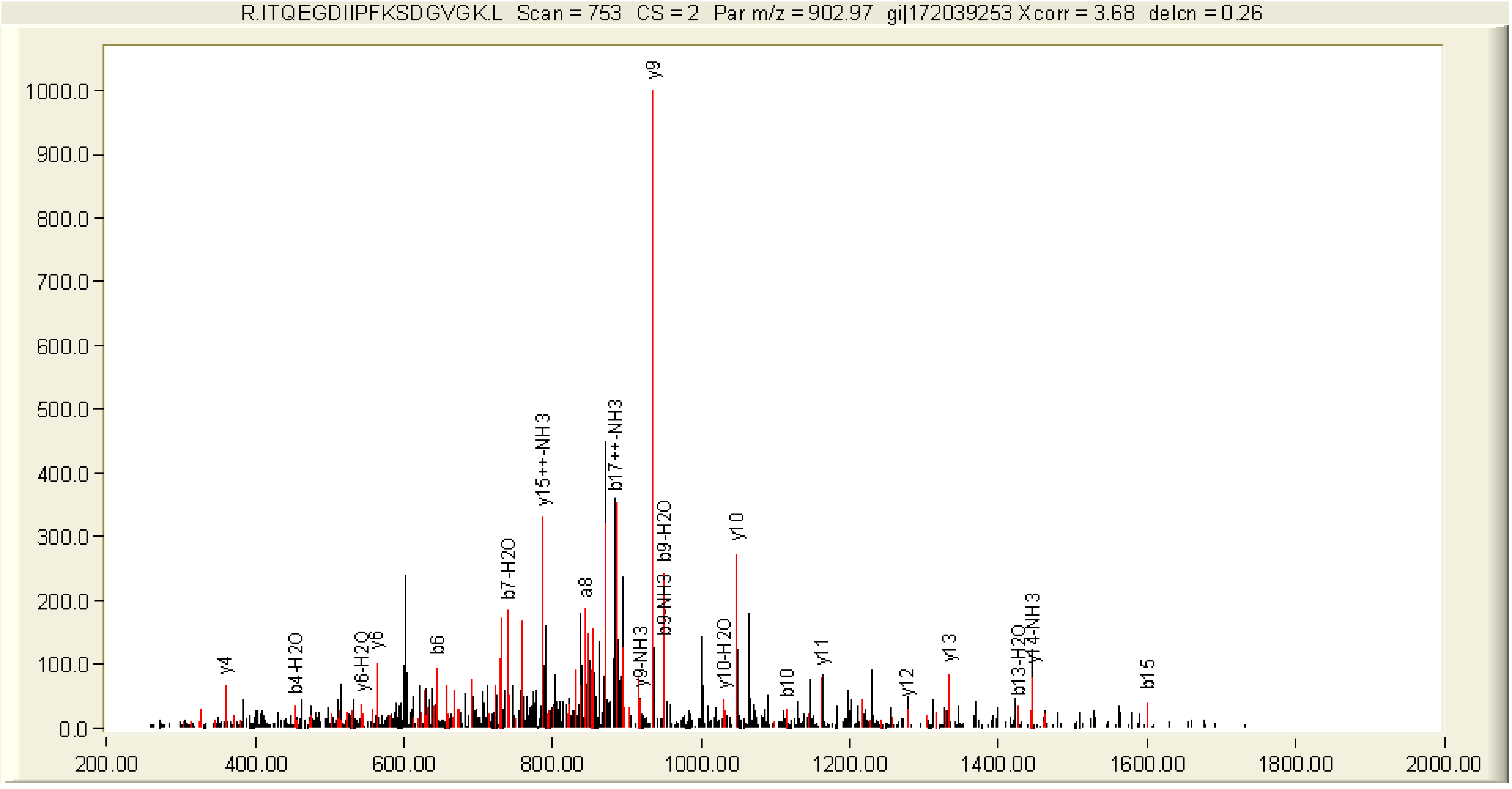
R.ITQEGDIIPFKSDGVGK.L Scan = 753 CS = 2 Par m/z = 902.97 gi|172039253 Xcorr = 3.68 delcn = 0.26
1000.0
900.0
800.0
700.0
600.0
500.0
400.0
300.0
200.0
100.0
0.0
200.00
400.00
600.00
800.00
1000.00
1200.00
1400.00
1600.00
1800.00
2000.00
y9
y4
b4-H2O
y6-H2O
y6
b6
b7-H2O
y15++-NH3
a8
b17++-NH3
y9-NH3
b9-NH3
b9-H2O
y10-H2O
y10
b10
y11
y12
y13
b13-H2O
y14-NH3
b15

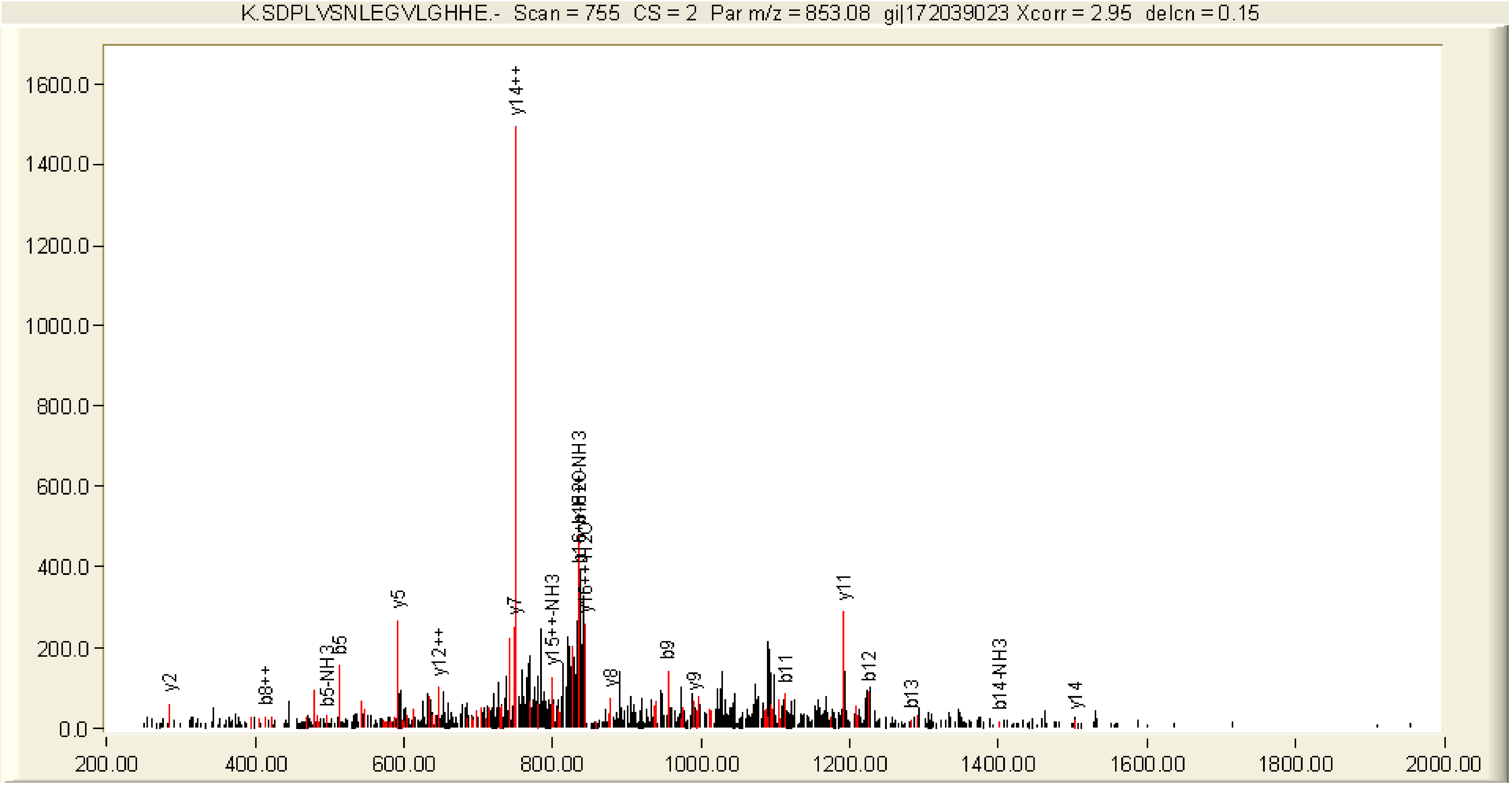
K.SDPLVSNLEGVLGHHE.- Scan = 755 CS = 2 Par m/z = 853.08 gi|172039023 Xcorr = 2.95 delcn = 0.15
1600.0
1400.0
1200.0
1000.0
800.0
600.0
400.0
200.0
0.0
y14++
y2
b8++
b5-NH3
b5
y5
y12++
y7
y15++-NH3
y8
b9
y9
b11
y11
b12
b13
b14-NH3
y14
200.00
400.00
600.00
800.00
1000.00
1200.00
1400.00
1600.00
1800.00
2000.00

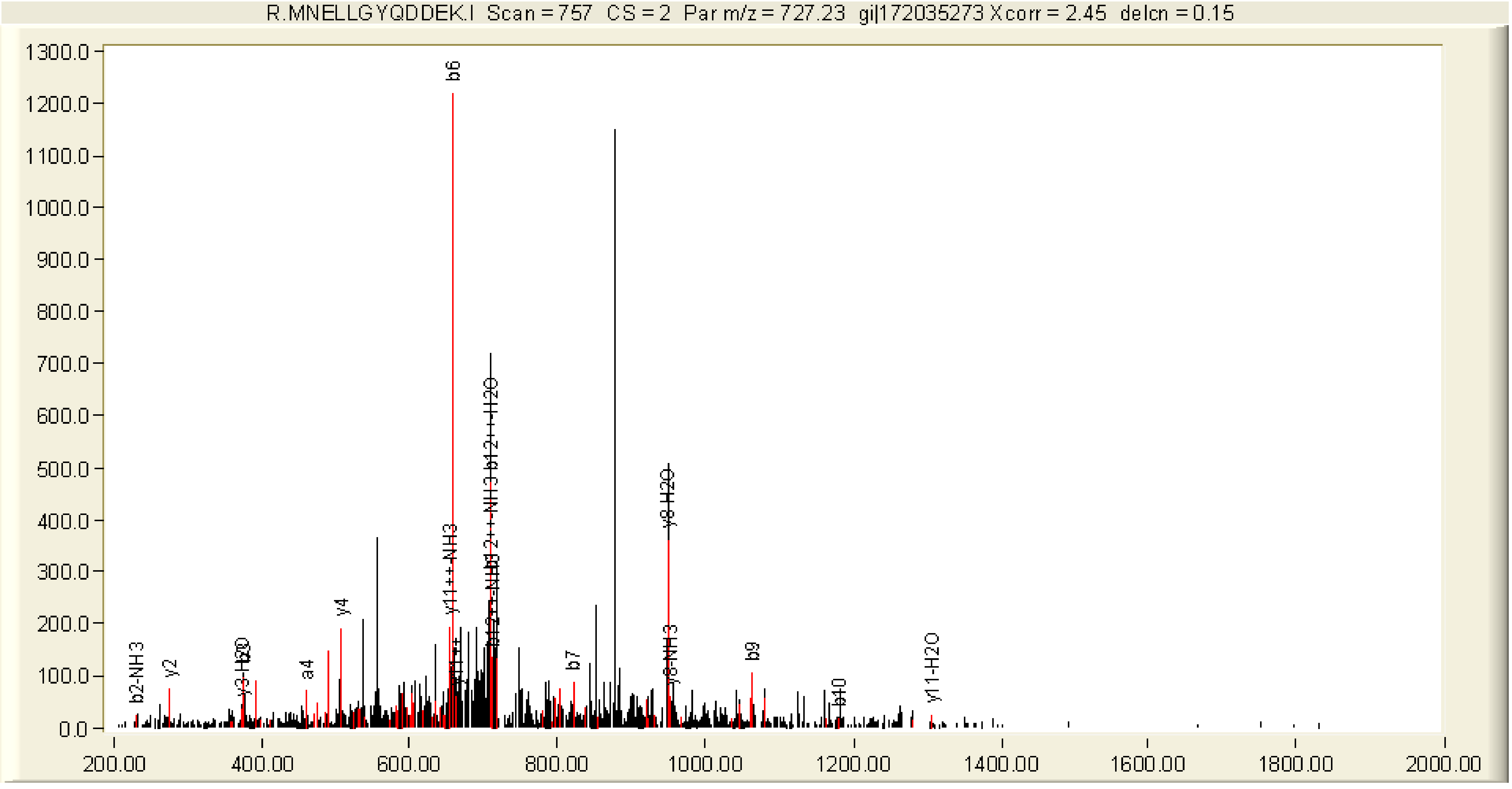
R.MNELLGYQDDEK.I Scan = 757 CS = 2 Par m/z = 727.23 gi|172035273 Xcorr = 2.45 delcn = 0.15
b6
y8-H2O
b7
b9
y11-H2O
b10
y8-NH3
y4
a4
y2
b2-NH3
y11++-NH3
0.0
100.0
200.0
300.0
400.0
500.0
600.0
700.0
800.0
900.0
1000.0
1100.0
1200.0
1300.0
200.00
400.00
600.00
800.00
1000.00
1200.00
1400.00
1600.00
1800.00
2000.00

R.NRLPNGSYLYGSSPQPDEIGQEYMVFQVHQGEVK.G Scan = 759 CS = 3 Par m/z = 1290.66 gi|172035121 Xcorr = 3.37 delcn = 0.17

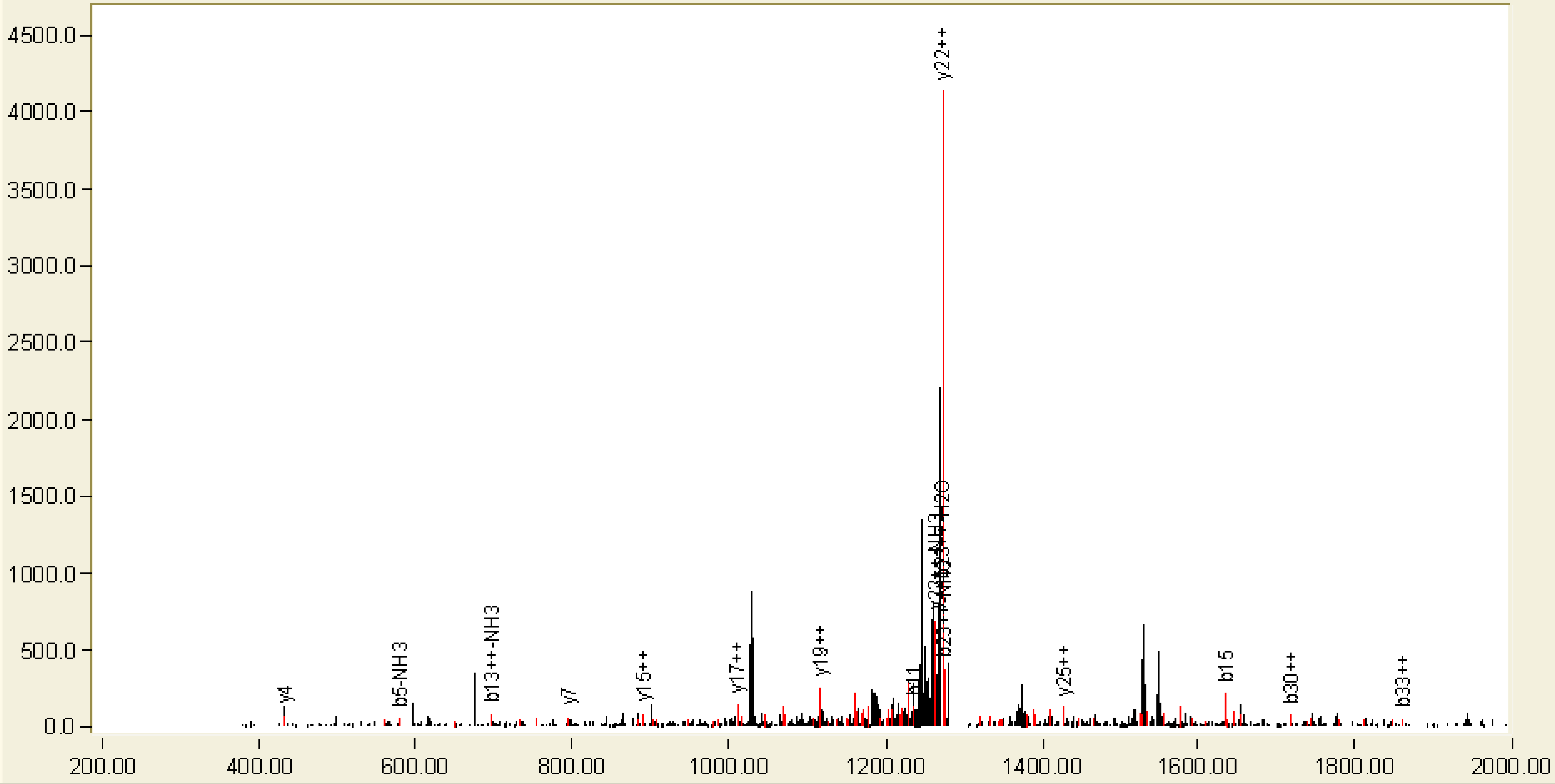

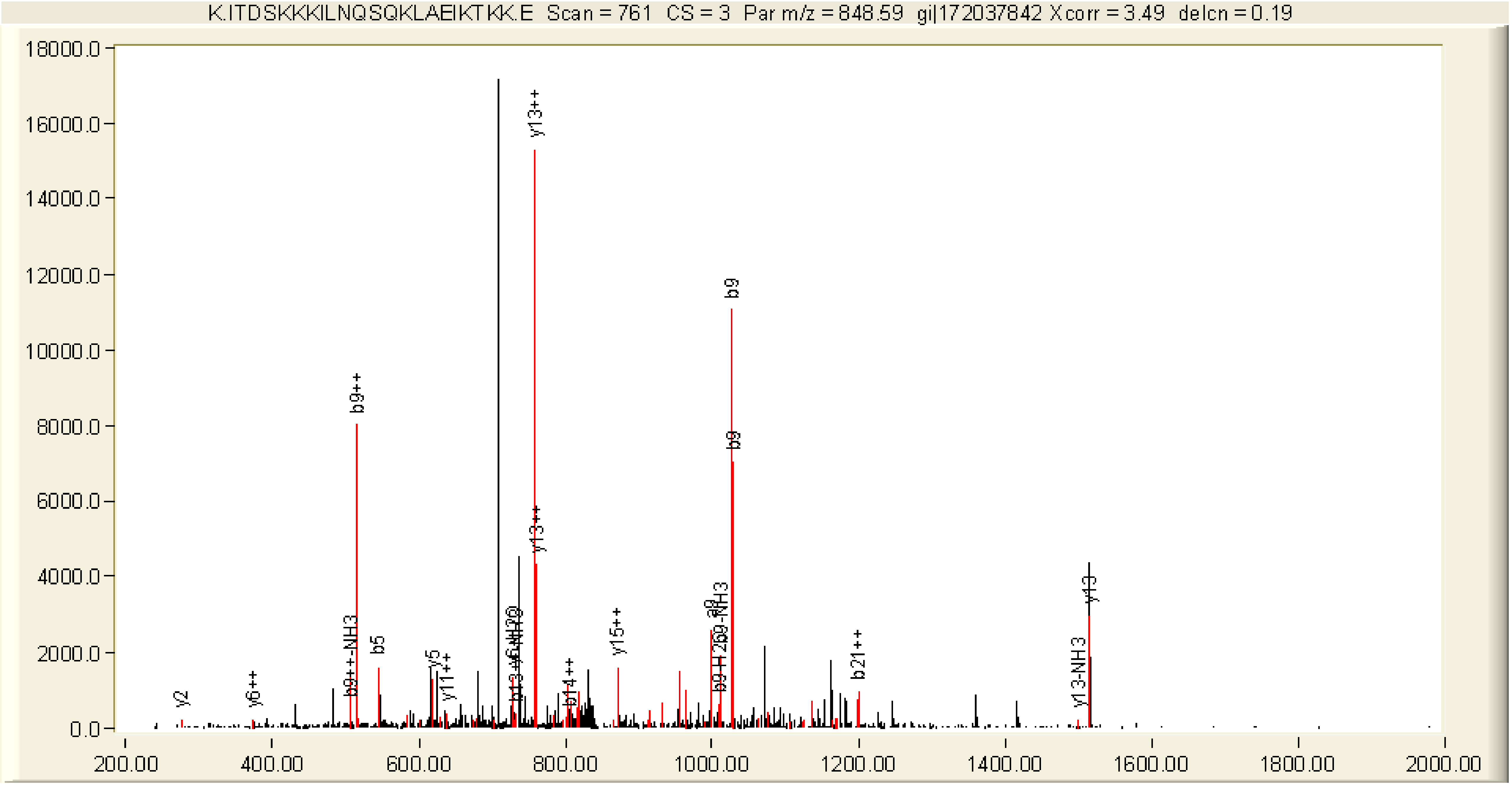

K.ITDSKKKILNQSQKLAEIKTKK.E Scan = 761 CS = 3 Par m/z = 848.59 gi|172037842 Xcorr = 3.49 delcn = 0.19
18000.0
16000.0
14000.0
12000.0
10000.0
8000.0
6000.0
4000.0
2000.0
0.0
200.00
400.00
600.00
800.00
1000.00
1200.00
1400.00
1600.00
1800.00
2000.00
y2
y6++
b9++-NH3
b9++
b5
y5
y11++
y13++
b9
b14++
y15++
b9-NH3
b21++
y13-NH3
y13

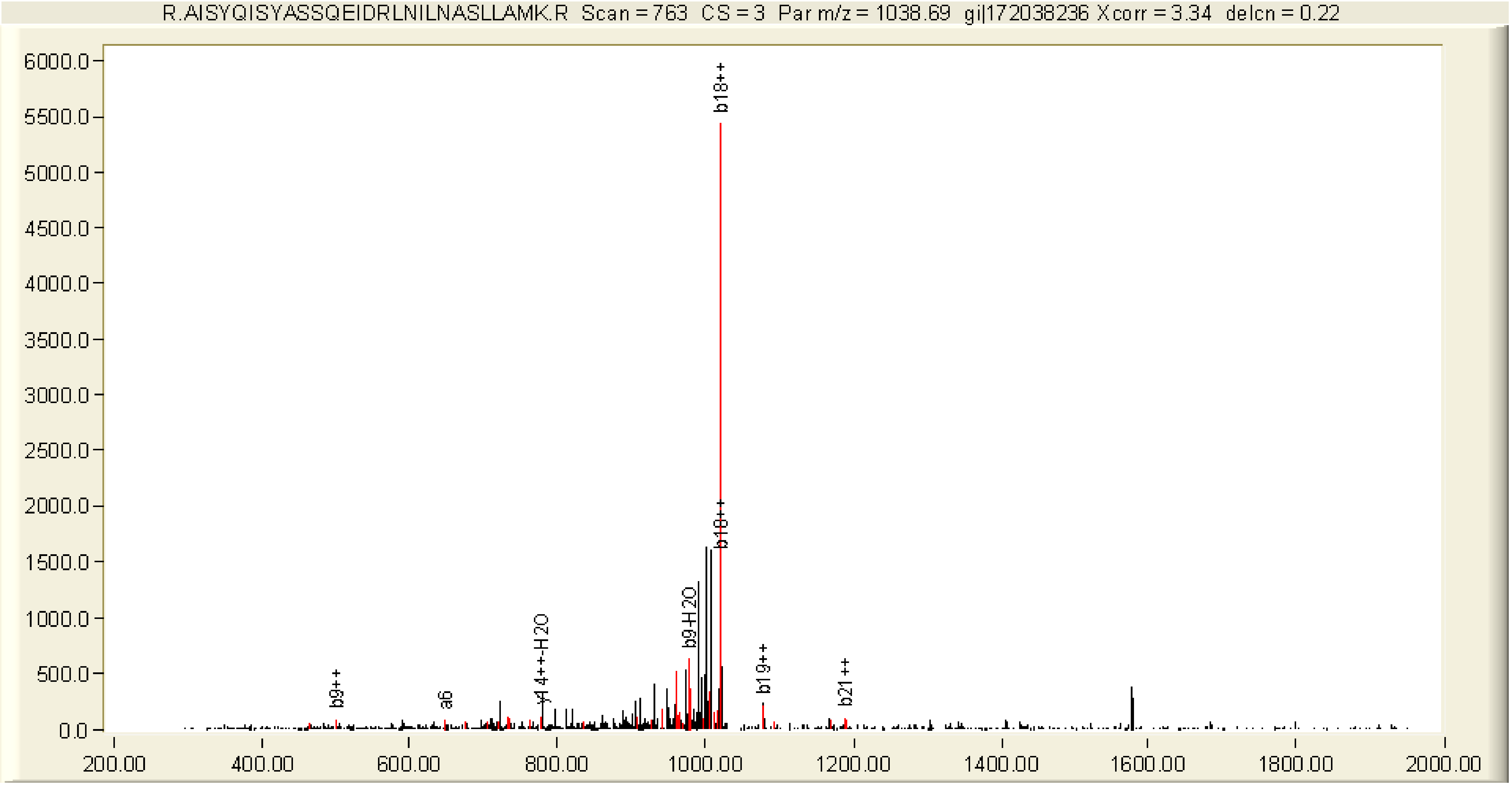
R.AISYQISYASSQEIDRLNILNASLLAMK.R Scan = 763 CS = 3 Par m/z = 1038.69 gi|172038236 Xcorr = 3.34 delcn = 0.22
b18++
b18++
b9-H2O
y14++-H2O
b9++
a6
b19++
b21++
6000.0
5500.0
5000.0
4500.0
4000.0
3500.0
3000.0
2500.0
2000.0
1500.0
1000.0
500.0
0.0
200.00
400.00
600.00
800.00
1000.00
1200.00
1400.00
1600.00
1800.00
2000.00

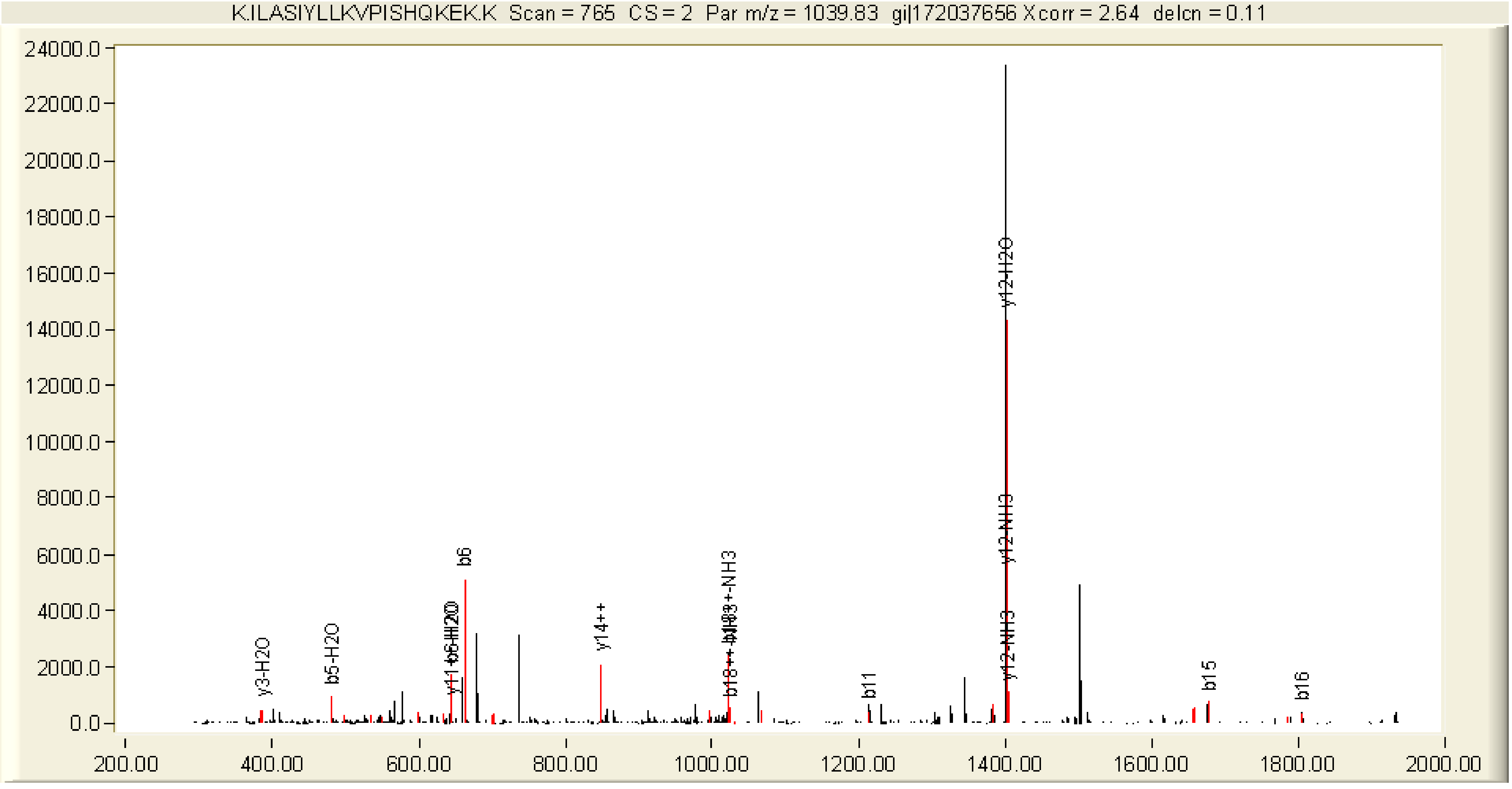
K.ILASIYLLKVPISHQKEK.K Scan = 765 CS = 2 Par m/z = 1039.83 gi|172037656 Xcorr = 2.64 delcn = 0.11
24000.0
22000.0
20000.0
18000.0
16000.0
14000.0
12000.0
10000.0
8000.0
6000.0
4000.0
2000.0
0.0
200.00
400.00
600.00
800.00
1000.00
1200.00
1400.00
1600.00
1800.00
2000.00
y3-H2O
b5-H2O
b6
y14++
b18++-NH3
y12-H2O
y12-NH3
b11
b15
b16

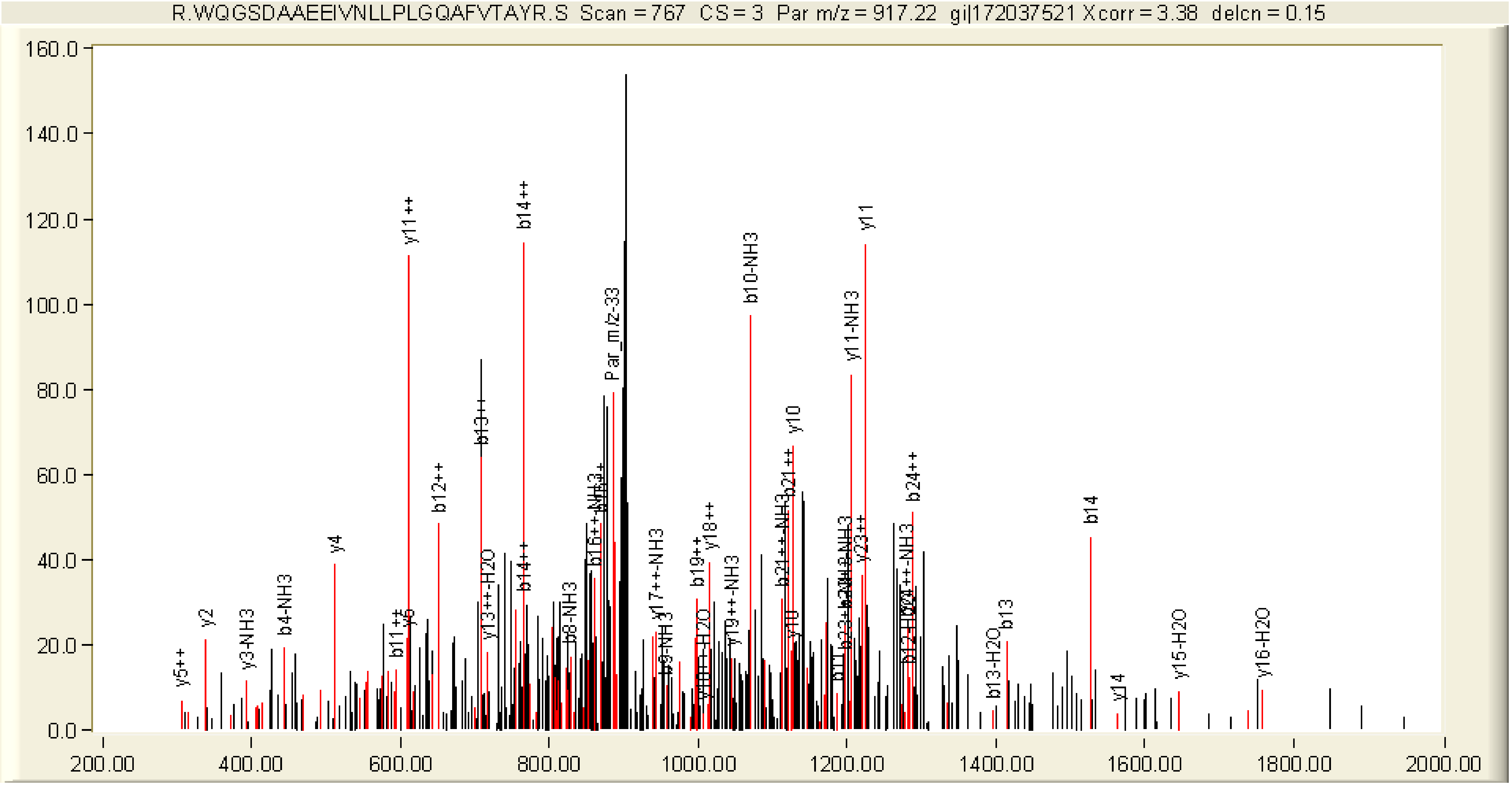
R.WQGSDAAEEIVNLLPLGQAFVTAYR.S Scan = 767 CS = 3 Par m/z = 917.22 gi|172037521 Xcorr = 3.38 delcn = 0.15
160.0
140.0
120.0
100.0
80.0
60.0
40.0
20.0
0.0
200.00
400.00
600.00
800.00
1000.00
1200.00
1400.00
1600.00
1800.00
2000.00
y5++
y2
y3-NH3
b4-NH3
y4
b11++
y11++
b12++
b13++
y13++-H2O
b14++
b14++
b8-NH3
Par_m/z-33
y17++-NH3
b19++
y18++
y19++-NH3
b10-NH3
y10
y11-NH3
y23++
y11
b24++
b13-H2O
b13
b14
y14
y15-H2O
y16-H2O

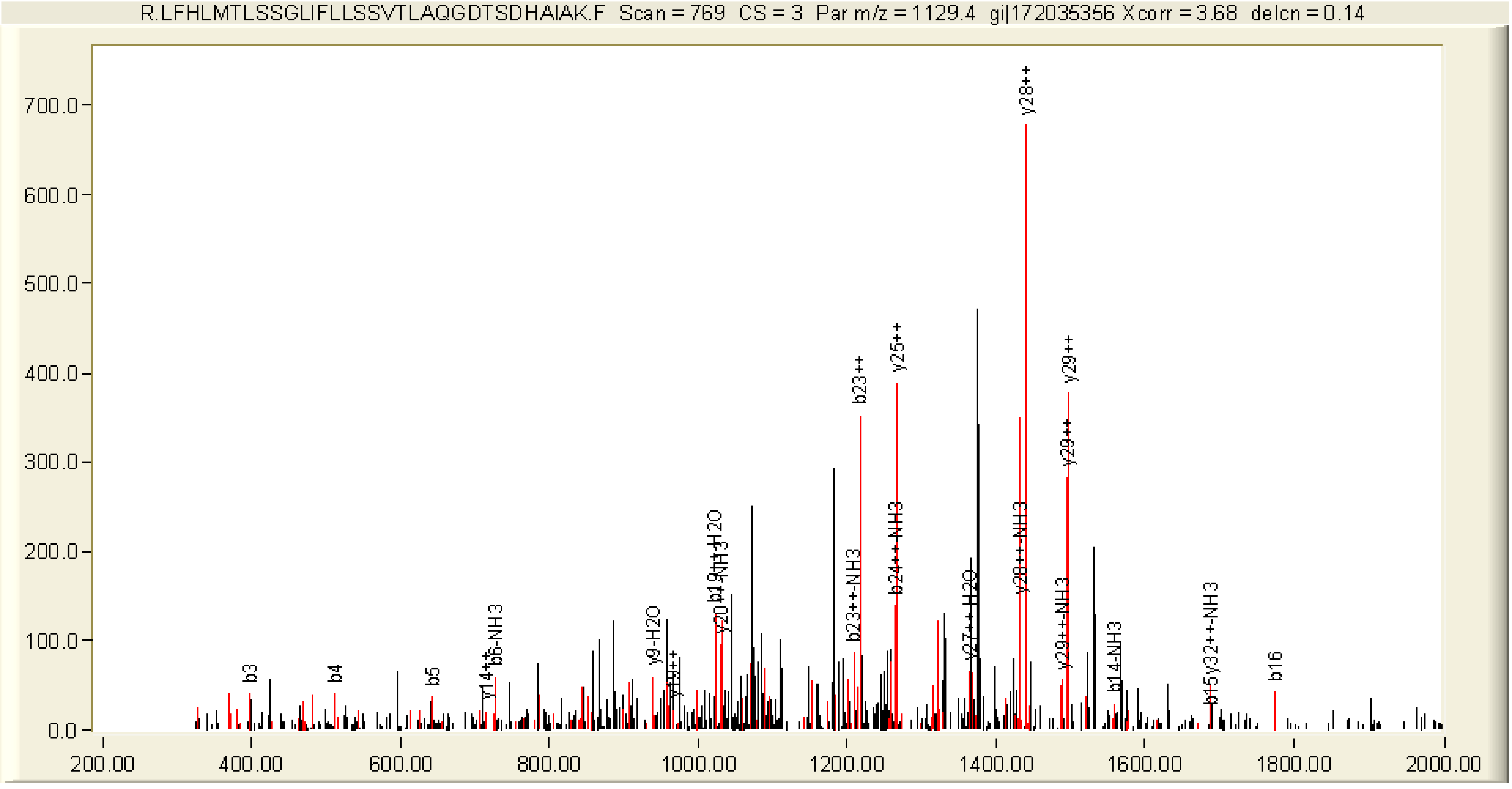
R.LFHLMTLSSGLIFLLSSVTLAQGDTSDHAIAK.F Scan = 769 CS = 3 Par m/z = 1129.4 gi|172035356 Xcorr = 3.68 delcn = 0.14
700.0
600.0
500.0
400.0
300.0
200.0
100.0
0.0
200.00
400.00
600.00
800.00
1000.00
1200.00
1400.00
1600.00
1800.00
2000.00
b3
b4
b5
b6-NH3
y9-H2O
b23++
b23++-NH3
y25++
b24++-NH3
y28++
y28++-NH3
y29++
y29++-NH3
b14-NH3
y32++-NH3
b16

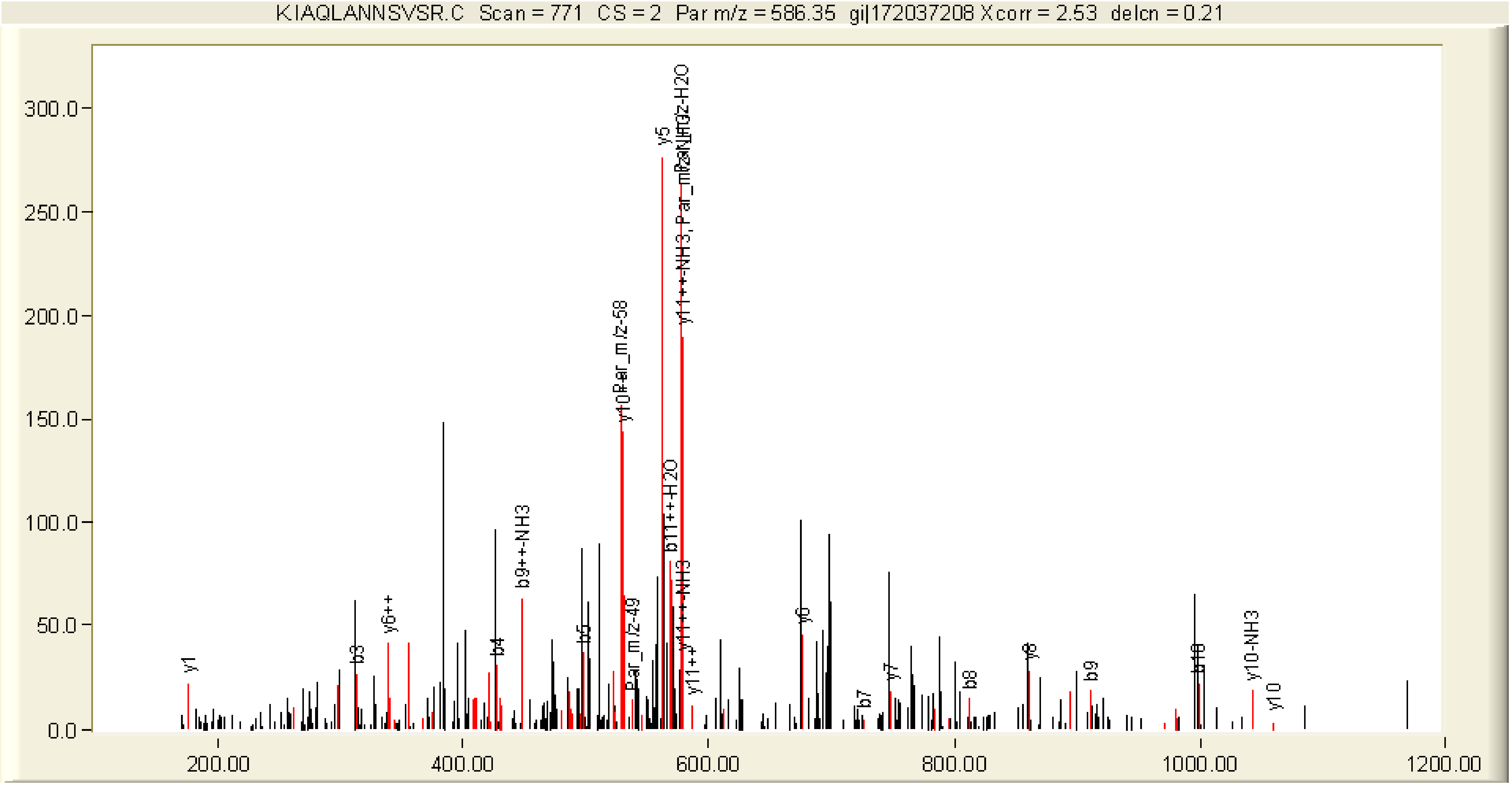

KIAQLANNSVSR.C Scan = 771 CS = 2 Par m/z = 586.35 gi|172037208 Xcorr = 2.53 delcn = 0.21
300.0
250.0
200.0
150.0
100.0
50.0
0.0
200.00
400.00
600.00
800.00
1000.00
1200.00
y1
b3
y6++
b4
b9++-NH3
b5
Par_m/z-49
Par_m/z-58
y5
b11++-H2O
y11++-NH3
y6
b7
y7
b8
b9
y10-NH3
y10

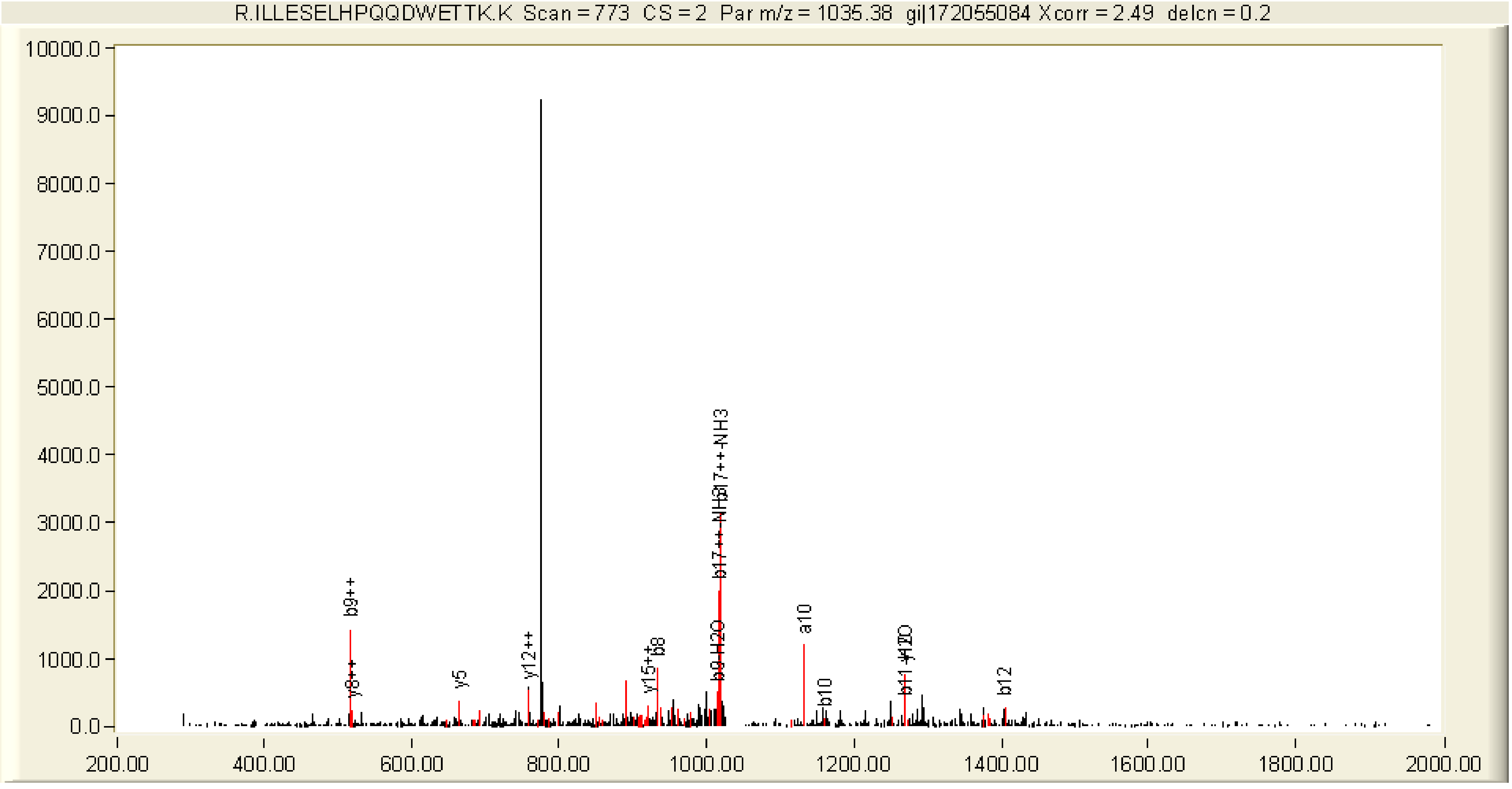
R.ILLESELHPQQDWETTK.K Scan = 773 CS = 2 Par m/z = 1035.38 gi|172055084 Xcorr = 2.49 delcn = 0.2
10000.0
9000.0
8000.0
7000.0
6000.0
5000.0
4000.0
3000.0
2000.0
1000.0
0.0
200.00
400.00
600.00
800.00
1000.00
1200.00
1400.00
1600.00
1800.00
2000.00
b9++
y8++
y5
y12++
y15++
b8
b17++-NH3
a10
b10
b12

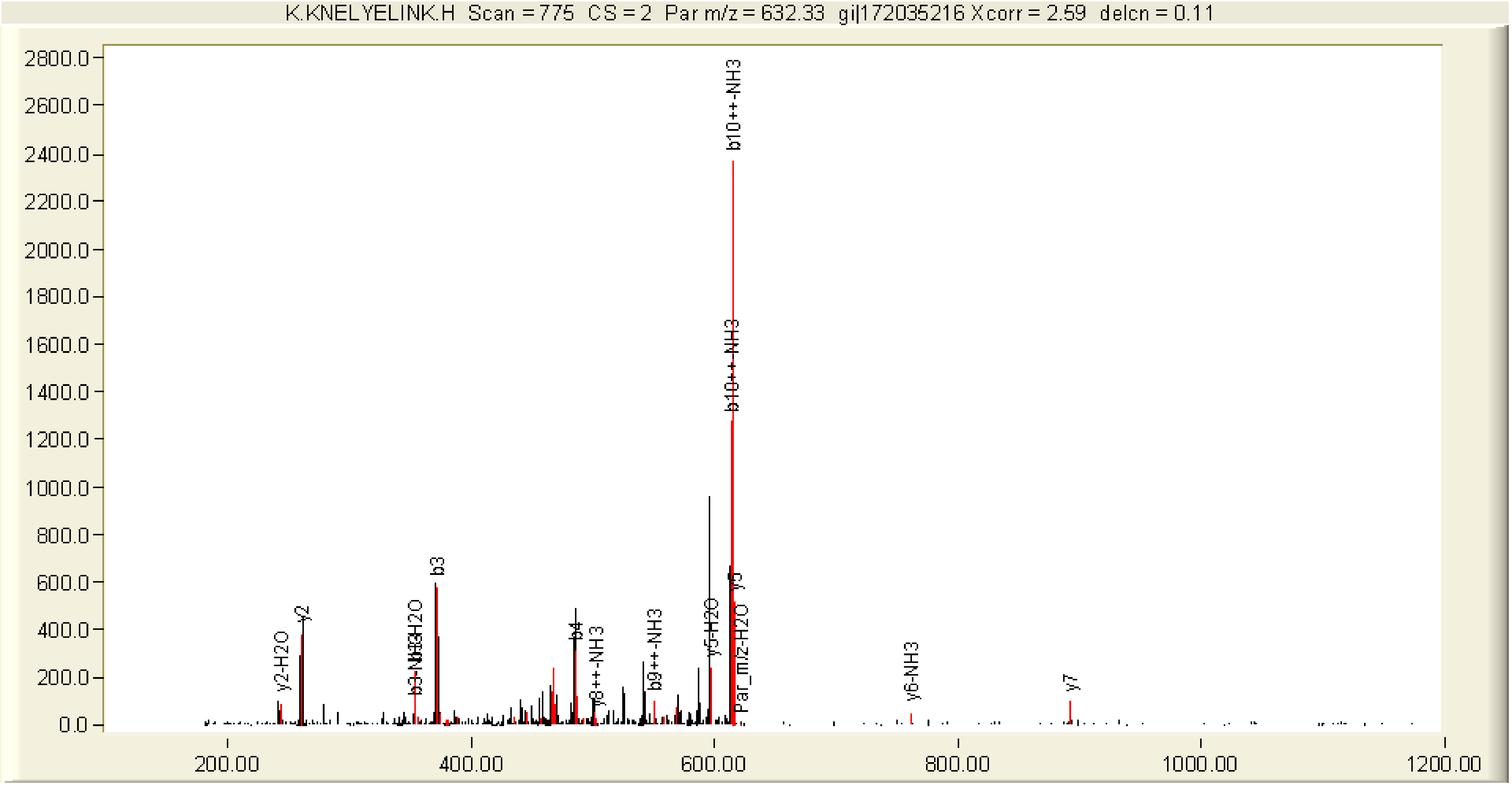
K.KNELYELINK.H Scan = 775 CS = 2 Par m/z = 632.33 gi|172035216 Xcorr = 2.59 delcn = 0.11
2800.0
2600.0
2400.0
2200.0
2000.0
1800.0
1600.0
1400.0
1200.0
1000.0
800.0
600.0
400.0
200.0
0.0
200.00
400.00
600.00
800.00
1000.00
1200.00
y2-H2O
y2
b3
b4
y8++-NH3
b9++-NH3
y5-H2O
b10++-NH3
b10++-NH3
y5
Par_m/z-H2O
y6-NH3
y7

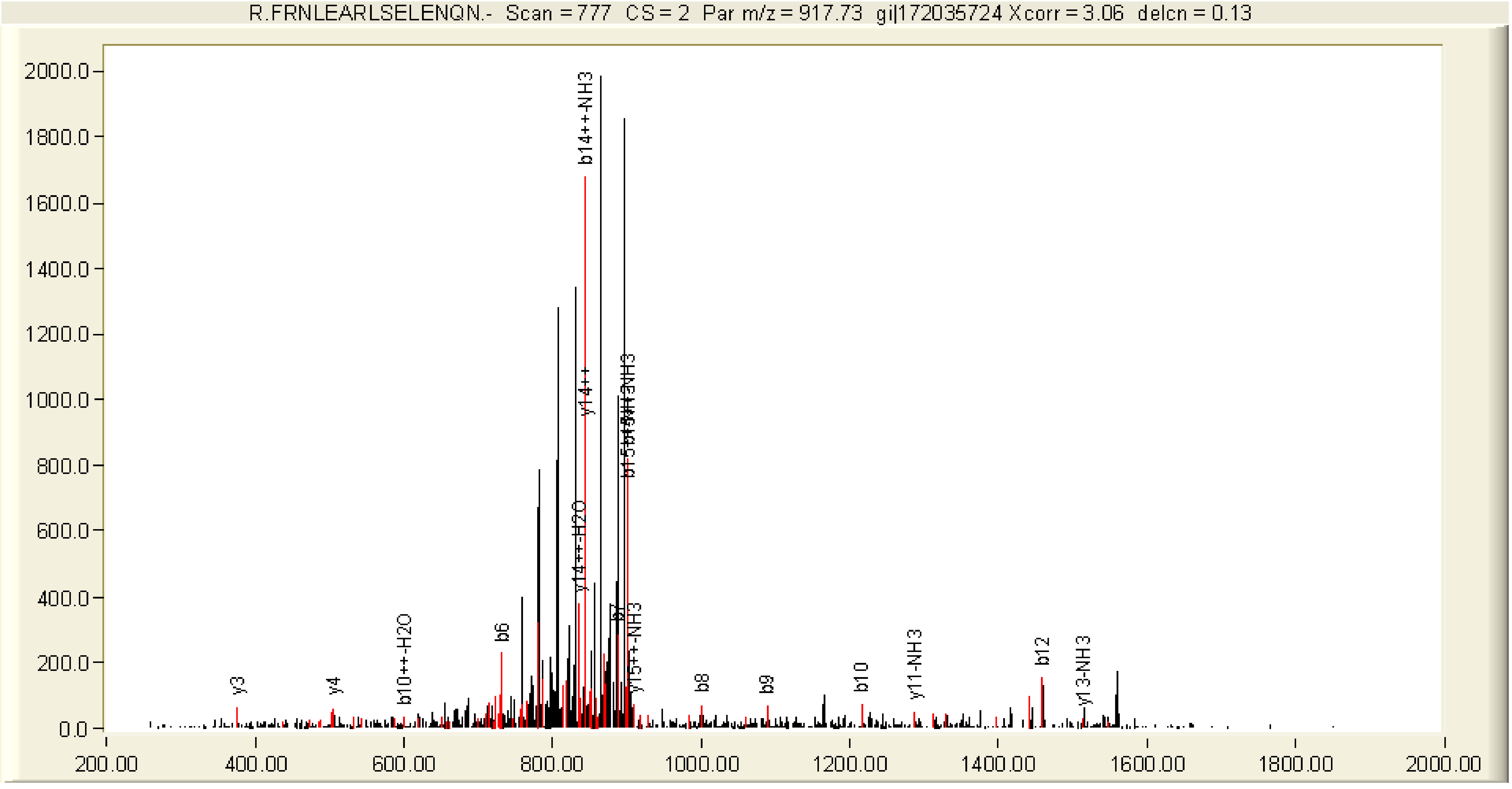

R.FRNLEARLSELENQN.- Scan = 777 CS = 2 Par m/z = 917.73 gi|172035724 Xcorr = 3.06 delcn = 0.13
2000.0
1800.0
1600.0
1400.0
1200.0
1000.0
800.0
600.0
400.0
200.0
0.0
200.00
400.00
600.00
800.00
1000.00
1200.00
1400.00
1600.00
1800.00
2000.00
y3
y4
b10++-H2O
b6
y14++-H2O
b14++-NH3
y14++
b7
y15++-NH3
b8
b9
b10
y11-NH3
b12
y13-NH3

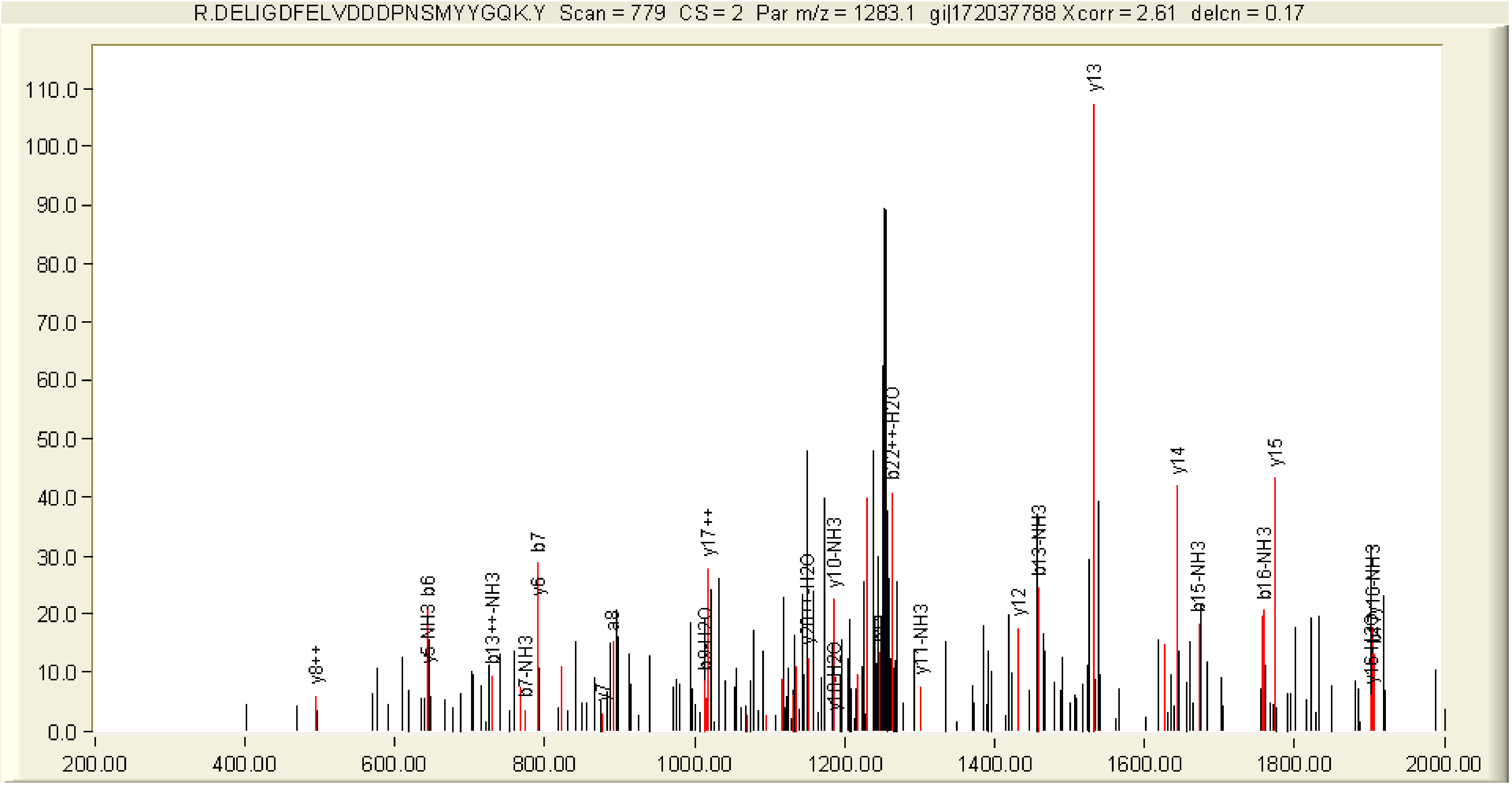
R.DELIGDFELVDDDPNSMYYGQK.Y Scan = 779 CS = 2 Par m/z = 1283.1 gi|172037788 Xcorr = 2.61 delcn = 0.17
110.0
100.0
90.0
80.0
70.0
60.0
50.0
40.0
30.0
20.0
10.0
0.0
200.00
400.00
600.00
800.00
1000.00
1200.00
1400.00
1600.00
1800.00
2000.00
y13
b22++-H2O
y14
y15
y17++
y10-NH3
b13-NH3
b15-NH3
b16-NH3
b7
y6
y5-NH3 b6
b13++-NH3
b7-NH3
a8
y8++
y12
y11-NH3

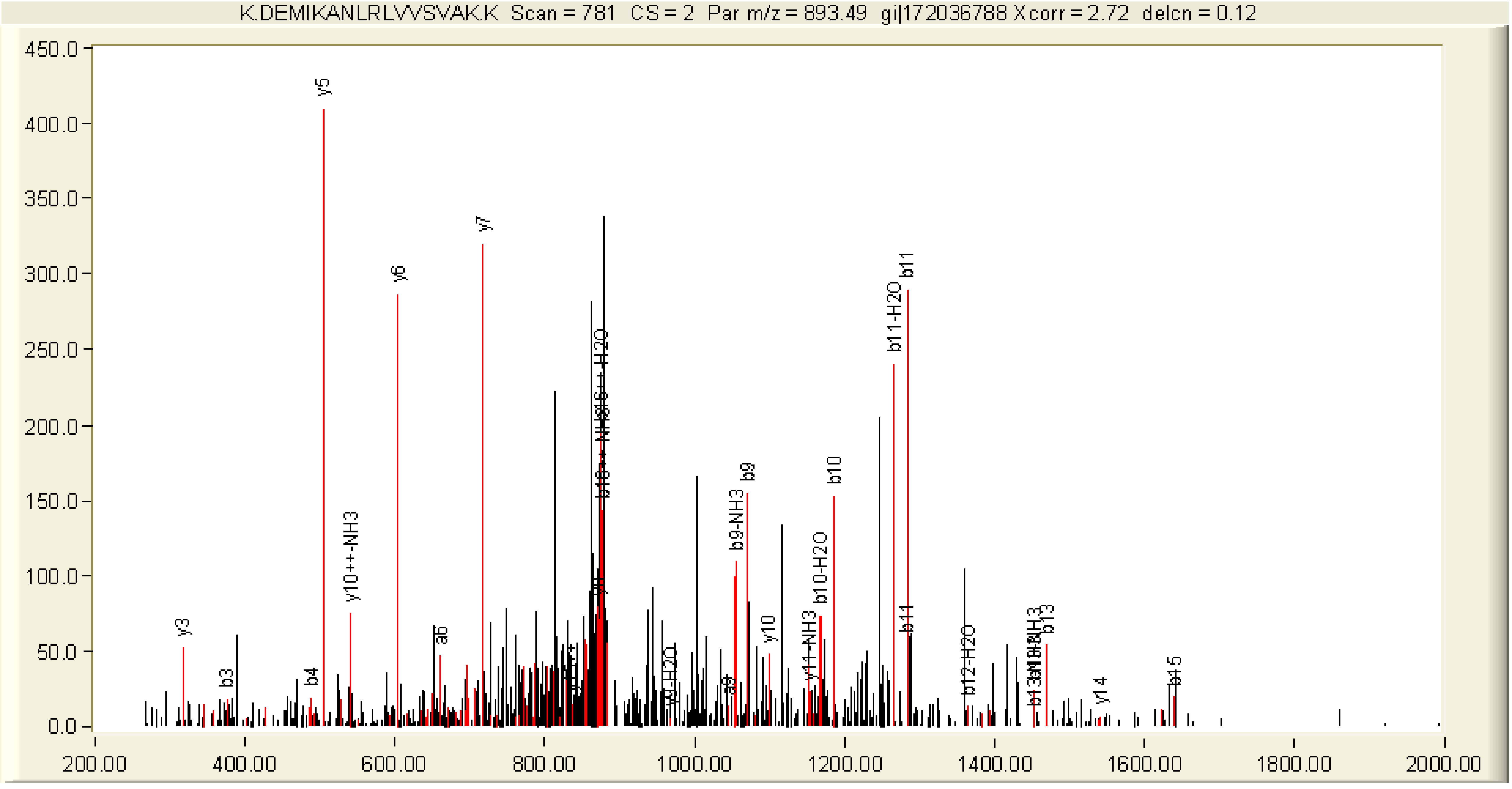

K.DEMIKANLRLVVSVAK.K Scan = 781 CS = 2 Par m/z = 893.49 gi|172036788 Xcorr = 2.72 delcn = 0.12
450.0
400.0
350.0
300.0
250.0
200.0
150.0
100.0
50.0
0.0
200.00
400.00
600.00
800.00
1000.00
1200.00
1400.00
1600.00
1800.00
2000.00
y3
b3
b4
y5
y10++-NH3
y6
a6
y7
b9-NH3
b9
y10
b10-H2O
b10
y11-NH3
b11-H2O
b11
b12-H2O
b13
y14
b15

K.DTIEAVGIPHPEVELILNNGNIIDFSYGIK.K Scan = 783 CS = 3 Par m/z = 1094.34 gi|172039179 Xcorr = 3.27 delcn = 0.11

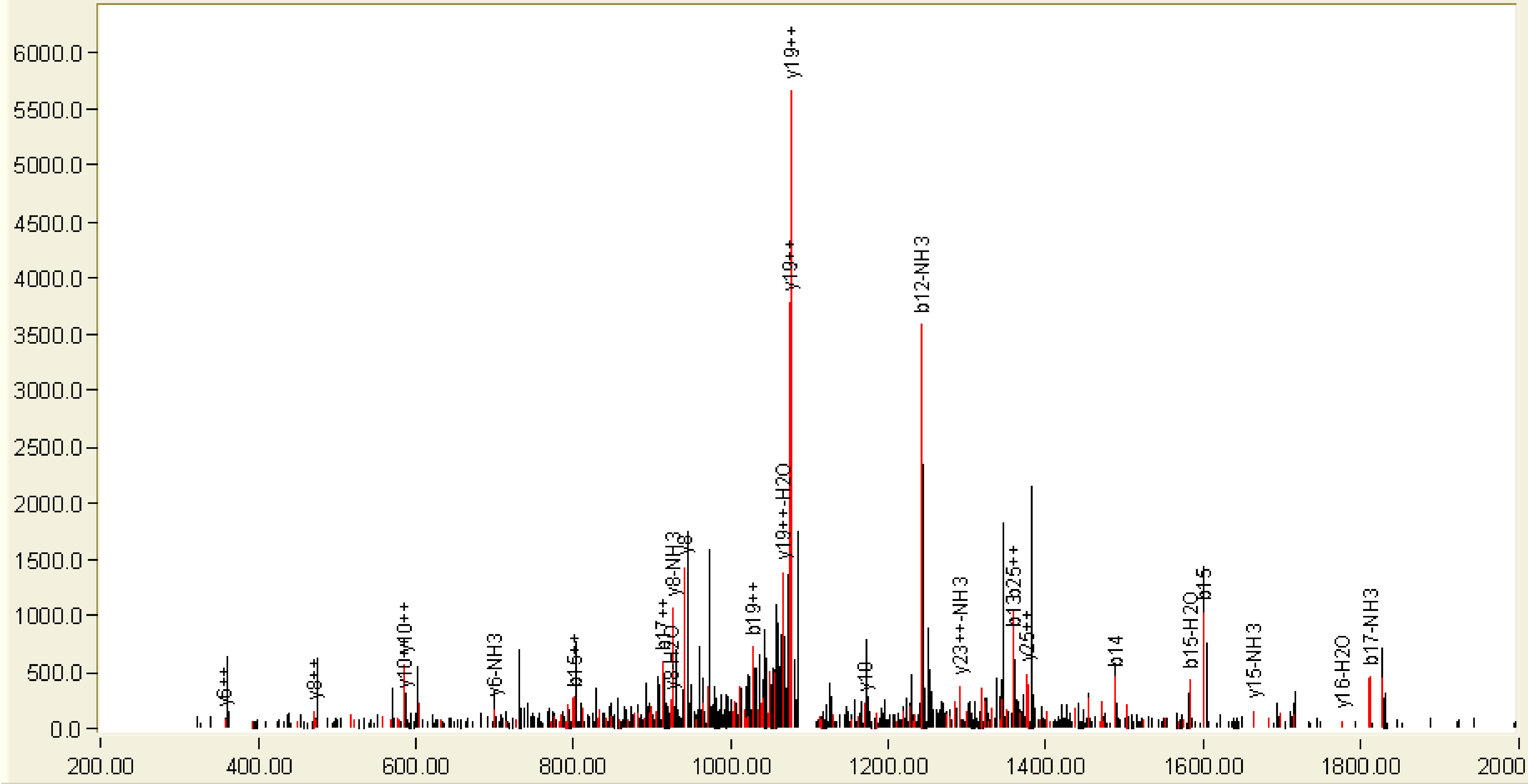

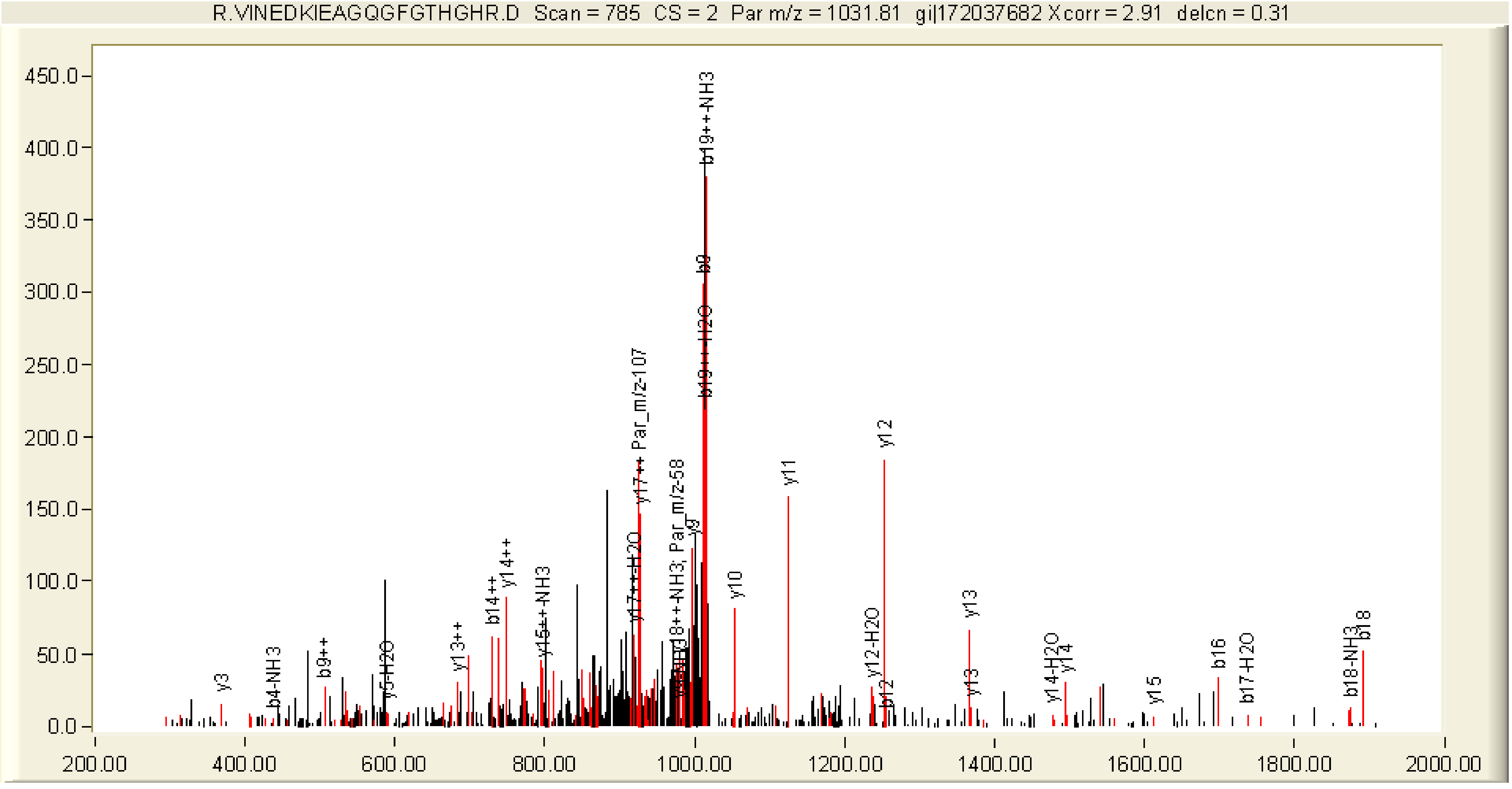

R.VINEDKIEAGQGFGTHGHR.D Scan = 785 CS = 2 Par m/z = 1031.81 gi|172037682 Xcorr = 2.91 delcn = 0.31
450.0
400.0
350.0
300.0
250.0
200.0
150.0
100.0
50.0
0.0
200.00
400.00
600.00
800.00
1000.00
1200.00
1400.00
1600.00
1800.00
2000.00
y3
b4-NH3
b9++
y5-H2O
y13++
b14++
y14++
y15++-NH3
y17++-H2O
y17++ Par_m/z-107
Par_m/z-58
y9
b9
b19++-H2O
b19++-NH3
y10
y11
y12-H2O
b12
y12
y13
y13
y14-H2O
y14
y15
b16
b17-H2O
b18-NH3
b18

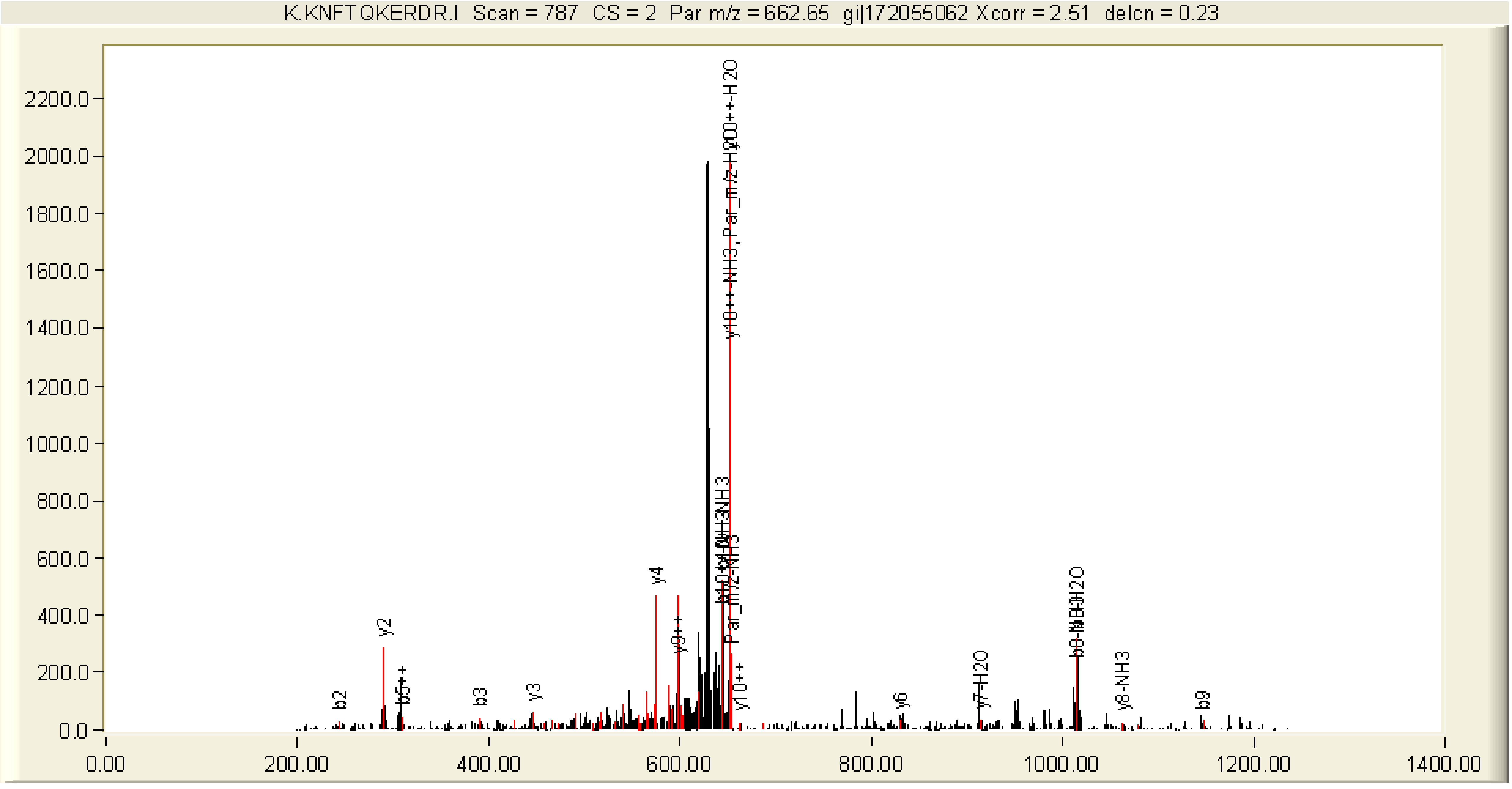
K.KNFTQKERDR.I Scan = 787 CS = 2 Par m/z = 662.65 gi|172055062 Xcorr = 2.51 delcn = 0.23
2200.0
2000.0
1800.0
1600.0
1400.0
1200.0
1000.0
800.0
600.0
400.0
200.0
0.0
0.00
200.00
400.00
600.00
800.00
1000.00
1200.00
1400.00
b2
y2
b5++
b3
y3
y4
y9++
y10++
y6
y7-H2O
y8-NH3
b9

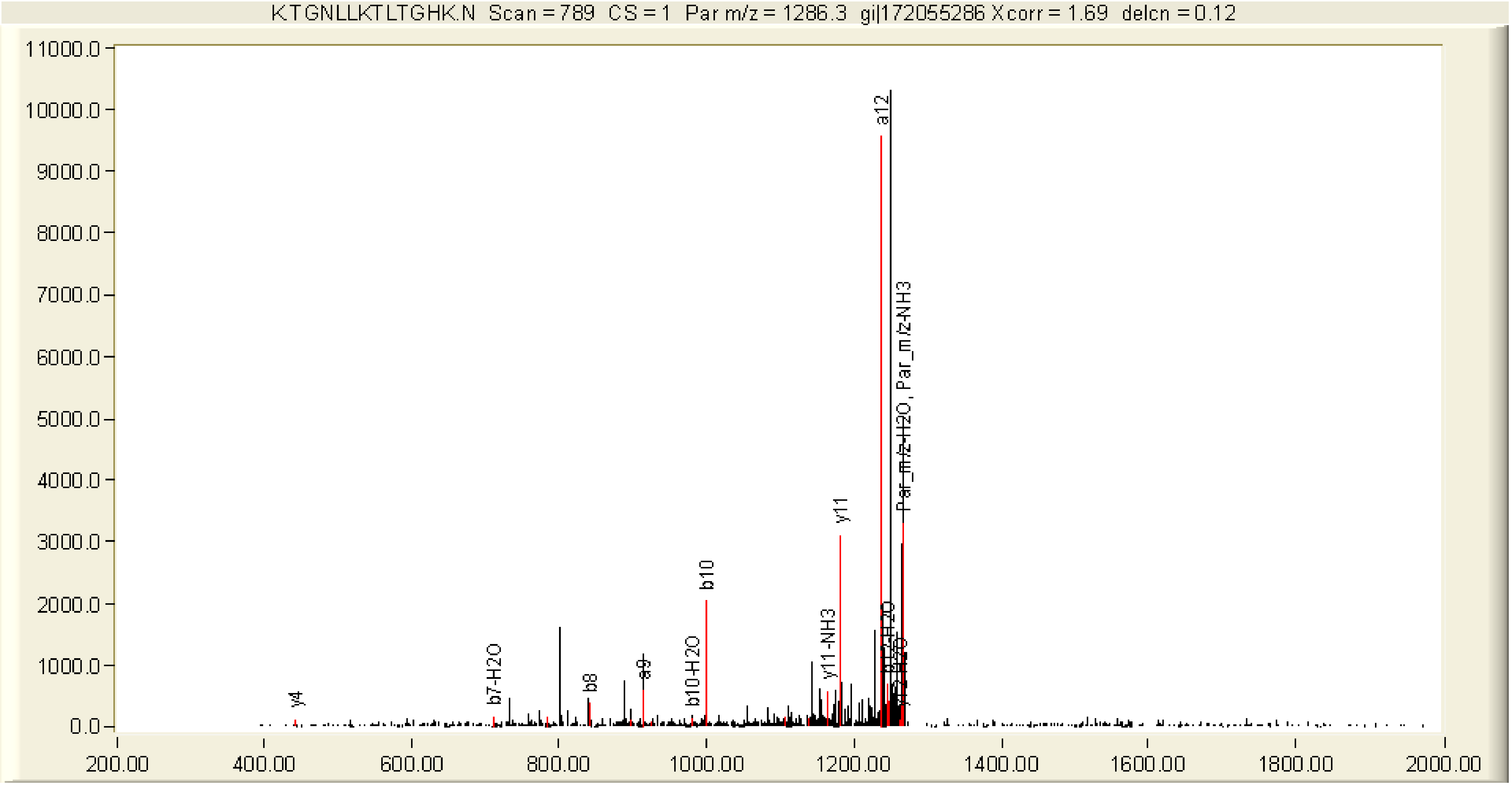
KTGNLLKTLTGHK.N Scan = 789 CS = 1 Par m/z = 1286.3 gi|172055286 Xcorr = 1.69 delcn = 0.12
11000.0
10000.0
9000.0
8000.0
7000.0
6000.0
5000.0
4000.0
3000.0
2000.0
1000.0
0.0
200.00
400.00
600.00
800.00
1000.00
1200.00
1400.00
1600.00
1800.00
2000.00
a12
Par_m/z-H2O, Par_m/z-NH3
y11
b10
y11-NH3
a9
b10-H2O
b8
b7-H2O
y4

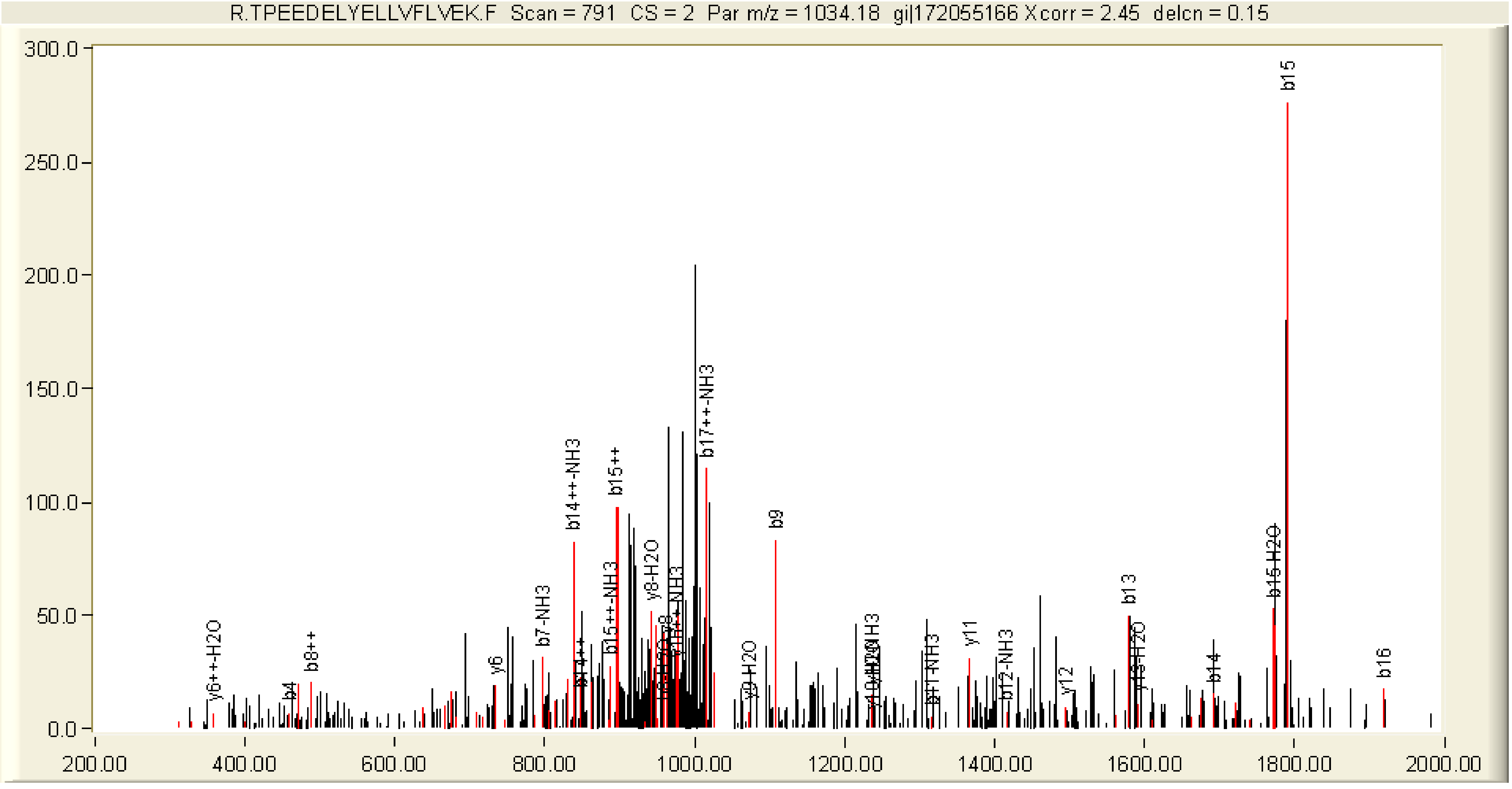
R.TPEEDELYELLVFLVEK.F Scan = 791 CS = 2 Par m/z = 1034.18 gi|172055166 Xcorr = 2.45 delcn = 0.15
300.0
250.0
200.0
150.0
100.0
50.0
0.0
200.00
400.00
600.00
800.00
1000.00
1200.00
1400.00
1600.00
1800.00
2000.00
b15
b17++-NH3
b14++-NH3
b15++
b9
y8-H2O
b15-H2O
b13
y6++-H2O
b8++
b4
y6
b7-NH3
b15++-NH3
y11
b12-NH3
y12
b14
b16

K.NYIIQAYQSAEFTYTQTDSNDLKDTEKITKK.S Scan = 793 CS = 3 Par m/z = 1221.19 gi|172055153 Xcorr = 3.35 delcn = 0.17

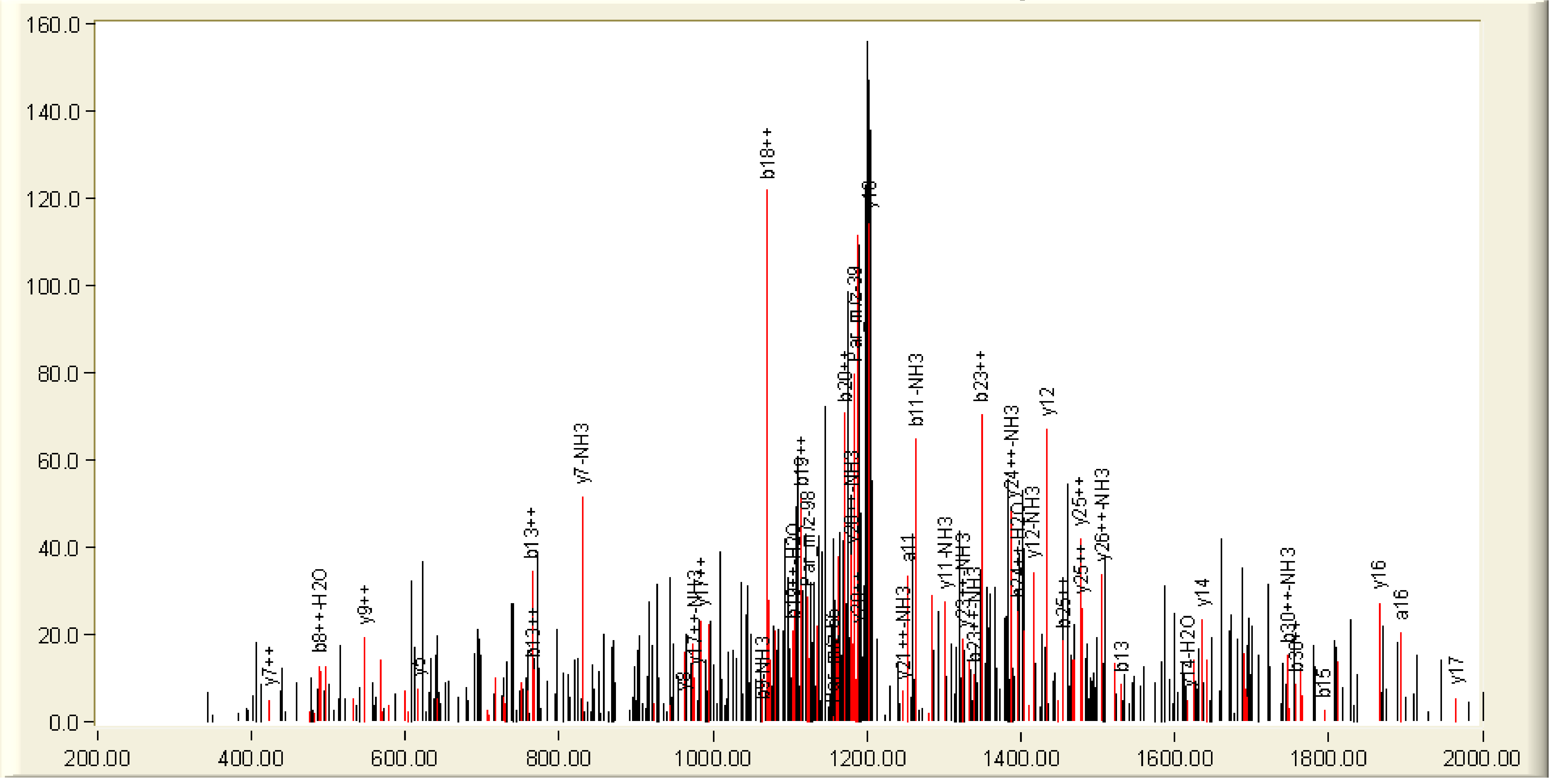

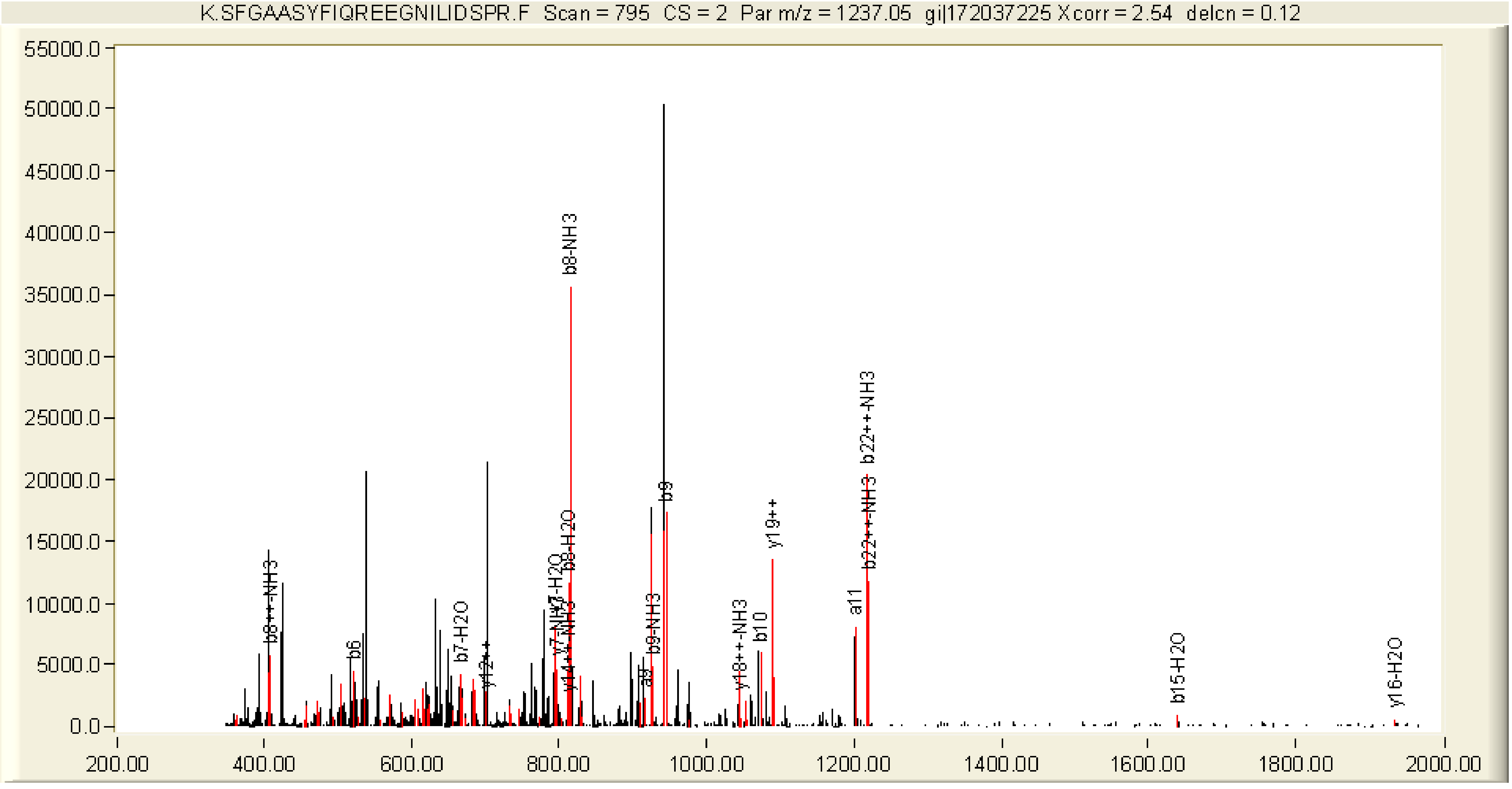
K.SFGAASYFIQREEGNILIDSPR.F Scan = 795 CS = 2 Par m/z = 1237.05 gi|172037225 Xcorr = 2.54 delcn = 0.12
55000.0
50000.0
45000.0
40000.0
35000.0
30000.0
25000.0
20000.0
15000.0
10000.0
5000.0
0.0
200.00
400.00
600.00
800.00
1000.00
1200.00
1400.00
1600.00
1800.00
2000.00
b8++-NH3
b6
b7-H2O
y12++
b8-H2O
b8-NH3
a9
b9-NH3
b9
y18++-NH3
b10
y19++
a11
b22++-NH3
b22++-NH3
b15-H2O
y16-H2O

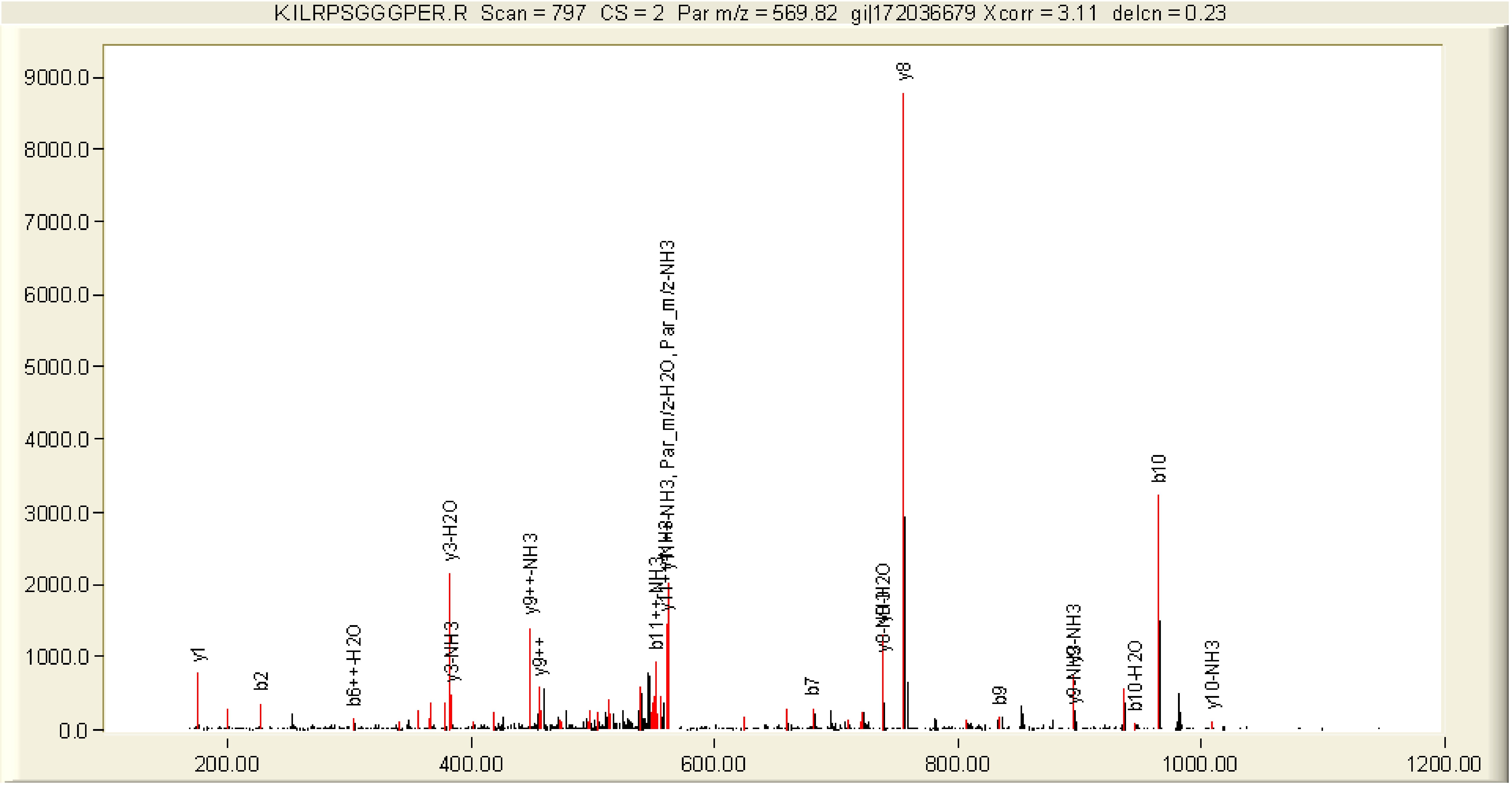
KILRPSGGGPER.R Scan = 797 CS = 2 Par m/z = 569.82 gi|172036679 Xcorr = 3.11 delcn = 0.23
9000.0
8000.0
7000.0
6000.0
5000.0
4000.0
3000.0
2000.0
1000.0
0.0
200.00
400.00
600.00
800.00
1000.00
1200.00
y1
b2
b6++-H2O
y3-NH3
y3-H2O
y9++
y9++-NH3
b11++-NH3
Par_m/z-H2O, Par_m/z-NH3
y8
b7
b9
b10-H2O
b10
y10-NH3

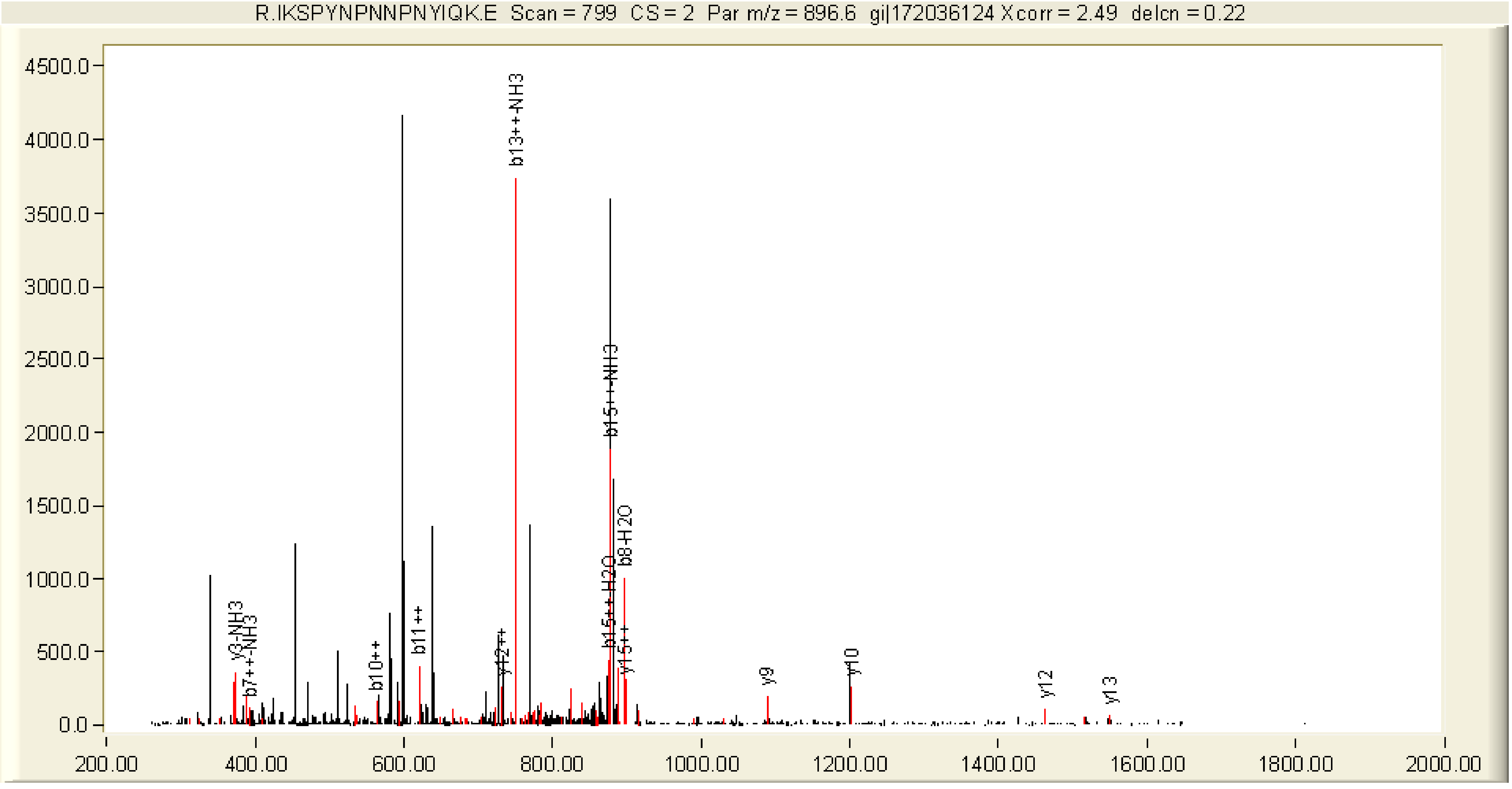
R.IKSPYNPNNPNYIQK.E Scan = 799 CS = 2 Par m/z = 896.6 gi|172036124 Xcorr = 2.49 delcn = 0.22
4500.0
4000.0
3500.0
3000.0
2500.0
2000.0
1500.0
1000.0
500.0
0.0
200.00
400.00
600.00
800.00
1000.00
1200.00
1400.00
1600.00
1800.00
2000.00
b13++-NH3
b15++-NH3
b8-H2O
b15++-H2O
y15++
y3-NH3
b7++-NH3
b10++
b11++
y12++
y9
y10
y12
y13

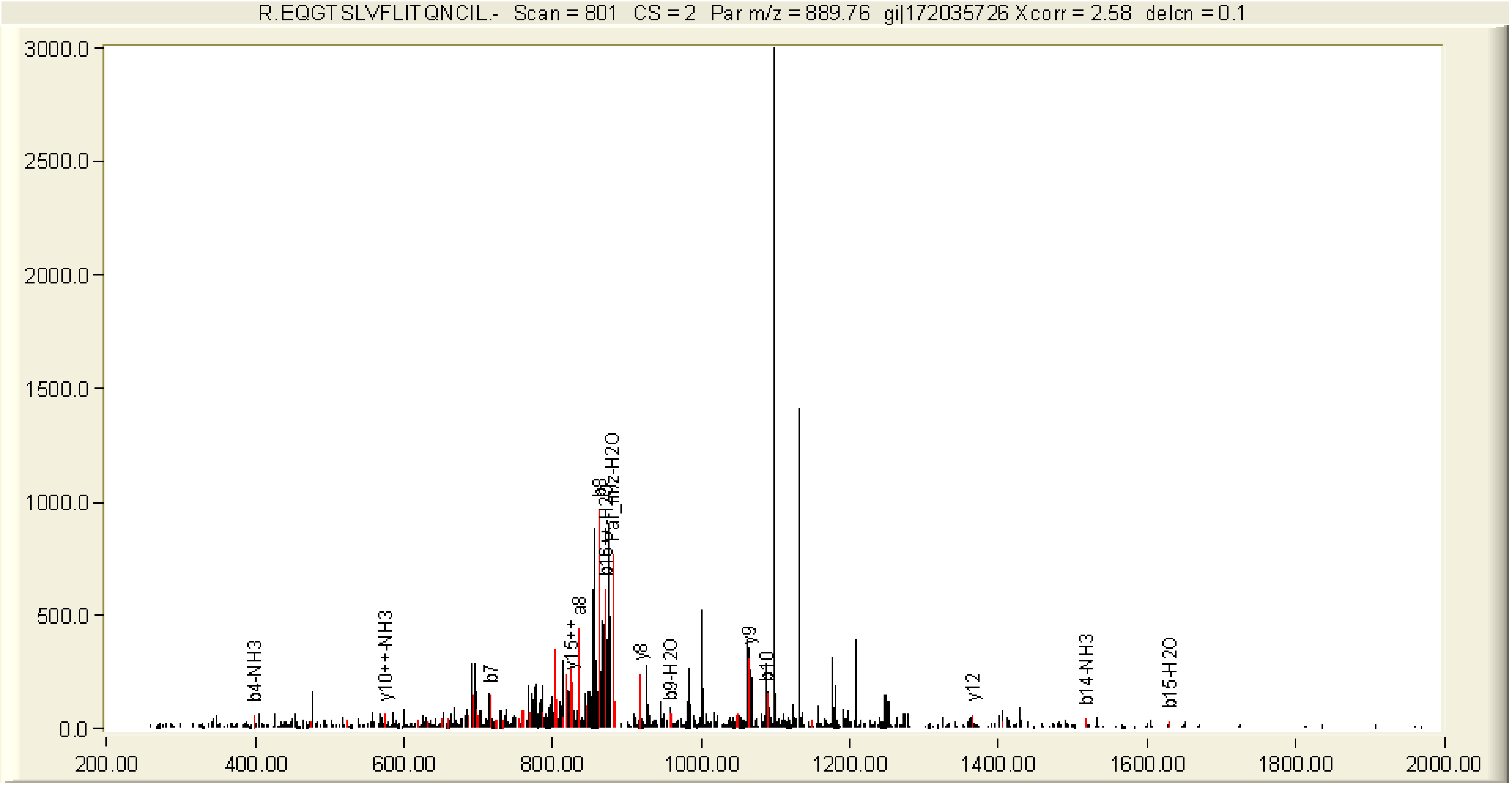
R.EQGTSLVFLITQNCIL.- Scan = 801 CS = 2 Par m/z = 889.76 gi|172035726 Xcorr = 2.58 delcn = 0.1
3000.0
2500.0
2000.0
1500.0
1000.0
500.0
0.0
200.00
400.00
600.00
800.00
1000.00
1200.00
1400.00
1600.00
1800.00
2000.00
b4-NH3
y10++-NH3
b7
y15++
a8
y8
b9-H2O
y9
b10
y12
b14-NH3
b15-H2O

K.TGHIYIDFGEETESEIEDN.- Scan = 803 CS = 2 Par m/z = 1100.95 gi|172035447 Xcorr = 2.42 delcn = 0.17

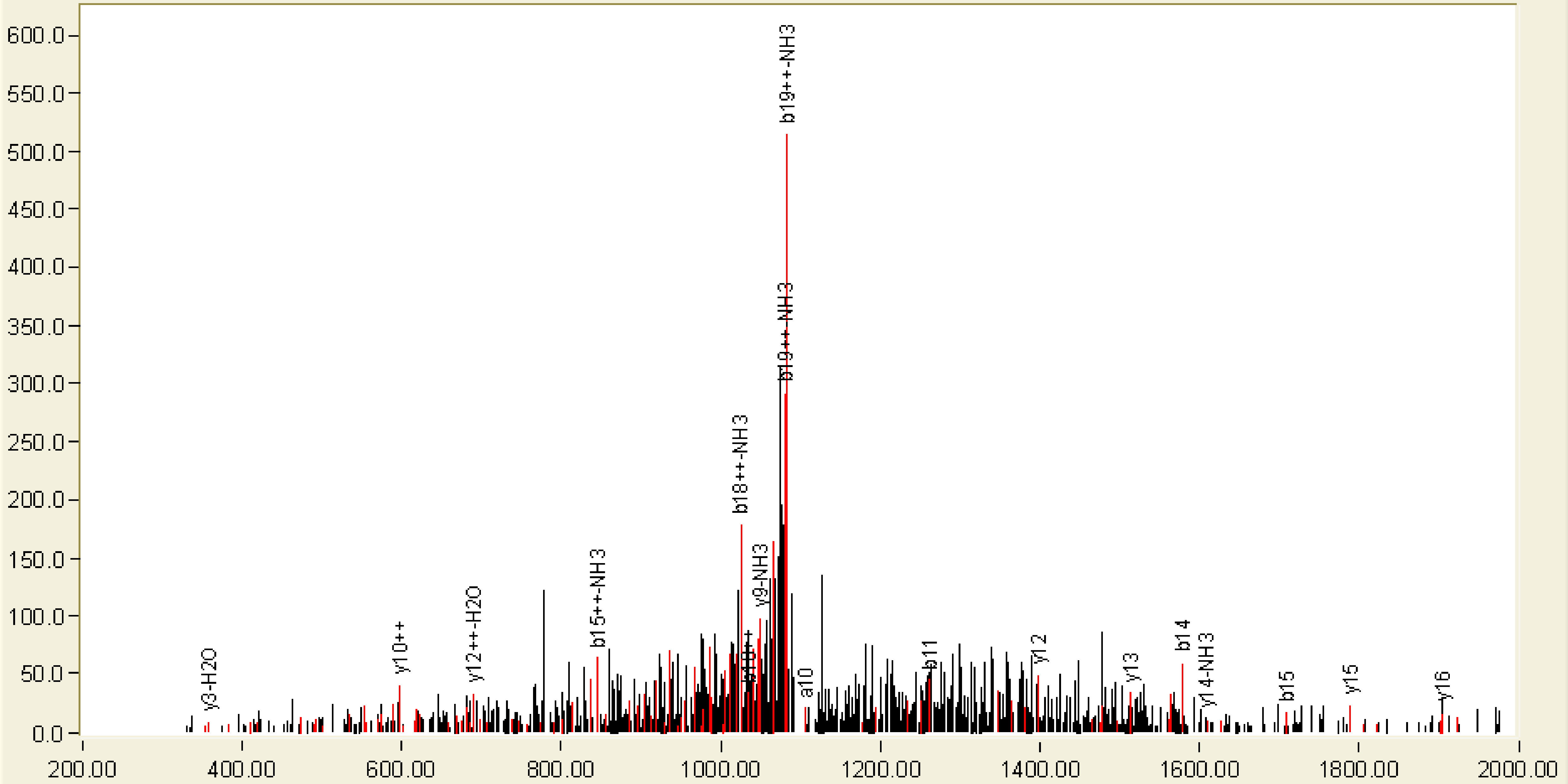

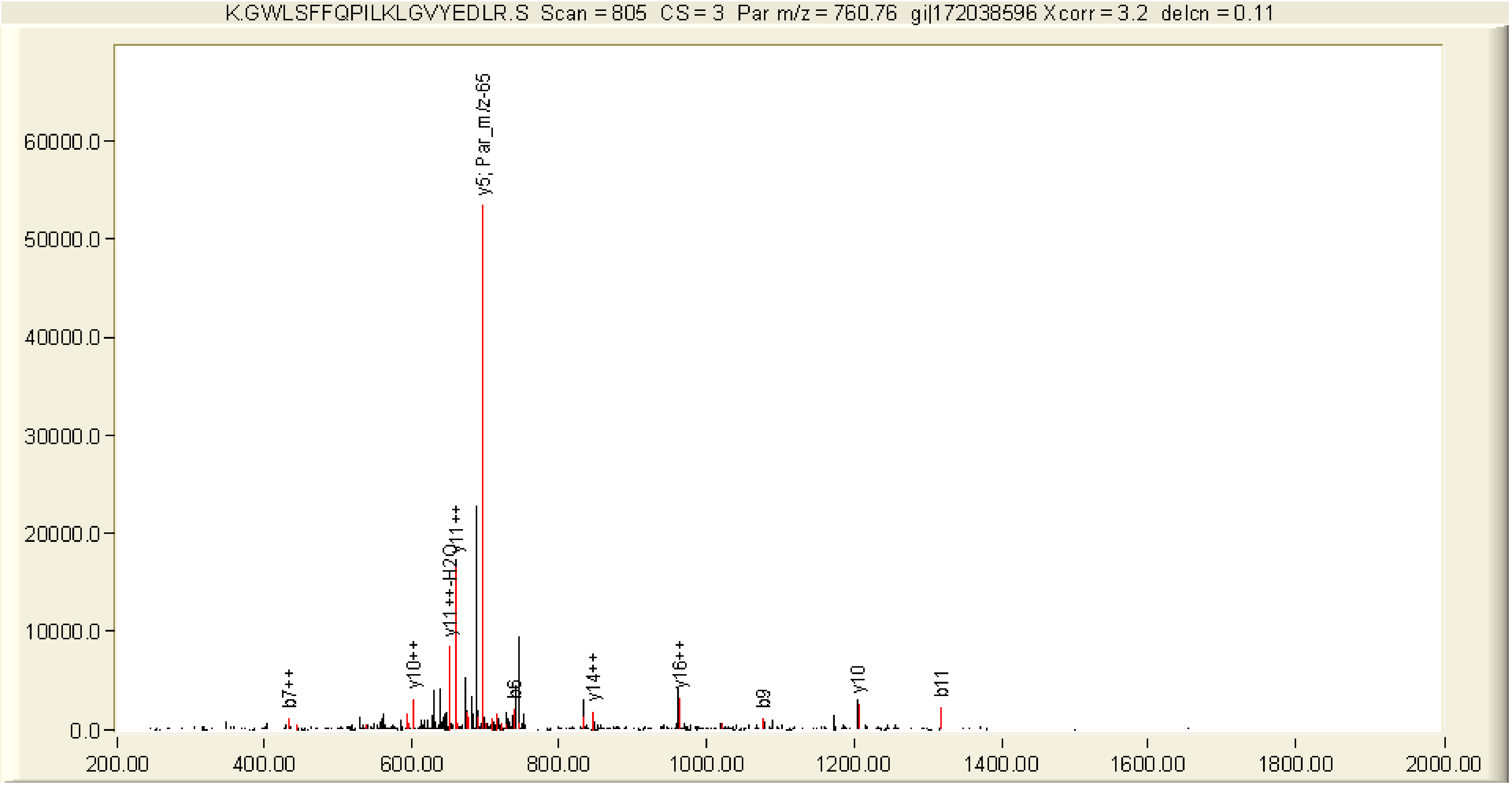

K.GWLSFFQPILKLGVYEDLR.S Scan = 805 CS = 3 Par m/z = 760.76 gi|172038596 Xcorr = 3.2 delcn = 0.11
y5; Par_m/z-65
y11++
y11++-H2O
y10++
b7++
b6
y14++
y16++
b9
y10
b11
60000.0
50000.0
40000.0
30000.0
20000.0
10000.0
0.0
200.00
400.00
600.00
800.00
1000.00
1200.00
1400.00
1600.00
1800.00
2000.00

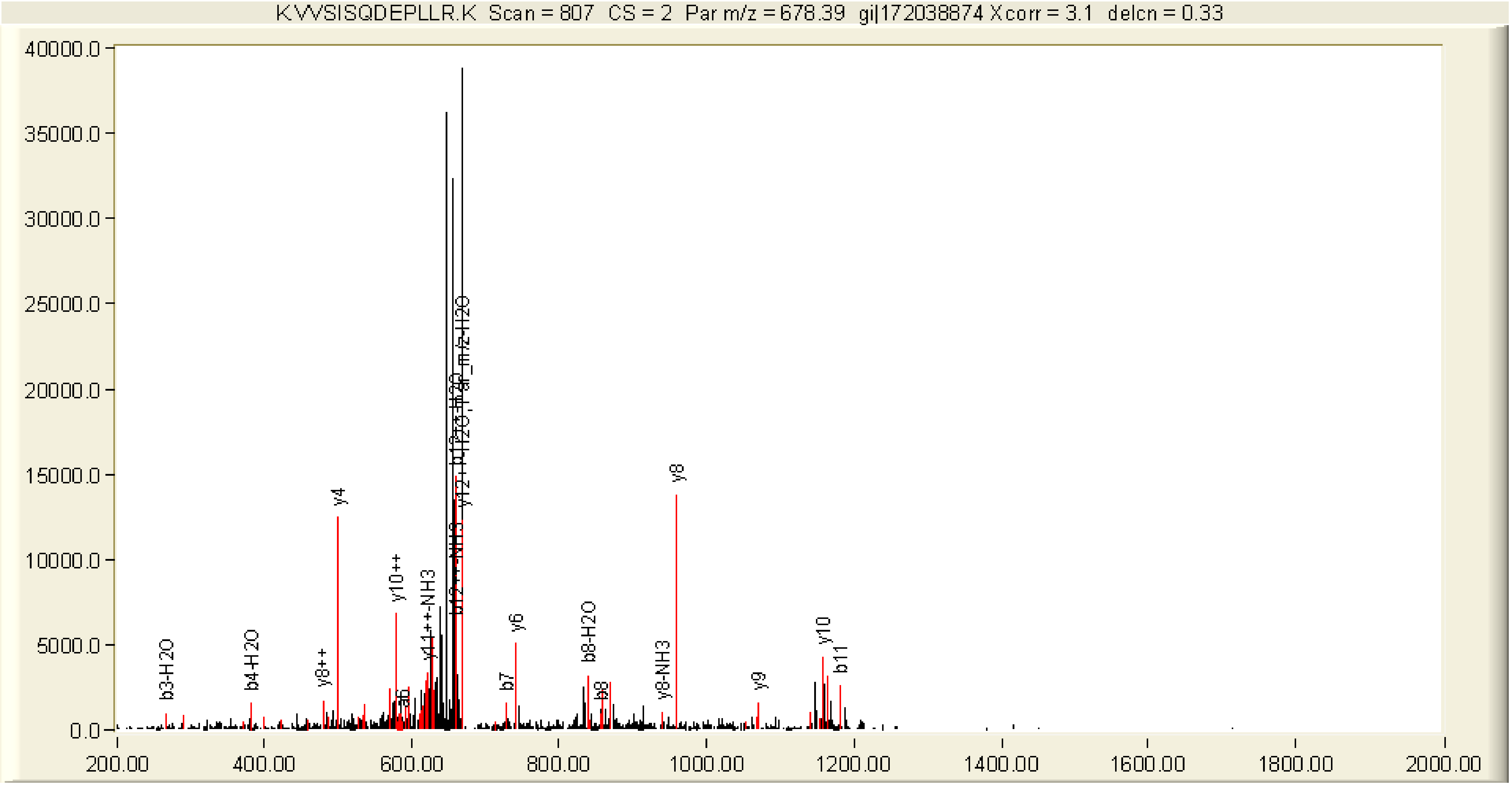
KVVSISQDEPLLR.K Scan = 807 CS = 2 Par m/z = 678.39 gi|172038874 Xcorr = 3.1 delcn = 0.33
40000.0
35000.0
30000.0
25000.0
20000.0
15000.0
10000.0
5000.0
0.0
200.00
400.00
600.00
800.00
1000.00
1200.00
1400.00
1600.00
1800.00
2000.00
b3-H2O
b4-H2O
y8++
y4
a6
y10++
y11++-NH3
y6
b7
b8-H2O
b8
y8-NH3
y8
y9
y10
b11

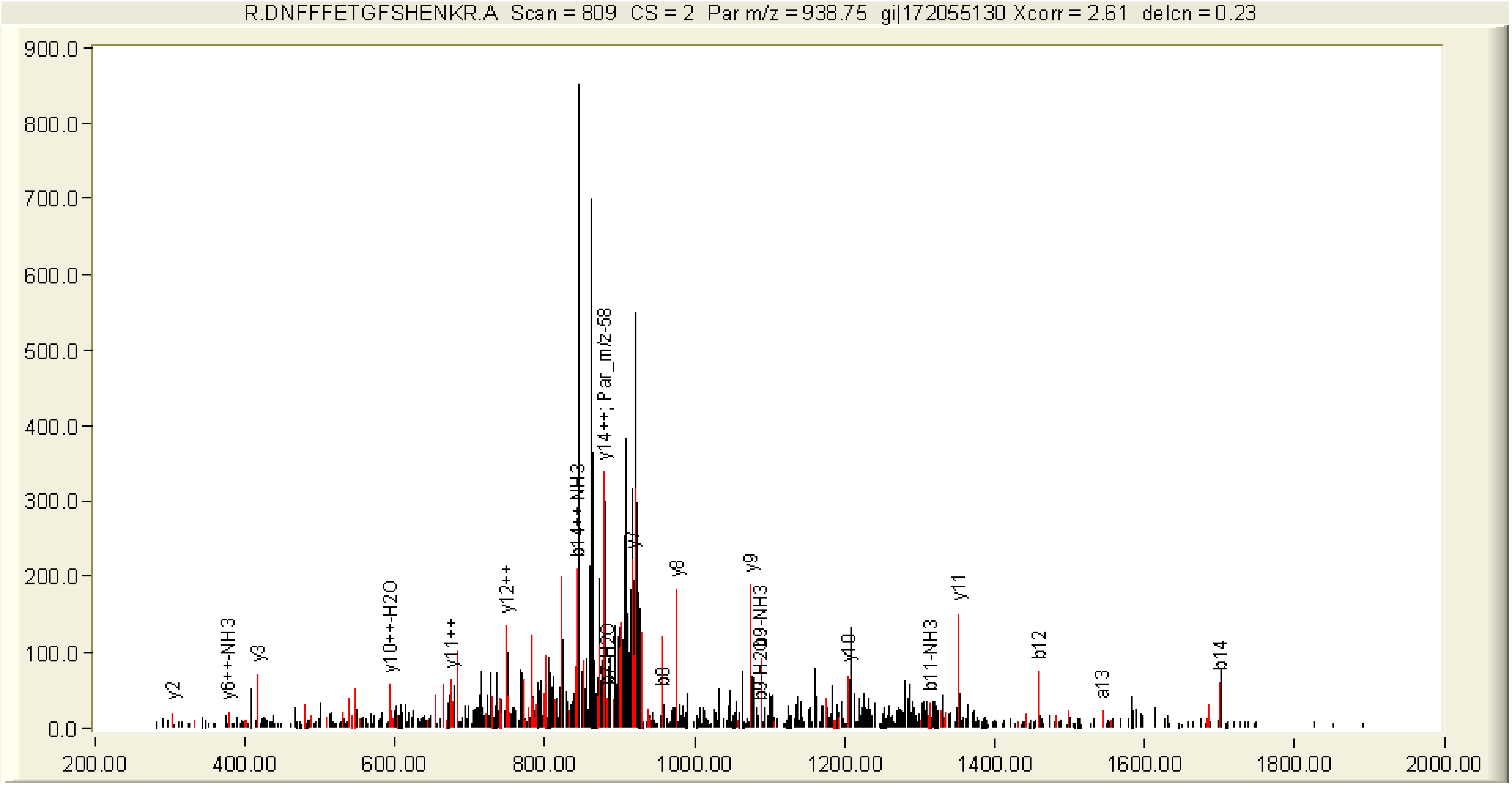

R.DNFFFETGFSHENKR.A Scan = 809 CS = 2 Par m/z = 938.75 gi|172055130 Xcorr = 2.61 delcn = 0.23
900.0
800.0
700.0
600.0
500.0
400.0
300.0
200.0
100.0
0.0
200.00
400.00
600.00
800.00
1000.00
1200.00
1400.00
1600.00
1800.00
2000.00
y2
y6++-NH3
y3
y10++-H2O
y11++
y12++
b14++-NH3
y14++; Par_m/z-58
y7
b8
y8
y9
b9-NH3
y10
b11-NH3
y11
b12
a13
b14

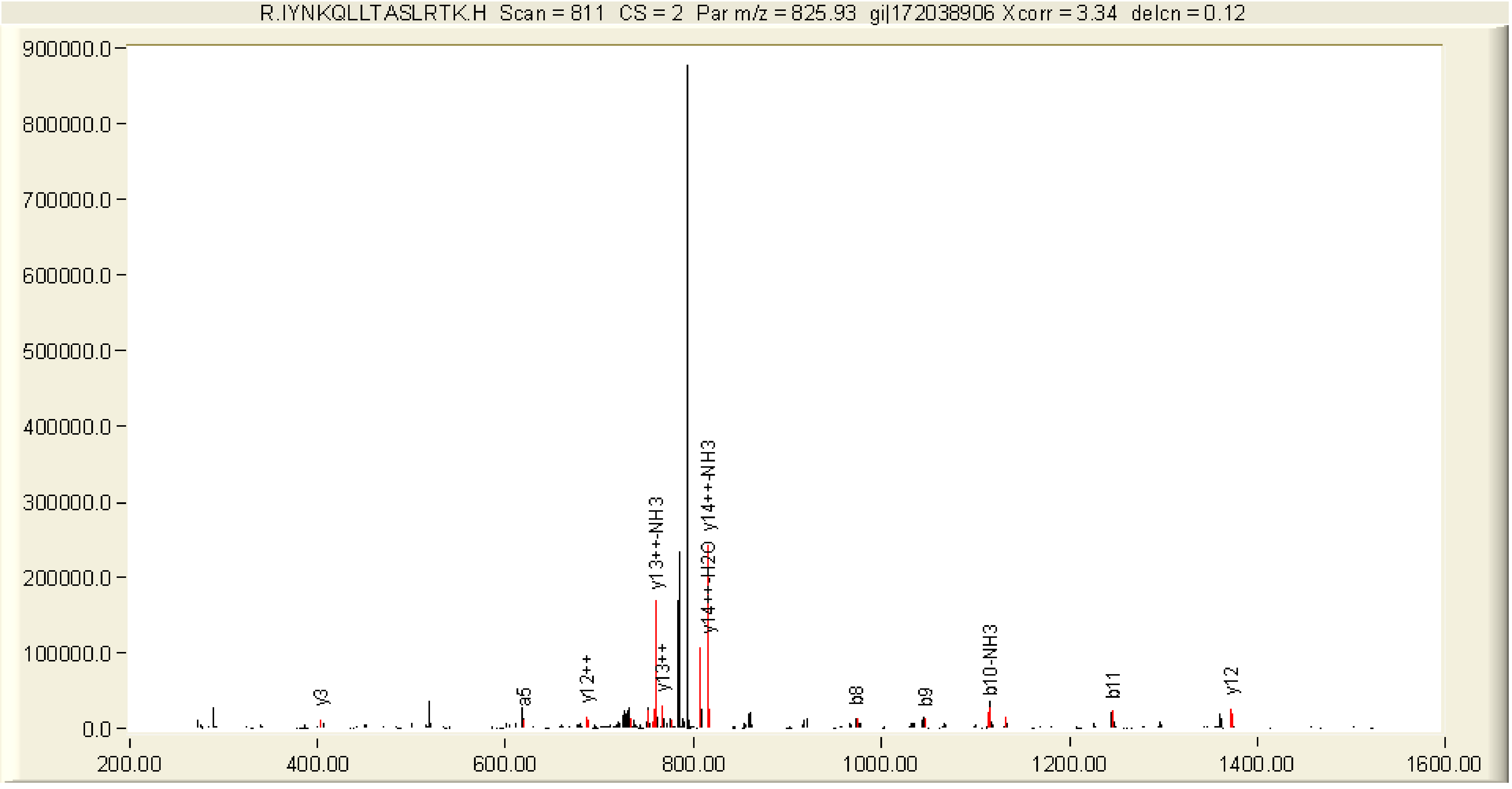
R.IYNKQLLTASLRTK.H Scan = 811 CS = 2 Par m/z = 825.93 gi|172038906 Xcorr = 3.34 delcn = 0.12
900000.0
800000.0
700000.0
600000.0
500000.0
400000.0
300000.0
200000.0
100000.0
0.0
y3
a5
y12++
y13++-NH3
y13++
y14++-H2O
y14++-NH3
b8
b9
b10-NH3
b11
y12
200.00
400.00
600.00
800.00
1000.00
1200.00
1400.00
1600.00

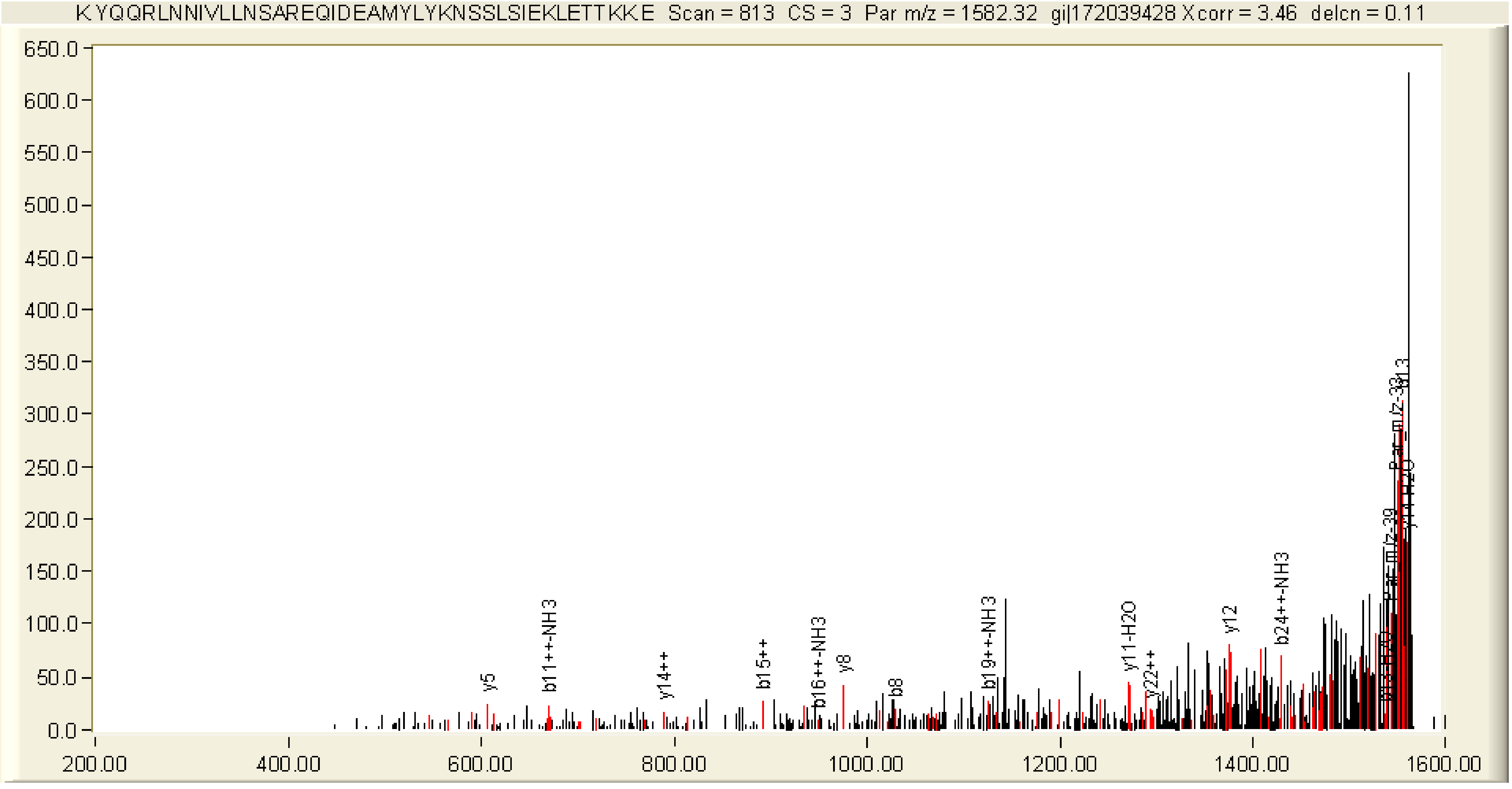
K YQQRLNNIVLLNSAREQIDEAMYLYKNSSLSIEKLETTKK E Scan = 813 CS = 3 Par m/z = 1582.32 gi|172039428 Xcorr = 3.46 delcn = 0.11
650.0
600.0
550.0
500.0
450.0
400.0
350.0
300.0
250.0
200.0
150.0
100.0
50.0
0.0
200.00
400.00
600.00
800.00
1000.00
1200.00
1400.00
1600.00
y5
b11++-NH3
y14++
b15++
b16++-NH3
y8
b8
b19++-NH3
y11-H2O
y22++
y12
b24++-NH3

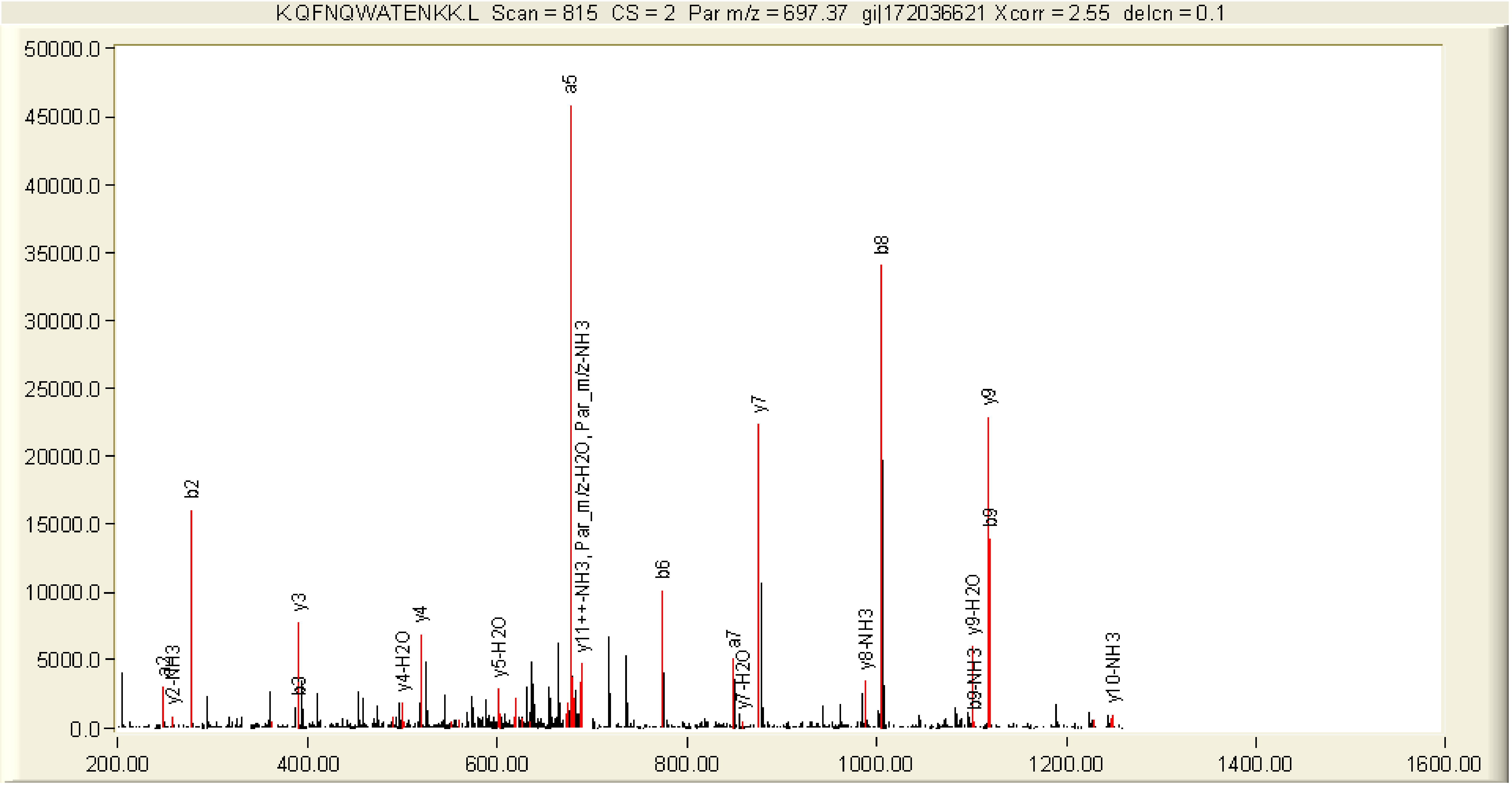

K.QFNQWATENKK.L Scan = 815 CS = 2 Par m/z = 697.37 gi|172036621 Xcorr = 2.55 delcn = 0.1
a5
b8
y11++-NH3, Par_m/z-H2O, Par_m/z-NH3
y7
y9
b2
b9
b6
y3
y4
y4-H2O
y5-H2O
a7
y7-H2O
y8-NH3
y9-H2O
b9-NH3
y10-NH3
y2-NH3
a2
b3
0.0
5000.0
10000.0
15000.0
20000.0
25000.0
30000.0
35000.0
40000.0
45000.0
50000.0
200.00
400.00
600.00
800.00
1000.00
1200.00
1400.00
1600.00

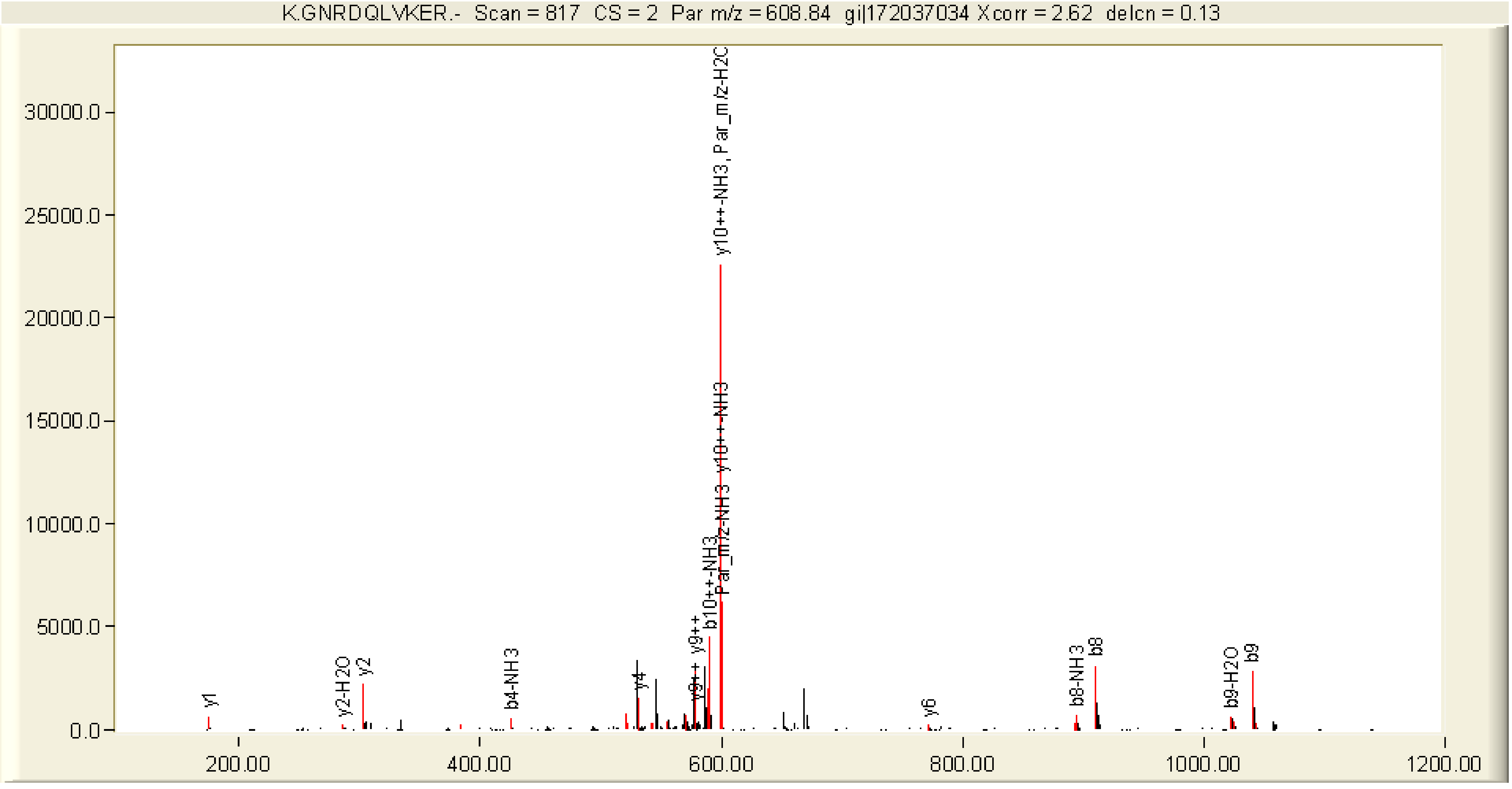

K.GNRDQLVKER.- Scan = 817 CS = 2 Par m/z = 608.84 gi|172037034 Xcorr = 2.62 delcn = 0.13
30000.0
25000.0
20000.0
15000.0
10000.0
5000.0
0.0
200.00
400.00
600.00
800.00
1000.00
1200.00
y1
y2-H2O
y2
b4-NH3
y4
y9++
b10++-NH3
Par_m/z-NH3
y10++-NH3
y10++-NH3, Par_m/z-H2O
y6
b8-NH3
b8
b9-H2O
b9

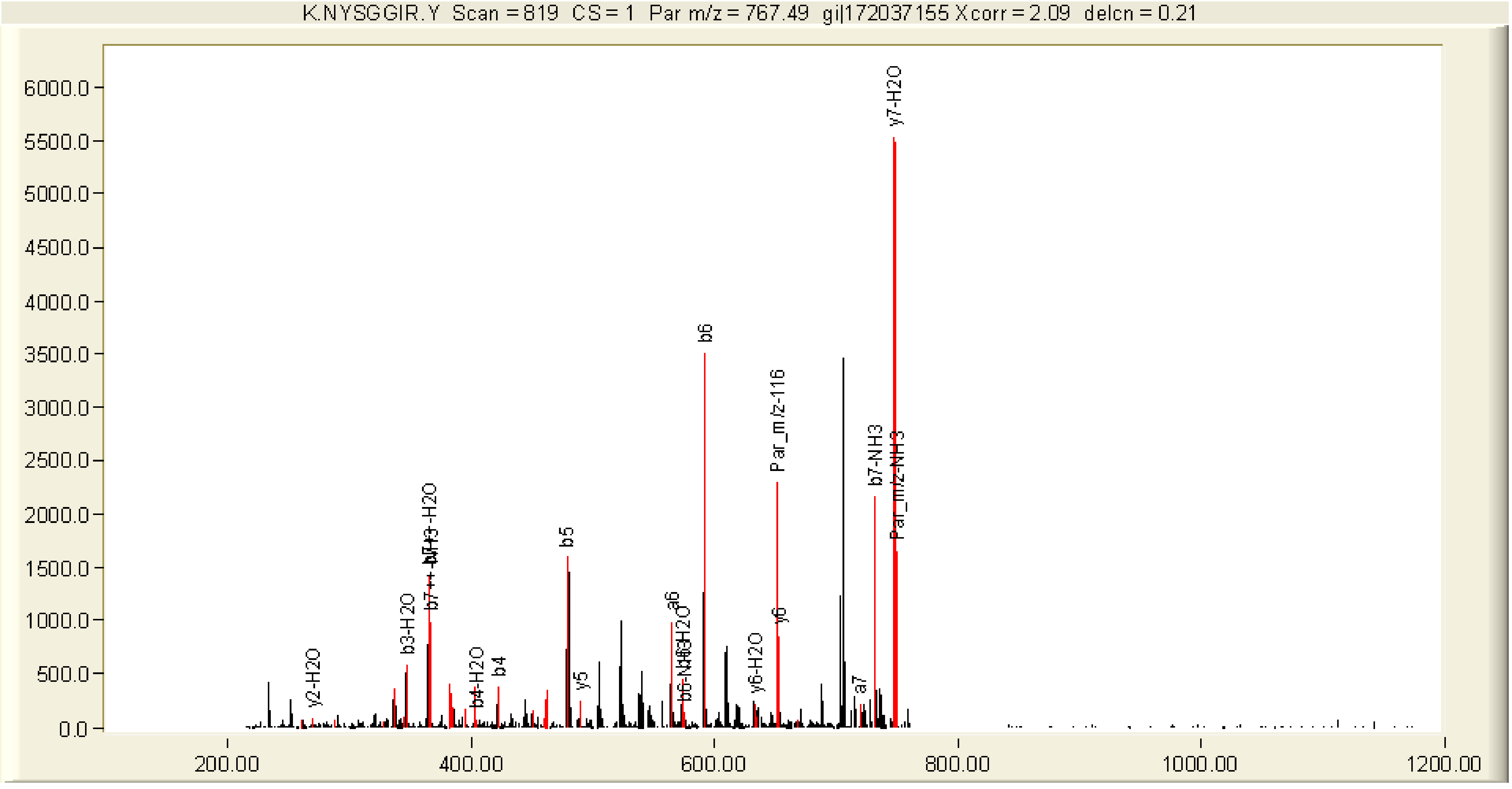
K.NYSGGIR.Y Scan = 819 CS = 1 Par m/z = 767.49 gi|172037155 Xcorr = 2.09 delcn = 0.21
6000.0
5500.0
5000.0
4500.0
4000.0
3500.0
3000.0
2500.0
2000.0
1500.0
1000.0
500.0
0.0
200.00
400.00
600.00
800.00
1000.00
1200.00
y7-H2O
b6
Par_m/z-116
b7-NH3
Par_m/z-NH3
b5
b3-H2O
y2-H2O
b4-H2O
b4
y5
a6
y6
y6-H2O
a7

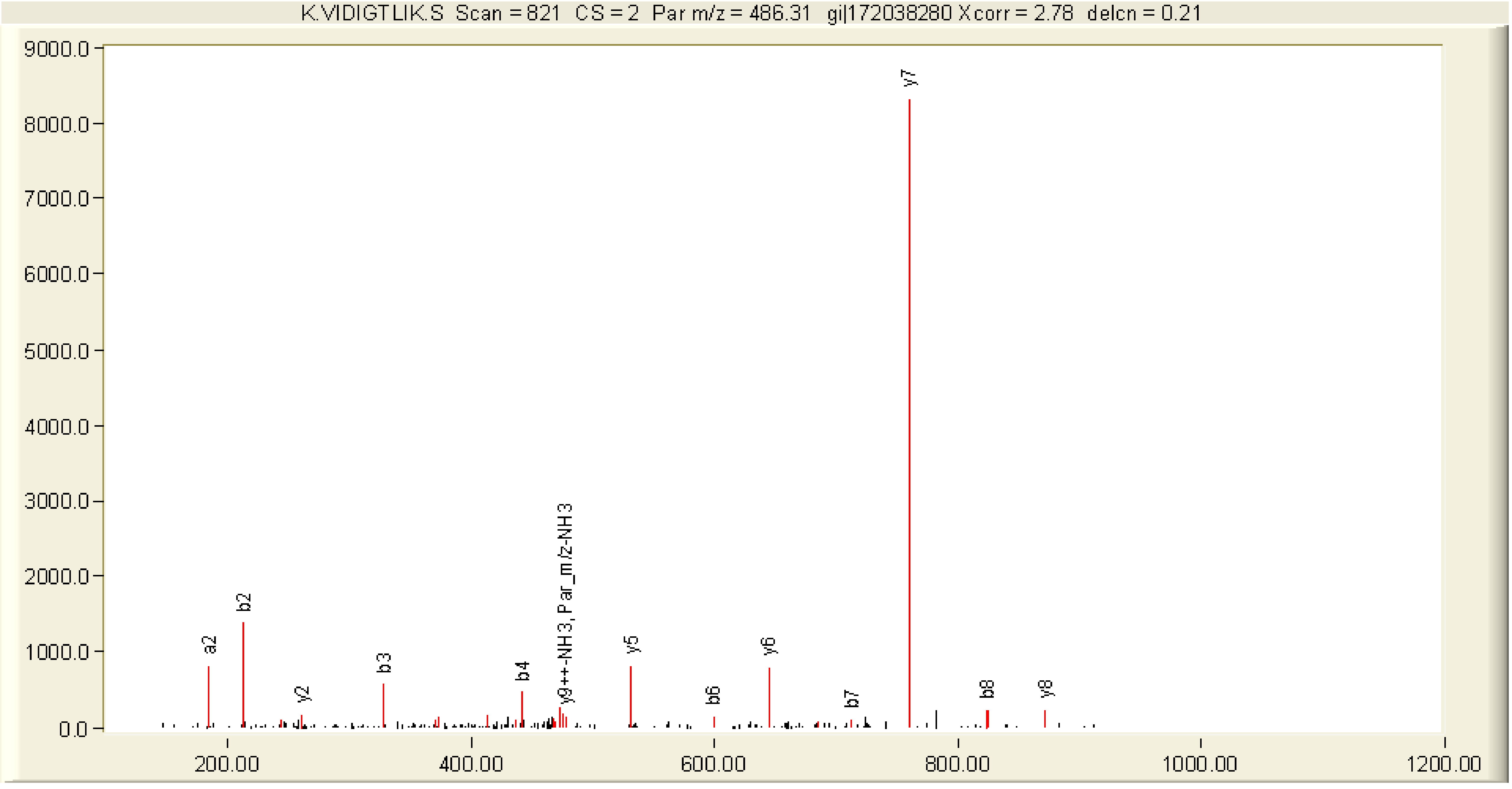

K.VIDIGTLIK.S Scan = 821 CS = 2 Par m/z = 486.31 gi|172038280 Xcorr = 2.78 delcn = 0.21
9000.0
8000.0
7000.0
6000.0
5000.0
4000.0
3000.0
2000.0
1000.0
0.0
200.00
400.00
600.00
800.00
1000.00
1200.00
a2
b2
y2
b3
b4
y9++-NH3, Par_m/z-NH3
y5
b6
y6
b7
y7
b8
y8

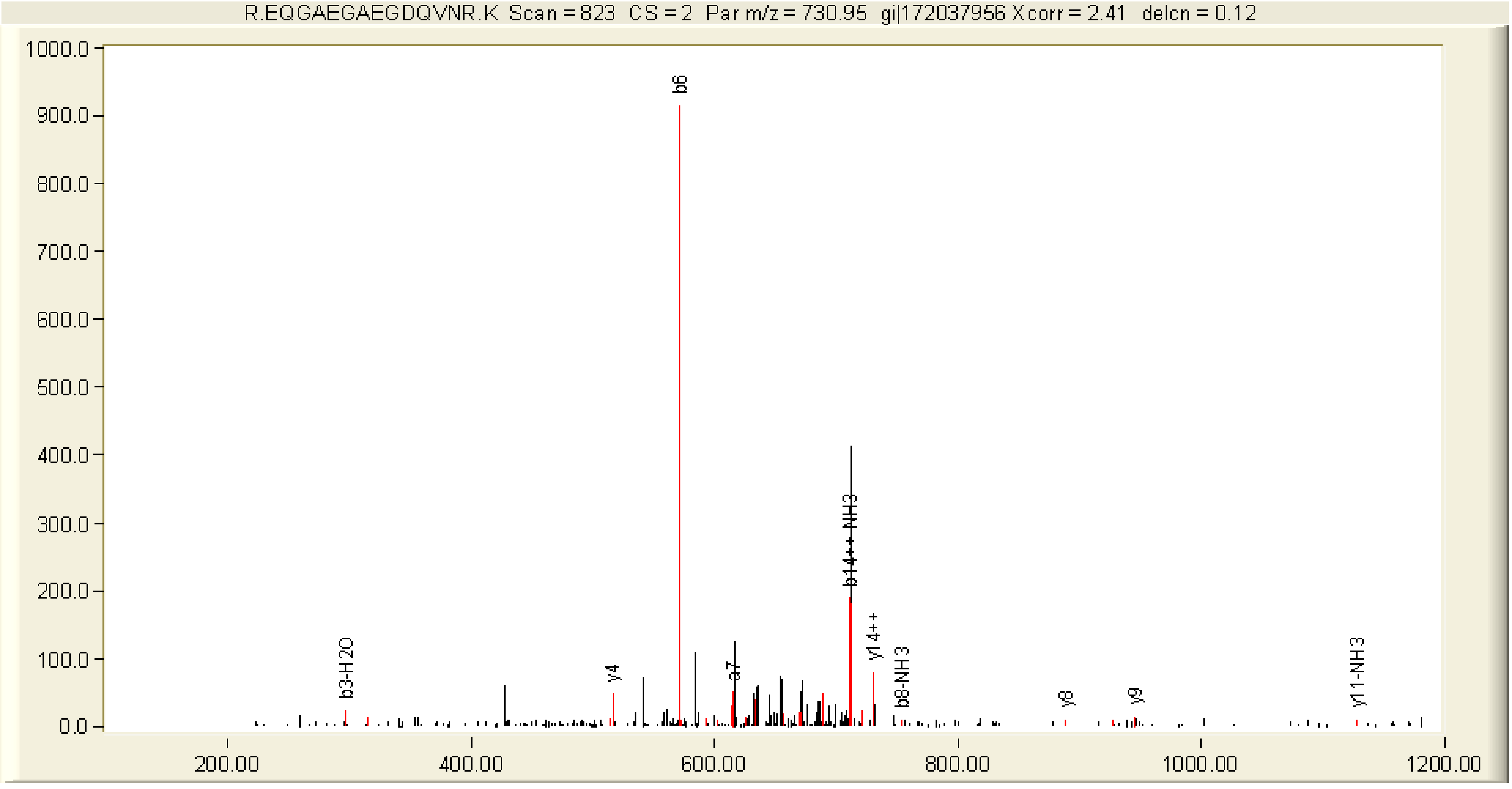

R.EQGAEGAEGDQVNR.K Scan = 823 CS = 2 Par m/z = 730.95 gi|172037956 Xcorr = 2.41 delcn = 0.12
1000.0
900.0
800.0
700.0
600.0
500.0
400.0
300.0
200.0
100.0
0.0
200.00
400.00
600.00
800.00
1000.00
1200.00
b6
b14++-NH3
y14++
b3-H2O
y4
a7
b8-NH3
y8
y9
y11-NH3

K.ALHETFPTLPIYASHVTK.Q Scan = 825 CS = 3 Par m/z = 675.69 gi|172039432 Xcorr = 4.15 delcn = 0.38

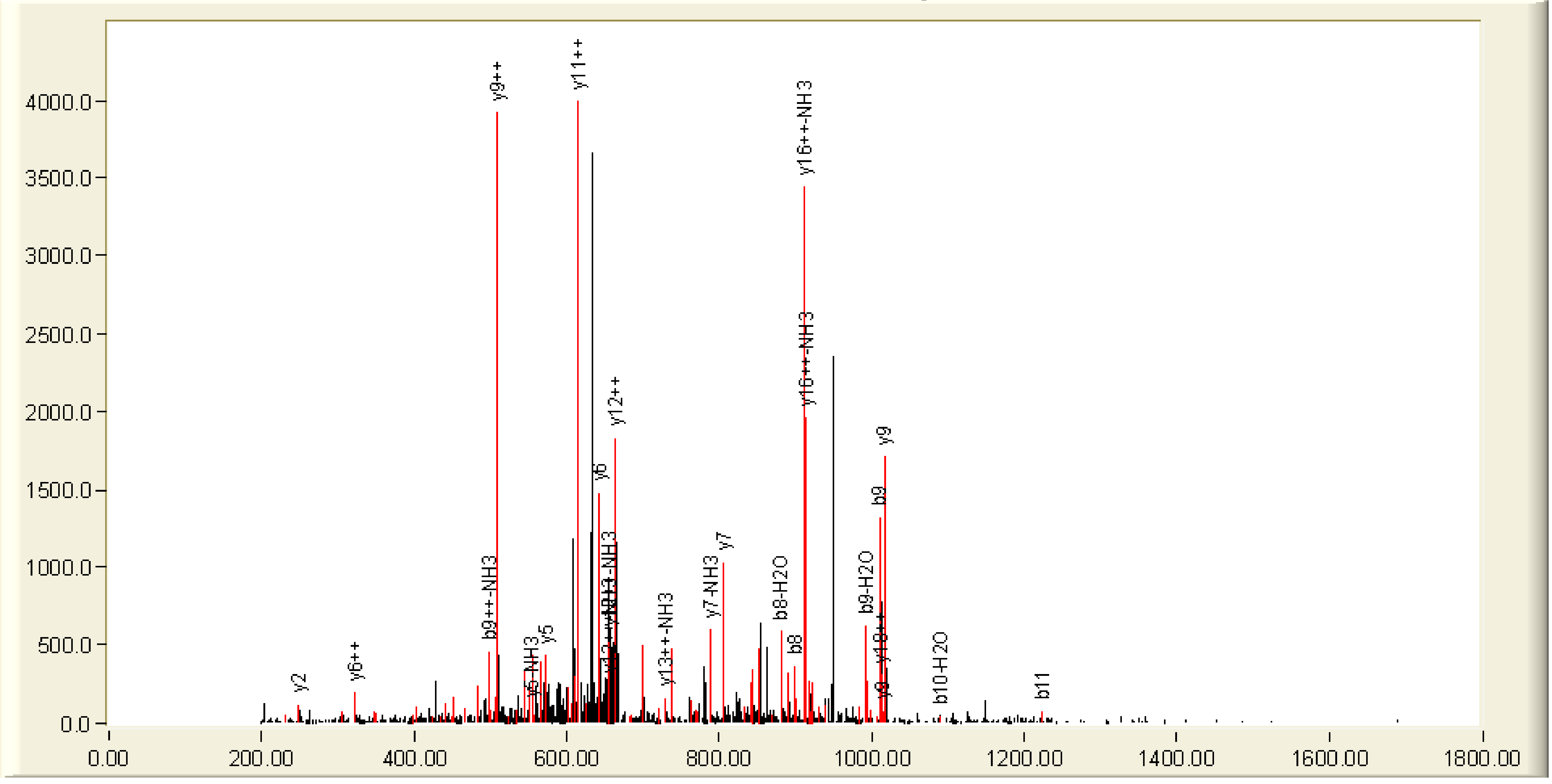

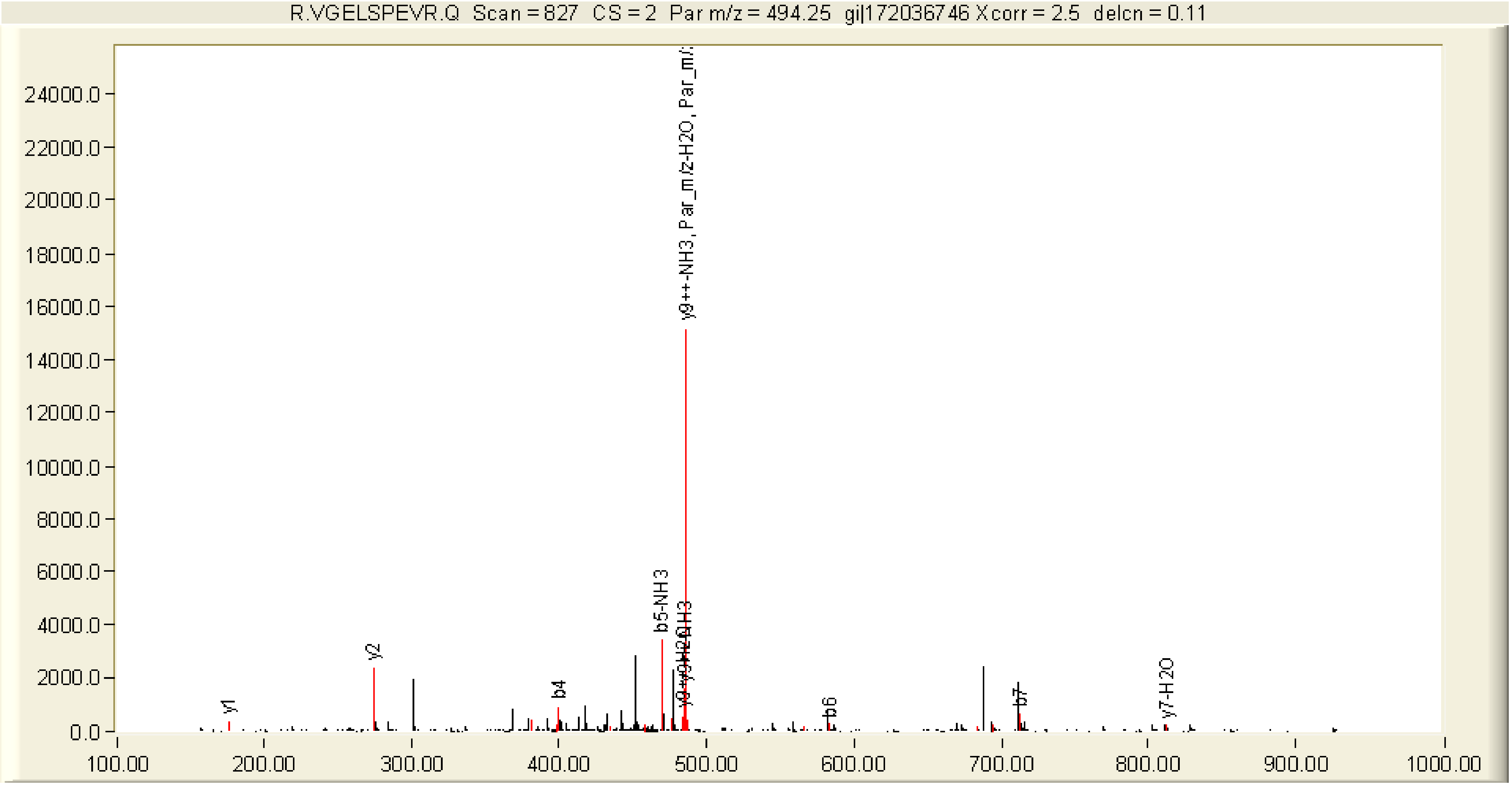
R.VGELSPEVR.Q Scan = 827 CS = 2 Par m/z = 494.25 gi|172036746 Xcorr = 2.5 delcn = 0.11
y9++-NH3, Par_m/z-H2O, Par_m/
b5-NH3
y1
y2
b4
b6
b7
y7-H2O
24000.0
22000.0
20000.0
18000.0
16000.0
14000.0
12000.0
10000.0
8000.0
6000.0
4000.0
2000.0
0.0
100.00
200.00
300.00
400.00
500.00
600.00
700.00
800.00
900.00
1000.00

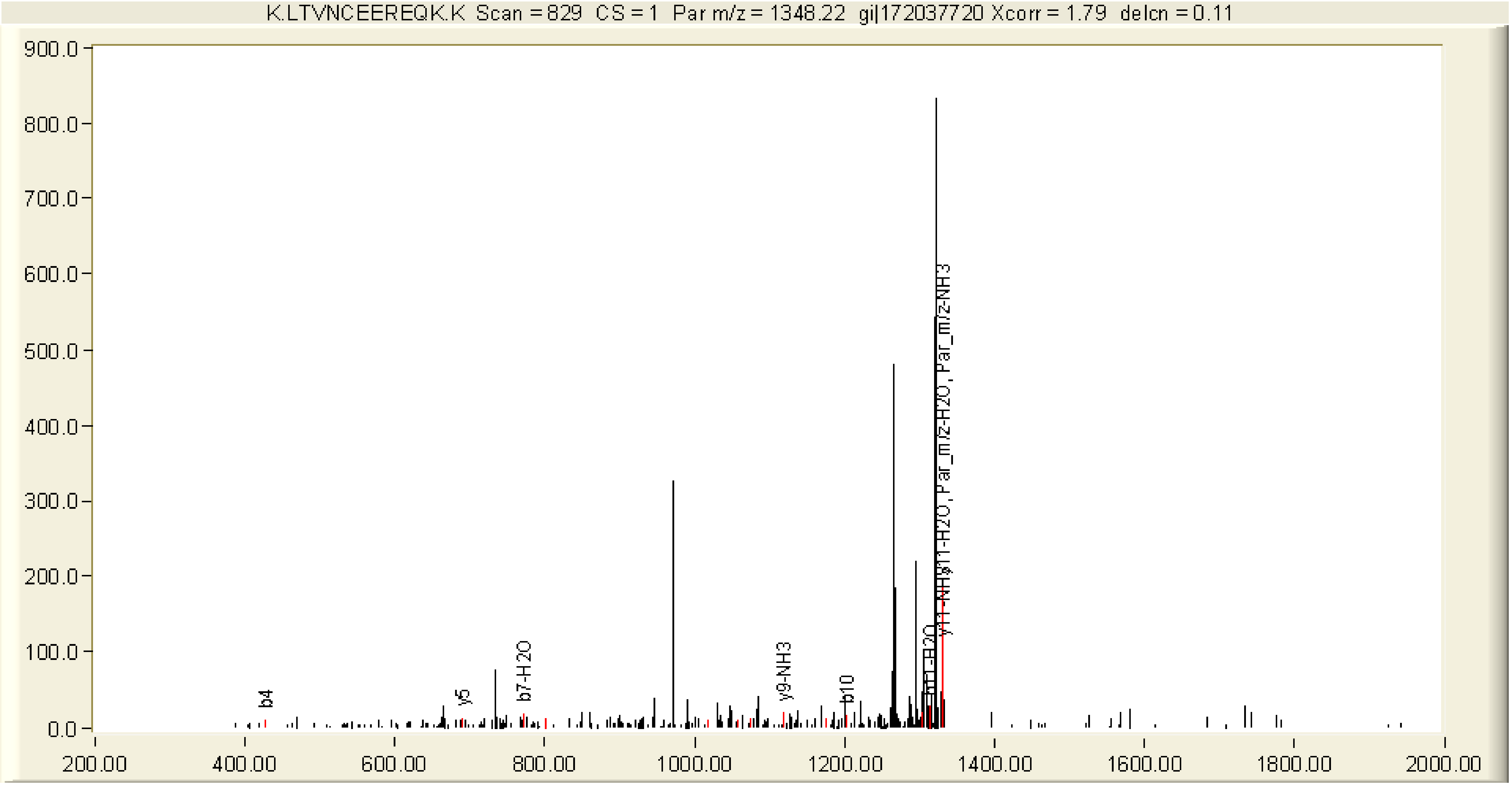
K.LTVNCEEREQK.K Scan = 829 CS = 1 Par m/z = 1348.22 gi|172037720 Xcorr = 1.79 delcn = 0.11
b4
y5
b7-H2O
y9-NH3
b10
y11-H2O, Par_m/z-H2O, Par_m/z-NH3
900.0
800.0
700.0
600.0
500.0
400.0
300.0
200.0
100.0
0.0
200.00
400.00
600.00
800.00
1000.00
1200.00
1400.00
1600.00
1800.00
2000.00

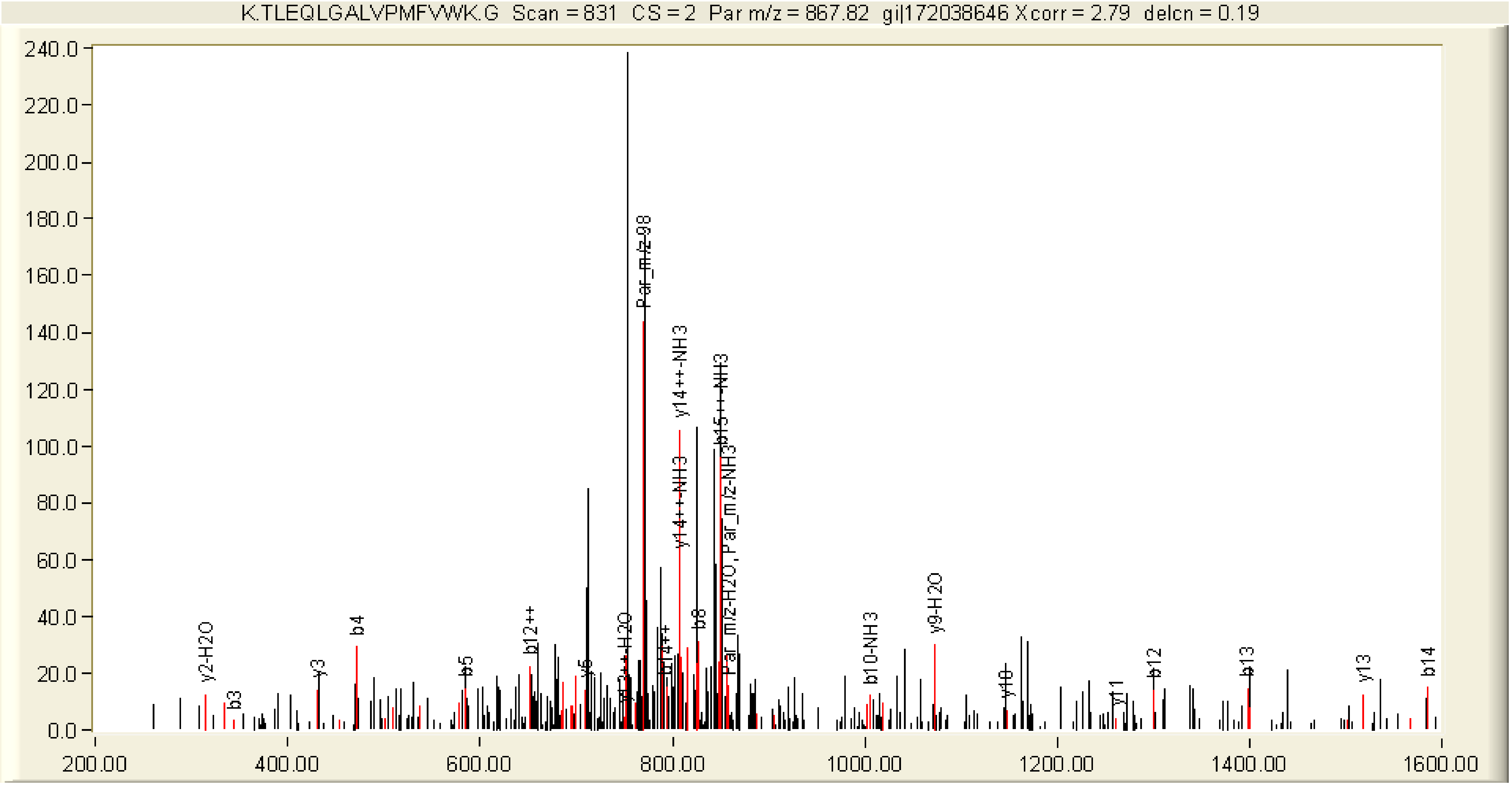

K.TLEQLGALVPMFVWK.G Scan = 831 CS = 2 Par m/z = 867.82 gi|172038646 Xcorr = 2.79 delcn = 0.19
240.0
220.0
200.0
180.0
160.0
140.0
120.0
100.0
80.0
60.0
40.0
20.0
0.0
200.00
400.00
600.00
800.00
1000.00
1200.00
1400.00
1600.00
y2-H2O
b3
y3
b4
b5
b12++
y6
Par_m/z-98
y14++-NH3
y14++-NH3
b15++-NH3
Par_m/z-H2O,Par_m/z-NH3
b8
b14++
b10-NH3
y9-H2O
y10
y11
b12
b13
y13
b14

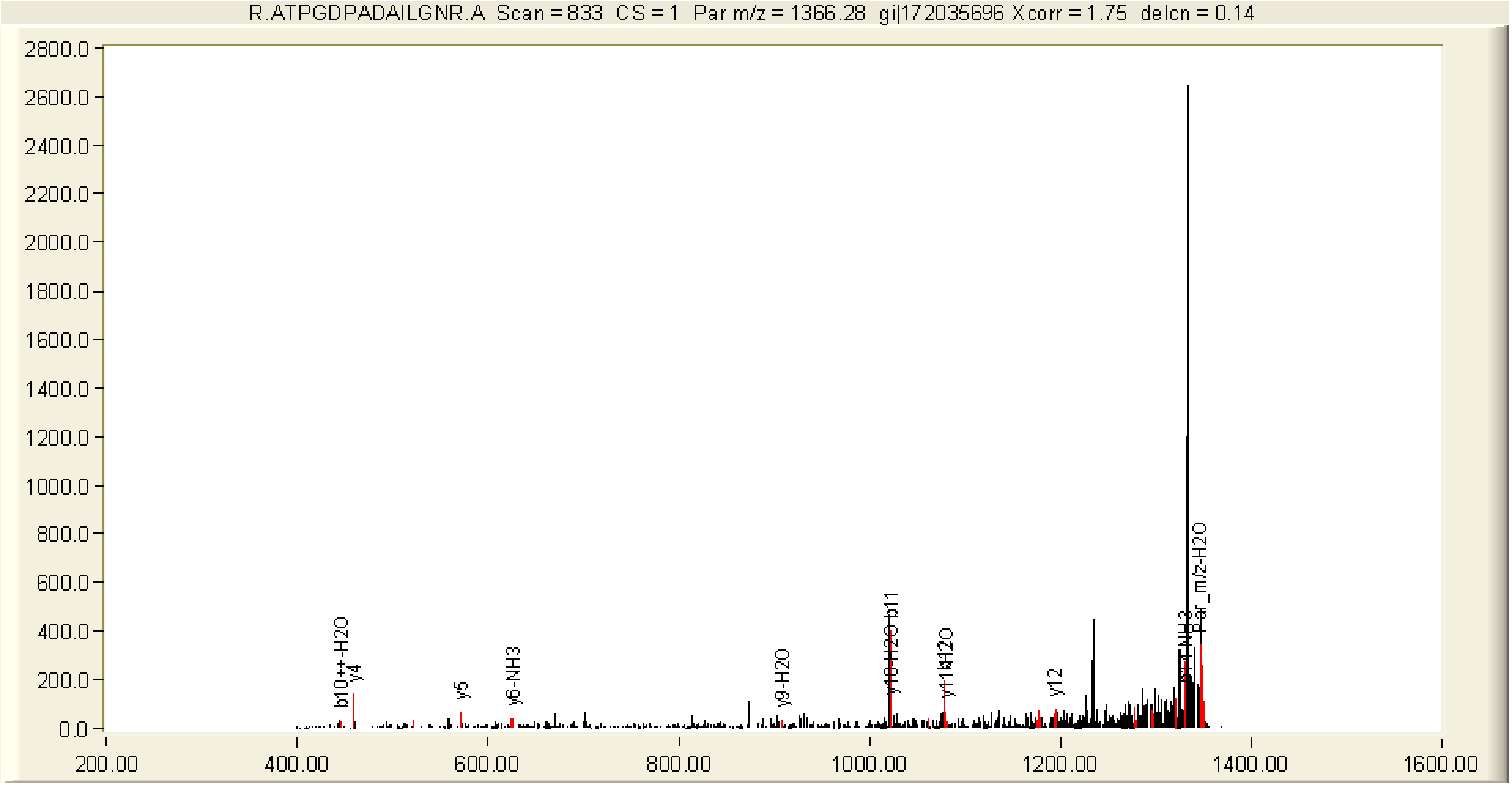

R.ATPGDPADAILGNR.A Scan = 833 CS = 1 Par m/z = 1366.28 gi|172035696 Xcorr = 1.75 delcn = 0.14
2800.0
2600.0
2400.0
2200.0
2000.0
1800.0
1600.0
1400.0
1200.0
1000.0
800.0
600.0
400.0
200.0
0.0
200.00
400.00
600.00
800.00
1000.00
1200.00
1400.00
1600.00
b10++-H2O
y4
y5
y6-NH3
y9-H2O
y10-H2O
b11
y11-H2O
y12
Par_m/z-H2O

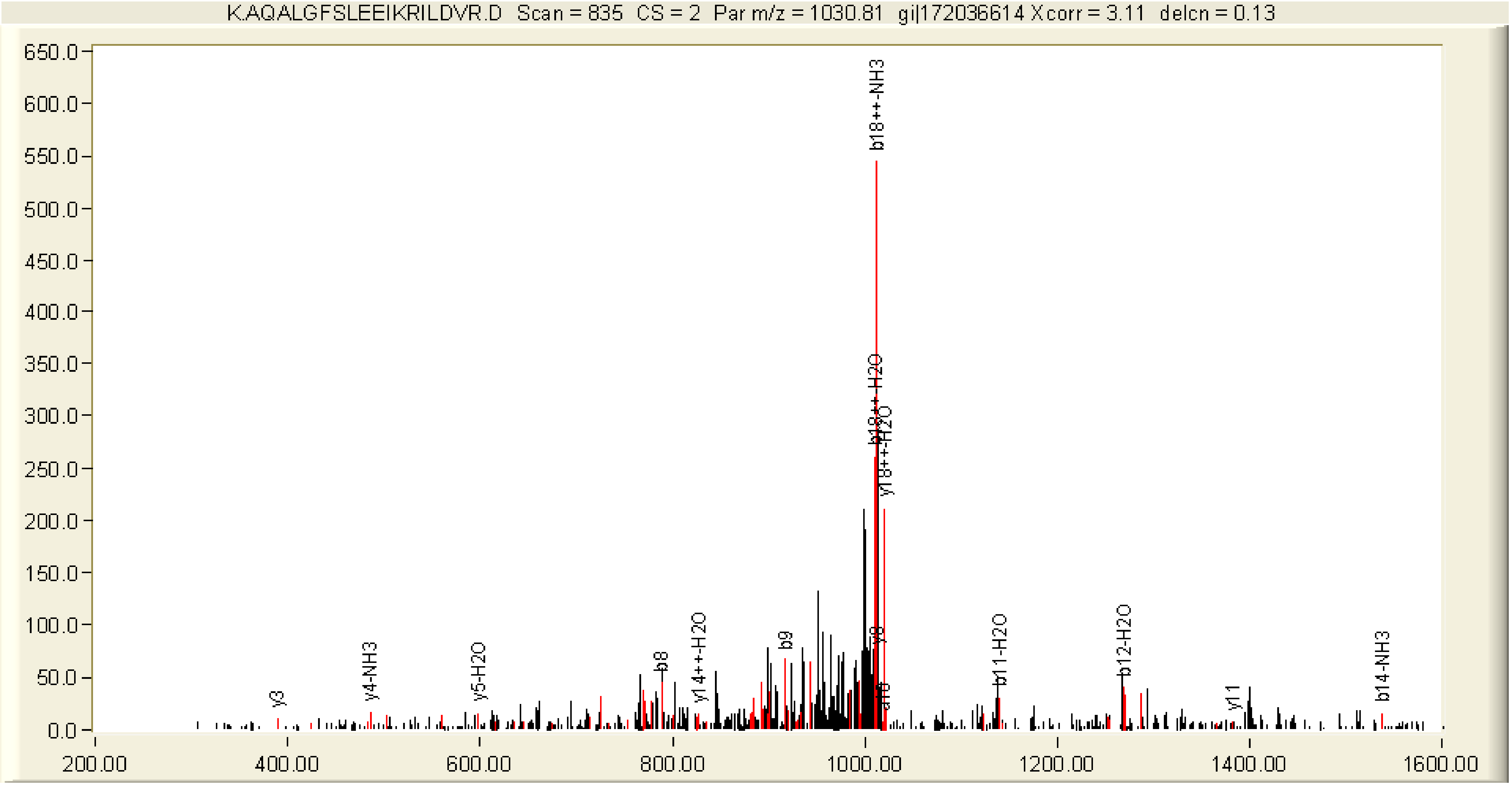

K.AQALGFSLEEIKRILDVR.D Scan = 835 CS = 2 Par m/z = 1030.81 gi|172036614 Xcorr = 3.11 delcn = 0.13
650.0
600.0
550.0
500.0
450.0
400.0
350.0
300.0
250.0
200.0
150.0
100.0
50.0
0.0
200.00
400.00
600.00
800.00
1000.00
1200.00
1400.00
1600.00
b18++-NH3
b18++-H2O
y18++-H2O
y3
y4-NH3
y5-H2O
b8
y14++-H2O
b9
y8
b11-H2O
b12-H2O
y11
b14-NH3

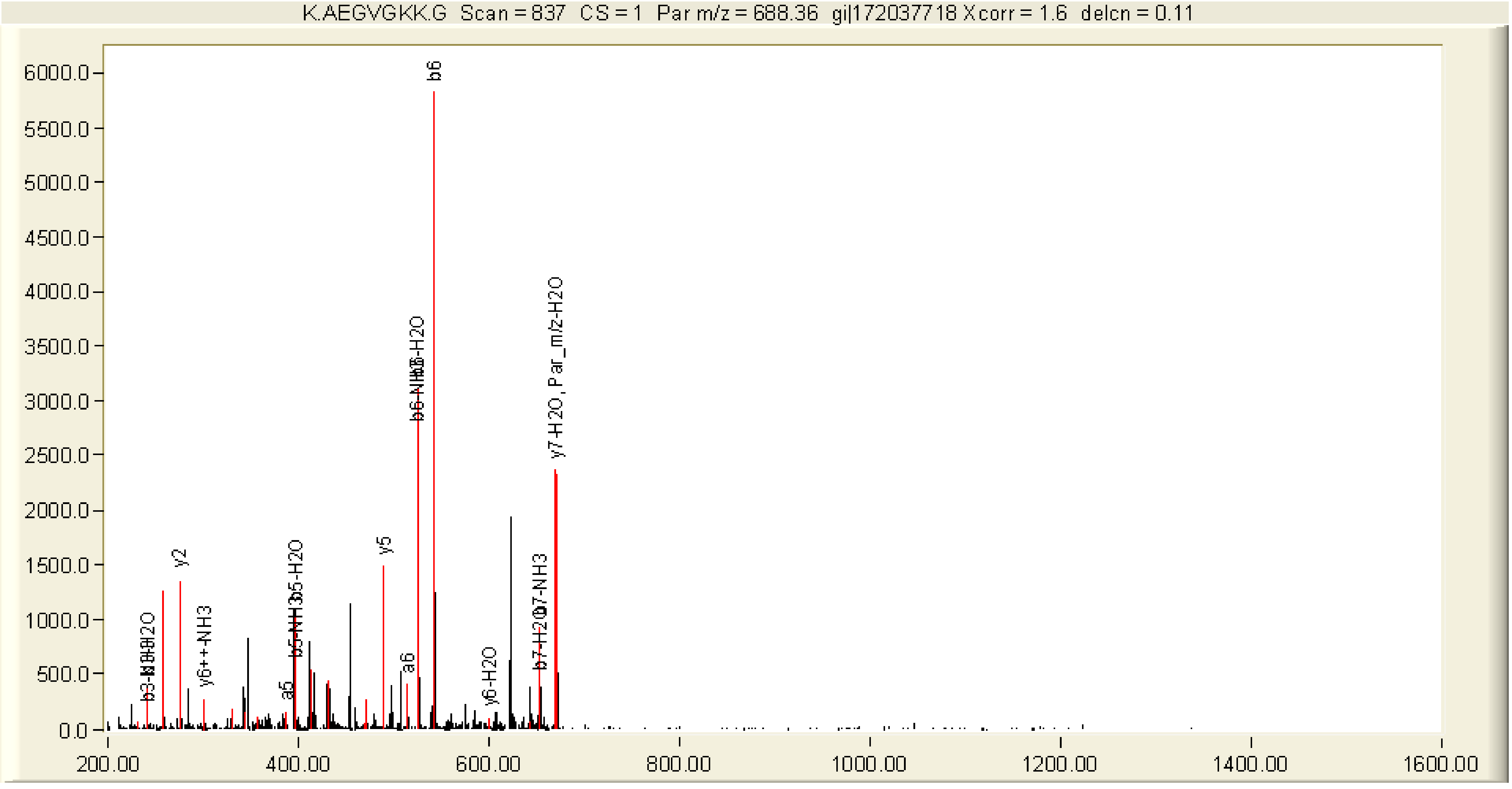

K.AEGVGKK.G Scan = 837 CS = 1 Par m/z = 688.36 gi|172037718 Xcorr = 1.6 delcn = 0.11
b6
y7-H2O, Par_m/z-H2O
y2
y6++-NH3
y5
a5
a6
b5-H2O
y6-H2O
b7-NH3
6000.0
5500.0
5000.0
4500.0
4000.0
3500.0
3000.0
2500.0
2000.0
1500.0
1000.0
500.0
0.0
200.00
400.00
600.00
800.00
1000.00
1200.00
1400.00
1600.00

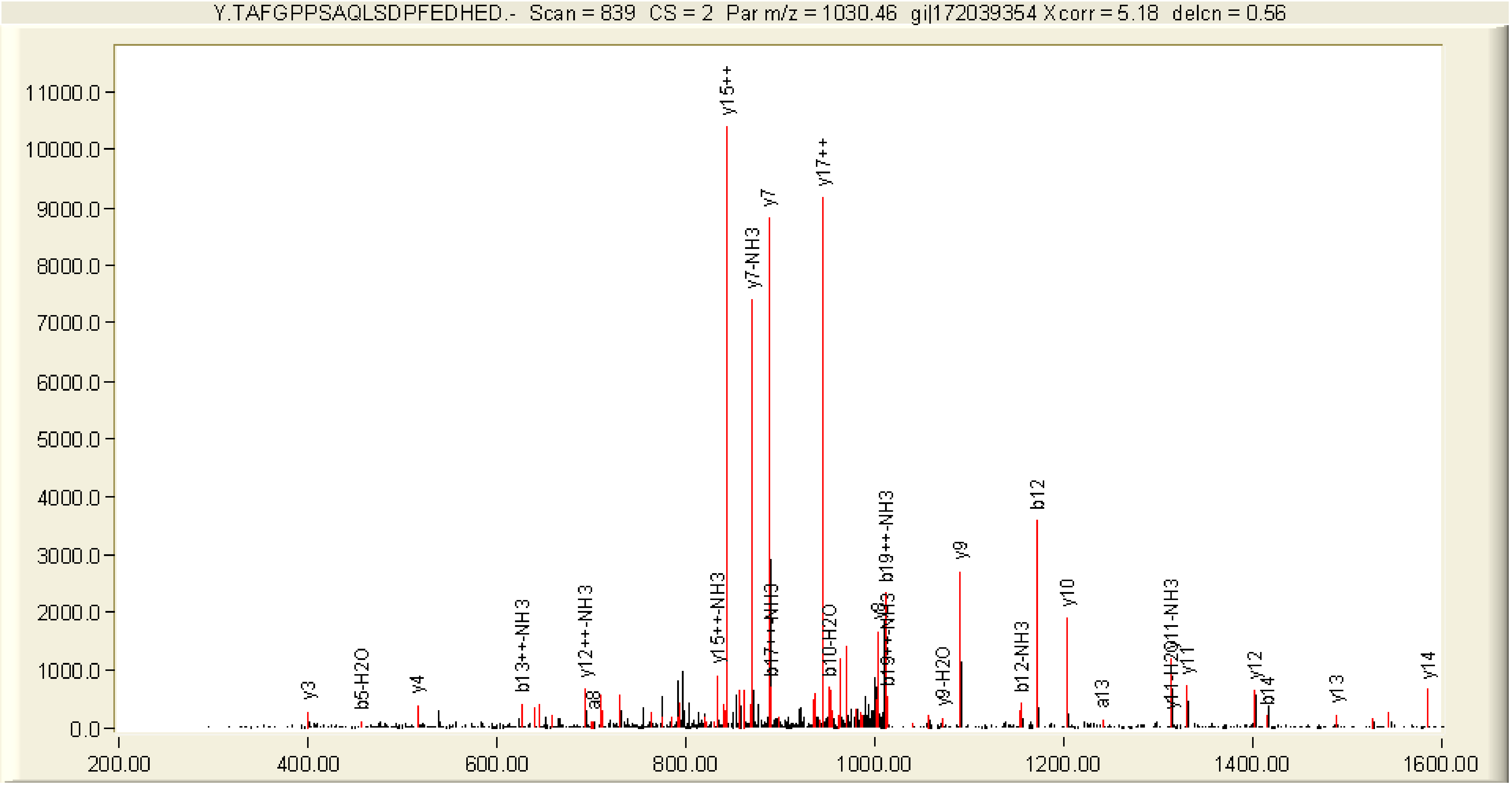
Y.TAFGPPSAQLSDPFEDHED.- Scan = 839 CS = 2 Par m/z = 1030.46 gi|172039354 Xcorr = 5.18 delcn = 0.56
y15++
y17++
y7
y7-NH3
b12
y9
b19++-NH3
y10
y15++-NH3
y12++-NH3
b13++-NH3
b10-H2O
y9-H2O
b12-NH3
y11-NH3
y11
y12
y14
y13
a13
b14
y4
b5-H2O
y3
a8

R.LYRTGDLVR.Y Scan = 841 CS = 2 Par m/z = 547.33 gi|172037947 Xcorr = 2.41 delcn = 0.13

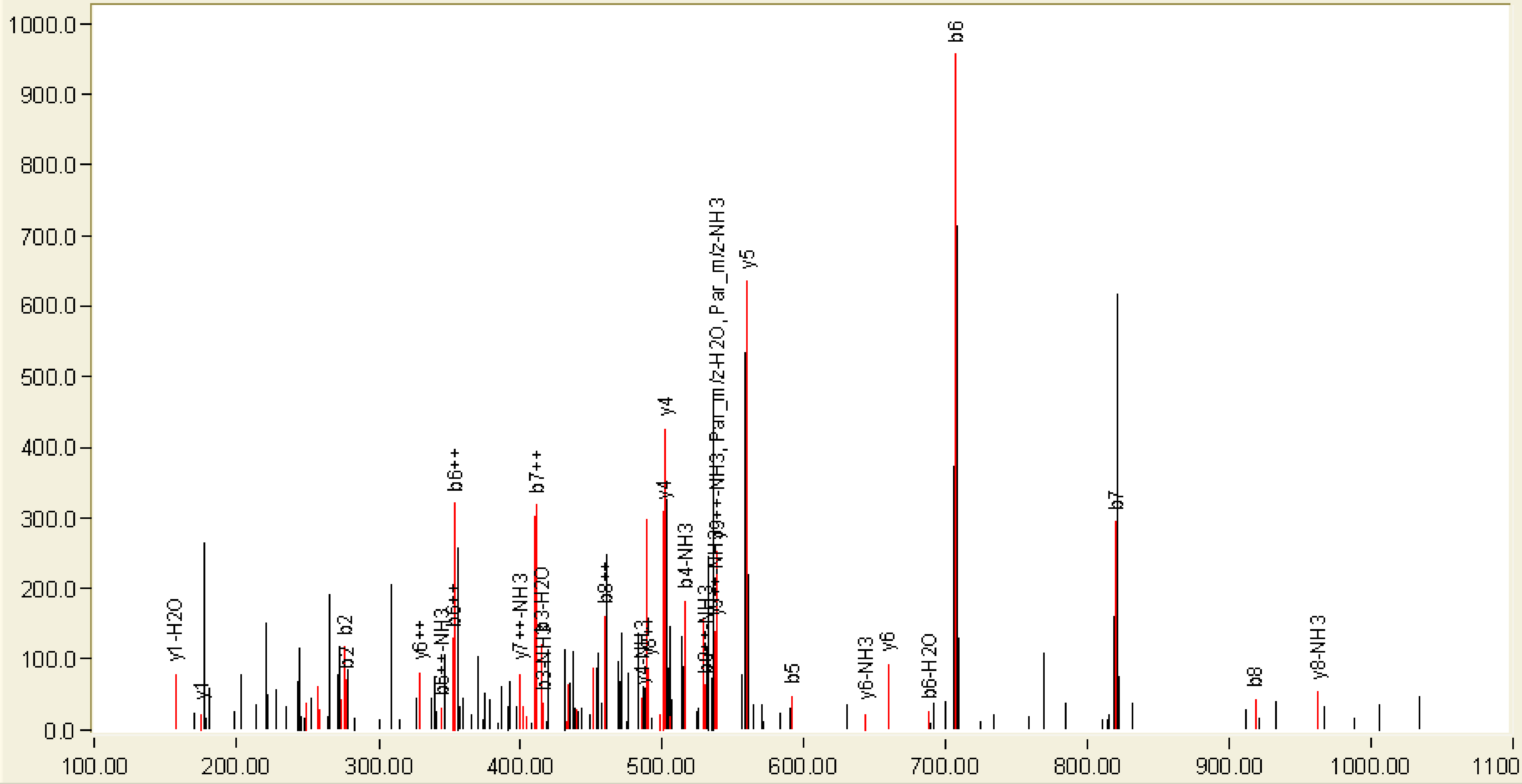

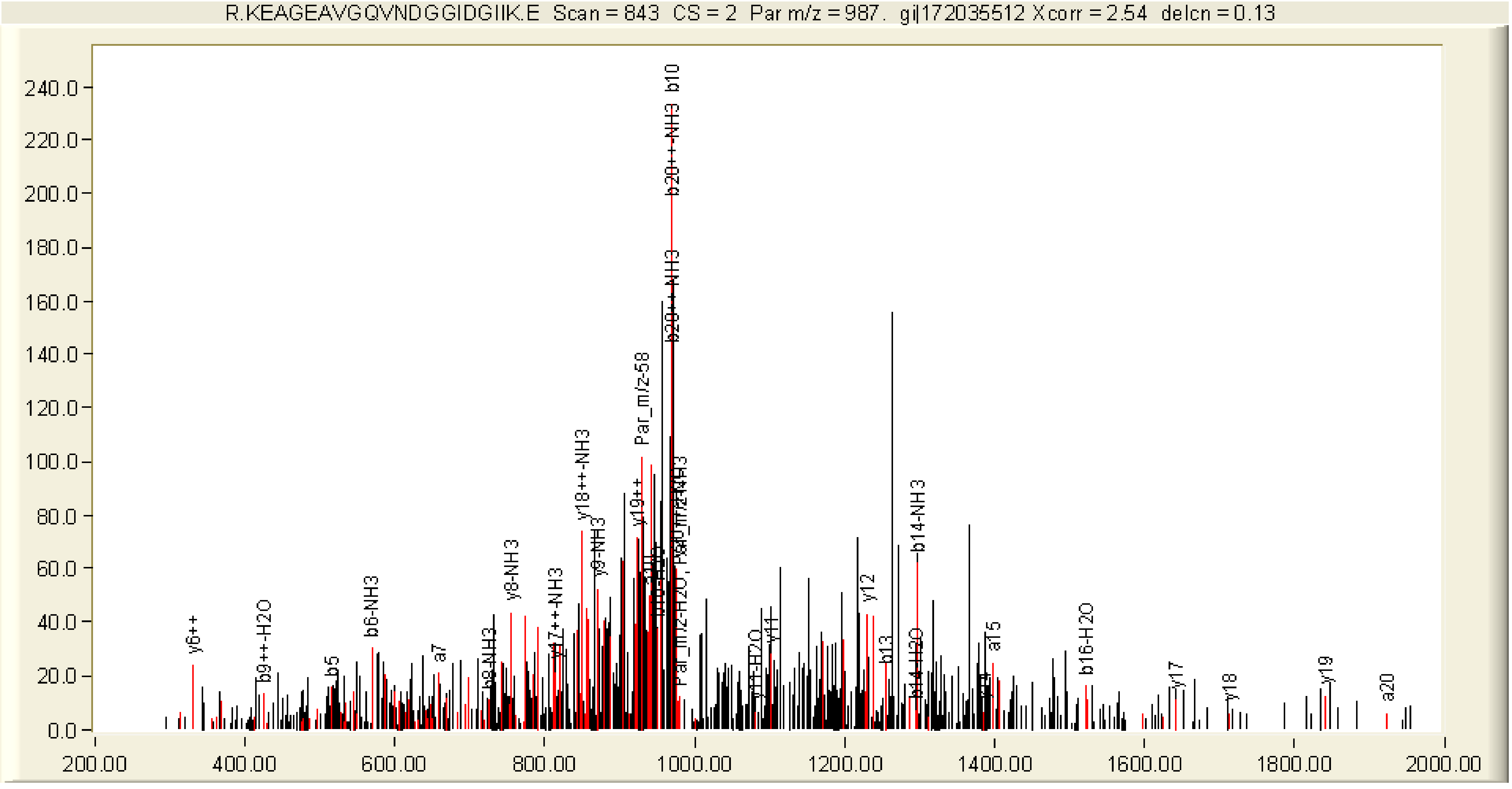
R.KEAGEAVGQVNDGGIDGIIK.E Scan = 843 CS = 2 Par m/z = 987. gi|172035512 Xcorr = 2.54 delcn = 0.13
240.0
220.0
200.0
180.0
160.0
140.0
120.0
100.0
80.0
60.0
40.0
20.0
0.0
200.00
400.00
600.00
800.00
1000.00
1200.00
1400.00
1600.00
1800.00
2000.00
y6++
b9++-H2O
b5
b6-NH3
a7
b8-NH3
y8-NH3
y17++-NH3
y18++-NH3
y9-NH3
y19++
Par_m/z-58
b20++-NH3
b10
y12
b13
b14-NH3
b14-H2O
a15
b16-H2O
y17
y18
y19
a20

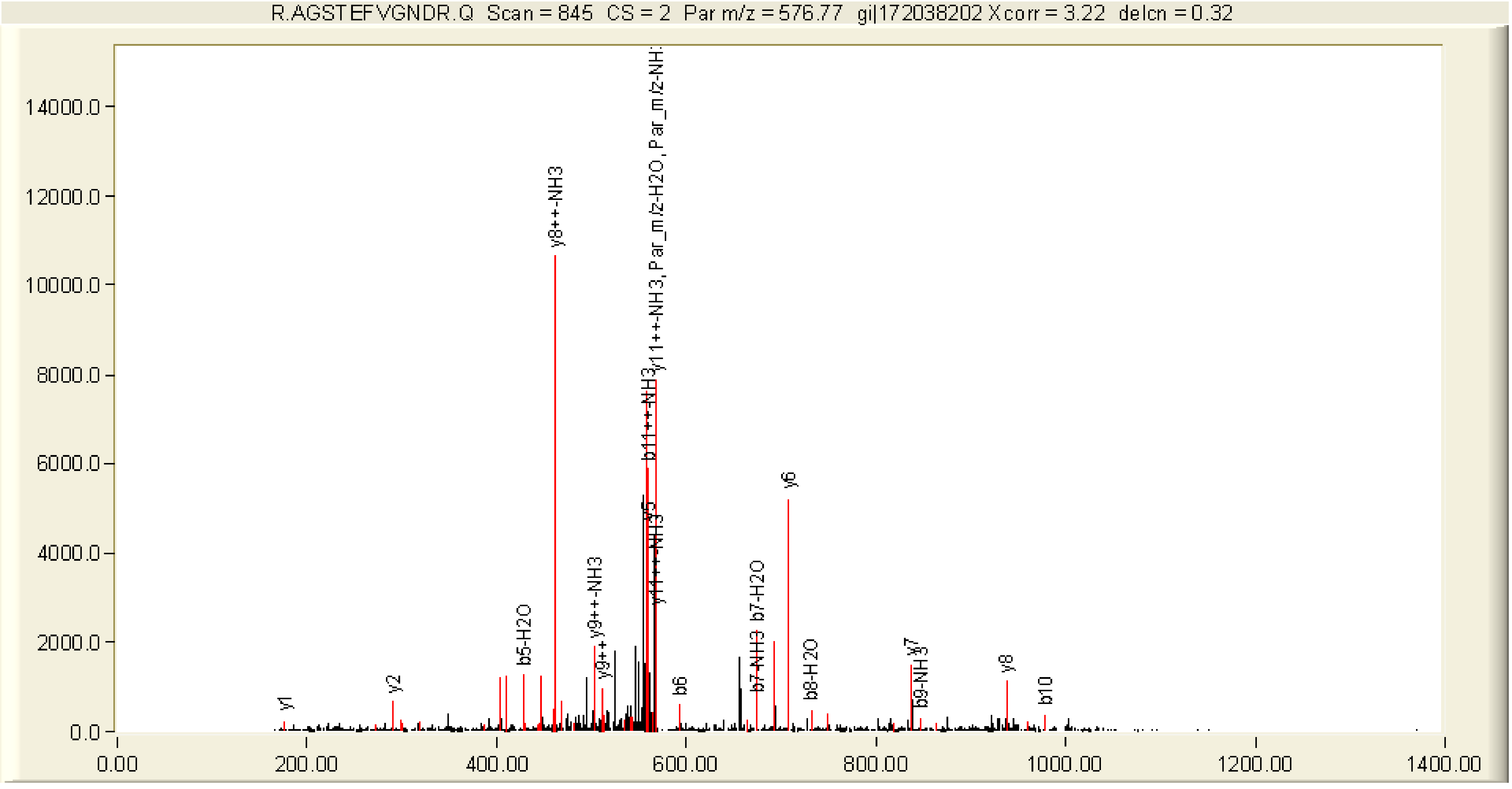
R.AGSTEFVGNDR.Q Scan = 845 CS = 2 Par m/z = 576.77 gi|172038202 Xcorr = 3.22 delcn = 0.32
14000.0
12000.0
10000.0
8000.0
6000.0
4000.0
2000.0
0.0
0.00
200.00
400.00
600.00
800.00
1000.00
1200.00
1400.00
y1
y2
b5-H2O
y8++-NH3
y9++
y9++-NH3
b11++-NH3
y5
y11++-NH3
y11++-NH3, Par_m/z-H2O, Par_m/z-NH3
b6
b7-NH3
b7-H2O
y6
b8-H2O
b9-NH3
y7
y8
b10

K.LPTPAEWVTVRRERVKDAVNTIWWLCKEPHPKANNR.N Scan = 847 CS = 3 Par m/z = 1438.26 gi|172037500 Xcorr = 3.35 delcn = 0.15

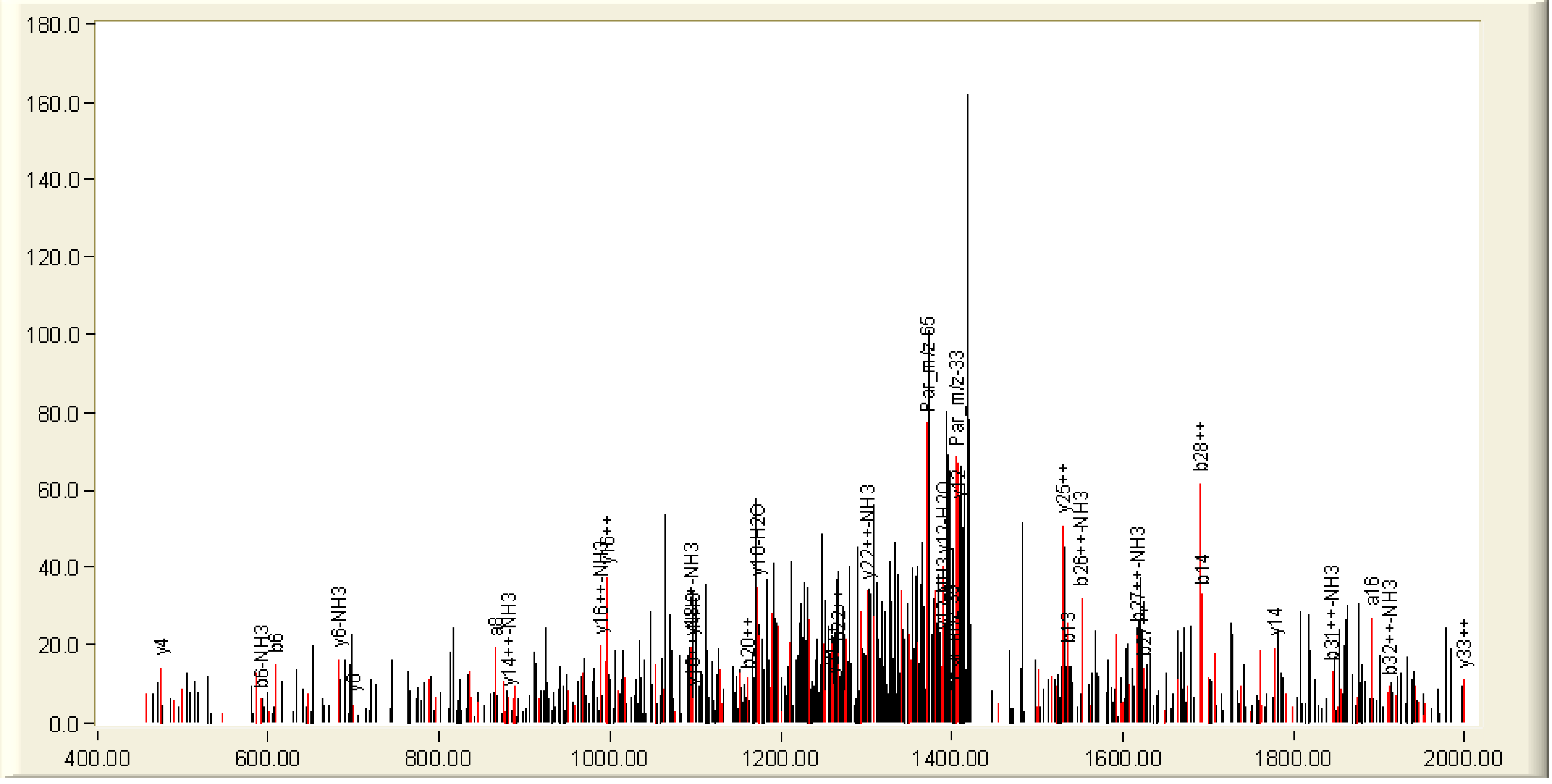

R.VLIHVNTSKPQK.E Scan = 849 CS = 2 Par m/z = 682.41 gi|172038285 Xcorr = 2.9 delcn = 0.32

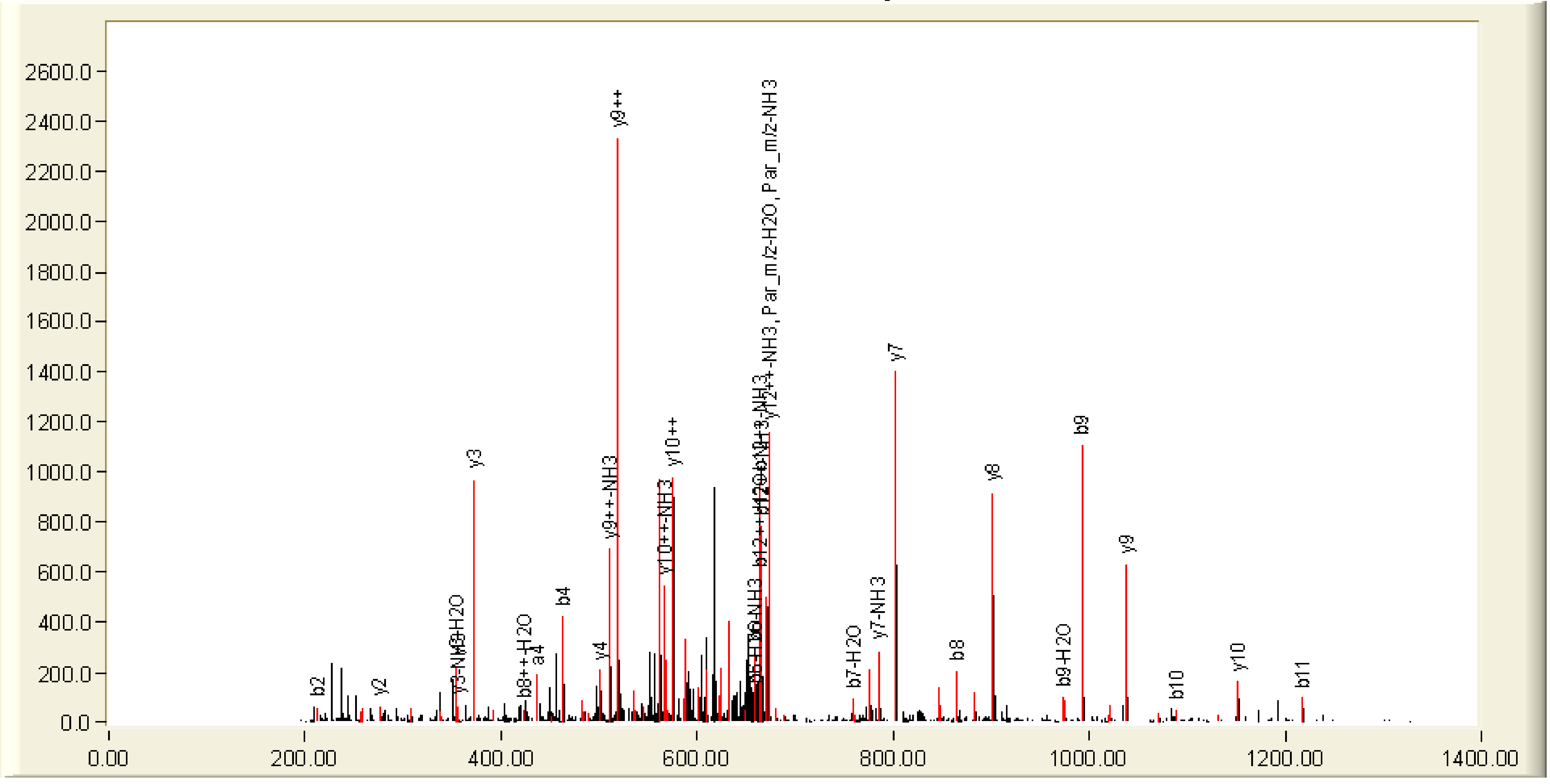

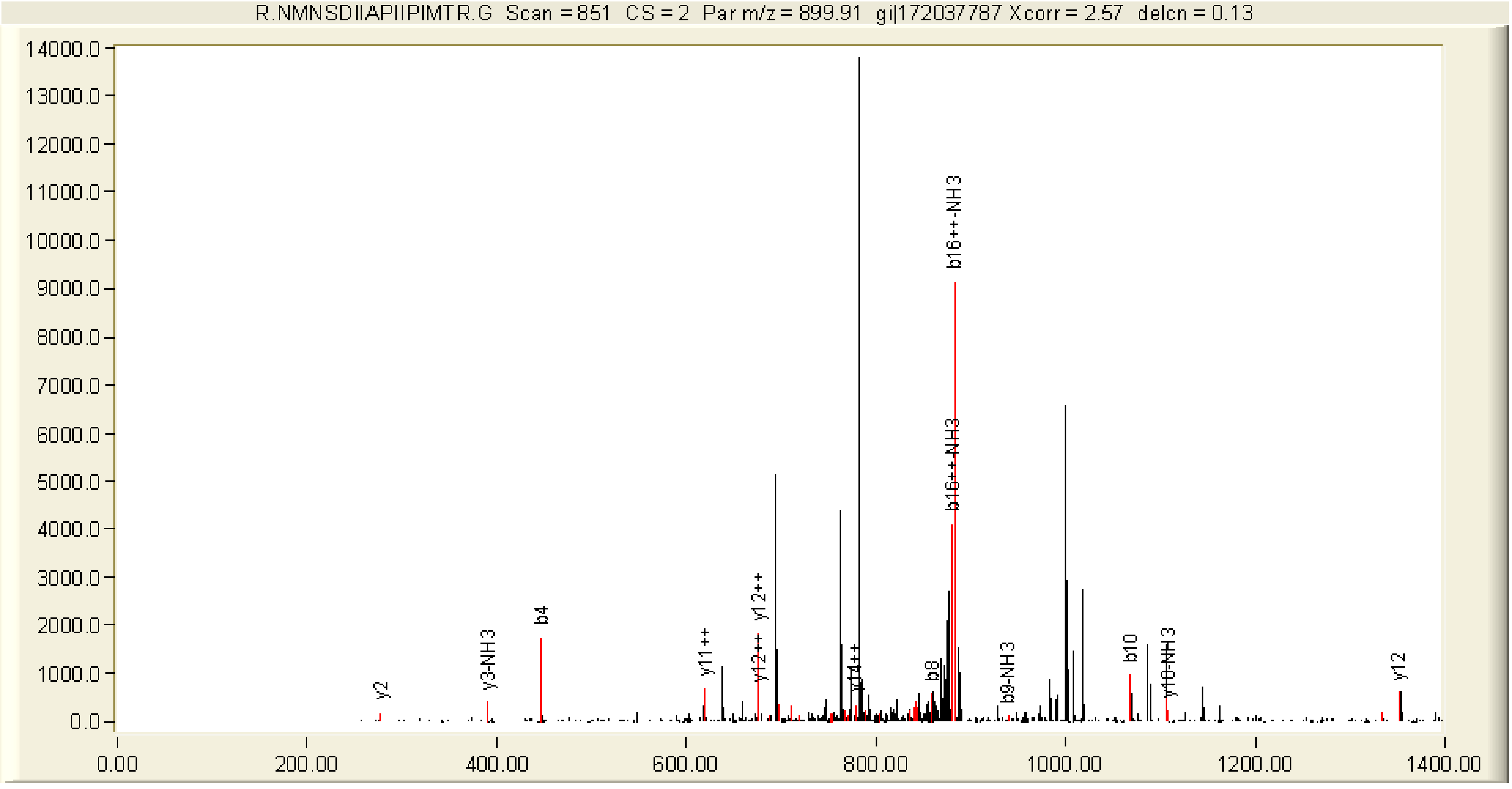

R.NMNSDIIAPIIPIMTR.G Scan = 851 CS = 2 Par m/z = 899.91 gi|172037787 Xcorr = 2.57 delcn = 0.13
14000.0
13000.0
12000.0
11000.0
10000.0
9000.0
8000.0
7000.0
6000.0
5000.0
4000.0
3000.0
2000.0
1000.0
0.0
0.00
200.00
400.00
600.00
800.00
1000.00
1200.00
1400.00
y2
y3-NH3
b4
y11++
y12++
y12++
b8
b16++-NH3
b16++-NH3
b9-NH3
b10
y10-NH3
y12

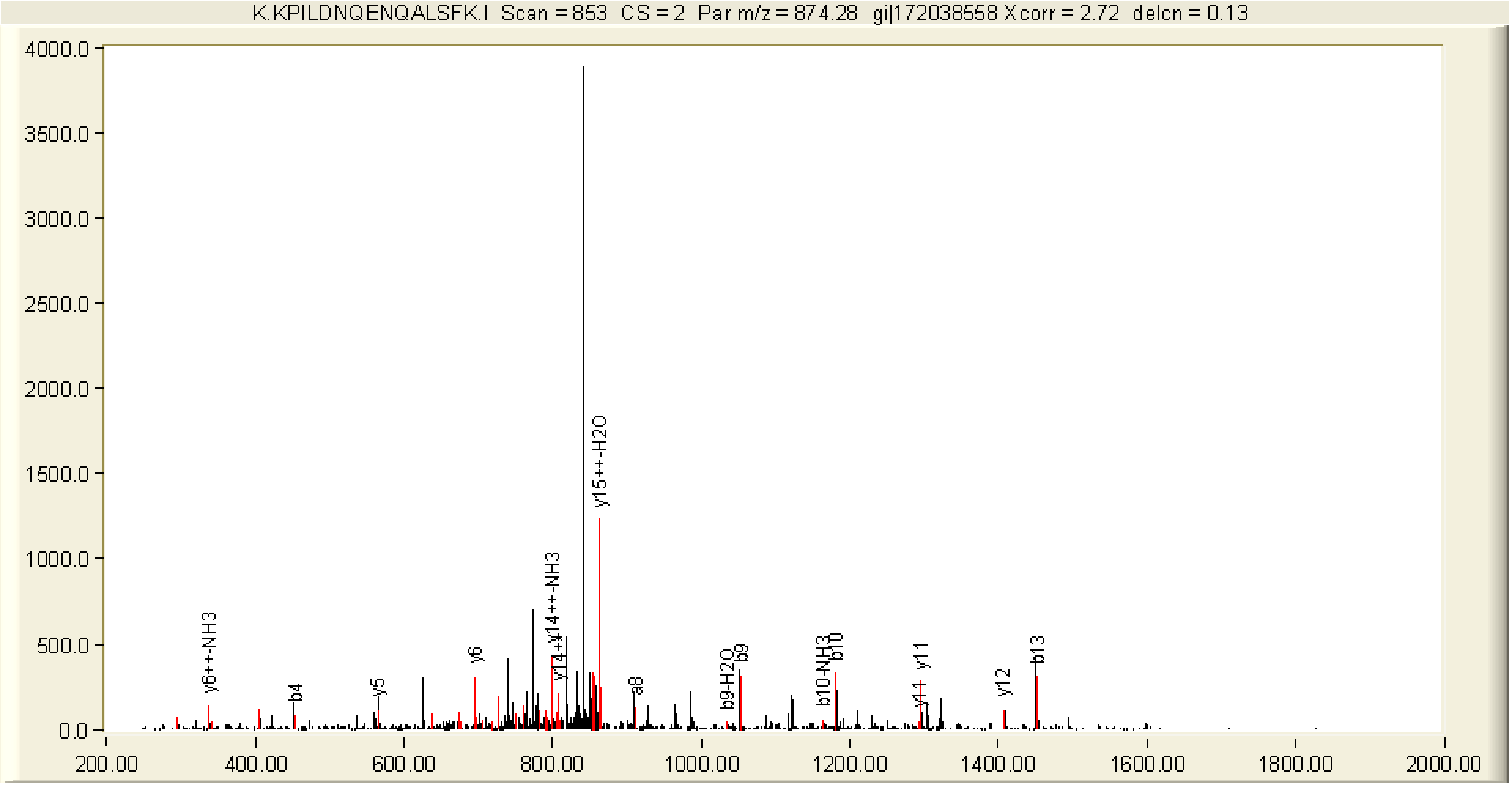

K.KPILDNQENQALSFK.I Scan = 853 CS = 2 Par m/z = 874.28 gi|172038558 Xcorr = 2.72 delcn = 0.13
4000.0
3500.0
3000.0
2500.0
2000.0
1500.0
1000.0
500.0
0.0
200.00
400.00
600.00
800.00
1000.00
1200.00
1400.00
1600.00
1800.00
2000.00
y6++-NH3
b4
y5
y6
y14++-NH3
y15++-H2O
a8
b9-H2O
b9
b10-NH3
b10
y11
y12
b13

K.IIMITKSDK.I Scan = 855 CS = 1 Par m/z = 1050.79 gi|172036767 Xcorr = 1.72 delcn = 0.13

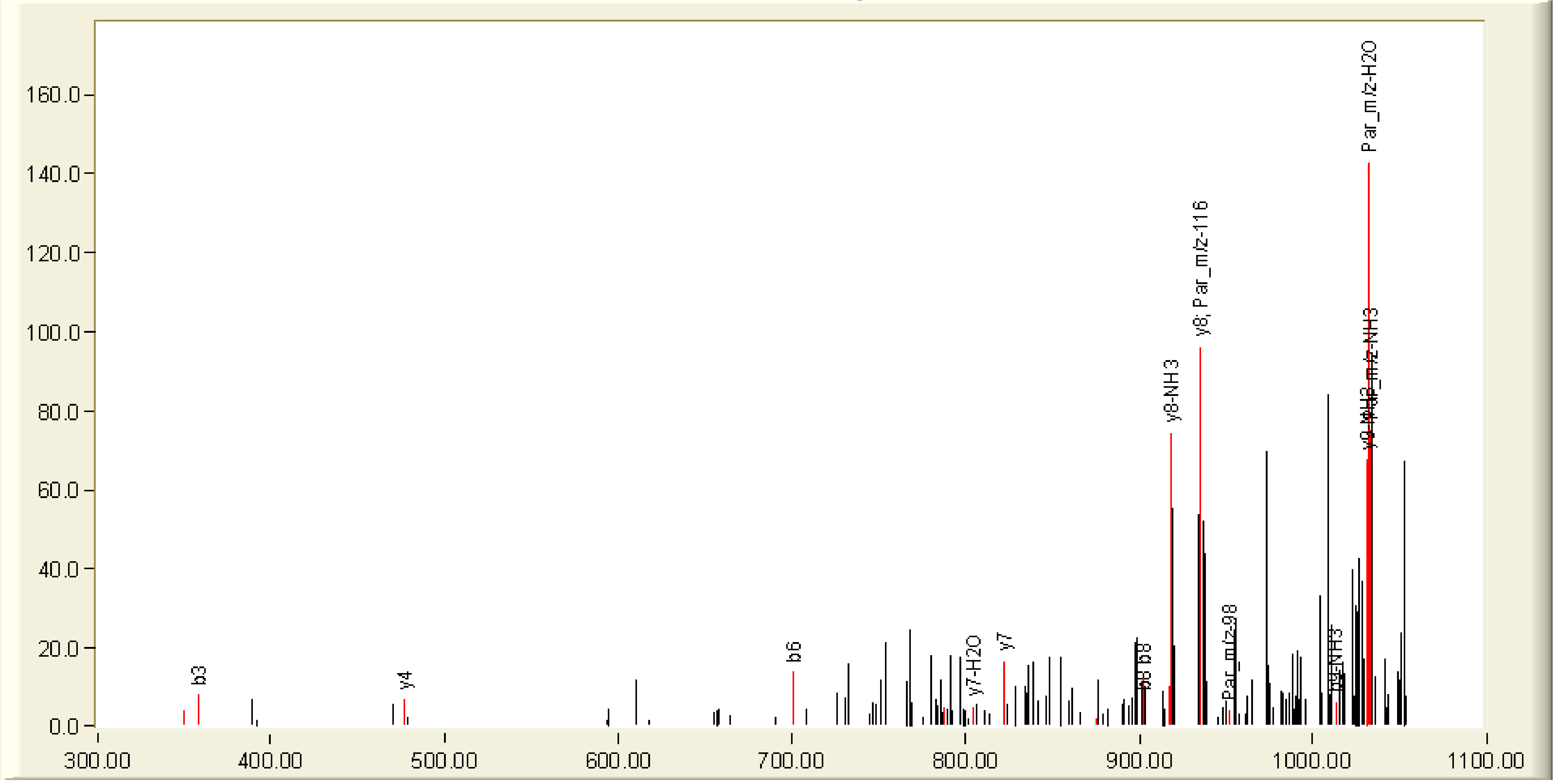

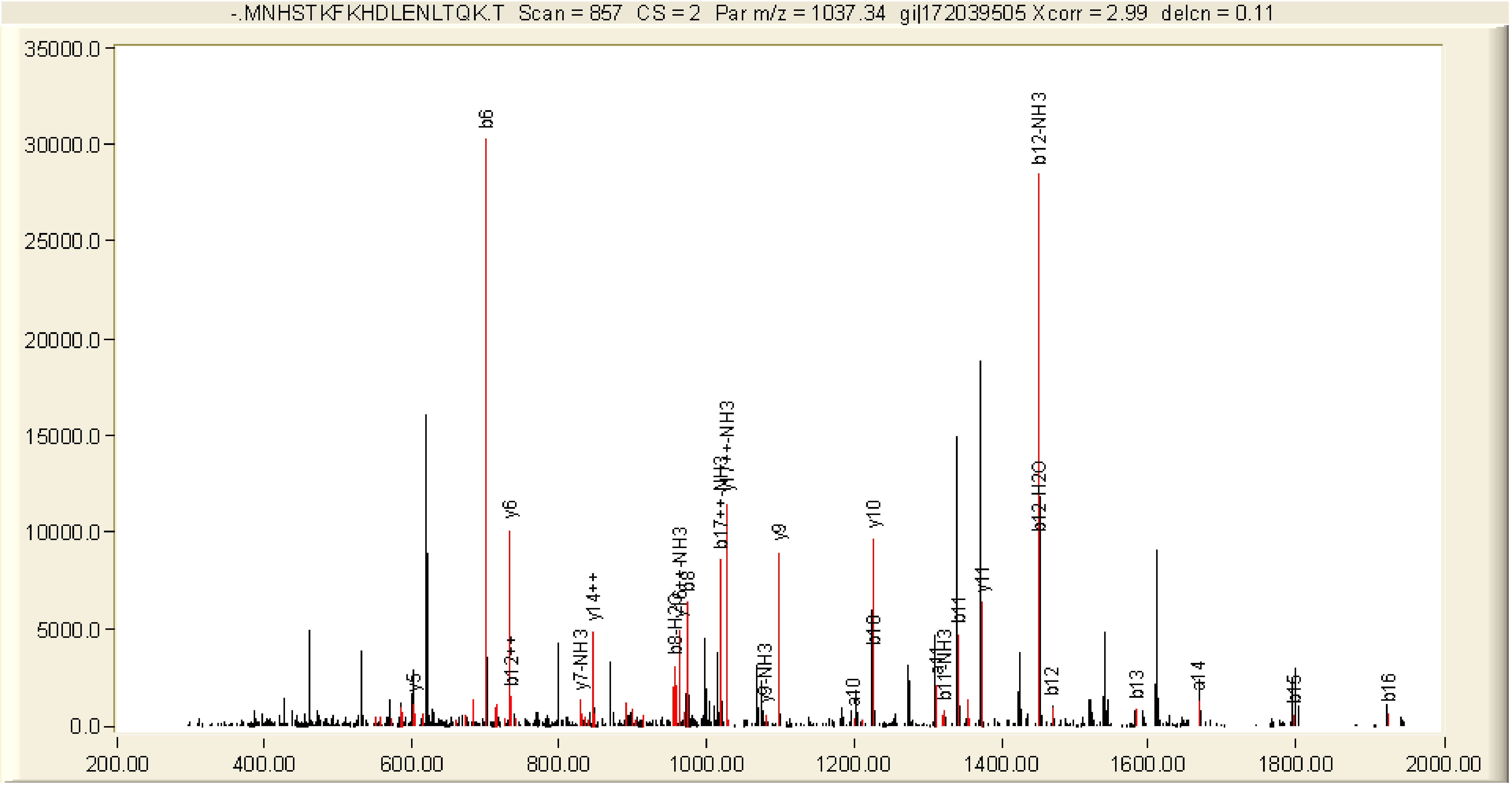

-.MNHSTKFKHDLENLTQK.T Scan = 857 CS = 2 Par m/z = 1037.34 gi|172039505 Xcorr = 2.99 delcn = 0.11
35000.0
30000.0
25000.0
20000.0
15000.0
10000.0
5000.0
0.0
200.00
400.00
600.00
800.00
1000.00
1200.00
1400.00
1600.00
1800.00
2000.00
b6
b12-NH3
y6
b12++
y5
y7-NH3
y14++
b8
y9
y10
b10
b11
y11
b12-H2O
b12
b13
a14
b16
a10

R.IVAVTFSRDGQRLVSGSIDETLQVWDWQNQR.L Scan = 859 CS = 3 Par m/z = 1201.71 gi|172037089 Xcorr = 3.25 delcn = 0.21

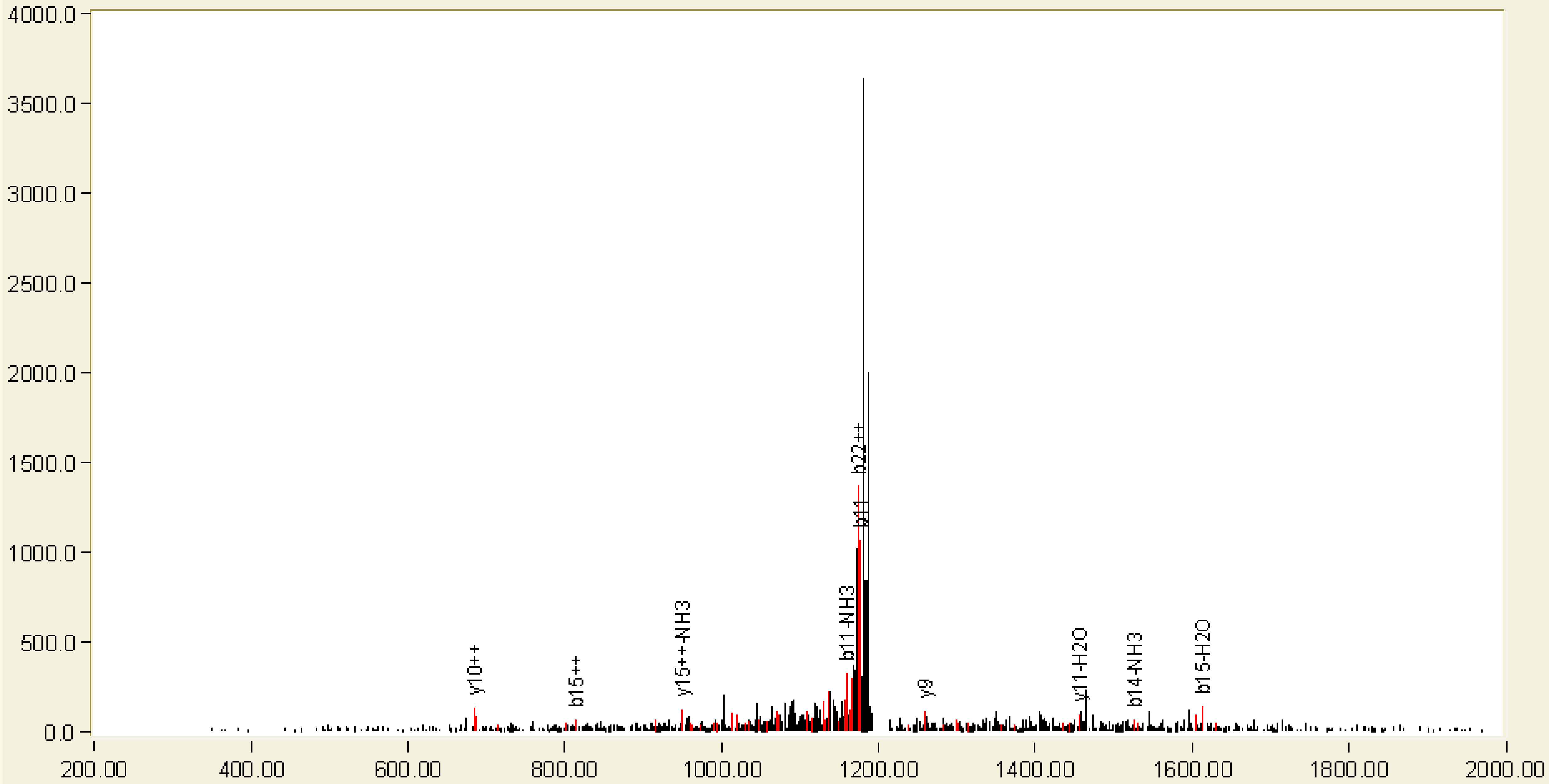

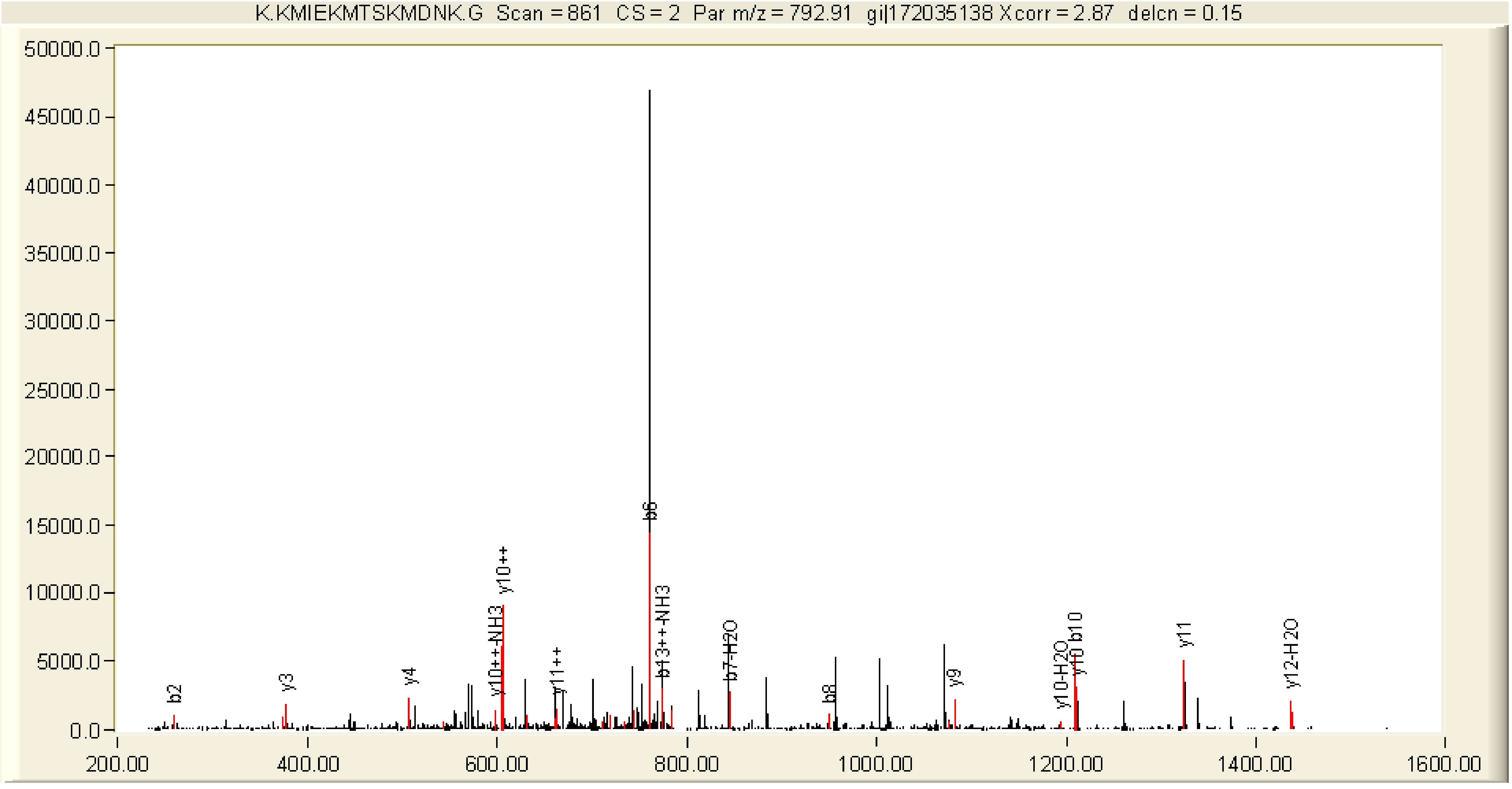

K.KMIEKMTSKMDNK.G Scan = 861 CS = 2 Par m/z = 792.91 gi|172035138 Xcorr = 2.87 delcn = 0.15
50000.0
45000.0
40000.0
35000.0
30000.0
25000.0
20000.0
15000.0
10000.0
5000.0
0.0
200.00
400.00
600.00
800.00
1000.00
1200.00
1400.00
1600.00
b2
y3
y4
y10++-NH3
y10++
y11++
b6
b13++-NH3
b7-H2O
b8
y9
y10-H2O
y10
b10
y11
y12-H2O

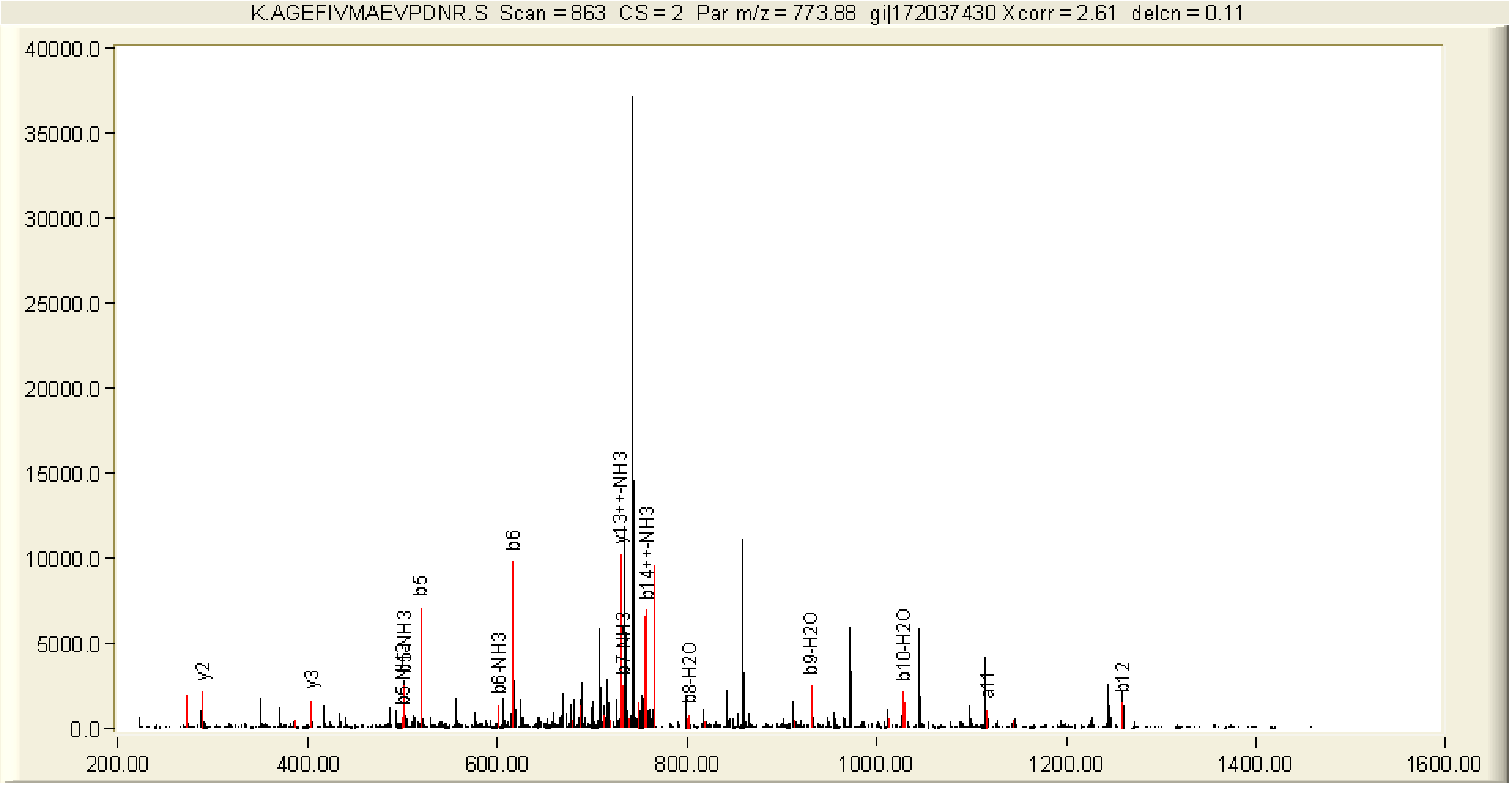

K.AGEFIVMAEVPDNR.S Scan = 863 CS = 2 Par m/z = 773.88 gi|172037430 Xcorr = 2.61 delcn = 0.11
40000.0
35000.0
30000.0
25000.0
20000.0
15000.0
10000.0
5000.0
0.0
200.00
400.00
600.00
800.00
1000.00
1200.00
1400.00
1600.00
y2
y3
b5
b6-NH3
b6
y13++-NH3
b14++-NH3
b8-H2O
b9-H2O
b10-H2O
a11
b12

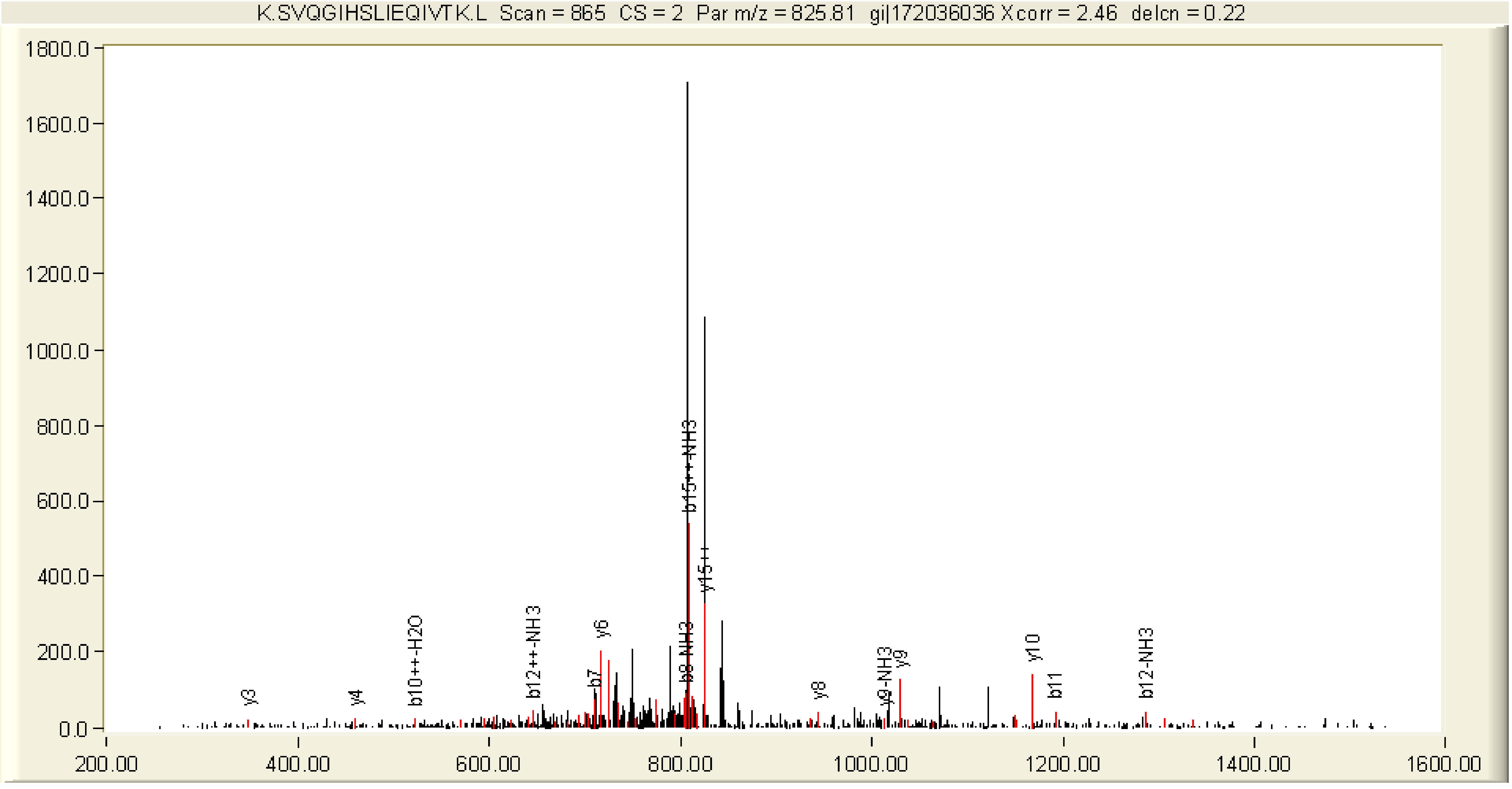
K.SVQGIHSLIEQIVTK.L Scan = 865 CS = 2 Par m/z = 825.81 gi|172036036 Xcorr = 2.46 delcn = 0.22
1800.0
1600.0
1400.0
1200.0
1000.0
800.0
600.0
400.0
200.0
0.0
200.00
400.00
600.00
800.00
1000.00
1200.00
1400.00
1600.00
y3
y4
b10++-H2O
b12++-NH3
b7
y6
b8-NH3
b15++-NH3
y15++
y8
y9-NH3
y9
y10
b11
b12-NH3

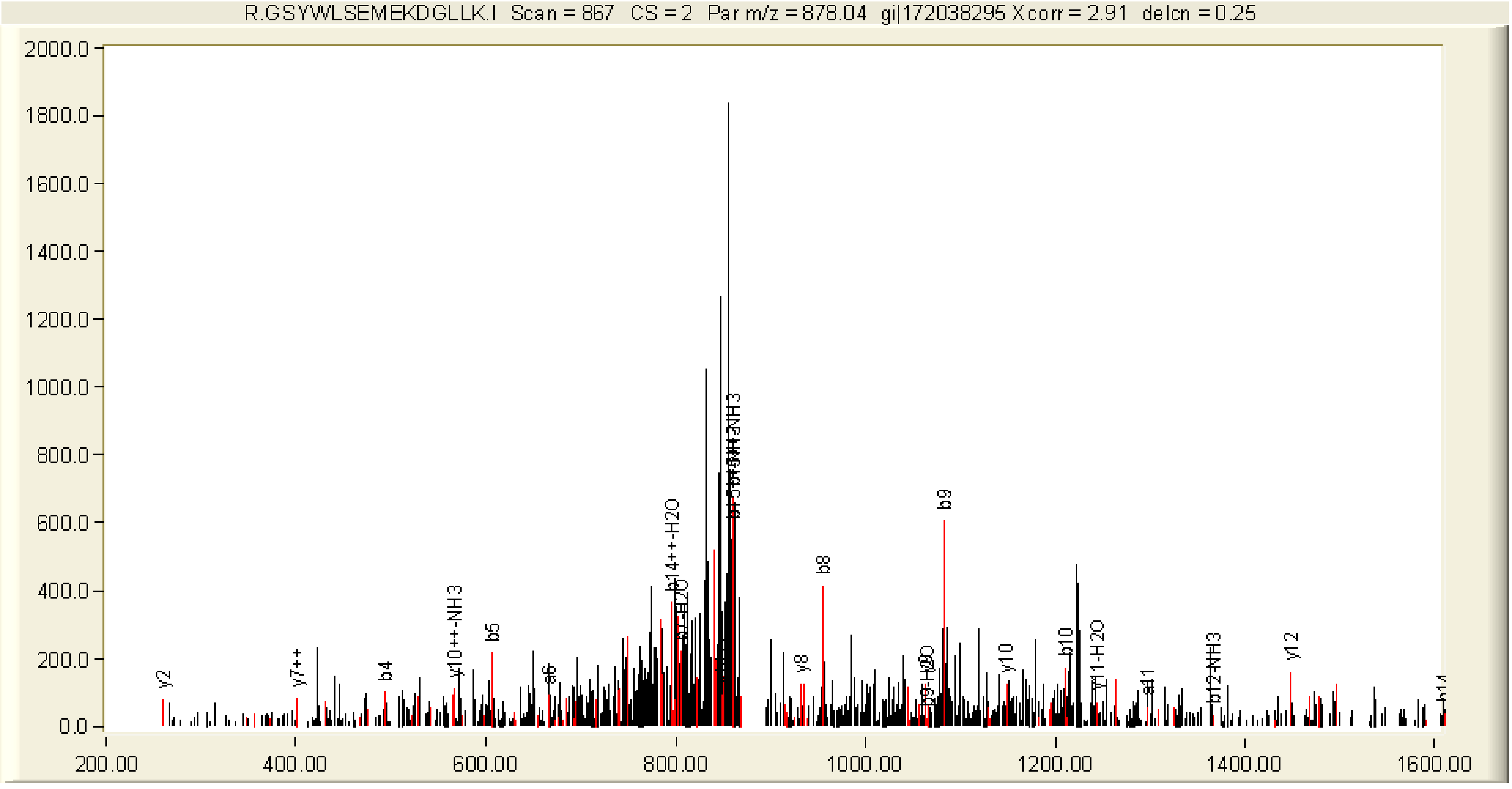
R.GSYWLSEMEKDGLLK.I Scan = 867 CS = 2 Par m/z = 878.04 gi|172038295 Xcorr = 2.91 delcn = 0.25
2000.0
1800.0
1600.0
1400.0
1200.0
1000.0
800.0
600.0
400.0
200.0
0.0
200.00
400.00
600.00
800.00
1000.00
1200.00
1400.00
1600.00
y2
y7++
b4
y10++-NH3
b5
b14++-H2O
b8
b9
y8
y10
b10
y11-H2O
y12
b12-NH3

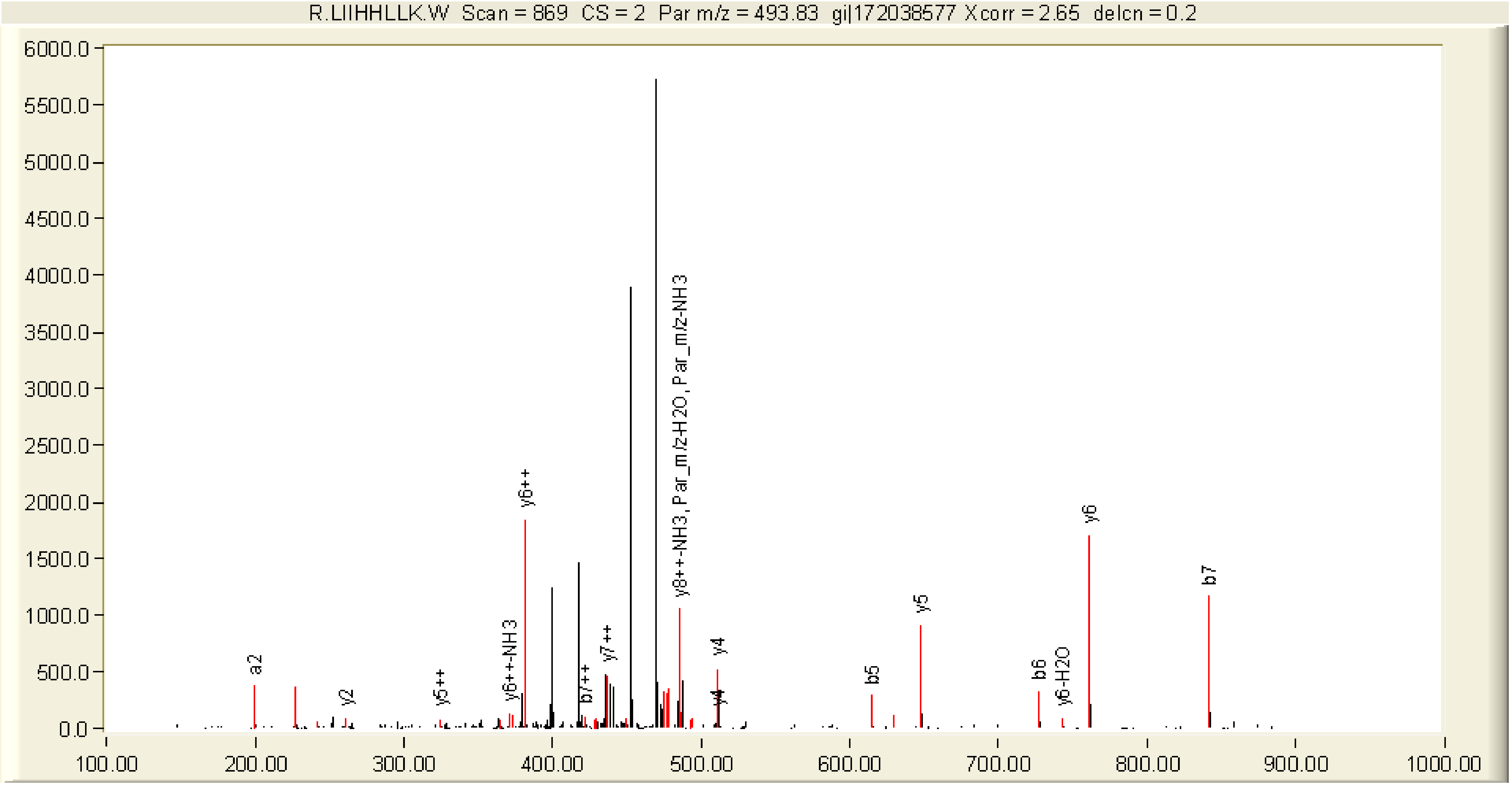

R.LIIHHLLK.W Scan = 869 CS = 2 Par m/z = 493.83 gi|172038577 Xcorr = 2.65 delcn = 0.2
a2
y2
y5++
y6++-NH3
y6++
b7++
y7++
y8++-NH3, Par_m/z-H2O, Par_m/z-NH3
y4
b5
y5
b6
y6-H2O
y6
b7

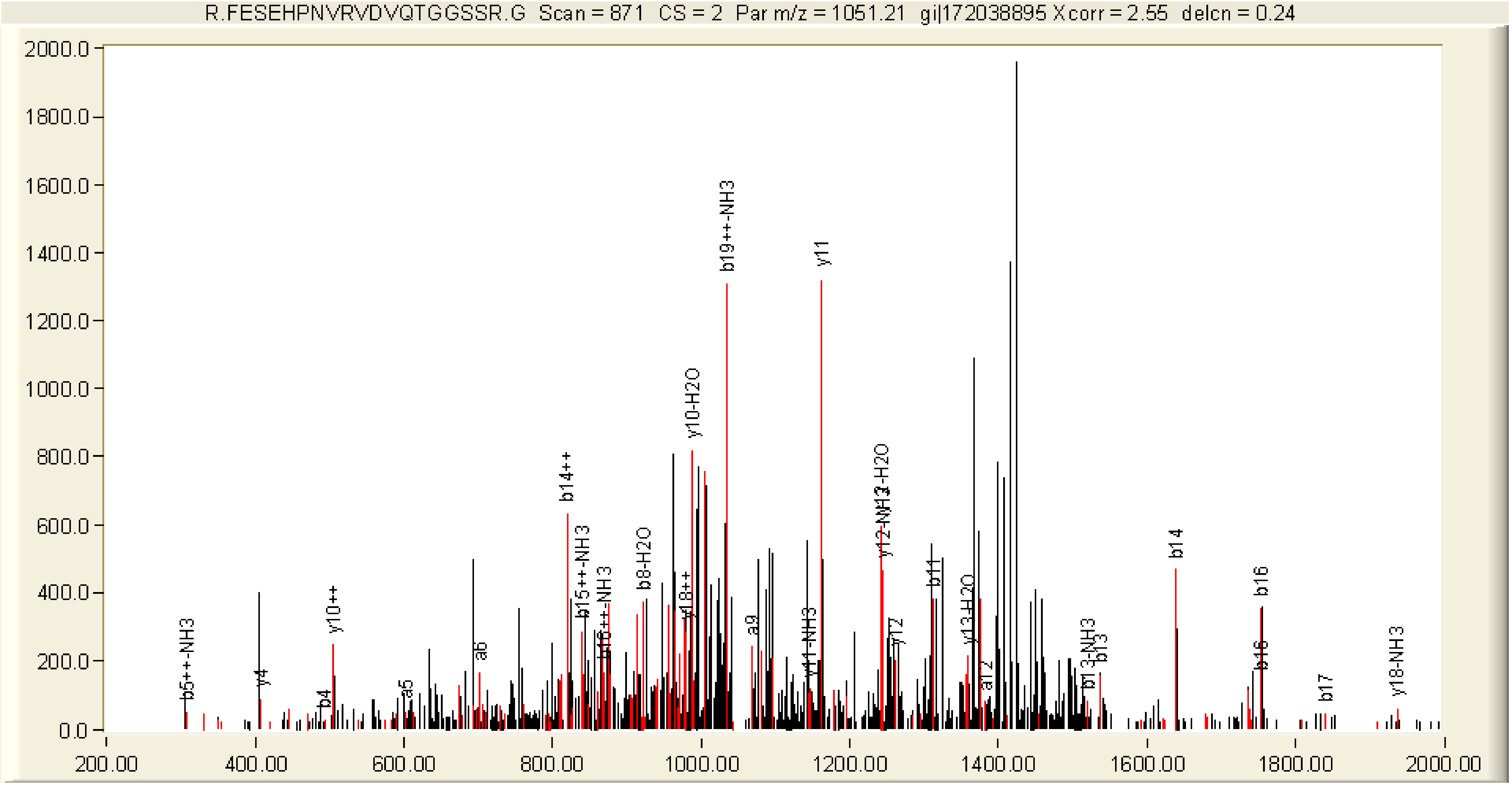

R.FESEHPNVRVDVQTGGSSR.G Scan = 871 CS = 2 Par m/z = 1051.21 gi|172038895 Xcorr = 2.55 delcn = 0.24
2000.0
1800.0
1600.0
1400.0
1200.0
1000.0
800.0
600.0
400.0
200.0
0.0
200.00
400.00
600.00
800.00
1000.00
1200.00
1400.00
1600.00
1800.00
2000.00
b5++-NH3
y4
b4
y10++
a5
a6
b14++
b15++-NH3
b8-H2O
y18++
y10-H2O
b19++-NH3
a9
y11-NH3
y11
y12
b11
y13-H2O
a12
b13
b14
b16
b17
y18-NH3

R.FELIYQNSLKYTK.R Scan = 873 CS = 2 Par m/z = 824.93 gi|172037833 Xcorr = 2.47 delcn = 0.25

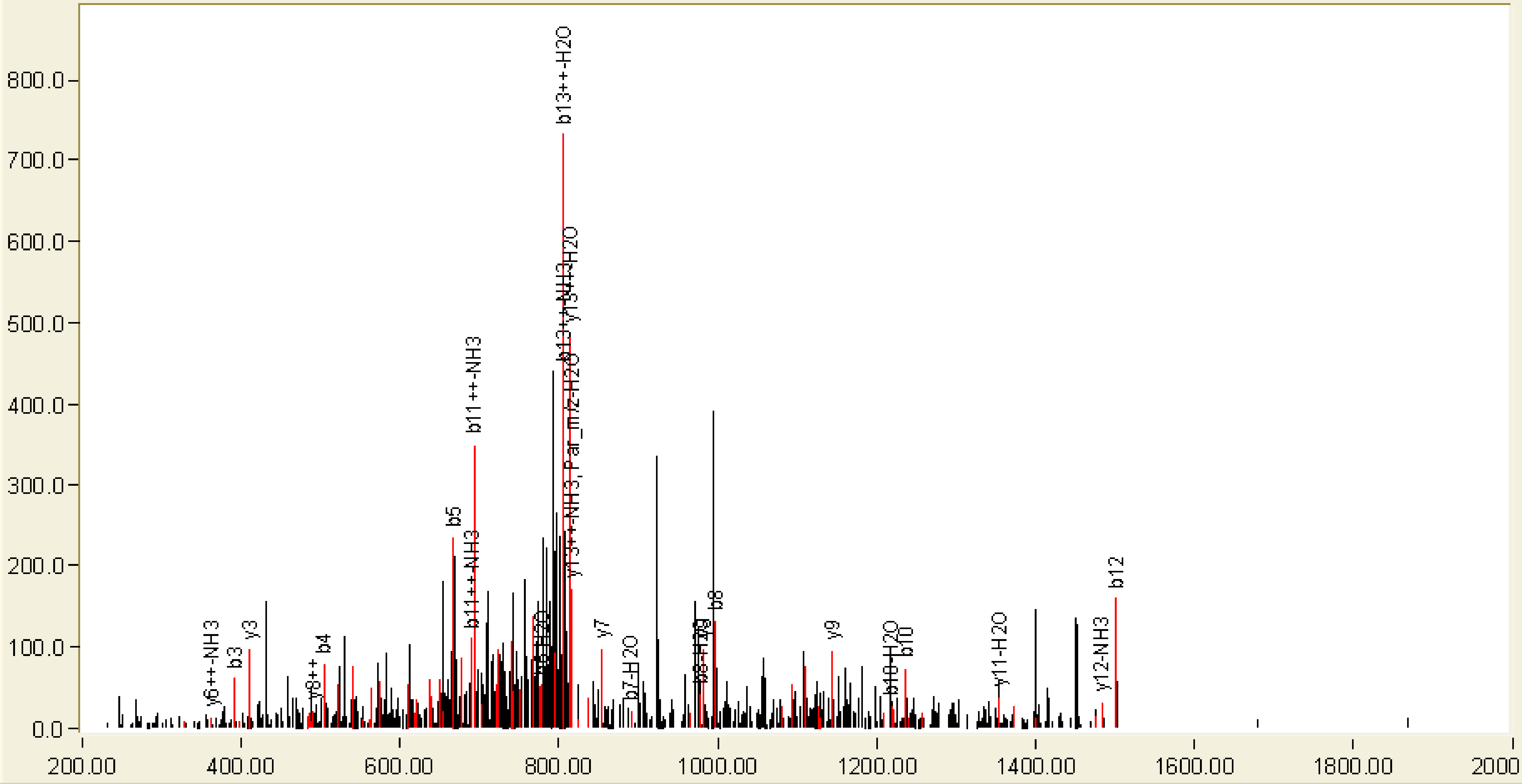

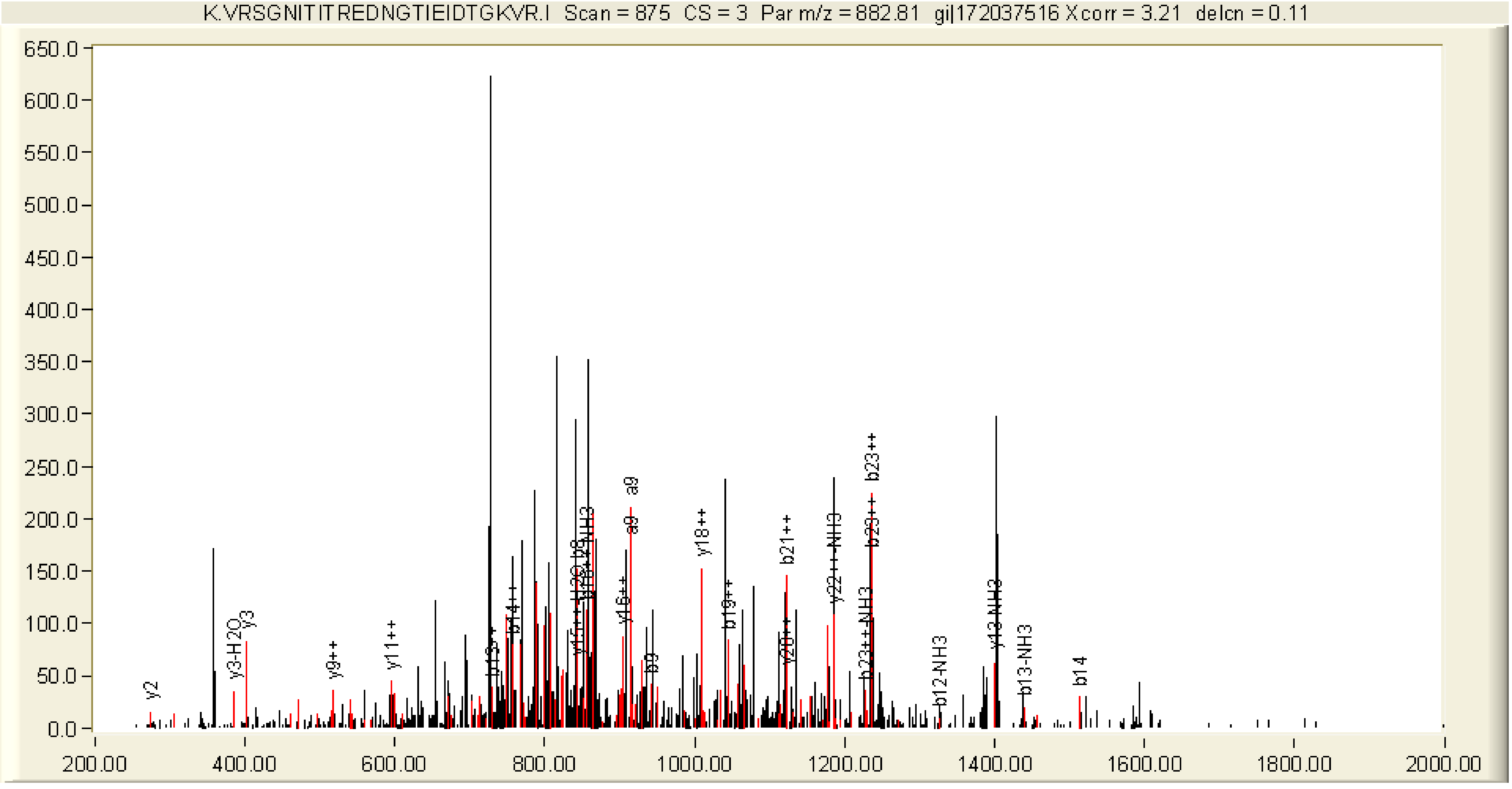
K.VRSGNITITREDNGTIEIDTGKVR.I Scan = 875 CS = 3 Par m/z = 882.81 gi|172037516 Xcorr = 3.21 delcn = 0.11
650.0
600.0
550.0
500.0
450.0
400.0
350.0
300.0
250.0
200.0
150.0
100.0
50.0
0.0
200.00
400.00
600.00
800.00
1000.00
1200.00
1400.00
1600.00
1800.00
2000.00
y2
y3-H2O
y3
y9++
y11++
b14++
y16++
a9
a9
b9
y18++
b19++
b21++
y20++
y22++-NH3
b23++-NH3
b23++
b23++
b12-NH3
y13-NH3
b13-NH3
b14

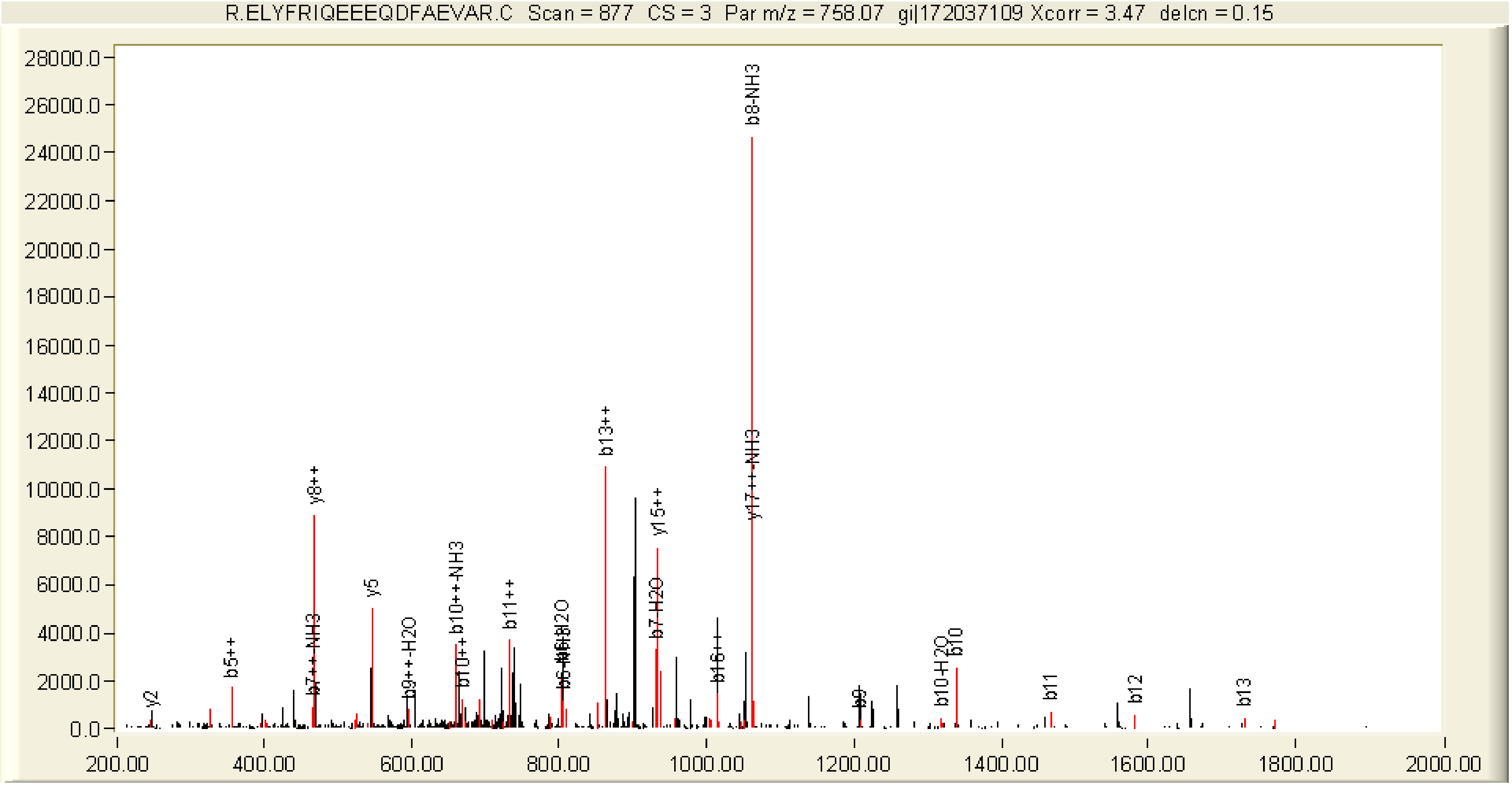
R.ELYFRIQEEEQDFAEVAR.C Scan = 877 CS = 3 Par m/z = 758.07 gi|172037109 Xcorr = 3.47 delcn = 0.15
28000.0
26000.0
24000.0
22000.0
20000.0
18000.0
16000.0
14000.0
12000.0
10000.0
8000.0
6000.0
4000.0
2000.0
0.0
200.00
400.00
600.00
800.00
1000.00
1200.00
1400.00
1600.00
1800.00
2000.00
b8-NH3
b13++
y17++-NH3
y8++
y15++
y5
b10++-NH3
b11++
b7-H2O
b7++-NH3
b9++-H2O
b5++
b10++
b16++
b10-H2O
b10
b11
b12
b13
y2

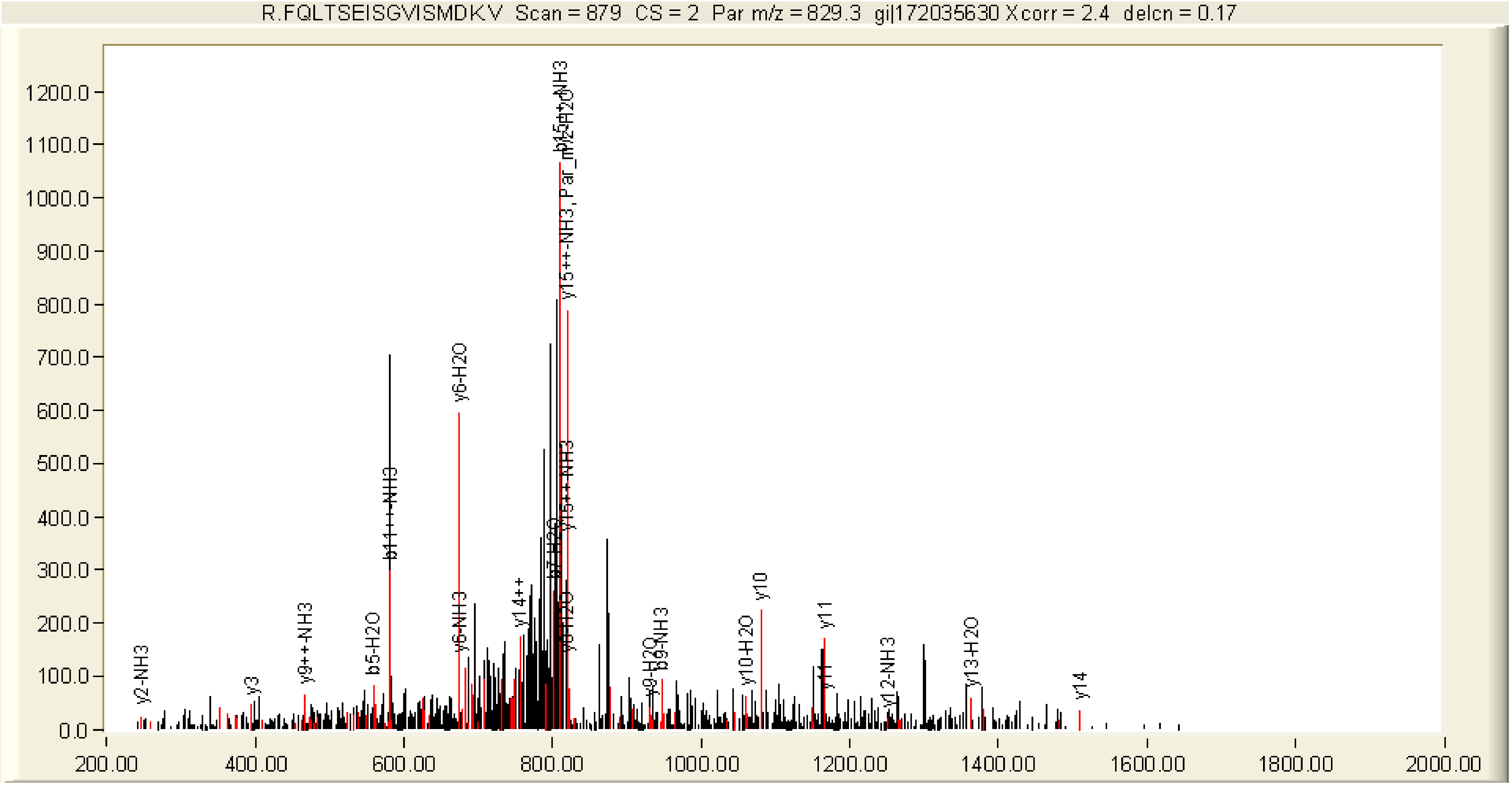
R.FQLTSEISGVISMDKV Scan = 879 CS = 2 Par m/z = 829.3 gi|172035630 Xcorr = 2.4 delcn = 0.17
y2-NH3
y3
y9++-NH3
b5-H2O
b14++-NH3
y6-NH3
y6-H2O
y14++
y15++-NH3
y9-NH3
b9-NH3
y10-H2O
y10
y11
y12-NH3
y13-H2O
y14
0.0
100.0
200.0
300.0
400.0
500.0
600.0
700.0
800.0
900.0
1000.0
1100.0
1200.0
200.00
400.00
600.00
800.00
1000.00
1200.00
1400.00
1600.00
1800.00
2000.00

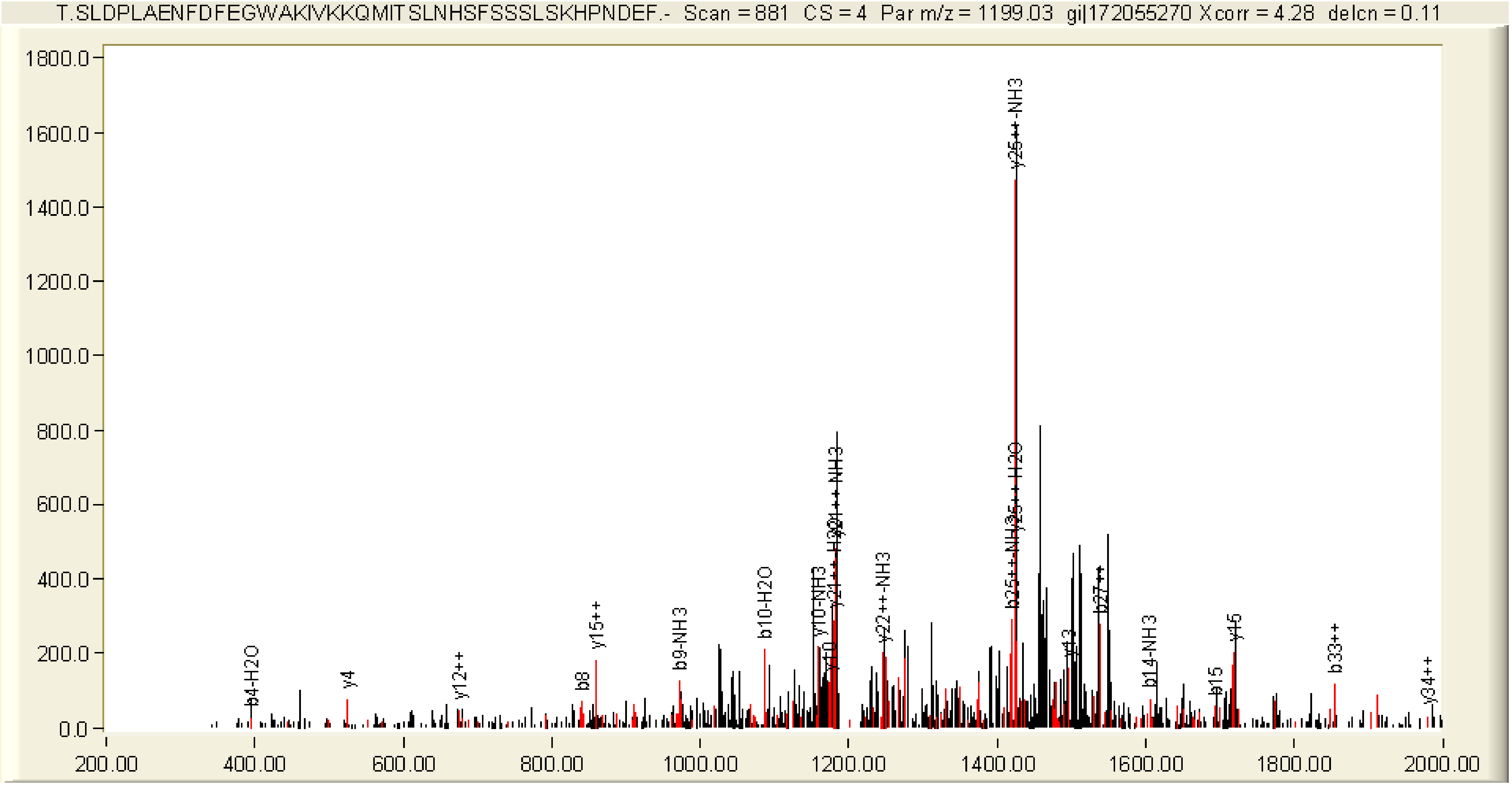
T.SLDPLAENFDFEGWAKIVKKQMITSLNHSFSSSLSKHPNDEF.- Scan = 881 CS = 4 Par m/z = 1199.03 gi|172055270 Xcorr = 4.28 delcn = 0.11
1800.0
1600.0
1400.0
1200.0
1000.0
800.0
600.0
400.0
200.0
0.0
200.00
400.00
600.00
800.00
1000.00
1200.00
1400.00
1600.00
1800.00
2000.00
b4-H2O
y4
y12++
b8
y15++
b9-NH3
b10-H2O
y10-NH3
y21++-NH3
y22++-NH3
b25++-NH3
y25++-H2O
y25++-NH3
b27++
b14-NH3
b15
y15
b33++
y34++

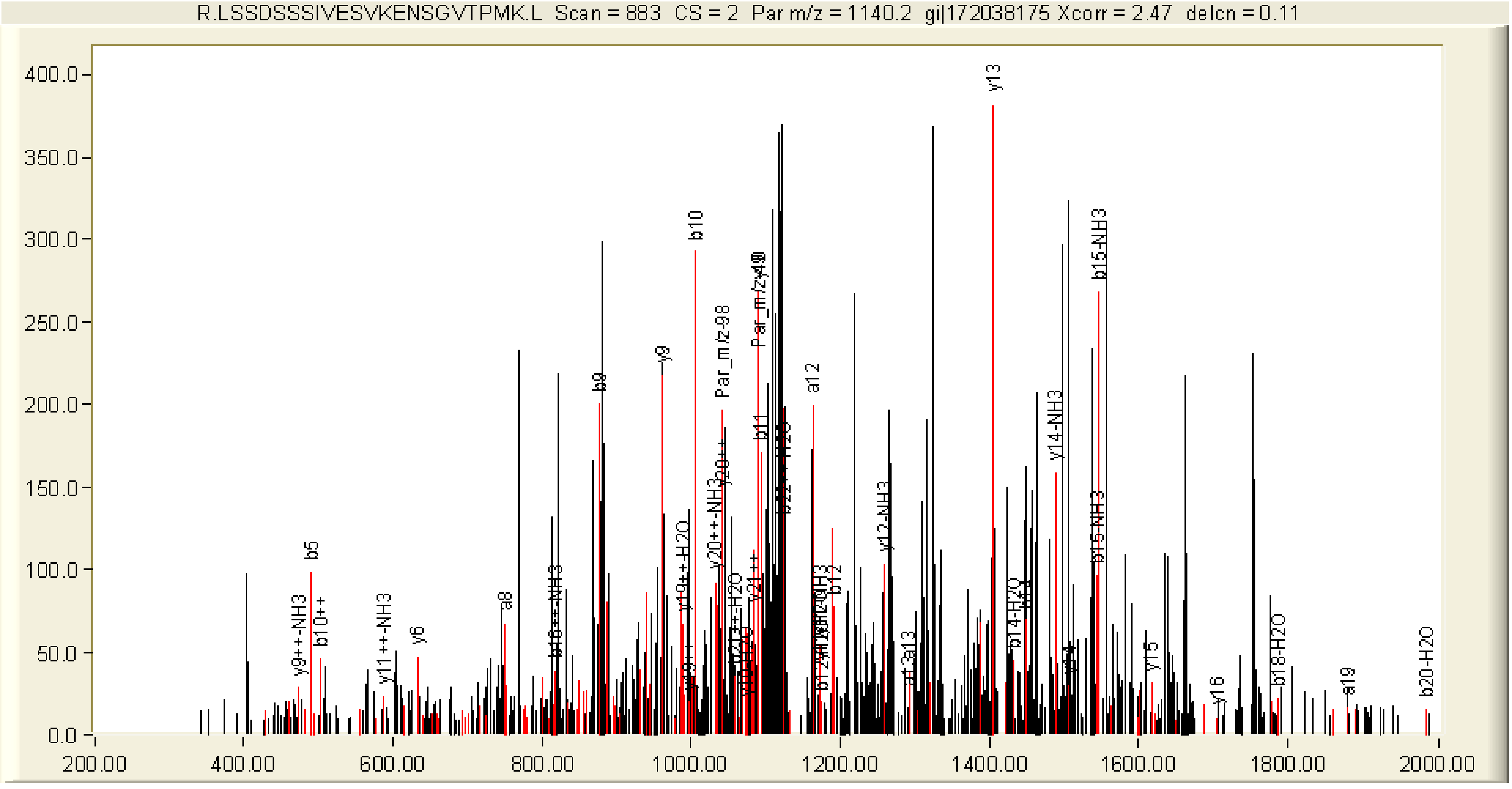
R.LSSDSSSIVESVKENSGVTPMK.L Scan = 883 CS = 2 Par m/z = 1140.2 gi|172038175 Xcorr = 2.47 delcn = 0.11
400.0
350.0
300.0
250.0
200.0
150.0
100.0
50.0
0.0
200.00
400.00
600.00
800.00
1000.00
1200.00
1400.00
1600.00
1800.00
2000.00
y13
b10
b15-NH3
Par_m/z-98
y9
a12
y14-NH3
y12-NH3
b5
y9++-NH3
b10++
y11++-NH3
y6
a8
y20++-NH3
b11
b12
b14-H2O
y15
y16
b18-H2O
a19
b20-H2O

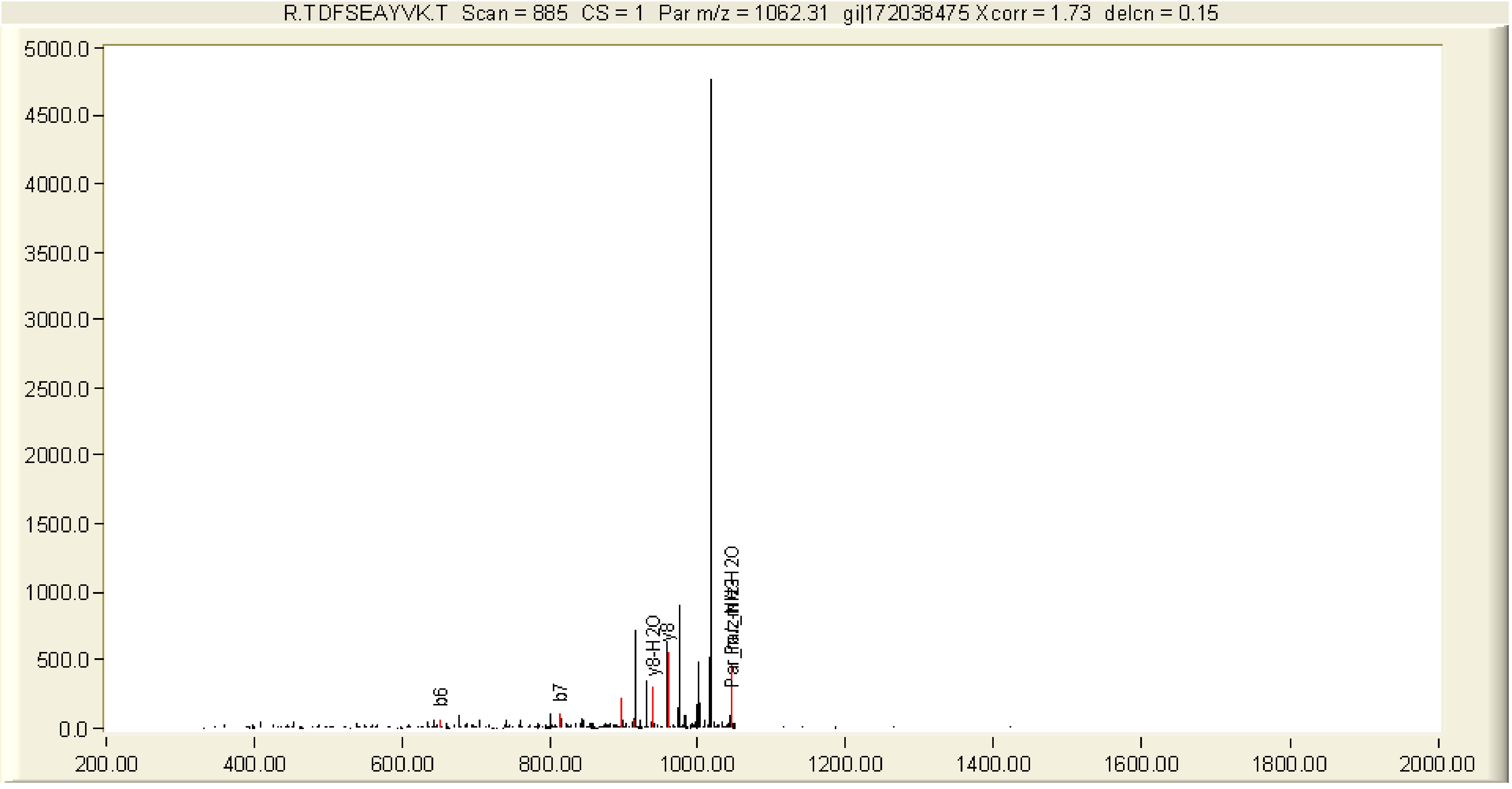
R.TDFSEAYVK.T Scan = 885 CS = 1 Par m/z = 1062.31 gi|172038475 Xcorr = 1.73 delcn = 0.15
5000.0
4500.0
4000.0
3500.0
3000.0
2500.0
2000.0
1500.0
1000.0
500.0
0.0
200.00
400.00
600.00
800.00
1000.00
1200.00
1400.00
1600.00
1800.00
2000.00
b6
b7
y8-H2O
y8

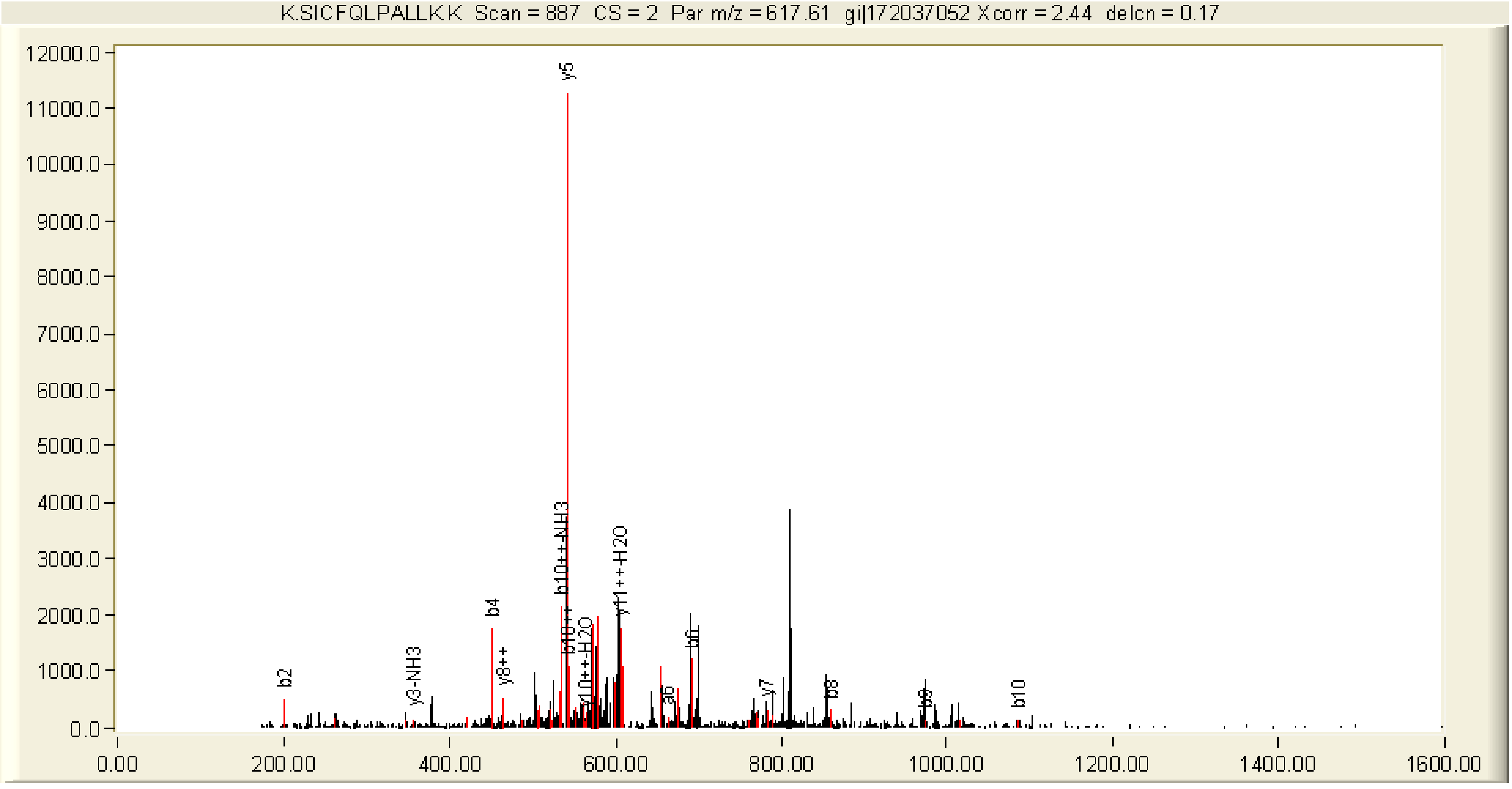

K.SICFQLPALLK.K Scan = 887 CS = 2 Par m/z = 617.61 gi|172037052 Xcorr = 2.44 delcn = 0.17
12000.0
11000.0
10000.0
9000.0
8000.0
7000.0
6000.0
5000.0
4000.0
3000.0
2000.0
1000.0
0.0
0.00
200.00
400.00
600.00
800.00
1000.00
1200.00
1400.00
1600.00
b2
y3-NH3
b4
y8++
b10++-NH3
y5
b10++
y11++-H2O
b6
y7
b8
b9
b10

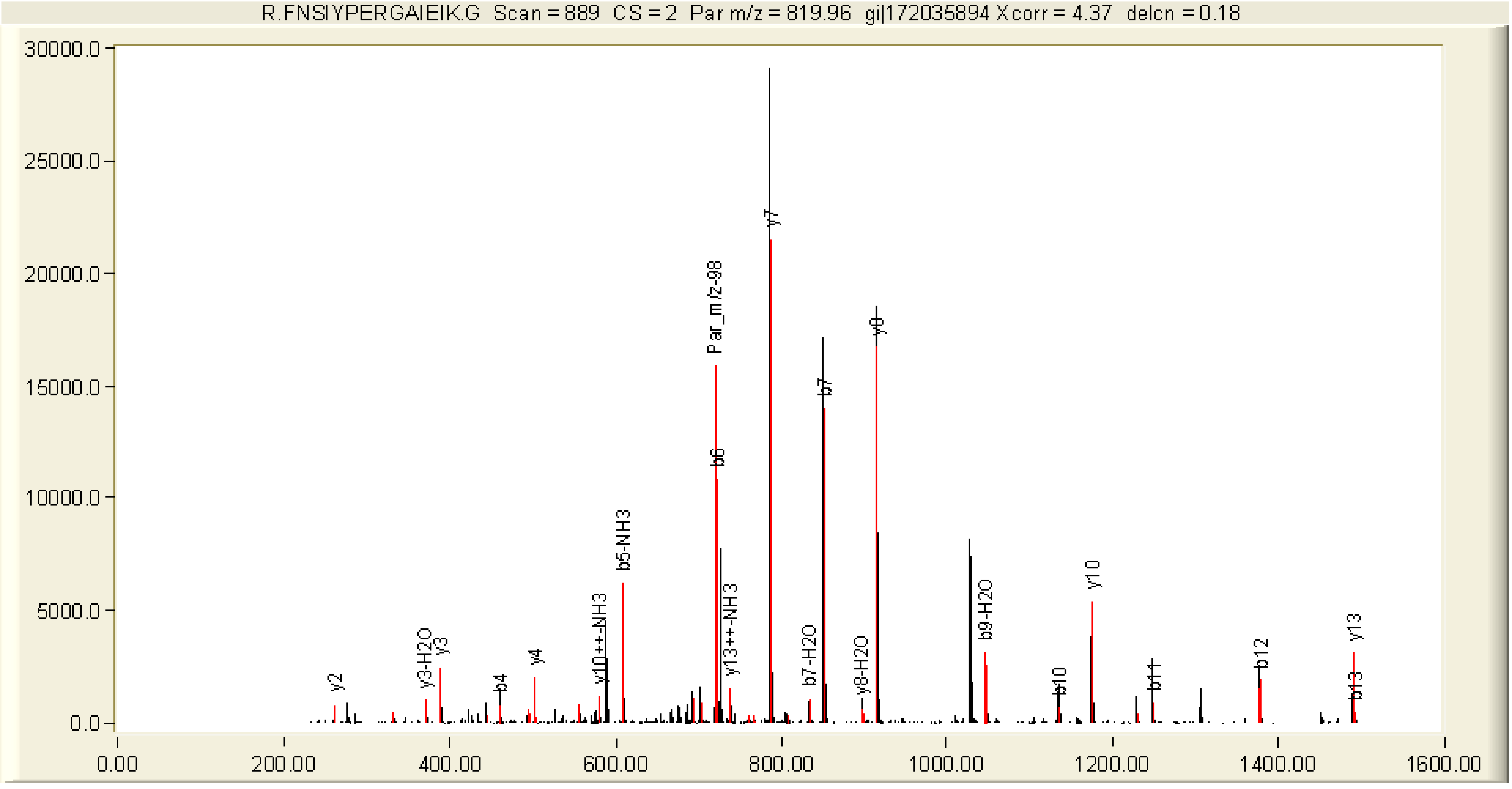

R.FNSIYPERGAIEIK.G Scan = 889 CS = 2 Par m/z = 819.96 gi|172035894 Xcorr = 4.37 delcn = 0.18
30000.0
25000.0
20000.0
15000.0
10000.0
5000.0
0.0
0.00
200.00
400.00
600.00
800.00
1000.00
1200.00
1400.00
1600.00
y2
y3-H2O
y3
b4
y4
y10++-NH3
b5-NH3
Par_m/z-98
b6
y13++-NH3
y7
b7-H2O
b7
y8-H2O
y8
b9-H2O
b10
y10
b11
b12
b13
y13

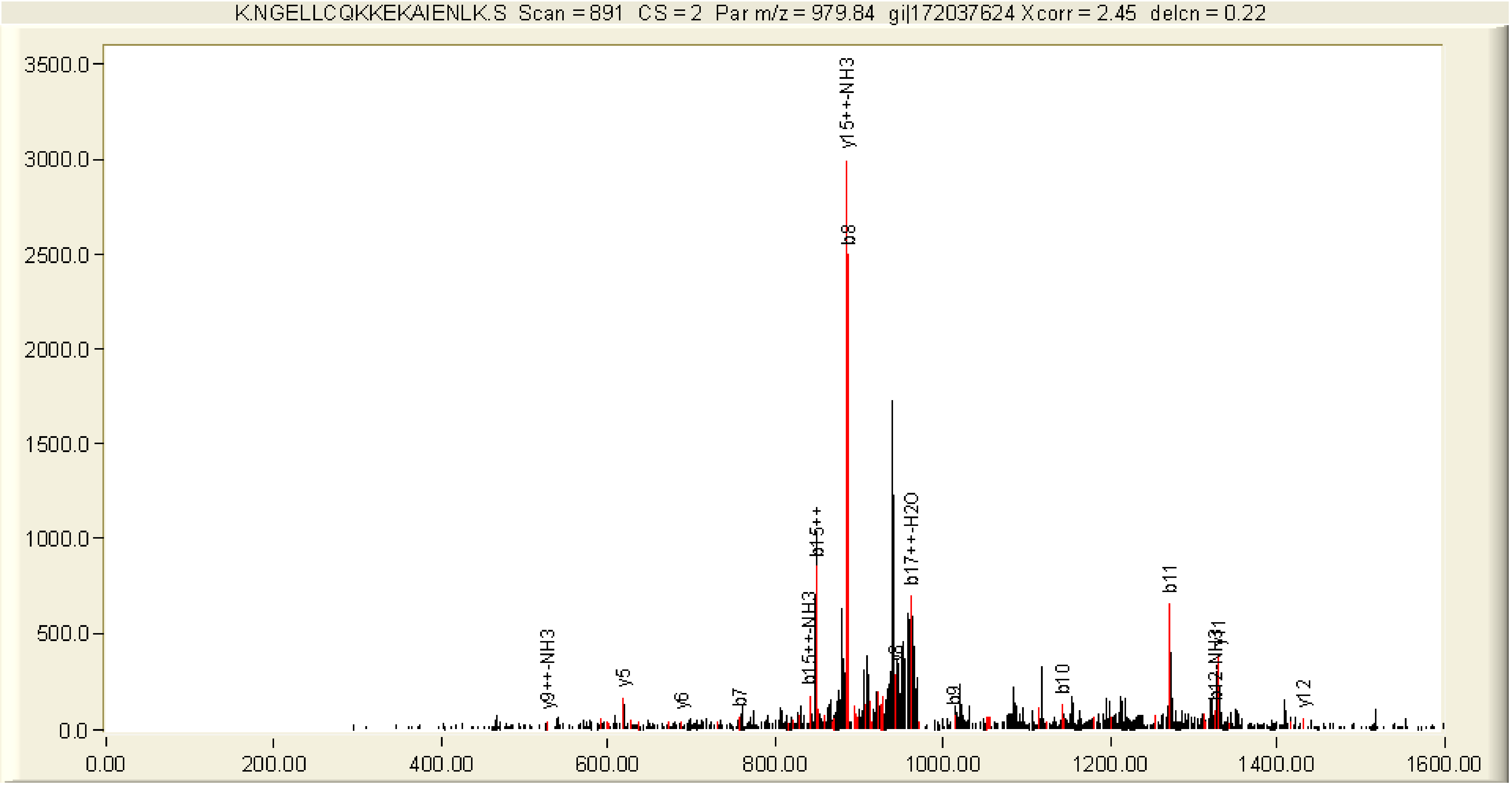

K.NGELLCQKKEKAIENLK.S Scan = 891 CS = 2 Par m/z = 979.84 gi|172037624 Xcorr = 2.45 delcn = 0.22
3500.0
3000.0
2500.0
2000.0
1500.0
1000.0
500.0
0.0
0.00
200.00
400.00
600.00
800.00
1000.00
1200.00
1400.00
1600.00
y15++-NH3
b8
b15++
b15++-NH3
b17++-H2O
b11
y9++-NH3
y5
y6
b7
b9
b10
y12

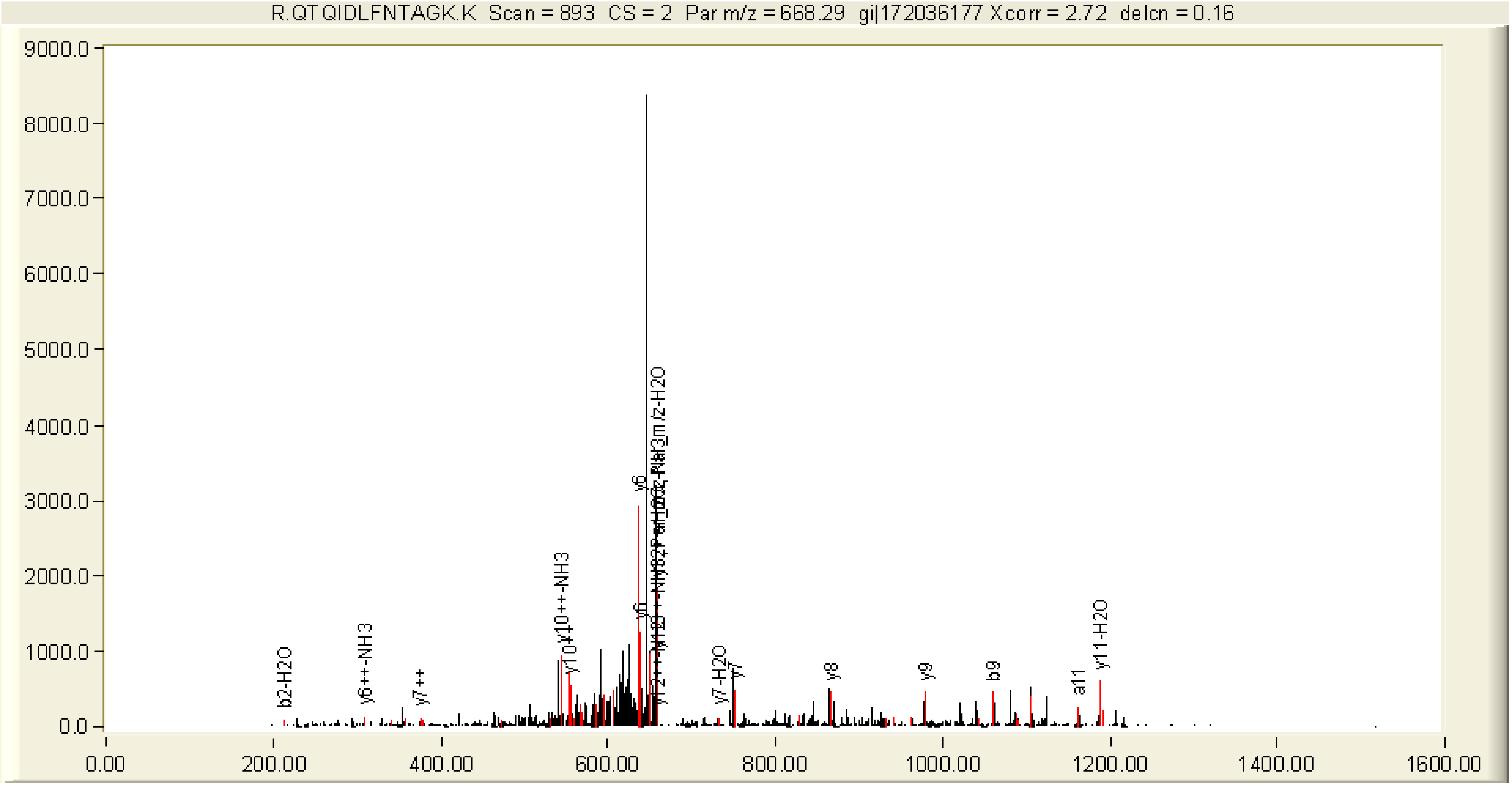

R.QTQIDLFNTAGK.K Scan = 893 CS = 2 Par m/z = 668.29 gi|172036177 Xcorr = 2.72 delcn = 0.16
9000.0
8000.0
7000.0
6000.0
5000.0
4000.0
3000.0
2000.0
1000.0
0.0
0.00
200.00
400.00
600.00
800.00
1000.00
1200.00
1400.00
1600.00
b2-H2O
y6++-NH3
y7++
y10++-NH3
y10++
y6
y7-H2O
y7
y8
y9
b9
a11
y11-H2O

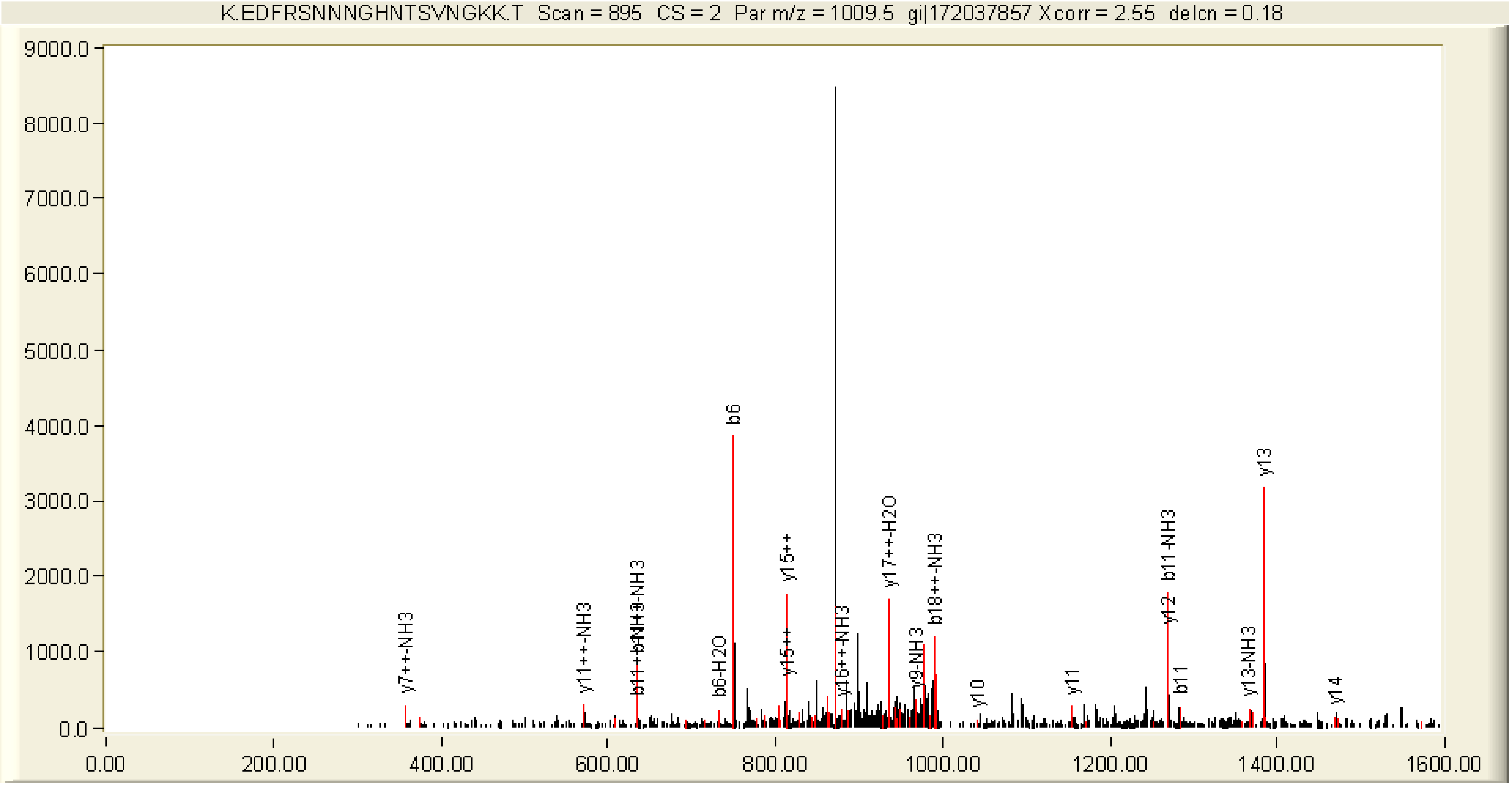

K.EDFRSNNNGHNTSVNGKK.T Scan = 895 CS = 2 Par m/z = 1009.5 gi|172037857 Xcorr = 2.55 delcn = 0.18
9000.0
8000.0
7000.0
6000.0
5000.0
4000.0
3000.0
2000.0
1000.0
0.0
0.00
200.00
400.00
600.00
800.00
1000.00
1200.00
1400.00
1600.00
y7++-NH3
y11++-NH3
b6-H2O
b6
y15++
y17++-H2O
b18++-NH3
y9-NH3
y10
y11
b11-NH3
y12
b11
y13-NH3
y13
y14

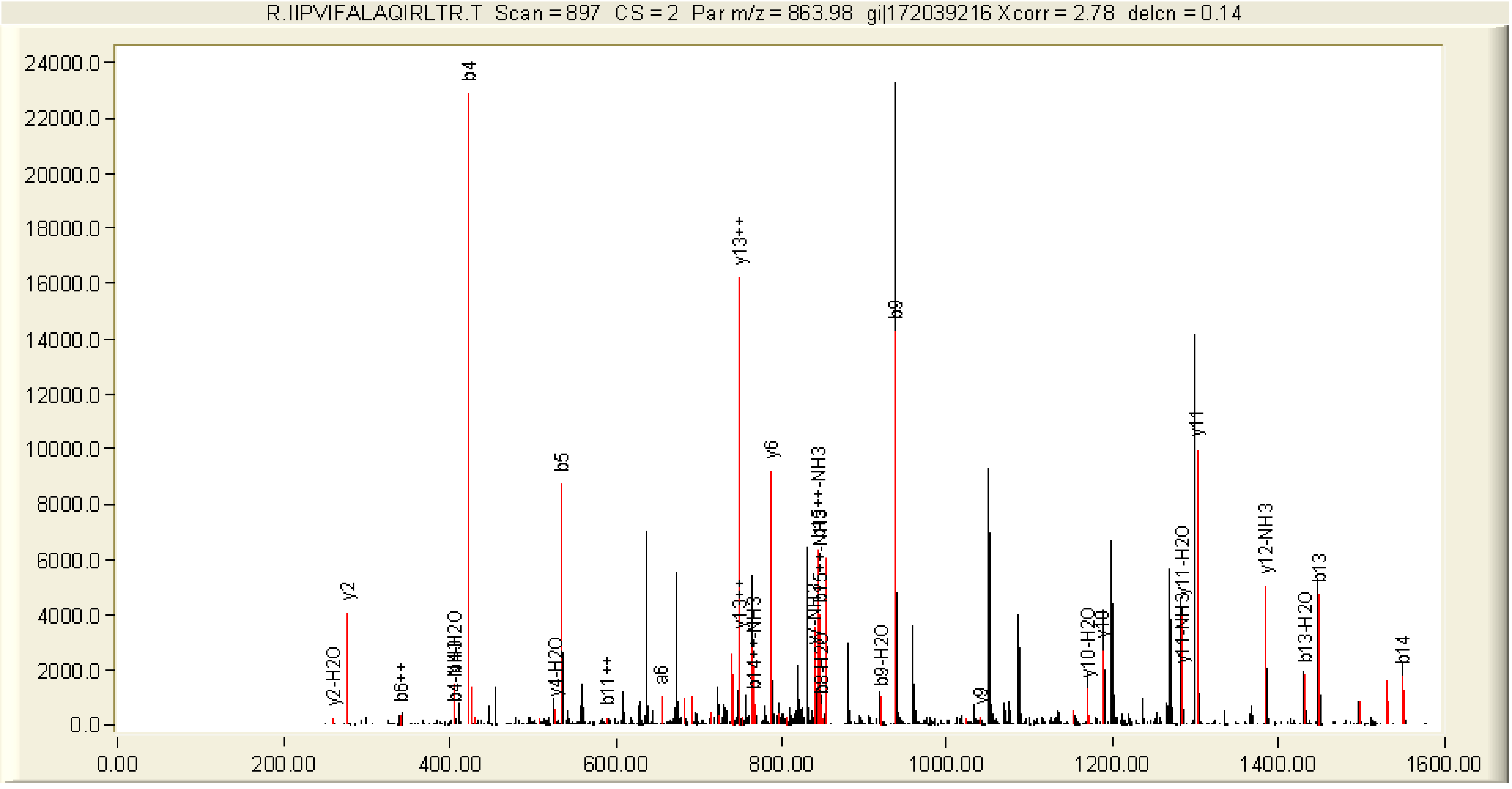

R.IIPVIFALAQIRLTR.T Scan = 897 CS = 2 Par m/z = 863.98 gi|172039216 Xcorr = 2.78 delcn = 0.14
b4
y13++
b9
y11
b5
y6
y2
y12-NH3
b13
b13-H2O
b14
y2-H2O
b6++
b11++
a6
y4-H2O
b9-H2O
y10-H2O
y10
y11-H2O
y11-NH3
y9
0.00
200.00
400.00
600.00
800.00
1000.00
1200.00
1400.00
1600.00
0.0
2000.0
4000.0
6000.0
8000.0
10000.0
12000.0
14000.0
16000.0
18000.0
20000.0
22000.0
24000.0

K.NAWEETFNKDPIDLR.P Scan = 899 CS = 2 Par m/z = 924.99 gi|172035119 Xcorr = 2.61 delcn = 0.18

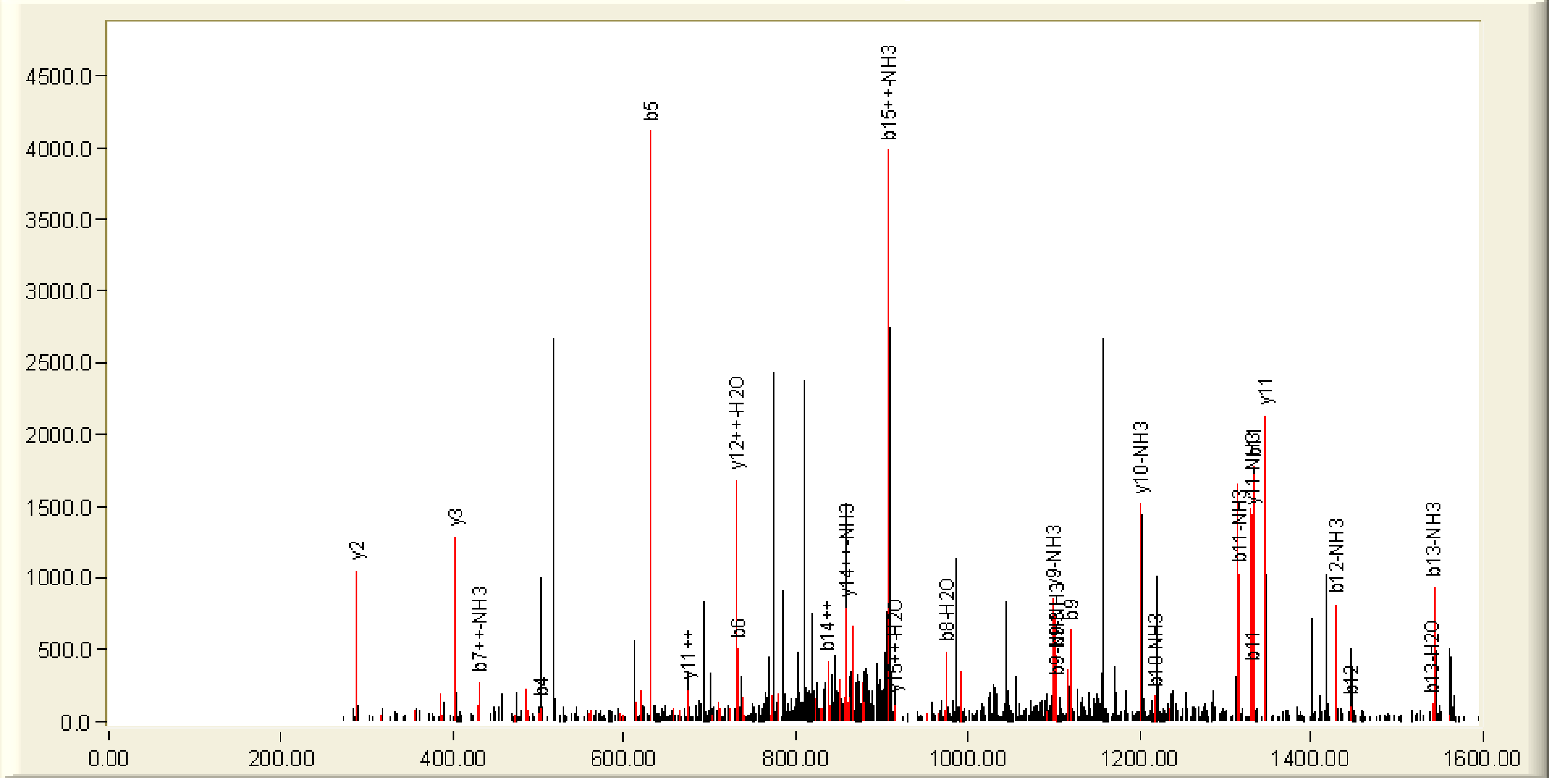

K.ELETHTVVGLDIETTGLDPHTSEISLIQIAAPNRPVILIEFR.G Scan = 901 CS = 3 Par m/z = 1546.11 gi|172054918 Xcorr = 3.49 delcn = 0.25

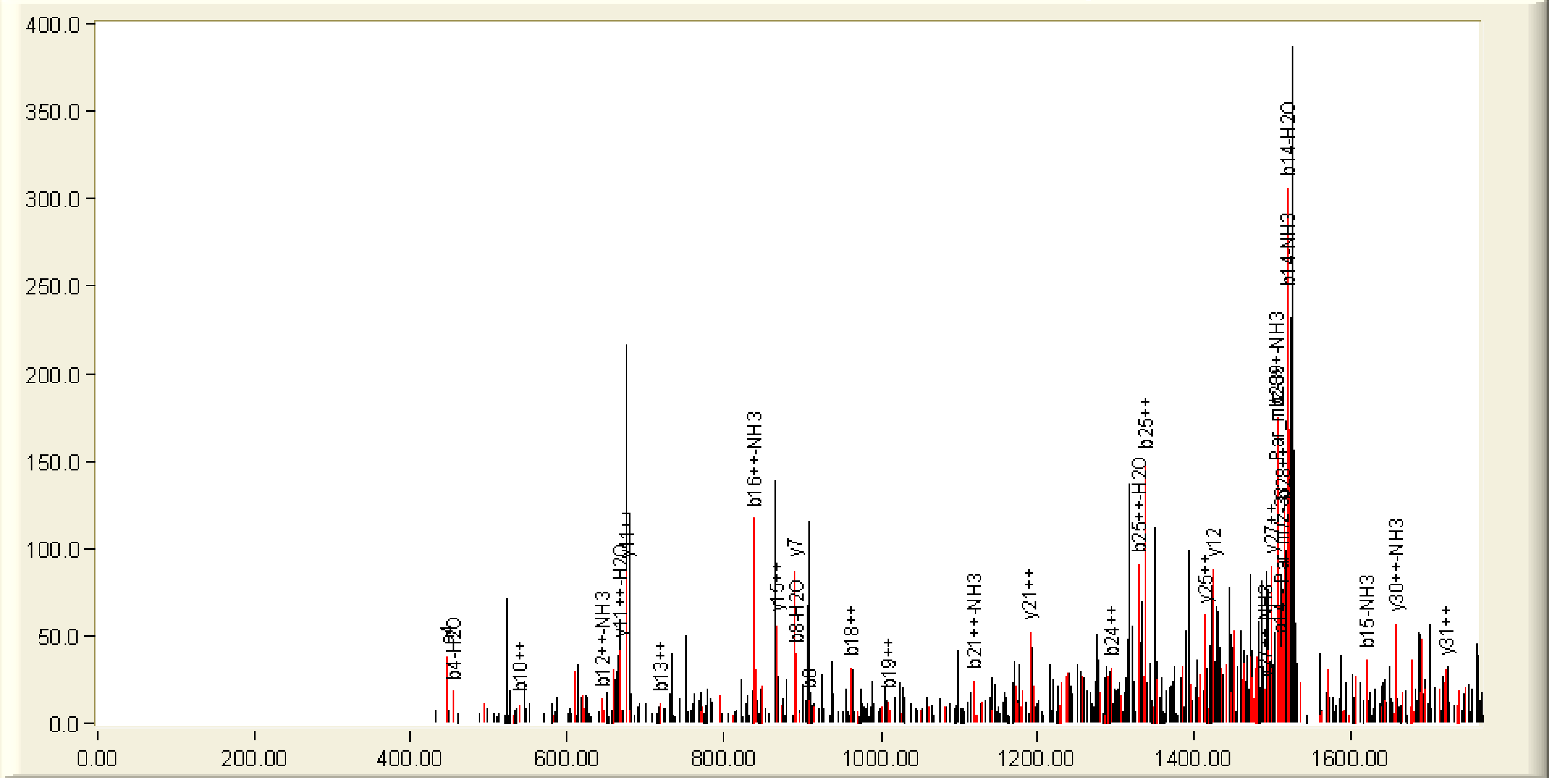

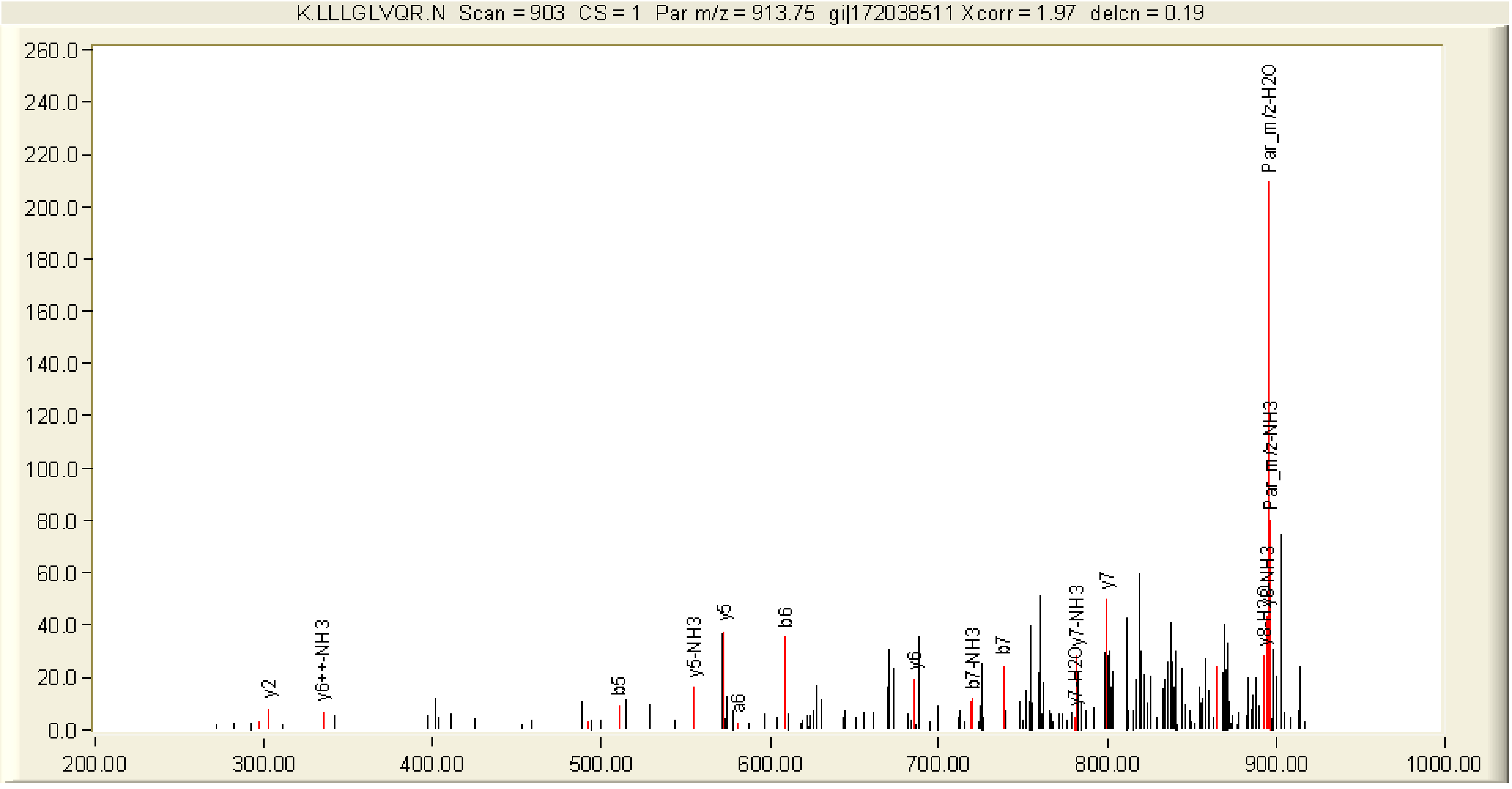

K.LLLGLVQR.N Scan = 903 CS = 1 Par m/z = 913.75 gi|172038511 Xcorr = 1.97 delcn = 0.19
260.0
240.0
220.0
200.0
180.0
160.0
140.0
120.0
100.0
80.0
60.0
40.0
20.0
0.0
200.00
300.00
400.00
500.00
600.00
700.00
800.00
900.00
1000.00
y2
y6++-NH3
b5
y5-NH3
y5
a6
b6
y6
b7-NH3
b7
y7-H2O
y7-NH3
y7
Par_m/z-H2O
Par_m/z-NH3

K.FYLGAILIVFAGVLVLKNQLPDTDR.S Scan = 905 CS = 3 Par m/z = 926.42 gi|172054988 Xcorr = 3.7 delcn = 0.11

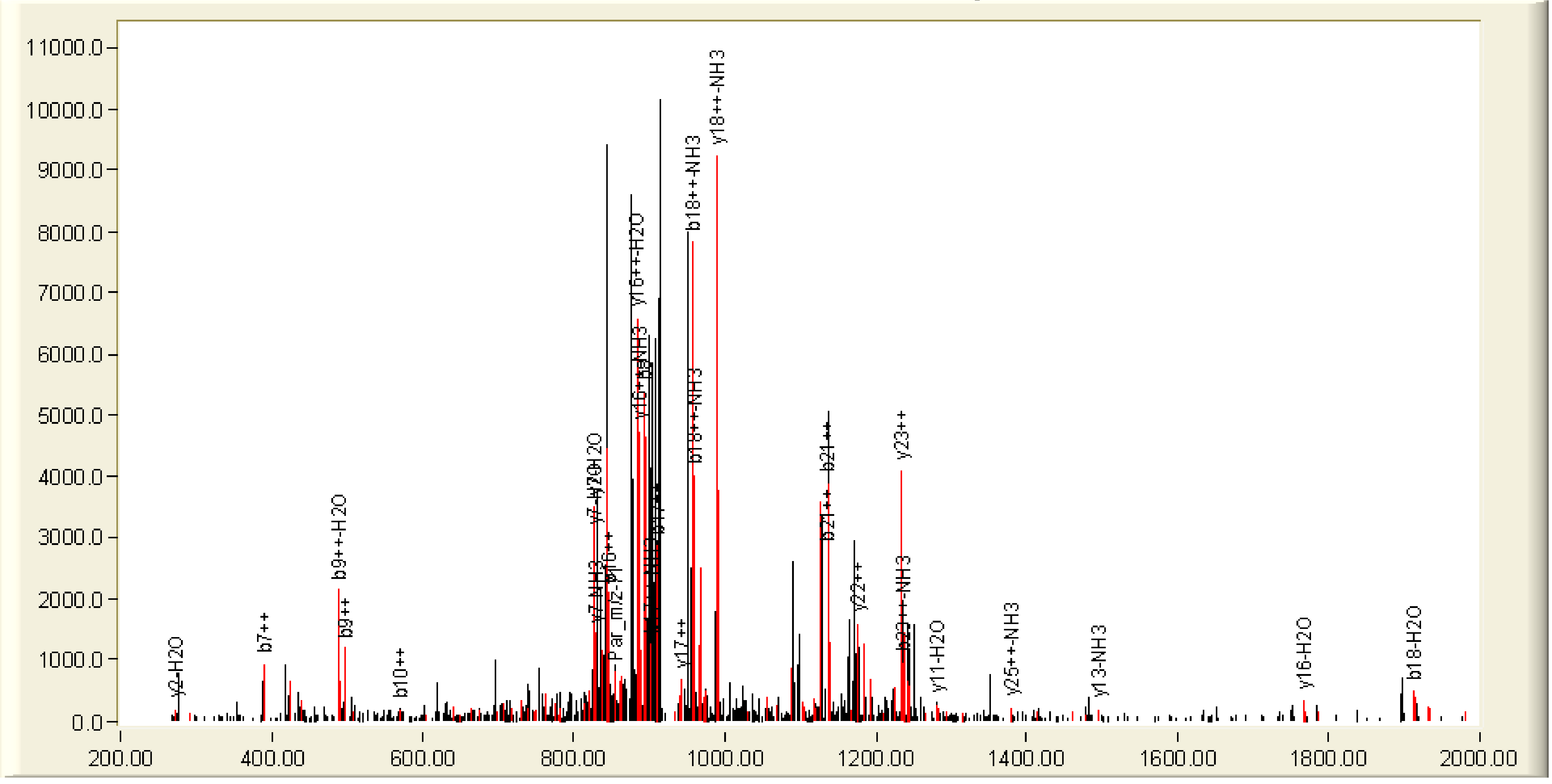

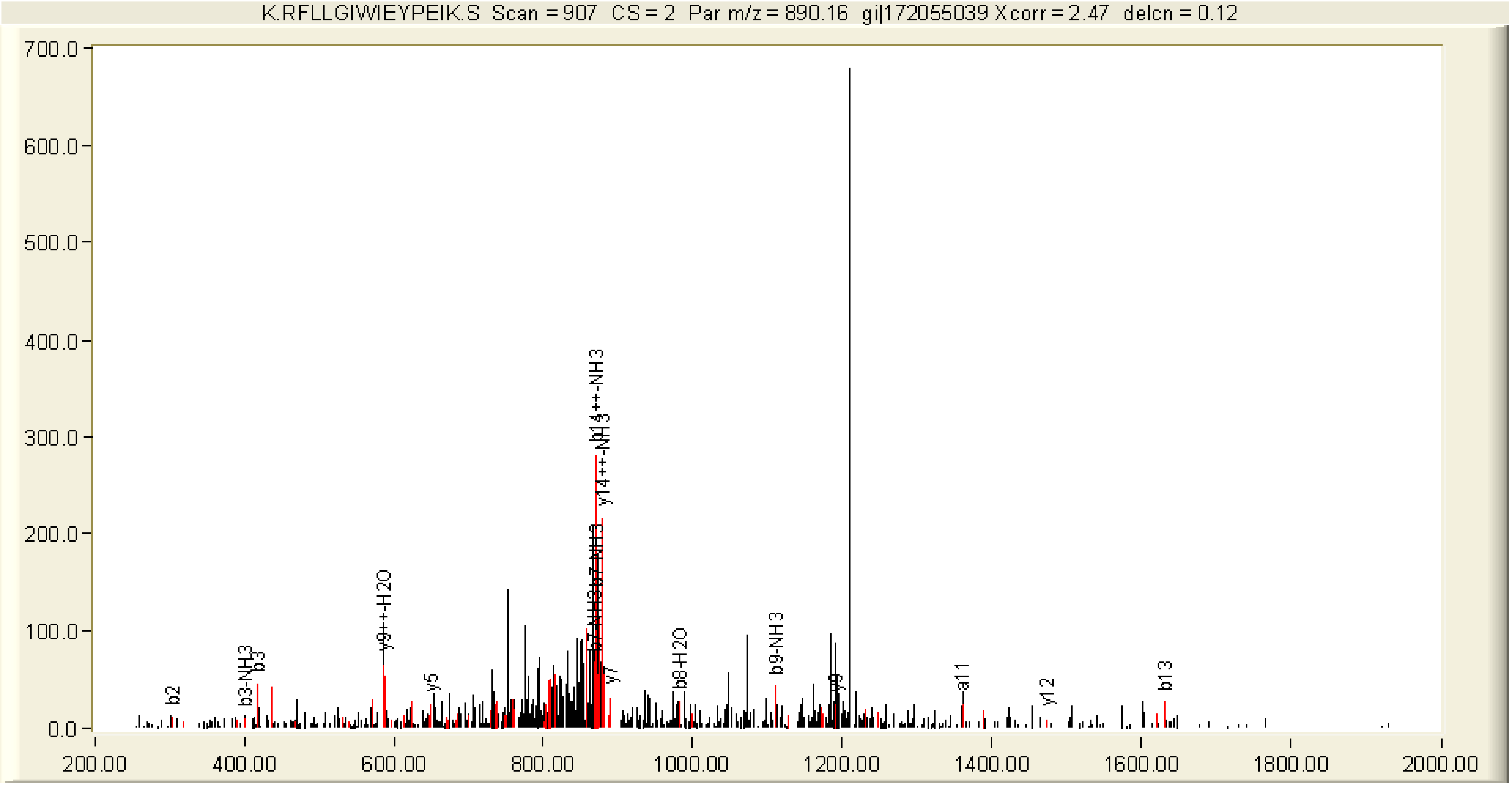
K.RFLLGIWIEYPEIK.S Scan = 907 CS = 2 Par m/z = 890.16 gi|172055039 Xcorr = 2.47 delcn = 0.12
700.0
600.0
500.0
400.0
300.0
200.0
100.0
0.0
200.00
400.00
600.00
800.00
1000.00
1200.00
1400.00
1600.00
1800.00
2000.00
b2
b3-NH3
b3
y9++-H2O
y5
y14++-NH3
y7
b8-H2O
b9-NH3
a11
y12
b13

R.TLLEDLGTICLNTVECTLESGKYVFDKITR.P Scan = 909 CS = 3 Par m/z = 1125.92 gi|172035985 Xcorr = 3.3 delcn = 0.11

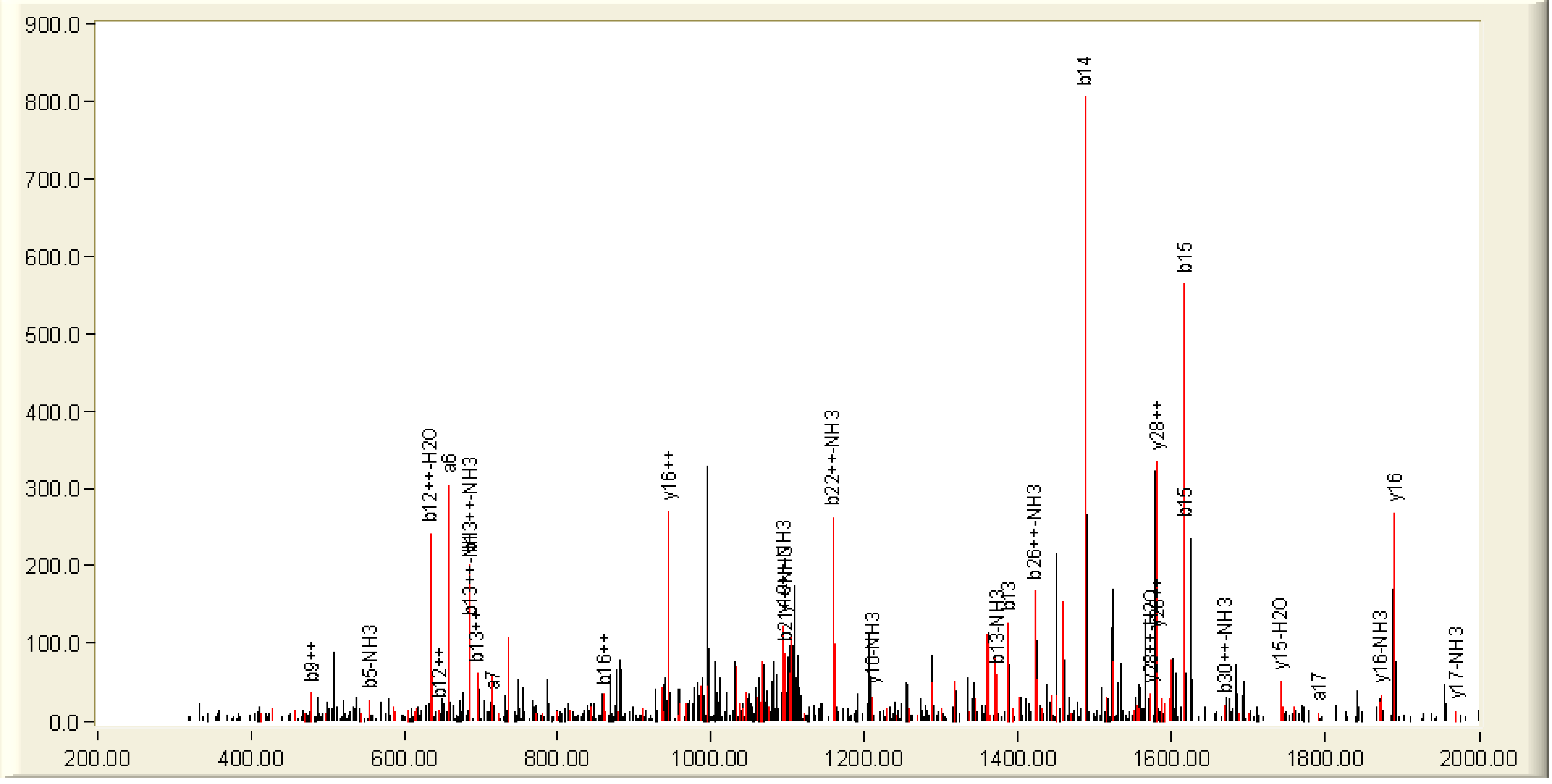

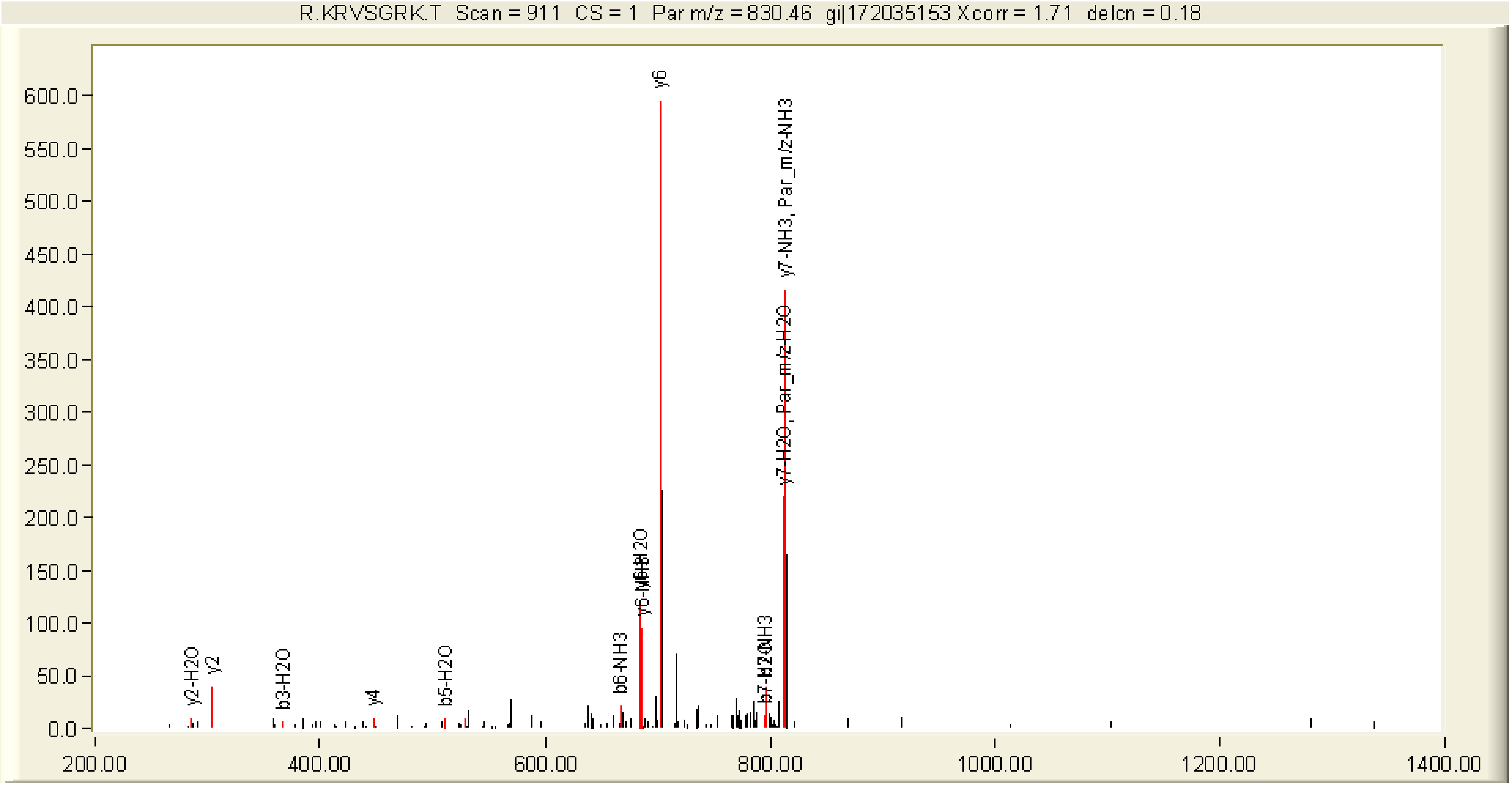
R.KRVSGRK.T Scan = 911 CS = 1 Par m/z = 830.46 gi|172035153 Xcorr = 1.71 delcn = 0.18
y6
y7-NH3, Par_m/z-NH3
y7-H2O, Par_m/z-H2O
y2-H2O
y2
b3-H2O
y4
b5-H2O
b6-NH3
600.0
550.0
500.0
450.0
400.0
350.0
300.0
250.0
200.0
150.0
100.0
50.0
0.0
200.00
400.00
600.00
800.00
1000.00
1200.00
1400.00

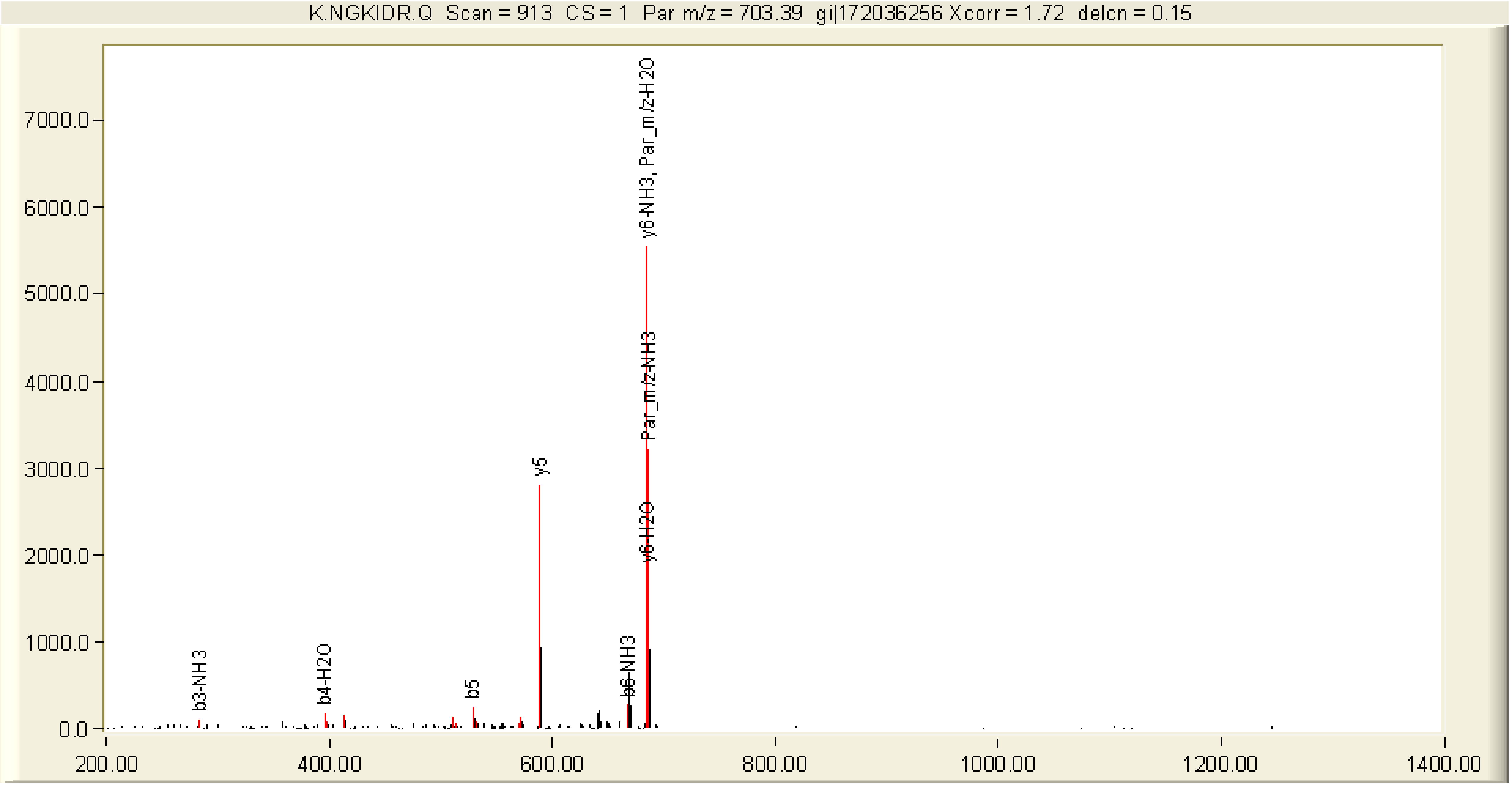

K.NGKIDR.Q Scan = 913 CS = 1 Par m/z = 703.39 gi|172036256 Xcorr = 1.72 delcn = 0.15
y6-NH3, Par_m/z-H2O
Par_m/z-NH3
y5
y6-H2O
b3-NH3
b4-H2O
b5
b6-NH3
7000.0
6000.0
5000.0
4000.0
3000.0
2000.0
1000.0
0.0
200.00
400.00
600.00
800.00
1000.00
1200.00
1400.00

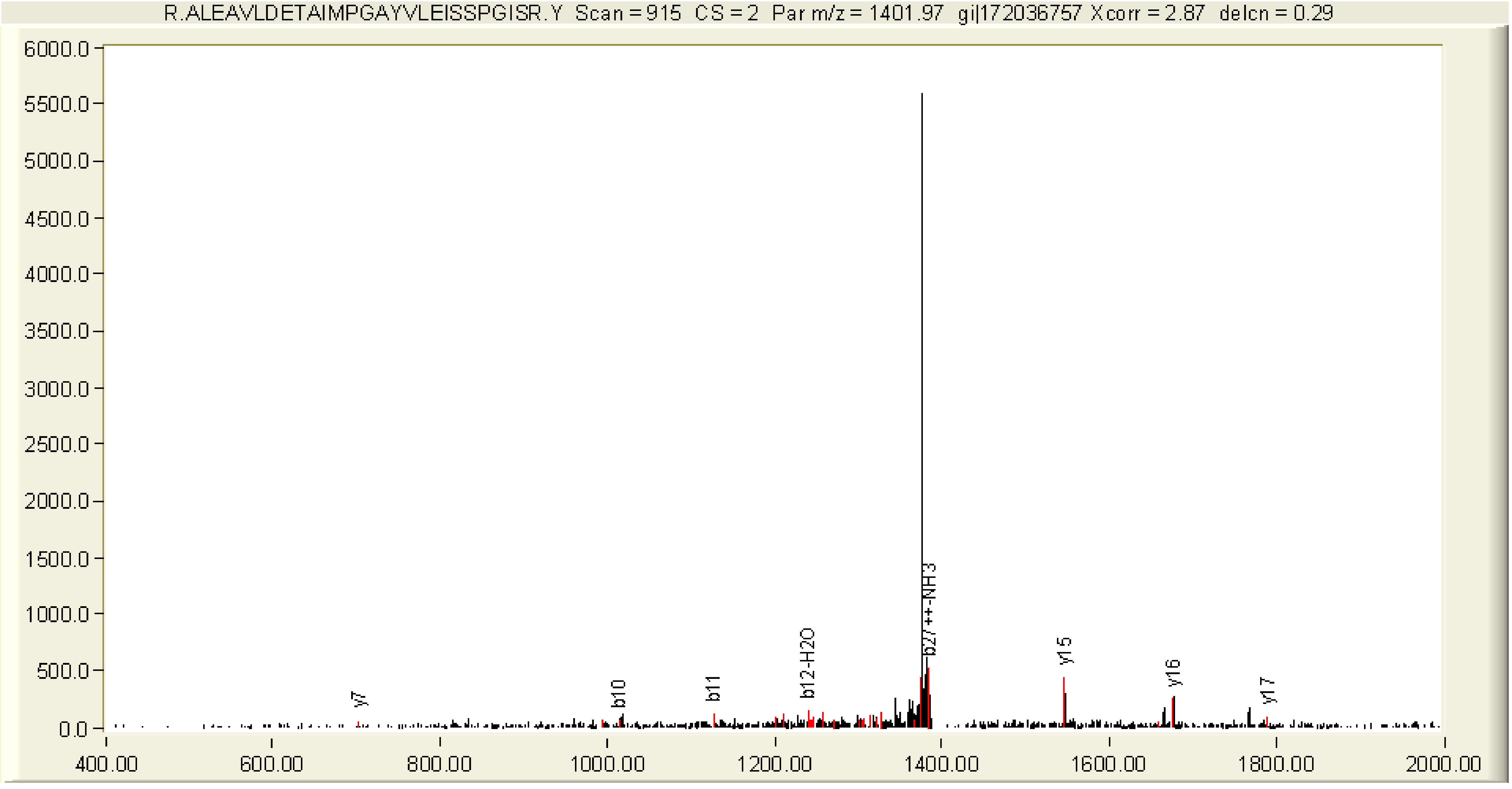
R.ALEAVLDETAIMPGAYVLEISSPGISR.Y Scan = 915 CS = 2 Par m/z = 1401.97 gi|172036757 Xcorr = 2.87 delcn = 0.29
6000.0
5500.0
5000.0
4500.0
4000.0
3500.0
3000.0
2500.0
2000.0
1500.0
1000.0
500.0
0.0
y7
b10
b11
b12-H2O
b27++-NH3
y15
y16
y17
400.00
600.00
800.00
1000.00
1200.00
1400.00
1600.00
1800.00
2000.00

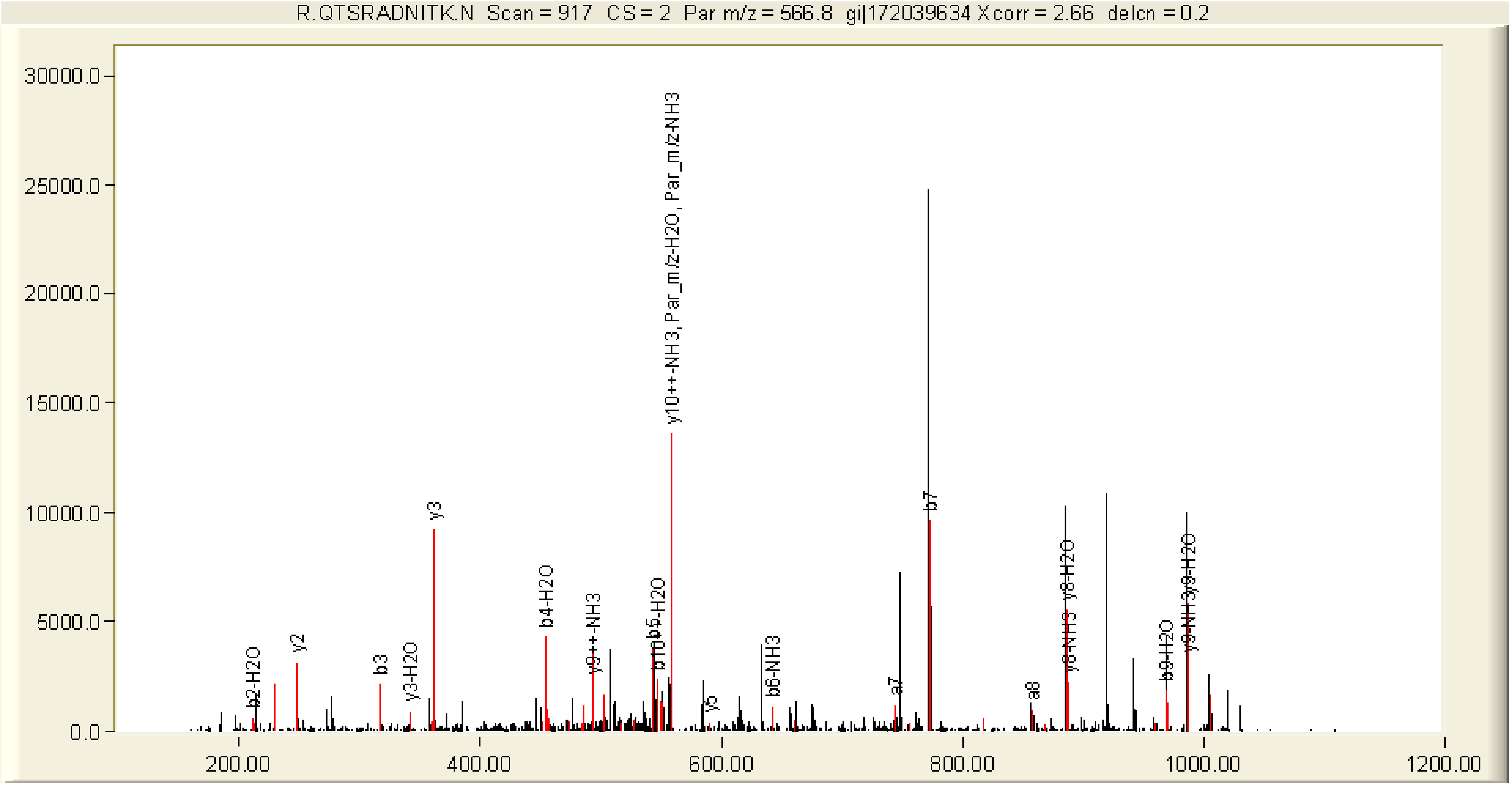

R.QTSRADNITK.N Scan = 917 CS = 2 Par m/z = 566.8 gi|172039634 Xcorr = 2.66 delcn = 0.2
30000.0
25000.0
20000.0
15000.0
10000.0
5000.0
0.0
200.00
400.00
600.00
800.00
1000.00
1200.00
y10++-NH3, Par_m/z-H2O, Par_m/z-NH3
b7
y3
b4-H2O
y9++-NH3
b5-H2O
b6-NH3
b2-H2O
y2
b3
y3-H2O
y5
a7
a8
y8-NH3
y8-H2O
b9-H2O
y9-H2O

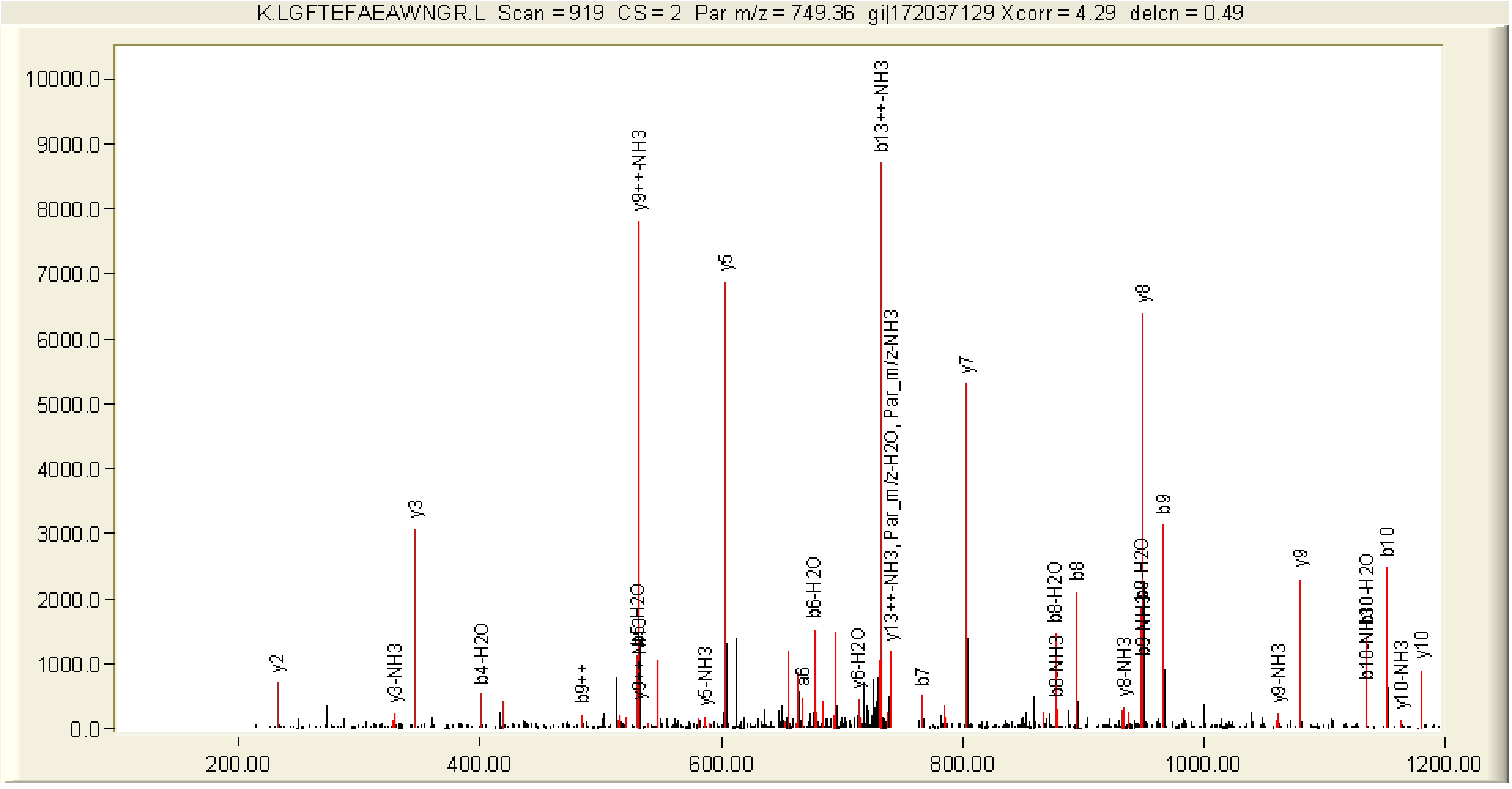

K.LGFTEFAEAWNGR.L Scan = 919 CS = 2 Par m/z = 749.36 gi|172037129 Xcorr = 4.29 delcn = 0.49
10000.0
9000.0
8000.0
7000.0
6000.0
5000.0
4000.0
3000.0
2000.0
1000.0
0.0
200.00
400.00
600.00
800.00
1000.00
1200.00
y2
y3-NH3
y3
b4-H2O
b9++
y9++-NH3
y5-NH3
y5
a6
b6-H2O
y6-H2O
y13++-NH3, Par_m/z-H2O, Par_m/z-NH3
b13++-NH3
b7
y7
b8-NH3
b8-H2O
b8
y8-NH3
y8
b9
y9-NH3
y9
b10-H2O
b10
y10-NH3
y10

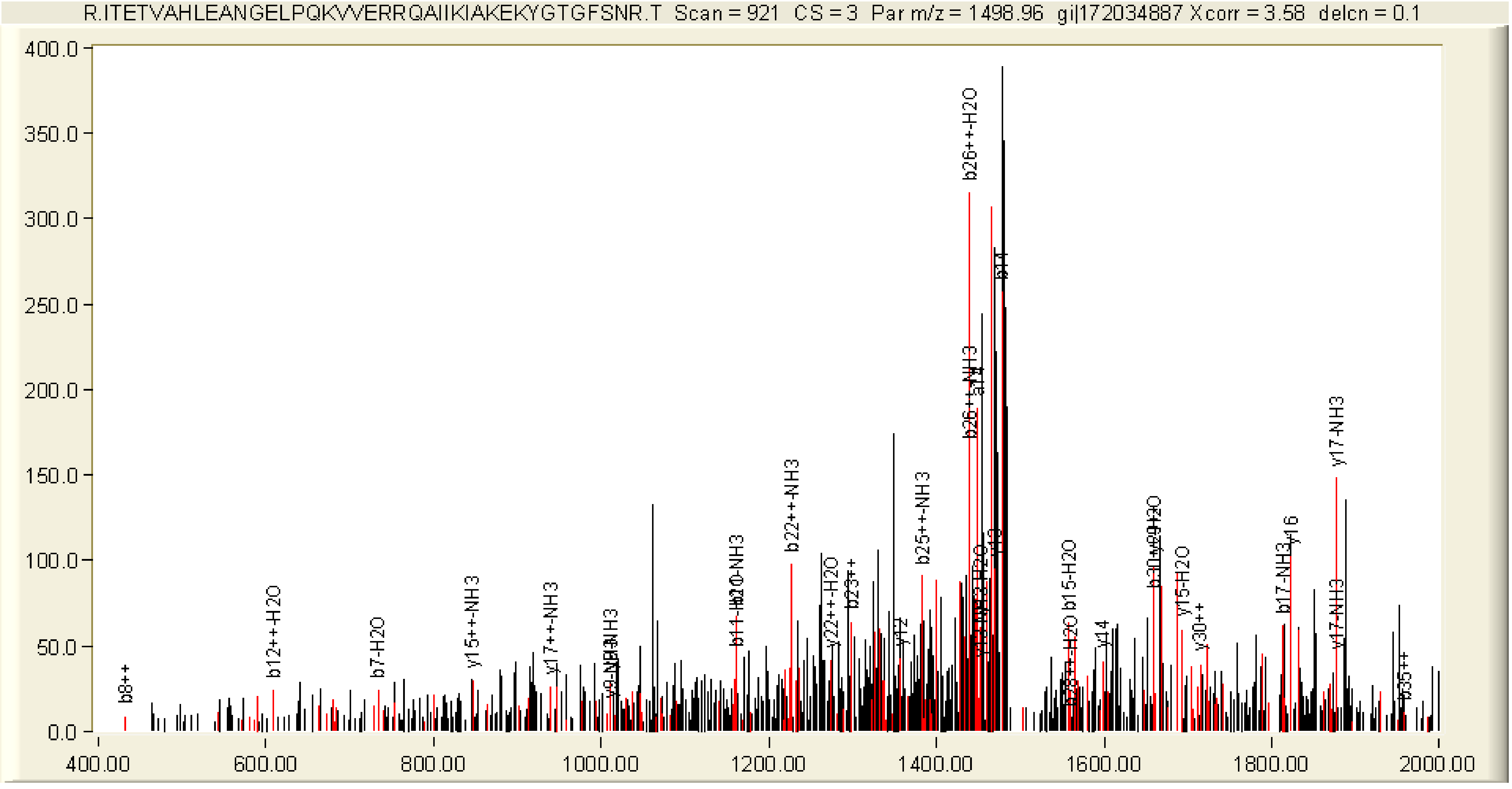
R.ITETVAHLEANGELPQKVVERRQAIIKIAKEKYGTGFSNR.T Scan = 921 CS = 3 Par m/z = 1498.96 gi|172034887 Xcorr = 3.58 delcn = 0.1
400.0
350.0
300.0
250.0
200.0
150.0
100.0
50.0
0.0
400.00
600.00
800.00
1000.00
1200.00
1400.00
1600.00
1800.00
2000.00
b8++
b12++-H2O
b7-H2O
y15++-NH3
y17++-NH3
b22++-NH3
b25++-NH3
y22++-H2O
b23++
y12
b26++-H2O
b26++-NH3
y14
b15-H2O
y15-H2O
y30++
b17-NH3
y16
y17-NH3
b35++

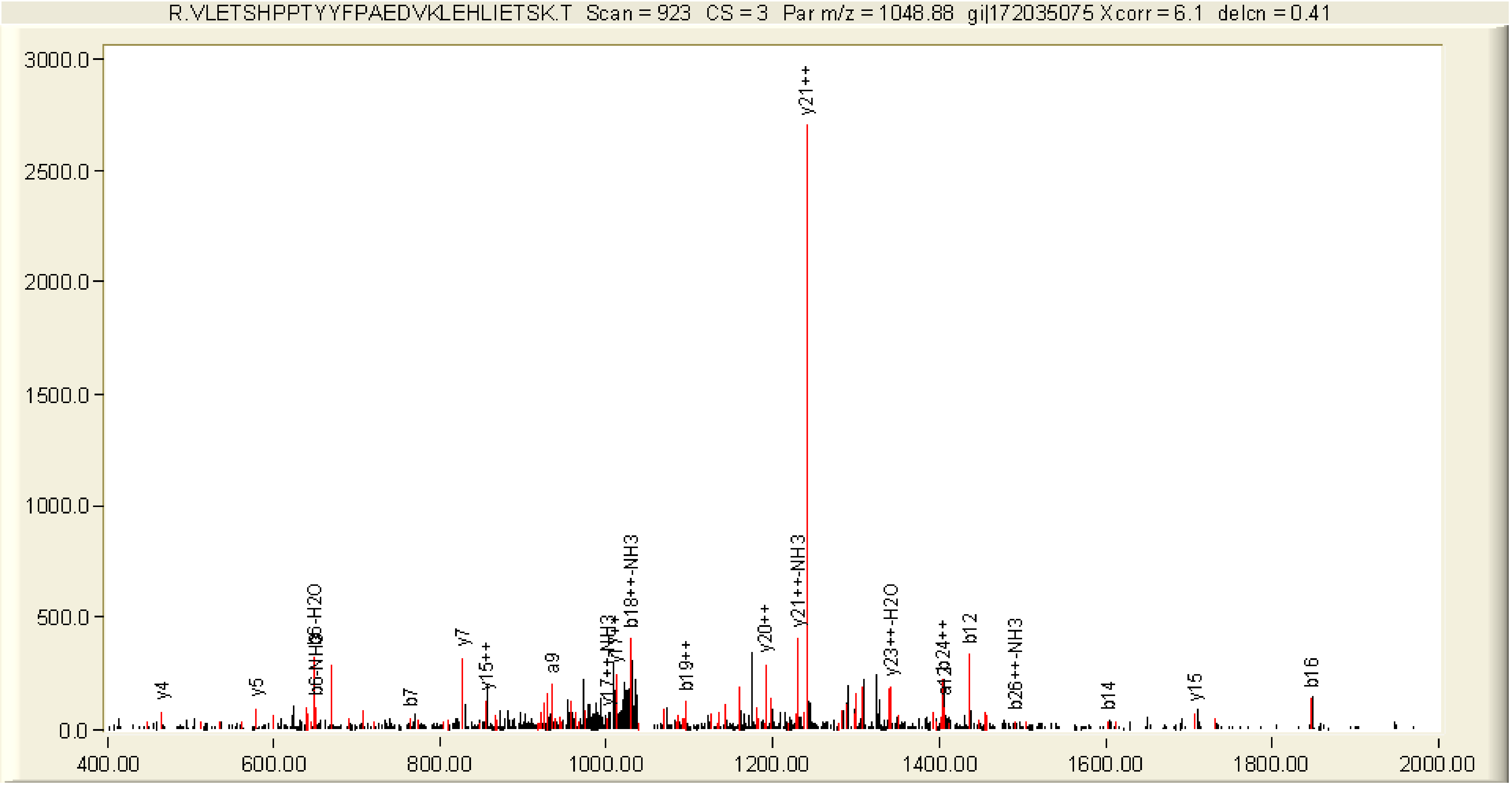
R.VLETSHPPTYYFPAEDVKLEHLIETSK.T Scan = 923 CS = 3 Par m/z = 1048.88 gi|172035075 Xcorr = 6.1 delcn = 0.41
3000.0
2500.0
2000.0
1500.0
1000.0
500.0
0.0
400.00
600.00
800.00
1000.00
1200.00
1400.00
1600.00
1800.00
2000.00
y21++
b18++-NH3
y21++-NH3
y23++-H2O
y20++
b12
b26++-NH3
y4
y5
b7
y7
y15++
a9
b19++
b14
y15
b16

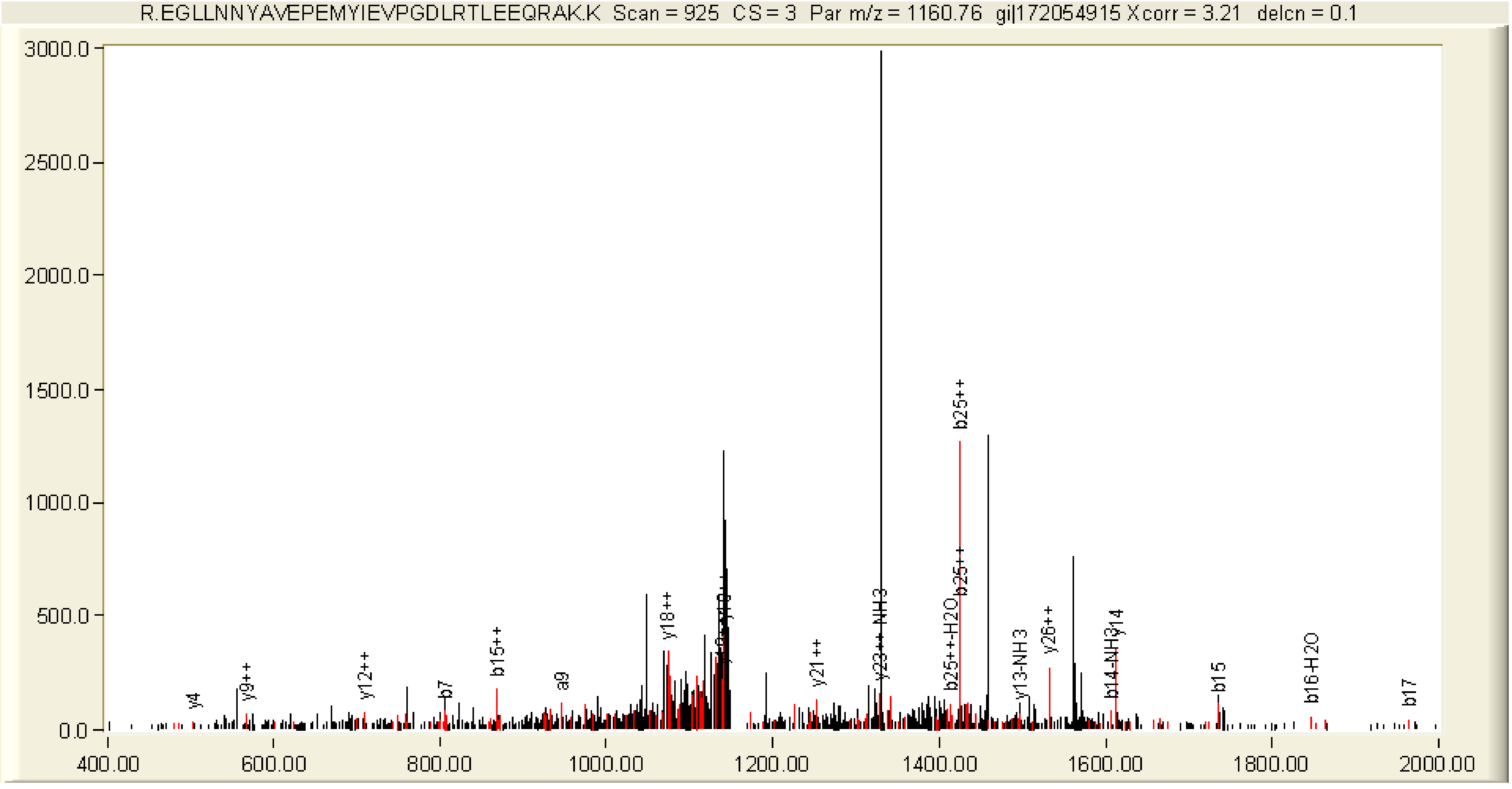
R.EGLLNNYAVEPEMYIEVPGDLRTLEEQRAK.K Scan = 925 CS = 3 Par m/z = 1160.76 gi|172054915 Xcorr = 3.21 delcn = 0.1
3000.0
2500.0
2000.0
1500.0
1000.0
500.0
0.0
400.00
600.00
800.00
1000.00
1200.00
1400.00
1600.00
1800.00
2000.00
y4
y9++
y12++
b7
b15++
a9
y18++
y21++
y23++-NH3
b25++-H2O
b25++
b25++
y13-NH3
y26++
b14-NH3
y14
b15
b16-H2O
b17

K.ELDDILTNDSEPSTKSDPAINTNTVFSSAQTFNQFK.T Scan = 927 CS = 3 Par m/z = 1325.7 gi|172038561 Xcorr = 3.22 delcn = 0.17

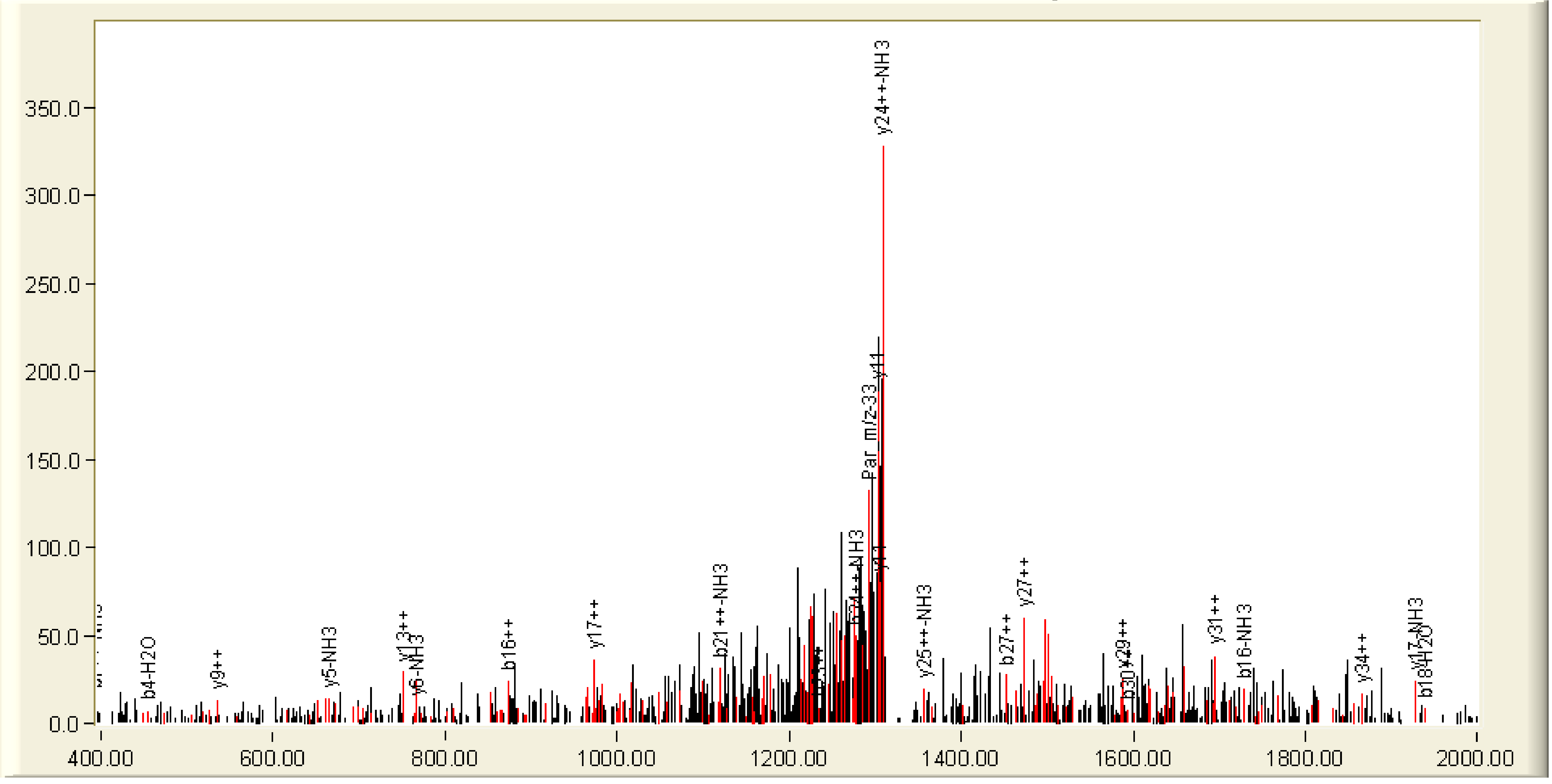

R.NRSFEQVIADGSLSLLSHHLQK.G Scan = 929 CS = 2 Par m/z = 1241.14 gi|172055208 Xcorr = 2.62 delcn = 0.13

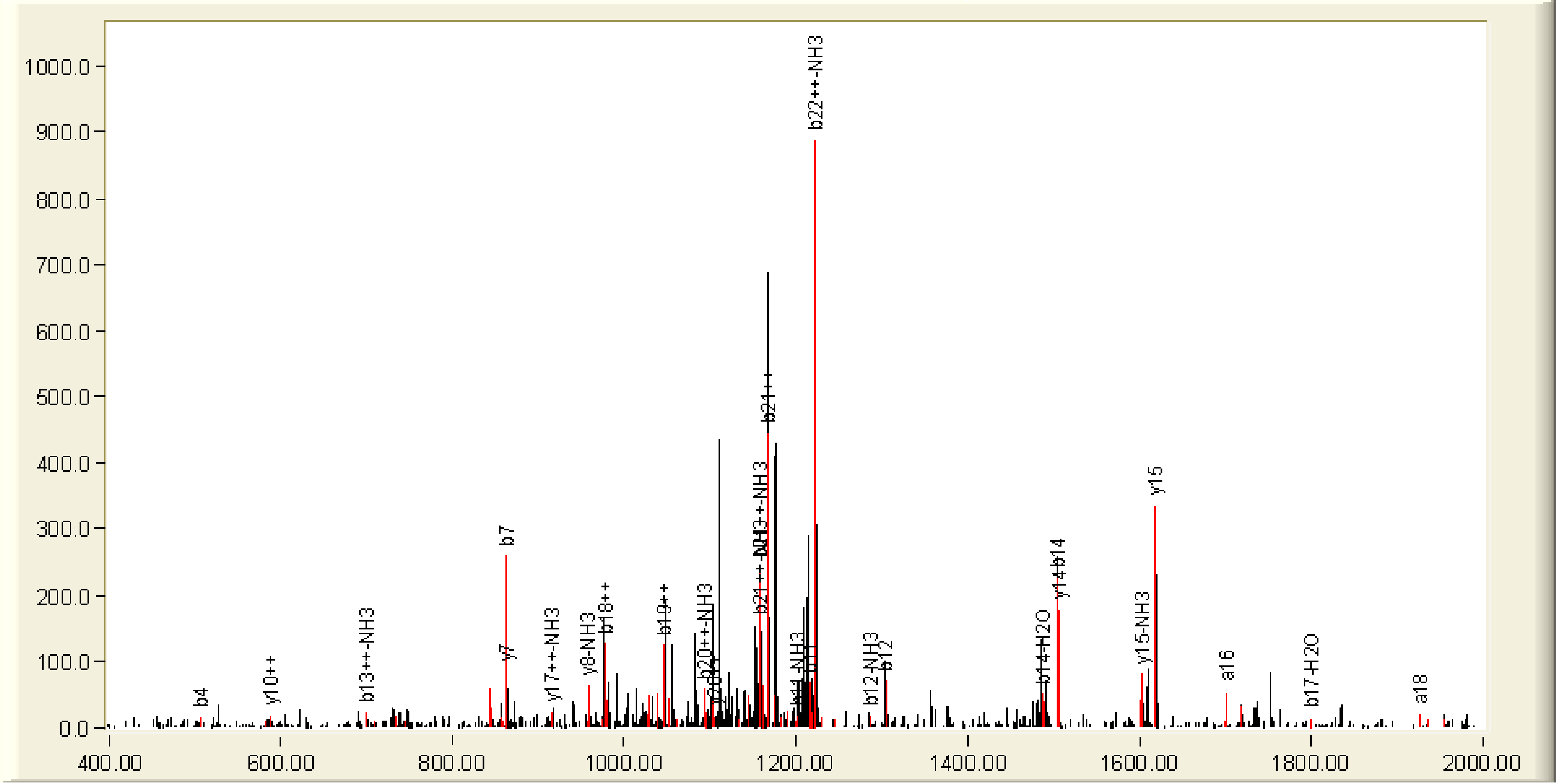

K.QWFTDTQNNGWDGIEHDYYKSVTKGHH.R Scan = 931 CS = 3 Par m/z = 1089.66 gi|172036643 Xcorr = 5.27 delcn = 0.36

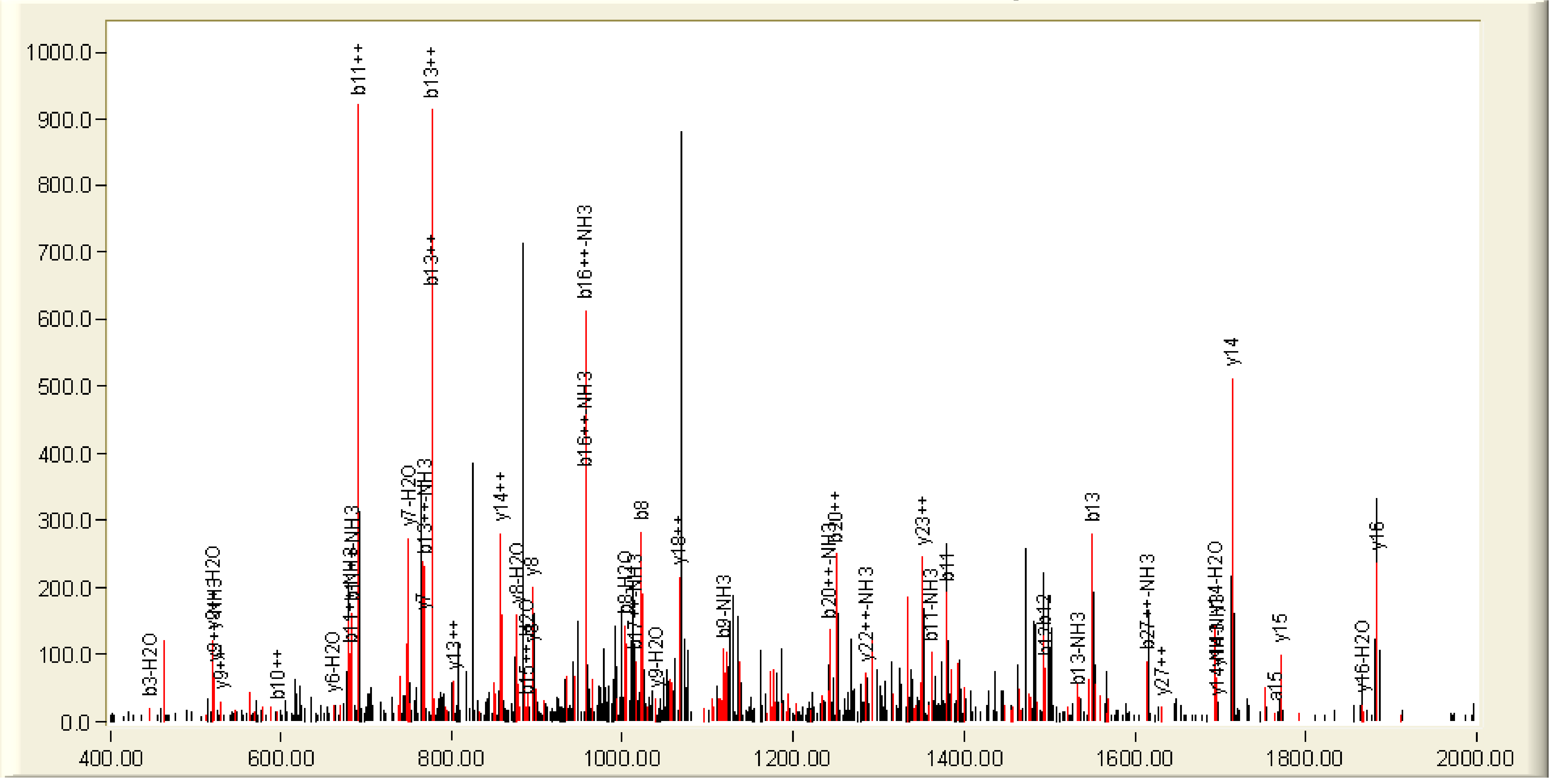

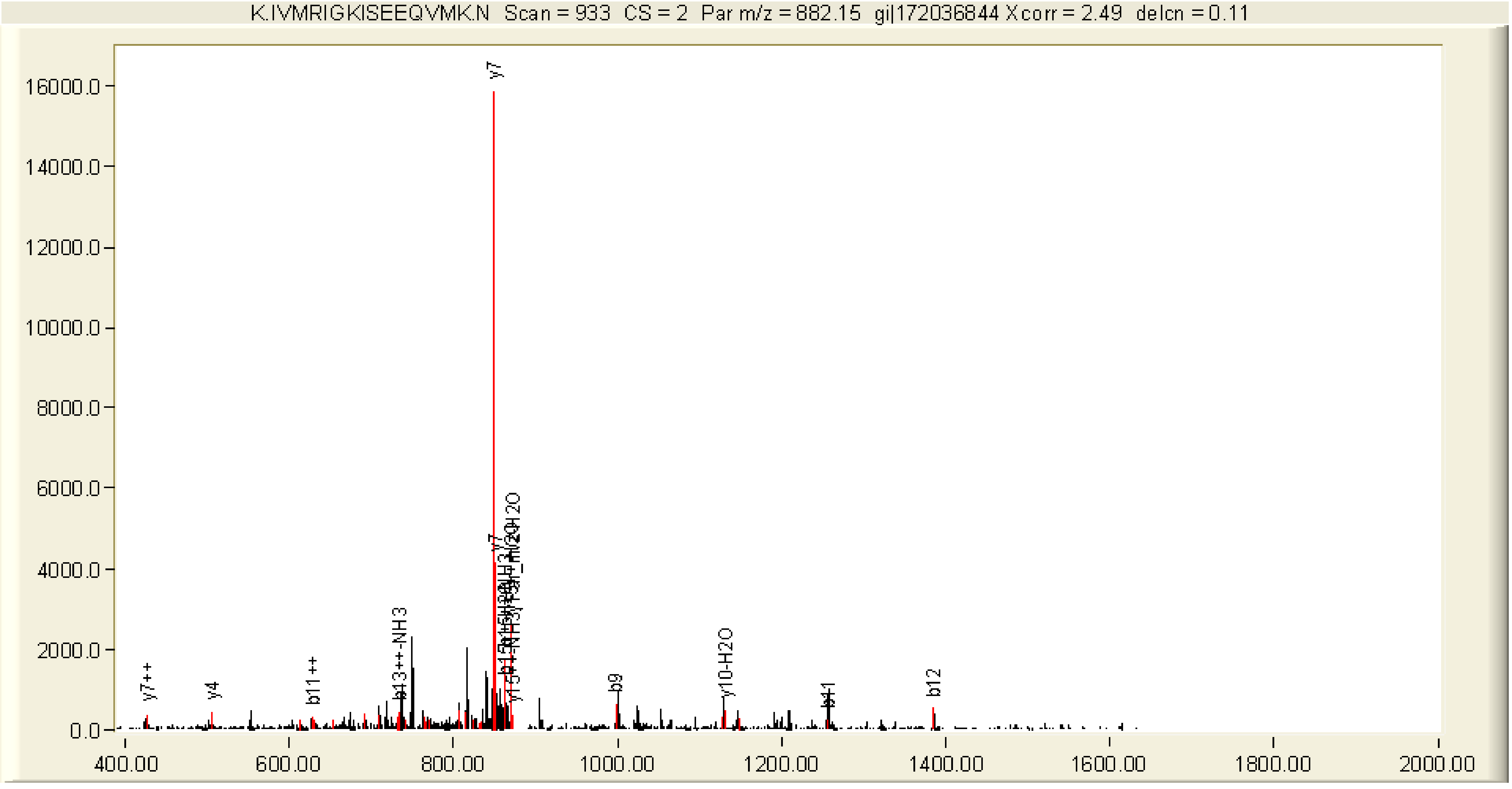

K.IVMRIGKISEEQVMK.N Scan = 933 CS = 2 Par m/z = 882.15 gi|172036844 Xcorr = 2.49 delcn = 0.11
16000.0
14000.0
12000.0
10000.0
8000.0
6000.0
4000.0
2000.0
0.0
400.00
600.00
800.00
1000.00
1200.00
1400.00
1600.00
1800.00
2000.00
y7
y7++
y4
b11++
b13++-NH3
b9
y10-H2O
b11
b12

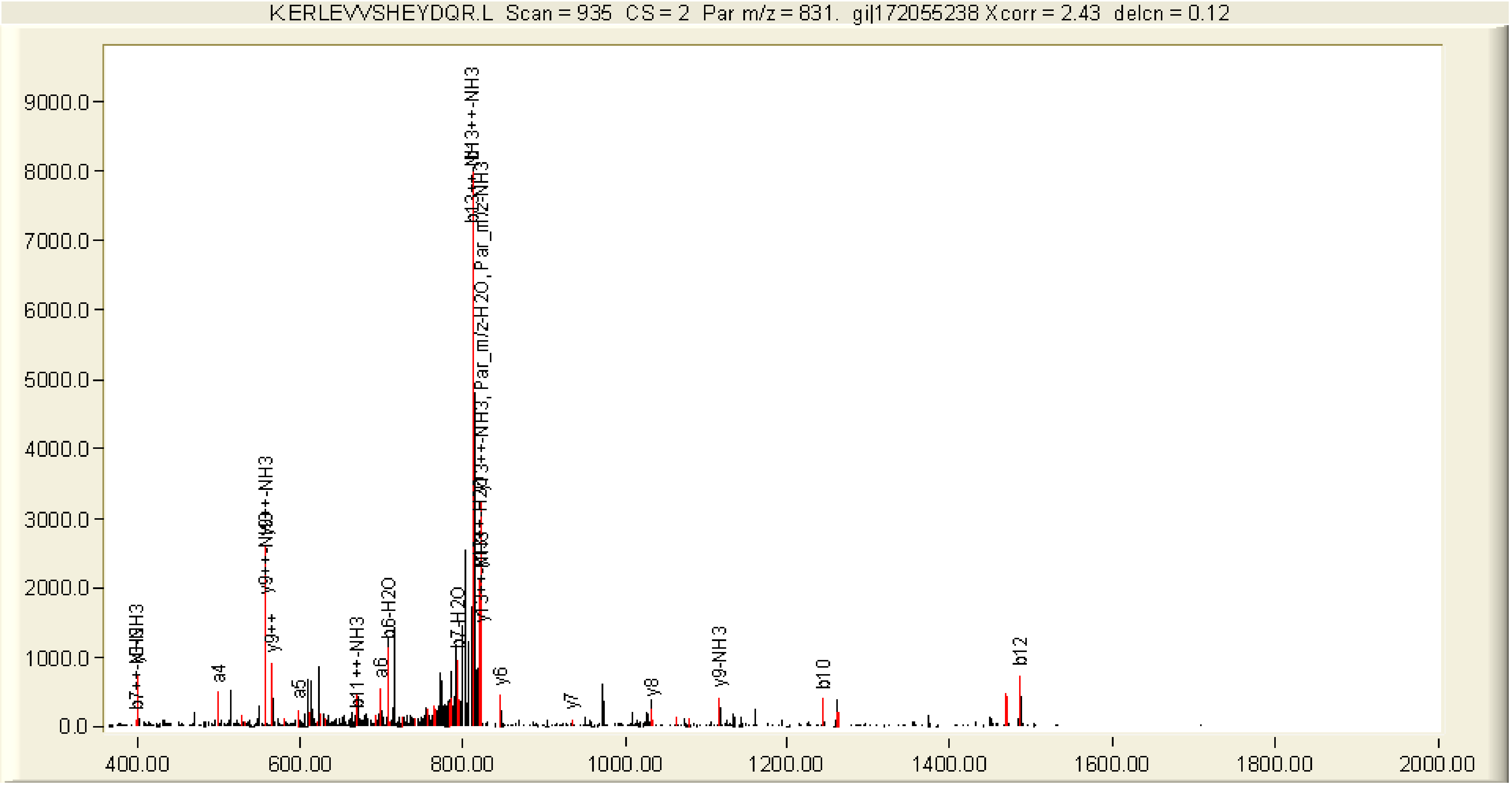

KERLEVVSHEYDQR.L Scan = 935 CS = 2 Par m/z = 831. gi|172055238 Xcorr = 2.43 delcn = 0.12
9000.0
8000.0
7000.0
6000.0
5000.0
4000.0
3000.0
2000.0
1000.0
0.0
400.00
600.00
800.00
1000.00
1200.00
1400.00
1600.00
1800.00
2000.00
a4
y9++
a5
b11++-NH3
a6
b6-H2O
b7-H2O
y6
y7
y8
y9-NH3
b10
b12

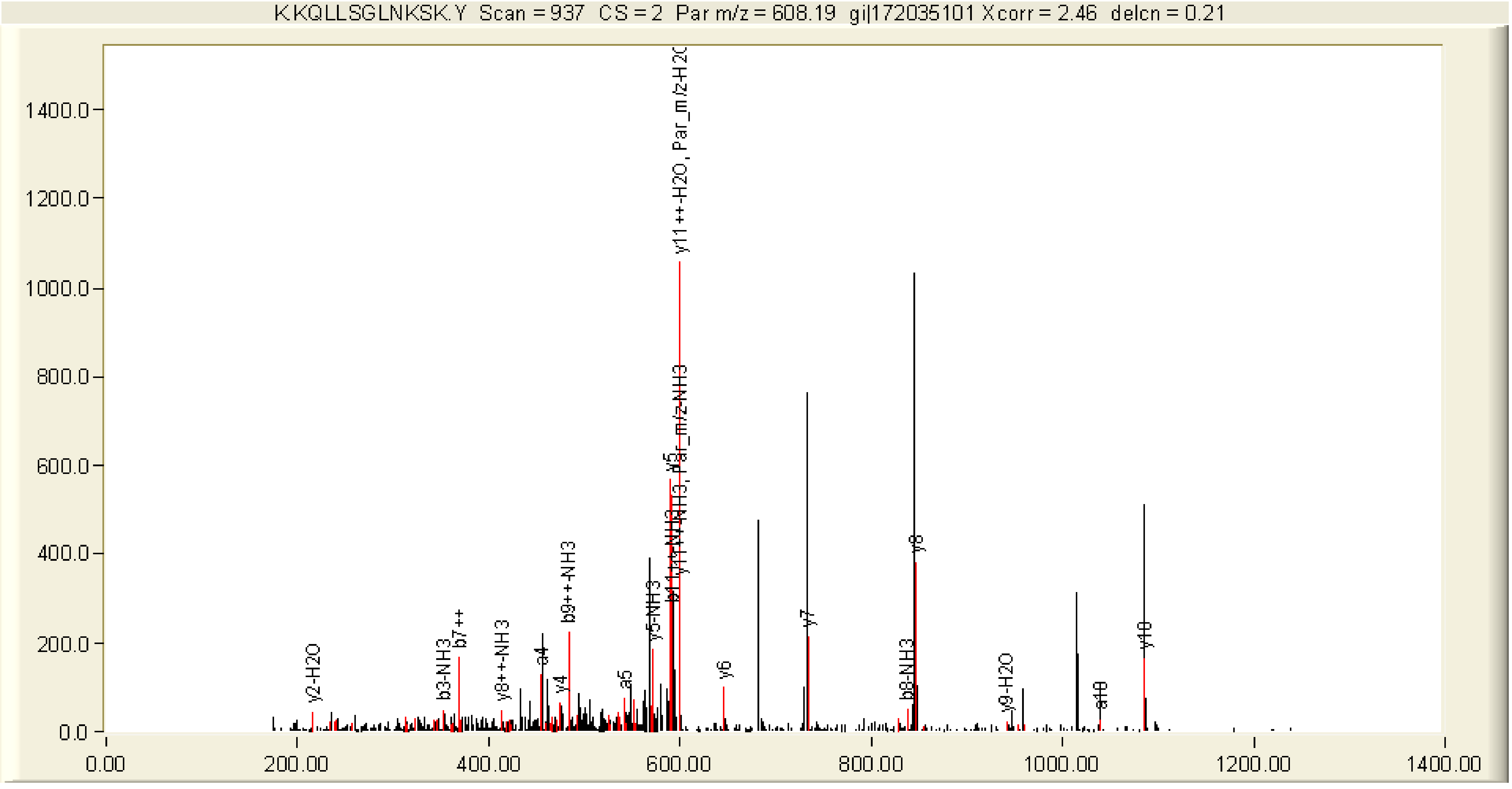

K.KQLLSGLNKSK.Y Scan = 937 CS = 2 Par m/z = 608.19 gi|172035101 Xcorr = 2.46 delcn = 0.21
1400.0
1200.0
1000.0
800.0
600.0
400.0
200.0
0.0
0.00
200.00
400.00
600.00
800.00
1000.00
1200.00
1400.00
y2-H2O
b3-NH3
b7++
y8++-NH3
a4
y4
b9++-NH3
a5
y5-NH3
y11++-H2O, Par_m/z-H2O
y6
y7
b8-NH3
y8
y9-H2O
a10
y10

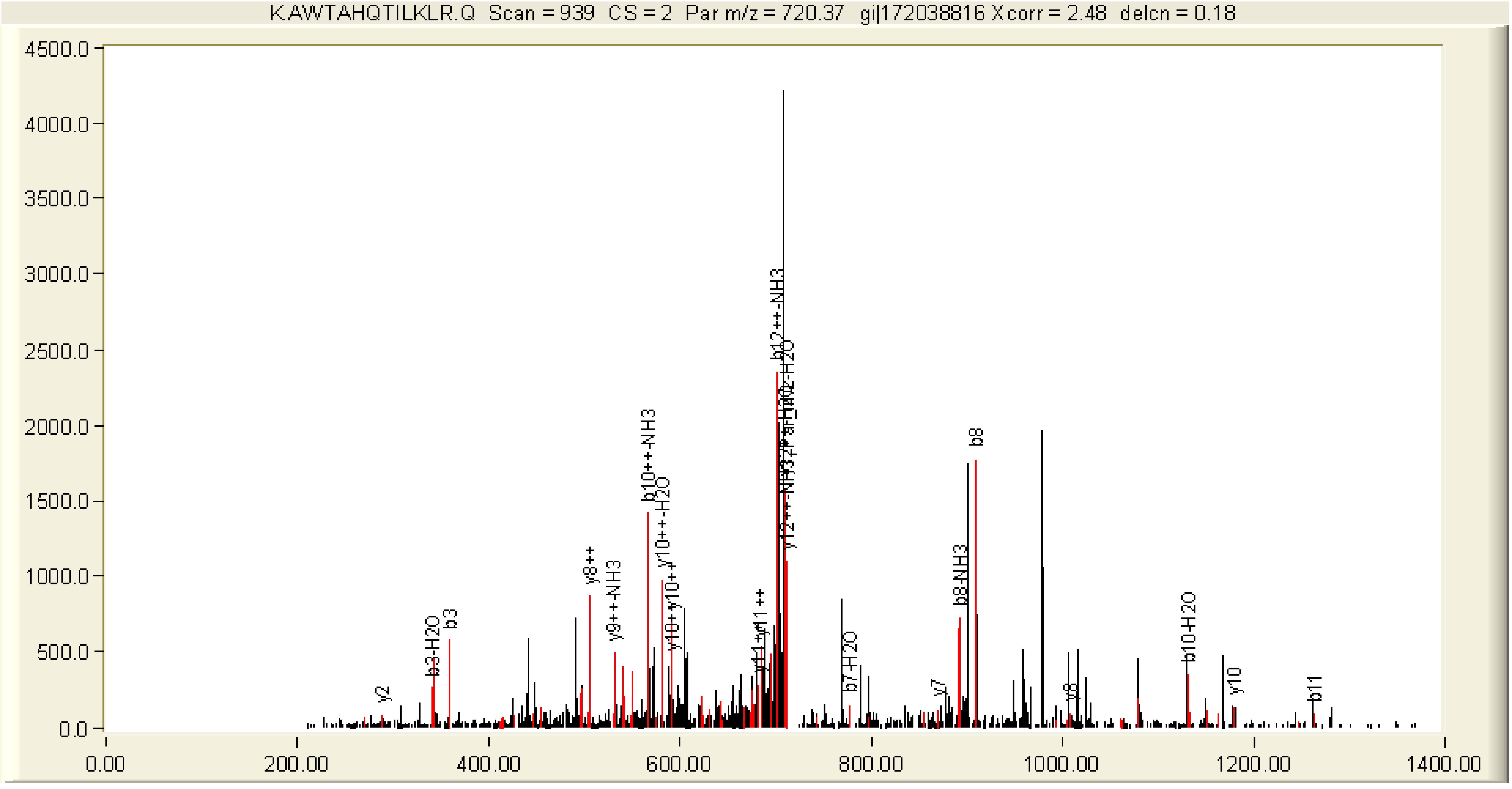

KAWTAHQTILKLR.Q Scan = 939 CS = 2 Par m/z = 720.37 gi|172038816 Xcorr = 2.48 delcn = 0.18
4500.0
4000.0
3500.0
3000.0
2500.0
2000.0
1500.0
1000.0
500.0
0.0
0.00
200.00
400.00
600.00
800.00
1000.00
1200.00
1400.00
y2
b3-H2O
b3
y8++
y9++-NH3
b10++-NH3
y10++-H2O
b12++-NH3
b8
b8-NH3
b7-H2O
y7
y8
b10-H2O
y10
b11

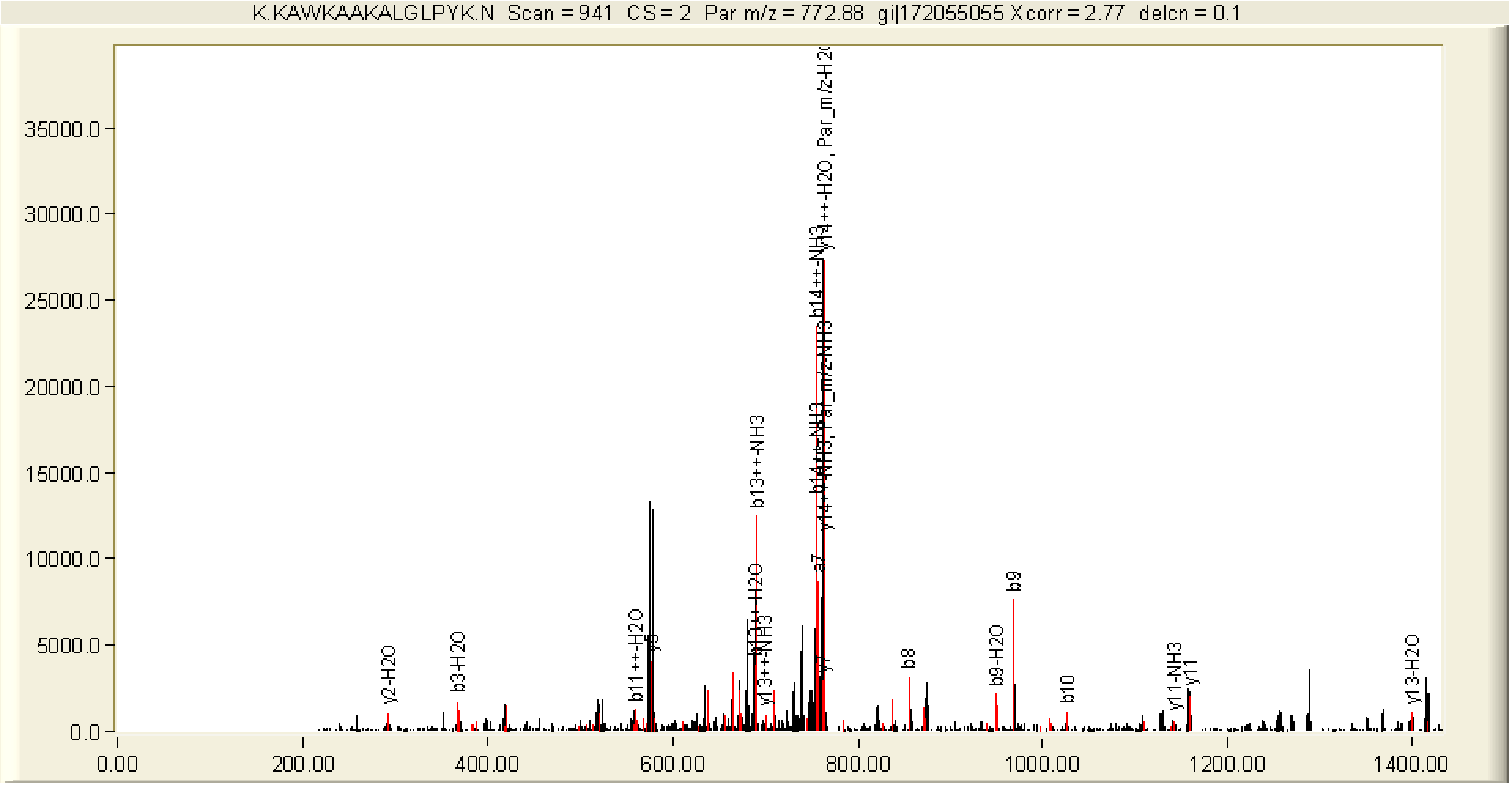
K.KAWKAAKALGLPYK.N Scan = 941 CS = 2 Par m/z = 772.88 gi|172055055 Xcorr = 2.77 delcn = 0.1
35000.0
30000.0
25000.0
20000.0
15000.0
10000.0
5000.0
0.0
0.00
200.00
400.00
600.00
800.00
1000.00
1200.00
1400.00
y2-H2O
b3-H2O
b11++-H2O
y5
b13++-NH3
b13++-H2O
y13++-NH3
a7
b14++-NH3
y14++-H2O, Par_m/z-H2O
b8
b9-H2O
b9
b10
y11-NH3
y11
y13-H2O

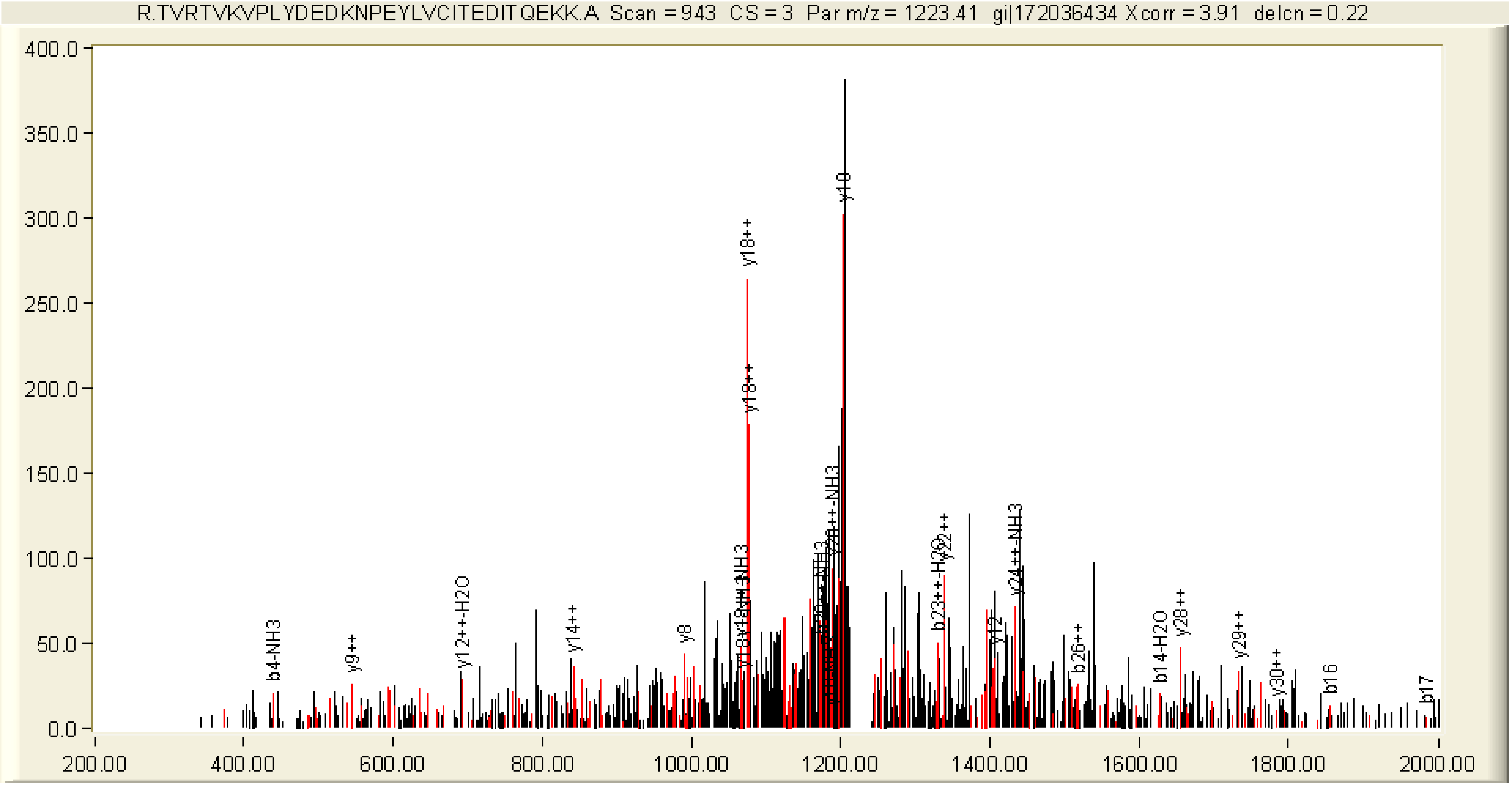
R.TVRTVKVPLYDEDKNPEYLVCITEDITQEKK.A Scan = 943 CS = 3 Par m/z = 1223.41 gi|172036434 Xcorr = 3.91 delcn = 0.22
b4-NH3
y9++
y12++-H2O
y14++
y8
y18++
y18++
y10
b23++-H2O
y22++
y12
y24++-NH3
b26++
b14-H2O
y28++
y29++
y30++
b16
b17
400.0
350.0
300.0
250.0
200.0
150.0
100.0
50.0
0.0
200.00
400.00
600.00
800.00
1000.00
1200.00
1400.00
1600.00
1800.00
2000.00

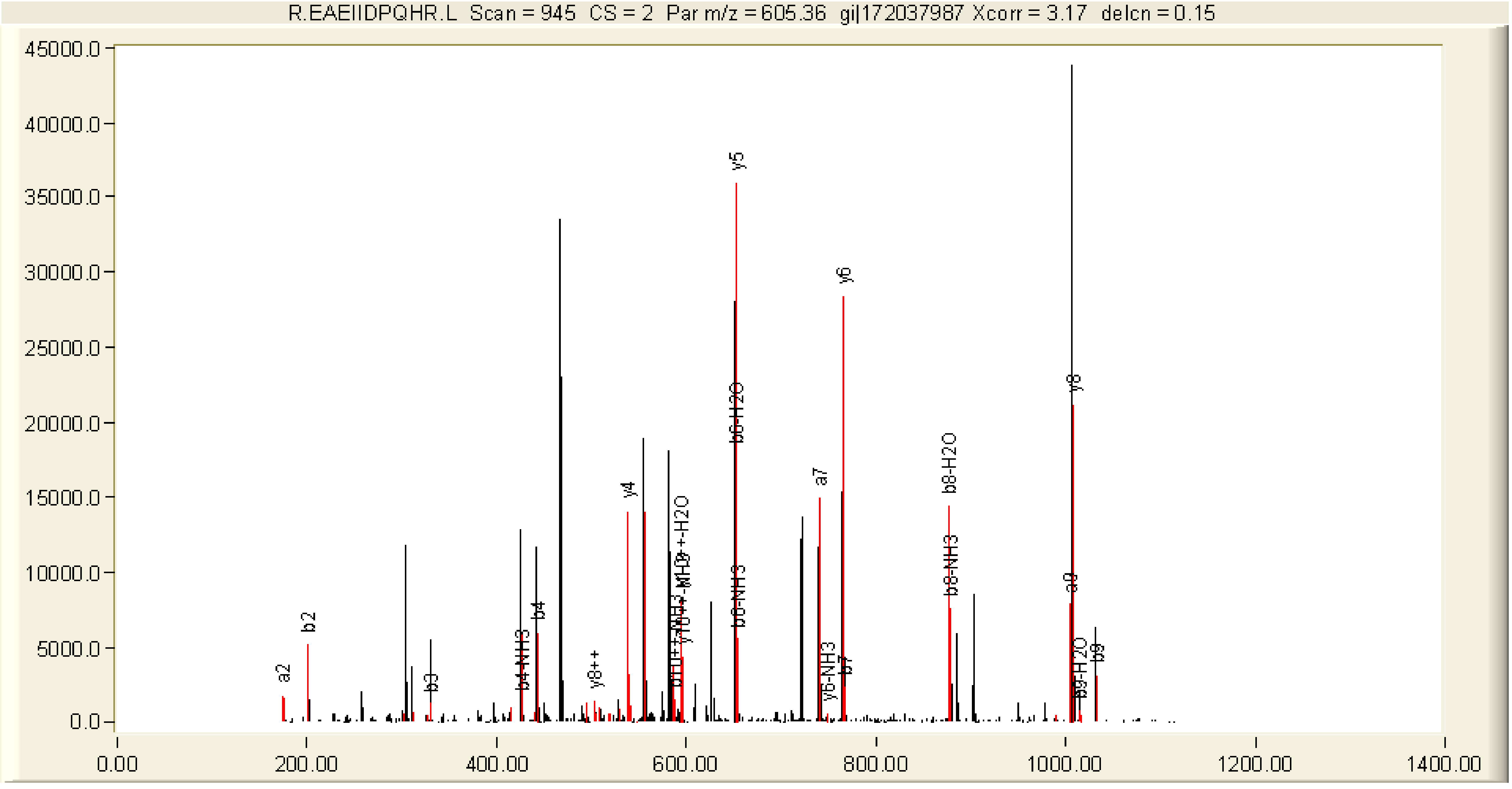
R.EAEIIDPQHR.L Scan = 945 CS = 2 Par m/z = 605.36 gi|172037987 Xcorr = 3.17 delcn = 0.15
45000.0
40000.0
35000.0
30000.0
25000.0
20000.0
15000.0
10000.0
5000.0
0.0
0.00
200.00
400.00
600.00
800.00
1000.00
1200.00
1400.00
a2
b2
b3
b4-NH3
b4
y8++
y4
y5
b6-H2O
b6-NH3
a7
y6-NH3
b7
y6
b8-H2O
b8-NH3
y8
a9
b9-H2O
b9

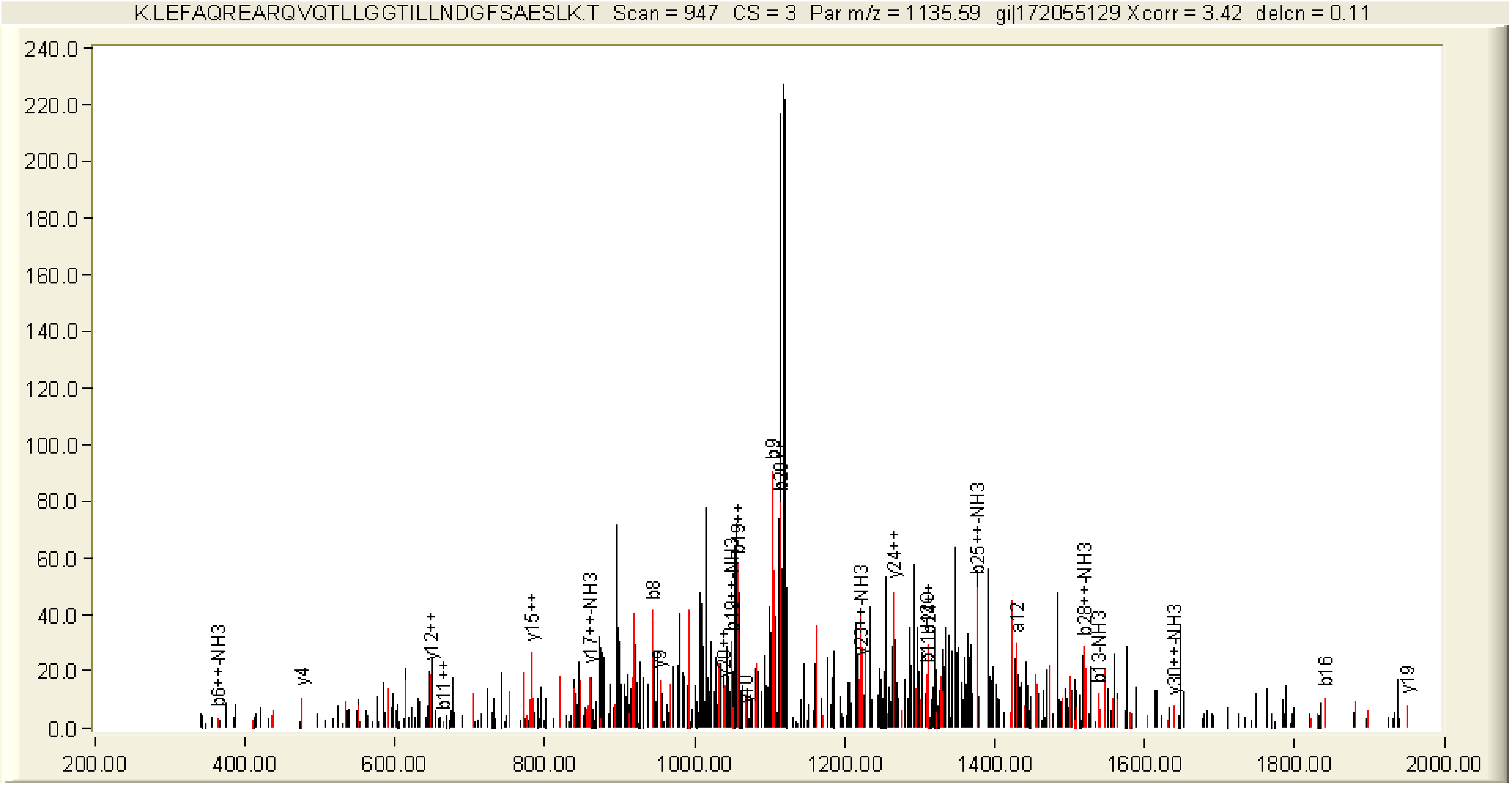
K.LEFAQREARQVQTLLGGTILLNDGFSAESLK.T Scan = 947 CS = 3 Par m/z = 1135.59 gi|172055129 Xcorr = 3.42 delcn = 0.11
240.0
220.0
200.0
180.0
160.0
140.0
120.0
100.0
80.0
60.0
40.0
20.0
0.0
200.00
400.00
600.00
800.00
1000.00
1200.00
1400.00
1600.00
1800.00
2000.00
b6++-NH3
y4
y12++
b11++
y15++
y17++-NH3
b8
b19++
b25++-NH3
y24++
a12
b28++-NH3
y30++-NH3
b16
y19

R.ARTILWTAGMKASPLANILAERAEAKL Scan = 949 CS = 3 Par m/z = 929.05 gi|172039016 Xcorr = 3.2 delcn = 0.13

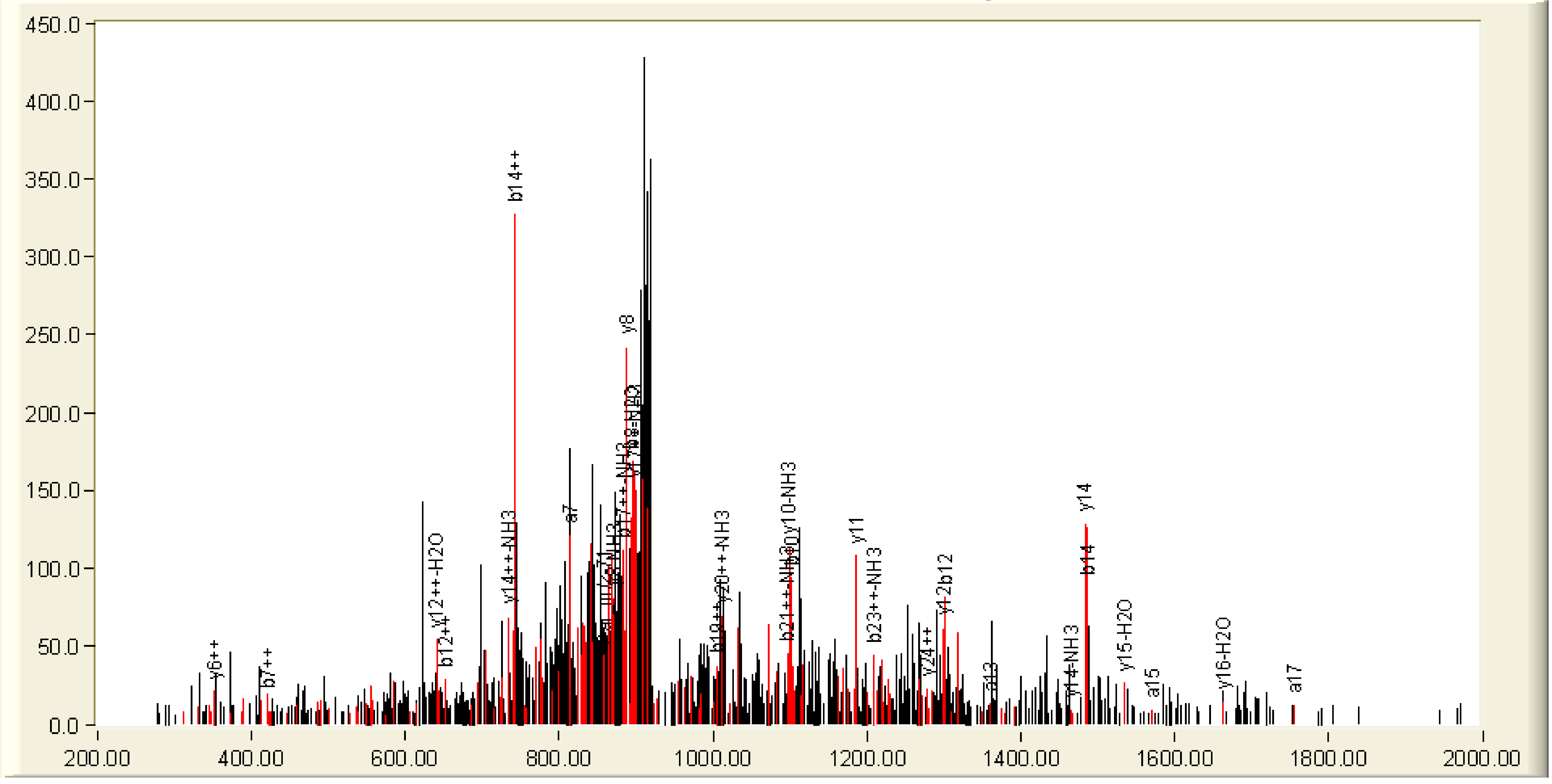

K.VGIDQVVAGELDILIGPVSITAER.F Scan = 951 CS = 2 Par m/z = 1233.46 gi|172037324 Xcorr = 3.1 delcn = 0.28

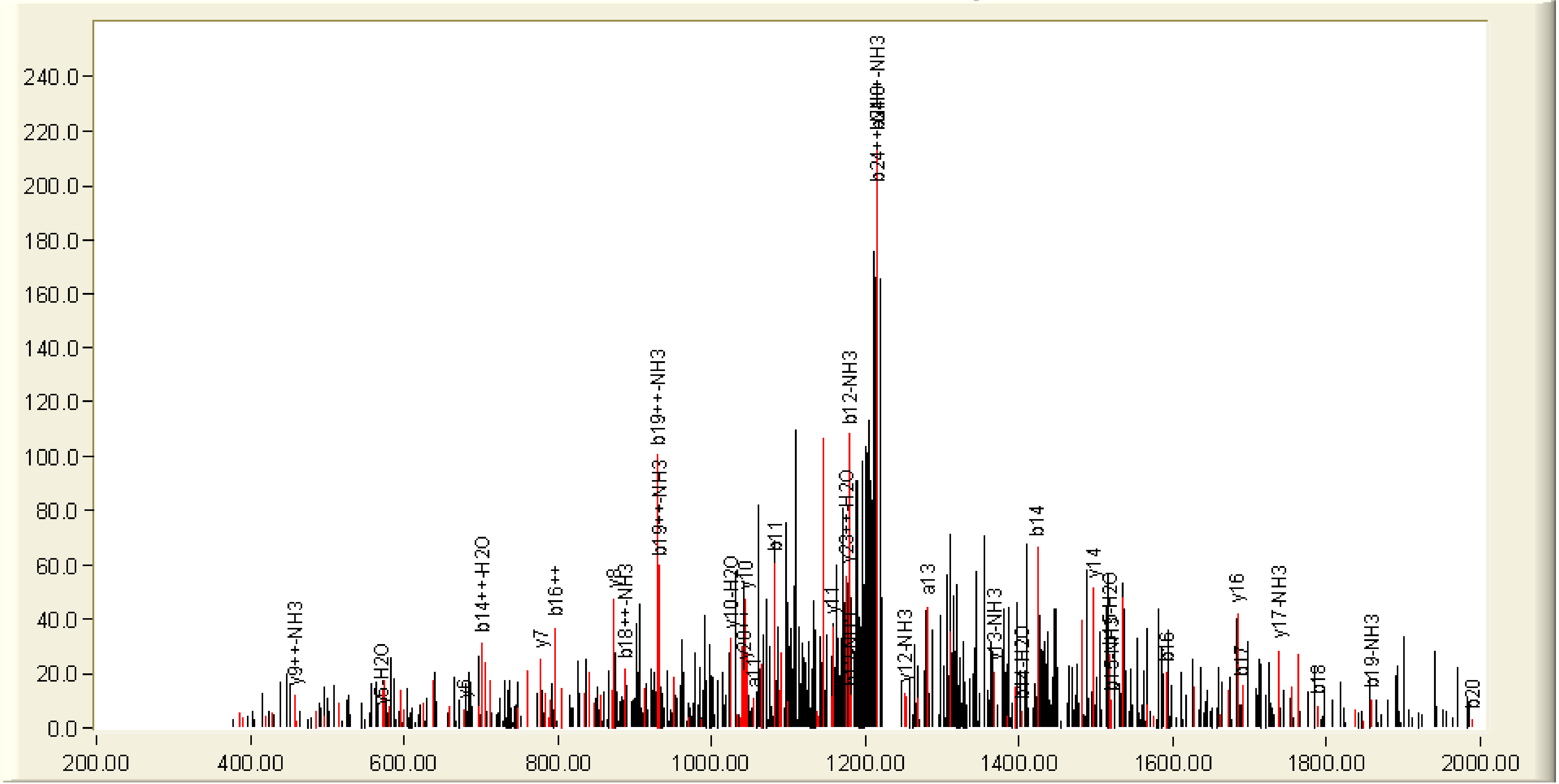

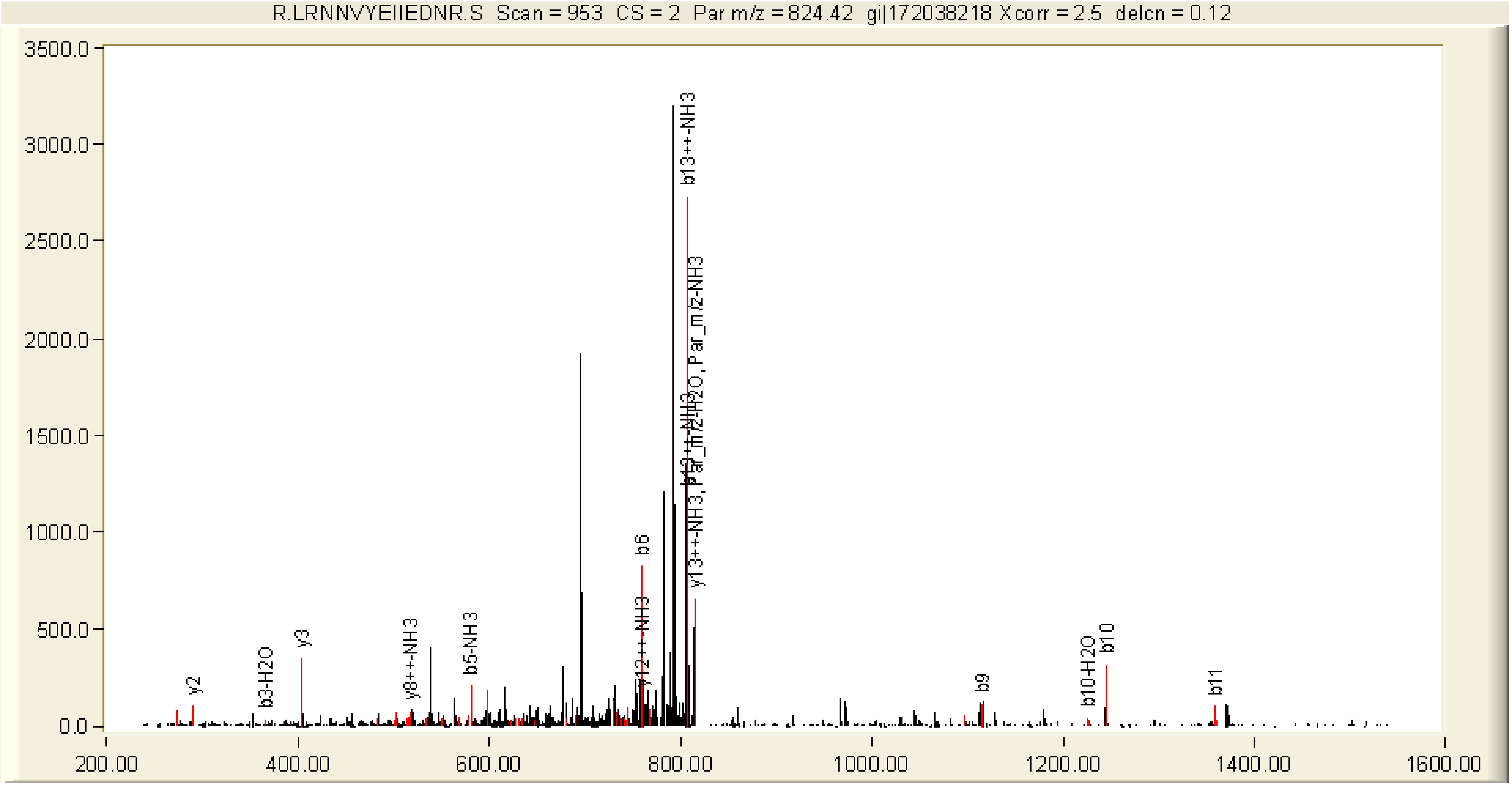
R.LRNNVYEIIEDNR.S Scan = 953 CS = 2 Par m/z = 824.42 gi|172038218 Xcorr = 2.5 delcn = 0.12
3500.0
3000.0
2500.0
2000.0
1500.0
1000.0
500.0
0.0
200.00
400.00
600.00
800.00
1000.00
1200.00
1400.00
1600.00
y2
b3-H2O
y3
y8++-NH3
b5-NH3
y12++-NH3
b6
b13++-NH3
y13++-NH3, Par_m/z-H2O, Par_m/z-NH3
b9
b10-H2O
b10
b11

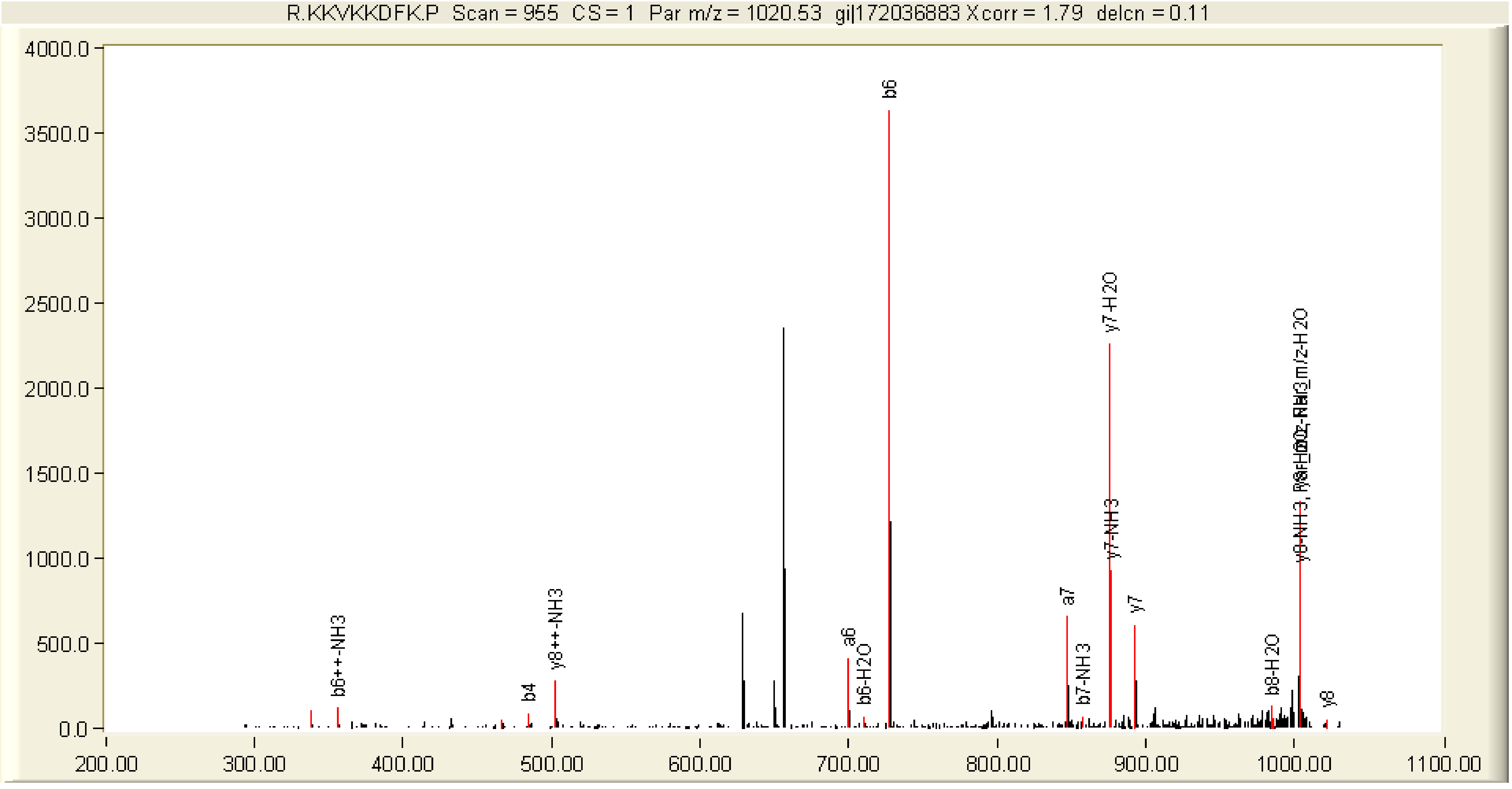

R.KKVKKDFK.P Scan = 955 CS = 1 Par m/z = 1020.53 gi|172036883 Xcorr = 1.79 delcn = 0.11
4000.0
3500.0
3000.0
2500.0
2000.0
1500.0
1000.0
500.0
0.0
200.00
300.00
400.00
500.00
600.00
700.00
800.00
900.00
1000.00
1100.00
b6++-NH3
b4
y8++-NH3
a6
b6-H2O
b6
a7
b7-NH3
y7-H2O
y7-NH3
y7
b8-H2O
y8-NH3
y8

R.TLAVGDIHGCTTAFDQLLEAIDLRPEDK.L Scan = 957 CS = 3 Par m/z = 1015.88 gi|172055283 Xcorr = 3.53 delcn = 0.16

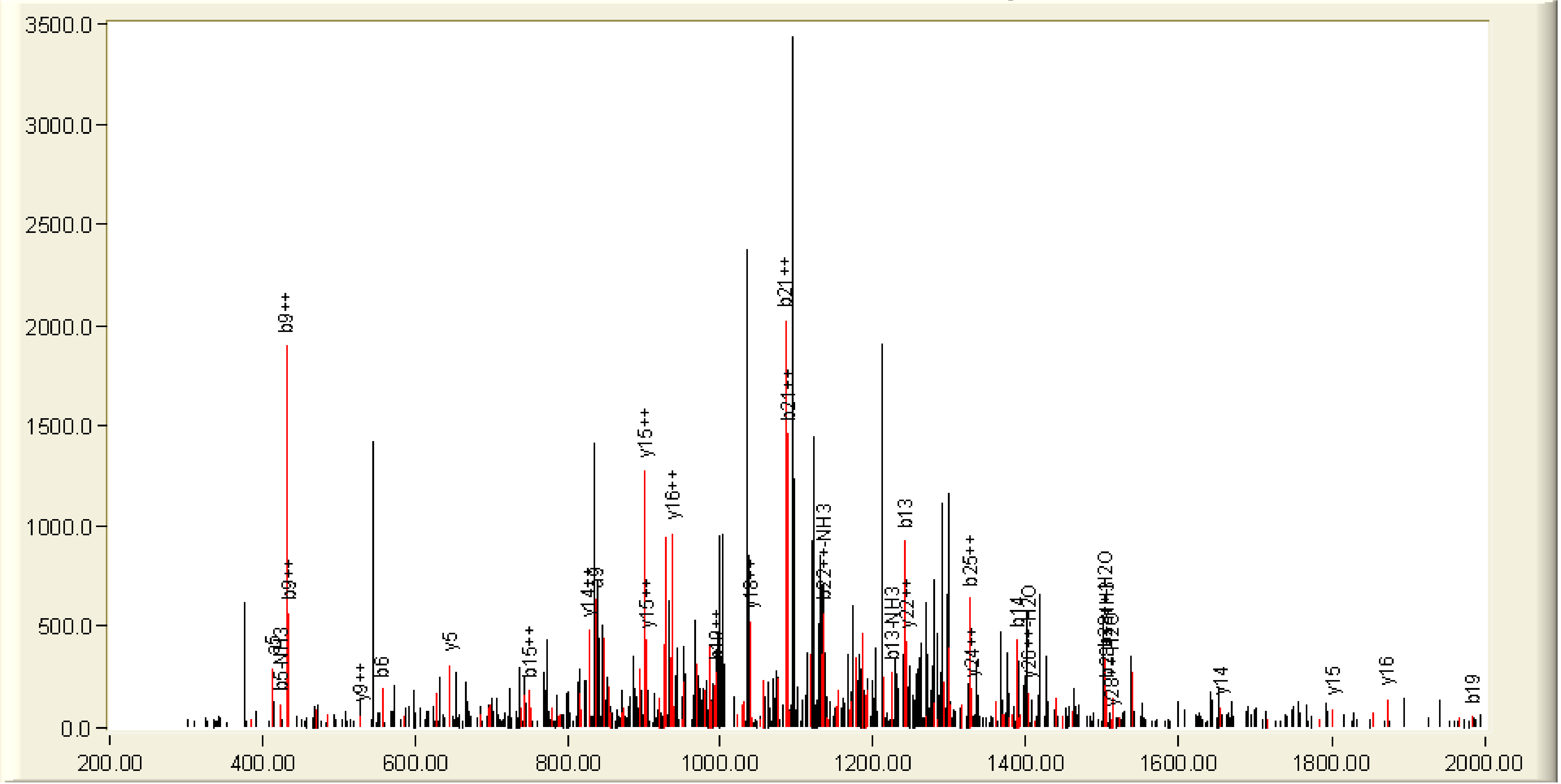

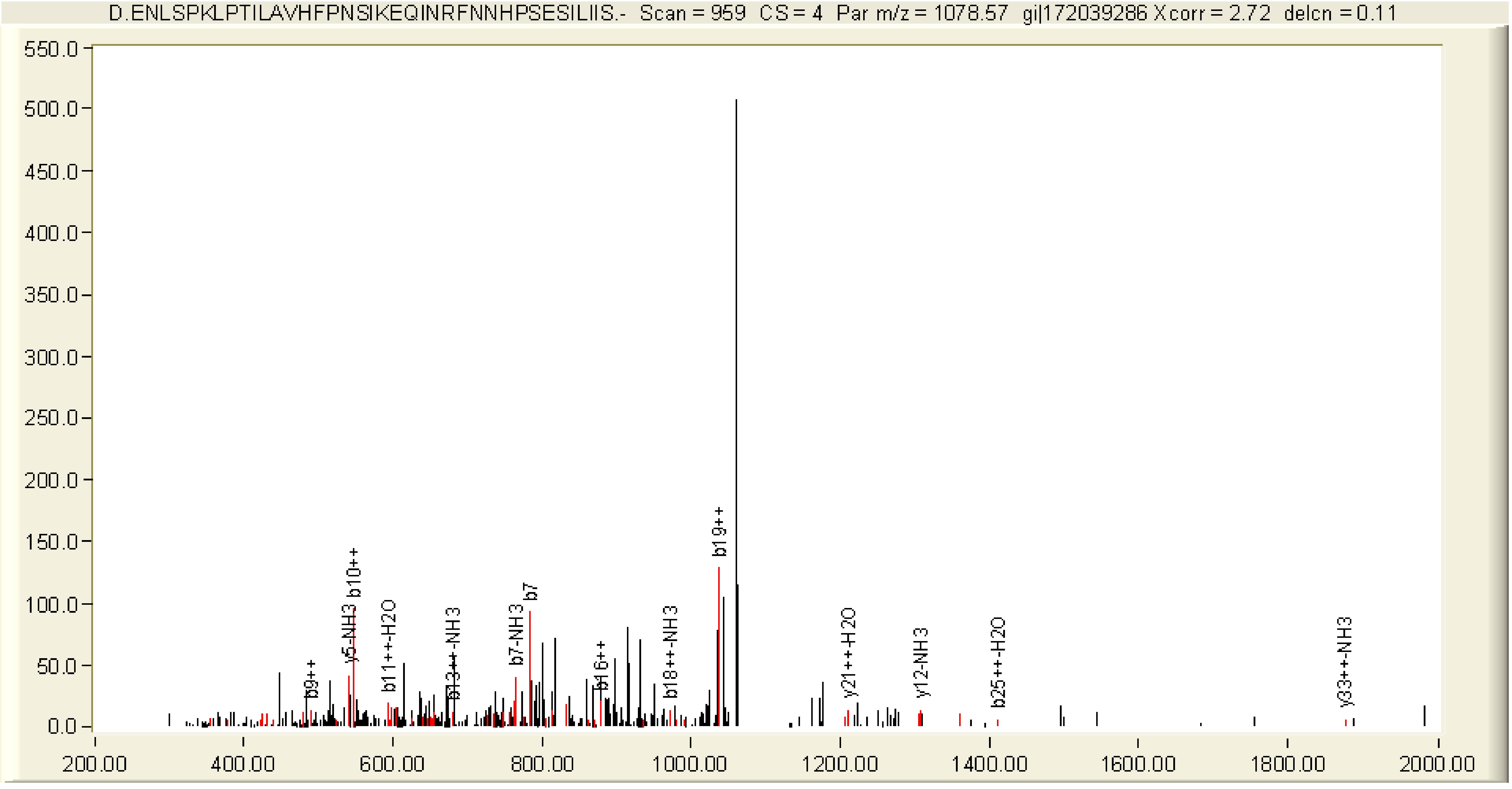
D.ENLSPKLPTILAVHFPNSIKEQINRFNNHPSESILIIS.- Scan = 959 CS = 4 Par m/z = 1078.57 gi|172039286 Xcorr = 2.72 delcn = 0.11
550.0
500.0
450.0
400.0
350.0
300.0
250.0
200.0
150.0
100.0
50.0
0.0
200.00
400.00
600.00
800.00
1000.00
1200.00
1400.00
1600.00
1800.00
2000.00
b9++
y5-NH3
b10++
b11++-H2O
b13++-NH3
b7-NH3
b7
b16++
b18++-NH3
b19++
y21++-H2O
y12-NH3
b25++-H2O
y33++-NH3

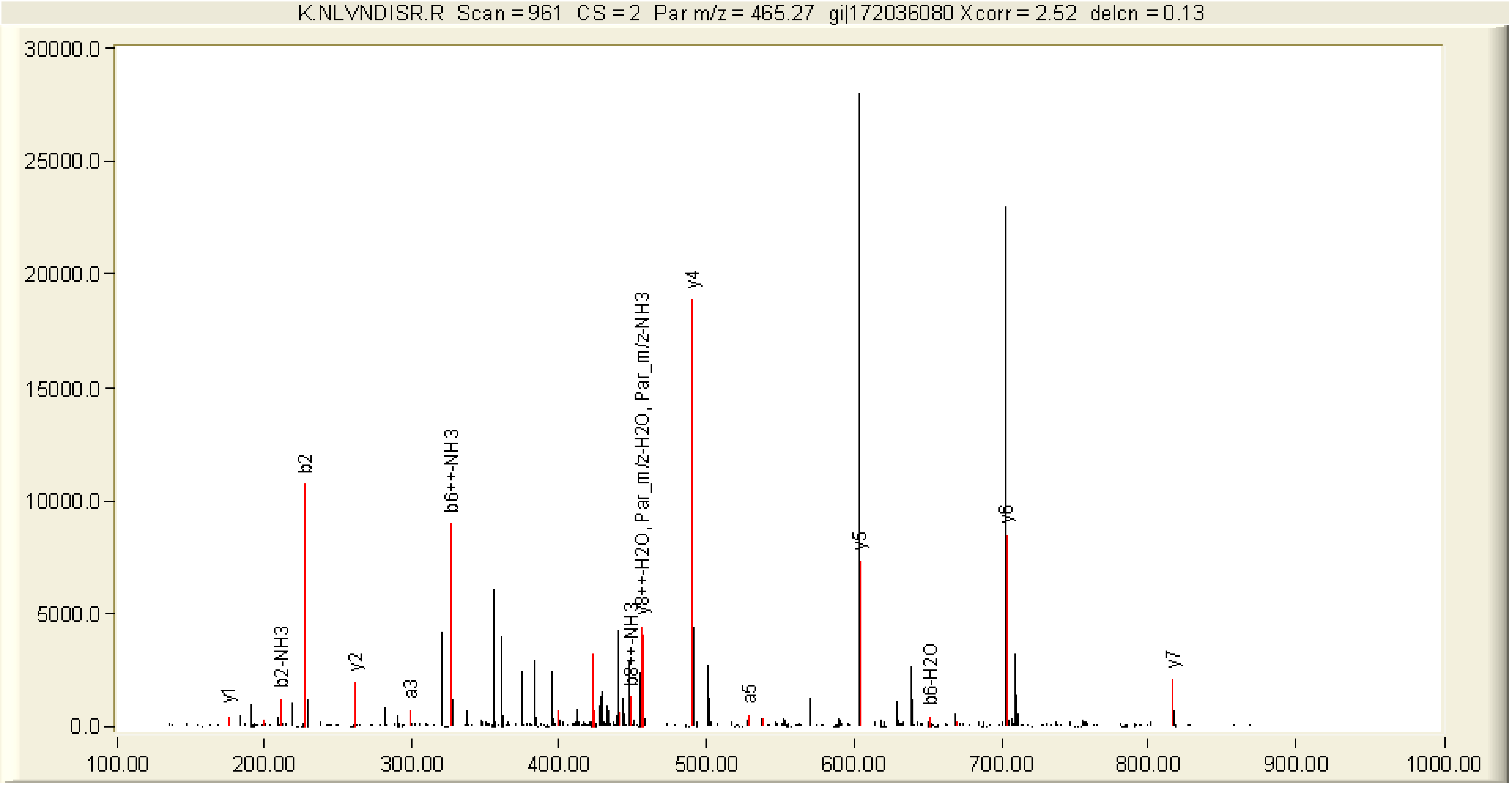
K.NLVNDISR.R Scan = 961 CS = 2 Par m/z = 465.27 gi|172036080 Xcorr = 2.52 delcn = 0.13
30000.0
25000.0
20000.0
15000.0
10000.0
5000.0
0.0
100.00
200.00
300.00
400.00
500.00
600.00
700.00
800.00
900.00
1000.00
y1
b2-NH3
b2
y2
a3
b6++-NH3
b8++-NH3
y8++-H2O, Par_m/z-H2O, Par_m/z-NH3
y4
a5
y5
b6-H2O
y6
y7

R.DTFGVTDKDILDAIR.N Scan = 963 CS = 2 Par m/z = 841.32 gi|172036902 Xcorr = 2.57 delcn = 0.27

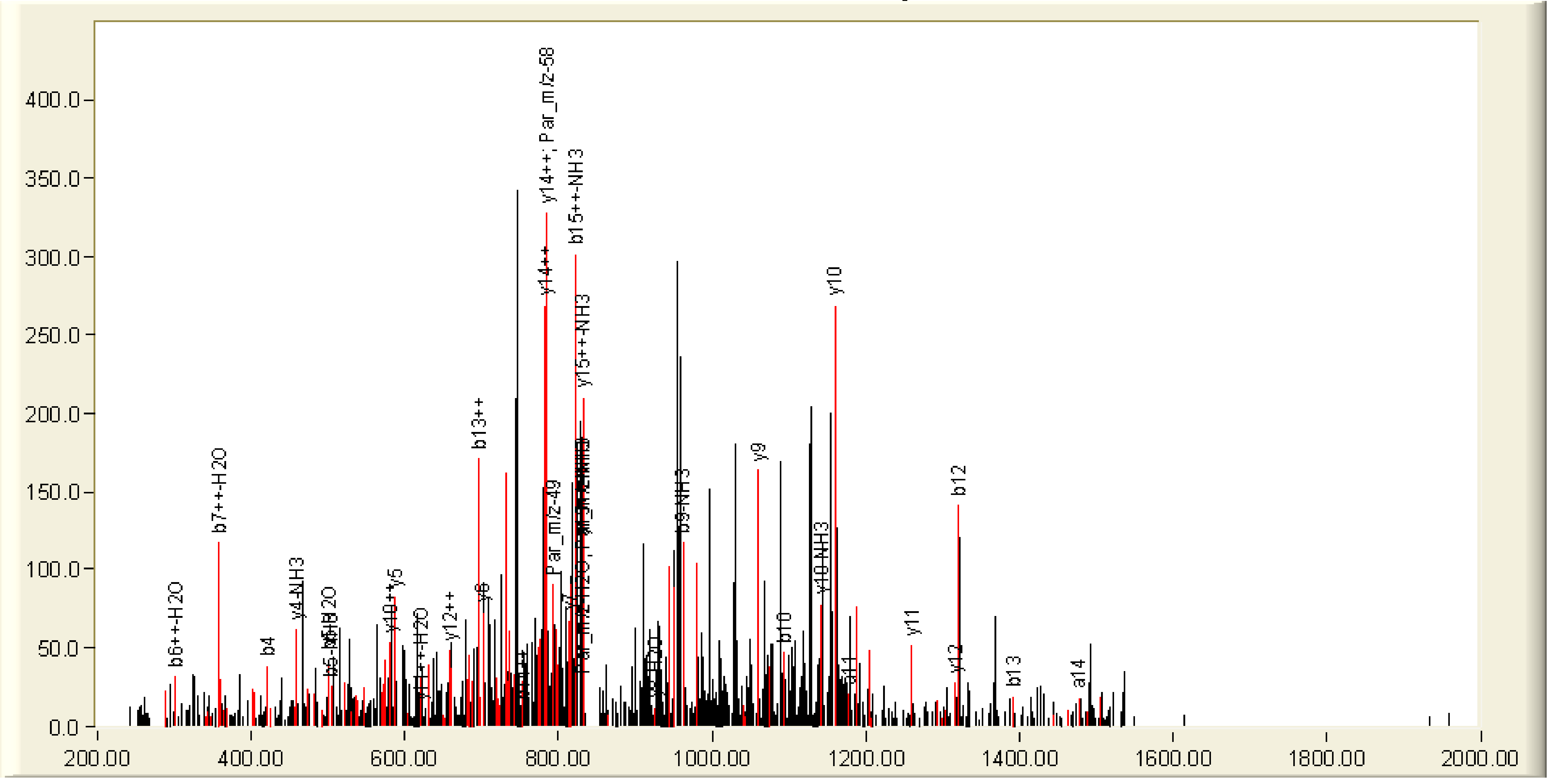

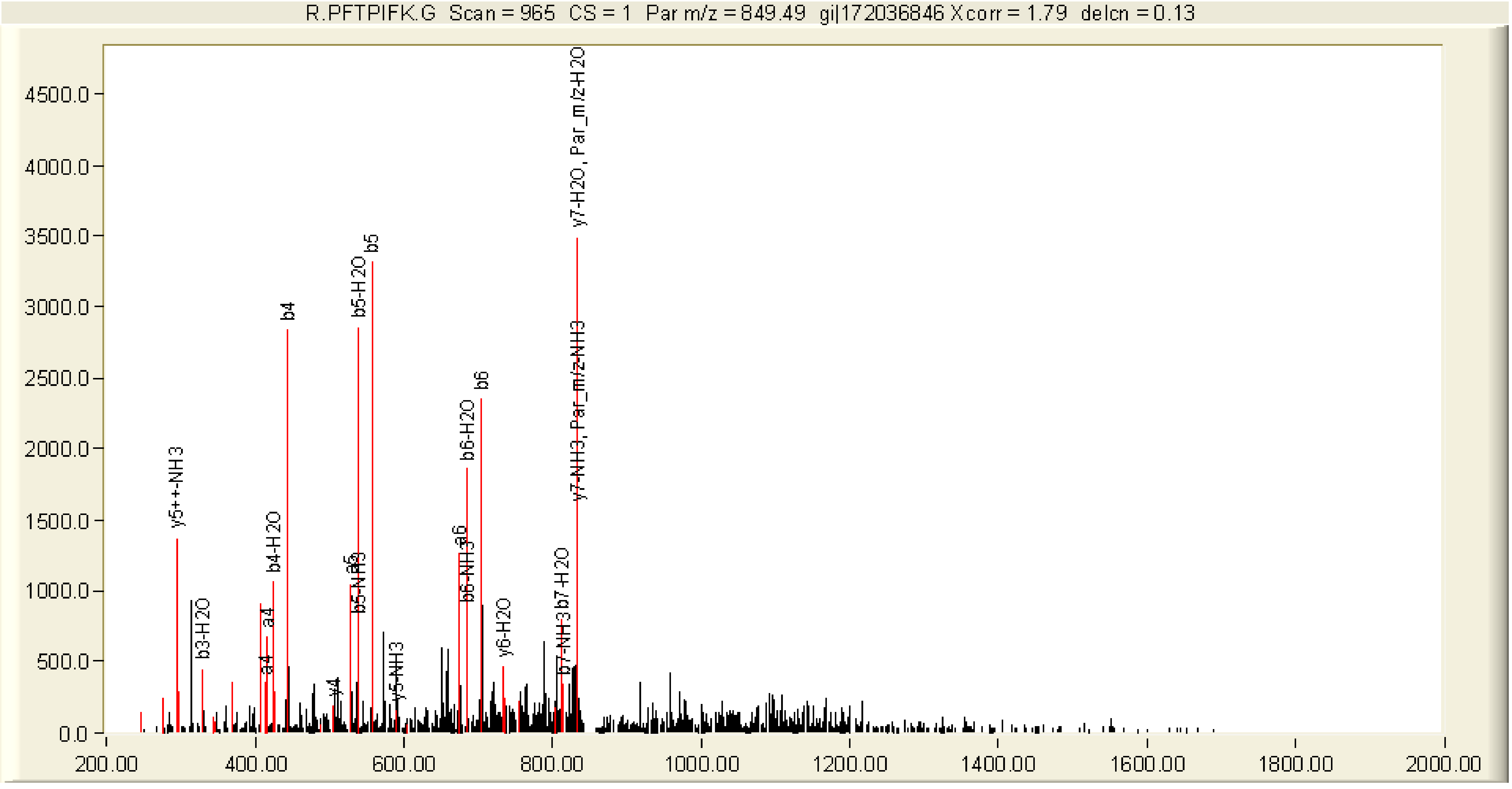

R.PFTPIFK.G Scan = 965 CS = 1 Par m/z = 849.49 gi|172036846 Xcorr = 1.79 delcn = 0.13
4500.0
4000.0
3500.0
3000.0
2500.0
2000.0
1500.0
1000.0
500.0
0.0
200.00
400.00
600.00
800.00
1000.00
1200.00
1400.00
1600.00
1800.00
2000.00
y5++-NH3
b3-H2O
a4
a4
b4-H2O
b4
y4
b5-NH3
a5
b5-H2O
b5
y5-NH3
b6-NH3
a6
b6-H2O
b6
y6-H2O
b7-NH3
b7-H2O
y7-NH3, Par_m/z-NH3
y7-H2O, Par_m/z-H2O

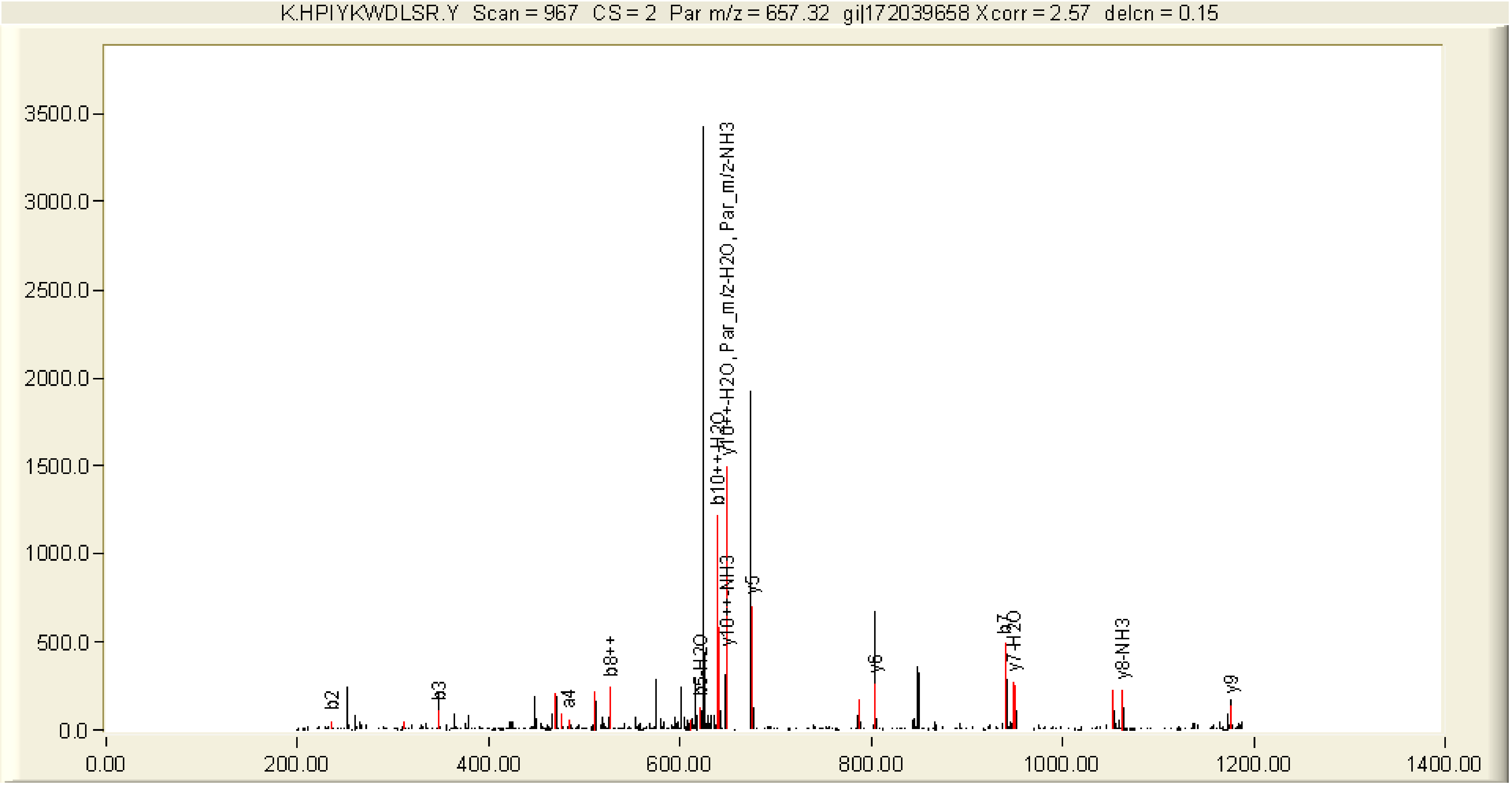

K.HPIYKWDLSR.Y Scan = 967 CS = 2 Par m/z = 657.32 gi|172039658 Xcorr = 2.57 delcn = 0.15
3500.0
3000.0
2500.0
2000.0
1500.0
1000.0
500.0
0.0
0.00
200.00
400.00
600.00
800.00
1000.00
1200.00
1400.00
b2
b3
a4
b8++
b5-H2O
b10++-H2O
y10++-H2O, Par_m/z-H2O, Par_m/z-NH3
y10++-NH3
y5
y6
b7
y7-H2O
y8-NH3
y9

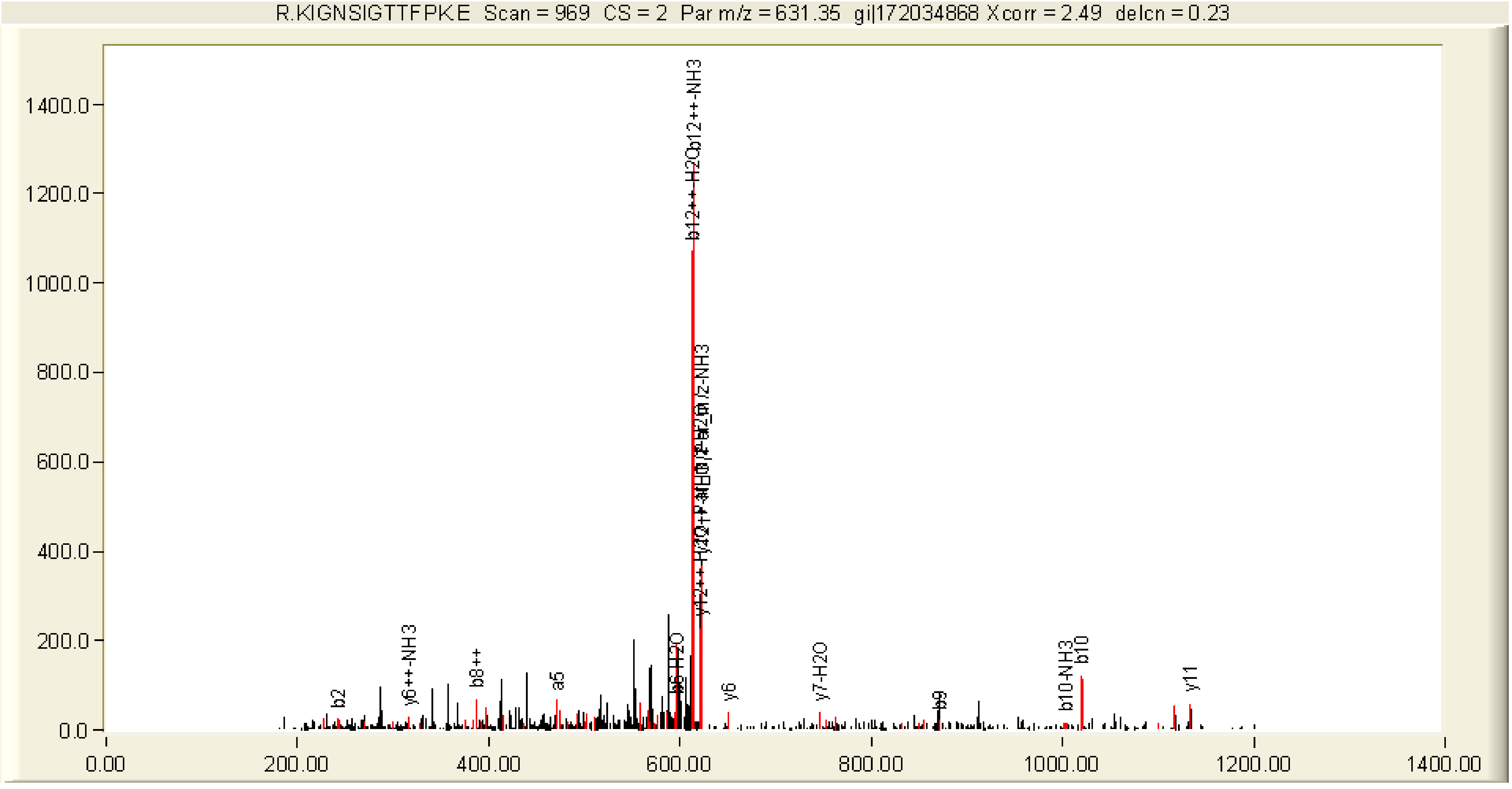
R.KIGNSIGTTFPKE Scan = 969 CS = 2 Par m/z = 631.35 gi|172034868 Xcorr = 2.49 delcn = 0.23
1400.0
1200.0
1000.0
800.0
600.0
400.0
200.0
0.0
0.00
200.00
400.00
600.00
800.00
1000.00
1200.00
1400.00
b2
y6++-NH3
b8++
a5
b5-H2O
b12++-NH3
y6
y7-H2O
b9
b10-NH3
b10
y11

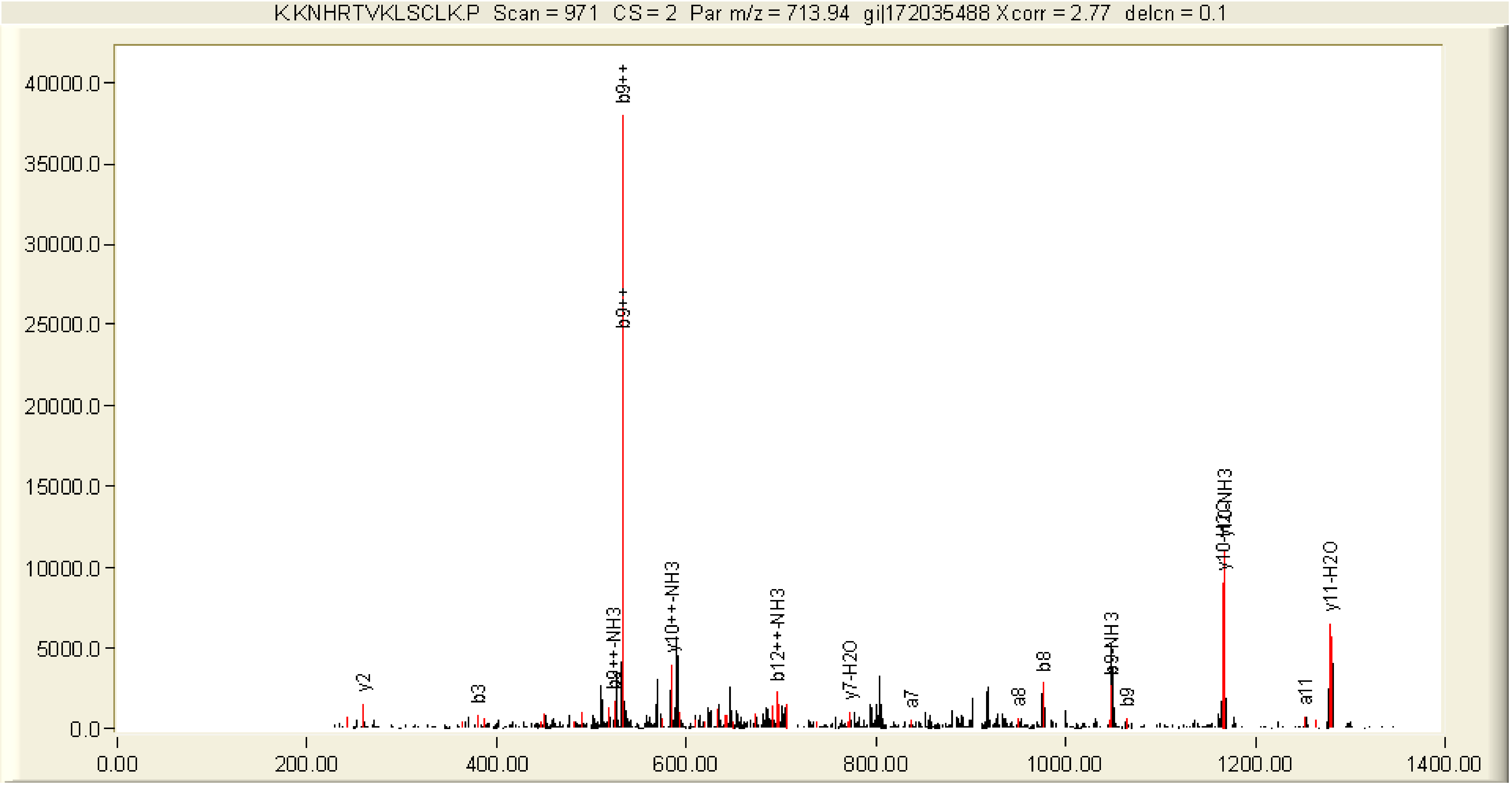
K.KNHRTVKLSCLK.P Scan = 971 CS = 2 Par m/z = 713.94 gi|172035488 Xcorr = 2.77 delcn = 0.1
b9++
b9++
y2
b3
b9++-NH3
y10++-NH3
b12++-NH3
y7-H2O
a7
a8
b8
b9-NH3
b9
y10-H2O
y11-H2O
a11
40000.0
35000.0
30000.0
25000.0
20000.0
15000.0
10000.0
5000.0
0.0
0.00
200.00
400.00
600.00
800.00
1000.00
1200.00
1400.00

R.SLKPSLLEKIATR.D Scan = 973 CS = 2 Par m/z = 728.4 gi|172038709 Xcorr = 3.05 delcn = 0.18

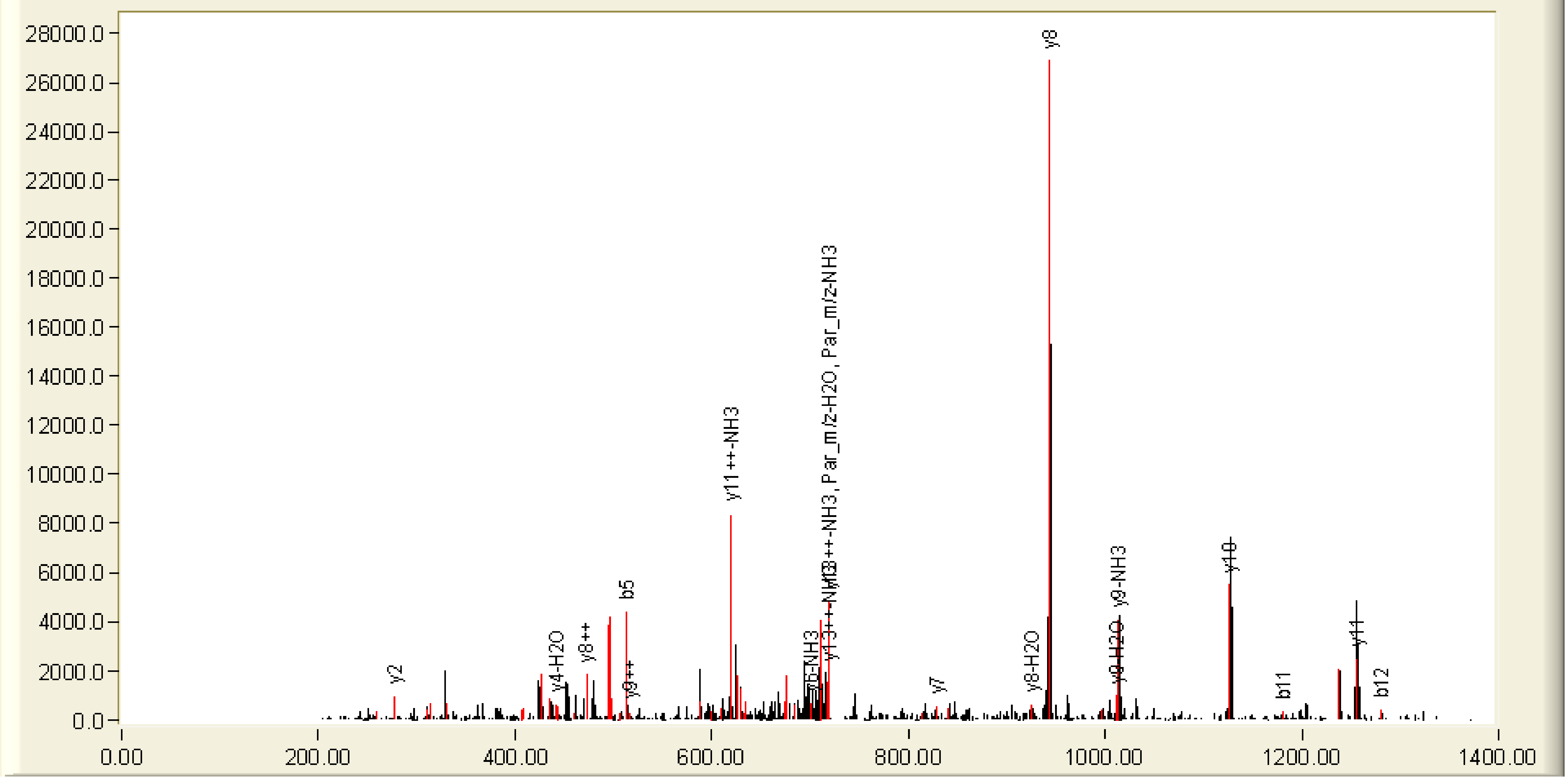

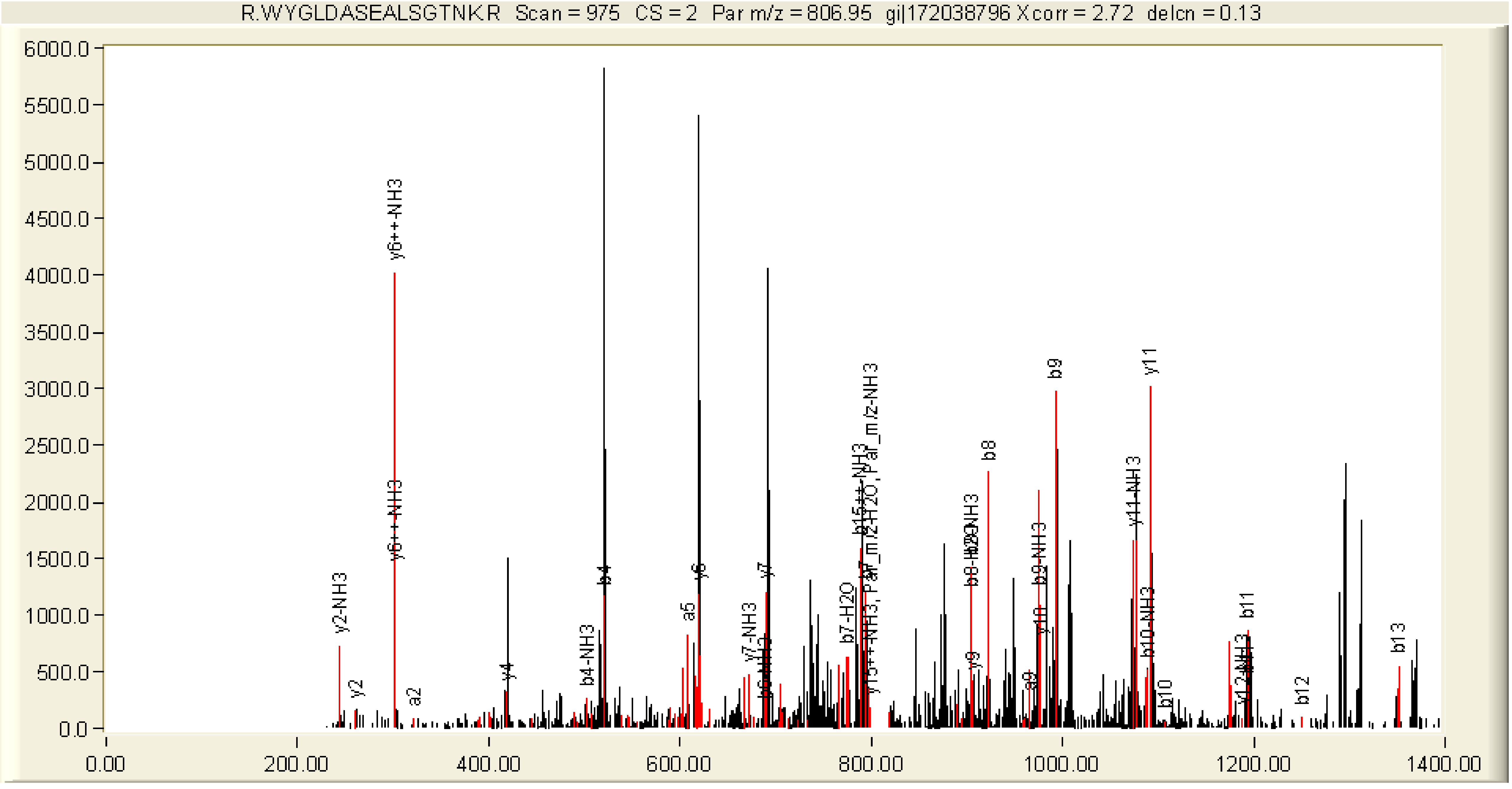
R.WYGLDASEALSGTNKR Scan = 975 CS = 2 Par m/z = 806.95 gi|172038796 Xcorr = 2.72 delcn = 0.13
6000.0
5500.0
5000.0
4500.0
4000.0
3500.0
3000.0
2500.0
2000.0
1500.0
1000.0
500.0
0.0
0.00
200.00
400.00
600.00
800.00
1000.00
1200.00
1400.00
y6++-NH3
y2-NH3
y2
a2
y4
b4-NH3
b4
a5
y6
y7-NH3
y7
b7-H2O
b8
b9
y11
b9-NH3
y10
y9
a9
y11-NH3
b10-NH3
b10
b11
b12
b13

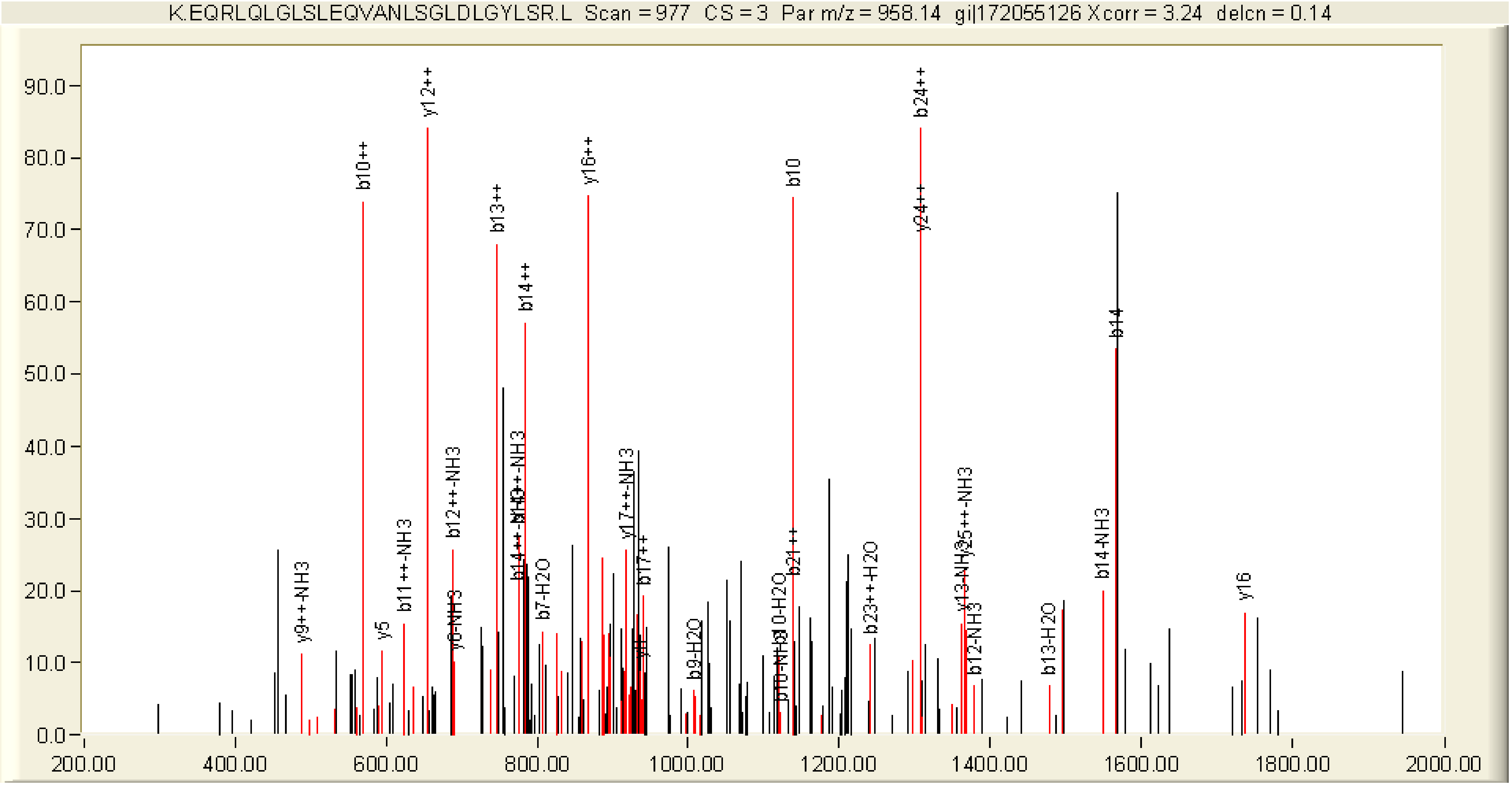
K.EQRLQLGLSLEQVANLSGLDLGYLSR.L Scan = 977 CS = 3 Par m/z = 958.14 gi|172055126 Xcorr = 3.24 delcn = 0.14
90.0
80.0
70.0
60.0
50.0
40.0
30.0
20.0
10.0
0.0
200.00
400.00
600.00
800.00
1000.00
1200.00
1400.00
1600.00
1800.00
2000.00
y9++-NH3
b10++
y5
b11++-NH3
y12++
b12++-NH3
b13++
b14++
b7-H2O
y16++
y17++-NH3
b17++
b9-H2O
b10-H2O
b21++
b10
b23++-H2O
b24++
y24++
y25++-NH3
b12-NH3
b13-H2O
b14-NH3
b14
y16

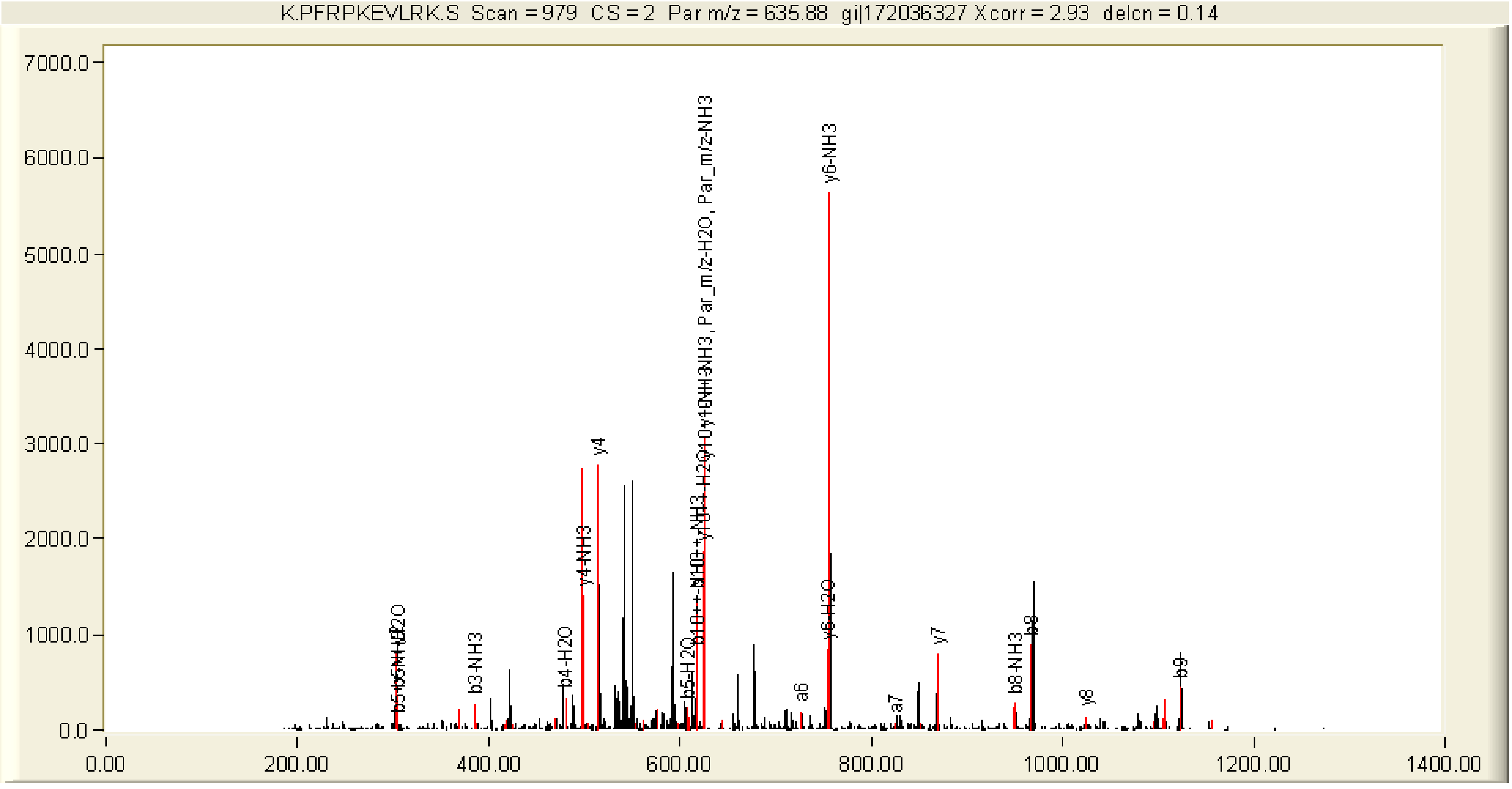

K.PFRPKEVLRK.S Scan = 979 CS = 2 Par m/z = 635.88 gi|172036327 Xcorr = 2.93 delcn = 0.14
7000.0
6000.0
5000.0
4000.0
3000.0
2000.0
1000.0
0.0
0.00
200.00
400.00
600.00
800.00
1000.00
1200.00
1400.00
y6-NH3
y4
y4-NH3
b3-NH3
b4-H2O
b5-H2O
y6-H2O
a6
a7
y7
b8-NH3
b8
y8
b9

R.AGETPMVYWNGQAIK.W Scan = 981 CS = 2 Par m/z = 833.47 gi|172038163 Xcorr = 2.95 delcn = 0.1

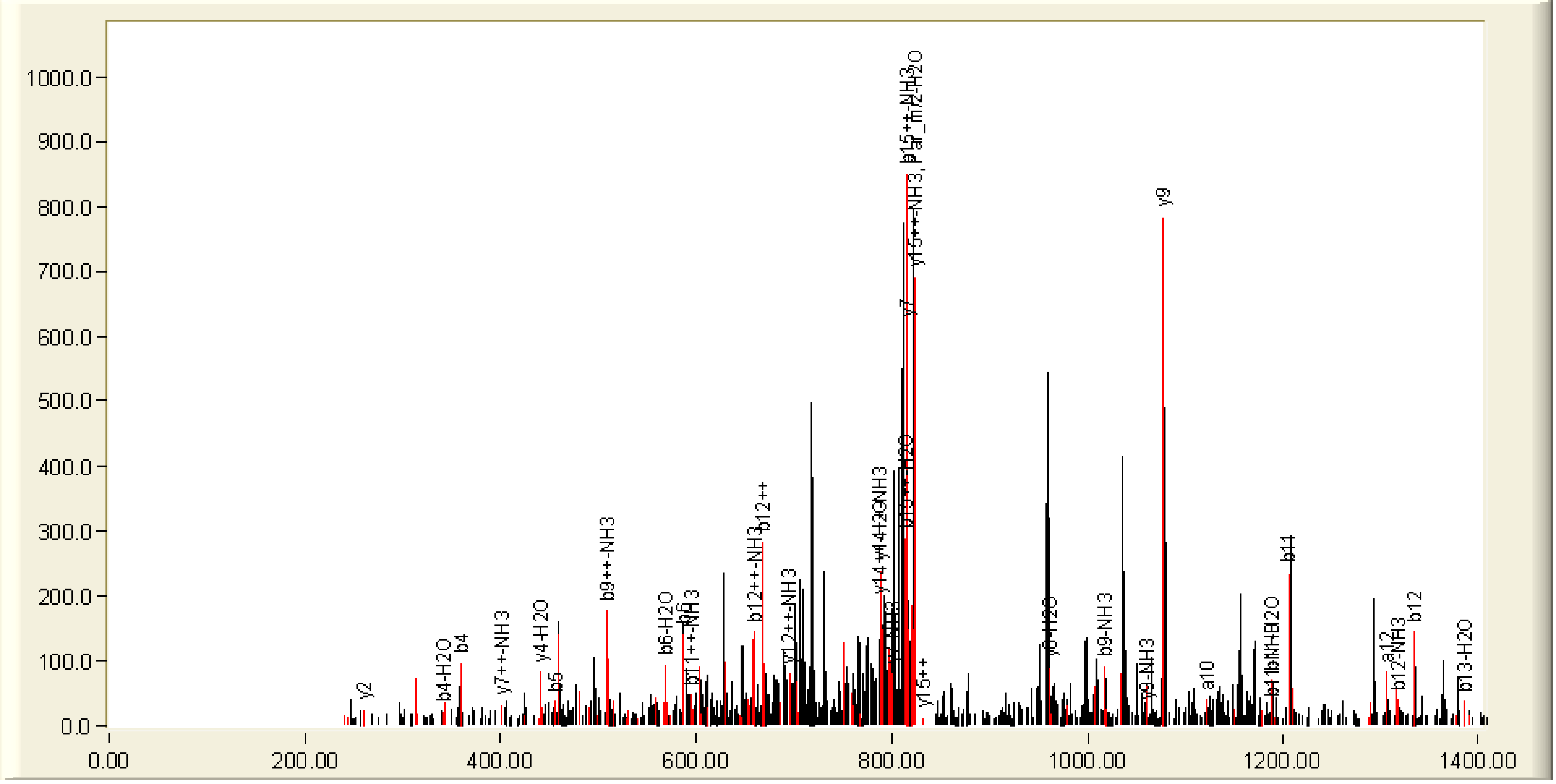

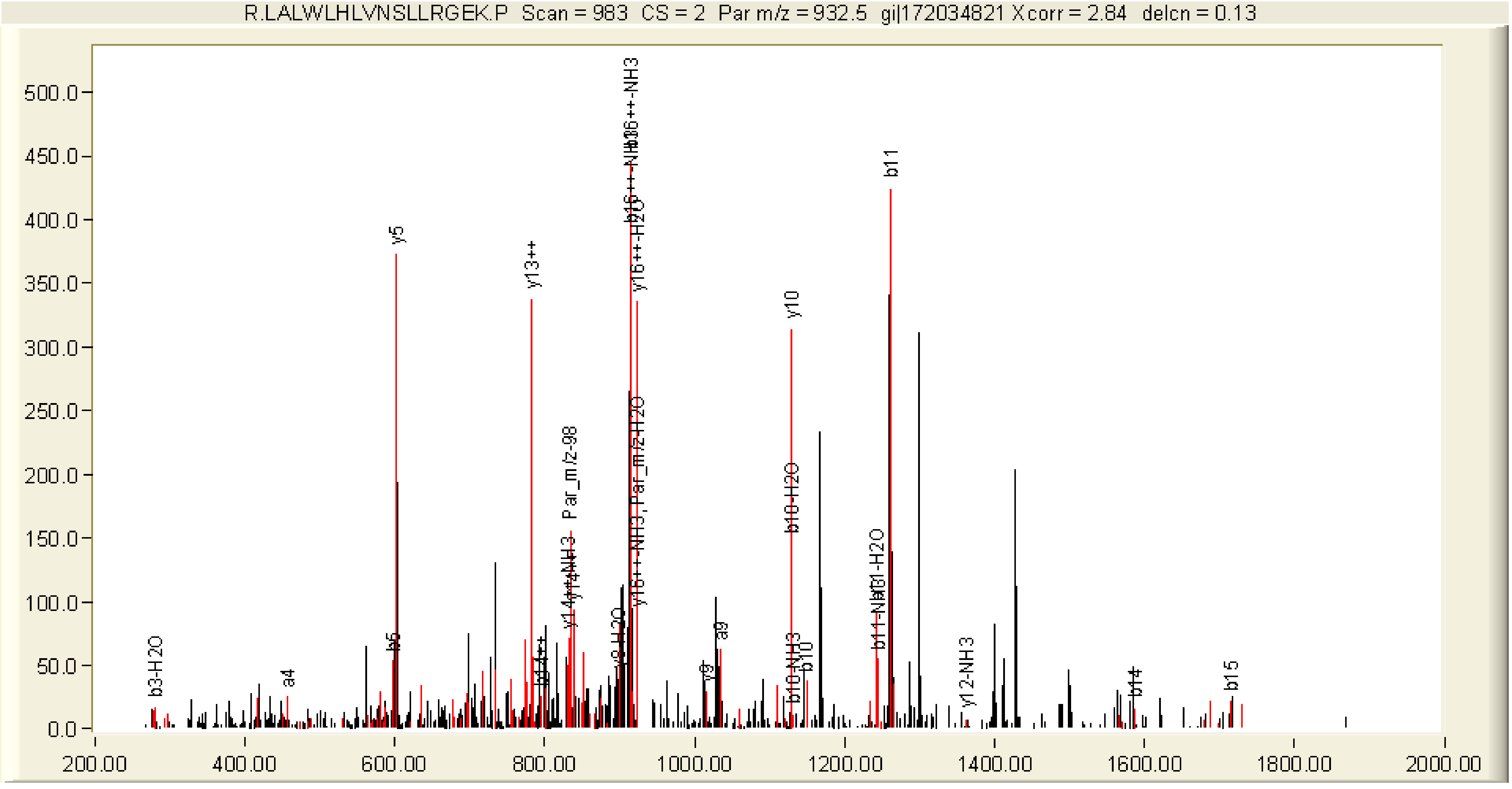
R.LALWLHLVNSLLRGEK.P Scan = 983 CS = 2 Par m/z = 932.5 gi|172034821 Xcorr = 2.84 delcn = 0.13
500.0
450.0
400.0
350.0
300.0
250.0
200.0
150.0
100.0
50.0
0.0
200.00
400.00
600.00
800.00
1000.00
1200.00
1400.00
1600.00
1800.00
2000.00
b3-H2O
a4
b5
y5
y13++
Par_m/z-98
y16++-NH3
y16++-H2O
y16++-NH3, Par_m/z-H2O
y10
b10-H2O
b10-NH3
b10
a9
b11
b11-H2O
y12-NH3
b14
b15

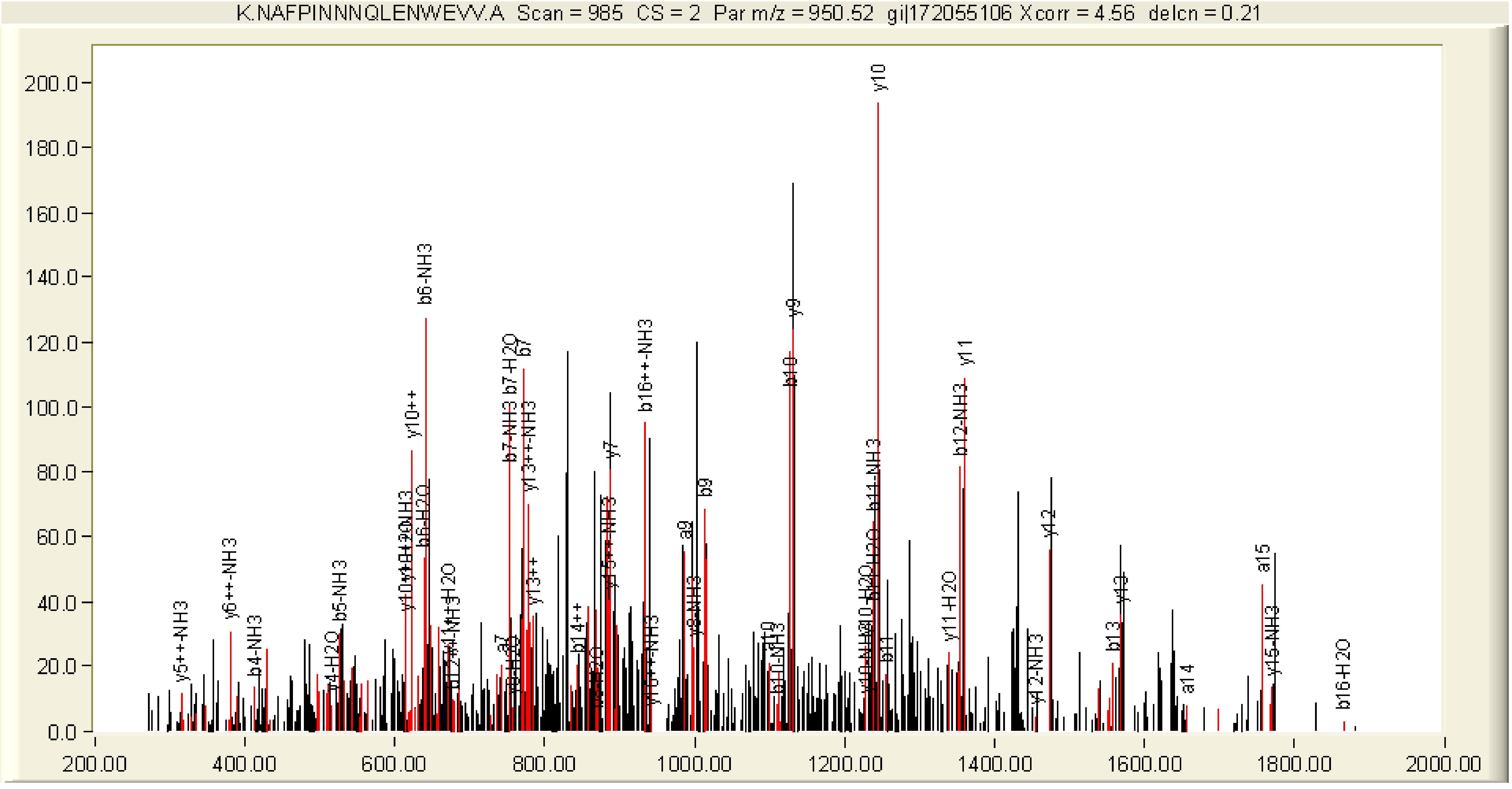

K.NAFPINNNQLENWEVV.A Scan = 985 CS = 2 Par m/z = 950.52 gi|172055106 Xcorr = 4.56 delcn = 0.21
y10
y9
b10
b6-NH3
b7-H2O
b7
b7-NH3
y13++-NH3
y10++
b16++-NH3
y11
b12-NH3
b11-NH3
y7
b9
a9
y12
y13
b13
a15
y15-NH3
b16-H2O
a14
y11-H2O
y12-NH3
y6++-NH3
y5++-NH3
b4-NH3
b5-NH3
y13++
b14++
200.00
400.00
600.00
800.00
1000.00
1200.00
1400.00
1600.00
1800.00
2000.00
0.0
20.0
40.0
60.0
80.0
100.0
120.0
140.0
160.0
180.0
200.0

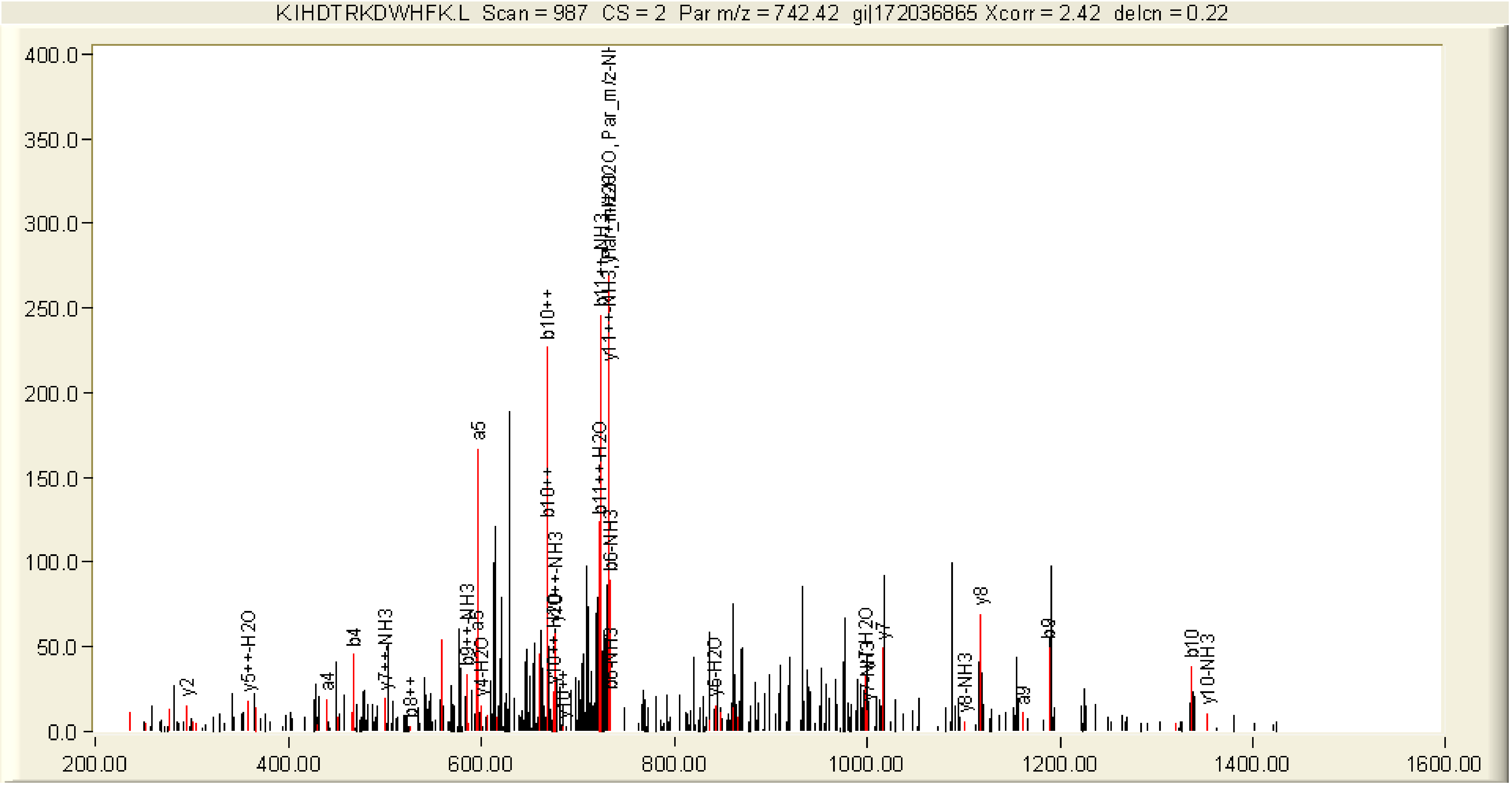
KIHDTRKDWHFK.L Scan = 987 CS = 2 Par m/z = 742.42 gi|172036865 Xcorr = 2.42 delcn = 0.22
400.0
350.0
300.0
250.0
200.0
150.0
100.0
50.0
0.0
200.00
400.00
600.00
800.00
1000.00
1200.00
1400.00
1600.00
y2
y5++-H2O
a4
b4
y7++-NH3
b8++
a5
b10++
b11++-H2O
b6-NH3
y8
b9
b10
y10-NH3
y8-NH3
a9
y7
y6-H2O

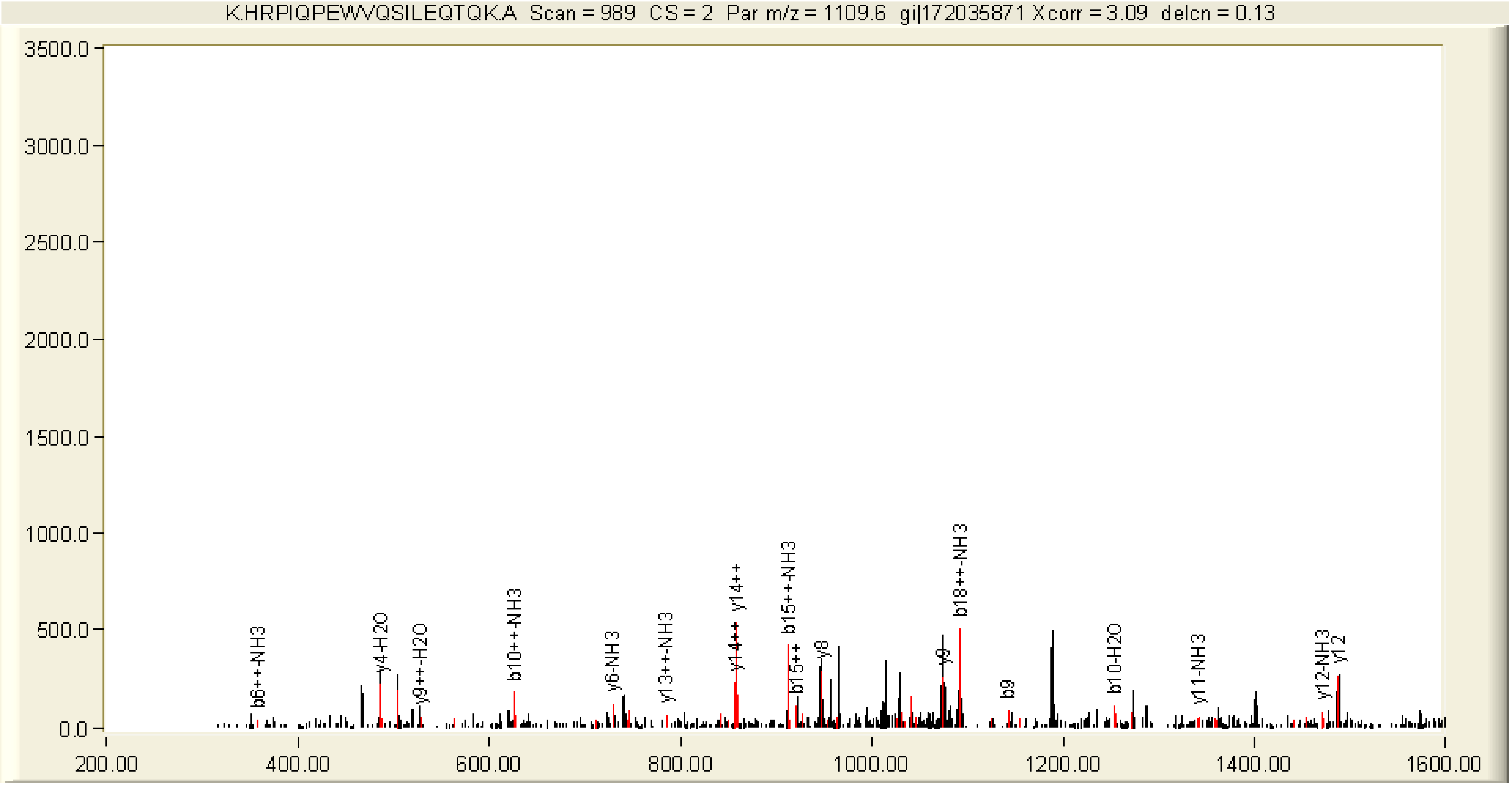

K.HRPIQPEWVQSILEQTQK.A Scan = 989 CS = 2 Par m/z = 1109.6 gi|172035871 Xcorr = 3.09 delcn = 0.13
3500.0
3000.0
2500.0
2000.0
1500.0
1000.0
500.0
0.0
200.00
400.00
600.00
800.00
1000.00
1200.00
1400.00
1600.00
b6++-NH3
y4-H2O
y9++-H2O
b10++-NH3
y6-NH3
y13++-NH3
y14++
y14++
b15++-NH3
b15++
y8
y9
b18++-NH3
b9
b10-H2O
y11-NH3
y12-NH3
y12

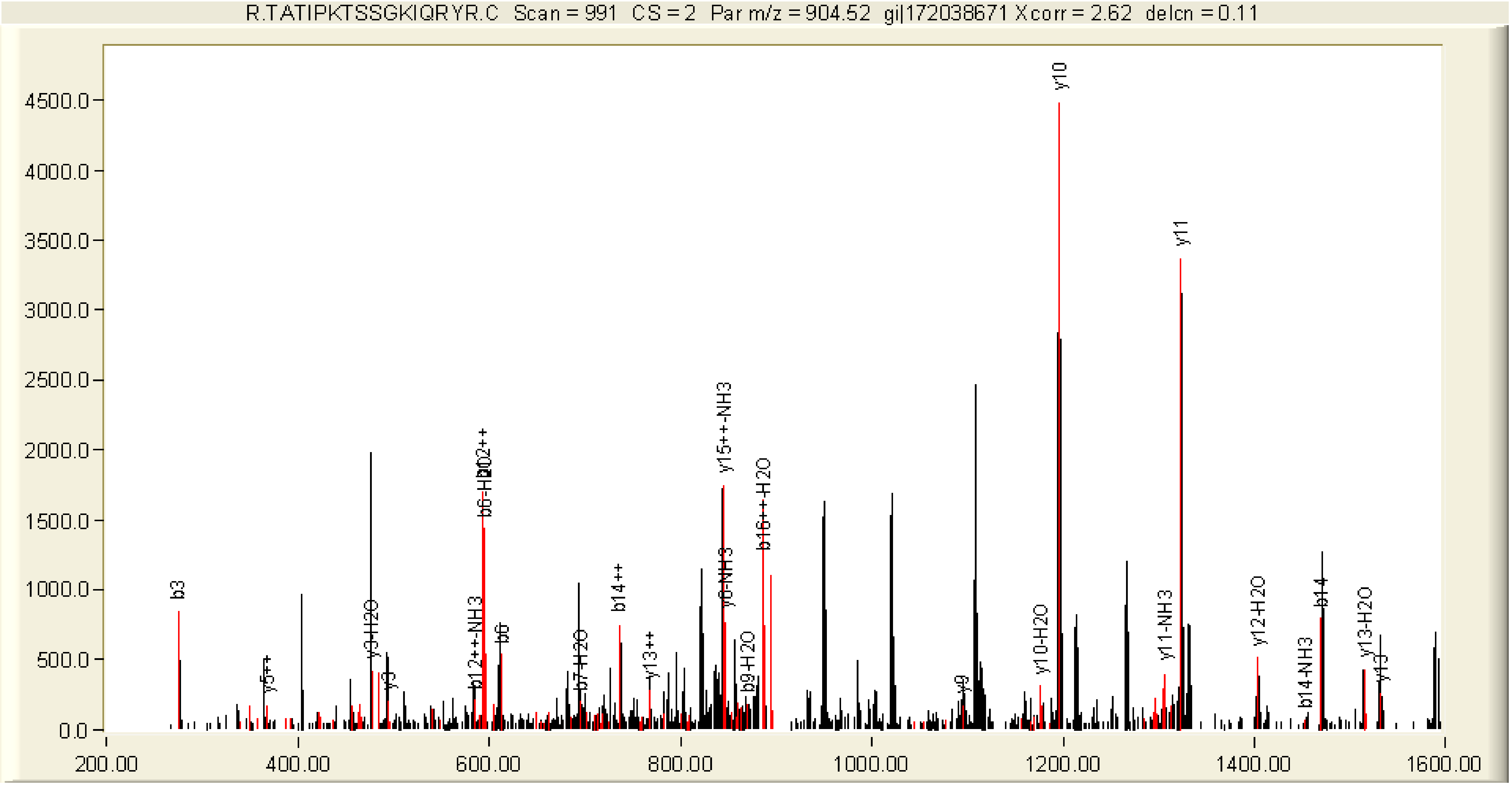

R.TATIPKTSSGKIQRYR.C Scan = 991 CS = 2 Par m/z = 904.52 gi|172038671 Xcorr = 2.62 delcn = 0.11
4500.0
4000.0
3500.0
3000.0
2500.0
2000.0
1500.0
1000.0
500.0
0.0
200.00
400.00
600.00
800.00
1000.00
1200.00
1400.00
1600.00
b3
y5++
y3-H2O
y3
b12++-NH3
b6
b7-H2O
b14++
y13++
y15++-NH3
y6-NH3
b9-H2O
b16++-H2O
y9
y10-H2O
y10
y11-NH3
y11
y12-H2O
b14-NH3
b14
y13-H2O
y13

K.CTSRELEKAAINR.F Scan = 993 CS = 2 Par m/z = 746.41 gi|172036761 Xcorr = 2.56 delcn = 0.13

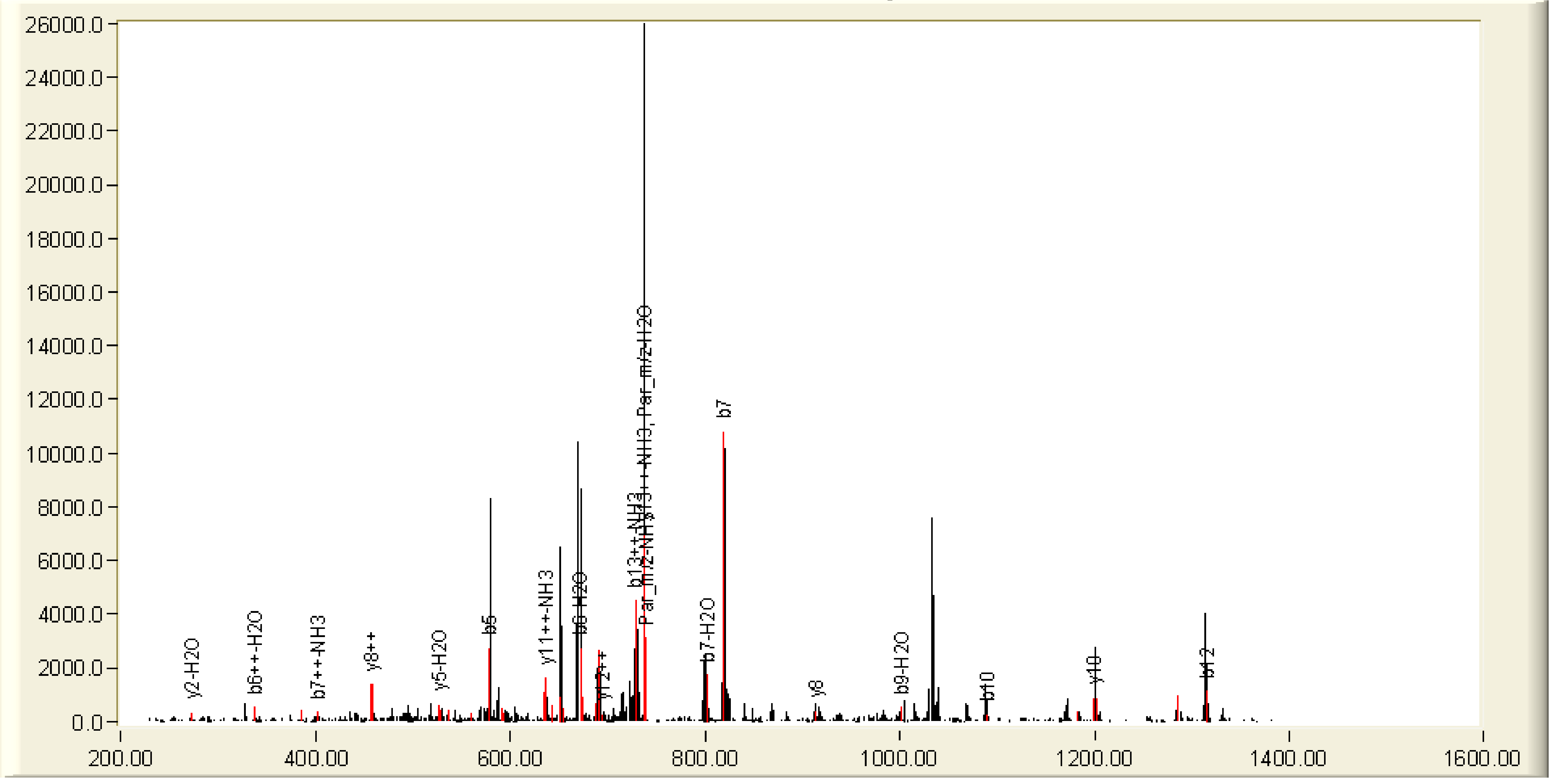

K.QETLDKVNKAIERLLK.T Scan = 995 CS = 2 Par m/z = 949.05 gi|172039477 Xcorr = 2.68 delcn = 0.11

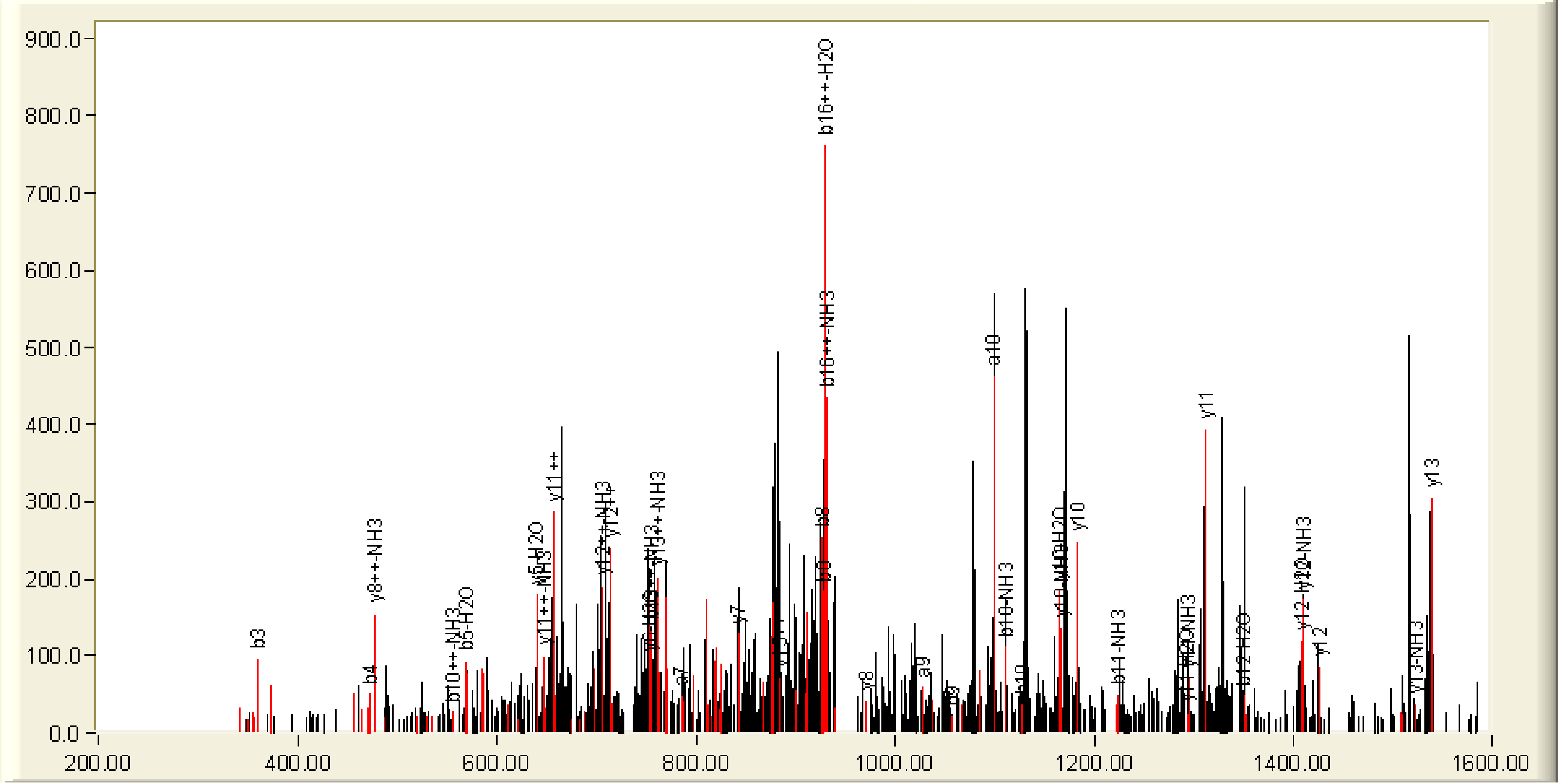

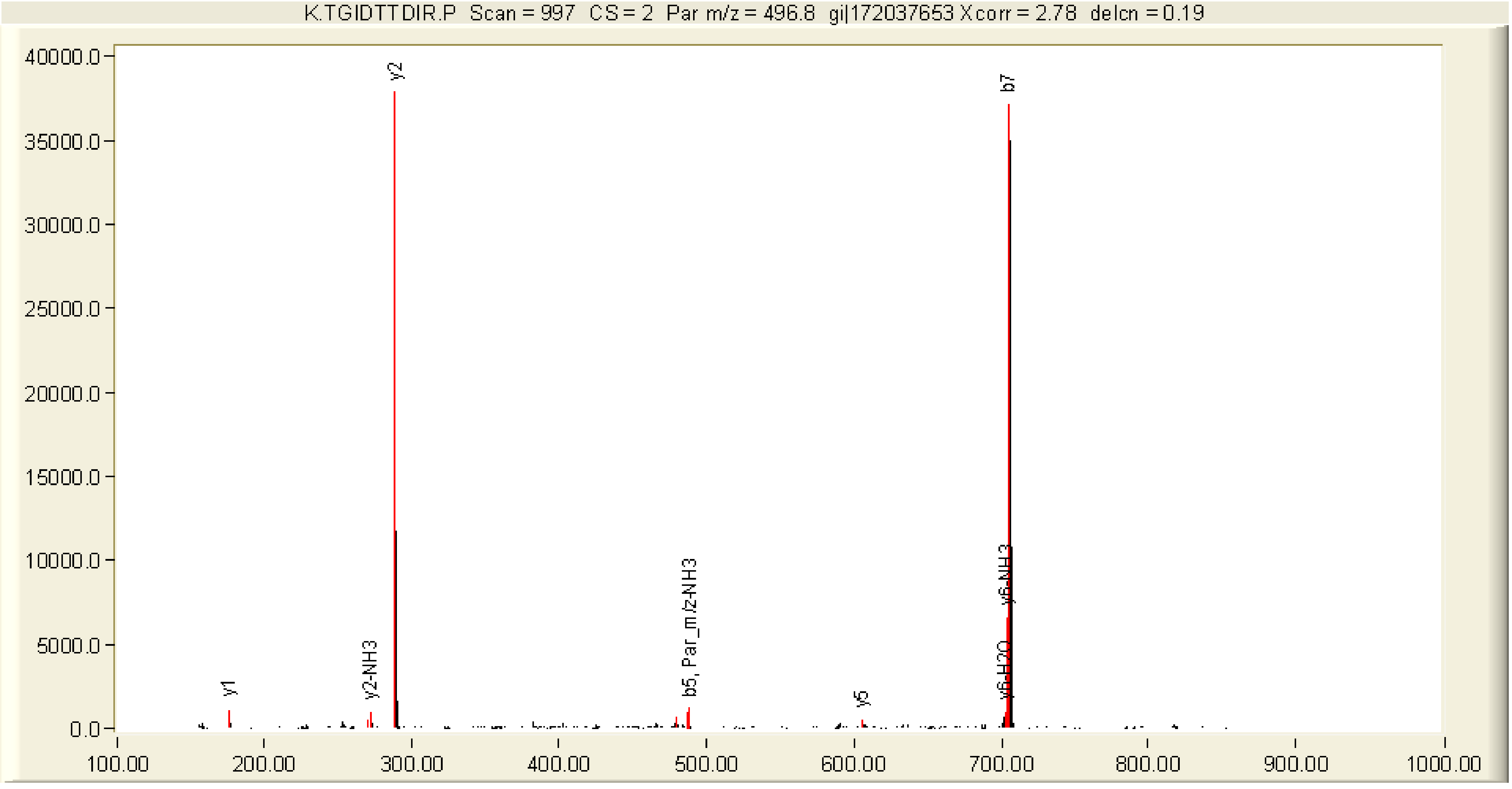

K.TGIDTTDIR.P Scan = 997 CS = 2 Par m/z = 496.8 gi|172037653 Xcorr = 2.78 delcn = 0.19
y2
b7
y1
y2-NH3
b5, Par_m/z-NH3
y5
y6-H2O
y6-NH3
40000.0
35000.0
30000.0
25000.0
20000.0
15000.0
10000.0
5000.0
0.0
100.00
200.00
300.00
400.00
500.00
600.00
700.00
800.00
900.00
1000.00

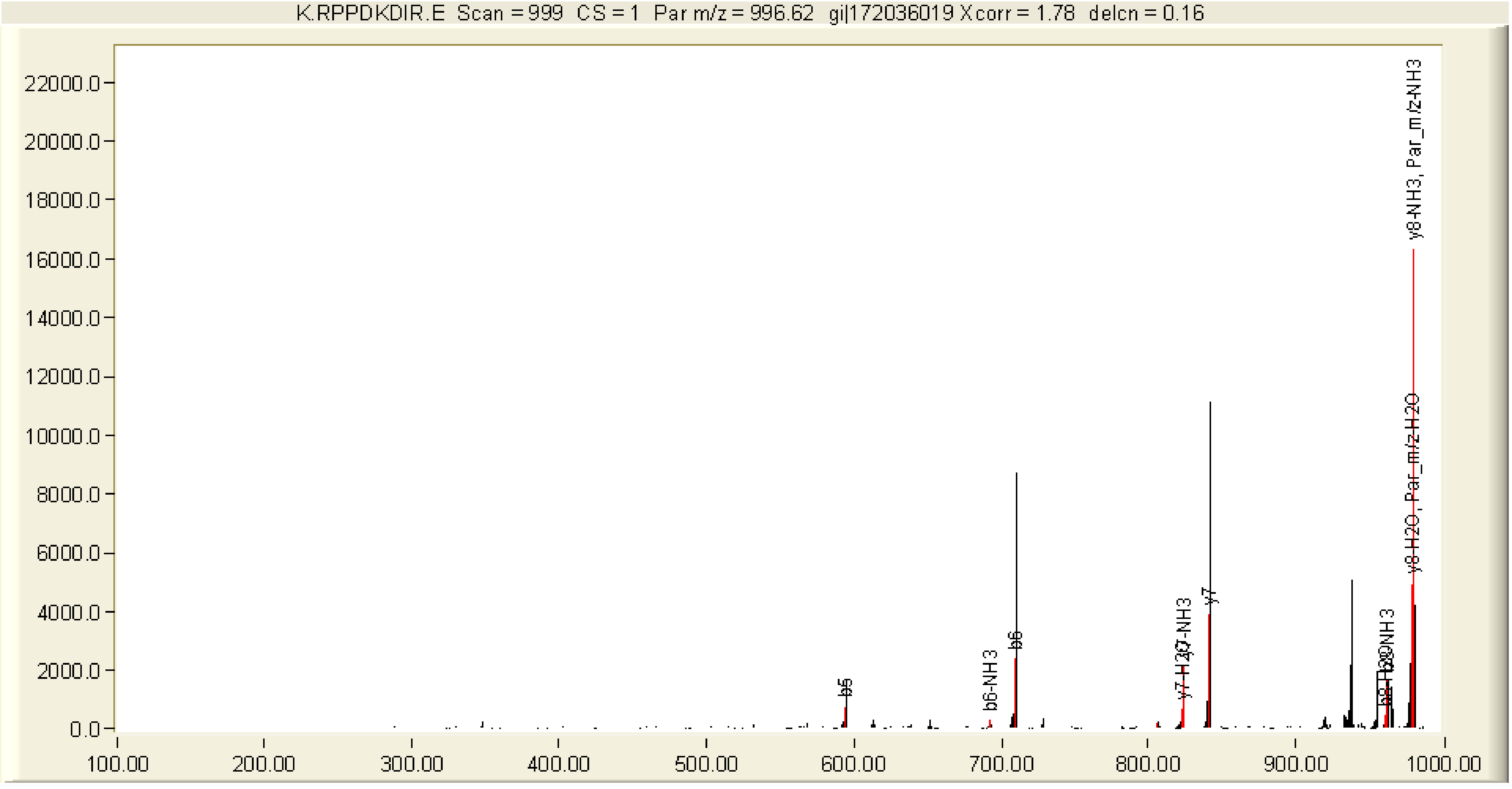
K.RPPDKDIR.E Scan = 999 CS = 1 Par m/z = 996.62 gi|172036019 Xcorr = 1.78 delcn = 0.16
22000.0
20000.0
18000.0
16000.0
14000.0
12000.0
10000.0
8000.0
6000.0
4000.0
2000.0
0.0
100.00
200.00
300.00
400.00
500.00
600.00
700.00
800.00
900.00
1000.00
b5
b6-NH3
b6
y7-NH3
y7
y8-H2O, Par_m/z-H2O
y8-NH3, Par_m/z-NH3

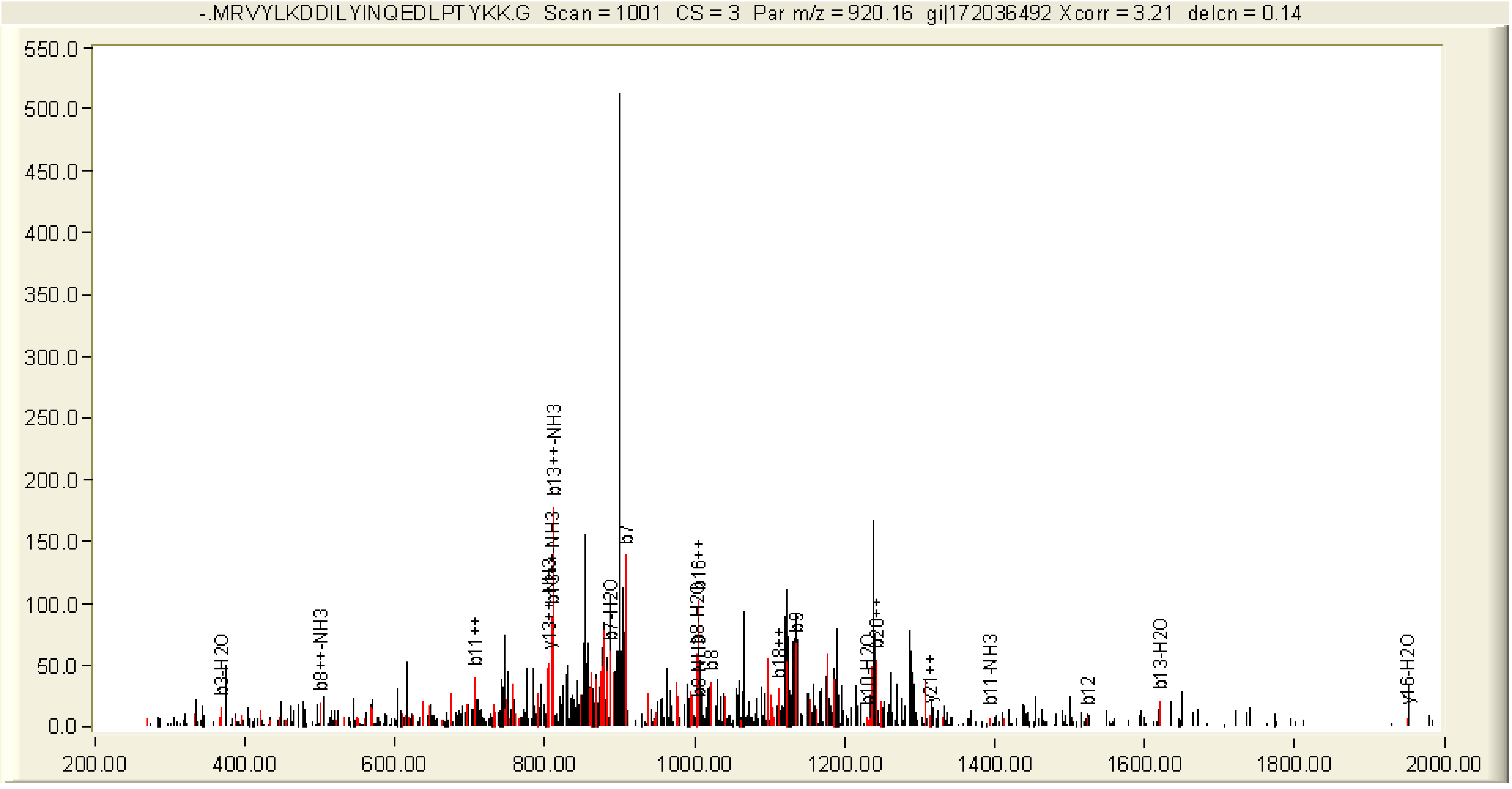

-.MRVYLKDDILYINQEDLPTYKK.G Scan = 1001 CS = 3 Par m/z = 920.16 gi|172036492 Xcorr = 3.21 delcn = 0.14
550.0
500.0
450.0
400.0
350.0
300.0
250.0
200.0
150.0
100.0
50.0
0.0
200.00
400.00
600.00
800.00
1000.00
1200.00
1400.00
1600.00
1800.00
2000.00
b3-H2O
b8++-NH3
b11++
b13++-NH3
b7-H2O
b7
b16++
b8
b18++
b9
b10-H2O
b20++
y21++
b11-NH3
b12
b13-H2O
y16-H2O

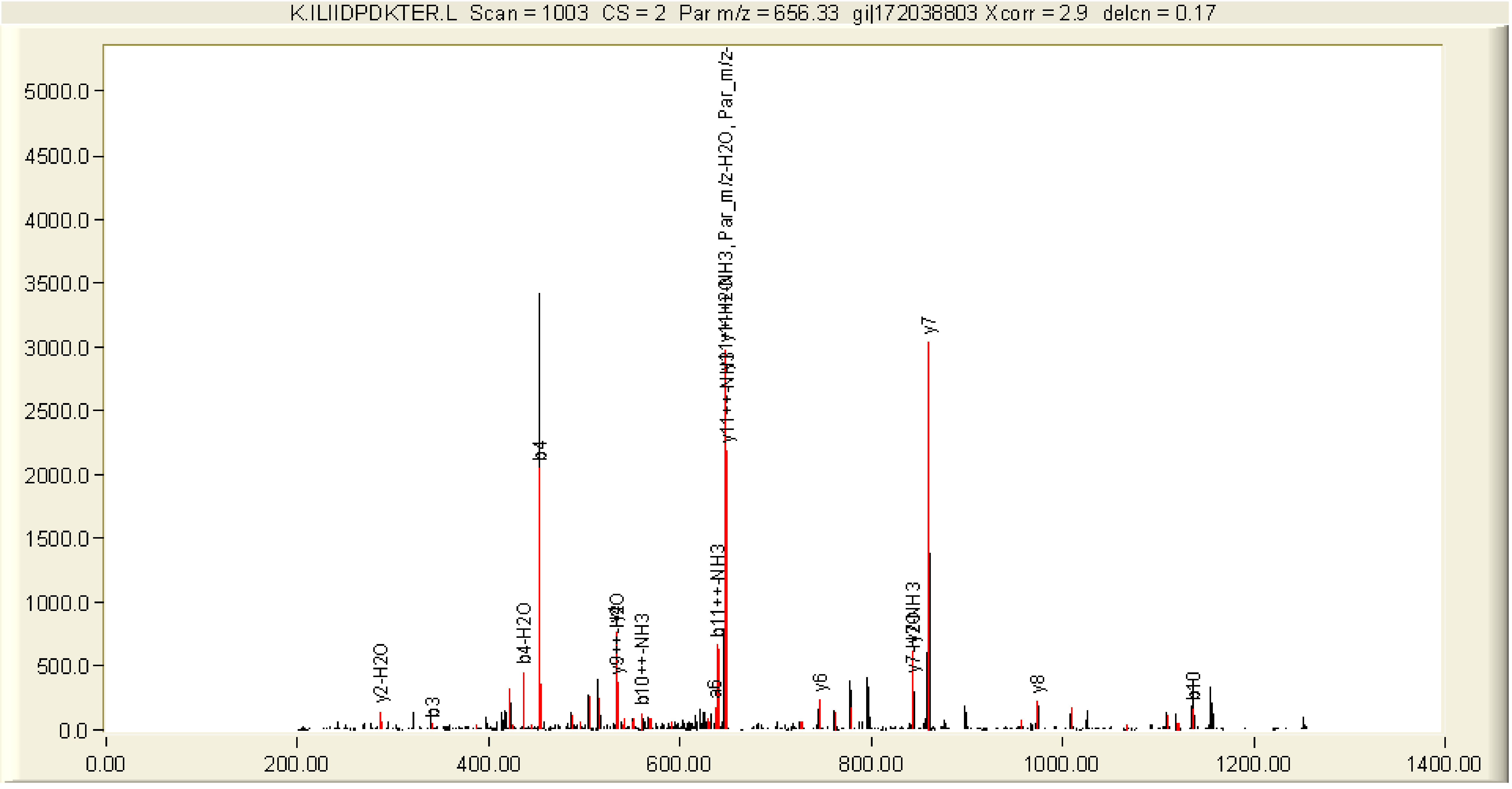

K.ILIIDPDKTER.L Scan = 1003 CS = 2 Par m/z = 656.33 gi|172038803 Xcorr = 2.9 delcn = 0.17
5000.0
4500.0
4000.0
3500.0
3000.0
2500.0
2000.0
1500.0
1000.0
500.0
0.0
0.00
200.00
400.00
600.00
800.00
1000.00
1200.00
1400.00
y2-H2O
b3
b4-H2O
b4
b10++-NH3
a6
b11++-NH3
y6
y7
y8
b10

K.SELGWADFRVTHYEQIQK.W Scan = 1005 CS = 2 Par m/z = 1103.02 gi|172035740 Xcorr = 2.51 delcn = 0.12

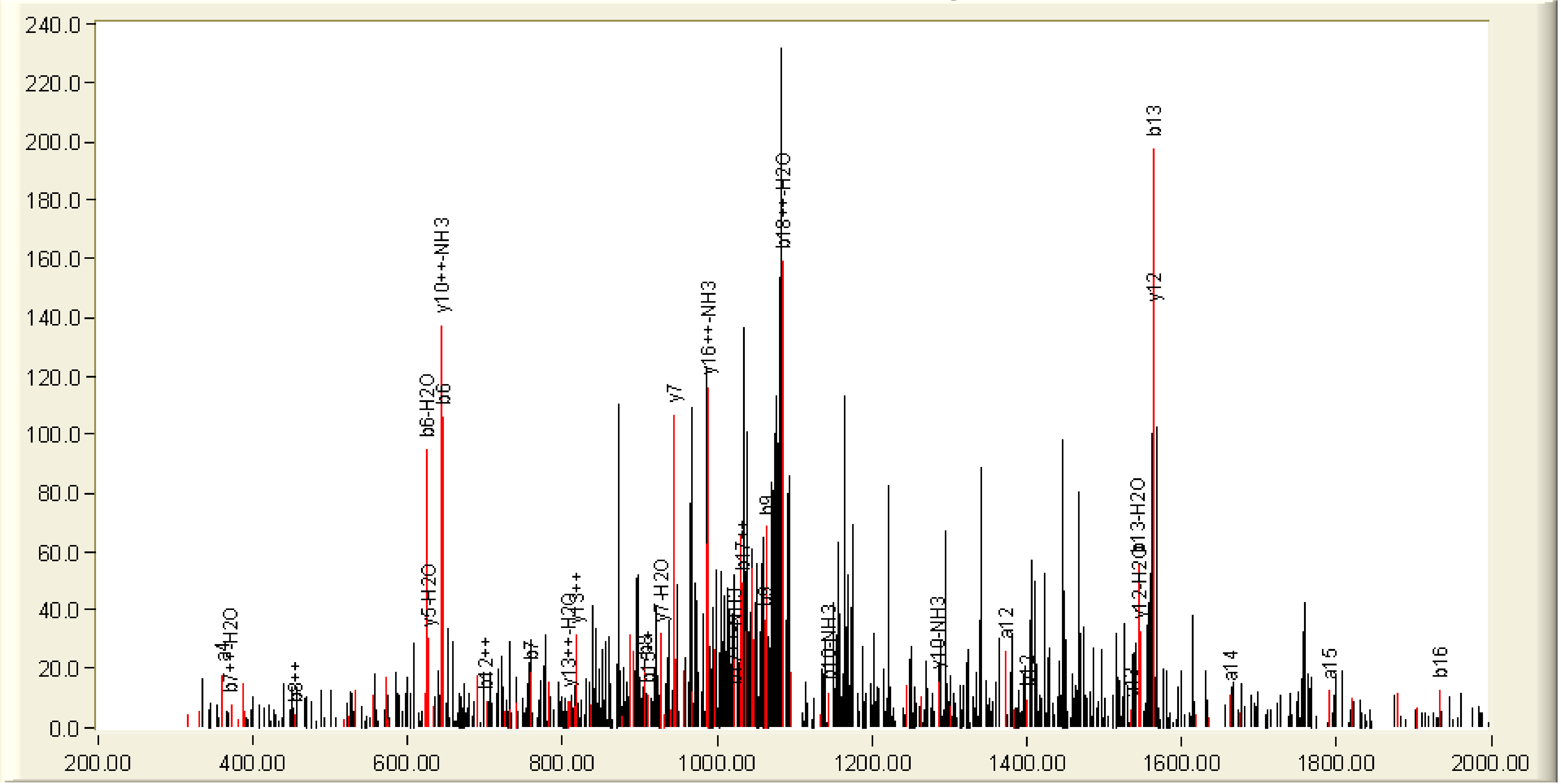

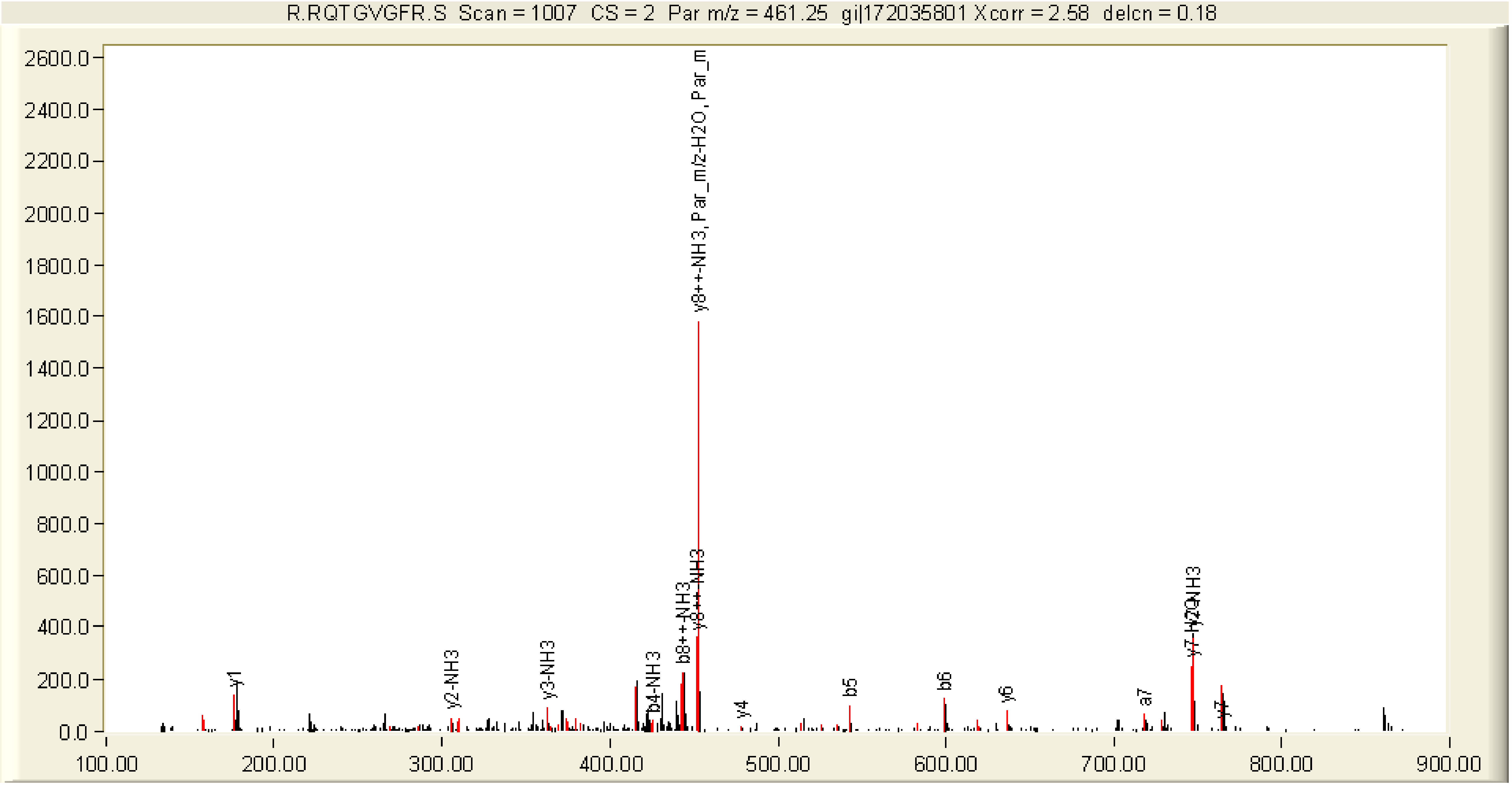
R.RQTGVGFR.S Scan = 1007 CS = 2 Par m/z = 461.25 gi|172035801 Xcorr = 2.58 delcn = 0.18
2600.0
2400.0
2200.0
2000.0
1800.0
1600.0
1400.0
1200.0
1000.0
800.0
600.0
400.0
200.0
0.0
100.00
200.00
300.00
400.00
500.00
600.00
700.00
800.00
900.00
y8++-NH3, Par_m/z-H2O, Par_m
y1
y2-NH3
y3-NH3
b4-NH3
b8++-NH3
y8++-NH3
y4
b5
b6
y6
a7
y7-H2O
y7-NH3

K.VLKEENNQ.- Scan = 1009 CS = 2 Par m/z = 487.25 gi|172037874 Xcorr = 3.34 delcn = 0.1

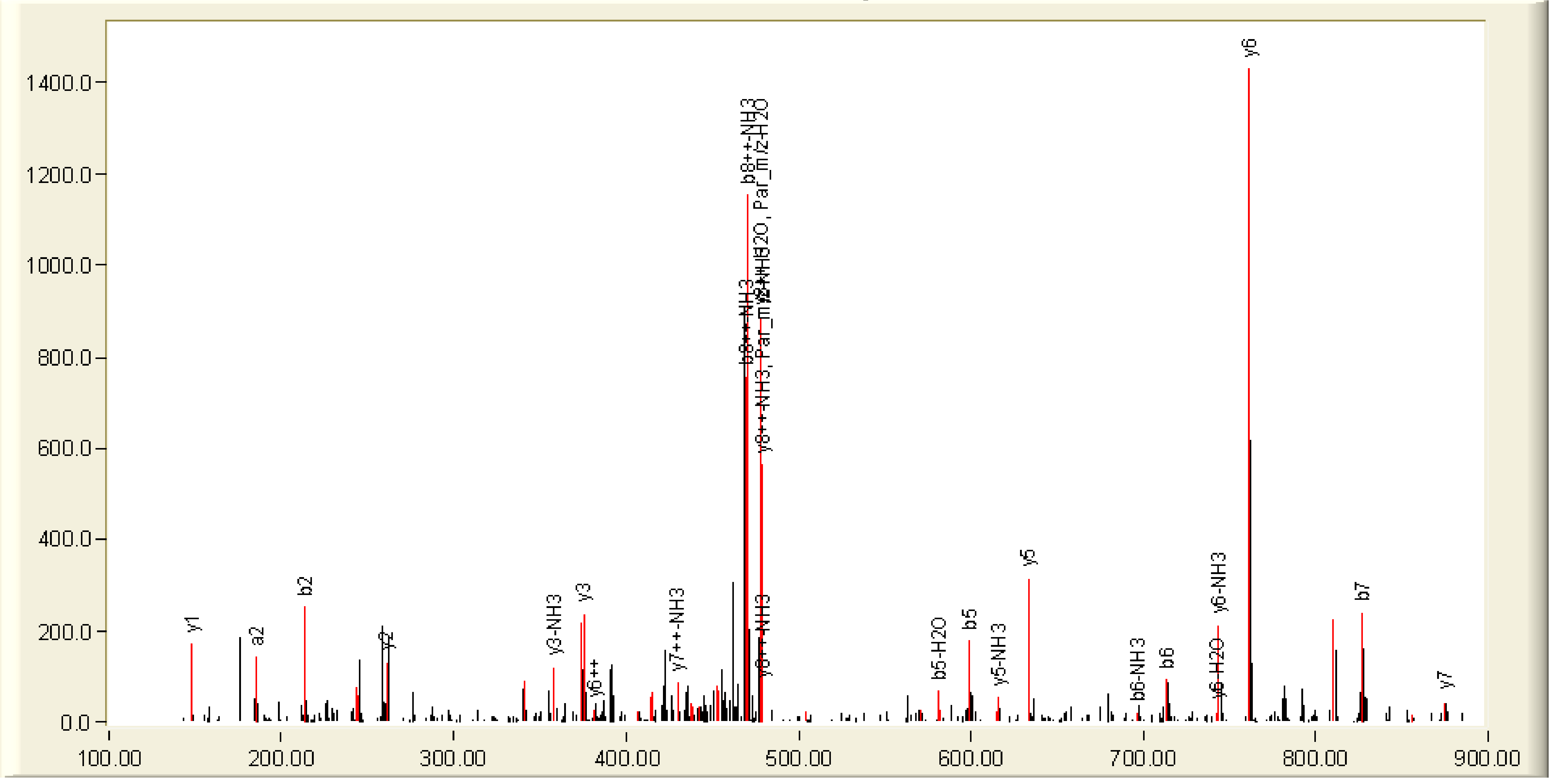

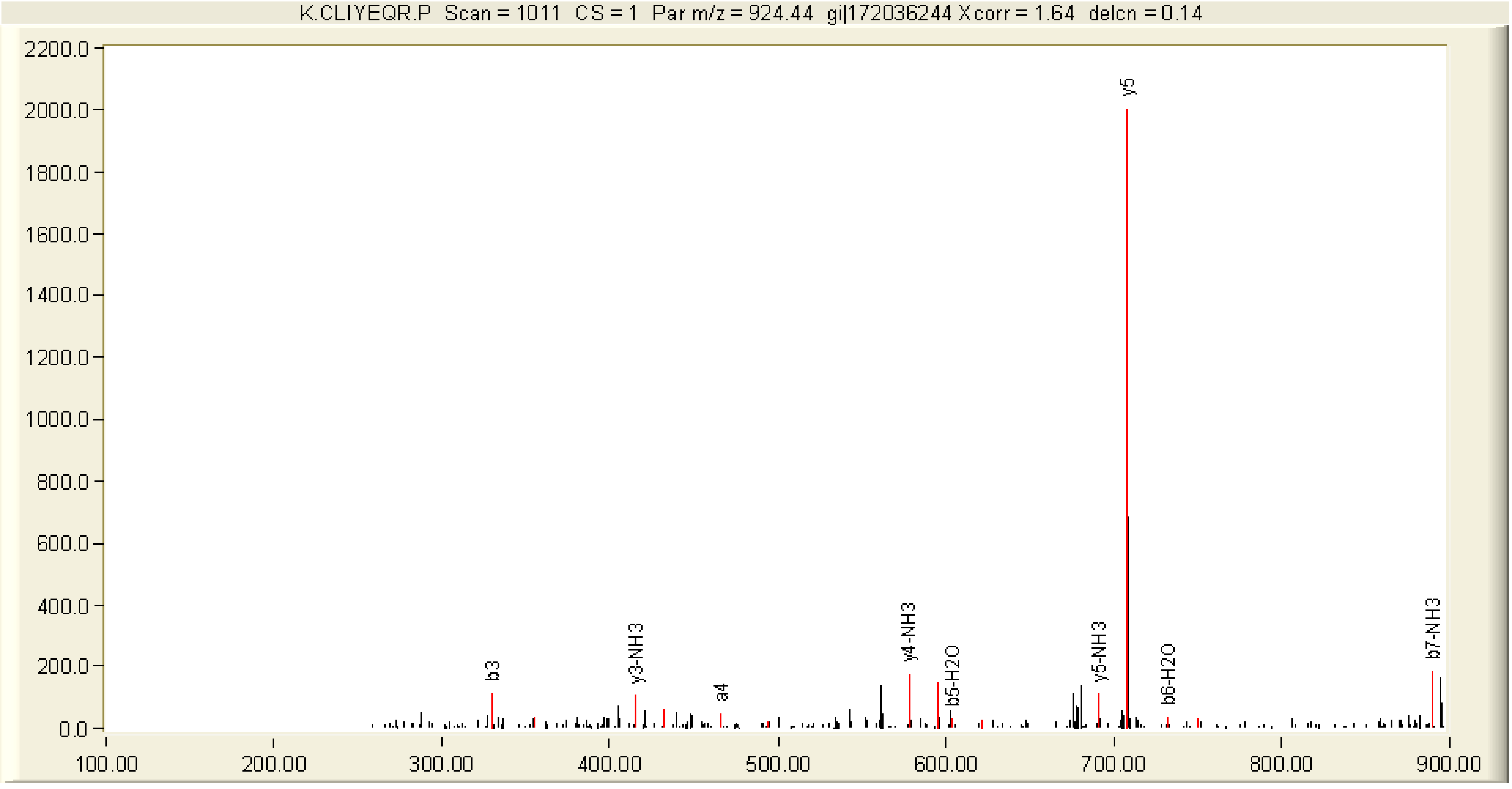

K.CLIYEQR.P Scan = 1011 CS = 1 Par m/z = 924.44 gi|172036244 Xcorr = 1.64 delcn = 0.14
0.0
200.0
400.0
600.0
800.0
1000.0
1200.0
1400.0
1600.0
1800.0
2000.0
2200.0
100.00
200.00
300.00
400.00
500.00
600.00
700.00
800.00
900.00
b3
y3-NH3
a4
y4-NH3
b5-H2O
y5-NH3
y5
b6-H2O
b7-NH3

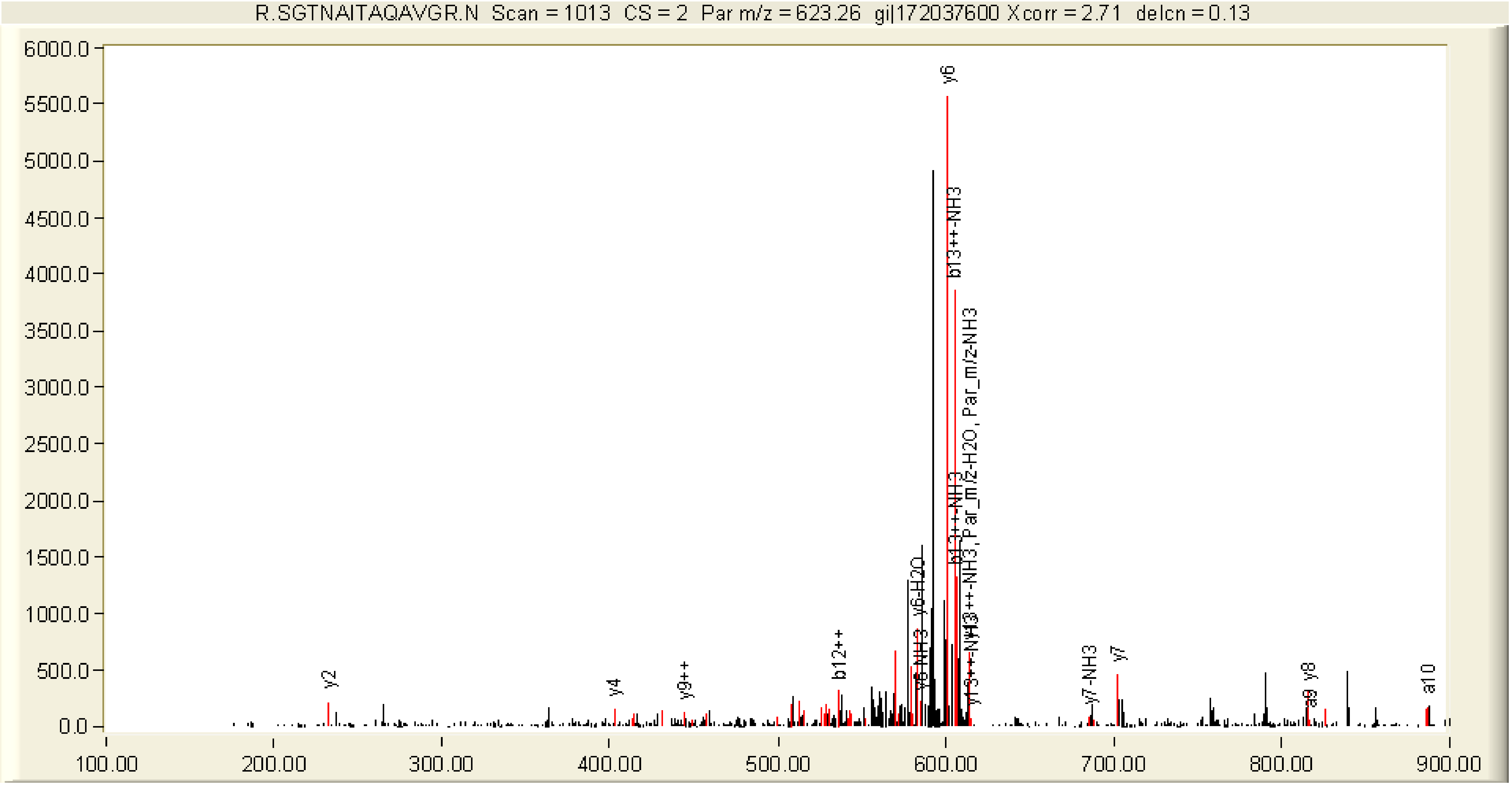
R.SGTNAITAQAVGR.N Scan = 1013 CS = 2 Par m/z = 623.26 gi|172037600 Xcorr = 2.71 delcn = 0.13
6000.0
5500.0
5000.0
4500.0
4000.0
3500.0
3000.0
2500.0
2000.0
1500.0
1000.0
500.0
0.0
100.00
200.00
300.00
400.00
500.00
600.00
700.00
800.00
900.00
y6
b13++-NH3
y6-H2O
y6-NH3
y2
y4
y9++
b12++
y7-NH3
y7
a9
y8
a10

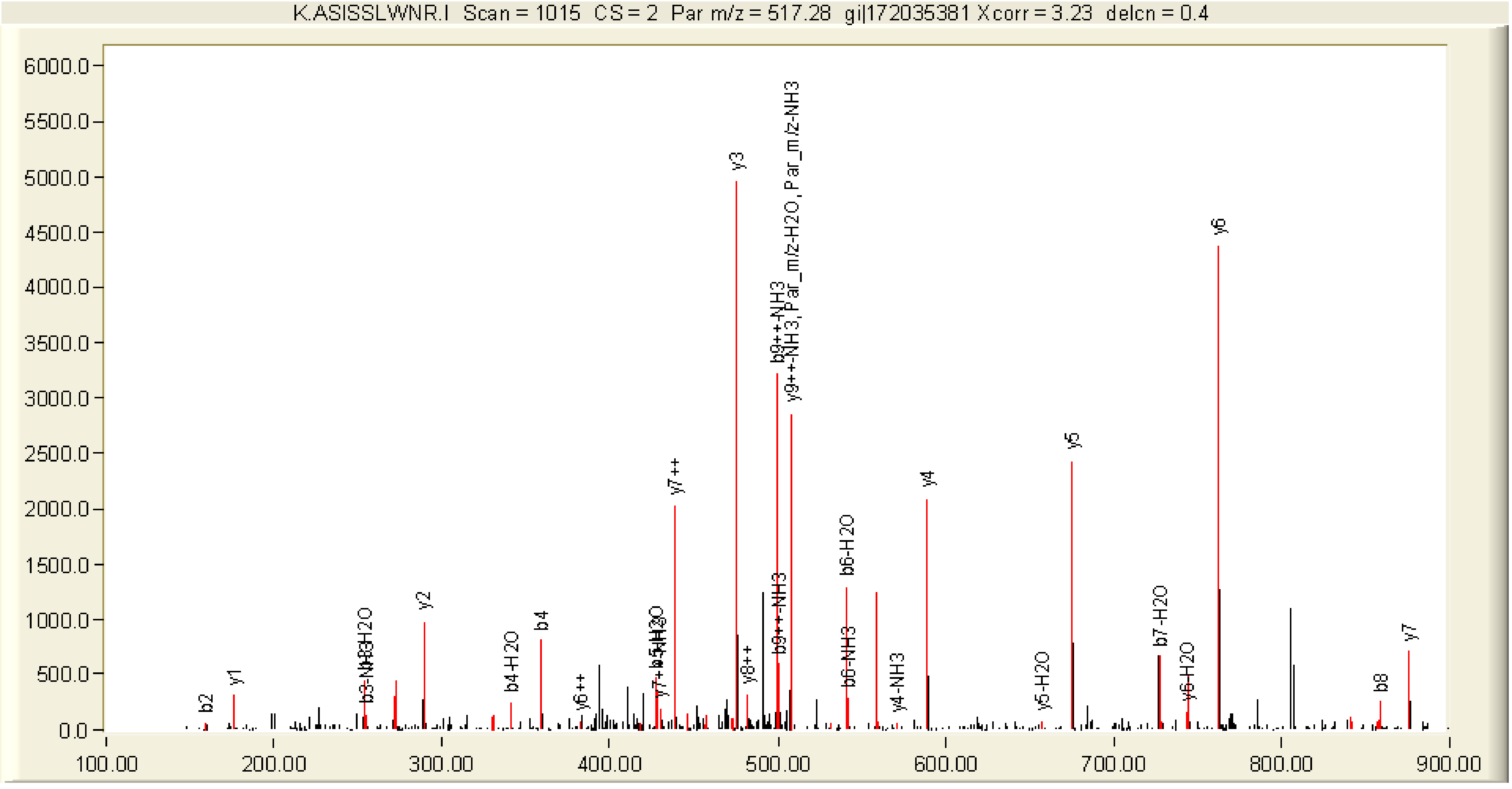

K.ASISSLWNR.I Scan = 1015 CS = 2 Par m/z = 517.28 gi|172035381 Xcorr = 3.23 delcn = 0.4
6000.0
5500.0
5000.0
4500.0
4000.0
3500.0
3000.0
2500.0
2000.0
1500.0
1000.0
500.0
0.0
100.00
200.00
300.00
400.00
500.00
600.00
700.00
800.00
900.00
b2
y1
y2
b4-H2O
b4
y6++
y7++
y3
y8++
b9++-NH3
y9++-NH3, Par_m/z-H2O, Par_m/z-NH3
b6-H2O
b6-NH3
y4-NH3
y4
y5-H2O
y5
b7-H2O
y6-H2O
y6
b8
y7

R.ILIHVEPPNYESSNISFGSDNNNK.S Scan = 1017 CS = 3 Par m/z = 896.77 gi|172035832 Xcorr = 5.8 delcn = 0.35

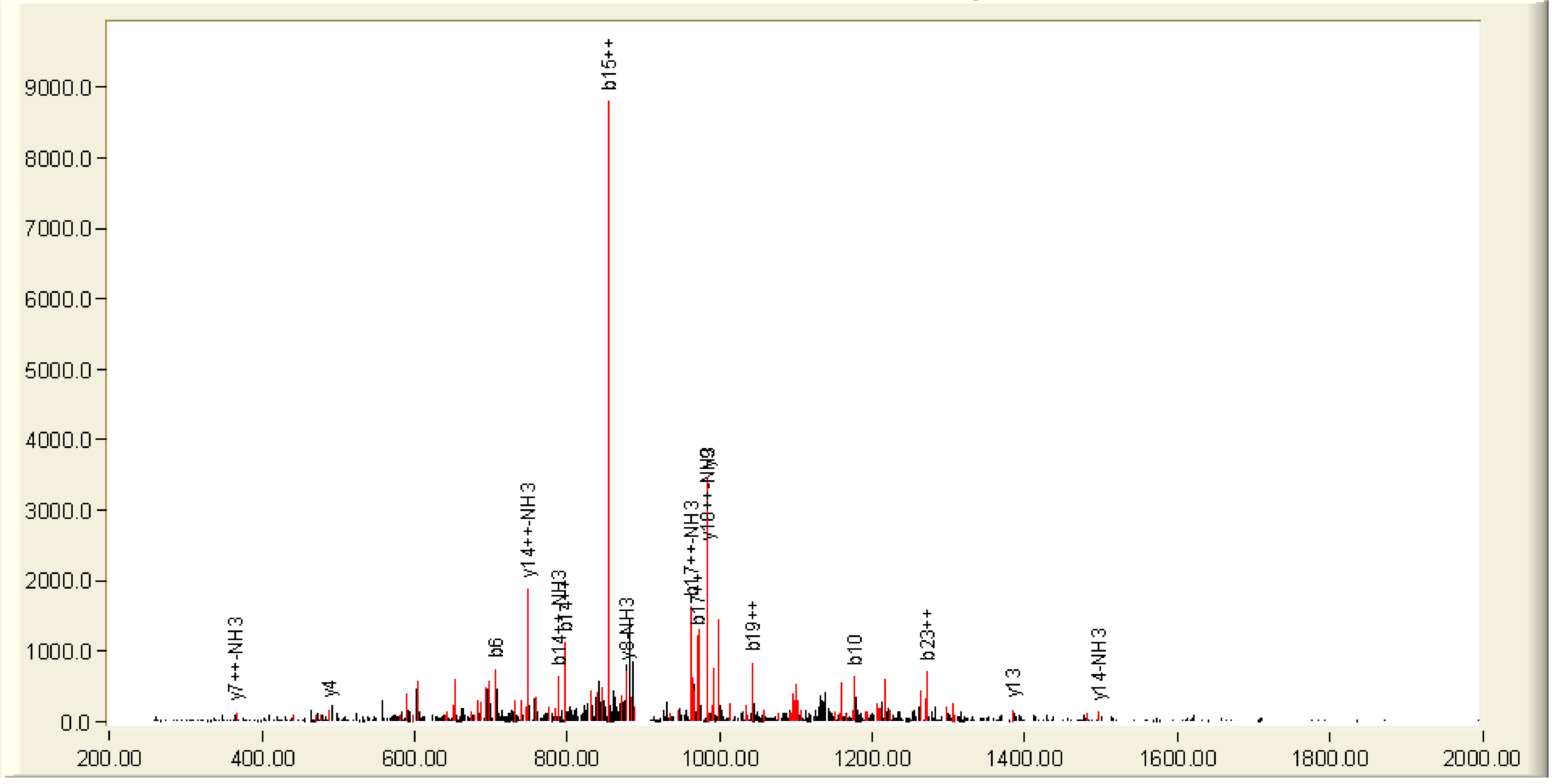

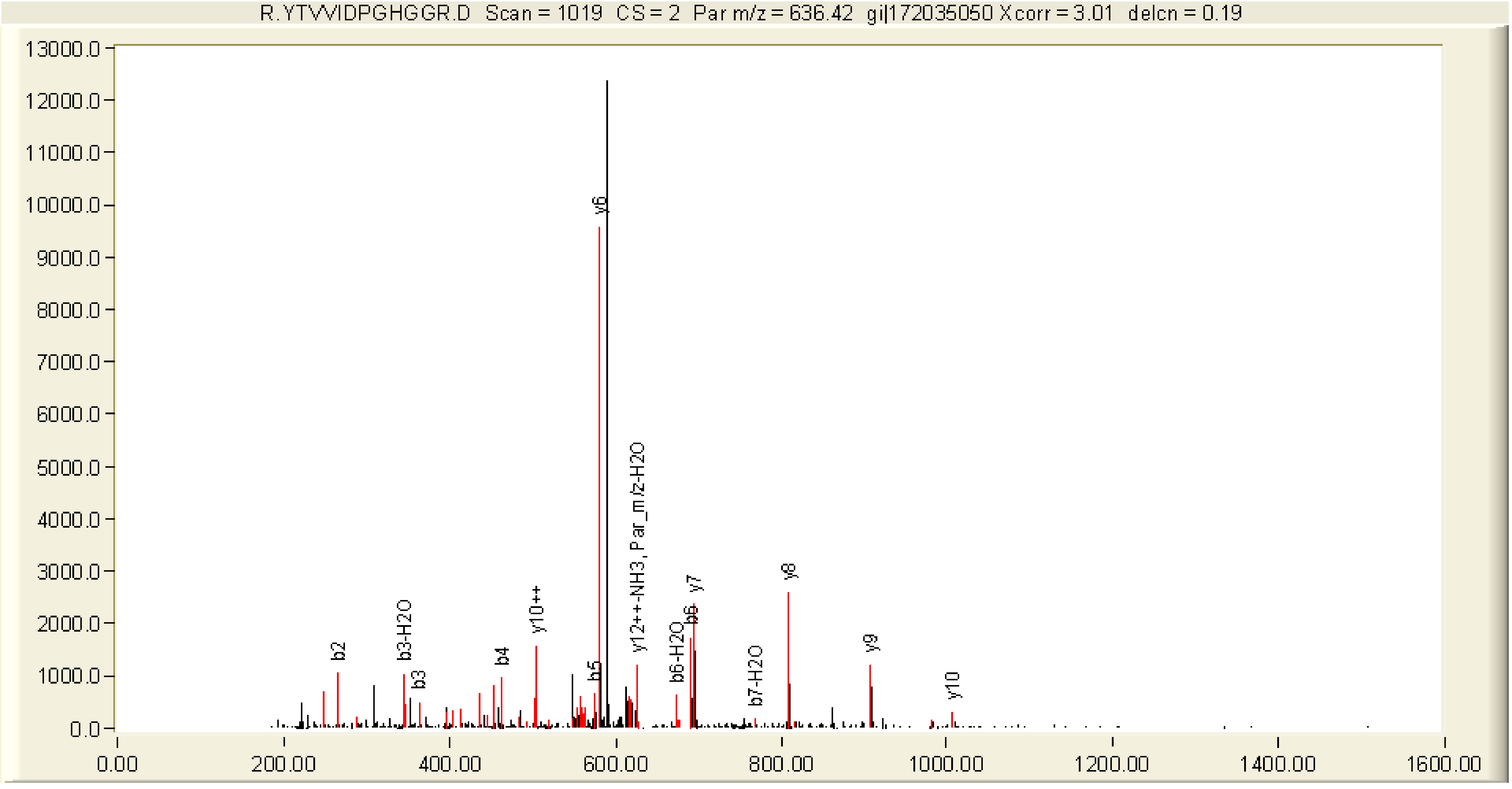
R.YTVVIDPGHGGR.D Scan = 1019 CS = 2 Par m/z = 636.42 gi|172035050 Xcorr = 3.01 delcn = 0.19
y6
y12++-NH3, Par_m/z-H2O
y7
y8
b6
b6-H2O
y10++
b3-H2O
b2
b4
b3
b5
b7-H2O
y9
y10
0.0
1000.0
2000.0
3000.0
4000.0
5000.0
6000.0
7000.0
8000.0
9000.0
10000.0
11000.0
12000.0
13000.0
0.00
200.00
400.00
600.00
800.00
1000.00
1200.00
1400.00
1600.00

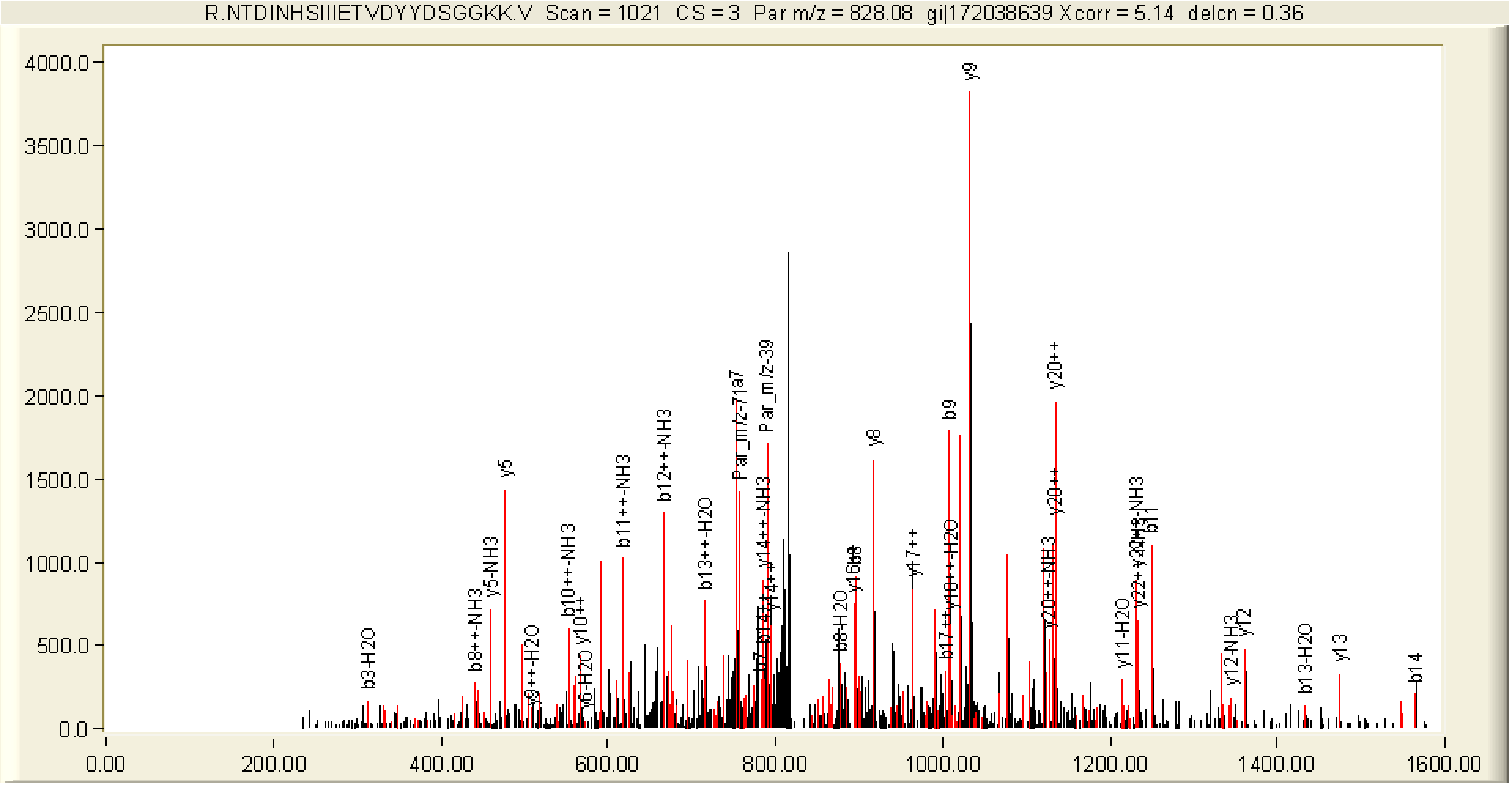
R.NTDINHSIIIETVDYYDSGGKK.V Scan = 1021 CS = 3 Par m/z = 828.08 gi|172038639 Xcorr = 5.14 delcn = 0.36
4000.0
3500.0
3000.0
2500.0
2000.0
1500.0
1000.0
500.0
0.0
0.00
200.00
400.00
600.00
800.00
1000.00
1200.00
1400.00
1600.00
y9
y20++
b9
y8
Par_m/z-39
y5
b12++-NH3
b11++-NH3
b13++-H2O
y5-NH3
b10++-NH3
b8++-NH3
b3-H2O
y11-H2O
y12-NH3
y12
b13-H2O
y13
b14

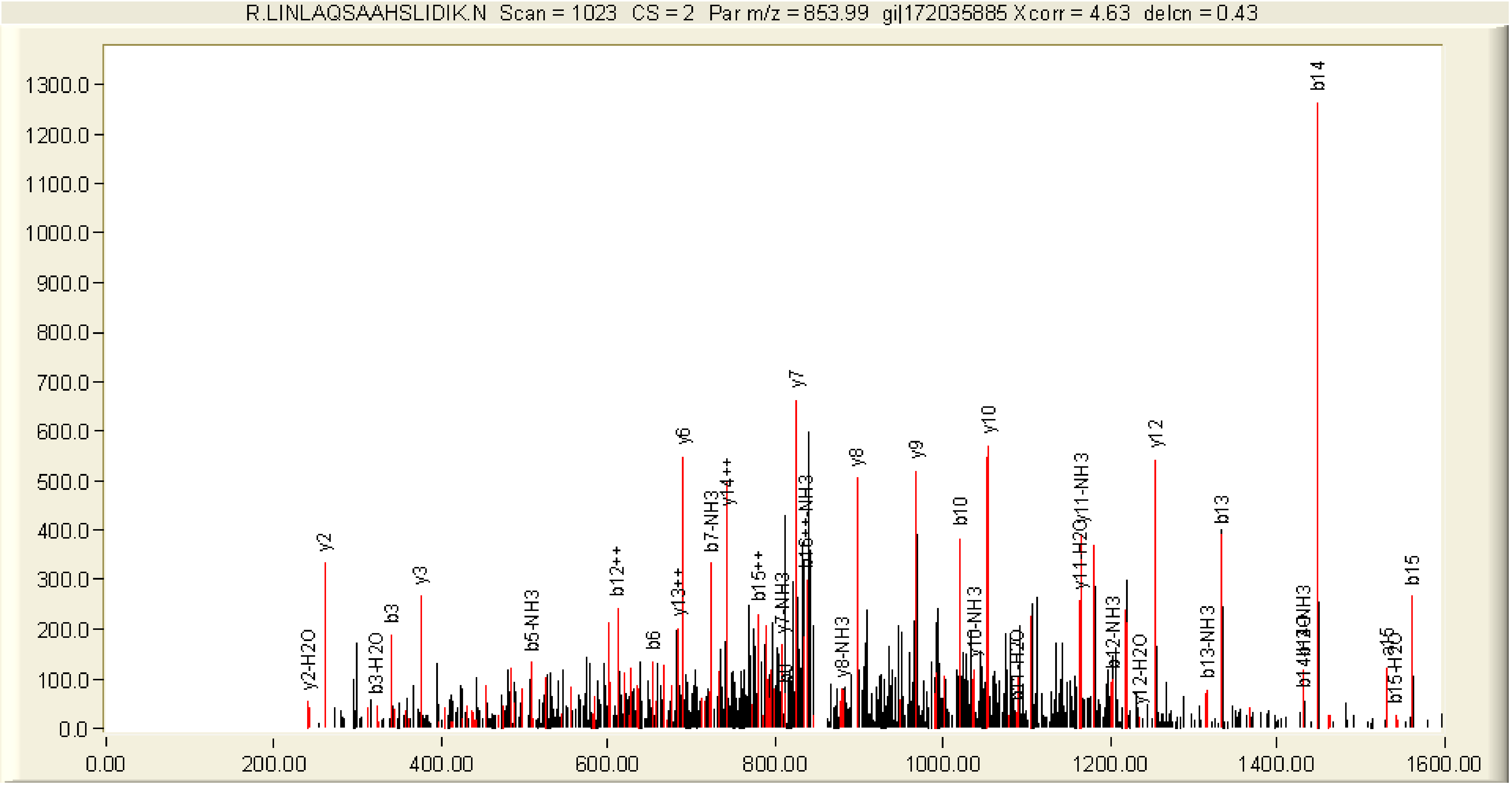
R.LINLAQSAAHSLIDIK.N Scan = 1023 CS = 2 Par m/z = 853.99 gi|172035885 Xcorr = 4.63 delcn = 0.43
1300.0
1200.0
1100.0
1000.0
900.0
800.0
700.0
600.0
500.0
400.0
300.0
200.0
100.0
0.0
0.00
200.00
400.00
600.00
800.00
1000.00
1200.00
1400.00
1600.00
y2-H2O
y2
b3-H2O
b3
y3
b5-NH3
b12++
b6
y13++
y6
b7-NH3
y14++
b15++
y7-NH3
y7
y8-NH3
y8
y9
b10
y10-NH3
y10
y11-H2O
y11-NH3
b12-NH3
y12-H2O
y12
b13-NH3
b13
b14
b15-H2O
b15

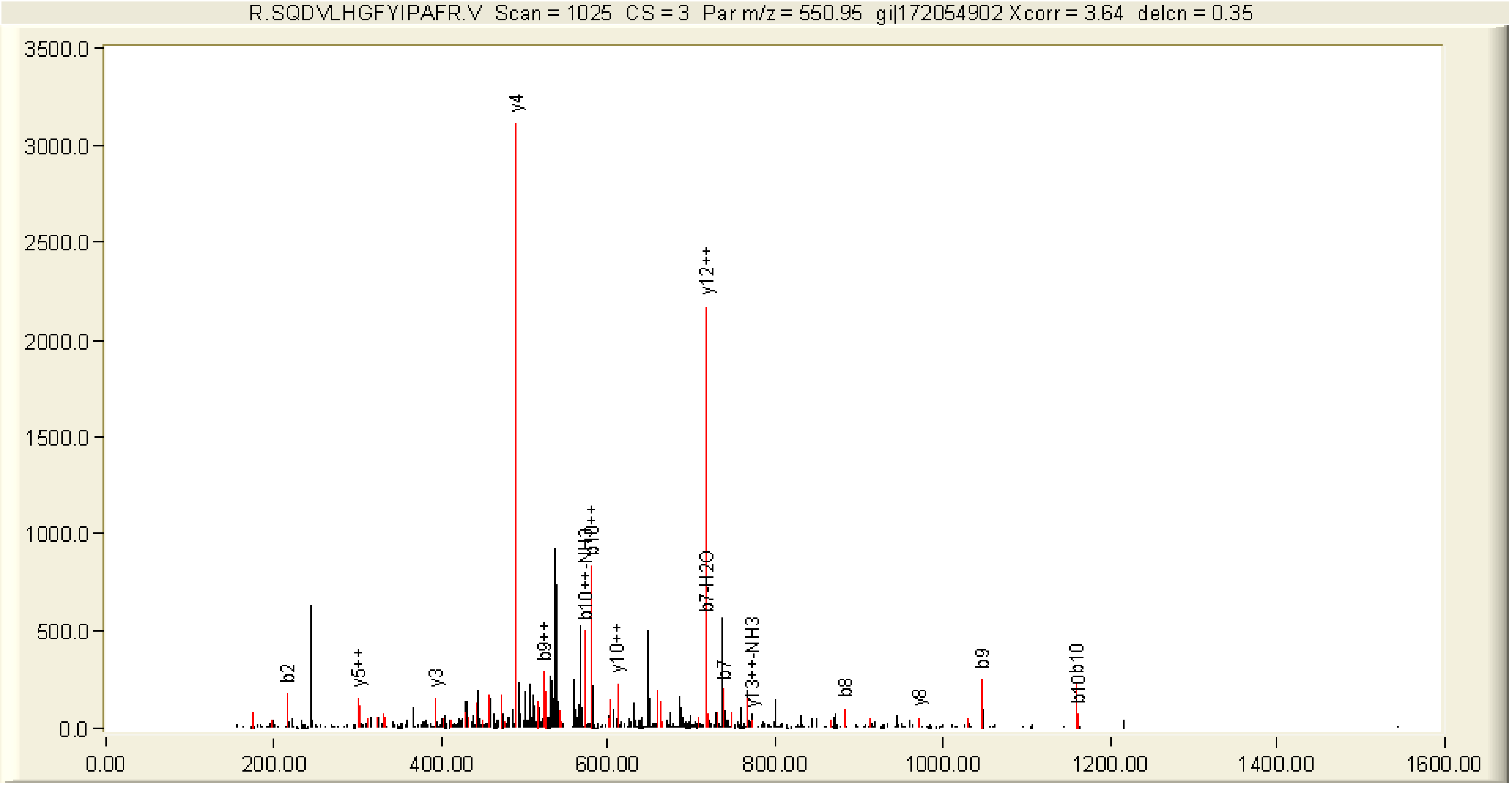
R.SQDVLHGFYIPAFR.V Scan = 1025 CS = 3 Par m/z = 550.95 gi|172054902 Xcorr = 3.64 delcn = 0.35
3500.0
3000.0
2500.0
2000.0
1500.0
1000.0
500.0
0.0
0.00
200.00
400.00
600.00
800.00
1000.00
1200.00
1400.00
1600.00
y4
y12++
b10++-NH3
b10++
b7-H2O
b9++
y10++
y13++-NH3
b7
b2
y5++
y3
b8
y8
b9
b10b10

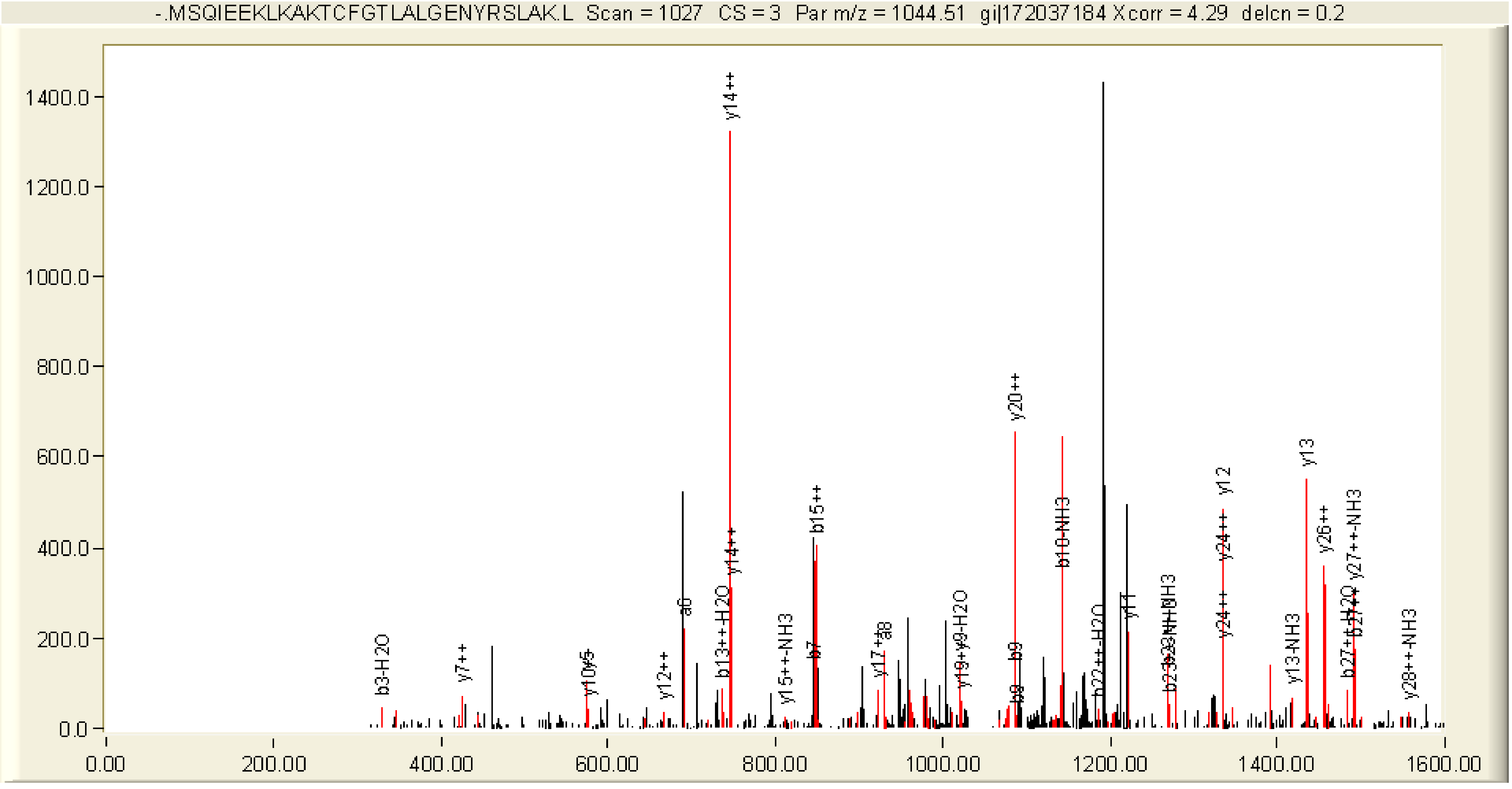

-.MSQIEEKLKAKTCFGTLALGENYRSLAK.L Scan = 1027 CS = 3 Par m/z = 1044.51 gi|172037184 Xcorr = 4.29 delcn = 0.2
1400.0
1200.0
1000.0
800.0
600.0
400.0
200.0
0.0
0.00
200.00
400.00
600.00
800.00
1000.00
1200.00
1400.00
1600.00
b3-H2O
y7++
y12++
a6
b13++-H2O
y14++
y14++
y15++-NH3
b7
b15++
y20++
b9
b10-NH3
b22++-H2O
y11
y24++
y24++
y12
y13-NH3
y13
y26++
b27++-H2O
y27++-NH3
y28++-NH3

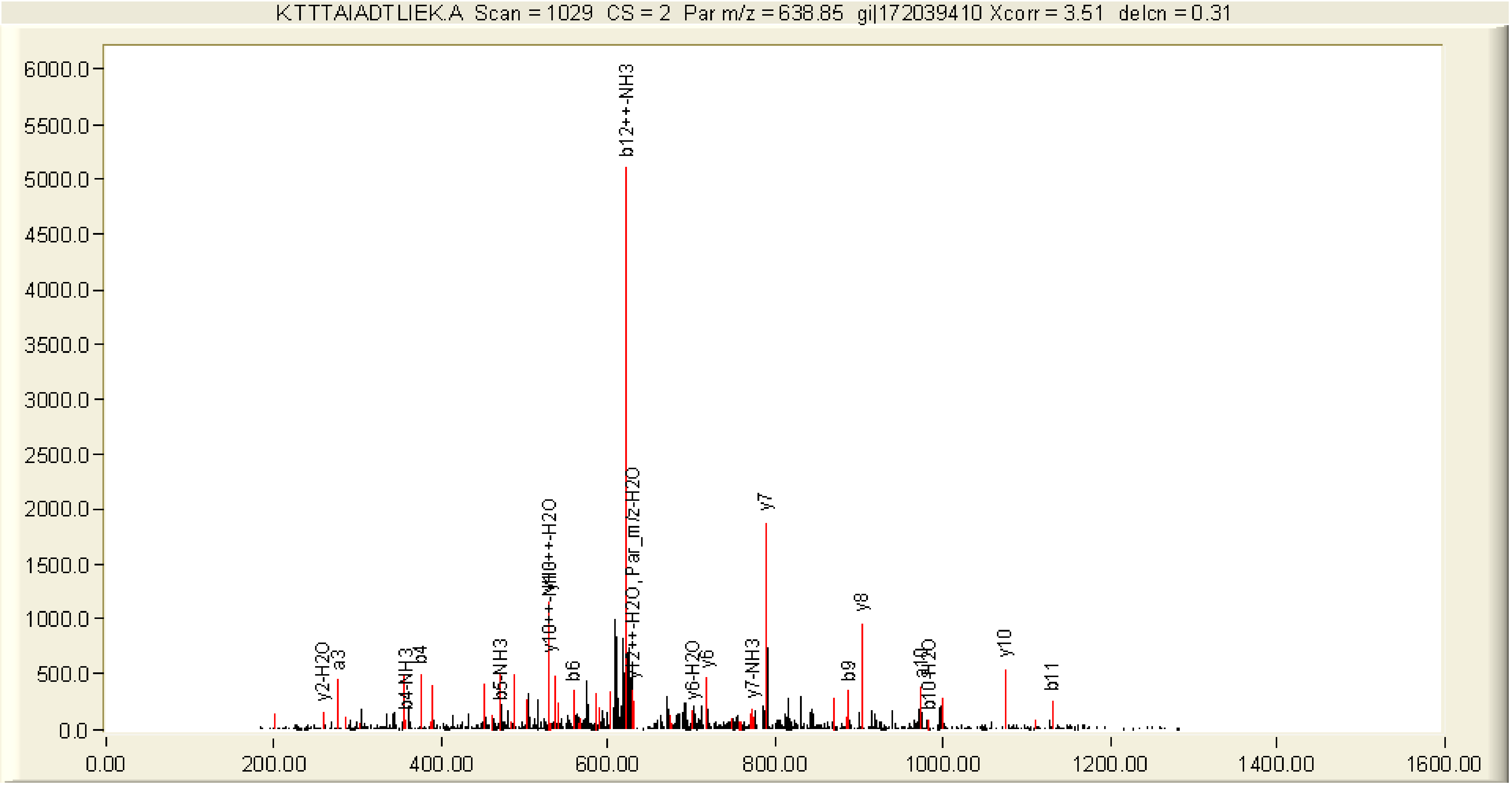

KTTTAIADTLIEK.A Scan = 1029 CS = 2 Par m/z = 638.85 gi|172039410 Xcorr = 3.51 delcn = 0.31
6000.0
5500.0
5000.0
4500.0
4000.0
3500.0
3000.0
2500.0
2000.0
1500.0
1000.0
500.0
0.0
0.00
200.00
400.00
600.00
800.00
1000.00
1200.00
1400.00
1600.00
b12++-NH3
y7
y8
y10
b11
b9
y2-H2O
a3
b4-NH3
b4
b5-NH3
b6
y6-H2O
y6
y7-NH3

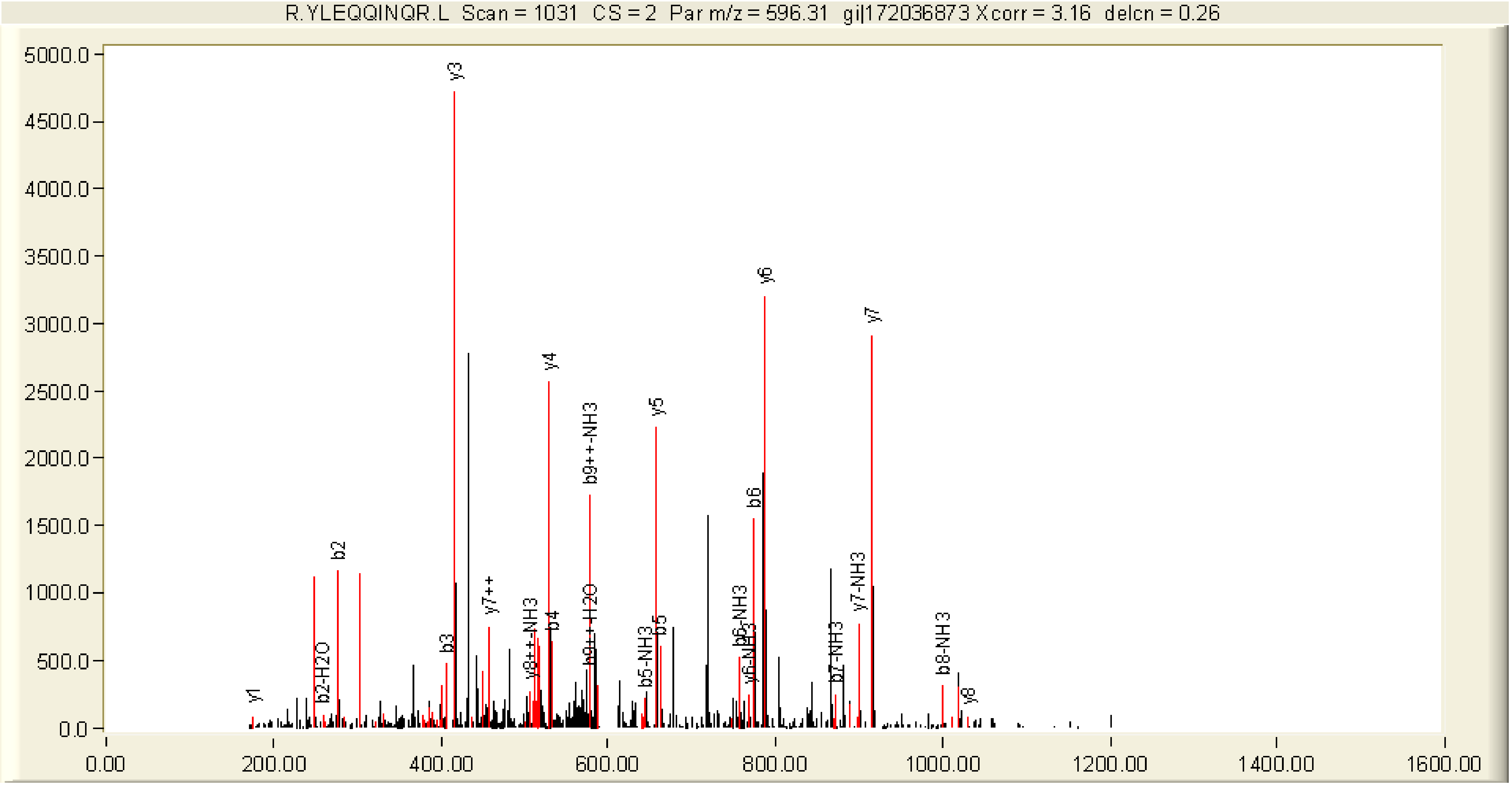

R.YLEQQINQR.L Scan = 1031 CS = 2 Par m/z = 596.31 gi|172036873 Xcorr = 3.16 delcn = 0.26
5000.0
4500.0
4000.0
3500.0
3000.0
2500.0
2000.0
1500.0
1000.0
500.0
0.0
0.00
200.00
400.00
600.00
800.00
1000.00
1200.00
1400.00
1600.00
y1
b2-H2O
b2
b3
y3
y7++
y8++-NH3
y4
b4
b9++-NH3
b9++-H2O
b5-NH3
y5
b5
b6-NH3
y6-NH3
b6
y6
b7-NH3
y7-NH3
y7
b8-NH3
y8

-.MIGEQNMINLEEITIKVSPELAQVYRQAS.T Scan = 1033 CS = 4 Par m/z = 825.68 gi|172036830 Xcorr = 3.08 delcn = 0.14

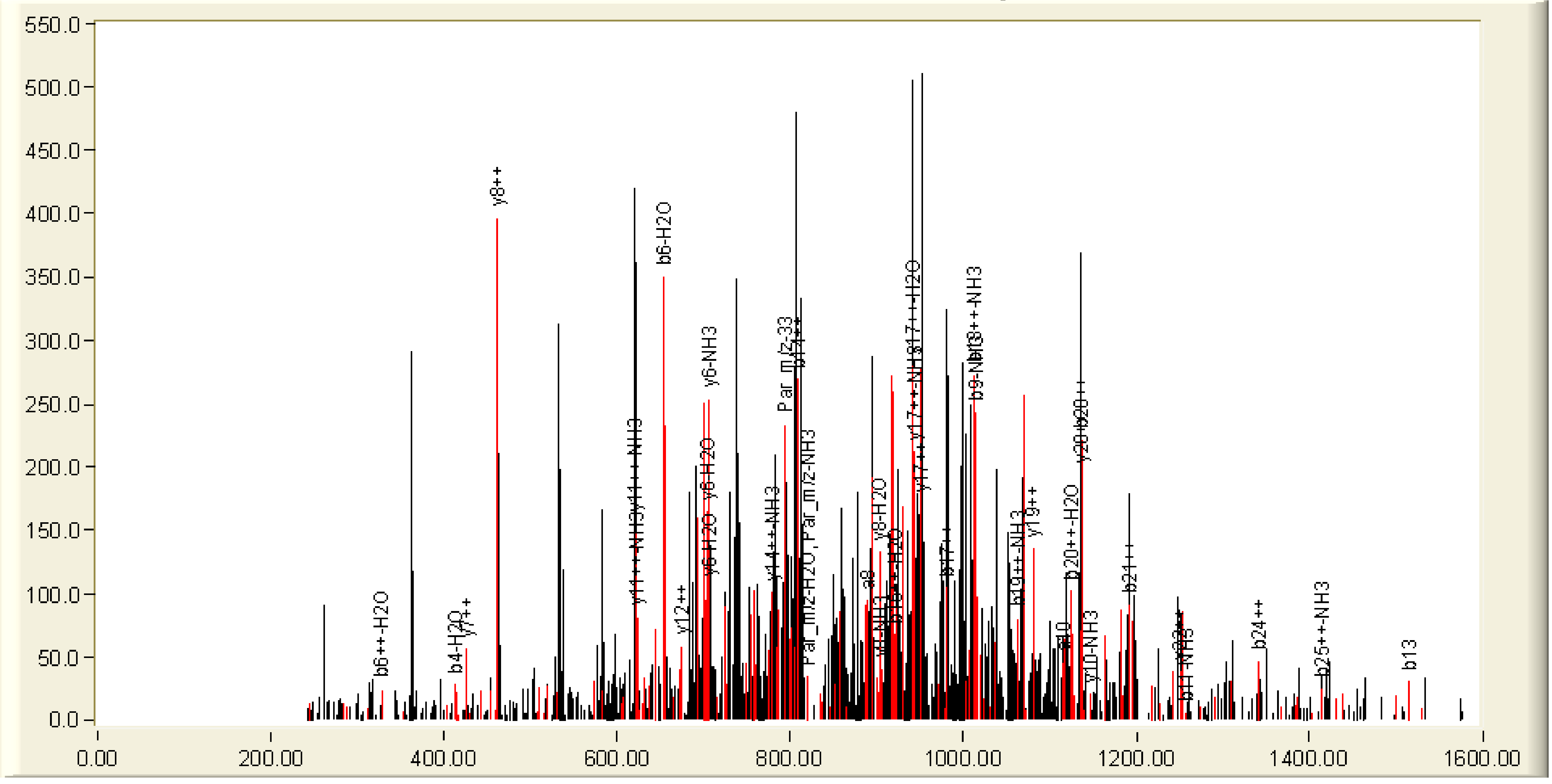

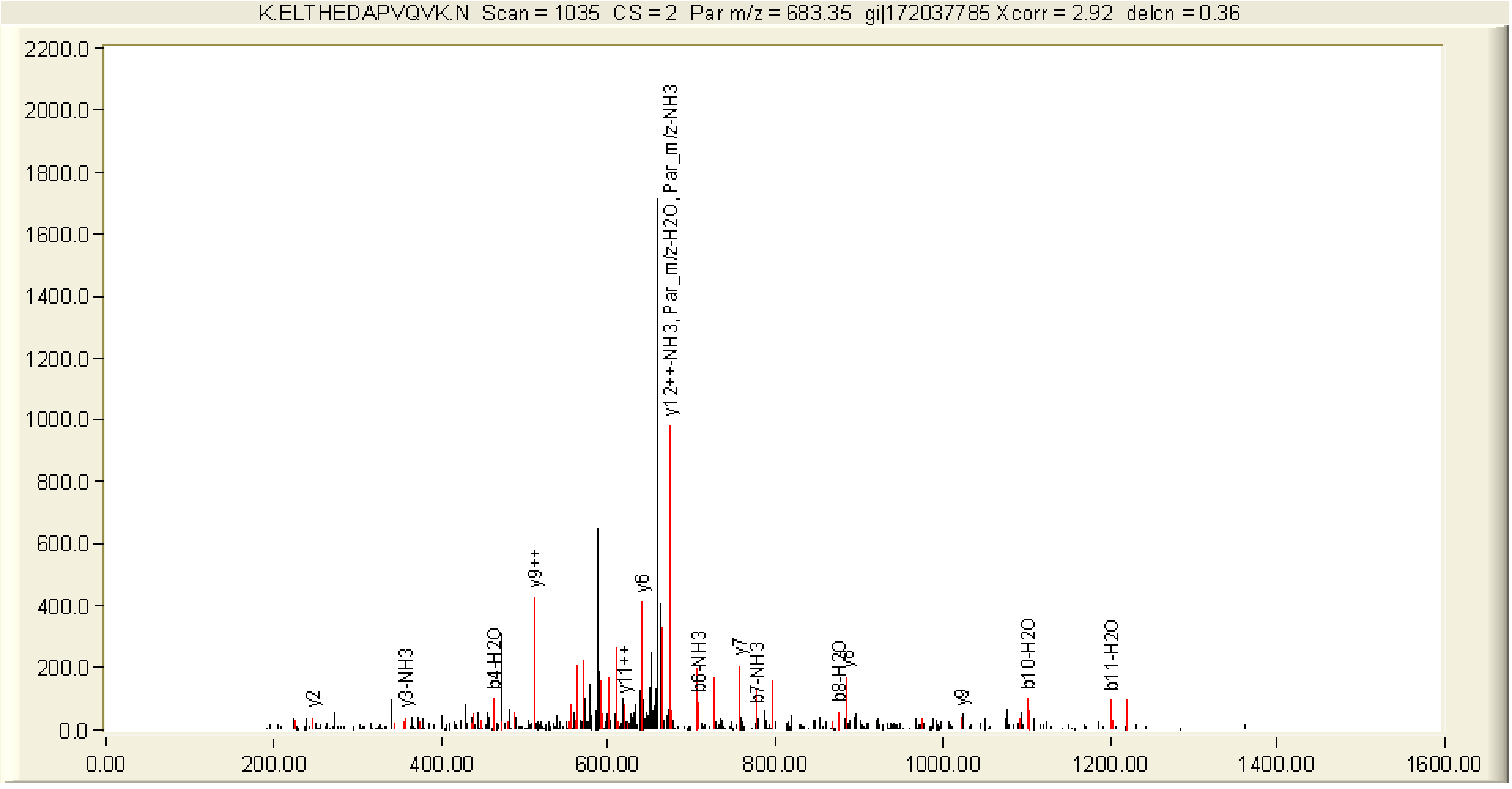

K.ELTHEDAPVQVK.N Scan = 1035 CS = 2 Par m/z = 683.35 gi|172037785 Xcorr = 2.92 delcn = 0.36
y12++-NH3, Par_m/z-H2O, Par_m/z-NH3
y9++
y6
y11++
y2
y3-NH3
b4-H2O
b6-NH3
y7
b7-NH3
b8-H2O
y8
y9
b10-H2O
b11-H2O
0.0
200.0
400.0
600.0
800.0
1000.0
1200.0
1400.0
1600.0
1800.0
2000.0
2200.0
0.00
200.00
400.00
600.00
800.00
1000.00
1200.00
1400.00
1600.00

R.GIENSDYPWLILAPACNKQAGLELVAFQPGTMK.P Scan = 1037 CS = 3 Par m/z = 1191.92 gi|172035972 Xcorr = 3.42 delcn = 0.11

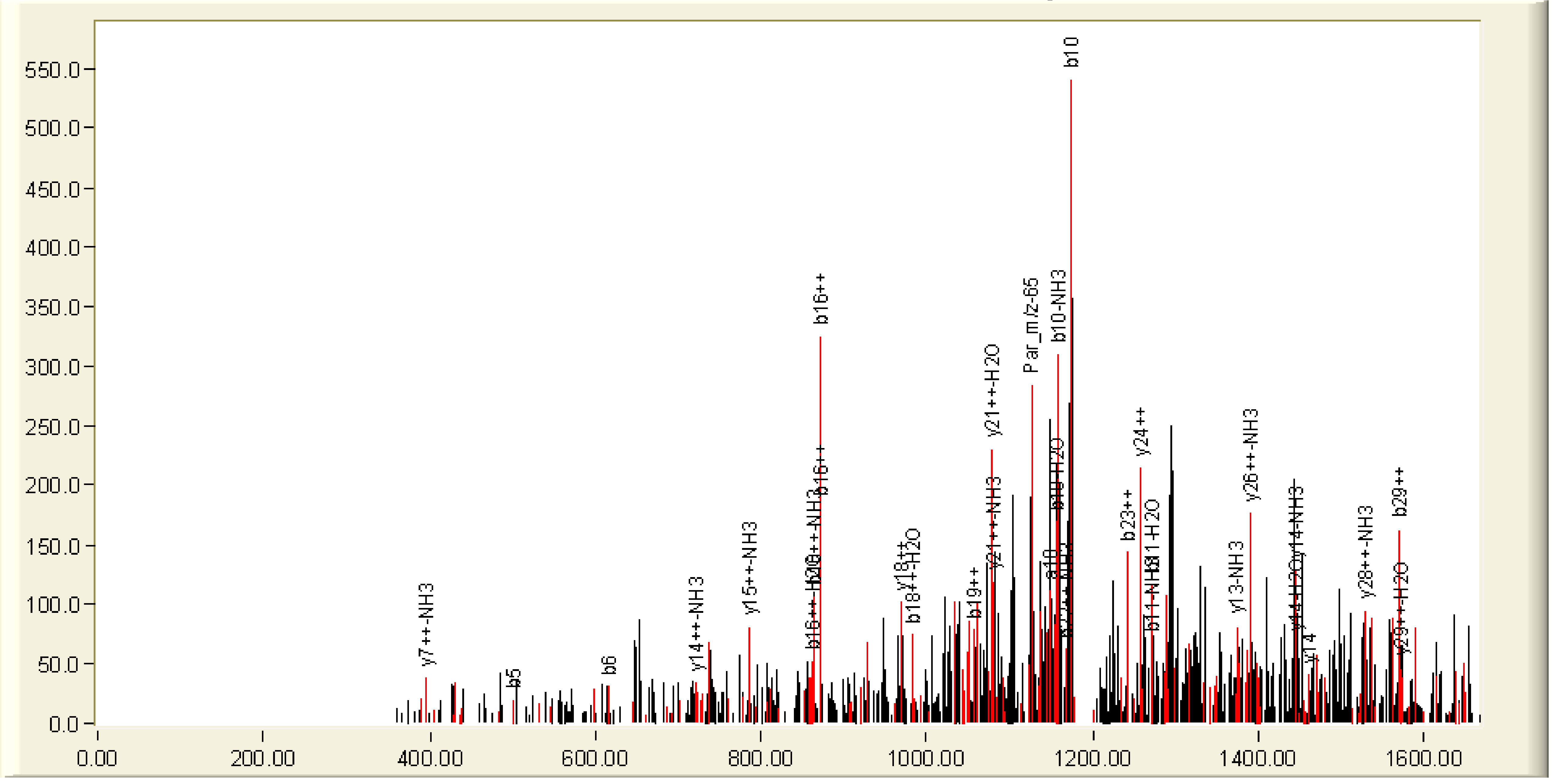

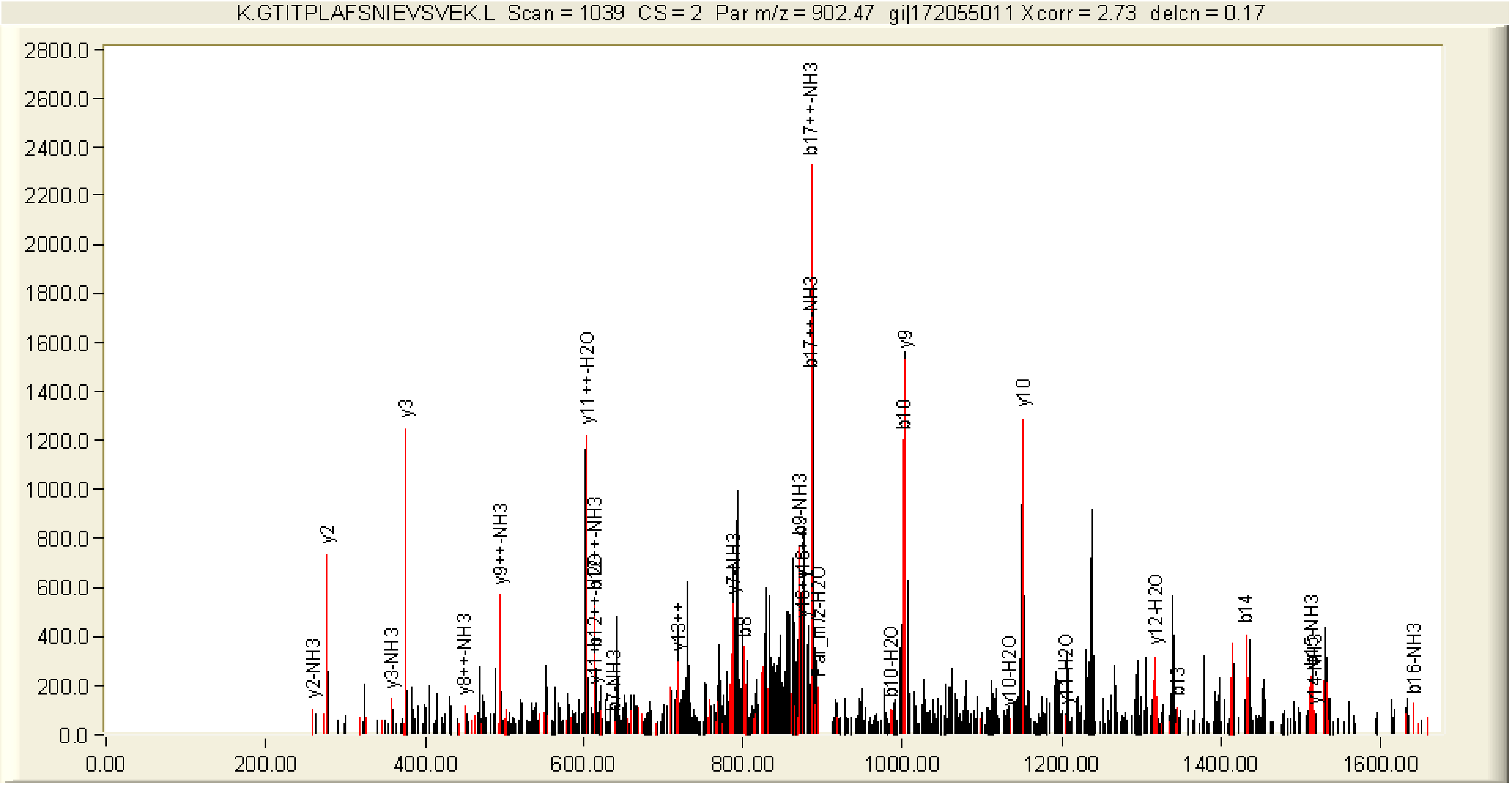

K.GTITPLAFSNIEVSVEK.L Scan = 1039 CS = 2 Par m/z = 902.47 gi|172055011 Xcorr = 2.73 delcn = 0.17
b17++-NH3
y9
b10
y10
y3
y11++-H2O
y2
y9++-NH3
y8++-NH3
y3-NH3
y2-NH3
y13++
y7-NH3
b9-NH3
y12-H2O
b14
b16-NH3
b13
b10-H2O
y10-H2O
0.00
200.00
400.00
600.00
800.00
1000.00
1200.00
1400.00
1600.00
0.0
200.0
400.0
600.0
800.0
1000.0
1200.0
1400.0
1600.0
1800.0
2000.0
2200.0
2400.0
2600.0
2800.0

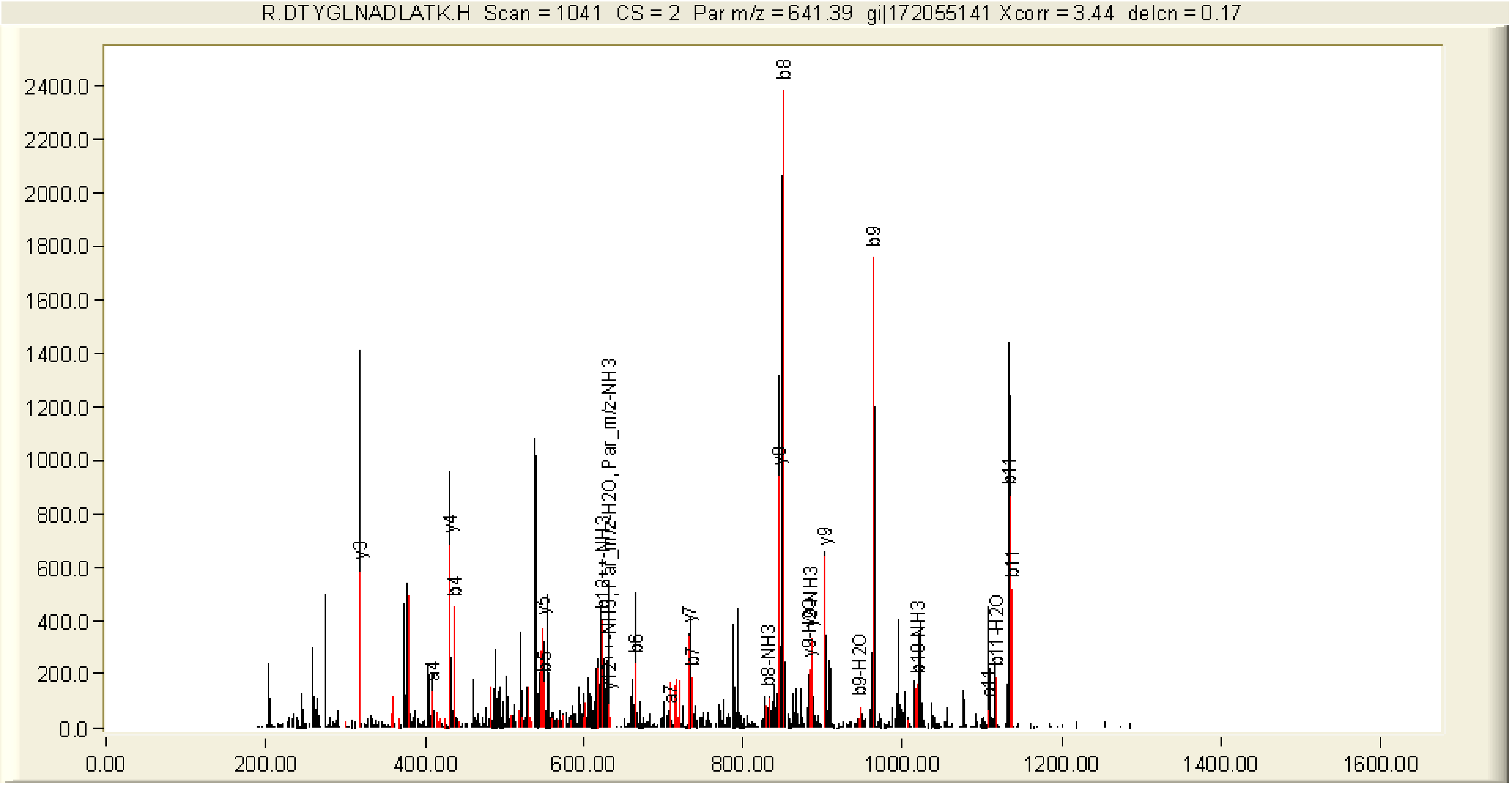

R.DTYGLNADLATK.H Scan = 1041 CS = 2 Par m/z = 641.39 gi|172055141 Xcorr = 3.44 delcn = 0.17
b8
b9
y3
y4
b4
a4
y5
b5
b6
a7
b7
y7
y8
y9
b8-NH3
y9-H2O
b9-H2O
b10-NH3
b11
b11-H2O
a11
2400.0
2200.0
2000.0
1800.0
1600.0
1400.0
1200.0
1000.0
800.0
600.0
400.0
200.0
0.0
0.00
200.00
400.00
600.00
800.00
1000.00
1200.00
1400.00
1600.00

K.FYTCSFTTEDHQRLCSLLQKNK.S Scan = 1043 CS = 3 Par m/z = 887.77 gi|172034931 Xcorr = 3.36 delcn = 0.14

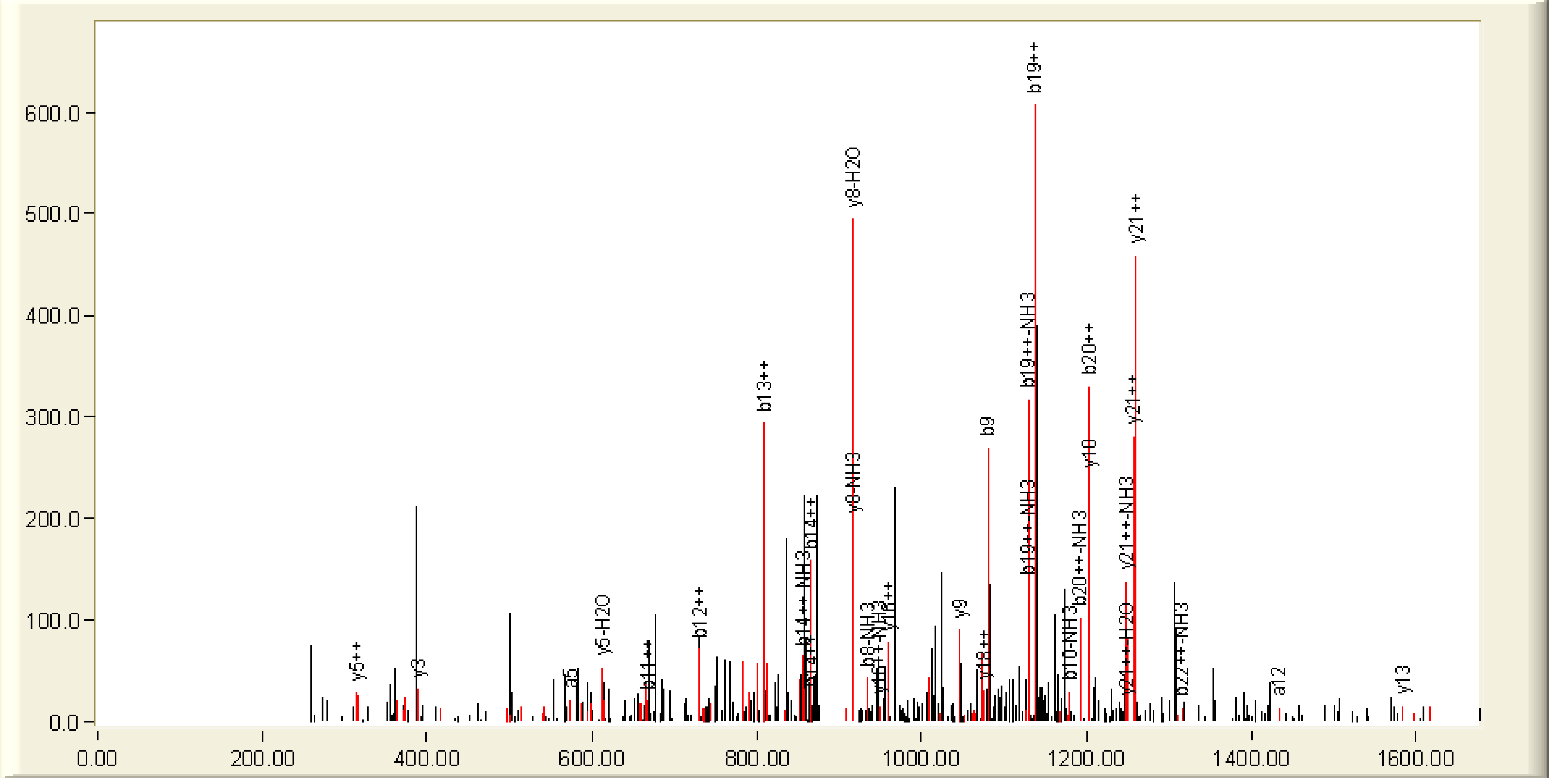

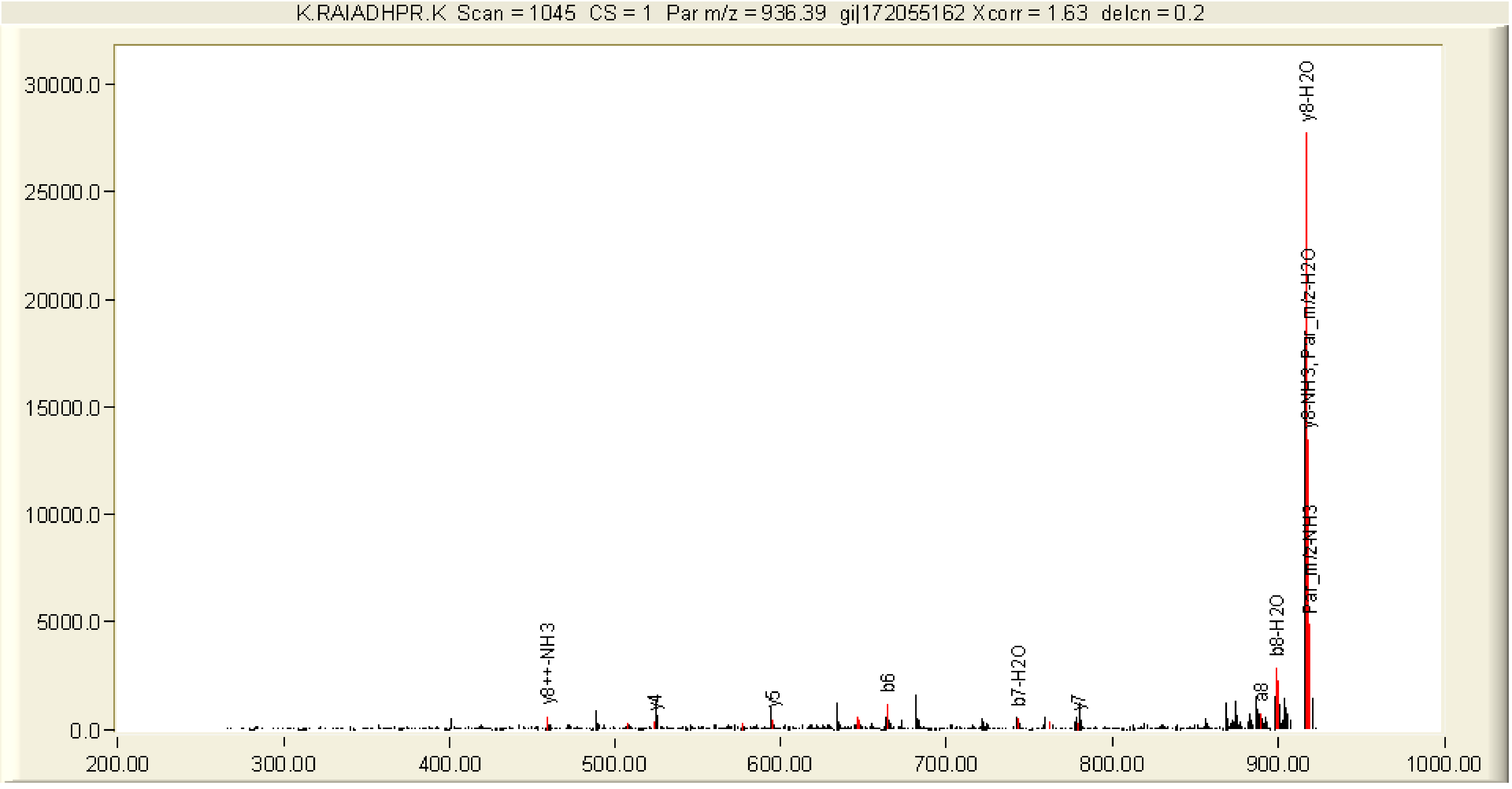
K.RAIADHPR.K Scan = 1045 CS = 1 Par m/z = 936.39 gi|172055162 Xcorr = 1.63 delcn = 0.2
30000.0
25000.0
20000.0
15000.0
10000.0
5000.0
0.0
200.00
300.00
400.00
500.00
600.00
700.00
800.00
900.00
1000.00
y8++-NH3
y4
y5
b6
b7-H2O
y7
a8
b8-H2O
Par_m/z-NH3
y8-NH3, Par_m/z-H2O
y8-H2O

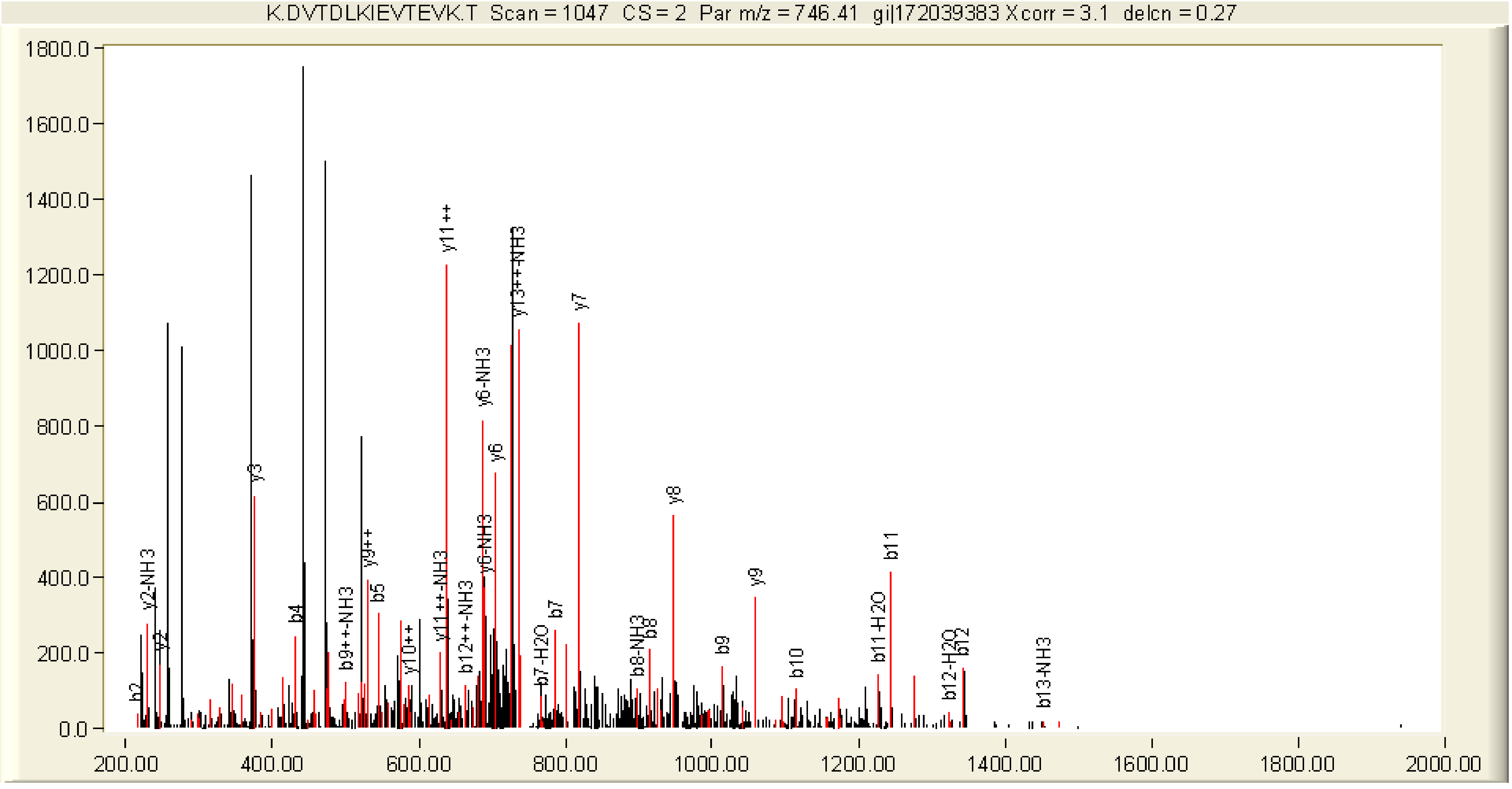
K.DVTDLKIEVTEVK.T Scan = 1047 CS = 2 Par m/z = 746.41 gi|172039383 Xcorr = 3.1 delcn = 0.27
1800.0
1600.0
1400.0
1200.0
1000.0
800.0
600.0
400.0
200.0
0.0
200.00
400.00
600.00
800.00
1000.00
1200.00
1400.00
1600.00
1800.00
2000.00
b2
y2-NH3
y2
y3
b4
b9++-NH3
y9++
b5
y10++
y11++-NH3
y11++
b12++-NH3
y6-NH3
y6-NH3
y6
y13++-NH3
b7-H2O
b7
y7
b8-NH3
b8
y8
b9
y9
b10
b11-H2O
b11
b12-H2O
b12
b13-NH3

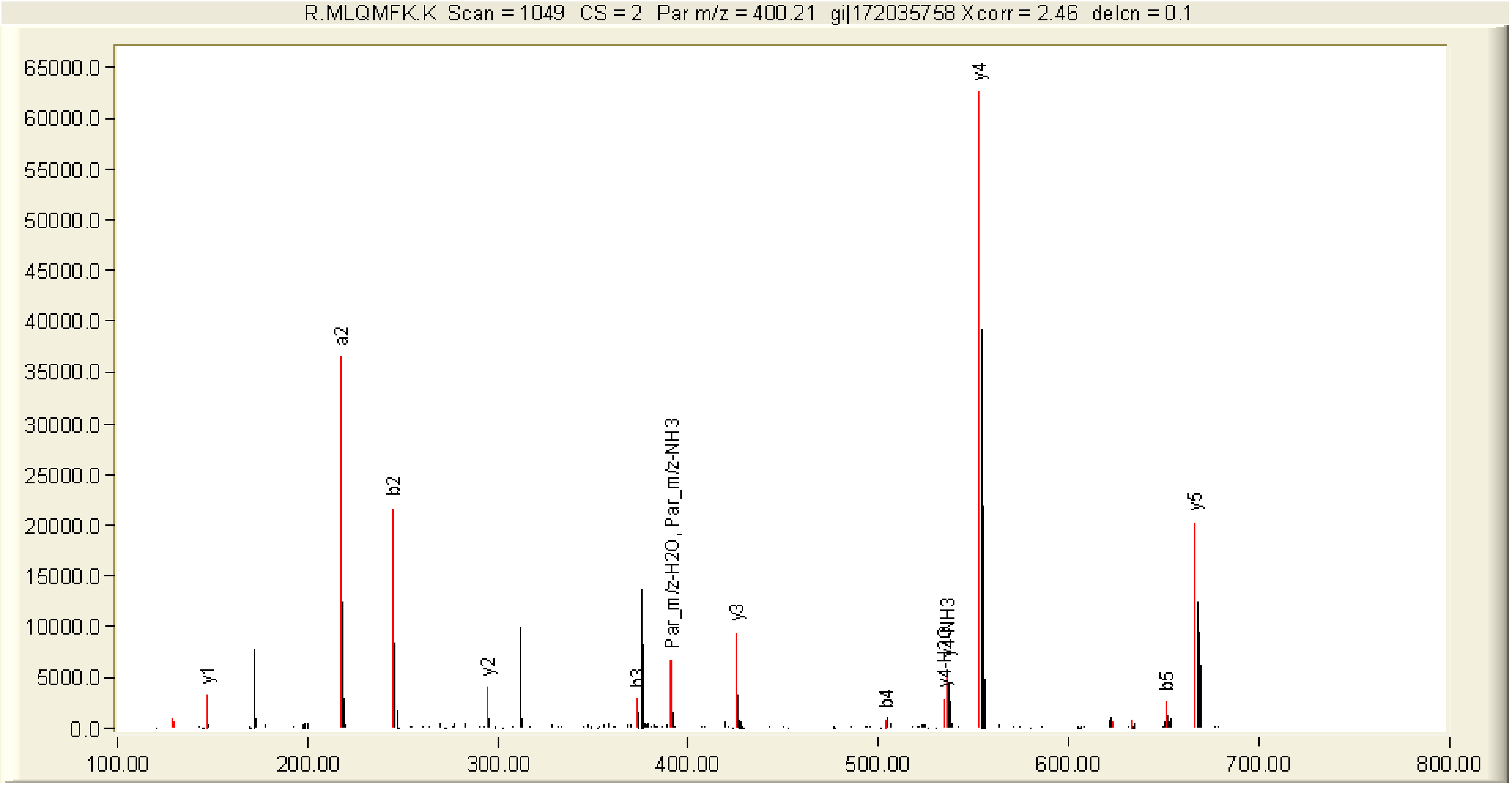
R.MLQMFK.K Scan = 1049 CS = 2 Par m/z = 400.21 gi|172035758 Xcorr = 2.46 delcn = 0.1
65000.0
60000.0
55000.0
50000.0
45000.0
40000.0
35000.0
30000.0
25000.0
20000.0
15000.0
10000.0
5000.0
0.0
100.00
200.00
300.00
400.00
500.00
600.00
700.00
800.00
y1
a2
b2
y2
b3
Par_m/z-H2O, Par_m/z-NH3
y3
b4
y4
b5
y5

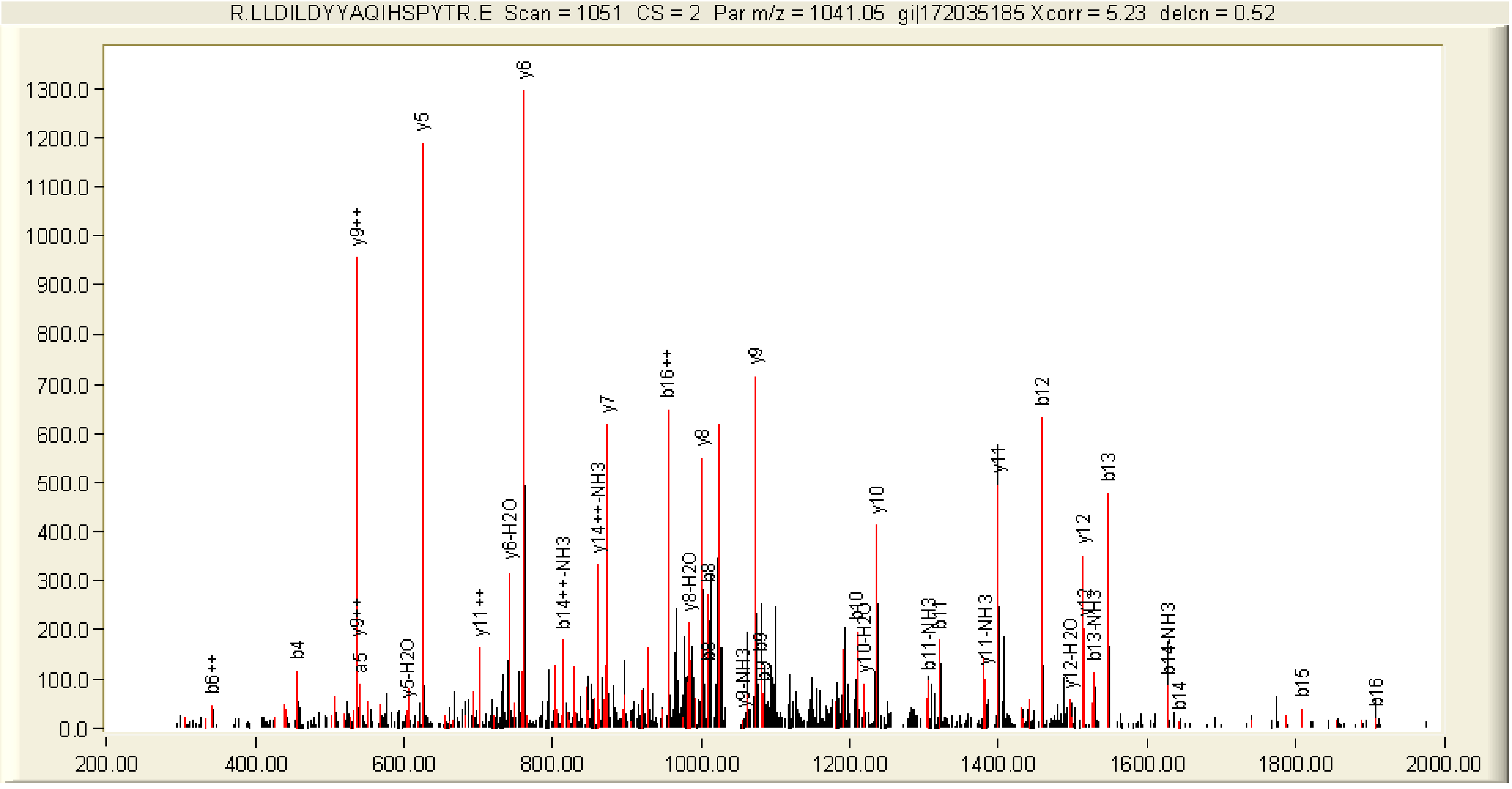
R.LLDILDYYAQIHSPYTR.E Scan = 1051 CS = 2 Par m/z = 1041.05 gi|172035185 Xcorr = 5.23 delcn = 0.52
1300.0
1200.0
1100.0
1000.0
900.0
800.0
700.0
600.0
500.0
400.0
300.0
200.0
100.0
0.0
200.00
400.00
600.00
800.00
1000.00
1200.00
1400.00
1600.00
1800.00
2000.00
y6
y5
y9++
y9
b16++
y7
b12
y8
y11
b13
y10
y12
y6-H2O
y14++-NH3
b14++-NH3
y8-H2O
b8
y11++
y9++
a5
b4
b6++
y5-H2O
b10
y10-H2O
b11-NH3
b11
y11-NH3
y12-H2O
b13-NH3
b14-NH3
b14
b15
b16
y9-NH3

KASGVAVQEVSQVALLSIDEFVRDELNNAAIK.F Scan = 1053 CS = 3 Par m/z = 1096.59 gi|172035438 Xcorr = 3.81 delcn = 0.24

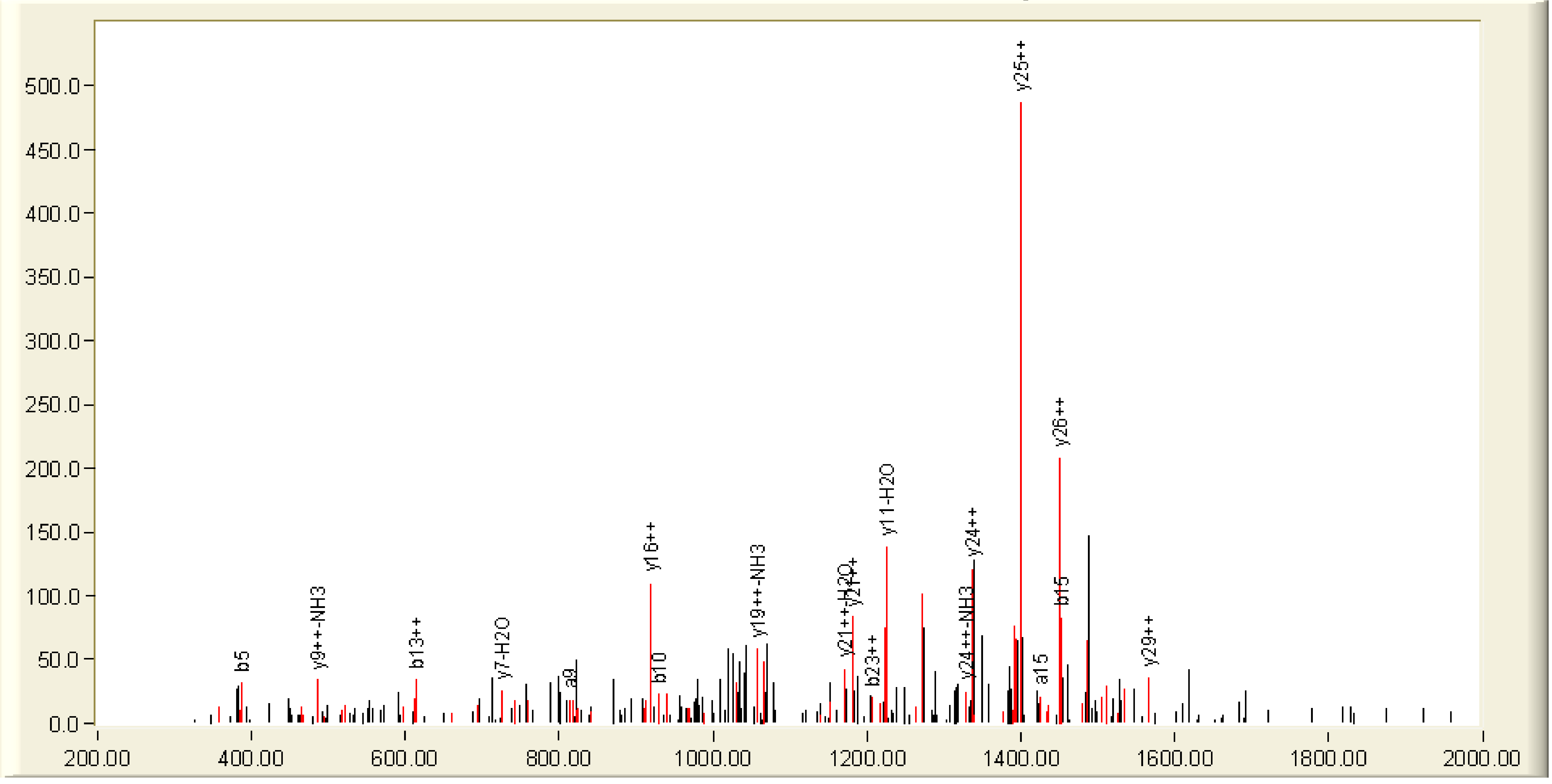

K.RGYIHNASCFNTATIADLITTDGSK.L Scan = 1055 CS = 3 Par m/z = 891.11 gi|172038593 Xcorr = 3.4 delcn = 0.15

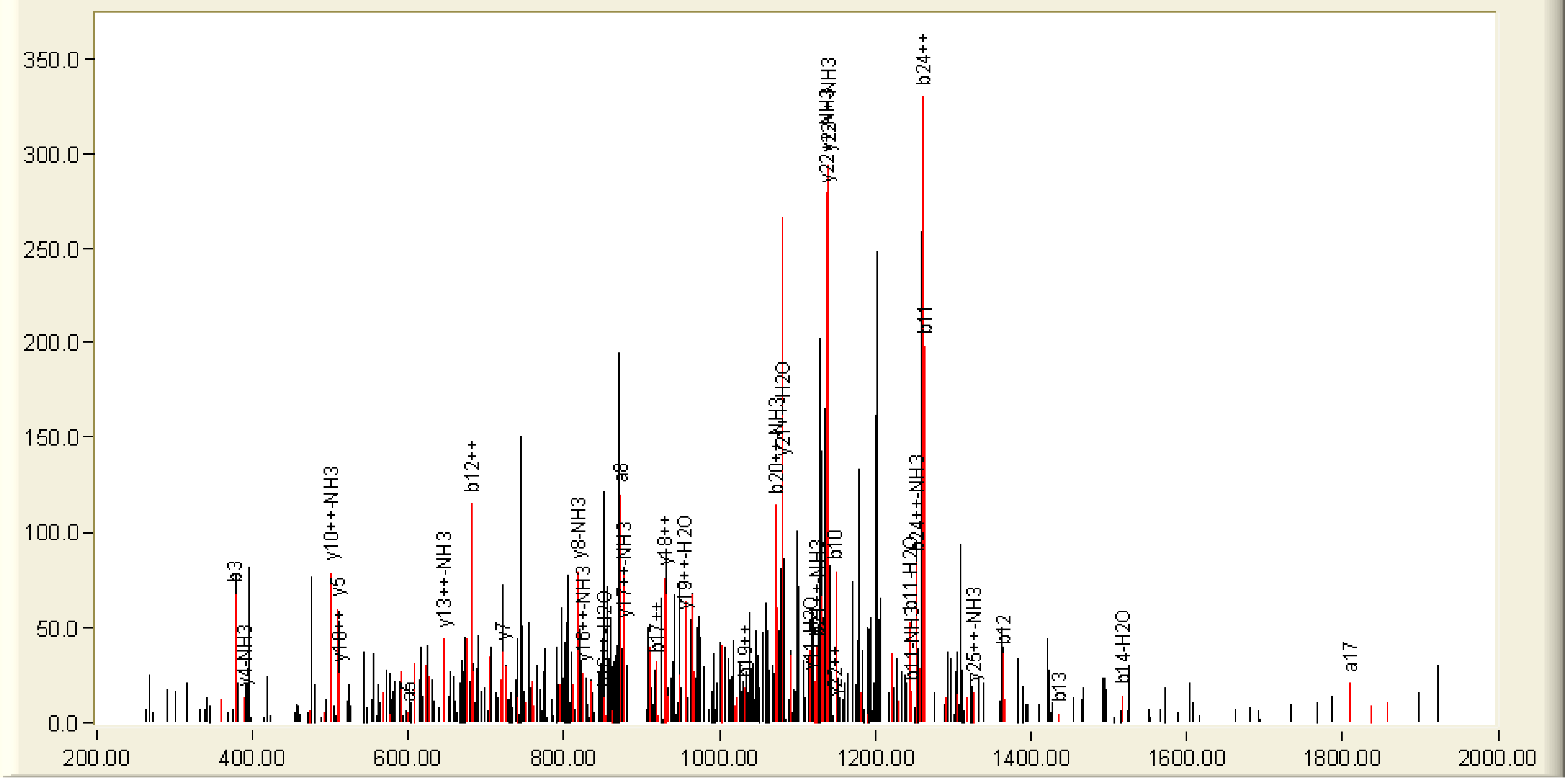

-.MTNTDFLKEFDDDITVSESIPYCQITNPD.N Scan = 1057 CS = 4 Par m/z = 838.43 gi|172054869 Xcorr = 2.79 delcn = 0.12

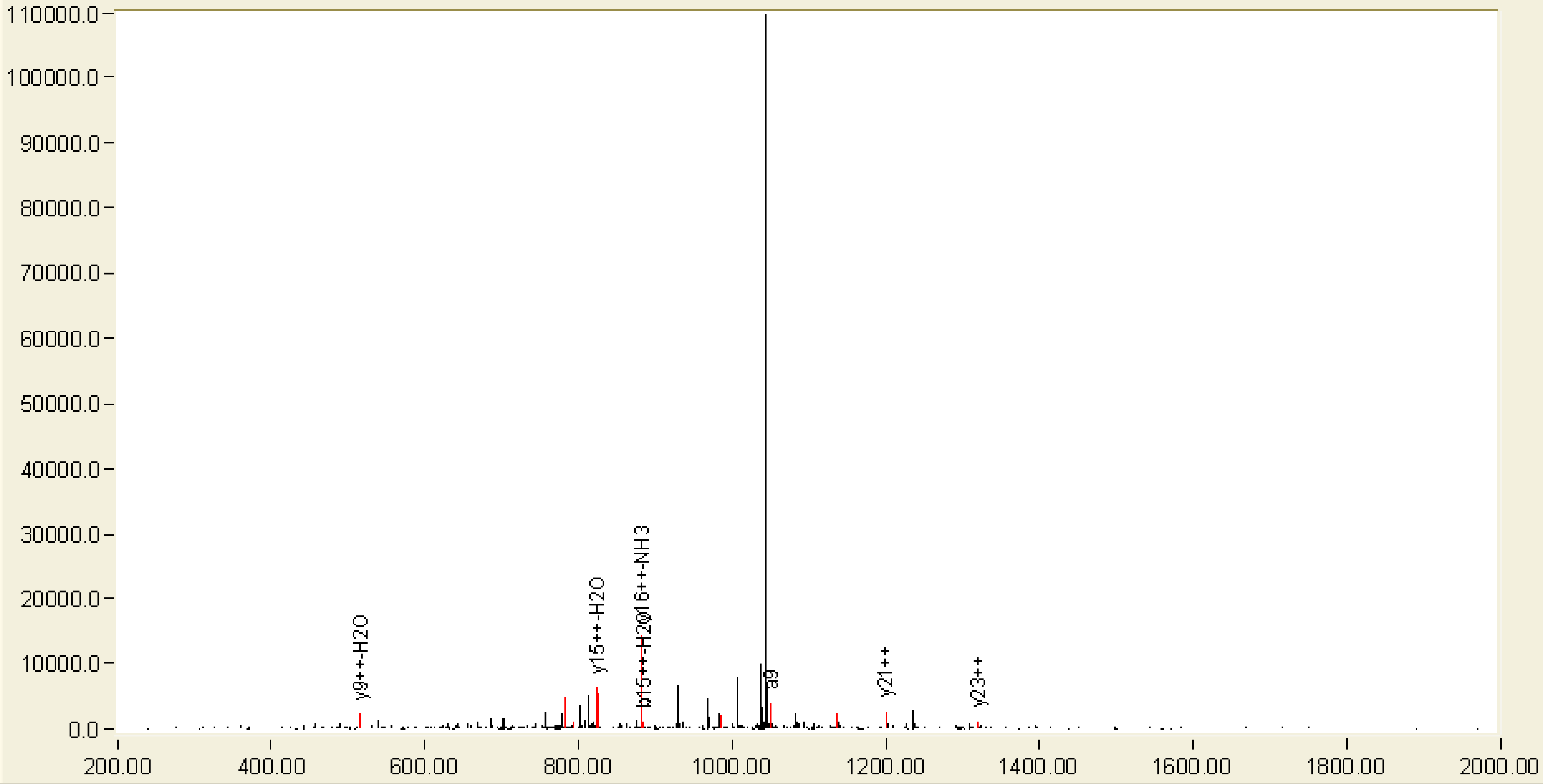

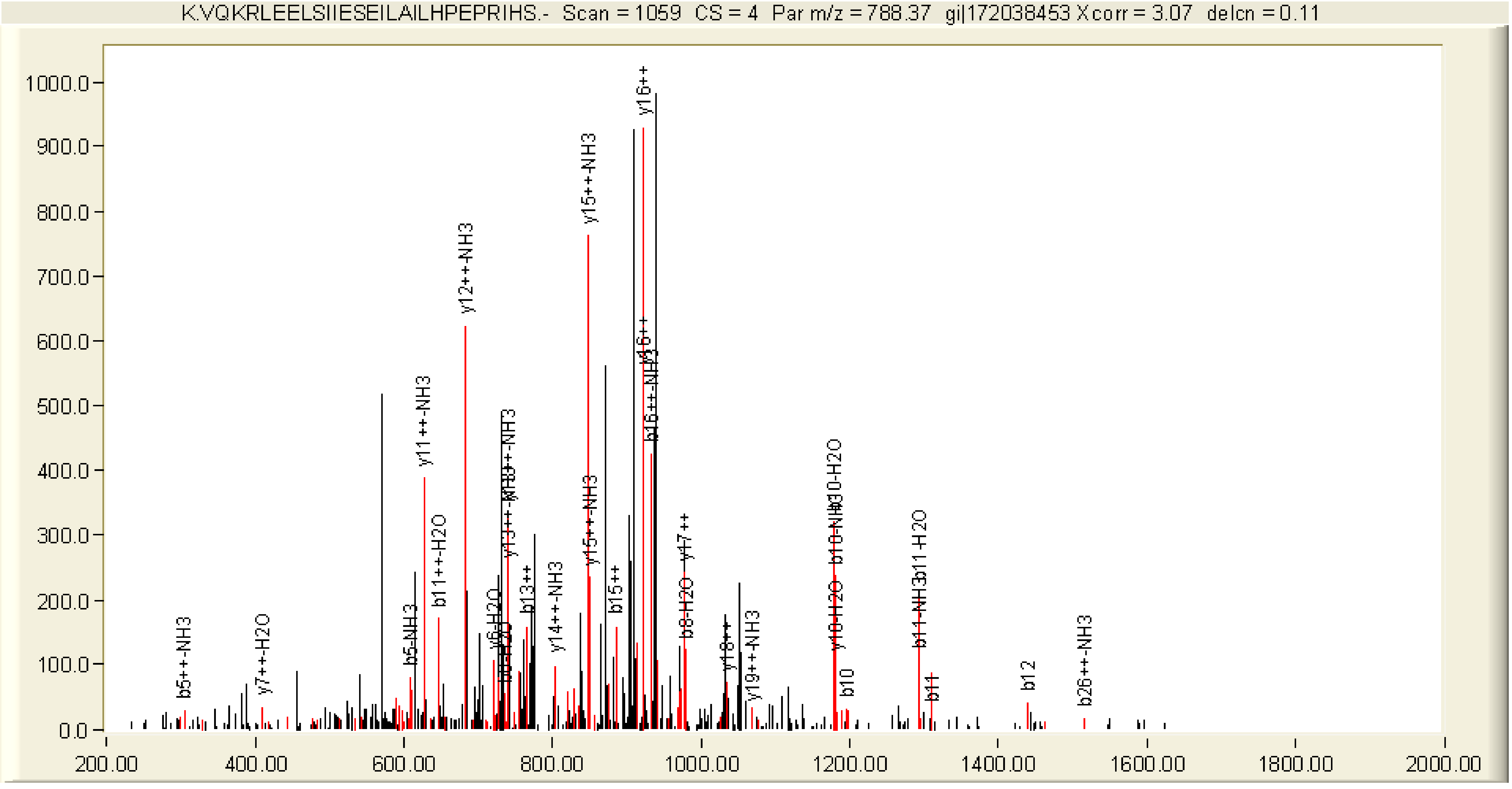
K.VQKRLEELSIIESEILAILHPEPRIHS.- Scan = 1059 CS = 4 Par m/z = 788.37 gi|172038453 Xcorr = 3.07 delcn = 0.11
1000.0
900.0
800.0
700.0
600.0
500.0
400.0
300.0
200.0
100.0
0.0
200.00
400.00
600.00
800.00
1000.00
1200.00
1400.00
1600.00
1800.00
2000.00
b5++-NH3
y7++-H2O
b5-NH3
y11++-NH3
b11++-H2O
y12++-NH3
y15++-NH3
y16++
b13++
y14++-NH3
y15++-NH3
b15++
b8-H2O
y17++
y18++
y19++-NH3
b10-H2O
b10
b11-NH3
b11-H2O
b11
b12
b26++-NH3

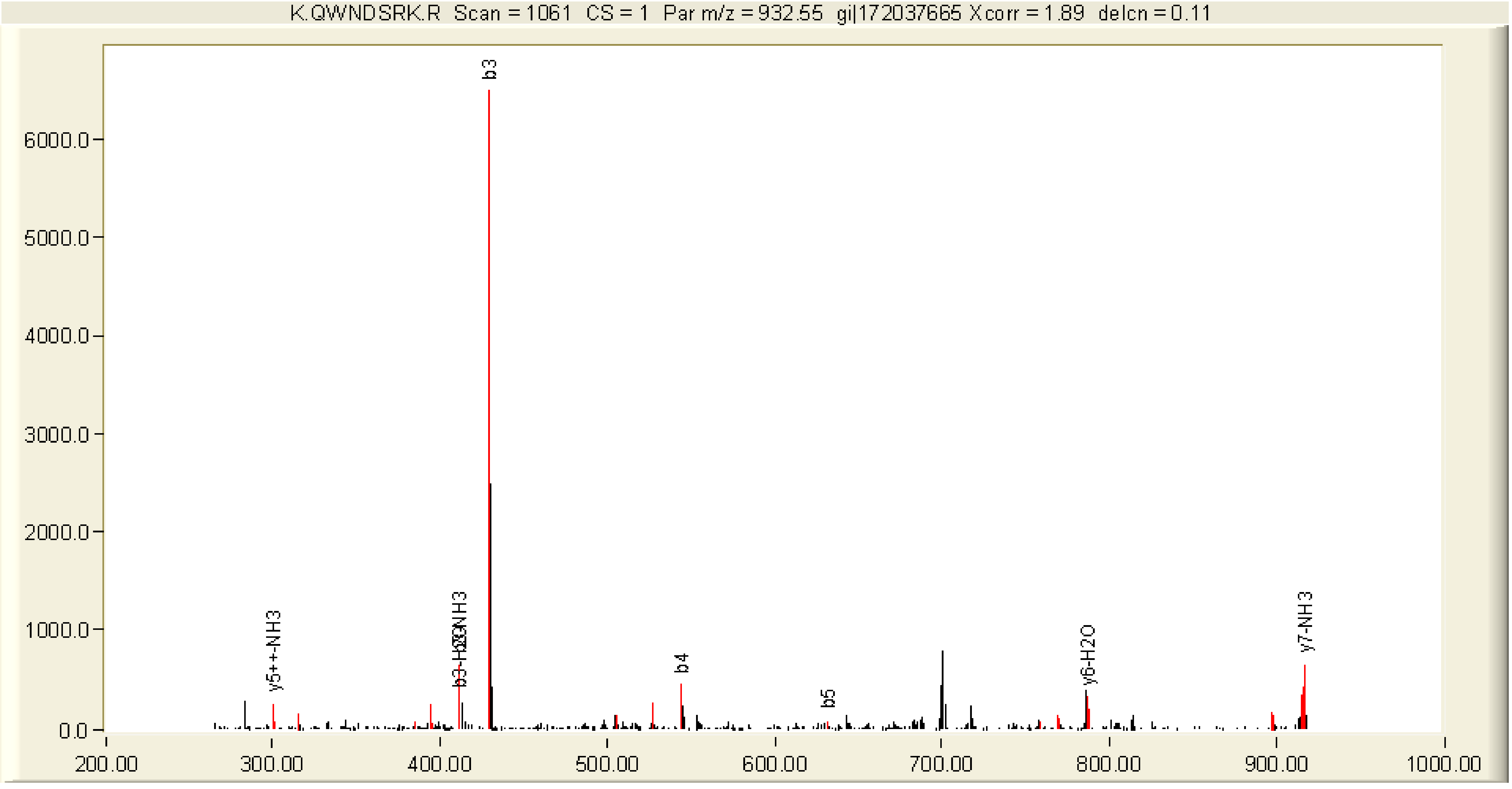

K.QWNDSRK.R Scan = 1061 CS = 1 Par m/z = 932.55 gi|172037665 Xcorr = 1.89 delcn = 0.11
b3
y5++-NH3
b3-NH3
b4
b5
y6-H2O
y7-NH3
6000.0
5000.0
4000.0
3000.0
2000.0
1000.0
0.0
200.00
300.00
400.00
500.00
600.00
700.00
800.00
900.00
1000.00

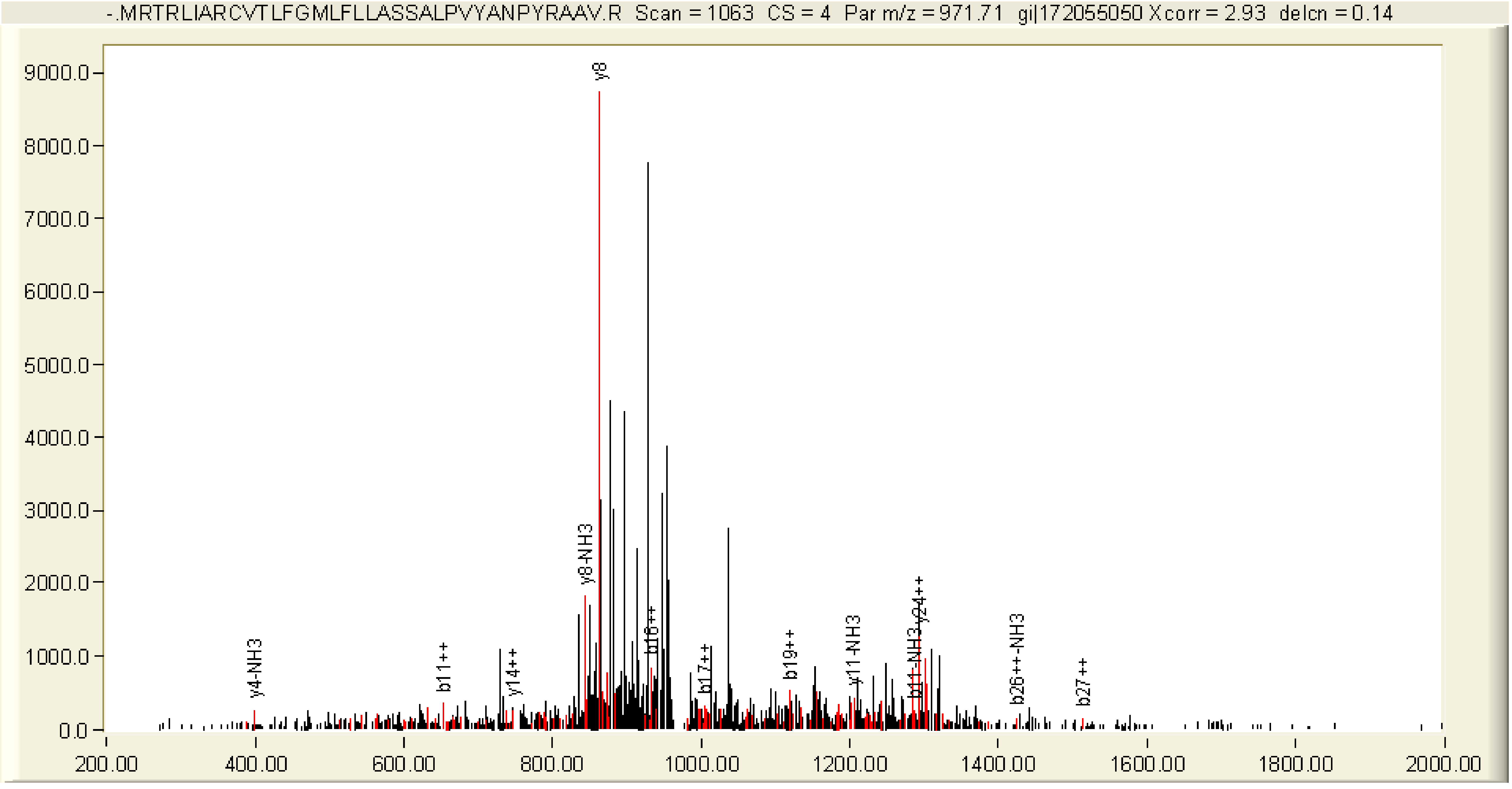

-.MRTRLIARCVTLFGMLFLLASSALPVYANPYRAAV.R Scan = 1063 CS = 4 Par m/z = 971.71 gi|172055050 Xcorr = 2.93 delcn = 0.14
9000.0
8000.0
7000.0
6000.0
5000.0
4000.0
3000.0
2000.0
1000.0
0.0
200.00
400.00
600.00
800.00
1000.00
1200.00
1400.00
1600.00
1800.00
2000.00
y8
y8-NH3
y4-NH3
b11++
y14++
b16++
b17++
b19++
y11-NH3
b11-NH3
y24++
b26++-NH3
b27++

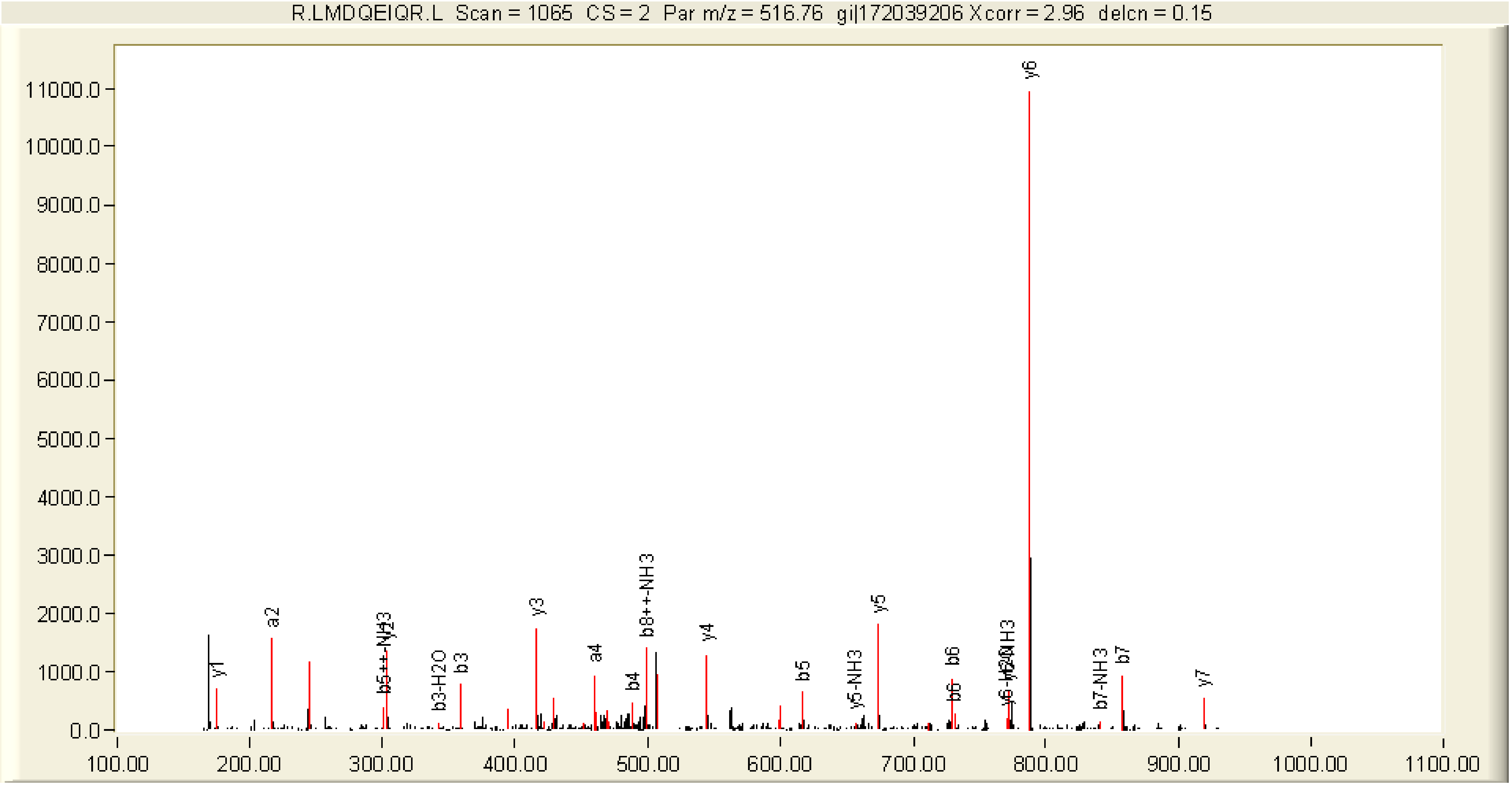

R.LMDQEIQR.L Scan = 1065 CS = 2 Par m/z = 516.76 gi|172039206 Xcorr = 2.96 delcn = 0.15
y6
y1
a2
b3-H2O
b3
y3
a4
b4
b8++-NH3
y4
b5
y5-NH3
y5
b6
b7-NH3
b7
y7

K.NIAIAFILLAASPAPPLLTK.R Scan = 1067 CS = 2 Par m/z = 1018.54 gi|172038756 Xcorr = 3.2 delcn = 0.12

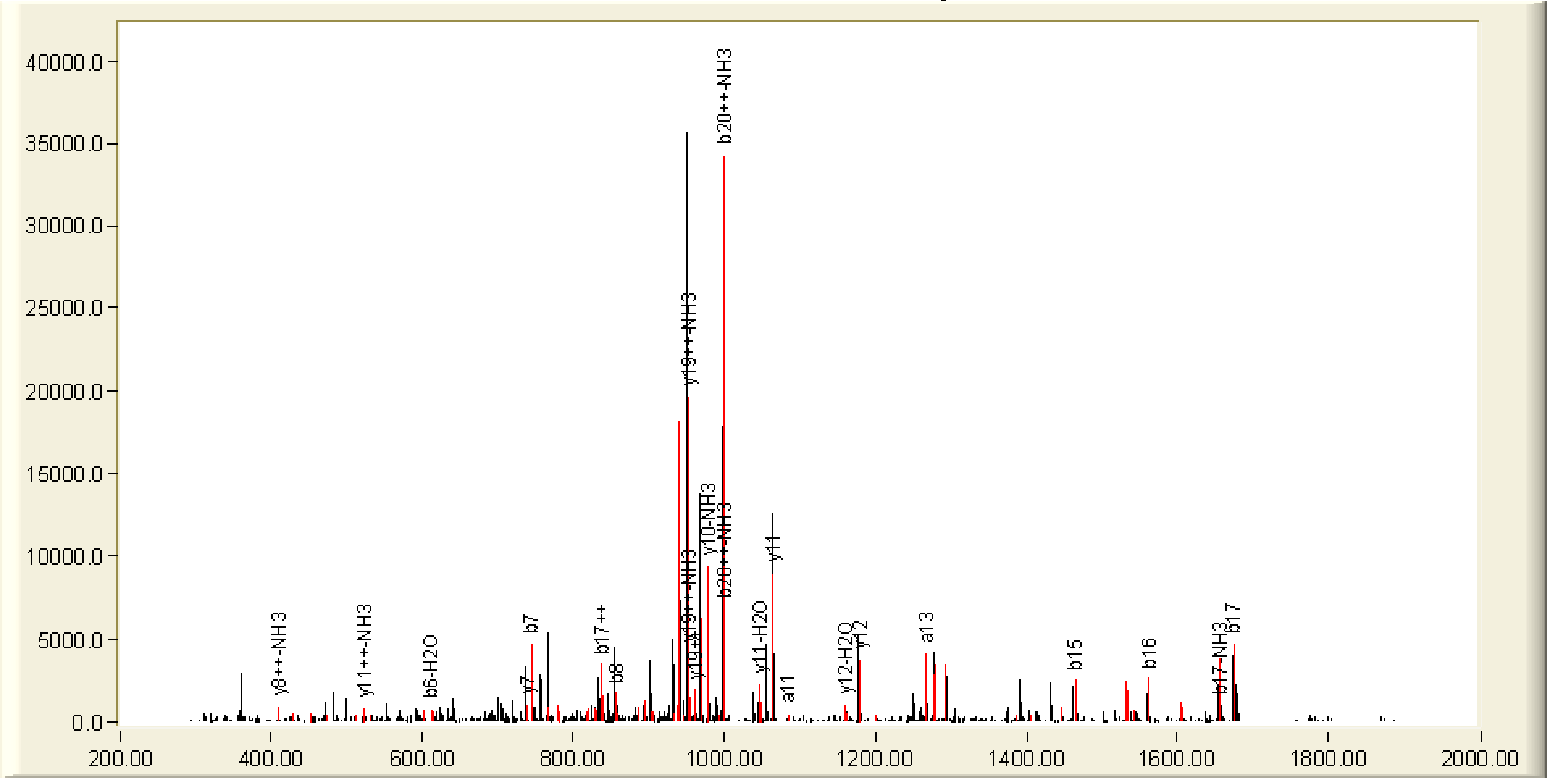

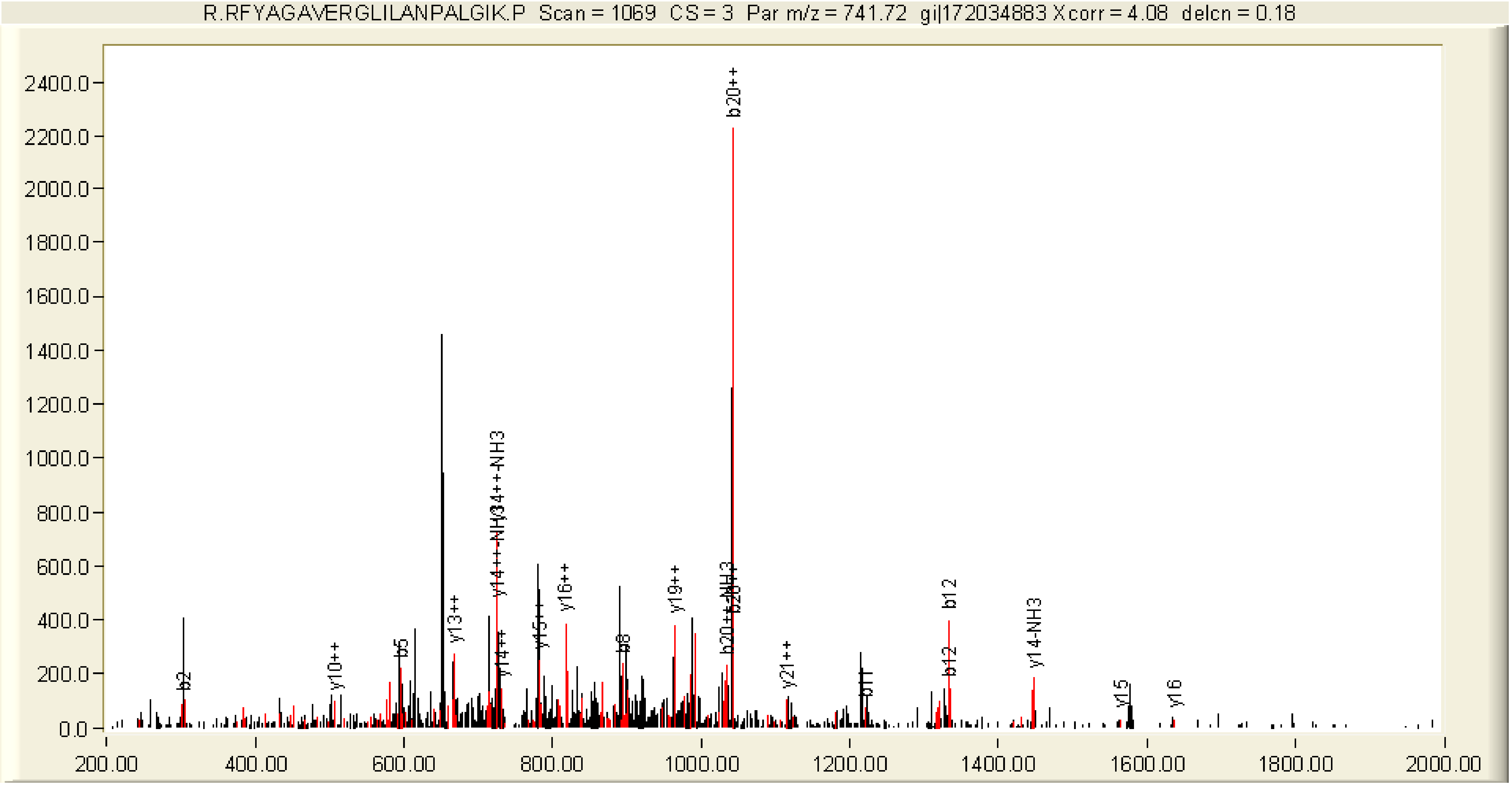
R.RFYAGAVERGLILANPALGIK.P Scan = 1069 CS = 3 Par m/z = 741.72 gi|172034883 Xcorr = 4.08 delcn = 0.18
2400.0
2200.0
2000.0
1800.0
1600.0
1400.0
1200.0
1000.0
800.0
600.0
400.0
200.0
0.0
200.00
400.00
600.00
800.00
1000.00
1200.00
1400.00
1600.00
1800.00
2000.00
b20++
b2
y10++
b5
y13++
y14++-NH3
y14++
y15++
y16++
b8
y19++
b20++-NH3
y21++
b11
b12
y14-NH3
y15
y16

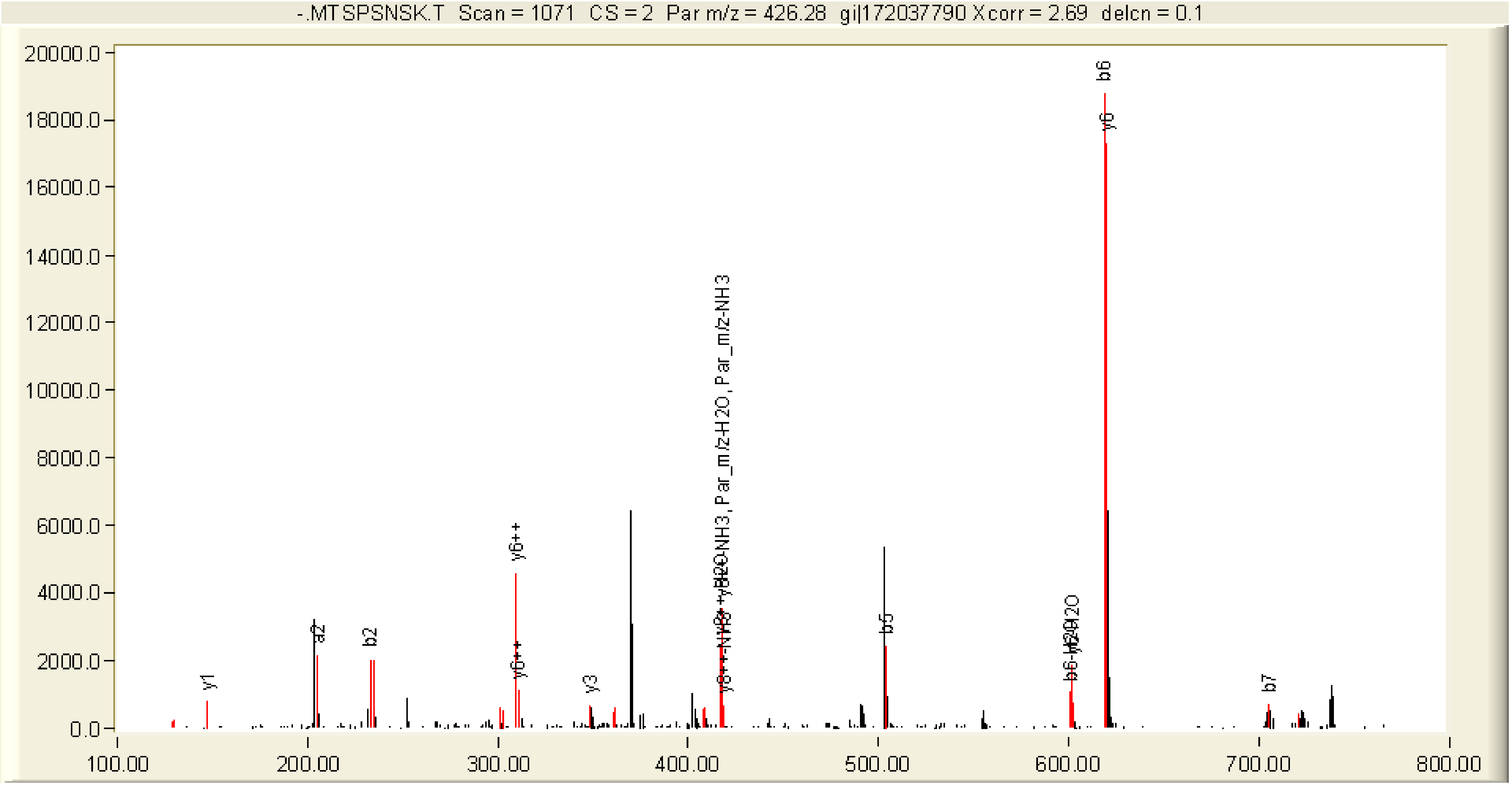

-.MTSPSNSK.T Scan = 1071 CS = 2 Par m/z = 426.28 gi|172037790 Xcorr = 2.69 delcn = 0.1
20000.0
18000.0
16000.0
14000.0
12000.0
10000.0
8000.0
6000.0
4000.0
2000.0
0.0
100.00
200.00
300.00
400.00
500.00
600.00
700.00
800.00
y1
a2
b2
y6++
y3
b5
b6
y6
b7
Par_m/z-NH3

-.MSVLNILHLVGSADNEFYCDLSR.L Scan = 1073 CS = 3 Par m/z = 865.13 gi|172038031 Xcorr = 3.27 delcn = 0.17

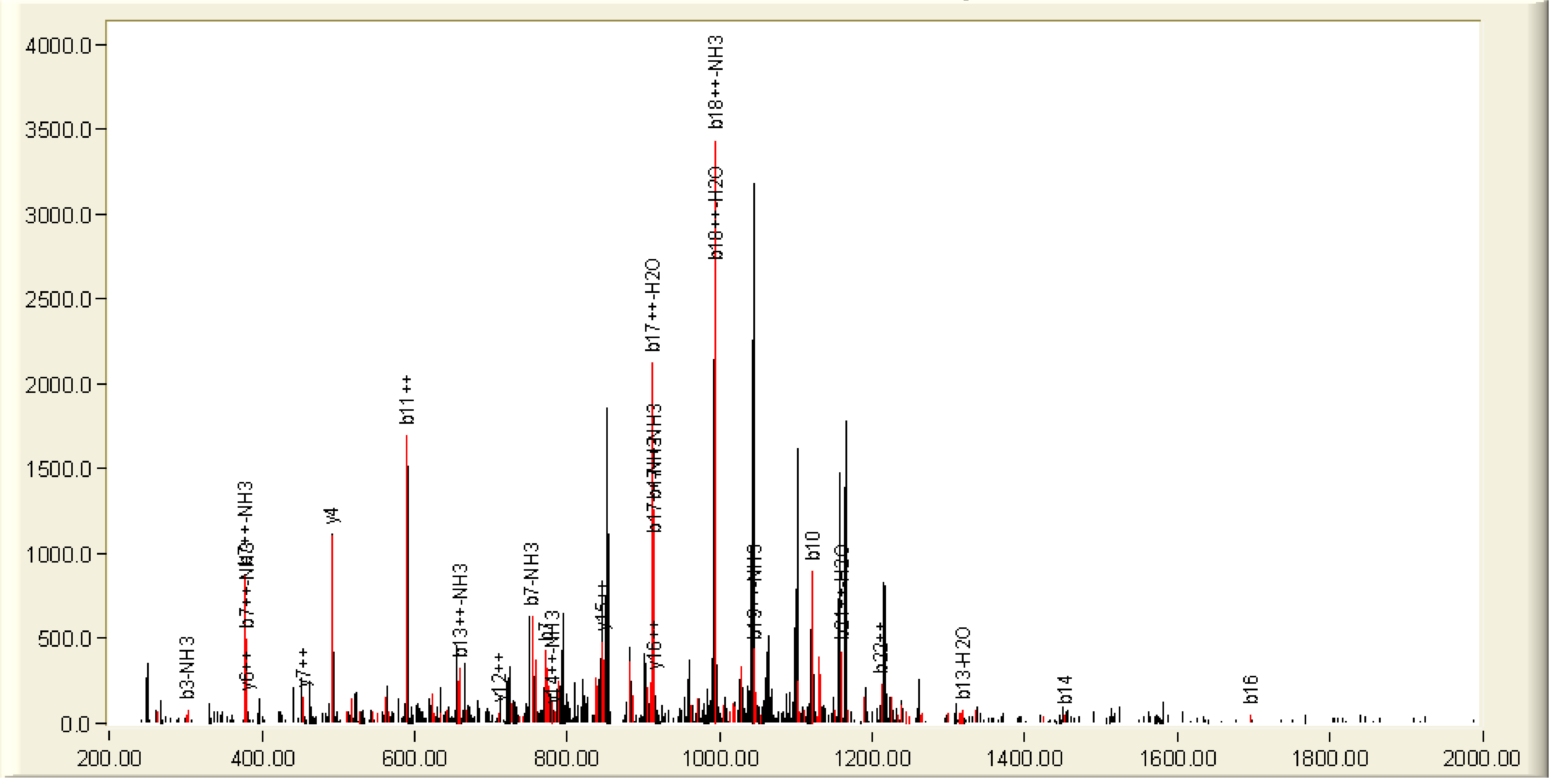

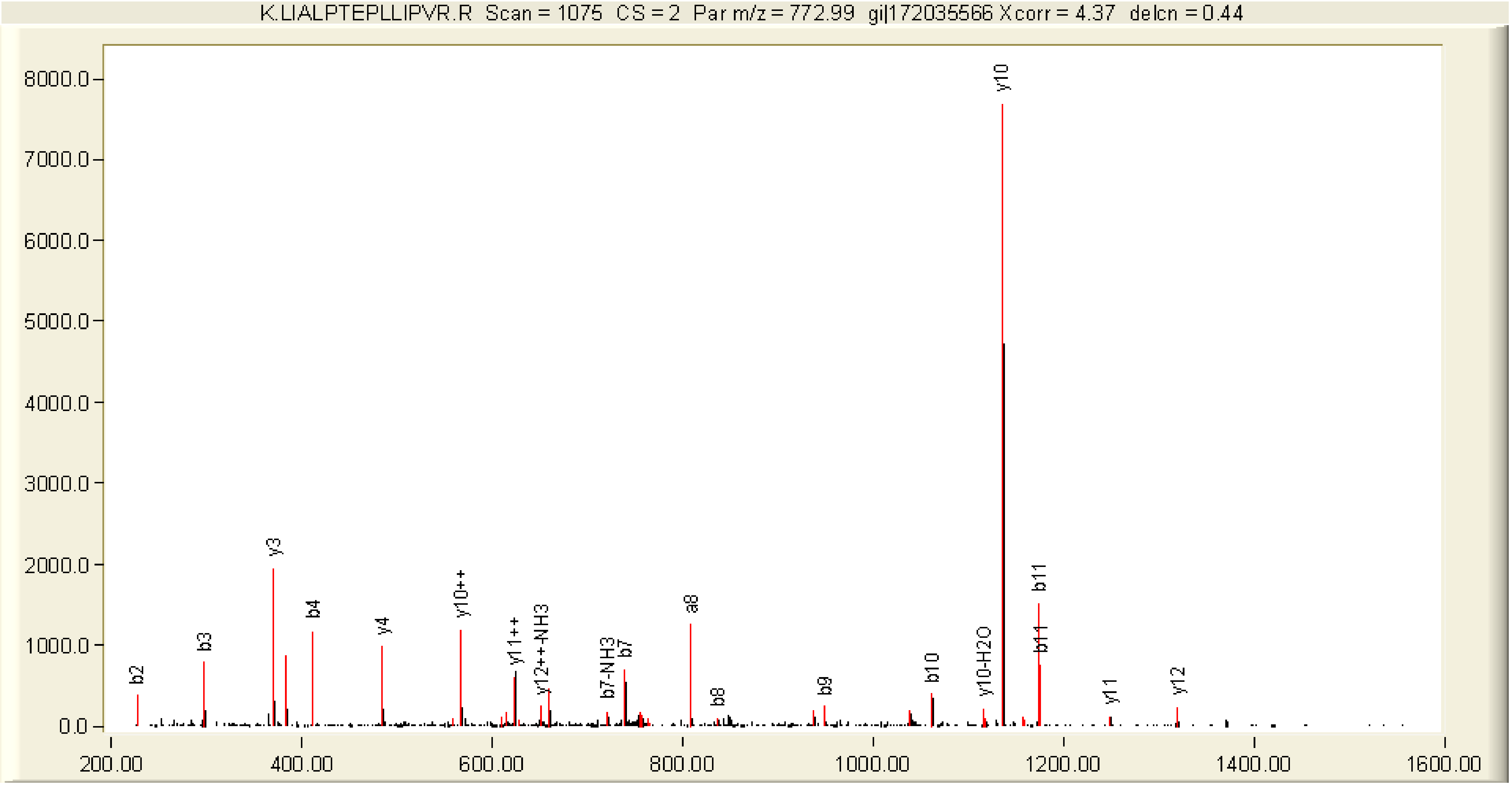

K.LIALPTEPLLIPVR.R Scan = 1075 CS = 2 Par m/z = 772.99 gi|172035566 Xcorr = 4.37 delcn = 0.44
8000.0
7000.0
6000.0
5000.0
4000.0
3000.0
2000.0
1000.0
0.0
200.00
400.00
600.00
800.00
1000.00
1200.00
1400.00
1600.00
b2
b3
y3
b4
y4
y10++
y11++
y12++-NH3
b7-NH3
b7
a8
b8
b9
b10
y10-H2O
y10
b11
b11
y11
y12

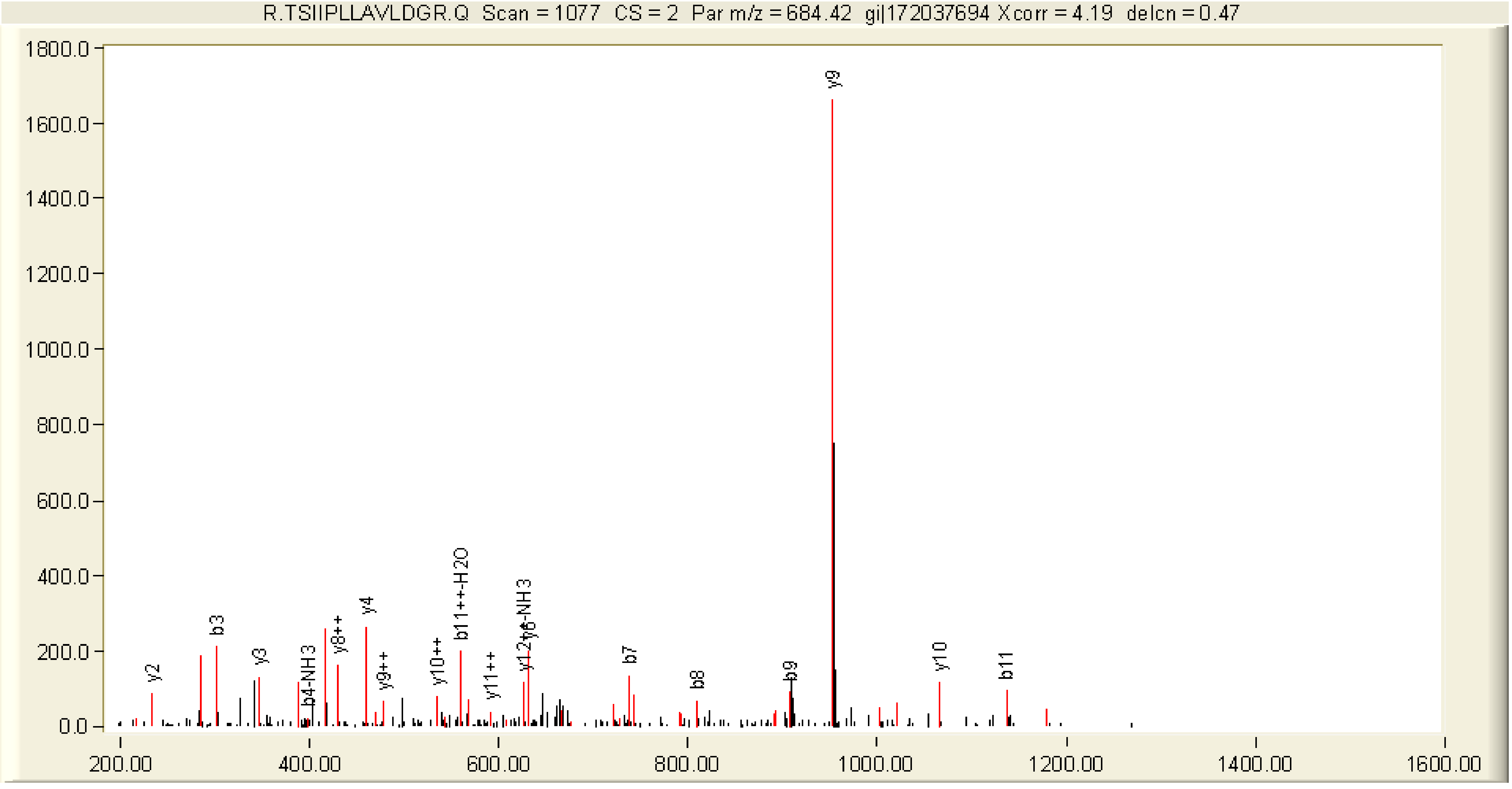

R.TSIIPLLAVLDGR.Q Scan = 1077 CS = 2 Par m/z = 684.42 gi|172037694 Xcorr = 4.19 delcn = 0.47
y9
y2
b3
y3
b4-NH3
y8++
y4
y9++
y10++
b11++-H2O
y11++
y12++
y6-NH3
b7
b8
b9
y10
b11
1800.0
1600.0
1400.0
1200.0
1000.0
800.0
600.0
400.0
200.0
0.0
200.00
400.00
600.00
800.00
1000.00
1200.00
1400.00
1600.00

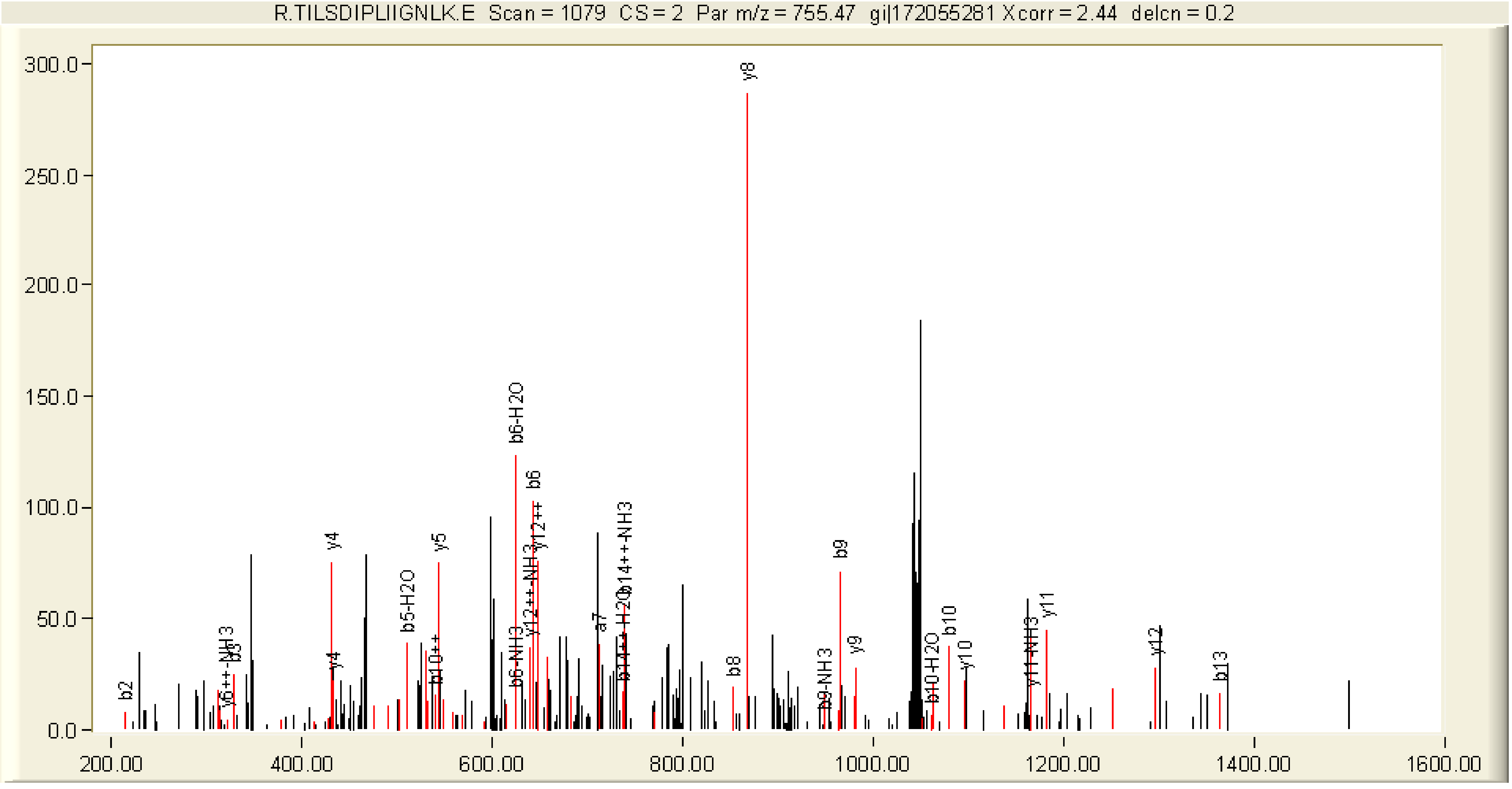
R.TILSDIPLIIGNLK.E Scan = 1079 CS = 2 Par m/z = 755.47 gi|172055281 Xcorr = 2.44 delcn = 0.2
300.0
250.0
200.0
150.0
100.0
50.0
0.0
200.00
400.00
600.00
800.00
1000.00
1200.00
1400.00
1600.00
y8
b6-H2O
b6
y12++
y4
y5
b9
b14++-NH3
b5-H2O
y12++-NH3
a7
y11
b10
y9
y12
b13
b8
y10
y11-NH3
b10-H2O
b9-NH3
b6-NH3
b10++
y6++-NH3
b3
b2

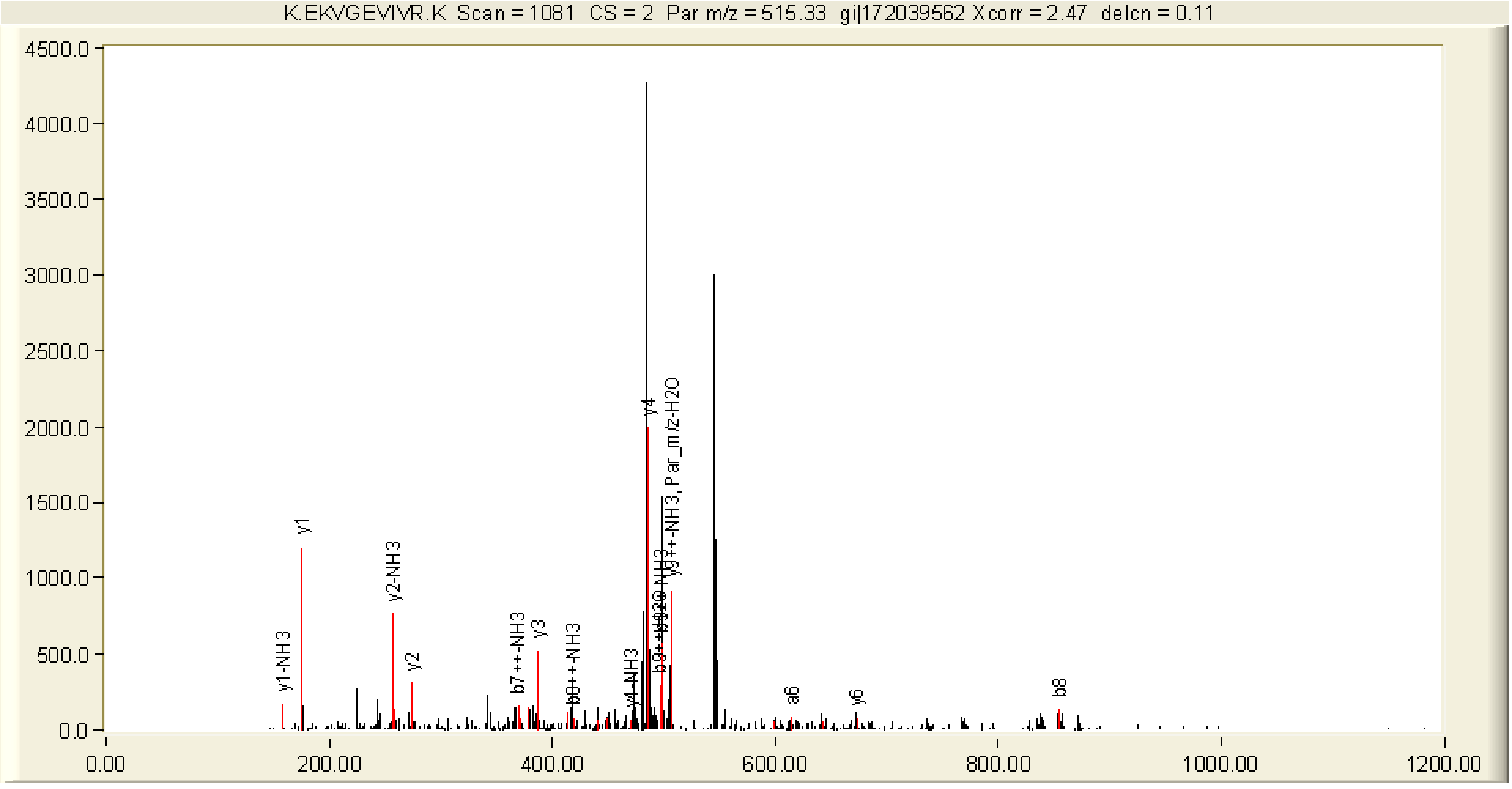
K.EKVGEVIVR.K Scan = 1081 CS = 2 Par m/z = 515.33 gi|172039562 Xcorr = 2.47 delcn = 0.11
4500.0
4000.0
3500.0
3000.0
2500.0
2000.0
1500.0
1000.0
500.0
0.0
0.00
200.00
400.00
600.00
800.00
1000.00
1200.00
y1-NH3
y1
y2-NH3
y2
b7++-NH3
y3
y4
y9++-NH3, Par_m/z-H2O
a6
y6
b8

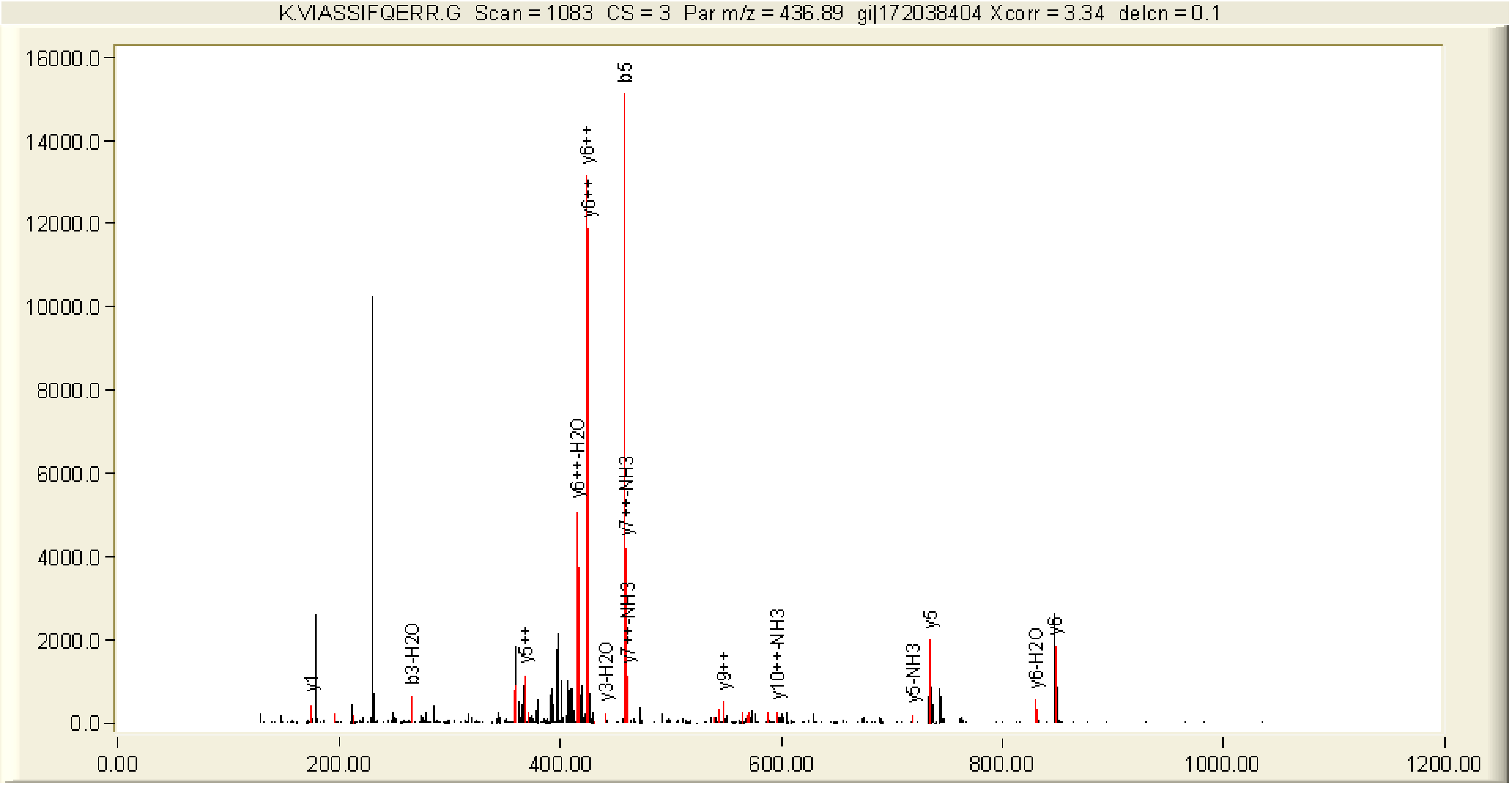
K.VIASSIFQERR.G Scan = 1083 CS = 3 Par m/z = 436.89 gi|172038404 Xcorr = 3.34 delcn = 0.1
16000.0
14000.0
12000.0
10000.0
8000.0
6000.0
4000.0
2000.0
0.0
0.00
200.00
400.00
600.00
800.00
1000.00
1200.00
b5
y6++
y6++
y6++-H2O
y7++-NH3
y7++-NH3
y1
b3-H2O
y5++
y3-H2O
y9++
y10++-NH3
y5-NH3
y5
y6-H2O
y6

K.ILLQAYHQPEAVTEELIELLLKPAQDK.G Scan = 1085 CS = 3 Par m/z = 1035.58 gi|172038441 Xcorr = 5.32 delcn = 0.3

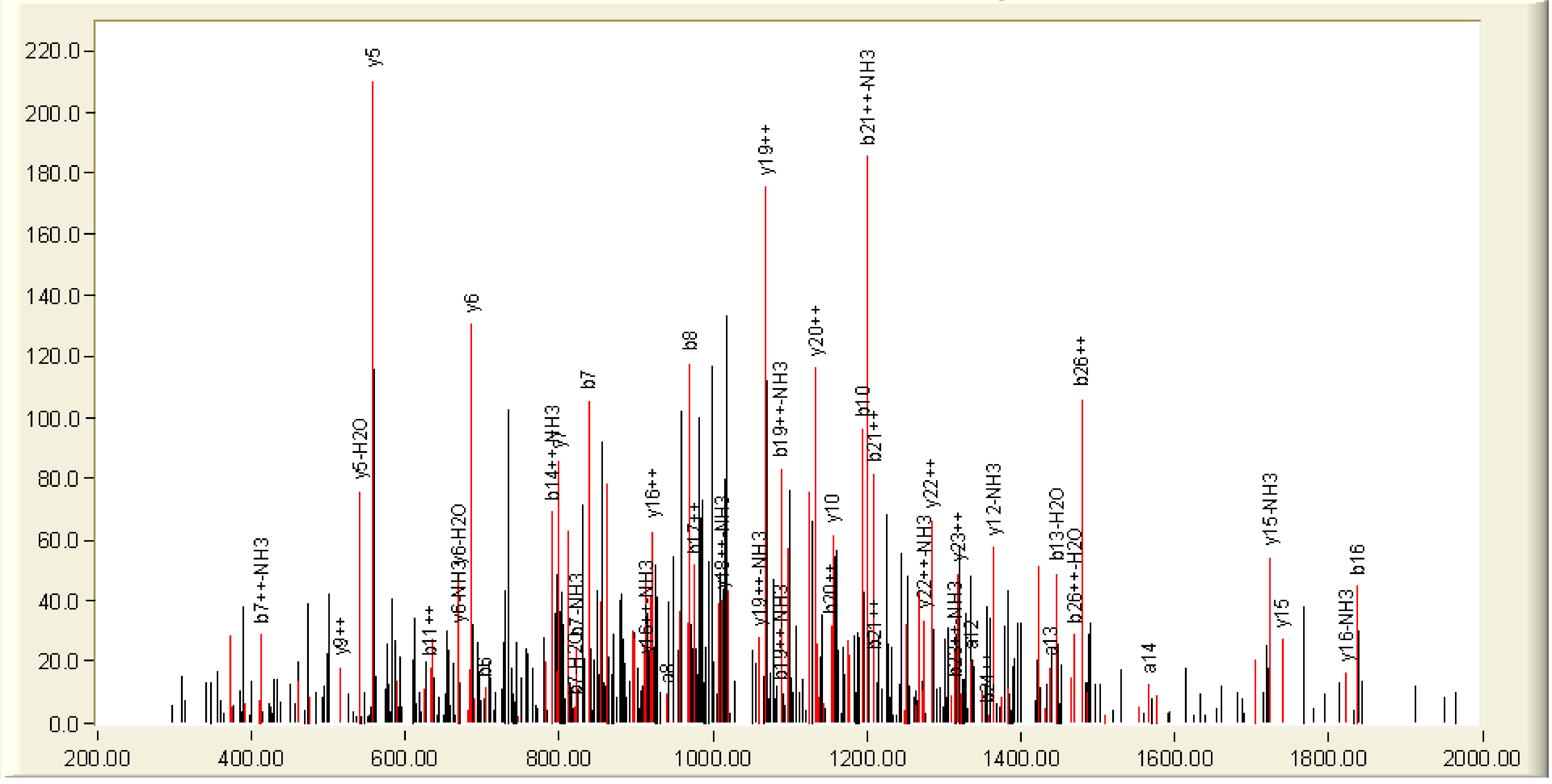

K.AGSGVGLVSPAGATFTTEDINIVQDAVK.A Scan = 1087 CS = 3 Par m/z = 906.47 gi|172038515 Xcorr = 3.93 delcn = 0.36

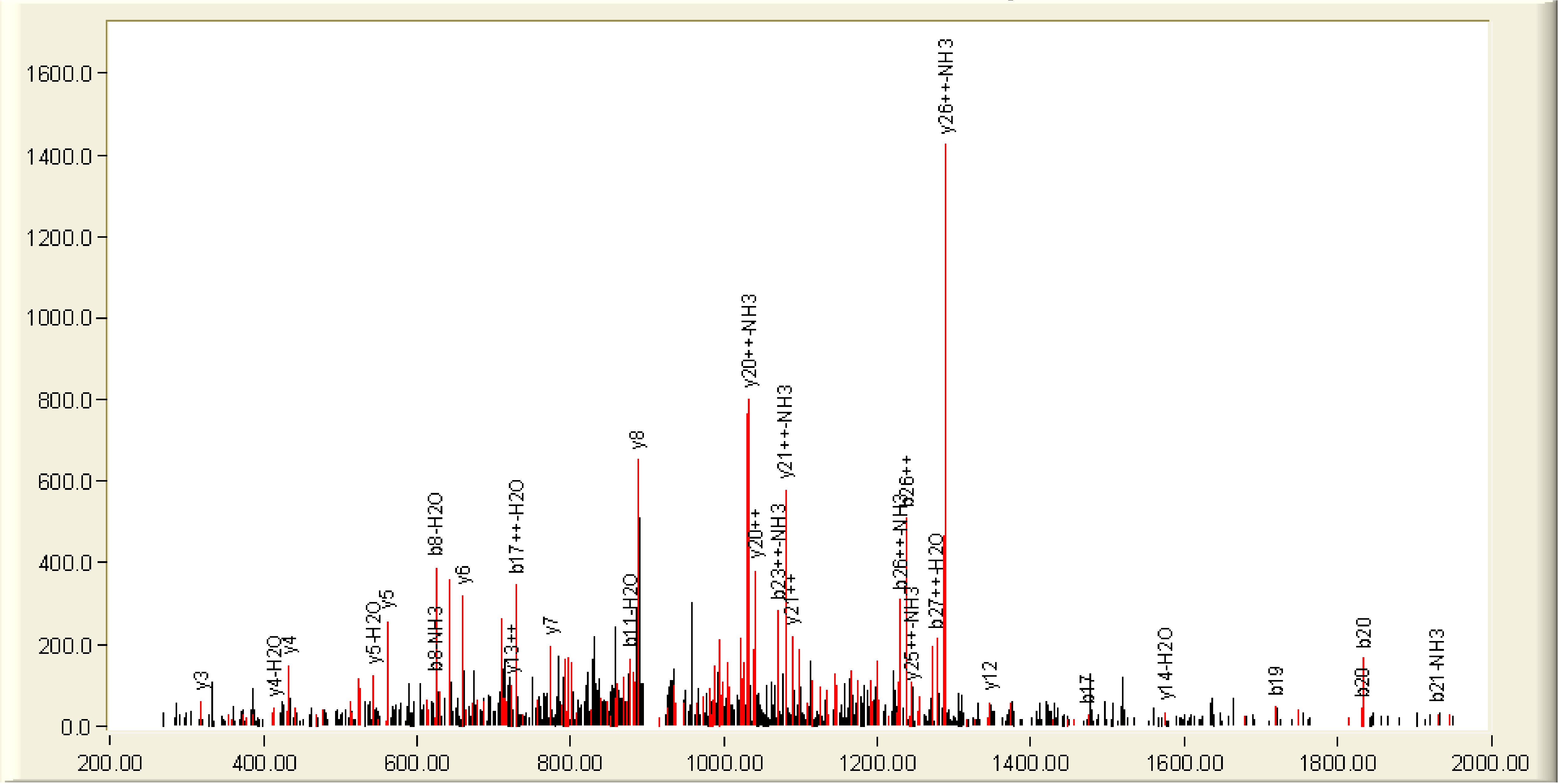

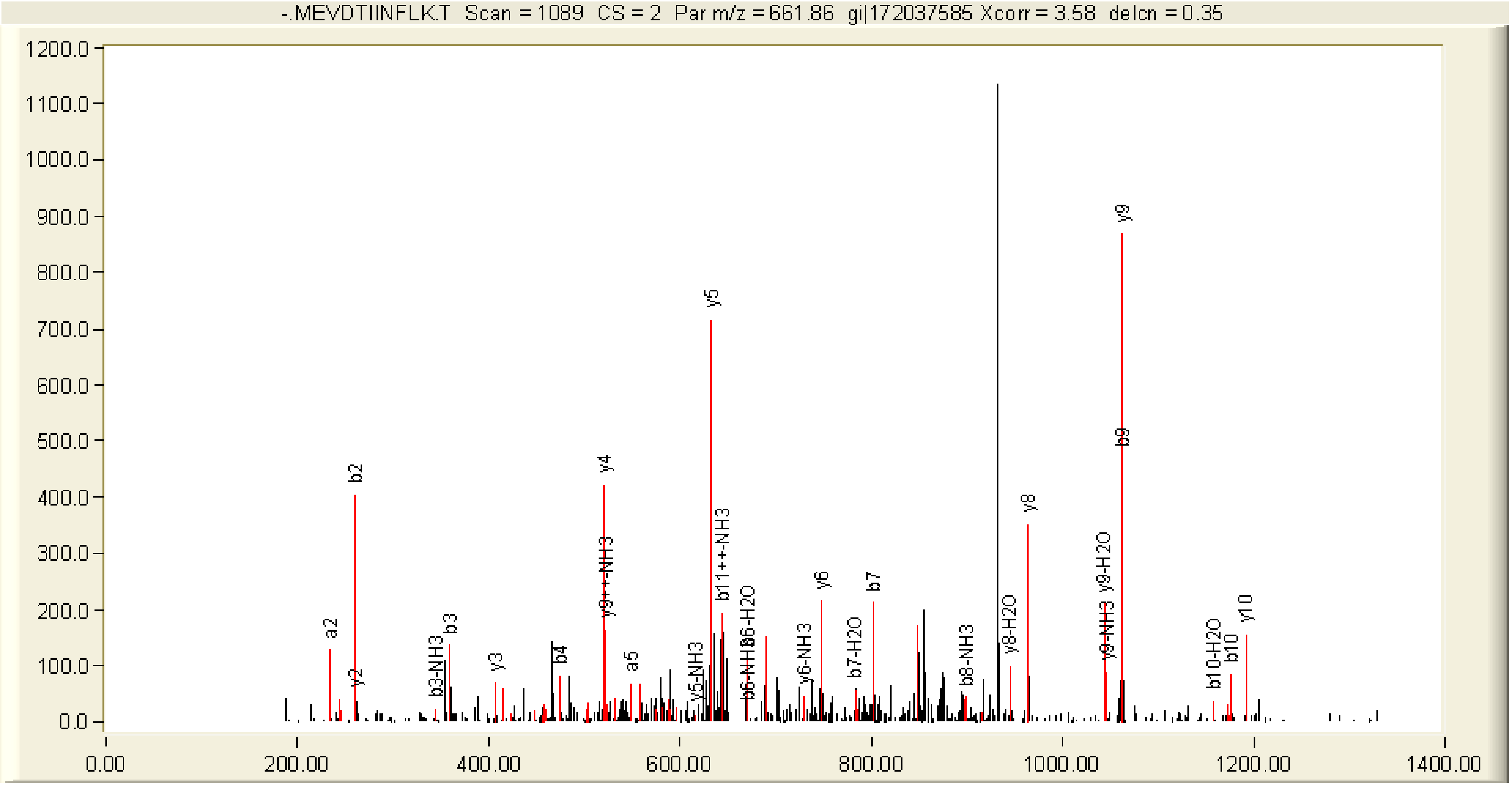
-.MEVDTIINFLK.T Scan = 1089 CS = 2 Par m/z = 661.86 gi|172037585 Xcorr = 3.58 delcn = 0.35
1200.0
1100.0
1000.0
900.0
800.0
700.0
600.0
500.0
400.0
300.0
200.0
100.0
0.0
0.00
200.00
400.00
600.00
800.00
1000.00
1200.00
1400.00
a2
b2
y2
b3-NH3
b3
y3
b4
y4
y9++-NH3
a5
y5-NH3
y5
b11++-NH3
b6-NH3
b6-H2O
y6-NH3
y6
b7-H2O
b7
b8-NH3
y8-H2O
y8
y9-NH3
y9-H2O
b9
y9
b10-H2O
b10
y10

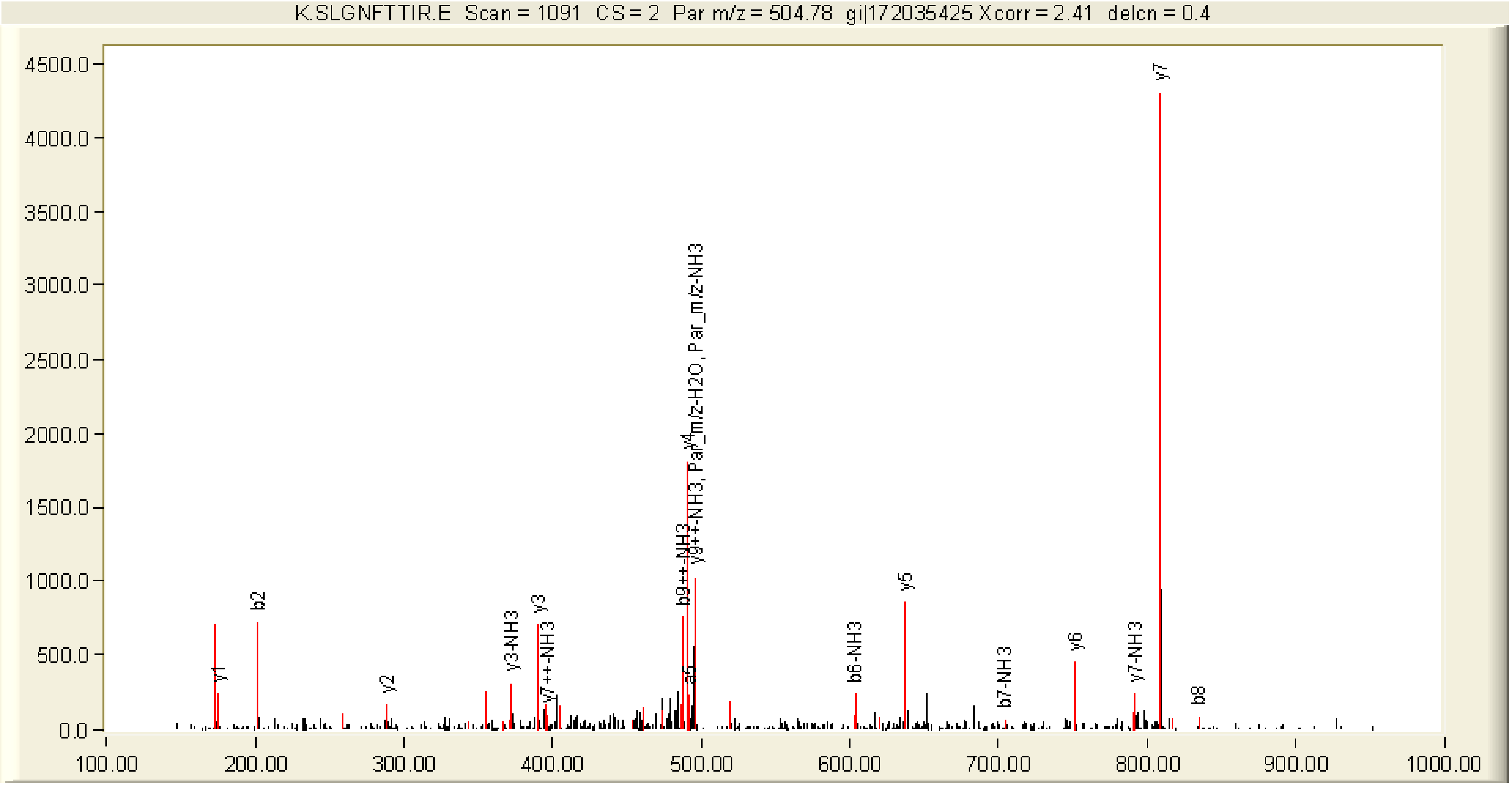

K.SLGNFTTIR.E Scan = 1091 CS = 2 Par m/z = 504.78 gi|172035425 Xcorr = 2.41 delcn = 0.4
y7
y4
b9++-NH3, y9++-NH3, Par_m/z-H2O, Par_m/z-NH3
y5
b2
y3
y3-NH3
y7++-NH3
b6-NH3
y6
y7-NH3
b7-NH3
b8
y1
y2
a5
4500.0
4000.0
3500.0
3000.0
2500.0
2000.0
1500.0
1000.0
500.0
0.0
100.00
200.00
300.00
400.00
500.00
600.00
700.00
800.00
900.00
1000.00

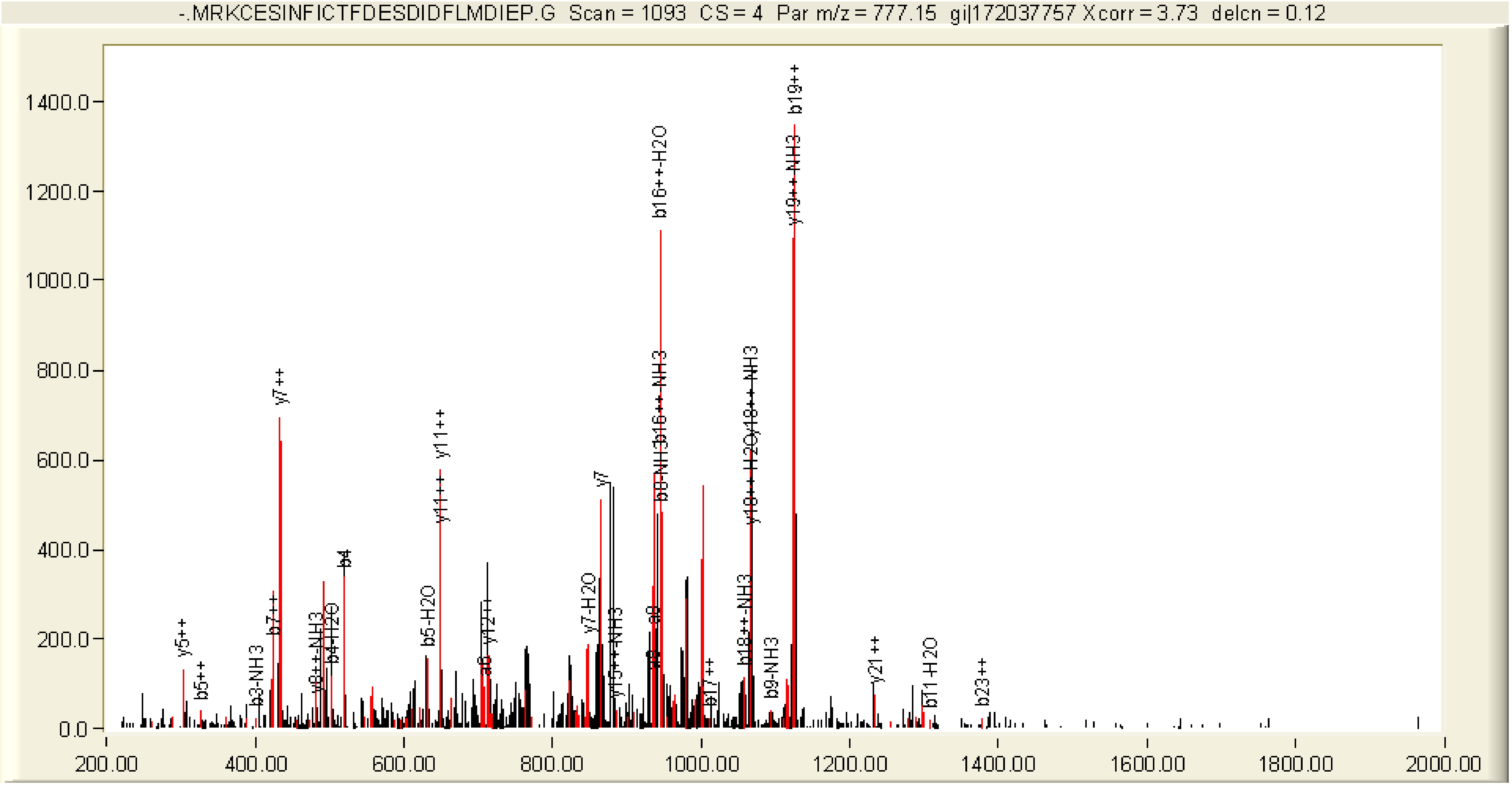
-.MRKCESINFICTFDESDIDFLMDIEP.G Scan = 1093 CS = 4 Par m/z = 777.15 gi|172037757 Xcorr = 3.73 delcn = 0.12
1400.0
1200.0
1000.0
800.0
600.0
400.0
200.0
0.0
200.00
400.00
600.00
800.00
1000.00
1200.00
1400.00
1600.00
1800.00
2000.00
y5++
b5++
b3-NH3
b7++
y7++
y8++-NH3
b4-H2O
b4
b5-H2O
y11++
y11++
a6
y12++
y7-H2O
y7
y15++-NH3
a8
a8
b8-NH3
b16++-NH3
b16++-H2O
b17++
b18++-NH3
y19++-H2O
y18++-NH3
b9-NH3
y19++-NH3
b19++
y21++
b11-H2O
b23++

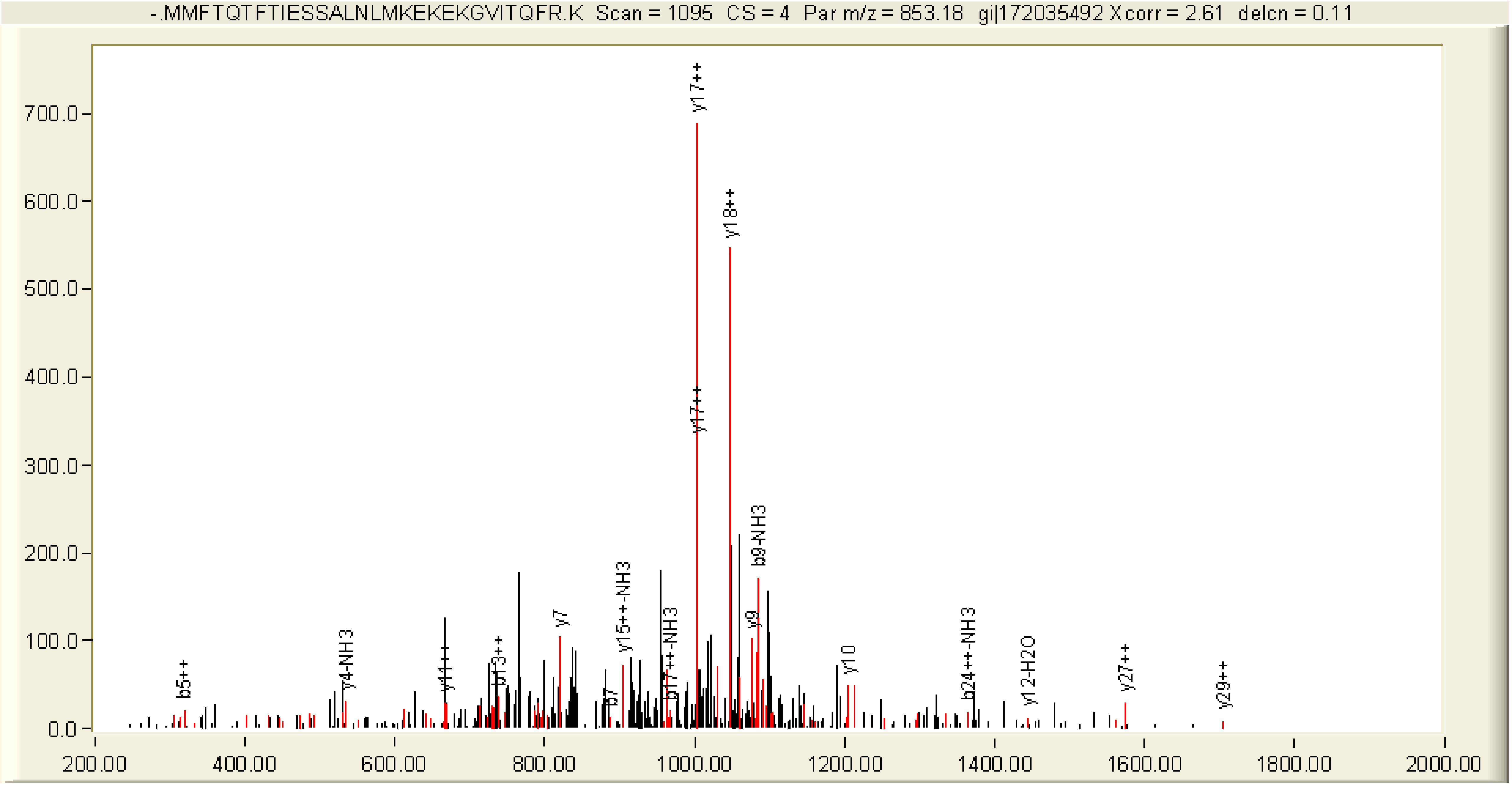
-.MMFTQTFTIESSALNLMKEKEKGVITQFR.K Scan = 1095 CS = 4 Par m/z = 853.18 gi|172035492 Xcorr = 2.61 delcn = 0.11
0.0
100.0
200.0
300.0
400.0
500.0
600.0
700.0
200.00
400.00
600.00
800.00
1000.00
1200.00
1400.00
1600.00
1800.00
2000.00
y17++
y18++
y17++
b9-NH3
y9
y7
y15++-NH3
b17++-NH3
b7
b13++
y11++
y4-NH3
b5++
y10
b24++-NH3
y12-H2O
y27++
y29++

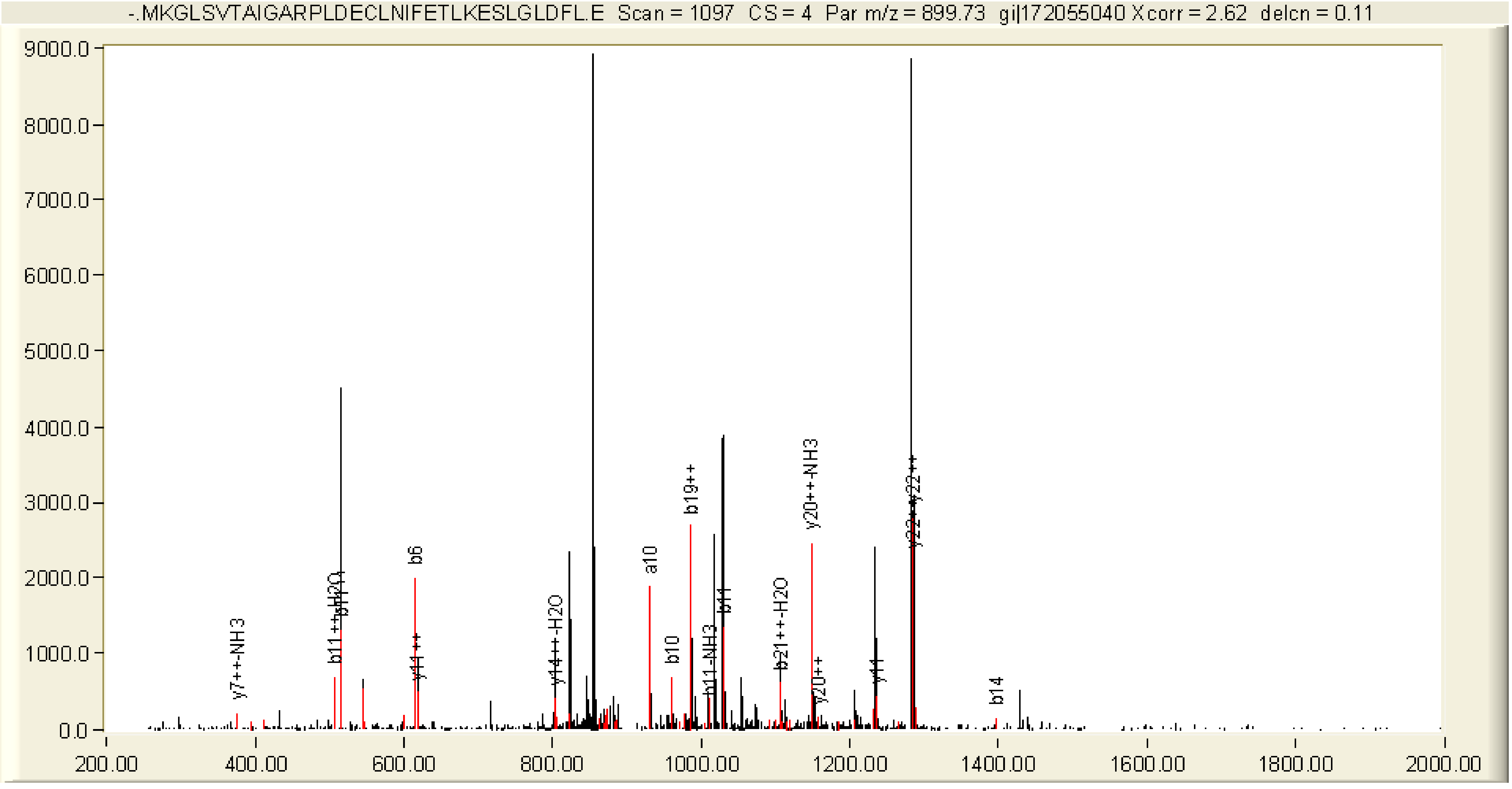
-.MKGLSVTAIGARPLDECLNIFETLKESLGLDFL.E Scan = 1097 CS = 4 Par m/z = 899.73 gi|172055040 Xcorr = 2.62 delcn = 0.11
9000.0
8000.0
7000.0
6000.0
5000.0
4000.0
3000.0
2000.0
1000.0
0.0
200.00
400.00
600.00
800.00
1000.00
1200.00
1400.00
1600.00
1800.00
2000.00
y7++-NH3
b11++-H2O
b6
y14++
y14++-H2O
a10
b10
b19++
b11-NH3
b11
b21++-H2O
y20++-NH3
y20++
y11
y22++
b14

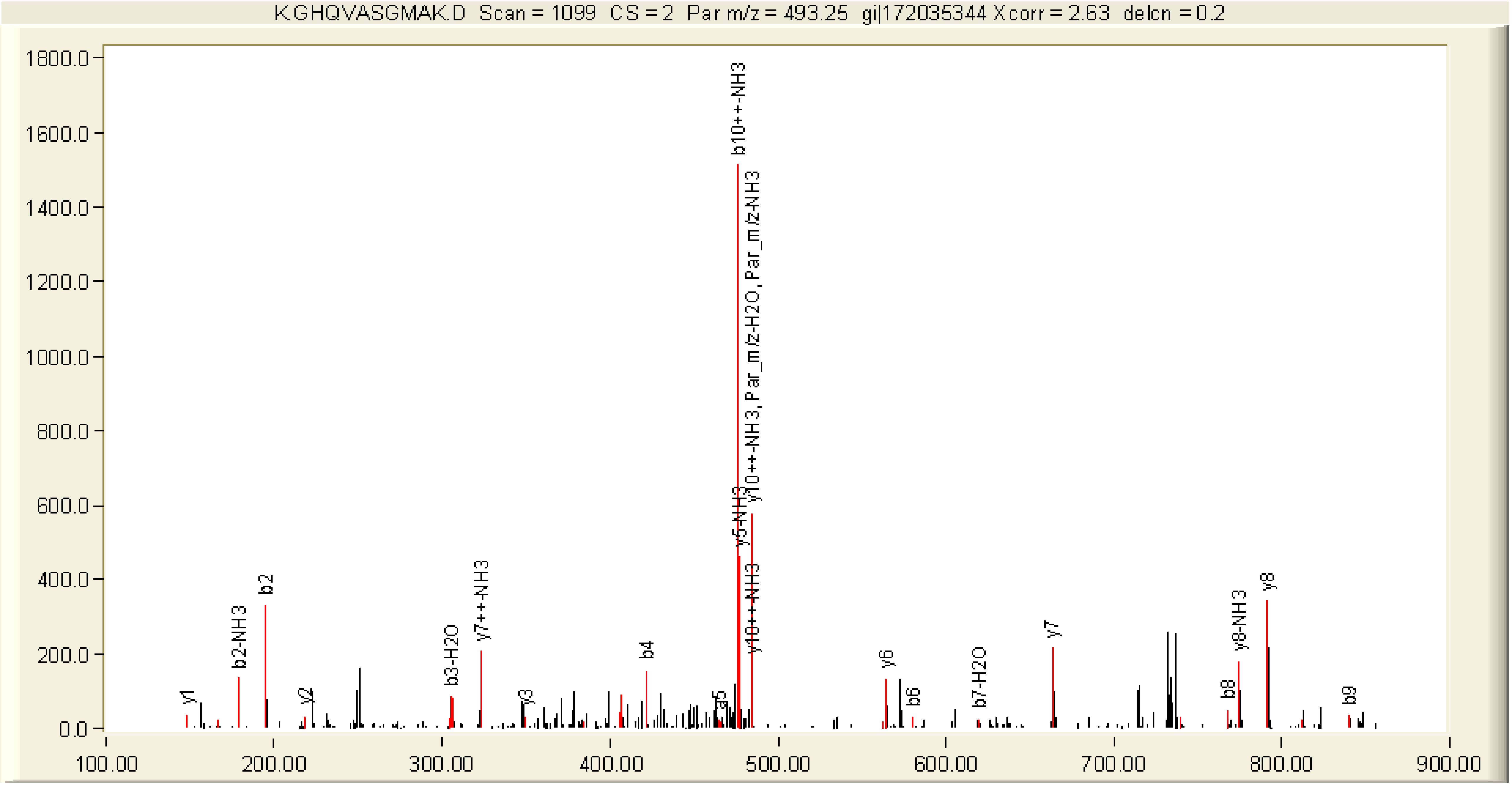

K.GHQVASGMAK.D Scan = 1099 CS = 2 Par m/z = 493.25 gi|172035344 Xcorr = 2.63 delcn = 0.2
1800.0
1600.0
1400.0
1200.0
1000.0
800.0
600.0
400.0
200.0
0.0
100.00
200.00
300.00
400.00
500.00
600.00
700.00
800.00
900.00
y1
b2-NH3
b2
y2
b3-H2O
y7++-NH3
y3
b4
a5
b10++-NH3
y5-NH3
y10++-NH3, Par_m/z-H2O, Par_m/z-NH3
y10++-NH3
y6
b6
b7-H2O
y7
b8
y8-NH3
y8
b9

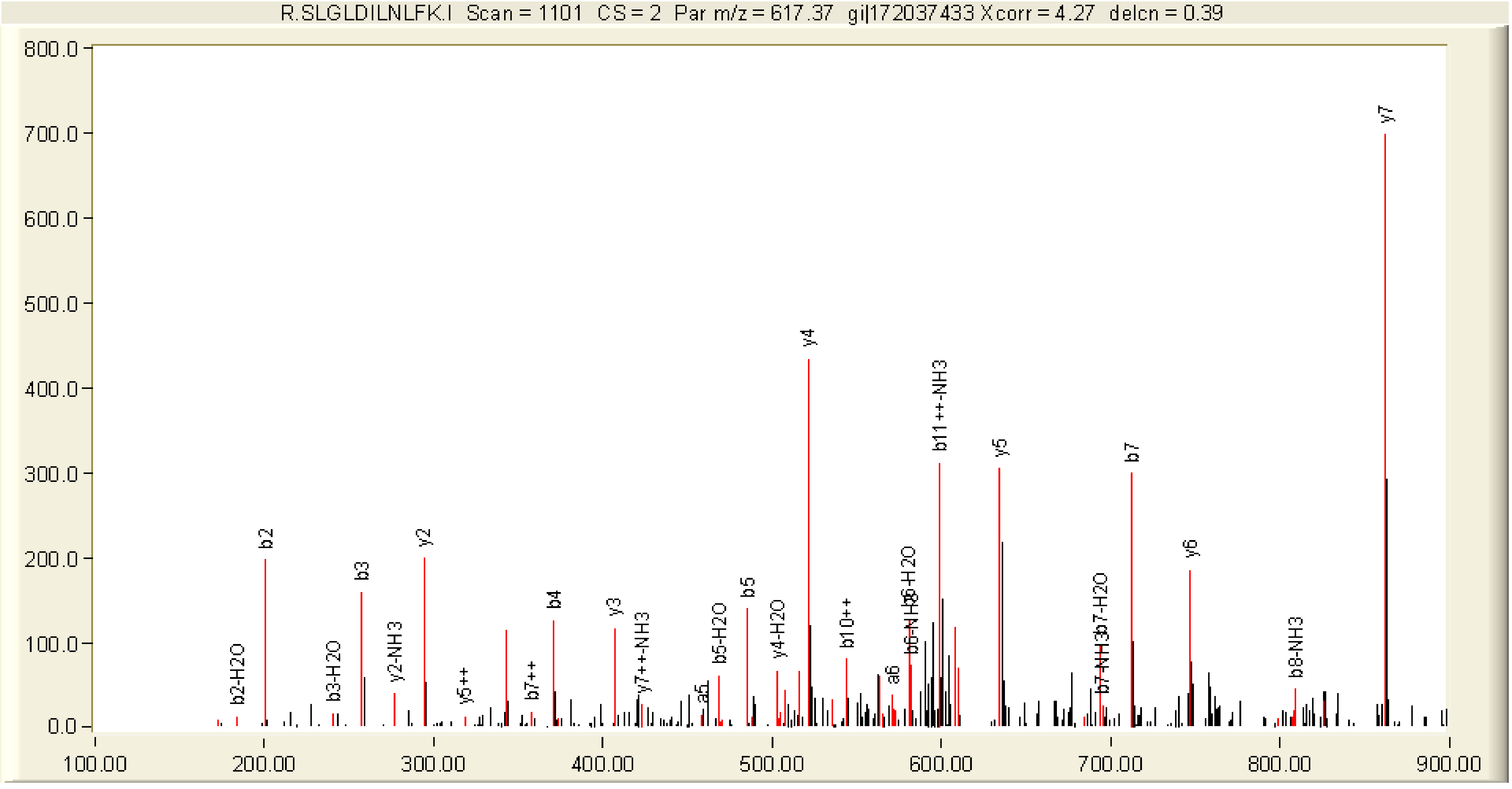
R.SLGLDILNLFK.I Scan = 1101 CS = 2 Par m/z = 617.37 gi|172037433 Xcorr = 4.27 delcn = 0.39
800.0
700.0
600.0
500.0
400.0
300.0
200.0
100.0
0.0
100.00
200.00
300.00
400.00
500.00
600.00
700.00
800.00
900.00
b2-H2O
b2
b3-H2O
b3
y2-NH3
y2
y5++
b7++
b4
y3
y7++-NH3
a5
b5-H2O
b5
y4-H2O
y4
b10++
a6
b11++-NH3
y5
b7-H2O
b7
y6
b8-NH3
y7

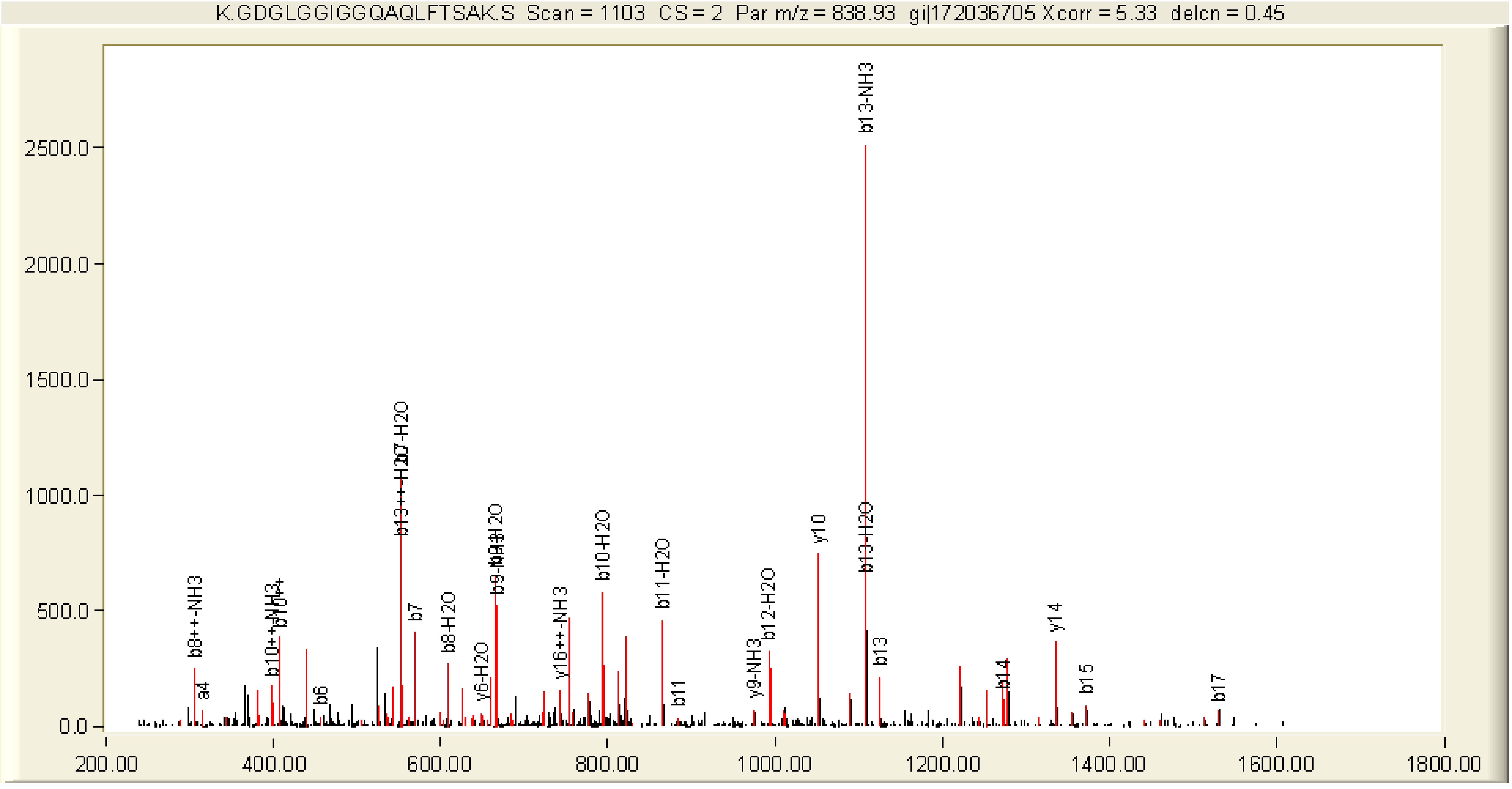
K.GDGLGGIGGQAQLFTSAK.S Scan = 1103 CS = 2 Par m/z = 838.93 gi|172036705 Xcorr = 5.33 delcn = 0.45
b13-NH3
b13-H2O
y10
b11-H2O
b10-H2O
b12-H2O
y9-NH3
y14
b13
b14
b15
b17
b11
b8++-NH3
a4
b6
b7
b8-H2O
y6-H2O
y16++-NH3
0.0
500.0
1000.0
1500.0
2000.0
2500.0
200.00
400.00
600.00
800.00
1000.00
1200.00
1400.00
1600.00
1800.00

K.QKGIITQK.L Scan = 1105 CS = 1 Par m/z = 915.51 gi|172036540 Xcorr = 1.88 delcn = 0.13

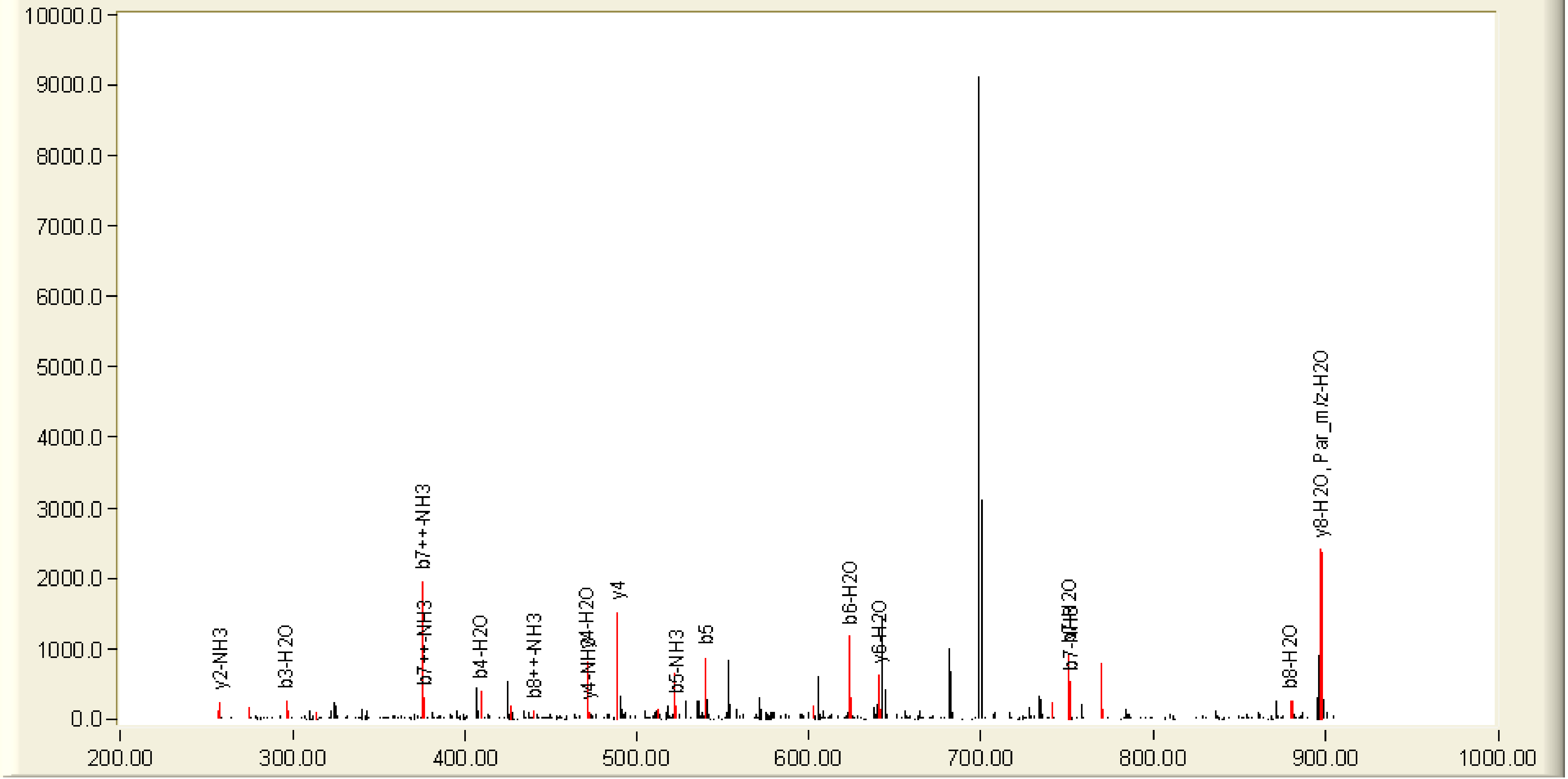

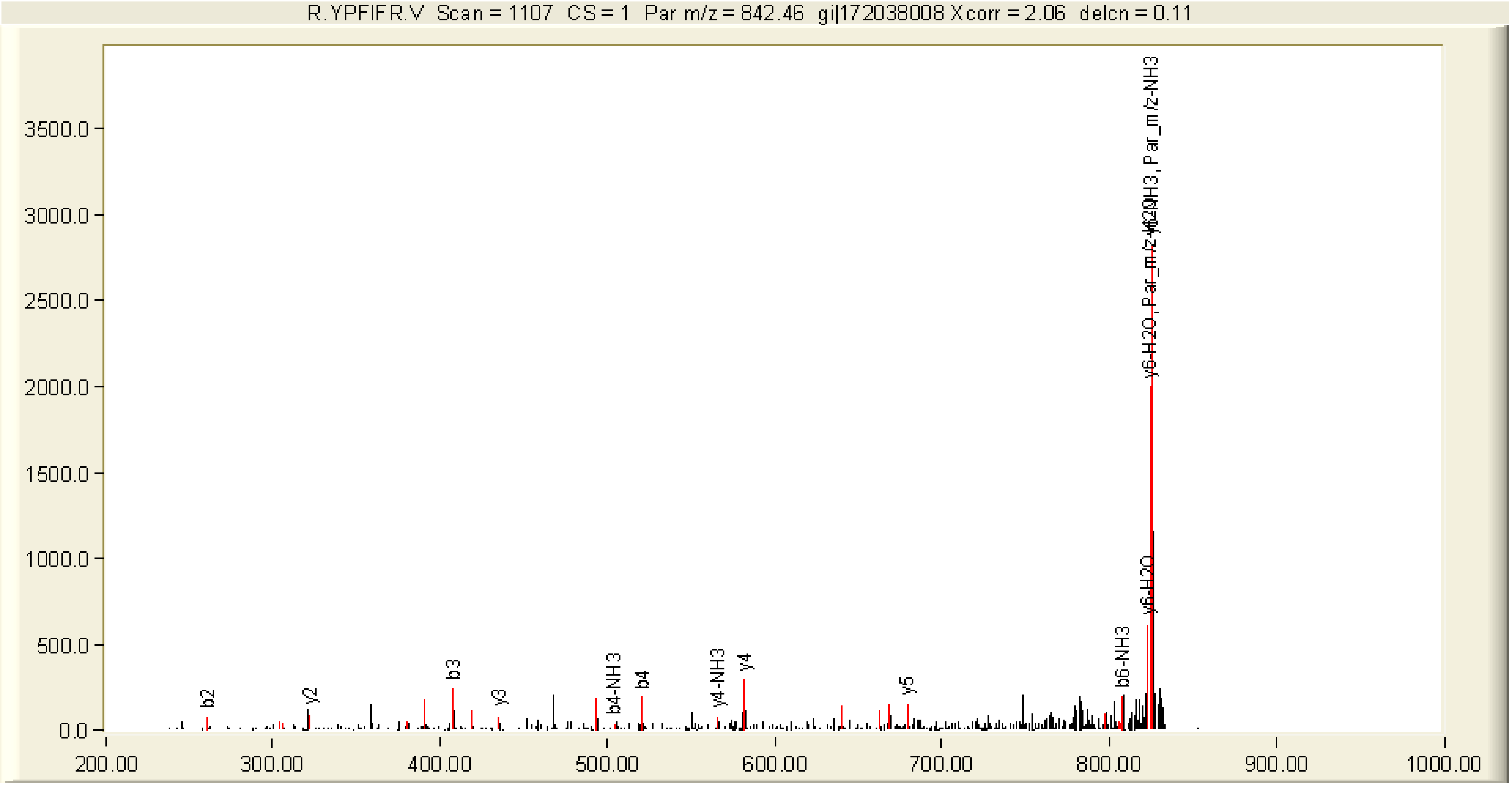

R.YPFIFR.V Scan = 1107 CS = 1 Par m/z = 842.46 gi|172038008 Xcorr = 2.06 delcn = 0.11
3500.0
3000.0
2500.0
2000.0
1500.0
1000.0
500.0
0.0
200.00
300.00
400.00
500.00
600.00
700.00
800.00
900.00
1000.00
b2
y2
b3
y3
b4-NH3
b4
y4-NH3
y4
y5
b6-NH3
y6-H2O
y6-H2O , Par_m/z-H2O
y6-NH3, Par_m/z-NH3

K.TISDTLSKPLK.I Scan = 1109 CS = 2 Par m/z = 601.85 gi|172039047 Xcorr = 2.99 delcn = 0.29

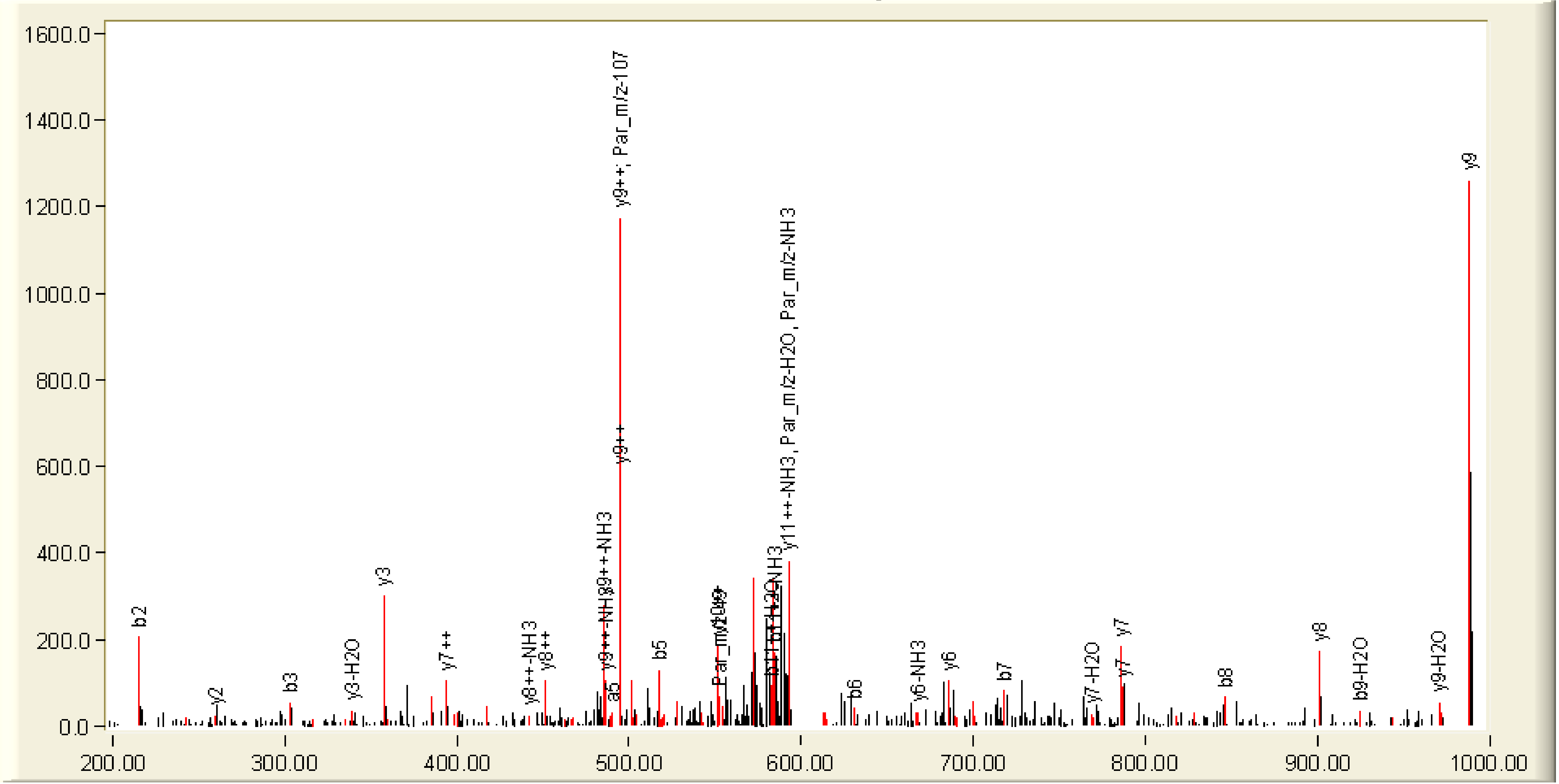

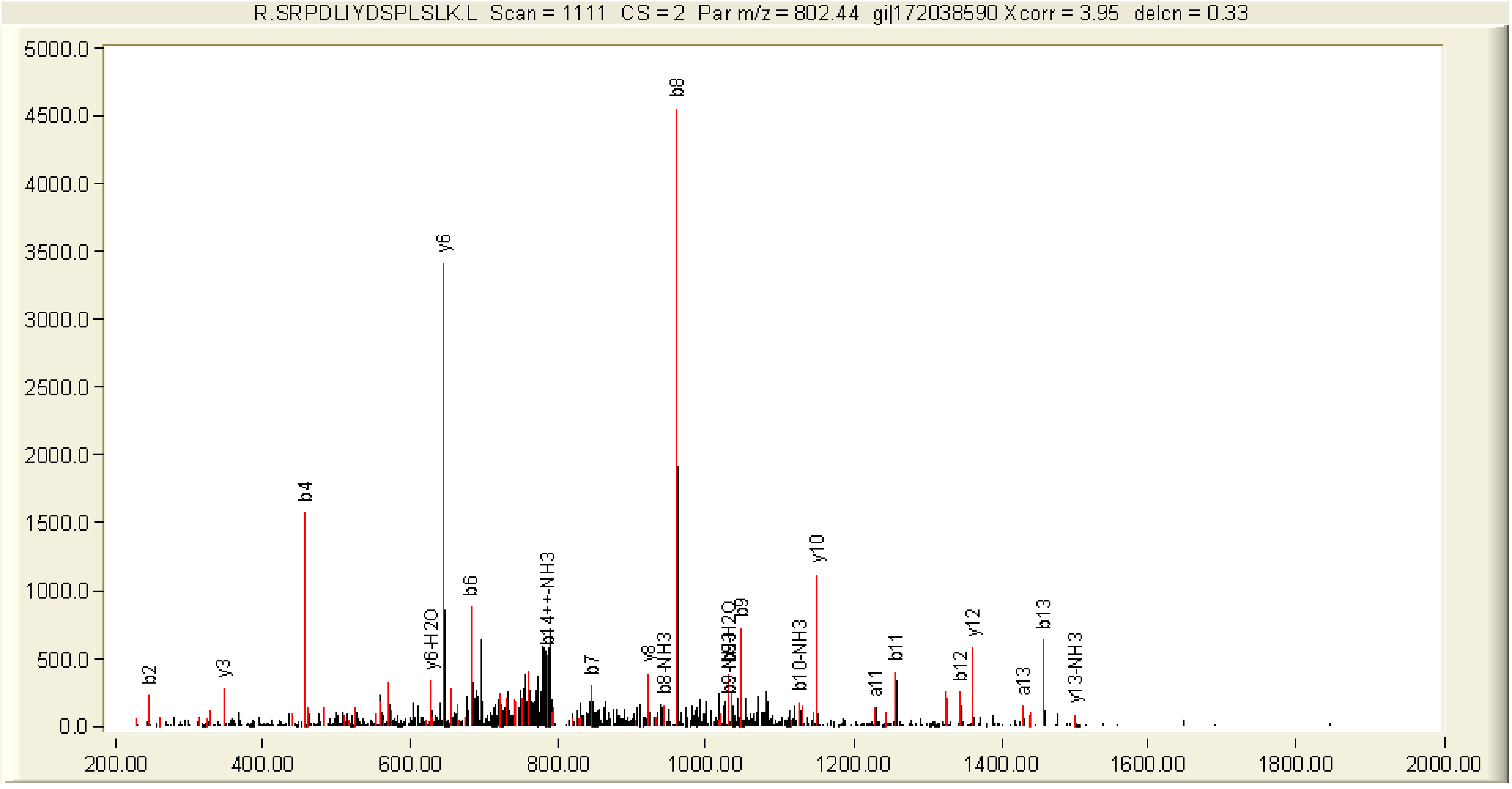

R.SRPDLIYDSPLSLK.L Scan = 1111 CS = 2 Par m/z = 802.44 gi|172038590 Xcorr = 3.95 delcn = 0.33
b8
y6
b4
b6
b14++-NH3
y10
b9
y12
b13
b11
y6-H2O
b10-NH3
y13-NH3
b8-NH3
y8
b12
a13
a11
b7
y3
b2
5000.0
4500.0
4000.0
3500.0
3000.0
2500.0
2000.0
1500.0
1000.0
500.0
0.0
200.00
400.00
600.00
800.00
1000.00
1200.00
1400.00
1600.00
1800.00
2000.00

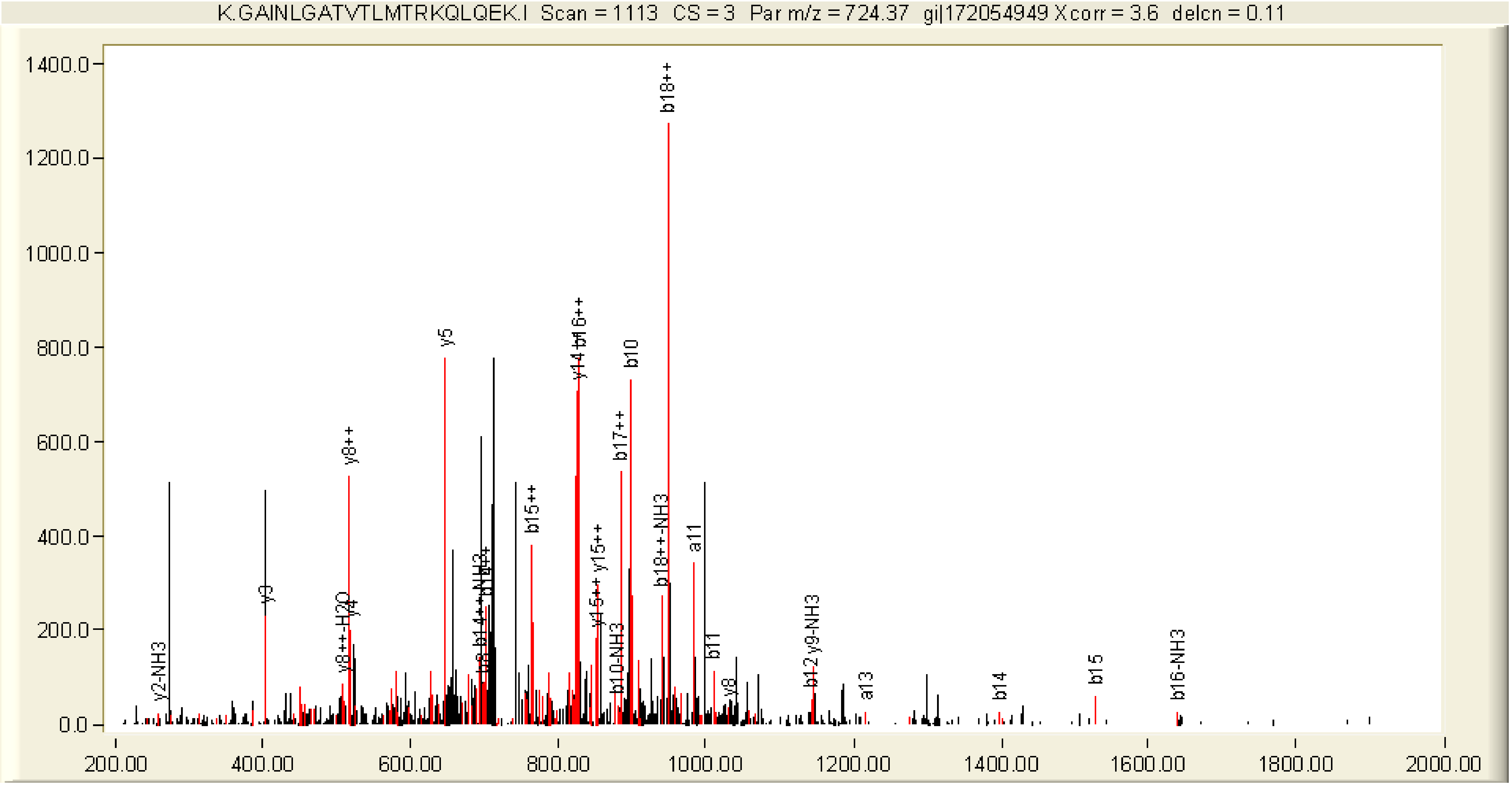

K.GAINLGATVTLMTRKQLQEK.I Scan = 1113 CS = 3 Par m/z = 724.37 gi|172054949 Xcorr = 3.6 delcn = 0.11
1400.0
1200.0
1000.0
800.0
600.0
400.0
200.0
0.0
200.00
400.00
600.00
800.00
1000.00
1200.00
1400.00
1600.00
1800.00
2000.00
y2-NH3
y3
y8++-H2O
y4
y8++
y5
b15++
y14
b16++
y15++
b17++
b10-NH3
b10
b18++-NH3
b18++
a11
b11
y8
b12
y9-NH3
a13
b14
b15
b16-NH3

K.GIVYTVYRLEDLIKLTLHHSSSPNR.S Scan = 1115 CS = 3 Par m/z = 971.15 gi|172038850 Xcorr = 3.46 delcn = 0.14

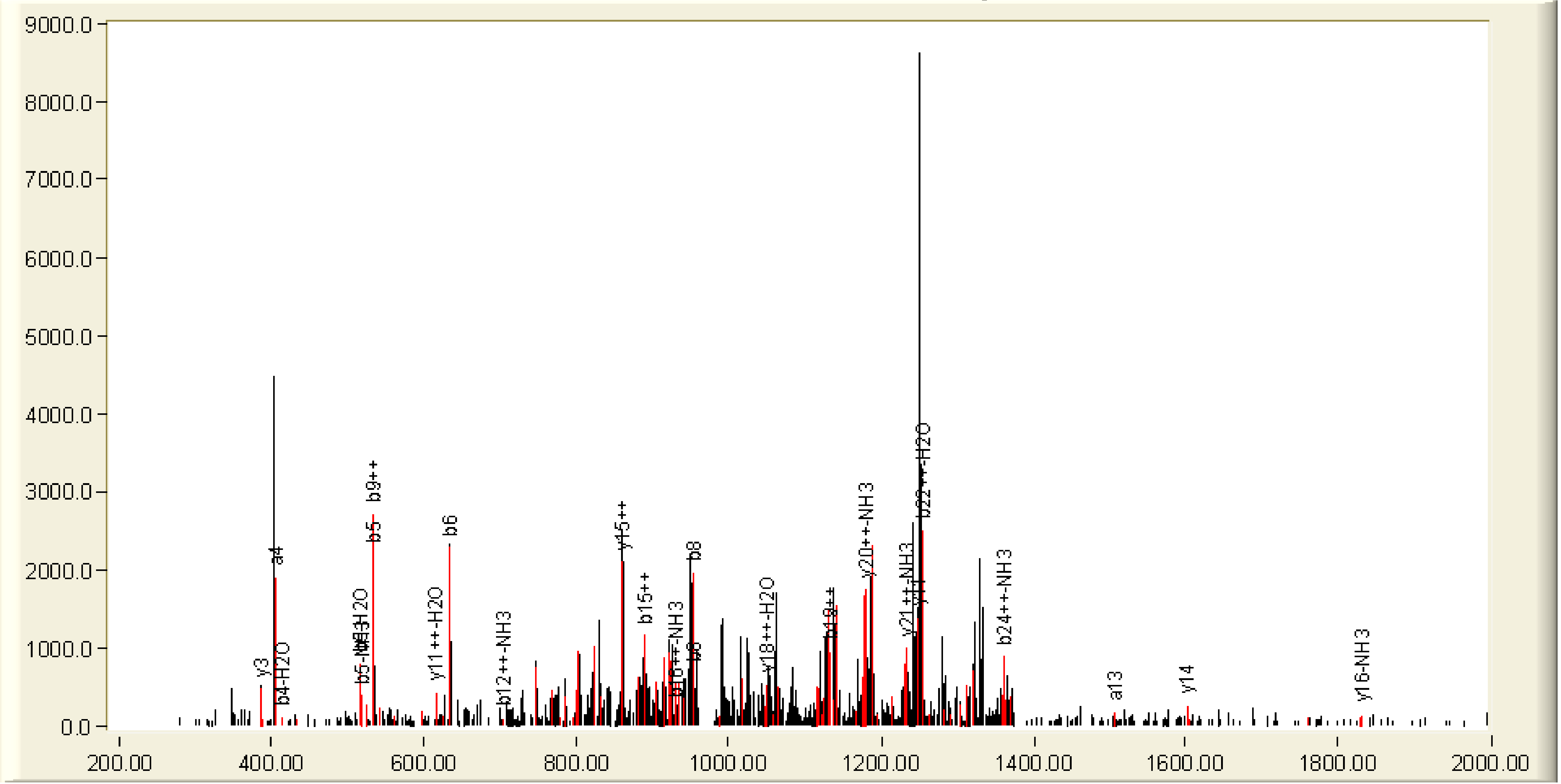

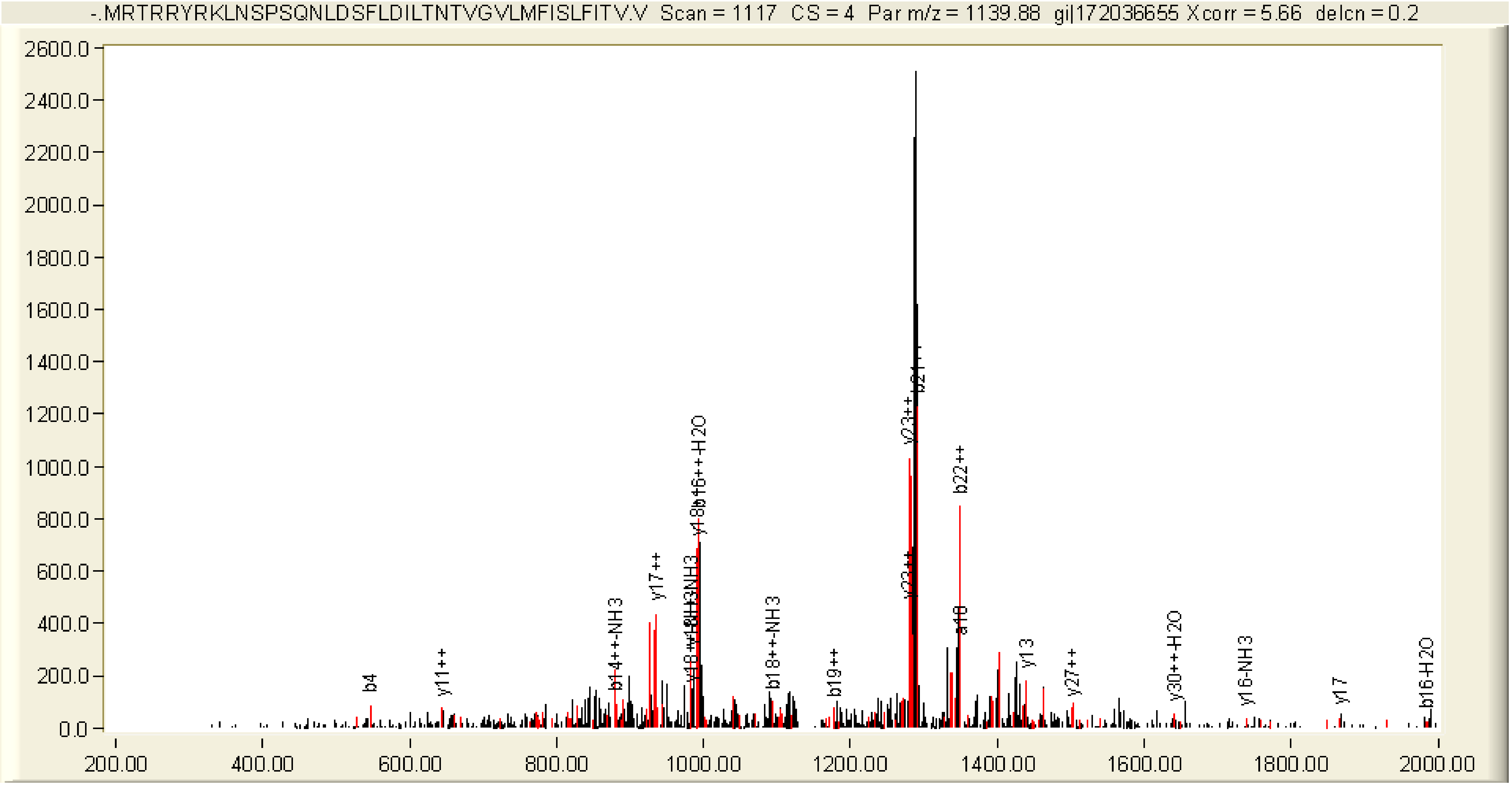

-.MRTRRYRKLNSPSQNLDSFLDILTNTVGVLMFISLFITV.V Scan = 1117 CS = 4 Par m/z = 1139.88 gi|172036655 Xcorr = 5.66 delcn = 0.2
2600.0
2400.0
2200.0
2000.0
1800.0
1600.0
1400.0
1200.0
1000.0
800.0
600.0
400.0
200.0
0.0
200.00
400.00
600.00
800.00
1000.00
1200.00
1400.00
1600.00
1800.00
2000.00
b4
y11++
b14++-NH3
y17++
b18++-NH3
b19++
y23++
b22++
y13
y27++
y30++-H2O
y16-NH3
y17
b16-H2O

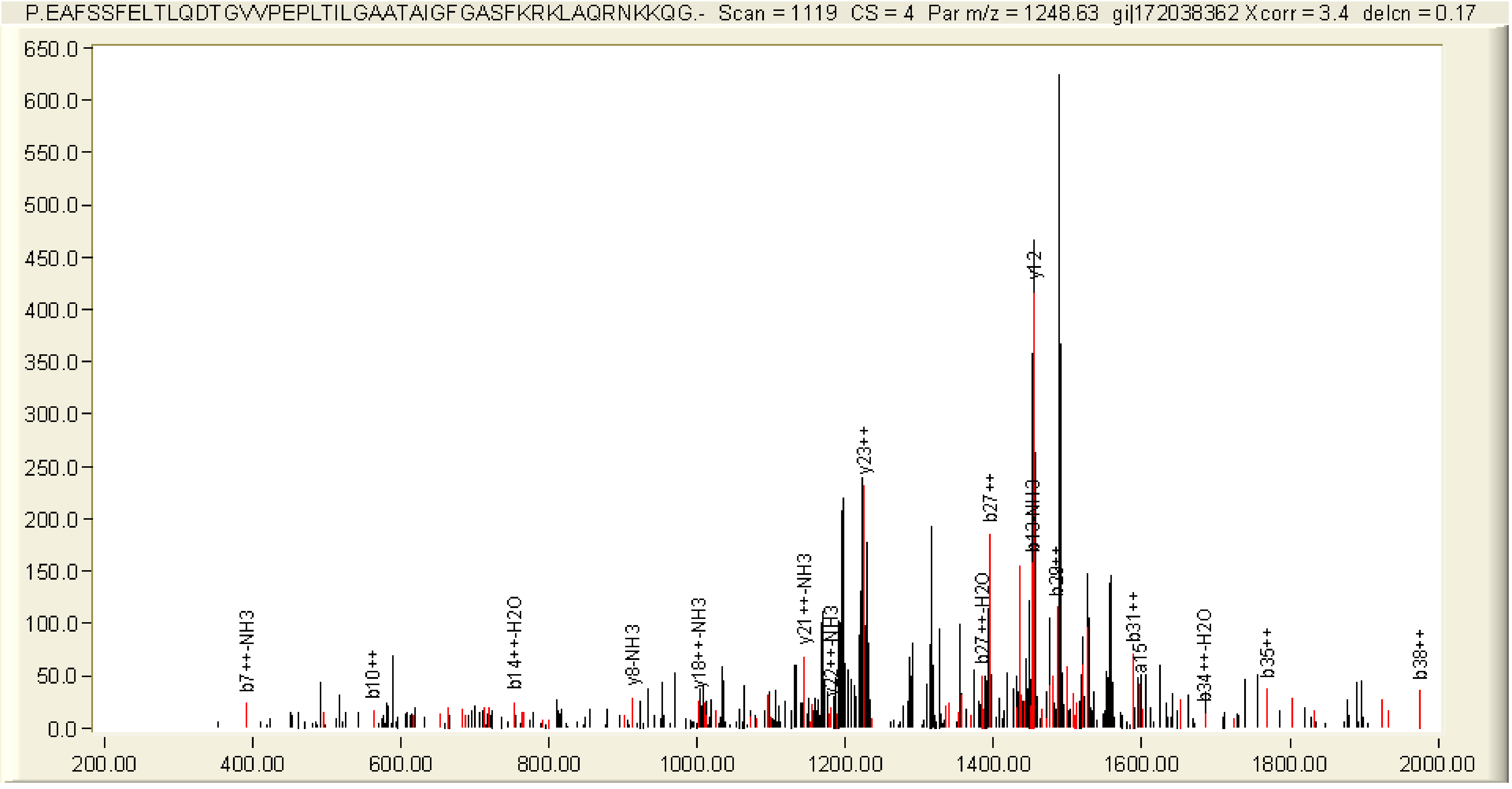
P.EAFSSFELTLQDTGVVPEPLTILGAATAIGFGASFKRKLAQRNKKQG.- Scan = 1119 CS = 4 Par m/z = 1248.63 gi|172038362 Xcorr = 3.4 delcn = 0.17
650.0
600.0
550.0
500.0
450.0
400.0
350.0
300.0
250.0
200.0
150.0
100.0
50.0
0.0
200.00
400.00
600.00
800.00
1000.00
1200.00
1400.00
1600.00
1800.00
2000.00
b7++-NH3
b10++
b14++-H2O
y8-NH3
y18++-NH3
y21++-NH3
y23++
b27++-H2O
b27++
y12
b13-NH3
a15
b31++
b34++-H2O
b35++
b38++

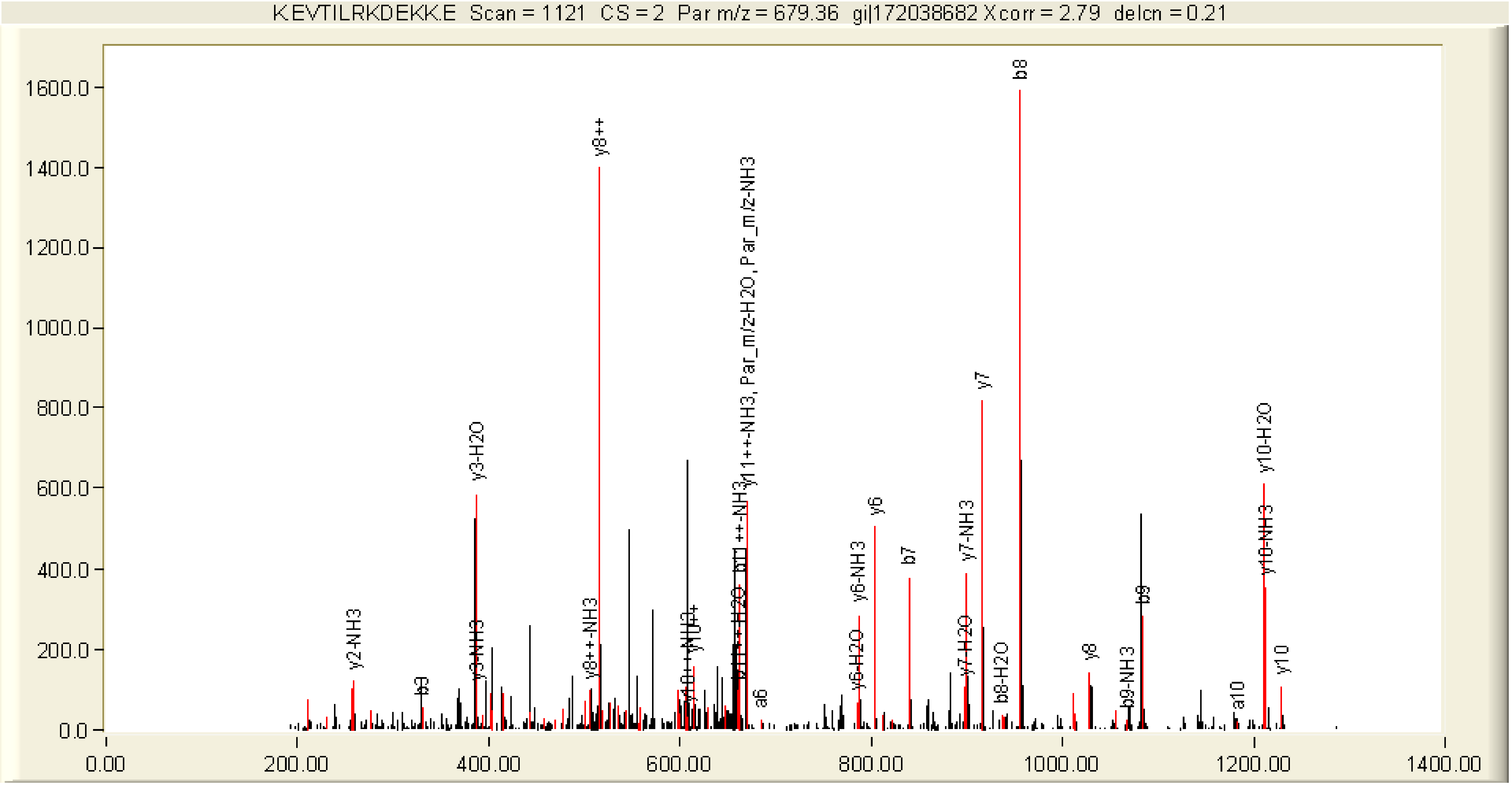
K.EVTILRKDEKK.E Scan = 1121 CS = 2 Par m/z = 679.36 gi|172038682 Xcorr = 2.79 delcn = 0.21
0.0
200.0
400.0
600.0
800.0
1000.0
1200.0
1400.0
1600.0
0.00
200.00
400.00
600.00
800.00
1000.00
1200.00
1400.00
y2-NH3
b3
y3-NH3
y3-H2O
y8++-NH3
y8++
y11++-NH3, Par_m/z-H2O, Par_m/z-NH3
a6
y6-H2O
y6-NH3
y6
b7
y7-H2O
y7-NH3
y7
b8-H2O
b8
y8
b9-NH3
b9
a10
y10-NH3
y10-H2O
y10

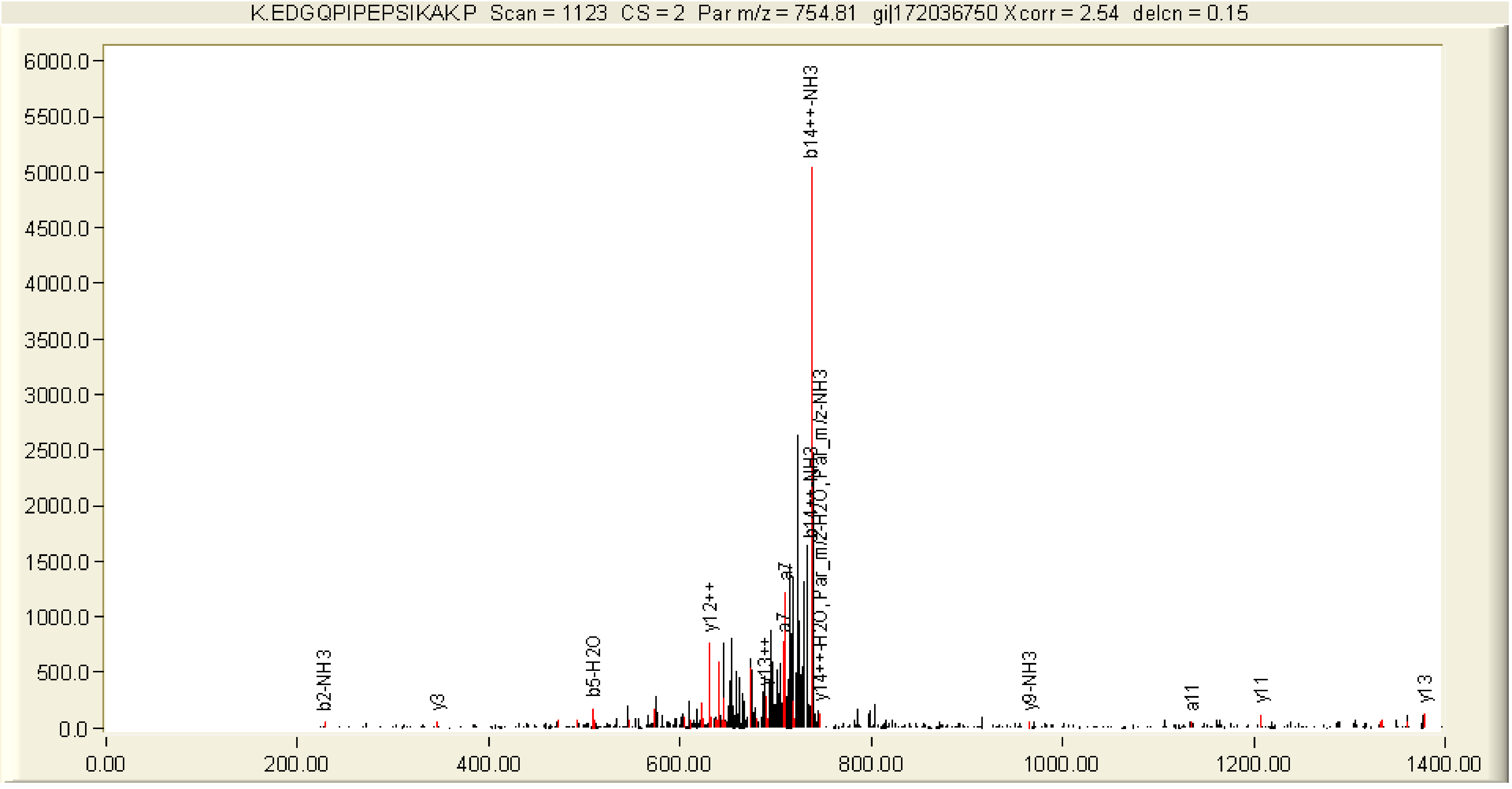
K.EDGQPIPEPSIKAKP Scan = 1123 CS = 2 Par m/z = 754.81 gi|172036750 Xcorr = 2.54 delcn = 0.15
6000.0
5500.0
5000.0
4500.0
4000.0
3500.0
3000.0
2500.0
2000.0
1500.0
1000.0
500.0
0.0
0.00
200.00
400.00
600.00
800.00
1000.00
1200.00
1400.00
b14++-NH3
y14++-H2O, Par_m/z-H2O, Par_m/z-NH3
b14++-NH3
a7
a7
y12++
y13++
b5-H2O
b2-NH3
y3
y9-NH3
a11
y11
y13

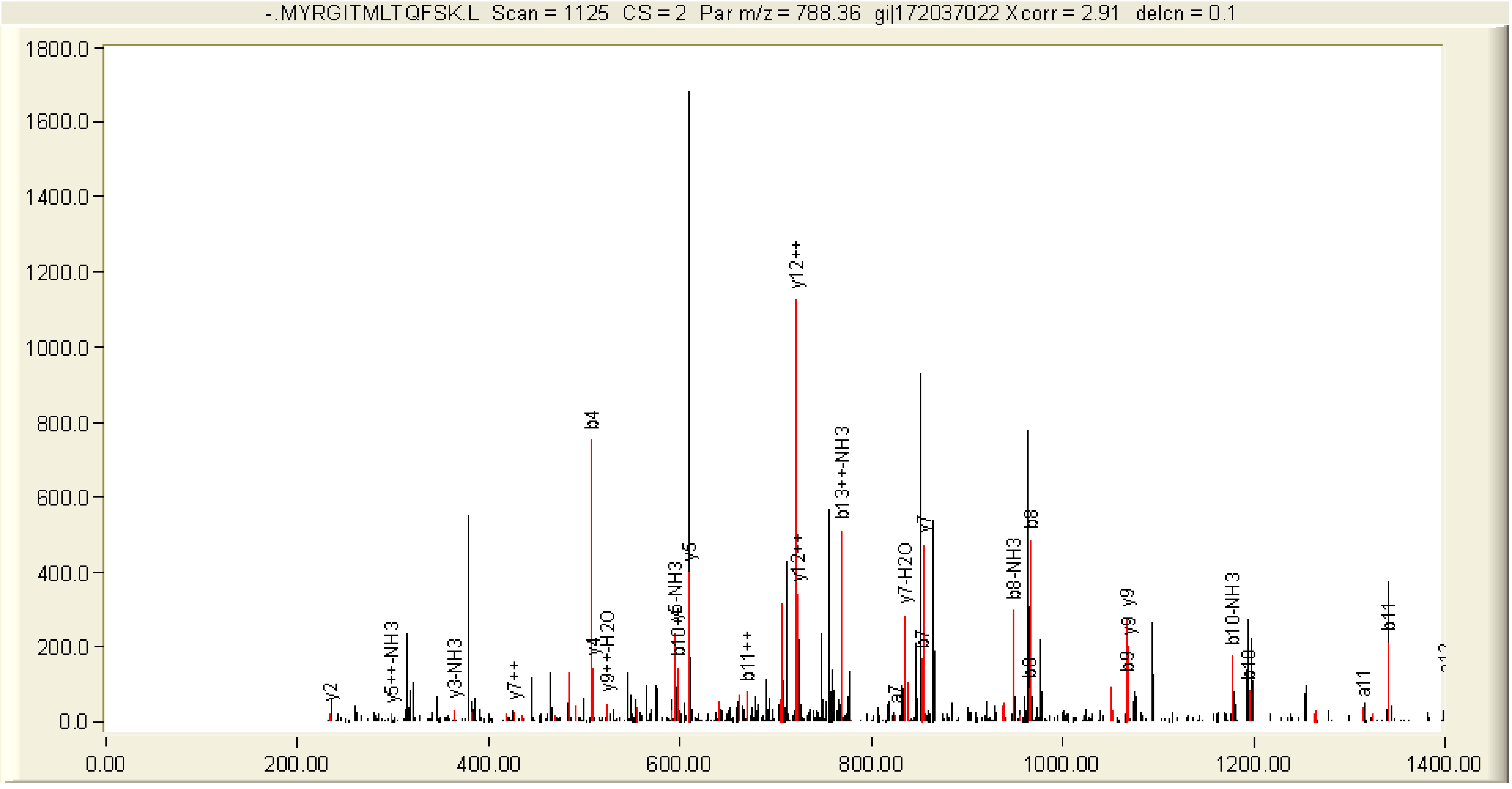

-.MYRGITMLTQFSK.L Scan = 1125 CS = 2 Par m/z = 788.36 gi|172037022 Xcorr = 2.91 delcn = 0.1
1800.0
1600.0
1400.0
1200.0
1000.0
800.0
600.0
400.0
200.0
0.0
0.00
200.00
400.00
600.00
800.00
1000.00
1200.00
1400.00
y2
y5++-NH3
y3-NH3
y7++
b4
y4
y9++-H2O
y5
y12++
y12++
b11++
b13++-NH3
y7-H2O
a7
y7
b7
b8-NH3
b8
b8
y9
y9
b9
b10-NH3
b10
a11
b11

R.KNFMAASTPLHENELIDCVR.A Scan = 1127 CS = 3 Par m/z = 764.06 gi|172038454 Xcorr = 4.53 delcn = 0.18

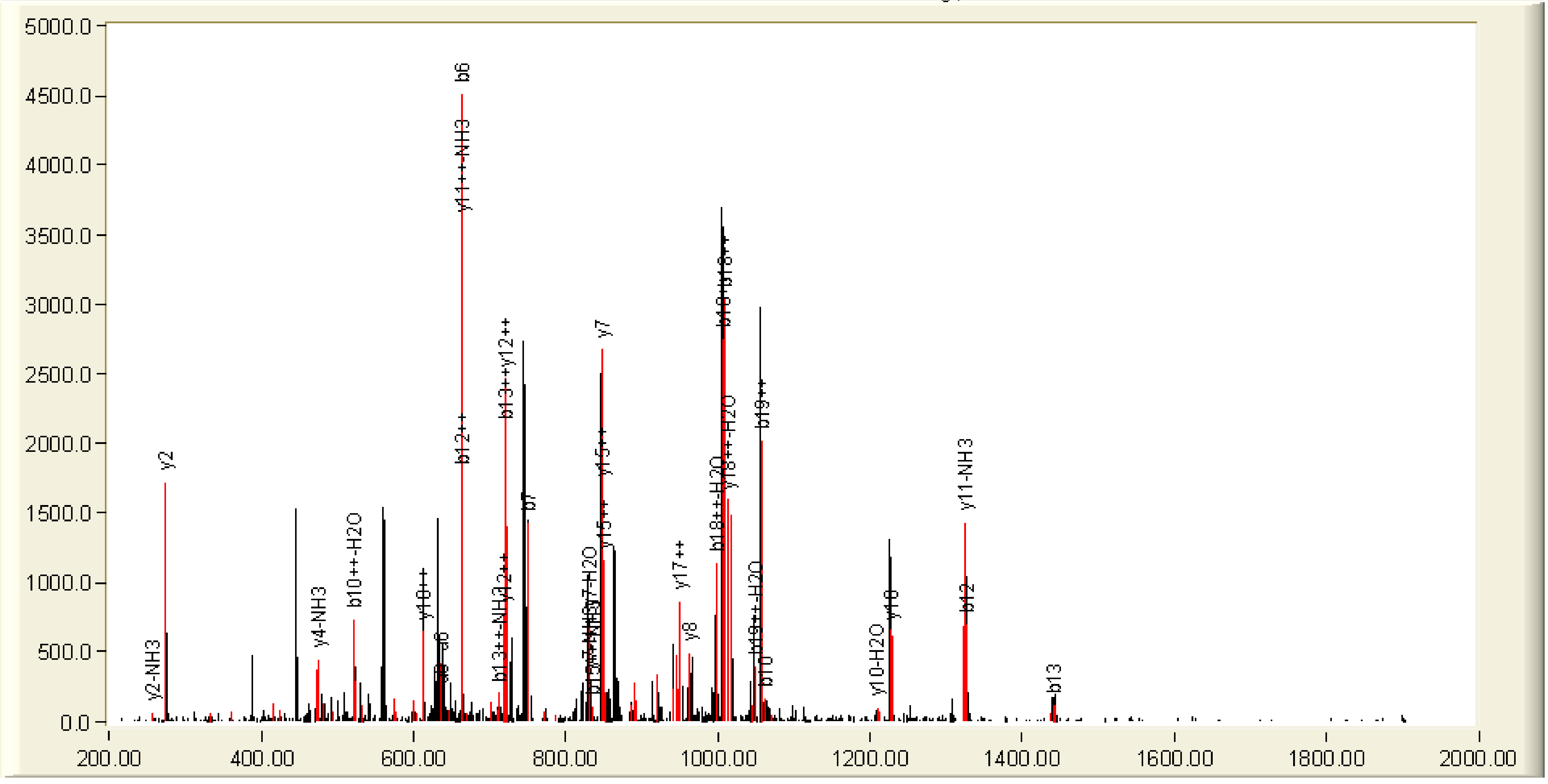

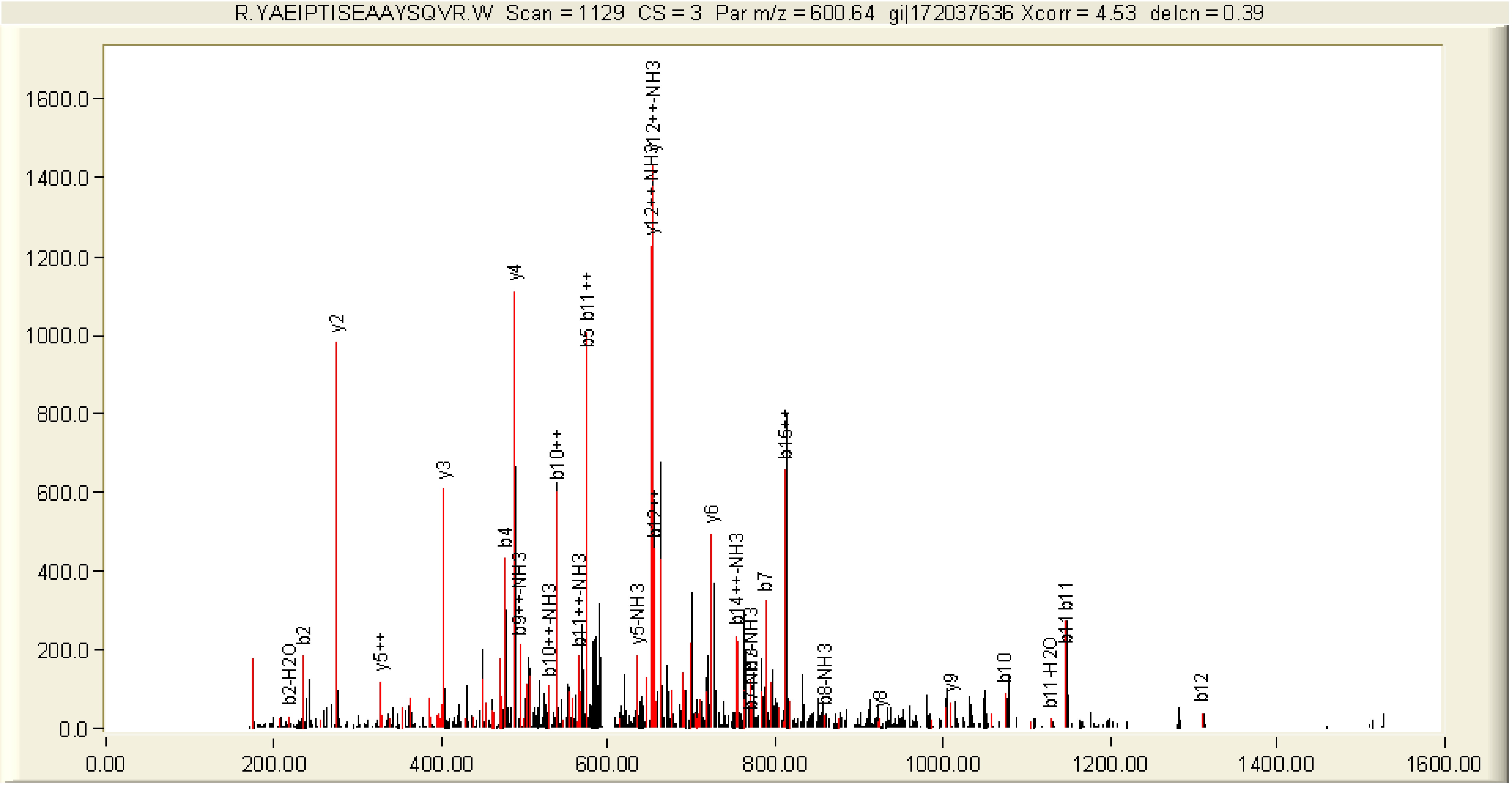
R.YAEIPTISEAAYSQVR.W Scan = 1129 CS = 3 Par m/z = 600.64 gi|172037636 Xcorr = 4.53 delcn = 0.39
1600.0
1400.0
1200.0
1000.0
800.0
600.0
400.0
200.0
0.0
0.00
200.00
400.00
600.00
800.00
1000.00
1200.00
1400.00
1600.00
y2
y3
y4
b4
b5 b11++
b10++
y12++-NH3
y12++-NH3
y6
b15++
b7
b14++-NH3
b2-H2O
b2
y5++
b9++-NH3
b10++-NH3
b11++-NH3
y5-NH3
b8-NH3
y8
y9
b10
b11-H2O
b11 b11
b12

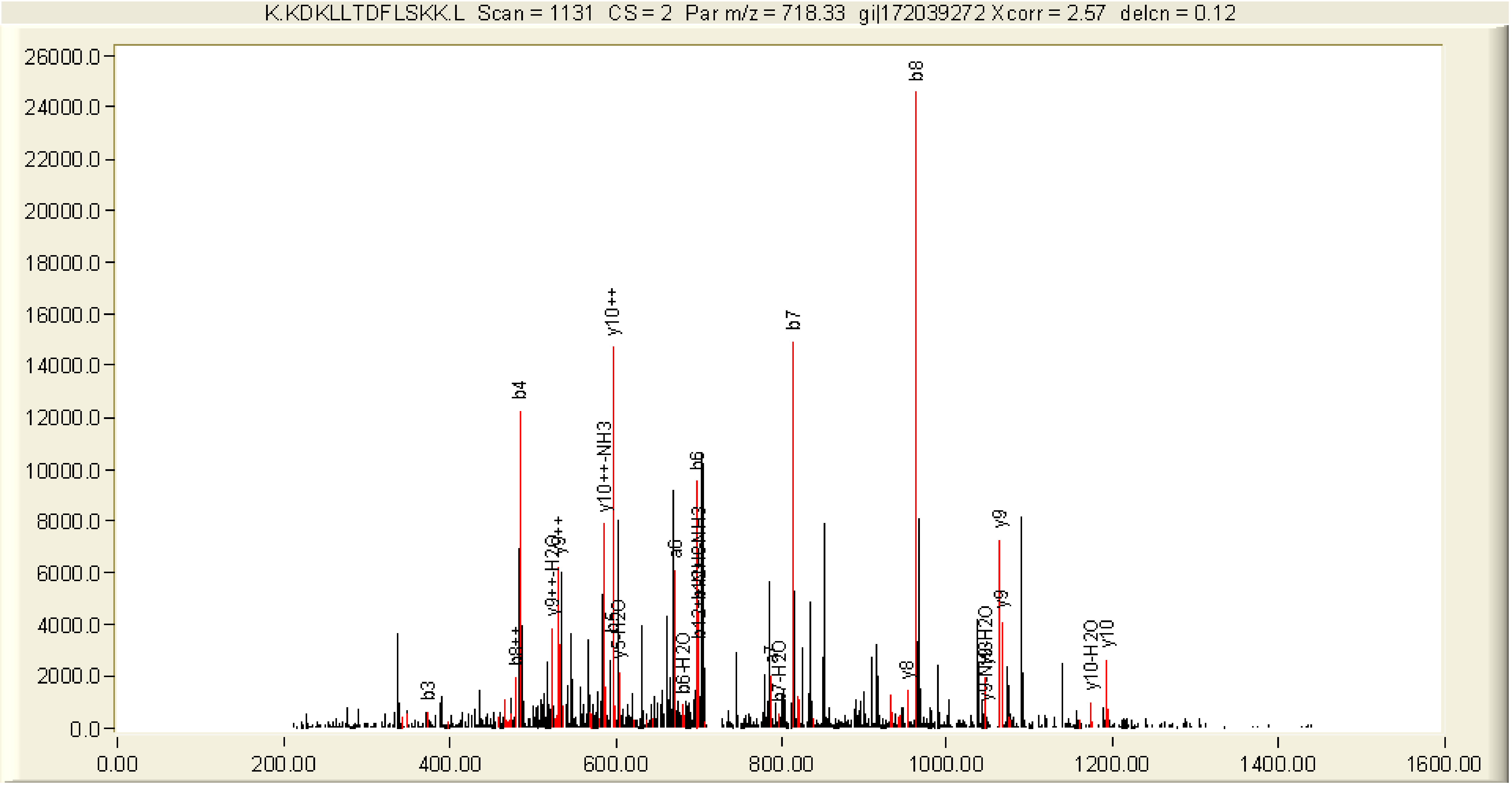
K.KDKLLTDFLSKK.L Scan = 1131 CS = 2 Par m/z = 718.33 gi|172039272 Xcorr = 2.57 delcn = 0.12
26000.0
24000.0
22000.0
20000.0
18000.0
16000.0
14000.0
12000.0
10000.0
8000.0
6000.0
4000.0
2000.0
0.0
0.00
200.00
400.00
600.00
800.00
1000.00
1200.00
1400.00
1600.00
b8
b7
y10++
b4
y10++-NH3
b6
a6
y9++
y9++-H2O
b8++
b3
y5-H2O
b6-H2O
b7-H2O
y8
y9
y10-H2O
y10

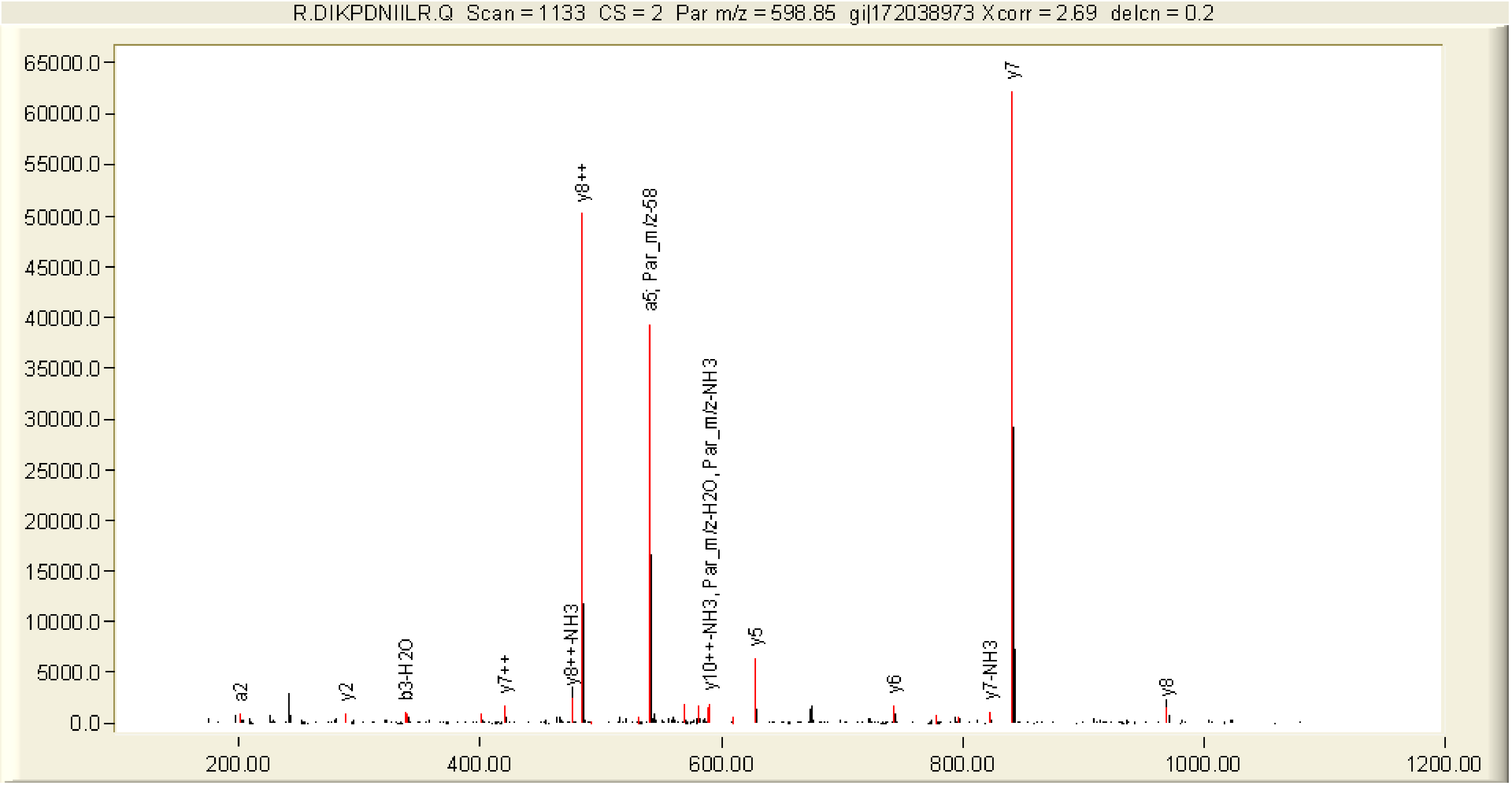

R.DIKPDNIILR.Q Scan = 1133 CS = 2 Par m/z = 598.85 gi|172038973 Xcorr = 2.69 delcn = 0.2
65000.0
60000.0
55000.0
50000.0
45000.0
40000.0
35000.0
30000.0
25000.0
20000.0
15000.0
10000.0
5000.0
0.0
200.00
400.00
600.00
800.00
1000.00
1200.00
a2
y2
b3-H2O
y7++
y8++-NH3
y8++
a5; Par_m/z-58
y10++-NH3, Par_m/z-H2O, Par_m/z-NH3
y5
y6
y7-NH3
y7
y8

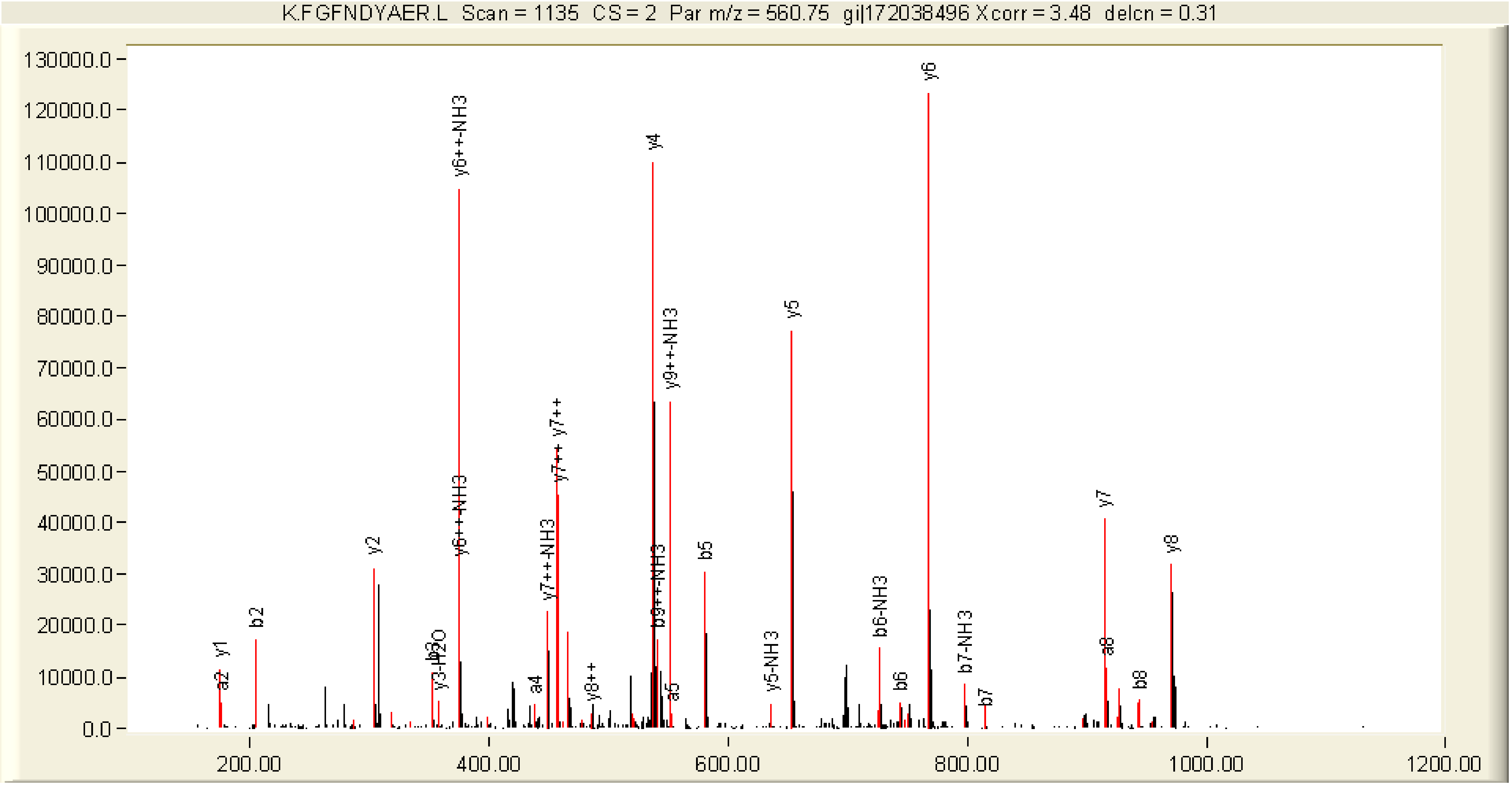
K.FGFNDYAER.L Scan = 1135 CS = 2 Par m/z = 560.75 gi|172038496 Xcorr = 3.48 delcn = 0.31
130000.0
120000.0
110000.0
100000.0
90000.0
80000.0
70000.0
60000.0
50000.0
40000.0
30000.0
20000.0
10000.0
0.0
200.00
400.00
600.00
800.00
1000.00
1200.00
a2
y1
b2
y2
y3-H2O
b3
y6++-NH3
y6++-NH3
a4
y7++-NH3
y7++
y7++
y8++
b9++-NH3
y4
y9++-NH3
a5
b5
y5-NH3
y5
b6-NH3
b6
y6
b7-NH3
b7
y7
a8
b8
y8

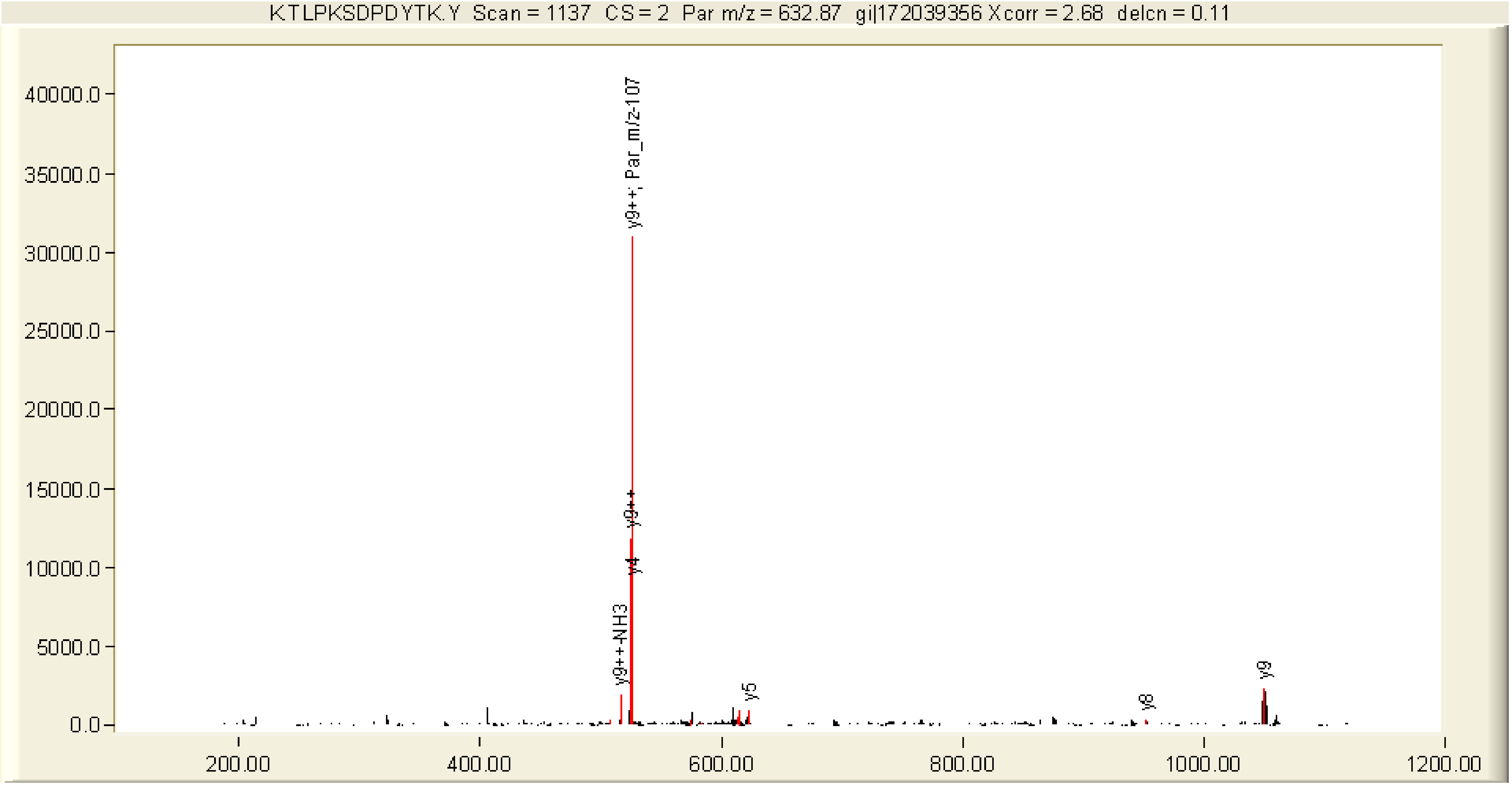

KTLPKSDPDYTK.Y Scan = 1137 CS = 2 Par m/z = 632.87 gi|172039356 Xcorr = 2.68 delcn = 0.11
y9++; Par_m/z-107
y9++
y4
y9++-NH3
y5
y8
y9
40000.0
35000.0
30000.0
25000.0
20000.0
15000.0
10000.0
5000.0
0.0
200.00
400.00
600.00
800.00
1000.00
1200.00

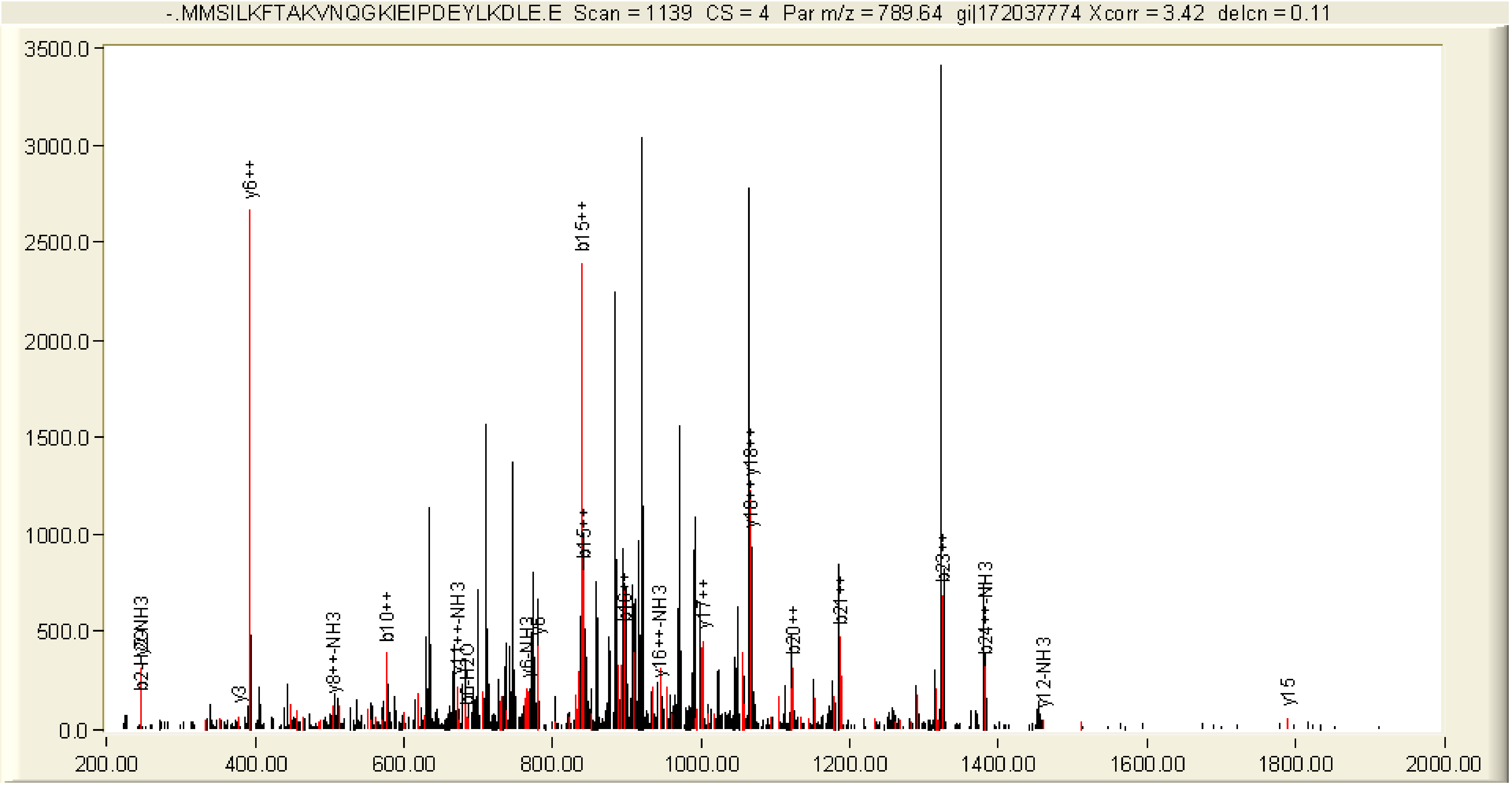

-.MMSILKFTAKVNQGKIEIPDEYLKDLE.E Scan = 1139 CS = 4 Par m/z = 789.64 gi|172037774 Xcorr = 3.42 delcn = 0.11
y6++
b15++
y18++
b23++
b21++
b20++
y17++
y16++-NH3
b24++-NH3
y12-NH3
y15
b10++
y8++-NH3
y3
y6-NH3
y6
3500.0
3000.0
2500.0
2000.0
1500.0
1000.0
500.0
0.0
200.00
400.00
600.00
800.00
1000.00
1200.00
1400.00
1600.00
1800.00
2000.00

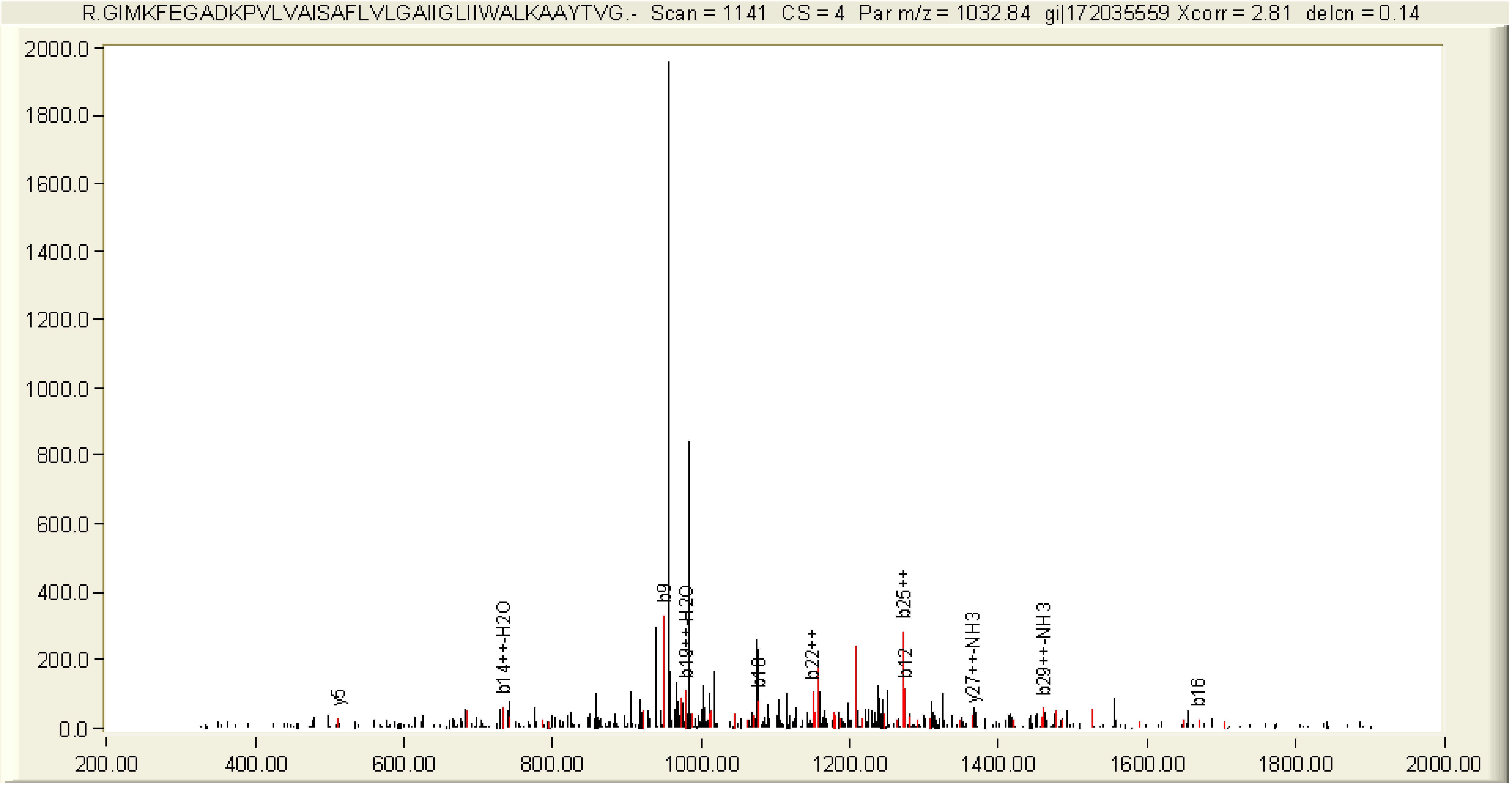

R.GIMKFEGADKPVLVAISAFLVLGAIIGLIIWALKAAYTVG.- Scan = 1141 CS = 4 Par m/z = 1032.84 gi|172035559 Xcorr = 2.81 delcn = 0.14
2000.0
1800.0
1600.0
1400.0
1200.0
1000.0
800.0
600.0
400.0
200.0
0.0
y5
b14++-H2O
b9
b19++-H2O
b19++
b18
b22++
b25++
b12
y27++-NH3
b29++-NH3
b16
200.00
400.00
600.00
800.00
1000.00
1200.00
1400.00
1600.00
1800.00
2000.00

K.ISLPTALDYVMACLTKYRLPETTRWADKLASDK.- Scan = 1143 CS = 3 Par m/z = 1257.26 gi|172039482 Xcorr = 3.73 delcn = 0.13

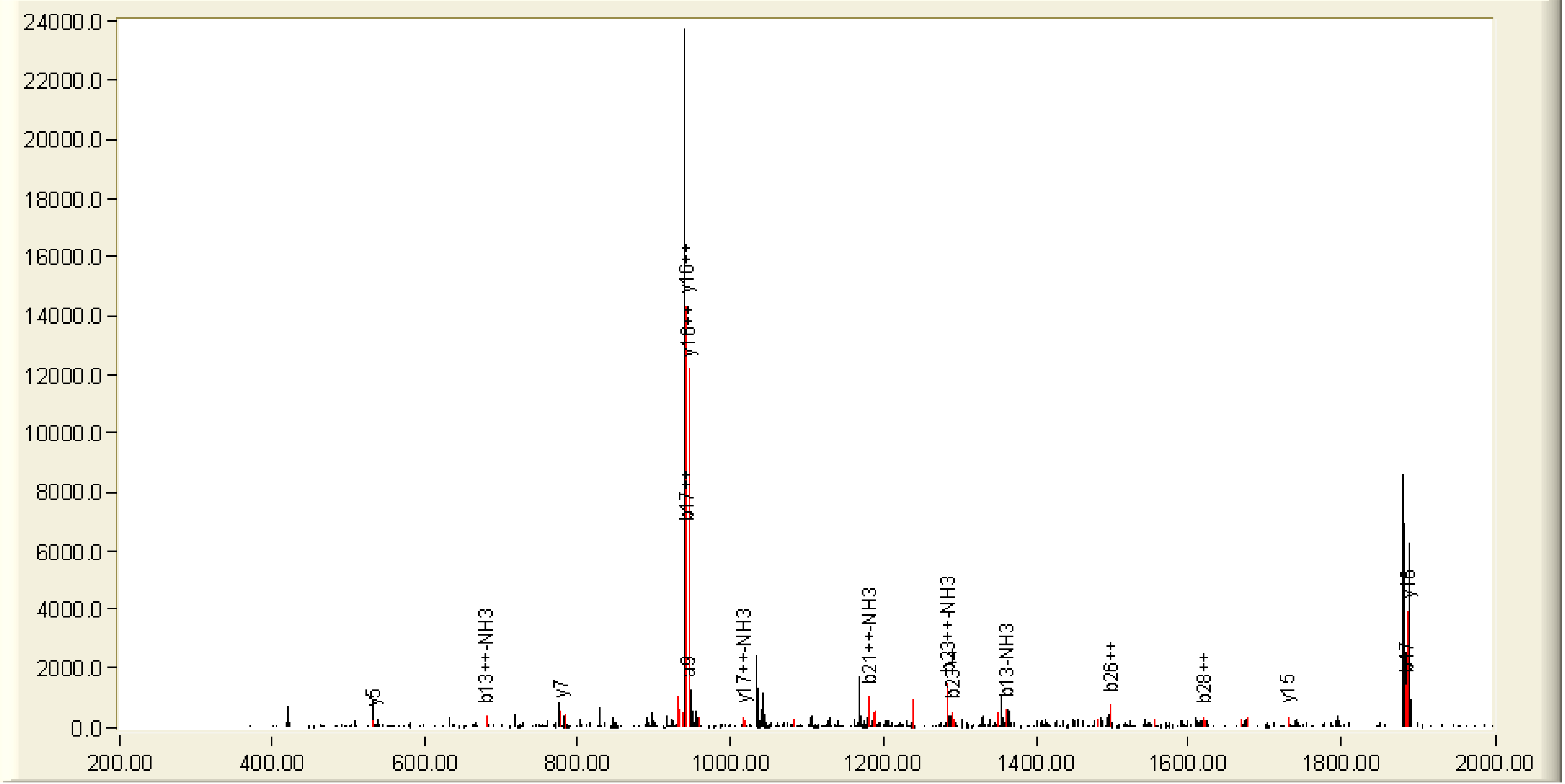

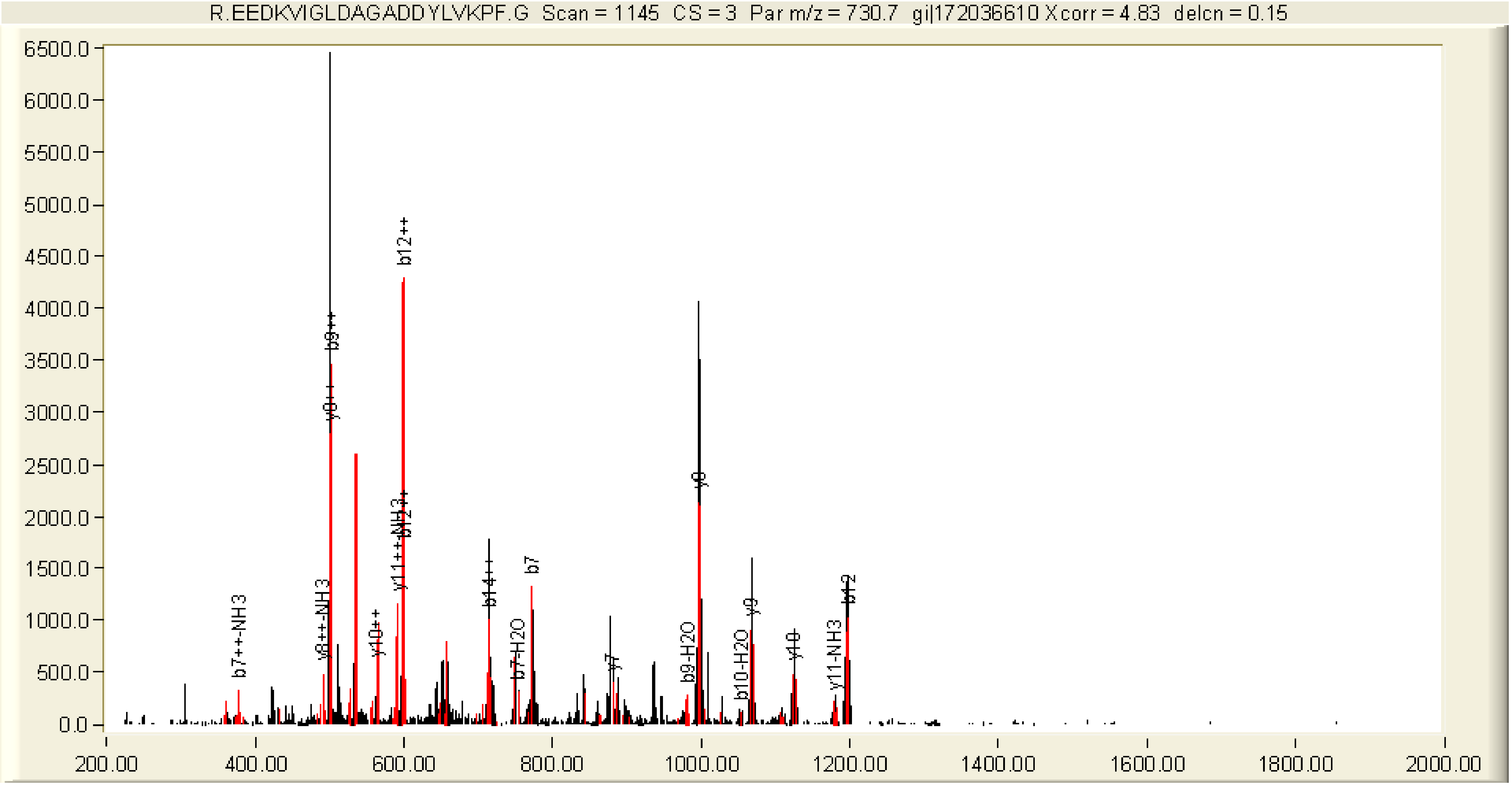
R.EEDKVIGLDAGADDYLVKPF.G Scan = 1145 CS = 3 Par m/z = 730.7 gi|172036610 Xcorr = 4.83 delcn = 0.15
6500.0
6000.0
5500.0
5000.0
4500.0
4000.0
3500.0
3000.0
2500.0
2000.0
1500.0
1000.0
500.0
0.0
b7++-NH3
y8++-NH3
b9++
y9++
y10++
y11++-NH3
b12++
b12++
b14++
b7-H2O
b7
y7
b9-H2O
y8
b10-H2O
y9
y10
y11-NH3
b12
200.00
400.00
600.00
800.00
1000.00
1200.00
1400.00
1600.00
1800.00
2000.00

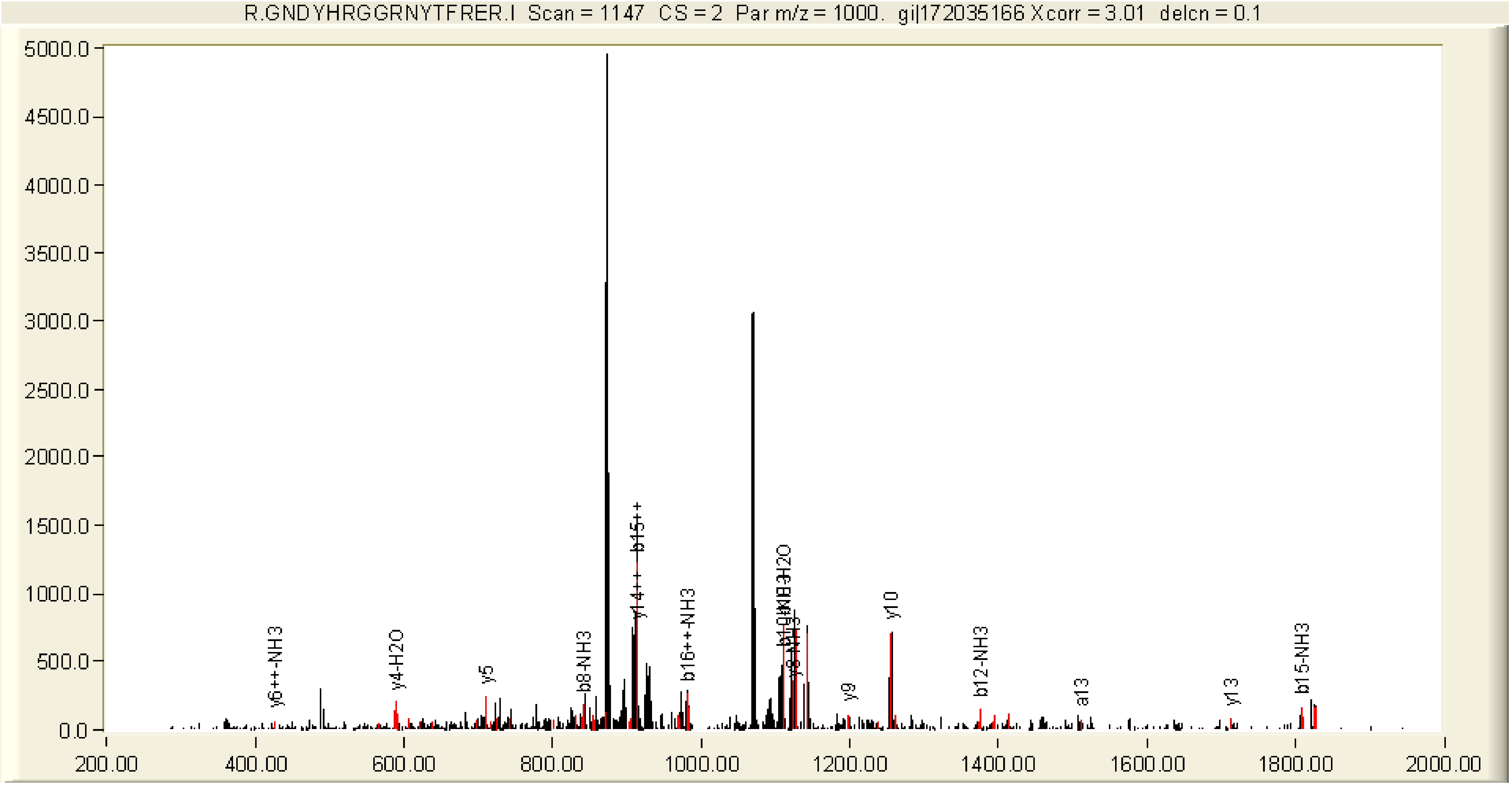
R.GNDYHRGGRNYTFRER.I Scan = 1147 CS = 2 Par m/z = 1000. gi|172035166 Xcorr = 3.01 delcn = 0.1
5000.0
4500.0
4000.0
3500.0
3000.0
2500.0
2000.0
1500.0
1000.0
500.0
0.0
y6++-NH3
y4-H2O
y5
b8-NH3
y14++
b15++
b16++-NH3
y9
y10
b12-NH3
a13
y13
b15-NH3
200.00
400.00
600.00
800.00
1000.00
1200.00
1400.00
1600.00
1800.00
2000.00

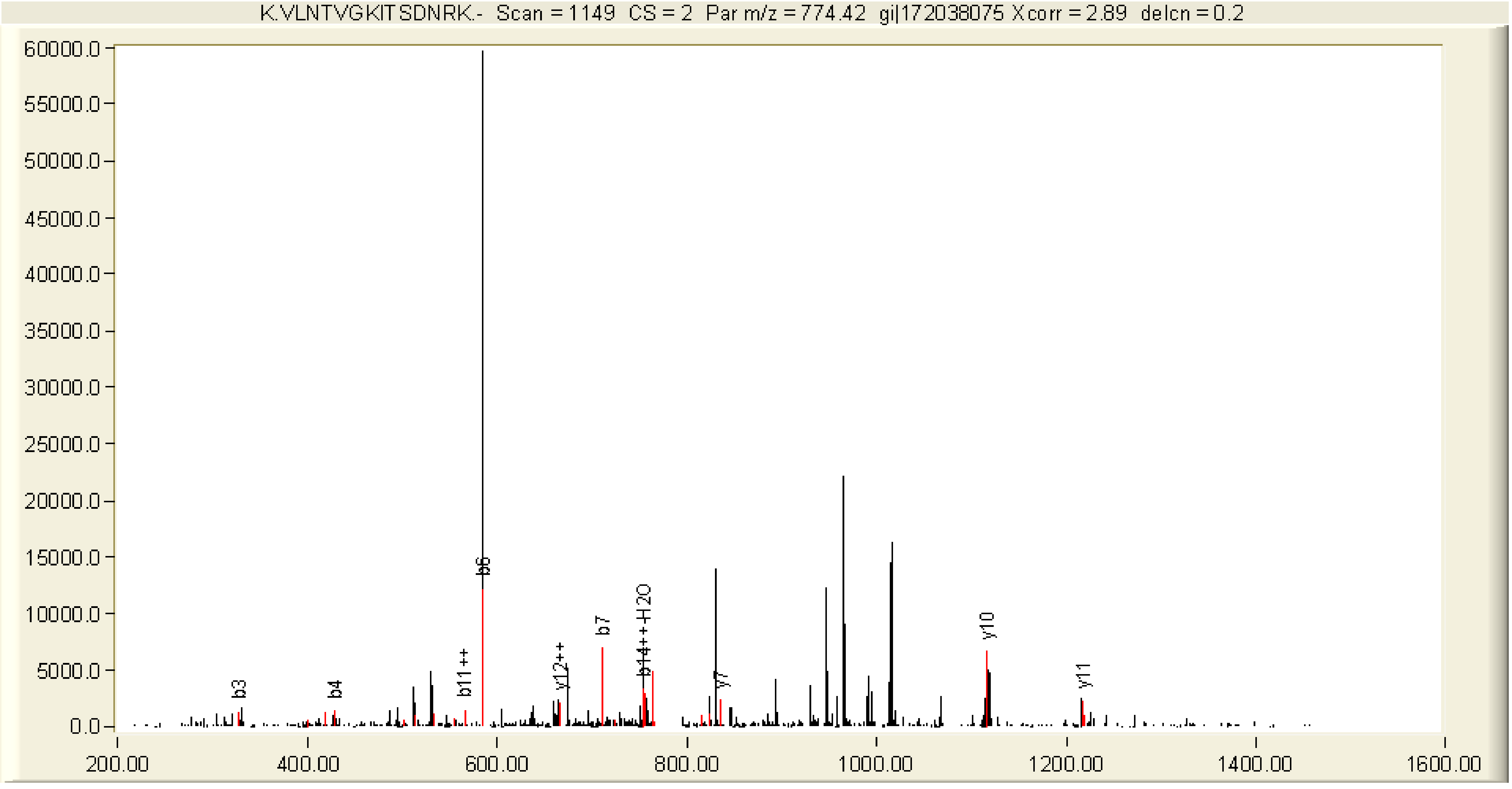
K.VLNTVGKITSDNRK.- Scan = 1149 CS = 2 Par m/z = 774.42 gi|172038075 Xcorr = 2.89 delcn = 0.2
60000.0
55000.0
50000.0
45000.0
40000.0
35000.0
30000.0
25000.0
20000.0
15000.0
10000.0
5000.0
0.0
200.00
400.00
600.00
800.00
1000.00
1200.00
1400.00
1600.00
b3
b4
b11++
b6
y12++
b7
b14++H2O
y7
y10
y11

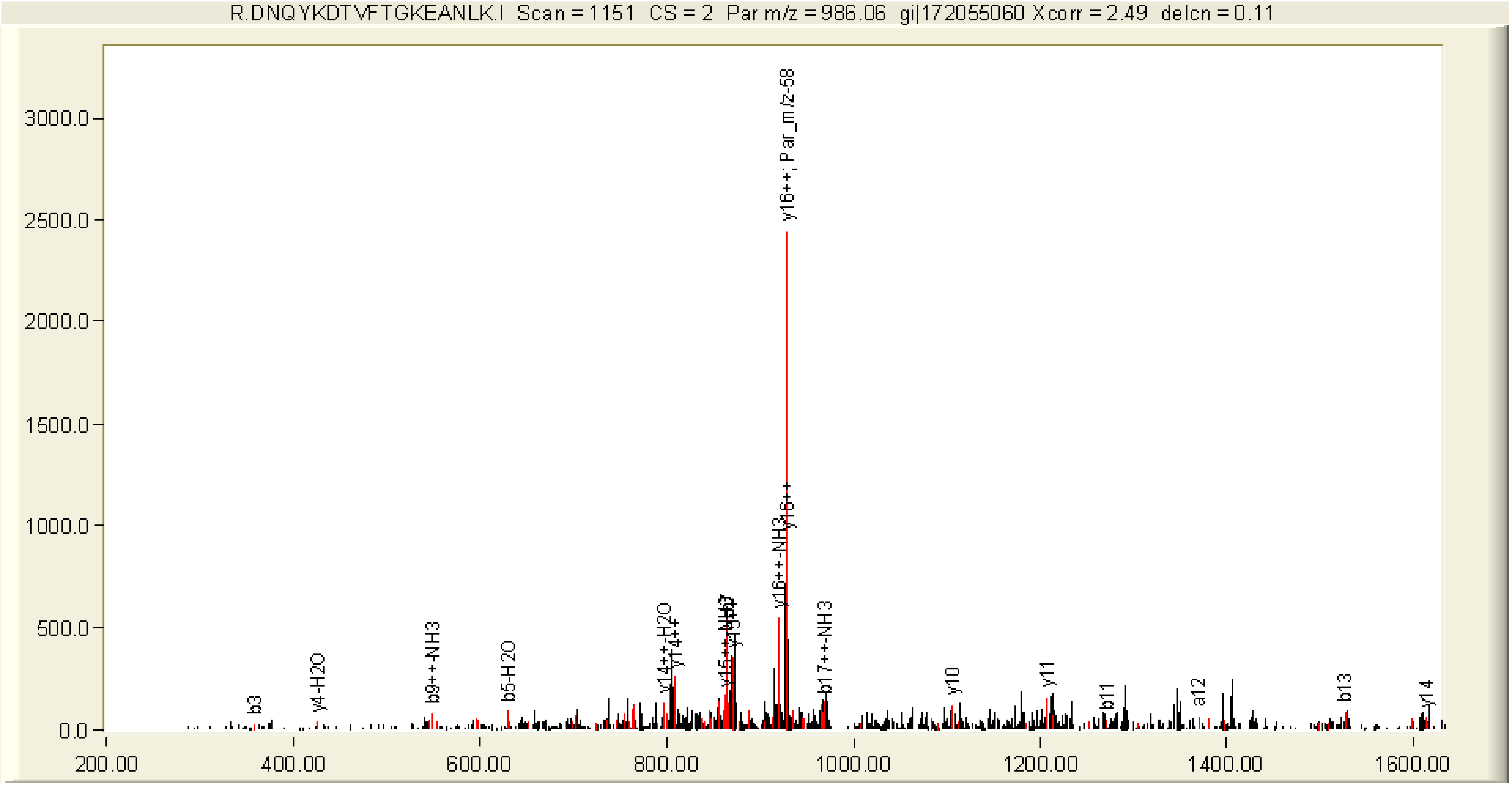
R.DNQYKDTVFTGKEANLK.I Scan = 1151 CS = 2 Par m/z = 986.06 gi|172055060 Xcorr = 2.49 delcn = 0.11
y16++; Par_m/z-58
y16++
y16++-NH3
b17++-NH3
y14++-H2O
y14++
b3
y4-H2O
b9++-NH3
b5-H2O
y10
y11
b11
a12
b13
y14
3000.0
2500.0
2000.0
1500.0
1000.0
500.0
0.0
200.00
400.00
600.00
800.00
1000.00
1200.00
1400.00
1600.00

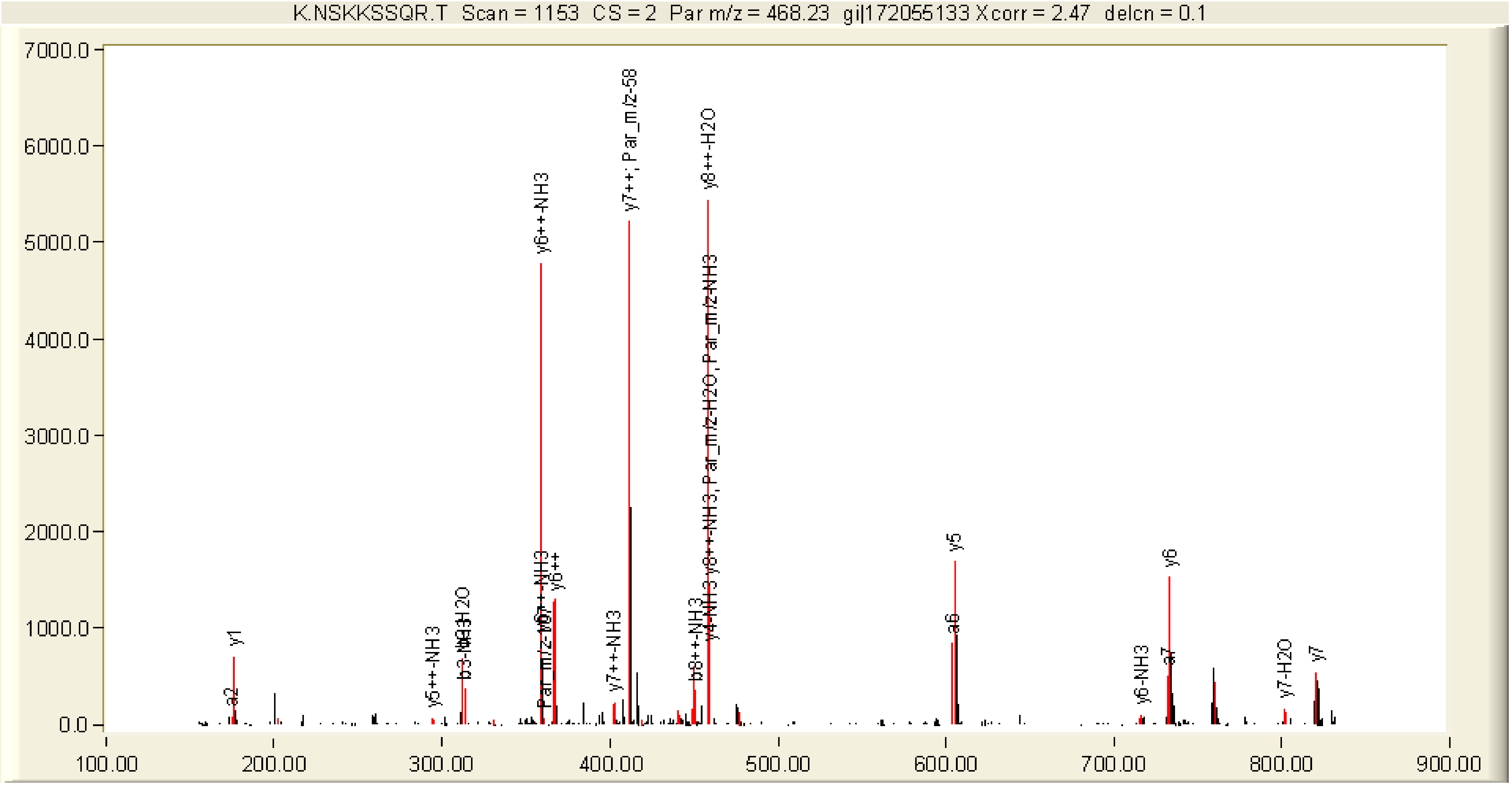
K.NSKKSSQR.T Scan = 1153 CS = 2 Par m/z = 468.23 gi|172055133 Xcorr = 2.47 delcn = 0.1
7000.0
6000.0
5000.0
4000.0
3000.0
2000.0
1000.0
0.0
100.00
200.00
300.00
400.00
500.00
600.00
700.00
800.00
900.00
y1
a2
y5++-NH3
y6++-NH3
y7++; Par_m/z-58
y8++-H2O
y6++
y7++-NH3
b8++-NH3
y5
a6
y6
y6-NH3
a7
y7-H2O
y7

R.VAIGNEVSR.G Scan = 1155 CS = 2 Par m/z = 472.76 gi|172038784 Xcorr = 2.47 delcn = 0.24

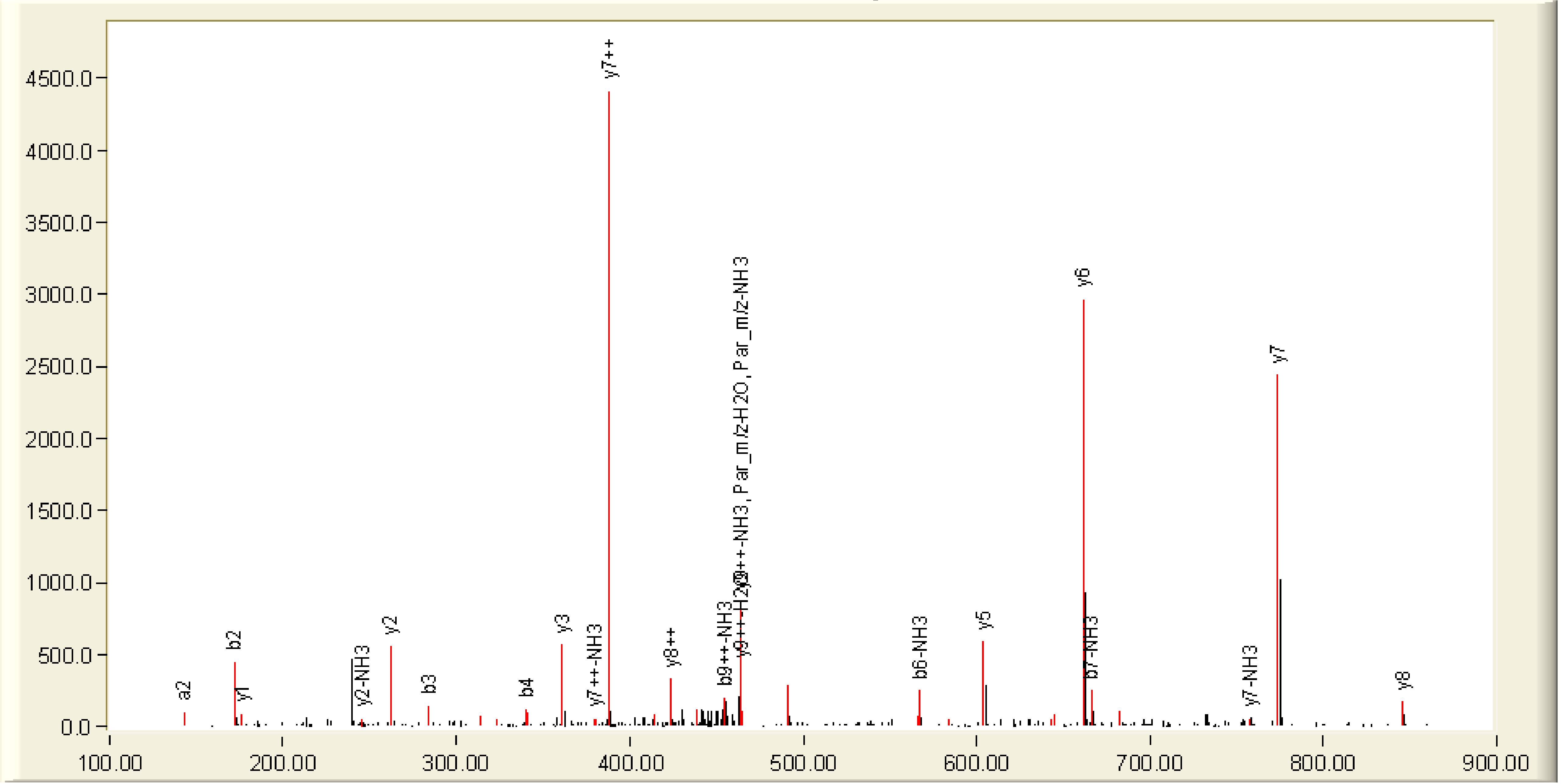

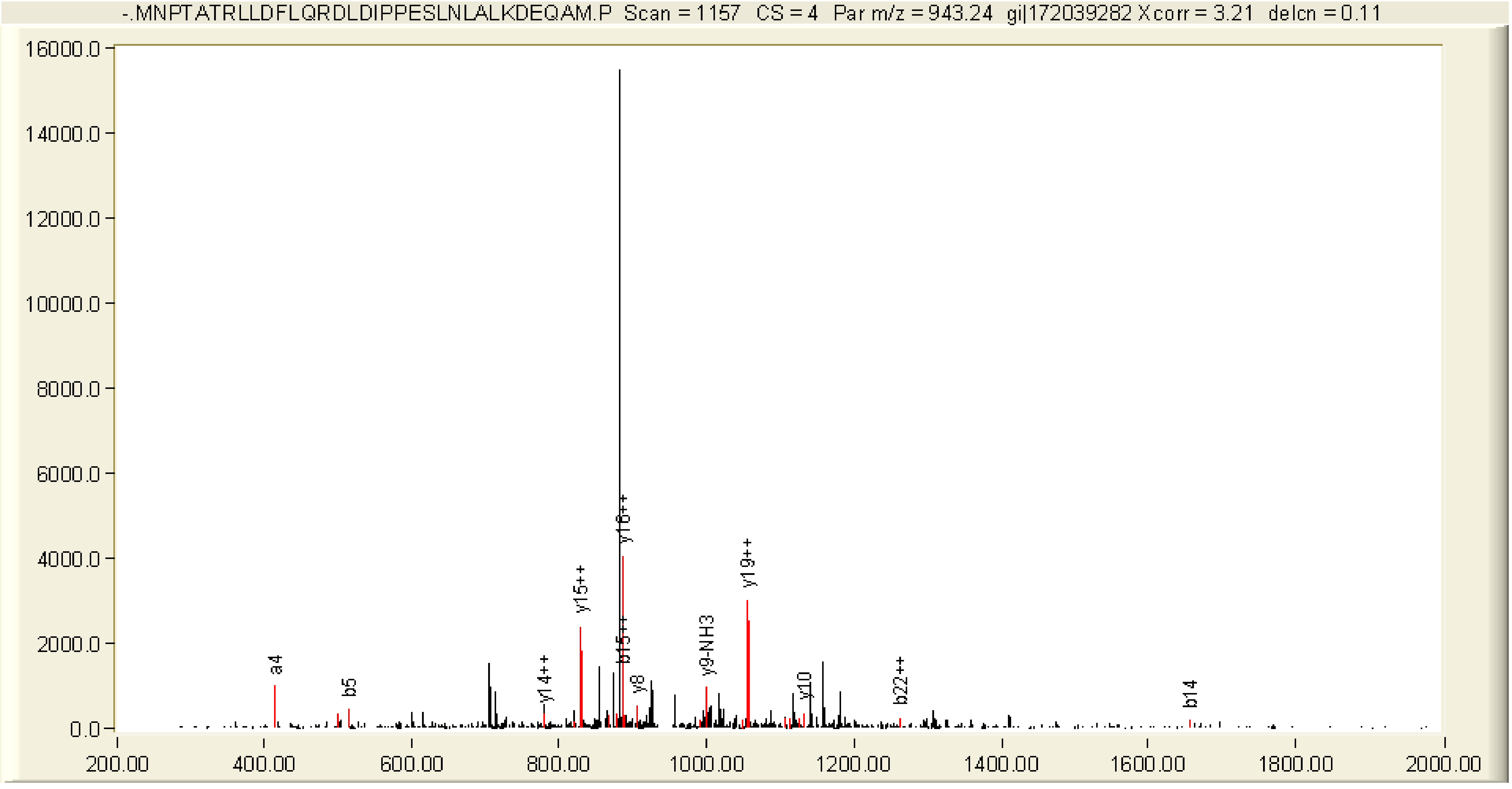

-.MNPTATRLLDFLQRDLDIPPESLNLALKDEQAM.P Scan = 1157 CS = 4 Par m/z = 943.24 gi|172039282 Xcorr = 3.21 delcn = 0.11
16000.0
14000.0
12000.0
10000.0
8000.0
6000.0
4000.0
2000.0
0.0
200.00
400.00
600.00
800.00
1000.00
1200.00
1400.00
1600.00
1800.00
2000.00
a4
b5
y14++
y15++
b15++
y16++
y8
y9-NH3
y19++
y10
b22++
b14

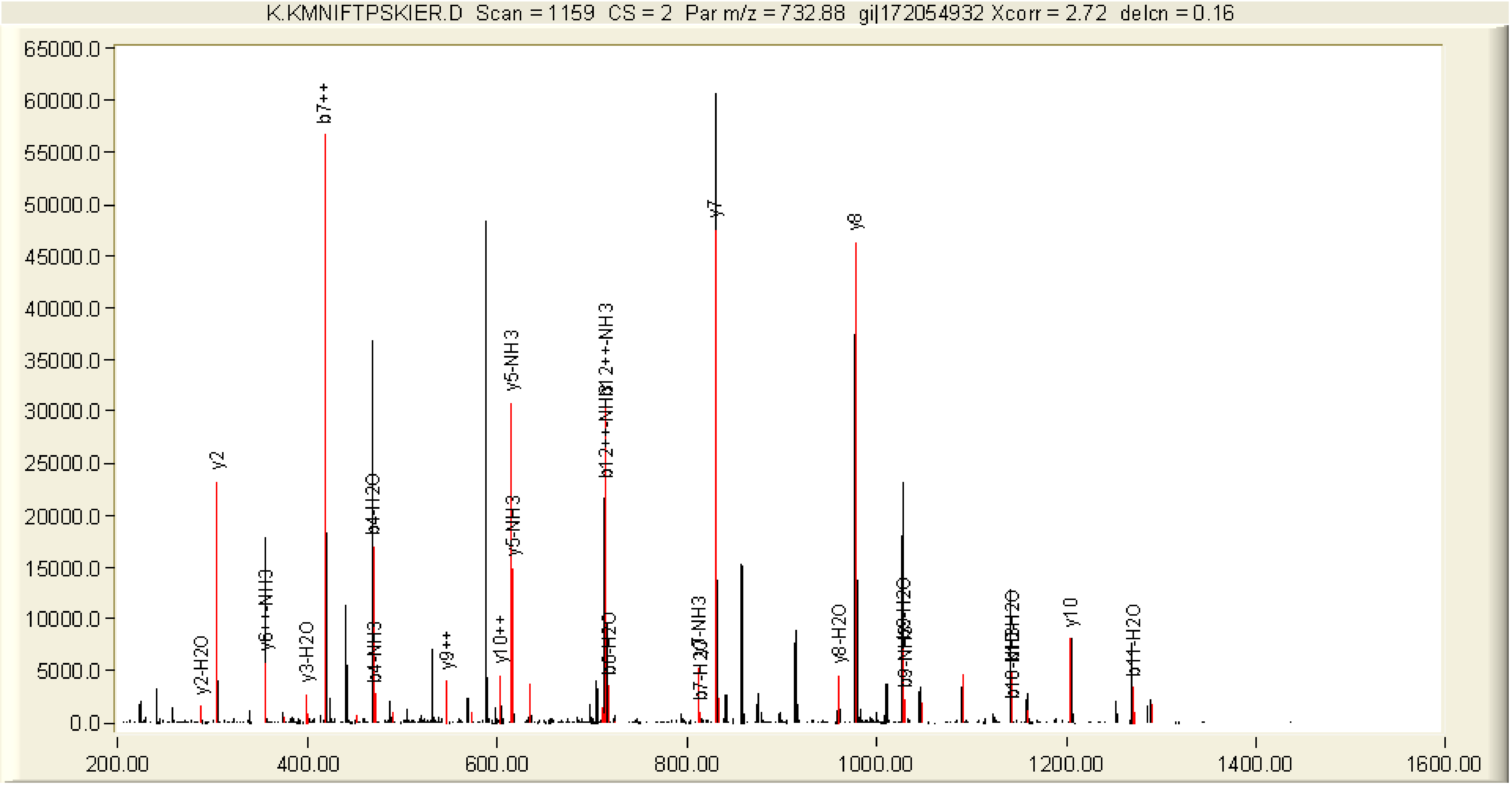
K.KMNIFTPSKIER.D Scan = 1159 CS = 2 Par m/z = 732.88 gi|172054932 Xcorr = 2.72 delcn = 0.16
65000.0
60000.0
55000.0
50000.0
45000.0
40000.0
35000.0
30000.0
25000.0
20000.0
15000.0
10000.0
5000.0
0.0
200.00
400.00
600.00
800.00
1000.00
1200.00
1400.00
1600.00
b7++
y2
y2-H2O
y6++-NH3
y3-H2O
b4-H2O
b4-NH3
y9++
y10++
y5-NH3
b12++-NH3
y7
y8
y8-H2O
y10
b11-H2O

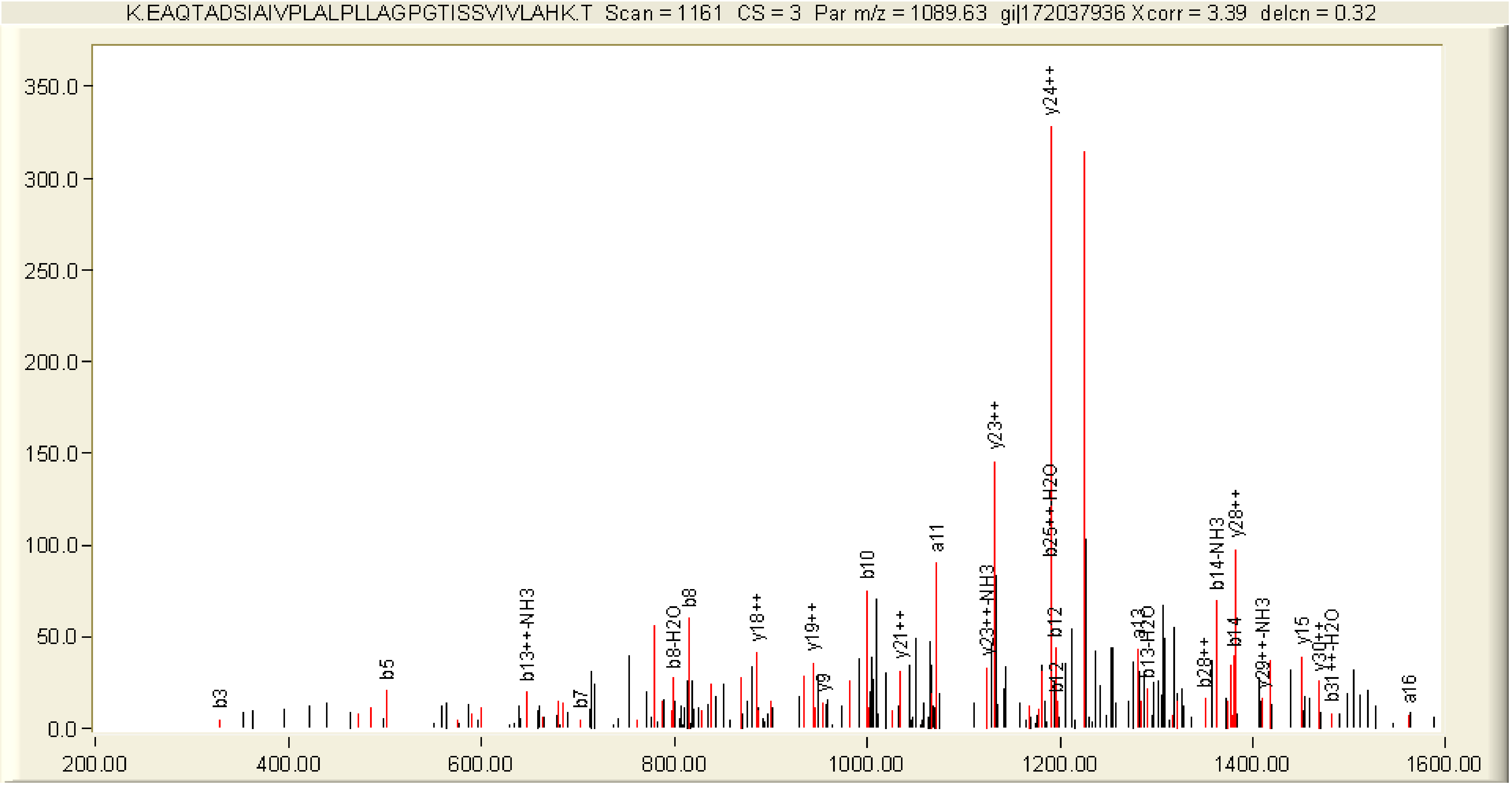

K.EAQTADSIAIVPLALPLLAGPGTISSVIVLAHK.T Scan = 1161 CS = 3 Par m/z = 1089.63 gi|172037936 Xcorr = 3.39 delcn = 0.32
y24++
y23++
b25++-H2O
a11
b10
y28++
b14-NH3
b13++-NH3
b8
b8-H2O
y18++
y19++
y21++
y23++-NH3
b12
y29++-NH3
b14
y15
b5
b3
b7
y9
b28++
a16

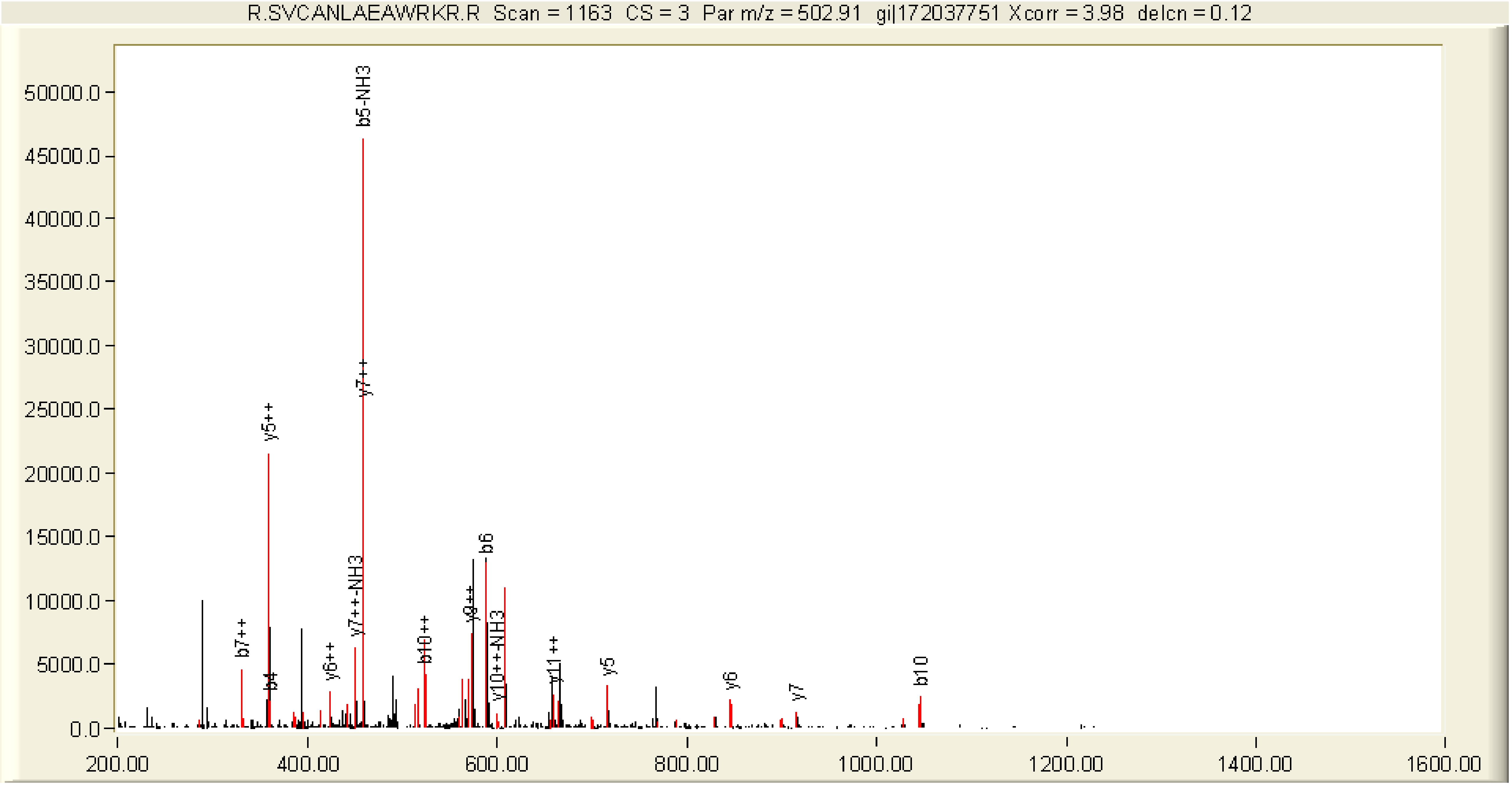
R.SVCANLAEAWRKR.R Scan = 1163 CS = 3 Par m/z = 502.91 gi|172037751 Xcorr = 3.98 delcn = 0.12
50000.0
45000.0
40000.0
35000.0
30000.0
25000.0
20000.0
15000.0
10000.0
5000.0
0.0
200.00
400.00
600.00
800.00
1000.00
1200.00
1400.00
1600.00
b5-NH3
y7++
y5++
b6
y7++-NH3
y9++
y10++-NH3
b7++
b4
y6++
b10++
y11++
y5
y6
y7
b10

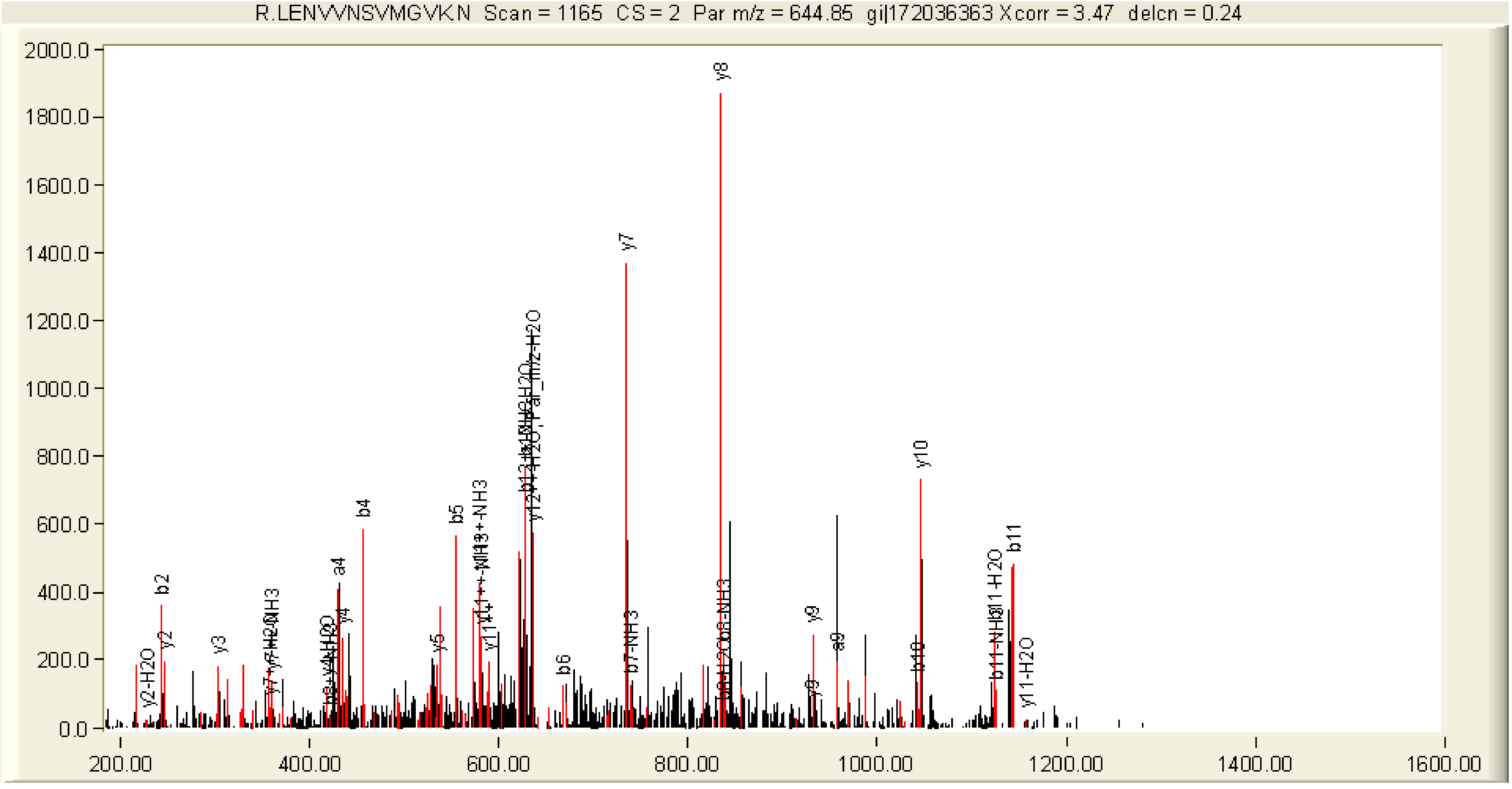

R.LENVVNSVMGVKN Scan = 1165 CS = 2 Par m/z = 644.85 gi|172036363 Xcorr = 3.47 delcn = 0.24
2000.0
1800.0
1600.0
1400.0
1200.0
1000.0
800.0
600.0
400.0
200.0
0.0
200.00
400.00
600.00
800.00
1000.00
1200.00
1400.00
1600.00
y8
y7
y10
b4
b5
b2
a4
y4
y2
y3
y5
b6
b7-NH3
y9
a9
b11
b11-H2O
b11-NH3
y11-H2O
y2-H2O

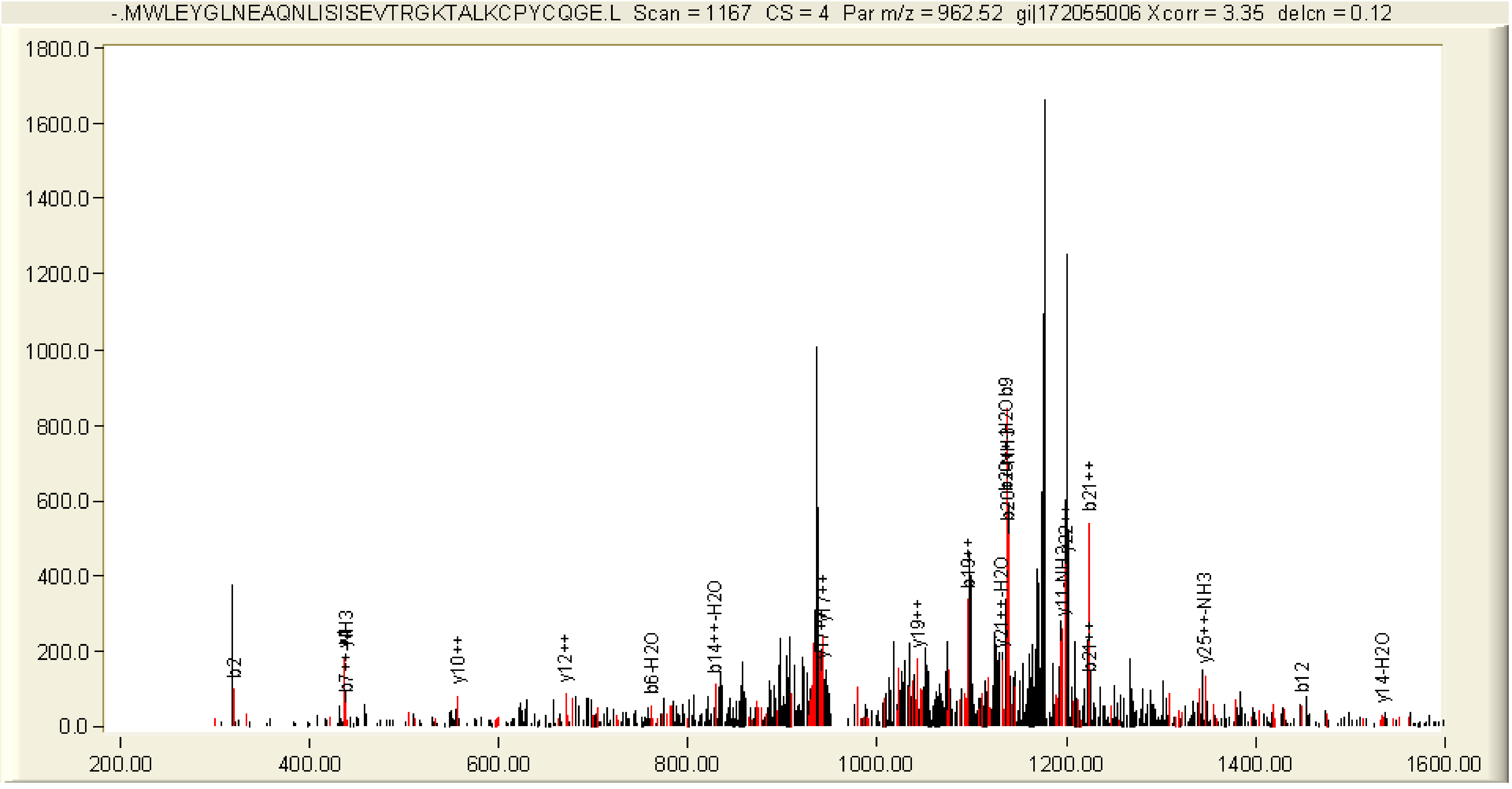
-.MWLEYGLNEAQNLISISEVTRGKTALKCPYCQGE.L Scan = 1167 CS = 4 Par m/z = 962.52 gi|172055006 Xcorr = 3.35 delcn = 0.12
1800.0
1600.0
1400.0
1200.0
1000.0
800.0
600.0
400.0
200.0
0.0
200.00
400.00
600.00
800.00
1000.00
1200.00
1400.00
1600.00
b2
y10++
y12++
b6-H2O
b14++-H2O
y19++
b19++
y21++-H2O
y11-NH3
b21++
y25++-NH3
b12
y14-H2O

K.QALEQGHEVTGFARNPLKLDIKHPK.L Scan = 1169 CS = 3 Par m/z = 943.79 gi|172036684 Xcorr = 3.31 delcn = 0.18

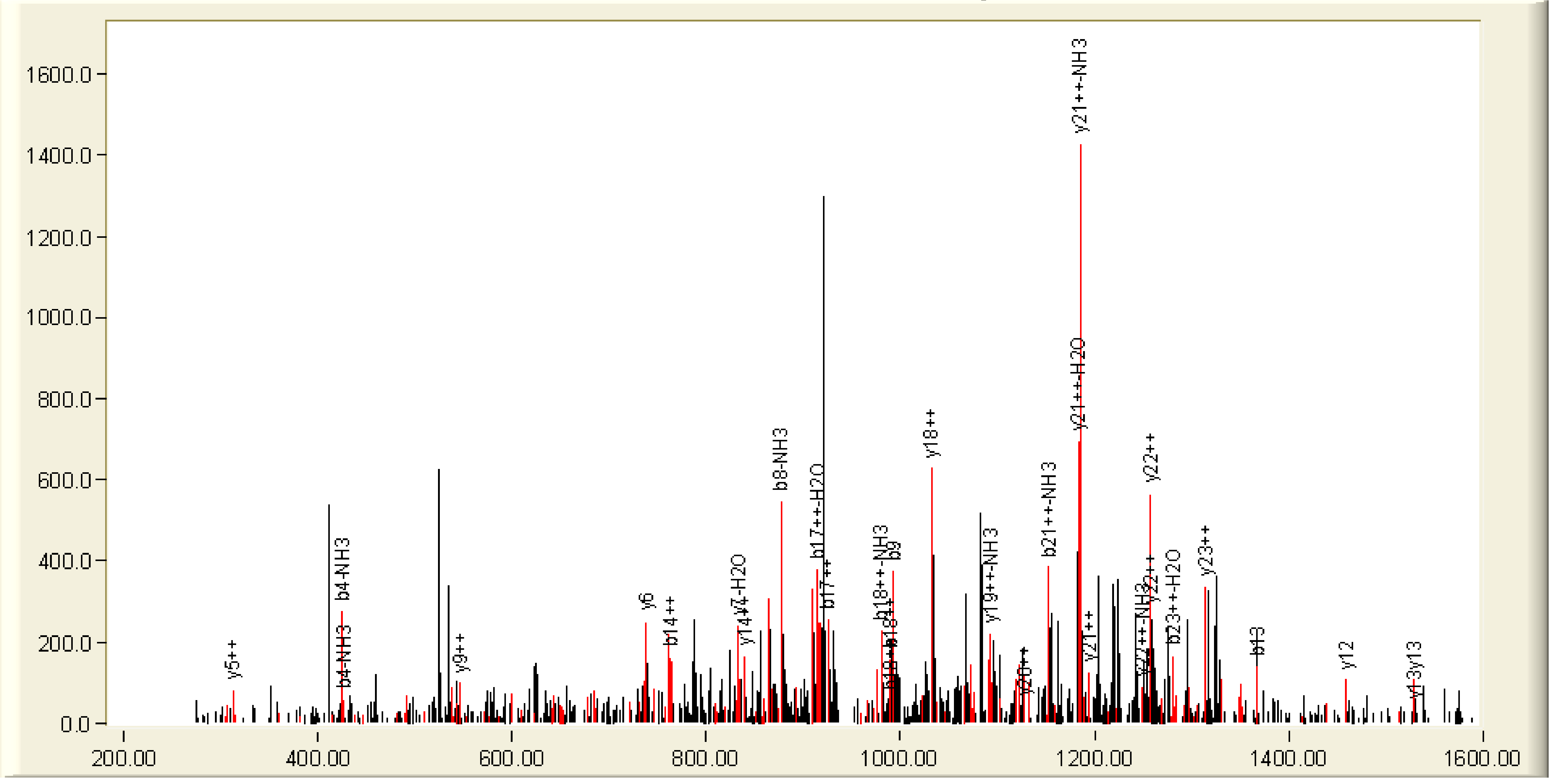

KAQPIATEDKPR.R Scan = 1171 CS = 2 Par m/z = 613.83 gi|172038495 Xcorr = 3.31 delcn = 0.26

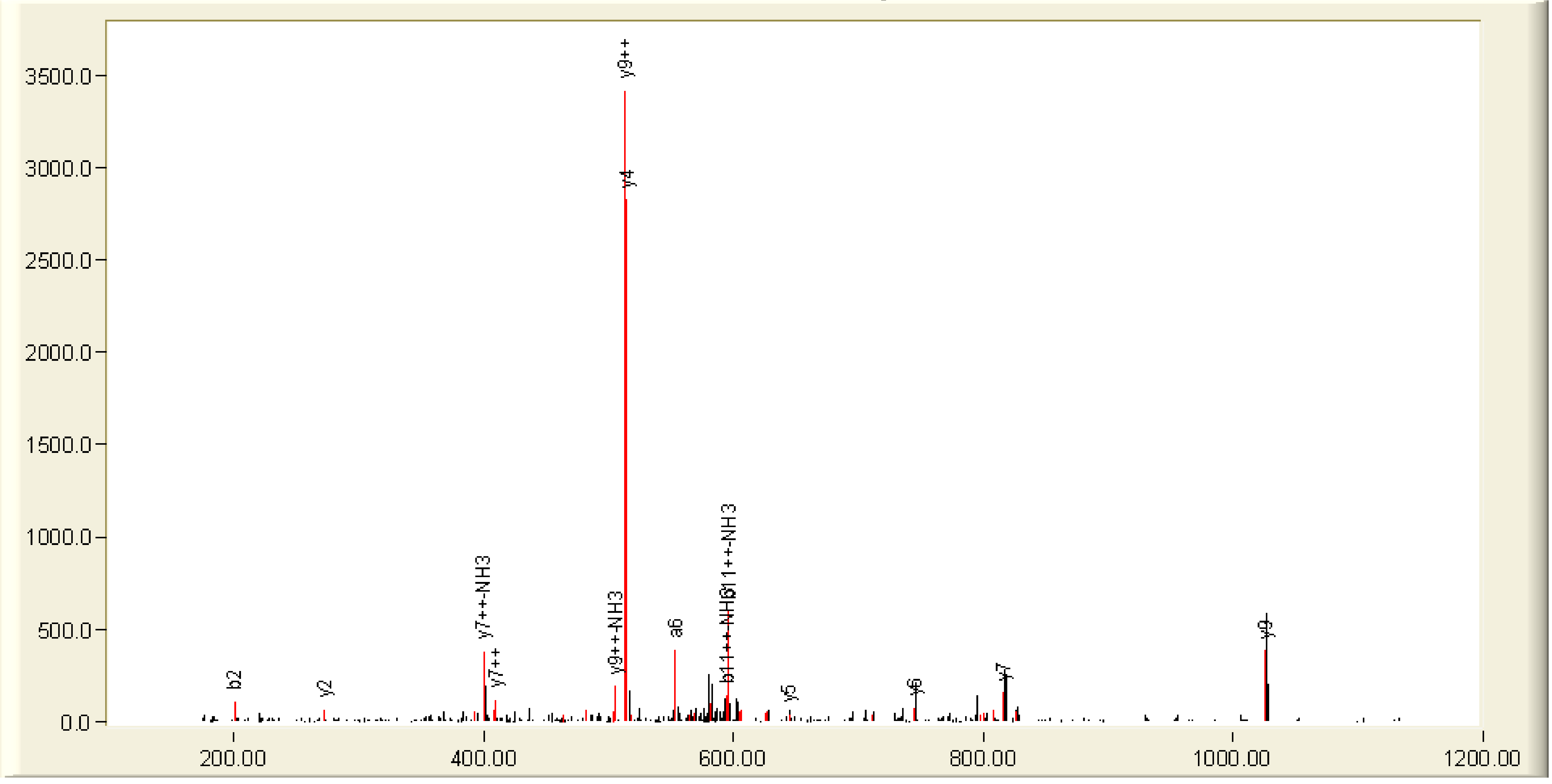

L.ETISLLILNRAVLRFIVEIKHEKMAKFMA.-   Scan = 1173   CS = 4   Par m/z = 853.63   gi|172054917 Xcorr = 3.01   delcn = 0.19

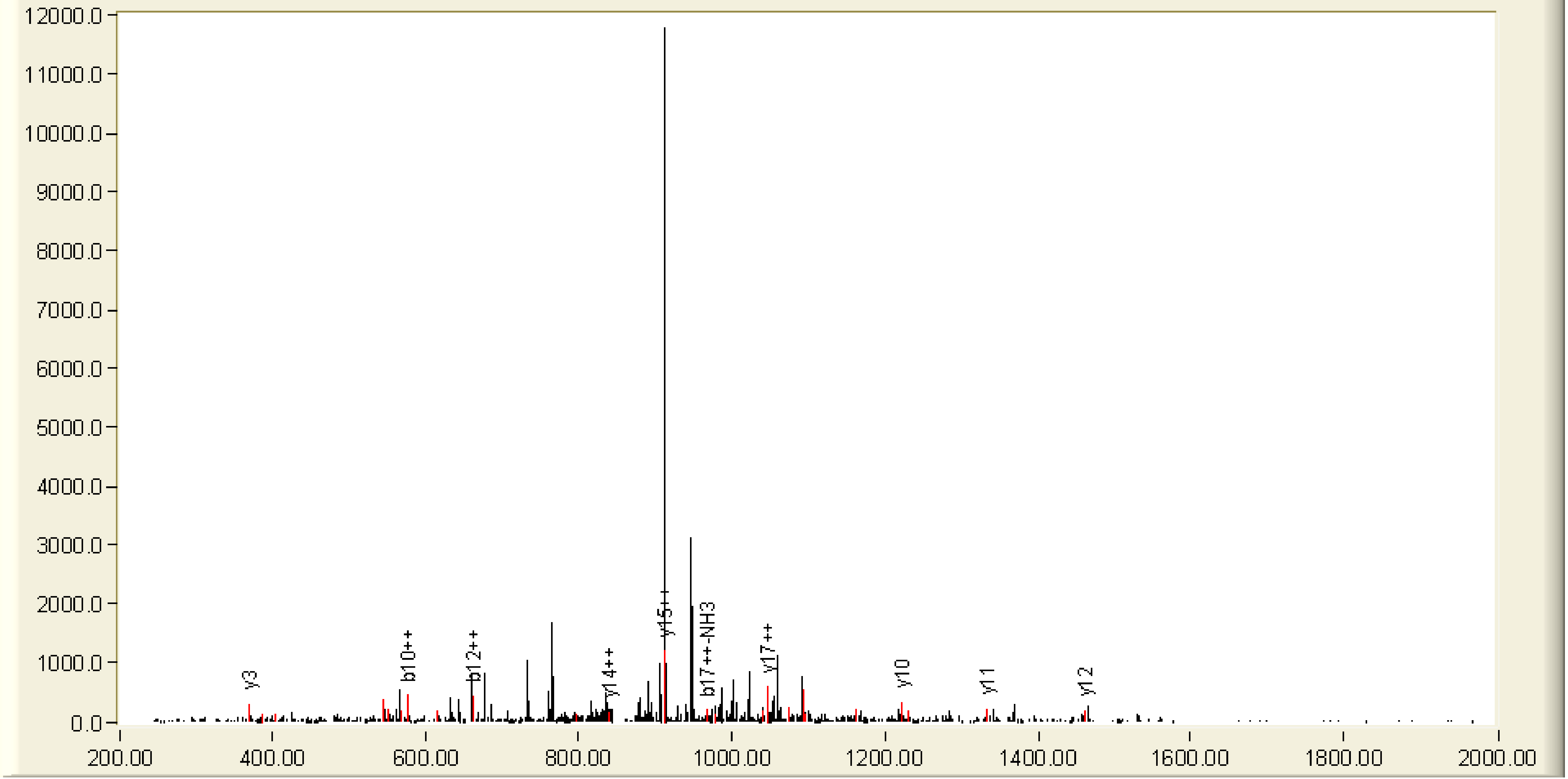

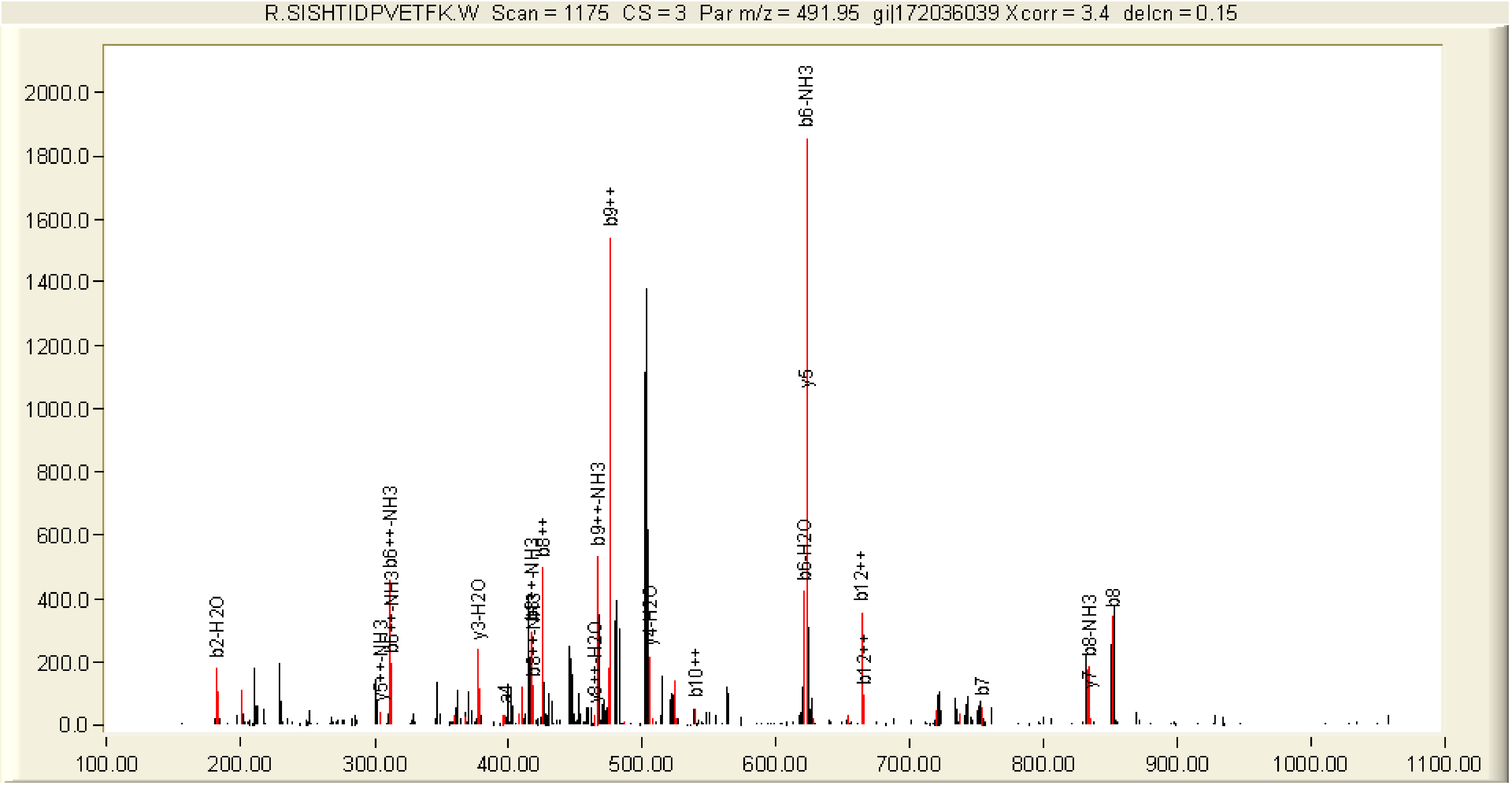
R.SISHTIDPVETFK.W Scan = 1175 CS = 3 Par m/z = 491.95 gi|172036039 Xcorr = 3.4 delcn = 0.15
2000.0
1800.0
1600.0
1400.0
1200.0
1000.0
800.0
600.0
400.0
200.0
0.0
100.00
200.00
300.00
400.00
500.00
600.00
700.00
800.00
900.00
1000.00
1100.00
b6-NH3
b9++
y5
b6-H2O
b9++-NH3
b8++
b6++-NH3
y3-H2O
b2-H2O
b12++
b10++
b8
b8-NH3
b7
y7
a4

K.YRGISYIIGQNKSVVK.Q Scan = 1177 CS = 2 Par m/z = 912.94 gi|172055177 Xcorr = 2.77 delcn = 0.11

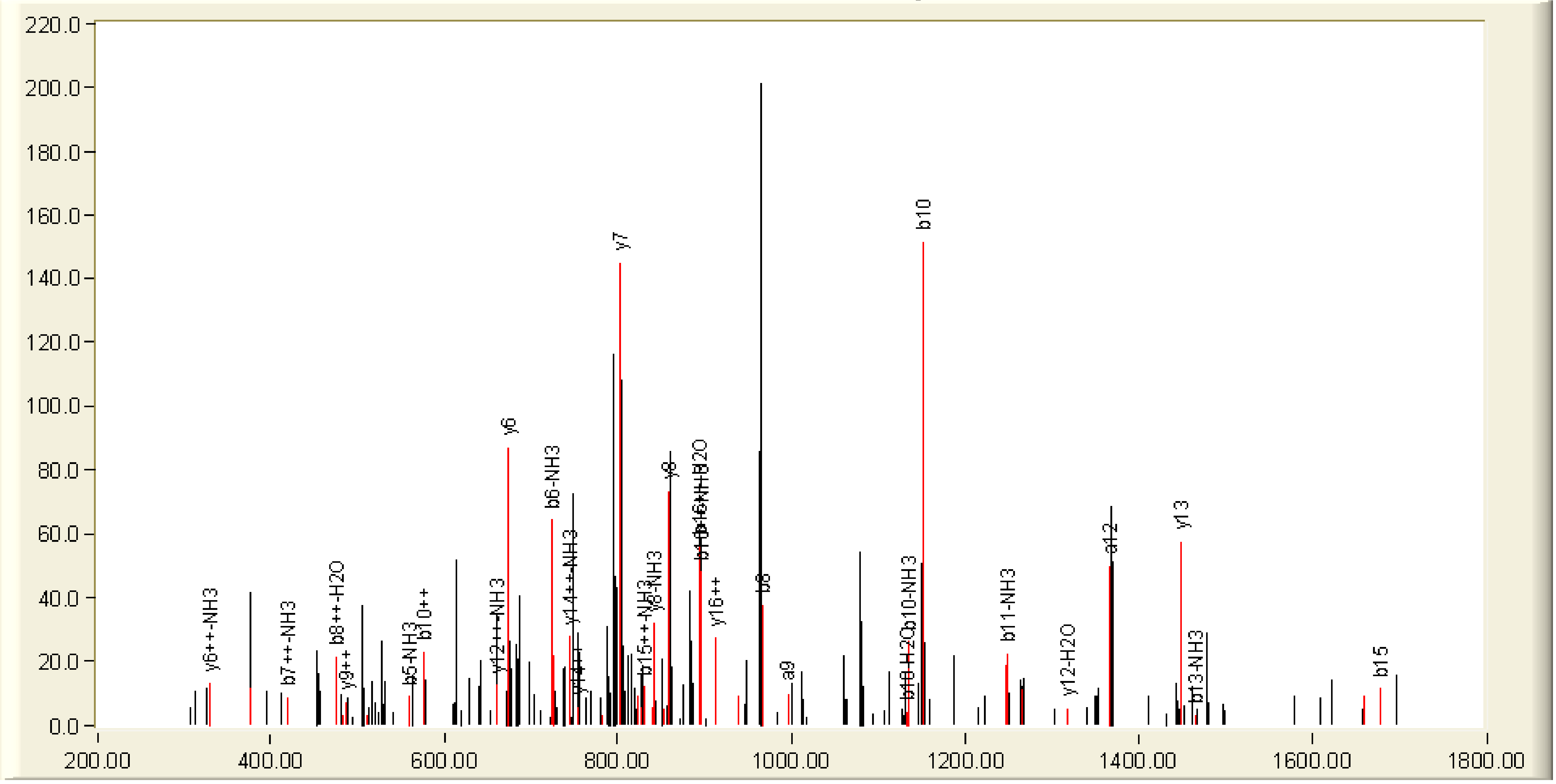

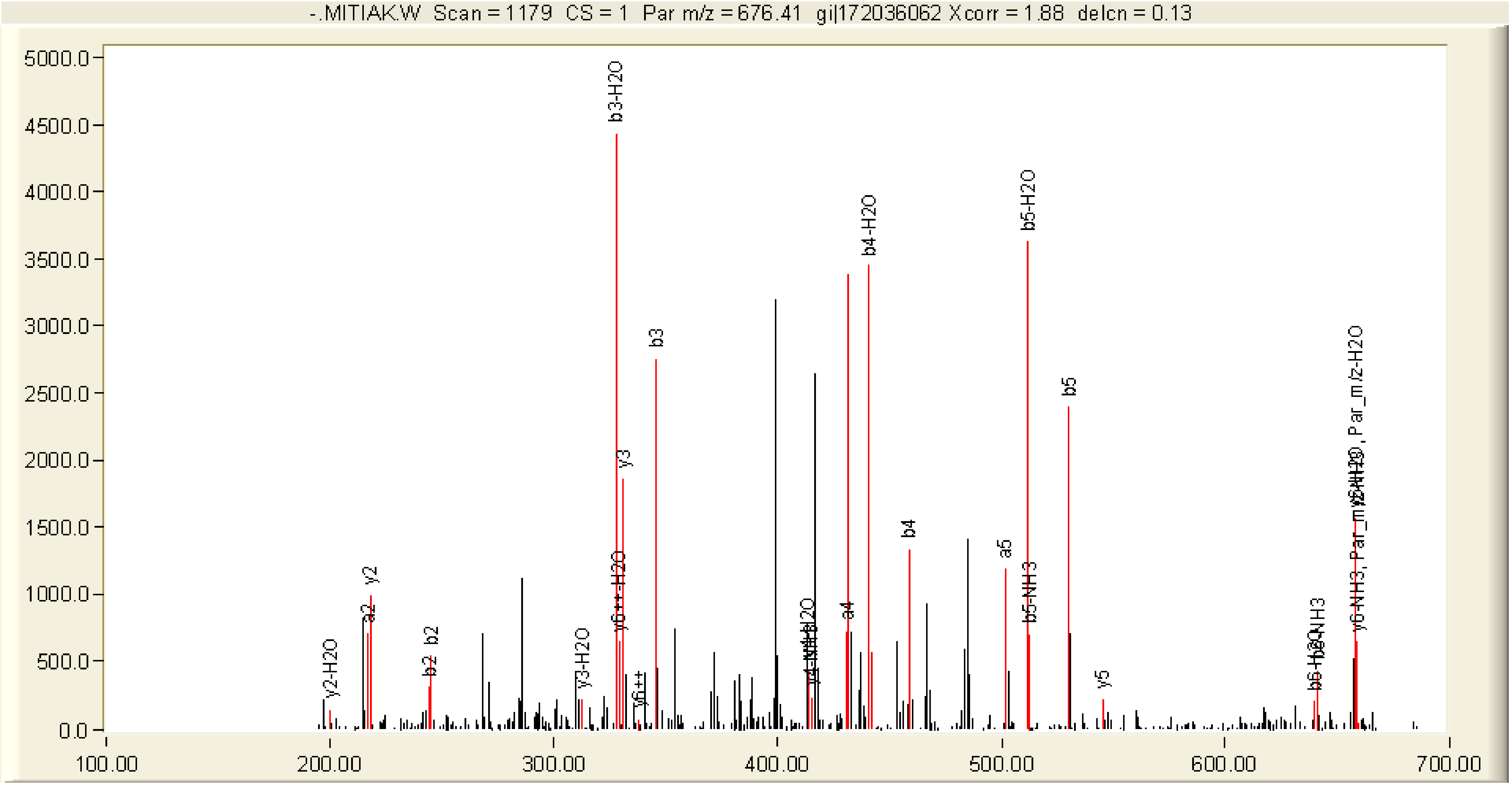

-.MITIAK.W Scan = 1179 CS = 1 Par m/z = 676.41 gi|172036062 Xcorr = 1.88 delcn = 0.13
5000.0
4500.0
4000.0
3500.0
3000.0
2500.0
2000.0
1500.0
1000.0
500.0
0.0
100.00
200.00
300.00
400.00
500.00
600.00
700.00
y2-H2O
a2
y2
b2
b2
y3-H2O
b3-H2O
y3
b3
y6++
a4
b4-H2O
b4
a5
b5-H2O
b5-NH3
b5
y5
y6-NH3, Par_m/z-H2O

W.NDAATIEGLTLRLDYGIPLISLPHFGNNLQEKGLYFQINYSP.- Scan = 1181 CS = 4 Par m/z = 1176.61 gi|172037157 Xcorr = 3.12 delcn = 0.13

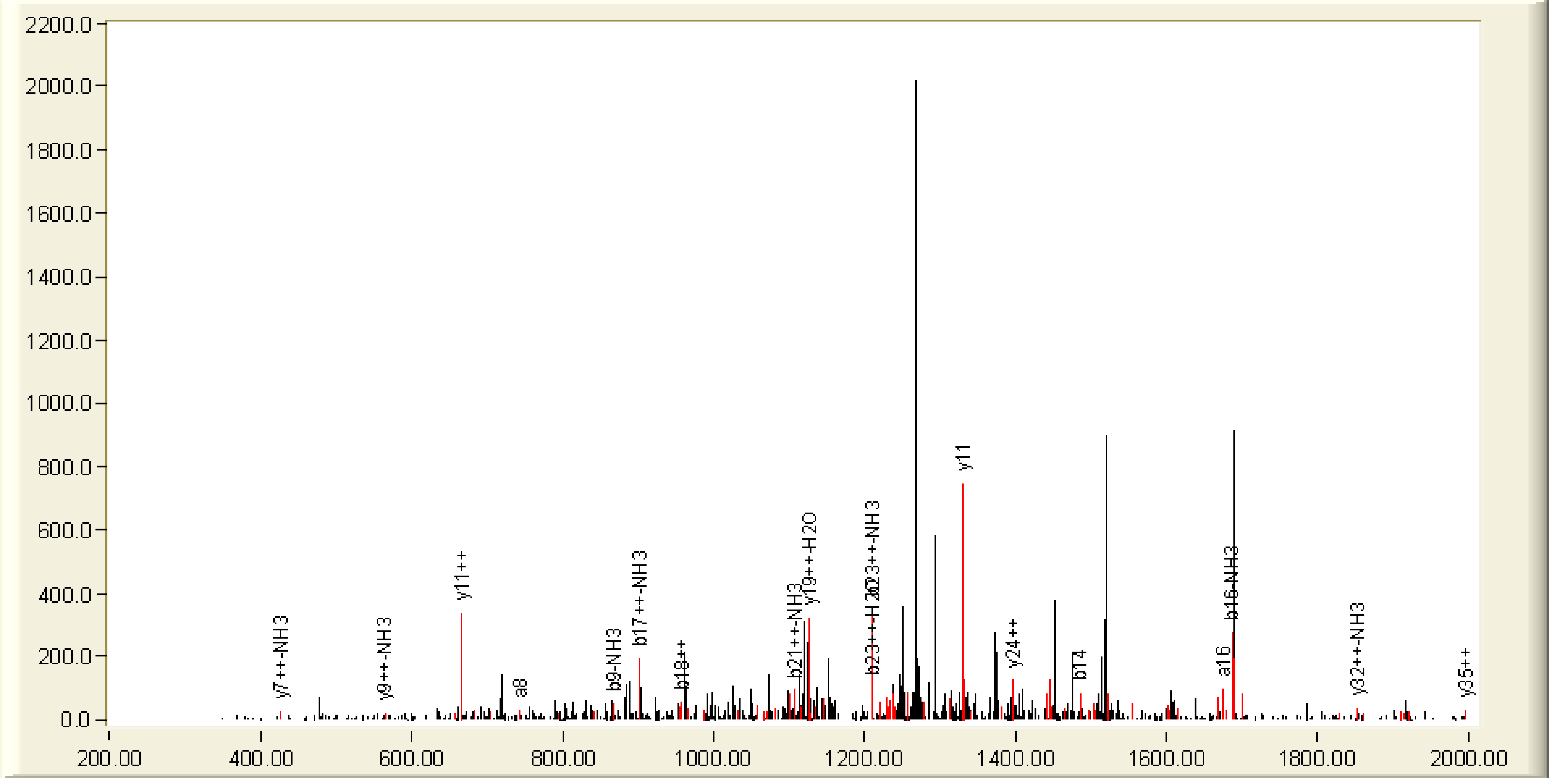

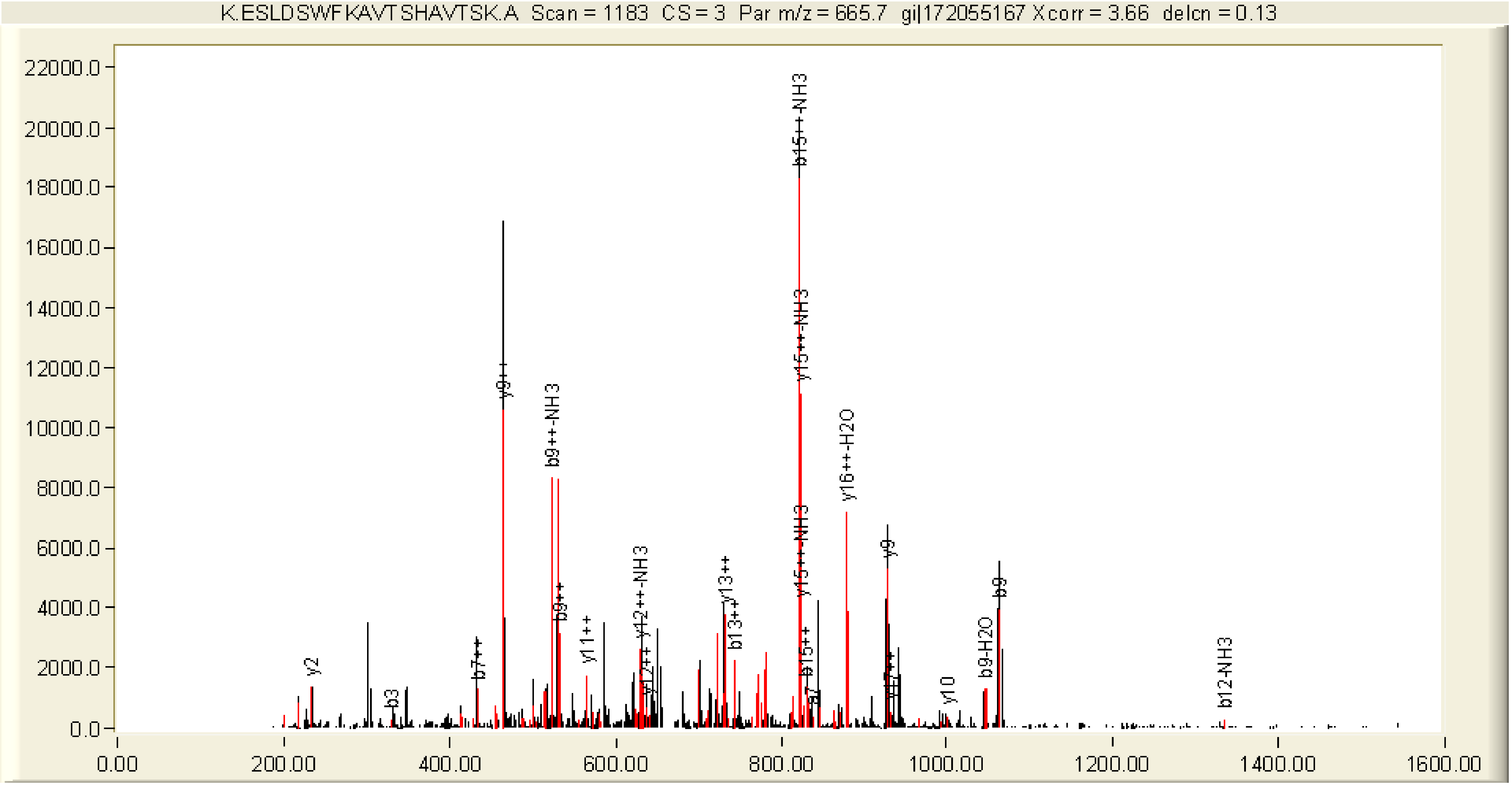
K.ESLDSWFKAVTSHAVTSK.A Scan = 1183 CS = 3 Par m/z = 665.7 gi|172055167 Xcorr = 3.66 delcn = 0.13
22000.0
20000.0
18000.0
16000.0
14000.0
12000.0
10000.0
8000.0
6000.0
4000.0
2000.0
0.0
0.00
200.00
400.00
600.00
800.00
1000.00
1200.00
1400.00
1600.00
y2
b3
b7++
y9++
b9++-NH3
b9++
y11++
y12++-NH3
y12++
y13++
b13++
b15++-NH3
y15++-NH3
y15++-NH3
b15++
y16++-H2O
y9
y10
b9-H2O
b9
b12-NH3

K.KTWLLKQNPLDKD Scan = 1185 CS = 2 Par m/z = 742.36 gi|172039513 Xcorr = 2.76 delcn = 0.11

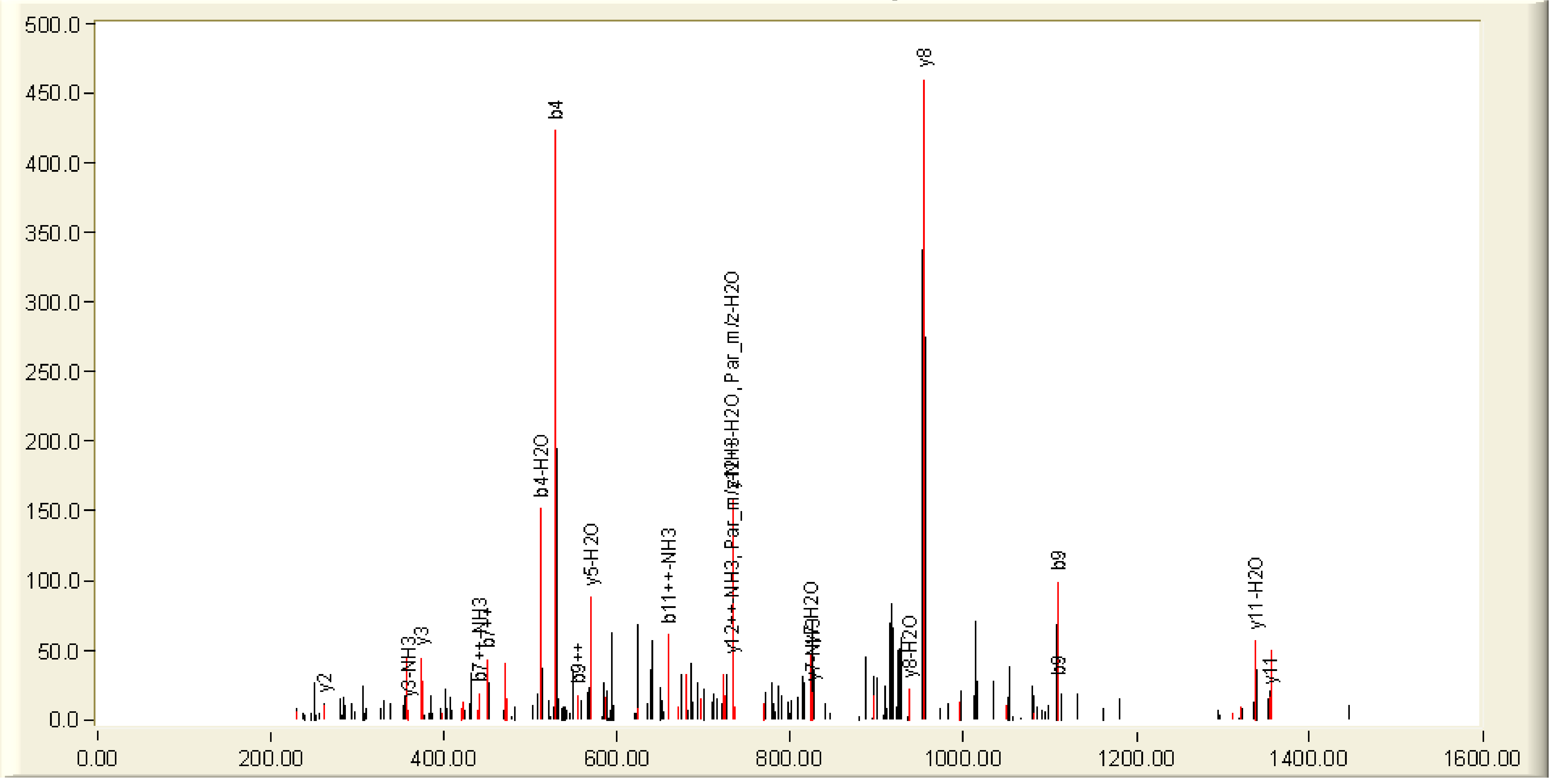

K.SLSLKRIKSLADR.Y Scan = 1187 CS = 2 Par m/z = 745.44 gi|172038167 Xcorr = 2.47 delcn = 0.11

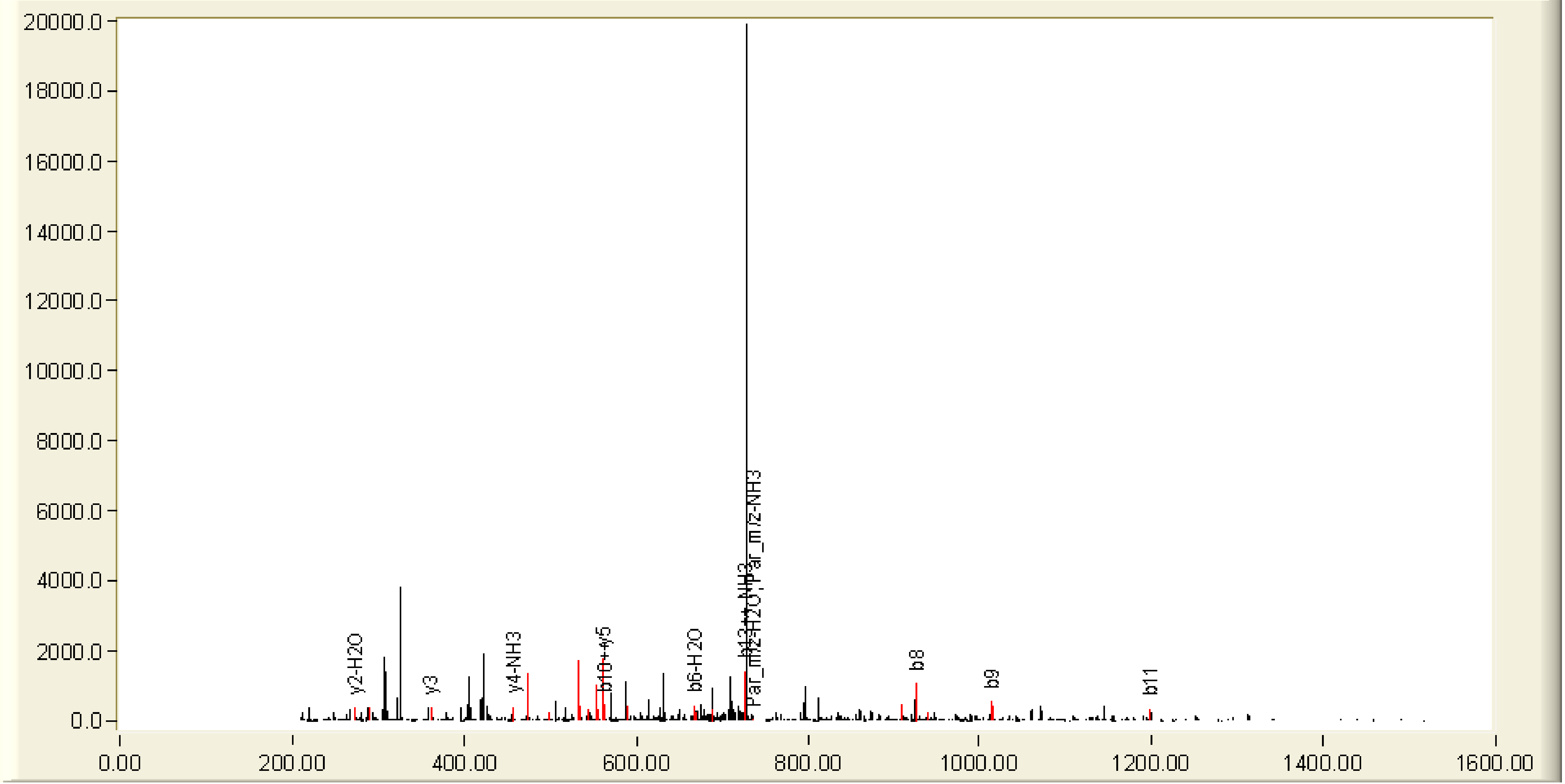

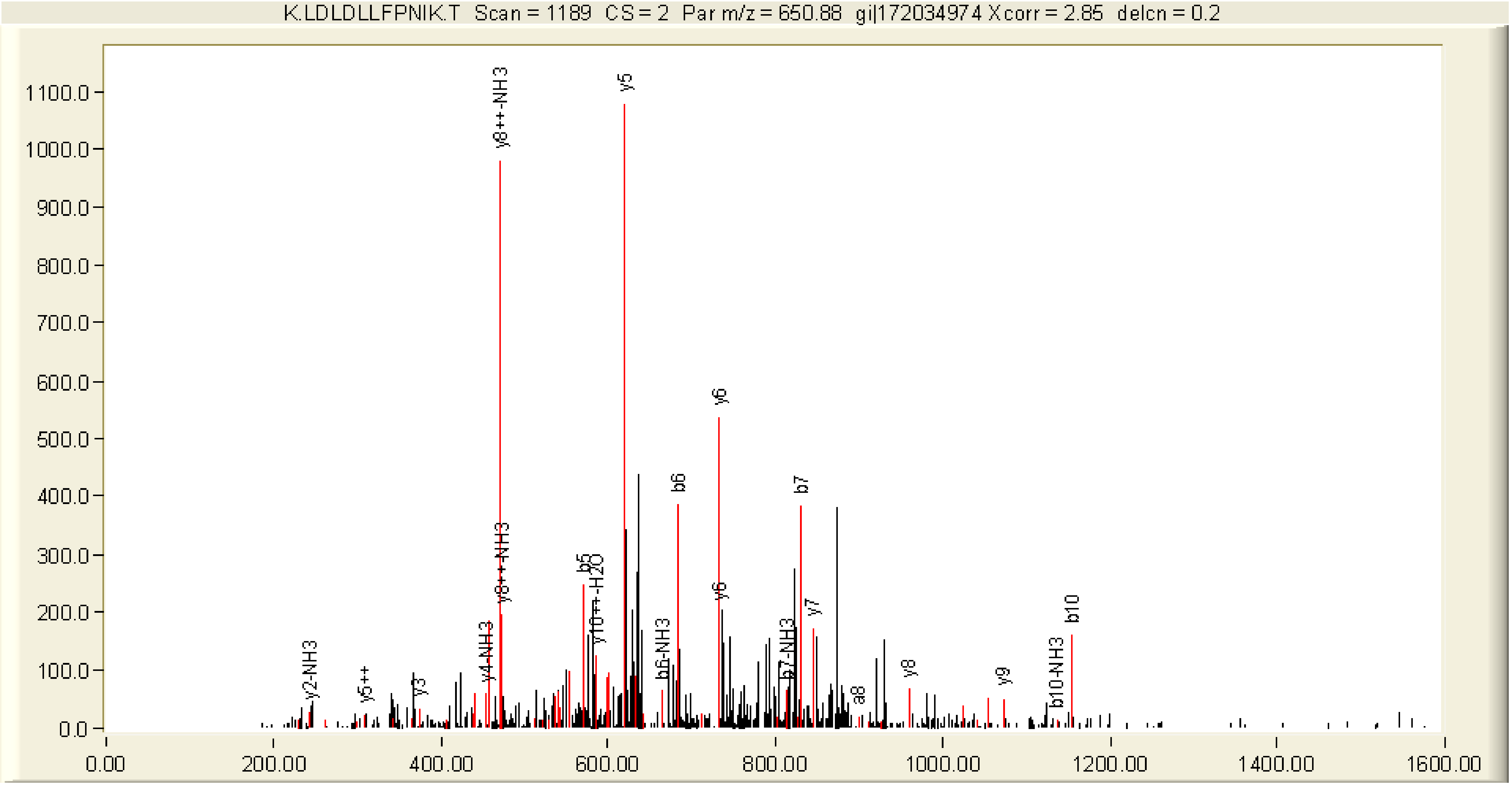

K.LDLDLLFPNIK.T Scan = 1189 CS = 2 Par m/z = 650.88 gi|172034974 Xcorr = 2.85 delcn = 0.2
y8++-NH3
y5
y6
b6
b7
y8++-NH3
b5
y10++-H2O
y6
y7
b10
y2-NH3
y5++
y3
y4-NH3
b6-NH3
b7-NH3
a8
y8
y9
b10-NH3
1100.0
1000.0
900.0
800.0
700.0
600.0
500.0
400.0
300.0
200.0
100.0
0.0
0.00
200.00
400.00
600.00
800.00
1000.00
1200.00
1400.00
1600.00

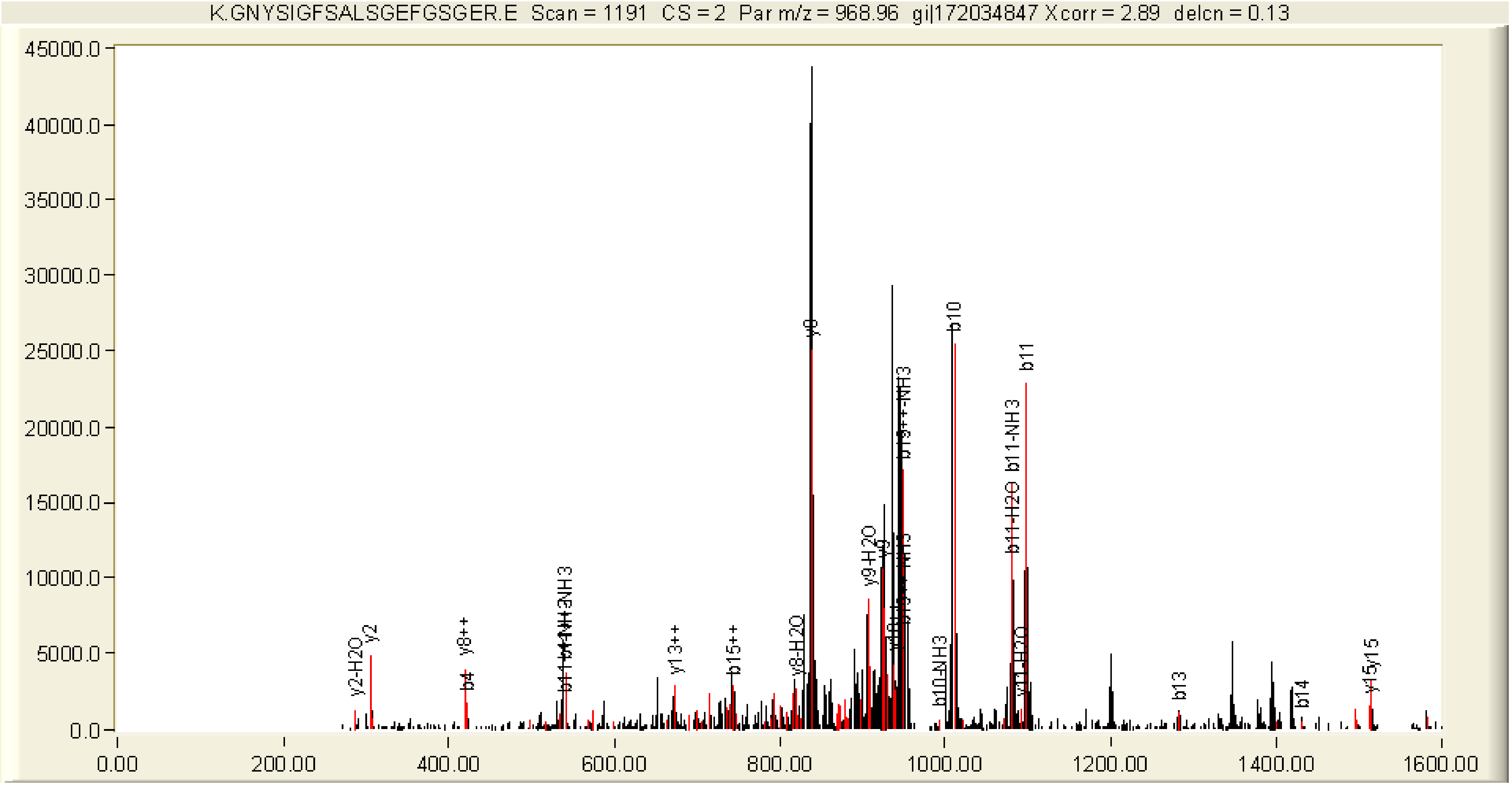
K.GNYSIGFSALSGEFGSGER.E Scan = 1191 CS = 2 Par m/z = 968.96 gi|172034847 Xcorr = 2.89 delcn = 0.13
45000.0
40000.0
35000.0
30000.0
25000.0
20000.0
15000.0
10000.0
5000.0
0.0
0.00
200.00
400.00
600.00
800.00
1000.00
1200.00
1400.00
1600.00
y2-H2O
y2
y8++
b4
y13++
b15++
y8-H2O
y9-H2O
b10
b10-NH3
b11
b11-NH3
b11-H2O
b13
b14
y15
y15

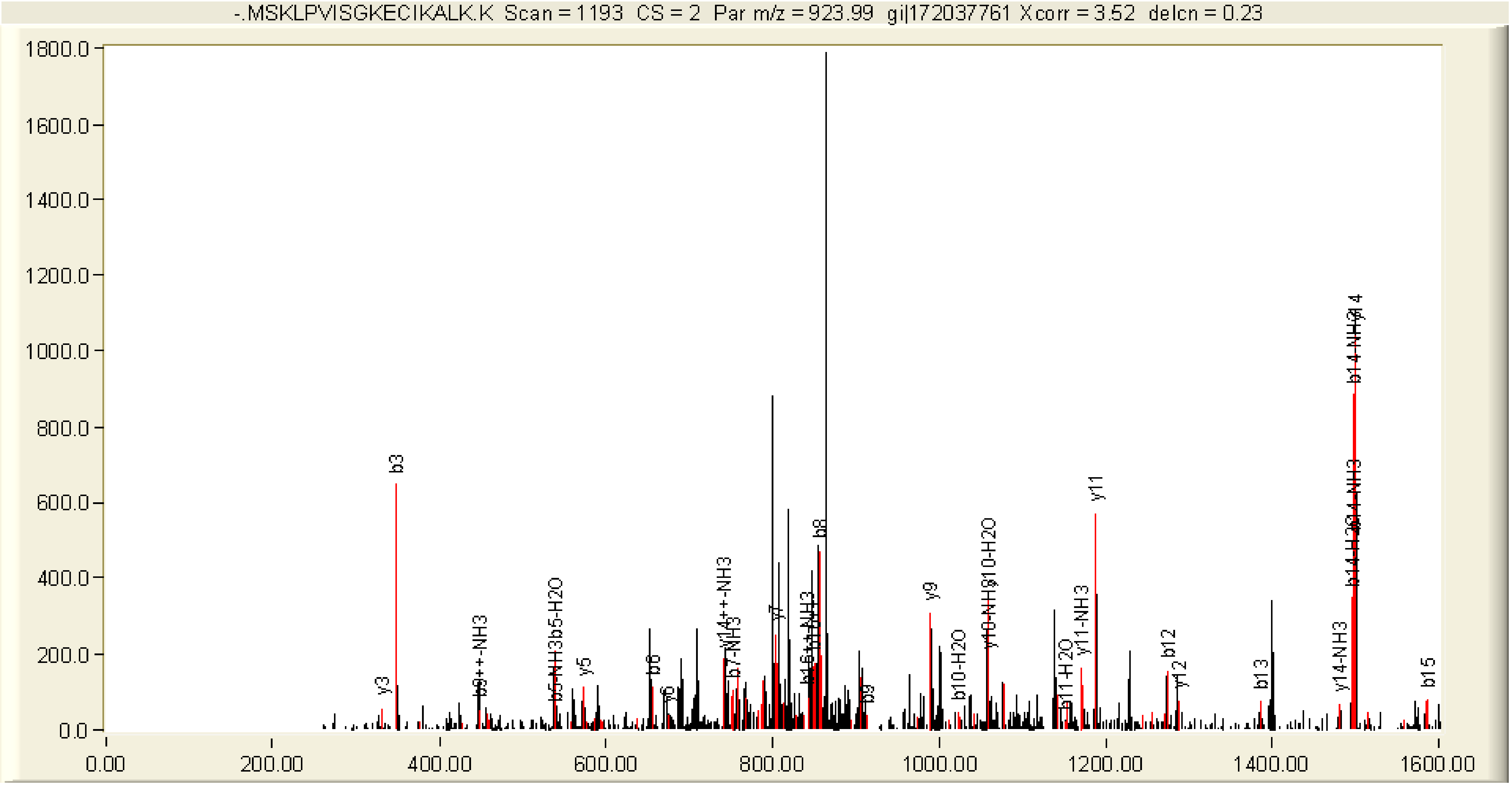
-.MSKLPVISGKECIKALK.K Scan = 1193 CS = 2 Par m/z = 923.99 gi|172037761 Xcorr = 3.52 delcn = 0.23
1800.0
1600.0
1400.0
1200.0
1000.0
800.0
600.0
400.0
200.0
0.0
0.00
200.00
400.00
600.00
800.00
1000.00
1200.00
1400.00
1600.00
b3
y3
y5
b6
y7
b8
b9
y9
b10-H2O
y11
y11-NH3
b12
y12
b13
y14-NH3
b15

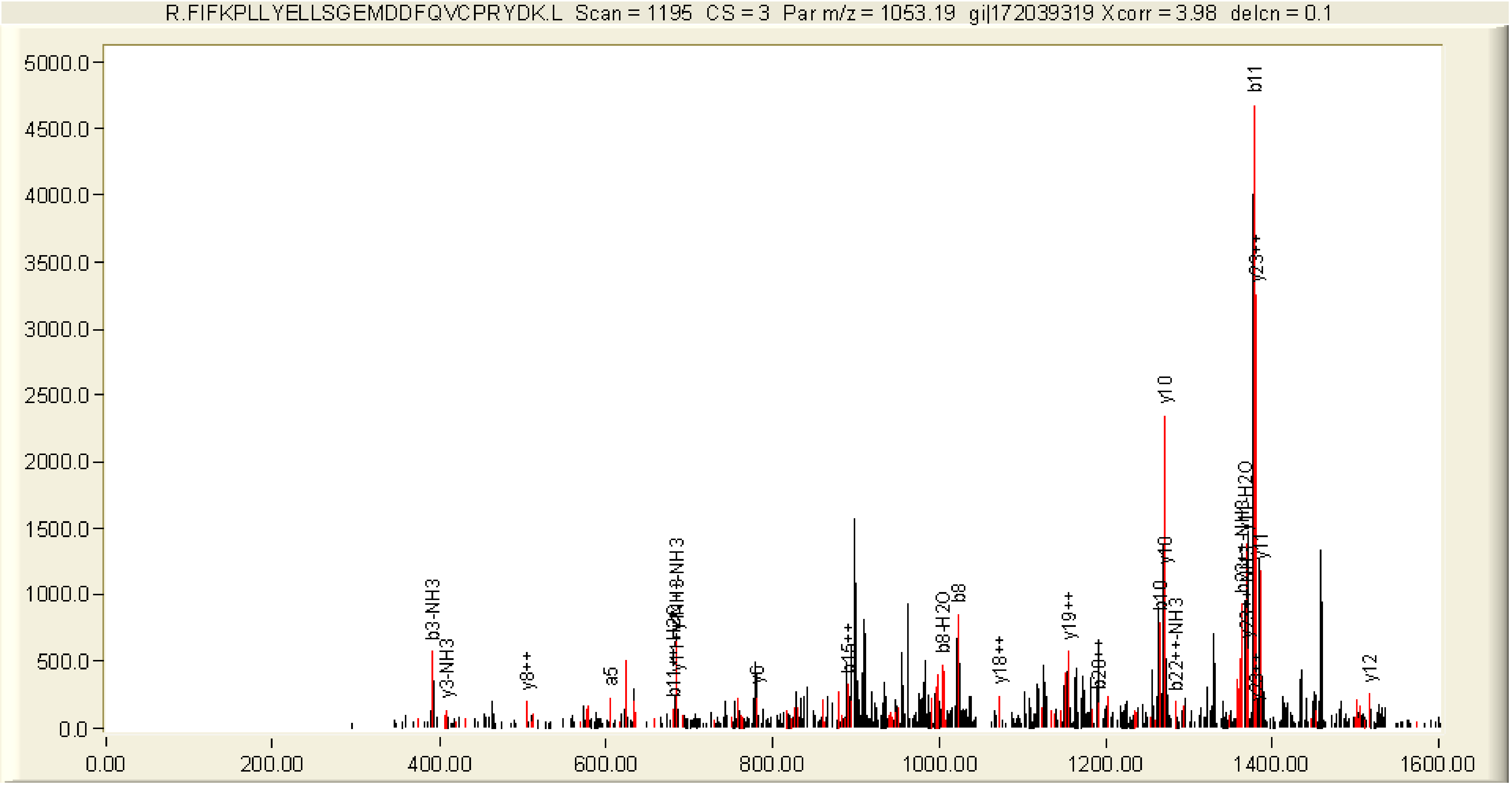
R.FIFKPLLYELLSGEMDDFQVCPRYDK.L Scan = 1195 CS = 3 Par m/z = 1053.19 gi|172039319 Xcorr = 3.98 delcn = 0.1
5000.0
4500.0
4000.0
3500.0
3000.0
2500.0
2000.0
1500.0
1000.0
500.0
0.0
0.00
200.00
400.00
600.00
800.00
1000.00
1200.00
1400.00
1600.00
b3-NH3
y3-NH3
y8++
a5
y6
b15++
b8-H2O
b8
y18++
y19++
b20++
b10
y10
y10
b22++-NH3
b11
y23++
y11
y12

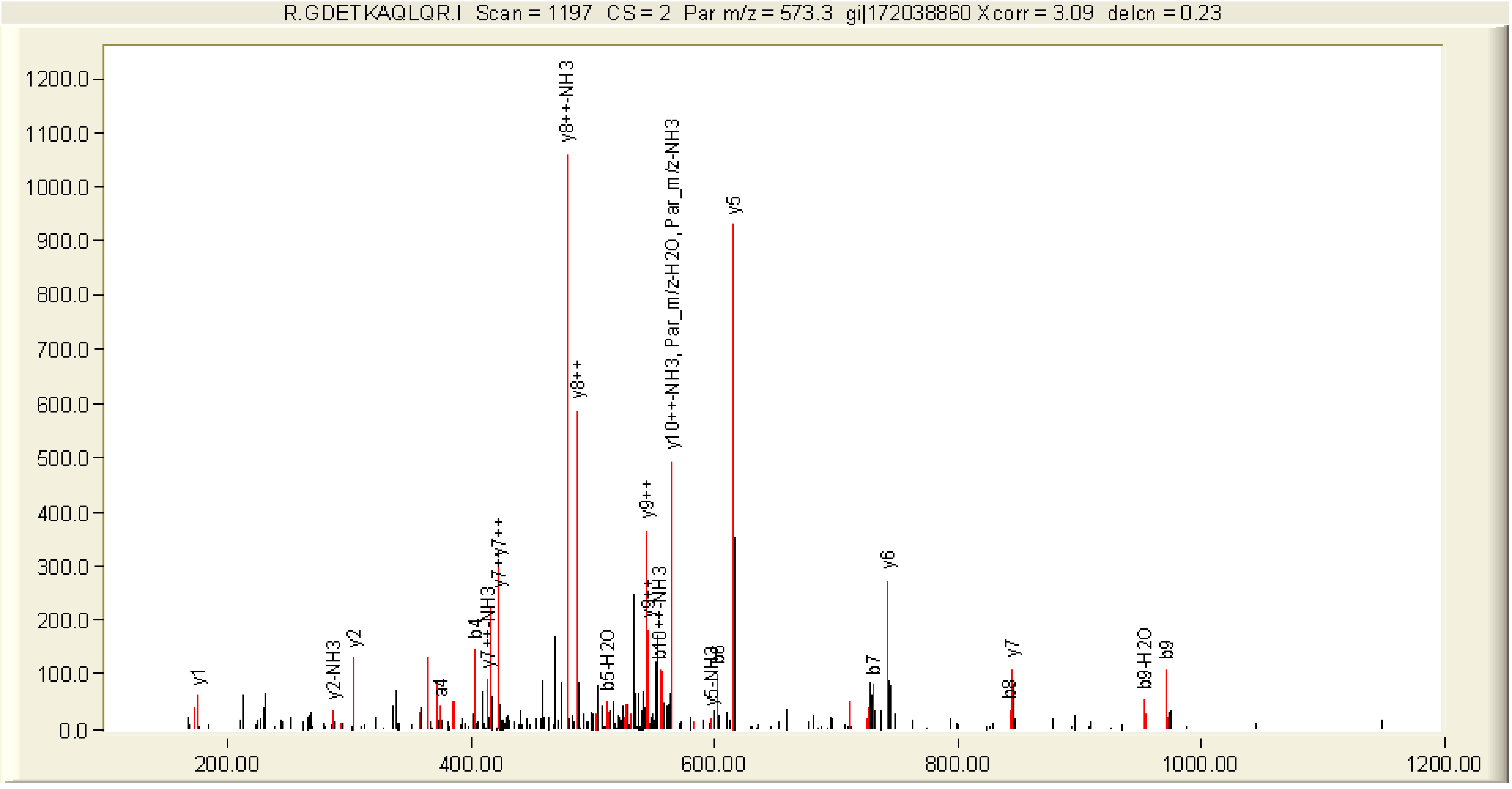
R.GDETKAQLQR.I Scan = 1197 CS = 2 Par m/z = 573.3 gi|172038860 Xcorr = 3.09 delcn = 0.23
y8++-NH3
y10++-NH3, Par_m/z-H2O, Par_m/z-NH3
y5
y8++
y9++
y7++
y6
b5-H2O
y2-NH3
y2
b4
a4
y1
b7
y7
b8
b9-H2O
b9
0.0
100.0
200.0
300.0
400.0
500.0
600.0
700.0
800.0
900.0
1000.0
1100.0
1200.0
200.00
400.00
600.00
800.00
1000.00
1200.00

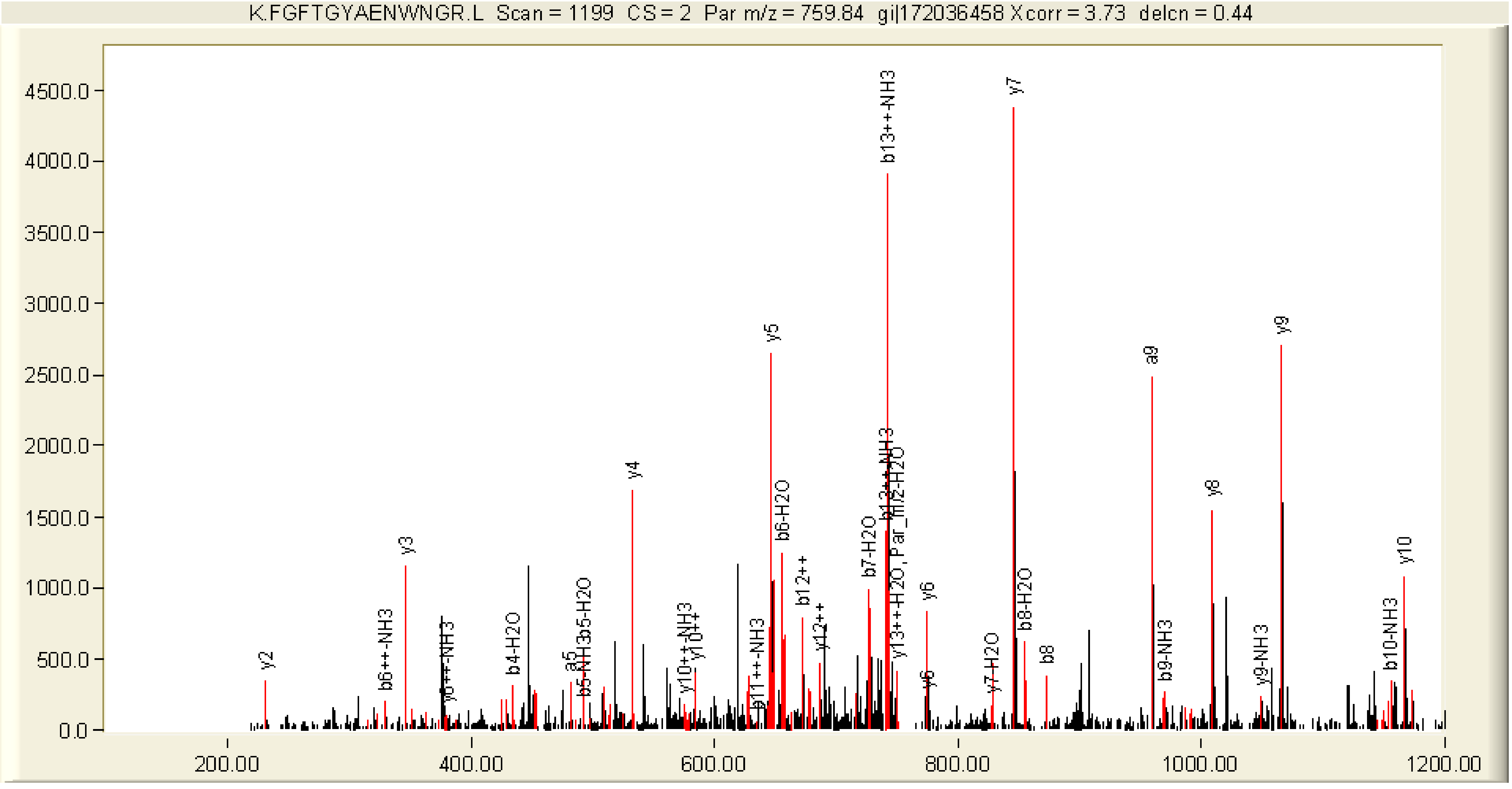

K.FGFTGYAENWNGR.L Scan = 1199 CS = 2 Par m/z = 759.84 gi|172036458 Xcorr = 3.73 delcn = 0.44
4500.0
4000.0
3500.0
3000.0
2500.0
2000.0
1500.0
1000.0
500.0
0.0
200.00
400.00
600.00
800.00
1000.00
1200.00
y2
b6++-NH3
y3
b4-H2O
b5-H2O
y4
y5
b6-H2O
b12++
b7-H2O
b13++-NH3
y6
y7
b8-H2O
b8
a9
b9-NH3
y8
y9-NH3
y9
b10-NH3
y10

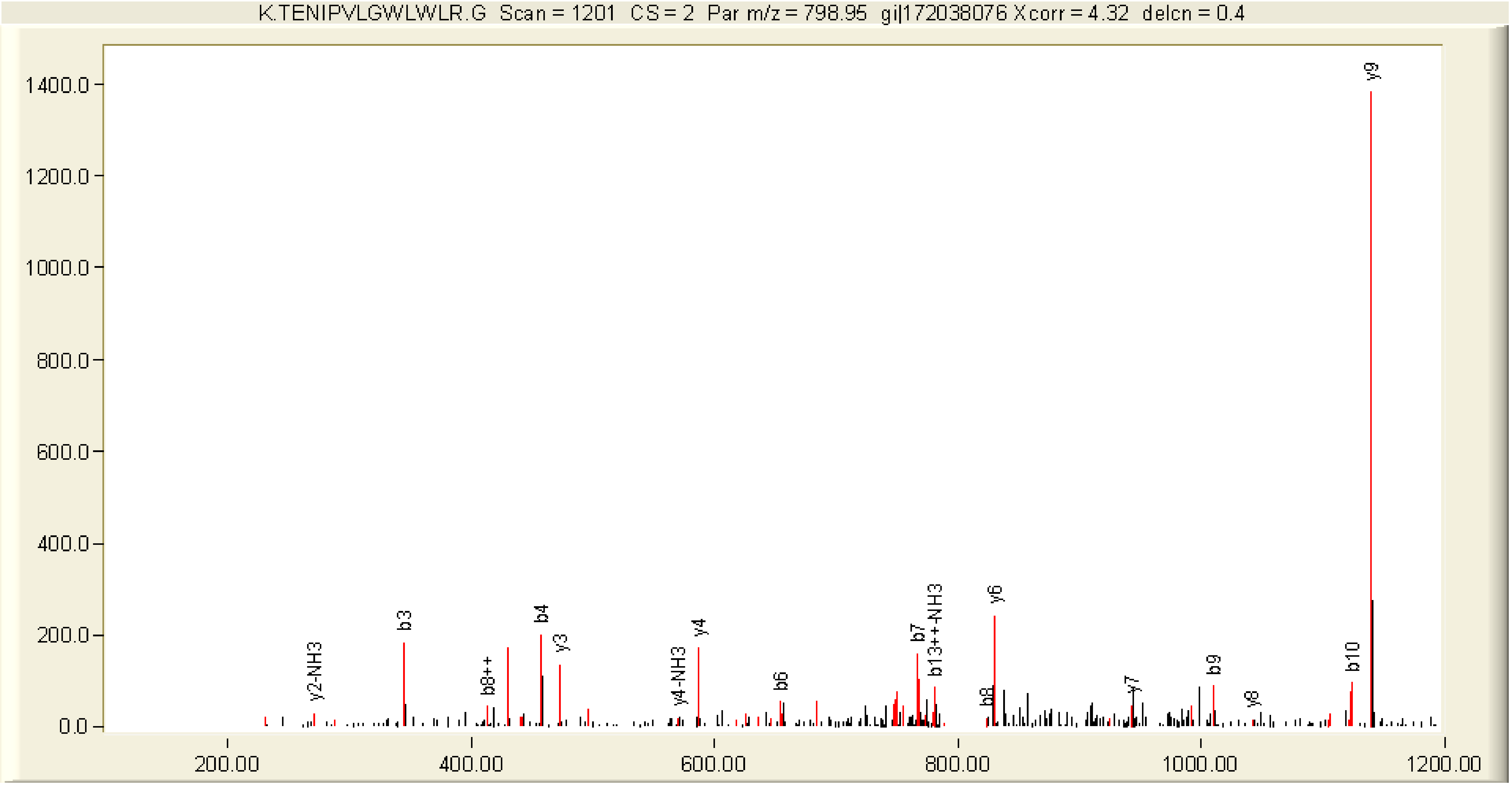

K.TENIPVLGWLWLR.G Scan = 1201 CS = 2 Par m/z = 798.95 gi|172038076 Xcorr = 4.32 delcn = 0.4
1400.0
1200.0
1000.0
800.0
600.0
400.0
200.0
0.0
200.00
400.00
600.00
800.00
1000.00
1200.00
y9
y2-NH3
b3
b8++
b4
y3
y4-NH3
y4
b6
b7
b13++-NH3
b8
y6
y7
b9
y8
b10

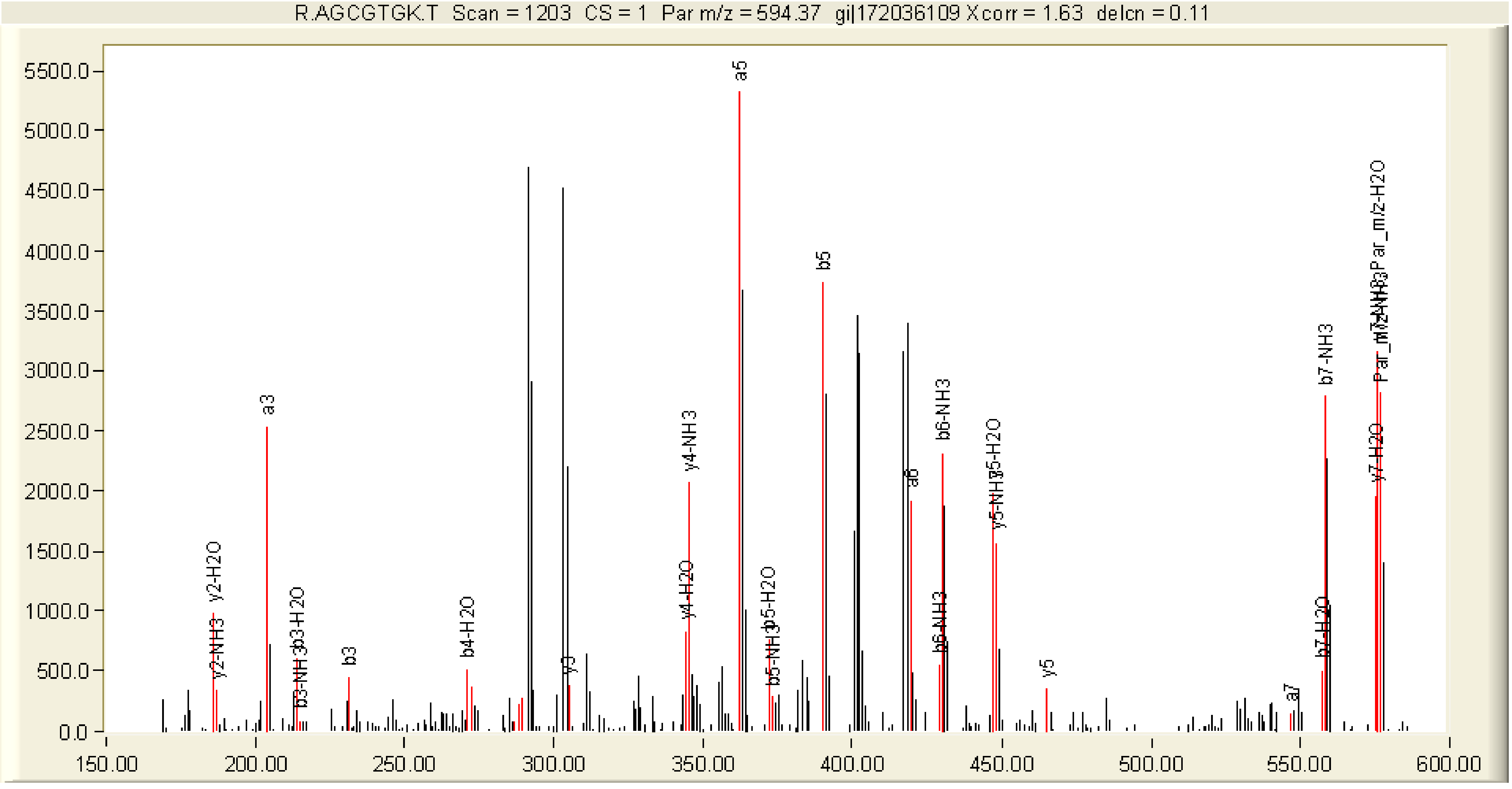
R.AGCGTGK.T Scan = 1203 CS = 1 Par m/z = 594.37 gi|172036109 Xcorr = 1.63 delcn = 0.11
a5
b5
a3
b7-NH3
Par_m/z-H2O
y4-NH3
b6-NH3
y5-H2O
a6
y7-H2O
y2-H2O
y4-H2O
b5-H2O
b3-H2O
b4-H2O
y2-NH3
b3-NH3
b5-NH3
b6-NH3
b7-H2O
b3
y3
y5
a7
0.0
500.0
1000.0
1500.0
2000.0
2500.0
3000.0
3500.0
4000.0
4500.0
5000.0
5500.0
150.00
200.00
250.00
300.00
350.00
400.00
450.00
500.00
550.00
600.00

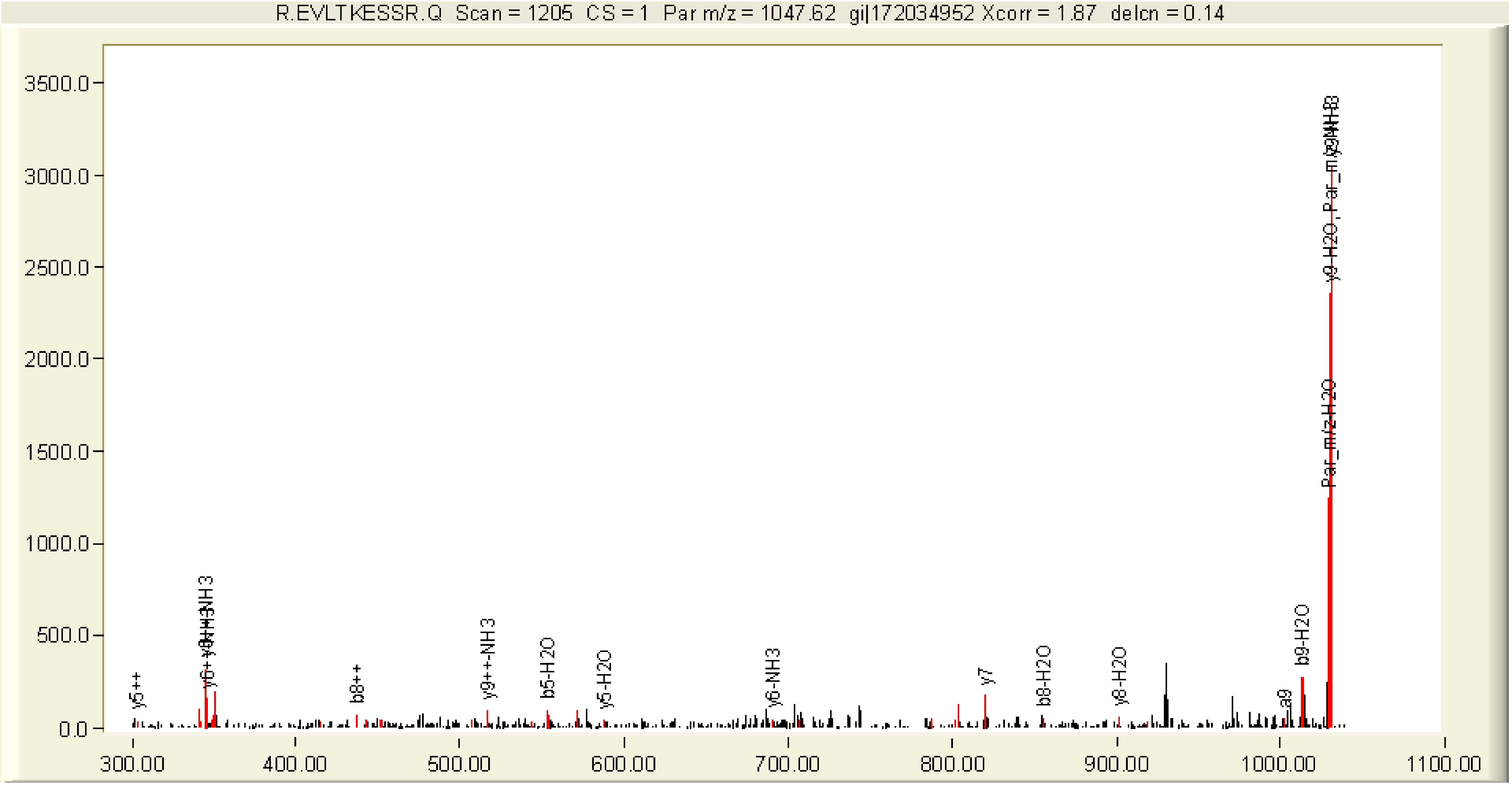
R.EVLTKESSR.Q Scan = 1205 CS = 1 Par m/z = 1047.62 gi|172034952 Xcorr = 1.87 delcn = 0.14
3500.0
3000.0
2500.0
2000.0
1500.0
1000.0
500.0
0.0
300.00
400.00
500.00
600.00
700.00
800.00
900.00
1000.00
1100.00
y5++
b8++
y9++-NH3
b5-H2O
y5-H2O
y6-NH3
y7
b8-H2O
y8-H2O
a9
b9-H2O
Par_m/z-H2O

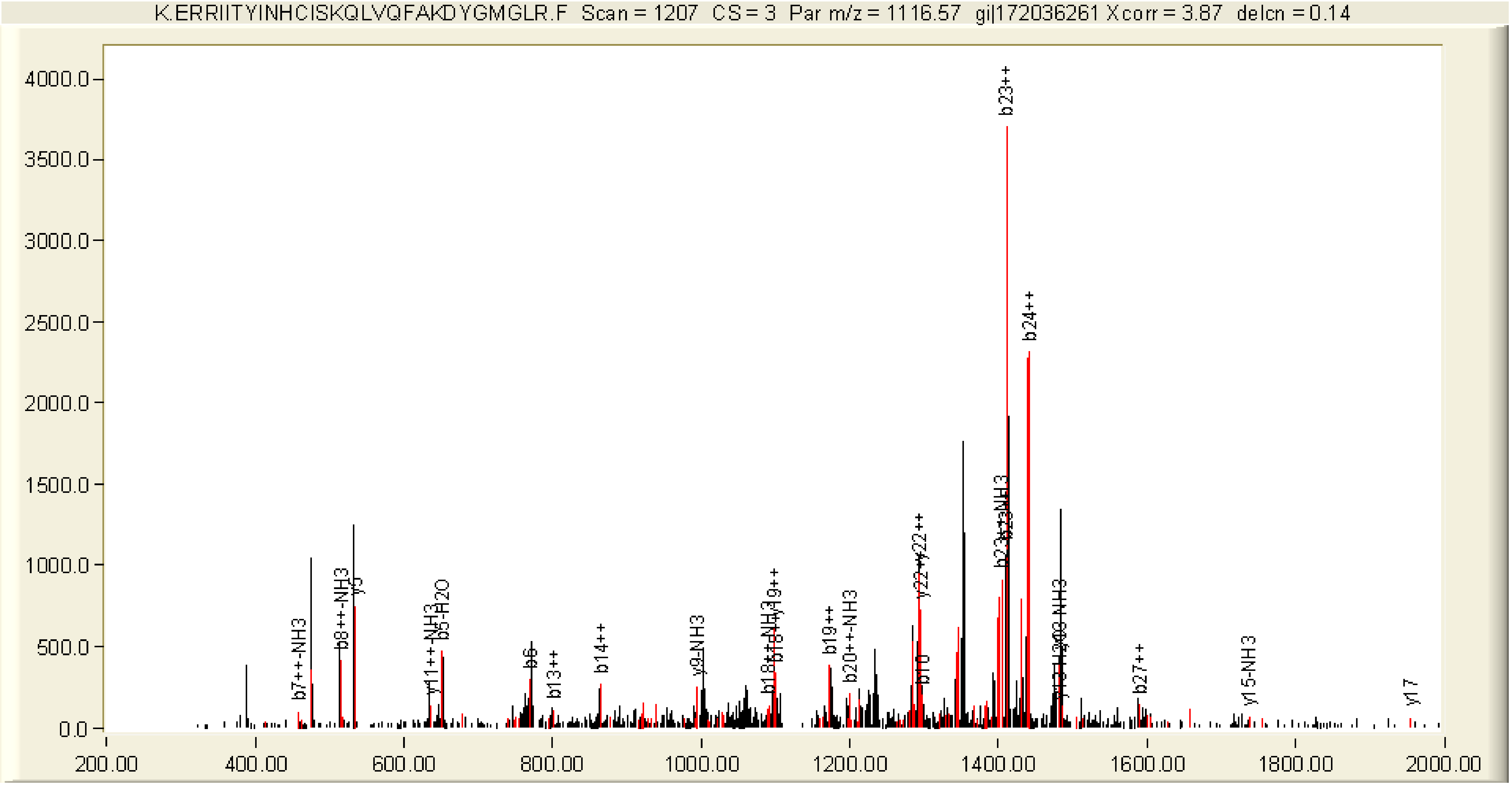
K.ERRIITYINHCISKQLVQFAKDYGMGLR.F Scan = 1207 CS = 3 Par m/z = 1116.57 gi|172036261 Xcorr = 3.87 delcn = 0.14
4000.0
3500.0
3000.0
2500.0
2000.0
1500.0
1000.0
500.0
0.0
200.00
400.00
600.00
800.00
1000.00
1200.00
1400.00
1600.00
1800.00
2000.00
b23++
b24++
b7++-NH3
b8++-NH3
y5
y11++-NH3
b5-H2O
b6
b13++
b14++
y9-NH3
b19++
b20++-NH3
b10
b27++
y15-NH3
y17

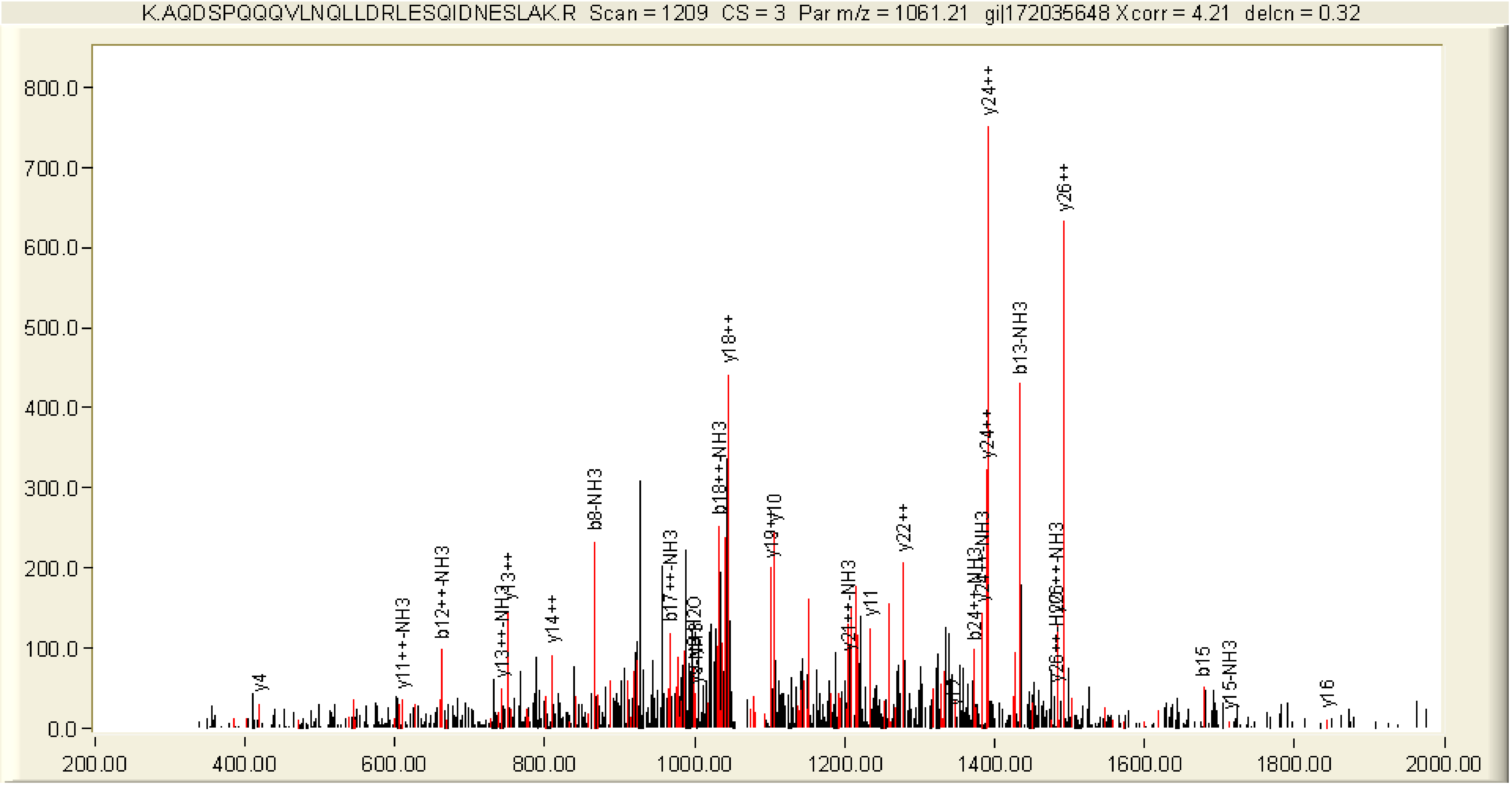

K.AQDSPQQQVLNQLLDRLESQIDNESLAK.R Scan = 1209 CS = 3 Par m/z = 1061.21 gi|172035648 Xcorr = 4.21 delcn = 0.32
800.0
700.0
600.0
500.0
400.0
300.0
200.0
100.0
0.0
200.00
400.00
600.00
800.00
1000.00
1200.00
1400.00
1600.00
1800.00
2000.00
y4
y11++-NH3
b12++-NH3
y13++-NH3
y13++
y14++
b8-NH3
b17++-NH3
b18++-NH3
y18++
y19+
y10
y21++-NH3
y11
y22++
b24++-NH3
y24++
b13-NH3
y26++-NH3
y26++
b15
y15-NH3
y16

-.MKLSQLSDNELMEAYKILIKRINLYKEERISSQNW.Q Scan = 1211 CS = 4 Par m/z = 1071.99 gi|172035845 Xcorr = 4.09 delcn = 0.15

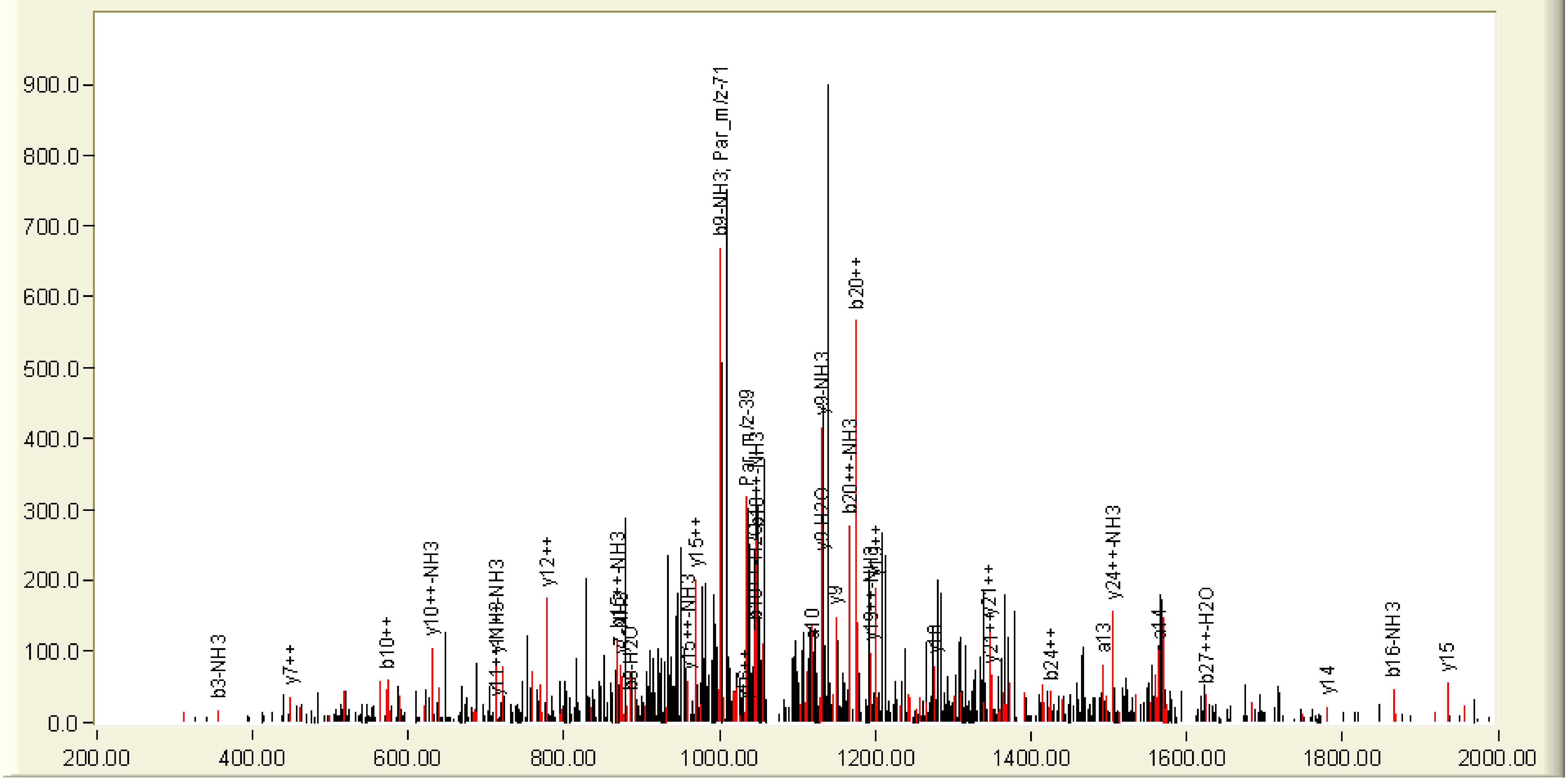

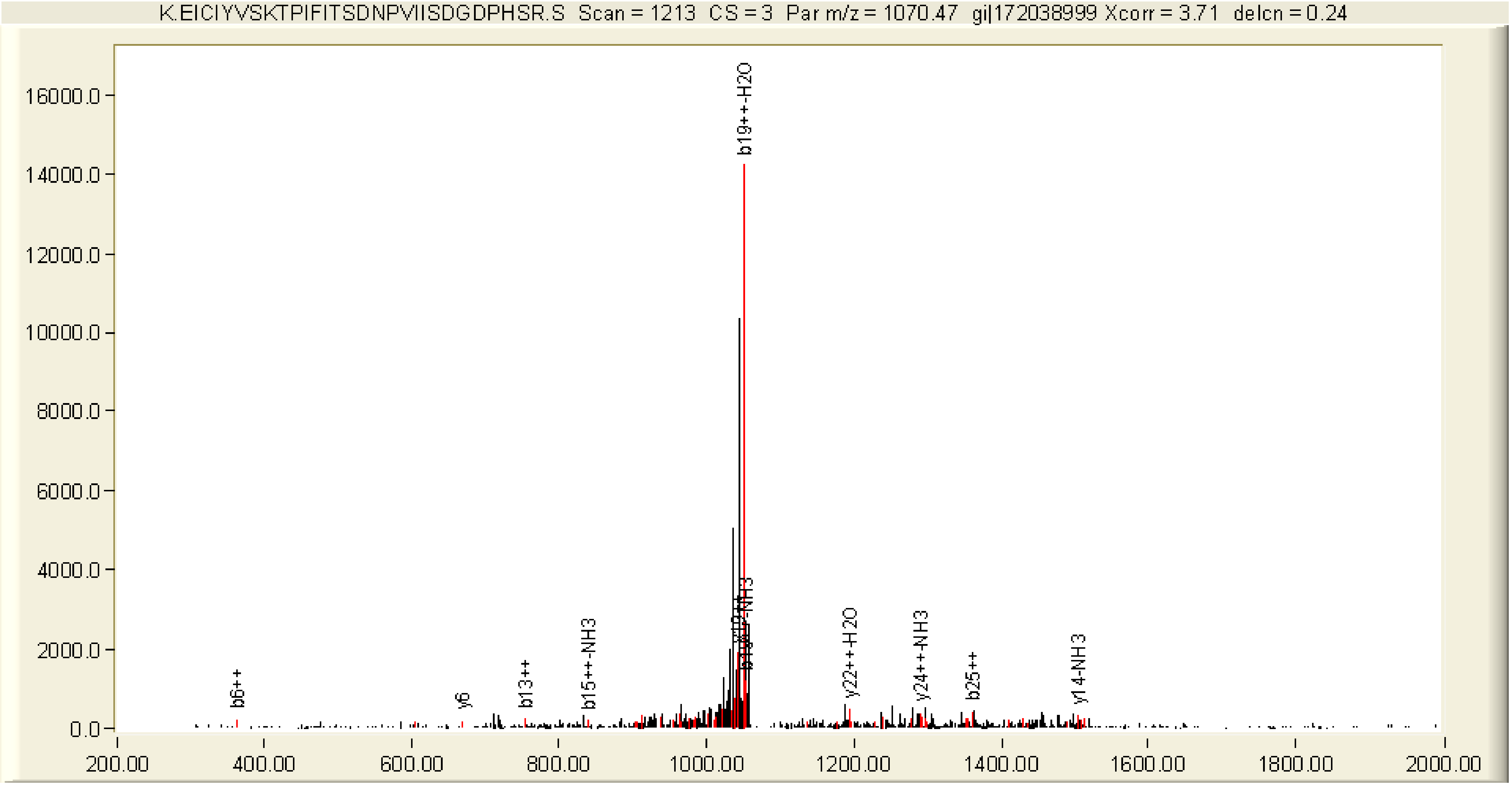
K.EICIYVSKTPIFITSDNPVIISDGDPHSR.S Scan = 1213 CS = 3 Par m/z = 1070.47 gi|172038999 Xcorr = 3.71 delcn = 0.24
16000.0
14000.0
12000.0
10000.0
8000.0
6000.0
4000.0
2000.0
0.0
200.00
400.00
600.00
800.00
1000.00
1200.00
1400.00
1600.00
1800.00
2000.00
b19++-H2O
b6++
y6
b13++
b15++-NH3
y22++-H2O
y24++-NH3
b25++
y14-NH3

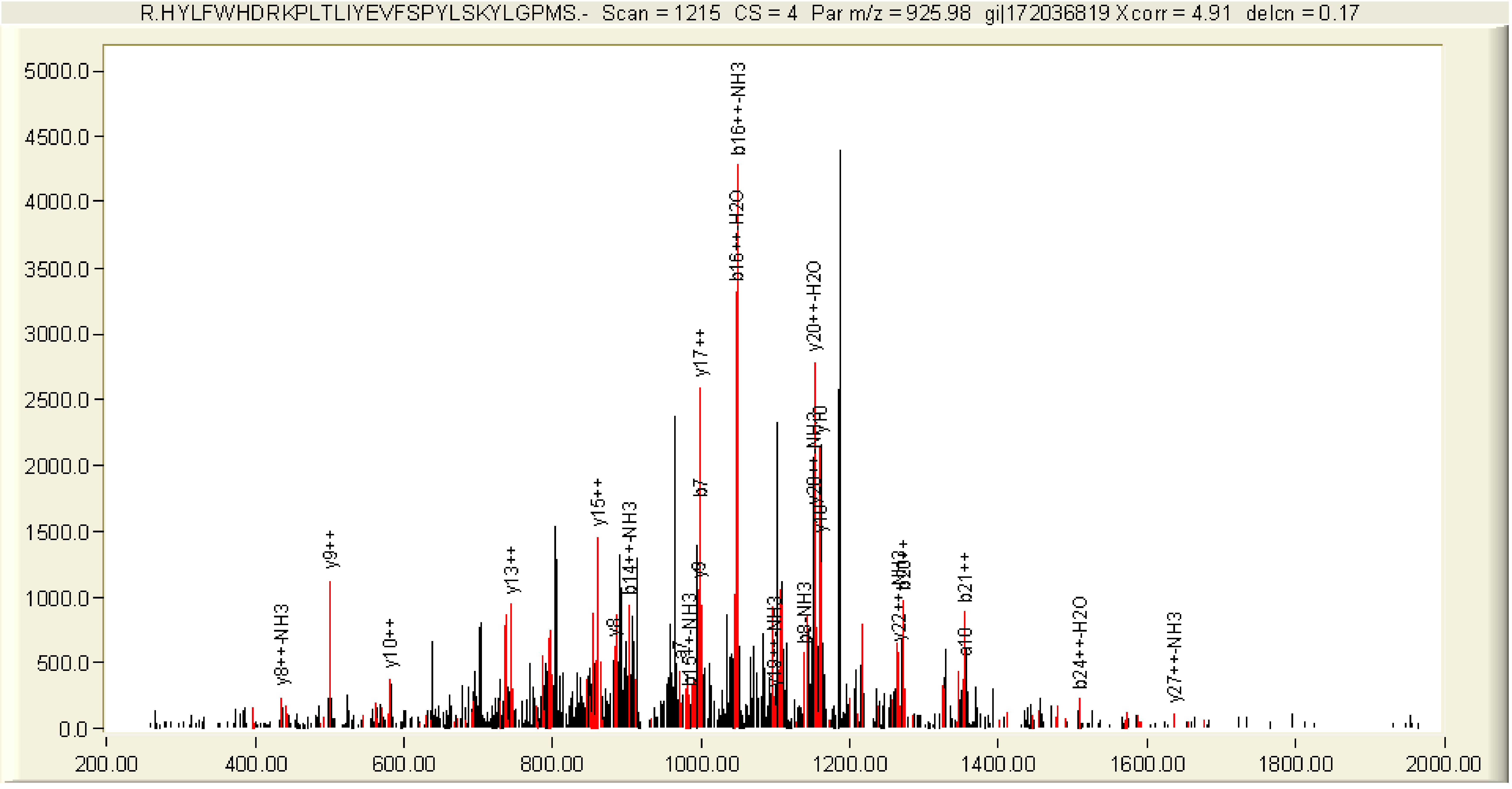
R.HYLFWHDRKPLTLIYEVFSPYLSKYLGPMS.- Scan = 1215 CS = 4 Par m/z = 925.98 gi|172036819 Xcorr = 4.91 delcn = 0.17
5000.0
4500.0
4000.0
3500.0
3000.0
2500.0
2000.0
1500.0
1000.0
500.0
0.0
200.00
400.00
600.00
800.00
1000.00
1200.00
1400.00
1600.00
1800.00
2000.00
y8++-NH3
y9++
y10++
y13++
y15++
b14++-NH3
y8
y17++
b7
y9
b16++-NH3
b16++-H2O
y20++-H2O
b8-NH3
y22++
b21++
a10
b24++-H2O
y27++-NH3

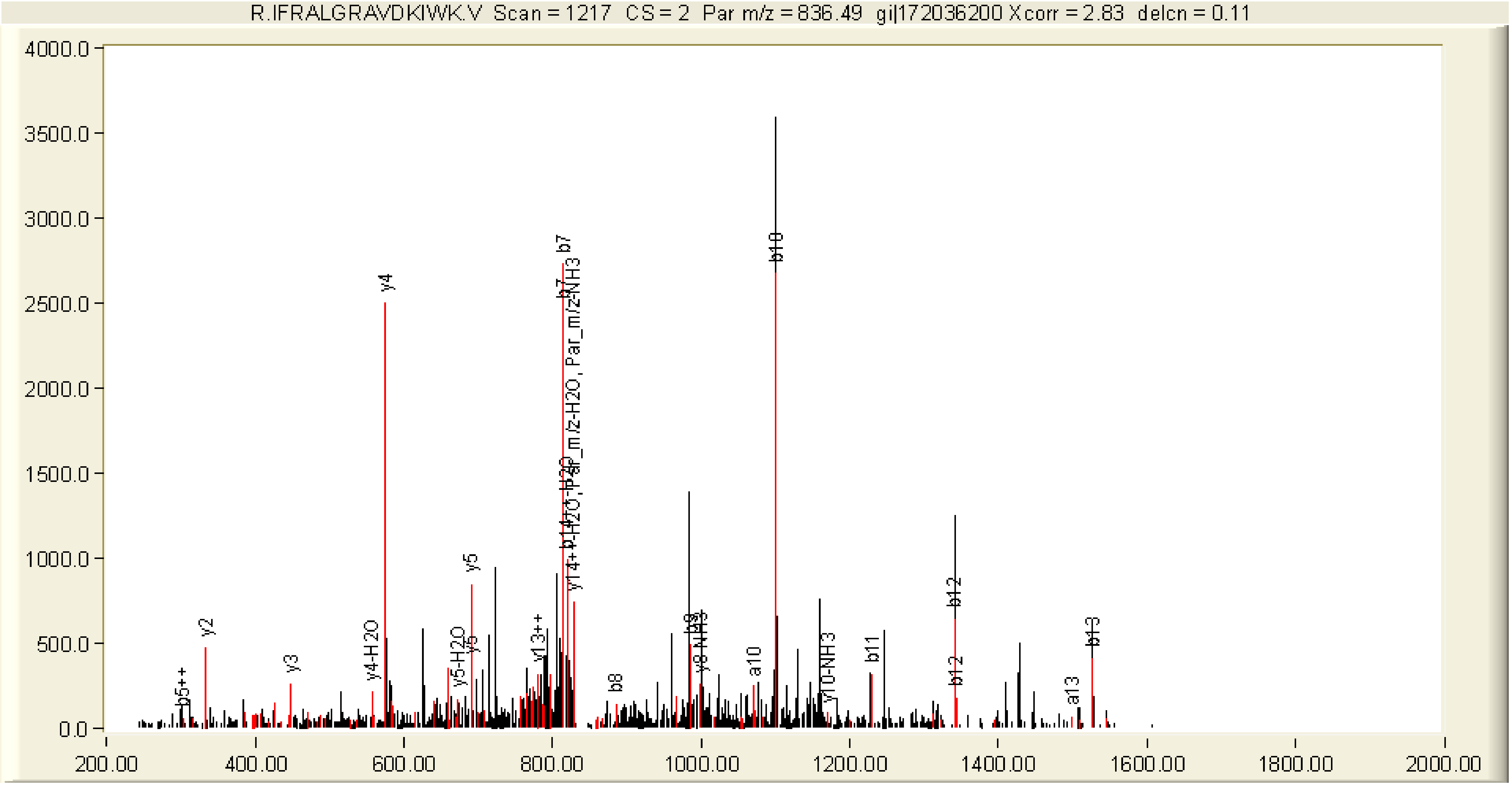
R.IFRALGRAVDKIWK.V Scan = 1217 CS = 2 Par m/z = 836.49 gi|172036200 Xcorr = 2.83 delcn = 0.11
4000.0
3500.0
3000.0
2500.0
2000.0
1500.0
1000.0
500.0
0.0
200.00
400.00
600.00
800.00
1000.00
1200.00
1400.00
1600.00
1800.00
2000.00
b5++
y2
y3
y4-H2O
y4
y5-H2O
y5
y5
y13++
b7
b7
Par_m/z-NH3
Par_m/z-H2O, Par_m/z-NH3
b8
y8
y8-NH3
a10
b10
y10-NH3
b11
b12
b12
a13
b13

L.ENLVRSLVGAFIGVRVISGLRAIGIMKPEAAIY.- Scan = 1219 CS = 4 Par m/z = 878.97 gi|172036444 Xcorr = 3.07 delcn = 0.11

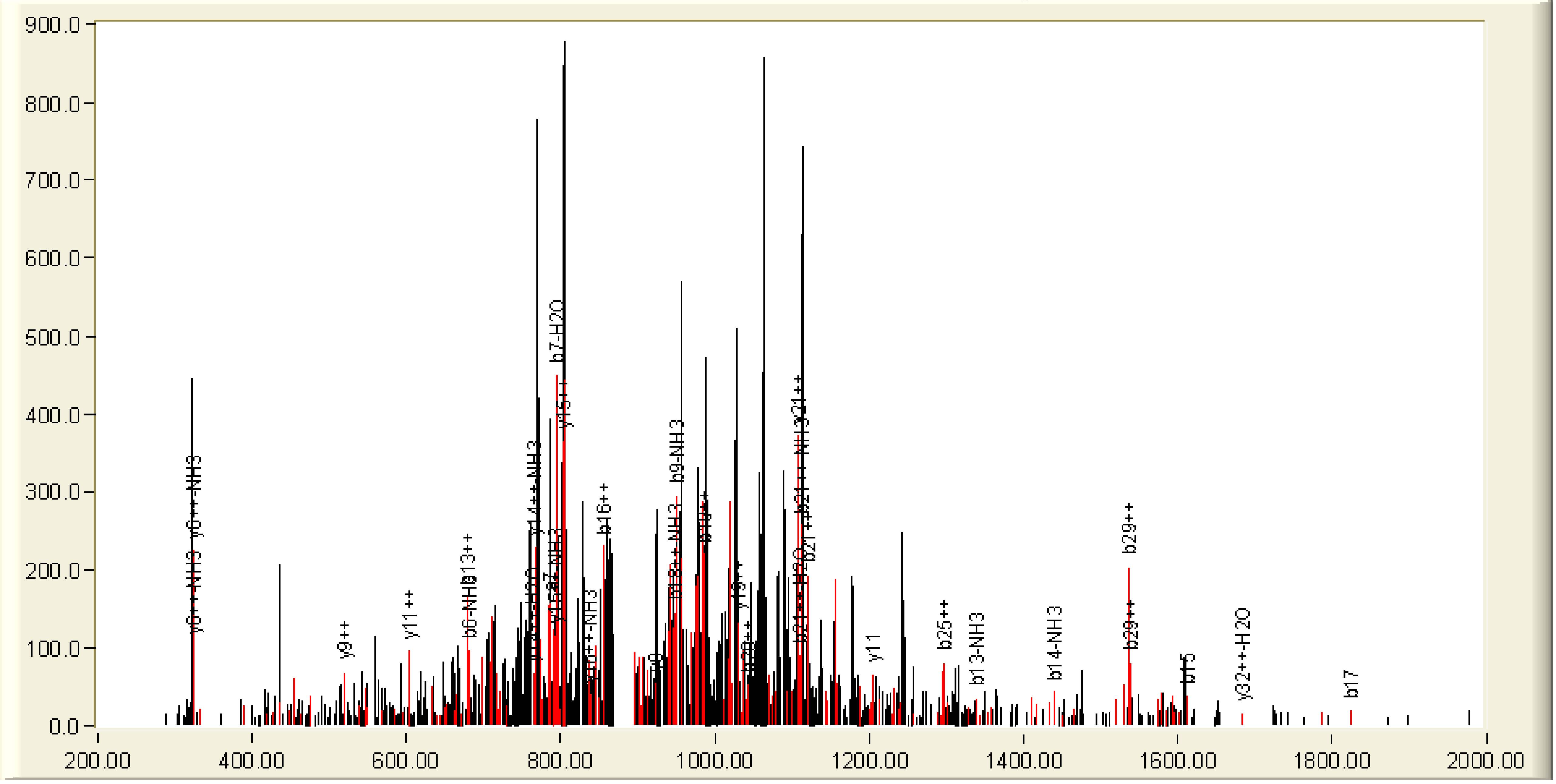

K.AVALQLDTSNIKTFDSFVGQLKQSLQNK.W Scan = 1221 CS = 3 Par m/z = 1032.19 gi|172055172 Xcorr = 3.41 delcn = 0.14

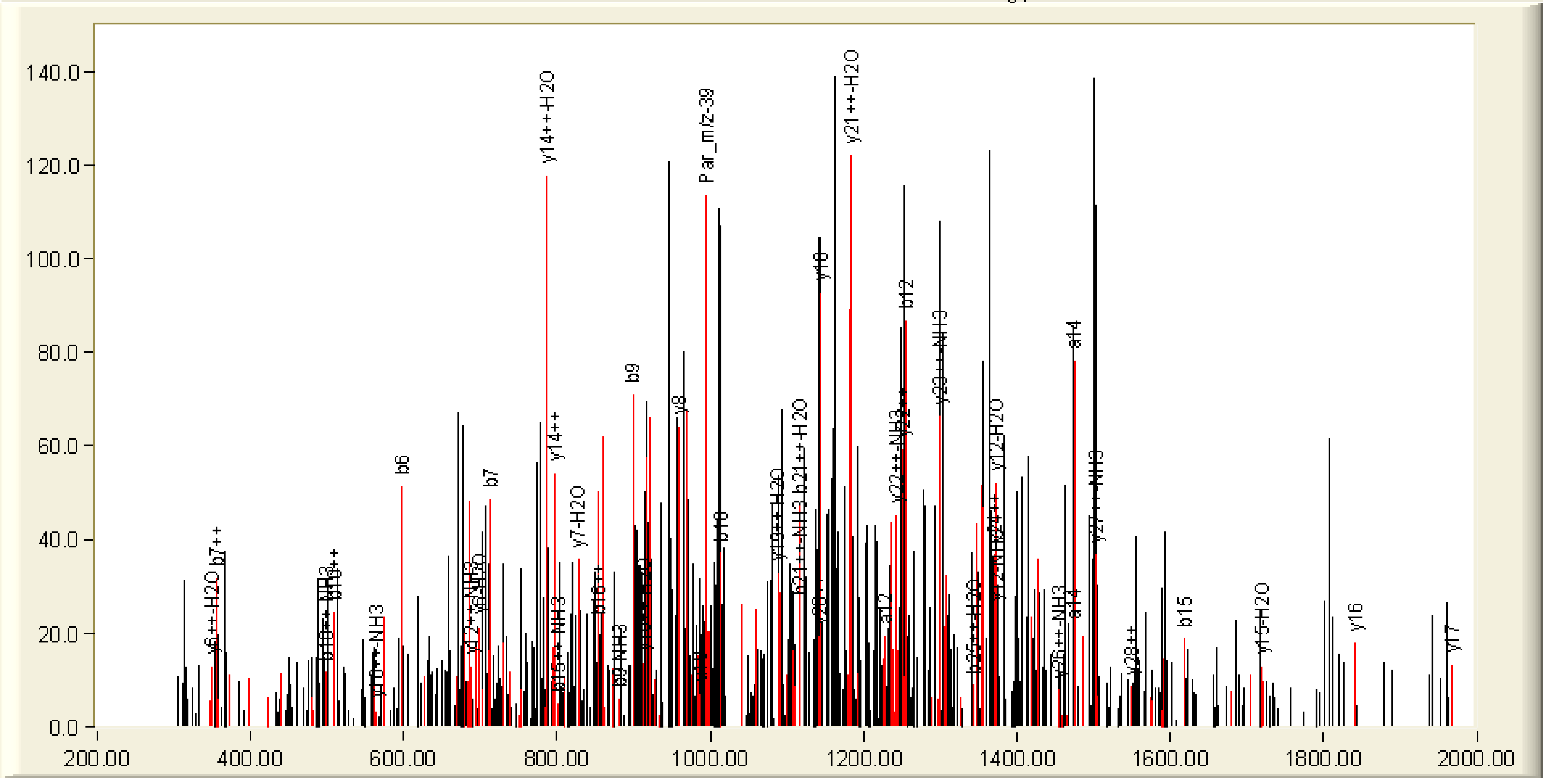

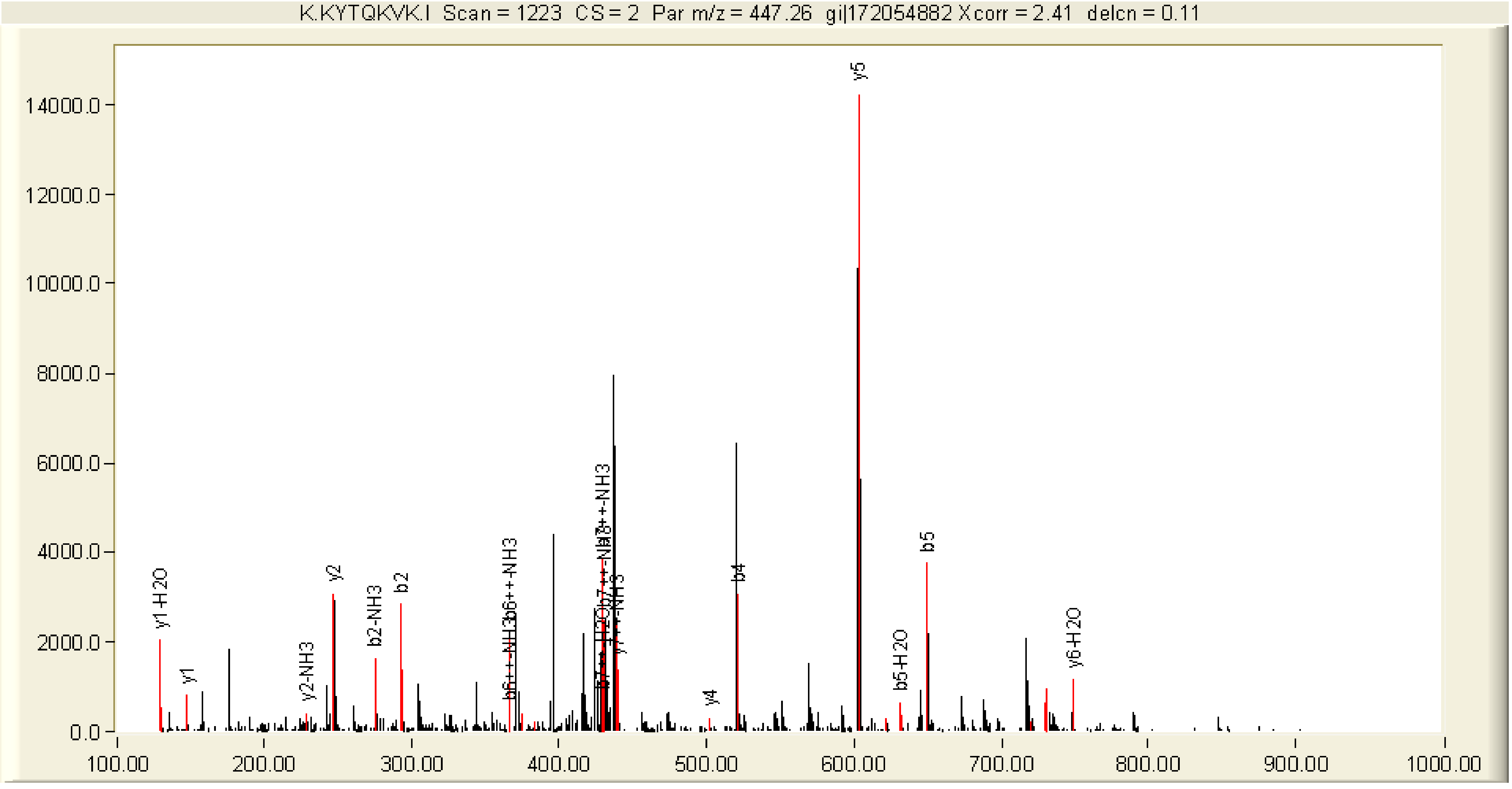

K.KYTQKVK.I Scan = 1223 CS = 2 Par m/z = 447.26 gi|172054882 Xcorr = 2.41 delcn = 0.11
14000.0
12000.0
10000.0
8000.0
6000.0
4000.0
2000.0
0.0
100.00
200.00
300.00
400.00
500.00
600.00
700.00
800.00
900.00
1000.00
y1-H2O
y1
y2-NH3
y2
b2-NH3
b2
b6++-NH3
b6++-NH3
y4
b4
y5
b5-H2O
b5
y6-H2O

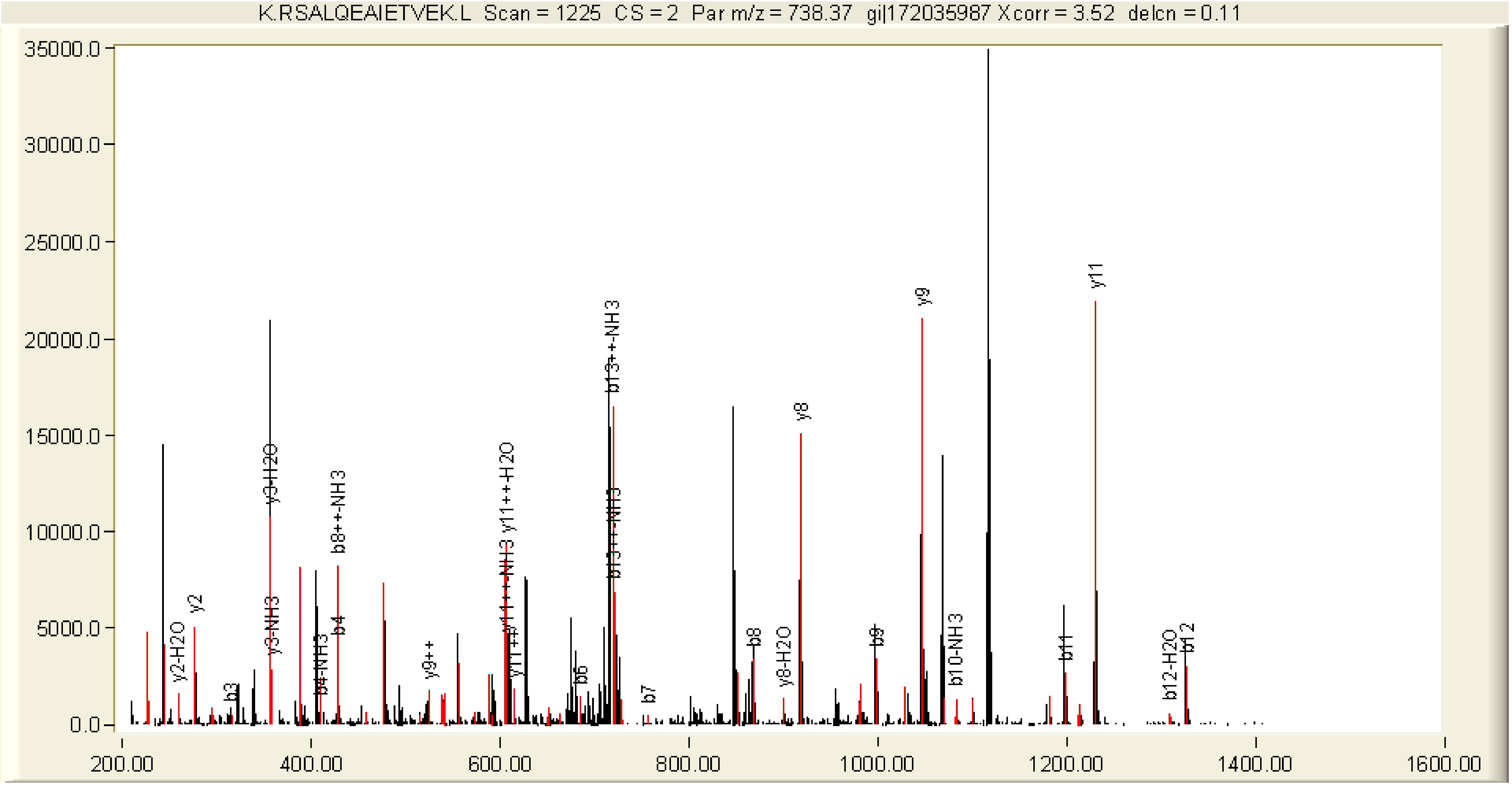
K.RSALQEAIETVEK.L Scan = 1225 CS = 2 Par m/z = 738.37 gi|172035987 Xcorr = 3.52 delcn = 0.11
35000.0
30000.0
25000.0
20000.0
15000.0
10000.0
5000.0
0.0
200.00
400.00
600.00
800.00
1000.00
1200.00
1400.00
1600.00
y2-H2O
y2
b3
y3-H2O
y3-NH3
b4-NH3
b8++-NH3
b4
y9++
y11++-NH3
y11++-H2O
y11++
b6
b13++-NH3
b7
b8
y8-H2O
y8
b9
y9
b10-NH3
b11
y11
b12-H2O
b12

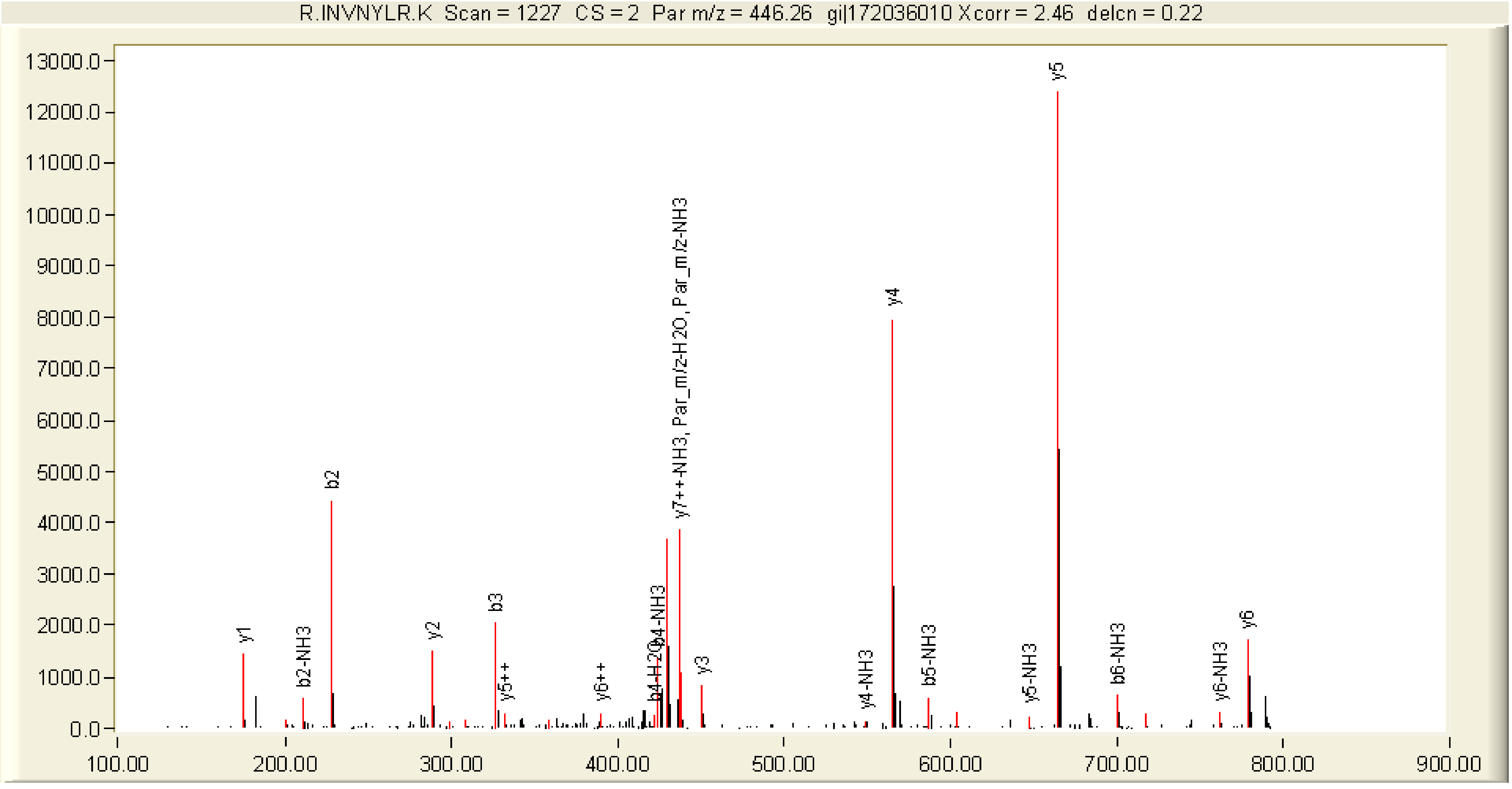

R.INVNYLR.K Scan = 1227 CS = 2 Par m/z = 446.26 gi|172036010 Xcorr = 2.46 delcn = 0.22
0.0
1000.0
2000.0
3000.0
4000.0
5000.0
6000.0
7000.0
8000.0
9000.0
10000.0
11000.0
12000.0
13000.0
100.00
200.00
300.00
400.00
500.00
600.00
700.00
800.00
900.00
y1
b2-NH3
b2
y2
b3
y5++
y6++
b4-H2O
b4-NH3
y7++-NH3, Par_m/z-H2O, Par_m/z-NH3
y3
y4-NH3
y4
b5-NH3
y5-NH3
y5
b6-NH3
y6-NH3
y6

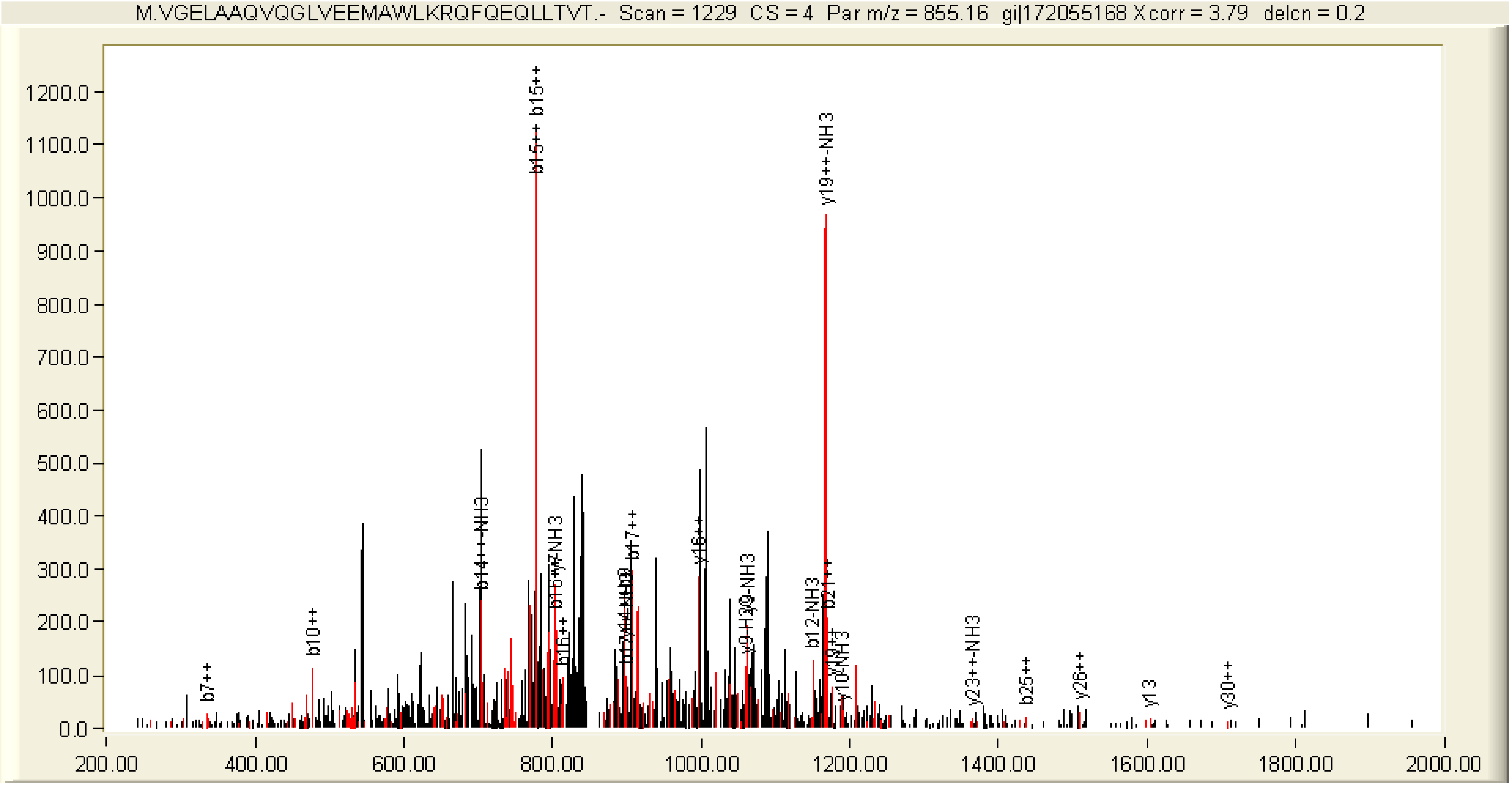
M.VGELAAQVQGLVEEMAWLKRQFQEQLLTVT.- Scan = 1229 CS = 4 Par m/z = 855.16 gi|172055168 Xcorr = 3.79 delcn = 0.2
b7++
b10++
b14++-NH3
b15++ b15++
y19++-NH3
b17++
y16++
y9-NH3
b12-NH3
b21++
y23++-NH3
b25++
y26++
y13
y30++
1200.0
1100.0
1000.0
900.0
800.0
700.0
600.0
500.0
400.0
300.0
200.0
100.0
0.0
200.00
400.00
600.00
800.00
1000.00
1200.00
1400.00
1600.00
1800.00
2000.00

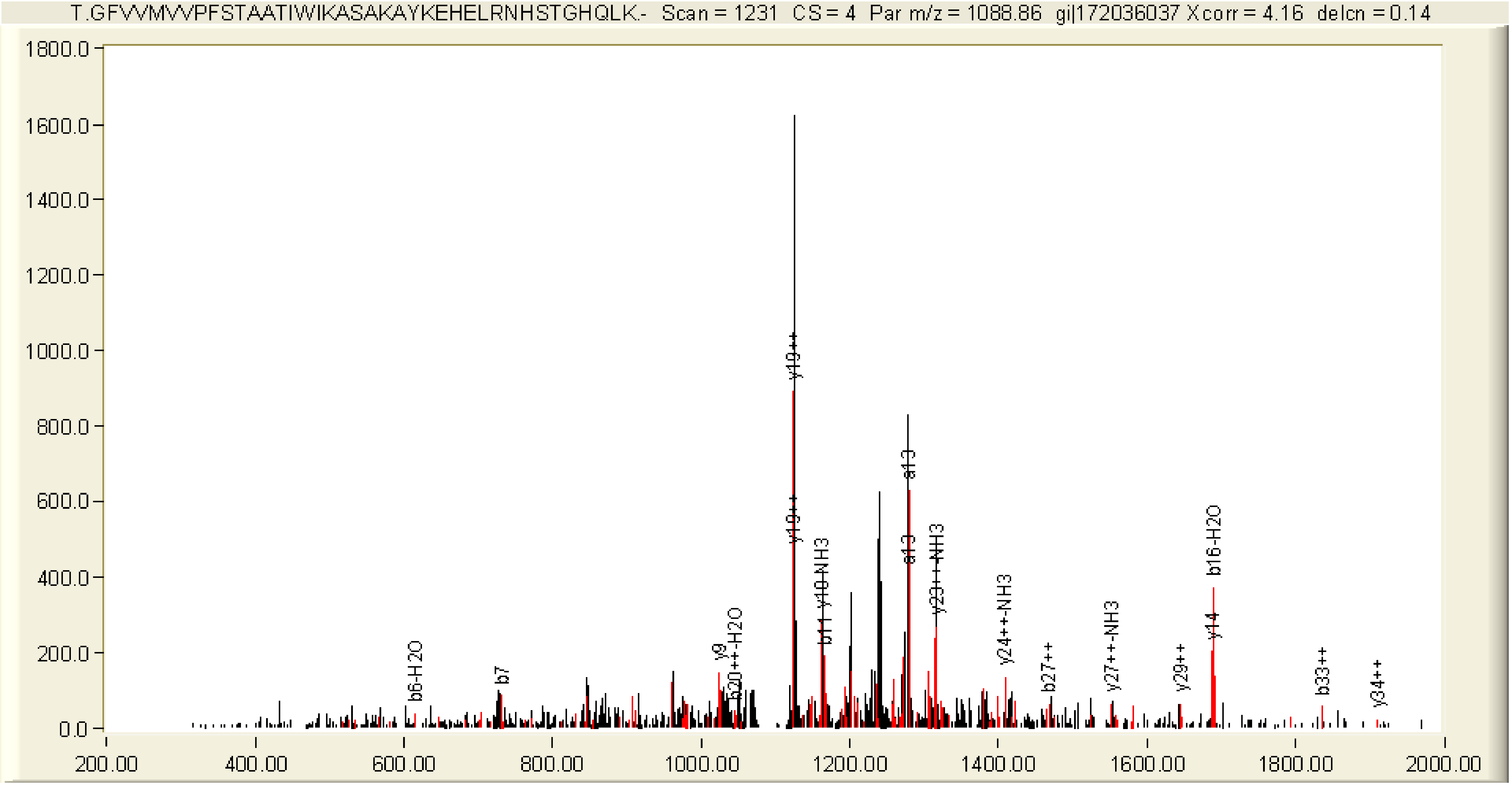

T.GFVVMVVPFSTAATIWIKASAKAYKEHELRNHSTGHQLK.- Scan = 1231 CS = 4 Par m/z = 1088.86 gi|172036037 Xcorr = 4.16 delcn = 0.14
b6-H2O
b7
y9
b20++-H2O
y19++
b11
y10-NH3
a13
y29++-NH3
y24++-NH3
b27++
y27++-NH3
y29++
y14
b16-H2O
b33++
y34++
0.0
200.0
400.0
600.0
800.0
1000.0
1200.0
1400.0
1600.0
1800.0
200.00
400.00
600.00
800.00
1000.00
1200.00
1400.00
1600.00
1800.00
2000.00

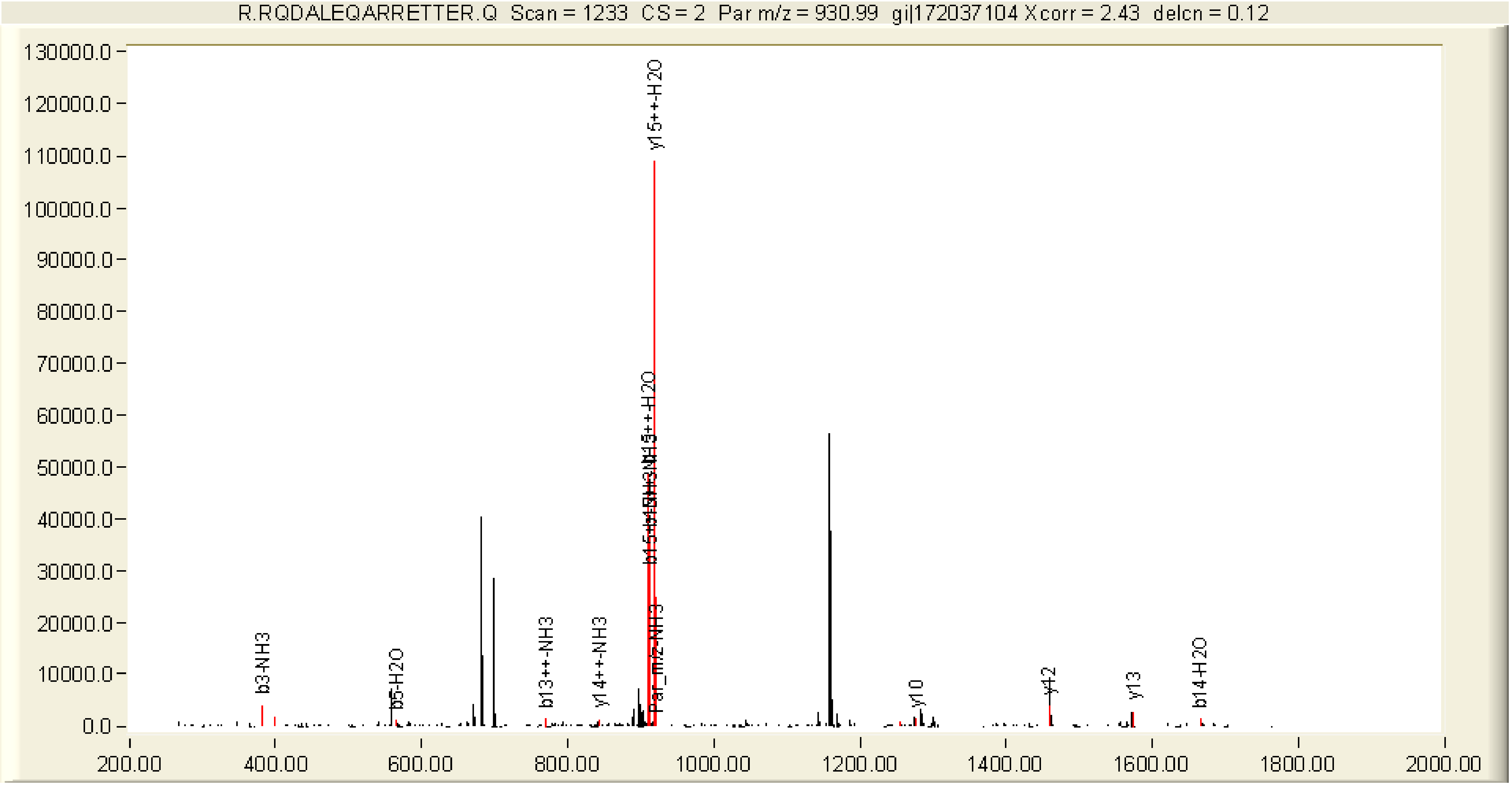

R.RQDALEQARRETTER.Q Scan = 1233 CS = 2 Par m/z = 930.99 gi|172037104 Xcorr = 2.43 delcn = 0.12
130000.0
120000.0
110000.0
100000.0
90000.0
80000.0
70000.0
60000.0
50000.0
40000.0
30000.0
20000.0
10000.0
0.0
y15++-H2O
b3-NH3
b5-H2O
b13++-NH3
y14++-NH3
Par_m/z-NH3
y10
y12
y13
b14-H2O
200.00
400.00
600.00
800.00
1000.00
1200.00
1400.00
1600.00
1800.00
2000.00

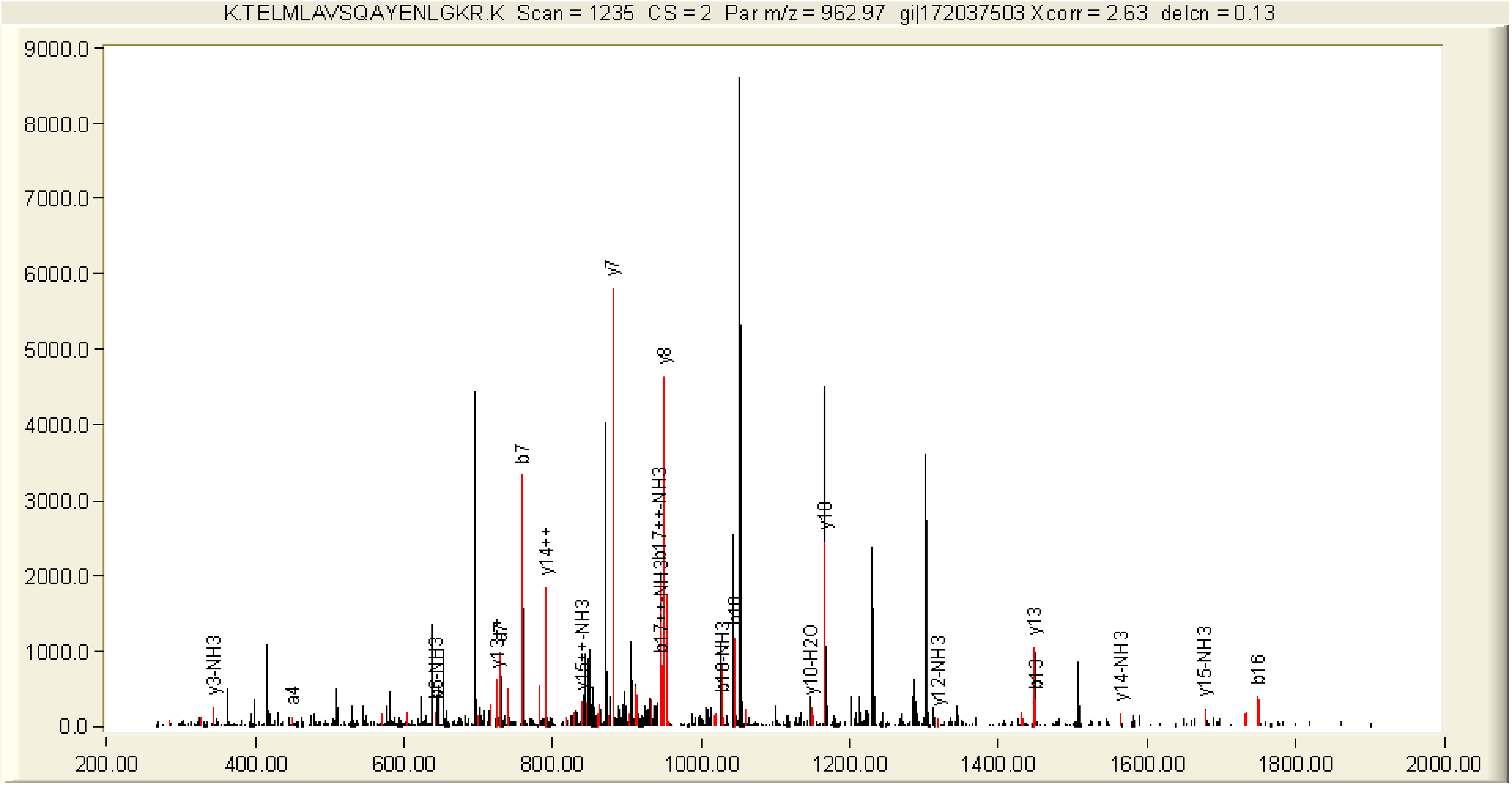

K.TELMLAVSQAYENLGKR.K Scan = 1235 CS = 2 Par m/z = 962.97 gi|172037503 Xcorr = 2.63 delcn = 0.13
9000.0
8000.0
7000.0
6000.0
5000.0
4000.0
3000.0
2000.0
1000.0
0.0
200.00
400.00
600.00
800.00
1000.00
1200.00
1400.00
1600.00
1800.00
2000.00
y3-NH3
a4
b7
y14++
y7
y8
y10
y10-H2O
y12-NH3
y13
y14-NH3
y15-NH3
b16

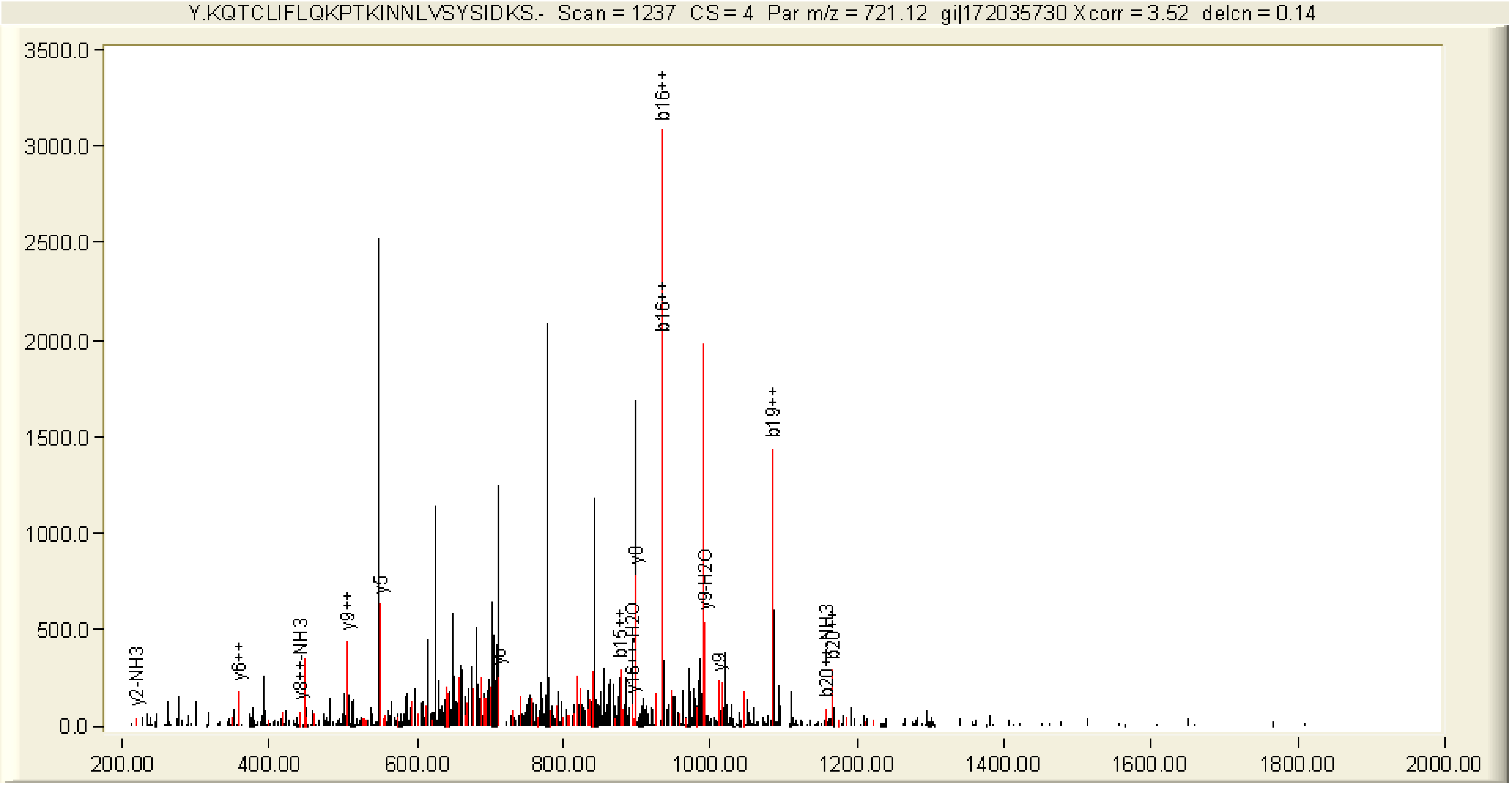
Y.KQTCLIFLQKPTKINNLVSYSIDKS.- Scan = 1237 CS = 4 Par m/z = 721.12 gi|172035730 Xcorr = 3.52 delcn = 0.14
3500.0
3000.0
2500.0
2000.0
1500.0
1000.0
500.0
0.0
200.00
400.00
600.00
800.00
1000.00
1200.00
1400.00
1600.00
1800.00
2000.00
b16++
b16++
b19++
y9-H2O
y8
y5
y9++
y8++-NH3
y6++
y2-NH3
b15++
y9
b20++
b20++-NH3

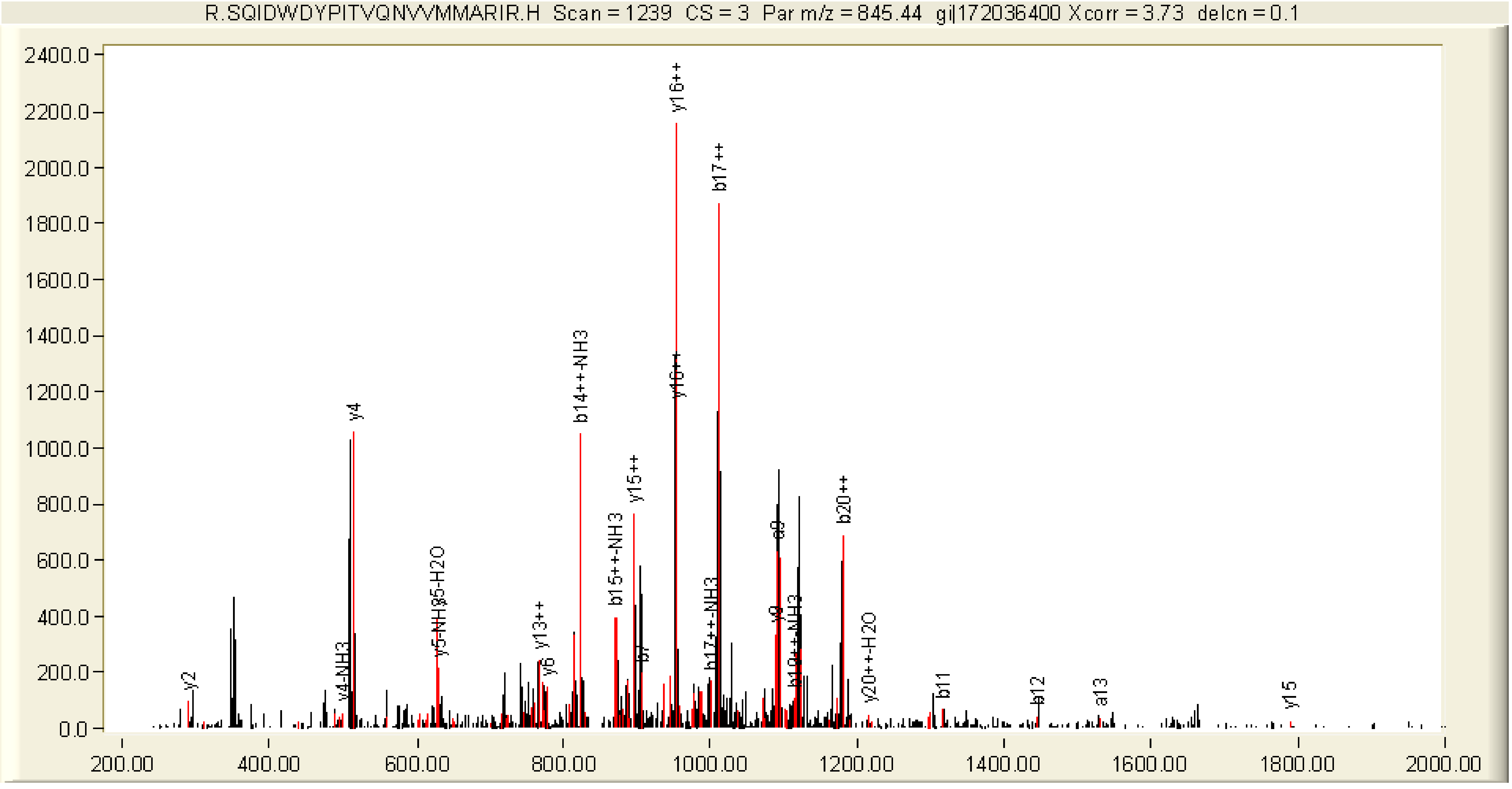

R.SQIDWDYPITVQNVVMMARIR.H Scan = 1239 CS = 3 Par m/z = 845.44 gi|172036400 Xcorr = 3.73 delcn = 0.1
2400.0
2200.0
2000.0
1800.0
1600.0
1400.0
1200.0
1000.0
800.0
600.0
400.0
200.0
0.0
y2
y4-NH3
y4
y5-H2O
y13++
y6
b14++-NH3
b15++-NH3
y15++
b7
y16++
y16++
b17++-NH3
b17++
y9
a9
b20++
y20++-H2O
b11
b12
a13
y15
200.00
400.00
600.00
800.00
1000.00
1200.00
1400.00
1600.00
1800.00
2000.00

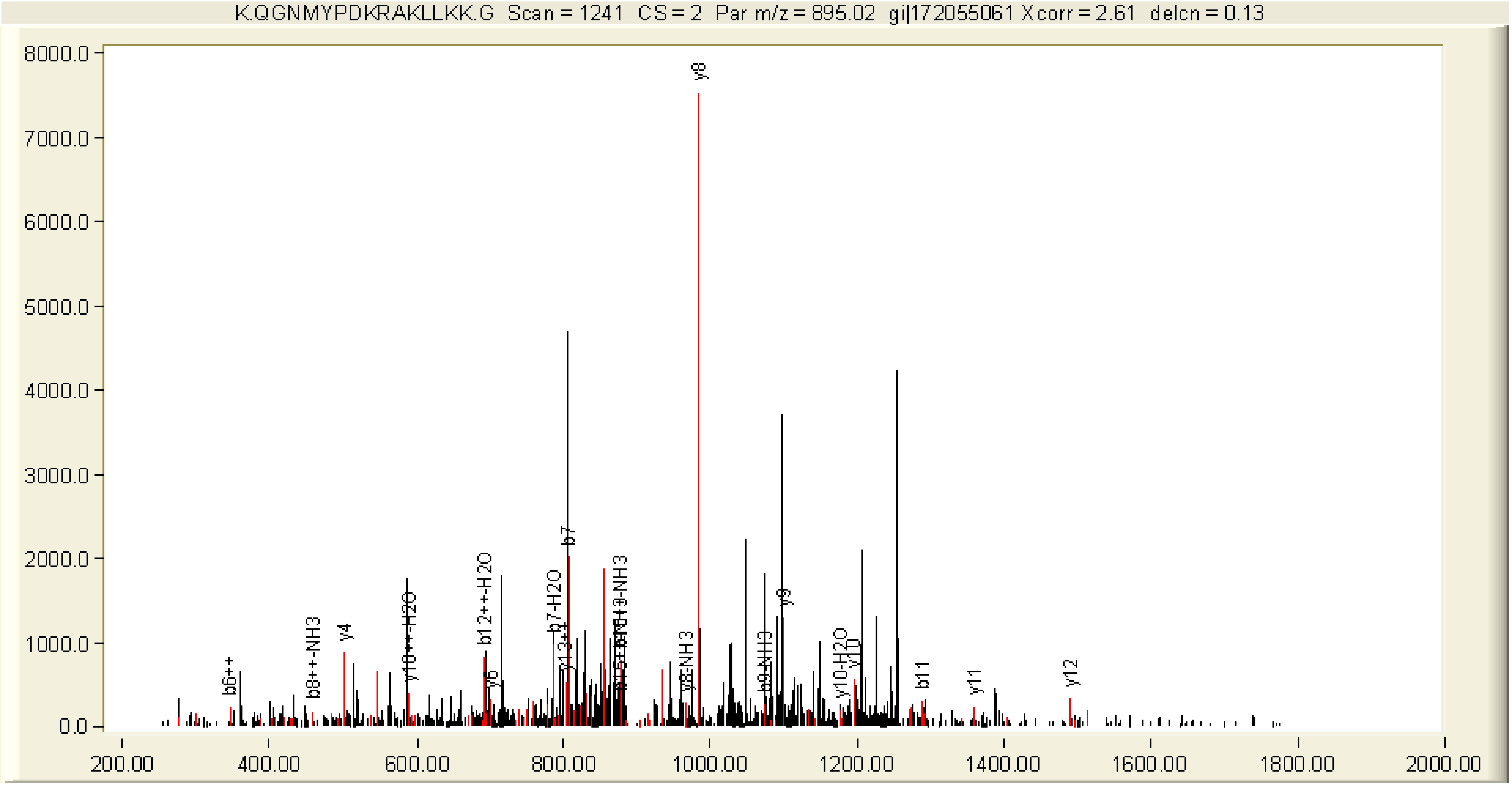
K.QGNMYPDKRAKLLKK.G Scan = 1241 CS = 2 Par m/z = 895.02 gi|172055061 Xcorr = 2.61 delcn = 0.13
8000.0
7000.0
6000.0
5000.0
4000.0
3000.0
2000.0
1000.0
0.0
200.00
400.00
600.00
800.00
1000.00
1200.00
1400.00
1600.00
1800.00
2000.00
y8
b7
b12++-H2O
b7-H2O
y4
y10++-H2O
b8++-NH3
b6++
y6
y8-NH3
b9-NH3
y9
y10-H2O
y10
b11
y11
y12

-.MTVLLPAIIPVAFIIIIGVIVGKTLEIHKQTLSQLI.V Scan = 1243 CS = 4 Par m/z = 974.54 gi|172036557 Xcorr = 2.83 delcn = 0.12

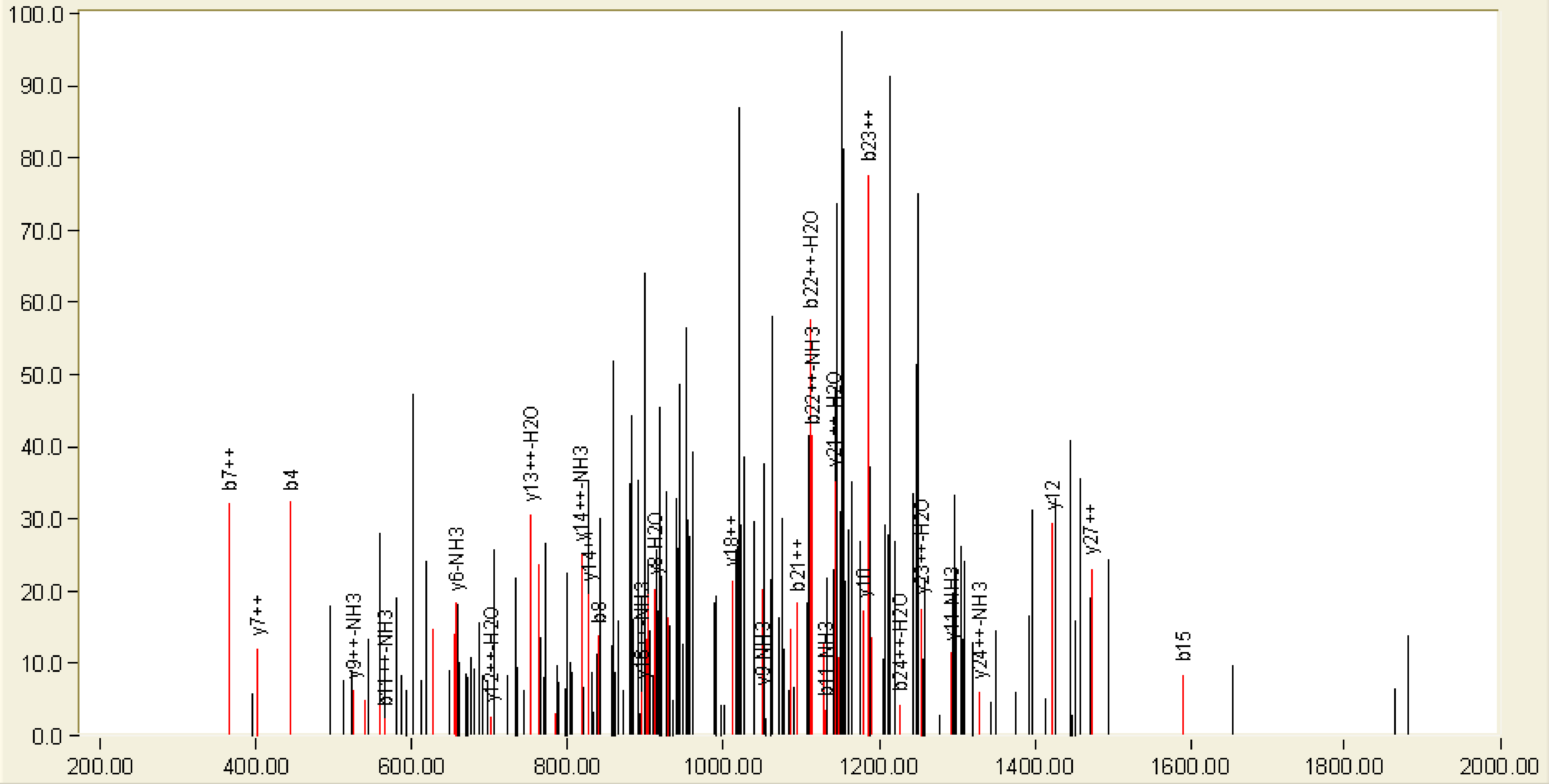

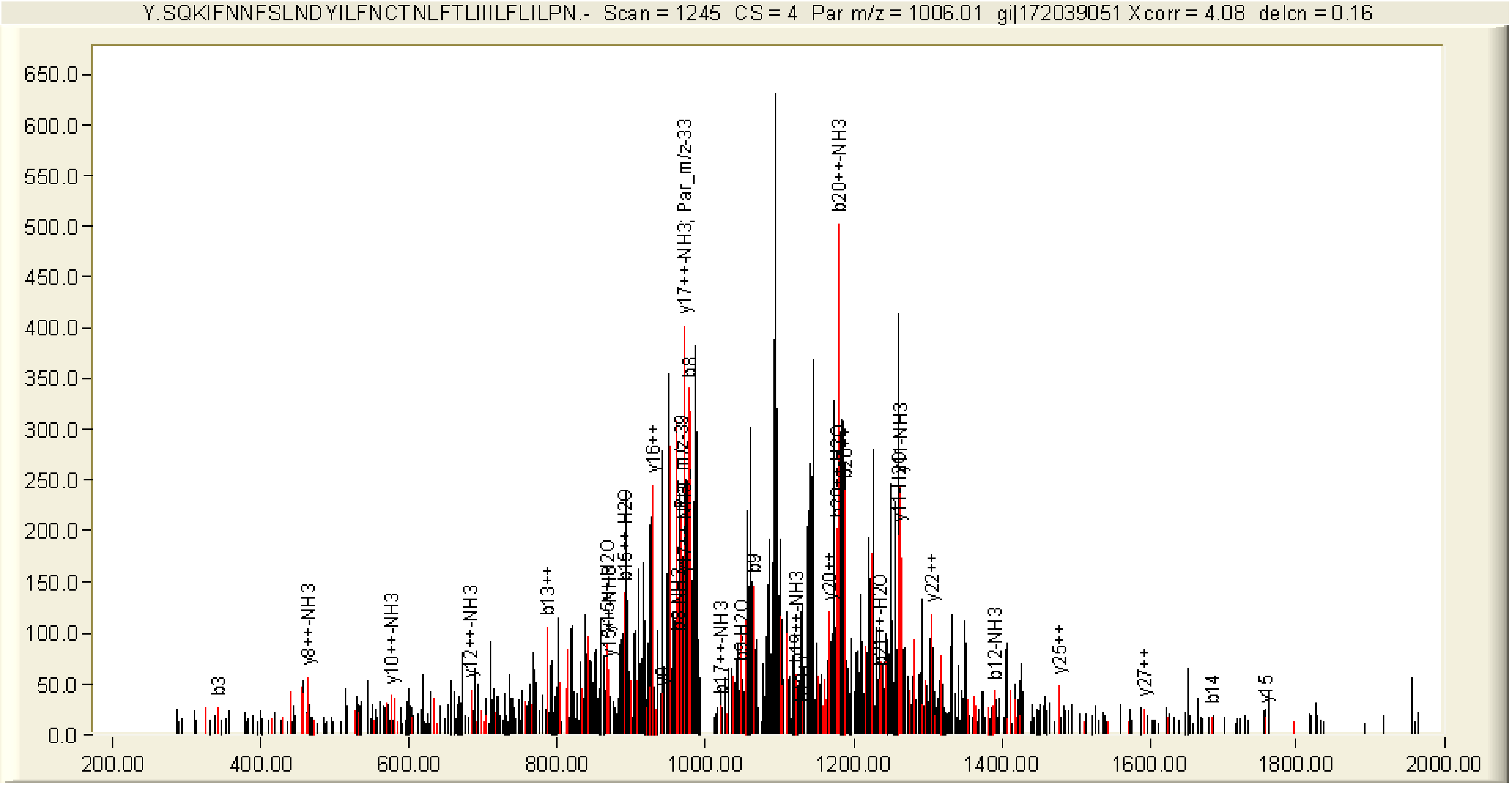
Y.SQKIFNNFSLNDYILFNCTNLFTLIIILFLILPN.- Scan = 1245 CS = 4 Par m/z = 1006.01 gi|172039051 Xcorr = 4.08 delcn = 0.16
650.0
600.0
550.0
500.0
450.0
400.0
350.0
300.0
250.0
200.0
150.0
100.0
50.0
0.0
200.00
400.00
600.00
800.00
1000.00
1200.00
1400.00
1600.00
1800.00
2000.00
b3
y8++-NH3
y10++-NH3
y12++-NH3
b13++
b15++-H2O
y16++
y17++-NH3; Par_m/z-33
b8
b20++-NH3
y20++
b9
y22++
b12-NH3
y25++
y27++
b14
y15

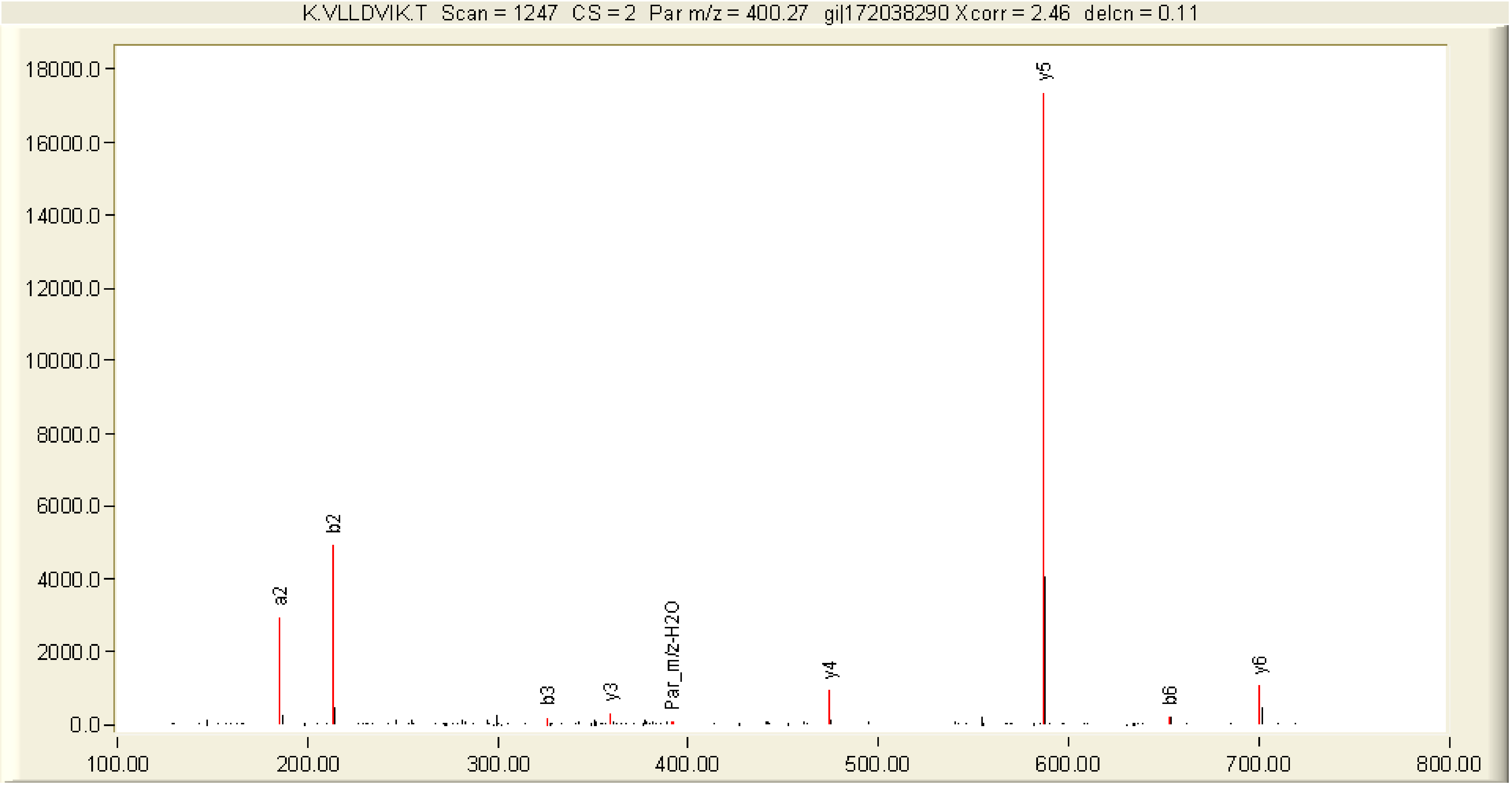
K.VLLDVIK.T Scan = 1247 CS = 2 Par m/z = 400.27 gi|172038290 Xcorr = 2.46 delcn = 0.11
18000.0
16000.0
14000.0
12000.0
10000.0
8000.0
6000.0
4000.0
2000.0
0.0
100.00
200.00
300.00
400.00
500.00
600.00
700.00
800.00
a2
b2
b3
y3
Par_m/z-H2O
y4
y5
b6
y6

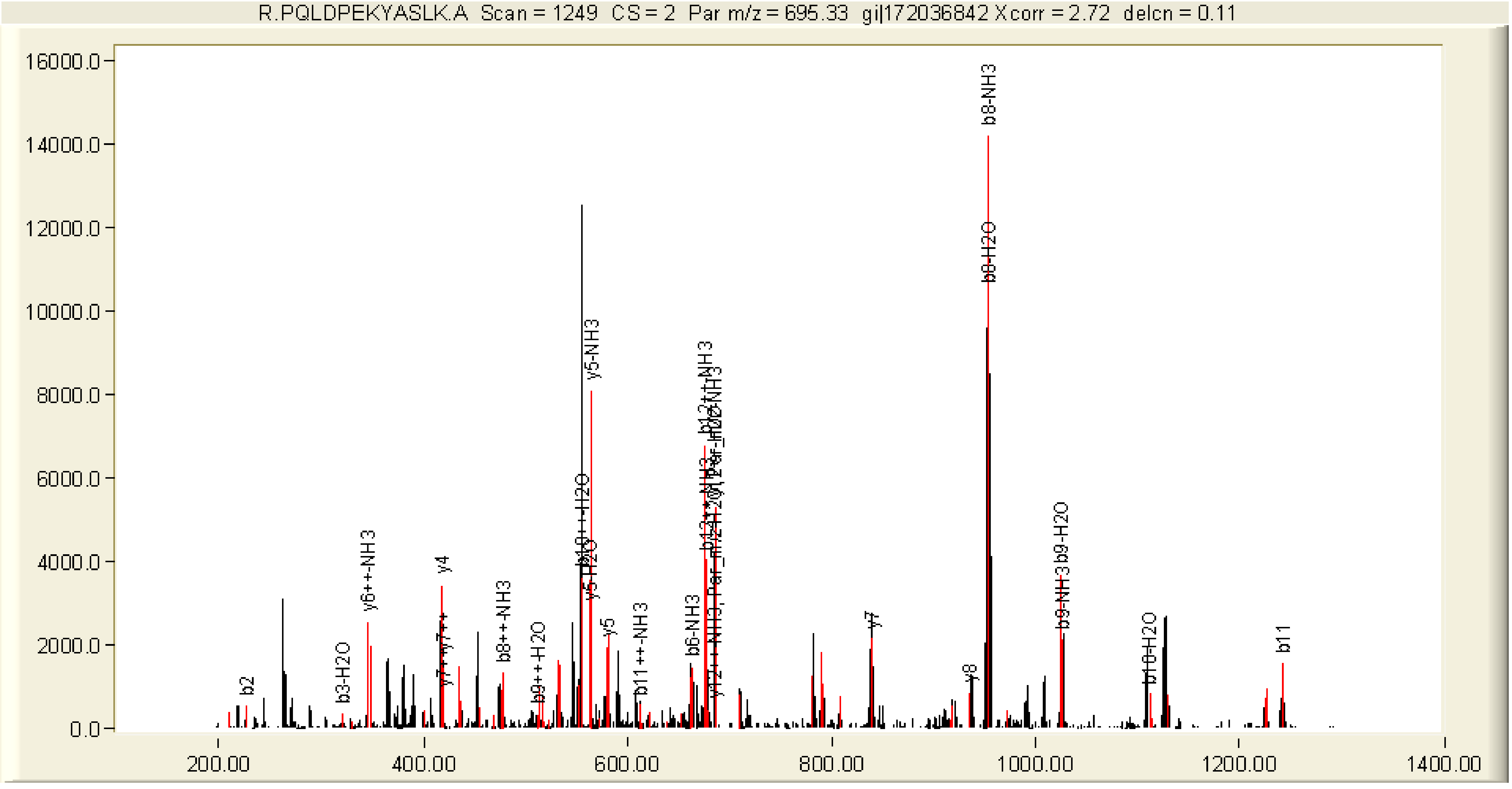
R.PQLDPEKYASLK.A Scan = 1249 CS = 2 Par m/z = 695.33 gi|172036842 Xcorr = 2.72 delcn = 0.11
16000.0
14000.0
12000.0
10000.0
8000.0
6000.0
4000.0
2000.0
0.0
200.00
400.00
600.00
800.00
1000.00
1200.00
1400.00
b2
b3-H2O
y6++-NH3
y4
b8++-NH3
b9++-H2O
y5-NH3
y5
b11++-NH3
b6-NH3
b8-NH3
b8-H2O
y7
y8
b9-NH3
b9-H2O
b10-H2O
b11

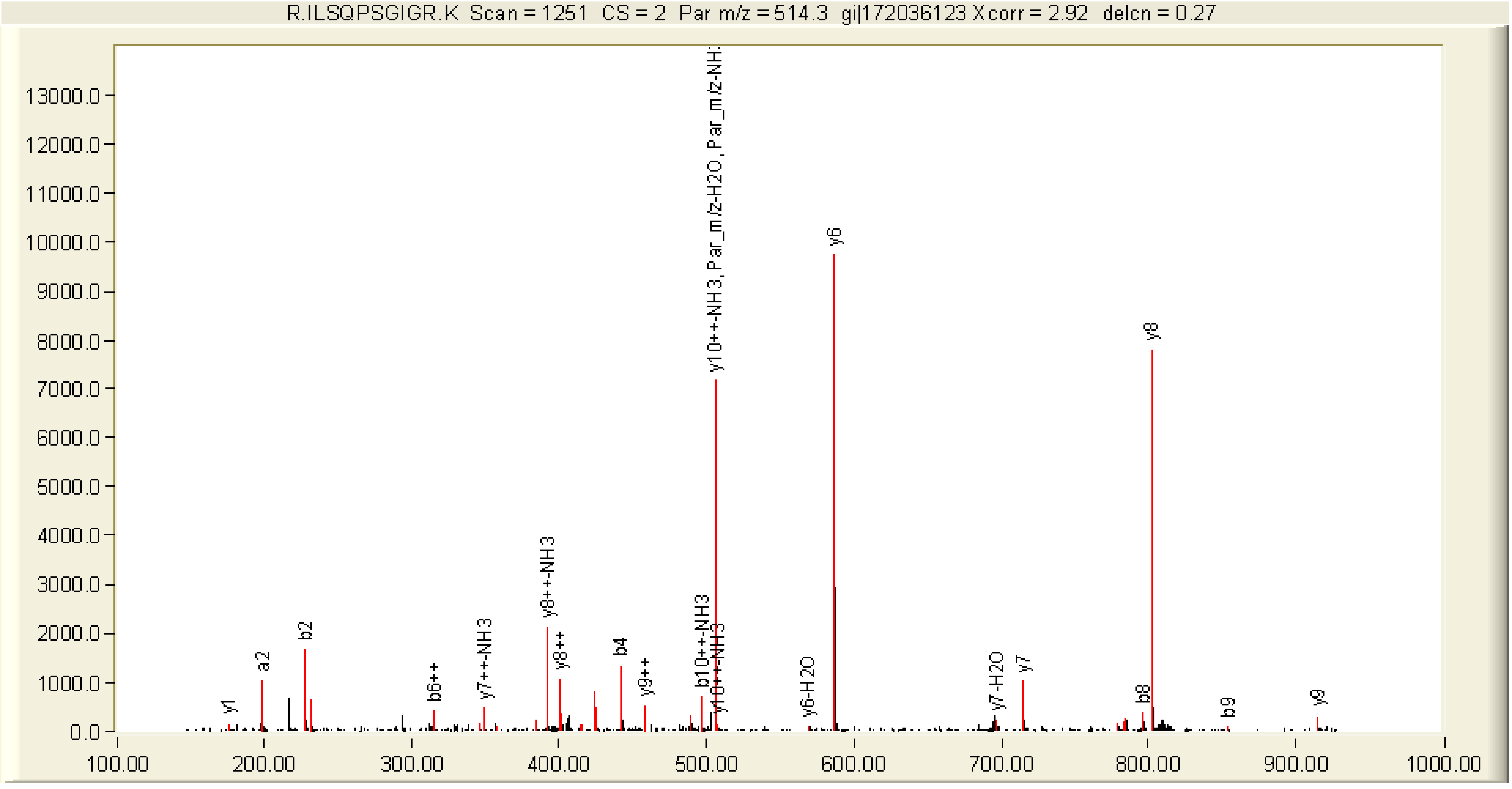
R.ILSQPSGIGR.K Scan = 1251 CS = 2 Par m/z = 514.3 gi|172036123 Xcorr = 2.92 delcn = 0.27
y10++-NH3, Par_m/z-H2O, Par_m/z-NH3
y6
y8
y8++-NH3
b2
a2
y1
b6++
y7++-NH3
y8++
b4
y9++
b10++-NH3
y10++-NH3
y6-H2O
y7-H2O
y7
b8
b9
y9
0.0
1000.0
2000.0
3000.0
4000.0
5000.0
6000.0
7000.0
8000.0
9000.0
10000.0
11000.0
12000.0
13000.0
100.00
200.00
300.00
400.00
500.00
600.00
700.00
800.00
900.00
1000.00

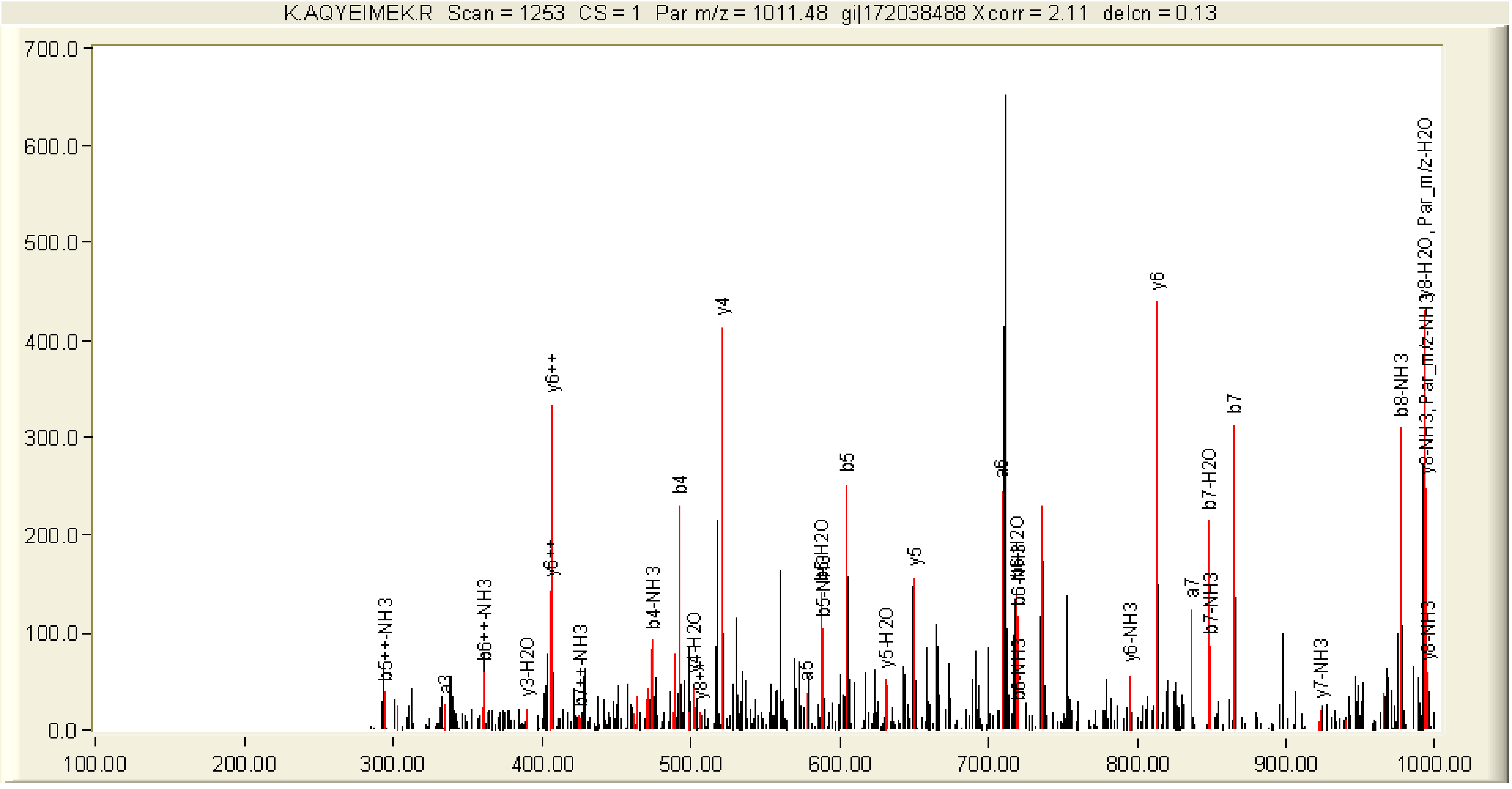

K.AQYEIMEK.R Scan = 1253 CS = 1 Par m/z = 1011.48 gi|172038488 Xcorr = 2.11 delcn = 0.13
700.0
600.0
500.0
400.0
300.0
200.0
100.0
0.0
100.00
200.00
300.00
400.00
500.00
600.00
700.00
800.00
900.00
1000.00
b5++-NH3
a3
b6++-NH3
y3-H2O
y6++
y6++
b7++-NH3
b4-NH3
b4
y4-H2O
y4
a5
b5-NH3
b5
y5-H2O
y5
a6
y6-NH3
y6
a7
b7-H2O
b7-NH3
b7
y7-NH3
b8-NH3
y8-NH3

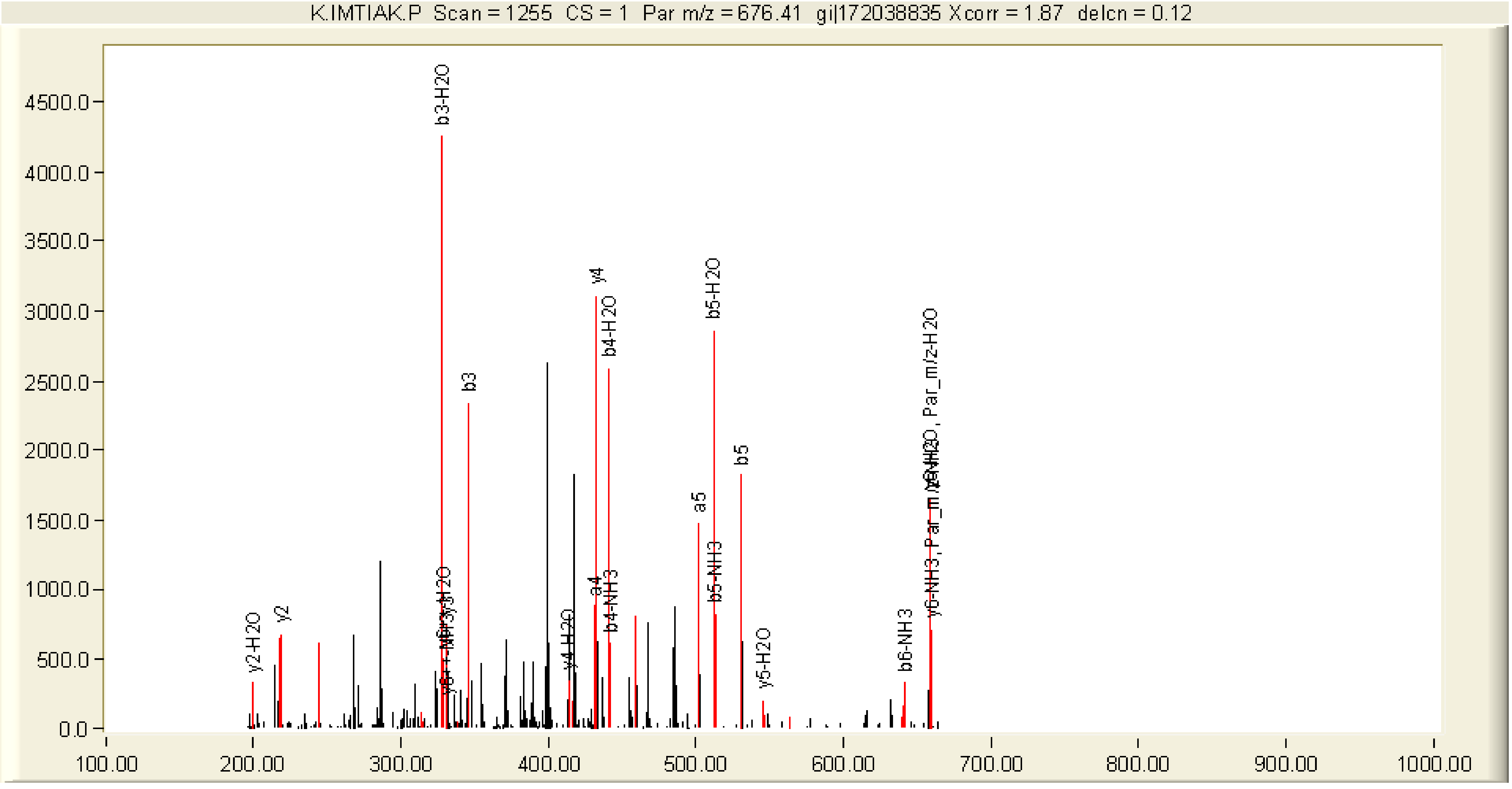

K.IMTIAK.P Scan = 1255 CS = 1 Par m/z = 676.41 gi|172038835 Xcorr = 1.87 delcn = 0.12
4500.0
4000.0
3500.0
3000.0
2500.0
2000.0
1500.0
1000.0
500.0
0.0
100.00
200.00
300.00
400.00
500.00
600.00
700.00
800.00
900.00
1000.00
b3-H2O
b3
y4
b4-H2O
b5-H2O
b5
a5
b5-NH3
a4
b4-NH3
y4-H2O
y2-H2O
y2
y5-H2O
b6-NH3
y6-NH3, Par_m/z-NH3
y6-H2O, Par_m/z-H2O

I.GNRTAAIADLKQAKTLLKQQGQTSRYPEIDNLLD.- Scan = 1257 CS = 4 Par m/z = 941.51 gi|172037193 Xcorr = 3.36 delcn = 0.17

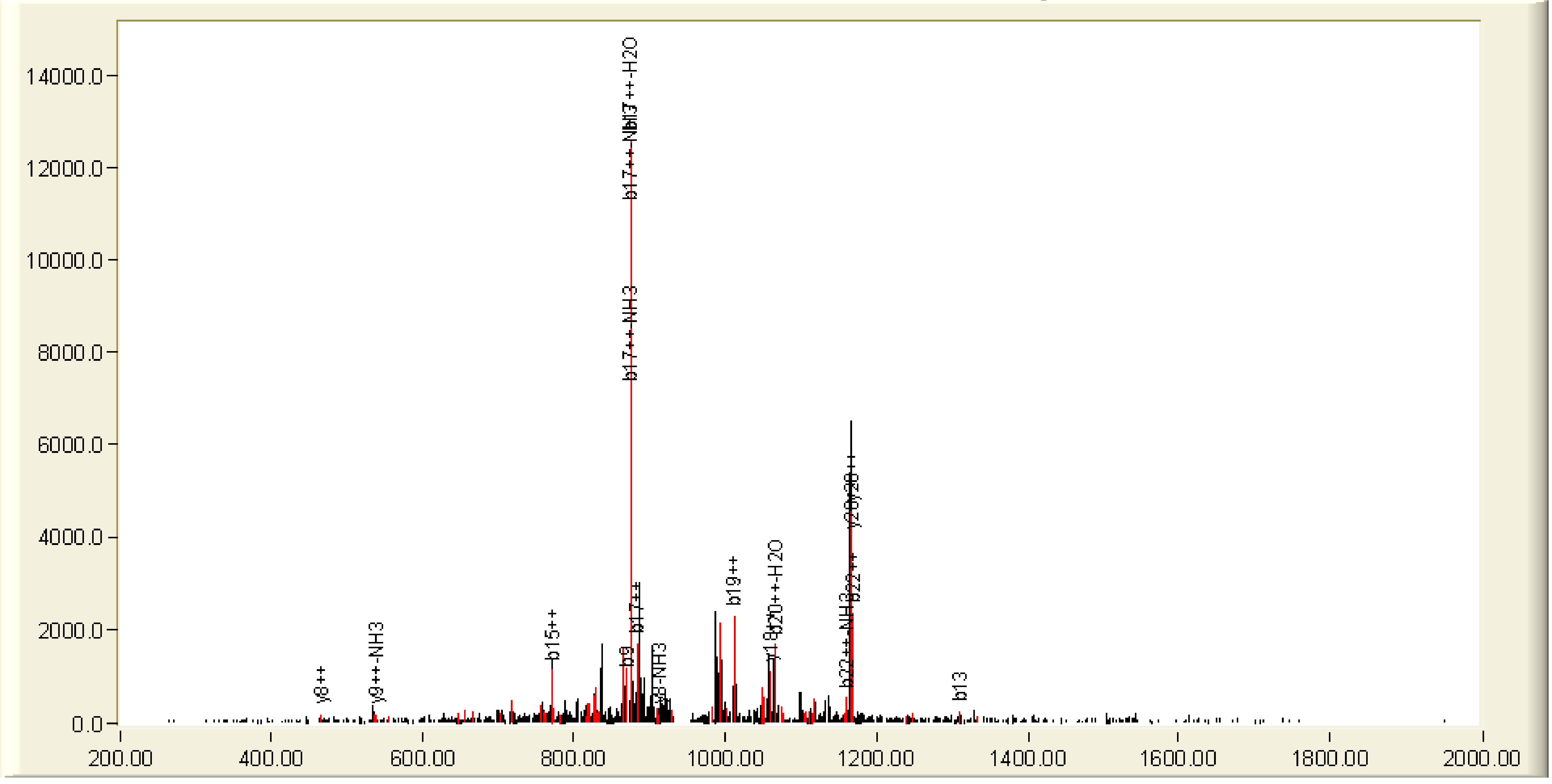

R.IFGPDADNLTDPDAIATVAR.D Scan = 1259 CS = 2 Par m/z = 1035.52 gi|172037929 Xcorr = 3.23 delcn = 0.13

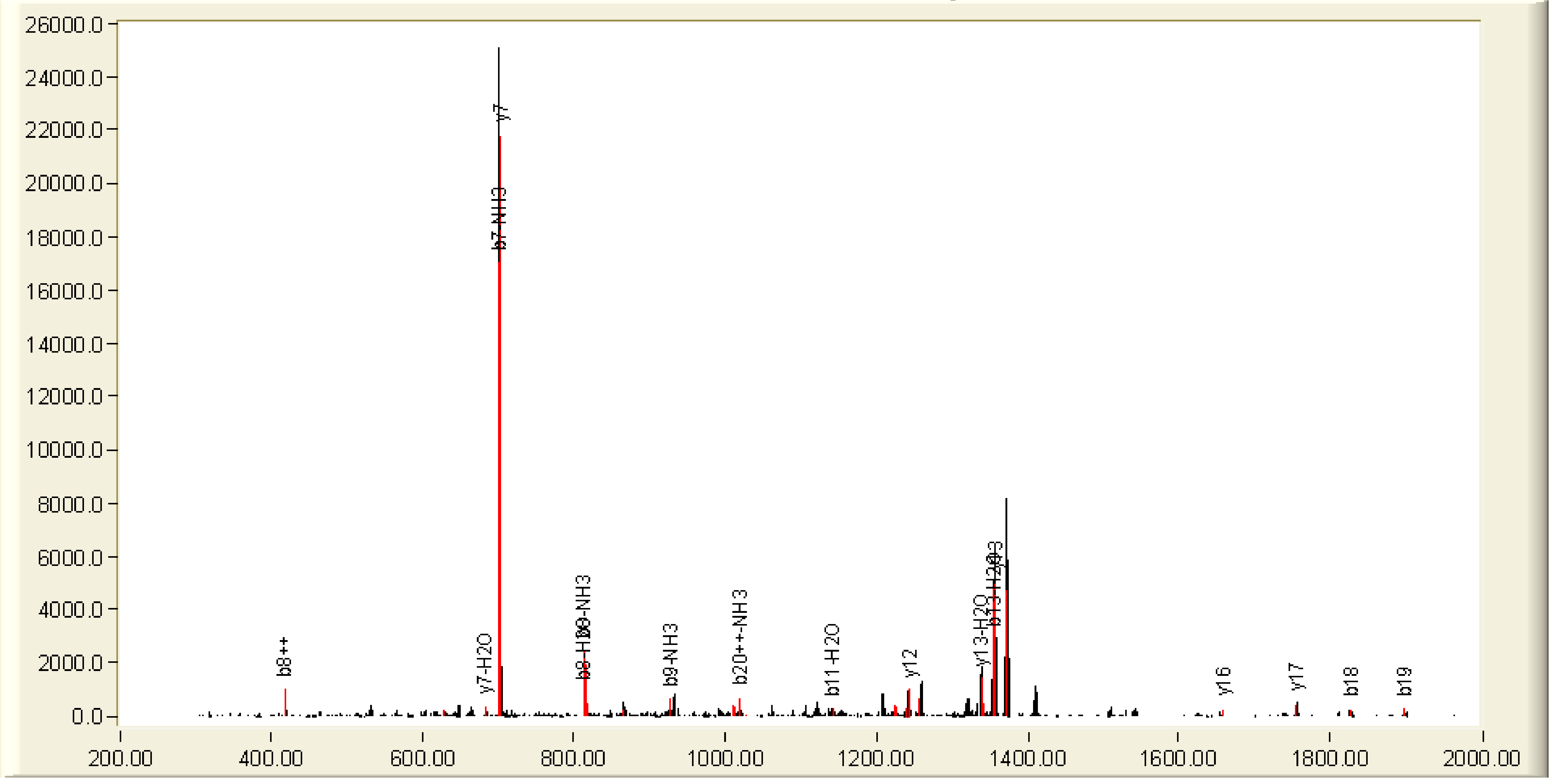

R.RDRWSYKFFLHLPNSAMKTK.K Scan = 1261 CS = 3 Par m/z = 840.78 gi|172037065 Xcorr = 4.41 delcn = 0.16

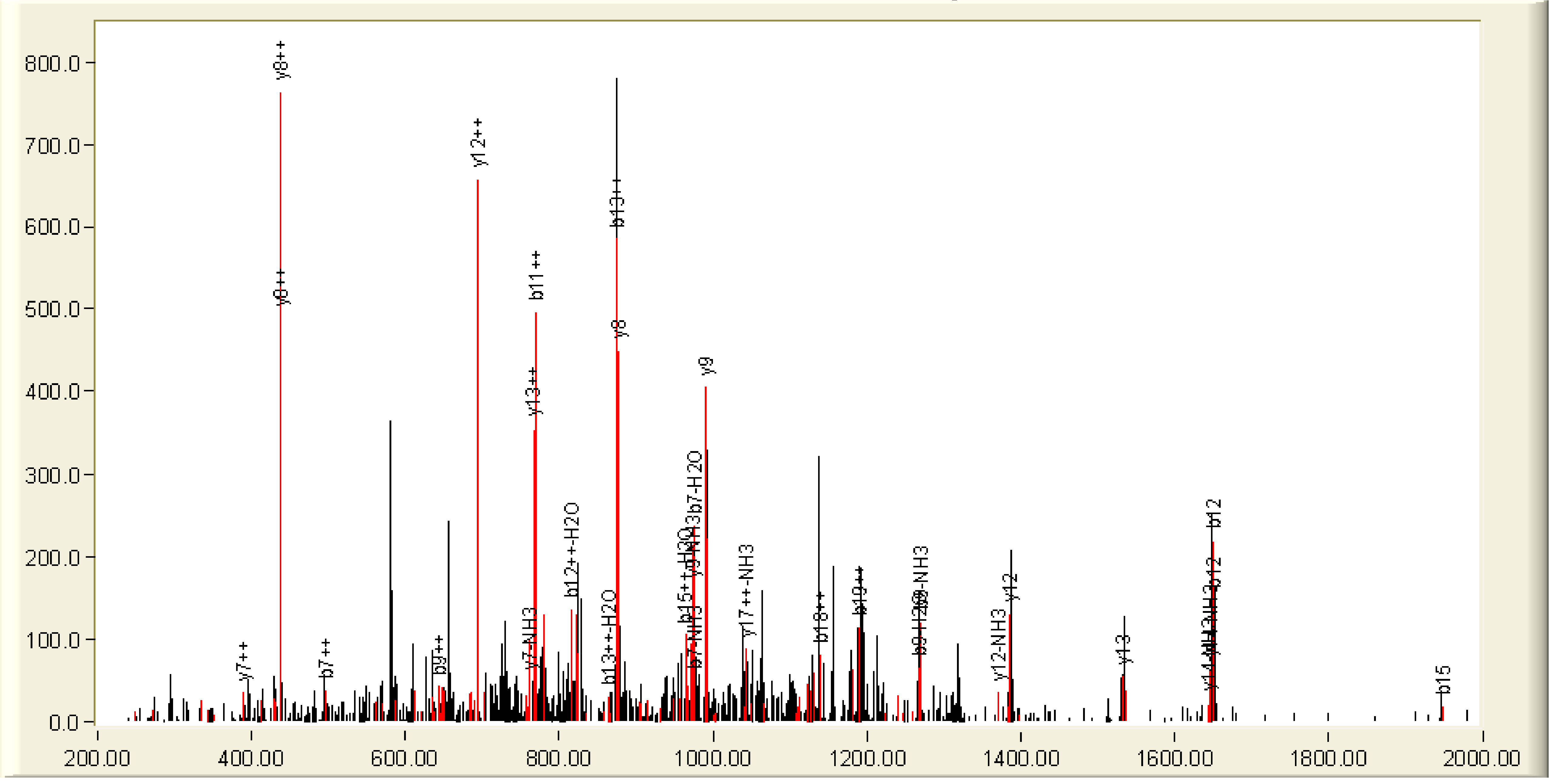

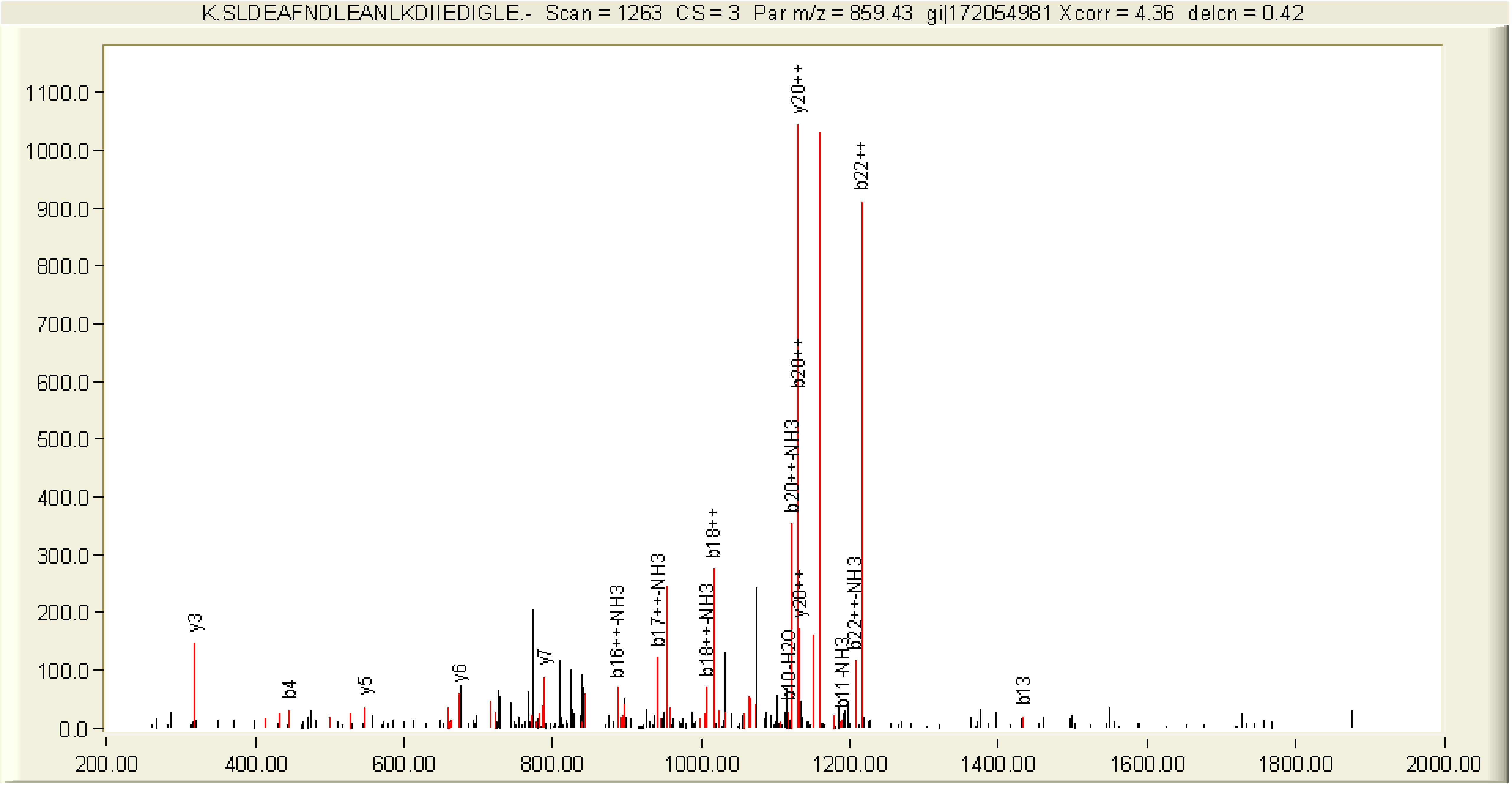
K.SLDEAFNDLEANLKDIIEDIGLE.- Scan = 1263 CS = 3 Par m/z = 859.43 gi|172054981 Xcorr = 4.36 delcn = 0.42
y3
b4
y5
y6
y7
b16++-NH3
b17++-NH3
b18++-NH3
b18++
b10-H2O
b20++-NH3
b20++
y20++
y20++
b11-NH3
b22++-NH3
b22++
b13
1100.0
1000.0
900.0
800.0
700.0
600.0
500.0
400.0
300.0
200.0
100.0
0.0
200.00
400.00
600.00
800.00
1000.00
1200.00
1400.00
1600.00
1800.00
2000.00

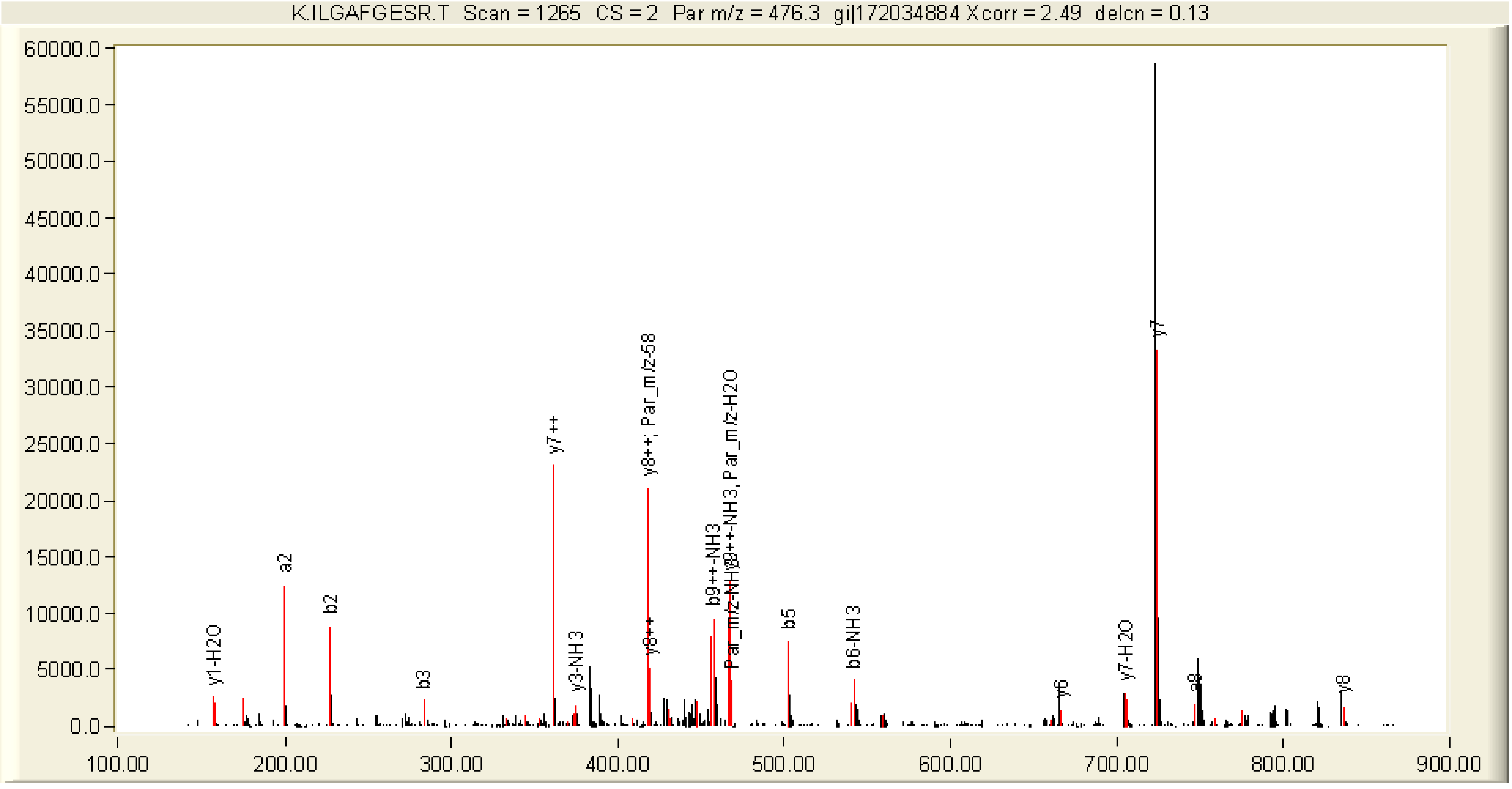

K.ILGAFGESR.T Scan = 1265 CS = 2 Par m/z = 476.3 gi|172034884 Xcorr = 2.49 delcn = 0.13
60000.0
55000.0
50000.0
45000.0
40000.0
35000.0
30000.0
25000.0
20000.0
15000.0
10000.0
5000.0
0.0
100.00
200.00
300.00
400.00
500.00
600.00
700.00
800.00
900.00
y1-H2O
a2
b2
b3
y7++
y3-NH3
y8++; Par_m/z-58
y8++
b9++-NH3
b9++-NH3, Par_m/z-H2O
b5
b6-NH3
y6
y7-H2O
y7
a8
y8

R.MLNYSLLTEAKLIEITQEATNKMTYR.E Scan = 1267 CS = 3 Par m/z = 1024.55 gi|172055114 Xcorr = 3.59 delcn = 0.17

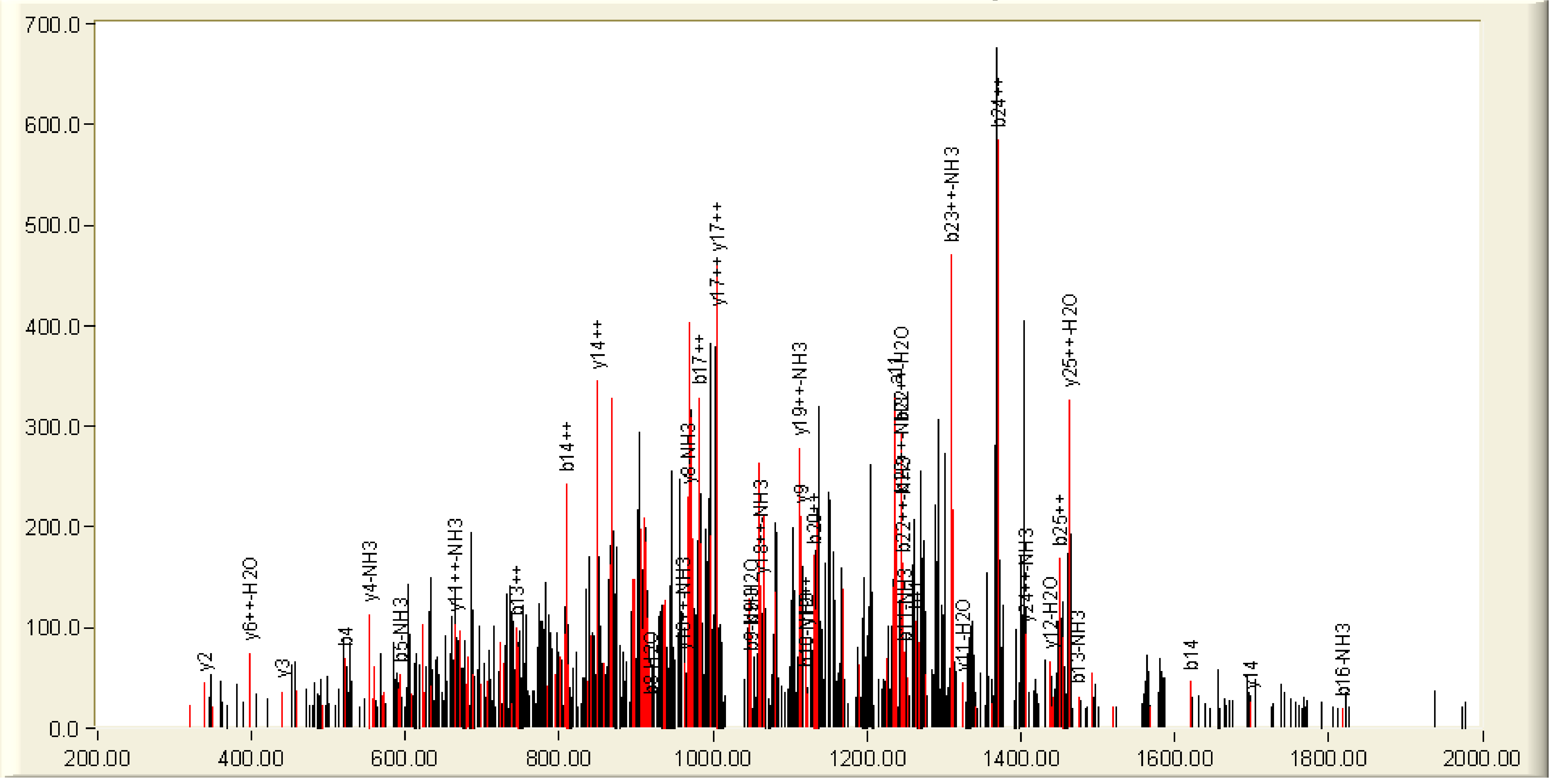

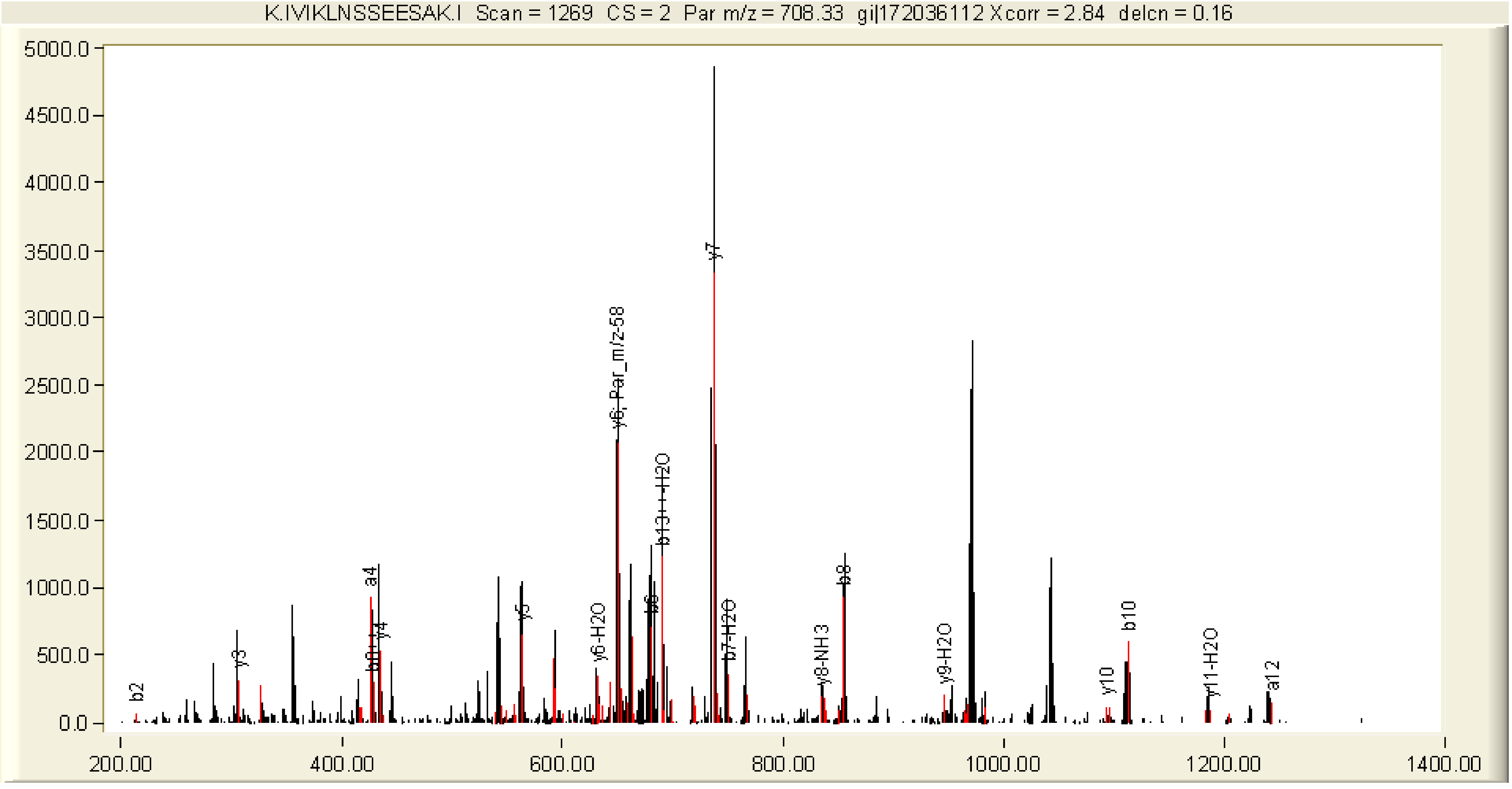
K.IVIKLNSSEESAK.I Scan = 1269 CS = 2 Par m/z = 708.33 gi|172036112 Xcorr = 2.84 delcn = 0.16
5000.0
4500.0
4000.0
3500.0
3000.0
2500.0
2000.0
1500.0
1000.0
500.0
0.0
200.00
400.00
600.00
800.00
1000.00
1200.00
1400.00
b2
y3
a4
y4
y5
y6-H2O
y6; Par_m/z-58
b6
b13++-H2O
y7
b7-H2O
y8-NH3
b8
y9-H2O
y10
b10
y11-H2O
a12

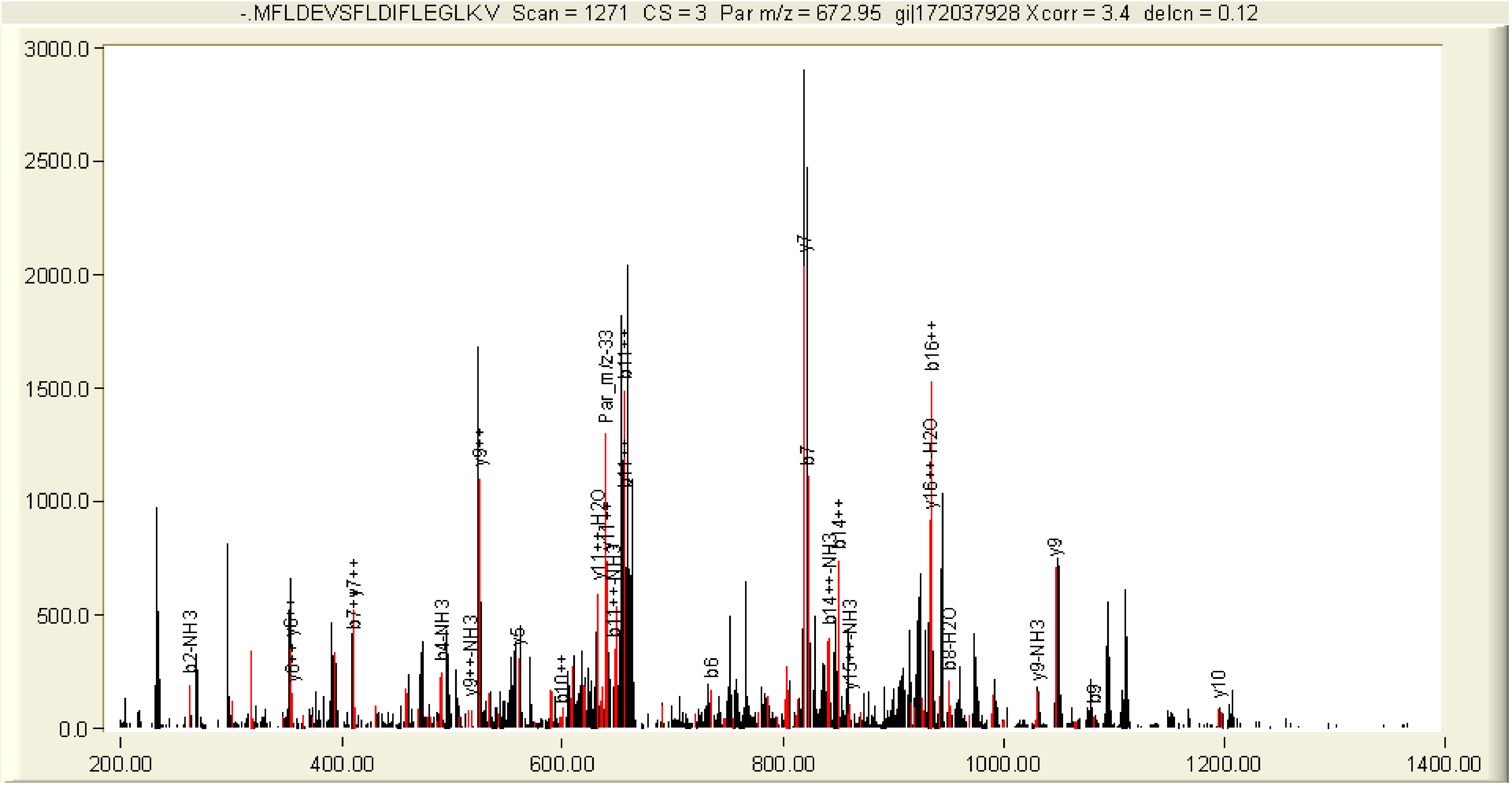
-.MFLDEVSFLDIFLEGLKV Scan = 1271 CS = 3 Par m/z = 672.95 gi|172037928 Xcorr = 3.4 delcn = 0.12
3000.0
2500.0
2000.0
1500.0
1000.0
500.0
0.0
200.00
400.00
600.00
800.00
1000.00
1200.00
1400.00
b2-NH3
y6++
b7+
y7++
b4-NH3
y9++-NH3
y9++
y5
b10++
Par_m/z-33
b11++
y7
b7
b14++-NH3
b14++
y15++-NH3
b16++
y16++-H2O
b8-H2O
b6
y9-NH3
y9
b9
y10

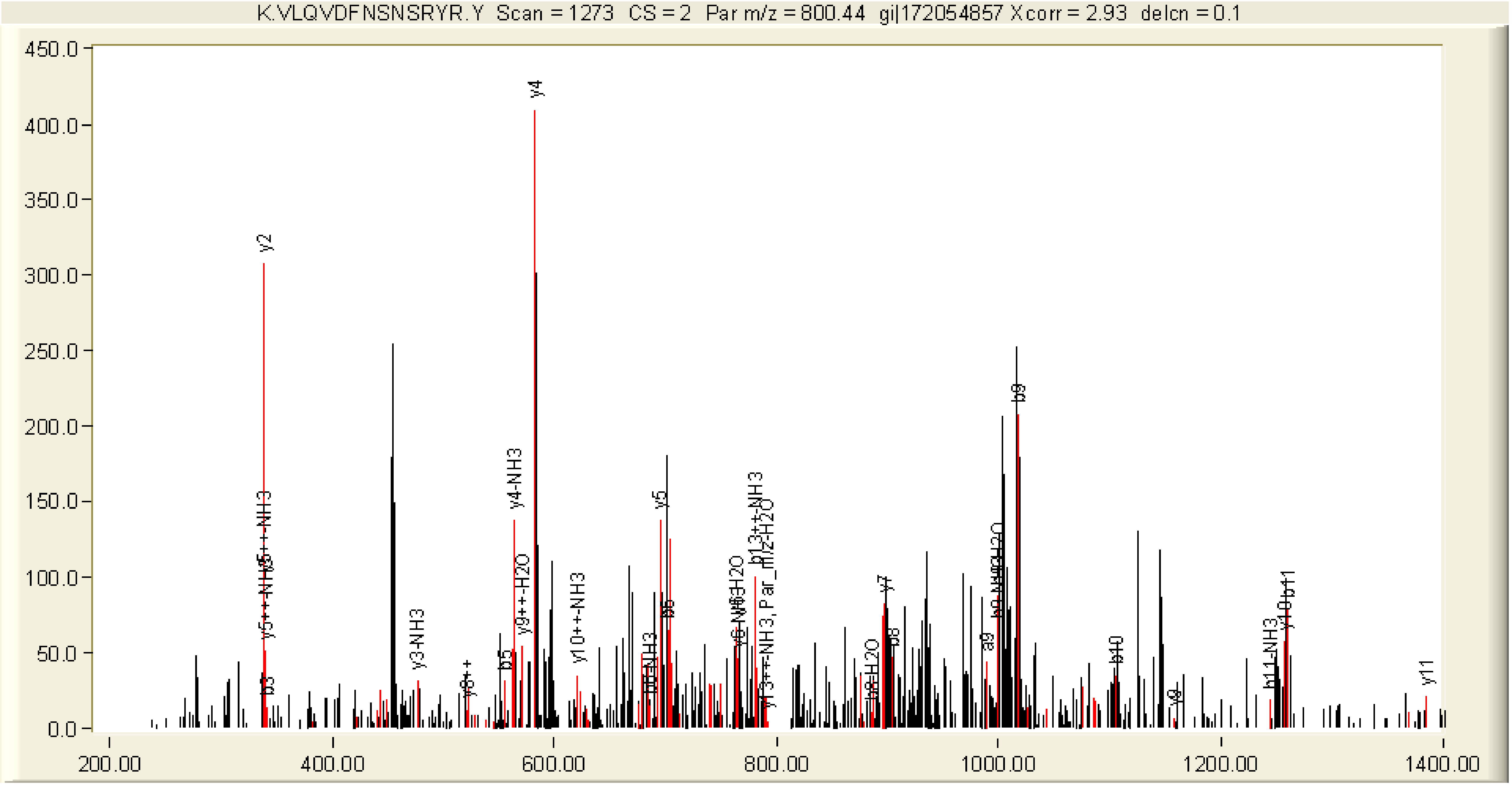

K.VLQVDFNSNSRYR.Y Scan = 1273 CS = 2 Par m/z = 800.44 gi|172054857 Xcorr = 2.93 delcn = 0.1
450.0
400.0
350.0
300.0
250.0
200.0
150.0
100.0
50.0
0.0
200.00
400.00
600.00
800.00
1000.00
1200.00
1400.00
y2
y4
y4-NH3
y5
b9
y3-NH3
y10++-NH3
y7
b8
a9
b10
b11-NH3
y11

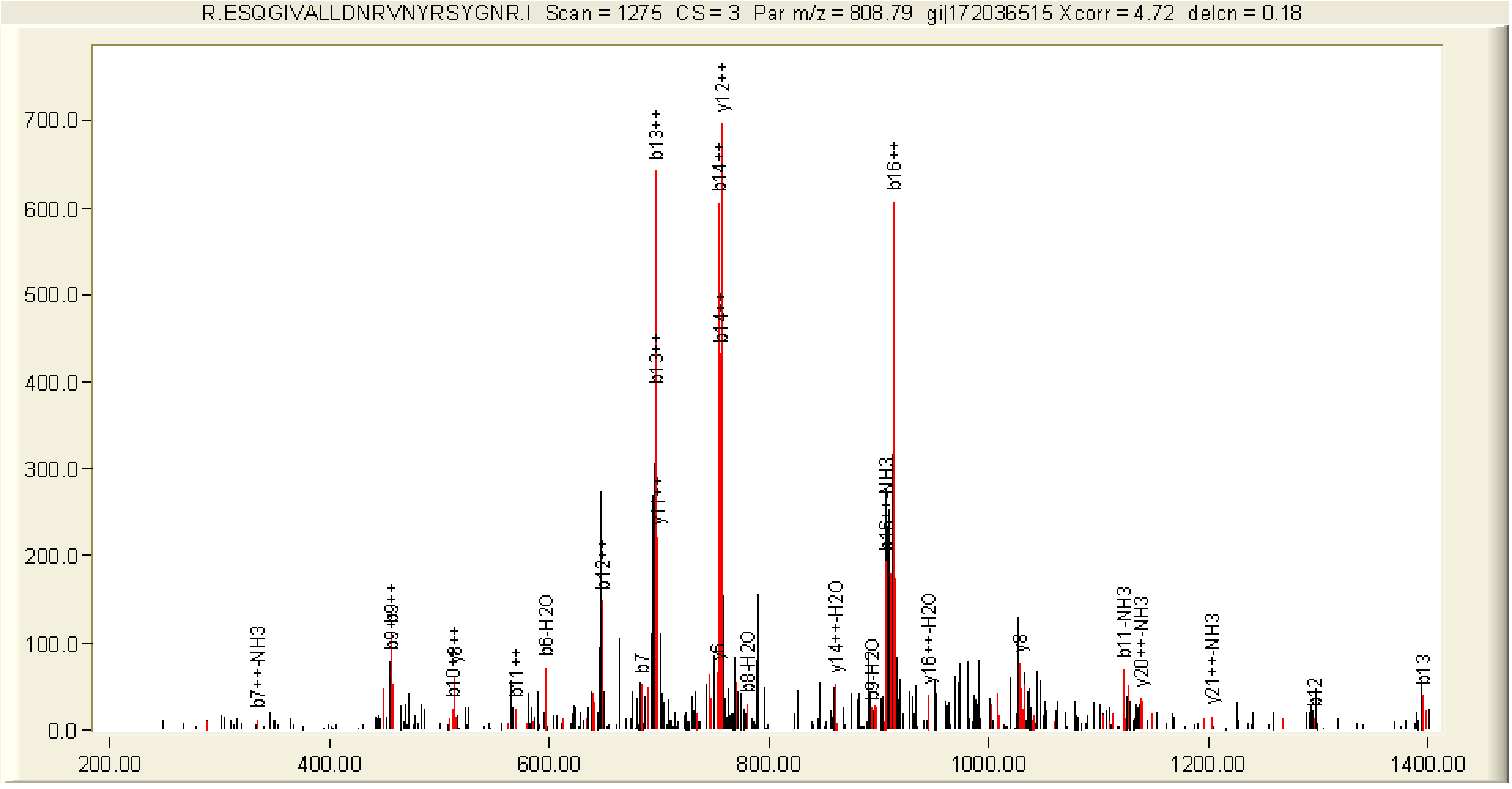

R.ESQGIVALLDNRVNYRSYGNR.I Scan = 1275 CS = 3 Par m/z = 808.79 gi|172036515 Xcorr = 4.72 delcn = 0.18
700.0
600.0
500.0
400.0
300.0
200.0
100.0
0.0
200.00
400.00
600.00
800.00
1000.00
1200.00
1400.00
y12++
b13++
b14++
b16++
y11++
b12++
b16++-NH3
b7++-NH3
b9++
b10++
b11++
b6-H2O
b7
y6
b8-H2O
y14++-H2O
b9-H2O
y16++-H2O
y8
b11-NH3
y20++-NH3
y21++-NH3
b12
b13

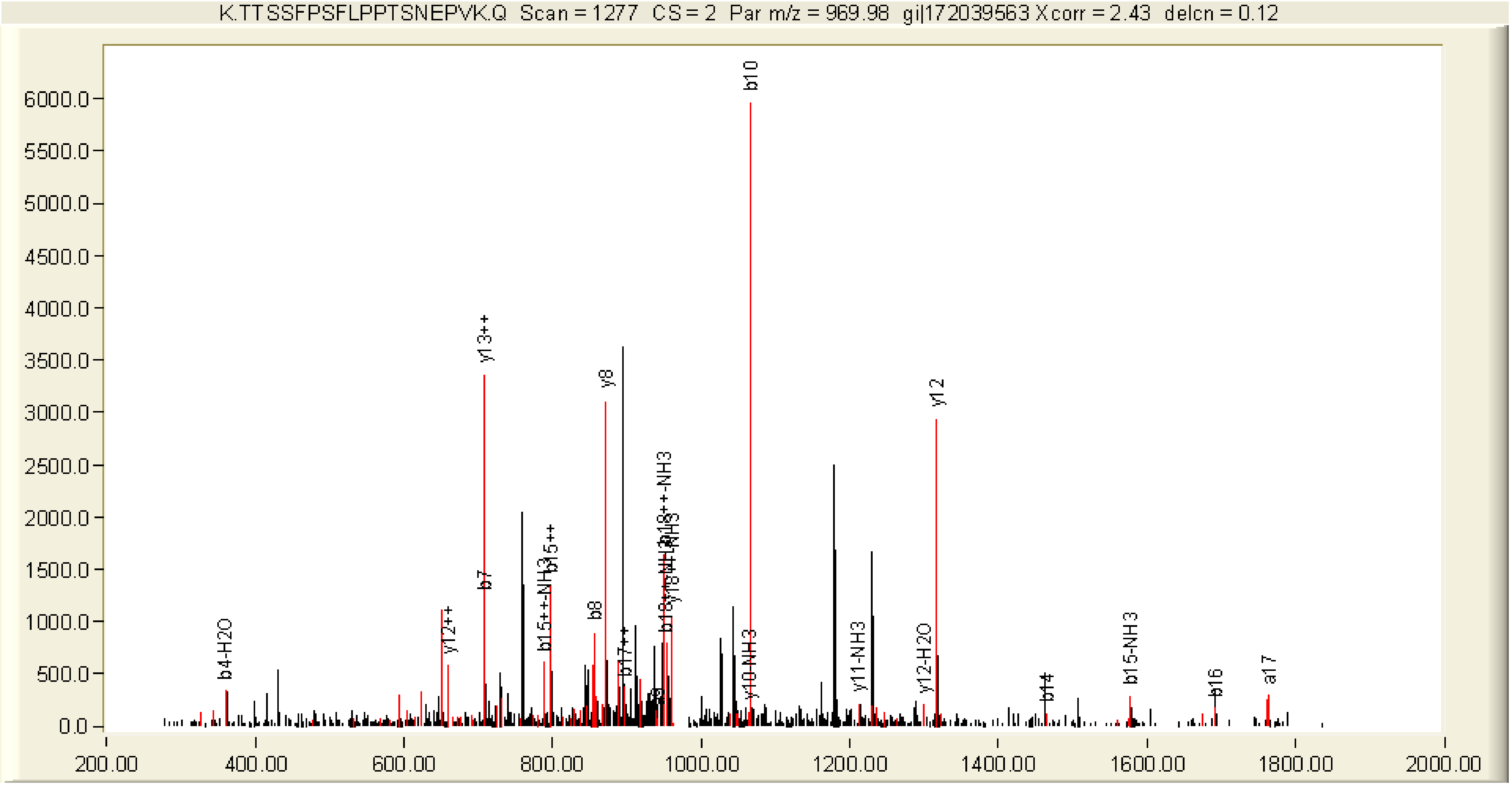
K.TTSSFPSFLPPTSNEPVK.Q Scan = 1277 CS = 2 Par m/z = 969.98 gi|172039563 Xcorr = 2.43 delcn = 0.12
6000.0
5500.0
5000.0
4500.0
4000.0
3500.0
3000.0
2500.0
2000.0
1500.0
1000.0
500.0
0.0
200.00
400.00
600.00
800.00
1000.00
1200.00
1400.00
1600.00
1800.00
2000.00
b10
y13++
y8
y12
b7
b15++
b15++-NH3
b8
y12++
b4-H2O
y10-NH3
y11-NH3
y12-H2O
b14
b15-NH3
b16
a17

K.EGSYAMGATRLQTALK.V Scan = 1279 CS = 2 Par m/z = 848.52 gi|172036079 Xcorr = 2.43 delcn = 0.13

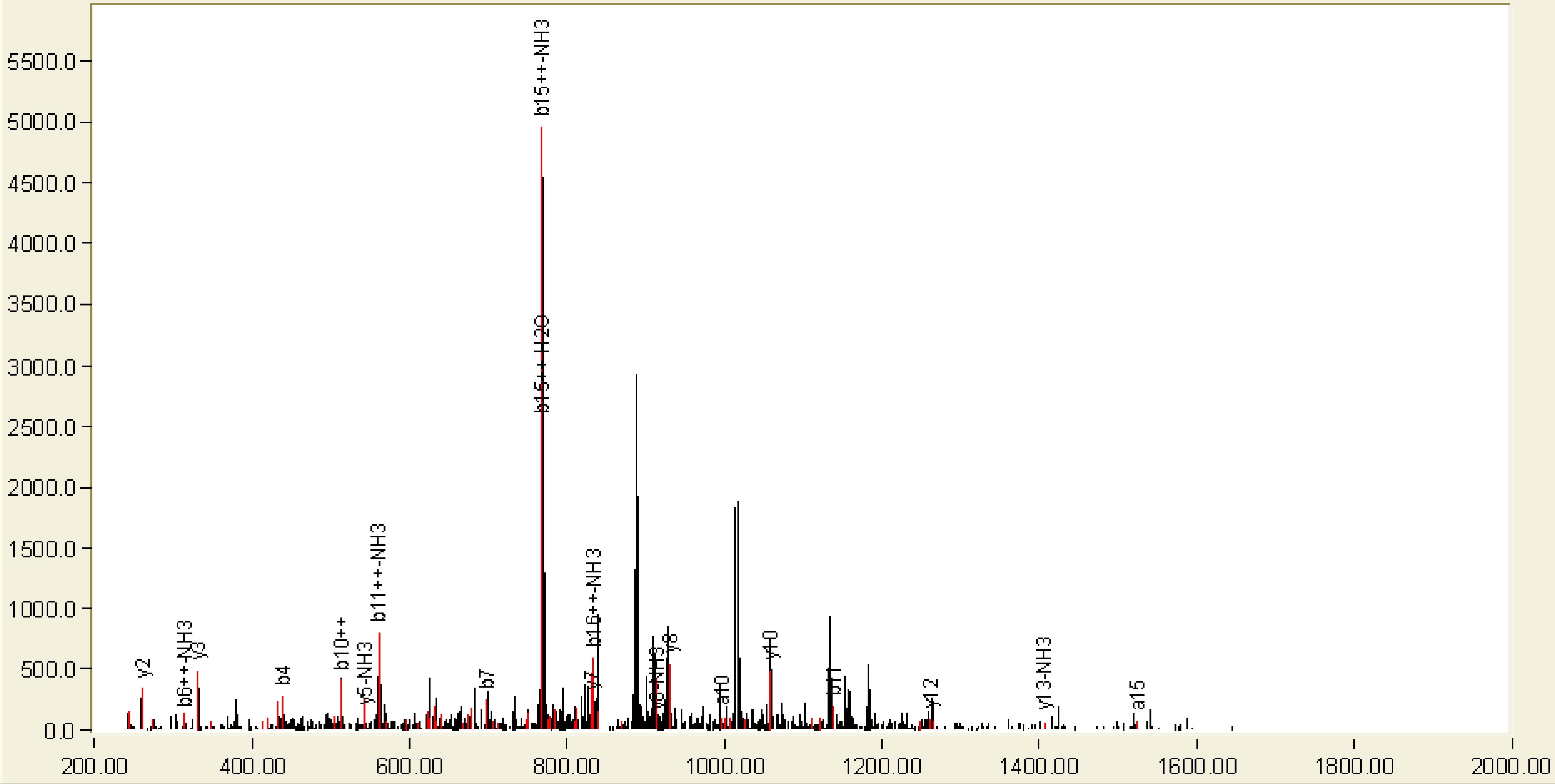

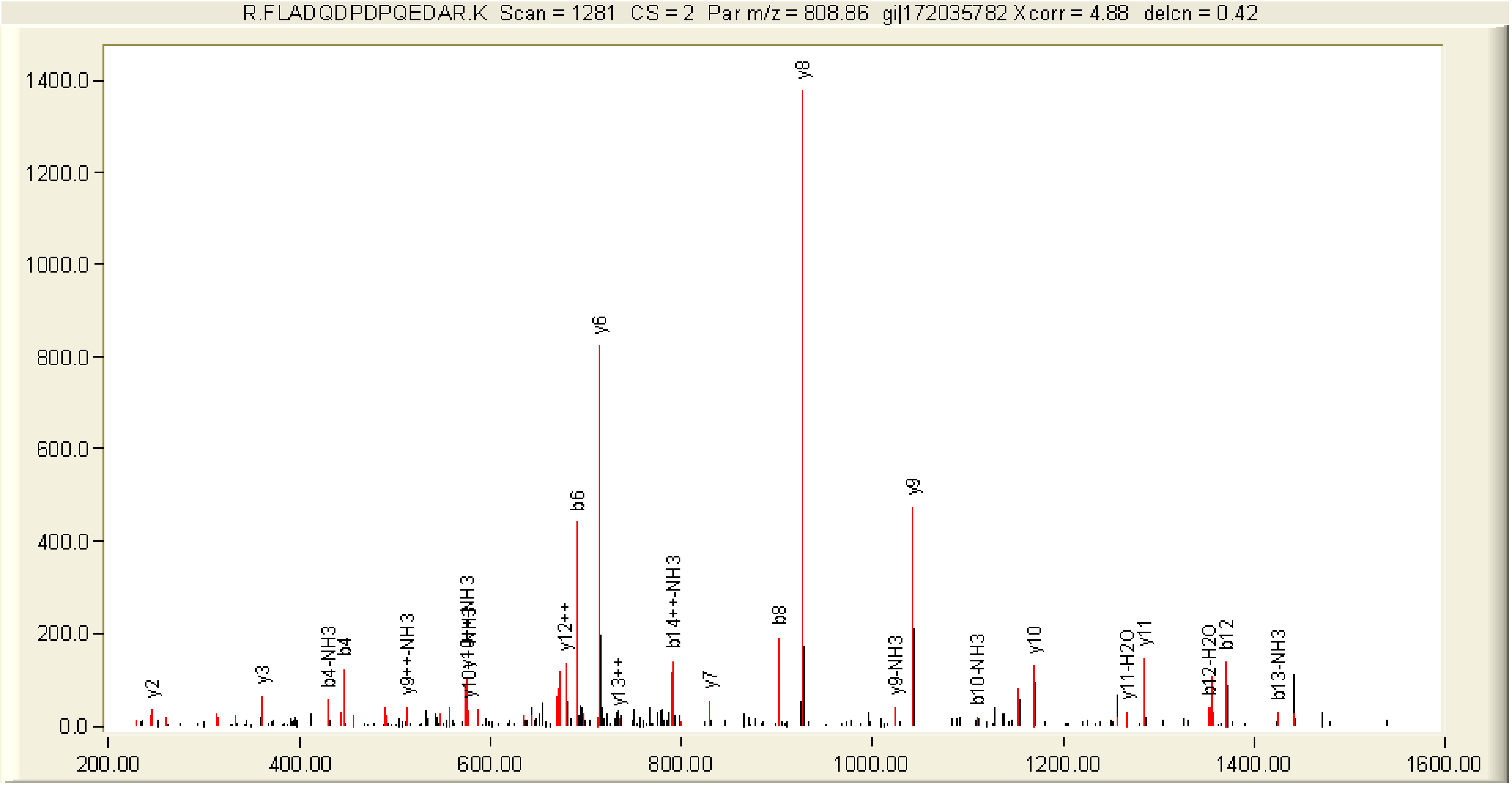
R.FLADQDPDPQEDAR.K Scan = 1281 CS = 2 Par m/z = 808.86 gi|172035782 Xcorr = 4.88 delcn = 0.42
1400.0
1200.0
1000.0
800.0
600.0
400.0
200.0
0.0
200.00
400.00
600.00
800.00
1000.00
1200.00
1400.00
1600.00
y2
y3
b4-NH3
b4
y9++-NH3
y12++
b6
y6
y13++
b14++-NH3
y7
b8
y8
y9-NH3
y9
b10-NH3
y10
y11-H2O
y11
b12-H2O
b12
b13-NH3

K.VFSESNSDGYTFISAED.K Scan = 1283 CS = 3 Par m/z = 623.65 gi|172039444 Xcorr = 4.88 delcn = 0.2

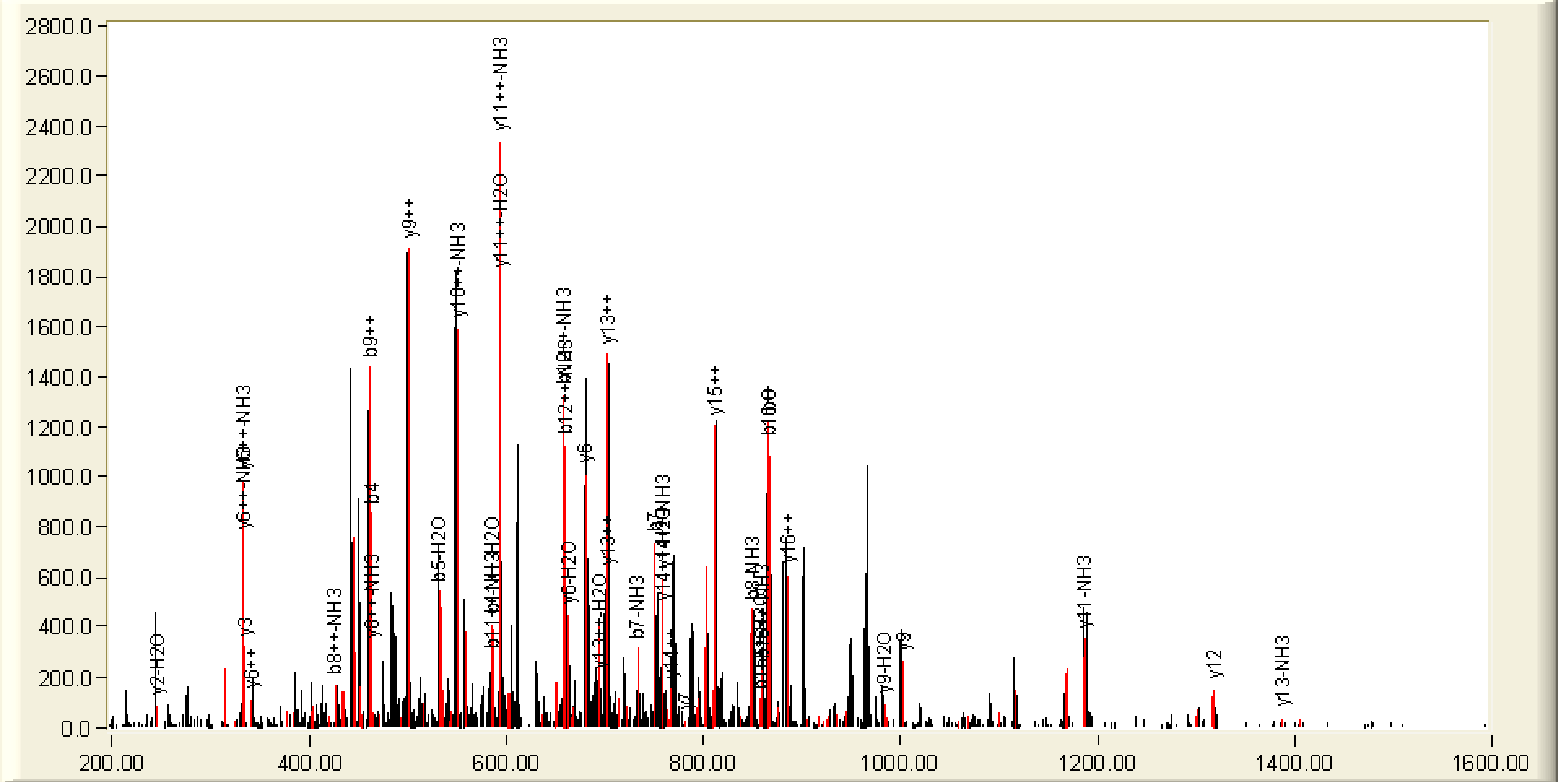

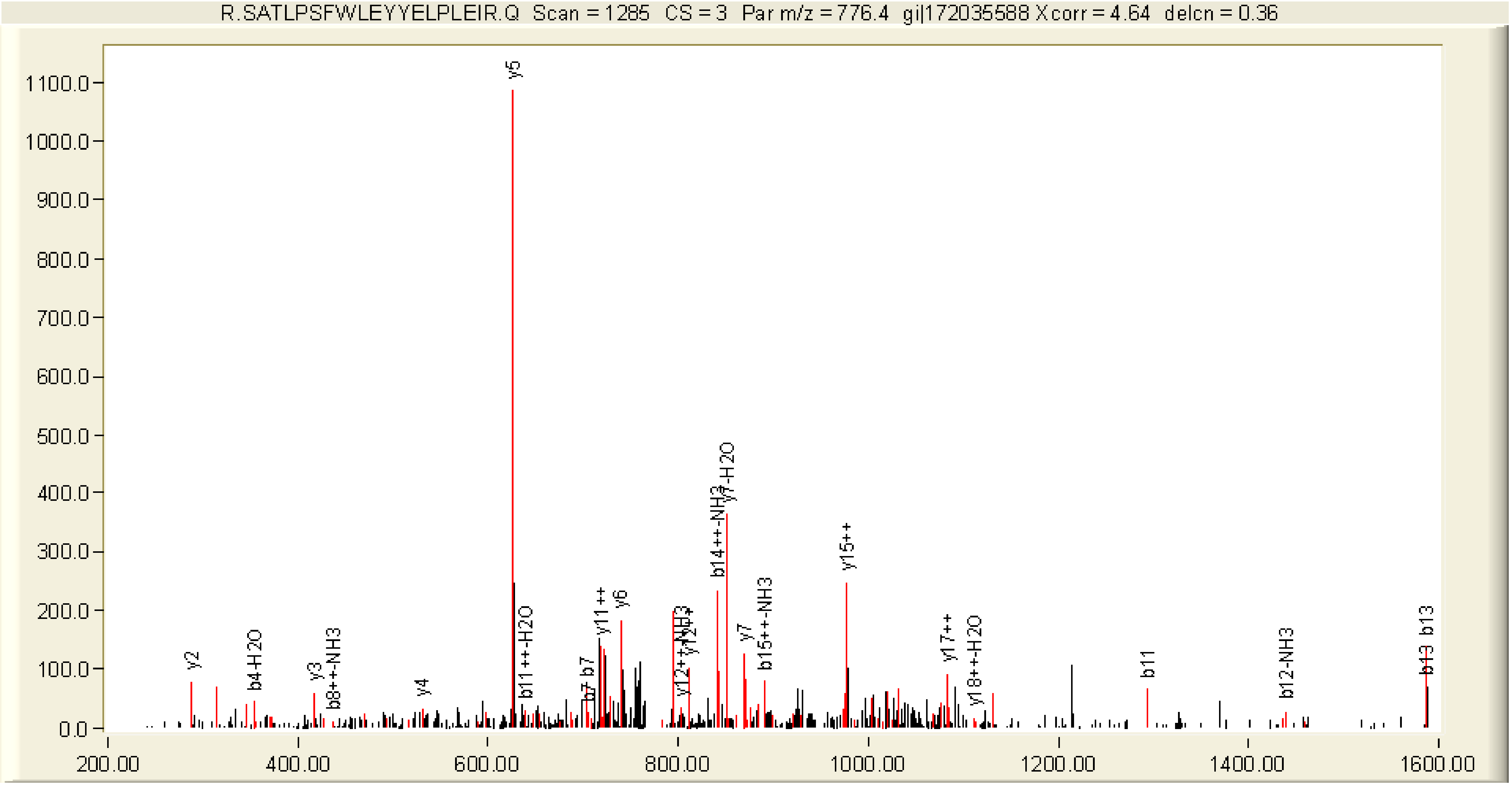
R.SATLPSFWLEYYELPLEIR.Q Scan = 1285 CS = 3 Par m/z = 776.4 gi|172035588 Xcorr = 4.64 delcn = 0.36
y5
y7-H2O
b14++-NH3
y15++
b15++-NH3
y7
y6
y11++
y12++-NH3
y12++
b11++-H2O
b7 b7
y2
b4-H2O
y3
b8++-NH3
y4
y17++
y18++-H2O
b11
b12-NH3
b13 b13
0.0
100.0
200.0
300.0
400.0
500.0
600.0
700.0
800.0
900.0
1000.0
1100.0
200.00
400.00
600.00
800.00
1000.00
1200.00
1400.00
1600.00

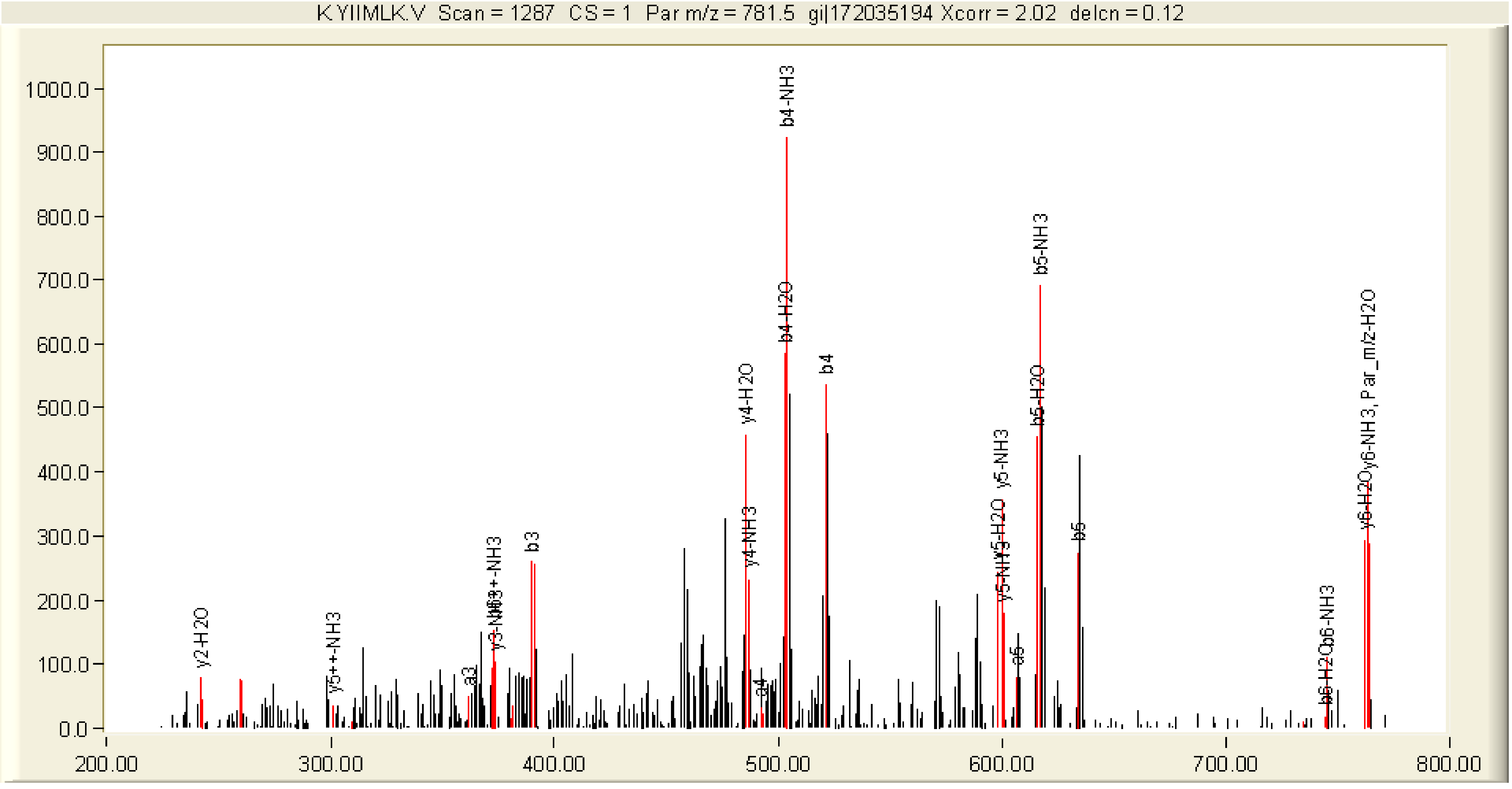

K.YIIMLK.V Scan = 1287 CS = 1 Par m/z = 781.5 gi|172035194 Xcorr = 2.02 delcn = 0.12
1000.0
900.0
800.0
700.0
600.0
500.0
400.0
300.0
200.0
100.0
0.0
200.00
300.00
400.00
500.00
600.00
700.00
800.00
y2-H2O
y5++-NH3
a3
b3
y4-H2O
y4-NH3
a4
b4-NH3
b4-H2O
b4
y5-H2O
y5-NH3
a5
b5-NH3
b5-H2O
b5
b6-H2O
b6-NH3
y6-H2O
y6-NH3, Par_m/z-H2O

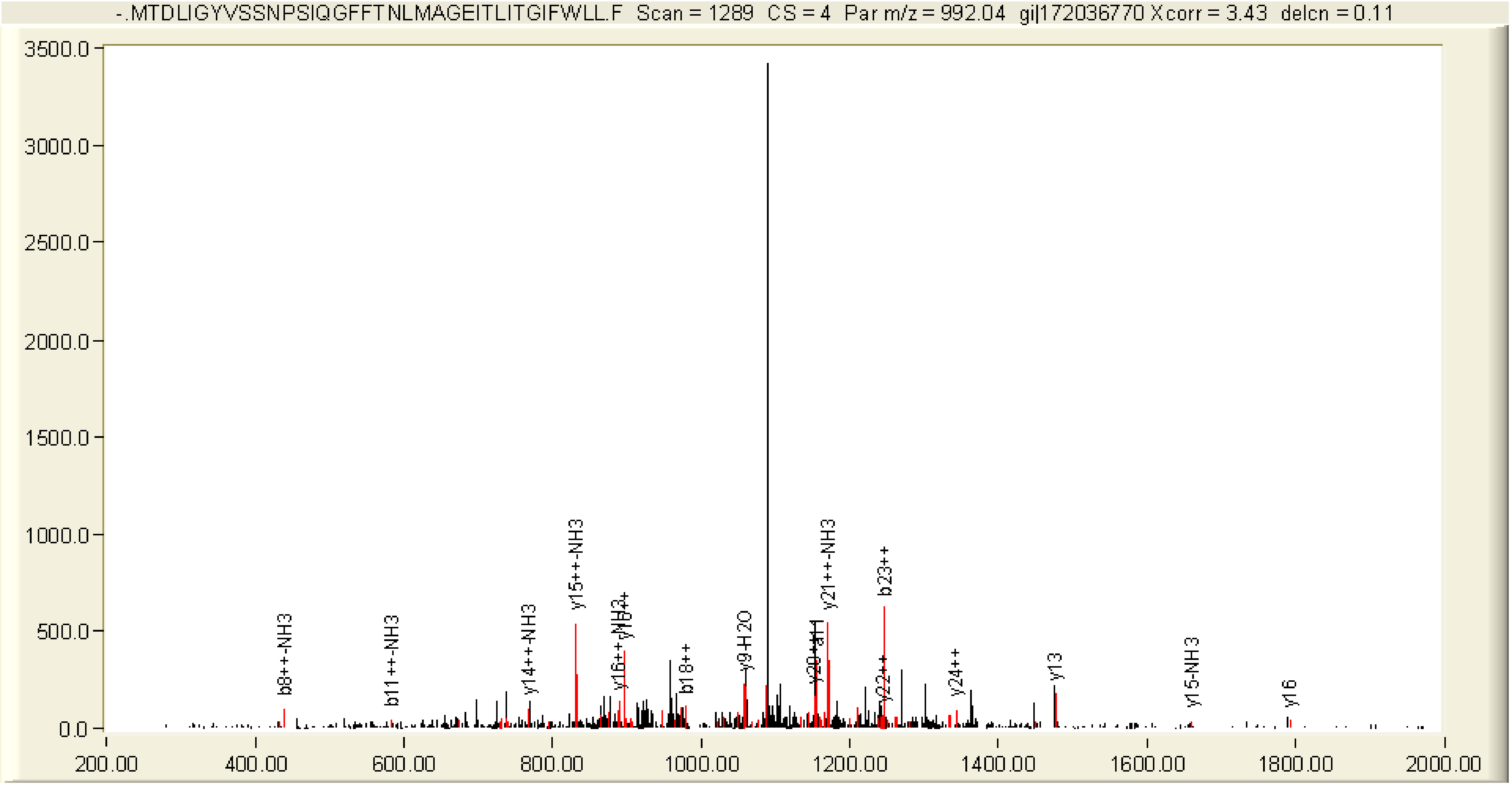
-.MTDLIGYVSSNPSIQGFFTNLMAGEITLITGIFWLL.F Scan = 1289 CS = 4 Par m/z = 992.04 gi|172036770 Xcorr = 3.43 delcn = 0.11
3500.0
3000.0
2500.0
2000.0
1500.0
1000.0
500.0
0.0
200.00
400.00
600.00
800.00
1000.00
1200.00
1400.00
1600.00
1800.00
2000.00
b8++-NH3
b11++-NH3
y14++-NH3
y15++-NH3
y16++
b18++
y9-H2O
y21++-NH3
b23++
y22++
y24++
y13
y15-NH3
y16

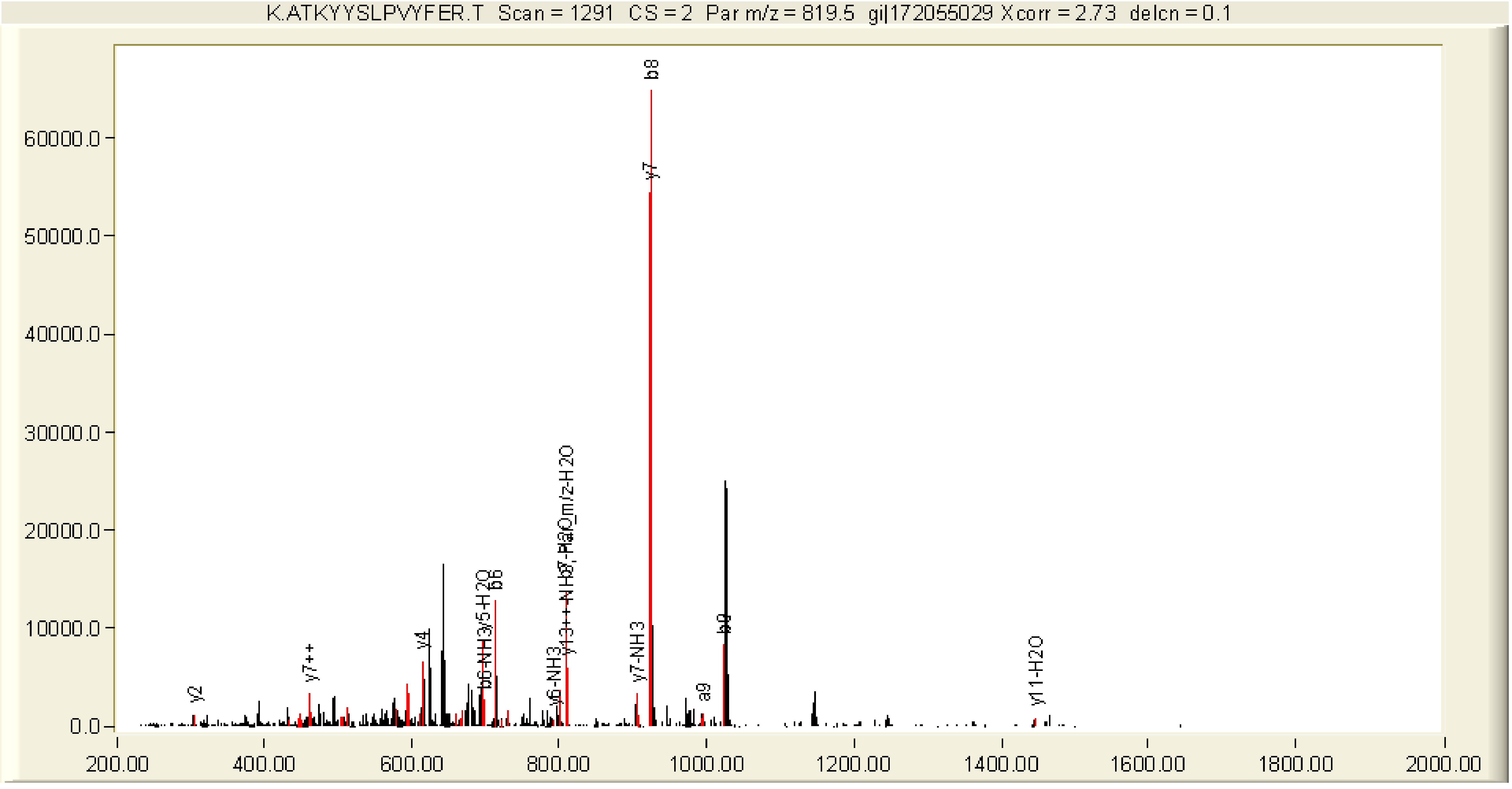

K.ATKYYSLPVYFER.T Scan = 1291 CS = 2 Par m/z = 819.5 gi|172055029 Xcorr = 2.73 delcn = 0.1
b8
y7
b6
b6-NH3/y5-H2O
y4
y7++
y2
y6-NH3
y7-NH3
a9
b9
y11-H2O
60000.0
50000.0
40000.0
30000.0
20000.0
10000.0
0.0
200.00
400.00
600.00
800.00
1000.00
1200.00
1400.00
1600.00
1800.00
2000.00

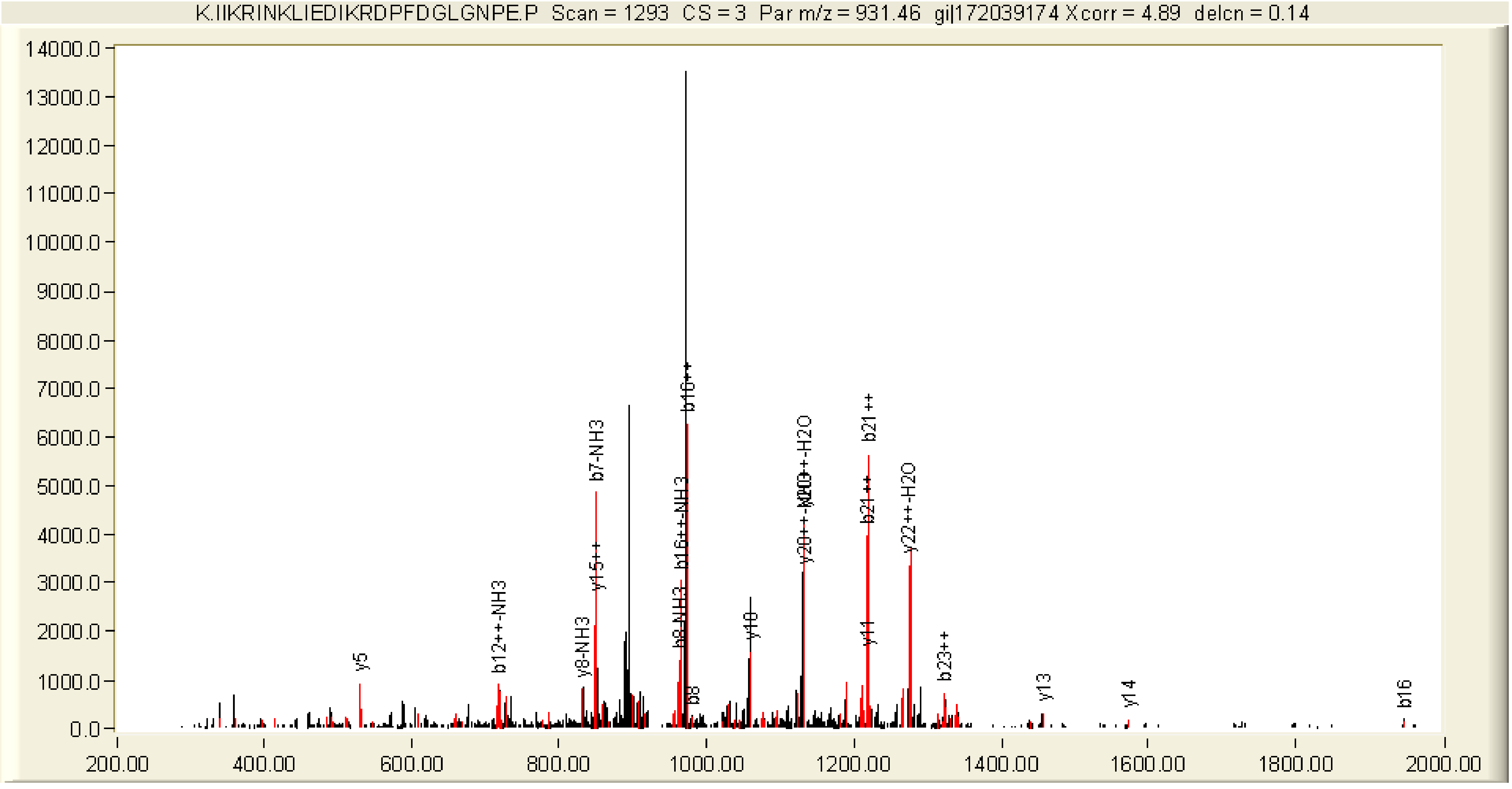
K.IIKRINKLIEDIKRDPFDGLGNPE.P Scan = 1293 CS = 3 Par m/z = 931.46 gi|172039174 Xcorr = 4.89 delcn = 0.14
14000.0
13000.0
12000.0
11000.0
10000.0
9000.0
8000.0
7000.0
6000.0
5000.0
4000.0
3000.0
2000.0
1000.0
0.0
200.00
400.00
600.00
800.00
1000.00
1200.00
1400.00
1600.00
1800.00
2000.00
y5
b12++-NH3
y8-NH3
y15++
b7-NH3
b8-NH3
b16++-NH3
b8
b16++
y10
y20++-H2O
y11
b21++
y22++-H2O
b23++
y13
y14
b16

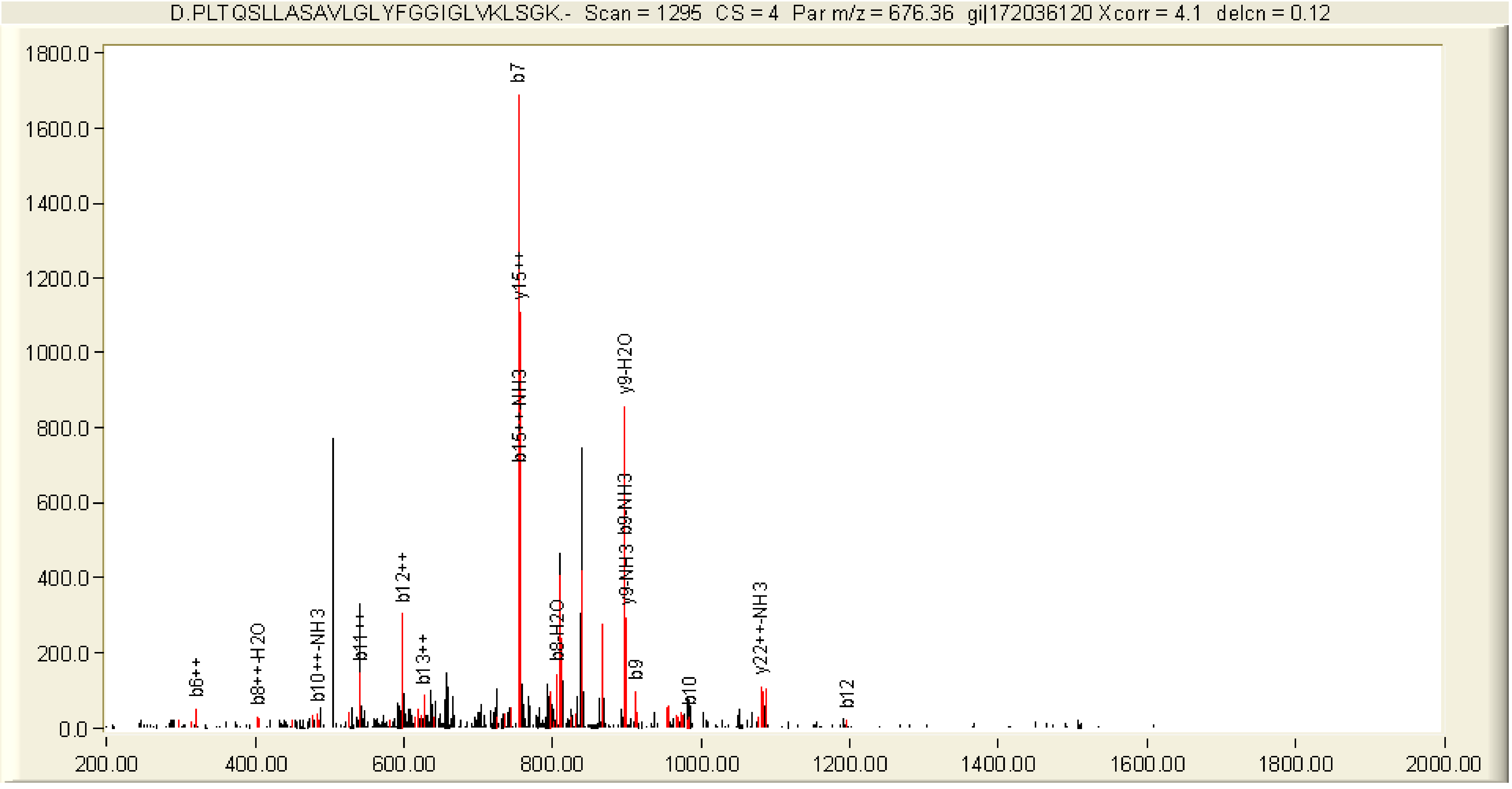

D.PLTQSLLASAVLGLYFGGIGLVKLSGK.- Scan = 1295 CS = 4 Par m/z = 676.36 gi|172036120 Xcorr = 4.1 delcn = 0.12
b7
y15++
b15++-NH3
y9-H2O
y9-NH3
b9-NH3
b12++
b11++
b13++
b8-H2O
b10++-NH3
b8++-H2O
b6++
b9
b10
y22++-NH3
b12
1800.0
1600.0
1400.0
1200.0
1000.0
800.0
600.0
400.0
200.0
0.0
200.00
400.00
600.00
800.00
1000.00
1200.00
1400.00
1600.00
1800.00
2000.00

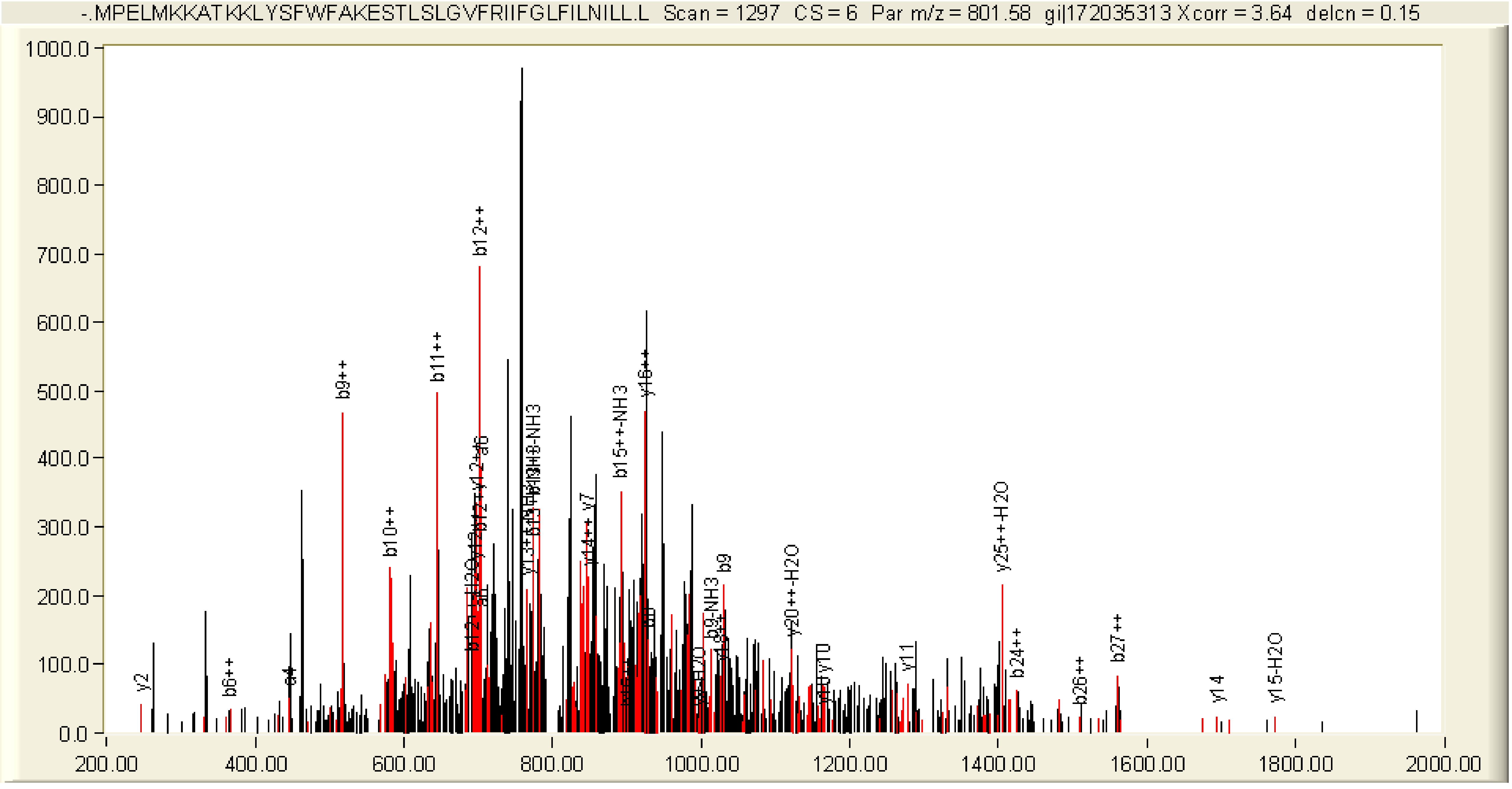
-.MPELMKKATKKLYSFWFAKESTLSLGVFRIIFGLFILNILL.L Scan = 1297 CS = 6 Par m/z = 801.58 gi|172035313 Xcorr = 3.64 delcn = 0.15
1000.0
900.0
800.0
700.0
600.0
500.0
400.0
300.0
200.0
100.0
0.0
200.00
400.00
600.00
800.00
1000.00
1200.00
1400.00
1600.00
1800.00
2000.00
y2
b6++
b9++
b10++
b11++
b12++
b15++-NH3
y16++
y7
b9
b9-NH3
y20++-H2O
y11
y25++-H2O
b24++
b26++
b27++
y14
y15-H2O

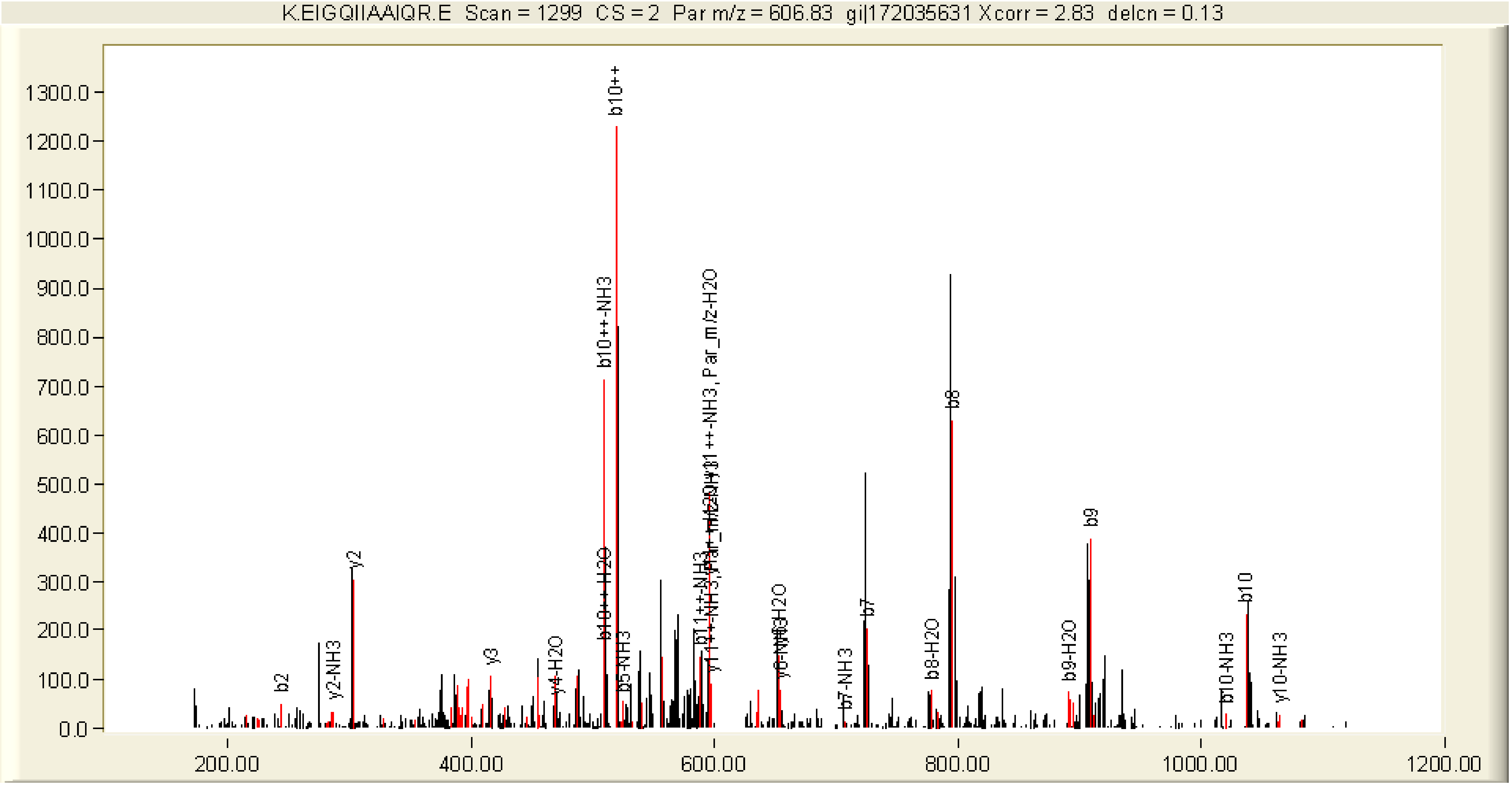
K.EIGQIIAAIQR.E Scan = 1299 CS = 2 Par m/z = 606.83 gi|172035631 Xcorr = 2.83 delcn = 0.13
1300.0
1200.0
1100.0
1000.0
900.0
800.0
700.0
600.0
500.0
400.0
300.0
200.0
100.0
0.0
200.00
400.00
600.00
800.00
1000.00
1200.00
b2
y2-NH3
y2
y3
y4-H2O
b10++-H2O
b10++-NH3
b10++
b5-NH3
b8
b7-NH3
b7
b8-H2O
b9-H2O
b9
b10-NH3
b10
y10-NH3

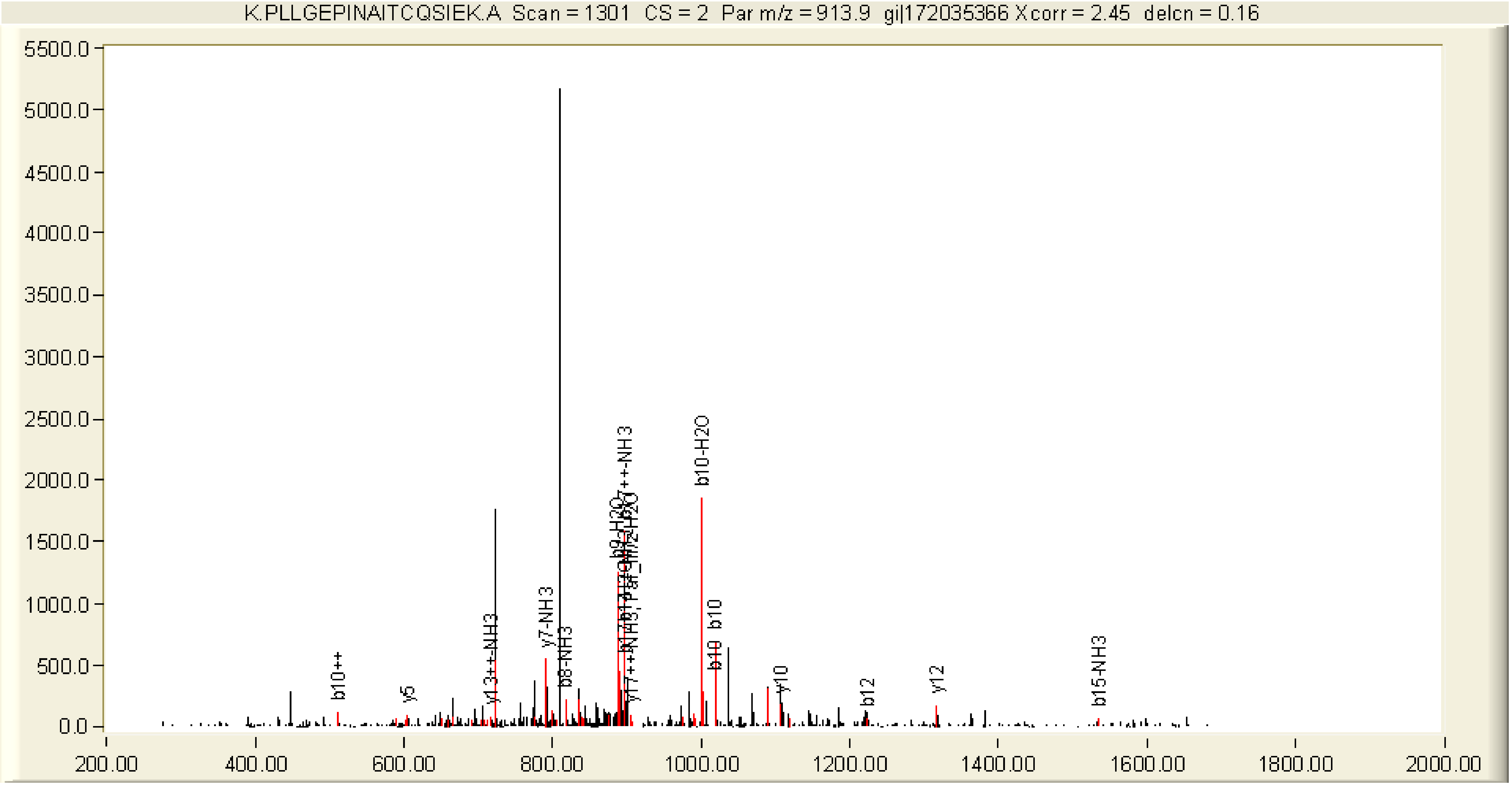
K.PLLGEPINAITCQSIEK.A Scan = 1301 CS = 2 Par m/z = 913.9 gi|172035366 Xcorr = 2.45 delcn = 0.16
b10++
y5
y13++-NH3
y7-NH3
b8-NH3
b10-H2O
b10
y10
b12
y12
b15-NH3

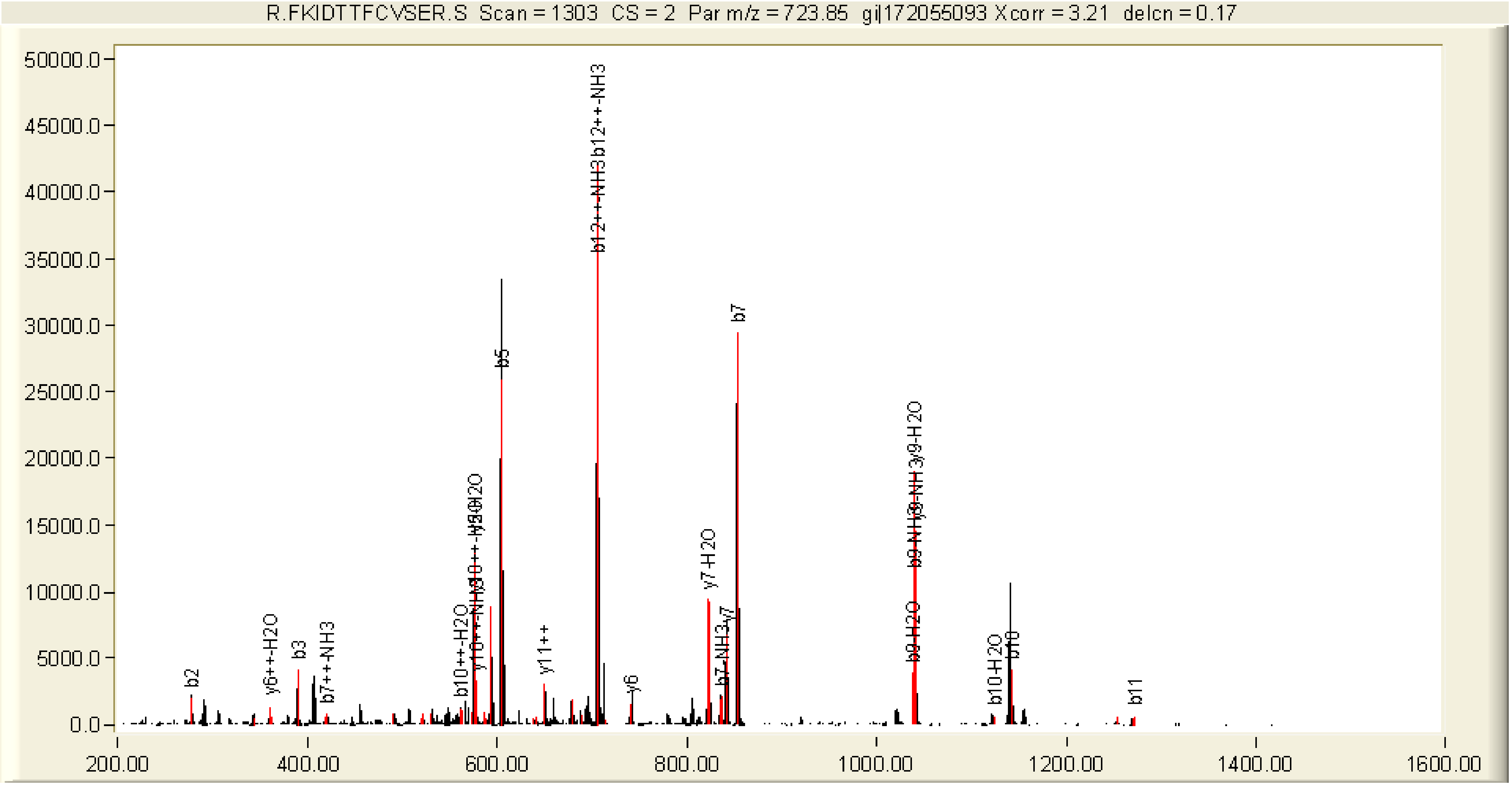
R.FKIDTTFCVSER.S Scan = 1303 CS = 2 Par m/z = 723.85 gi|172055093 Xcorr = 3.21 delcn = 0.17
50000.0
45000.0
40000.0
35000.0
30000.0
25000.0
20000.0
15000.0
10000.0
5000.0
0.0
200.00
400.00
600.00
800.00
1000.00
1200.00
1400.00
1600.00
b2
y6++-H2O
b3
b7++-NH3
b10++-H2O
y11++
b5
b12++-NH3b12++-NH3
y6
y7-H2O
b7-NH3
y7
b7
b9-H2O
b10-H2O
b10
b11

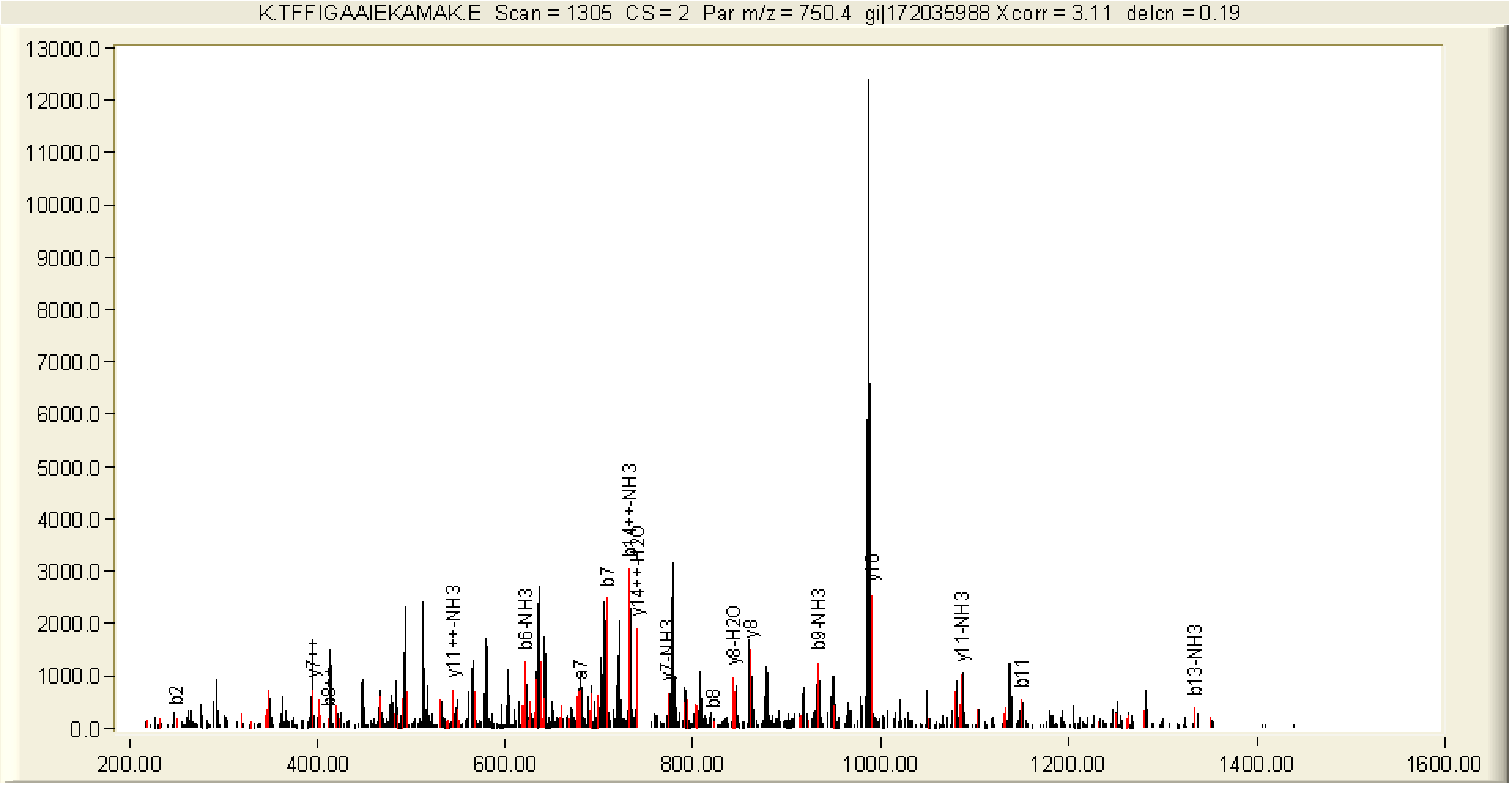

K.TFFIGAAIEKAMAK.E Scan = 1305 CS = 2 Par m/z = 750.4 gi|172035988 Xcorr = 3.11 delcn = 0.19
13000.0
12000.0
11000.0
10000.0
9000.0
8000.0
7000.0
6000.0
5000.0
4000.0
3000.0
2000.0
1000.0
0.0
200.00
400.00
600.00
800.00
1000.00
1200.00
1400.00
1600.00
b2
y7++
y11++-NH3
b6-NH3
a7
b7
y14++-NH3
y14++-H2O
y7-NH3
b8
y8-H2O
y8
b9-NH3
y10
y11-NH3
b11
b13-NH3

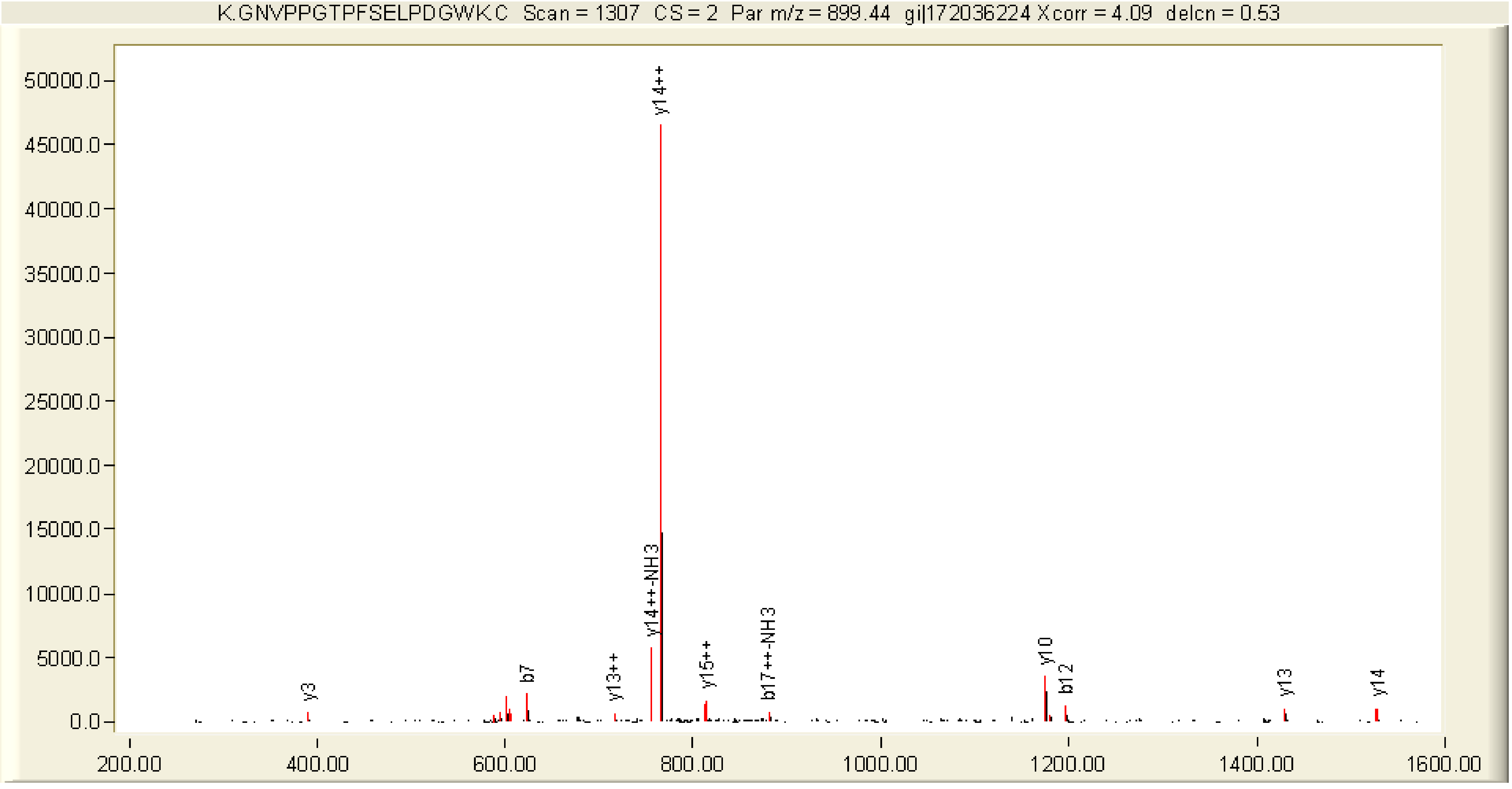

K.GNVPPGTPFSELPDGWK.C Scan = 1307 CS = 2 Par m/z = 899.44 gi|172036224 Xcorr = 4.09 delcn = 0.53
50000.0
45000.0
40000.0
35000.0
30000.0
25000.0
20000.0
15000.0
10000.0
5000.0
0.0
y14++
y14++-NH3
y3
b7
y13++
y15++
b17++-NH3
y10
b12
y13
y14
200.00
400.00
600.00
800.00
1000.00
1200.00
1400.00
1600.00

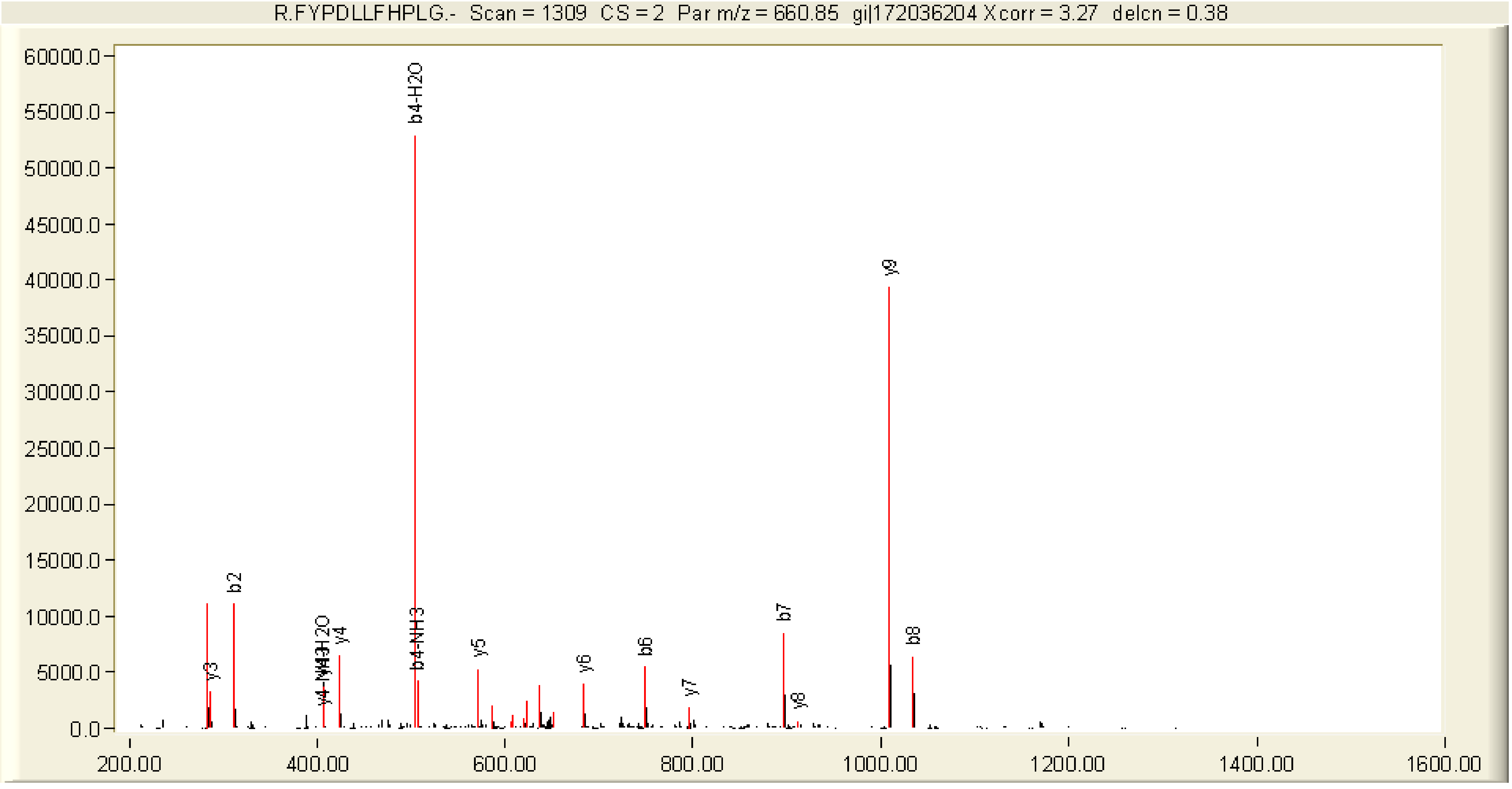

R.FYPDLLFHPLG.- Scan = 1309 CS = 2 Par m/z = 660.85 gi|172036204 Xcorr = 3.27 delcn = 0.38
60000.0
55000.0
50000.0
45000.0
40000.0
35000.0
30000.0
25000.0
20000.0
15000.0
10000.0
5000.0
0.0
200.00
400.00
600.00
800.00
1000.00
1200.00
1400.00
1600.00
b4-H2O
y9
b2
y3
y4
b4-NH3
y5
y6
b6
y7
b7
y8
b8

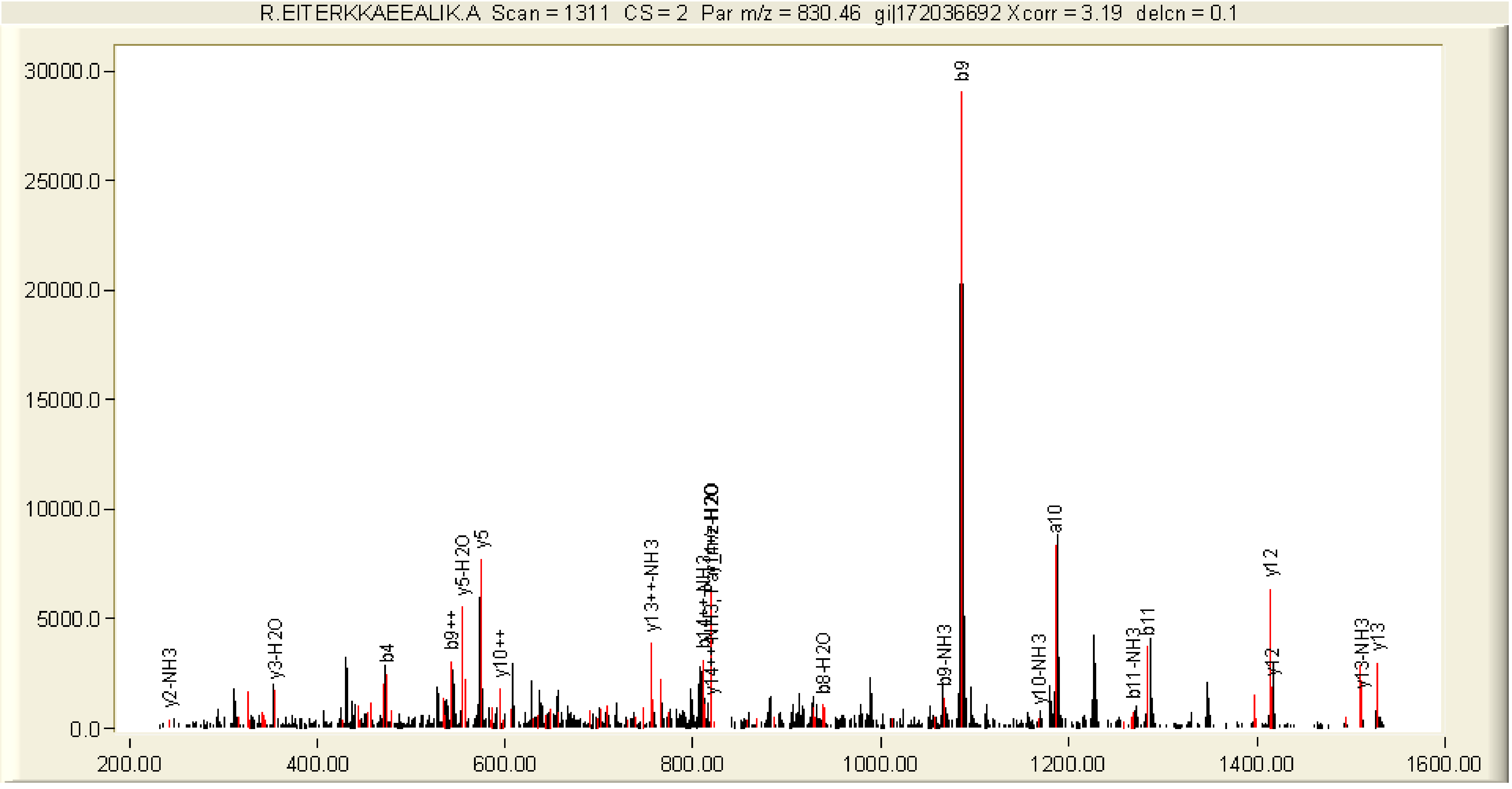
R.EITERKKAEEALIK.A Scan = 1311 CS = 2 Par m/z = 830.46 gi|172036692 Xcorr = 3.19 delcn = 0.1
30000.0
25000.0
20000.0
15000.0
10000.0
5000.0
0.0
200.00
400.00
600.00
800.00
1000.00
1200.00
1400.00
1600.00
y2-NH3
y3-H2O
b4
b9++
y5-H2O
y5
y10++
y13++-NH3
b8-H2O
b9-NH3
b9
y10-NH3
a10
b11-NH3
b11
y12
y13-NH3
y13

V.QKHKSKISLIQQVISGNTPPMPKFQPSPQDMADLLSYLEKL.- Scan = 1313 CS = 6 Par m/z = 773.73 gi|172038177 Xcorr = 3.71 delcn = 0.17

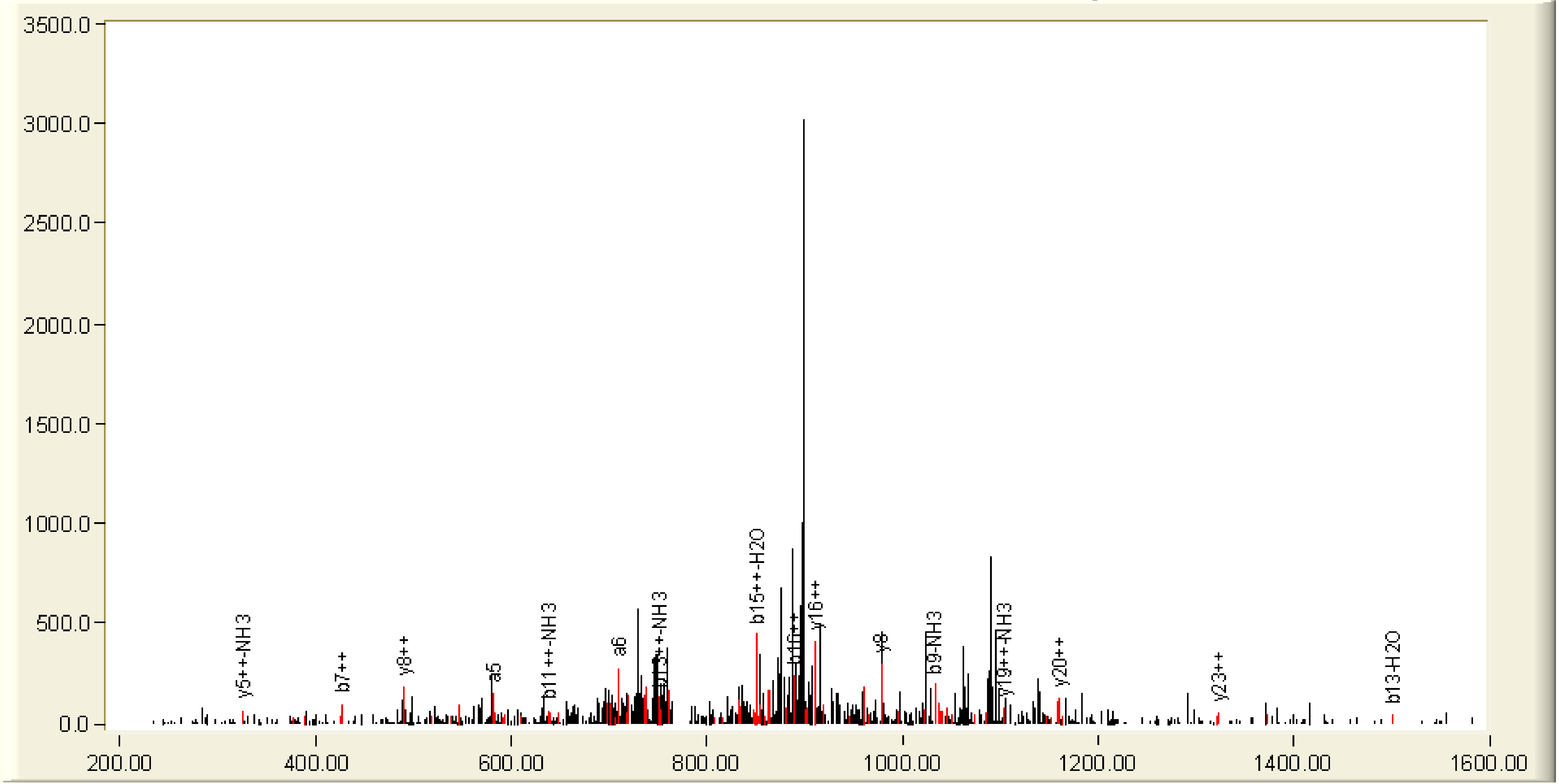

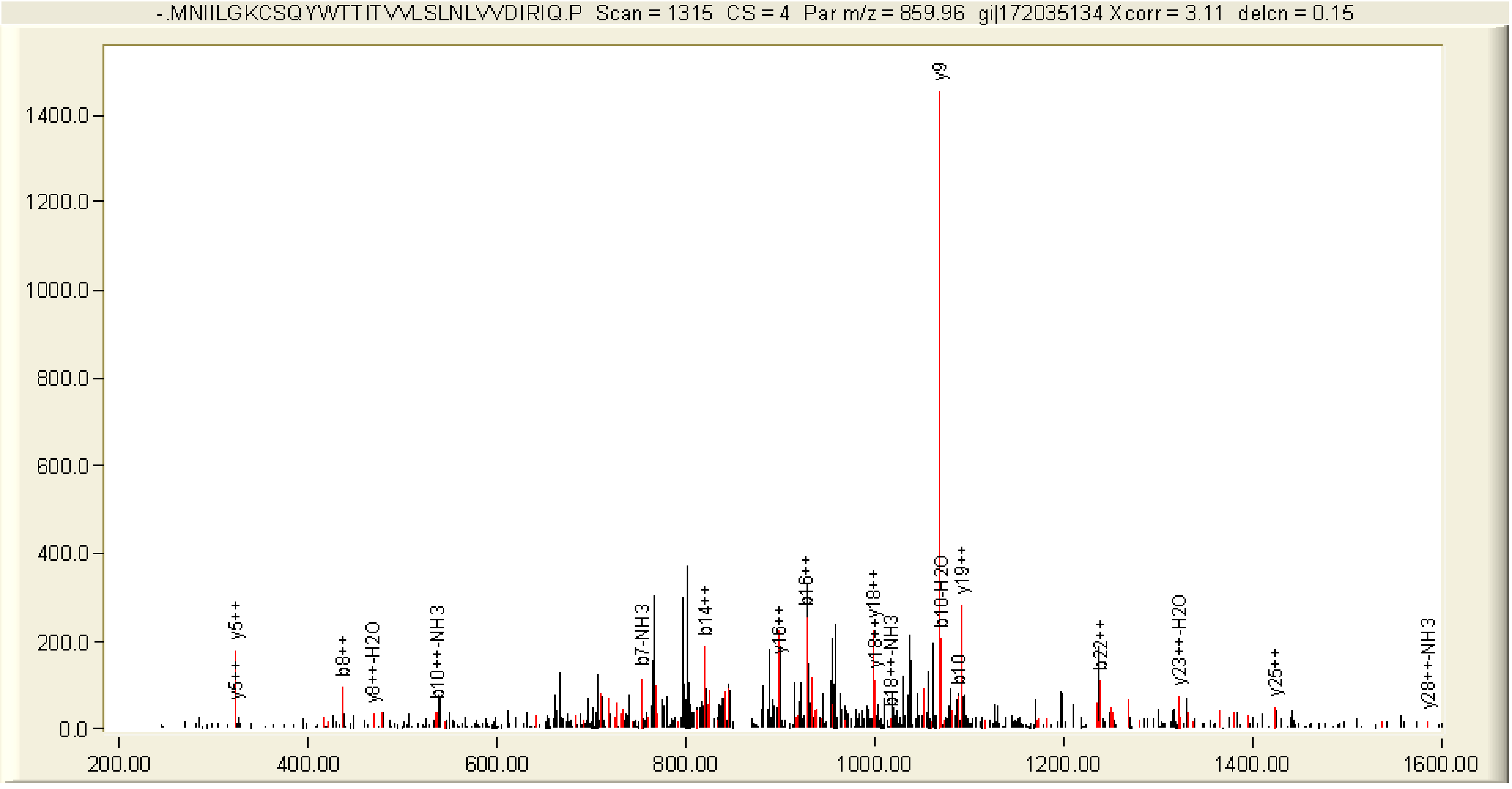
-.MNIILGKCSQYWTTITVVLSLNLVVDIRIQ.P Scan = 1315 CS = 4 Par m/z = 859.96 gi|172035134 Xcorr = 3.11 delcn = 0.15
y9
y5++
y5++
b8++
y8++-H2O
b10++-NH3
b7-NH3
b14++
y16++
b16++
y18++ y18++
b18++-NH3
b10-H2O
y19++
b10
b22++
y23++-H2O
y25++
y28++-NH3
1400.0
1200.0
1000.0
800.0
600.0
400.0
200.0
0.0
200.00
400.00
600.00
800.00
1000.00
1200.00
1400.00
1600.00

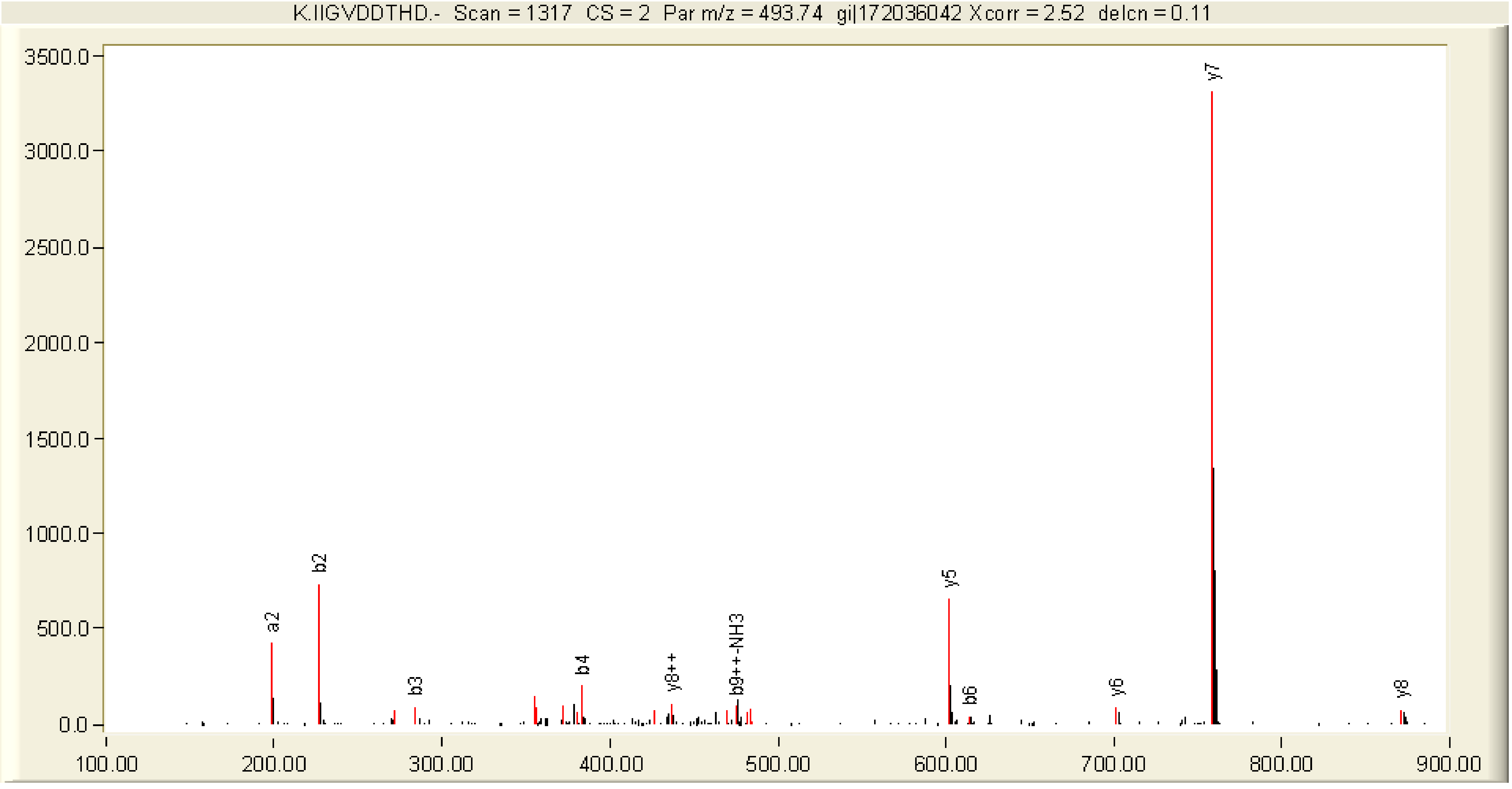

K.IIGVDDTHD.- Scan = 1317 CS = 2 Par m/z = 493.74 gi|172036042 Xcorr = 2.52 delcn = 0.11
3500.0
3000.0
2500.0
2000.0
1500.0
1000.0
500.0
0.0
100.00
200.00
300.00
400.00
500.00
600.00
700.00
800.00
900.00
a2
b2
b3
b4
y8++
b9++-NH3
y5
b6
y6
y7
y8

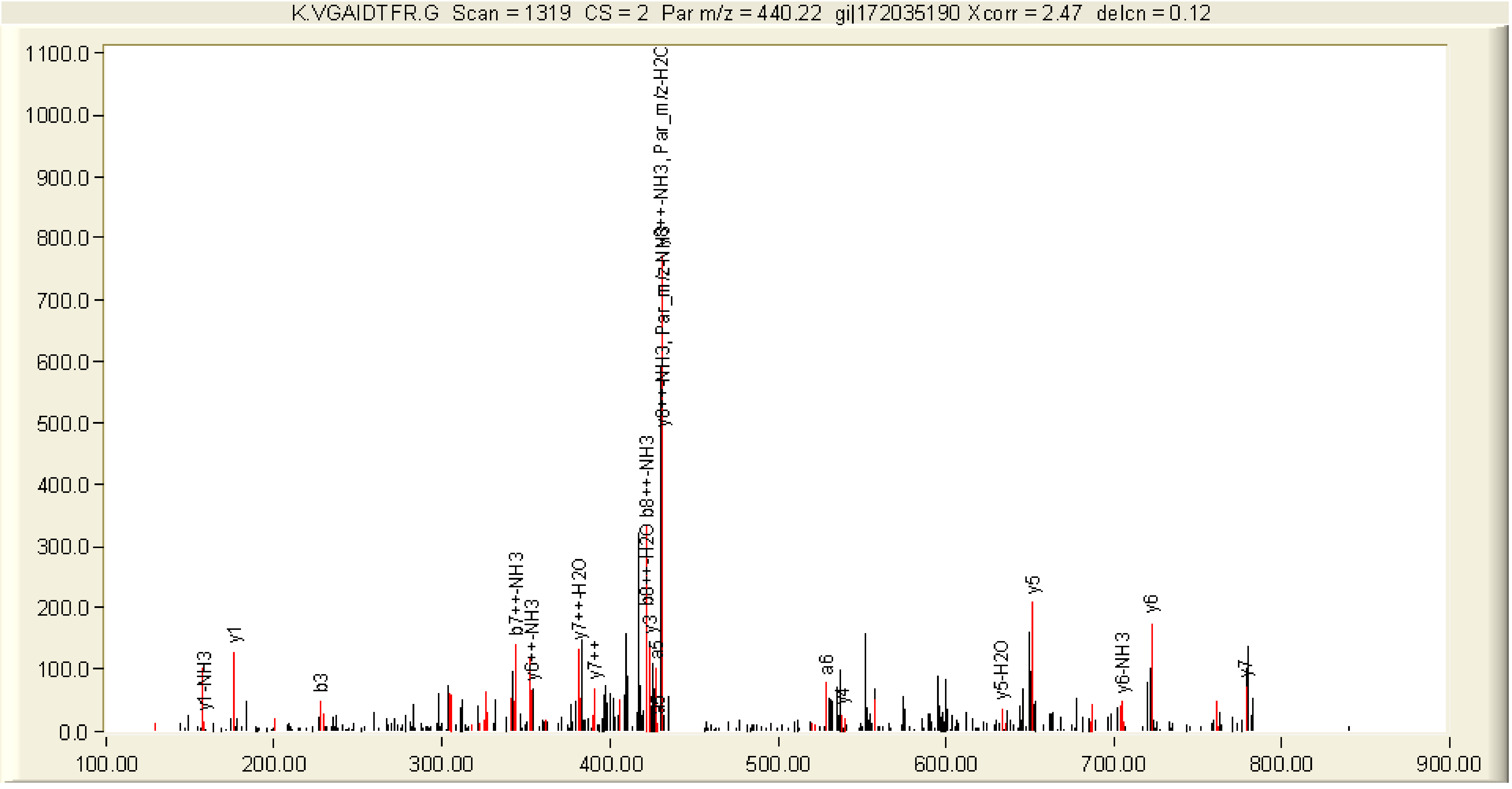

K.VGAIDTFR.G Scan = 1319 CS = 2 Par m/z = 440.22 gi|172035190 Xcorr = 2.47 delcn = 0.12
1100.0
1000.0
900.0
800.0
700.0
600.0
500.0
400.0
300.0
200.0
100.0
0.0
100.00
200.00
300.00
400.00
500.00
600.00
700.00
800.00
900.00
y1-NH3
y1
b3
b7++-NH3
y6++-NH3
y7++-H2O
y7++
b8++-NH3
y8++-NH3, Par_m/z-H2O
a5
y3
a6
y4
y5-H2O
y5
y6-NH3
y6
y7

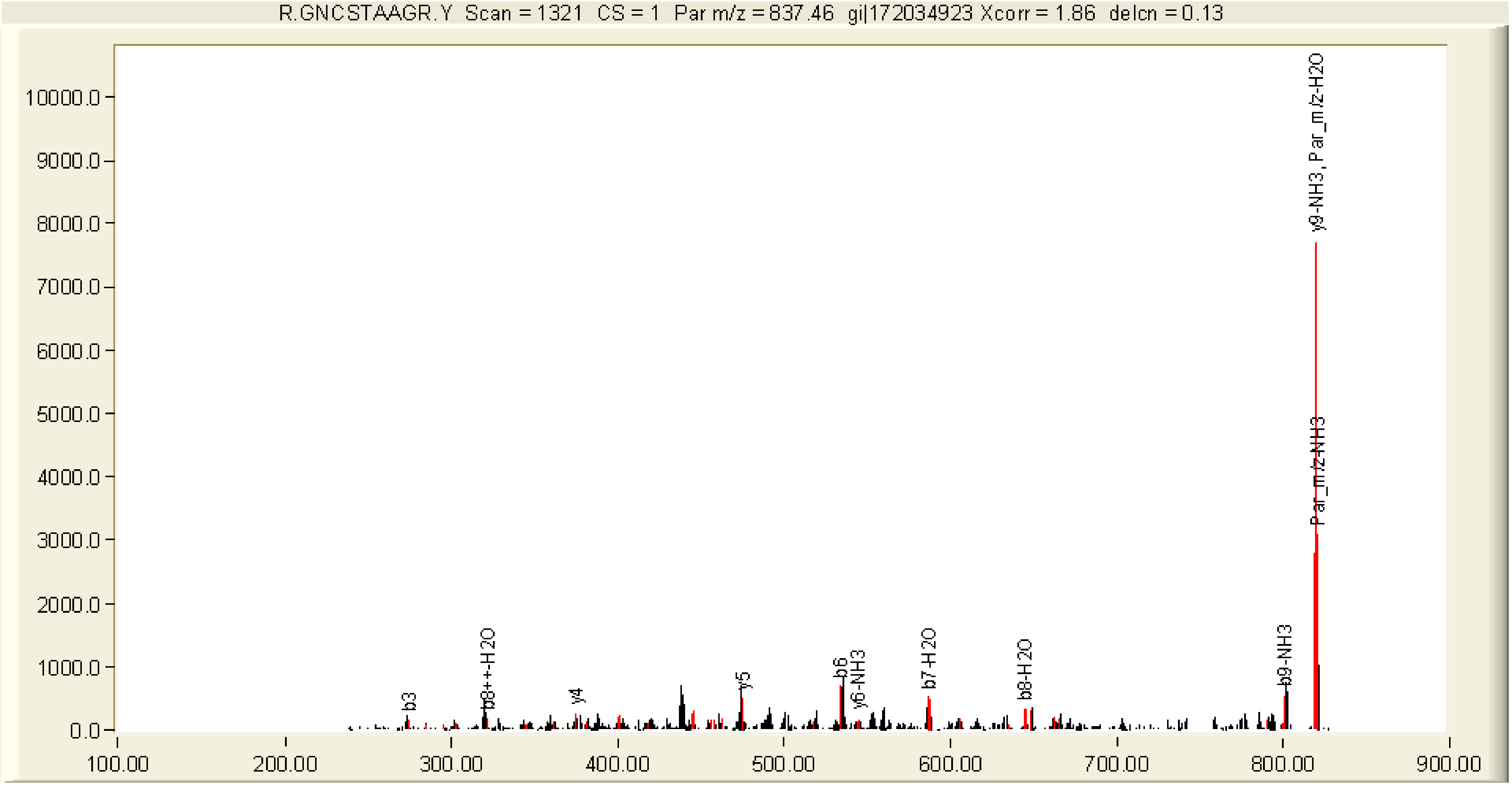

R.GNCSTAAGR.Y Scan = 1321 CS = 1 Par m/z = 837.46 gi|172034923 Xcorr = 1.86 delcn = 0.13
10000.0
9000.0
8000.0
7000.0
6000.0
5000.0
4000.0
3000.0
2000.0
1000.0
0.0
100.00
200.00
300.00
400.00
500.00
600.00
700.00
800.00
900.00
b3
b8++-H2O
y4
y5
b6
y6-NH3
b7-H2O
b8-H2O
b9-NH3
Par_m/z-NH3
y9-NH3, Par_m/z-H2O

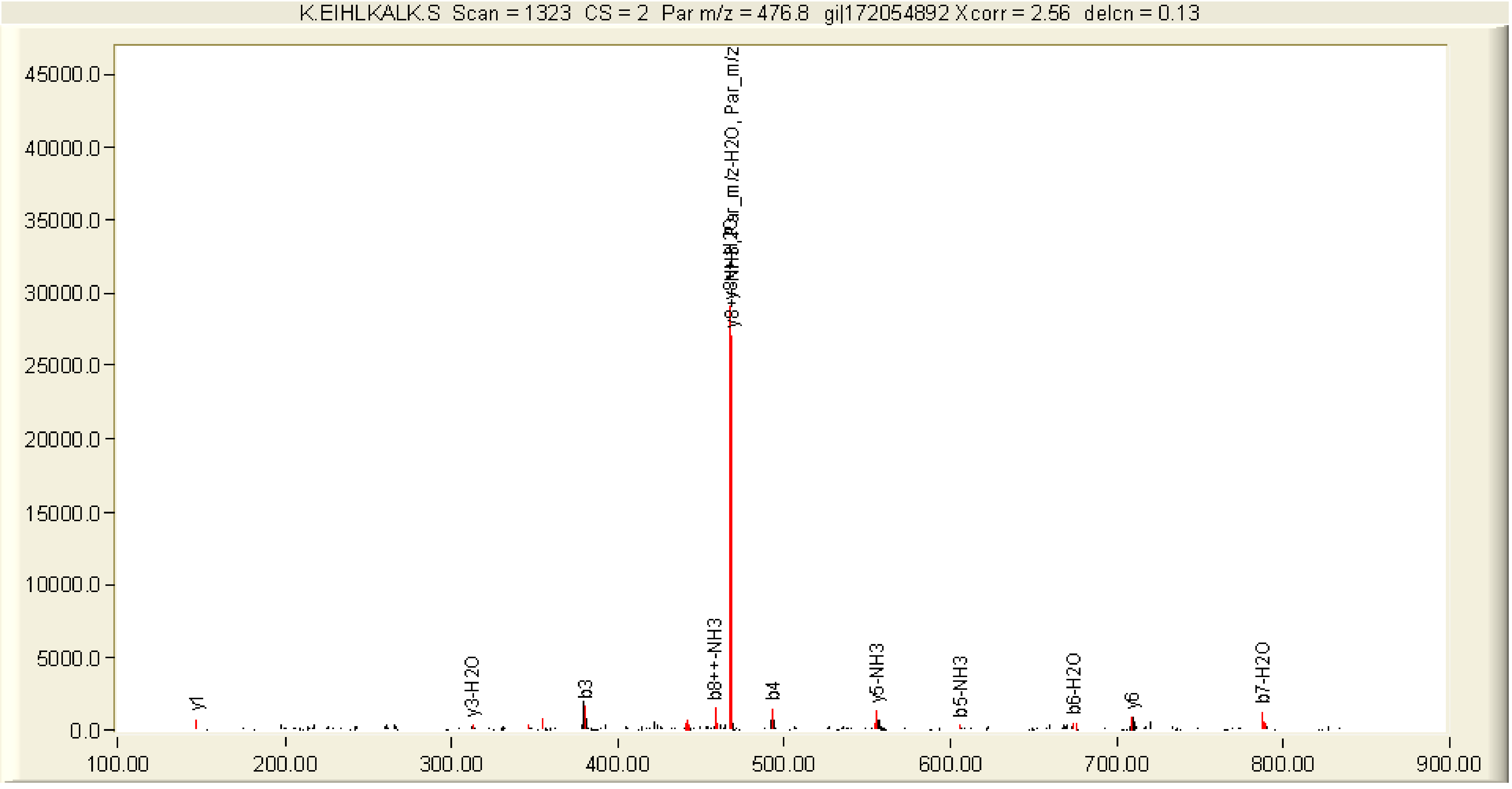

K.EIHLKALK.S Scan = 1323 CS = 2 Par m/z = 476.8 gi|172054892 Xcorr = 2.56 delcn = 0.13
45000.0
40000.0
35000.0
30000.0
25000.0
20000.0
15000.0
10000.0
5000.0
0.0
100.00
200.00
300.00
400.00
500.00
600.00
700.00
800.00
900.00
y1
y3-H2O
b3
b8++-NH3
Par_m/z-H2O, Par_m/z
b4
y5-NH3
b5-NH3
b6-H2O
y6
b7-H2O

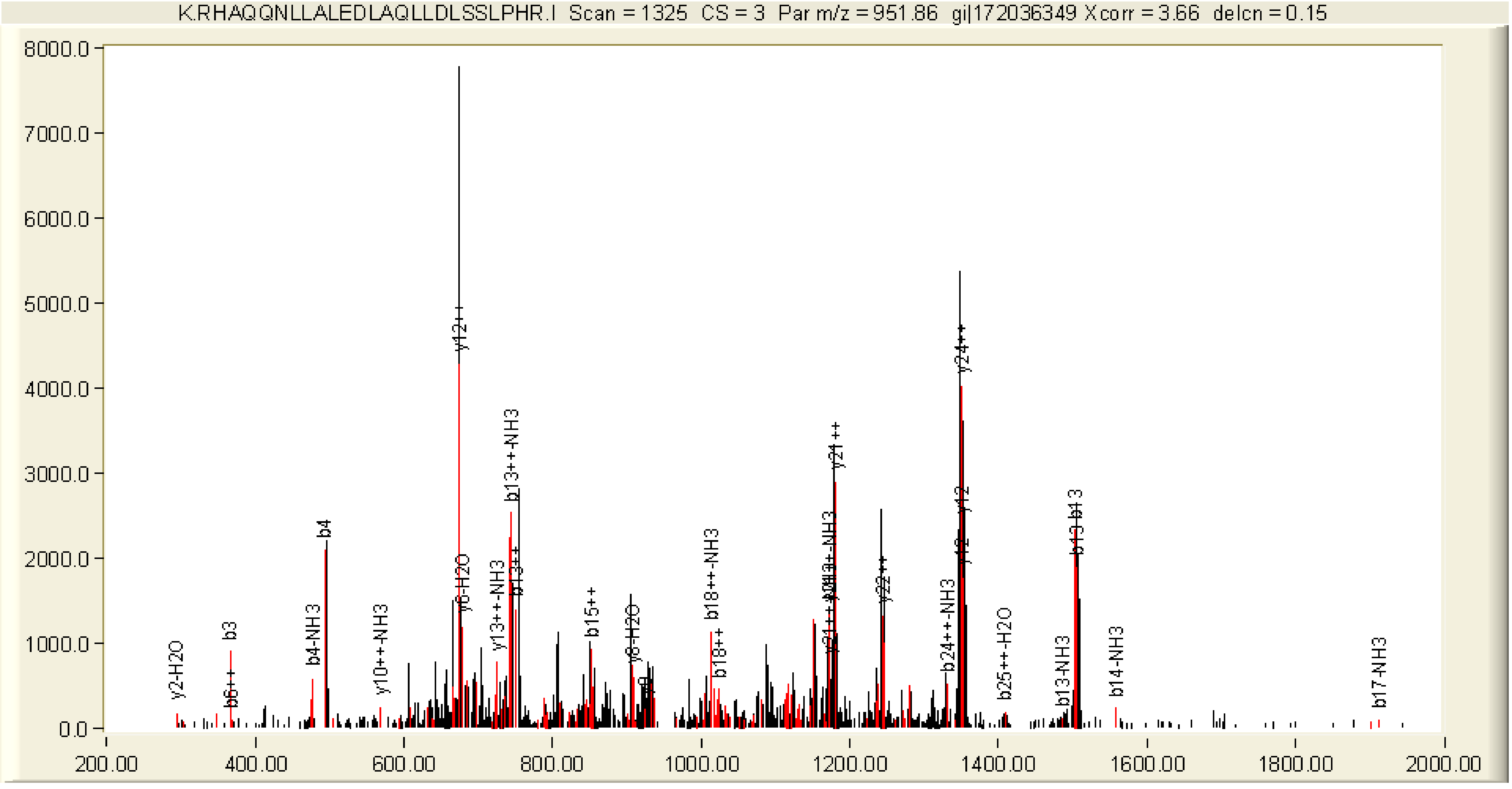
K.RHAQQNLLALEDLAQLLDLSSLPHR.I Scan = 1325 CS = 3 Par m/z = 951.86 gi|172036349 Xcorr = 3.66 delcn = 0.15
8000.0
7000.0
6000.0
5000.0
4000.0
3000.0
2000.0
1000.0
0.0
200.00
400.00
600.00
800.00
1000.00
1200.00
1400.00
1600.00
1800.00
2000.00
y2-H2O
b6++
b3
b4-NH3
b4
y10++-NH3
y6-H2O
y12++
y13++-NH3
b13++
b13++-NH3
b15++
y8-H2O
b18++
b18++-NH3
y21++
y22++
b24++-NH3
y24++
y12
b25++-H2O
b13-NH3
b13
b14-NH3
b17-NH3

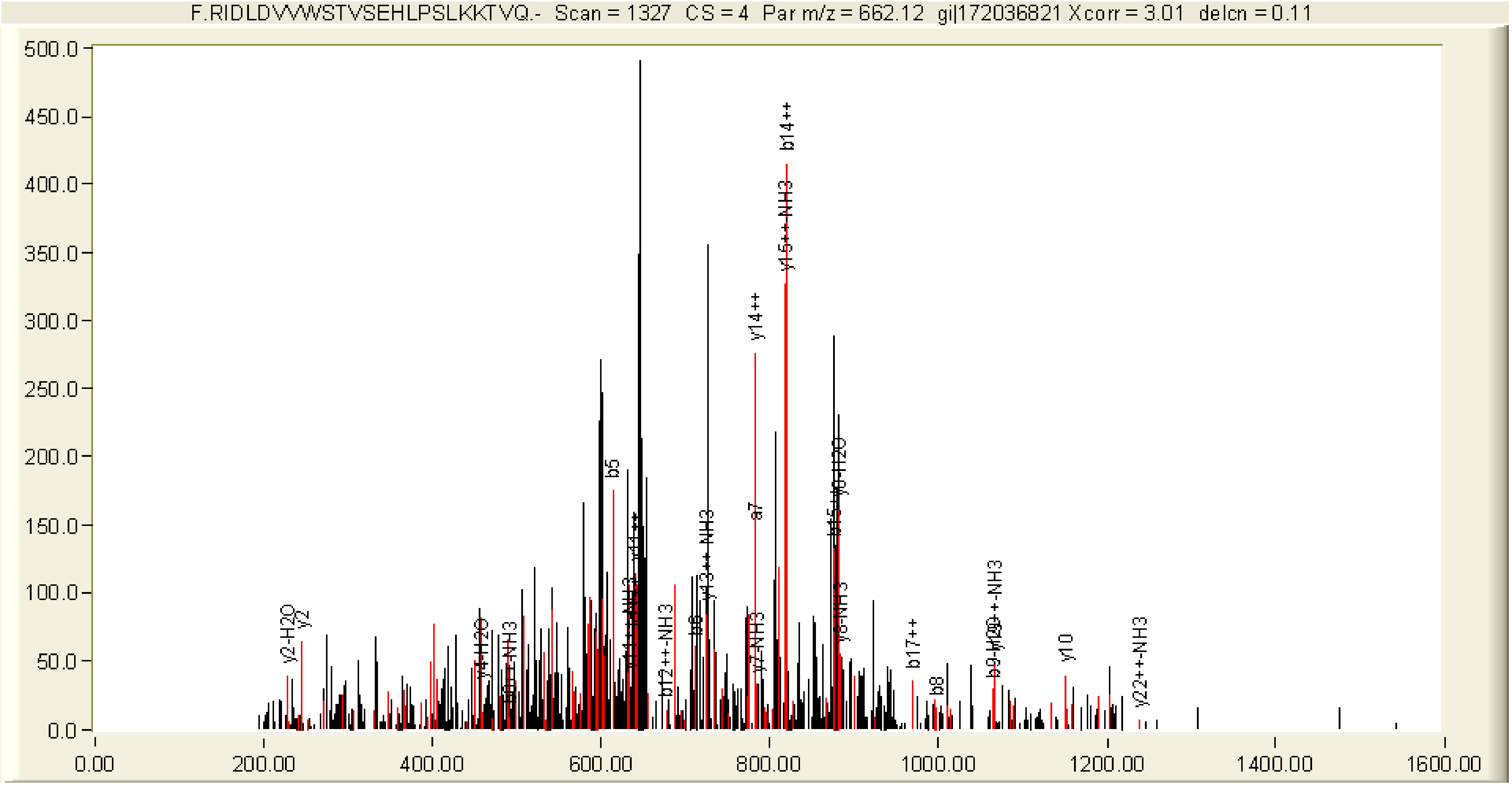
F.RIDLDVVWSTVSEHLPSLKKTVQ.- Scan = 1327 CS = 4 Par m/z = 662.12 gi|172036821 Xcorr = 3.01 delcn = 0.11
500.0
450.0
400.0
350.0
300.0
250.0
200.0
150.0
100.0
50.0
0.0
0.00
200.00
400.00
600.00
800.00
1000.00
1200.00
1400.00
1600.00
y2-H2O
y2
y4-H2O
b5
y14++
a7
y15++-NH3
b14++
y8-H2O
y8-NH3
y7-NH3
b12++-NH3
y13++-NH3
b17++
b8
y10
y22++-NH3

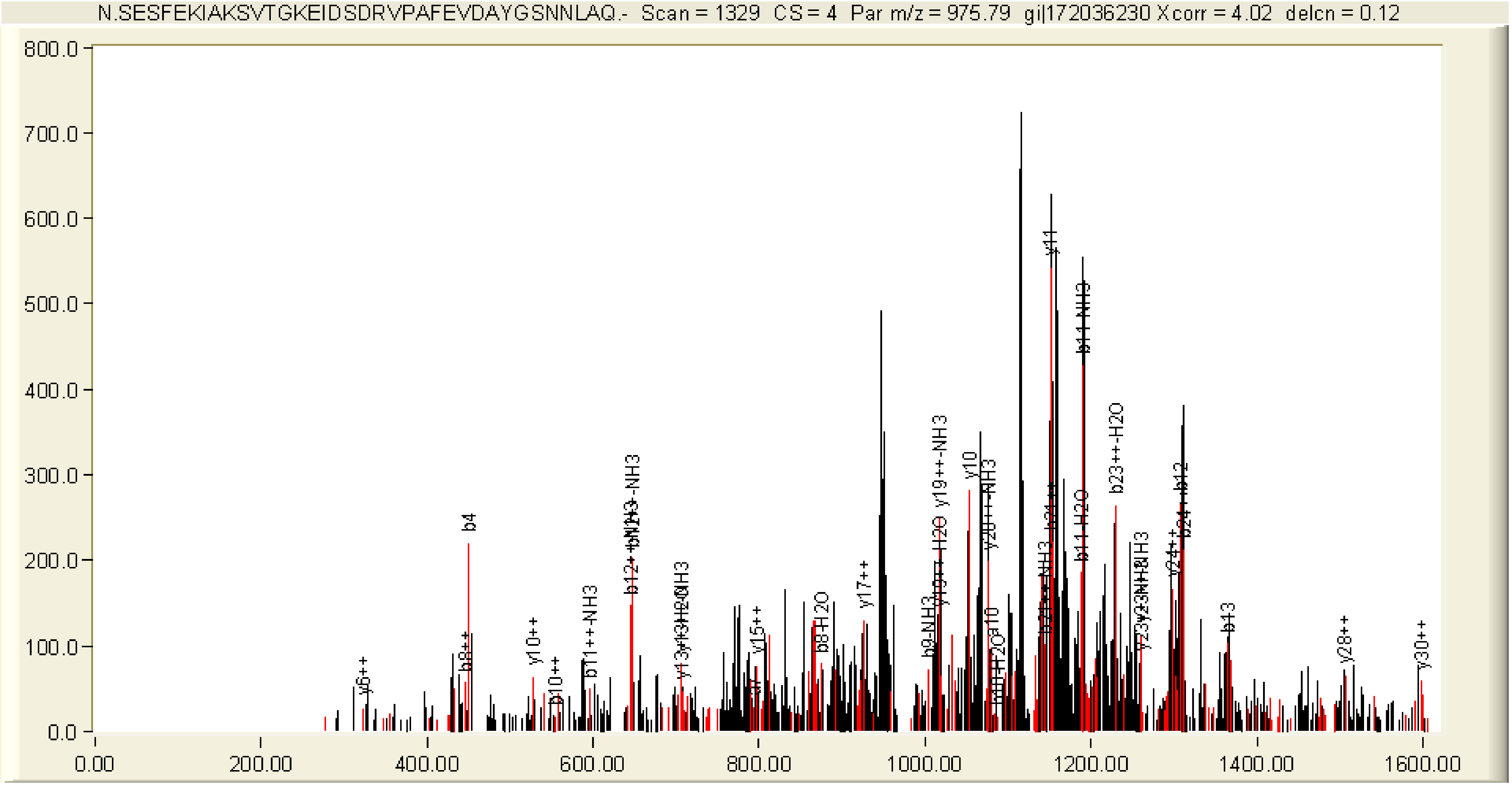

N.SESFEKIAKSVTGKEIDSDRVPAFEVDAYGSNNLAQ.- Scan = 1329 CS = 4 Par m/z = 975.79 gi|172036230 Xcorr = 4.02 delcn = 0.12
800.0
700.0
600.0
500.0
400.0
300.0
200.0
100.0
0.0
0.00
200.00
400.00
600.00
800.00
1000.00
1200.00
1400.00
1600.00
y6++
b8++
b4
y10++
b10++
b11++-NH3
y15++
b8-H2O
y17++
b9-NH3
y19++-NH3
y10
y11
b11-NH3
b11-H2O
b23++-H2O
y24++
b12
b13
y28++
y30++

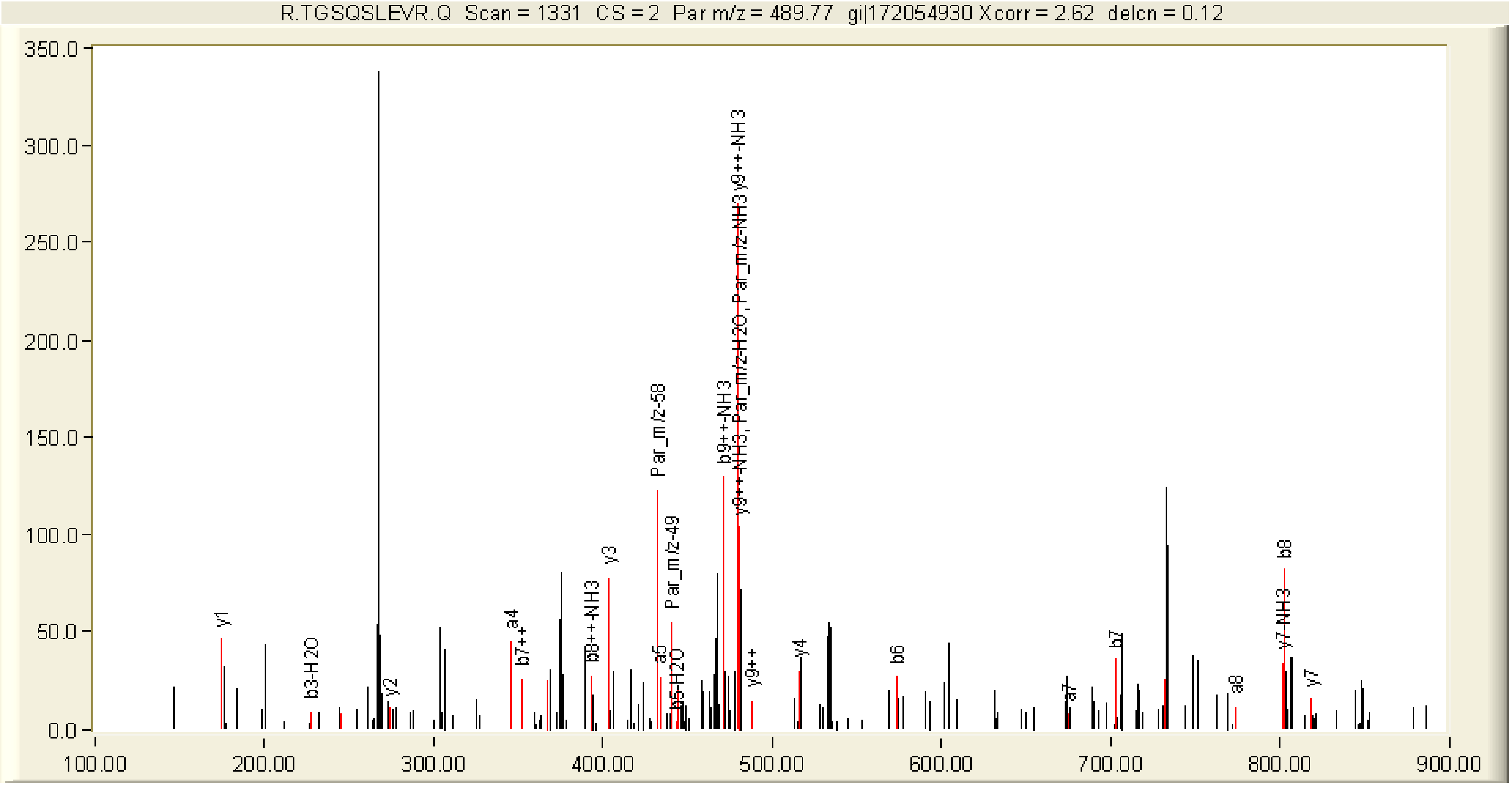
R.TGSQSLEVR.Q Scan = 1331 CS = 2 Par m/z = 489.77 gi|172054930 Xcorr = 2.62 delcn = 0.12
350.0
300.0
250.0
200.0
150.0
100.0
50.0
0.0
100.00
200.00
300.00
400.00
500.00
600.00
700.00
800.00
900.00
y1
b3-H2O
y2
a4
b7++
b8++-NH3
y3
Par_m/z-58
a5
Par_m/z-49
b5-H2O
b9++-NH3
y9++-NH3, Par_m/z-NH3, Par_m/z-H2O, Par_m/z-NH3 y9++-NH3
y9++
y4
b6
a7
b7
a8
y7-NH3
b8
y7

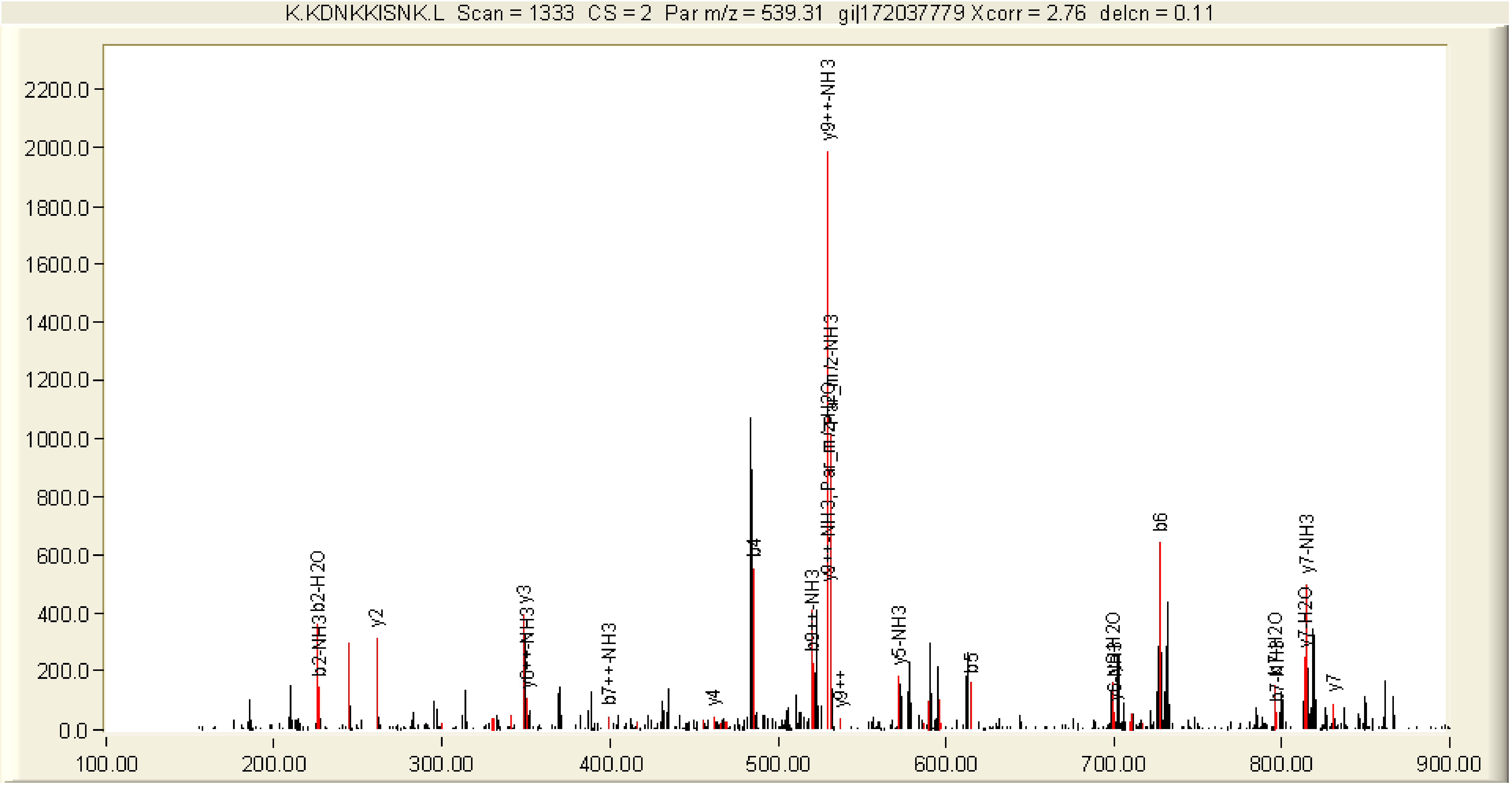
K.KDNKKISNK.L Scan = 1333 CS = 2 Par m/z = 539.31 gi|172037779 Xcorr = 2.76 delcn = 0.11
2200.0
2000.0
1800.0
1600.0
1400.0
1200.0
1000.0
800.0
600.0
400.0
200.0
0.0
100.00
200.00
300.00
400.00
500.00
600.00
700.00
800.00
900.00
b2-NH3
b2-H2O
y2
y6++-NH3
y3
b7++-NH3
y4
b4
b9++-NH3
y9++-NH3
y9++
y5-NH3
b5
b6
y7-NH3
y7-H2O
y7

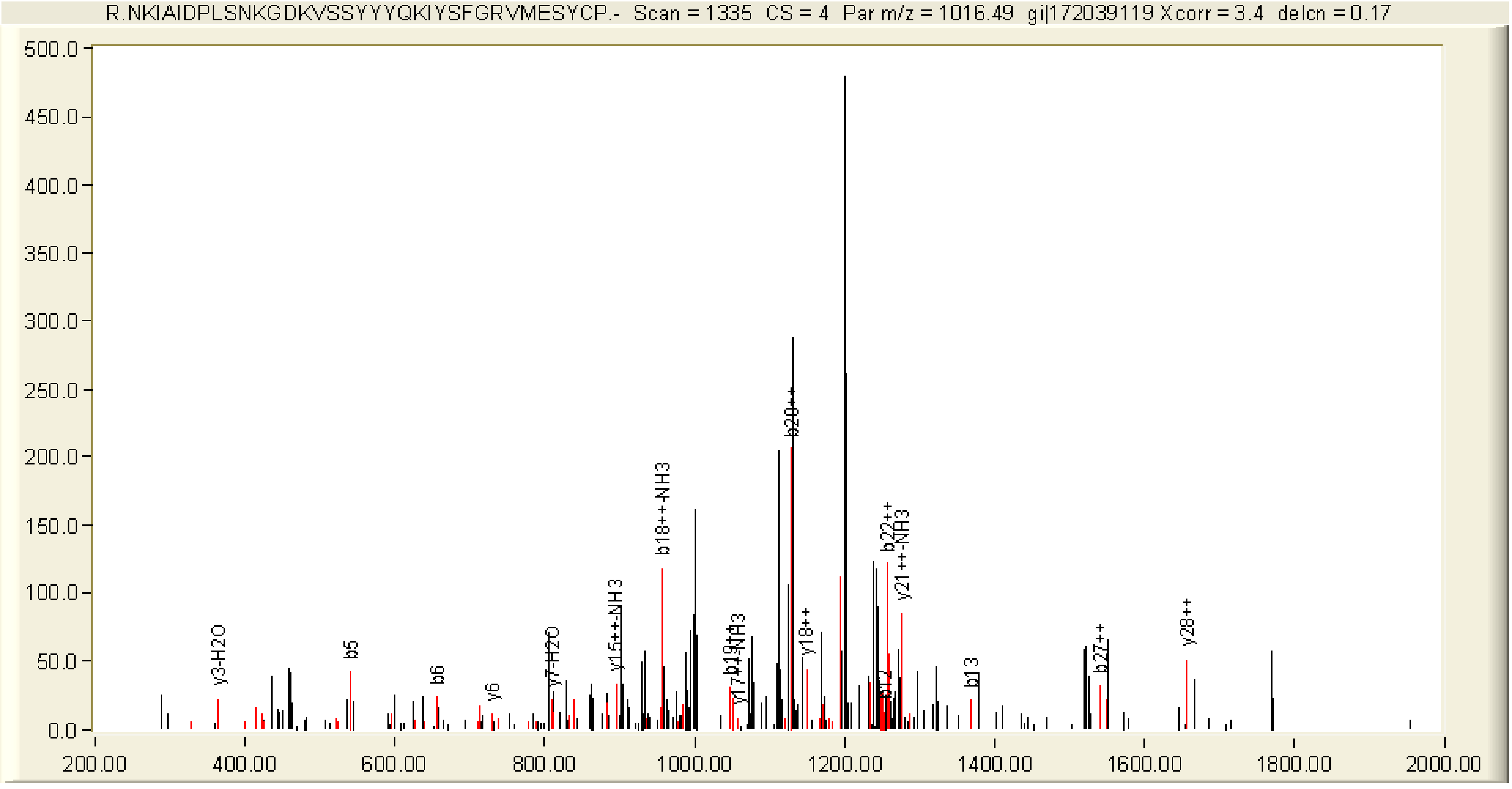

R.NKIAIDPLSNKGDKVSSYYYQKIYSFGRVMESYCP.- Scan = 1335 CS = 4 Par m/z = 1016.49 gi|172039119 Xcorr = 3.4 delcn = 0.17
y3-H2O
b5
b6
y6
y7-H2O
y15++-NH3
b18++-NH3
b19++-NH3
y17
b20++
y18++
b22++
y21++-NH3
b13
b27++
y28++

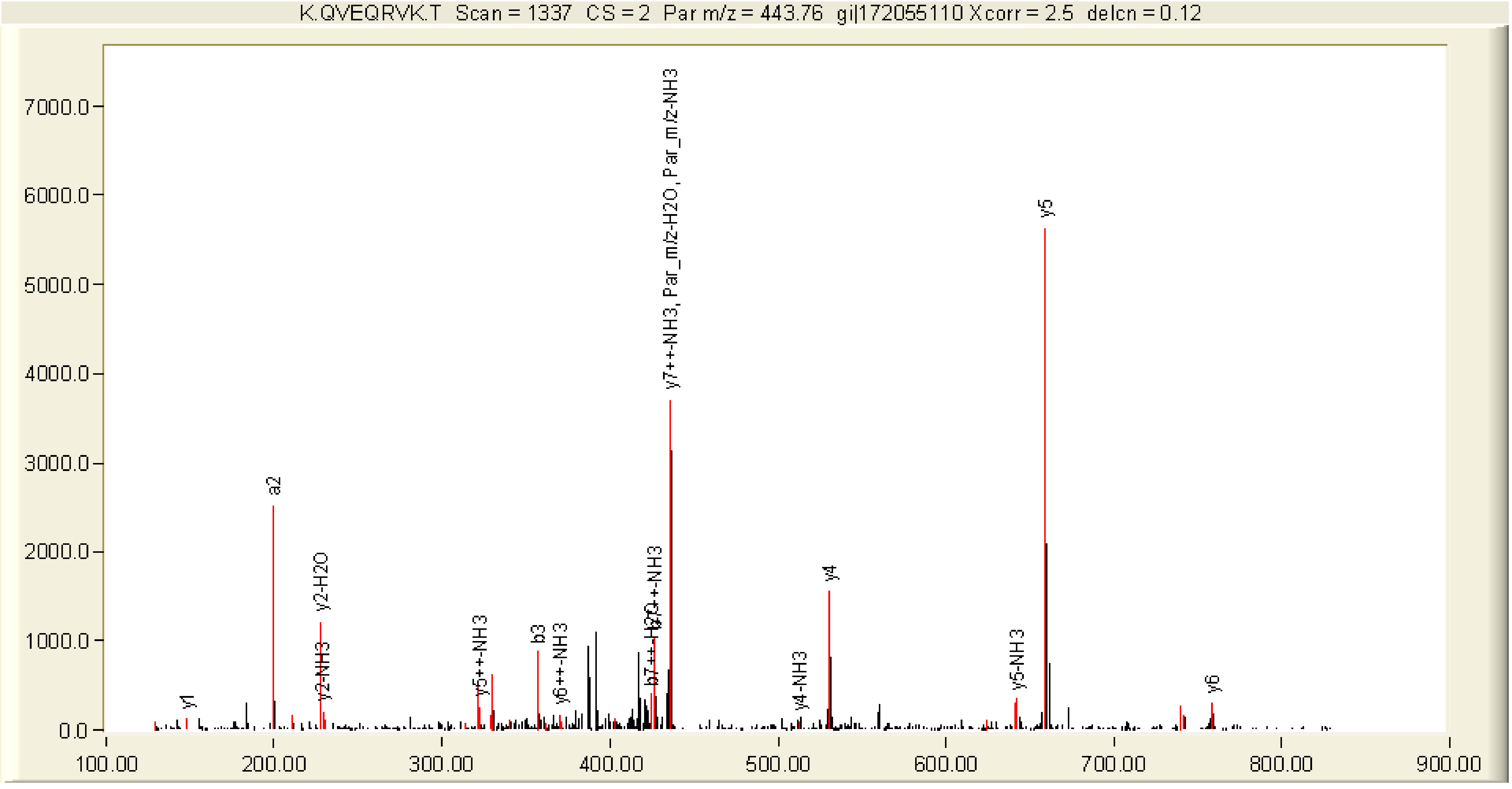
K.QVEQRVK.T Scan = 1337 CS = 2 Par m/z = 443.76 gi|172055110 Xcorr = 2.5 delcn = 0.12
7000.0
6000.0
5000.0
4000.0
3000.0
2000.0
1000.0
0.0
100.00
200.00
300.00
400.00
500.00
600.00
700.00
800.00
900.00
y1
a2
y2-H2O
y2-NH3
y5++-NH3
b3
y6++-NH3
y7++-NH3, Par_m/z-H2O, Par_m/z-NH3
y4-NH3
y4
y5-NH3
y5
y6

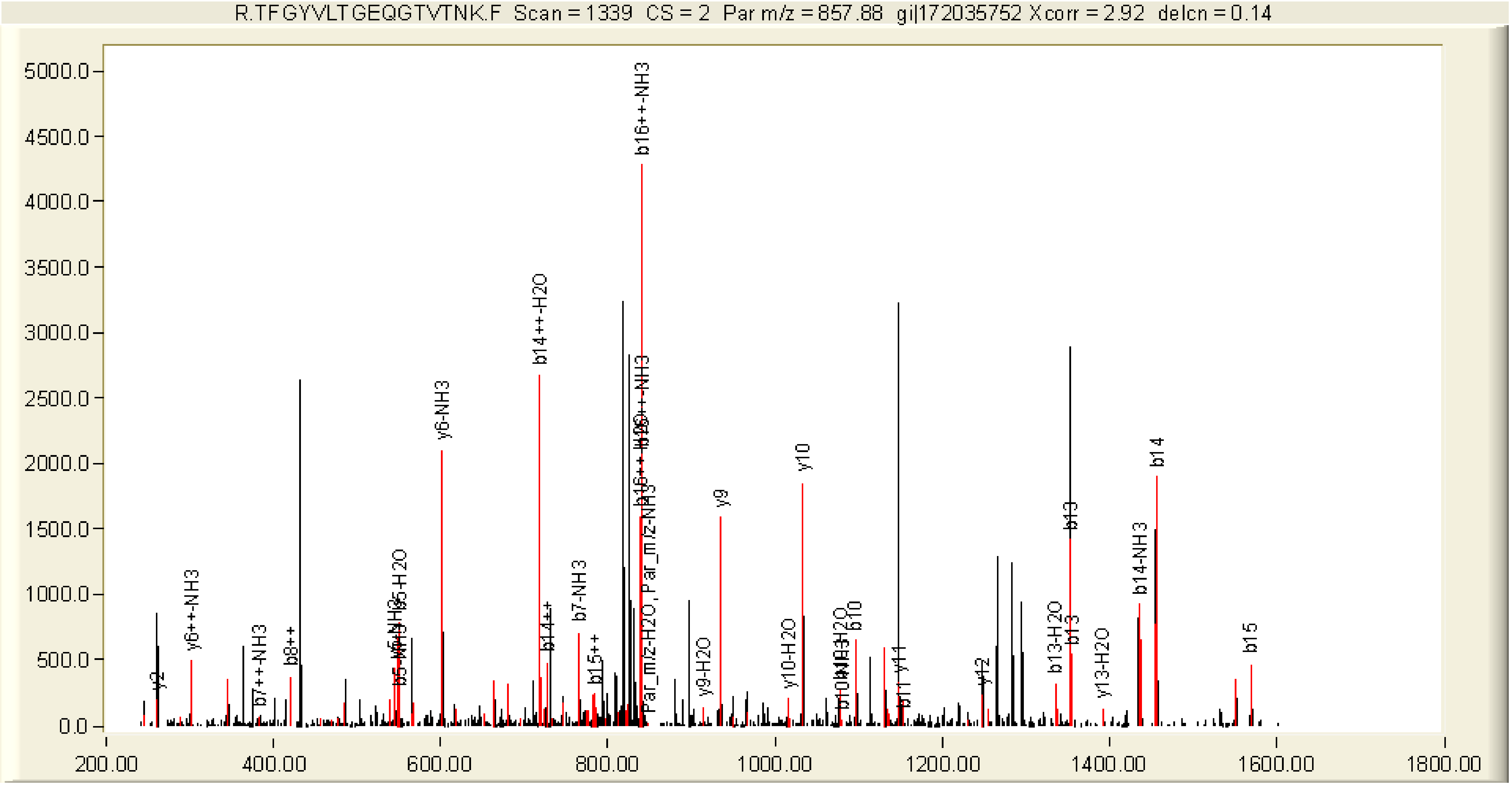
R.TFGYVLTGEQGTVTNK.F Scan = 1339 CS = 2 Par m/z = 857.88 gi|172035752 Xcorr = 2.92 delcn = 0.14
5000.0
4500.0
4000.0
3500.0
3000.0
2500.0
2000.0
1500.0
1000.0
500.0
0.0
200.00
400.00
600.00
800.00
1000.00
1200.00
1400.00
1600.00
1800.00
b16++-NH3
b14++-H2O
y6-NH3
y6++-NH3
y2
b7++-NH3
b8++
b7-NH3
b15++
b14++
y9
y9-H2O
y10
y10-H2O
b10
b13
b13-H2O
y13-H2O
b14-NH3
b14
b15
y12
b11

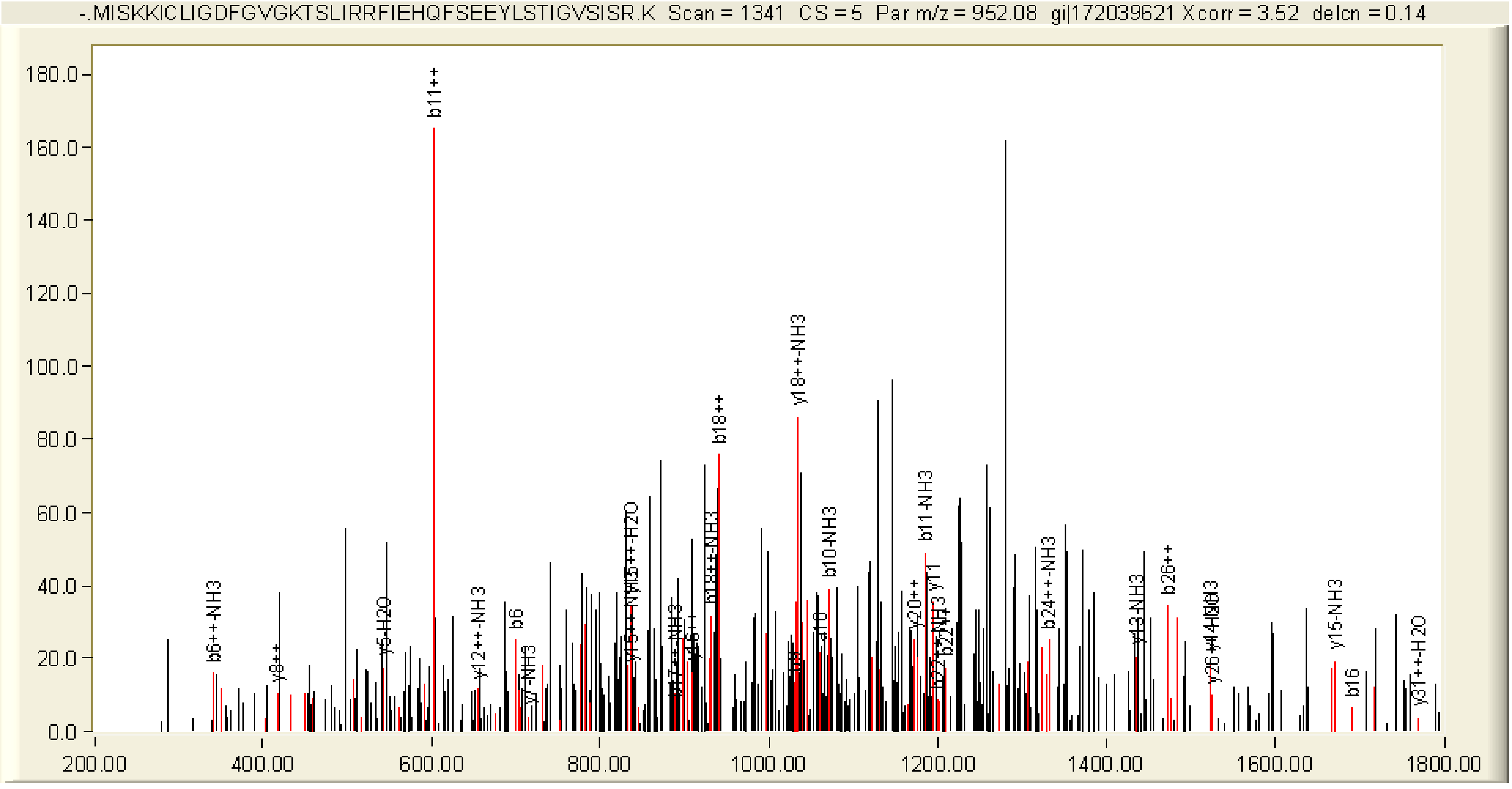
-.MISKKICLIGDFGVGKTSLIRRFIEHQFSEEYLSTIGVSISR.K Scan = 1341 CS = 5 Par m/z = 952.08 gi|172039621 Xcorr = 3.52 delcn = 0.14
180.0
160.0
140.0
120.0
100.0
80.0
60.0
40.0
20.0
0.0
200.00
400.00
600.00
800.00
1000.00
1200.00
1400.00
1600.00
1800.00
b11++
b6++-NH3
y8++
y5-H2O
y12++-NH3
b6
y7-NH3
b18++
y18++-NH3
b10-NH3
b11-NH3
y20++
y11
b24++-NH3
y13-NH3
b26++
y15-NH3
b16
y31++-H2O

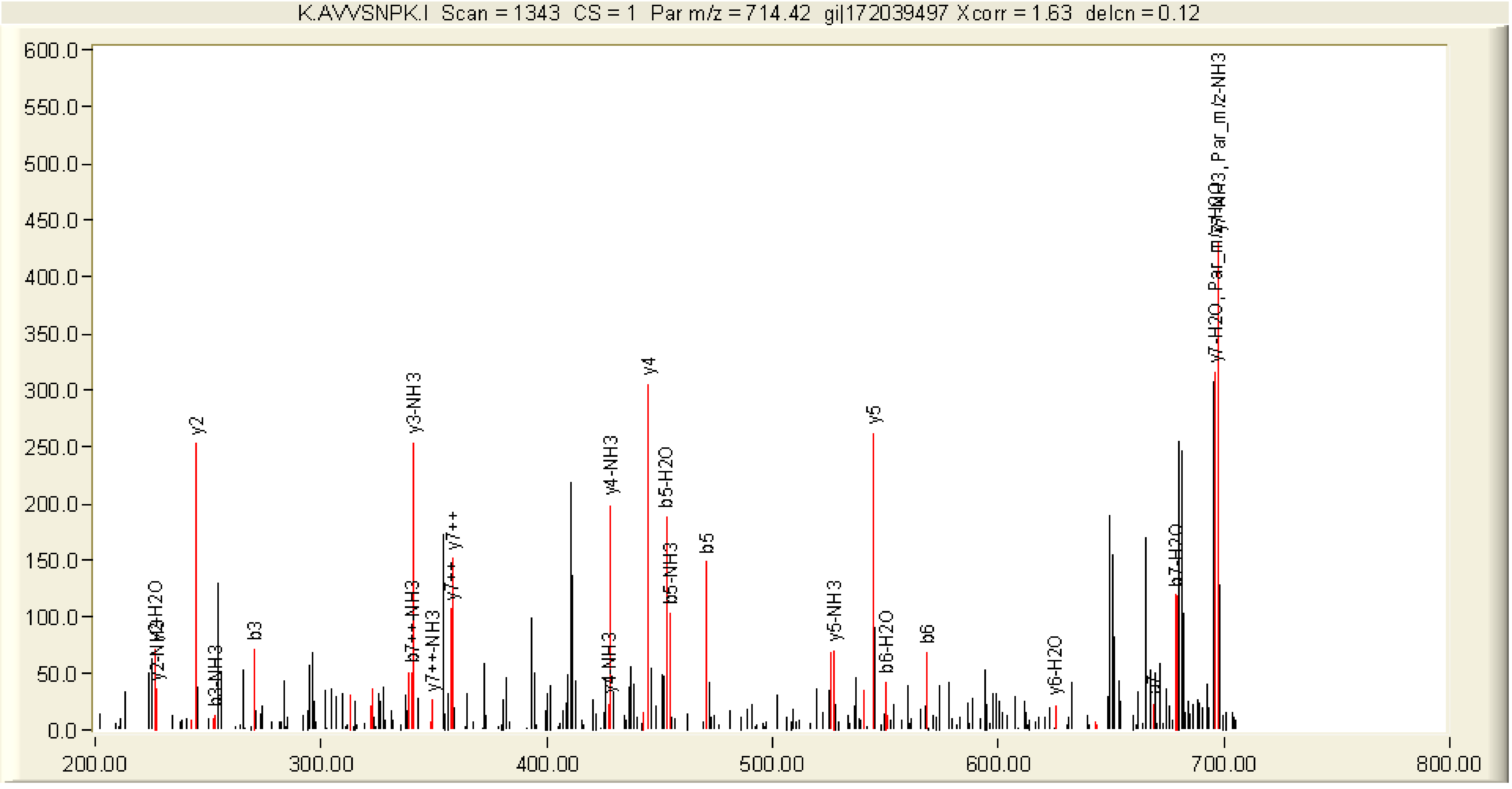
K.AVVSNPK.I Scan = 1343 CS = 1 Par m/z = 714.42 gi|172039497 Xcorr = 1.63 delcn = 0.12
600.0
550.0
500.0
450.0
400.0
350.0
300.0
250.0
200.0
150.0
100.0
50.0
0.0
200.00
300.00
400.00
500.00
600.00
700.00
800.00
y2
b3
b3-NH3
y3-NH3
b7++-NH3
y7++-NH3
y7++
y4-NH3
y4
b5-H2O
b5-NH3
b5
y5-NH3
y5
b6-H2O
b6
y6-H2O
b7-H2O
y7-H2O, Par_m/z-H2O
Par_m/z-NH3

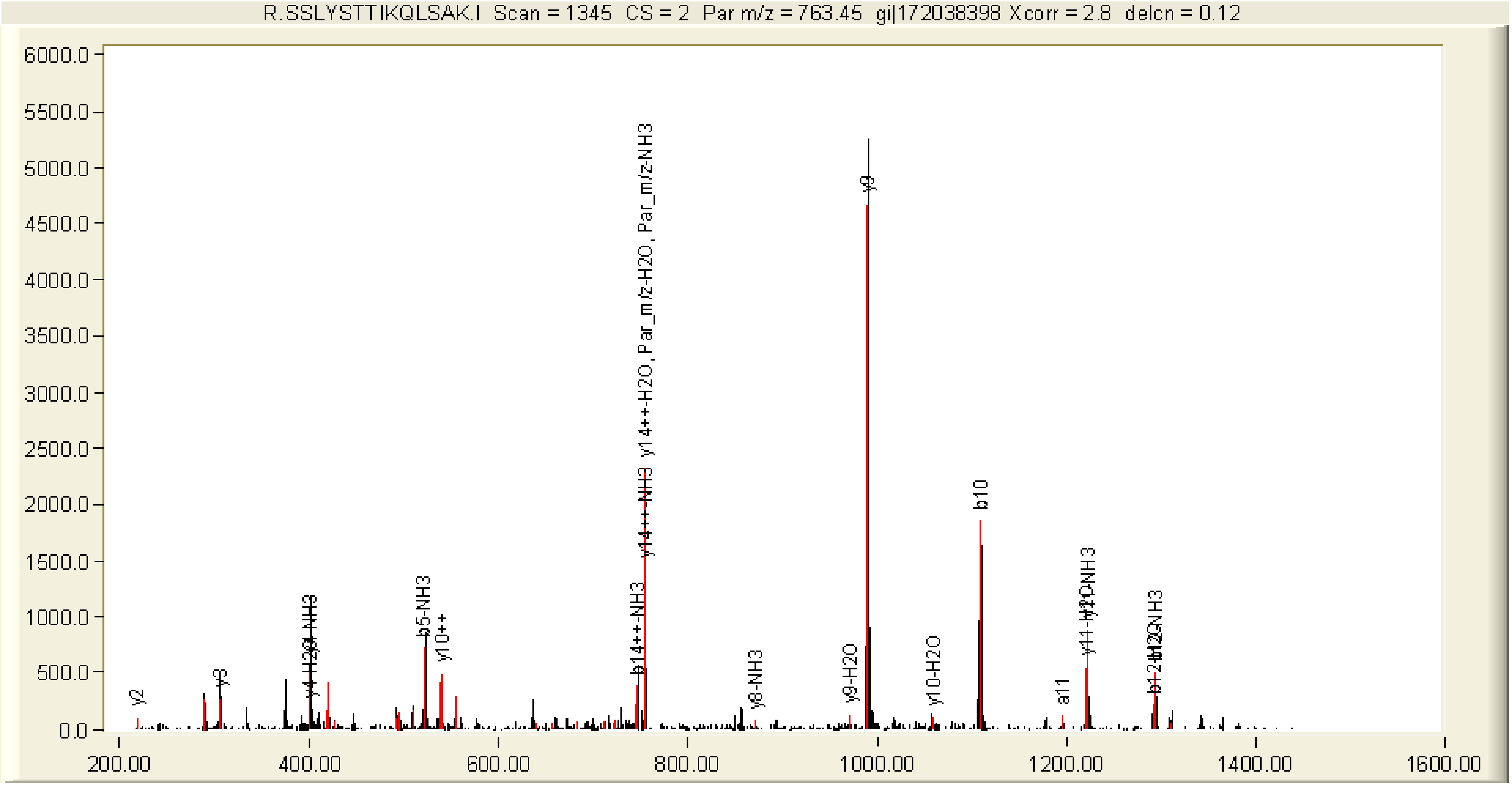
R.SSLYSTTIKQLSAK.I Scan = 1345 CS = 2 Par m/z = 763.45 gi|172038398 Xcorr = 2.8 delcn = 0.12
6000.0
5500.0
5000.0
4500.0
4000.0
3500.0
3000.0
2500.0
2000.0
1500.0
1000.0
500.0
0.0
200.00
400.00
600.00
800.00
1000.00
1200.00
1400.00
1600.00
y2
y3
b5-NH3
y10++
y14++-NH3
y14++-H2O, Par_m/z-H2O, Par_m/z-NH3
y8-NH3
y9-H2O
y9
y10-H2O
b10
a11

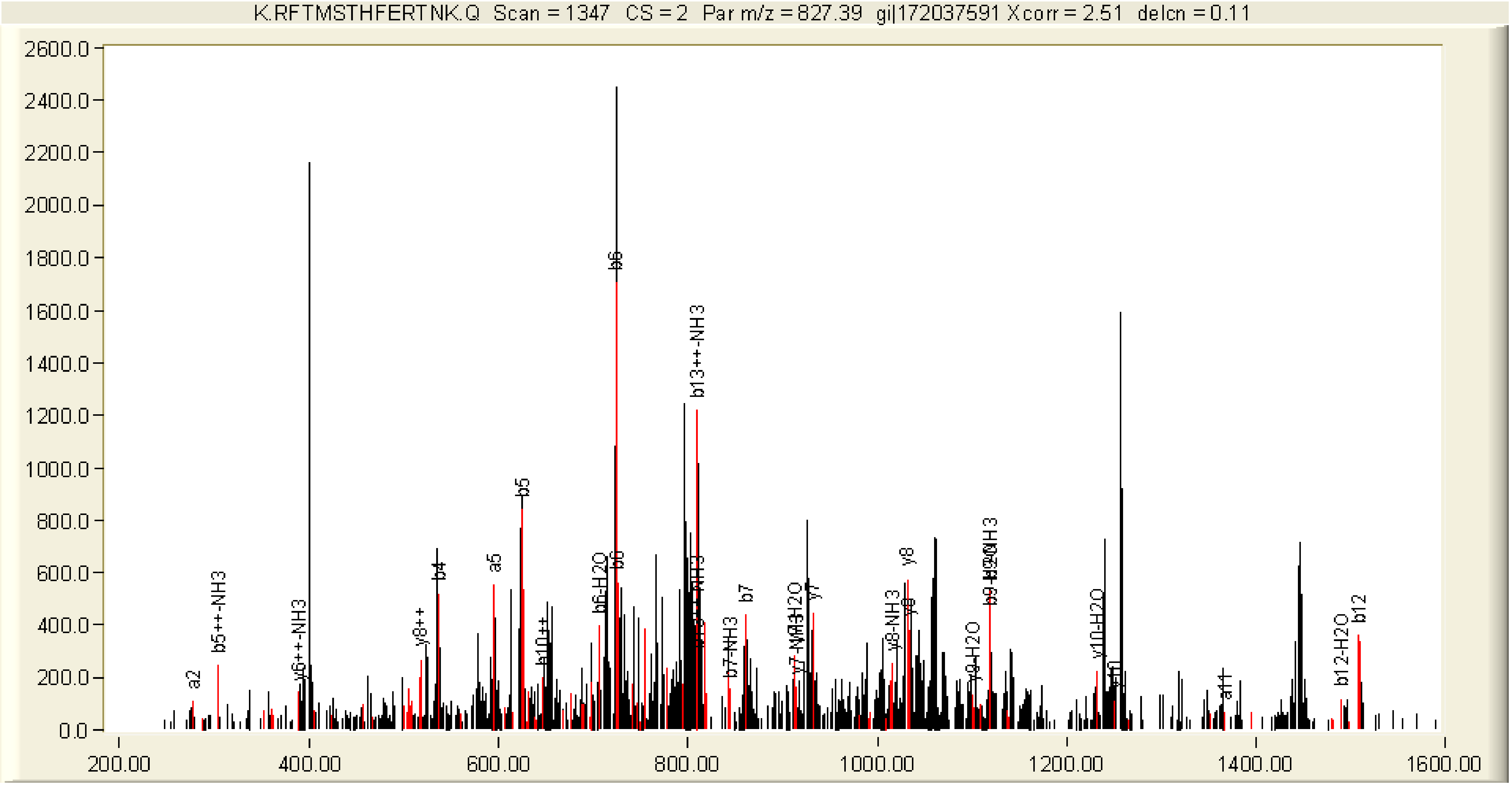
K.RFTMSTHFERTNK.Q Scan = 1347 CS = 2 Par m/z = 827.39 gi|172037591 Xcorr = 2.51 delcn = 0.11
2600.0
2400.0
2200.0
2000.0
1800.0
1600.0
1400.0
1200.0
1000.0
800.0
600.0
400.0
200.0
0.0
200.00
400.00
600.00
800.00
1000.00
1200.00
1400.00
1600.00
a2
b5++-NH3
y6++-NH3
y8++
b4
a5
b5
b10++
b6-H2O
b6
b13++-NH3
b7-NH3
b7
y7
y8-NH3
y8
y10-H2O
b12-H2O
b12

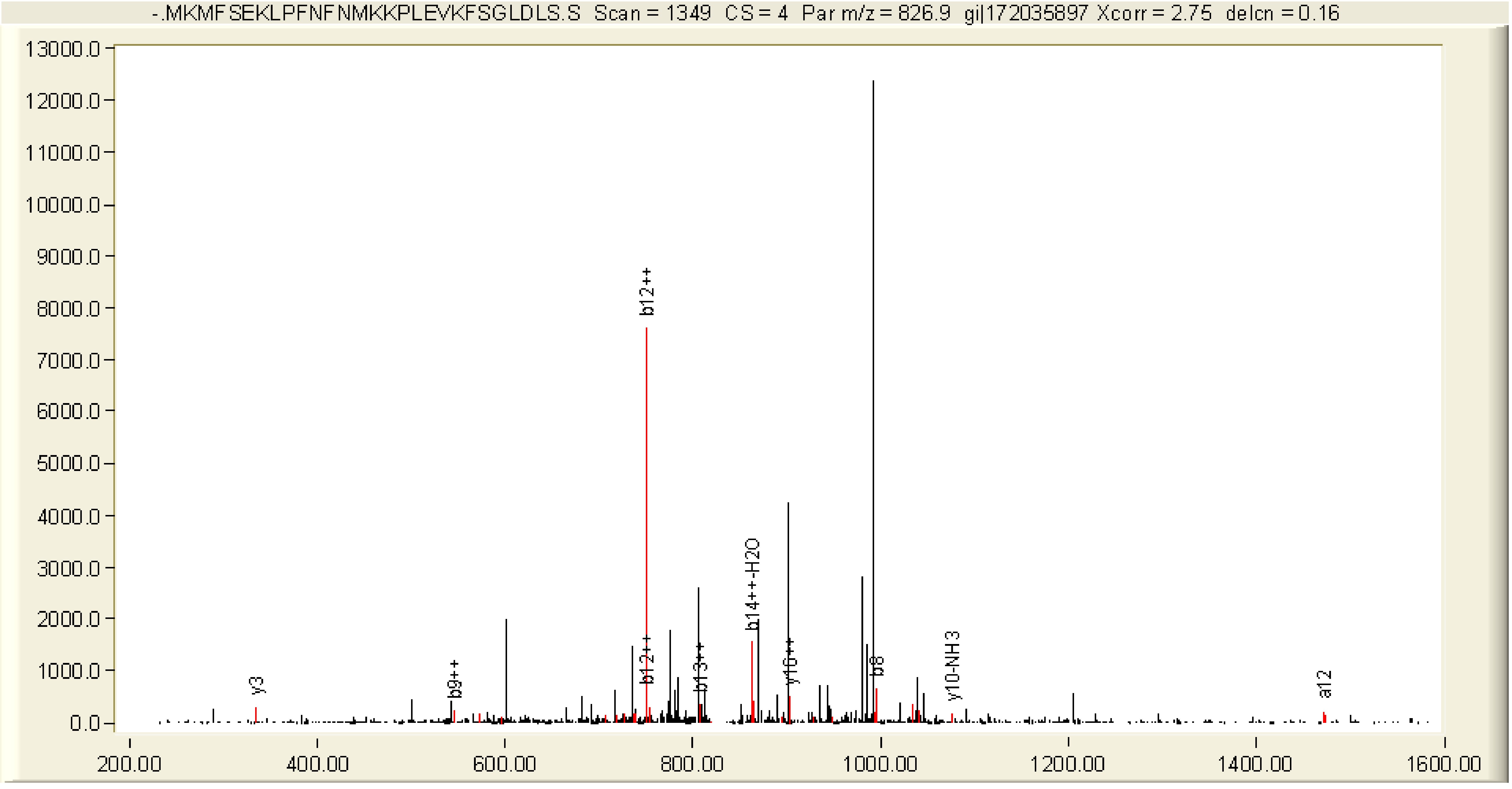
-.MKMFSEKLPFNFNMKKPLEVKFSGLDLS.S Scan = 1349 CS = 4 Par m/z = 826.9 gi|172035897 Xcorr = 2.75 delcn = 0.16
13000.0
12000.0
11000.0
10000.0
9000.0
8000.0
7000.0
6000.0
5000.0
4000.0
3000.0
2000.0
1000.0
0.0
200.00
400.00
600.00
800.00
1000.00
1200.00
1400.00
1600.00
b12++
b14++-H2O
y3
b9++
b12++
b13++
y16++
b8
y10-NH3
a12

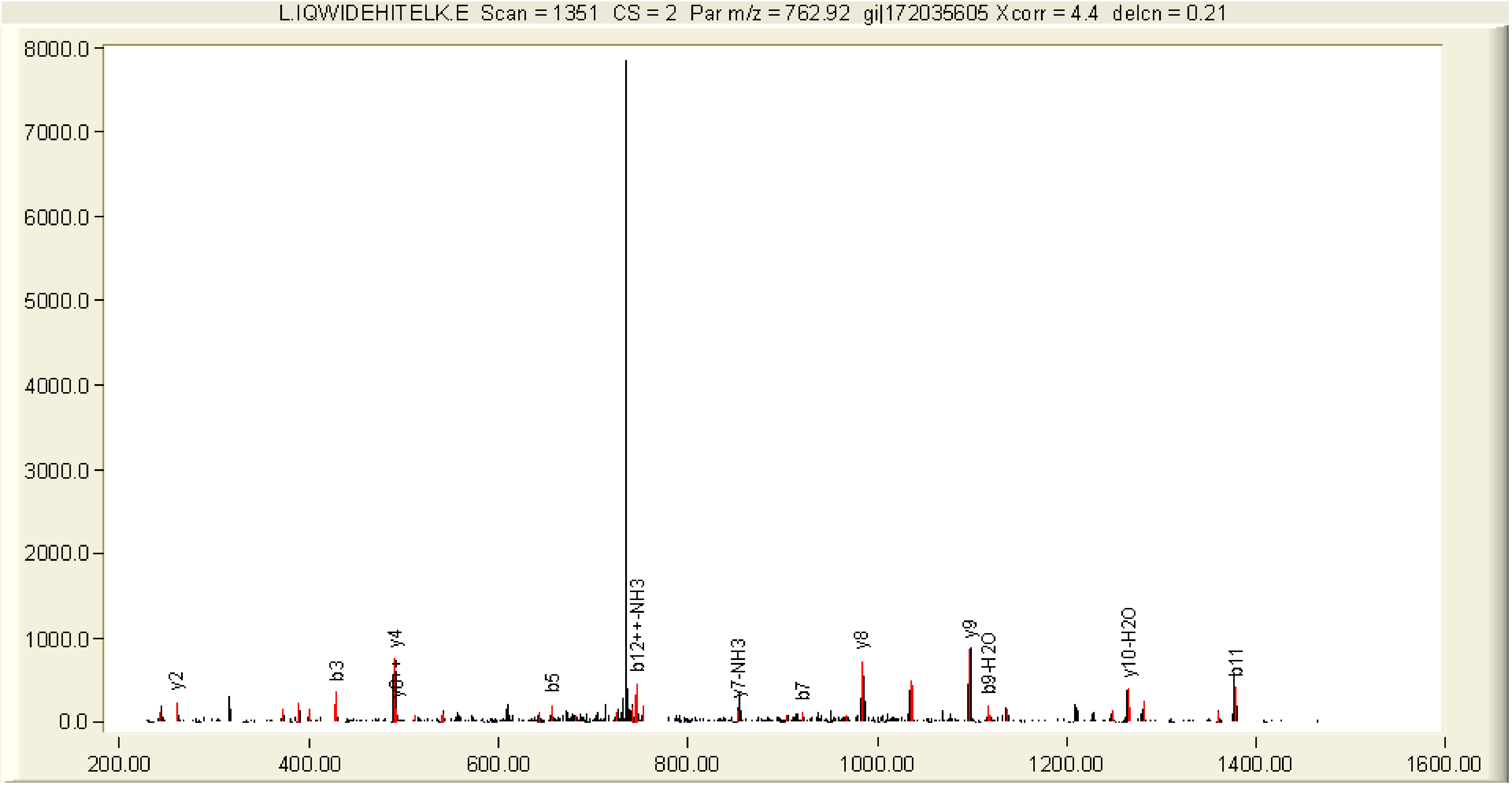

L.IQWIDEHITELK.E Scan = 1351 CS = 2 Par m/z = 762.92 gi|172035605 Xcorr = 4.4 delcn = 0.21
8000.0
7000.0
6000.0
5000.0
4000.0
3000.0
2000.0
1000.0
0.0
y2
b3
y4
b5
b12++-NH3
y7-NH3
b7
y8
y9
b9-H2O
y10-H2O
b11
200.00
400.00
600.00
800.00
1000.00
1200.00
1400.00
1600.00

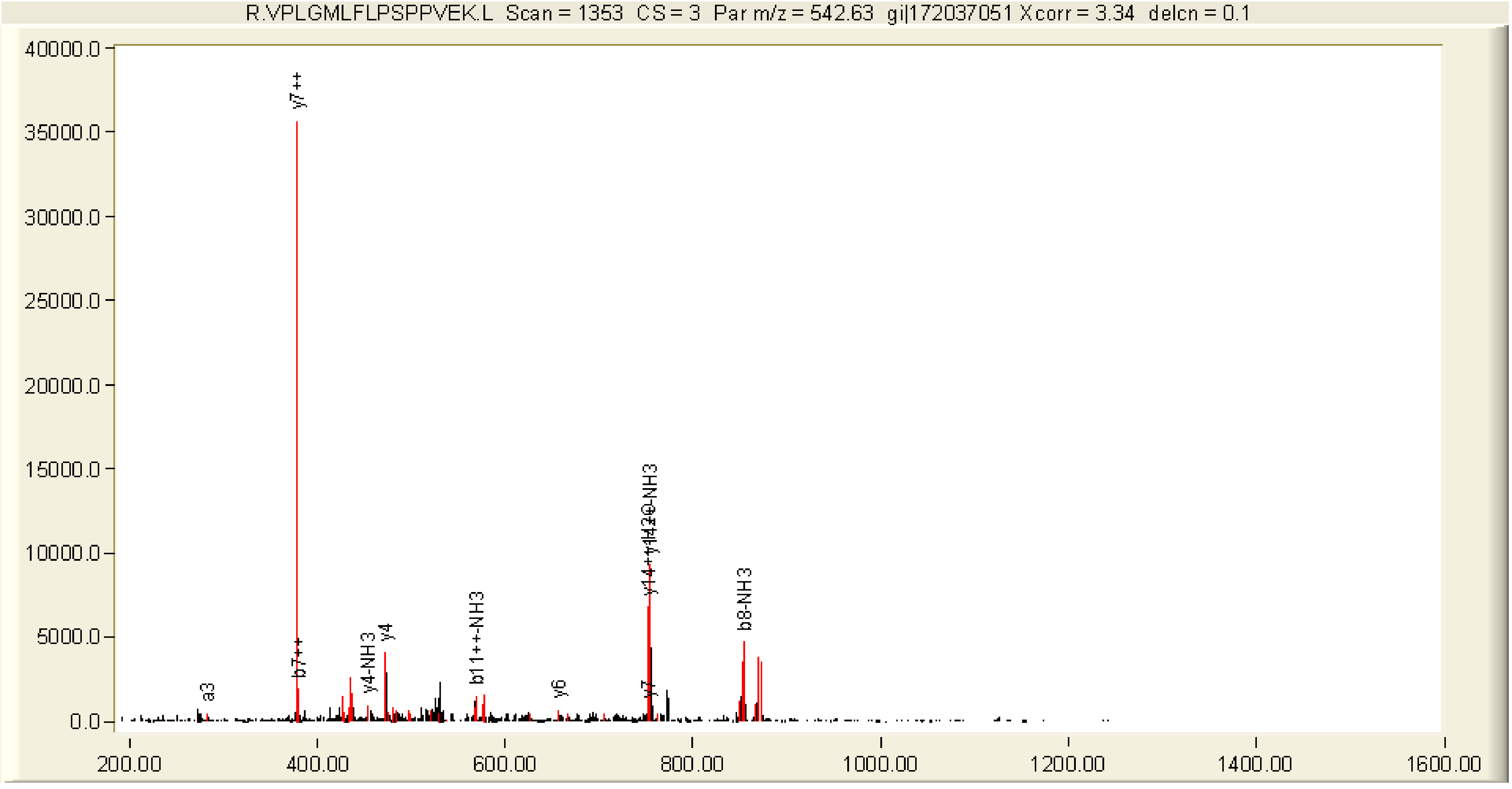

R.VPLGMLFLPSPPVEK.L Scan = 1353 CS = 3 Par m/z = 542.63 gi|172037051 Xcorr = 3.34 delcn = 0.1
40000.0
35000.0
30000.0
25000.0
20000.0
15000.0
10000.0
5000.0
0.0
200.00
400.00
600.00
800.00
1000.00
1200.00
1400.00
1600.00
y7++
a3
b7++
y4-NH3
y4
b11++-NH3
y6
y7
b8-NH3

R.SLIPYMMVIINAISIVLIVGLTNLYFK.S Scan = 1355 CS = 3 Par m/z = 1012.51 gi|172039436 Xcorr = 3.56 delcn = 0.14

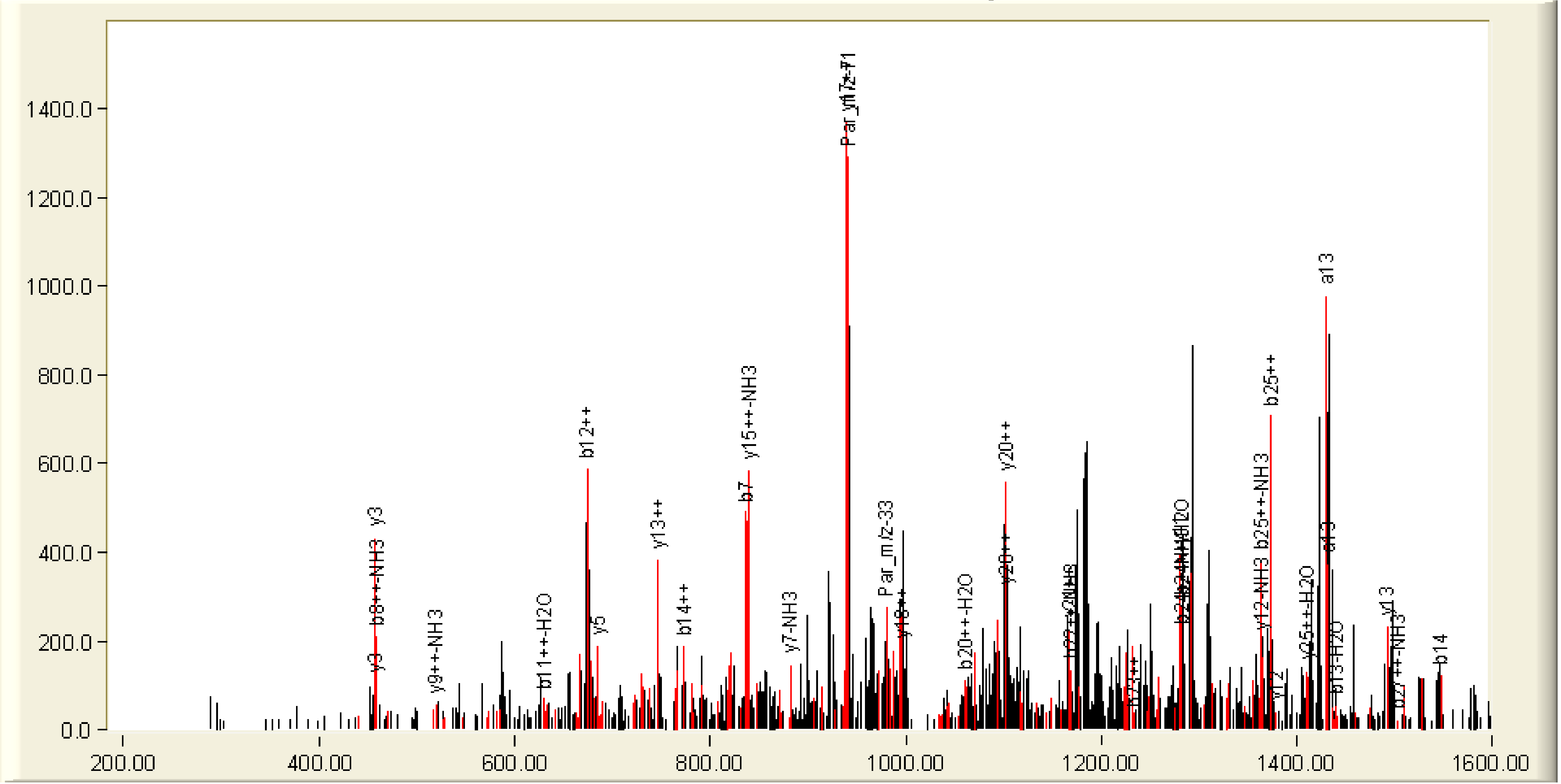

R.GRGGFRGSGGGGASPRINRSTSLQNRGSFNSSGR.T Scan = 1357 CS = 3 Par m/z = 1132.56 gi|172035108 Xcorr = 3.44 delcn = 0.18

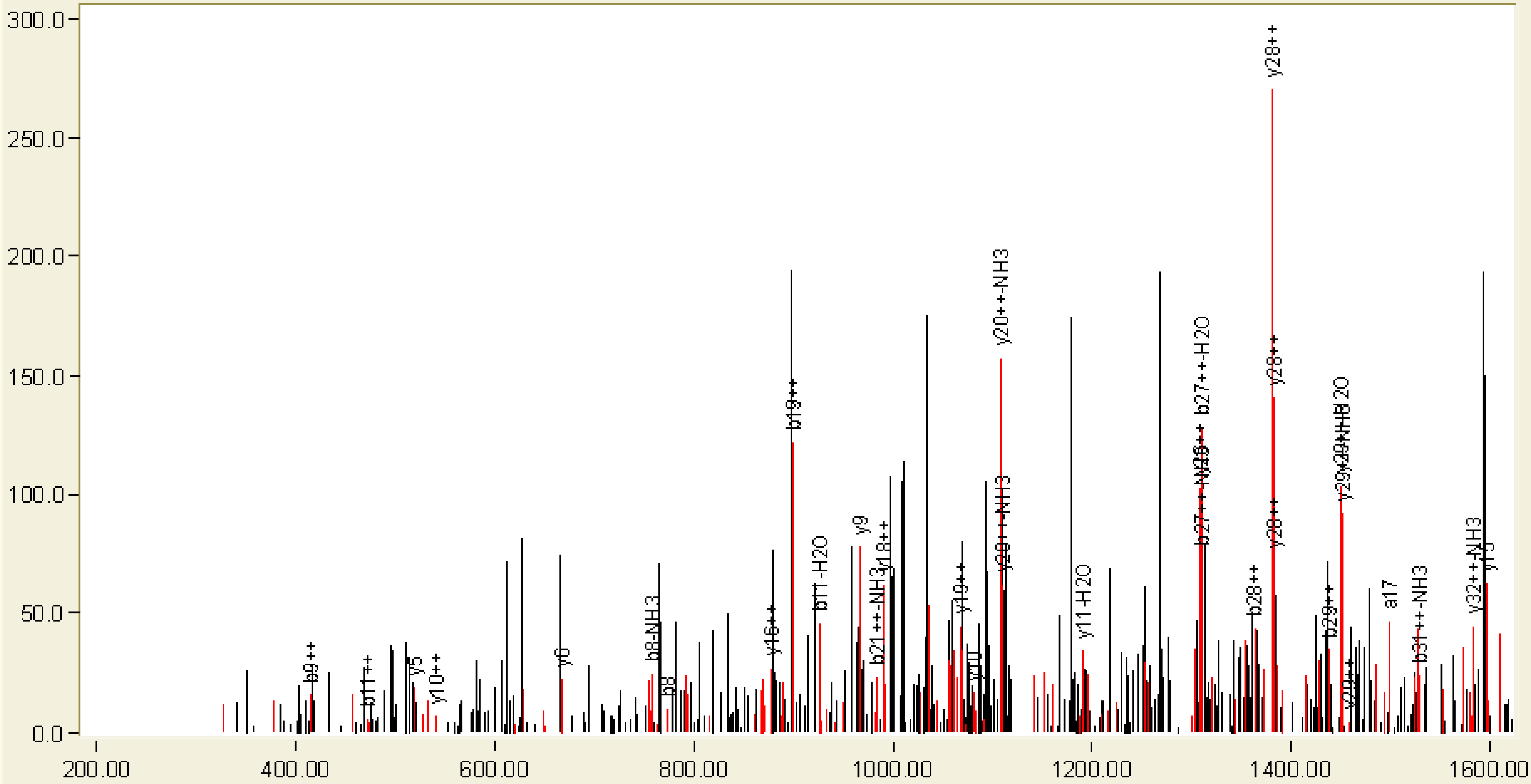

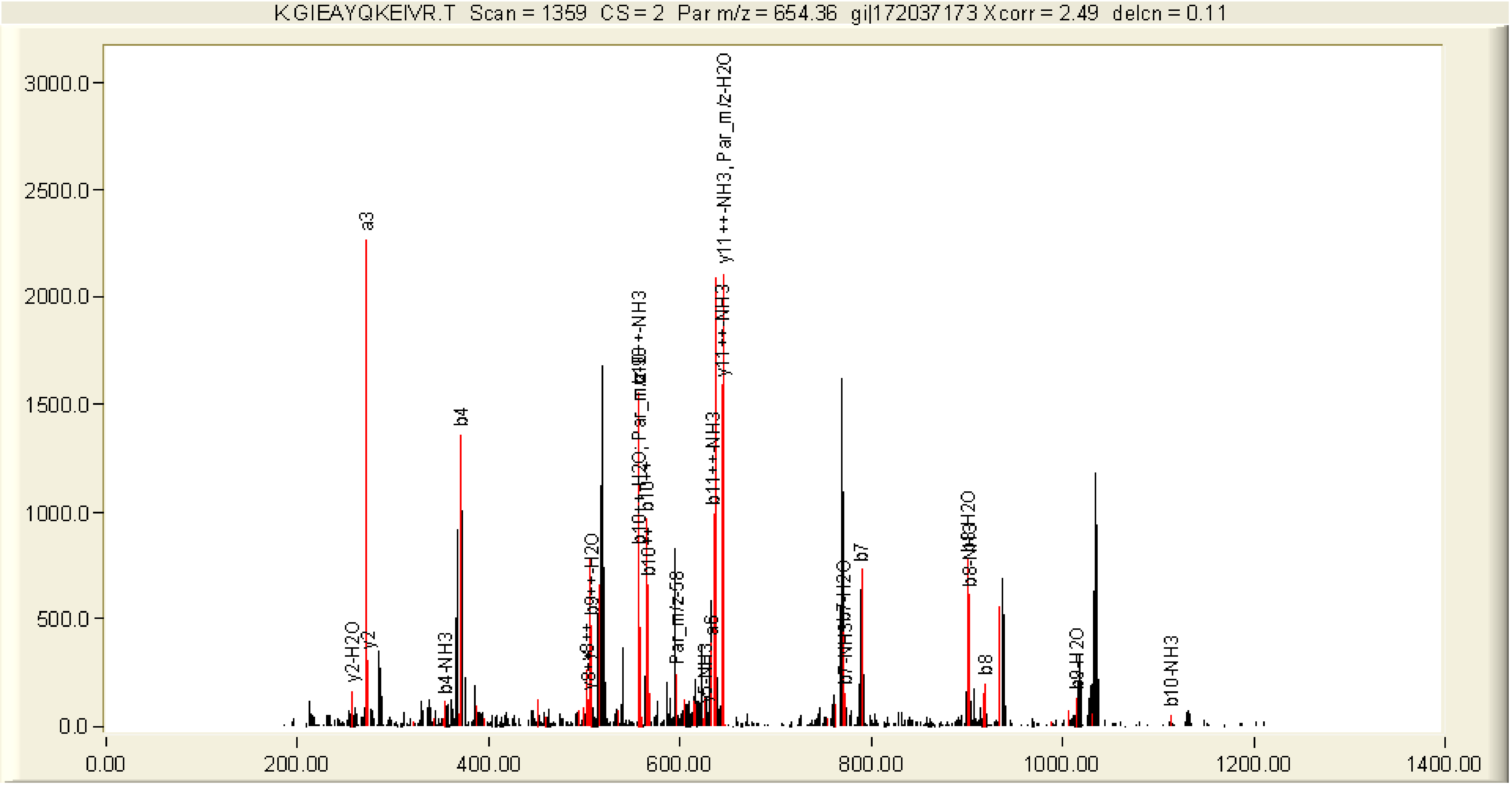

KGIEAYQKEIVR.T Scan = 1359 CS = 2 Par m/z = 654.36 gi|172037173 Xcorr = 2.49 delcn = 0.11
3000.0
2500.0
2000.0
1500.0
1000.0
500.0
0.0
0.00
200.00
400.00
600.00
800.00
1000.00
1200.00
1400.00
a3
y2-H2O
y2
b4
b4-NH3
b9++-H2O
b10++-H2O; Par_m/z-H2O
b10++
Par_m/z-58
a6
b11++-NH3
y11++-NH3
y11++-NH3, Par_m/z-H2O
b7-NH3
b7
b8-NH3
b8-H2O
b8
b9-H2O
b10-NH3

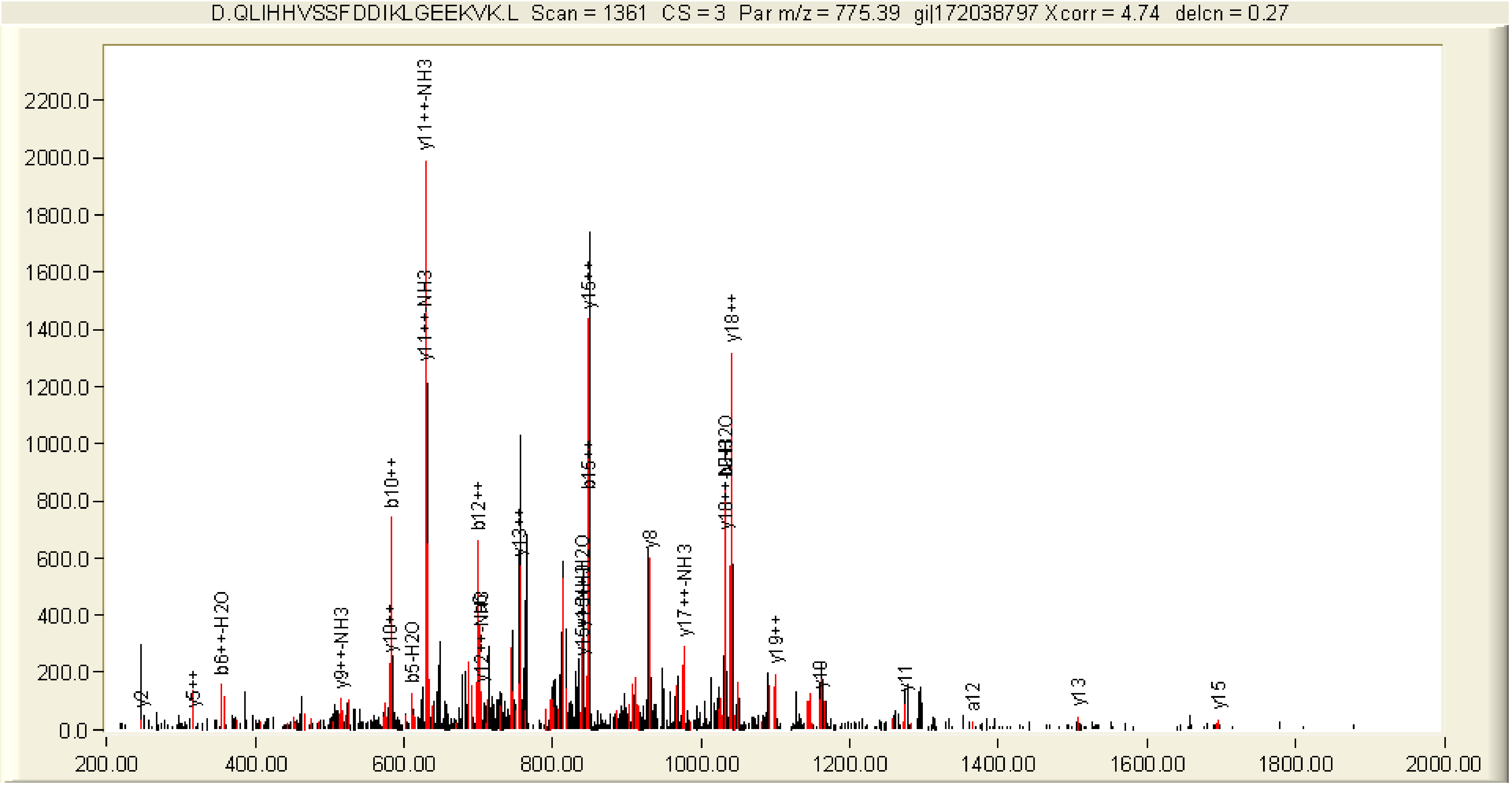
D.QLIHHVSSFDDIKLGEEKVK.L Scan = 1361 CS = 3 Par m/z = 775.39 gi|172038797 Xcorr = 4.74 delcn = 0.27
2200.0
2000.0
1800.0
1600.0
1400.0
1200.0
1000.0
800.0
600.0
400.0
200.0
0.0
200.00
400.00
600.00
800.00
1000.00
1200.00
1400.00
1600.00
1800.00
2000.00
y11++-NH3
y11++
y15++
y18++
b15++
b10++
b12++
y13++
y8
y17++-NH3
y10++
b5-H2O
b6++-H2O
y9++-NH3
y12++-NH3
y19++
y5++
y2
y11
a12
y13
y15

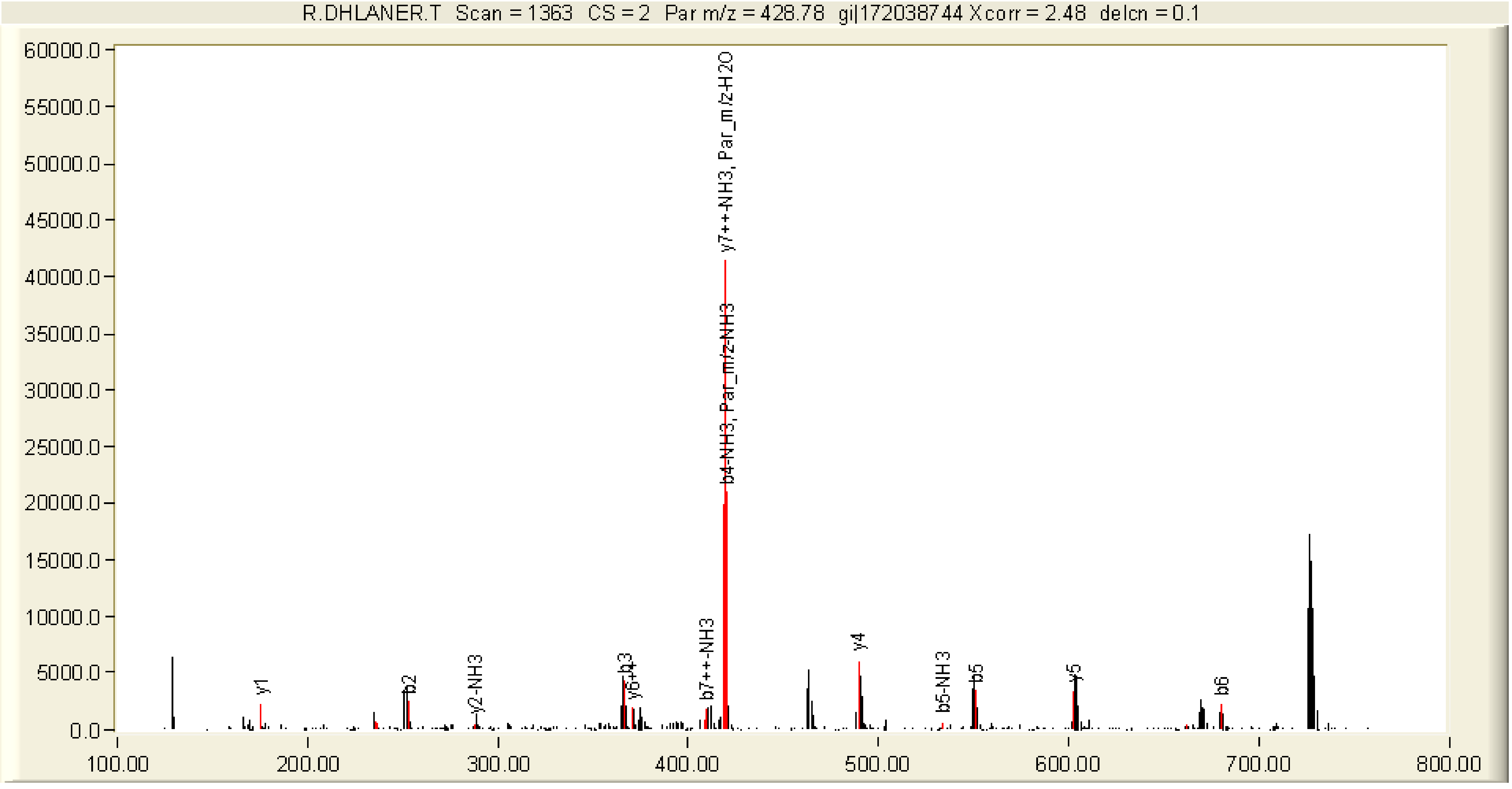

R.DHLANER.T Scan = 1363 CS = 2 Par m/z = 428.78 gi|172038744 Xcorr = 2.48 delcn = 0.1
60000.0
55000.0
50000.0
45000.0
40000.0
35000.0
30000.0
25000.0
20000.0
15000.0
10000.0
5000.0
0.0
100.00
200.00
300.00
400.00
500.00
600.00
700.00
800.00
y7++-NH3, Par_m/z-H2O
b4-NH3, Par_m/z-NH3
y1
b2
y2-NH3
b3
y6++
b7++-NH3
y4
b5-NH3
b5
y5
b6

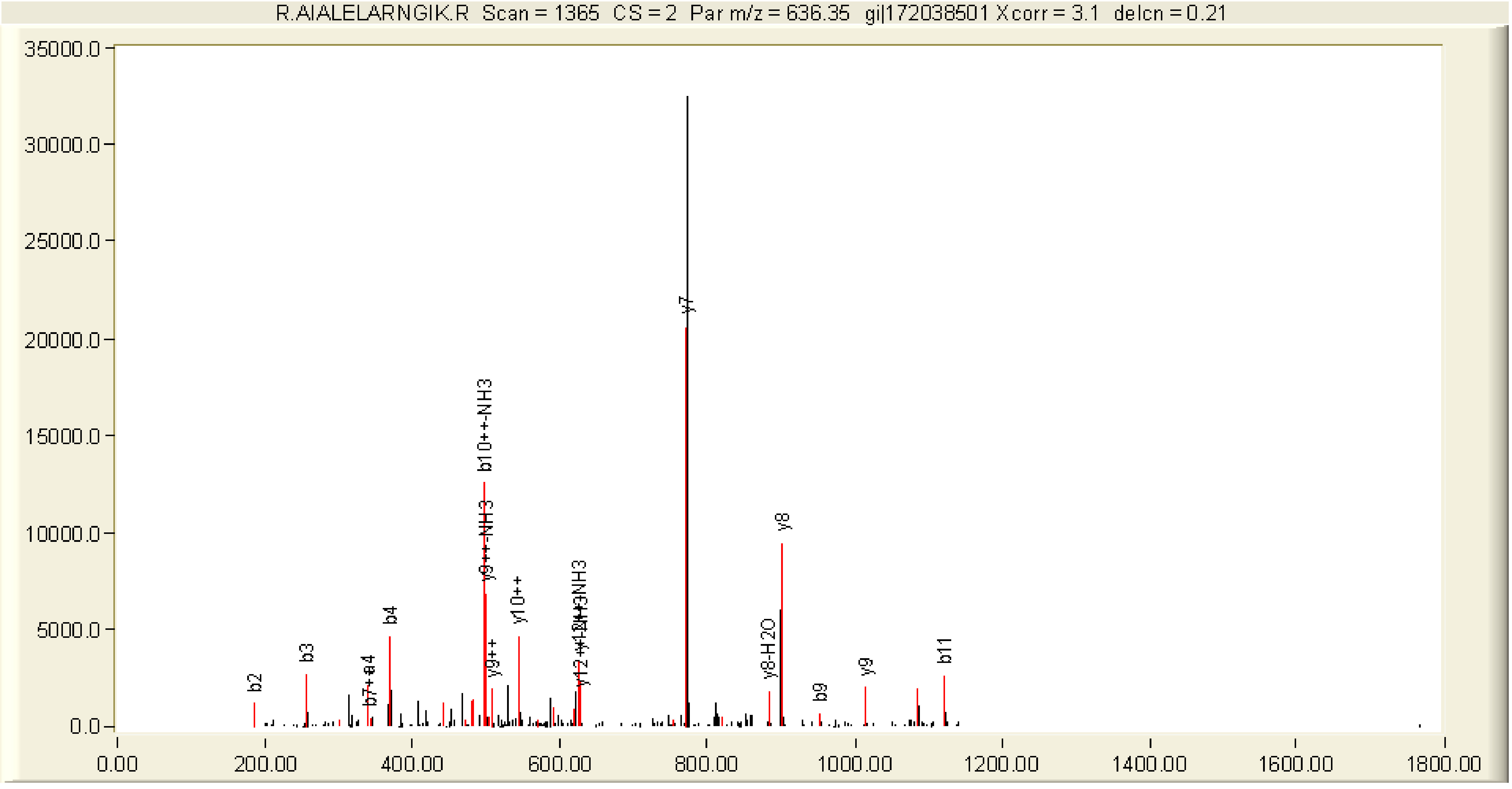

R.AIALELARNGIK.R Scan = 1365 CS = 2 Par m/z = 636.35 gi|172038501 Xcorr = 3.1 delcn = 0.21
35000.0
30000.0
25000.0
20000.0
15000.0
10000.0
5000.0
0.0
0.00
200.00
400.00
600.00
800.00
1000.00
1200.00
1400.00
1600.00
1800.00
b2
b3
b4
b10++-NH3
y9++-NH3
y9++
y10++
y7
y8-H2O
y8
b9
y9
b11

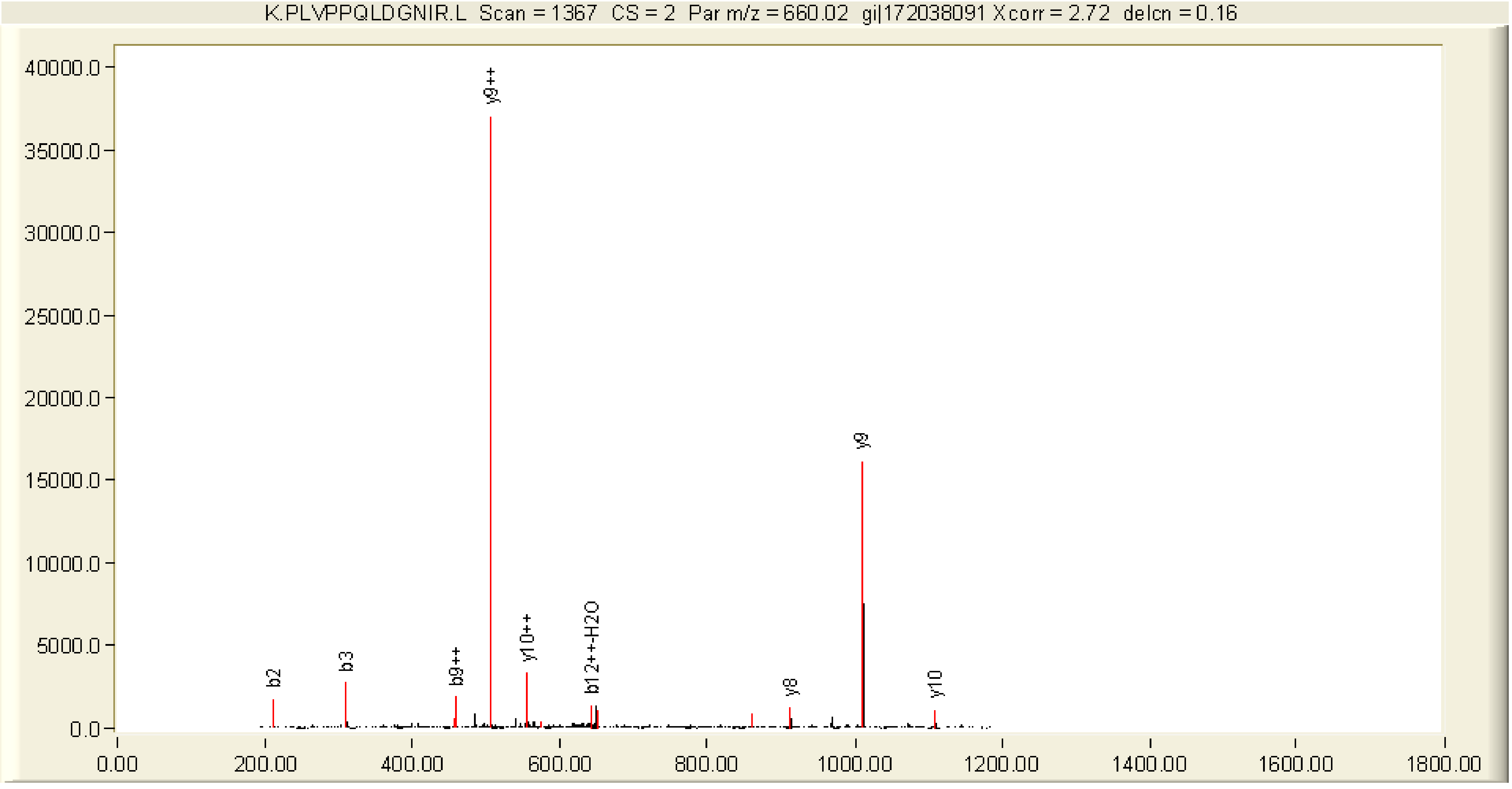
K.PLVPPQLDGNIR.L Scan = 1367 CS = 2 Par m/z = 660.02 gi|172038091 Xcorr = 2.72 delcn = 0.16
40000.0
35000.0
30000.0
25000.0
20000.0
15000.0
10000.0
5000.0
0.0
0.00
200.00
400.00
600.00
800.00
1000.00
1200.00
1400.00
1600.00
1800.00
y9++
y9
b2
b3
b9++
y10++
b12++-H2O
y8
y10

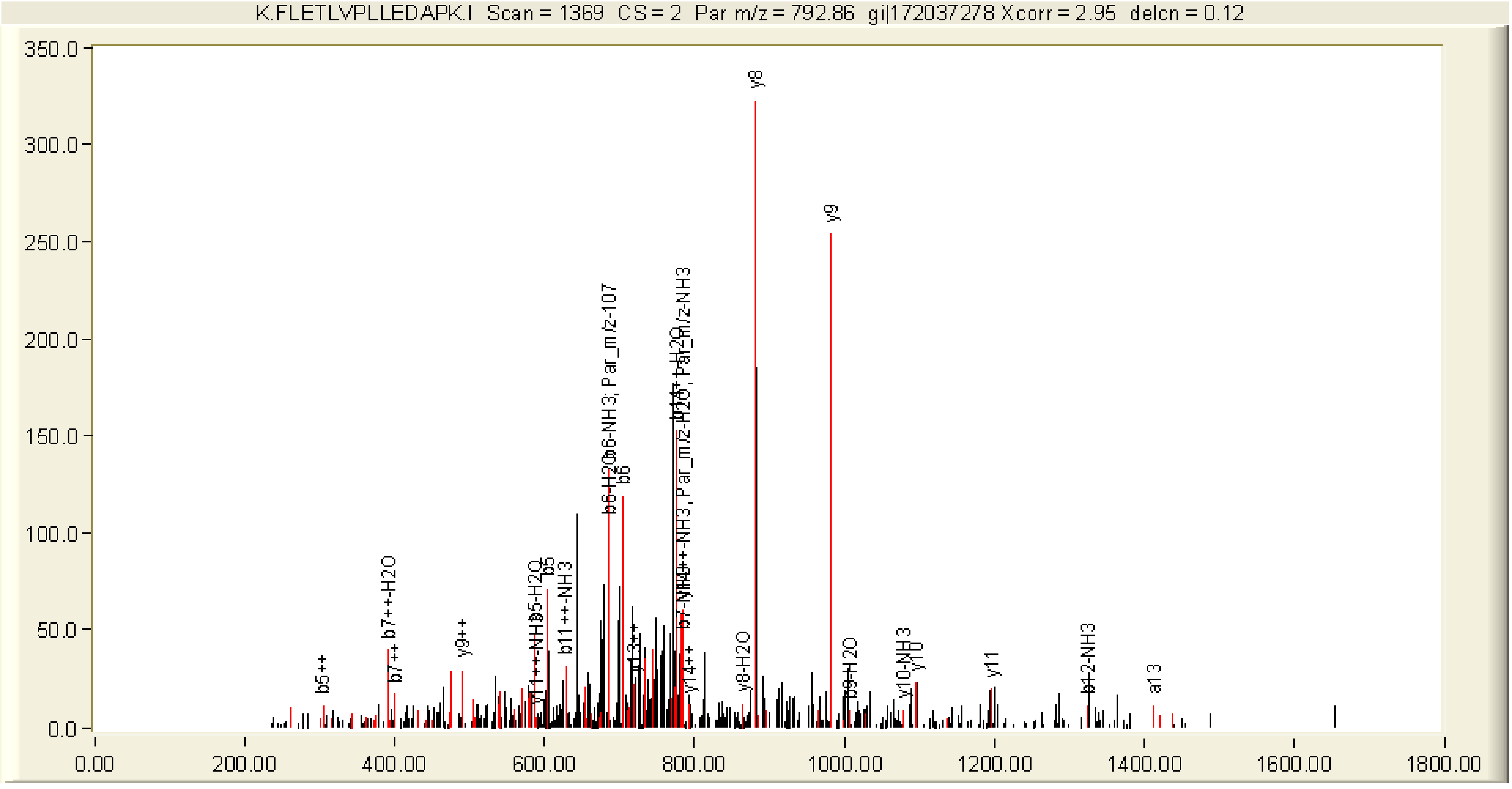
K.FLETLVPLLEDAPK.I Scan = 1369 CS = 2 Par m/z = 792.86 gi|172037278 Xcorr = 2.95 delcn = 0.12
350.0
300.0
250.0
200.0
150.0
100.0
50.0
0.0
0.00
200.00
400.00
600.00
800.00
1000.00
1200.00
1400.00
1600.00
1800.00
y8
y9
b5++
b7++
b7++-H2O
y9++
b5
b11++-NH3
b6
b6-H2O
b6-NH3; Par_m/z-107
y14++
y8-H2O
b9-H2O
y10-NH3
y10
y11
b12-NH3
a13

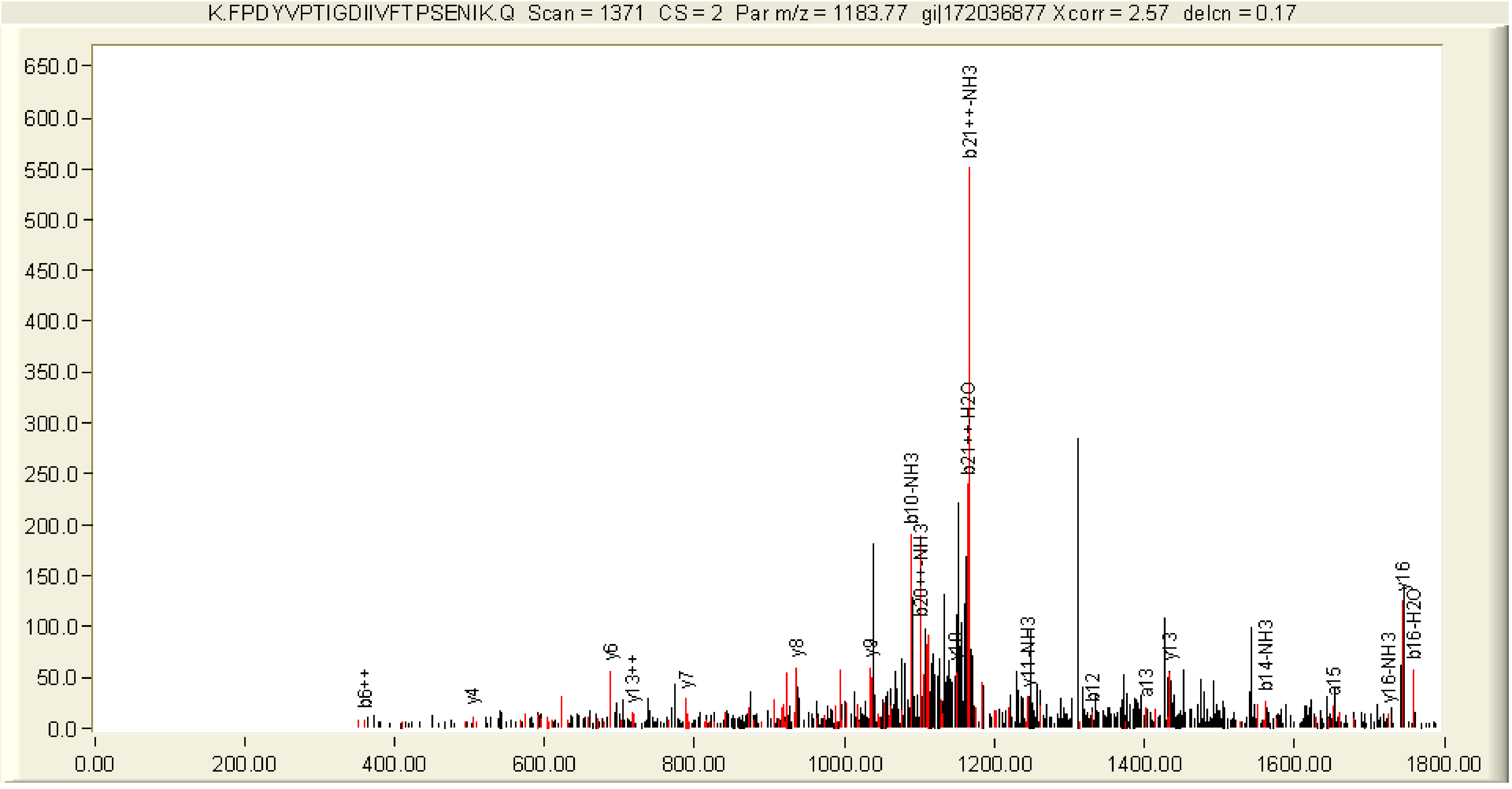
K.FPDYVPTIGDIIVFTPSENIK.Q Scan = 1371 CS = 2 Par m/z = 1183.77 gi|172036877 Xcorr = 2.57 delcn = 0.17
b21++-NH3
b21++-H2O
b10-NH3
b20++-NH3
b6++
y4
y6
y13++
y7
y8
y9
y10
y11-NH3
b12
a13
y13
b14-NH3
a15
y16-NH3
y16
b16-H2O
0.0
50.0
100.0
150.0
200.0
250.0
300.0
350.0
400.0
450.0
500.0
550.0
600.0
650.0
0.00
200.00
400.00
600.00
800.00
1000.00
1200.00
1400.00
1600.00
1800.00

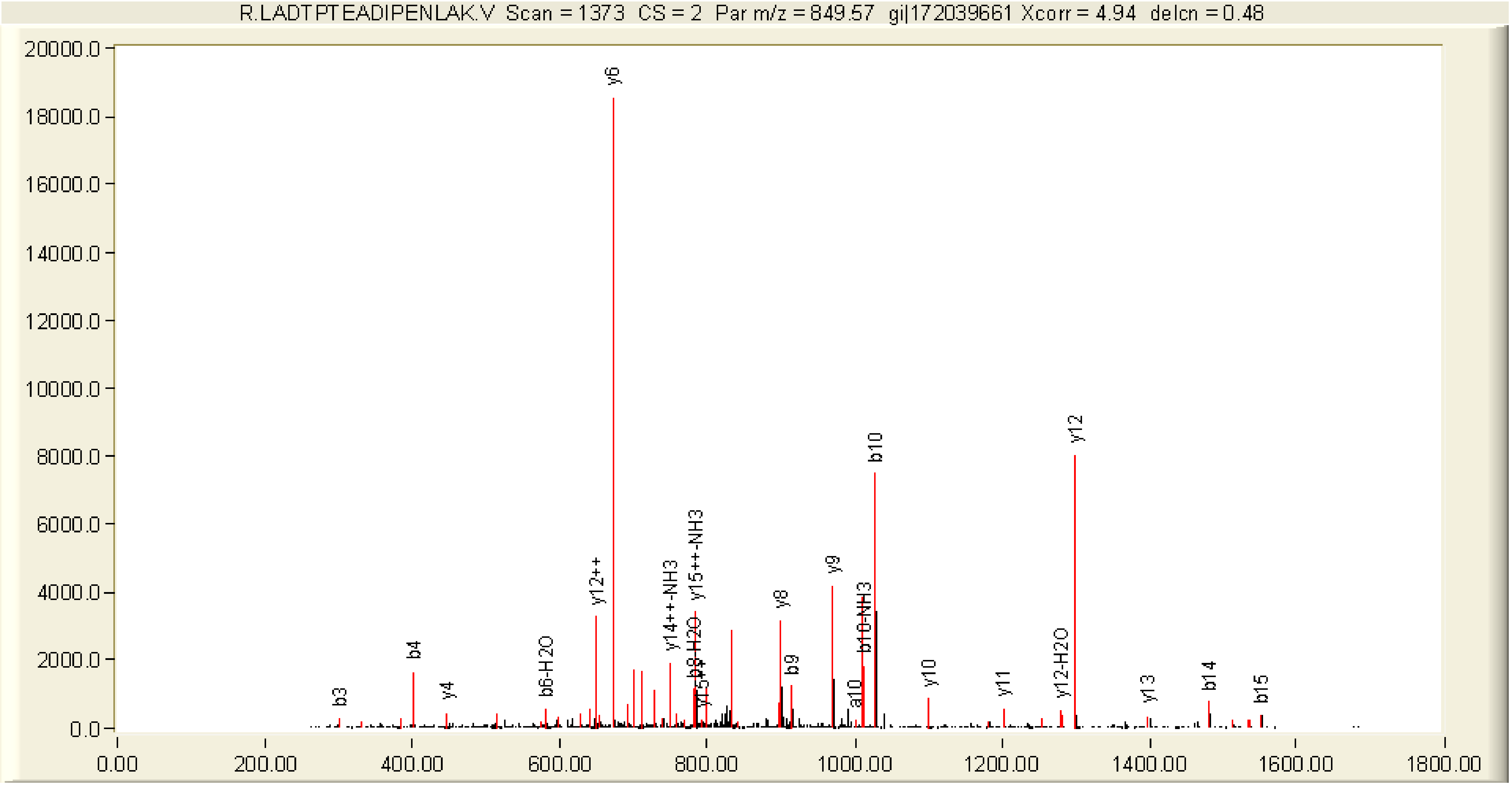
R.LADTPTEADIPENLAK.V Scan = 1373 CS = 2 Par m/z = 849.57 gi|172039661 Xcorr = 4.94 delcn = 0.48
y6
y12
b10
y15++-NH3
y14++-NH3
y12++
y9
y8
b10-NH3
b8-H2O
y15++
b9
a10
b4
y4
b3
b6-H2O
y10
y11
y12-H2O
y13
b14
b15
20000.0
18000.0
16000.0
14000.0
12000.0
10000.0
8000.0
6000.0
4000.0
2000.0
0.0
0.00
200.00
400.00
600.00
800.00
1000.00
1200.00
1400.00
1600.00
1800.00

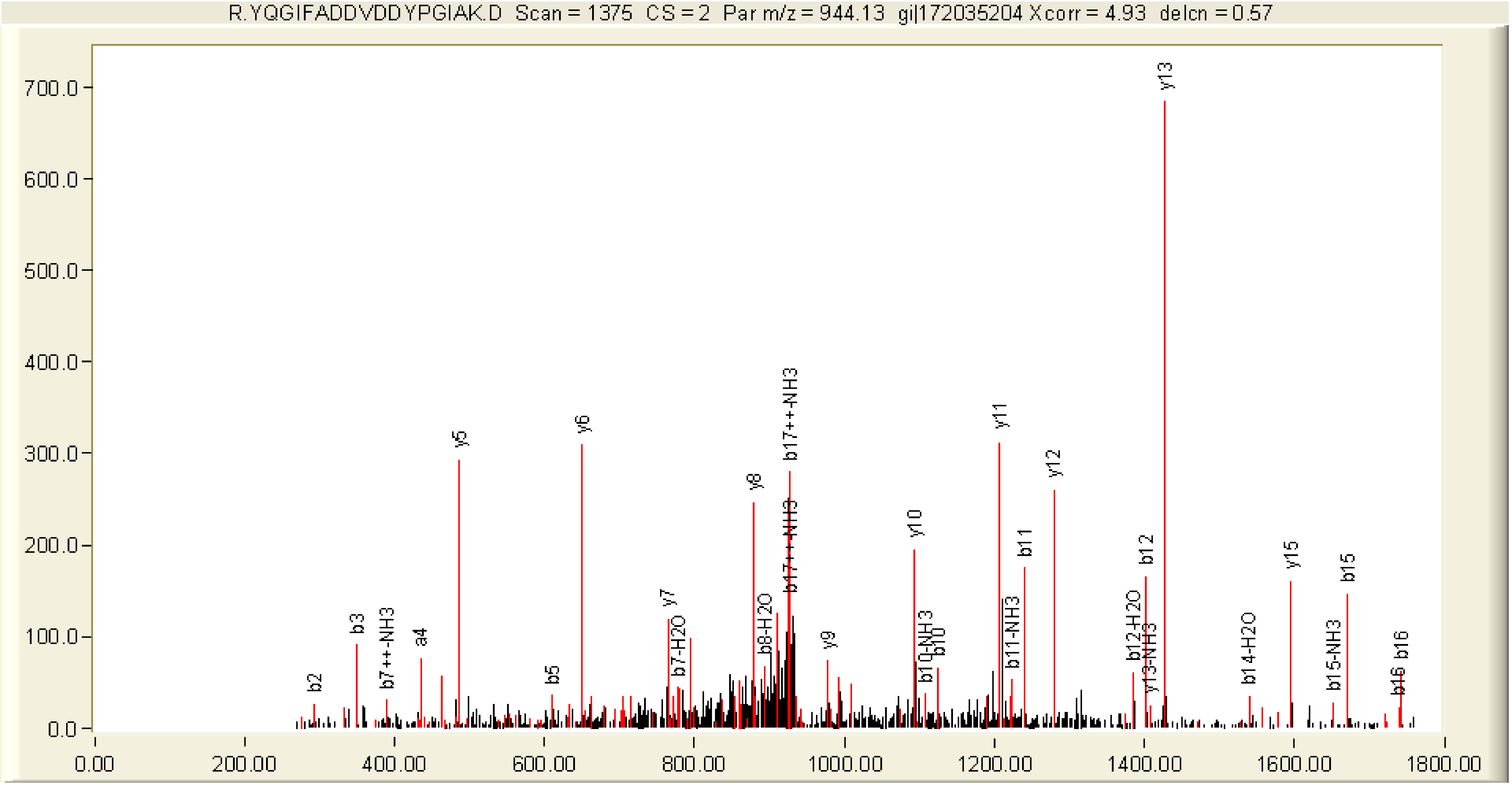
R.YQGIFADDVDDYPGIAK.D Scan = 1375 CS = 2 Par m/z = 944.13 gi|172035204 Xcorr = 4.93 delcn = 0.57
700.0
600.0
500.0
400.0
300.0
200.0
100.0
0.0
0.00
200.00
400.00
600.00
800.00
1000.00
1200.00
1400.00
1600.00
1800.00
b2
b3
b7++-NH3
a4
y5
b5
y6
y7
b7-H2O
b8-H2O
y8
b17++-NH3
y9
y10
b10-NH3
b10
y11
b11-NH3
b11
y12
b12-H2O
y13-NH3
b12
y13
b14-H2O
y15
b15-NH3
b15
b16
b16

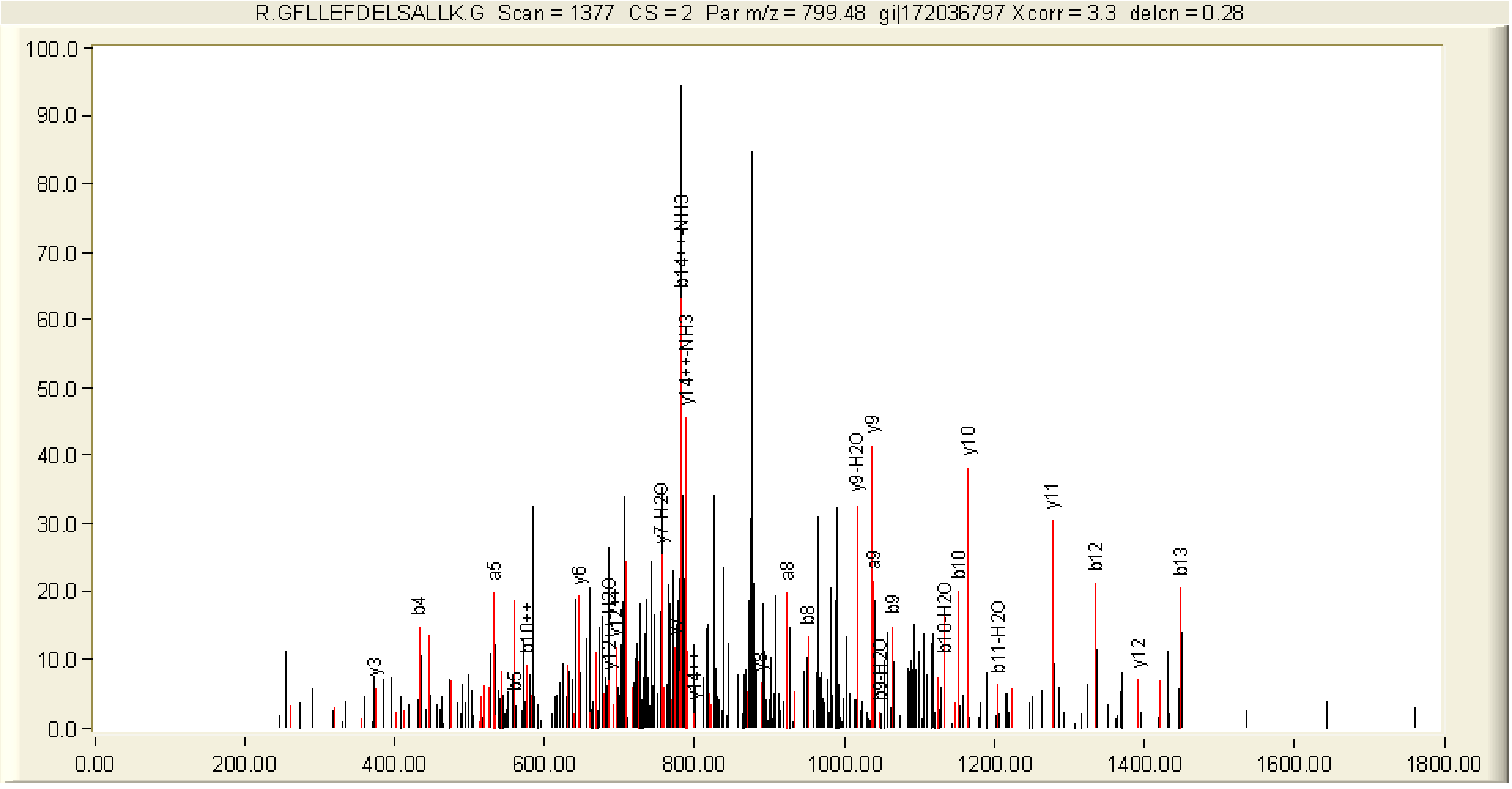
R.GFLLEFDELSALLK.G Scan = 1377 CS = 2 Par m/z = 799.48 gi|172036797 Xcorr = 3.3 delcn = 0.28
b14++-NH3
y14++-NH3
y9
y10
y9-H2O
y11
y7-H2O
a5
y6
a8
a9
b10
b12
b13
b4
b9
b8
b10-H2O
b11-H2O
y12
y3
b10++
b5
0.0
10.0
20.0
30.0
40.0
50.0
60.0
70.0
80.0
90.0
100.0
0.00
200.00
400.00
600.00
800.00
1000.00
1200.00
1400.00
1600.00
1800.00

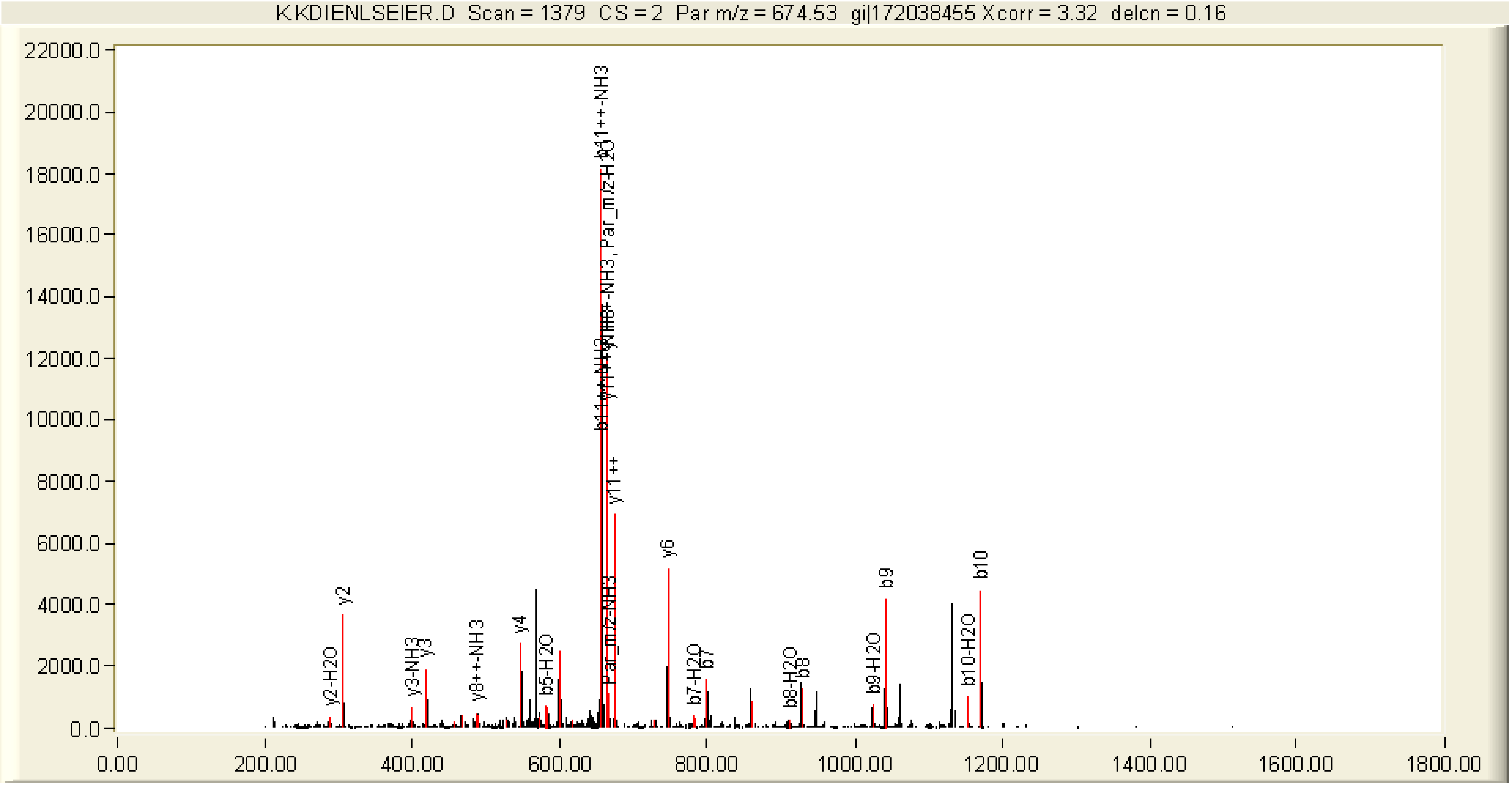

K KDIENLSEIER.D Scan = 1379 CS = 2 Par m/z = 674.53 gi|172038455 Xcorr = 3.32 delcn = 0.16
22000.0
20000.0
18000.0
16000.0
14000.0
12000.0
10000.0
8000.0
6000.0
4000.0
2000.0
0.0
0.00
200.00
400.00
600.00
800.00
1000.00
1200.00
1400.00
1600.00
1800.00
y2-H2O
y2
y3-NH3
y3
y8++-NH3
y4
b5-H2O
Par_m/z-NH3
y11++
y6
b7-H2O
b7
b8-H2O
b8
b9-H2O
b9
b10-H2O
b10

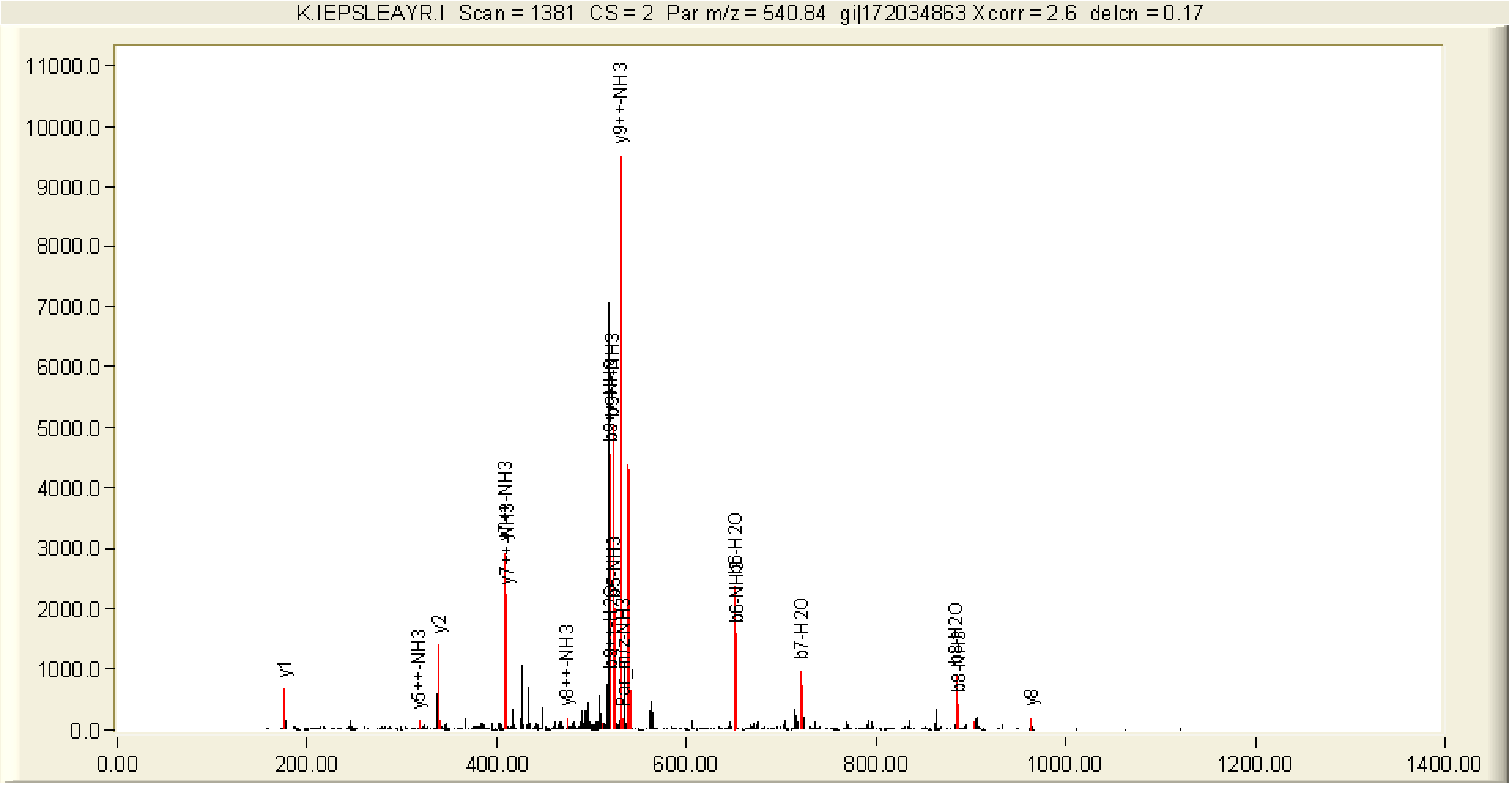
K.IEPSLEAYR.I Scan = 1381 CS = 2 Par m/z = 540.84 gi|172034863 Xcorr = 2.6 delcn = 0.17
11000.0
10000.0
9000.0
8000.0
7000.0
6000.0
5000.0
4000.0
3000.0
2000.0
1000.0
0.0
0.00
200.00
400.00
600.00
800.00
1000.00
1200.00
1400.00
y1
y5++-NH3
y2
y8++-NH3
y9++-NH3
b7-H2O
y8

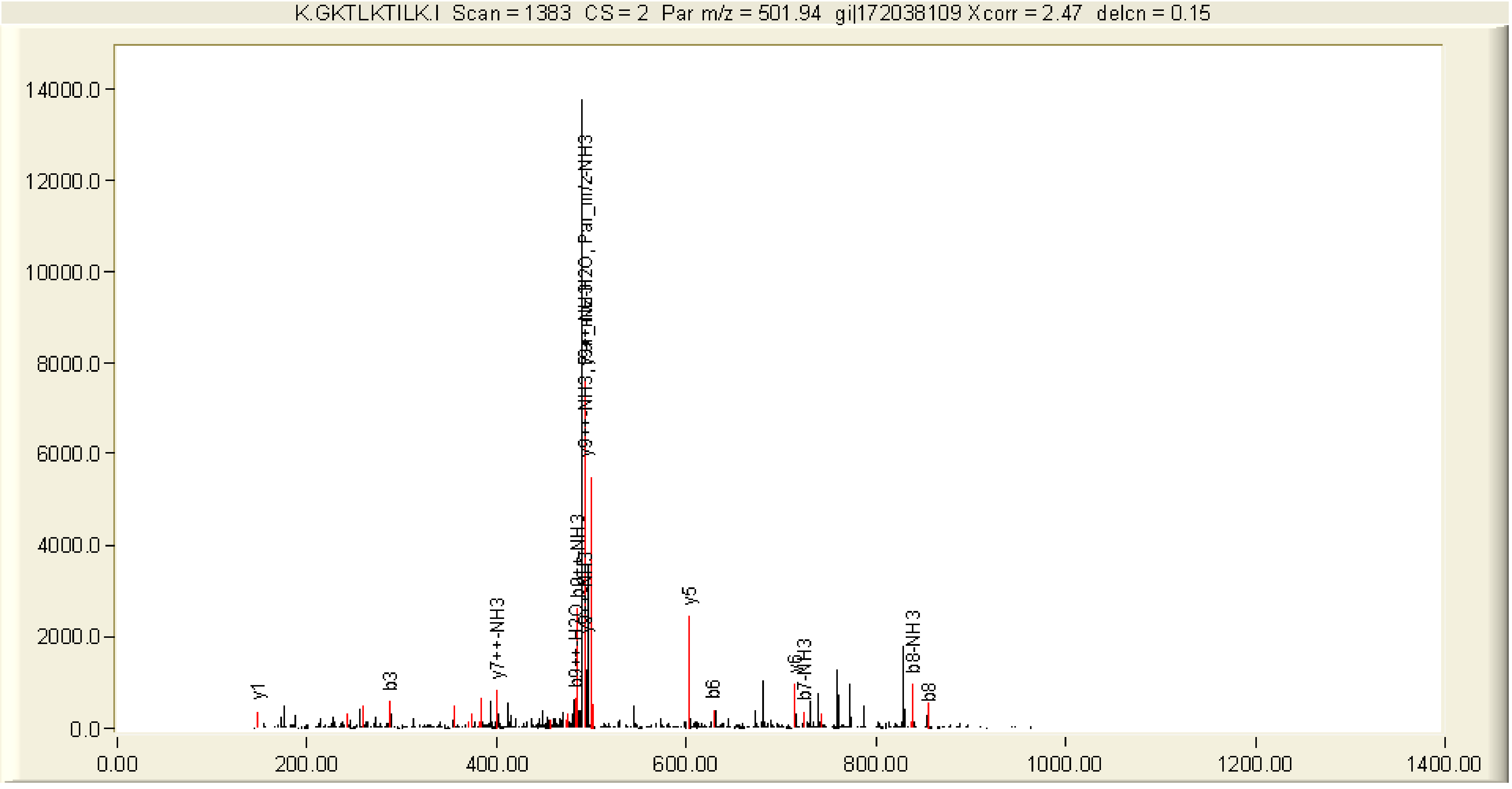

K.GKTLKTILK.I Scan = 1383 CS = 2 Par m/z = 501.94 gi|172038109 Xcorr = 2.47 delcn = 0.15
14000.0
12000.0
10000.0
8000.0
6000.0
4000.0
2000.0
0.0
0.00
200.00
400.00
600.00
800.00
1000.00
1200.00
1400.00
y1
b3
y7++-NH3
y5
b6
b7-NH3
b8-NH3
b8

R.SEDDPTGDSEVFGIVPALK.H Scan = 1385 CS = 2 Par m/z = 989.14 gi|172039025 Xcorr = 5.05 delcn = 0.49

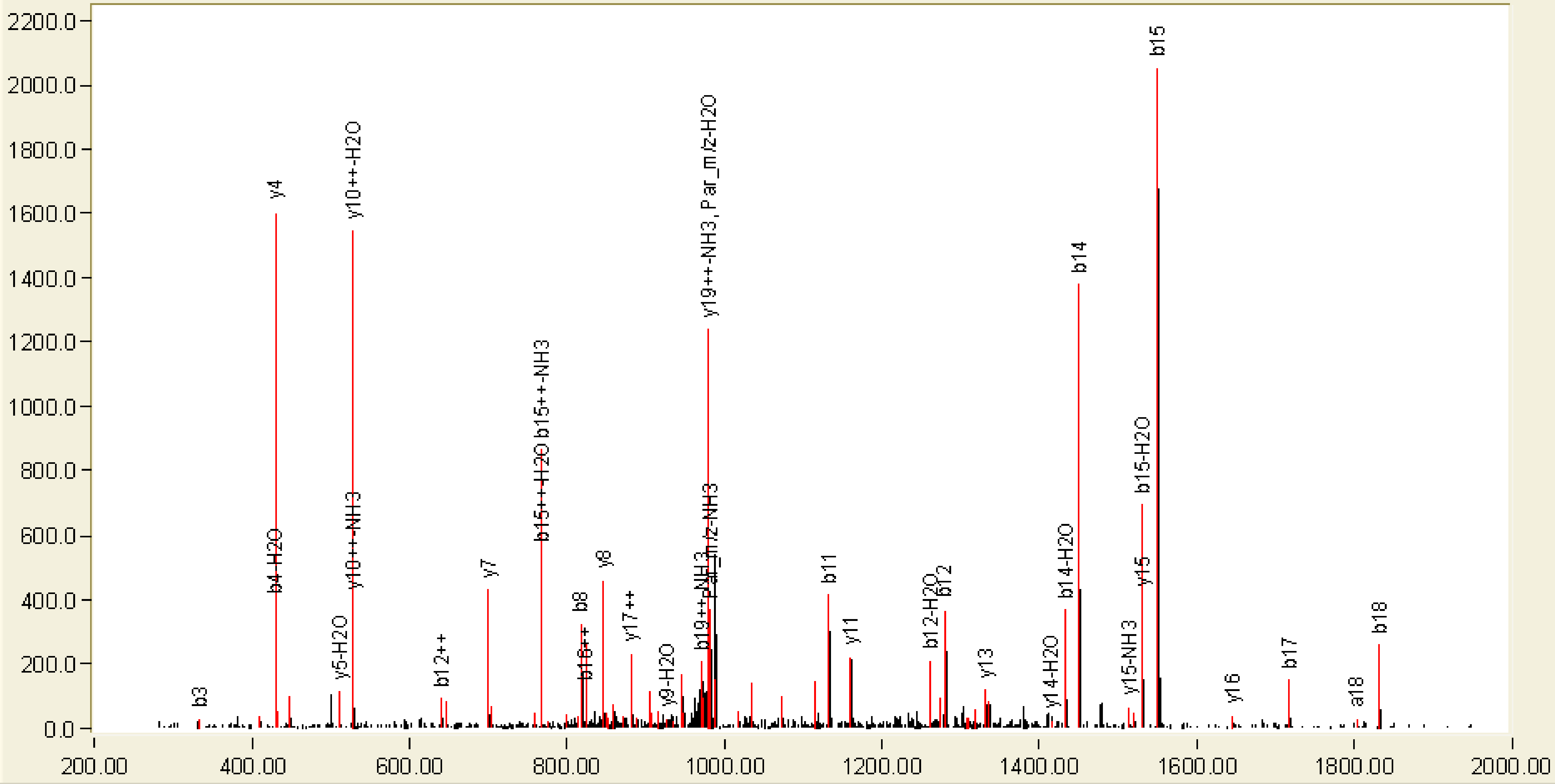

K.EELTPGRIRFIGAIFASVSVPLLYAVAR.E Scan = 1387 CS = 3 Par m/z = 1016.57 gi|172036531 Xcorr = 3.23 delcn = 0.28

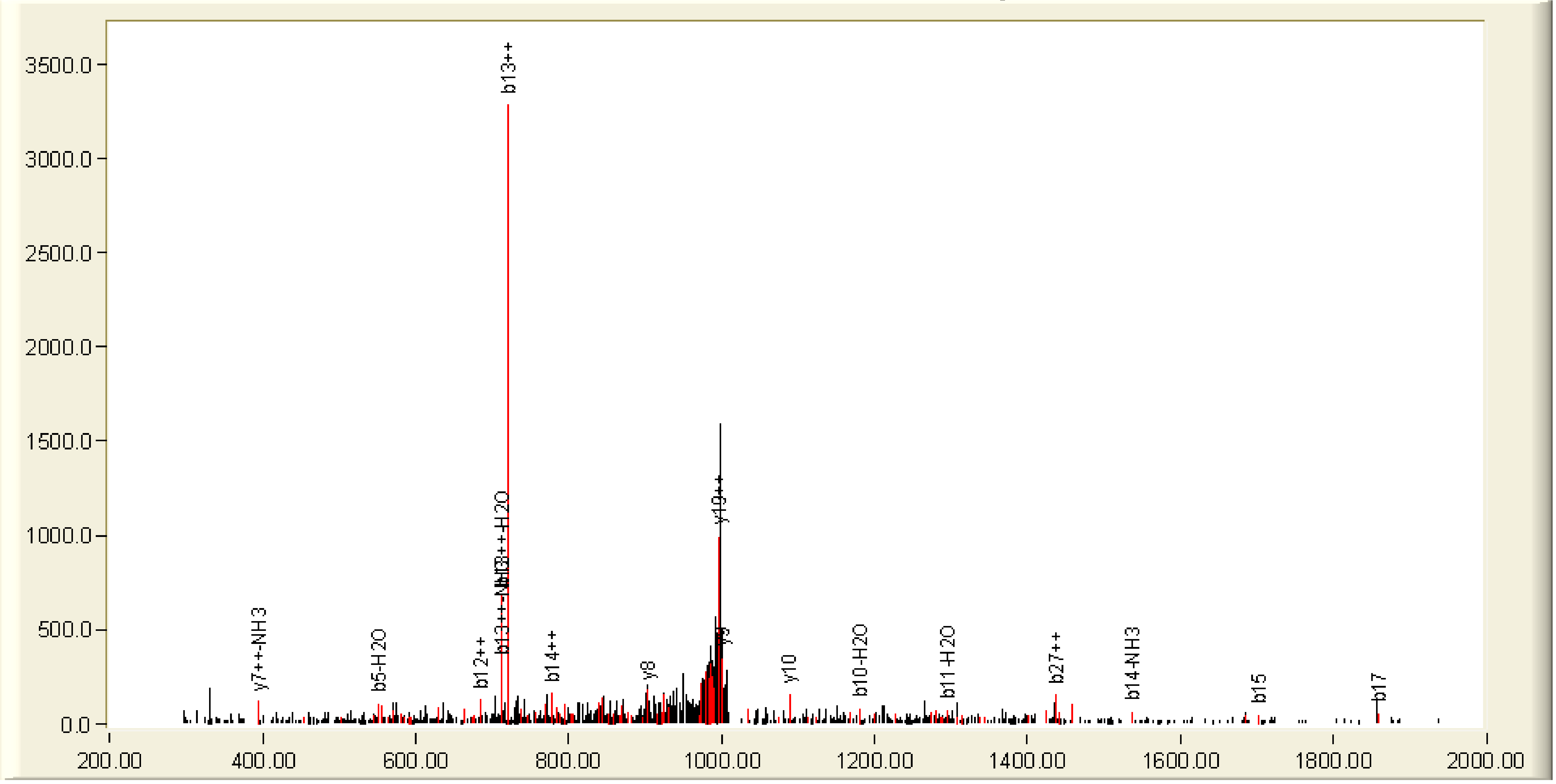

KQSYDLVYNHLQKI Scan = 1389 CS = 2 Par m/z = 753.86 gi|172055102 Xcorr = 2.8 delcn = 0.3

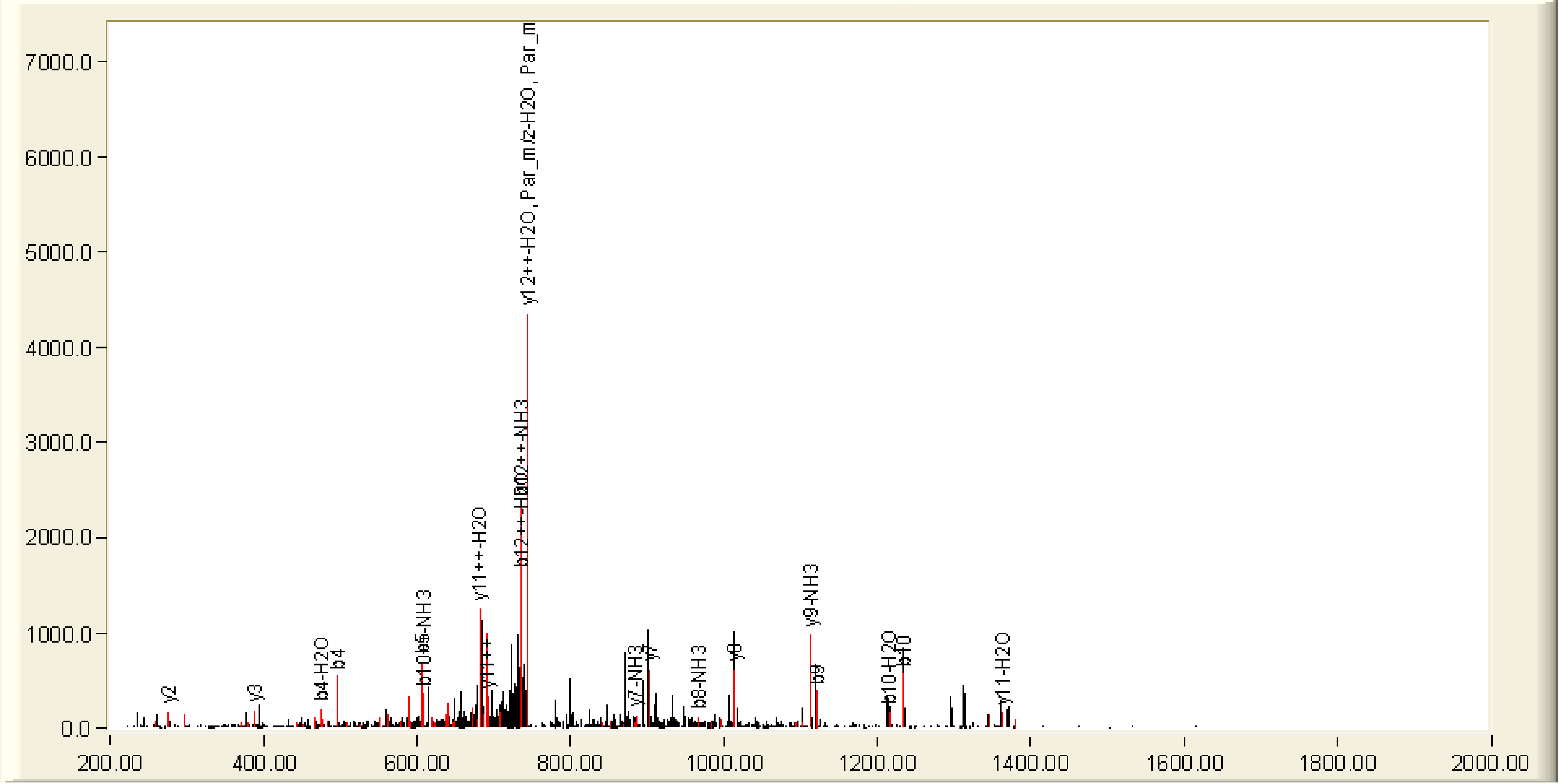

R.ISINSIISVGGEEK.K Scan = 1391 CS = 2 Par m/z = 723.77 gi|172037589 Xcorr = 3.73 delcn = 0.32

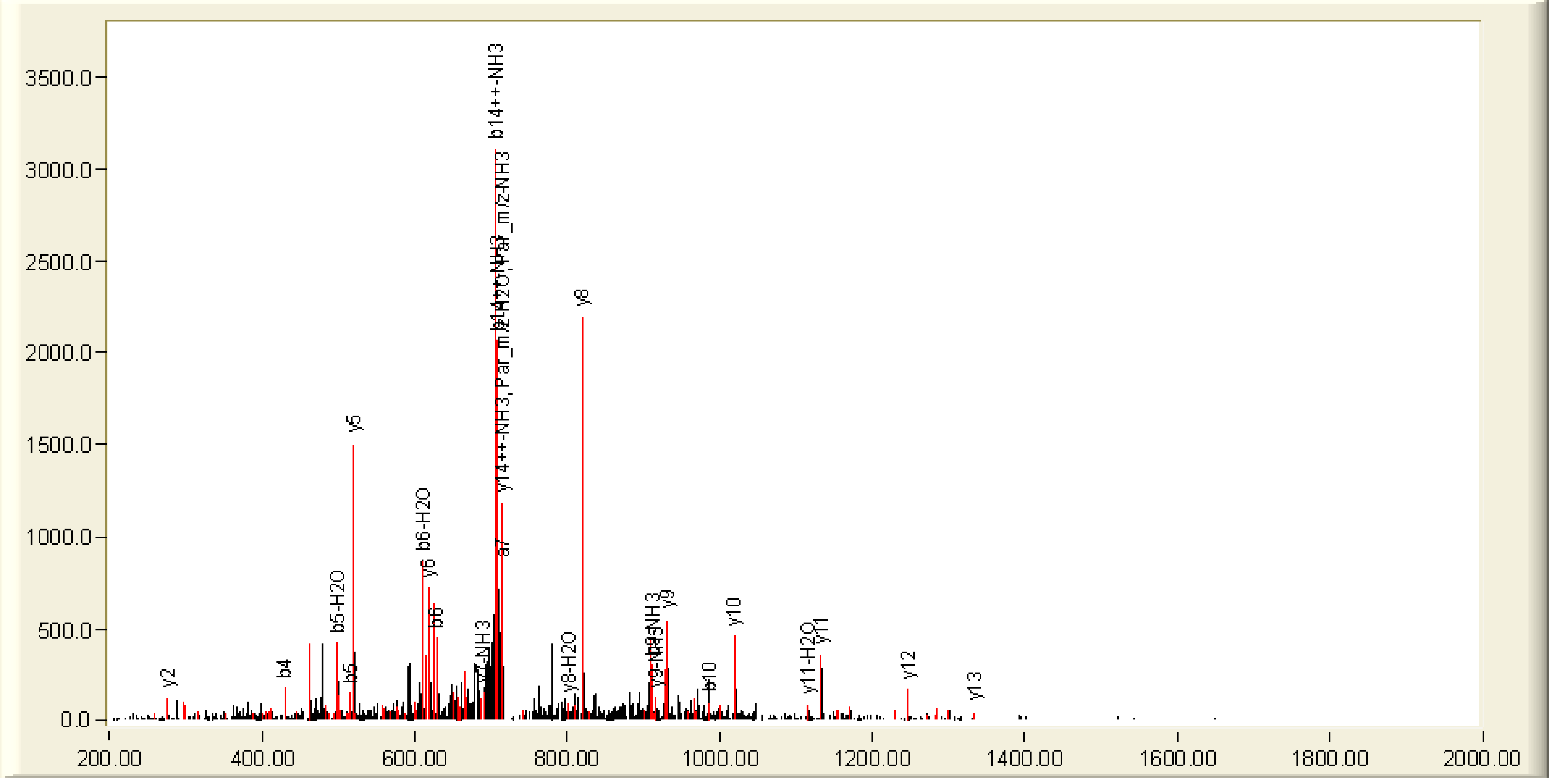

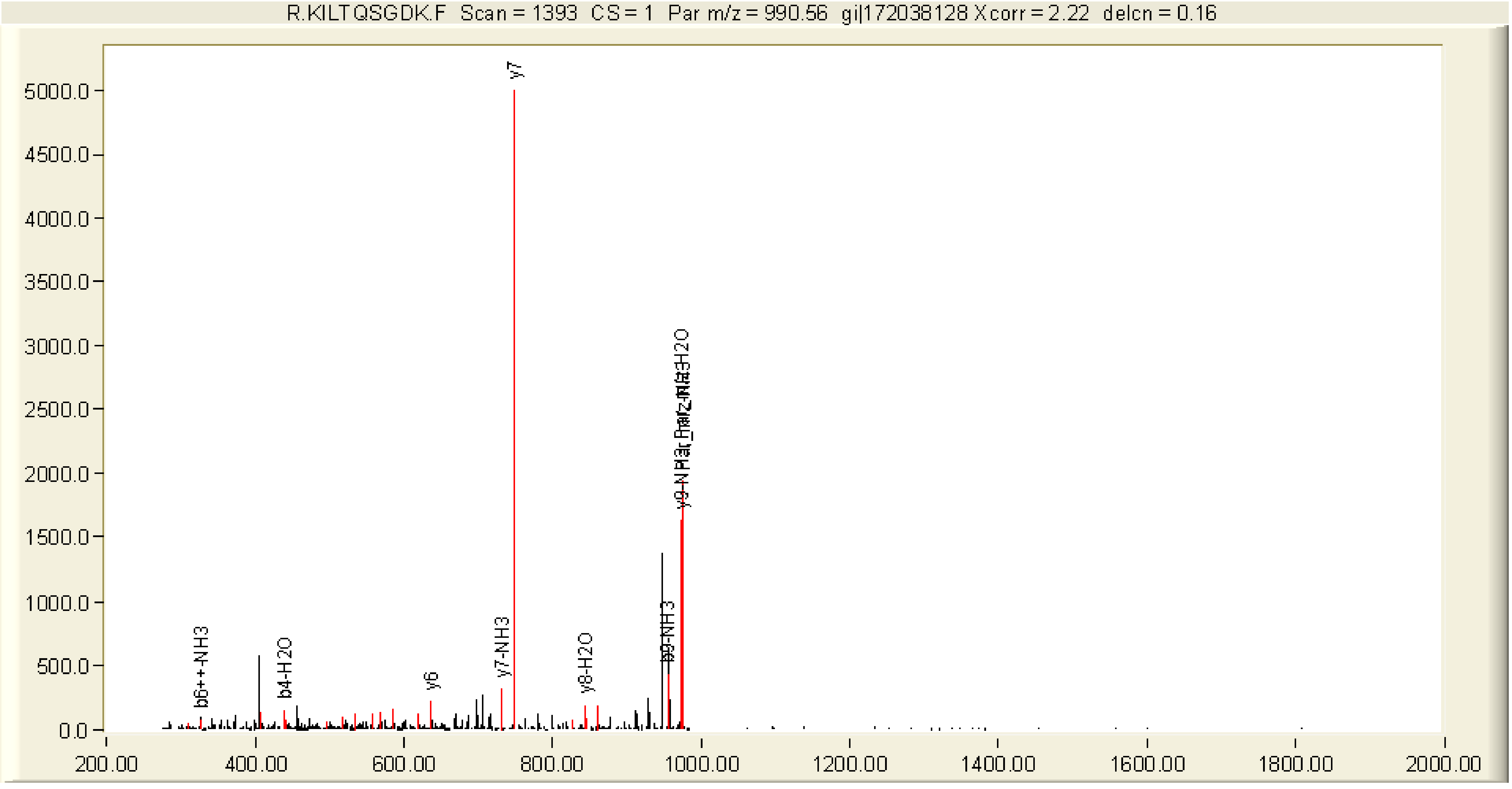
R.KILTQSGDK.F Scan = 1393 CS = 1 Par m/z = 990.56 gi|172038128 Xcorr = 2.22 delcn = 0.16
y7
b6++-NH3
b4-H2O
y6
y7-NH3
y8-H2O
b9-NH3
y9-NH3
5000.0
4500.0
4000.0
3500.0
3000.0
2500.0
2000.0
1500.0
1000.0
500.0
0.0
200.00
400.00
600.00
800.00
1000.00
1200.00
1400.00
1600.00
1800.00
2000.00

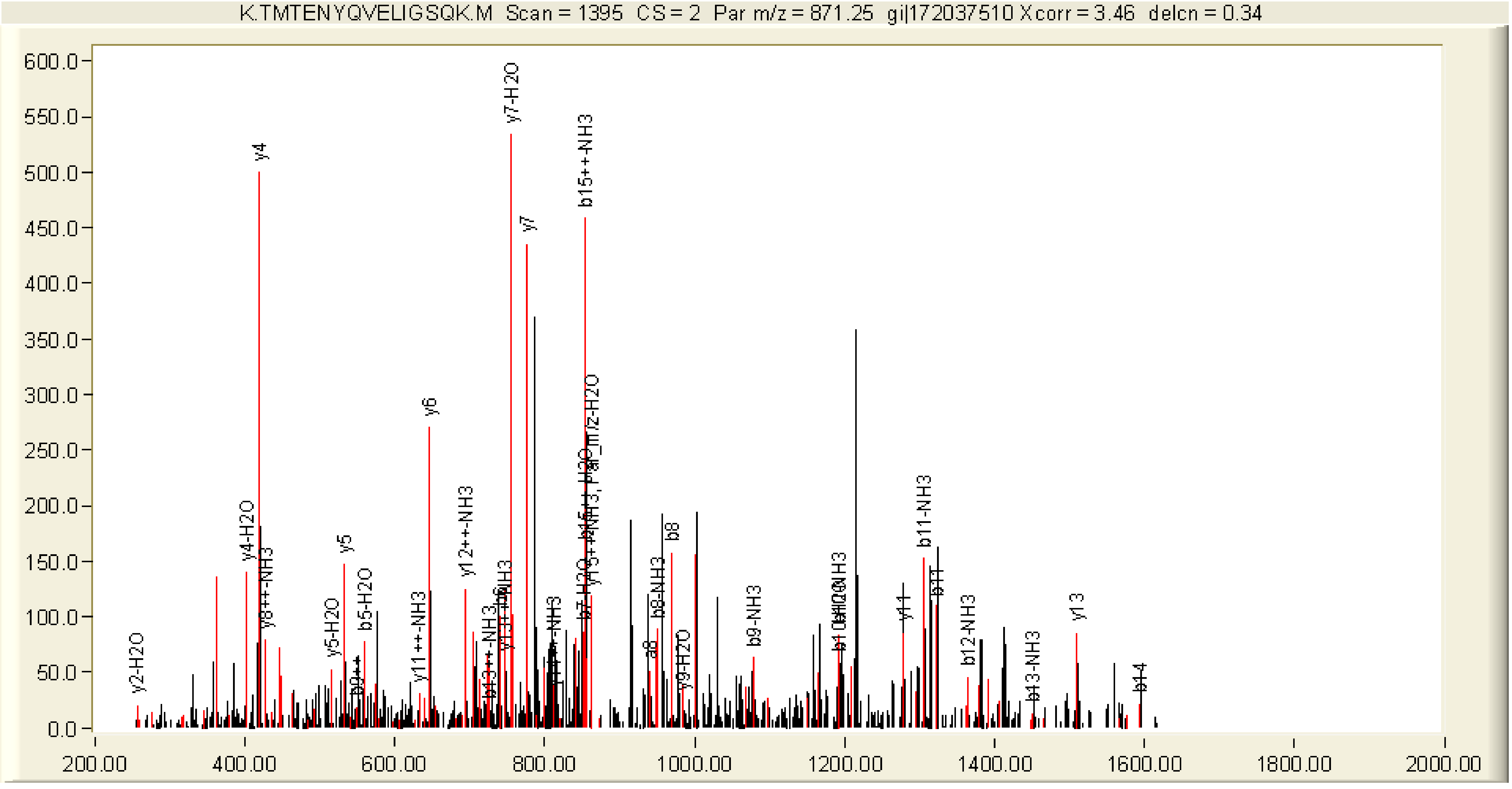

K.TMTENYQVELIGSQK.M Scan = 1395 CS = 2 Par m/z = 871.25 gi|172037510 Xcorr = 3.46 delcn = 0.34
600.0
550.0
500.0
450.0
400.0
350.0
300.0
250.0
200.0
150.0
100.0
50.0
0.0
200.00
400.00
600.00
800.00
1000.00
1200.00
1400.00
1600.00
1800.00
2000.00
y2-H2O
y4-H2O
y4
y8++-NH3
y5-H2O
y5
b5-H2O
y11 ++-NH3
y6
y12++-NH3
y7-H2O
y7
b15++-NH3
a8
b8-NH3
b8
y9-H2O
b9-NH3
b11-NH3
y11
b12-NH3
b13-NH3
y13
b14

-.MIKPELFDLVELLINLPEAK.Q Scan = 1397 CS = 2 Par m/z = 1163.58 gi|172037174 Xcorr = 3.74 delcn = 0.12

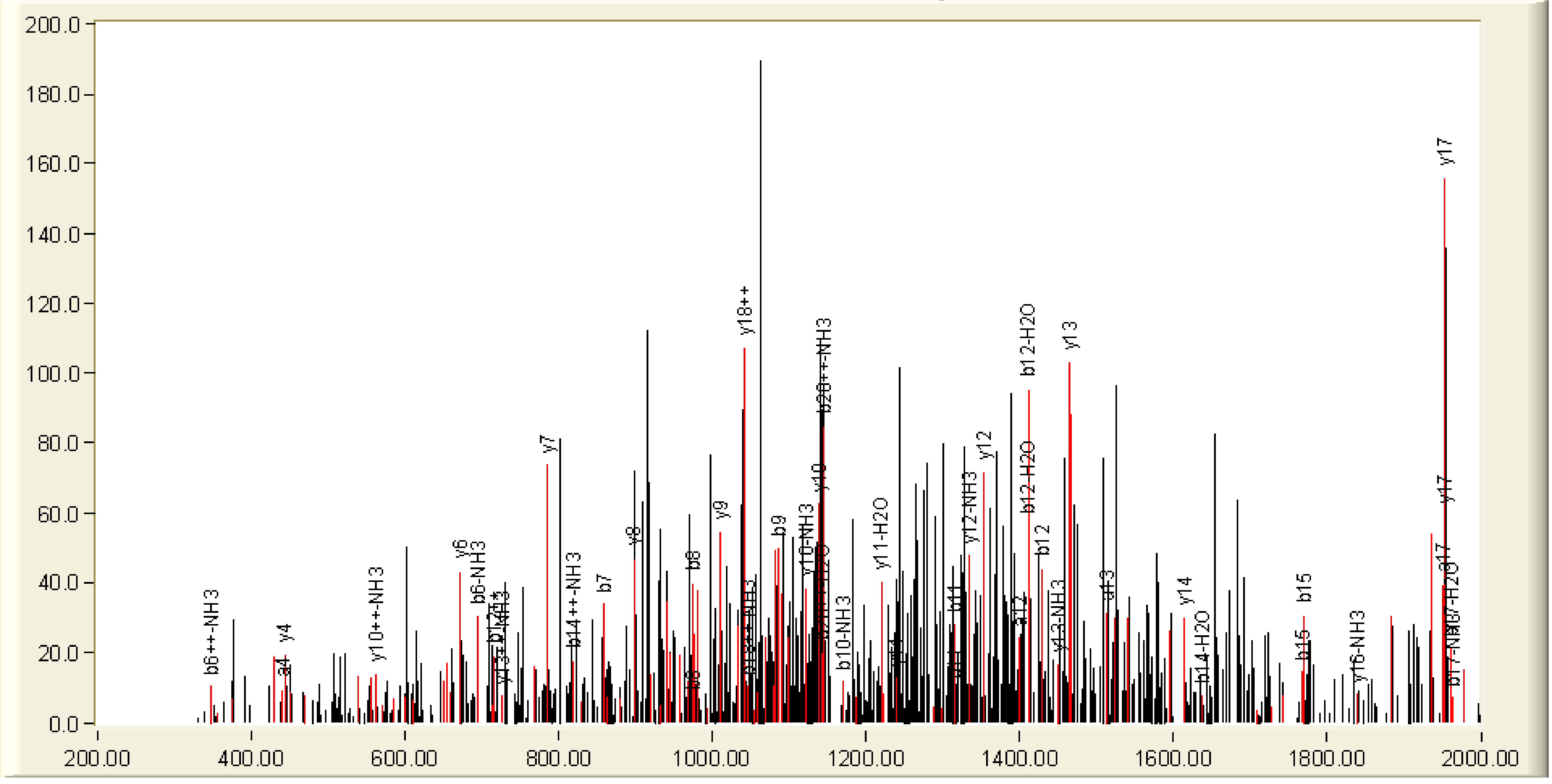

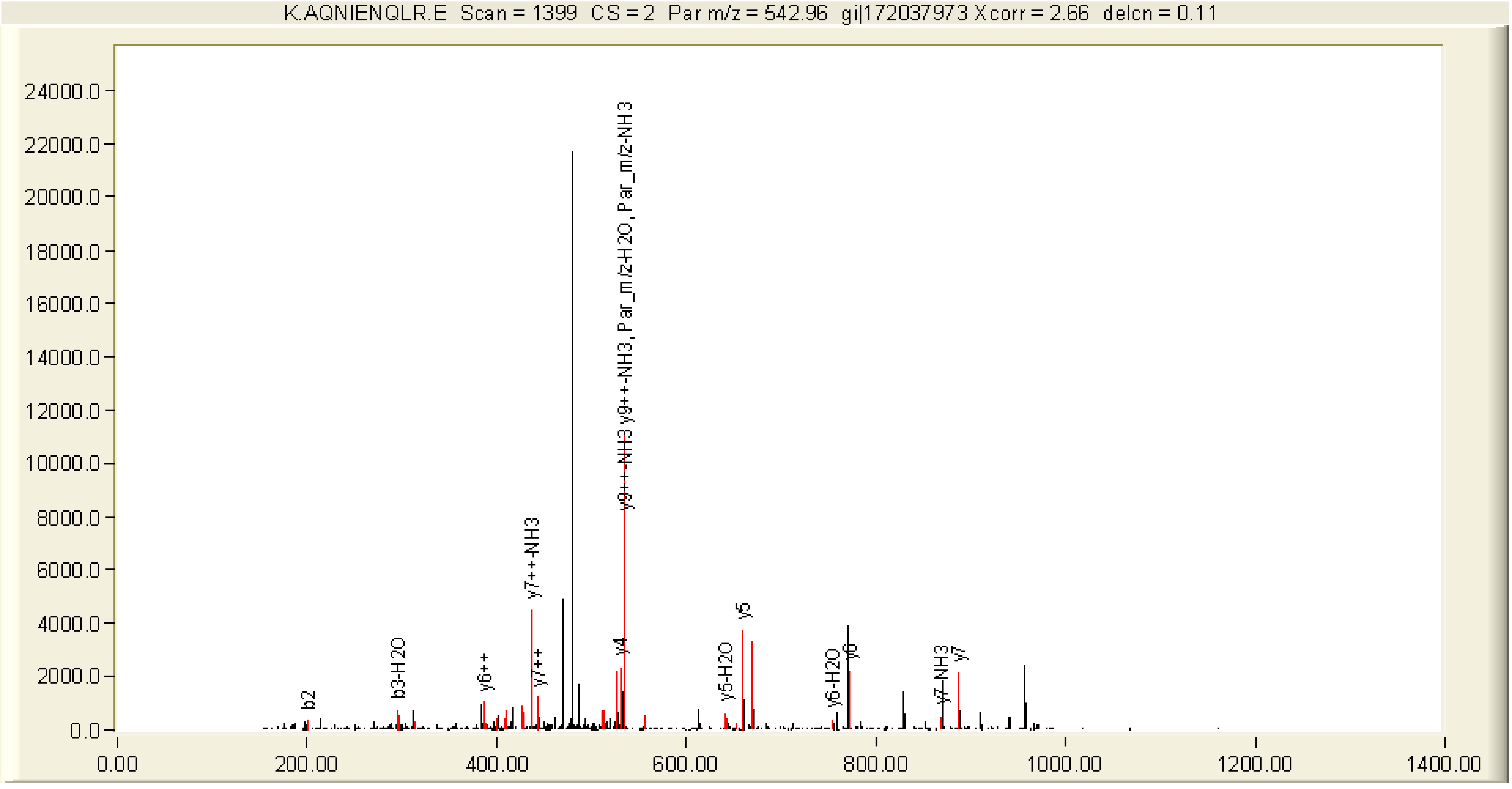

K.AQNIENQLR.E Scan = 1399 CS = 2 Par m/z = 542.96 gi|172037973 Xcorr = 2.66 delcn = 0.11
24000.0
22000.0
20000.0
18000.0
16000.0
14000.0
12000.0
10000.0
8000.0
6000.0
4000.0
2000.0
0.0
0.00
200.00
400.00
600.00
800.00
1000.00
1200.00
1400.00
b2
b3-H2O
y6++
y7++
y7++-NH3
y4
y9++-NH3 y9++-NH3, Par_m/z-H2O, Par_m/z-NH3
y5-H2O
y5
y6-H2O
y6
y7-NH3
y7

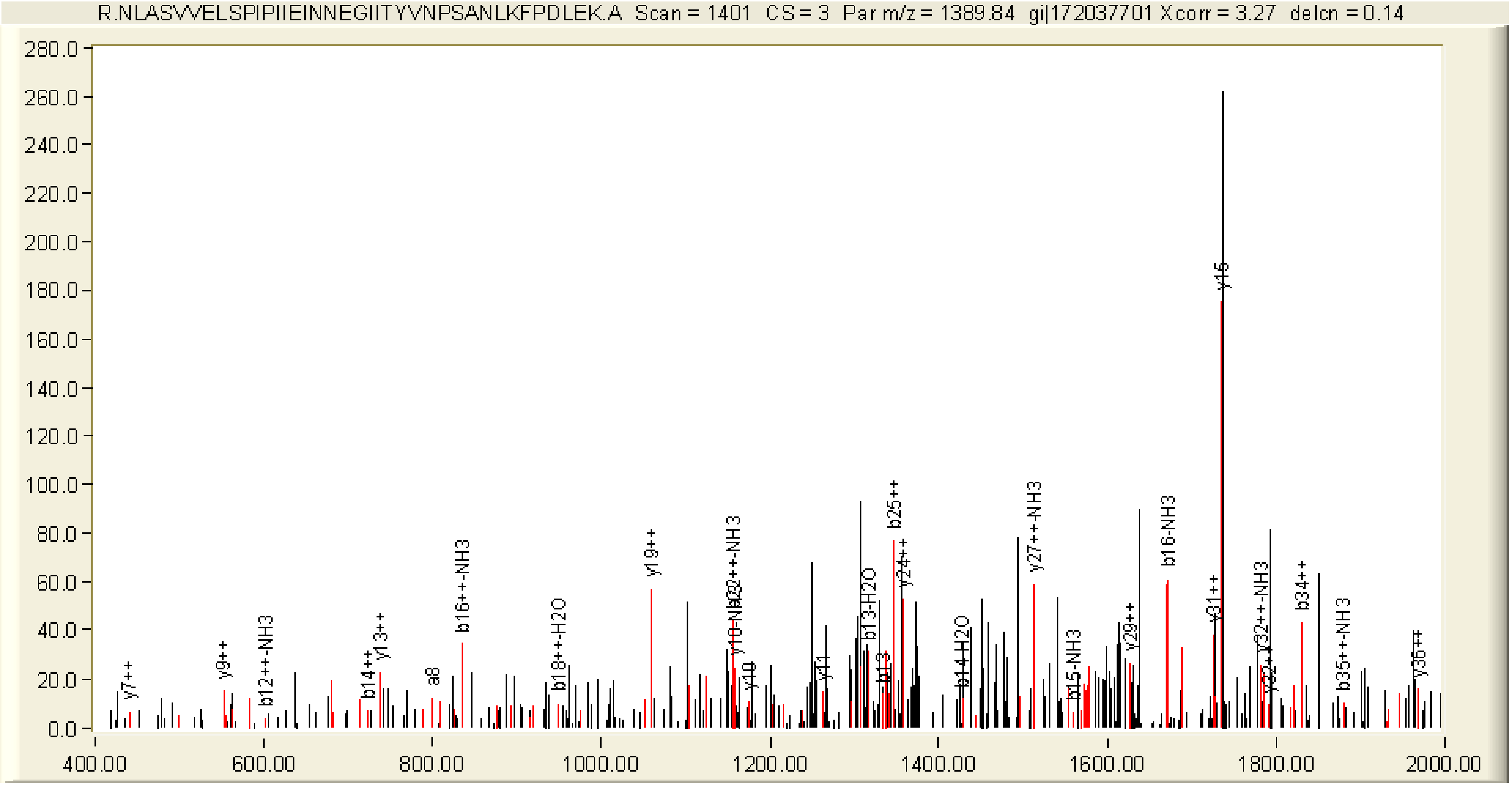

R.NLASVVELSPIPIIEINNEGIITYVNPSANLKFPDLEK.A Scan = 1401 CS = 3 Par m/z = 1389.84 gi|172037701 Xcorr = 3.27 delcn = 0.14
280.0
260.0
240.0
220.0
200.0
180.0
160.0
140.0
120.0
100.0
80.0
60.0
40.0
20.0
0.0
400.00
600.00
800.00
1000.00
1200.00
1400.00
1600.00
1800.00
2000.00
y7++
y9++
b12++-NH3
b14++
y13++
a8
b16++-NH3
b18++-H2O
y19++
b22++-NH3
y10
y11
b13-H2O
b13
b25++
y24++
b14-H2O
y27++-NH3
b15-NH3
y29++
b16-NH3
y31++
y15
b32++-NH3
b34++
b35++-NH3
y36++

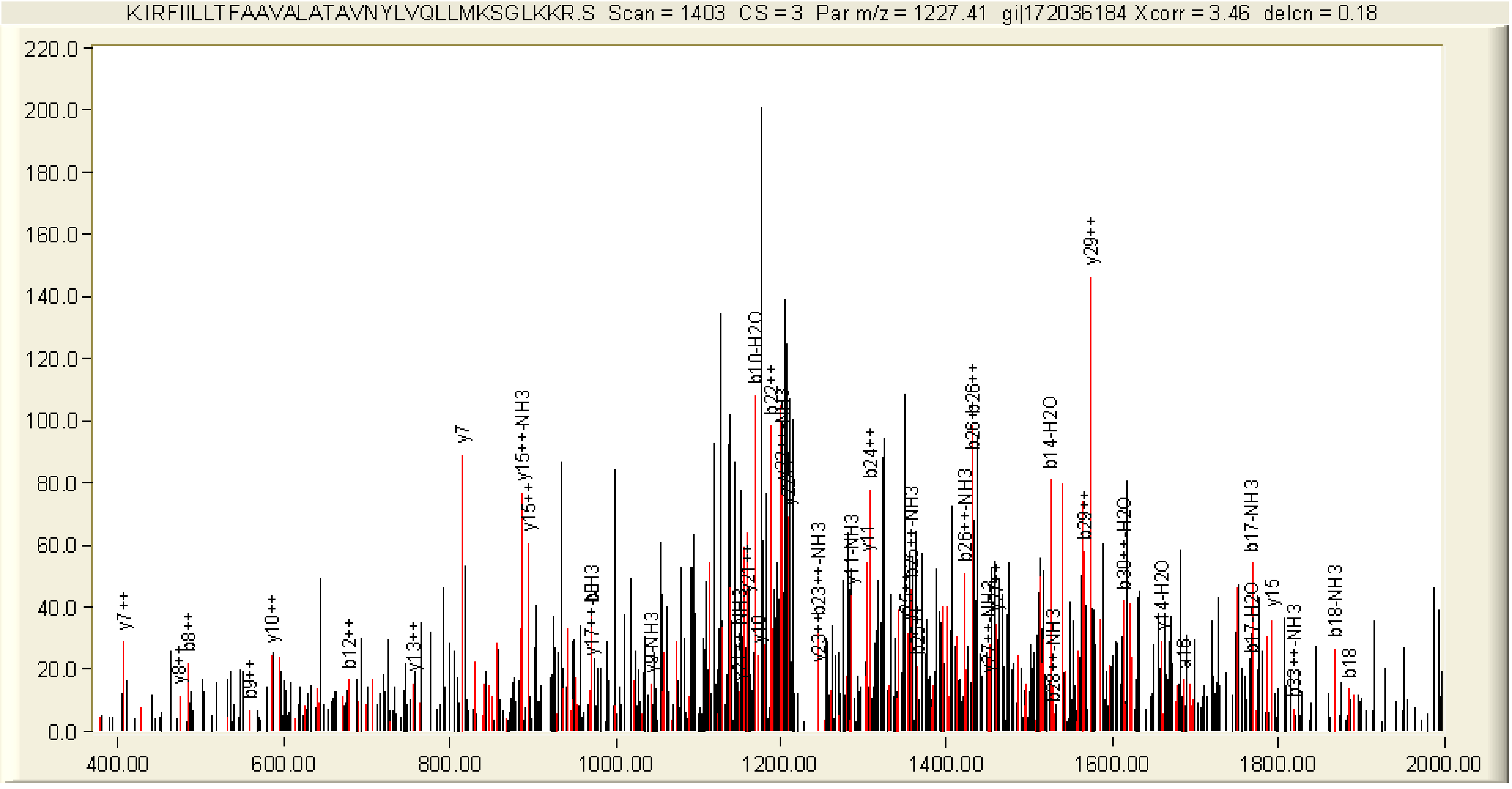
KIRFIILLTFAAVALATAVNYLVQLLMKSGLKKR.S Scan = 1403 CS = 3 Par m/z = 1227.41 gi|172036184 Xcorr = 3.46 delcn = 0.18
220.0
200.0
180.0
160.0
140.0
120.0
100.0
80.0
60.0
40.0
20.0
0.0
400.00
600.00
800.00
1000.00
1200.00
1400.00
1600.00
1800.00
2000.00
y7++
b8++
y10++
b12++
y13++
y7
y15++-NH3
b10-H2O
b22++
b24++
b26++
b14-H2O
y29++
b29++
b30++-H2O
b17-NH3
y15
b18-NH3
b18

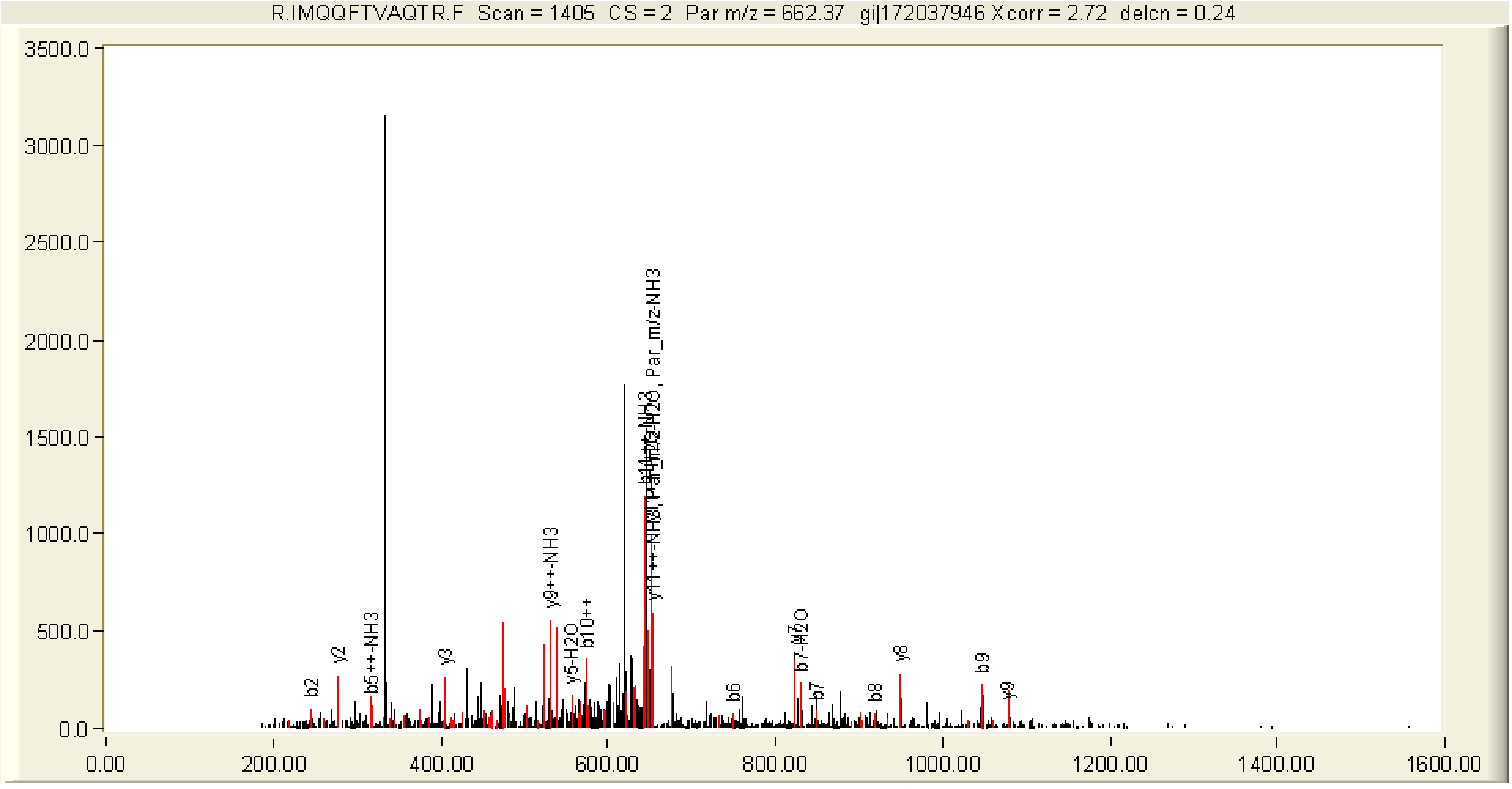
R.IMQQFTVAQTR.F Scan = 1405 CS = 2 Par m/z = 662.37 gi|172037946 Xcorr = 2.72 delcn = 0.24
3500.0
3000.0
2500.0
2000.0
1500.0
1000.0
500.0
0.0
0.00
200.00
400.00
600.00
800.00
1000.00
1200.00
1400.00
1600.00
b2
y2
b5++-NH3
y3
y9++-NH3
y5-H2O
b10++
b6
b7-H2O
b7
b8
y8
b9
y9

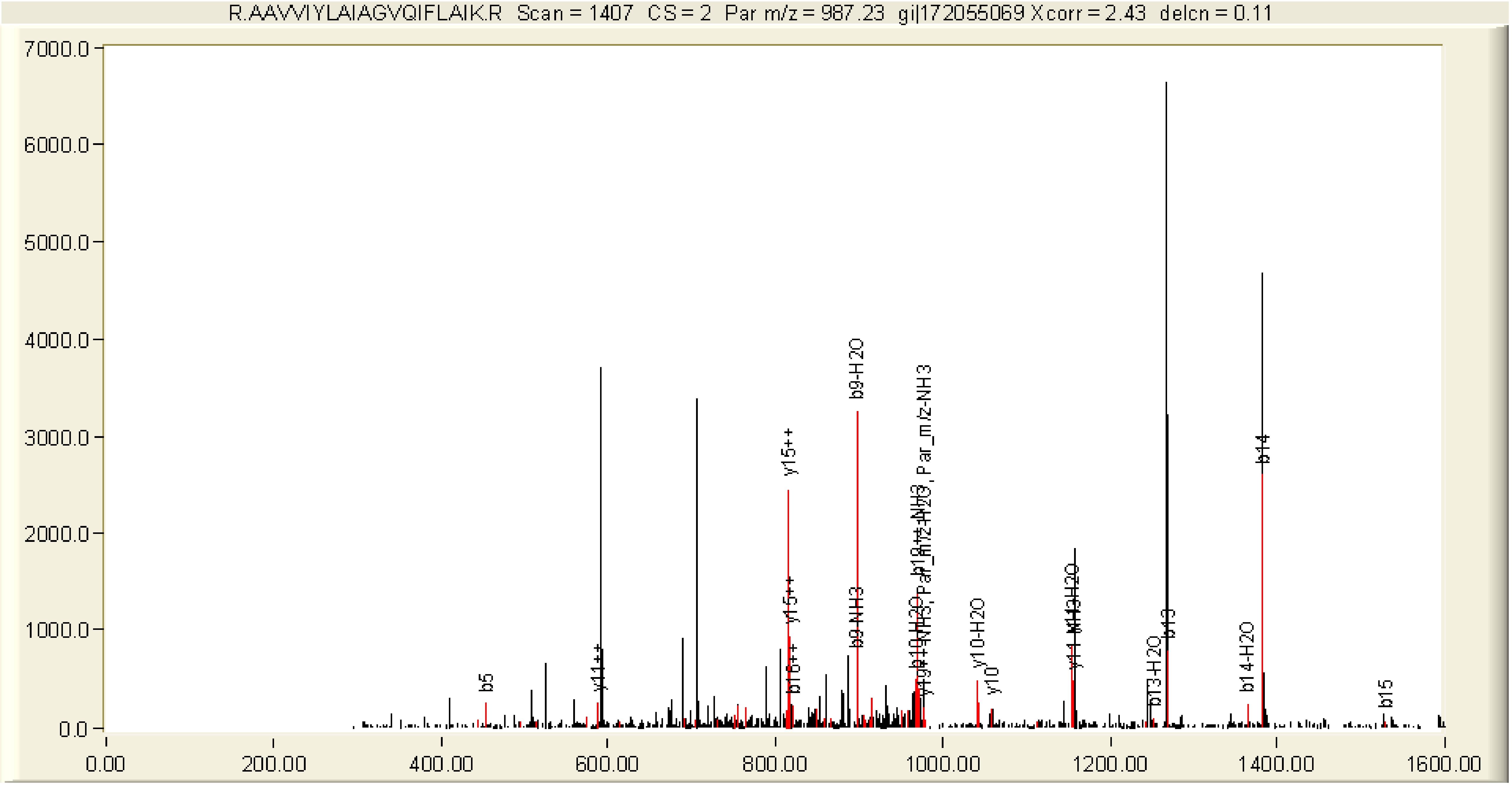
R.AAVVIYLAIAGVQIFLAIK.R Scan = 1407 CS = 2 Par m/z = 987.23 gi|172055069 Xcorr = 2.43 delcn = 0.11
7000.0
6000.0
5000.0
4000.0
3000.0
2000.0
1000.0
0.0
0.00
200.00
400.00
600.00
800.00
1000.00
1200.00
1400.00
1600.00
b5
y11++
b16++
y15++
y15++
b9-NH3
b9-H2O
y10-H2O
y10
b13-H2O
b13
b14-H2O
b14
b15

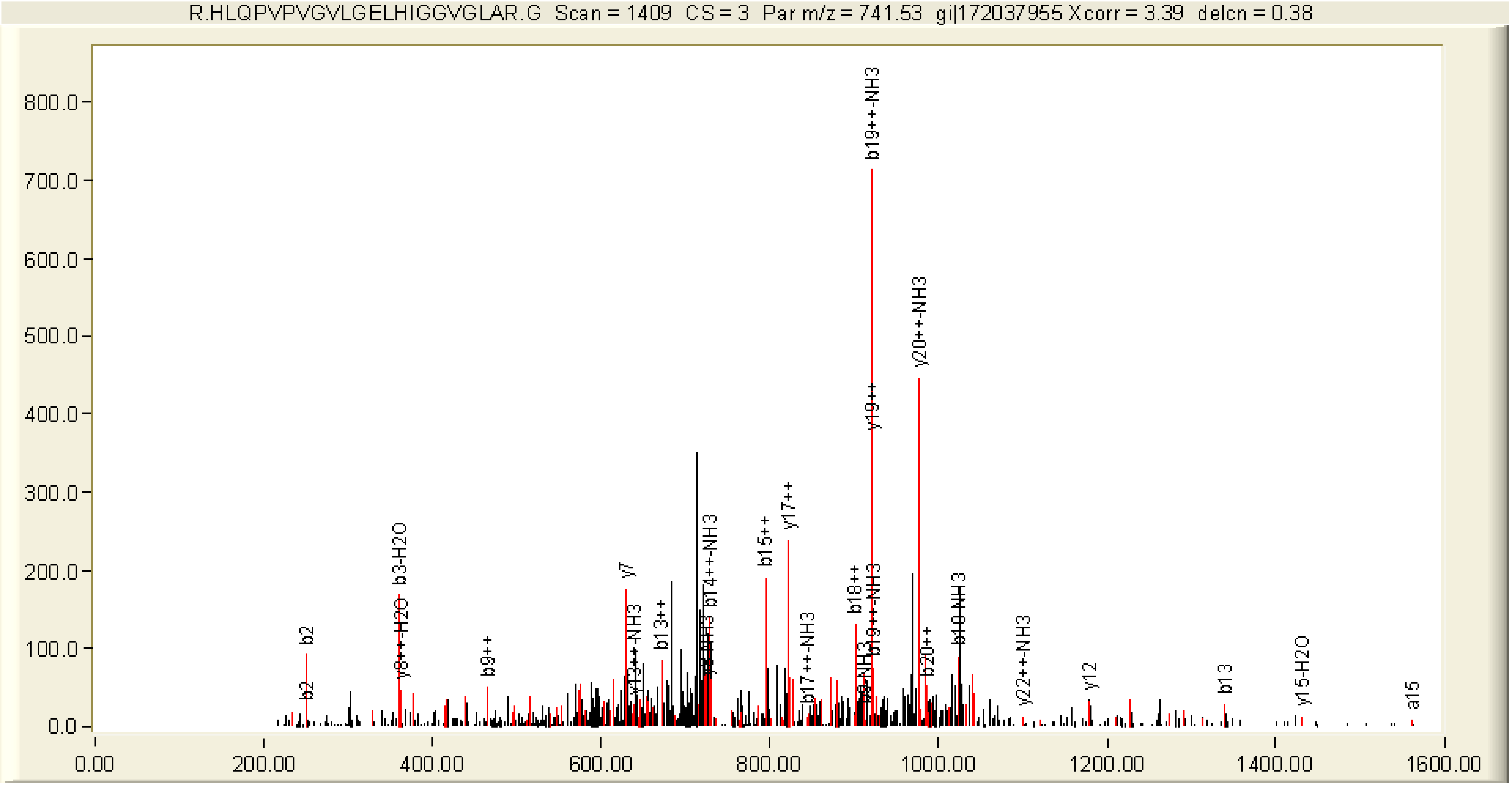
R.HLQPVPVGVLGELHIGGVGLAR.G Scan = 1409 CS = 3 Par m/z = 741.53 gi|172037955 Xcorr = 3.39 delcn = 0.38
800.0
700.0
600.0
500.0
400.0
300.0
200.0
100.0
0.0
0.00
200.00
400.00
600.00
800.00
1000.00
1200.00
1400.00
1600.00
b2
b2
y8++-H2O
b3-H2O
b9++
y7
b13++
b14++-NH3
b15++
y17++
b17++-NH3
b18++
b19++-NH3
y19++
y20++-NH3
b20++
b10-NH3
y22++-NH3
y12
b13
y15-H2O
a15

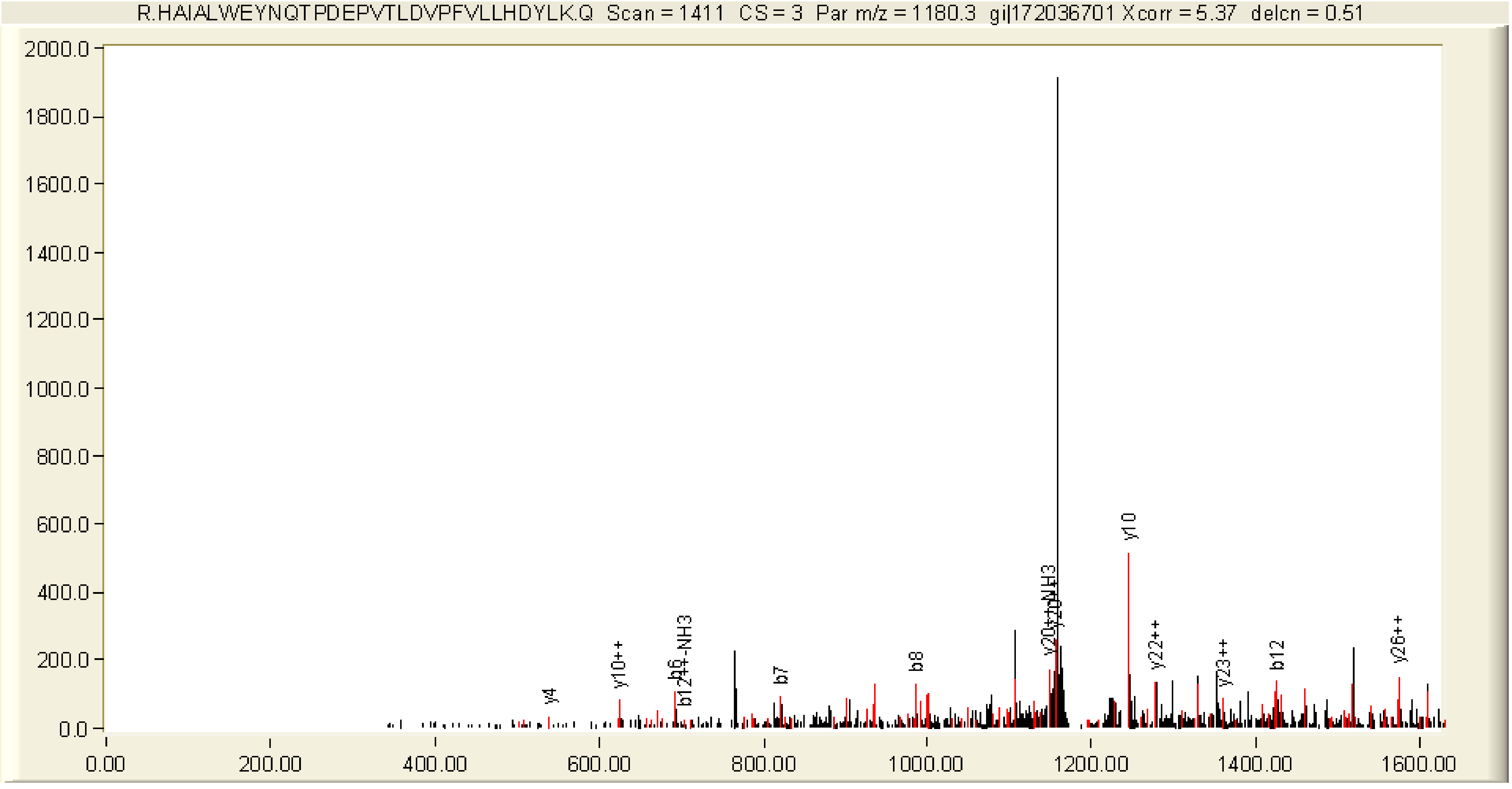

R.HAIALWEYNQTPDEPVTLDVPFVLLHDYLK.Q Scan = 1411 CS = 3 Par m/z = 1180.3 gi|172036701 Xcorr = 5.37 delcn = 0.51
2000.0
1800.0
1600.0
1400.0
1200.0
1000.0
800.0
600.0
400.0
200.0
0.0
0.00
200.00
400.00
600.00
800.00
1000.00
1200.00
1400.00
1600.00
y4
y10++
b6
b12++-NH3
b7
b8
y20++-NH3
y10
y22++
y23++
b12
y26++

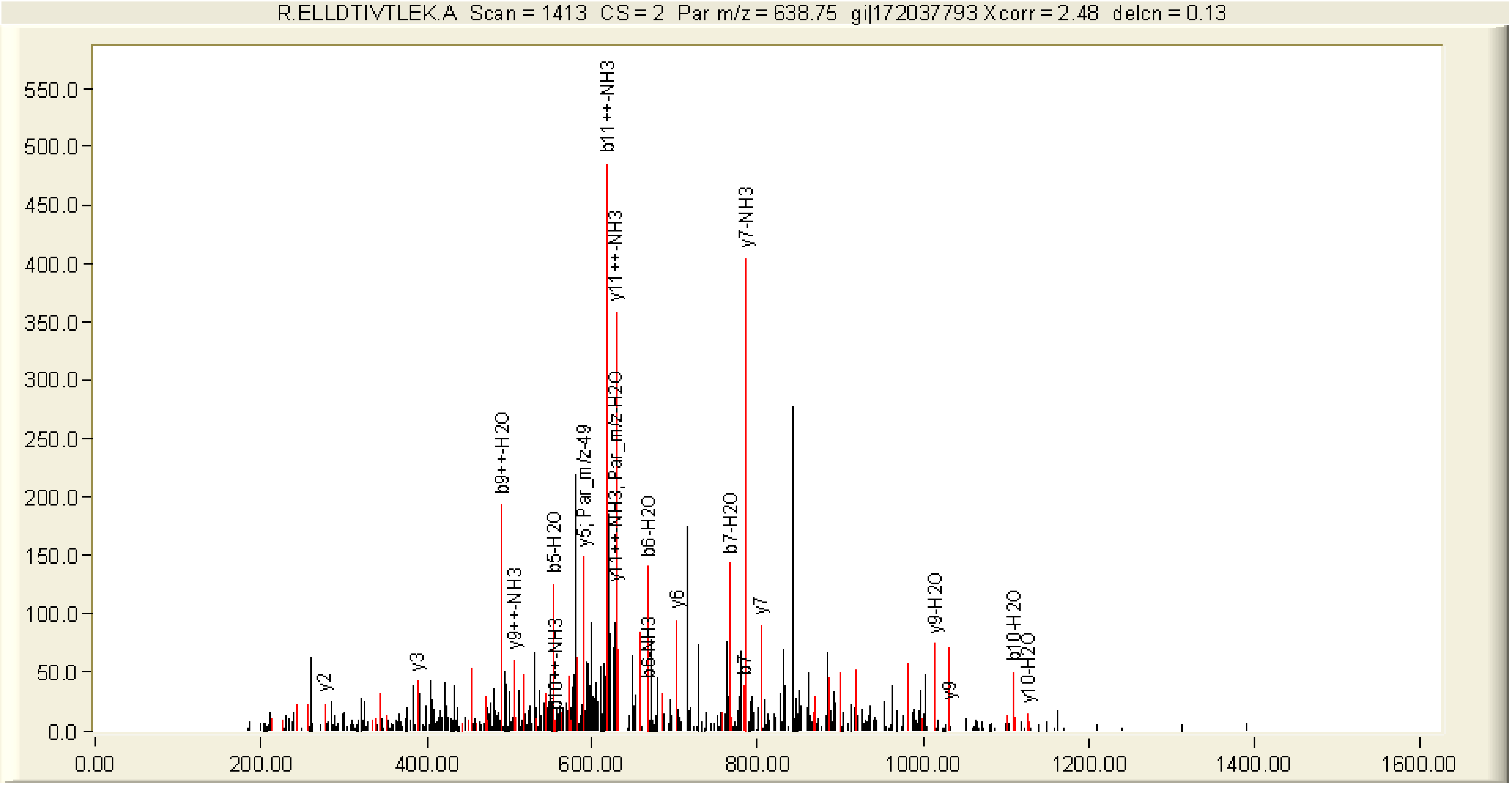
R.ELLDTIVTLEK.A Scan = 1413 CS = 2 Par m/z = 638.75 gi|172037793 Xcorr = 2.48 delcn = 0.13
550.0
500.0
450.0
400.0
350.0
300.0
250.0
200.0
150.0
100.0
50.0
0.0
0.00
200.00
400.00
600.00
800.00
1000.00
1200.00
1400.00
1600.00
b11++-NH3
y7-NH3
y11++-NH3
y11++-NH3, Par_m/z-H2O
y5; Par_m/z-49
b9++-H2O
b5-H2O
b6-H2O
b7-H2O
y9++-NH3
b10++-NH3
b6-NH3
y6
y7
b7
y2
y3
y9-H2O
y9
b10-H2O
y10-H2O

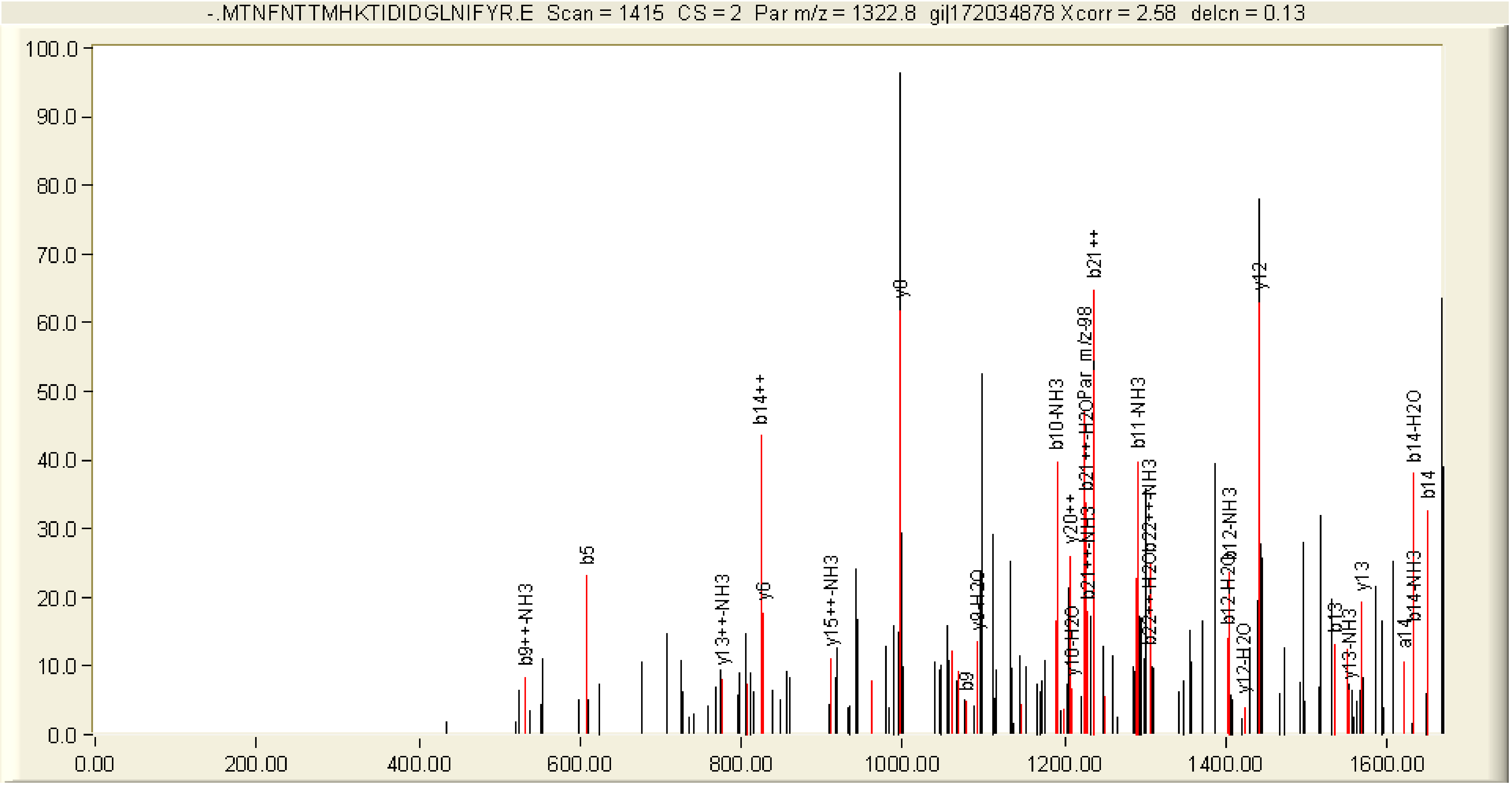

-.MTNFNTTMHKTIDIDGLNIFYR.E Scan = 1415 CS = 2 Par m/z = 1322.8 gi|172034878 Xcorr = 2.58 delcn = 0.13
b9++-NH3
b5
y13++-NH3
b14++
y6
y15++-NH3
y8
b9
y9-H2O
b10-NH3
y10-H2O
y20++
b21++-NH3
b21++-H2OPar m/z-98
b21++
b11-NH3
b22++-H2Ob22++-NH3
b12-H2O
b12-NH3
y12-H2O
y12
b13
y13-NH3
y13
a14
b14-NH3
b14-H2O
b14

R.VSWLADAFEAVLGALYLSTK.N Scan = 1417 CS = 2 Par m/z = 1077.78 gi|172038048 Xcorr = 2.57 delcn = 0.25

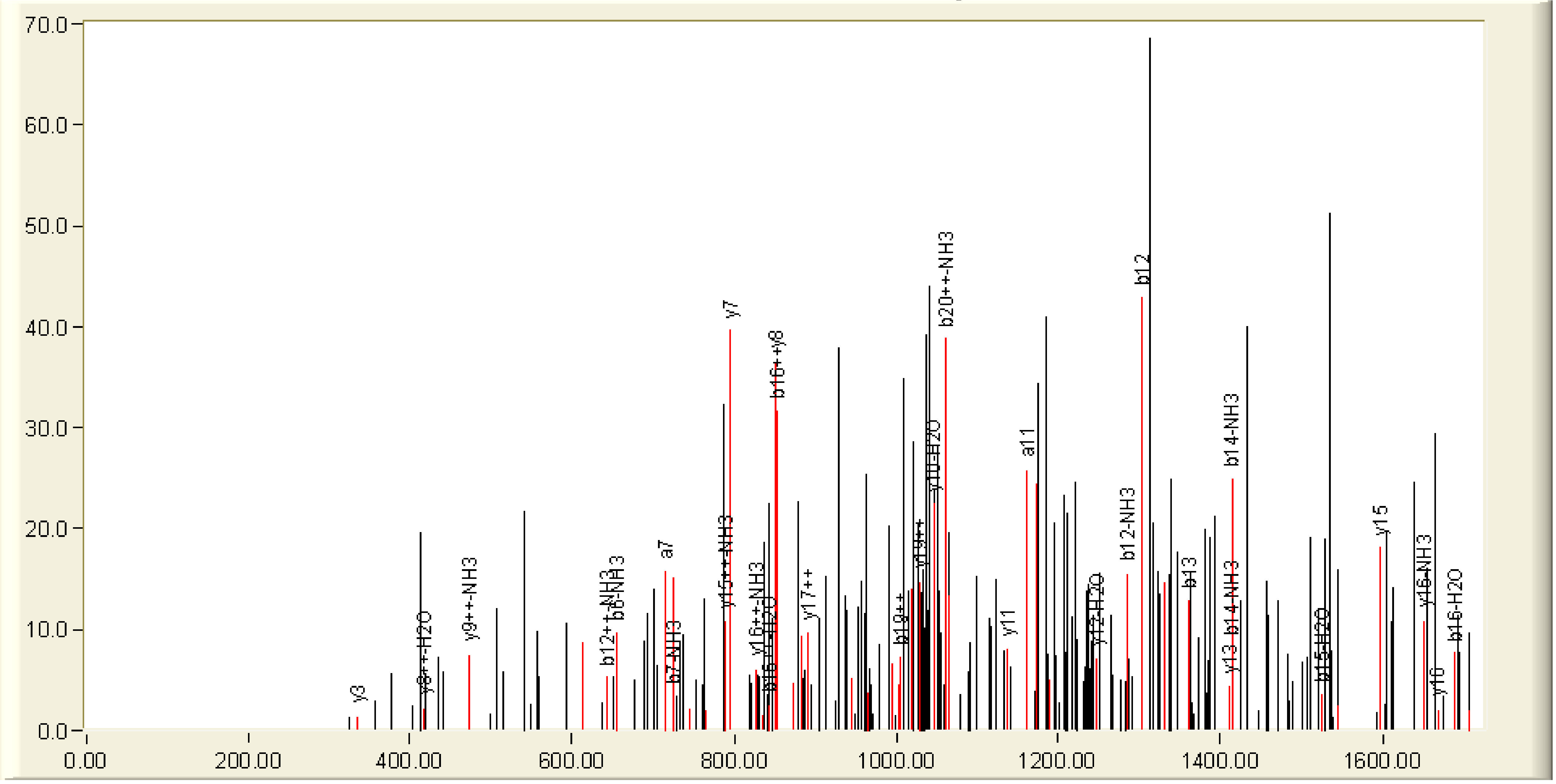

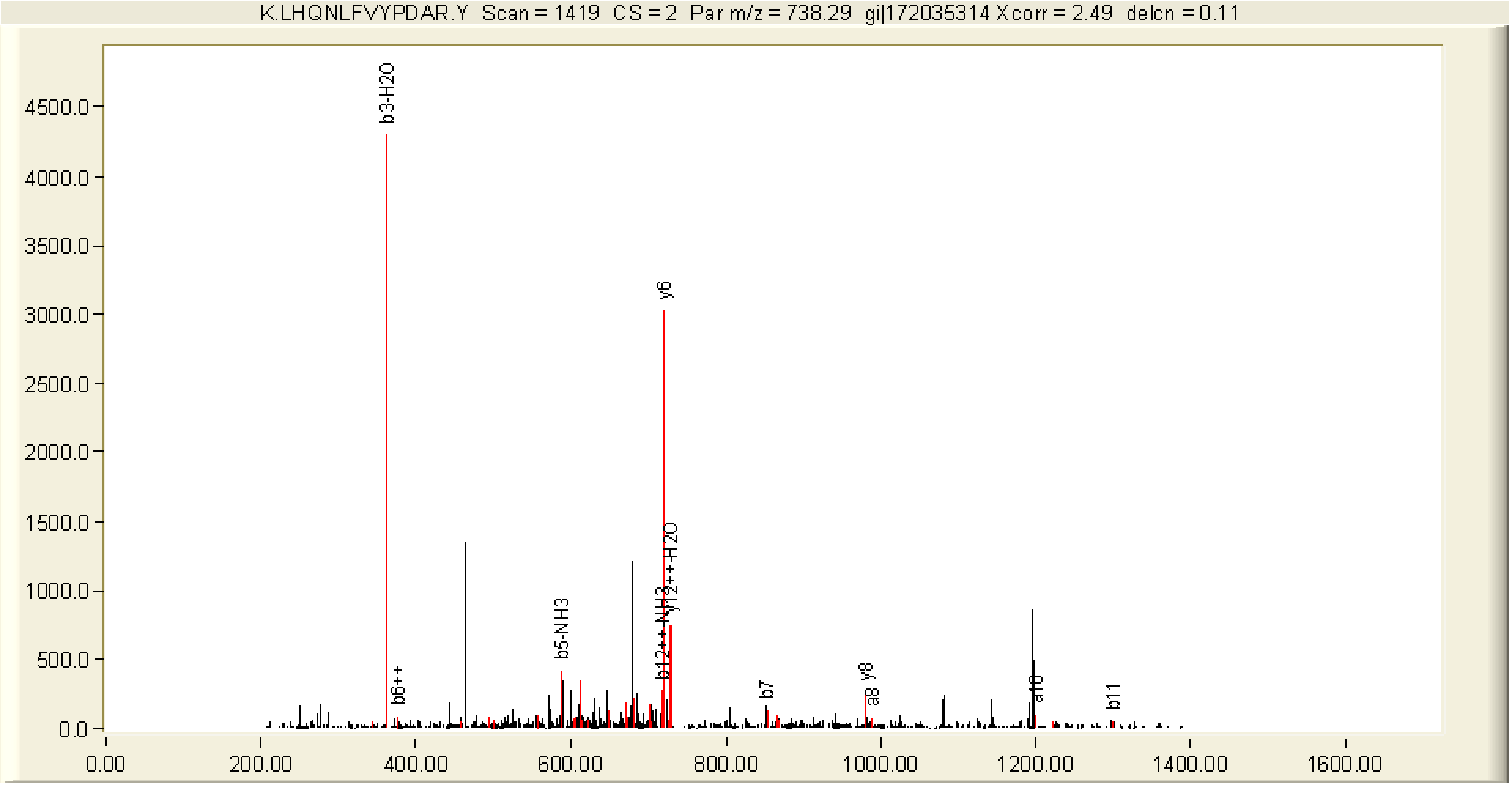

K.LHQNLFVYPDAR.Y Scan = 1419 CS = 2 Par m/z = 738.29 gi|172035314 Xcorr = 2.49 delcn = 0.11
b3-H2O
y6
y12++-H2O
b12++-NH3
b5-NH3
b6++
b7
a8 y8
a10
b11
4500.0
4000.0
3500.0
3000.0
2500.0
2000.0
1500.0
1000.0
500.0
0.0
0.00
200.00
400.00
600.00
800.00
1000.00
1200.00
1400.00
1600.00

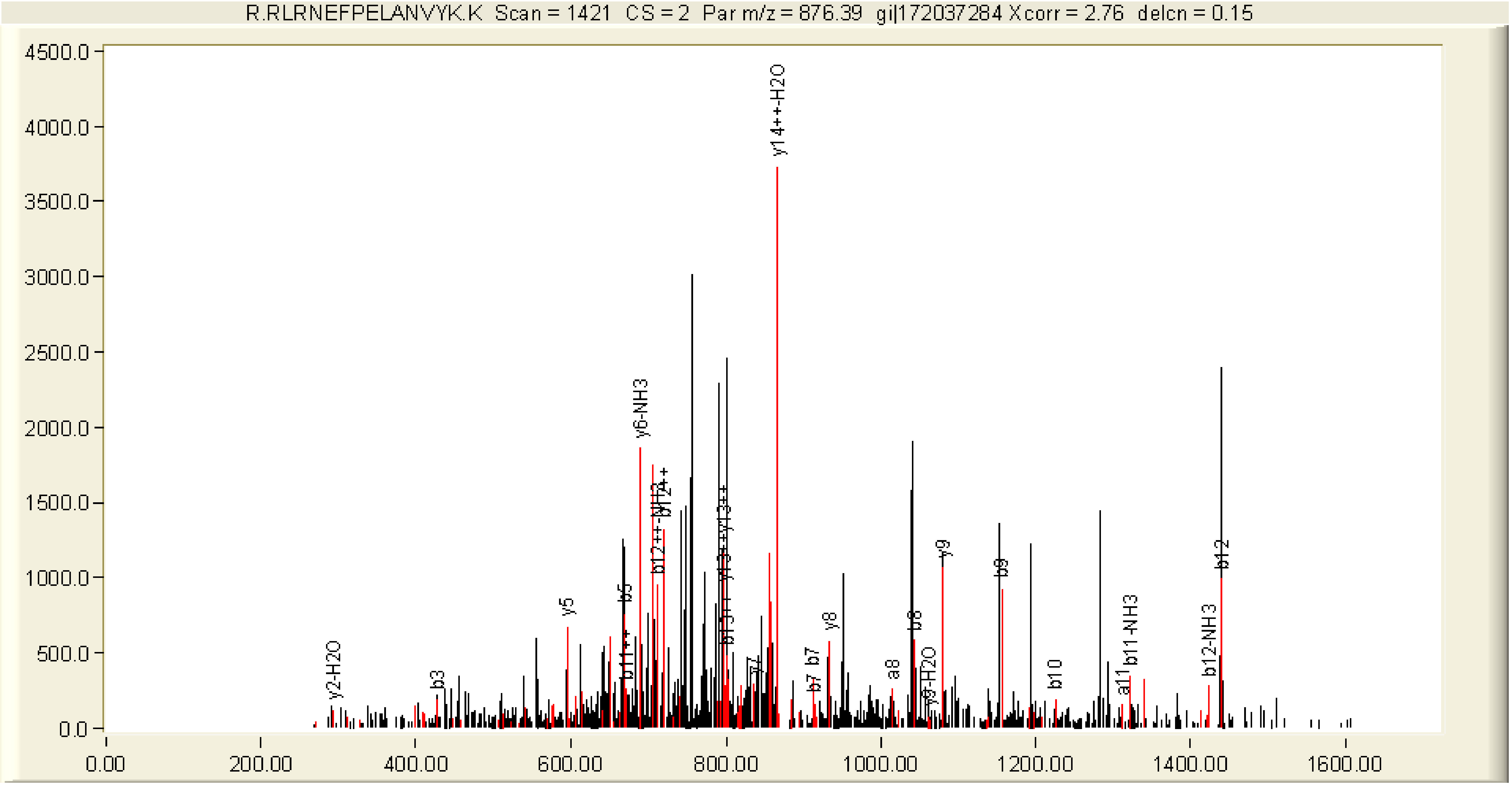
R.RLRNEFPELANVYK.K Scan = 1421 CS = 2 Par m/z = 876.39 gi|172037284 Xcorr = 2.76 delcn = 0.15
y14++-H2O
y6-NH3
y5
b5
y8
y9
b9
b12
b12-NH3
b11-NH3
a11
b10
a8
b8
b7
y2-H2O
b3
4500.0
4000.0
3500.0
3000.0
2500.0
2000.0
1500.0
1000.0
500.0
0.0
0.00
200.00
400.00
600.00
800.00
1000.00
1200.00
1400.00
1600.00

K.VSAEDQGEIIQGSIDVLVIQEQLWVTVIEAK.N Scan = 1423 CS = 3 Par m/z = 1137.43 gi|172055010 Xcorr = 3.24 delcn = 0.17

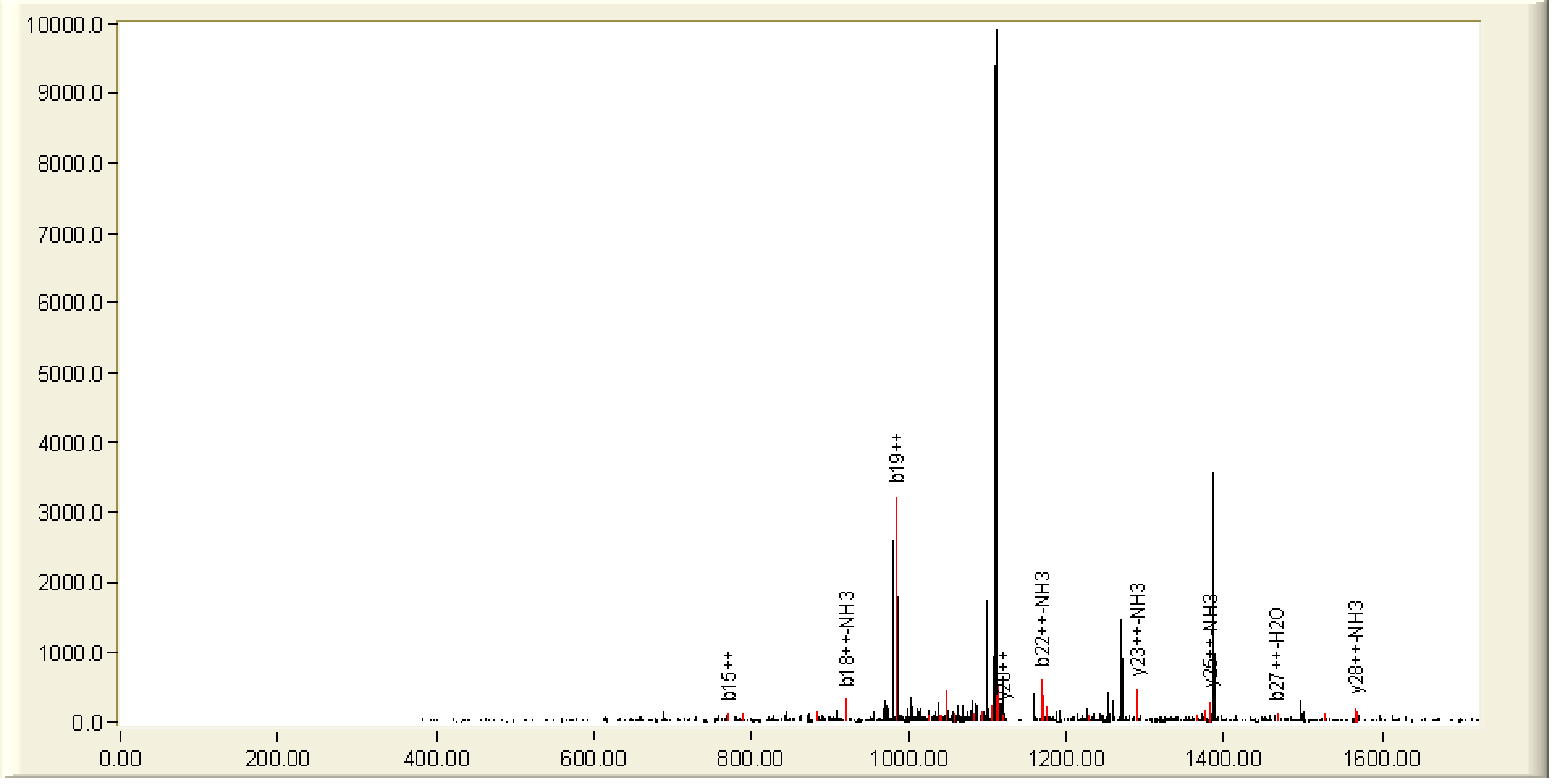

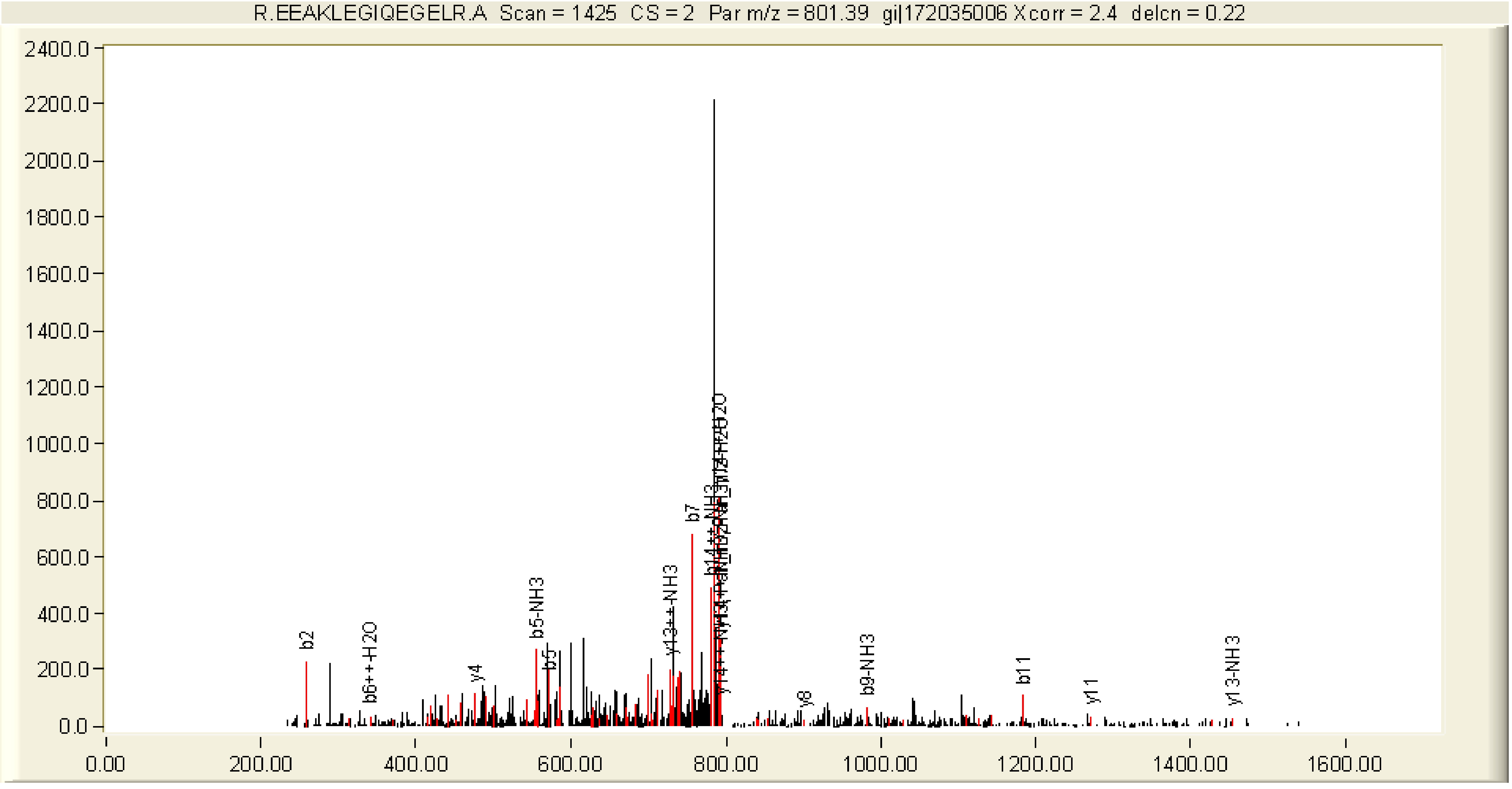
R.EEAKLEGIQEGELR.A Scan = 1425 CS = 2 Par m/z = 801.39 gi|172035006 Xcorr = 2.4 delcn = 0.22
2400.0
2200.0
2000.0
1800.0
1600.0
1400.0
1200.0
1000.0
800.0
600.0
400.0
200.0
0.0
0.00
200.00
400.00
600.00
800.00
1000.00
1200.00
1400.00
1600.00
b2
b6++-H2O
y4
b5-NH3
b5
y13++-NH3
b7
b14++-NH3
y8
b9-NH3
b11
y11
y13-NH3

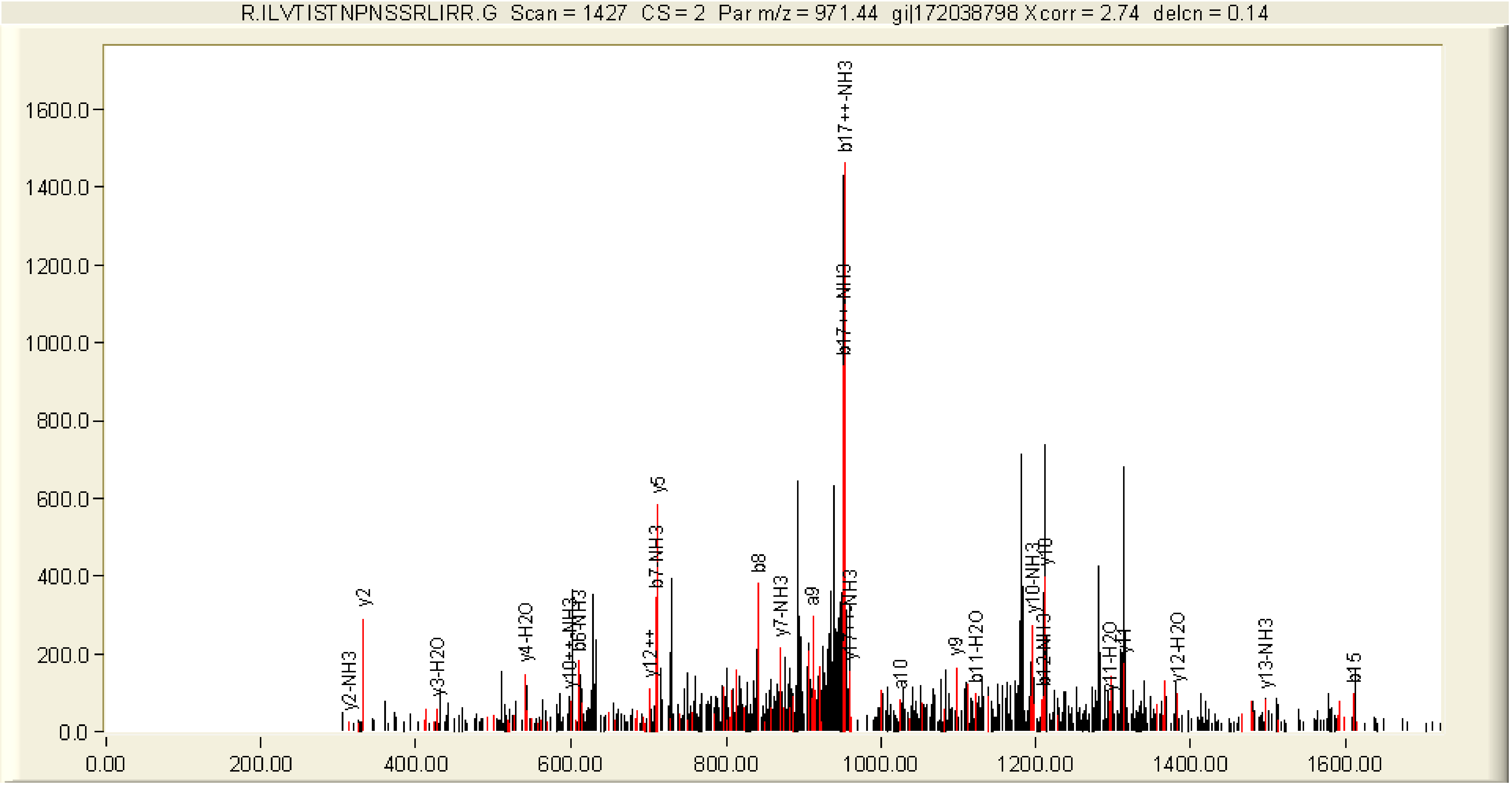
R.ILVTISTNPNSSRLIRR.G Scan = 1427 CS = 2 Par m/z = 971.44 gi|172038798 Xcorr = 2.74 delcn = 0.14
1600.0
1400.0
1200.0
1000.0
800.0
600.0
400.0
200.0
0.0
0.00
200.00
400.00
600.00
800.00
1000.00
1200.00
1400.00
1600.00
b17++-NH3
y5
b7-NH3
b8
y7-NH3
a9
y2
y2-NH3
y3-H2O
y4-H2O
y12++
a10
y9
b11-H2O
y10-NH3
y10
y12-H2O
y13-NH3
b15

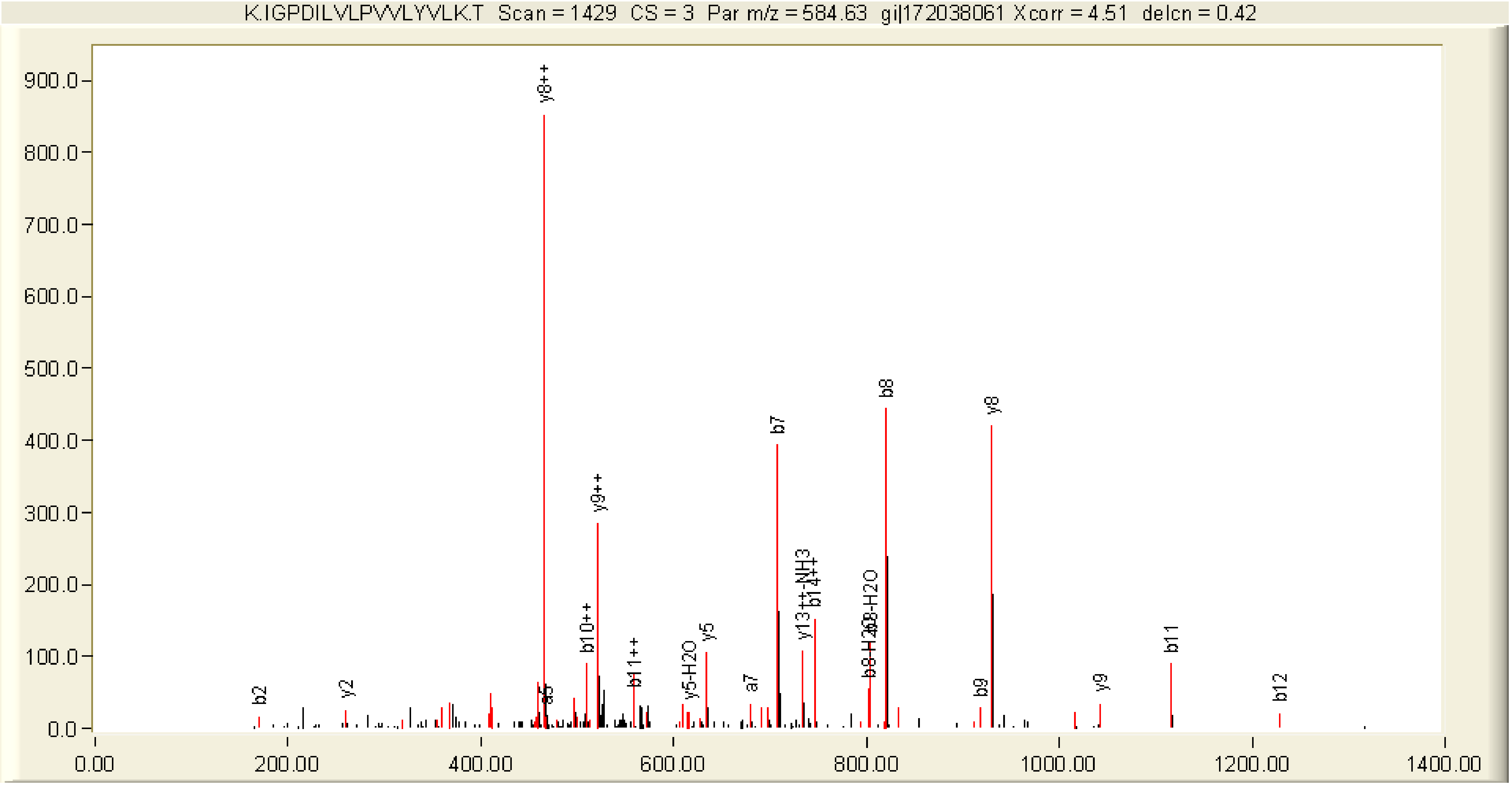

K.IGPDILVLPVVLYVLK.T Scan = 1429 CS = 3 Par m/z = 584.63 gi|172038061 Xcorr = 4.51 delcn = 0.42
y8++
y9++
b10++
b11++
y5-H2O
y5
a7
b7
b8
y8
b9
y9
b11
b12
b2
y2
a5
b8-H2O
0.0
100.0
200.0
300.0
400.0
500.0
600.0
700.0
800.0
900.0
0.00
200.00
400.00
600.00
800.00
1000.00
1200.00
1400.00

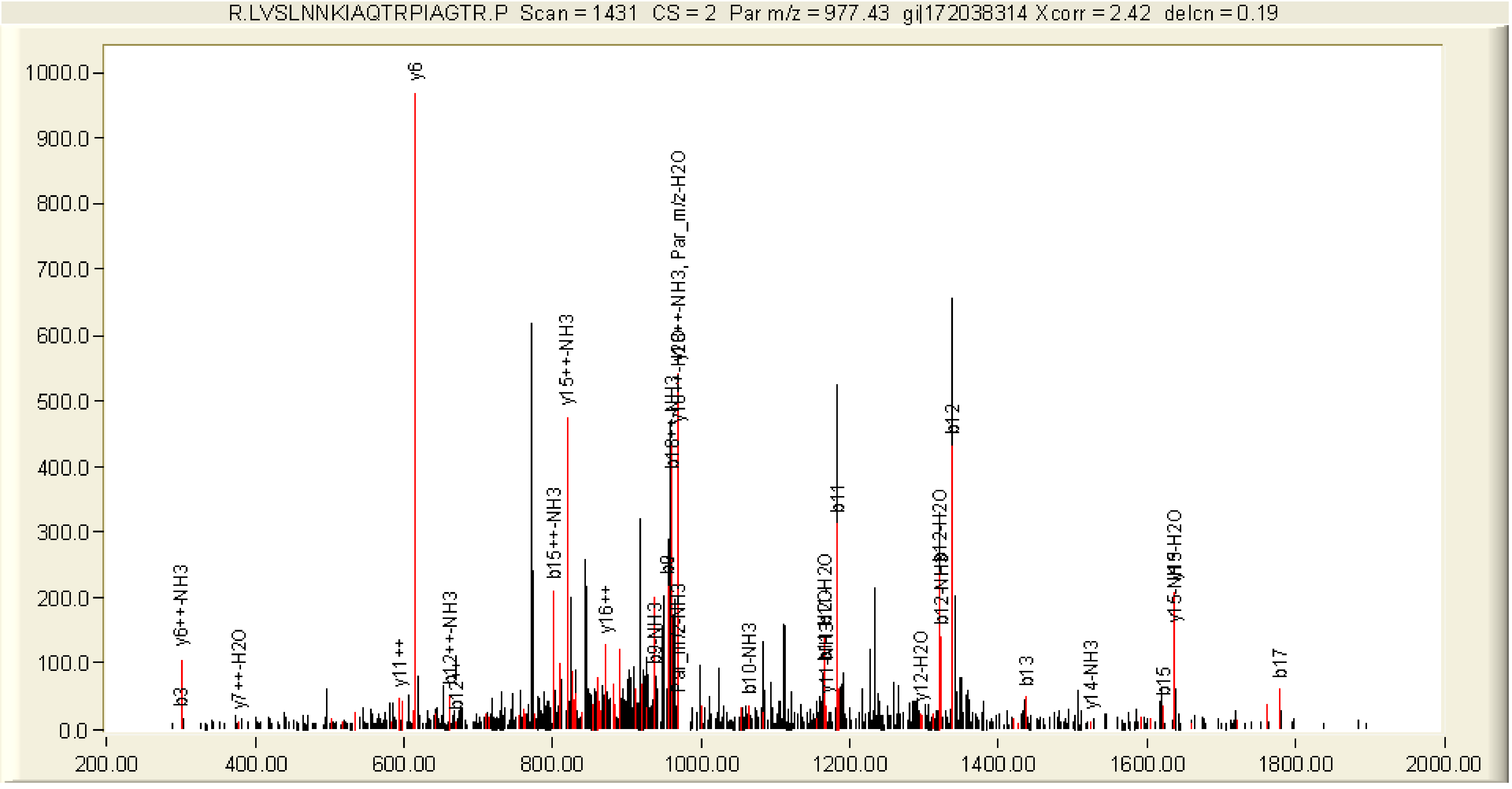

R.LVSLNNKIAQTRPIAGTR.P Scan = 1431 CS = 2 Par m/z = 977.43 gi|172038314 Xcorr = 2.42 delcn = 0.19
1000.0
900.0
800.0
700.0
600.0
500.0
400.0
300.0
200.0
100.0
0.0
200.00
400.00
600.00
800.00
1000.00
1200.00
1400.00
1600.00
1800.00
2000.00
y6
y15++-NH3
b15++-NH3
y16++
y6++-NH3
b3
y7++-H2O
y11++
b12++-NH3
b9-NH3
b10-NH3
b11
b12
b12-H2O
b12-NH3
y12-H2O
b13
y14-NH3
b15
b17

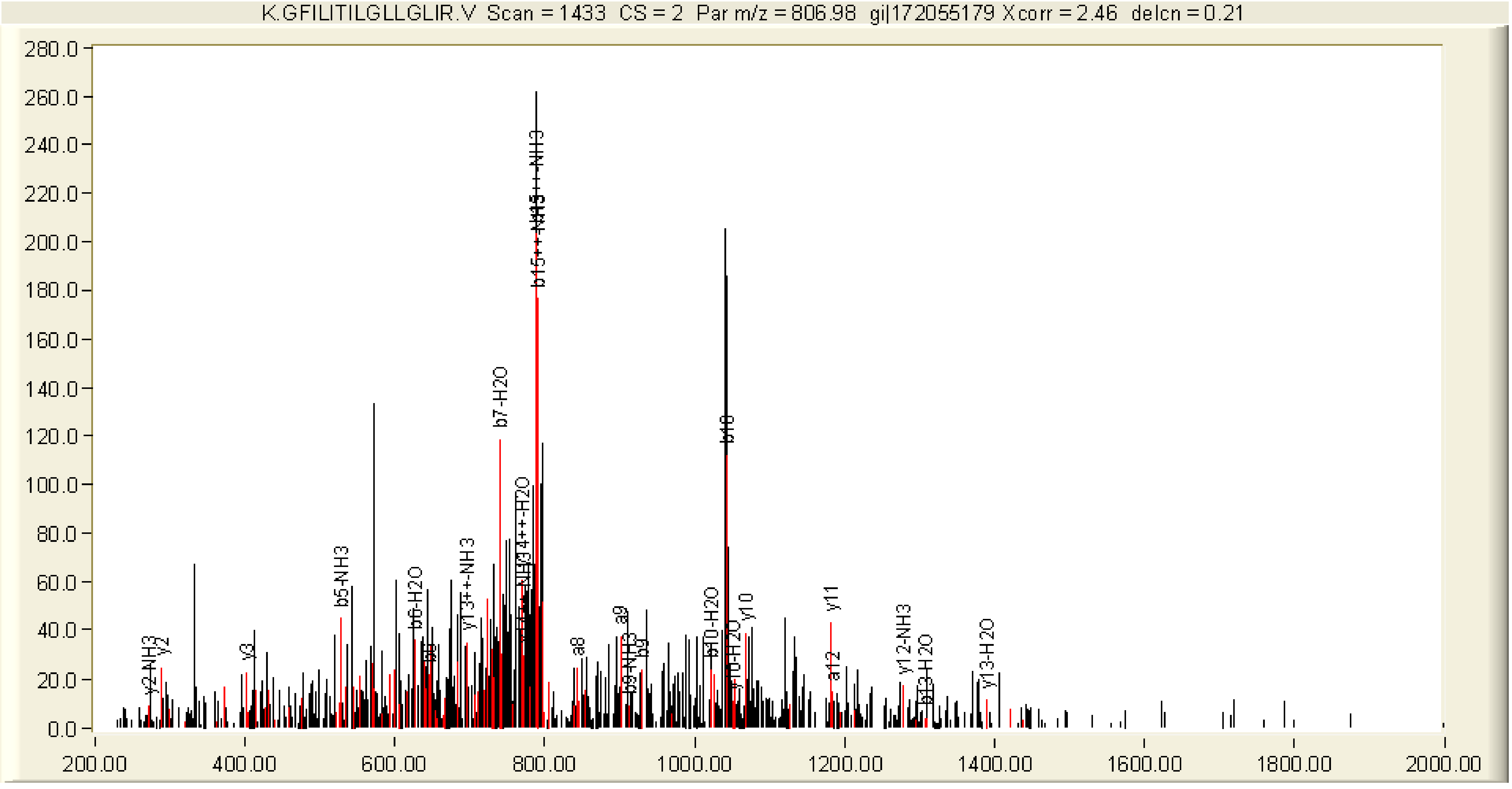

K.GFILITILGLLGLIR.V Scan = 1433 CS = 2 Par m/z = 806.98 gi|172055179 Xcorr = 2.46 delcn = 0.21
280.0
260.0
240.0
220.0
200.0
180.0
160.0
140.0
120.0
100.0
80.0
60.0
40.0
20.0
0.0
200.00
400.00
600.00
800.00
1000.00
1200.00
1400.00
1600.00
1800.00
2000.00
y2-NH3
y2
y3
b5-NH3
b6-H2O
y13++-NH3
b7-H2O
a8
a9
b9-NH3
b10-H2O
y10
y11
a12
y12-NH3
y13-H2O

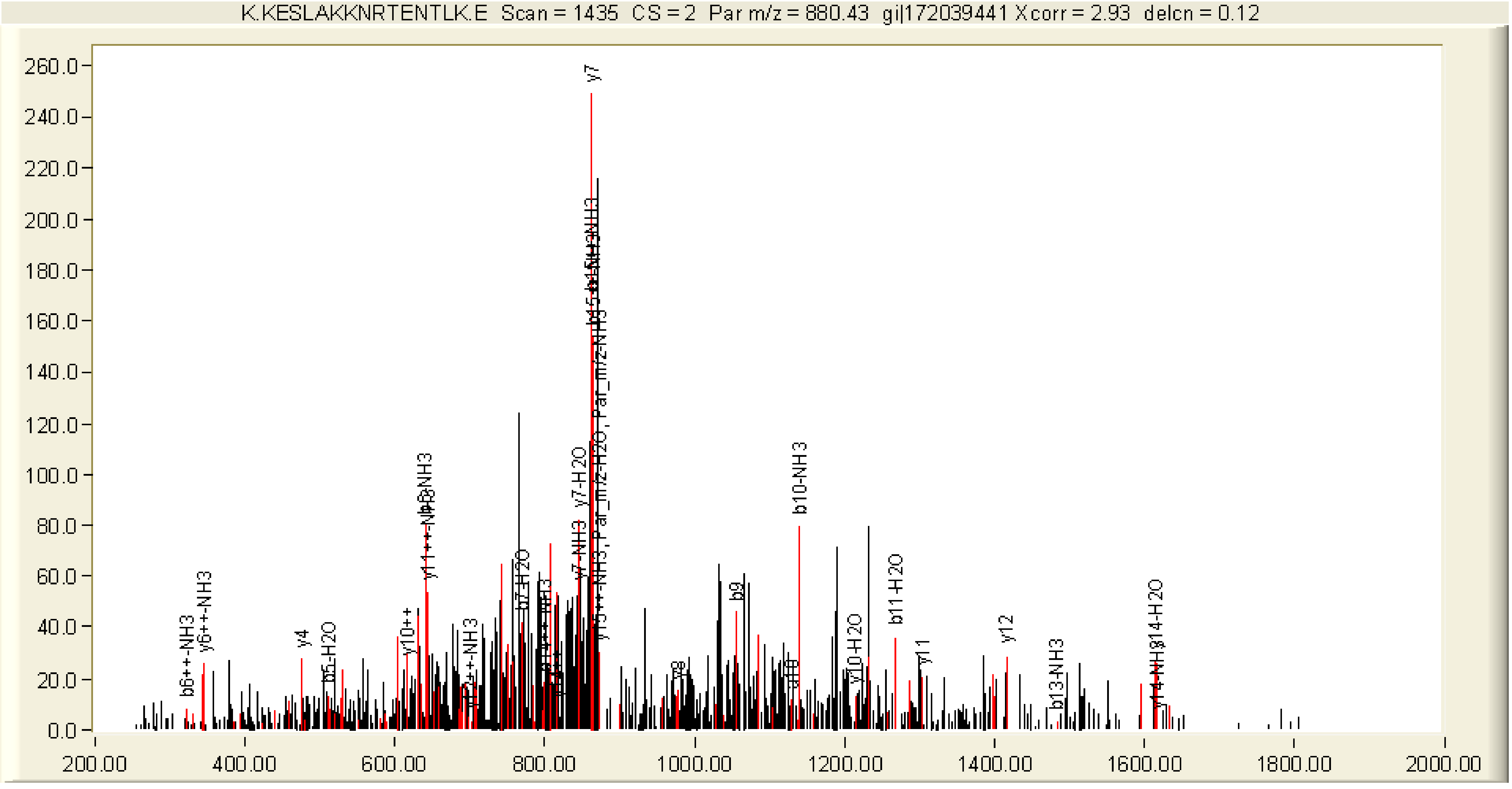
K.KESLAKKNRTENTLK.E Scan = 1435 CS = 2 Par m/z = 880.43 gi|172039441 Xcorr = 2.93 delcn = 0.12
260.0
240.0
220.0
200.0
180.0
160.0
140.0
120.0
100.0
80.0
60.0
40.0
20.0
0.0
200.00
400.00
600.00
800.00
1000.00
1200.00
1400.00
1600.00
1800.00
2000.00
y7
b6++-NH3
y6++-NH3
y4
b5-H2O
y10++
b7-H2O
y7-NH3
y7-H2O
b10-NH3
b9
y8
y10-H2O
b11-H2O
y11
y12
b13-NH3
y14-H2O

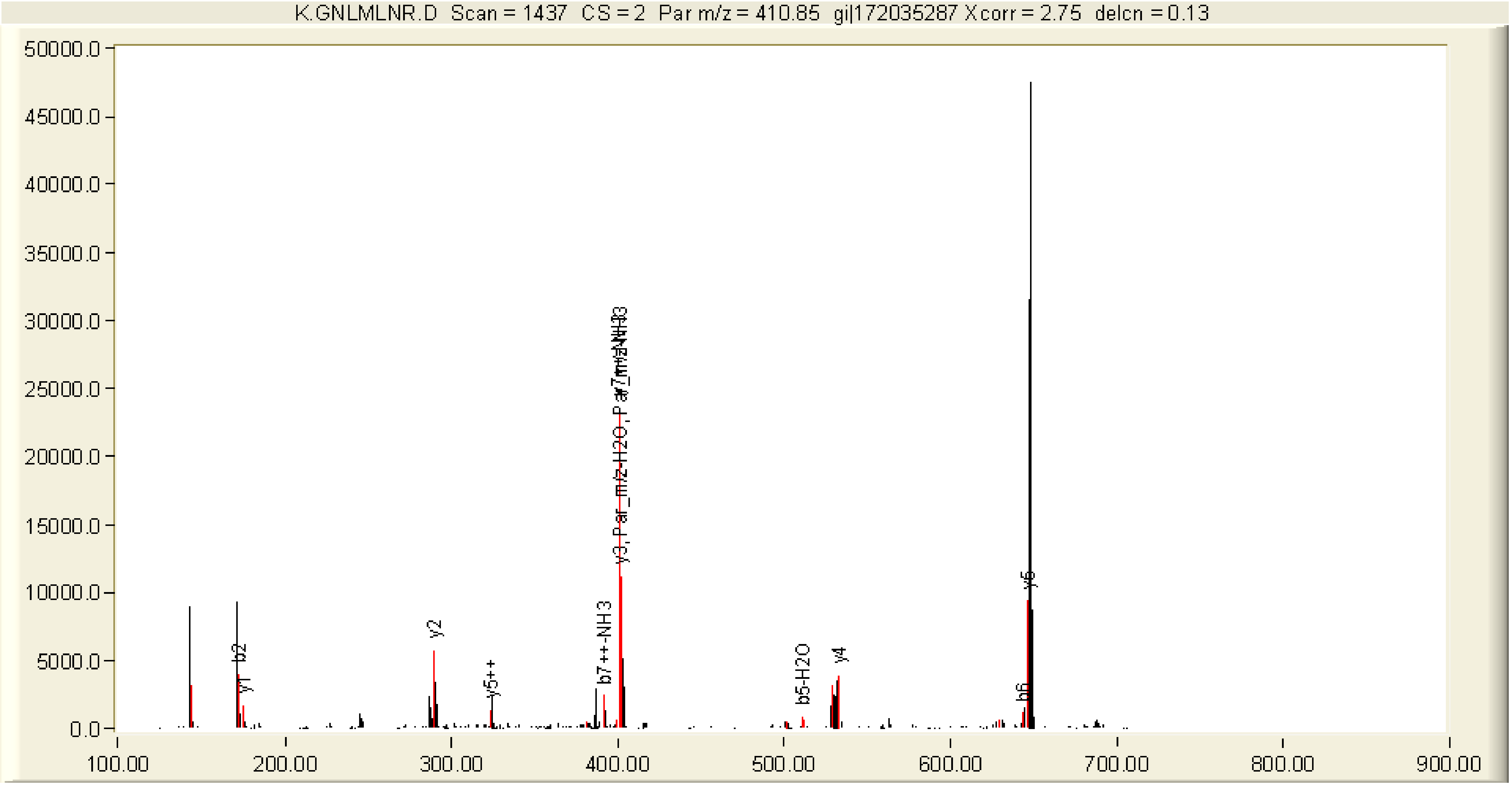

K.GNLMLNR.D Scan = 1437 CS = 2 Par m/z = 410.85 gi|172035287 Xcorr = 2.75 delcn = 0.13
50000.0
45000.0
40000.0
35000.0
30000.0
25000.0
20000.0
15000.0
10000.0
5000.0
0.0
100.00
200.00
300.00
400.00
500.00
600.00
700.00
800.00
900.00
y1
b2
y2
y5++
b7++-NH3
b5-H2O
y4
b6
y6

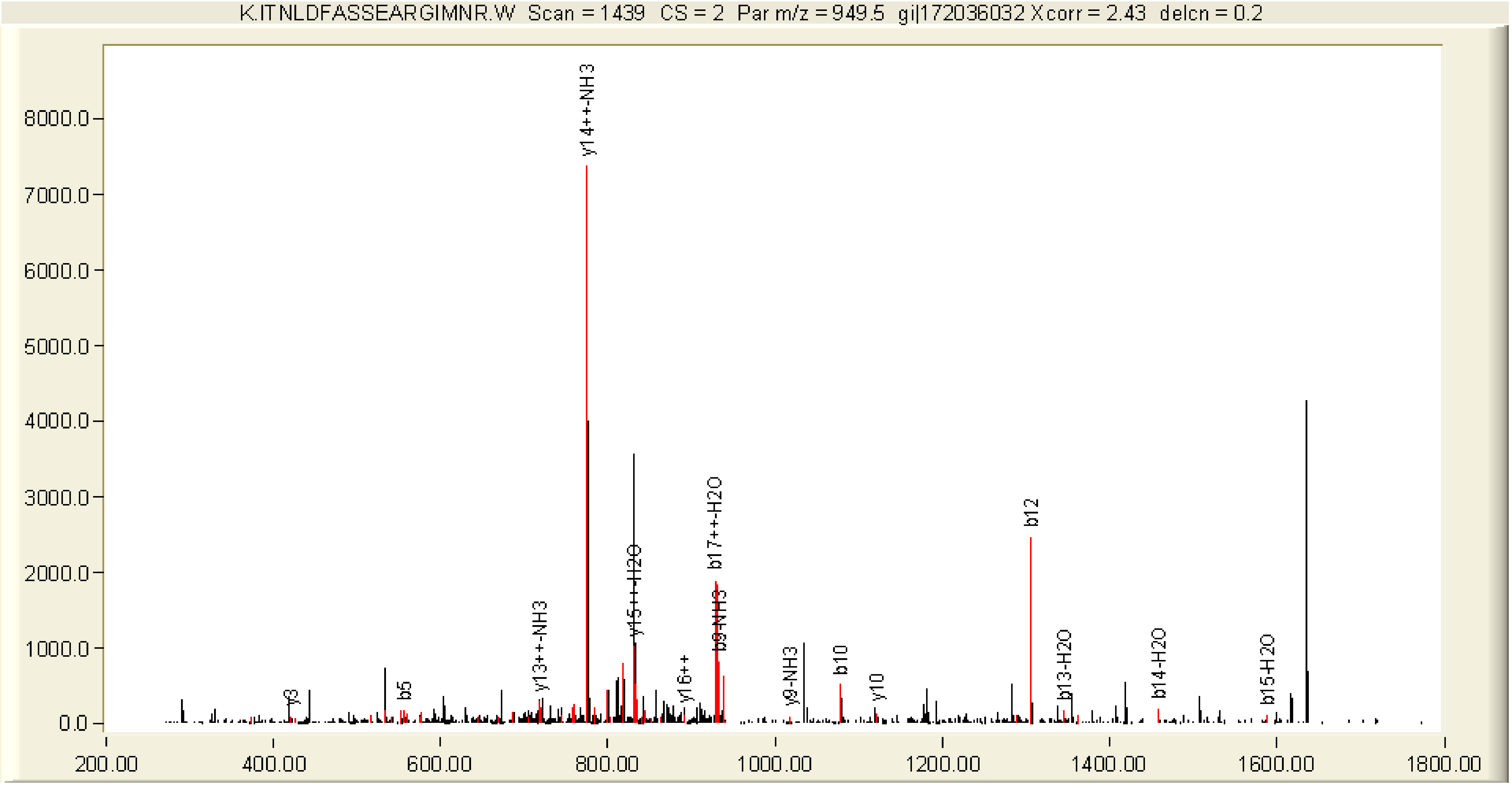
K.ITNLDFASSEARGIMNR.W Scan = 1439 CS = 2 Par m/z = 949.5 gi|172036032 Xcorr = 2.43 delcn = 0.2
y14++-NH3
y15++-H2O
b17++-H2O
b9-NH3
y13++-NH3
y16++
y3
b5
y9-NH3
b10
y10
b12
b13-H2O
b14-H2O
b15-H2O
8000.0
7000.0
6000.0
5000.0
4000.0
3000.0
2000.0
1000.0
0.0
200.00
400.00
600.00
800.00
1000.00
1200.00
1400.00
1600.00
1800.00

K.TIFILARNDNNEELQIVIFANGIWR.F Scan = 1441 CS = 3 Par m/z = 988.37 gi|172038026 Xcorr = 3.24 delcn = 0.12

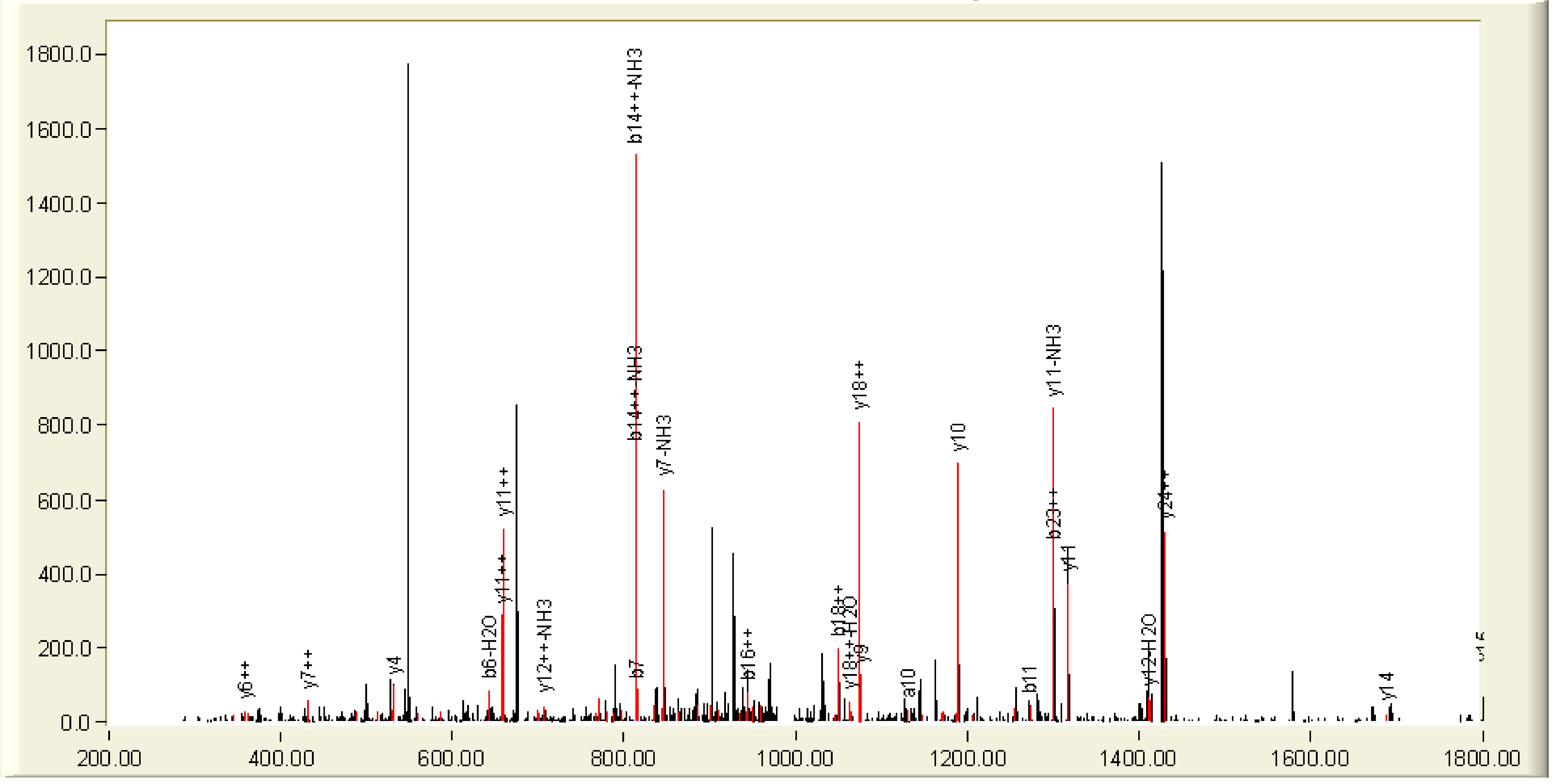

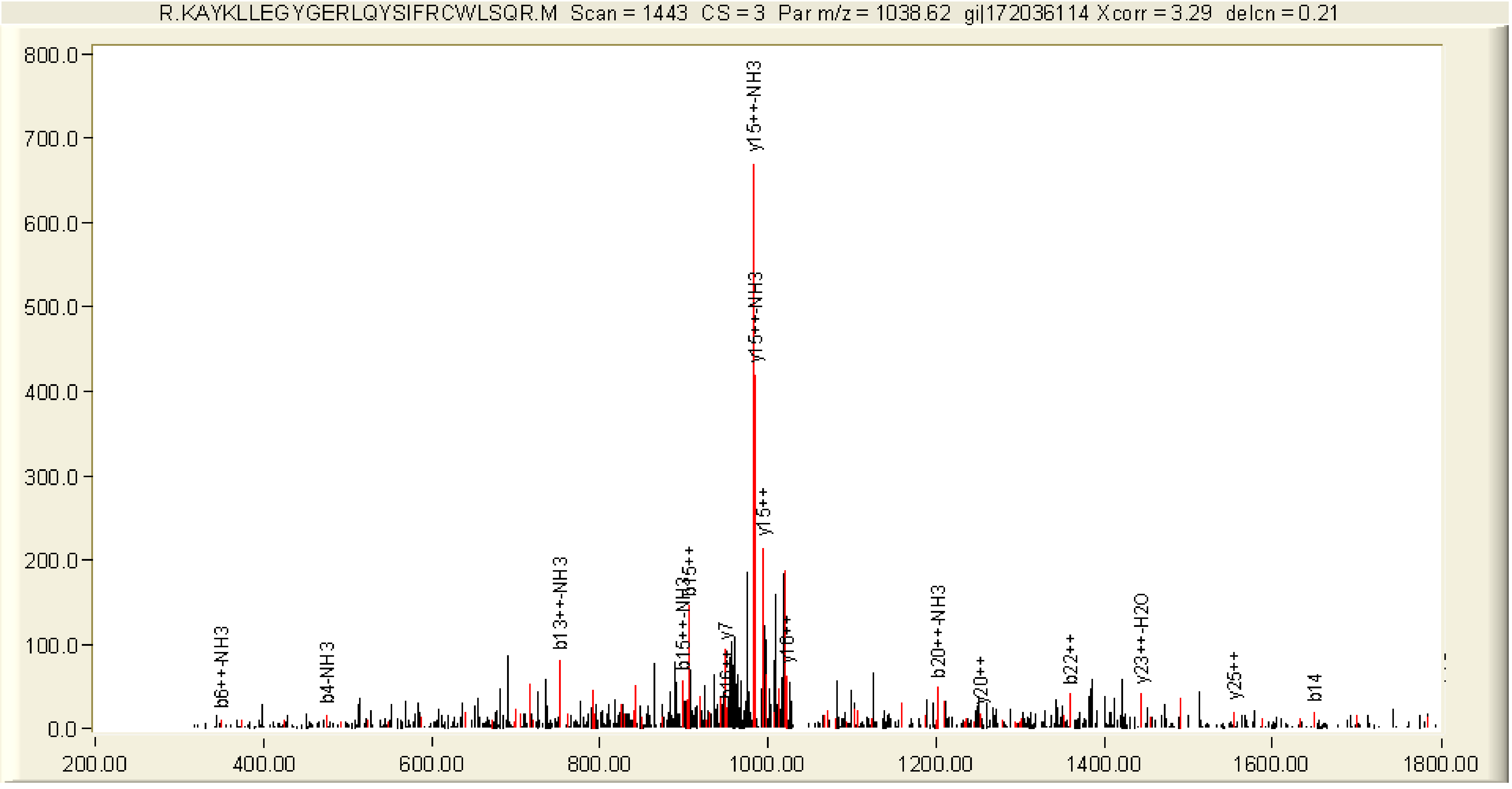

R.KAYKLLEGYGERLQYSIFRCWLSQR.M Scan = 1443 CS = 3 Par m/z = 1038.62 gi|17203614 Xcorr = 3.29 delcn = 0.21
y15++-NH3
y15++-NH3
y15++
b15++
b15++-NH3
b13++-NH3
y7
y16++
b20++-NH3
y20++
b22++
y23++-H2O
y25++
b14
b6++-NH3
b4-NH3
800.0
700.0
600.0
500.0
400.0
300.0
200.0
100.0
0.0
200.00
400.00
600.00
800.00
1000.00
1200.00
1400.00
1600.00
1800.00

K.QILRHKKSVRISLAKFLSK.L Scan = 1445 CS = 2 Par m/z = 1128.25 gi|172039158 Xcorr = 2.4 delcn = 0.27

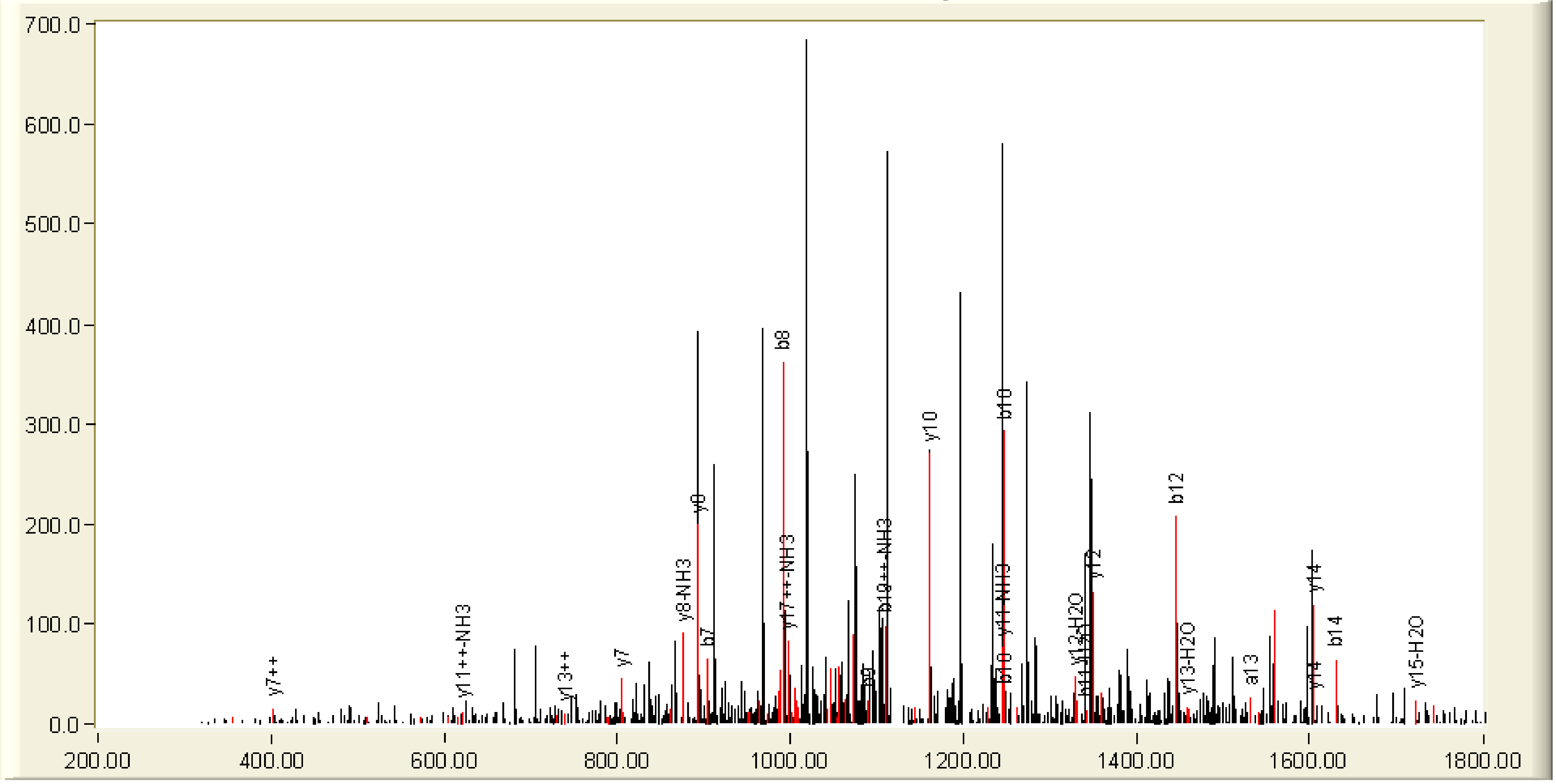

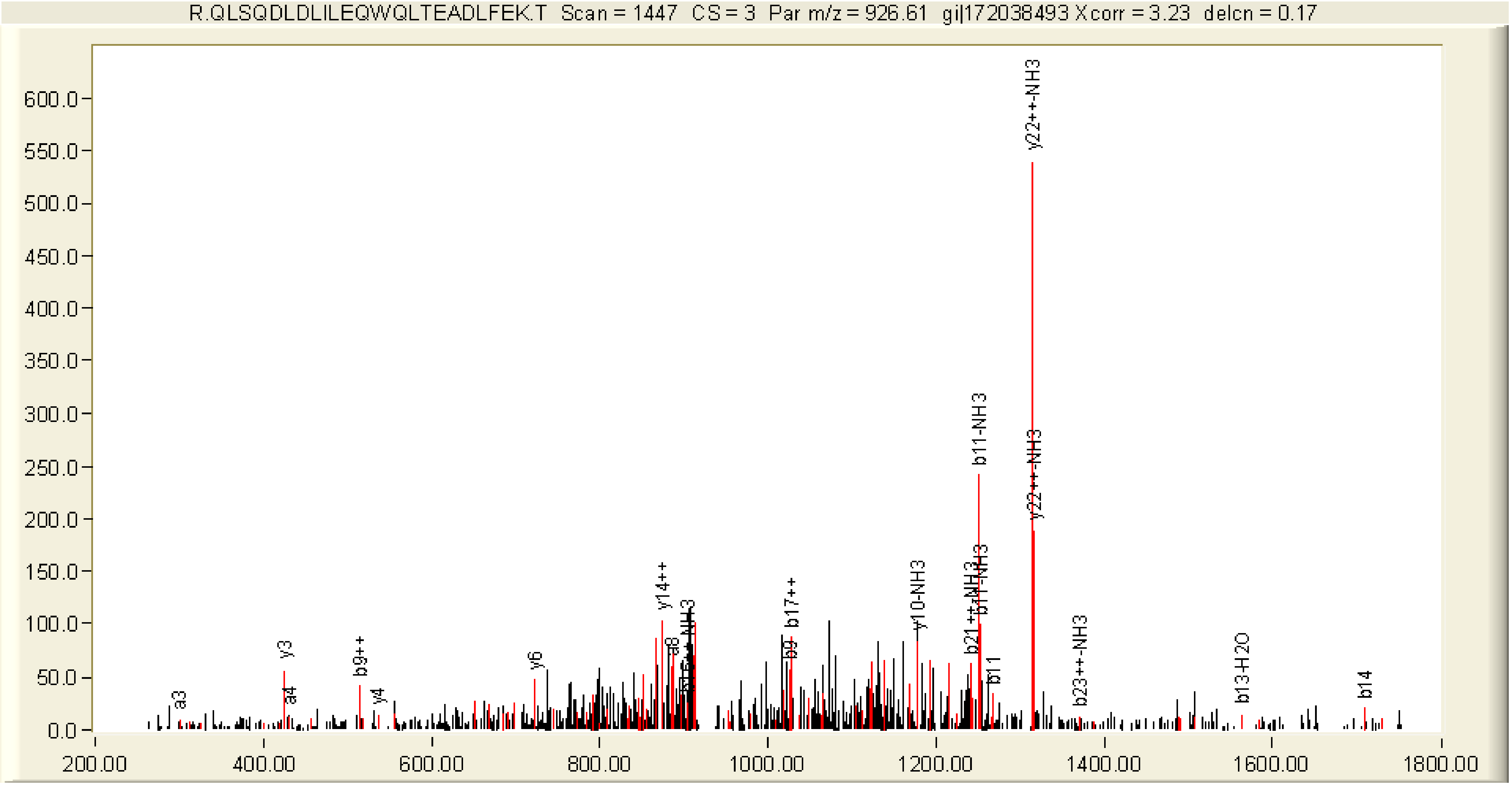

R.QLSQDLDLILEQWQLTEADLFEK.T Scan = 1447 CS = 3 Par m/z = 926.61 gi|172038493 Xcorr = 3.23 delcn = 0.17
600.0
550.0
500.0
450.0
400.0
350.0
300.0
250.0
200.0
150.0
100.0
50.0
0.0
200.00
400.00
600.00
800.00
1000.00
1200.00
1400.00
1600.00
1800.00
y22++-NH3
b11-NH3
y22++-NH3
y14++
b17++
y10-NH3
b21++-NH3
b11-NH3
a8
b9
b11
b23++-NH3
b13-H2O
b14
y3
a4
b9++
y4
y6
a3

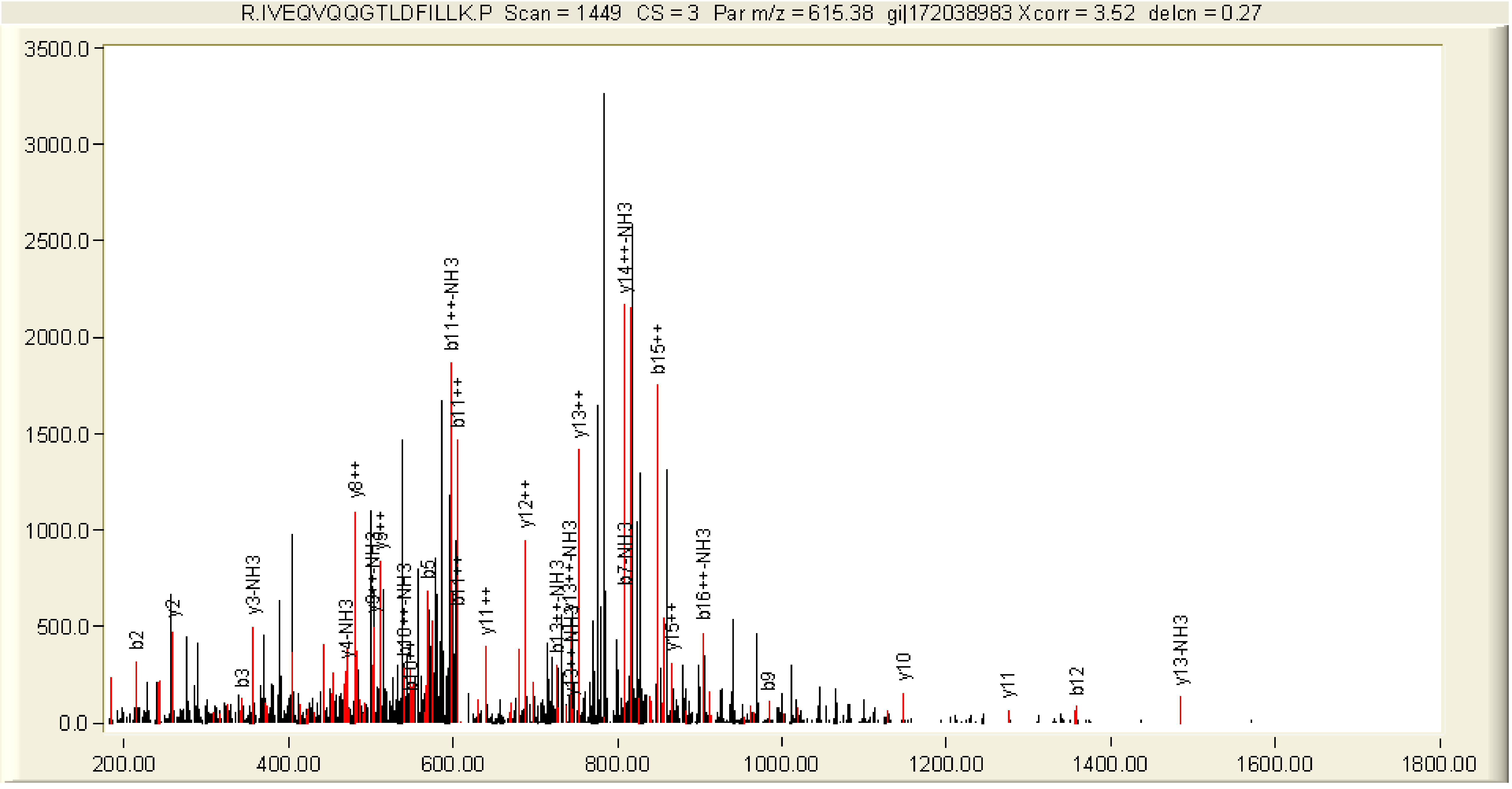
R.IVEQVQQGTLDFILLK.P Scan = 1449 CS = 3 Par m/z = 615.38 gi|172038983 Xcorr = 3.52 delcn = 0.27
3500.0
3000.0
2500.0
2000.0
1500.0
1000.0
500.0
0.0
200.00
400.00
600.00
800.00
1000.00
1200.00
1400.00
1600.00
1800.00
b2
y2
b3
y3-NH3
y4-NH3
y8++
y9++
b5
b11++-NH3
b11++
y11++
y12++
y13++
y14++-NH3
b7-NH3
b15++
y15++
b16++-NH3
b9
y10
y11
b12
y13-NH3

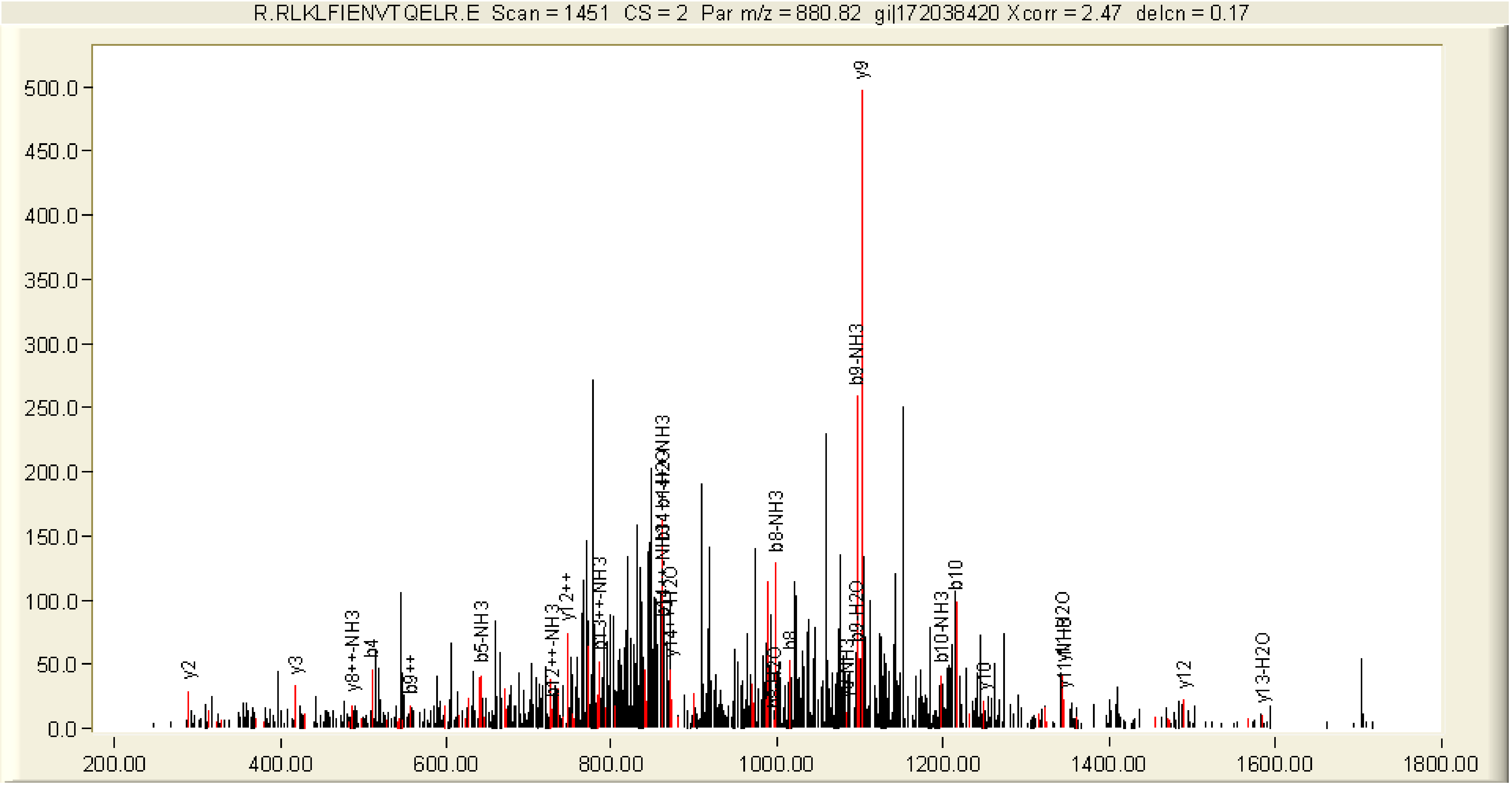

R.RLKLFIENVTQELR.E Scan = 1451 CS = 2 Par m/z = 880.82 gi|172038420 Xcorr = 2.47 delcn = 0.17
500.0
450.0
400.0
350.0
300.0
250.0
200.0
150.0
100.0
50.0
0.0
200.00
400.00
600.00
800.00
1000.00
1200.00
1400.00
1600.00
1800.00
y2
y3
y8++-NH3
b4
b9++
b5-NH3
y12++
b13++-NH3
b8-NH3
b8
y9
b9-NH3
b9-H2O
b10-NH3
b10
y10
y12
y13-H2O

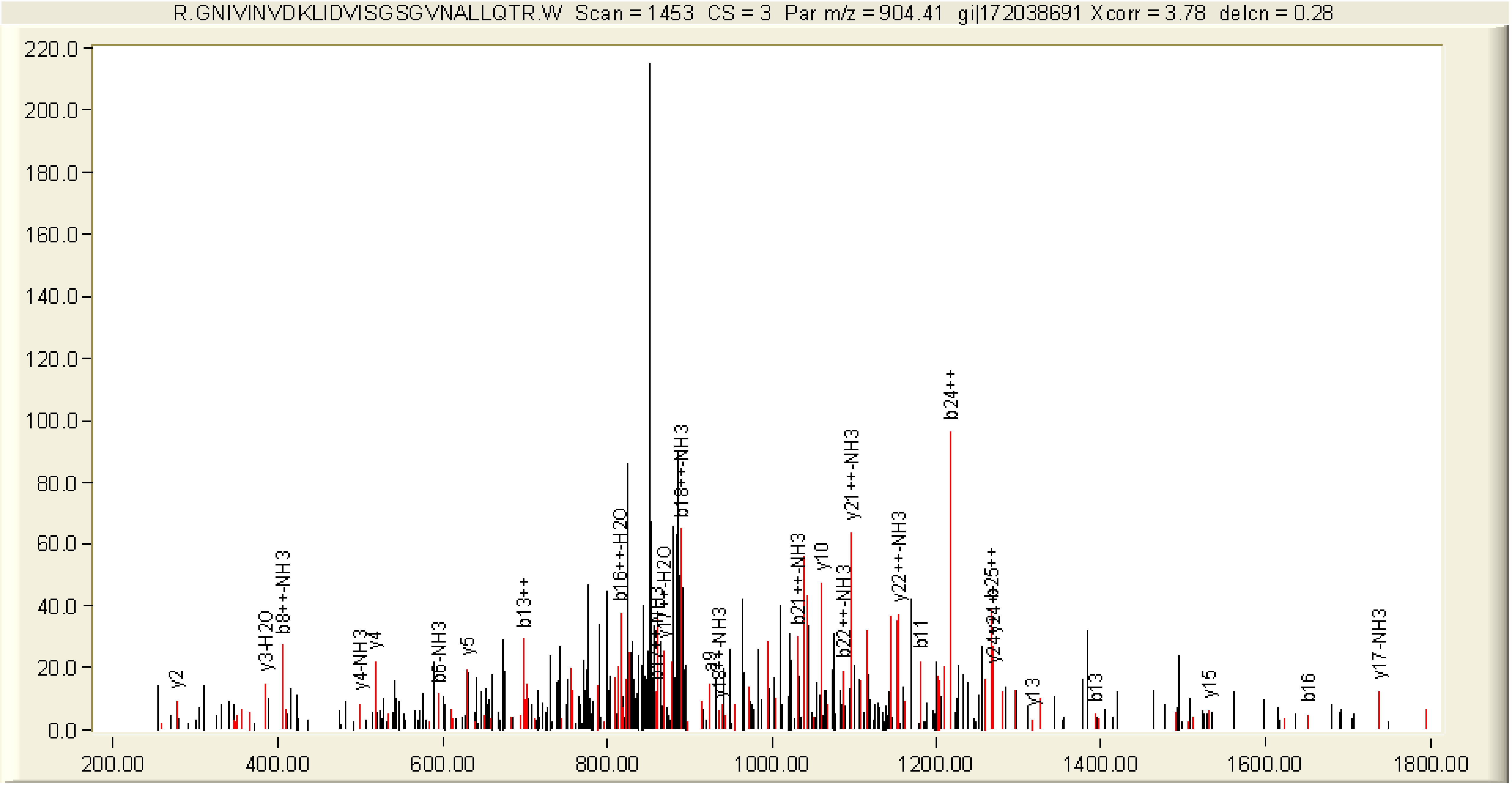

R.GNIVINVDKLIDVISGSGVNALLQTR.W Scan = 1453 CS = 3 Par m/z = 904.41 gi|172038691 Xcorr = 3.78 delcn = 0.28
220.0
200.0
180.0
160.0
140.0
120.0
100.0
80.0
60.0
40.0
20.0
0.0
200.00
400.00
600.00
800.00
1000.00
1200.00
1400.00
1600.00
1800.00
y2
y3-H2O
b8++-NH3
y4-NH3
y4
b6-NH3
y5
b13++
b16++-H2O
b18++-NH3
y21++-NH3
y10
b21++-NH3
b22++-NH3
y22++-NH3
b24++
b11
b25++
y13
b13
y15
b16
y17-NH3

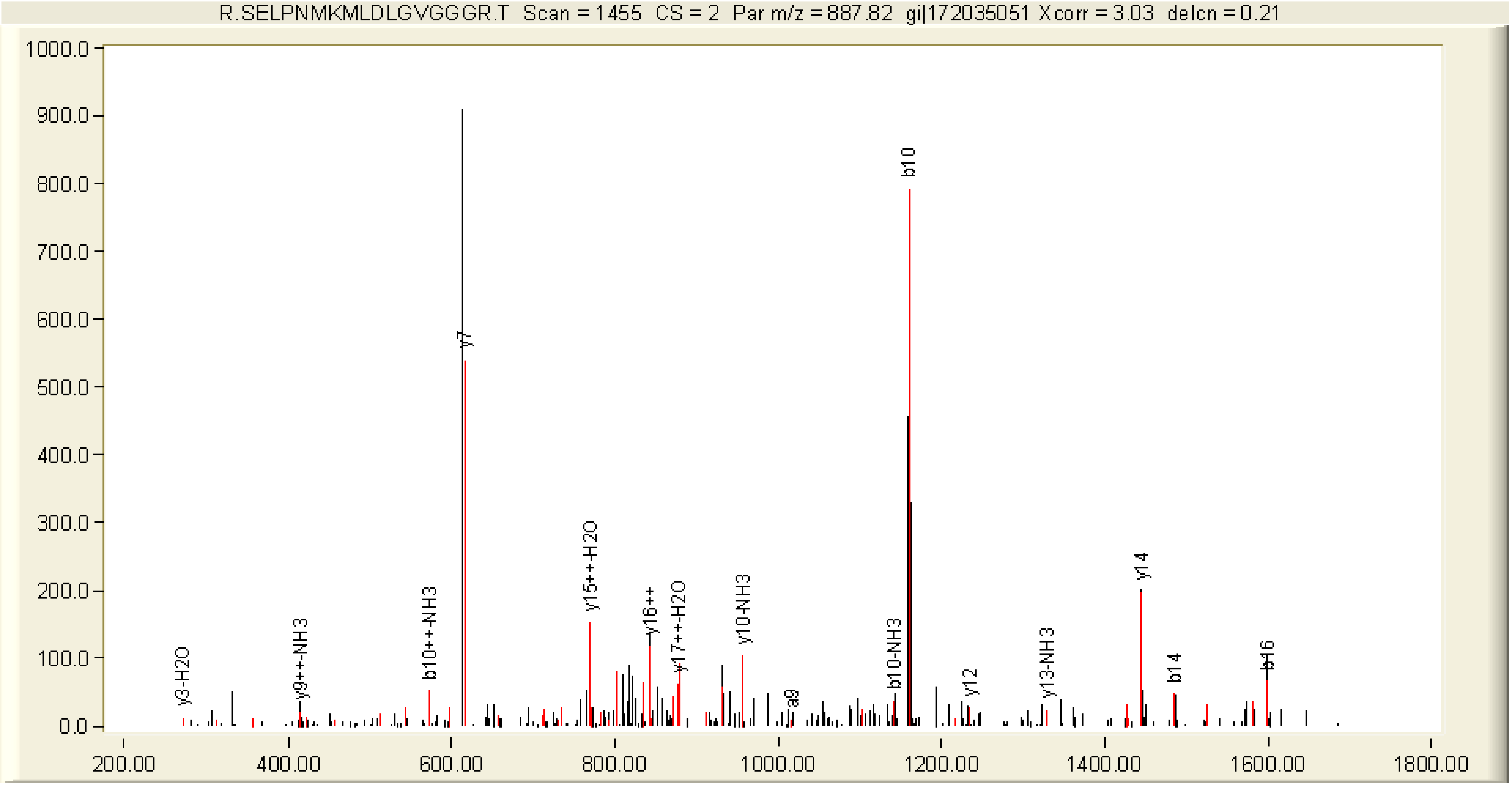
R.SELPNMKMLDLGVGGGR.T Scan = 1455 CS = 2 Par m/z = 887.82 gi|172035051 Xcorr = 3.03 delcn = 0.21
1000.0
900.0
800.0
700.0
600.0
500.0
400.0
300.0
200.0
100.0
0.0
200.00
400.00
600.00
800.00
1000.00
1200.00
1400.00
1600.00
1800.00
y3-H2O
y9++-NH3
b10++-NH3
y7
y15++-H2O
y16++
y17++-H2O
y10-NH3
a9
b10-NH3
b10
y12
y13-NH3
y14
b14
b16

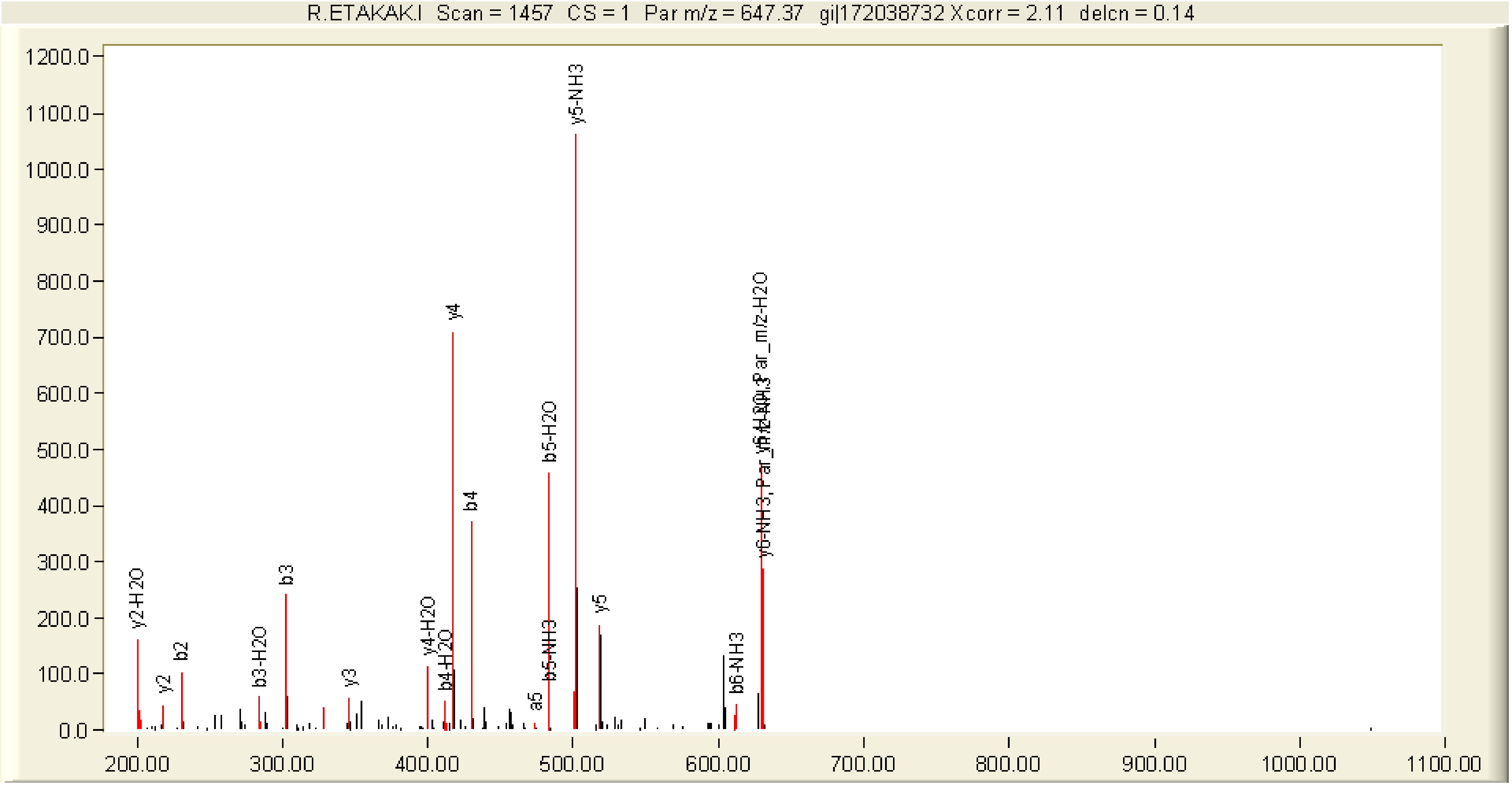
R.ETAKAK.I Scan = 1457 CS = 1 Par m/z = 647.37 gi|172038732 Xcorr = 2.11 delcn = 0.14
1200.0
1100.0
1000.0
900.0
800.0
700.0
600.0
500.0
400.0
300.0
200.0
100.0
0.0
200.00
300.00
400.00
500.00
600.00
700.00
800.00
900.00
1000.00
1100.00
y2-H2O
y2
b2
b3-H2O
b3
y3
y4-H2O
b4-H2O
y4
b4
a5
b5-NH3
b5-H2O
y5-NH3
y5
b6-NH3
y6-NH3, Par_m/z-H2O

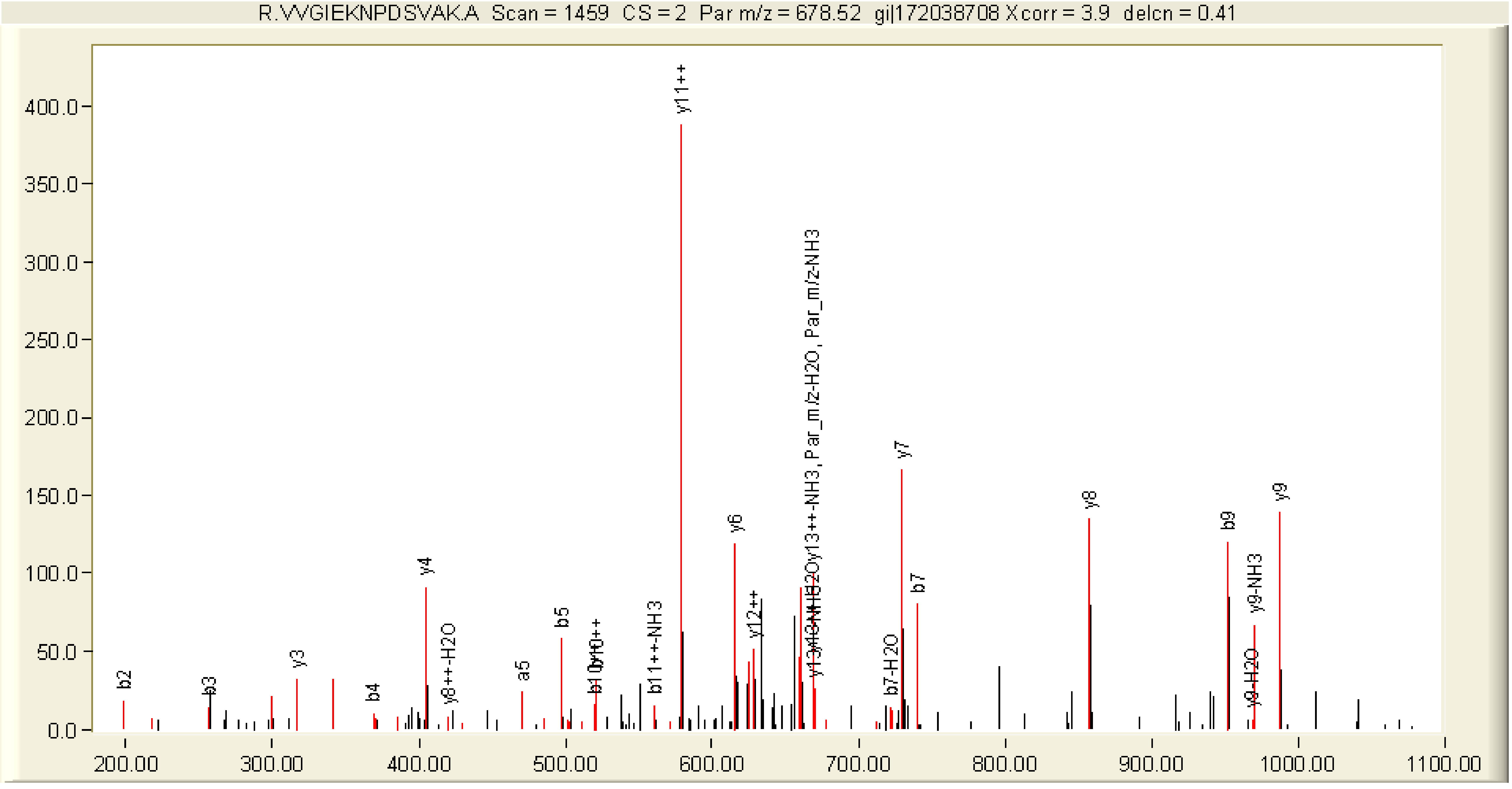
R.VVGIEKNPDSVAK.A Scan = 1459 CS = 2 Par m/z = 678.52 gi|172038708 Xcorr = 3.9 delcn = 0.41
0.0
50.0
100.0
150.0
200.0
250.0
300.0
350.0
400.0
200.00
300.00
400.00
500.00
600.00
700.00
800.00
900.00
1000.00
1100.00
b2
b3
y3
b4
y4
y8++-H2O
a5
b5
b11++-NH3
y11++
y6
y12++
y13++-NH3, Par_m/z-H2O, Par_m/z-NH3
b7-H2O
y7
b7
y8
b9
y9-H2O
y9-NH3
y9

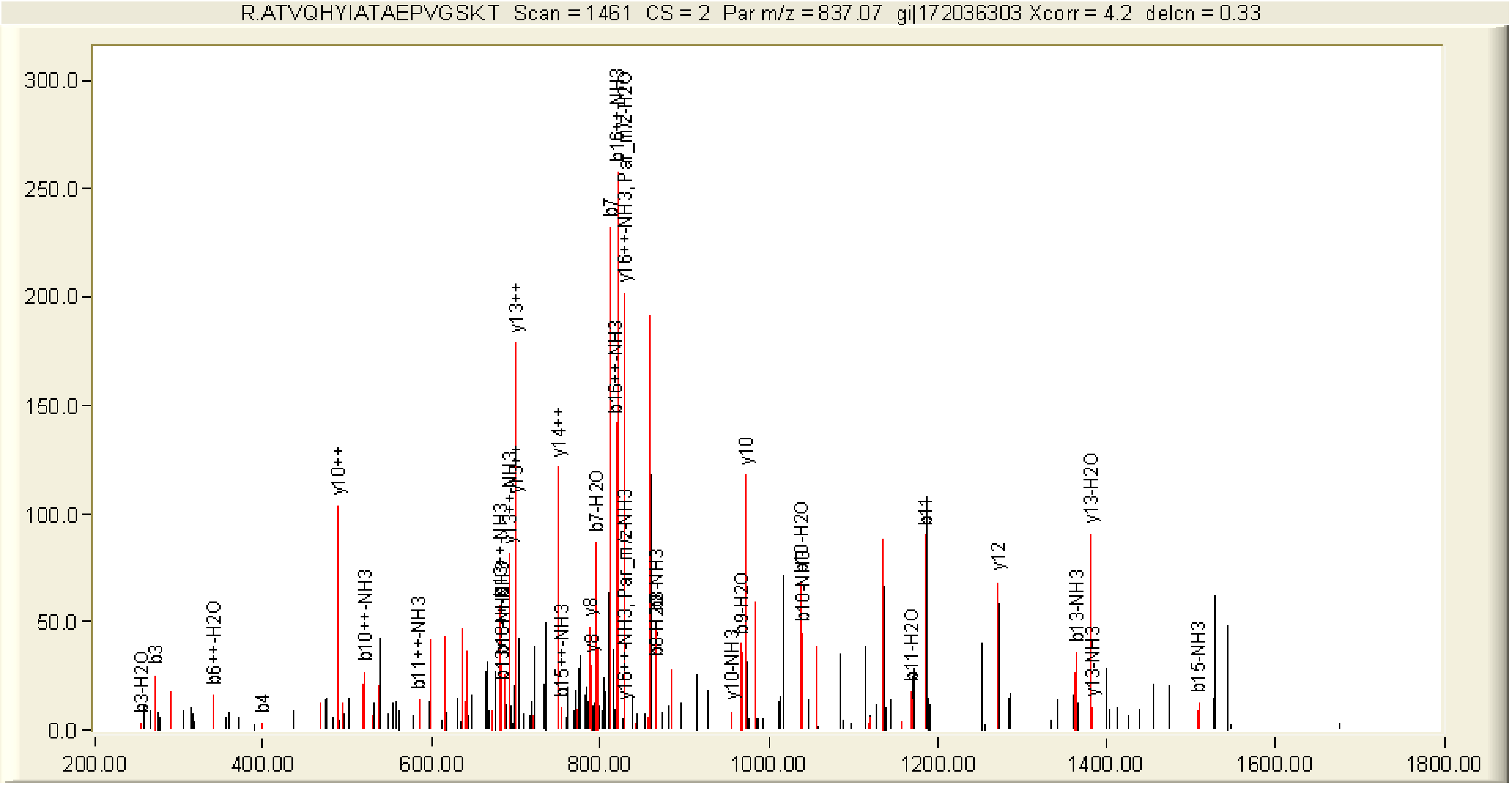
R.ATVQHYIATAEPVGSKT Scan = 1461 CS = 2 Par m/z = 837.07 gi|172036303 Xcorr = 4.2 delcn = 0.33
300.0
250.0
200.0
150.0
100.0
50.0
0.0
200.00
400.00
600.00
800.00
1000.00
1200.00
1400.00
1600.00
1800.00
b3-H2O
b3
b6++-H2O
b4
y10++
b10++-NH3
b11++-NH3
y13++
y14++
b15++-NH3
b7-H2O
y8
b7
y16++-NH3, Par_m/z-NH3
y10
y10-NH3
b9-H2O
b10-H2O
b11-H2O
b11
y12
y13-H2O
b13-NH3
y13-NH3
b15-NH3

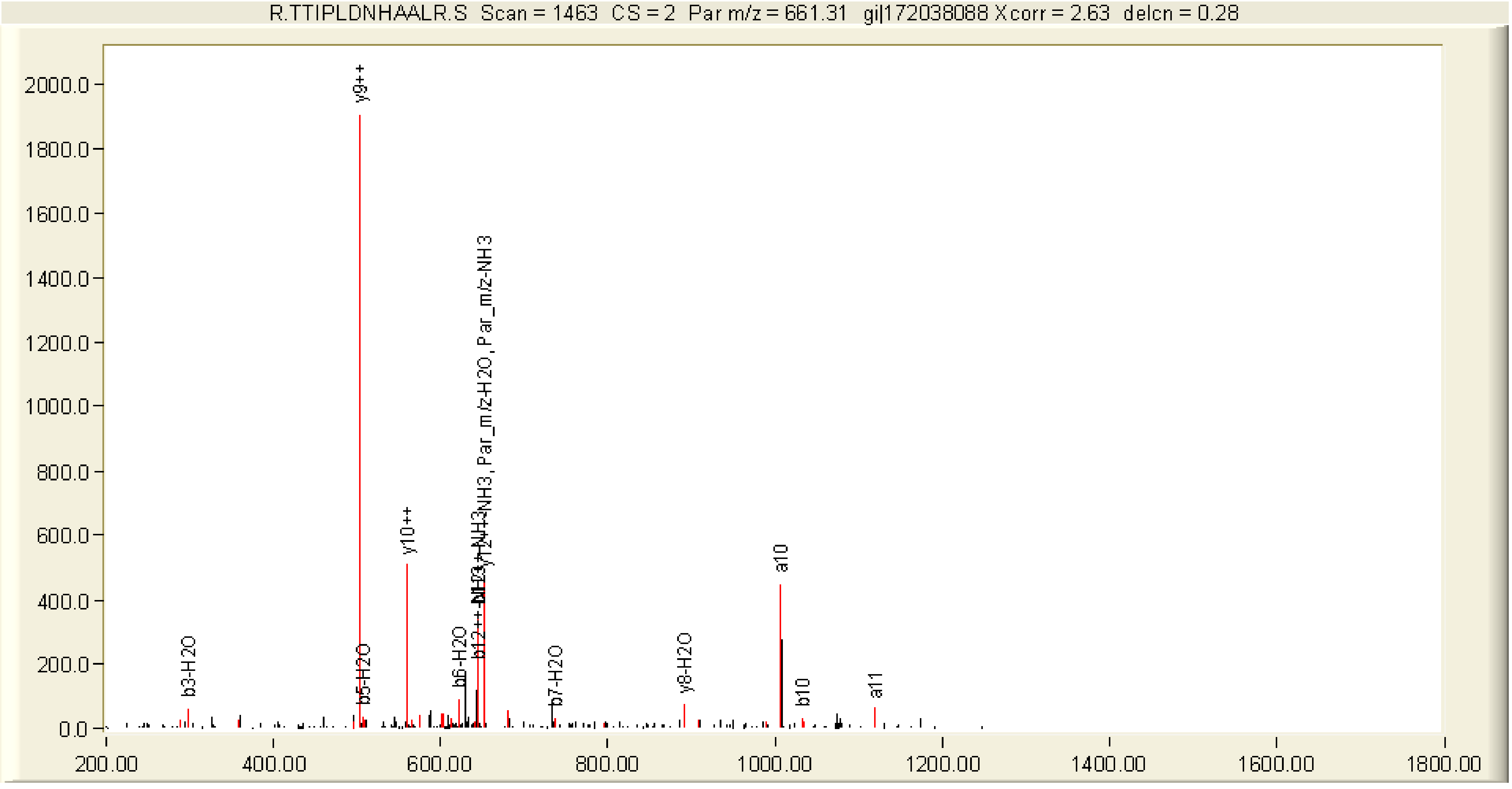
R.TTIPLDNHAALR.S Scan = 1463 CS = 2 Par m/z = 661.31 gi|172038088 Xcorr = 2.63 delcn = 0.28
2000.0
1800.0
1600.0
1400.0
1200.0
1000.0
800.0
600.0
400.0
200.0
0.0
200.00
400.00
600.00
800.00
1000.00
1200.00
1400.00
1600.00
1800.00
y9++
y10++
a10
b3-H2O
b5-H2O
b6-H2O
b7-H2O
y8-H2O
b10
a11
y12++-NH3, Par_m/z-H2O, Par_m/z-NH3

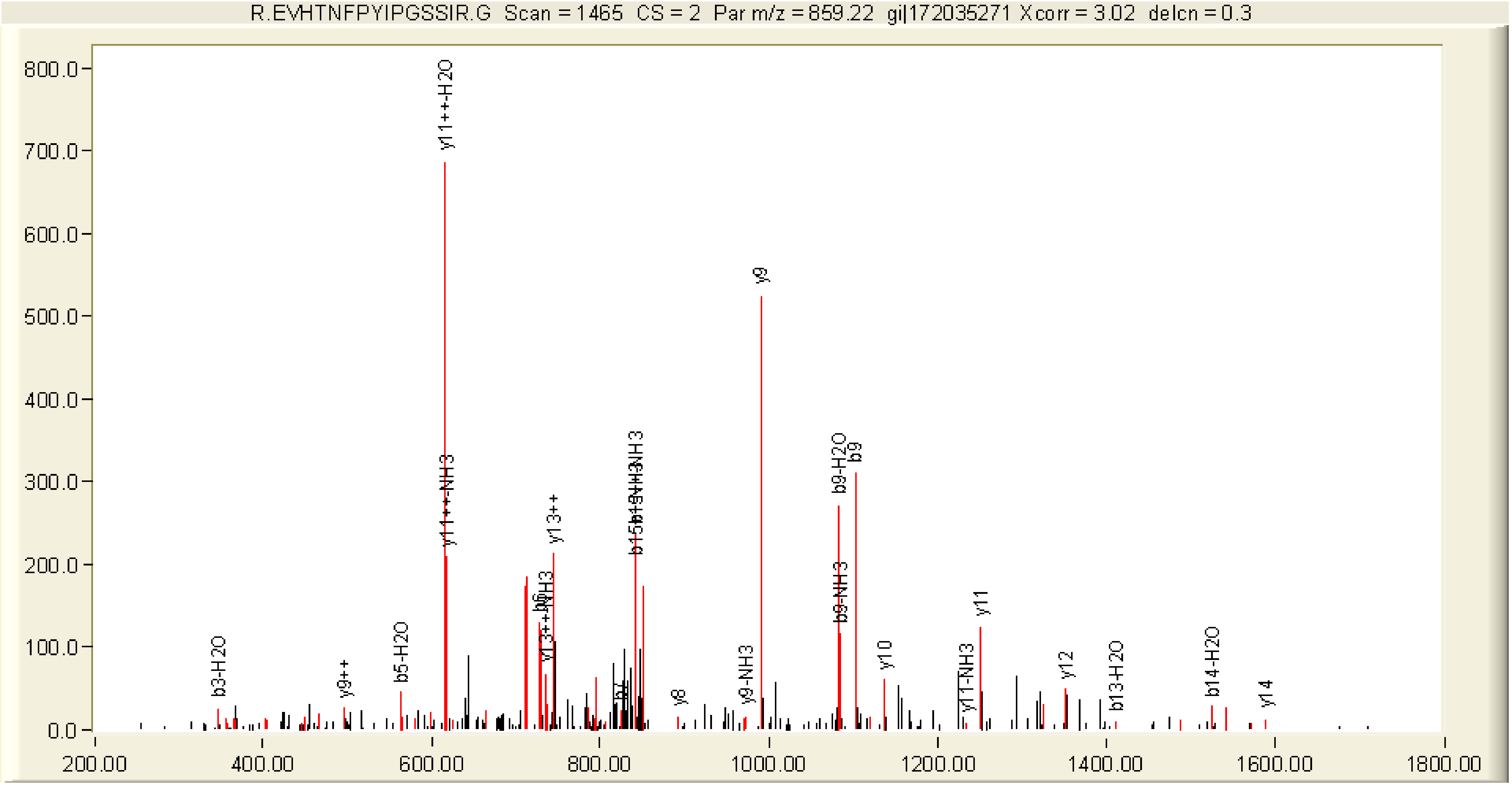
R.EVHTNFPYIPGSSIR.G Scan = 1465 CS = 2 Par m/z = 859.22 gi|172035271 Xcorr = 3.02 delcn = 0.3
y11++-H2O
y9
b9-H2O
b9
y11++-NH3
y13++
b9-NH3
y11
b3-H2O
y9++
b5-H2O
y10
y12
b13-H2O
b14-H2O
y14
y8
y9-NH3
y11-NH3
800.0
700.0
600.0
500.0
400.0
300.0
200.0
100.0
0.0
200.00
400.00
600.00
800.00
1000.00
1200.00
1400.00
1600.00
1800.00

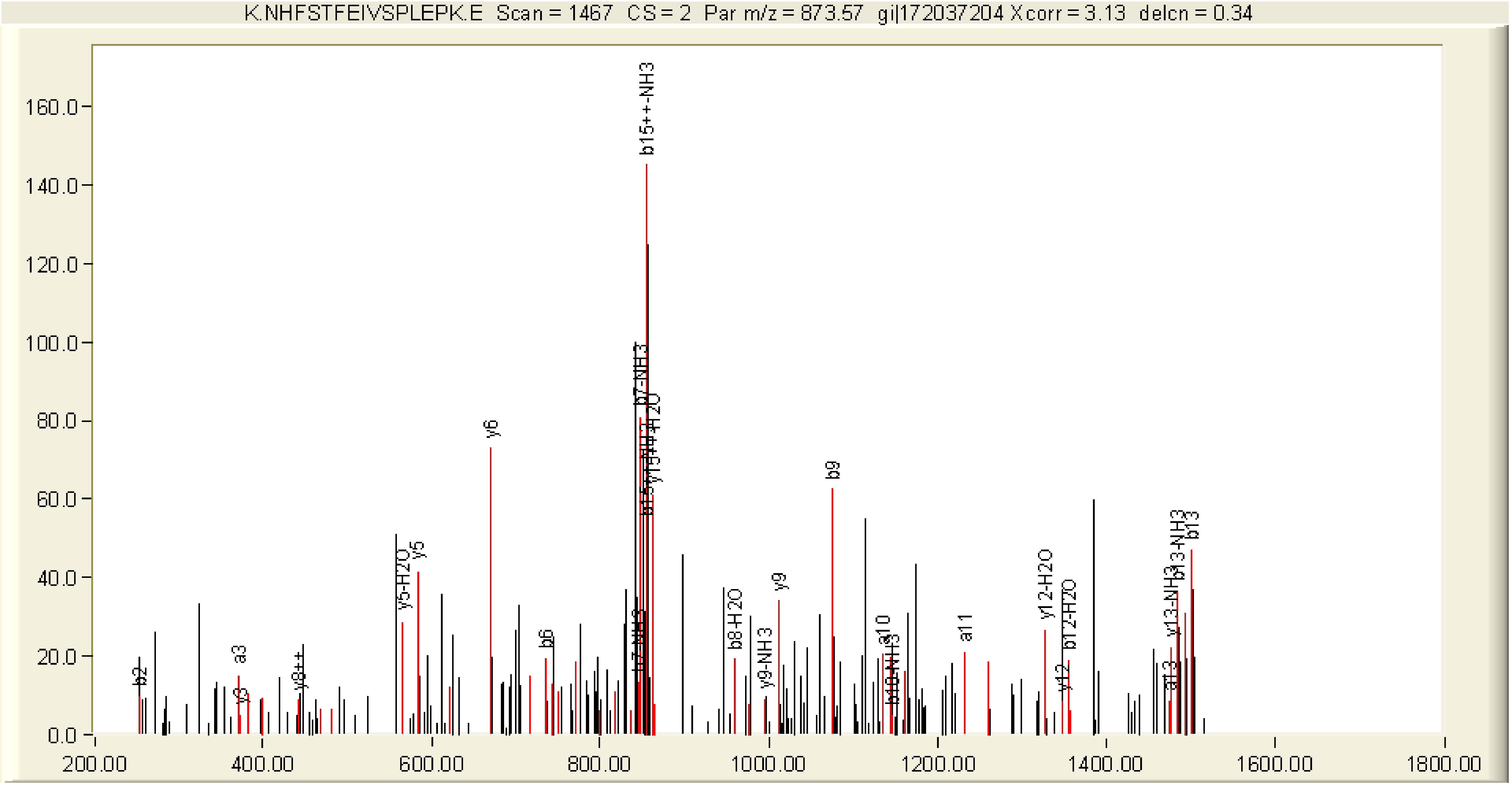

K.NHFSTFEIVSPLEPK.E Scan = 1467 CS = 2 Par m/z = 873.57 gi|172037204 Xcorr = 3.13 delcn = 0.34
b15++-NH3
y6
b9
y5-H2O
y5
a3
b2
y3
y8++
b6
b8-H2O
y9-NH3
y9
a10
a11
y12-H2O
b12-H2O
y12
a13
y13-NH3
b13-NH3
b13
b7-NH3
160.0
140.0
120.0
100.0
80.0
60.0
40.0
20.0
0.0
200.00
400.00
600.00
800.00
1000.00
1200.00
1400.00
1600.00
1800.00

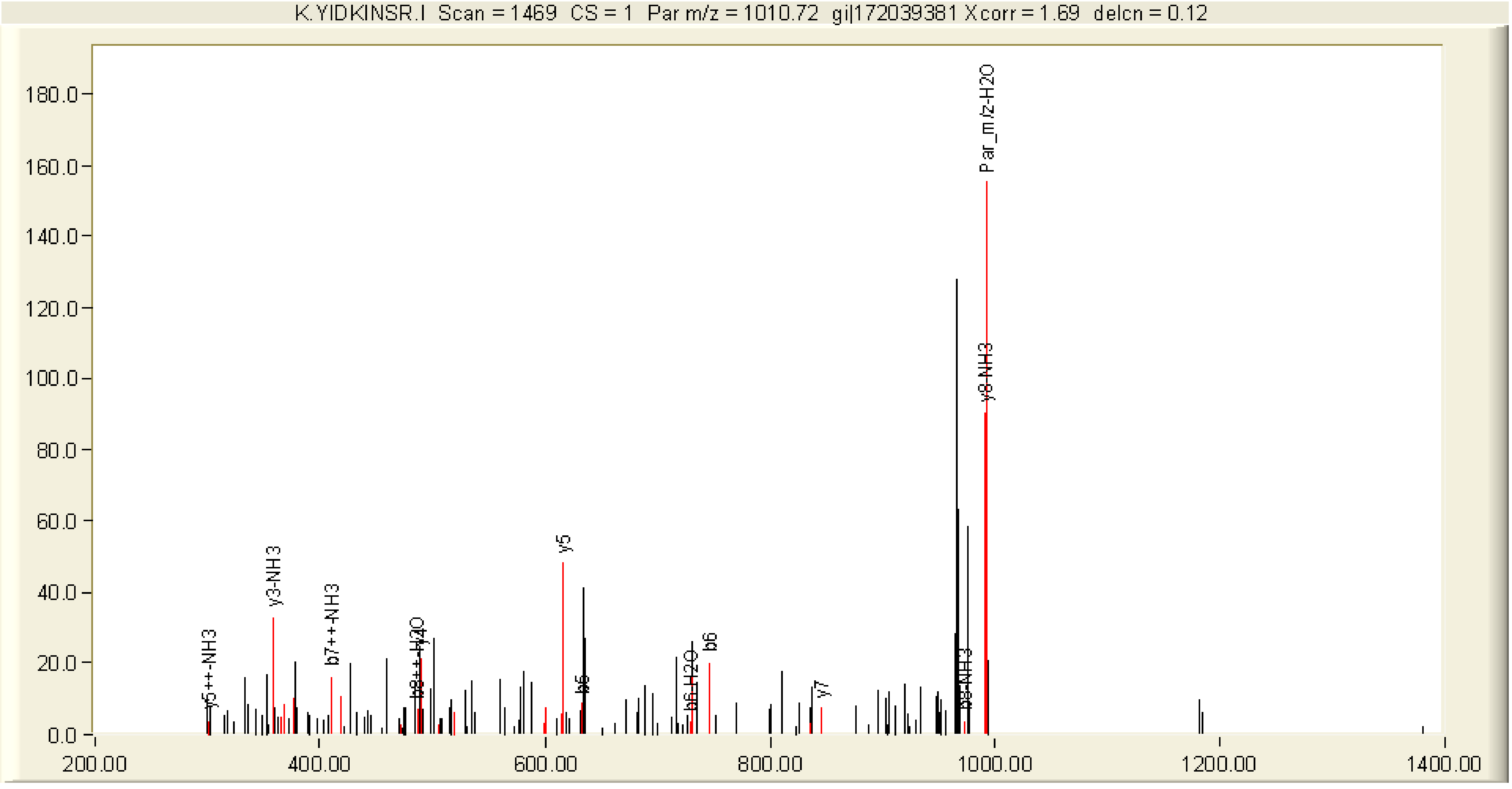
K.YIDKINSR.I Scan = 1469 CS = 1 Par m/z = 1010.72 gi|172039381 Xcorr = 1.69 delcn = 0.12
Par_m/z-H2O
y8-NH3
y5
y3-NH3
b7++-NH3
y5++-NH3
b6
b6-H2O
y7
b8-NH3
180.0
160.0
140.0
120.0
100.0
80.0
60.0
40.0
20.0
0.0
200.00
400.00
600.00
800.00
1000.00
1200.00
1400.00

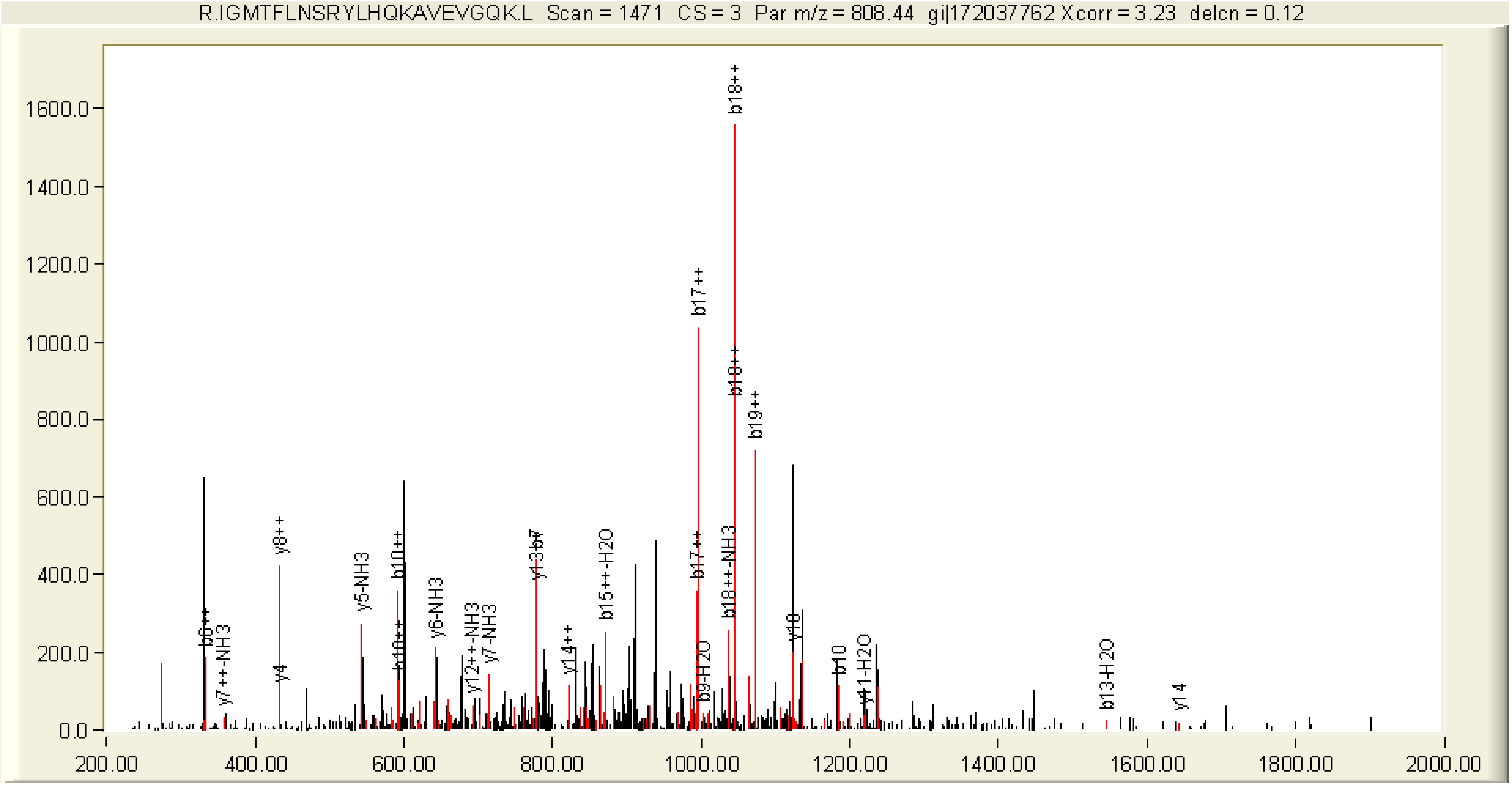
R.IGMTFLNSRYLHQKAVEVGQK.L Scan = 1471 CS = 3 Par m/z = 808.44 gi|172037762 Xcorr = 3.23 delcn = 0.12
1600.0
1400.0
1200.0
1000.0
800.0
600.0
400.0
200.0
0.0
200.00
400.00
600.00
800.00
1000.00
1200.00
1400.00
1600.00
1800.00
2000.00
b18++
b17++
b19++
y8++
y5-NH3
b10++
y6-NH3
y12++-NH3
y7-NH3
y7++-NH3
y4
b15++-H2O
b18++-NH3
b9-H2O
y14++
y10
b10
y11-H2O
b13-H2O
y14
